# enDNA-Prot: Identification of DNA-Binding Proteins by Applying Ensemble Learning

**DOI:** 10.1155/2014/294279

**Published:** 2014-05-26

**Authors:** Ruifeng Xu, Jiyun Zhou, Bin Liu, Lin Yao, Yulan He, Quan Zou, Xiaolong Wang

**Affiliations:** ^1^School of Computer Science and Technology, Harbin Institute of Technology Shenzhen Graduate School, Shenzhen, Guangdong 518055, China; ^2^Key Laboratory of Network Oriented Intelligent Computation, Harbin Institute of Technology Shenzhen Graduate School, Shenzhen, Guangdong 518055, China; ^3^Shanghai Key Laboratory of Intelligent Information Processing, Shanghai 518055, China; ^4^Gordon Life Science Institute, Belmont, Massachusetts, USA; ^5^PKU-HKUST ShenZhen-Hong Kong Institution, Shenzhen, Guangdong 518055, China; ^6^Peking University Shenzhen Graduate School, Shenzhen, Guangdong 518055, China; ^7^School of Engineering & Applied Science, Aston University, Birmingham B47ET, UK; ^8^School of Information Science and Technology, Xiamen University, Xiamen, Fujian 316005, China

## Abstract

DNA-binding proteins are crucial for various cellular processes, such as recognition of specific nucleotide, regulation of transcription, and regulation of gene expression. Developing an effective model for identifying DNA-binding proteins is an urgent research problem. Up to now, many methods have been proposed, but most of them focus on only one classifier and cannot make full use of the large number of negative samples to improve predicting performance. This study proposed a predictor called enDNA-Prot for DNA-binding protein identification by employing the ensemble learning technique. Experiential results showed that enDNA-Prot was comparable with DNA-Prot and outperformed DNAbinder and iDNA-Prot with performance improvement in the range of 3.97–9.52% in ACC and 0.08–0.19 in MCC. Furthermore, when the benchmark dataset was expanded with negative samples, the performance of enDNA-Prot outperformed the three existing methods by 2.83–16.63% in terms of ACC and 0.02–0.16 in terms of MCC. It indicated that enDNA-Prot is an effective method for DNA-binding protein identification and expanding training dataset with negative samples can improve its performance. For the convenience of the vast majority of experimental scientists, we developed a user-friendly web-server for enDNA-Prot which is freely accessible to the public.

## 1. Introduction

DNA-binding proteins are very important constituent of proteomes of living body, including eukaryotic and prokaryotic. They play crucial roles in various cellular processes, such as DNA packaging, replication, transcription regulation, and other activities associated with DNA. In the early days of DNA-binding protein identification, it was tackled mainly by experimental techniques, including filter binding assays, genetic analysis, chromatin immunoprecipitation on microarrays, and X-ray crystallography. Although they achieved superior performance, its characteristics of time consumption and expensive cost make it low practical value. Later automated methods have been developed to work out the difficulty of experimental methods. In the past, many efforts have been made for developing automated methods, and several predictors have been proposed. Broadly, these methods can be divided into four categories: (1) methods based on support vector machine (SVM), (2) methods based on Random Tree, (3) methods based on artificial neural network (ANN), and (4) other methods.

SVM-based predictive methods are the most commonly used methods. Cai and Lin [1] introduced SVM and the pseudo amino acid composition, a collection of nonlinear features extractable from protein sequence to the field of protein function prediction. Yu et al. [2] integrated SVMs, protein sequence amino acid composition, and associated physicochemical properties for rRNA-, RNA-, and DNA-binding protein identification. Nanni and Lumini [3] proposed to combine the feature extraction method based on grouped weight with a set of amino acid alphabets obtained by Genetic Algorithm to produce features fed into SVMs for identifying DNA-binding proteins. Kumar et al. [4] developed a predictor called DNAbinder. It is the first study in which the combination of evolutionary information in form of PSSM profiles with SVMs has been used successfully for DNA-binding protein identification. Nanni and Lumini [3] proposed a parallel fusion between a SVM classifier trained with the features extracted from the gene ontology database and a 1-nearest neighbor classifier trained using the dipeptide composition of the sequence. Bhardwaj et al. [5] used SVMs as the classifier and information derived from characteristics (surface and overall composition, overall charge, and positive potential patches on the protein surface) as features to develop a predictor for DNA-binding proteins. Fang et al. [6] encoded a protein sequence into a feature vector by autocross-covariance transform, pseudo amino acid composition, and dipeptide composition, respectively, and also the different combinations of the three encoded methods, then fed them into a SVM classifier for DNA-binding protein identification. Bhardwaj and Lu [7] applied three steps to tackle the problem, including the development of an automated approach for fast and reliable recognition of DNA-binding sites, improving the prediction by distance-dependent refinement, and using these predictions to identify DNA-binding proteins.

Random Tree based methods were also commonly used. Kumar et al. [8] proposed a random forest method predictor called DNA-Prot to identify DNA binding proteins from protein sequence which used the fusion of sequence information and structure information as features. Nimrod et al. [9] presented a random forests classifier for identifying DNA-binding proteins with known information, such as electrostatic potential, cluster-based amino acid conservation patterns, and the secondary structure content of the patches, as well as the whole protein. Later they developed a web server called iDBPs which used the three-dimensional structure of a query protein to predict whether it binds DNA [10].

Up to present, several predictors applying ANN have been proposed. Stawiski et al. [11] presented an automated approach based on characterizing the structural and sequence properties of large, positively charged electrostatic patches on DNA-binding protein surfaces and used ANN as classifier. Keil et al. [12] introduced an algorithm which realized on the basis of a neural network strategy and the segmentation of the molecular surface into overlapping patches. Ahmad and Sarai et al. [13] demonstrated that Net charge, electric dipole moment, and quadrupole moment are important for DNA-binding protein identification. Patel et al. [14] implemented an approach for predicting the DNA-binding proteins from its amino acid sequence using ANN. Furthermore, they also tried implementing a two-layered artificial neural network for the same problem [15]. Molparia et al. [16] developed a method for predicting recognition helices for C2H2 zinc fingers that bind to specific target DNA sites based on ANN and constructed a web server called ZIF-Predict.

In addition to the aforementioned predictors, there are also other studies that made DNA-binding protein identification. For example, Neumann et al. [17] and Cai et al. [18] used boosted decision trees and nearest neighbor as classifier, respectively. Shanahan et al. [19] showed that a protein sequence of known structure and unknown function can be identified as a DNA-binding protein by employing structure features. Ahmad et al. [20] demonstrated that net charge, net dipole moment, and quadrupole moment are important features in this field. Nordhoff et al. [21] used mass spectrometry for DNA-binding protein identification.

Many efforts have been made for DNA-binding protein identification and many predictors have been proposed. However, most of predictors applied only one classifier. Otherwise, the number of newly discovered protein entries has been increasing extremely fast. In 1986, the number of protein entries in the Swiss-Prot [22] is only 3,939, but the UniProtKB/Swiss-Prot (released 2013_12 on December 11, 2013) has increased to 541,954 protein sequence, and most of them are non-DNA-binding proteins, meaning that the negative samples can be obtained easily for DNA-binding protein identification. Therefore, in this study, we attempted to adopt ensemble learning to perform DNA-binding protein identification and expand the benchmark dataset with negative samples to further improve its predictive performance.

## 2. Methods

As shown by a series of recent publications [23–35] and summarized in a comprehensive review [36], to develop a useful statistical prediction method or model for a biological system, one needs to engage the following procedures: (i) construct or select a valid benchmark dataset to train and test the predictor; (ii) formulate the samples with an effective mathematical expression that can truly reflect their intrinsic correlation with the target to be predicted; (iii) introduce or develop a powerful algorithm (or engine) to operate the prediction; (iv) properly perform cross-validation tests to objectively evaluate the anticipated accuracy of the predictor; (v) establish a user-friendly web-server for the predictor that is accessible to the public. Below, we describe our proposed method that followed such a general procedure.

### 2.1. Data

In this study, four datasets are used, including benchmark dataset, expanded benchmark dataset, independent dataset1 and independent dataset2, where benchmark dataset and expanded benchmark dataset were used as training dataset while independent dataset1 and independent dataset2 were used as two independent testing datasets.

#### 2.1.1. Benchmark Dataset

The benchmark dataset is used to train enDNA-Prot. It can be expressed as

(1) $$\begin{array}{c} S=S^{+}\cup S^{-}, \end{array}$$

where subset *S* ^+^ contains 146 DNA-binding proteins and subset *S* ^−^ contains 250 non-DNA-binding proteins, while the symbol ∪ represents the “union” in the set theory. Both the two subsets have a pairwise sequence identity cutoff of 25%. The DNA-binding proteins and non-DNA-binding proteins were obtained from the work of Kumar et al. [4] and Stawiski et al. [11]. A complete list of all the codes and sequence for the benchmark dataset can be found in Supplementary Material S1, available online at http://dx.doi.org/10.1155/2014/294279.

#### 2.1.2. Expanded Benchmark Dataset

In order to analyze the influence of the number of negative samples in benchmark dataset on the performance of enDNA-Prot, we constructed an expanded benchmark dataset based on benchmark dataset by adding sufficient number of non-DNA-binding proteins. It can be denoted as follows:

(2) $$\begin{array}{c} S=S_{e}^{+}\cup S_{e}^{-}, \end{array}$$

where *S*_*e*_^+^ and *S*_*e*_^−^ denote the set of DNA-binding proteins and non-DNA-binding proteins, respectively. *S*_*e*_^+^ is represented as *S*_*e*_^+^ = *S* ^+^, meaning that *S*_*e*_ contains the same positive samples as *S*. And *S*_*e*_^−^ can be calculated as *S*_*e*_^−^ = *S* ^−^ ∪ *S*_*a*_, which means that the set of negative samples of *S*_*e*_ is constructed by combining all the negative samples from *S* and the samples from another set *S*_*a*_, where *S*_*a*_ is a set of non-DNA-binding proteins obtained by adopting following processing procedure. At first, randomly extract a number of non-DNA-binding protein sequences from the latest release of PDB (Protein Data Bank release: December 2013) [37] with pairwise sequence identity cutoff of 25%. Next, remove all the sequences having ≥25% pairwise sequence identity with any sequence from benchmark dataset CD-HIT program [38]. Thus *S*_*e*_ contains 146 DNA-binding proteins and 2125 non-DNA-binding proteins. A complete list of all the codes and sequence for the expanded benchmark dataset can be found in Supplementary Material S2.

#### 2.1.3. Independent Dataset1

Independent dataset1 was obtained from Wang and Brown [39] which can be formulated as

(3) $$\begin{array}{c} S_{\text{Ind}1}^{}=S_{\text{Ind}1}^{+}\cup S_{\text{Ind}1}^{-}, \end{array}$$

where subsets *S*_Ind1_^+^ and *S*_Ind1_^−^ originally contain 92 DNA-binding proteins obtained from Protein Data Bank [37] and 100 non-DNA-binding protein entries obtained from Swiss-Prot database [40], respectively. And both subsets *S*_Ind1_^+^ and *S*_Ind1_^−^ have a pairwise sequence identity cutoff of 25%. Moreover, in order to avoid overestimating the current method, any sequence in the two subsets that has ≥40% pairwise sequence identity to any sequence in benchmark dataset or expanded benchmark dataset was removed using CD-HIT program [38]. Thus subsets *S*_Ind1_^+^ and *S*_Ind1_^−^ consist of 82 DNA-binding proteins and 100 non-DNA-binding proteins. A complete list of all the codes and sequences for the independent dataset1 can be found in Supplementary Material S3.

#### 2.1.4. Independent Dataset2

Independent dataset2 was constructed by first collected 823 DNA-binding domains and 823 non-DNA-binding domains from the work of Kumar et al. [8], in which the 823 DNA-binding domains were extracted from Pfam dataset [41] with keywords of “DNA-binding domain” and pairwise sequence identity cutoff of 25% while the 823 non-DNA-binding domains were randomly selected from it with the same pairwise sequence identity cutoff. And then remove the sequences that have ≥40% pairwise sequence identity to any sequence from benchmark dataset or expanded benchmark dataset using CD-HIT program [38] to avoid overestimating the current method. Finally, the independent dataset2 can be formulated as

(4) $$\begin{array}{c} S_{\text{Ind}2}^{}=S_{\text{Ind}2}^{+}\cup S_{\text{Ind}2}^{-}, \end{array}$$

where subset *S*_Ind2_^+^ contains 770 DNA-binding proteins and subset *S*_Ind2_^−^ contains 815 non-DNA-binding proteins. The summarization of the four datasets is given in Table 1. A complete list of all the codes and sequence for the independent dataset2 can be found in Supplementary Material S4. The four Supplementary Material files can be downloaded from http://bioinformatics.hitsz.edu.cn/Ensemble-DNA-Prot/download.jsp.

### 2.2. Features Extraction

A step that converts a sequence into a feature vector should be conducted, which dramatically affects the predictive performance. Inspired by the work of Cai et al. [42] and the study of Lin et al. [43], our present feature vector concluded the composition, distribution, and physicochemical properties of the amino acids in a sequence. Given that the respective occurrences of the 20 standard amino acids were represented as *o*_1_, *o*_2_, *o*_3_,…, *o*_20_, the composition part of present feature vector was calculated as

(5) $$\begin{array}{c} \left(f_{1},f_{2},\ldots ,f_{20}\right)=\left(\frac{o_{1}}{L},\frac{o_{2}}{L},\ldots ,\frac{o_{20}}{L}\right), \end{array}$$

where *L* denotes the sequence length.

With the exception of the effect of the composition, the properties including content (*C*), distribution (*D*), and dipeptide composition (DI) contributed to the predictive performance. First the 20 standard amino acids were divided into three groups based on each physicochemical property, which were listed in Table 2. In this section, hydrophobicity (*H*) was taken as an example to calculate these three properties. For a sequence, the amino acids were distributed to three groups according to their *H* property and the respective size of the three groups is calculated as *CH*_1_, *CH*_2_, and *CH*_3_. So the content for *H* was denoted as

(6) $$\begin{array}{c} \left(f_{21},f_{22},f_{23}\right)=\left(\frac{CH_{1}}{L},\frac{CH_{2}}{L},\frac{CH_{3}}{L}\right), \end{array}$$

*DH*_*ij*_(*i* = 1,2, 3; *j* = 1,2, 3,4, 5) are used to measure the respective location of the first (*j* = 1), 25 (*j* = 2), 50 (*j* = 3), 75 (*j* = 4), and 100% (*j* = 5) of amino acids with property *i*. Then the distribution for *H* was defined as

(7) (f24,…,f28;f29,…,f33;f34,…,f38)  =(DH11L,…,DH15L;DH21L,…,    DH25L;DH31L,…,DH35L).

To our knowledge, there are (*L* − 1) dipeptides in a sequence with length *L*. The parameters DI*H*_1_, DI*H*_2_, and DI*H*_3_ are used to count the respective number of the three types of dipeptides that contained two amino acids from different groups. Then the dipeptide composition for *H* was calculated as

(8) $$\begin{array}{c} \left(f_{39},f_{40},f_{41}\right)=\left(\frac{\text{DI}H_{1}}{L},\frac{\text{DI}H_{2}}{L},\frac{\text{DI}H_{3}}{L}\right). \end{array}$$

A feature vector with dimension 21 was calculated for each physicochemical property. We finally get a feature vector with dimension 188 after all properties were calculated.

### 2.3. Ensemble Classifier

#### 2.3.1. Definition

Ensemble learning is a machine learning method, in which multileaners are applied to tackle a same problem. While ordinary classifiers usually try to learn one hypothesis from training data, ensemble learning firstly learns a set of hypotheses and then combines them into an ensemble classifier. There are two kinds of ensemble classifiers. One kind constructs a set of base learners called homogeneous base learners with a single base learning algorithm; the other kind produces base learners by adopting multiple learning algorithms, which are called heterogeneous learners.

In this regard, three ensemble methods are usually adopted including Boosting [44, 45], Bagging [46], and Stacking [47]. Bagging trains a set of base learners each from a different dataset with the same size as the training dataset obtained by subsampling the training dataset with replacement. Stacking is implemented by first generating a number of first-level individual learners from the training dataset with different learning algorithms and then combining them by adopting a second-level learner called metalearner [47]. Boosting is a typical ensemble method and often used to train base learners. It has many effective variants and its representative algorithm is AdaBoost [45]. Due to the fact that the current benchmark dataset contains sufficient number of negative samples and a small amount of positive samples, we proposed an improved AdaBoost called Unbalanced-AdaBoost to make the best of the negative samples.

#### 2.3.2. Constructing the enDNA-Prot

**Figure 1:**
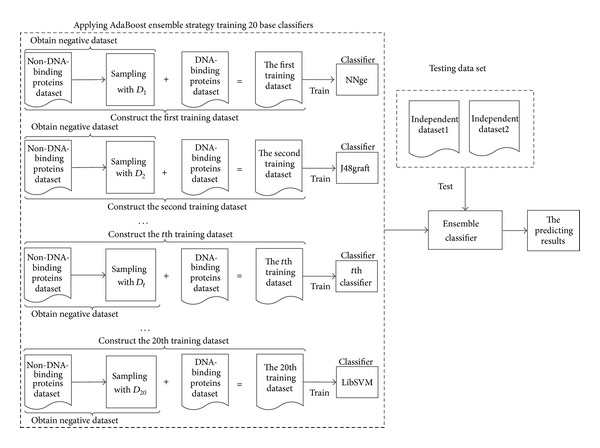
The frame diagram of enDNA-Prot.

The flowchart of enDNA-Prot is shown in Figure 1. From it we can see that *T* base learners were firstly trained by adopting Unbalanced-AdaBoost and then combined into an ensemble classifier with weighted vote rule. Before introducing the details of Unbalanced-AdaBoost, we described some symbols. *X* and *Y* are the instance space and the class labels, respectively, and *Y* = {−1, +1}. A train dataset with *m* samples is represented as *S*_Train_ = {(*x*_1_, *y*_1_), (*x*_2_, *y*_2_),…, (*x*_*m*_, *y*_*m*_)}, where *x*_*i*_ ∈ *X* and *y*_*i*_ ∈ *Y*  (*i* = 1,…, *m*). Its positive samples subset and negative samples subset are *S*_Train_^+^ = {(*x*_1_, *y*_1_), (*x*_2_, *y*_2_),…, (*x*_*n*_, *y*_*n*_)} and *S*_Train_^−^ = {(*x*_1_, *y*_1_), (*x*_2_, *y*_2_),…, (*x*_*l*_, *y*_*l*_)}, respectively, where *n* + *l* = *m*. *W*_*t*_ is the weight distribution on *S*_*T*_^−^ at the *t*th learning round and its element *W*_*t*_(*i*) means the probability with which the corresponding sample (*x*_*i*_, *y*_*i*_) in *S*_Train_^−^ will be sampled by a weighted sampling process. The pseudocode of Unbalanced-AdaBoost is shown in Algorithm 1. At first, initialize a uniform weight distribution *W*_1_ for *S*_Train_^−^, meaning that all the samples in *S*_Train_^−^ have a same probability to be sampled. Next, train a base learner *h*_*t*_ : *X* → *Y*  (1 ≤ *t* ≤ *T*) on a dataset that contains all the positive samples and the negative samples sampled from *S*_Train_^−^ with weight distribution *W*_*t*_; then test it on the negative train dataset *S*_Train_^−^ and multiply the weights of the incorrectly classified negative samples by a factor, meaning an updated weight distribution *W*_*t*+1_ was produced based on *W*_*t*_. Finally, iterate the above process for *T* times; *T* base learners were produced and combined into an ensemble classifier by adopting weighted vote rule.

Unbalanced-AdaBoost made two adjustments over AdaBoost. At first, the base learners were trained on datasets generated by combining all the positive samples and same number of negative ones sampled from *S*_Train_^−^ with different weight distribution. Next, in order to prevent overfitting and make full use of the large number of negative samples, the weight increasing speed of the incorrectly classified negative samples in every round was controlled. For example, in the *t*th (1 ≤ *t* ≤ *T*) round of the Unbalanced-AdaBoost, the weight increasing factor of the incorrectly classified negative samples was set as follows:

(9) $$\begin{array}{c} f=\log_{\text{size}}\left(\text{size}+\frac{1-\varepsilon_{t}}{\varepsilon_{t}}\right), \end{array}$$

where *f* is the weight increasing factor of the incorrectly classified negative samples, *ε*_*t*_ denotes the error rate of base learner *h*_*t*_ on the negative train dataset *S*_Train_^−^, and size is the number of negative samples in *S*_Train_^−^.

Previous research [48] indicated that the diversity of the base classifiers facilitates further improvement. Accordingly we used four types of classifiers including classifiers based on tree, classifiers based on KNN, classifiers based on rule, and classifiers based on function. There are 20 classifier algorithms taken as base classifier algorithms in this study, namely, IB1, IB5, IB15, J48graft, JRip, J48, NNge, PART, RandomForest, RandomTree, REPTree, Ridor, SimpleCairt, SMO, and conjunctiveRule, DecisionStump, DecisionTable, BFTree, ZeroR, and LibSVM. For more details about these learning algorithms, please refer to Weka [49].

## 3. Experiments

To evaluate the predictive performance of enDNA-Prot, we conducted a series of experiments. Firstly, we test the predictive performance of enDNA-Prot trained with benchmark dataset on the two independent datasets. Then we discussed the influence of the number of negative samples in benchmark dataset on the performance of enDNA-Prot.

### 3.1. Evaluation Metrics

Sensitivity (SE), specificity (SP), accuracy (ACC), Matthew's correlation coefficient (MCC) value, and F1_Measure (F1_M) are the top five commonly used evaluation metrics in this regard. In order to evaluate the enDNA-Prot objectively and without bias, they are adopted as the metrics of our study. Their computational formulae are written as follows:

(10) $$\begin{array}{c} \text{SE}=\frac{\text{TP}}{\text{TP}+\text{FN}},\\ \text{SP}=\frac{\text{TN}}{\text{TN}+\text{FP}},\\ \text{ACC}=\frac{\text{TP}+\text{TN}}{\text{TP}+\text{FP}+\text{TN}+\text{FN}},\\ \text{MCC}=\frac{\text{TP}*\text{TN}-\text{FP}*\text{FN}}{\sqrt{\left(\text{TP}+\text{FN}\right)\left(\text{TP}+\text{FP}\right)\left(\text{TN}+\text{FP}\right)\left(\text{TN}+\text{FN}\right)}},\\ \text{F}1\_\text{M}=\frac{2*\text{P}*\text{R}}{\text{P}+\text{R}},\\ \text{P}=\frac{\text{TP}}{\text{TP}+\text{FP}},\\ \text{R}=\frac{\text{TP}}{\text{TP}+\text{FN}}, \end{array}$$

where TP refers to the number of positive samples that are classified correctly, FP denotes the number of negative samples that are classified as positive sample, TN denotes the number of negative samples that are classified correctly, and FN denotes that number of positive samples that are classified as negative samples, while P and R refer to Precision value and Recall value, respectively.

### 3.2. Predictive Performance

In this experiment, the enDNA-Prot is trained with the benchmark dataset and then tested on the two independent datasets. Furthermore, its predictive performance was compared with some state-of-the-art methods, including DNAbinder [4], DNA-Port [8], and iDNA-Prot [50]. DNAbinder firstly extracts evolutionary information in form of PSSM from the corresponding protein sequence and then feeds it into SVMs for identifying DNA-binding proteins. It proposed three ways to encode the evolutionary information from PSSM. One is to encode the evolutionary information into a feature vector of 21 dimensions called PSSM-21 and its element is simple composition of occurrence of each type of amino acids, calculated by summing over each column (residual position) of PSSM. The second way is to encode a sequence into a feature vector with 420 dimensions called PSSM-420, of which the element is composition of occurrences of each type of amino acid corresponding to each type of amino acids in protein sequence, meaning that it has 20 values instead of one for each column. The last one is called PSSM-400 which is similar to PSSM-420 except dummy residue “X” is ignored. As the sequences in our dataset almost have no dummy residue “X,” we will not refer to the PSSM-420 based DNAbinder. DNA-Prot is a predictor that encodes a sequence by using several types of information including sequence information and structure information, such as amino acid composition, dipeptide composition, amino acid composition in the secondary structures, and secondary structures itself. The Random Forest is adopted by it as a learning algorithm. iDNA-Prot represents each sequence as pseudo amino acid composition by applied grey model [51]. All these methods are in-house implemented and tested on the same datasets to give an unbiased comparison with the present method enDNA-Prot.

The results of different methods on independent dataset1 are given in Table 3. From this table we can see that enDNA-Prot and DNA-Prot achieved highly comparable performance and outperform other methods by 4.51–7.15% in terms of ACC and 0.08–0.15 in terms of MCC.

In order to objectively evaluate the performance of our method and fairly compare it with other methods, the present method was further evaluated on another independent dataset. The results on independent dataset2 are given in Table 4. From Table 4 we can see that among all the methods enDNA-Prot achieves the best performance. It outperforms other methods with improvement in the range of 3.97–9.52% in terms of ACC and in the range of 0.08–0.19 in terms of MCC, which indicated that enDNA-Prot is an effective method for DNA-binding protein identification.

### 3.3. Impact of the Number of Negative Samples

**Figure 2:**
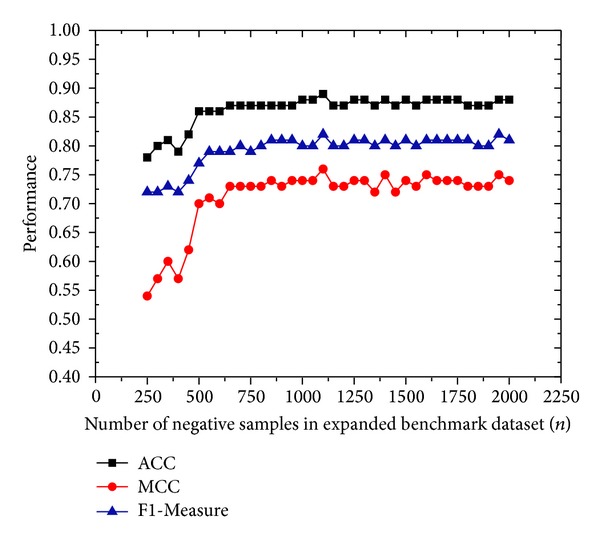
The influence of *n* on performance.

To analyze the influence of the number of negative samples in benchmark dataset on the performance of enDNA-Prot, a training dataset and a validation dataset are constructed based on *S*_*e*_. At first, extract the former 73 DNA-binding proteins and 125 non-DNA-binding proteins from *S*_*e*_ to create a validation dataset. Next, collect the remaining 73 DNA-binding proteins and *n* different non-DNA-binding proteins from the remaining non-DNA-binding proteins to compose a training dataset, where the *n* is a variable ranging from 250 to 2000. By changing the value of *n*, we can obtain number of different training datasets. Through a validation dataset and multiple training datasets, the compact of the number of negative samples in training dataset on the performance of current method is achieved, which are given in Figure 2. As shown in this figure, the performance of enDNA-Prot increases to a maximum value as the value of *n* increases from 250 to 1100 and then tends to be steady when *n* is larger than 1100. It indicated that 1100 negative samples can render enDNA-Prot to achieve the best predictive performance. Therefore, for further analysis, we create a dataset called expanded benchmark dataset1100 with all the positive samples and the former 1100 negative sample from expanded benchmark dataset, which will be employed as another training dataset for further evaluating the present method.

The predictive performance of enDNA-Prot trained with different training datasets for the two independent datasets is given in Table 5. As shown from this table, the enDNA-Prot training on expanded benchmark dataset1100 outperforms the one training on benchmark dataset with improvement in the range of 1.77–4.94% in terms of ACC and 0.03–0.09 in terms of MCC. It indicated that expanding the training dataset with negative samples can indeed improve the predictive performance of enDNA-Prot.

In order to further analyze the advantage of current method over other methods, the expanded benchmark dataset1100 is also used as training dataset to evaluate the performance of the three methods mentioned above and our method. The results of the proposed method and other methods on independent dataset1 are given in Table 6. From this table we can see that enDNA-Prot achieved 89.56% in terms of ACC and 0.79 in terms of MCC, which outperforms other methods with improvement in the range of 11.11–16.63% in terms of ACC and 0.18–0.27 in terms MCC. The results on independent dataset2 are given in Table 7, from which we can see that enDNA-Prot achieved 83.48% in terms of ACC and 0.67 in terms of MCC, which outperforms other methods with improvement in the range of 2.83–8.37% in ACC and 0.02–0.16 in MCC. It indicated that enDNA-Prot can perform better than other existing methods on unbalanced dataset.

### 3.4. Web-Server Guide

For the convenience of the vast majority of experimental scientists to use enDNA-Prot, a detailed step-by-step guide on how to use the web-server of enDNA-Prot is provided as follows.

*Step* *1.* Open the web-server at http://bioinformatics.hitsz.edu.cn/Ensemble-DNA-Prot/ and you will see the home page of enDNA-Prot on your screen. Click on the “How to use” button to see a brief introduction about the predictor.

*Step* *2.* Click on the “Server” button and you will see the server page of enDNA-Prot on your screen. Either type or copy/paste the query protein sequence of FASTA format into the input box at the center of the server page. For more detailed information about the format of query protein sequence, please click on the “fasta format” above the input box. Note that number of query protein sequence inputted should be no more than 50. Then click on the Submit button.

*Step* *3.* Input your information into the corresponding input box, such as your name and your email address, and click on the Submit button. Then you will get the results whether it is a DNA-binding protein or non-DNA-binding protein of your inputted query protein sequences.

*Step* *4.* If you need to get the source code of enDNA-Prot and the dataset employed in this paper, you can click on Download in the home page.

*Step* *5.* If you have any problem regarding the predictor enDNA-Prot or using the web-server of enDNA-Prot, please click on Contact in home page to get our email address.

## 4. Conclusions

In the field of DNA-binding protein identification, many predictors have been proposed, but most of them focus on only one classification algorithm and cannot make full use of the large number of negative samples to improve its performance. Accordingly, we proposed a new predictor called enDNA-Prot which firstly encoded each protein sequence into a feature vector with dimension of 188 with features only extracted from protein sequence and then fed into an ensemble classifier constructed with 20 different machine learning classifiers. The experimental results showed that the proposed method outperforms most existing state-of-the-art methods, indicating that enDNA-Prot is an effective method for DNA-binding protein identification for both balanced dataset and unbalanced dataset. Furthermore, it also showed that the performance of enDNA-Prot trained with expanded benchmark dataset is better than the one trained with benchmark dataset, which indicates that expanding training dataset with negative samples can improve its predicative performance.

## Supplementary Material

Supplementary Material S1 lists all the codes and sequences for the benchmark dataset. It contains 396 proteins, classified into 146 DNA-binding proteins and 250 non DNA-binding proteins.
Supplementary Material S2 lists all the codes and sequences for the expanded benchmark dataset. It contains 2271 proteins, classified into 146 DNA-binding proteins and 2125 non DNA-binding proteins.
Supplementary Material S3 lists all the codes and sequences for the independent dataset1. It contains 182 proteins, classified into 82 DNA-binding proteins and 100 non DNA-binding proteins.
Supplementary Material S4 lists all the codes and sequences for the independent dataset2. It contains 1585 proteins, classified into 770 DNA-binding proteins and 815 non DNA-binding proteins.

## Figures and Tables

**Algorithm 1:**
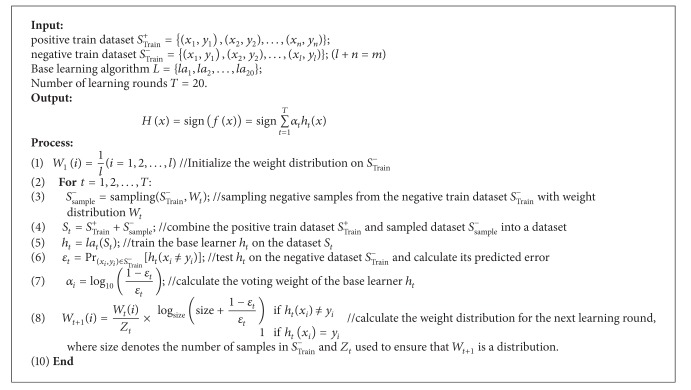
The pseudocode of Unbalanced-AdaBoost.

**Table 1:** The summarization of datasets.

Dataset	DNA-binding proteins	Non-DNA-binding proteins
Benchmark dataset	146	250
Expanded benchmark dataset	146	2125
Independent dataset1	82	100
Independent dataset2	770	815

**Table 2:** The three groups of amino acids for each physicochemical property.

Physicochemical property	The 1st group	The 2nd group	The 3rd group
Hydrophobicity	RKEDQN	GASTPHT	CVLIMFW
Normalized van der Waals volume	GASCTPD	NVEQIL	MHKFRYW
Polarity	LIFWCMVY	PATGS	HQRKNED
Polarizability	GASDT	CPNVEQIL	KMHFRYW
Charge	KR	ANCQGHILMFPSTWYV	DE
Surface tension	GQDNAHR	KTSEC	ILMFPWYV
Secondary structure	EALMQKRH	VIYCWFT	GNPSD
Solvent accessibility	ALFCGIVW	RKQEND	MPSTHY

**Table 3:** Performance for independent dataset1 (trained on benchmark dataset).

Method	ACC (%)	MCC	SE (%)	SP (%)	F1-M (%)
DNAbinder(P21)	79.00	0.61	54.87	98.08	70.31
DNAbinder(P400)	80.11	0.62	58.53	97.97	72.73
DNA-Prot	84.61	0.69	73.17	94.00	81.08
iDNA-Prot	77.47	0.55	78.05	77.00	75.73
enDNA-Prot	84.62	0.70	73.18	94.00	84.62

P400 and P21 denote the two vectorization methods PSSM-400 based DNAbinder and PSSM-21 based DNAbinder, respectively.

**Table 4:** Performance for independent datset2 (trained on benchmark dataset).

Method	ACC (%)	MCC	SE (%)	SP (%)	F1-M (%)
DNAbinder(P21)	76.64	0.55	86.18	67.57	74.89
DNAbinder(P400)	76.38	0.52	72.35	80.19	75.23
DNA-Prot	77.74	0.56	85.19	70.71	78.79
iDNA-Prot	72.19	0.45	77.01	67.64	72.89
enDNA-Prot	81.71	0.64	84.55	79.05	81.71

P400 and P21 denote the two vectorization methods PSSM-400 based DNAbinder and PSSM-21 based DNAbinder, respectively.

**Table 5:** Performance of enDNA-Prot trained on different dataset.

Testing dataset	Training dataset	ACC (%)	MCC	SE (%)	SP (%)	F1-M (%)
ID1	BD	84.62	0.70	73.18	94.00	84.62
	EBD1100	89.56	0.79	80.48	97.00	87.42

ID2	BD	81.71	0.64	84.55	79.05	81.71
	EBD1100	83.48	0.67	84.29	82.72	83.21

ID1 and ID2 denote the independent dataset1 and independent dataset2, respectively; BD and EBD1100 denote the benchmark dataset and expanded benchmark dataset1100, respectively.

**Table 6:** Performance for independent dataset1 (trained on expanded benchmark dataset1100).

Method	ACC (%)	MCC	SE (%)	SP (%)	F1-M (%)
DNAbinder(P21)	72.93	0.52	42.24	100	57.39
DNAbinder(P400)	78.45	0.61	52.44	100	68.80
DNA-Prot	76.37	0.58	47.56	100	64.46
iDNA-Prot	76.92	0.58	50.00	99.00	66.13
enDNA-Prot	89.56	0.79	80.48	97.00	87.42

P400 and P21 denote the two vectorization methods PSSM-400 based DNAbinder and PSSM-21 based DNAbinder, respectively.

**Table 7:** Performance for independent datase2 (trained on expanded benchmark dataset1100).

Method	ACC (%)	MCC	SE (%)	SP (%)	F1-M (%)
DNAbinder(P21)	75.11	0.51	64.41	85.27	71.59
DNAbinder(P400)	81.65	0.65	67.14	95.42	78.09
DNA-Prot	79.07	0.60	65.32	92.03	75.19
iDNA-Prot	75.60	0.54	57.01	93.14	69.41
enDNA-Prot	83.48	0.67	84.29	82.72	83.21

P400 and P21 denote the two vectorization methods PSSM-400 based DNAbinder and PSSM-21 based DNAbinder, respectively.
