# Supplementary material for: enDNA-Prot: Identification of DNA-Binding Proteins by Applying Ensemble Learning
Source: Biomed Res Int. 2014 May 26;2014:294279. doi: 10.1155/2014/294279 (PMC4058174; doi:10.1155/2014/294279)
Supplement: Supplementary file 1 — Supplementary Material S1 lists all the codes and sequences for the benchmark dataset. It contains 396 proteins, classified into 146 DNA-binding proteins and 250 non DNA-binding proteins. Supplementary Material S2 lists all the codes and sequences for the expanded benchmark dataset. It contains 2271 proteins, classified into 146 DNA-binding proteins and 2125 non DNA-binding proteins. Supplementary Material S3 lists all the codes and sequences for the independent dataset1. It contains 182 proteins, classified into 82 DNA-binding proteins and 100 non DNA-binding proteins. Supplementary Material S4 lists all the codes and sequences for the independent dataset2. It contains 1585 proteins, classified into 770 DNA-binding proteins and 815 non DNA-binding proteins. [file 294279.f1.zip › 294279.f1/S1.docx]

Online Supporting Information S1. The 396 protein sequences and its identifiers in benchmark dataset classified 146 DNA-binding proteins and 250 non DNA-binding proteins are shown here.

| (1). DNA-binding proteins |
| --- |

>D1A04A2

EPATILLIDDHPMLRTGVKQLISMAPDITVVGEASNGEQGIELAESLDPDLILLDLNMPGMNGLETLDKLREKSLSGRIVVFSVSNHEEDVVTALKRGADGYLLKDMEPEDLLKALHQAAAGEMVLSEALTPVLAASL

>D1A0AA_

MKRESHKHAEQARRNRLAVALHELASLIPAEWKQQNVSAAPSKATTVEAACRYIRHLQQNGST

>D1A1IA1

RPYACPVESCDRRFSRSADLTRHIRIHTG

>D1A3QA2

GPYLVIVEQPKQRGFRFRYGCEGPSHGGLPGASSEKGRKTYPTVKICNYEGPAKIEVDLVTHSDPPRAHAHSLVGKQCSELGICAVSVGPKDMTAQFNNLGVLHVTKKNMMGTMIQKLQRQRLRSRPQGLTEAEQRELEQEAKELKKVMDLSIVRLRFSAFLRSLPLKPVISQPIHDSKSPGAS

>D1A73A_

ALTNAQILAVIDSWEETVGQFPVITHHVPLGGGLQGTLHCYEIPLAAPYGVGFAKNGPTRWQYKRTINQVVHRWGSHTVPFLLEPDNINGKTCTASHLCHNTRCHNPLHLCWESLDDNKGRNWCPGPNGGCVHAVVCLRQGPLYGPGATVAGPQQRGSHFVV

>D1AISB1

NLAFALSELDRITAQLKLPRHVEEEAARLYREAVRKGLIRGRSIESVMAACVYAACRLLKVPRTLDEIADIARVDKKEIGRSYRFIARNLNLTPKKLF

>D1AISB2

VKPTDYVNKFADELGLSEKVRRRAIEILDEAYKRGLTSGKSPAGLVAAALYIASLLEGEKRTQREVAEVARVTEVTVRNRYKELVEKLKIKVPIA

>D1AZPA_

MVKVKFKYKGEEKEVDTSKIKKVWRVGKMVSFTYDDNGKTGRGAVSEKDAPKELLDMLARAEREKK

>D1B3TA_

KGGWFGKHRGQGGSNPKFENIAEGLRALLARSHVERTTDEGTWVAGVFVYGGSKTSLYNLRRGTALAIPQCRLTPLSRLPFGMAPGPGPQPGPLRESIVCYFMVFLQTHIFAEVLKDAIKDLVMTKPAPTCNIRVTVCSFDDGVDLP

>D1BG1A1

VVTEKQQMLEQHLQDVRKRVQDLEQKMKVVENLQDDFDFNYKTLKSQGDMQDLNGNNQSVTRQKMQQLEQMLTALDQMRRSIVSELAGLLSAMEYVQKTLTDEELADWKRRQQIACIGGPPNICLDRLENWITSLAESQLQTRQQIKKLEELQQKVSYKGDPIVQHRPMLEERIVELFRNLMKSAF

>D1BG1A2

VVERQPCMPMHPDRPLVIKTGVQFTTKVRLLVKFPELNYQLKIKVCIDKDSGDVAALRGSRKFNILGTNTKVMNMEESNNGSLSAEFKHLTLREQRCGNGGRANCDASLIVTEELHLITFETEVYHQGLKIDLETHSLPVVVISNICQMPNAWASILWYNMLTNNPKNVNFFTKPPIGTWDQVAEVLSWQFSSTTKRGLSIEQLTTLAEKLLGPGVNYSGCQITWAKFCKENMAGKGFSFWVWLDNIIDLVKKY

>D1BG1A3

ILALWNEGYIMGFISKERERAILSTKPPGTFLLRFSESSKEGGVTFTWVEKDISGSTQIQSVEPYTKQQLNNMSFAEIIMGYKIMDATNILVSPLVYLYPDIPKEEAFGKYCRPESQEHPEADPGSAAPYLKTKFICVTPF

>D1BL0A1

DAITIHSILDWIEDNLESPLSLEKVSERSGYSKWHLQRMFKKETGHSLGQYIRS

>D1BL0A2

RKMTEIAQKLKESNEPILYLAERYGFESQQTLTRTFKNYFDVPPHKYRMTNMQGESRFLHPL

>D1CDWA2

NMVGSCDVKFPIRLEGLVLTHQQFSSYEPELFPGLIYRMIKPRIVLLIFVSGKVVLTGAKVRAEIYEAFENIYPILKGFRK

>D1CF7A_

SRHEKSLGLLTTKFVSLLQEAKDGVLDLKLAADTLAVRQKRRIYDITNVLEGIGLIEKKSKNSIQWK

>D1CF7B_

GKGLRHFSMKVCEKVQRKGTTSYNEVADELVSEFTNSNNHLAADSAYDQKNIRRRVYDALNVLMAMNIISKEKKEIKWIGLP

>D1CKQA_

SQGVIGIFGDYAKAHDLAVGEVSKLVKKALSNEYPQLSFRYRDSIKKTEINEALKKIDPDLGGTLFVSNSSIKPDGGIVEVKDDYGEWRVVLVAEAKHQGKDIINIRNGLLVGKRGDQDLMAAGNAIERSHKNISEIANFMLSESHFPYVLFLEGSNFLTENISITRPDGRVVNLEYNSGILNRLDRLTAANYGMPINSNLCINKFVNHKDKSIMLQAASIYTQGDGREWDSKIMFEIMFDISTTSLRVLGRDLFEQLTSK

>D1D02A_

LSGRLNWQALAGLKASGAEQNLYNVFNAVFEGTKYVLYEKPKHLKNLYAQVVLPDDVIKEIFNPLIDLSTTQWGVSPAFAIENTETHKILFGEIKRQDGWVEGKDPSAGRGNAHERSCKLFTPGLLKAYRTIGGINDEEILPFWVVFEGDITRDPKRVREITFWYDHYQDNYFMWRPNESGEKLVQHFNEKLKKYLD

>D1D5YA3

EFTMPEHKFVTLEDTPLIGVTQSYSCSLEQISDFRHEMRYQFWHDFLGNAPTIPPVLYGLNETRPSQDKDDEQEVFYTTALAQDQADGYVLTGHPVMLQGGEYVMFTYEGLGTGVQEFILTVYGTCMPMLNLTRRKGQDIERYYPAEDAKAGDRPINLRCELLIPIRRKLAAA

>D1D66A1

EQACDICRLKKLKCSKEKPKCAKCLKNNWECRYSPKTKRSP

>D1DC1A_

KPFENHLKSVDDLKTTYEEYRAGFIAFALEKNKRSTPYIERARALKVAASVAKTPKDLLYLEDIQDALLYASGISDKAKKFLTEDDKKESINNLIENFLEPAGEEFIDELIFRYLLFQGDSLGGTMRNIAGALAQQKLTRAIISALDIANIPYKWLDSRDKKYTNWMDKPEDDYELETFAKGISWTINGKHRTLMYNITVSLVKKNVDICLFNCEPEIYTPQKVHQQPEKYLLLGELKGGIDPAGADEHWKTANTALTRIRNKFSEKGLSPKTIFIGAAIEHSMAEEIWDQLQSGSLTNSANLTKTEQVGSLCRWIINI

>D1DCTA_

MNLISLFSGAGGLDLGFQKAGFRIICANEYDKSIWKTYESNHSAKLIKGDISKISSDEFPKCDGIIGGPPCQSWSEGGSLRGIDDPRGKLFYEYIRILKQKKPIFFLAENVKGMMAQRHNKAVQEFIQEFDNAGYDVHIILLNANDYGVAQDRKRVFYIGFRKELNINYLPPIPHLIKPTFKDVIWDLKDNPIPALDKNKTNGNKCIYPNHEYFIGSYSTIFMSRNRVRQWNEPAFTVQASGRQCQLHPQAPVMLKVSKNLNKFVEGKEHLYRRLTVRECARVQGFPDDFIFHYESLNDGYKMIGNAVPVNLAYEIAKTIKSAL

>D1DFMA_

MKIDITDYNHADEILNPQLWKEIEETLLKMPLHVKASDQASKVGSLIFDPVGTNQYIKDELVPKHWKNNIPIPKRFDFLGTDIDFGKRDTLVEVQFSNYPFLLNNTVRSELFHKSNMDIDEEGMKVAIIITKGHMFPASNSSLYYEQAQNQLNSLAEYNVFDVPIRLVGLIEDFETDIDIVSTTYADKRYSRTITKRDTVKGKVIDTNTPNTRRRKRGTIVTY

>D1DMUA_

MYNLHREKIFMSYNQNKQYLEDNPEIQEKIELYGLNLLNEVISDNEEEIRADYNEANFLHPFWMNYPPLDRGKMPKGDQIPWIEVGEKAVGSKLTRLVSQREDITVREIGLPTGPDERYLLTSPTIYSLTNGFTDSIMMFVDIKSVGPRDSDYDLVLSPNQVSGNGDWAQLEGGIQNNQQTIQGPRSSQIFLPTIPPLYILSDGTIAPVVHLFIKPIYAMRSLTKGDTGQSLYKIKLASVPNGLGLFCNPGYAFDSAYKFLFRPGKDDRTKSLLQKRVRVDLRVLDKIGPRVMTIDMDK

>D1DP7P_

TVQWLLDNYETAEGVSLPRSTLYNHYLLHSQEQKLEPVNAASFGKLIRSVFMGLRTRRLGTRGNSKYHYYGLRIKA

>D1DSZA_

PCFVCQDKSSGYHYGVSACEGCKGFFRRSIQKNMVYTCHRDKNCIINKVTRNRCQYCRLQKCFEVGMSKESVRND

>D1E3MA3

GTISDEALLQERQDNLLAAIWQDSKGFGYATLDISSGRFRLSEPADRETMAAELQRTNPAELLYAEDFAEMSLIEGRRGLRRRPLWEFEIDTARQQLNLQFGTRDLVGFGVENAPRGLCAAGCLLQYAKDTQRTTLPHIRSITMEREQDSIIM

>D1E3OC2

EEPSDLEELEQFAKTFKQRRIKLGFTQGDVGLAMGKLYGNDFSQTTISRFEALNLSFKNMSKLKPLLEKWLNDAE

>D1ECRA_

DLVDRLNTTFRQMEQELAIFAAHLEQHKLLVARVFSLPEVKKEDEHNPLNRIEVKQHLGNDAQSLALRHFRHLFIQQQSENRSSKAAVRLPGVLCYQVDNLSQAALVSHIQHINKLKTTFEHIVTVESELPTAARFEWVHRHLPGLITLNAYRTLTVLHDPATLRFGWANKHIIKNLHRDEVLAQLEKSLKSPRSVAPWTREEWQRKLEREYQDIAALPQNAKLKIKRPVKVQPIARVWYKGDQKQVQHACPTPLIALINRDNGAGVPDVGELLNYDADNVQHRYKPQAQPLRLIIPRLHLYVAD

>D1EGWA_

GRKKIQITRIMDERNRQVTFTKRKFGLMKKAYELSVLCDCEIALIIFNSSNKLFQYASTDMDKVLLKYTEY

>D1EWNA_

HLTRLGLEFFDQPAVPLARAFLGQVLVRRLPNGTELRGRIVETQAYLGPEDEAAHSRGGRQTPRNRGMFMKPGTLYVYIIYGMYFCMNISSQGDGACVLLRALEPLEGLETMRQLRSTLRKGTASRVLKDRELCSGPSKLCQALAINKSFDQRDLAQDEAVWLERGPLEPSEPAVVAAARVGVGHAGEWARKPLRFYVRGSPWVSVVDRVAEQD

>D1EWQA1

RGQDTLFSVLDETRTAPGRRLLQSWLRHPLLDRGPLEARLDRVEGFVREGALREGVRRLLYRLADLERLATRLELGRASPKDLGALRRSLQILPELRALLGEEVGLPDLSPLKEELEAALVEDPPLKVSEGGLIREGYDPDLDALRAAHREGVAYFLELEERERERTGIPTLKVGYNAVFGYYLEVTRPYYERVPKEYRPVQTLKDRQRYTLPEMKEKEREVYRLEALIRRREEEVFLEVRERAKRQAEALREAARILAELDVYAALAEVAVRYG

>D1EWQA2

YVRPRFGDRLQIRAGRHPVVERRTEFVPNDLEMAHELVLITGPNMAGKSTFLRQTALIALLAQVGSFVPAEEAHLPLFDGIYTRIGASDDLAGGKSTFMVEMEEVALILKEATENSLVLLDEVGRGTSSLDGVAIATAVAEALHERRAYTLFATHYFELTALGLPRLKNLHVAAREEAGGLVFYHQVLPGPASKSYGVEVAAMAGLPKEVVARARALLQAMAAR

>D1EWQA3

LLQESLLPREANYLAAIATGDGWGLAFLDVSTGEFKGTVLKSKSALYDELFRHRPAEVLLAPELLENGAFLDEFRKRFPVMLSEAPFEPEGEGPLALRRARGALLAYAQRTQGGALSLQPFRFYDPGAFMRLPEATLRALEVFEPL

>D1EWQA4

MEGMLKGEGPGPLPPLLQQYVELRDQYPDYLLLFQVGDFYECFGEDAERLARALGLVLTHKTSKDFTTPMAGIPLRAFEAYAERLLKMGFRLAVADQVEPAEEAEGLVRREVTQLLTPGT

>D1F44A1

SDEVRKNLMDMFRDRQAFSEHTWKMLLSVCRSWAAWCKLNNRKWFPAEPEDVRDYLLYLQARGLAVKTIQQHLGQLNMLHRRSGLPRPSDSNAVSLVMRRIRKENVDAGE

>D1F44A2

RAKQALAFERTDFDQVRSLMENSDRCQDIRNLAFLGIAYNTLLRIAEIARIRVKDISRTDGGRMLIHIGRTKTLVSTAGVEKALSLGVTKLVERWISVSGVADDPNNYLFCRVRKNGVAAPSATSQLSTRALEGIFEATHRLIYGAKDDSGQRYLAWSGHSARVGAARDMARAGVSIPEIMQAGGWTNVNIVMNFIRNLDSETGAMVRLLEDGD

>D1FIUA_

MQPLFTQERRIFHKKLLDGNILATNNRGVVSNADGSNTRSFNIAKGIADLLHSETVSERLPGQTSGNAFEAICSEFVQSAFEKLQHIRPGDWNVKQVGSRNRLEIARYQQYAHLTALAKAAEENPELAAALGSDYTITPDIIVTRNLIADAEINRNEFLVDENIATYASLRAGNGNMPLLHASISCKWTIRSDRAQNARSEGLNLVRNRKGRLPHIVVVTAEPTPSRISSIALGTGEIDCVYHFALYELEQILQSLNYEDALDLFYIMVNGKRLKDISDLPLDLAV

>D1FJLA_

KQRRSRTTFSASQLDELERAFERTQYPDIYTREELAQRTNLTEARIQVWFQNRRARLRKQHTSVS

>D1FLOA1

PQFDILCKTPPKVLVRQFVERFERPSGEKIALCAAELTYLCWMITHNGTAIKRATFMSYNTIISNSLSFDIVNKSLQFKYKTQKATILEASLKKLIPAWEFTIIPYYGQKHQSDITDIVSSLQLQFES

>D1FLOA2

KGNSHSKKMLKALLSEGESIWEITEKILNSFEYTSRFTKTKTLYQFLFLATFINCGRFSDIKNVDPKSFKLVQNKYLGVIIQCLVTETKTSVSRHIYFFSARGRIDPLVYLDEFLRNSEPVLKRVNRTGNSSSNKQEYQLLKDNLVRSYNKALKKNAPYSIFAIKNGPKSHIGRHLMTSFLSMKGLTELTNVVGNWSDKRASAVARTTYTHQITAIPDHYFALVSRYYAYDPISKEMIALKDETNPIEEWQHIEQLKGSAEGSIRYPAWNGIISQEVLDYLSSYINRRI

>D1G38A_

VETPPEVVDFMVSLAEAPRGGRVLEPACAHGPFLRAFREAHGTGYRFVGVEIDPKALDLPPWAEGILADFLLWEPGEAFDLILGNPPYGIVGEASKYPIHVFKAVKDLYKKAFSTWKGKYNLYGAFLEKAVRLLKPGGVLVFVVPATWLVLEDFALLREFLAREGKTSVYYLGEVFPQKKVSAVVIRFQKSGKGLSLWDTQESESGFTPILWAEYPHWEGEIIRFETEETRKLEISGMPLGDLFHIRFAARSPEFKKHPAVRKEPGPGLVPVLTGRNLKPGWVDYEKNHSGLWMPKERAKELRDFYATPHLVVAHTKGTRVVAAWDERAYPWREEFHLLPKEGVRLDPSSLVQWLNSEAMQKHVRTLYRDFVPHLTLRMLERLPVRREYGFHT

>D1G9ZA_

NTKYNKEFLLYLAGFVDGDGSIIAQIKPNQSYKFKHQLSLTFQVTQKTQRRWFLDKLVDEIGVGYVRDRGSVSDYILSEIKPLHNFLTQLQPFLKLKQKQANLVLKIIEQLPSAKESPDKFLEVCTWVDQIAALNDSKTRKTTSETVRAVLD

>D1GT0D_

DRVKRPMNAFMVWSRGQRRKMAQENPKMHNSEISKRLGAEWKLLSETEKRPFIDEAKRLRALHMKEHPDYKYRPRRKTKT

>D1H6FA_

DPKVHLEAKELWDQFHKRGTEMVITKSGRRMFPPFKVRCSGLDKKAKYILLMDIIAADDCRYKFHNSRWMVAGKADPEMPKRMYIHPDSPATGEQWMSKVVTFHKLKLTNNISDKHGFTILNSMHKYQPRFHIVRANDILKLPYSTFRTYLFPETEFIAVTAYQNDKITQLKIDNNPFAKGFRD

>D1H9DB_

PRVVPDQRSKFENEEFFRKLSRECEIKYTGFRDRPHEERQARFQNACRDGRSEIAFVATGTNLSLQFFPASWQGEQRQTPSREYVDLEREAGKVYLKAPMILNGVCVIWKGWIDLQRLDGMGCLEFDEERAQQE

>D1HCRA_

GRPRAINKHEQEQISRLLEKGHPRQQLAIIFGIGVSTLYRYFPASSIKKRMN

>D1HLVA1

MGPKRRQLTFREKSRIIQEVEENPDLRKGEIARRFNIPPSTLSTILKNKRAILASERKYGVASTCR

>D1HLVA2

KTNKLSPYDKLEGLLIAWFQQIRAAGLPVKGIILKEKALRIAEELGMDDFTASNGWLDRFRRRRS

>D1I3JA_

KFCKCGVRIQTSAYTCSKCRNRSGENNSFFNHKHSDITKSKISEKMKGKKPSNIKKISCDGVIFDCAADAARHFKISSGLVTYRVKSDKWNWFYIN

>D1I7DA_

MRLFIAEKPSLARAIADVLPKPHRKGDGFIECGNGQVVTWCIGHLLEQAQPDAYDSRYARWNLADLPIVPEKWQLQPRPSVTKQLNVIKRFLHEASEIVHAGDPDREGQLLVDEVLDYLQLAPEKRQQVQRCLINDLNPQAVERAIDRLRSNSEFVPLCVSALARARADWLYGINMTRAYTILGRNAGYQGVLSVGRVQTPVLGLVVRRDEEIENFVAKDFFEVKAHIVTPADERFTAIWQPSEACEPYQDEEGRLLHRPLAEHVVNRISGQPAIVTSYNDKRESESAPLPFSLSALQIEAAKRFGLSAQNVLDICQKLYETHKLITFPRSDCRYLPEEHFAGRHAVMNAISVHAPDLLPQPVVDPDIRNRCWDDKKVDAHHAIIPTARSSAINLTENEAKVYNLIARQYLMQFCPDAVFRKCVIELDIAKGKFVAKARFLAEAGWRTLLGSKERDEENDGTPLPVVAKGDELLCEKGEVVERQTQPPRHFTDATLLSAMTGIARFVQDKDLKKILRATDGLGTEATRAGIIELLFKRGFLTKKGRYIHSTDAGKALFHSLPEMATRPDMTAHWESVLTQISEKQCRYQDFMQPLVGTLYQLIDQAKRTPVRQFRGIVAP

>D1IC8A2

KELENLSPEEAAHQKAVVETLLQEDPWRVAKMVKSYLQQHNIPQREVVDTTGLNQSHLSQHLNKGTPMKTQKRAALYTWYVRKQREVAQQFTHA

>D1IGNA1

KASFTDEEDEFILDVVRKNPTRRTTHTLYDEISHYVPNHTGNSIRHRFRVYLSKRLEYVYEVDKFGKLVRDDDGNLIKTKVLPPSI

>D1IGNA2

KRKFSADEDYTLAIAVKKQFYRDLFQIDPDTGRSLITDEDTPTAIARRNMTMDPNHVPGSEPNFAAYRTQSRRGPIAREFFKHFAEEHAAHTENAWRDRFRKFLLAYGIDDYISYYEAEKAQNREPEPMKNLTNRPKRPGVPTPGNYNS

>D1J1VA_

VTIDNIQKTVAEYYKIKVADLLSKRRSRSVARPRQMAMALAKELTNHSLPEIGDAFGGRDHTTVLHACRKIEQLREESHDIKEDFSNLIRTLSS

>D1J3EA_

PLGSAMRELLLSDEYAEQKRAVNRFMLLLSTLYSLDAQAFAEATESLHGRTRVYFAADEQTLLKNGNQTKPKHVPGTPYWVITNTNTGRKCSMIEHIMQSMQFPAELIEKVCGTI

>D1J75A_

NLEQKILQVLSDDGGPVKIGQLVKKCQVPKKTLNQVLYRLKKEDRVSSPEPATWSIG

>D1JB7A1

YEYVELAKASLTSAQPQHFYAVVIDATFPYKTNQERYICSLKIVDPTLYLKQQKGAGDASDYATLVLYAKRFEDLPIIHRAGDIIRVHRATLRLYNGQRQFNANVFYSSSWALFSTDKRSVTQEINNQDAVSDTTPFSFSSKHATIEKNEISILQNLRKWANQYFSSYS

>D1JB7A2

VISSDMYTALNKAQAQKGDFDVVAKILQVHELDEYTNELKLKDASGQVFYTLSLKLKFPHVRTGEVVRIRSATYDETSTQKKVLILSHYSNIITFIQSSKLAKELRAKIQDDHSVEVASLKKNV

>D1JB7A3

SLNAVVLTEVDKKHAALPSTSLQDLFHHADSDKELQAQDTFRTQFYVTKIEPSDVKEWVKGYDRKTKKSSSLKGASGKGDNIFQVQFLVKDASTQLNNNTYRVLLYTQDGLGANFFNVKADNLHKNADARKKLEDSAELLTKFNSYVDAVVERRNGFYLIKDTKLIY

>D1JB7B_

QQQSAFKQLYTELFNNEGDFSKVSSNLKKPLKCYVKESYPHFLVTDGYFFVAPYFTKEAVNEFHAKFPNVNIVDLTDKVIVINNWSLELRRVNSAEVFTSYANLEARLIVHSFKPNLQERLNPTRYPVNLFRDDEFKTTIQHFRHTALQAAINKTVKGDNLVDISKVADAAGKKGKVDAGIVKASASKGDEFSDFSFKEGNTATLKIADIFVQEKG

>D1JEYA1

RALSRLKLKLNKDIVISVGIYNLVQKALKPPPIKLYRETNEPVKTKTRTFNTSTGGLLLPSDTKRSQIYGSRQIILEKEETEELKRFDDPGLMLMGFKPLVLLKKHHYLRPSLFVYPEESLVIGSSTLFSALLIKCLEKEVAALCRYTPRRNIPPYFVALVPQEEELDDQKIQVTPPGFQLVFLPFADDKRKMPFTEKIMATPEQVGKMKAIVEKLRFTYRSDSFENPVLQQHFRNLEALALDLMEPEQAVDLTLPKVEAMNKRLGSLVDEFKELVYPPDY

>D1JEYB1

RHSIHWPCRLTIGSNLSIRIAAYKSILQERVKKTWTVVDAKTLKKEDIQKETVYCLNDDDETEVLKEDIIQGFRYGSDIVPFSKVDEEQMKYKSEGKCFSVLGFCKSSQVQRRFFMGNQVLKVFAARDDEAAAVALSSLIHALDDLDMVAIVRYAYDKRANPQVGVAFPHIKHNYECLVYVQLPFMEDLRQYMFSSLKNSKKYAPTEAQLNAVDALIDSMSLAKKDEKTDTLEDLFPTTKIPNPRFQRLFQCLLHRALHPREPLPPIQQHIWNMLNPPAEVTTKSQIPLSKIKTLFPLIEAKKK

>D1JEYA2

GRDSLIFLVDASKAMFESQSEDELTPFDMSIQCIQSVYISKIISSDRDLLAVVFYGTEKDKNSVNFKNIYVLQELDNPGAKRILELDQFKGQQGQKRFQDMMGHGSDYSLSEVLWVCANLFSDVQFKMSHKRIMLFTNEDNPHGNDSAKASRARTKAGDLRDTGIFLDLMHLKKPGGFDISLFYRDIISIAEDEDLRVHFEESSKLEDLLRKVRAKETRK

>D1JEYB2

NKAAVVLCMDVGFTMSNSIPGIESPFEQAKKVITMFVQRQVFAENKDEIALVLFGTDGTDNPLSGGDQYQNITVHRHLMLPDFDLLEDIESKIQPGSQQADFLDALIVSMDVIQHETIGKKFEKRHIEIFTDLSSRFSKSQLDIIIHSLKKCDISLQFFLPFSLGKEDGSGDRGDGPFRLGGHGPSFPLKGITEQQKEGLEIVKMVMISLEGEDGLDEIYSFSESLRKLCVFKKIE

>D1JFIB_

DDLTIPRAAINKMIKETLPNVRVANDARELVVNCCTEFIHLISSEANEICNKSEKKTISPEHVIQALESLGFGSYISEVKEVLQECKTVALKRRKASSRLENLGIPEEELLRQQQELFAKARQQQAELAQQEWLQ

>D1JX4A1

VRKSIGRIVTMKRNSRNLEEIKPYLFRAIEESYYKLDKRIPKAIHVVAVTEDLDIVSRGRTFPHGISKETAYSESVKLLQKILEEDERKIRRIGVRFSKFI

>D1JX4A2

MIVLFVDFDYFYAQVEEVLNPSLKGKPVVVCVFSGRFEDSGAVATANYEARKFGVKAGIPIVEAKKILPNAVYLPMRKEVYQQVSSRIMNLLREYSEKIEIASIDEAYLDISDKVRDYREAYNLGLEIKNKILEKEKITVTVGISKNKVFAKIAADMAKPNGIKVIDDEEVKRLIRELDIADVPGIGNITAEKLKKLGINKLVDTLSIEFDKLKGMIGEAKAKYLISLARDEYNEPIRTR

>D1K3XA1

VGPDVLDPNLTPEVVKERLLSPRFRNRQFAGLLLDQAFLAGLGNYLRVEILWQVGLTGNHKAKDLNAAQLDALAHALLEIPRFSYATRG

>D1K3XA2

PEGPEIRRAADNLEAAIKGKPLTDVWFAFPQLKTYQSQLIGQHVTHVETRGKALLTHFSNDLTLYSHNQLYGVWRVVDTGEEPQTTRVLRVKLQTADKTILLYSASDIEMLRPEQLTTHPFLQR

>D1K3XA3

ALFRFKVFHRDGEPCERCGSIIEKTTLSSRPFYWCPGCQH

>D1K4TA1

EKSMMNLQTKIDAKKEQLADARRDLKSAKADAKVMKDAKTKKVVESKKKAVQRLEEQLMKLEVQATDREENK

>D1K4TA2

PSSRIKGEKDWQKYETARRLKKCVDKIRNQYREDWKSKEMKVRQRAVALYFIDKLALRAGNEKEEGETADTVGCCSLRVEHINLHPELDGQEYVVEFDFLGKDSIRYYNKVPVEKRVFKNLQLFMENKQPEDDLFDRLNTGILNKHLQDLMEGLTAKVFRTYNASITLQQQLKELTAPDENIPAKILSYNRANRAVAILCNHQRAPPKTFXQIALGTSKLNYLDPRITVAWCKKWGVPIEKIYNKTQREKFAWAIDMADEDYEF

>D1K61A_

RGHRFTKENVRILESWFAKNIENPYLDTKGLENLMKNTSLSRIQIKNWVSNRRRKEKTIT

>D1KFSA1

MISYDNYVTILDEETLKAWIAKLEKAPVFAFDTETDSLDNISANLVGLSFAIEPGVAAYIPVAHDYLDAPDQISRERALELLKPLLEDEKALKVGQNLKYDRGILANYGIELRGIAFDTMLESYILNSVAGRHDMDSLAERWLKHKTITFEEIAGKGKNQLTFNQIALEEAGRYAAEDADVTLQLHLKMWPDLQK

>D1L3LA1

DAAWLDPKEATYLRWIAVGKTMEEIADVEGVKYNSVRVKLREAMKRFDVRSKAHLTALAIRRKLI

>D1L3LA2

QHWLDKLTDLAAIEGDECILKTGLADIADHFGFTGYAYLHIQHRHITAVTNYHRQWQSTYFDKKFEALDPVVKRARSRKHIFTWSGEHERPTLSKDERAFYDHASDFGIRSGITIPIKTANGFMSMFTMASDKPVIDLDREIDAVAAAATIGQIHARISFLRTTPTAE

>D1LLMC2

EKPFACDICGRKFARSDERKRHRDIQHI

>D1LMB3_

PLTQEQLEDARRLKAIYEKKKNELGLSQESVADKMGMGQSGVGALFNGINALNAYNAALLAKILKVSVEEFSPSIAREIYEMYEAVS

>D1M3QA1

DPIECLFSFICSSNNNIARITGMVERLCQAFGPRLIQLDDVTYHGFPSLQALAGPEVEAHLRKLGLGYRARYVSASARAILEEQGGLAWLQQLRESSYEEAHKALCILPGVGTKVADCICLMALDKPQAVPVEVHMWHIAQRDYSWHPTTSQAKGPSPQTNKELGNFFRSLWGPYAGWAQAVLFSADLRQ

>D1M3QA2

GSEGHRTLASTPALWASIPCPRSELRLDLVLPSGQSFRWREQSPAHWSGVLADQVWTLTQTEEQLHCTVYRGDKSQASRPTPDELEAVRKYFQLDVTLAQLYHHWGSVDSHFQEVAQKFQGVRLLRQ

>D1MDYB_

TTNADRRKAATMRERRRLSKVNEAFETLKRSTSSNPNQRLPKVEILRNAIRYIEGLQALLRD

>D1MNNA_

VILTQLNEDGTTSNYFDKRKLKIAPRSTLQFKVGPPFELVRDYCPVVESHTGRTLDLRIIPRIDRGFDHIDEEWVGYKRNYFTLVSTFETANCDLDTFLKSSFDLLVEDSSVEGRLRVQYFAIKIKAKNDDDDTEINLVQHTAKRDKGPQFCPSVCPLVPSPLPKHQTIREASNVRNITKMKKYDSTFYLHRDHVNYEEYGVDSLLFSYPEDSIQKVARYERVQFASSISVKKPSQQNKHFSLHVILGAVVDPDTFHGENPGIPYDELALKNGSKGMFVYLQEMKTPPLIIRGRSPSNYASSQ

>D1MUSA_

SALHRAADWAKSVFSSAALGDPRRTARLVNVAAQLAKYSGKSITISSEGSKAAQEGAYRFIRNPNVSAEAIRKAGAMQTVKLAQEFPELLAIEDTTSLSYRHQVAEELGKLGSIQKASRGWWVHSVLLLEATTFRTVGLLHQEWWMRPDDPADADEKESGKWLAAAATSRLRMGSMMSNVIAVCDREADIHAYLQDKLAHNERFVVRSKHPRKDVESGLYLYDHLKNQPELGGYQISIPQKGVVDKRGKRKNRPARKASLSLRSGRITLKQGNITLNAVLAEEINPPKGETPLKWLLLTSEPVESLAQALRVIDIYTHRWRIEEFHKAWKTGAGAERQRMEKPDNLERMVSILSFVAVRLLQLRESFTPPQALRAQGLLKEAEHVESQSAETVLTPDECQLLGYLDKGKRKRKEKAGSLQWAYMAIARLGGFMDSKRTGIASWGALWEGWEALQSKLDGFLAAKDLMAQGIKIG

>D1NH2B_

NAEASRVYEIIVESVVNEVREDFENAGIDEQTLQDLKNIWQKKLTE

>D1NH2D1

GYYELYRRSTIGNSLVDALDTLISDGRIEASLAMRVLETFDKVVAETLKD

>D1NH2C_

DYLISEGEEDGPDENLMLCLYDKVTRTKARWKCSLKDGVVTINRNDYTFQKAQVEAEWV

>D1NH2D2

NTQSKLTVKGNLDTYGFCDDVWTFIVKNCQVTVEDSHRDASQNGSGDSQSVISVDKLRIVACNSKKS

>D1NH2A1

SGIVPTLQNIVATVTLGCRLDLKTVALHARNAEYNPKRFAAVIMRIREPKTTALIFASGKMVVTGAKSEDDSKLASRKYARIIQKIGFAAKFTDF

>D1ODHA_

LSWDINDVKLPQNVKTTDWFQEWPDSYVKHIYSSDDRNAQRHLSSWAMRNTNNHNSRILKKSCLGVVVCSRDCSTEEGRKIYLRPAICDKARQKQQRKSCPNCNGPLKLIPCRGHGGFPVTNFWRHDGRFIFFQSKGEHDHPRPETKLEAEARRAMKK

>D1OE4A_

ESPADSFLKVELELNLKLSNLVFQDPVQYVYNPLVYAWAPHENYVQTYCKSKKEVLFLGMNPGPFGMAQTGVPFGEVNHVRDWLQIEGPVSKPEVEHPKRRIRGFECPQSEVSGARFWSLFKSLCGQPETFFKHCFVHNHCPLIFMNHSGKNLTPTDLPKAQRDTLLEICDEALCQAVRVLGVKLVIGVGRFSEQRARKALMAEGIDVTVKGIMHPSPRNPQANKGWEGIVRGQLLELGVLSLLT

>D1OJ8A_

EDWDTFQKKHLTDTKKVKCDVEMKKALFDCKKTNTFIFARPPRVQALCKNIKNNTNVLSRDVFYLPQCNRKKLPCHYRLDGSTNTICLTCMKELPIHFAGVGKCP

>D1OMHA_

MLSHMVLTRQDIGRAASYYEDGADDYYAKDGDASEWQGKGAEELGLSGEVDSKRFRELLAGNIGEGHRIMRSATRQDSKERIGLDLTFSAPKSVSLQALVAGDAEIIKAHDRAVARTLEQAEARAQARQKIQGKTRIETTGNLVIGKFRHETSRERDPQLHTHAVILNMTKRSDGQWRALKNDEIVKATRYLGAVYNAELAHELQKLGYQLRYGKDGNFDLAHIDRQQIEGFSKRTEQIAEWYAARGLDPNSVSLEQKQAAKVLSRAKKTSVDREALRAEWQATAKELGIDFS

>D1OZJA_

FTPPIVKRLLGWKKGEQNGQEEKWCEKAVKSLVKKLKKTGQLDELEKAITTQNVNTKCITIPRSLDGRLQVSHRKGLPHVIYCRLWRWPDLHSHHELRAMELCEFAFNMKKDEVCVNPYHYQRVET

>D1P7HL1

ELPMVERQDTDSCLVYGGQQMILTGQNFTSESKVVFTEKTTDGQQIWEMEATVDKDKSQPNMLFVEIPEYRNKHIRTPVKVNFYVINGKRKRSQPQHFTYHPV

>D1P7HL2

SSVPLEWPLSSQSGSYELRIEVQPKPHHRAHYETEGSRGAVKAPTGGHPVVQLHGYMENKPLGLQIFIGTADERILKPHAFYQVHRITGKTVTTTSYEKIVGNTKVLEIPLEPKNNMRATIDCAGILKLRNADIELRKGETDIGRKNTRVRLVFRVHIPESSGRIVSLQTASNPIECSQRSAH

>D1PDNC_

QGRVNQLGGVFINGRPLPNNIRLKIVEMAADGIRPCVISRQLRVSHGCVSKILNRYQETGSIRPGVIGGSKPRIATPEIENRIEEYKRSSPGMFSWEIREKLIREGVCDRSTAPSVSAISRLV

>D1QRVA_

SDKPKRPLSAYMLWLNSARESIKRENPGIKVTEVAKRGGELWRAMKDKSEWEAKAAKAKDDYDRAVKEFEANG

>D1QTMA1

ALEEAPWPPPEGAFVGFVLSRKEPMWADLLALAAARGGRVHRAPEPYKALRDLKEARGLLAKDLSVLALREGLGLPPGDDPMLLAYLLDPSNTTPEGVARRYGGEWTEEAGERAALSERLFANLWGRLEG

>D1QUQA_

HIVPCTISQLLSATLVDEVFRIGNVEISQVTIVGIIRHAEKAPTNIVYKIDDMTAAPMDVRQWVDTDDTSSENTVVPPETYVKVAGHLRSFQNKKSLVAFKIMPLEDMNEFTTHILEVINAHMVLSK

>D1QUQB_

DMMDLPRSRINAGMLAQFIDKPVCFVGRLEKIHPTGKMFILSDGEGKNGTIELMEPLDEEISGIVEVVGRVTAKATILCTSYVQFKEDSHPFDLGLYNEAVKIIHDFPQFYPLG

>D1QZGA_

VIDSLQLNELLNAGEYKIGELTFQSIRSSQELQKKNTIVNLFGIVKDFTPSRQSLHGTKDWVTTVYLWDPTCDTSSIGLQIHLFSKQGNDLPVIKQVGQPLLLHQITLRSYRDRTQGLSKDQFRYALWPDFSSNSKDTLCPQPMPRLMKTGDKEEQFALLLNKIWDEQTN

>D1R2ZA2

PQLPEVETIRRTLLPLIVGKTIEDVRIFWPNIIRHPRDSEAFAARMIGQTVRGLERRGKFLKFLLDRDALISHLRMEGRYAVASALEPLEPHTHVVFCFTDGSELRYRDVRKFGTMHVYAKEEADRRPPLAEL

>D1R71A_

EADQVIENLQRNELTPREIADFIGRELAKGKKKGDIAKEIGKSPAFITQHVTLLDLPEKIADAFNTGRVRDVTVVNELVTAFKKRPEEVEAWLDDDTQEITRGTVKLLREFLDE

>D1R8EA1

ESYYSIGEVSKLANVSIKALRYYDKIDLFKPAYVDPDTSYRYYTDSQLIHLDLIKSLKYIGTPLEEMKKAQDLEMEELFAFYTEQERQIREKLDFLSALEQTISLVKKRMKRQMEYPA

>D1R8EA2

LGEVFVLDEEEIRIIQTEAEGIGPENVLNASYSKLKKFIESADGFTNNSYGATFSFQPYTSIDEMTYRHIFTPVLTNKQISSITPDMEITTIPKGRYACIAYNFSPEHYFLNLQKLIKYIADRQLTVVSDVYELIIPIHYSPKKQEEYRVEMKIRIA

>D1REPC1

SPRIVQSNDLTEAAYSLSRDQKRMLYLFVDQIRKSDGTLQEHDGICEIHVAKYAEIFGLTSAEASKDIRQALKSFAGKEVVFYRPEEDAGDEKGYESFPWFIKPAHSPSRGLYSVHINPYLIPFFIGLQ

>D1REPC2

NRFTQFRLSETKEITNPYAMRLYESLCQYRKPDGSGIVSLKIDWIIERYQLPQSYQRMPDFRRRFLQVCVNEINSRTPMRLSYIEKKKGRQTTHIVFSFRDIT

>D1RH6A_

MYLTLQEWNARQRRPRSLETVRRWVRESRIFPPPVKDGREYLFHESAVKVDLNRP

>D1RRQA2

VKQVPLAVAVLADDEGRVLIRKRDSTGLLANLWEFPSCETDGADGKEKLEQMVGEQYGLQVELTEPIVSFEHAFSHLVWQLTVFPGRLVHGGPVEEPYRLAPEDELKAYAFPVSHQRVWREYKEWAS

>D1RXWA1

LTREQLIDIAILVGTDYNEGVKGVGVKKALNYIKTYGDIFRALKALKVNIDHVEEIRNFFLNPPVTDDYRIEFREPDFEKAIEFLCEEHDFSRERVEKALEKLKA

>D1RXWA2

ADIGDLFEREEVELEYFSGKKIAVDAFNTLYQFISIIRQPDGTPLKDSQGRITSHLSGILYRVSNMVEVGIRPVFVFDGEPPEFKKAEIEERKKRRAEAEEMWIAALQAGDKDAKKYAQAAGRVDEYIVDSAKTLLSYMGIPFVDAPSEGEAQAAYMAAKGDVEYTGSQDYDSLLFGSPRLARNLAITGKRKLPGKNVYVDVKPEIIILESNLKRLG

>D1RZTA1

ATNHNLHITEKLEVLAKAYSVQGDKWRALGYAKAINALKSFHKPVTSYQEACSIPGIGKRMAEKIIEILESGHLRKLDHI

>D1RZTA2

SESVPVLELFSNIWGAGTKTAQMWYQQGFRSLEDIRSQASLTTQQAIGLKHYSDFLE

>D1RZTA3

RMPREEATEIEQTVQKAAQAFNSGLLCVACGSYRRGKATCGDVDVLITHPDGRSHRGIFSRLLDSLRQEGFLTDDLVSQEENGQQQKYLGVCRLPGPGRRHRRLDIIVVPYSEFACALLYFTGSAHFNRSMRALAKTKGMSLSEHALSTAVVRNTHGCKVGPGRVLPTPTEKDVFRLLGLPYREPAERDW

>D1S32F_

AKRHRKVLRDNIQGITKPAIRRLARRGGVKRISGLIYEETRGVLKVFLENVIRDAVTYTEHAKRKTVTAMDVVYALKRQGRTLYGFGG

>D1SA3A_

MRTELLSKLYDDFGIDQLPHTQHGVTSDRLGKLYEKYILDIFKDIESLKKYNTNAFPQEKDISSKLLKALNLDLDNIIDVSSSDTDLGRTIAGGSPKTDATIRFTFHNQSSRLVPLNIKHSSKKKVSIAEYDVETICTGVGISDGELKELIRKHQNDQSAKLFTPVQKQRLTELLEPYRERFIRWCVTLRAEKSEGNILHPDLLIRFQVIDREYVDVTIKNIDDYVSDRIAEGSKARKPGFGTGLNWTYASGSKAKKMQFKG

>D1SFUA_

CTVNDAEIFSLVKKEVLSLNTNDYTTAISLSNRLKINKKKINQQLYKLQKEDTVKMVPSNPPKWFKNYNC

>D1SKNP_

GRQSKDEQLASDNELPVSAFQISEMSLSELQQVLKNESLSEYQRQLIRKIRRRGKNKVAARTCRQRRTDRHDKM

>D1SX5A_

SLRSDLINALYDENQKYDVCGIISAEGKIYPLGSDTAVLSTIFELFSRPIINKIAEKHGYIVEEPKQQNHYPDFTLYKPSEPNKKIAIDIKTTYTNKENEKIKFTLGGYTSFIRNNTKNIVYPFDQYIAHWIIGYVYTRVATRKSSLKTYNINELNEIPKPYKGVKVFLQDKWVIAGDLAGSGNTTNIGSIHAHYKDFVEGKGIFDSEDEFLDYWRNYERTSQLRNDKYNNISEYRNWIYRGRK

>D1T3NA1

QSFSEEDSFKKCSSEVEAKNKIEELLASLLNRVCQDGRKPHTVRLIIRRYSSEKHYGRESRQCPIPSHVIQKLGTGNYDVMTPMVDILMKLFRNMVNVKMPFHLTLLSVCFCNLK

>D1TC3C_

PRGSALSDTERAQLDVMKLLNVSLHEMSRKISRSRHCIRVYLKDPVSYGTS

>D1TDZA1

GPEPTYEDFDEKLFREKLRKSTKKIKPYLLEQTLVAGLGNIYVDEVLWLAKIHPEKETNQLIESSIHLLHDSIIEILQKAIKLGGSSI

>D1TK5A1

MIVSDIEANALLESVTKFHCGVIYDYSTAEYVSYRPSDFGAYLDALEAEVARGGLIVFHNGHKYDVPALTKLAKLQLNREFHLPRENCIDTLVLSRLIHSNLKDTDMGLLRSGKLPGALEAWGYRLGEMKGEYKDDFKRMLEEQGEEYVDGMEWWNFNEEMMDYNVQDVVVTKALLEKLLSDKHYFPPEIDFTDVGYTTFWSES

>D1TK5A2

LEAVDIEHRAAWLLAKQERNGFPFDTKAIEELYVELAARRSELLRKLTETFGSWYQPKGGTEMFCHPRTGKPLPKYPRIKTPKVGGIFKKPKNKAQREGREPCELDTREYVAGAPYTPVEHVVFNPSSRDHIQKKLQEAGWVPTKYTDKGAPVVDDEVLEGVRVDDPEKQAAIDLIKEYLMIQKRIGQSAEGDKAWLRYVAEDGKIHGSVNPNGAVTGRATHAFPNLAQIPGVRSPYGEQCRAAFGAEHHLDGITGKPWVQAGIDASGLELRCLAHFMARFDNGEYAHEILNGDIHTKNQIAAELPTRDNAKTFIYGFLYGAGDEKIGQIVGAGKERGKELKKKFLENTPAIAALRESIQQTLVESSQWVAGEQQVKWKRRWIKGLDGRKVHVRSPHAALNTLLQSAGALICKLWIIKTEEMLVEKGLKHGWDGDFAYMAWVHDEIQVGCRTEEIAQVVIETAQEAMRWVGDHWNFRCLLDTEGKMGPNWAICH

>D1TTUA1

DKAEYRFFEAMGQVANPISPCPVVGSLEVDGHGEASRVELHGRDFKPNLKVWFGATPVETTFRSEESLHCSIPPVSQVRNEQTHWMFTNRTTGDVEVPISLVRDDGVVYSSGLTFSYKS

>D1TTUA2

QSLTSDRMIDFLSNKEKYECVISIFHAKVAQKSYGNEKRFFCPPPCIYLIGQGWKLKKDRVAQLYKTLKASAQKDAAIENDPIHEQQATELVAYIGIGSDTSERQQLDFSTGKVRHPGDQRQDPNIYDYCAAKTLYISDSDKRKYFDLNAQFFYGCGMEIGGFVSQRIKVISKPSKKKQSMKNTD

>D1TTUA3

CKYLCIASGTKVALFNRLRSQTVSTRYLHVEGNAFHASSTKWGAFTIHLFDDERGLQETDNFAVRDGFVYYGSVVKLVDSVTGIALPRLRIRKVDKQQVILDASCSEEPVSQLHKCAFQMIDNELVYLCLSHDKIIQHQATAINEHRHQINDGAAWTIIST

>D1U3EM1

MEWKDIKGYEGHYQVSNTGEVYSIKSGKTLKHQIPKDGYHRIGLFKGGKGKTFQVHRLVAIHFCEGYEEGLVVDHKDGNKDNNLSTNLRWVTQKINVENQMSRGT

>D1U3EM2

LNVSKAQQIAKIKNQKPIIVISPDGIEKEYPSTKCACEELGLTRGKVTDVLKGHRIHHKGYTFRYKLNG

>D1U4BA1

MAFTLADRVTEEMLADKAALVVEVVEENYHDAPIVGIAVVNEHGRFFLRPETALADPQFVAWLGDETKKKSMFDSKRAAVALKWKGIELCGVSFDLLLAAYLLDPAQGVDDVAAAAKMKQYEAVRPDEAVYGKGAKRAVPDEPVLAEHLVRKAAAIWELERPFLDELRRN

>D1U4BA2

EQDRLLVELEQPLSSILAEMEFAGVKVDTKRLEQMGKELAEQLGTVEQRIYELAGQEFNINSPKQLGVILFEKLQLPVLKKTKTGYSTSADVLEKLAPYHEIVENILHYRQLGKLQSTYIEGLLKVVRPDTKKVHTIFNQALTQTGRLSSTEPNLQNIPIRLEEGRKIRQAFVPSESDWLIFAADYSQIELRVLAHIAEDDNLMEAFRRDLDIHTKTAMDIFQVSEDEVTPNMRRQAKAVNFGIVYGISDYGLAQNLNISRKEAAEFIERYFESFPGVKRYMENIVQEAKQKGYVTTLLHRRRYLPDITSRNFNVRSFAERMAMNTPIQGSAADIIKKAMIDLNARLKEERLQAHLLLQVHDELILEAPKEEMERLCRLVPEVMEQAVTLRVPLKVDYHYGSTWYDAK

>D1U78A2

APRRKALSVRDERNVIRAASNSCKTARDIRNELQLSASKRTILNVIKRSG

>D1UBDC1

TIACPHKGCTKMFRDNSAMRKHLHTHGP

>D1UBDC2

RVHVCAECGKAFVESSKLKRHQLVHTGE

>D1VPWA1

TIKDVAKRANVSTTTVSHVINKTRFVAEETRNAVWAAIKELHYSPSAVARSMKVNH

>D1ZMEC1

SVACLSCRKRHIKCPGGNPCQKCVTSNAICEYLEPS

>D2BOPA_

SCFALISGTANQVKCYRFRVKKNHRHRYENCTTTWFTVADNGAERQGQAQILITFGSPSQRQDFLKHVPLPPGMNISGFTASLDF

>D2DNJA_

LKIAAFNIRTFGETKMSNATLASYIVRIVRRYDIVLIQEVRDSHLVAVGKLLDYLNQDDPNTYHYVVSEPLGRNSYKERYLFLFRPNKVSVLDTYQYDDGCESCGNDSFSREPAVVKFSSHSTKVKEFAIVALHSAPSDAVAEINSLYDVYLDVQQKWHLNDVMLMGDFNADCSYVTSSQWSSIRLRTSSTFQWLIPDSADTTATSTNCAYDRIVVAGSLLQSSVVPGSAAPFDFQAAYGLSNEMALAISDHYPVEVTLT

>D2DRPA1

FTKEGEHTYRCKVCSRVYTHISNFCRHYVTSHKRNVK

>D2DRPA2

VYPCPFCFKEFTRKDNMTAHVKIIHK

>D2GLIA1

ETDCRWDGCSQEFDSQEQLVHHINSEHIHGER

>D2GLIA2

KEFVCHWGGCSRELRPFKAQYMLVVHMRRHTGE

>D2IRFG_

RMRMRPWLEEQINSNTIPGLKWLNKEKKIFQIPWMHAARHGWDVEKDAPLFRNWAIHTGKHQPGIDKPDPKTWKANFRCAMNSLPDIEEVKDRSIKKGNNAFRVYRMLP

>D3PVIA_

SHPDLNKLLELWPHIQEYQDLALKHGINDIFQGNGGKLLQVLLITGLTVLPGREGNDAVDNAGQEYELKSINIDLTKGFSTHHHMNPVIIAKYRQVPWIFAIYRGIAIEAIYRLEPKDLEFYYDKWERKWYSDGHKDINNPKIPVKYVMEHGTKIY

(2). non DNA-binding proteins

>1GND-_

MDEEYDVIVLGTGLTECILSGIMSVNGKKVLHMDRNPYYGGESSSITPLEELYKRFQLLEGPPETMGRGRDWNVDLIPKFLMANGQLVKMLLYTEVTRYLDFKVVEGSFVYKGGKIYKVPSTETEALASNLMGMFEKRRFRKFLVFVANFDENDPKTFEGVDPQNTSMRDVYRKFDLGQDVIDFTGHALALYRTDDYLDQPCLETINRIKLYSESLARYGKSPYLYPLYGLGELPQGFARLSAIYGGTYMLNKPVDDIIMENGKVVGVKSEGEVARCKQLICDPSYVPDRVRKAGQVIRIICILSHPIKNTNDANSCQIIIPQNQVNRKSDIYVCMISYAHNVAAQGKYIAIASTTVETTDPEKEVEPALELLEPIDQKFVAISDLYEPIDDGSESQVFCSCSYDATTHFETTCNDIKDIYKRMAGSAFDFENMKRKQNDVFGEADQ

>1PHP-_

MNKKTIRDVDVRGKRVFCRVDFNVPMEQGAITDDTRIRAALPTIRYLIEHGAKVILASHLGRPKGKVVEELRLDAVAKRLGELLERPVAKTNEAVGDEVKAAVDRLNEGDVLLLENVRFYPGEEKNDPELAKAFAELADLYVNDAFGAAHRAHASTEGIAHYLPAVAGFLMEKELEVLGKALSNPDRPFTAIIGGAKVKDKIGVIDNLLEKVDNLIIGGGLAYTFVKALGHDVGKSLLEEDKIELAKSFMEKAKEKGVRFYMPVDVVVADRFANDANTKVVPIDAIPADWSALDIGPKTRELYRDVIRESKLVVWNGPMGVFEMDAFAHGTKAIAEALAEALDTYSVIGGGDSAAAVEKFGLADKMDHISTGGGASLEFMEGKQLPGVVALEDK

>1LKI-_

SPLPITPVNATCAIRHPCHGNLMNQIKNQLAQLNGSANALFISYYTAQGEPFPNNVEKLCAPNMTDFPSFHGNGTEKTKLVELYRMVAYLSASLTNITRDQKVLNPTAVSLQVKLNATIDVMRGLLSNVLCRLCNKYRVGHVDVPPVPDHSDKEAFQRKKLGCQLLGTYKQVISVVVQAF

>1MRP-_

DITVYNGQHKEAATAVAKAFEQETGIKVTLNSGKSEQLAGQLKEEGDKTPADVFYTEQTATFADLSEAGLLAPISEQTIQQTAQKGVPLAPKKDWIALSGRSRVVVYDHTKLSEKDMEKSVLDYATPKWKGKIGYVSTSGAFLEQVVALSKMKGDKVALNWLKGLKENGKLYAKNSVALQAVENGEVPAALINNYYWYNLAKEKGVENLKSRLYFVRHQDPGALVSYSGAAVLKASKNQAEAQKFVDFLASKKGQEALVAARAEYPLRADVVSPFNLEPYEKLEAPVVSATTAQDKEHAIKLIEEAGLK

>1MAI-_

MHGLQDDPDLQALLKGSQLLKVKSSSWRRERFYKLQEDCKTIWQESRKVMRSPESQLFSIEDIQEVRMGHRTEGLEKFARDIPEDRCFSIVFKDQRNTLDLIAPSPADAQHWVQGLRKIIHHSGSMDQRQK

>1A8E-_

DKTVRWCAVSEHEATKCQSFRDHMKSVIPSDGPSVACVKKASYLDCIRAIAANEADAVTLDAGLVYDAYLAPNNLKPVVAEFYGSKEDPQTFYYAVAVVKKDSGFQMNQLRGKKSCHTGLGRSAGWNIPIGLLYCDLPEPRKPLEKAVANFFSGSCAPCADGTDFPQLCQLCPGCGCSTLNQYFGYSGAFKCLKDGAGDVAFVKHSTIFENLANKADRDQYELLCLDNTRKPVDEYKDCHLAQVPSHTVVARSMGGKEDLIWELLNQAQEHFGKDKSKEFQLFSSPHGKDLLFKDSAHGFLKVPPRMDAKMYLGYEYVTAIRNLREGTC

>1CZJ-_

ETFEIPESVTMSPKQFEGYTPKKGDVTFNHASHMDIACQQCHHTVPDTYTIESCMTEGCHDNIKERTEISSVYRTFHTTKDSEKSCVGCHRELKRQGPSDAPLACNSCHVQ

>1A53-_

PRYLKGWLKDVVQLSLRRPSFRASRQRPIISLNERILEFNKRNITAIIAEYKRKSPSGLDVERDPIEYSKFMERYAVGLSILTEEKYFNGSYETLRKIASSVSIPILMKDFIVKESQIDDAYNLGADTVLLIVKILTERELESLLEYARSYGMEPLIEINDENDLDIALRIGARFIGINSRDLETLEINKENQRKLISMIPSNVVKVAESGISERNEIEELRKLGVNAFLIGSSLMRNPEKIKEFIL

>1BR9-_

CSCSPVHPQQAFCNADVVIRAKAVSEKEVDSGNDIYGNPIKRIQYEIKQIKMFKGPEKDIEFIYTAPSSAVCGVSLDVGGKKEYLIAGKAEGDGKMHITLCDFIVPWDTLSTTQKKSLNHRYQMGCECKITRCPMIPCYISSPDECLWMDWVTEKNINGHQAKFFACIKRSDGSCAWYRGAAPPKQEFLDIEDP

>1PPN-_

IPEYVDWRQKGAVTPVKNQGSCGSCWAFSAVVTIEGIIKIRTGNLNEYSEQELLDCDRRSYGCNGGYPWSALQLVAQYGIHYRNTYPYEGVQRYCRSREKGPYAAKTDGVRQVQPYNEGALLYSIANQPVSVVLEAAGKDFQLYRGGIFVGPCGNKVDHAVAAVGYGPNYILIKNSWGTGWGENGYIRIKRGTGNSYGVCGLYTSSFYPVKN

>1PBV-_

ANEGSKTLQRNRKMAMGRKKFNMDPKKGIQFLVENELLQNTPEEIARFLYKGEGLNKTAIGDYLGEREELNLAVLHAFVDLHEFTDLNLVQALRQFLWSFRLPGEAQKIDRMMEAFAQRYCLCNPGVFQSTDTCYVLSFAVIMLNTSLHNPNVRDKPGLERFVAMNRGINEGGDLPEELLRNLYDSIRNEPFKIP

>1FIT-_

MSFRFGQHLIKPSVVFLKTELSFALVNRKPVVPGHVLVCPLRPVERFHDLRPDEVADLFQTTQRVGTVVEKHFHGTSLTFSXQDGPEAGQTVKHVHVHVLPRKAGDFHRNDSIYEELQKHDKEDFPASWRSEEEXAAEAAALRVYFQ

>1BDB-_

MKLKGEAVLITGGASGLGRALVDRFVAEGAKVAVLDKSAERLAELETDHGDNVLGIVGDVRSLEDQKQAASRCVARFGKIDTLIPNAGIWDYSTALVDLPEESLDAAFDEVFHINVKGYIHAVKACLPALVASRGNVIFTISNAGFYPNGGGPLYTAAKHAIVGLVRELAFELAPYVRVNGVGSGGINSDLRGPSSLGMGSKAISTVPLADMLKSVLPIGRMPEVEEYTGAYVFFATRGDAAPATGALLNYDGGLGVRGFFSGAGGNDLLEQLNIHP

>1LID-_

CDAFVGTWKLVSSENFDDYMKEVGVGFATRKVAGMAKPNMIISVNGDLVTIRSESTFKNTEISFKLGVEFDEITADDRKVKSIITLDGGALVQVQKWDGKSTTIKRKRDGDKLVVECVMKGVTSTRVYERA

>1RCB-_

HKCDITLQEIIKTLNSLTEQKTLCTELTVTDIFAASKNTTEKETFCRAATVLRQFYSHHEKDTRCLGATAQQFHRHKQLIRFLKRLDRNLWGLAGLNSCPVKEANQSTLENFLERLKTIMREKYSKCSS

>1COT-_

QDGDAAKGEKEFNKCKACHMIQAPDGTDIIKGGKTGPNLYGVVGRKIASEEGFKYGEGILEVAEKNPDLTWTEADLIEYVTDPKPWLVKMTDDKGAKTKMTFKMGKNQADVVAFLAQNSPDAGGDGEAA

>1CSH-_

STNLKDVLASLIPKEQARIKTFRQQHGNTAVGQITVDMSYGGMRGMKGLIYETSVLDPDEGIRFRGFSIPECQKLLPKAGGGEEPLPEGLFWLLVTGQIPTPEQVSWVSKEWAKRAALPSHVVTMLDNFPTNLHPMSQLSAAITALNSESNFARAYAEGINRTKYWEFVYEDAMDLIAKLPCVAAKIYRNLYRAGSSIGAIDSKLDWSHNFTNMLGYTDPQFTELMRLYLTIHSDHEGGNVSAHTSHLVGSALSDPYLSFAAAMNGLAGPLHGLANQEVLLWLSQLQKDLGADASDEKLRDYIWNTLNSGRVVPGYGHAVLRKTDPRYTCQREFALKHLPSDPMFKLVAQLYKIVPNVLLEQGKAKNPWPNVDAHSGVLLQYYGMTEMNYYTVLFGVSRALGVLAQLIWSRALGFPLERPKSMSTAGLEKLSAGG

>1A8P-_

MSNLNVERVLSVHHWNDTLFSFKTTRNPSLRFENGQFVMIGLEVDGRPLMRAYSIASPNYEEHLEFFSIKVQNGPLTSRLQHLKEGDELMVSRKPTGTLVTSDLLPGKHLYMLSTGTGLAPFMSLIQDPEVYERFEKVVLIHGVRQVNELAYQQFITEHLPQSEYFGEAVKEKLIYYPTVTRESFHNQGRLTDLMRSGKLFEDIGLPPINPQDDRAMICGSPSMLDESCEVLDGFGLKISPRMGEPGDYLIERAFVEK

>1ATG-_

ELKVVTATNFLGTLEQLAGQFAKQTGHAVVISSGSSGPVYAQIVNGAPYNVFFSADEKSPEKLDNQGFALPGSRFTYAIGKLVLWSAKPGLVDNQGKVLAGNGWRHIAISNPQIAPYGLAGTQVLTHLGLLDKLTAQERIVEANSVGQAHSQTASGAADLGFVALAQIIQAAAKIPGSHWFPPANYYEPIVQQAVITKSTAEKANAEQFMSWMKGPKAVAIIKAAGYVLPQ

>1BG2-_

MADLAECNIKVMCRFRPLNESEVNRGDKYIAKFQGEDTVVIASKPYAFDRVFQSSTSQEQVYNDCAKKIVKDVLEGYNGTIFAYGQTSSGKTHTMEGKLHDPEGMGIIPRIVQDIFNYIYSMDENLEFHIKVSYFEIYLDKIRDLLDVSKTNLSVHEDKNRVPYVKGCTERFVCSPDEVMDTIDEGKSNRHVAVTNMNEHSSRSHSIFLINVKQENTQTEQKLSGKLYLVDLAGSEKVSKTGAEGAVLDEAKNINKSLSALGNVISALAEGSTYVPYRDSKMTRILQDSLGGNCRTTIVICCSPSSYNESETKSTLLFGQRAKTI

>1FMK-_

MVTTFVALYDYESRTETDLSFKKGERLQIVNNTEGDWWLAHSLSTGQTGYIPSNYVAPSDSIQAEEWYFGKITRRESERLLLNAENPRGTFLVRESETTKGAYCLSVSDFDNAKGLNVKHYKIRKLDSGGFYITSRTQFNSLQQLVAYYSKHADGLCHRLTTVCPTSKPQTQGLAKDAWEIPRESLRLEVKLGQGCFGEVWMGTWNGTTRVAIKTLKPGTMSPEAFLQEAQVMKKLRHEKLVQLYAVVSEEPIYIVTEYMSKGSLLDFLKGETGKYLRLPQLVDMAAQIASGMAYVERMNYVHRDLRAANILVGENLVCKVADFGLARLIEDNEYTARQGAKFPIKWTAPEAALYGRFTIKSDVWSFGILLTELTTKGRVPYPGMVNREVLDQVERGYRMPCPPECPESLHDLMCQCWRKEPEERPTFEYLQAFLEDYFTSTEPQXQPGENL

>1NDH-_

STPAITLENPDIKYPLRLIDKEVVNHDTRRFRFALPSPEHILGLPVGQHIYLSARIDGNLVIRPYTPVSSDDDKGFVDLVIKVYFKDTHPKFPAGGKMSQYLESMKIGDTIEFRGPNGLLVYQGKGKFAIRPDKKSSPVIKTVKSVGMIAGGTGITPMLQVIRAIMKDPDDHTVCHLLFANQTEKDILLRPELEELRNEHSARFKLWYTVDRAPEAWDYSQGFVNEEMIRDHLPPPEEEPLVLMCGPPPMIQYACLPNLERVGHPKERCFAF

>1CSN-_

MSGQNNVVGVHYKVGRRIGEGSFGVIFEGTNLLNNQQVAIKFEPRRSDAPQLRDEYRTYKLLAGCTGIPNVYYFGQEGLHNVLVIDLLGPSLEDLLDLCGRKFSVKTVAMAAKQMLARVQSIHEKSLVYRDIKPDNFLIGRPNSKNANMIYVVDFGMVKFYRDPVTKQHIPYREKKNLSGTARYMSINTHLGREQSRRDDLEALGHVFMYFLRGSLPWQGLKAATNKQKYERIGEKKQSTPLRELCAGFPEEFYKYMHYARNLAFDATPDYDYLQGLFSKVLERLNTTEDENFDWNLL

>1NSJ-_

MVRVKICGITNLEDALFSVESGADAVGFVFYPKSKRYISPEDARRISVELPPFVFRVGVFVNEEPEKILDVASYVQLNAVQLHGEEPIELCRKIAERILVIKAVGVSNERDMERALNYREFPILLDTKTPEYGGSGKTFDWSLILPYRDRFRYLVLSGGLNPENVRSAIDVVRPFAVDVSSGVEAFPGKKDHDSIKMFIKNAKGL

>1OYC-_

MSFVKDFKPQALGDTNLFKPIKIGNNELLHRAVIPPLTRMRALHPGNIPNRDWAVEYYTQRAQRPGTMIITEGAFISPQAGGYDNAPGVWSEEQMVEWTKIFNAIHEKKSFVWVQLWVLGWAAFPDNLARDGLRYDSASDNVFMDAEQEAKAKKANNPQHSLTKDEIKQYIKEYVQAAKNSIAAGADGVEIHSANGYLLNQFLDPHSNTRTDEYGGSIENRARFTLEVVDALVEAIGHEKVGLRLSPYGVFNSMSGGAETGIVAQYAYVAGELEKRAKAGKRLAFVHLVEPRVTNPFLTEGEGEYEGGSNDFVYSIWKGPVIRAGNFALHPEVVREEVKDKRTLIGYGRFFISNPDLVDRLEKGLPLNKYDRDTFYQMSAHGYIDYPTYEEALKLGWDKK

>1LFO-_

XMNFSGKYQVQSQENFEPFMKAMGLPEDLIQKGKDIKGVSEIVHEGKKVKLTITYGSKVIHNEFTLGEEXELETMTGEKVKAVVKMEGDNKMVTTFKGIKSVTEFNGDTITNTMTLGDIVYKRVSKRI

>1CIY-_

IETGYTPIDISLSLTQFLLSEFVPGAGFVLGLVDIIWGIFGPSQWDAFLVQIEQLINQRIEEFARNQAISRLEGLSNLYQIYAESFREWEADPTNPALREEMRIQFNDMNSALTTAIPLLAVQNYQVPLLSVYVQAANLHLSVLRDVSVFGQRWGFDAATINSRYNDLTRLIGNYTDYAVRWYNTGLERVWGPDSRDWVRYNQFRRELTLTVLDIVALFSNYDSRRYPIRTVSQLTREIYTNPVLENFDGSFRGMAQRIEQNIRQPHLMDILNSITIYTDVHRGFNYWSGHQITASPVGFSGPEFAFPLFGNAGNAAPPVLVSLTGLGIFRTLSSPLYRRIILGSGPNNQELFVLDGTEFSFASLTTNLPSTIYRQRGTVDSLDVIPPQDNSVPPRAGFSHRLSHVTMLSQAAGAVYTLRAPTFSWQHRSAEFNNIIPSSQITQIPLTKSTNLGSGTSVVKGPGFTGGDILRRTSPGQISTLRVNITAPLSQRYRVRIRYASTTNLQFHTSIDGRPINQGNFSATMSSGSNLQSGSFRTVGFTTPFNFSNGSSVFTLSAHVFNSGNEVYIDRIEFVPAEVTFEAEYDLER

>1GKY-_

XSRPIVISGPSGTGKSTLLKKLFAEYPDSFGFSVSSTTRTPRAGEVNGKDYNFVSVDEFKSMIKNNEFIEWAQFSGNYYGSTVASVKQVSKSGKTCILDIDMQGVKSVKAIPELNARFLFIAPPSVEDLKKRLEGRGTETEESINKRLSAAQAELAYAETGAHDKVIVNDDLDKAYKELKDFIFAEK

>1OPR-_

MKPYQRQFIEFALNKQVLKFGEFTLKSGRKSPYFFNAGLFNTGRDLALLGRFYAEALVDSGIEFDLLFGPAYKGIPIATTTAVALAEHHDKDLPYCFNRKEAKDHGEGGSLVGSALQGRVMLVDDVITAGTAIRESMEIIQAHGATLAGVLISLDRQERGRGEISAIQEVERDYGCKVISIITLKDLIAYLEEKPDMAEHLAAVRAYREEFGV

>1AXN-_

SASIWVGHRGTVRDYPDFSPSVDAEAIQKAIRGIGTDEKMLISILTERSNAQRQLIVKEYQAAYGKELKDDLKGDLSGHFEHLMVALVTPPAVFDAKQLKKSMKGAGTNEDALIEILTTRTSRQMKDISQAYYTVYKKSLGDDISSETSGDFRKALLTLADGRRDESLKVDEHLAKQDAQILYKAGENRWGTDEDKFTEILCLRSFPQLKLTFDEYRNISQKDIVDSIKGELSGHFEDLLLAIVNCVRNTPAFLAERLHRALKGIGTDEFTLNRIMVSRSEIDLLDIRTEFKKHYGYSLYSAIKSDTSGDYEITLLKICGGDD

>1FDS-_

ARTVVLITGCSSGIGLHLAVRLASDPSQSFKVYATLRDLKTQGRLWEAARALACPPGSLETLQLDVRDSKSVAAARERVTEGRVDVLVCNAGLGLLGPLEALGEDAVASVLDVNVVGTVRMLQAFLPDMKRRGSGRVLVTGSVGGLMGLPFNDVYCASKFALEGLCESLAVLLLPFGVHLSLIECGPVHTAFMEKVLGSPEEVLDRTDIHTFHRFYQYLAHSKQVFREAAQNPEEVAEVFLTALRAPKPTLRYFTTERFLPLLRMRLDDPSGSNYVTAMHREVFGDVPAKAEAGAEAGGGRGPGAEDEAGRSAVGDPELGDPPAAPQ

>1DRW-_

MHDANIRVAIAGAGGRMGRQLIQAALALEGVQLGAALEREGSSLLGSDAGELAGAGKTGVTVQSSLDAVKDDFDVFIDFTRPEGTLNHLAFCRQHGKGMVIGTTGFDEAGKQAIRDAAADIAIVFAANFSVGVNVMLKLLEKAAKVMGDYTDIEIIEAHHRHKVDAPSGTALAMGEAIAHALDKDLKDCAVYSREGHTGERVPGTIGFATVRAGDIVGEHTAMFADIGERLEITHKASSRMTFANGAVRSALWLSGKESGLFDMRDVLDLNNL

>1IDO-_

GCPQEIFADSDIAFLIDGSGSIIPHDFRRMKEFVSTVMEQLKKSKTLFSLMQYSEEFRIHFTFKEFQNNPNPRSLVKPITQLLGRTHTATGIRKVVRELFNITNGARKNAFKILVVITDGEKFGDPLGYEDVIPEADREGVIRYVIGVGDAFRSEKSRQELNTIASKPPRDHVFQVNNFEALKTIQNQLREK

>1FRB-_

ATFVELSTKAKMPIVGLGTWKSPPNQVKEAVKAAIDAGYRHIDCAYAYCNENEVGEAIQEKIKEKAVQREDLFIVSKLWPTCFEKKLLKEAFQKTLTDLKLDYLDLYLIHWPQGLQPGKELFPKDDQGRILTSKTTFLEAWEGMEELVDQGLVKALGVSNFNHFQIERLLNKPGLKHKPVTNQVECHPYLTQEKLIQYCHSKGISVTAYSPLGSPDRPSAKPEDPSLLEDPKIKEIAAKHEKTSAQVLIRFHIQRNVVVIPKSVTPSRIQENIQVFDFQLSDEEMATILSFNRNWRACLLPETVNMEEYPYDAEY

>1LAM-_

TKGLVLGIYSKEKEEDEPQFTSAGENFNKLVSGKLREILNISGPPLKAGKTRTFYGLHEDFPSVVVVGLGKKTAGIDEQENWHEGKENIRAAVAAGCRQIQDLEIPSVEVDPCGDAQAAAEGAVLGLYEYDDLKQKRKVVVSAKLHGSEDQEAWQRGVLFASGQNLARRLMETPANEMTPTKFAEIVEENLKSASIKTDVFIRPKSWIEEQEMGSFLSVAKGSEEPPVFLEIHYKGSPNASEPPLVFVGKGITFDSGGISIKAAANMDLMRADMGGAATICSAIVSAAKLDLPINIVGLAPLCENMPSGKANKPGDVVRARNGKTIQVDNTDAEGRLILADALCYAHTFNPKVIINAATLTGAMDIALGSGATGVFTNSSWLWNKLFEASIETGDRVWRMPLFEHYTRQVIDCQLADVNNIGKYRSAGACTAAAFLKEFVTHPKWAHLDIAGVMTNKDEVPYLRKGMAGRPTRTLIEFLFRFSQ

>1PDA-_

MLDNVLRIATRQSPLALWQAHYVKDKLMASHPGLVVELVPMVTRGDVILDTPLAKVGGKGLFVKELEVALLENRADIAVHSMKDVPVEFPQGLGLVTICEREDPRDAFVSNNYDSLDALPAGSIVGTSSLRRQCQLAERRPDLIIRSLRGNVGTRLSKLDNGEYDAIILAVAGLKRLGLESRIRAALPPEISLPAVGQGAVGIECRLDDSRTRELLAALNHHETALRVTAERAMNTRLEGGCQVPIGSYAELIDGEIWLRALVGAPDGSQIIRGERRGAPQDAEQMGISLAEELLNNGAREILAEVYNGDAPA

>1CPT-_

MDARATIPEHIARTVILPQGYADDEVIYPAFKWLRDEQPLAMAHIEGYDPMWIATKHADVMQIGKQPGLFSNAEGSEILYDQNNEAFMRSISGGCPHVIDSLTSMDPPTHTAYRGLTLNWFQPASIRKLEENIRRIAQASVQRLLDFDGECDFMTDCALYYPLHVVMTALGVPEDDEPLMLKLTQDFFGVEAARRFHETIATFYDYFNGFTVDRRSCPKDDVMSLLANSKLDGNYIDDKYINAYYVAIATAGHDTTSSSSGGAIIGLSRNPEQLALAKSDPALIPRLVDEAVRWTAPVKSFMRTALADTEVRGQNIKRGDRIMLSYPSANRDEEVFSNPDEFDITRFPNRHLGFGWGAHMCLGQHLAKLEMKIFFEELLPKLKSVELSGPPRLVATNFVGGPKNVPIRFTKA

>1FNC-_

QIASDVEAPPPAPAKVEKHSKKMEEGITVNKFKPKTPYVGRCLLNTKITGDDAPGETWHMVFSHEGEIPYREGQSVGVIPDGEDKNGKPHKLRLYSIASSALGDFGDAKSVSLCVKRLIYTNDAGETIKGVCSNFLCDLKPGAEVKLTGPVGKEMLMPKDPNATIIMLGTGTGIAPFRSFLWKMFFEKHDDYKFNGLAWLFLGVPTSSSLLYKEEFEKMKEKAPDNFRLDFAVSREQTNEKGEKMYIQTRMAQYAVELWEMLKKDNTYVYMCGLKGMEKGIDDIMVSLAAAEGIDWIEYKRQLKKAEQWNVEVY

>1HCZ-_

YPIFAQQNYENPREATGRIVCANCHLASKPVDIEVPQAVLPDTVFEAVVKIPYDMQLKQVLANGKKGALNVGAVLILPEGFELAPPDRISPEMKEKIGNLSFQNYRPNKKNILVIGPVPGQKYSEITFPILAPDPATNKDVHFLKYPIYVGGNRGRGQIYPDGSKSNNTVYNATAGGIISKILRKEKGGYEITIVDASNERQVIDIIPRGLELLVSEGESIKLDQPLTSNPNVGGFGQGDAEIVLQDPLRVQ

>1AYL-_

MRVNNGLTPQELEAYGISDVHDIVYNPSYDLLYQEELDPSLTGYERGVLTNLGAVAVDTGIFTGRSPKDKYIVRDDTTRDTFWWADKGKGKNDNKPLSPETWQHLKGLVTRQLSGKRLFVVDAFCGANPDTRLSVRFITEVAWQAHFVKNMFIRPSDEELAGFKPDFIVMNGAKCTNPQWKEQGLNSENFVAFNLTERMQLIGGTWYGGEMKKGMFSMMNYLLPLKGIASMHCSANVGEKGDVAVFFGLSGTGKTTLSTDPKRRLIGDDEHGWDDDGVFNFEGGCYAKTIKLSKEAEPEIYNAIRRDALLENVTVREDGTIDFDDGSKTENTRVSYPIYHIDNIVKPVSKAGHATKVIFLTADAFGVLPPVSRLTADQTQYHFLSGFTAKLAGTERGITEPTPTFSACFGAAFLSLHPTQYAEVLVKRMQAAGAQAYLVNTGWNGTGKRISIKDTRAIIDAILNGSLDNAETFTLPMFNLAIPTELPGVDTKILDPRNTYASPEQWQEKAETLAKLFIDNFDKYTDTPAGAALVAAGPKLS

>1UAE-_

MDKFRVQGPTKLQGEVTISGAKNAALPILFAALLAEEPVEIQNVPKLKDVDTSMKLLSQLGAKVERNGSVHIDARDVNVFCAPYDLVKTMRASIWALGPLVARFGQGQVSLPGGCTIGARPVDLHISGLEQLGATIKLEEGYVKASVDGRLKGAHIVMDKVSVGATVTIMCAATLAEGTTIIENAAREPEIVDTANFLITLGAKISGQGTDRIVIEGVERLGGGVYRVLPDRIETGTFLVAAAISRGKIICRNAQPDTLDAVLAKLRDAGADIEVGEDWISLDMHGKRPKAVNVRTAPHPAFPTDMQAQFTLLNLVAEGTGFITETVFENRFMHVPELSRMGAHAEIESNTVICHGVEKLSGAQVMATDLRASASLVLAGCIAEGTTVVDRIYHIDRGYERIEDKLRALGANIERVKGE

>1A6O-A

MSKARVYADVNVLRPKEYWDYEALTVQWGEQDDYEVVRKVGRGKYSEVFEGINVNNNEKCIIKILKPVKKKKIKREIKILQNLCGGPNIVKLLDIVRDQHSKTPSLIFEYVNNTDFKVLYPTLTDYDIRYYIYELLKALDYCHSQGIMHRDVKPHNVMIDHELRKLRLIDWGLAEFYHPGKEYNVRVASRYFKGPELLVDLQDYDYSLDMWSLGCMFAGMIFRKEPFFYGHDNHDQLVKIAKVLGTDGLNVYLNKYRIELDPQLEALVGRHSRKPWLKFMNADNQHLVSPEAIDFLDKLLRYDHQERLTALEAMTHPYFQQVRAAENSRTRA

>1GOX-_

XMEITNVNEYEAIAKQKLPKMVYDYYASGAEDQWTLAENRNAFSRILFRPRILIDVTNIDMTTTILGFKISMPIMIAPTAMQKMAHPEGEYATARAASAAGTIMTLSSWATSSVEEVASTGPGIRFFQLYVYKDRNVVAQLVRRAERAGFKAIALTVDTPRLGRREADIKNRFVLPPFLTLKNFEGIDLGKMDKANDSGGLSSYVAGQIDRSLSWKDVAWLQTITSLPILVKGVITAEDARLAVQHGAAGIIVSNHGARQLDYVPATIMALEEVVKAAQGRIPVFLDGGVRRGTDVFKALALGAAGVFIGRPVVFSLAAEGEAGVKKVLQMMRDEFELTMALSGCRSLKEISRSHIAADWDPSSRAVARL

>1OAA-_

ADGLGCAVCVLTGASRGFGRALAPQLARLLSPGSVMLVSARSESMLRQLKEELGAQQPDLKVVLAAADLGTEAGVQRLLSAVRELPRPEGLQRLLLINNAATLGDVSKGFLNVNDLAEVNNYWALNLTSMLCLTSGTLNAFQDSPGLSKTVVNISSLCALQPYKGWGLYCAGKAARDMLYQVLAAEEPSVRVLSYAPGPLDNDMQQLARETSKDPELRSKLQKLKSDGALVDCGTSAQKLLGLLQKDTFQSGAHVDFYD

>1A7S-_

IVGGRKARPRQFPFLASIQNQGRHFCGGALIHARFVMTAASCFPGVSTVVLGAYDLRRRERQSRQTFSISSMSENGYDPQQNLNDLMLLQLDREANLTSSVTILPLPLQNATVEAGTRCQVAGWGSQRSGGRLSRFPRFVNVTVTPEDQCRPNNVCTGVLTRRGGICNGDGGTPLVCEGLAHGVASFSLGPCGRGPDFFTRVALFRDWIDGVLNNPGPGPA

>1HCL-_

MENFQKVEKIGEGTYGVVYKARNKLTGEVVALKKIRLDTETEGVPSTAIREISLLKELNHPNIVKLLDVIHTENKLYLVFEFLHQDLKKFMDASALTGIPLPLIKSYLFQLLQGLAFCHSHRVLHRDLKPQNLLINTEGAIKLADFGLARAFGVPVRTYTHEVVTLWYRAPEILLGCKYYSTAVDIWSLGCIFAEMVTRRALFPGDSEIDQLFRIFRTLGTPDEVVWPGVTSMPDYKPSFPKWARQDFSKVVPPLDEDGRSLLSQMLHYDPNKRISAKAALAHPFFQDVTKPVPHLRL

>1A17-_

RDEPPADGALKRAEELKTQANDYFKAKDYENAIKFYSQAIELNPSNAIYYGNRSLAYLRTECYGYALGDATRAIELDKKYIKGYYRRAASNMALGKFRAALRDYETVVKVKPHDKDAKMKYQECNKIVKQKAFERAIAGDEHKRSVVDSLDIESMTIEDEYSGPKL

>1PBE-_

MKTQVAIIGAGPSGLLLGQLLHKAGIDNVILERQTPDYVLGRIRAGVLEQGMVDLLREAGVDRRMARDGLVHEGVEIAFAGQRRRIDLKRLSGGKTVTVYGQTEVTRDLMEAREACGATTVYQAAEVRLHDLQGERPYVTFERDGERLRLDCDYIAGCDGFHGISRQSIPAERLKVFERVYPFGWLGLLADTPPVSHELIYANHPRGFALCSQRSATRSRYYVQVPLTEKVEDWSDERFWTELKARLPAEVAEKLVTGPSLEKSIAPLRSFVVEPMQHGRLFLAGDAAHIVPPTGAKGLNLAASDVSTLYRLLLKAYREGRGELLERYSAICLRRIWKAERFSWWMTSVLHRFPDTDAFSQRIQQTELEYYLGSEAGLATIAENYVGLPYEEIE

>1SKF-_

VTKPTIAAVGGYAMNNGTGTTLYTKAADTRRSTGSTTKIMTAKVVLAQSNLNLDAKVTIQKAYSDYVVANNASQAHLIVGDKVTVRQLLYGLMLPSGCDAAYALADKYGSGSTRAARVKSFIGKMNTAATNLGLHNTHFDSFDGIGNGANYSTPRDLTKIASSAMKNSTFRTVVKTKAYTAKTVTKTGSIRTMDTWKNTNGLLSSYSGAIGVKTGSGPEAKYCLVFAATRGGKTVIGTVLASTSIPARESDATKIMNYGFAL

>1AF7-_

SVLLQMTQRLALSDAHFRRICQLIYQRAGIVLADHKRDMVYNRLVRRLRALGLDDFGRYLSMLEANQNSAEWQAFINALTTNLTAFFREAHHFPILAEHARRRHGEYRVWSAAASTGEEPYSIAITLADALGMAPGRWKVFASDIDTEVLEKARSGIYRLSELKTLSPQQLQRYFMRGTGPHEGLVRVRQELANYVEFSSVNLLEKQYNVPGPFDAIFCRNVMIYFDKTTQEDILRRFVPLLKPDGLLFAGHSENFSNLVREFSLRGQTVYALS

>1AJ2-_

MKLFAQGTSLDLSHPHVMGILNVTPDSFSDGGTHNSLIDAVKHANLMINAGATIIDVGGESTRPGAAEVSVEEELQRVIPVVEAIAQRFEVWISVDTSKPEVIRESAKVGAHIINDIRSLSEPGALEAAAETGLPVCLMHMQGNPKTMQEAPKYDDVFAEVNRYFIEQIARCEQAGIAKEKLLLDPGFGFGKNLSHNYSLLARLAEFHHFNLPLLVGMSRKSMIGQLLNVGPSERLSGSLACAVIAAMQGAHIIRVHDVKETVEAMRVVEATLSAKENKRYE

>153L-_

RTDCYGNVNRIDTTGASCKTAKPEGLSYCGVSASKKIAERDLQAMDRYKTIIKKVGEKLCVEPAVIAGIISRESHAGKVLKNGWGDRGNGFGLMQVDKRSHKPQGTWNGEVHITQGTTILINFIKTIQKKFPSWTKDQQLKGGISAYNAGAGNVRSYARMDIGTTHDDYANDVVARAQYYKQHGY

>1A1X-_

GSAGEDVGAPPDHLWVHQEGIYRDEYQRTWVAVVEEETSFLRARVQQIQVPLGDAARPSHLLTSQLPLMWQLYPEERYMDNNSRLWQIQHHLMVRGVQELLLKLLPDD

>1A44-_

PVDLSKWSGPLSLQEVDERPQHPLQVKYGGAEVDELGKVLTPTQVKNRPTSITWDGLDPGKLYTLVLTDPDAPSRKDPKYREWHHFLVVNMKGNNISSGTVLSDYVGSGPPKGTGLHRYVWLVYEQEGPLKCDEPILSNRSGDHRGKFKVASFRKKYELGAPVAGTCYQAEWDDYVPKLYEQLSG

>1A48-_

XSITKTELDGILPLVARGKVRDIYEVDAGTLLFVATDRISAYDVIMENSIPEKGILLTKLSEFWFKFLSNDVRNHLVDIAPGKTIFDYLPAKLSEPKYKTQLEDRSLLVHKHKLIPLEVIVRGYITGSAWKEYVKTGTVHGLKQPQGLKESQEFPEPIFTPSTKAEQGEHDENISPAQAAELVGEDLSRRVAELAVKLYSKCKDYAKEKGIIIADTKFEFGIDEKTNEIILVDEVLTPDSSRFWNGASYKVGESQDSYDKQFLRDWLTANKLNGVNGVKMPQDIVDRTRAKYIEAYETLTGSKWSH

>1A6M-_

VLSEGEWQLVLHVWAKVEADVAGHGQDILIRLFKSHPETLEKFDRFKHLKTEAEMKASEDLKKHGVTVLTALGAILKKKGHHEAELKPLAQSHATKHKIPIKYLEFISEAIIHVLHSRHPGDFGADAQGAMNKALELFRKDIAAKYKELGY

>1A6Q-_

MGAFLDKPKMEKHNAQGQGNGLRYGLSSMQGWRVEMEDAHTAVIGLPSGLESWSFFAVYDGHAGSQVAKYCCEHLLDHITNNQDFKGSAGAPSVENVKNGIRTGFLEIDEHMRVMSEKKHGADRSGSTAVGVLISPQHTYFINCGDSRGLLCRNRKVHFFTQDHKPSNPLEKERIQNAGGSVMIQRVNGSLAVSRALGDFDYKCVHGKGPTEQLVSPEPEVHDIERSEEDDQFIILACDGIWDVMGNEELCDFVRSRLEVTDDLEKVCNEVVDTCLYKGSRDNMSVILICFPNAPKVSPEAVKKEAELDKYLECRVEEIIKKQGEGVPDLVHVMRTLASENIPSLPPGGELASKRNVIEAVYNRLNPYKNDDTDSTSTDDMW

>1A8L-_

MGLISDADKKVIKEEFFSKMVNPVKLIVFVRKDHCQYCDQLKQLVQELSELTDKLSYEIVDFDTPEGKELAKRYRIDRAPATTITQDGKDFGVRYFGLPAGHEFAAFLEDIVDVSREETNLMDETKQAIRNIDQDVRILVFVTPTCPYCPLAVRMAHKFAIENTKAGKGKILGDMVEAIEYPEWADQYNVMAVPKIVIQVNGEDRVEFEGAYPEKMFLEKLLSALS

>1A8Y-_

EEGLDFPEYDGVDRVINVNAKNYKNVFKKYEVLALLYHEPPEDDKASQRQFEMEELILELAAQVLEDKGVGFGLVDSEKDAAVAKKLGLTEEDSIYVFKEDEVIEYDGEFSADTLVEFLLDVLEDPVELIEGERELQAFENIEDEIKLIGYFKNKDSEHYKAFKEAAEEFHPYIPFFATFDSKVAKKLTLKLNEIDFYEAFMEEPVTIPDKPNSEEEIVNFVEEHRRSTLRKLKPESMYETWEDDMDGIHIVAFAEEADPDGYEFLEILKSVAQDNTDNPDLSIIWIDPDDFPLLVPYWEKTFDIDLSAPQIGVVNVTDADSVWMEMDDEEDLPSAEELEDWLEDVLEGEINTEDDDDEDDDDDDDD

>1AAC-_

DKATIPSESPFAAAEVADGAIVVDIAKMKYETPELHVKVGDTVTWINREAMPHNVHFVAGVLGEAALKGPMMKKEQAYSLTFTEAGTYDYHCTPHPFMRGKVVVE

>1ABE-_

ENLKLGFLVKQPEEPWFQTEWKFADKAGKDLGFEVIKIAVPDGEKTLNAIDSLAASGAKGFVICTPDPKLGSAIVAKARGYDMKVIAVDDQFVNAKGKPMDTVPLVMMAATKIGERQGQELYKEMQKRGWDVKESAVMAITANELDTARRRTTGSMDALKAAGFPEKQIYQVPTKSNDIPGAFDAANSMLVQHPEVKHWLIVGMNDSTVLGGVRATEGQGFKAADIIGIGINGVDAVSELSKAQATGFYGSLLPSPDVHGYKSSEMLYNWVAKDVEPPKFTEVTDVVLITRDNFKEELEKKGLGGK

>1AC5-_

LPSSEEYKVAYELLPGLSEVPDPSNIPQMHAGHIPLRSEDADEQDSSDLEYFFWKFTNNDSNGNVDRPLIIWLNGGPGCSSMDGALVESGPFRVNSDGKLYLNEGSWISKGDLLFIDQPTGTGFSVEQNKDEGKIDKNKFDEDLEDVTKHFMDFLENYFKIFPEDLTRKIILSGESYAGQYIPFFANAILNHNKFSKIDGDTYDLKALLIGNGWIDPNTQSLSYLPFAMEKKLIDESNPNFKHLTNAHENCQNLINSASTDEAAHFSYQECENILNLLLSYTRESSQKGTADCLNMYNFNLKDSYPSCGMNWPKDISFVSKFFSTPGVIDSLHLDSDKIDHWKECTNSVGTKLSNPISKPSIHLLPGLLESGIEIVLFNGDKDLICNNKGVLDTIDNLKWGGIKGFSDDAVSFDWIHKSKSTDDSEEFSGYVKYDRNLTFVSVYNASHMVPFDKSLVSRGIVDIYSNDVMIIDNNGKNVMITT

>1AEW-_

SSQIRQNYSTEVEAAVNRLVNLYLRASYTYLSLGFYFDRDDVALEGVCHFFRELAEEKREGAERLLKMQNQRGGRALFQDLQKPSQDEWGTTPDAMKAAIVLEKSLNQALLDLHALGSAQADPHLCDFLESHFLDEEVKLIKKMGDHLTNIQRLVGSQAGLGEYLFERLTLKHD

>1AH7-_

WSAEDKHKEGVNSHLWIVNRAIDIMSRNTTLVKQDRVAQLNEWRTELENGIYAADYENPYYDNSTFASHFYDPDNGKTYIPFAKQAKETGAKYFKLAGESYKNKDMKQAFFYLGLSLHYLGDVNQPMHAANFTNLSYPQGFHSKYENFVDTIKDNYKVTDGNGYWNWKGTNPEEWIHGAAVVAKQDYSGIVNDNTKDWFVKAAVSQEYADKWRAEVTPMTGKRLMDAQRVTAGYIQLWFDTYGDR

>1AHO-_

VKDGYIVDDVNCTYFCGRNAYCNEECTKLKGESGYCQWASPYGNACYCYKLPDHVRTKGPGRCH

>1AIR-_

ATDTGGYAATAGGNVTGAVSKTATSMQDIVNIIDAARLDANGKKVKGGAYPLVITYTGNEDSLINAAAANICGQWSKDPRGVEIKEFTKGITIIGANGSSANFGIWIKKSSDVVVQNMRIGYLPGGAKDGDMIRVDDSPNVWVDHNELFAANHECDGTPDNDTTFESAVDIKGASNTVTVSYNYIHGVKKVGLDGSSSSDTGRNITYHHNYYNDVNARLPLQRGGLVHAYNNLYTNITGSGLNVRQNGQALIENNWFEKAINPVTSRYDGKNFGTWVLKGNNITKPADFSTYSITWTADTKPYVNADSWTSTGTFPTVAYNYSPVSAQCVKDKLPGYAGVGKNLATLTSTACK

>1AJJ-_

PCSAFEFHCLSGECIHSSWRCDGGPDCKDKSDEENCA

>1AL3-_

MKLQQLRYIVEVVNHNLNVSSTAEGLYTSQPGISKQVRMLEDELGIQIFARSGKHLTQVTPAGQEIIRIAREVLSKVDAIKSVAGEHTWPDKGSLYVATTHTQARYALPGVIKGFIERYPRVSLHMHQGSPTQIAEAVSKGNADFAIATEALHLYDDLVMLPCYHWNRSIVVTPEHPLATKGSVSIEELAQYPLVTYTFGFTGRSELDTAFNRAGLTPRIVFTATDADVIKTYVRLGLGVGVIASMAVDPVSDPDLVKLDANGIFSHSTTKIGFRRSTFLRSYMYDFIQRFAPHLTRDVVDTAVALRSNEDIEAMFKDIKLPEK

>1ALU-_

MAPVPPGEDSKDVAAPHRQPLTSSERIDKQIRYILDGISALRKETCNKSNMCESSKEALAENNLNLPKMAEKDGCFQSGFNEETCLVKIITGLLEFEVYLEYLQNRFESSEEQARAVQMSTKVLIQFLQKKAKNLDAITTPDPTTNASLLTKLQAQNQWLQDMTTHLILRSFKEFLQSSLRALRQM

>1ALY-_

GDQNPQIAAHVISEASSKTTSVLQWAEKGYYTMSNNLVTLENGKQLTVKRQGLYYIYAQVTFCSNREASSQAPFIASLCLKSPGRFERILLRAANTHSSAKPCGQQSIHLGGVFELQPGASVFVNVTDPSQVSHGTGFTSFGLLKL

>1AMF-_

DEGKITVFAAASLTNAMQDIATQFKKEKGVDVVSSFASSSTLARQIEAGAPADLFISADQKWMDYAVDKKAIDTATRQTLLGNSLVVVAPKASVQKDFTIDSKTNWTSLLNGGRLAVGDPEHVPAGIYAKEALQKLGAWDTLSPKLAPAEDVRGALALVERNEAPLGIVYGSDAVASKGVKVVATFPEDSHKKVEYPVAVVEGHNNATVKAFYDYLKGPQAAEIFKRYGFTIK

>1AMK-_

MSAKPQPIAAANWKCNGTTASIEKLVQVFNEHTISHDVQCVVAPTFVHIPLVQAKLRNPKYVISAENAIAKSGAFTGEVSMPILKDIGVHWVILGHSERRTYYGETDEIVAQKVSEACKQGFMVIACIGETLQQREANQTAKVVLSQTSAIAAKLTKDAWNQVVLAYEPVWAIGTGKVATPEQAQEVHLLLRKWVSENIGTDVAAKLRILYGGSVNAANAATLYAKPDINGFLVGGASLKPEFRDIIDATR

>1AMM-_

GKITFYEDRGFQGHCYECSSDCPNLQPYFSRCNSIRVDSGCWMLYERPNYQGHQYFLRRGDYPDYQQWMGFNDSIRSCRLIPQHTGTFRMRIYERDDFRGQMSEITDDCPSLQDRFHLTEVHSLNVLEGSWVLYEMPSYRGRQYLLRPGEYRRYLDWGAMNAKVGSLRRVMDFY

>1AMP-_

MPPITQQATVTAWLPQVDASQITGTISSLESFTNRFYTTTSGAQASDWIASEWQALSASLPNASVKQVSHSGYNQKSVVMTITGSEAPDEWIVIGGHLDSTIGSHTNEQSVAPGADDDASGIAAVTEVIRVLSENNFQPKRSIAFMAYAAEEVGLRGSQDLANQYKSEGKNVVSALQLDMTNYKGSAQDVVFITDYTDSNFTQYLTQLMDEYLPSLTYGFDTCGYACSDHASWHNAGYPAAMPFESKFNDYNPRIHTTQDTLANSDPTGSHAKKFTQLGLAYAIEMGSATG

>1AMX-_

MRGSHHHHHHGSITSGNKSTNVTVHKSEAGTSSVFYYKTGDMLPEDTTHVRWFLNINNEKSYVSKDITIKDQIQGGQQLDLSTLNINVTGTHSNYYSGQSAITDFEKAFPGSKITVDNTKNTIDVTIPQGYGSYNSFSINYKTKITNEQQKEFVNNSQAWYQEHGKEEVNGKSFNHTVHN

>1AOA-_

EGICALGGTSELSSEGTQHSYSEEEKYAFVNWINKALENDPDCRHVIPMNPNTDDLFKAVGDGIVLCKMINLSVPDTIDERAINKKKLTPFIIQENLNLALNSASAIGCHVVNIGAEDLRAGKPHLVLGLLWQIIKIGLFADIELSRNEALAALLRDGETLEELMKLSPEELLLRWANFHLENSGWQKINNFSADIKDSKAYFHLLNQIAPKGQKEGEPRIDINMSGFNETDDLKRAESMLQQADKLGCRQFVTPADVVSGNPKLNLAFVANLFN

>1AOL-_

QVYNITWEVTNGDRETVWAISGNHPLWTWWPVLTPDLCMLALSGPPHWGLEYQAPYSSPPGPPCCSGSSGSSAGCSRDCDEPLTSLTPRCNTAWNRLKLDQVTHKSSEGFYVCPGSHRPREAKSCGGPDSFYCASWGCETTGRVYWKPSSSWDYITVDNNLTTSQAVQVCKDNKWCNPLAIQFTNAGKQVTSWTTGHYWGLRLYVSGRDPGLTFGIRLRYQNLGPRVP

>1AQP-_

KETAAAKFERQHMDSSTSAASSSNYCNQMMKSRNLTKDRCKPVNTFVHESLADVQAVCSQKNVACKNGQTNCYQSYSTMSITDCRETGSSKYPNCAYKTTQANKHIIVACEGNPYVPVHFDASV

>1ARB-_

GVSGSCNIDVVCPEGDGRRDIIRAVGAYSKSGTLACTGSLVNNTANDRKMYFLTAHHCGMGTASTAASIVVYWNYQNSTCRAPNTPASGANGDGSMSQTQSGSTVKATYATSDFTLLELNNAANPAFNLFWAGWDRRDQNYPGAIAIHHPNVAEKRISNSTSPTSFVAWGGGAGTTHLNVQWQPSGGVTEPGSSGSPIYSPEKRVLGQLHGGPSSCSATGTNRSDQYGRVFTSWTGGGAAASRLSDWLDPASTGAQFIDGLDSGGGTP

>1ARU-_

QGPGGGGGSVTCPGGQSTSNSQCCVWFDVLDDLQTNFYQGSKCESPVRKILRIVFHDAIGFSPALTAAGQFGGGGADGSIIAHSNIELAFPANGGLTDTIEALRAVGINHGVSFGDLIQFATAVGMSNCPGSPRLEFLTGRSNSSQPSPPSLIPGPGNTVTAILDRMGDAGFSPDEVVDLLAAHSLASQEGLNSAIFRSPLDSTPQVFDTQFYIETLLKGTTQPGPSLGFAEELSPFPGEFRMRSDALLARDSRTACRWQSMTSSNEVMGQRYRAAMAKMSVLGFDRNALTDCSDVIPSAVSNNAAPVIPGGLTVDDIEVSCPSEPFPEIATASGPLPSLAPAP

>1ASH-_

ANKTRELCMKSLEHAKVDTSNEARQDGIDLYKHMFENYPPLRKYFKSREEYTAEDVQNDPFFAKQGQKILLACHVLCATYDDRETFNAYTRELLDRHARDHVHMPPEVWTDFWKLFEEYLGKKTTLDEPTKQAWHEIGREFAKEINKHGR

>1ASS-_

MSGIVIDKEKVHSKMPDVVKNAKIALIDSALEIKKTEIEAKVQISDPSKIQDFLNQETNTFKQMVEKIKKSGANVVLCQKGIDDVAQHYLAKEGIYAVRRVKKSDMEKLAKATGAKIVTDLDDLTPSVLGEAETVEERKIGDDRMTFVMGCKNHHHHHH

>1AT0-_

CFTPESTALLESGVRKPLGELSIGDRVLSXTANGQAVYSEVILFXDRNLEQXQNFVQLHTDGGAVLTVTPAHLVSVWQPESQKLTFVFADRIEEKNQVLVRDVETGELRPQRVVKVGSVRSKGVVAPLTREGTIVVNSVAASCYA

>1AUK-_

RPPNIVLIFADDLGYGDLGCYGHPSSTTPNLDQLAAGGLRFTDFYVPVSLXTPSRAALLTGRLPVRMGMYPGVLVPSSRGGLPLEEVTVAEVLAARGYLTGMAGKWHLGVGPEGAFLPPHQGFHRFLGIPYSHDQGPCQNLTCFPPATPCDGGCDQGLVPIPLLANLSVEAQPPWLPGLEARYMAFAHDLMADAQRQDRPFFLYYASHHTHYPQFSGQSFAERSGRGPFGDSLMELDAAVGTLMTAIGDLGLLEETLVIFTADNGPETMRMSRGGCSGLLRCGKGTTYEGGVREPALAFWPGHIAPGVTHELASSLDLLPTLAALAGAPLPNVTLDGFDLSPLLLGTGKSPRQSLFFYPSYPDEVRGVFAVRTGKYKAHFFTQGSAHSDTTADPACHASSSLTAHEPPLLYDLSKDPGENYNLLGGVAGATPEVLQALKQLQLLKAQLDAAVTFGPSQVARGEDPALQICCHPGCTPRPACCHCPDPHA

>1AV4-_

MTPSTIQTASPFRLASAGEISEVQGILRTAGLLGPEKRIAYLGVLDPARGAGSEAEDRRFRVFIHDVSGARPQEVTVSVTNGTVISAVELDTAATGELPVLEEEFEVVEQLLATDERWLKALAARNLDVSKVRVAPLSAGVFEYAEERGRRILRGLAFVQDFPEDSAWAHPVDGLVAYVDVVSKEVTRVIDTGVFPVPAEHGNYTDPELTGPLRTTQKPISITQPEGPSFTVTGGNHIEWEKWSLDVGFDVREGVVLHNIAFRDGDRLRPIINRASIAEMVVPYGDPSPIRSWQNYFDTGEYLVGQYANSLELGCDCLGDITYLSPVISDAFGNPREIRNGICMHEEDWGILAKHSDLWSGINYTRRNRRMVISFFTTIGNXDYGFYWYLYLDGTIEFEAKATGVVFTSAFPEGGSDNISQLAPGLGAPFHQHIFSARLDMAIDGFTNRVEEEDVVRQTMGPGNERGNAFSRKRTVLTRESEAVREADARTGRTWIISNPESKNRLNEPVGYKLHAHNQPTLLADPGSSIARRAAFATKDLWVTRYADDERYPTGDFVNQHSGGAGLPSYIAQDRDIDGQDIVVWHTFGLTHFPRVEDWPIMPVDTVGFKLRPEGFFDRSPVLDVPANPSQSGSHCHG

>1AZ9-A

SEISRQEFQRRRQALVEQMQPGSAALIFAAPEVTRSADSEYPYRQNSDFWYFTGFNEPEAVLVLIKSDDTHNHSVLFNRVRDLTAEIWFGRRLGQDAAPEKLGVDRALAFSEINQQLYQLLNGLDVVYHAQGEYAYADVIVNSALEKLRKGSRQNLTAPATMIDWRPVVHEMRLFKSPEEIAVLRRAGEITAMAHTRAMEKCRPGMFEYHLEGEIHHEFNRHGARYPSYNTIVGSGENGCILHYTENEXEMRDGDLVLIDAGCEYKGYAGDITRTFPVNGKFTQAQREIYDIVLESLETSLRLYRPGTSILEVTGEVVRIMVSGLVKLGILKGDVDELIAQNAHRPFFMHGLSHWLGLDVHDVGVYGQDRSRILEPGMVLTVEPGLYIAPDAEVPEQYRGIGIRIEDDIVITETGNENLTASVVKKPEEIEALMVAARKQ

>1B0B-_

XLSAAQKDNVKSSWAKASAAWGTAGPEFFMALFDAHDDVFAKFSGLFSGAAKGTVKNTPEMAAQAQSFKGLVSNWVDNLDNAGALEGQCKTFAANHKARGISAGQLEAAFKVLAGFMKSYGGDEGAWTAVAGALMGMIRPDM

>1B5L-_

CYLSRKLMLDARENLKLLDRMNRLSPHSCLQDRKDFGLPQEMVEGDQLQKDQAFPVLYEMLQQSFNLFYTEHSSAAWDTTLLEQLCTGLQQQLDHLDTCRGQVMGEEDSELGNMDPIVTVKKYFQGIYDYLQEKGYSDCAWEIVRVEMMRALTVSTTLQKRLTKMGGDLNSP

>1B6A-_

MAGVEEVAASGSHLNGDLDPDDREEGAASTAEEAAKKKRRKKKKSKGPSAAGEQEPDKESGASVDEVARQLERSALEDKERDEDDEDGDGDGDGATGKKKKKKKKKRGPKVQTDPPSVPICDLYPNGVFPKGQECEYPPTQDGRTAAWRTTSEEKKALDQASEEIWNDFREAAEAHRQVRKYVMSWIKPGMTMIEICEKLEDCSRKLIKENGLNAGLAFPTGCSLNNCAAHYTPNAGDTTVLQYDDICKIDFGTHISGRIIDCAFTVTFNPKYDTLLKAVKDATNTGIKCAGIDVRLCDVGEAIQEVMESYEVEIDGKTYQVKPIRNLNGHSIGQYRIHAGKTVPIVKGGEATRMEEGEVYAIETFGSTGKGVVHDDMECSHYMKNFDVGHVPIRLPRTKHLLNVINENFGTLAFCRRWLDRLGESKYLMALKNLCDLGIVDPYPPLCDIKGSYTAQFEHTILLRPTCKEVVSRGDDY

>1BA3-_

MEDAKNIKKGPAPFYPLEDGTAGEQLHKAMKRYALVPGTIAFTDAHIEVNITYAEYFEMSVRLAEAMKRYGLNTNHRIVVCSENSLQFFMPVLGALFIGVAVAPANDIYNERELLNSMNISQPTVVFVSKKGLQKILNVQKKLPIIQKIIIMDSKTDYQGFQSMYTFVTSHLPPGFNEYDFVPESFDRDKTIALIMNSSGSTGLPKGVALPHRTACVRFSHARDPIFGNQIIPDTAILSVVPFHHGFGMFTTLGYLICGFRVVLMYRFEEELFLRSLQDYKIQSALLVPTLFSFFAKSTLIDKYDLSNLHEIASGGAPLSKEVGEAVAKRFHLPGIRQGYGLTETTSAILITPEGDDKPGAVGKVVPFFEAKVVDLDTGKTLGVNQRGELCVRGPMIMSGYVNNPEATNALIDKDGWLHSGDIAYWDEDEHFFIVDRLKSLIKYKGYQVAPAELESILLQHPNIFDAGVAGLPDDDAGELPAAVVVLEHGKTMTEKEIVDYVASQVTTAKKLRGGVVFVDEVPKGLTGKLDARKIREILIKAKKGGKSKL

>1BB9-_

MGSSHHHHHHSSGLVPRGSHMATVNGAVEGSTTTGRLDLPPGFMFKVQAQHDYTATDTDELQLKAGDVVLVIPFQNPEEQDEGWLMGVKESDWNQHKELEKCRGVFPENFTERVQ

>1BD8-_

RAGDRLSGAAARGDVQEVRRLLHRELVHPDALNRFGKTALQVMMFGSTAIALELLKQGASPNVQDTSGTSPVHDAARTGFLDTLKVLVEHGADVNVPDGTGALPIHLAVQEGHTAVVSFLAAESDLHRRDARGLTPLELALQRGAQDLVDILQGHM

>1BDO-_

EISGHIVRSPMVGTFYRTPSPDAKAFIEVGQKVNVGDTLCIVEAMKMMNQIEADKSGTVKAILVESGQPVEFDEPLVVIE

>1BEA-_

SAGTSCVPGWAIPHNPLPSCRWYVTSRTCGIGPRLPWPELKRRCCRELADIPAYCRCTALSILMDGAIPPGPDAQLEGRLEDLPGCPREVQRGFAATLVTEAECNLATISGVAECPWILGGGTMPSK

>1BEO-_

TACTATQQTAAYKTLVSILSDASFNQCSTDSGYSMLTAKALPTTAQYKLMCASTACNTMIKKIVTLNPPNCDLTVPTSGLVLNVYSYANGFSNKCSSL

>1BFD-_

MASVHGTTYELLRRQGIDTVFGNPGSNELPFLKDFPEDFRYILALQEACVVGIADGYAQASRKPAFINLHSAAGTGNAMGALSNAWNSHSPLIVTAGQQTRAMIGVEALLTNVDAANLPRPLVKWSYEPASAAEVPHAMSRAIHMASMAPQGPVYLSVPYDDWDKDADPQSHHLFDRHVSSSVRLNDQDLDILVKALNSASNPAIVLGPDVDAANANADCVMLAERLKAPVWVAPSAPRCPFPTRHPCFRGLMPAGIAAISQLLEGHDVVLVIGAPVFRYHQYDPGQYLKPGTRLISVTCDPLEAARAPMGDAIVADIGAMASALANLVEESSRQLPTAAPEPAKVDQDAGRLHPETVFDTLNDMAPENAIYLNESTSTTAQMWQRLNMRNPGSYYFCAAGGLGFALPAAIGVQLAEPERQVIAVIGDGSANYSISALWTAAQYNIPTIFVIMNNGTYGALRWFAGVLEAENVPGLDVPGIDFRALAKGYGVQALKADNLEQLKGSLQEALSAKGPVLIEVSTVSPVK

>1BG6-_

MIESKTYAVLGLGNGGHAFAAYLALKGQSVLAWDIDAQRIKEIQDRGAIIAEGPGLAGTAHPDLLTSDIGLAVKDADVILIVVPAIHHASIAANIASYISEGQLIILNPGATGGALEFRKILRENGAPEVTIGETSSMLFTCRSERPGQVTVNAIKGAMDFACLPAAKAGWALEQIGSVLPQYVAVENVLHTSLTNVNAVMHPLPTLLNAARCESGTPFQYYLEGITPSVGSLAEKVDAERIAIAKAFDLNVPSVCEWYKESYGQSPATIYEAVQGNPAYRGIAGPINLNTRYFFEDVSTGLVPLSELGRAVNVPTPLIDAVLDLISSLIDTDFRKEGRTLEKLGLSGLTAAGIRSAVE

>1BGC-_

TPLGPARSLPQSFLLKCLEQVRKIQADGAELQERLCAAHKLCHPEELMLLRHSLGIPQAPLSSCSSQSLQLRGCLNQLHGGLFLYQGLLQALAGISPELAPTLDTLQLDVTDFATNIWLQMEDLGAAPAVQPTQGAMPTFTSAFQRRAGGVLVASQLHRFLELAYRGLRYLAEP

>1BHE-_

SDSRTVSEPKTPSSCTTLKADSSTATSTIQKALNNCDQGKAVRLSAGSTSVFLSGPLSLPSGVSLLIDKGVTLRAVNNAKSFENAPSSCGVVDKNGKGCDAFITAVSTTNSGIYGPGTIDGQGGVKLQDKKVSWWELAADAKVKKLKQNTPRLIQINKSKNFTLYNVSLINSPNFHVVFSDGDGFTAWKTTIKTPSTARNTDGIDPMSSKNITIAYSNIATGDDNVAIKAYKGRAETRNISILHNDFGTGHGMSIGSETMGVYNVTVDDLKMNGTTNGLRIKSDKSAAGVVNGVRYSNVVMKNVAKPIVIDTVYEKKEGSNVPDWSDITFKDVTSETKGVVVLNGENAKKPIEVTMKNVKLTSDSTWQIKNVNVKK

>1BHP-_

KSCCKSTLGRNCYNLCRARGAQKLCANVCRCKLTSGLSCPKDFPK

>1BJ7-_

QETPAEIDPSKIPGEWRIIYAAADNKDKIVEGGPLRNYYRRIECINDCESLSITFYLKDQGTCLLLTEVAKRQEGYVYVLEFYGTNTLEVIHVSENMLVTYVENYDGERITKMTEGLAKGTSFTPEELEKYQQLNSERGVPNENIENLIKTDNCPP

>1BK0-_

MGSVSKANVPKIDVSPLFGDDQAAKMRVAQQIDAASRDTGFFYAVNHGINVQRLSQKTKEFHMSITPEEKWDLAIRAYNKEHQDQVRAGYYLSIPGKKAVESFCYLNPNFTPDHPRIQAKTPTHEVNVWPDETKHPGFQDFAEQYYWDVFGLSSALLKGYALALGKEENFFARHFKPDDTLASVVLIRYPYLDPYPEAAIKTAADGTKLSFEWHEDVSLITVLYQSNVQNLQVETAAGYQDIEADDTGYLINCGSYMAHLTNNYYKAPIHRVKWVNAERQSLPFFVNLGYDSVIDPFDPREPNGKSDREPLSYGDYLQNGLVSLINKNGQT

>1BOB-_

MSANDFKPETWTSSANEALRVSIVGENAVQFSPLFTYPIYGDSEKIYGYKDLIIHLAFDSVTFKPYVNVKYSAKLGDDNIVDVEKKLLSFLPKDDVIVRDEAKWVDCFAEERKTHNLSDVFEKVSEYSLNGEEFVVYKSSLVDDFARRMHRRVQIFSLLFIEAANYIDETDPSWQIYWLLNKKTKELIGFVTTYKYWHYLGAKSFDEDIDKKFRAKISQFLIFPPYQNKGHGSCLYEAIIQSWLEDKSITEITVEDPNEAFDDLRDRNDIQRLRKLGYDAVFQKHSDLSDEFLESSRKSLKLEERQFNRLVEMLLLLNNS

>1BPI-_

RPDFCLEPPYTGPCKARIIRYFYNAKAGLCQTFVYGGCRAKRNNFKSAEDCMRTCGGA

>1BQK-_

ADFEVHMLNKGKDGAMVFEPASLKVAPGDTVTFIPTDKGHNVETIKGMIPDGAEAFKSKINENYKVTFTAPGVYGVKCTPHYGMGMVGVVQVGDAPANLEAVKGAKNPKKAQERLDAALAALGN

>1BS9-_

SCPAIHVFGARETTASPGYGSSSTVVNGVLSAYPGSTAEAINYPACGGQSSCGGASYSSSVAQGIAAVASAVNSFNSQCPSTKIVLVGYSQGGEIMDVALCGGGDPNQGYTNTAVQLSSSAVNMVKAAIFMGDPMFRAGLSYEVGTCAAGGFDQRPAGFSCPSAAKIKSYCDASDPYCCNGSNAATHQGYGSEYGSQALAFVKSKLG

>1BTN-_

MEGFLNRKHEWEAHNKKASSRSWHNVYCVINNQEMGFYKDAKSAASGIPYHSEVPVSLKEAICEVALDYKKKKHVFKLRLSDGNEYLFQAKDDEEMNTWIQAISSA

>1BV1-_

GVFNYETETTSVIPAARLFKAFILDGDNLFPKVAPQAISSVENIEGNGGPGTIKKISFPEGLPFKYVKDRVDEVDHTNFKYNYSVIEGGPIGDTLEKISNEIKIVATPDGGSILKISNKYHTKGDHEVKAEQVKASKEMGETLLRAVESYLLAHSDAYN

>1BX7-_

TQGNTCGGETCSAAQVCLKGKCVCNEVHCRIRCKYGLKKDENGCEYPCSCAKASQ

>1BYB-_

ATSDSNMLLNYVPVYVMLPLGVVNVDNVFEDPDGLKEQLLQLRAAGVDGVMVDVWWGIIELKGPKQYDWRAYRSLFQLVQECGLTLQAIMSFHQCGGNVGDIVNIPIPQWVLDIGESNHDIFYTNRSGTRNKEYLTVGVDNEPIFHGRTAIEIYSDYMKSFRENMSDFLESGLIIDIEVGLGPAGELRYPSYPQSQGWEFPRIGEFQCYDKYLKADFKAAVARAGHPEWELPDDAGKYNDVPESTGFFKSNGTYVTEKGKFFLTWYSNKLLNHGDQILDEANKAFLGCKVKLAIKVSGIHWWYKVENHAAELTAGYYNLNDRDGYRPIARMLSRHHAILNFTCLEMRDSEQPSDAKSGPQELVQQVLSGGWREDIRVAGENALPRYDATAYNQIILNAKPQGVNNNGPPKLSMFGVTYLRLSDDLLQKSNFNIFKKFVLKMHADQDYCANPQKYNHAITPLKPSAPKIPIEVLLEATKPTLPFPWLPETDMKVDG

>1C52-_

QADGAKIYAQCAGCHQQNGQGIPGAFPPLAGHVAEILAKEGGREYLILVLLYGLQGQIEVKGMKYNGVMSSFAQLKDEEIAAVLNHIATAWGDAKKVKGFKPFTAEEVKKLRAKKLTPQQVLAERKKLGLK

>1CA1-_

WDGKIDGTGTHAMIVTQGVSILENDLSKNEPESVRKNLEILKENMHELQLGSTYPDYDKNAYDLYQDHFWDPDTDNNFSKDNSWYLAYSIPDTGESQIRKFSALARYEWQRGNYKQATFYLGEAMHYFGDIDTPYHPANVTAVDSAGHVKFETFAEERKEQYKINTVGCKTNEDFYADILKNKDFNAWSKEYARGFAKTGKSIYYSHASMSHSWDDWDYAAKVTLANSQKGTAGYIYRFLHDVSEGNDPSVGKNVKELVAYISTSGEKDAGTDDYMYFGIKTKDGKTQEWEMDNPGNDFMTGSKDTYTFKLKDENLKIDDIQNMWIRKRKYTAFPDAYKPENIKVIANGKVVVDKDINEWISGNSTYNIK

>1CEC-_

MVSFKAGINLGGWISQYQVFSKEHFDTFITEKDIETIAEAGFDHVRLPFDYPIIESDDNVGEYKEDGLSYIDRCLEWCKKYNLGLVLDMHHAPGYRFQDFKTSTLFEDPNQQKRFVDIWRFLAKRYINEREHIAFELLNEVVEPDSTRWNKLMLECIKAIREIDSTMWLYIGGNNYNSPDELKNLADIDDDYIVYNFHFYNPFFFTHQKAHWSESAMAYNRTVKYPGQYEGIEEFVKNNPKYSFMMELNNLKLNKELLRKDLKPAIEFREKKKCKLYCGEFGVIAIADLESRIKWHEDYISLLEEYDIGGAVWNYKKMDFEIYNEDRKPVSQELVNILARRKT

>1CEM-_

AGVPFNTKYPYGPTSIADNQSEVTAMLKAEWEDWKSKRITSNGAGGYKRVQRDASTNYDTVSEGMGYGLLLAVCFNEQALFDDLYRYVKSHFNGNGLMHWHIDANNNVTSHDGGDGAATDADEDIALALIFADKQWGSSGAINYGQEARTLINNLYNHCVEHGSYVLKPGDRWGGSSVTNPSYFAPAWYKVYAQYTGDTRWNQVADKCYQIVEEVKKYNNGTGLVPDWCTASGTPASGQSYDYKYDATRYGWRTAVDYSWFGDQRAKANCDMLTKFFARDGAKGIVDGYTIQGSKISNNHNASFIGPVAAASMTGYDLNFAKELYRETVAVKDSEYYGYYGNSLRLLTLLYITGNFPNPLSDL

>1CFB-_

IVQDVPNAPKLTGITCQADKAEIHWEQQGDNRSPILHYTIQFNTSFTPASWDAAYEKVPNTDSSFVVQMSPWANYTFRVIAFNKIGASPPSAHSDSCTTQPDVPFKNPDNVVGQGTEPNNLVISWTPMPEIEHNAPNFHYYVSWKRDIPAAAWENNNIFDWRQNNIVIADQPTFVKYLIKVVAINDRGESNVAAEEVVGYSGEDR

>1CHD-_

LKAGPLLSSEKLIAIGASTGGTEAIRHVLQPLPLSSPAVIITQHMPPGFTRSFAERLNKLCQISVKEAEDGERVLPGHAYIAPGDKHMELARSGANYQIKIHDGPPVNRHRPSVDVLFHSVAKHAGRNAVGVILTGMGNDGAAGMLAMYQAGAWTIAQNEASCVVFGMPREAINMGGVSEVVDLSQVSQQMLAKISAGQAIRI

>1CLC-_

TMITNSRGSVDLQPSLTGVFPSGLIETKVSAAKITENYQFDSRIRLNSIGFIPNHSKKATIAANCSTFYVVKEDGTIVYTGTATSMFDNDTKETVYIADFSSVNEEGTYYLAVPGVGKSVNFKIAMNVYEDAFKTAMLGMYLLRCGTSVSATYNGIHYSHGPCHTNDAYLDYINGQHTKKDSTKGWHDAGDYNKYVVNAGITVGSMFLAWEHFKDQLEPVALEIPEKNNSIPDFLDELKYEIDWILTMQYPDGSGRVAHKVSTRNFGGFIMPENEHDERFFVPWSSAATADFVAMTAMAARIFRPYDPQYAEKCINAAKVSYEFLKNNPANVFANQSGFSTGEYATVSDADDRLWAAAEMWETLGDEEYLRDFENRAAQFSKKIEADFDWDNVANLGMFTYLLSERPGKNPALVQSIKDSLLSTADSIVRTSQNHGYGRTLGTTYYWGCNGTVVRQTMILQVANKISPNNDYVNAALDAISHVFGRNYYNRSYVTGLGINPPMNPHDRRSGADGIWEPWPGYLVGGGWPGPKDWVDIQDSYQTNEIAINWNAALIYALAGFVNYNSPQNEVLYGDVNDDGKVNSTDLTLLKRYVLKAVSTLPSSKAEKNADVNRDGRVNSSDVTILSRYLIRVIEKLPI

>1CNV-_

DISSTEIAVYWGQREDGLLRDTCKTNNYKIVFISFLDKFGCEIRKPELELEGVCGPSVGNPCSFLESQIKECQRMGVKVFLALGGPKGTYSACSADYAKDLAEYLHTYFLSERREGPLGKVALDGIHFDIQKPVDELNWDNLLEELYQIKDVYQSTFLLSAAPGCLSPDEYLDNAIQTRHFDYIFVRFYNDRSCQYSTGNIQRIRNAWLSWTKSVYPRDKNLFLELPASQATAPGGGYIPPSALIGQVLPYLPDLQTRYAGIALWNRQADKETGYSTNIIRYLNATAMPFTSNLLKYPS

>1CPO-_

XEPGSGIGYPYDNNTLPYVAPGPTDSRAPCPALNALANHGYIPHDGRAISRETLQNAFLNHMGIANSVIELALTNAFVVCEYVTGSDCGDSLVNLTLLAEPHAFEHDHSFSRKDYKQGVANSNDFIDNRNFDAETFQTSLDVVAGKTHFDYADMNEIRLQRESLSNELDFPGWFTESKPIQNVESGFIFALVSDFNLPDNDENPLVRIDWWKYWFTNESFPYHLGWHPPSPAREIEFVTSASSAVLAASVTSTPSSLPSGAIGPGAEAVPLSFASTMTPFLLATNAPYYAQDPTLGPND

>1CPQ-_

ADTKEVLEAREAYFKSLGGSMKAMTGVAKAFDAEAAKVEAAKLEKILATDVAPLFPAGTSSTDLPGQTEAKAAIWANMDDFGAKGKAMHEAGGAVIAAANAGDGAAFGAALQKLGGTCKACHDDYREED

>1CTJ-_

EADLALGKAVFDGNCAACHAGGGNNVIPDHTLQKAAIEQFLDGGFNIEAIVYQIENGKGAMPAWDGRLDEDEIAGVAAYVYDQAAGNKW

>1CTT-_

MHPRFQTAFAQLADNLQSALEPILADKYFPALLTGEQVSSLKSATGLDEDALAFALLPLAAACARTPLSNFNVGAIARGVSGTWYFGANMEFIGATMQQTVHAEQSAISHAWLSGEKALAAITVNYTPCGHCRQFMNELNSGLDLRIHLPGREAHALRDYLPDAFGPKDLEIKTLLMDEQDHGYALTGDALSQAAIAAANRSHMPYSKSPSGVALECKDGRIFSGSYAENAAFNPTLPPLQGALILLNLKGYDYPDIQRAVLAEKADAPLIQWDATSATLKALGCHSIDRVLLA

>1CV8-_

YNEQYVNKLENFKIRETQGNNGWCAGYTMSALLNATYNTNKYHAEAVMRFLHPNLQGQQFQFTGLTPREMIYFGQTQGRSPQLLNRMTTYNEVDNLTKNNKGIAILGSRVESRNGMHAGHAMAVVGNAKLNNGQEVIIIWNPWDNGFMTQDAKNNVIPVSNGDHYQWYSSIYGY

>1CVL-_

ADTYAATRYPVILVHGLAGTDKFANVVDYWYGIQSDLQSHGAKVYVANLSGFQSDDGPNGRGEQLLAYVKQVLAATGATKVNLIGHSQGGLTSRYVAAVAPQLVASVTTIGTPHRGSEFADFVQDVLKTDPTGLSSTVIAAFVNVFGTLVSSSHNTDQDALAALRTLTTAQTATYNRNFPSAGLGAPGSCQTGAATETVGGSQHLLYSWGGTAIQPTSTVLGVTGATDTSTGTLDVANVTDPSTLALLATGAVMINRASGQNDGLVSRCSSLFGQVISTSYHWNHLDEINQLLGVRGANAEDPVAVIRTHVNRLKLQGV

>1CYO-_

SKAVKYYTLEEIQKHNNSKSTWLILHYKVYDLTKFLEEHPGGEEVLREQAGGDATENFEDVGHSTDARELSKTFIIGELHPDDRSKITKPSES

>1DDT-_

GADDVVDSSKSFVMENFSSYHGTKPGYVDSIQKGIQKPKSGTQGNYDDDWKGFYSTDNKYDAAGYSVDNENPLSGKAGGVVKVTYPGLTKVLALKVDNAETIKKELGLSLTEPLMEQVGTEEFIKRFGDGASRVVLSLPFAEGSSSVEYINNWEQAKALSVELEINFETRGKRGQDAMYEYMAQACAGNRVRRSVGSSLSCINLDWDVIRDKTKTKIESLKEHGPIKNKMSESPNKTVSEEKAKQYLEEFHQTALEHPELSELKTVTGTNPVFAGANYAAWAVNVAQVIDSETADNLEKTTAALSILPGIGSVMGIADGAVHHNTEEIVAQSIALSSLMVAQAIPLVGELVDIGFAAYNFVESIINLFQVVHNSYNRPAYSPGHKTQPFLHDGYAVSWNTVEDSIIRTGFQGESGHDIKITAENTPLPIAGVLLPTIPGKLDVNKSKTHISVNGRKIRMRCRAIDGDVTFCRPKSPVYVGNGVHANLHVAFHRSSSEKIHSNEISSDSIGVLGYQKTVDHTKVNSKLSLFFEIKS

>1DFX-_

PKHLEVYKCTHCGNIVEVLHGGGAELVCCGEPMKHMVEGSTDGAMEKHVPVIEKVDGGYLIKVGSVPHPMEEKHWIEWIELLADGRSYTKFLKPGDAPEAFFAIDASKVTAREYCNLHGHWKAEN

>1DHN-_

MQDTIFLKGMRFYGYHGALSAENEIGQIFKVDVTLKVDLSEAGRTDNVIDTVHYGEVFEEVKSIMEGKAVNLLEHLAERIANRINSQYNRVMETKVRITKENPPIPGHYDGVGIEIVRENK

>1DHR-_

MAASGEARRVLVYGGRGALGSRCVQAFRARNWWVASIDVVENEEASASVIVKMTDSFTEQADQVTAEVGKLLGDQKVDAILCVAGGWAGGNAKSKSLFKNCDLMWKQSIWTSTISSHLATKHLKEGGLLTLAGAKAALDGTPGMIGYGMAKGAVHQLCQSLAGKNSGMPSGAAAIAVLPVTLDTPMNRKSMPEADFSSWTPLEFLVETFHDWITGNKRPNSGSLIQVVTTDGKTELTPAYF

>1DIN-_

MLTEGISIQSYDGHTFGALVGSPAKAPAPVIVIAQEIFGVNAFMRETVSWLVDQGYAAVCPDLYARQAPGTALDPQDERQREQAYKLWQAFDMEAGVGDLEAAIRYARHQPYSNGKVGLVGYXLGGALAFLVAAKGYVDRAVGYYGVGLEKQLNKVPEVKHPALFHMGGQDHFVPAPSRQLITEGFGANPLLQVHWYEEAGHSFARTSSSGYVASAAALANERTLDFLAPLQSKKP

>1DOI-_

PTVEYLNYEVVDDNGWDMYDDDVFGEASDMDLDDEDYGSLEVNEGEYILEAAEAQGYDWPFSCRAGACANCAAIVLEGDIDMDMQQILSDEEVEDKNVRLTCIGSPDADEVKIVYNAKHLDYLQNRVI

>1DPE-_

KTLVYCSEGSPEGFNPQLFISGTTYDASSVPLYNRLVEFKIGTTEVIPGLAEKWEVSEDGKTYTFHLRKGVKWHDNKEFKPTRELNADDVVFSFDRQKNAQNPYHKVSGGSYEYFEGMGLPELISEVKKVDDNTVQFVLTRPEAPFLADLAMDFASILSKEYADAMMKAGTPEKLDLNPIGTGPFQLQQYQKDSRIRYKAFDGYWGTKPQIDTLVFSITPDASVRYAKLQKNECQVMPYPNPADIARMKQDKSINLMEMPGLNVGYLSYNVQKKPLDDVKVRQALTYAVNKDAIIKAVYQGAGVSAKNLIPPTMWGYNDDVQDYTYDPEKAKALLKEAGLEKGFSIDLWAMPVQRPYNPNARRMAEMIQADWAKVGVQAKIVTYEWGEYLKRAKDGEHQTVMMGWTGDNGDPDNFFATEFSCAASEQGSNYSKWCYKPFEDLIQPARATDDHNKRVELYKQAQVVMHDQAPALIIAHSTVFEPVRKEVKGYVVDPLGKHHFENVSIE

>1DUN-_

MLAYQGTQIKEKRDEDAGFDLCVPYDIMIPVSDTKIIPTDVKIQVPPNSFGWVTGKSSMAKQGLLINGGIIDEGYTGEIQVICTNIGKSNIKLIEGQKFAQLIILQHHSNSRQPWDENKISQRGDKGFGSTGVF

>1DXY-_

MKIIAYGARVDEIQYFKQWAKDTGNTLEYHTEFLDENTVEWAKGFDGINSLQTTPYAAGVFEKMHAYGIKFLTIRNVGTDNIDMTAMKQYGIRLSNVPAYSPAAIAEFALTDTLYLLRNMGKVQAQLQAGDYEKAGTFIGKELGQQTVGVMGTGHIGQVAIKLFKGFGAKVIAYDPYPMKGDHPDFDYVSLEDLFKQSDVIDLHVPGIEQNTHIINEAAFNLMKPGAIVINTARPNLIDTQAMLSNLKSGKLAGVGIDTYEYETEDLLNLAKHGSFKDPLWDELLGMPNVVLSPHIAYYTETAVHNMVYFSLQHLVDFLTKGETSTEVTGPAK

>1EAF-_

IPPIPPVDFAKYGEIEEVPMTRLMQIGATNLHRSWLNVPHVTQFESADITELEAFRVAQKAVAKKAGVKLTVLPLLLKACAYLLKELPDFNSSLAPSGQALIRKKYVHIGFAVDTPDGLLVPVIRNVDQKSLLQLAAEAAELAEKARSKKLGADAMQGACFTISSLGHIGGTAFTPIVNAPEVAILGVSKASMQPVWDGKAFQPRLMLPLSLSYDHRVINGAAAARFTKRLGDLLADIRAILL

>1ECY-_

AESVQPLEKIAPYPQAEKGMKRQVIQLTPQEDESTLKVELLIGQTLEVDCNLHRLGGKLENKTLEGWGYDYYVFDKVSSPVSTMMACPDGKKEKKFVTAYLGDAGMLRYNSKLPIVVYTPDNVDVKYRVWKAEEKIDNAVVR

>1EDG-_

MYDASLIPNLQIPQKNIPNNDGMNFVKGLRLGWNLGNTFDAFNGTNITNELDYETSWSGIKTTKQMIDAIKQKGFNTVRIPVSWHPHVSGSDYKISDVWMNRVQEVVNYCIDNKMYVILNTHHDVDKVKGYFPSSQYMASSKKYITSVWAQIAARFANYDEHLIFEGMNEPRLVGHANEWWPELTNSDVVDSINCINQLNQDFVNTVRATGGKNASRYLMCPGYVASPDGATNDYFRMPNDISGNNNKIIVSVHAYCPWNFAGLAMADGGTNAWNINDSKDQSEVTWFMDNIYNKYTSRGIPVIIGECGAVDKNNLKTRVEYMSYYVAQAKARGILCILWDNNNFSGTGELFGFFDRRSCQFKFPEIIDGMVKYAFGLIN

>1ESC-_

APADPVPTVFFGDSYTANFGIAPVTNQDSERGWCFQAKENYPAVATRSLADKGITLDVQADVSCGGALIHHFWEKQELPFGAGELPPQQDALKQDTQLTVGSLGGNTLGFNRILKQCSDELRKPSLLPGDPVDGDEPAAKCGEFFGTGDGKQWLDDQFERVGAELEELLDRIGYFAPDAKRVLVGYPRLVPEDTTKCLTAAPGQTQLPFADIPQDALPVLDQIQKRLNDAMKKAAADGGADFVDLYAGTGANTACDGADRGIGGLLEDSQLELLGTKIPWYAHPNDKGRDIQAKQVADKIEEILNR

>1EUR-_

VPPGGEPLYTEQDLAVNGREGFPNYRIPALTVTPDGDLLASYDGRPTGIDAPGPNSILQRRSTDGGRTWGEQQVVSAGQTTAPIKGFSDPSYLVDRETGTIFNFHVYSQRQGFAGSRPGTDPADPNVLHANVATSTDGGLTWSHRTITADITPDPGWRSRFAASGEGIQLRYGPHAGRLIQQYTIINAAGAFQAVSVYSDDHGRTWRAGEAVGVGMDENKTVELSDGRVLLNSRDSARSGYRKVAVSTDGGHSYGPVTIDRDLPDPTNNASIIRAFPDAPAGSARAKVLLFSNAASQTSRSQGTIRMSCDDGQTWPVSKVFQPGSMSYSTLTALPDGTYGLLYEPGTGIRYANFNLAWLGGICAP

>1EZM-_

AEAGGPGGNQKIGKYTYGSDYGPLIVNDRCEMDDGNVITVDMNSSTDDSKTTPFRFACPTNTYKQVNGAYSPLNDAHFFGGVVFKLYRDWFGTSPLTHKLYMKVHYGRSVENAYWDGTAMLFGDGATMFYPLVSLDVAAHEVSHGFTEQNSGLIYRGQSGGMNEAFSDMAGEAAEFYMRGKNDFLIGYDIKKGSGALRYMDQPSRDGRSIDNASQYYNGIDVHHSSGVYNRAFYLLANSPGWDTRKAFEVFVDANRYYWTATSNYNSGACGVIRSAQNRNYSAADVTRAFSTVGVTCPSAL

>1FCE-_

ASSPANKVYQDRFESMYSKIKDPANGYFSEQGIPYHSIETLMVEAPDYGHVTTSEAMSYYMWLEAMHGRFSGDFTGFDKSWSVTEQYLIPTEKDQPNTSMSRYDANKPATYAPEFQDPSKYPSPLDTSQPVGRDPINSQLTSAYGTSMLYGMHWILDVDNWYGFGARADGTSKPSYINTFQRGEQESTWETIPQPCWDEHKFGGQYGFLDLFTKDTGTPAKQFKYTNAPDADARAVQATYWADQWAKEQGKSVSTSVGKATKMGDYLRYSFFDKYFRKIGQPSQAGTGYDAAHYLLSWYYAWGGGIDSTWSWIIGSSHNHFGYQNPFAAWVLSTDANFKPKSSNGASDWAKSLDRQLEFYQWLQSAEGAIAGGATNSWNGRYEAVPSGTSTFYGMGYVENPVYADPGSNTWFGMQVWSMQRVAELYYKTGDARAKKLLDKWAKWINGEIKFNADGTFQIPSTIDWEGQPDTWNPTQGYTGNANLHVKVVNYGTDLGCASSLANTLTYYAAKSGDETSRQNAQKLLDAMWNNYSDSKGISTVEQRGDYHRFLDQEVFVPAGWTGKMPNGDVIKSGVKFIDIRSKYKQDPEWQTMVAALQAGQVPTQRLHRFWAQSEFAVANGVYAILFPD

>1FKJ-_

GVQVETISPGDGRTFPKRGQTCVVHYTGMLEDGKKFDSSRDRNKPFKFMLGKQEVIRGWEEGVAQMSVGQRAKLTISPDYAYGATGHPGIIPPHATLVFDVELLKLE

>1FUA-_

MERNKLARQIIDTCLEMTRLGLNQGTAGNVSVRYQDGMLITPTGIPYEKLTESHIVFIDGNGKHEEGKLPSSEWRFHMAAYQSRPDANAVVHNHAVHCTAVSILNRSIPAIHYMIAAAGGNSIPCAPYATFGTRELSEHVALALKNRKATLLQHHGLIACEVNLEKALWLAHEVEVLAQLYLTTLAITDPVPVLSDEEIAVVLEKFKTYGLRIEE

>1FUS-_

XSATTCGSTNYSASQVRAAANAACQYYQNDDTAGSSTYPHTYNNYEGFDFPVDGPYQEFPIKSGGVYTGGSPGADRVVINTNCEYAGAITHTGASGNNFVGCSGTN

>1G3P-_

AETVESCLAKSHTENSFTNVXKDDKTLDRYANYEGCLWNATGVVVCTGDETQCYGTWVPIGLAIPENEGGGSEGGGSEGGGSEGGGTKPPEYGDTPIPGYTYINPLDGTYPPGTEQNPANPNPSLEESQPLNTFMFQNNRFRNRQGALTVYTGTVTQGTDPVKTYYQYTPVSSKAMYDAYWNGKFRDCAFHSGFNEDIFVCEYQGQSSDLPQPPVNA

>1GAI-_

ATLDSWLSNEATVARTAILNNIGADGAWVSGADSGIVVASPSTDNPDYFYTWTRDSGLVIKTLVDLFRNGDTDLLSTIEHYISSQAIIQGVSNPSGDLSSGGLGEPKFNVDETAYTGSWGRPQRDGPALRATAMIGFGQWLLDNGYTSAATEIVWPLVRNDLSYVAQYWNQTGYDLWEEVNGSSFFTIAVQHRALVEGSAFATAVGSSCSWCDSQAPQILCYLQSFWTGSYILANFDSSRSGKDTNTLLGSIHTFDPEAGCDDSTFQPCSPRALANHKEVVDSFRSIYTLNDGLSDSEAVAVGRYPEDSYYNGNPWFLCTLAAAEQLYDALYQWDKQGSLEITDVSLDFFKALYSGAATGTYSSSSSTYSSIVSAVKTFADGFVSIVETHAASNGSLSEQFDKSDGDELSARDLTWSYAALLTANNRRNSVVPPSWGETSASSVPGTCAATSASGTYSSVTVTSWPSIVATG

>1GCA-_

ADTRIGVTIYKYDDNFMSVVRKAIEKDGKSAPDVQLLMNDSQNDQSKQNDQIDVLLAKGVKALAINLVDPAAAGTVIEKARGQNVPVVFFNKEPSRKALDSYDKAYYVGTDSKESGVIQGDLIAKHWQANQGWDLNKDGKIQYVLLKGEPGHPDAEARTTYVVKELNDKGIQTEQLALDTAMWDTAQAKDKMDAWLSGPNANKIEVVIANNDAMAMGAVEALKAHNKSSIPVFGVDALPEALALVKSGAMAGTVLNDANNQAKATFDLAKNLAEGKGAADGTSWKIENKIVRVPYVGVDKDNLSEFTQK

>1GEN-_

ELYGASPDIDLGTGPTPTLGPVTPEICKQDIVFDGIAQIRGEIFFFKDRFIWRTVTPRDKPMGPLLVATFWPELPEKIDAVYEAPQEEKAVFFAGNEYWIYSASTLERGYPKPLTSLGLPPDVQRVDAAFNWSKNKKTYIFAGDKFWRYNEVKKKMDPGFPKLIADAWNAIPDNLDAVVDLQGGGHSYFFKGAYYLKLENQSLKSVKFGSIKSDWLGC

>1GOF-_

ASAPIGSAISRNNWAVTCDSAQSGNECNKAIDGNKDTFWHTFYGANGDPKPPHTYTIDMKTTQNVNGLSMLPRQDGNQNGWIGRHEVYLSSDGTNWGSPVASGSWFADSTTKYSNFETRPARYVRLVAITEANGQPWTSIAEINVFQASSYTAPQPGLGRWGPTIDLPIVPAAAAIEPTSGRVLMWSSYRNDAFGGSPGGITLTSSWDPSTGIVSDRTVTVTKHDMFCPGISMDGNGQIVVTGGNDAKKTSLYDSSSDSWIPGPDMQVARGYQSSATMSDGRVFTIGGSWSGGVFEKNGEVYSPSSKTWTSLPNAKVNPMLTADKQGLYRSDNHAWLFGWKKGSVFQAGPSTAMNWYYTSGSGDVKSAGKRQSNRGVAPDAMCGNAVMYDAVKGKILTFGGSPDYQDSDATTNAHIITLGEPGTSPNTVFASNGLYFARTFHTSVVLPDGSTFITGGQRRGIPFEDSTPVFTPEIYVPEQDTFYKQNPNSIVRVYHSISLLLPDGRVFNGGGGLCGDCTTNHFDAQIFTPNYLYNSNGNLATRPKITRTSTQSVKVGGRITISTDSSISKASLIRYGTATHTVNTDQRRIPLTLTNNGGNSYSFQVPSDSGVALPGYWMLFVMNSAGVPSVASTIRVTQ

>1GPR-_

MIAEPLQNEIGEEVFVSPITGEIHPITDVPDQVFSGKMMGDGFAILPSEGIVVSPVRGKILNVFPTKHAIGLQSDGGREILIHFGIDTVSLKGEGFTSFVSEGDRVEPGQKLLEVDLDAVKPNVPSLMTPIVFTNLAEGETVSIKASGSVNREQEDIVKIEK

>1GSA-_

MIKLGIVMDPIANINIKKDSSFAMLLEAQRRGYELHYMEMGDLYLINGEARAHTRTLNVKQNYEEWFSFVGEQDLPLADLDVILMRKDPPFDTEFIYATYILERAEEKGTLIVNKPQSLRDCNEKLFTAWFSDLTPETLVTRNKAQLKAFWEKHSDIILKPLDGMGGASIFRVKEGDPNLGVIAETLTEHGTRYCMAQNYLPAIKDGDKRVLVVDGEPVPYCLARIPQGGETRGNLAAGGRGEPRPLTESDWKIARQIGPTLKEKGLIFVGLDIIGDRLTEINVTSPTCIREIEAEFPVSITGMLMDAIEARLQQQ

>1GYM-_

ASSVNELENWSKWMQPIPDSIPLARISIPGTHDSGTFKLQNPIKQVWGMTQEYDFRYQMDHGARIFDIRGRLTDDNTIVLHHGPLYLYVTLHEFINEAKQFLKDNPSETIIMSLKKEYEDMKGAEDSFSSTFEKKYFVDPIFLKTEGNIKLGDARGKIVLLKRYSGSNEPGGYNNFYWPDNETFTTTVNQNANVTVQDKYKVSYDEKVKSIKDTMDETMNNSEDLNHLYINFTSLSSGGTAWNSPYYYASYINPEIANYIKQKNPARVGWVIQDYINEKWSPLLYQEVIRANKSLIKE

>1HFC-_

VLTEGNPRWEQTHLTYRIENYTPDLPRADVDHAIEKAFQLWSNVTPLTFTKVSEGQADIMISFVRGDHRDNSPFDGPGGNLAHAFQPGPGIGGDAHFDEDERWTNNFREYNLHRVAAHELGHSLGLSHSTDIGALMYPSYTFSGDVQLAQDDIDGIQAIYGRSQNPVQP

>1HKA-_

TVAYIAIGSNLASPLEQVNAALKALGDIPESHILTVSSFYRTPPLGPQDQPDYLNAAVALETSLAPEELLNHTQRIELQQGRVRKAERWGPRTLDLDIMLFGNEVINTERLTVPHYDMKNRGFMLWPLFEIAPELVFPDGEMLRQILHTRAFDKLNKW

>1HOE-_

DTTVSEPAPSCVTLYQSWRYSQADNGCAETVTVKVVYEDDTEGLCYAVAPGQITTVGDGYIGSHGHARYLARCL

>1HPM-_

MSKGPAVGIDLGTTYSCVGVFQHGKVEIIANDQGNRTTPSYVAFTDTERLIGDAAKNQVAMNPTNTVFDAKRLIGRRFDDAVVQSDMKHWPFMVVNDAGRPKVQVEYKGETKSFYPEEVSSMVLTKMKEIAEAYLGKTVTNAVVTVPAYFNDSQRQATKDAGTIAGLNVLRIINEPTAAAIAYGLDKKVGAERNVLIFDLGGGTFDVSILTIEDGIFEVKSTAGDTHLGGEDFDNRMVNHFIAEFKRKHKKDISENKRAVRRLRTACERAKRTLSSSTQASIEIDSLYEGIDFYTSITRARFEELNADLFRGTLDPVEKALRDAKLDKSQIHDIVLVGGSTRIPKIQKLLQDFFNGKELNKSINPDEAVAYGAAVQAAILSGDKSE

>1HTP-_

SNVLDGLKYAPSHEWVKHEGSVATIGITDHAQDHLGEVVFVELPEPGVSVTKGKGFGAVESVKATSDVNSPISGEVIEVNTGLTGKPGLINSSPYEDGWMIKIKPTSPDELESLLGAKEYTKFCEEEDAAH

>1HXN-_

HRNSTQHGHESTRCDPDLVLSAMVSDNHGATYVFSGSHYWRLDTNRDGWHSWPIAHQWPQGPSTVDAAFSWEDKLYLIQDTKVYVFLTKGGYTLVNGYPKRLEKELGSPPVISLEAVDAAFVCPGSSRLHIMAGRRLWWLDLKSGAQATWTELPWPHEKVDGALCMEKPLGPNSCSTSGPNLYLIHGPNLYCYRHVDKLNAAKNLPQPQRVSRLLGCTH

>1HYP-_

ALITRPSCPDLSICLNILGGSLGTVDDCCALIGGLGDIEAIVCLCIQLRALGILNLNRNLQLILNSCGRSYPSNATCPRT

>1IAE-_

AAILGDEYLWSGGVIPYTFAGVSGADQSAILSGMQELEEKTCIRFVPRTTESDYVEIFTSGSGCWSYVGRISGAQQVSLQANGCVYHGTIIHELMHAIGFYHEHTRMDRDNYVTINYQNVDPSMTSNFDIDTYSRYVGEDYQYYSIMHYGKYSFSIQWGVLETIVPLQNGIDLTDPYDKAHMLQTDANQINNLYTNECSL

>1IFC-_

MAFDGTWKVDRNENYEKFMEKMGINVVKRKLGAHDNLKLTITQEGNKFTVKESSNFRNIDVVFELGVDFAYSLADGTELTGTWTMEGNKLVGKFKRVDNGKELIAVREISGNELIQTYTYEGVEAKRIFKKE

>1INP-_

MSDILQELLRVSEKAANIARACRQQETLFQLLIEEKKEGEKNKKFAVDFKTLADVLVQEVIKENMENKFPGLGKKIFGEESNELTNDLGEKIIMRLGPTEEETVALLSKVLNGNKLASEALAKVVHQDVFFSDPALDSVEINIPQDILGIWVDPIDSTYQYIKGSADITPNQGIFPSGLQCVTVLIGVYDIQTGVPLMGVINQPFVSQDLHTRRWKGQCYWGLSYLGTNIHSLLPPVSTRSNSEAQSQGTQNPSSEGSCRFSVVISTSEKETIKGALSHVCGERIFRAAGAGYKSLCVILGLADIYIFSEDTTFKWDSCAAHAILRAMGGGMVDLKECLERNPDTGLDLPQLVYHVGNEGAAGVDQWANKGGLIAYRSEKQLETFLSRLLQHLAPVATHT

>1IOV-_

MTDKIAVLLGGTSAEREVSLNSGAAVLAGLREGGIDAYPVDPKEVDVTQLKSMGFQKVFIALHGRGGEDGTLQGMLELMGLPYTGSGVMASALSMDKLRSKLLWQGAGLPVAPWVALTRAEFEKGLSDKQLAEISALGLPVIVKPSREGSSVGMSKVVAENALQDALRLAFQHDEEVLIEKWLSGPEFTVAILGEEILPSIRIQPSGTFYDYEAKYLSDETQYFCPAGLEASQEANLQALVLKAWTTLGCKGWGRIDVMLDSDGQFYLLEANTSPGMTSHSLVPMAARQAGMSFSQLVVRILELAD

>1JDW-_

MLRVRCLRGGSRGAEAVHYIGSRLGRTLTGWVQRTFQSTQAATASSRNSCAADDKATEPLPKDCPVSSYNEWDPLEEVIVGRAENACVPPFTIEVKANTYEKYWPFYQKQGGHYFPKDHLKKAVAEIEEMCNILKTEGVTVRRPDPIDWSLKYKTPDFESTGLYSAMPRDILIVVGNEIIEAPMAWRSRFFEYRAYRSIIKDYFHRGAKWTTAPKPTMADELYNQDYPIHSVEDRHKLAAQGKFVTTEFEPCFDAADFIRAGRDIFAQRSQVTNYLGIEWMRRHLAPDYRVHIISFKDPNPMHIDATFNIIGPGIVLSNPDRPCHQIDLFKKAGWTIITPPTPIIPDDHPLWMSSKWLSMNVLMLDEKRVMVDANEVPIQKMFEKLGITTIKVNIRNANSLGGGFHCWTCDVRRRGTLQSYLD

>1JER-_

MQSTVHIVGDNTGWSVPSSPNFYSQWAAGKTFRVGDSLQFNFPANAHNVHEMETKQSFDACNFVNSDNDVERTSPVIERLDELGMHYFVCTVGTHCSNGQKLSINVVAANATVSMPPPSSSPPSSVMPPPVMPPPSPS

>1KLO-_

CPCPGGSSCAIVPKTKEVVCTHCPTGTAGKRCELCDDGYFGDPLGSNGPVRLCRPCQCNDNIDPNAVGNCNRLTGECLKCIYNTAGFYCDRCKEGFFGNPLAPNPADKCKACACNPYGTVQQQSSCNPVTGQCQCLPHVSGRDCGTCDPGYYNLQSGQGCER

>1KOE-_

QPVLHLVALNTPLSGGMRGIRGADFQCFQQARAVGLSGTFRAFLSSRLQDLYSIVRRADRGSVPIVNLKDEVLSPSWDSLFSGSQGQLQPGARIFSFDGRDVLRHPAWPQKSVWHGSDPSGRRLMESYCETWRTETTGATGQASSLLSGRLLEQKAASCHNSYIVLCIENSF

>1KPF-_

XADEIAKAQVARPGGDTIFGKIIRKEIPAKIIFEDDRCLAFHDISPQAPTHFLVIPKKHISQISVAEDDDESLLGHLMIVGKKCAADLGLNKGYRMVVNEGSDGGQSVYHVHLHVLGGRQMHWPPG

>1KTE-_

AQAFVNSKIQPGKVVVFIKPTCPFCRKTQELLSQLPFKEGLLEFVDITATSDTNEIQDYLQQLTGARTVPRVFIGKECIGGCTDLESMHKRGELLTRLQQVGAVK

>1KUH-_

TVTVTYDPSNAPSFQQEIANAAQIWNSSVRNVQLRAGGNADFSYYEGNDSRGSYAQTDGHGRGYIFLDYQQNQQYDSTRVTAHETGHVLGLPDHYQGPCSELMSGGGPGPSCTNPYPNAQERSRVNALWANG

>1LBU-_

DGCYTWSGTLSEGSSGEAVRQLQIRVAGYPGTGAQLAIDGQFGPATKAAVQRFQSAYGLAADGIAGPATFNKIYQLQDDDCTPVNFTYAELNRCNSDWSGGKVSAATARANALVTMWKLQAMRHAMGDKPITVNGGFRSVTCNSNVGGASNSRHMYGHAADLGAGSQGFCALAQAARNHGFTEILGPGYPGHNDHTHVAGGDGRFWSAPSCGI

>1LCL-_

MSLLPVPYTEAASLSTGSTVTIKGRPLVCFLNEPYLQVDFHTEMKEESDIVFHFQVCFGRRVVMNSREYGAWKQQVESKNMPFQDGQEFELSISVLPDKYQVMVNGQSSYTFDHRIKPEAVKMVQVWRDISLTKFNVSYLKR

>1LED-_

XNTVNFTYPDFWSYSLKNGTEITFLGDATRIPGALQLTKTDANGNPVRSSAGQASYSEPVFLWDSTGKAASFYTSFTFLLKNYGAPTADGLAFFLAPVDSSVKDYGGFLGLFRHETAADPSKNQVVAVEFDTWINKDWNDPPYPHIGIDVNSIVSVATTRWENDDAYGSSIATAHITYDARSKILTVLLSYEHGRDYILSHVVDLAKVLPQKVRIGFSAGVGYDEVTYILSWHFFSTLDGTNK

>1LIT-_

QEAQTELPQARISCPEGTNAYRSYCYYFNEDRETWVDADLYCQNMNSGNLVSVLTQAEGAFVASLIKESGTDDFNVWIGLHDPKKNRRWHWSSGSLVSYKSWGIGAPSSVNPGYCVSLTSSTGFQKWKDVPCEDKFSFVCKFKN

>1LST-_

ALPQTVRIGTDTTYAPFSSKDAKGEFIGFDIDLGNEMCKRMQVKCTWVASDFDALIPSLKAKKIDAIISSLSITDKRQQEIAFSDKLYAADSRLIAAKGSPIQPTLESLKGKHVGVLQGSTQEAYANDNWRTKGVDVVAYANQDLIYSDLTAGRLDAALQDEVAASEGFLKQPAGKEYAFAGPSVKDKKYFGDGTGVGLRKDDTELKAAFDKALTELRQDGTYDKMAKKYFDFNVYGDK

>1LTM-_

EPQHNVMQMGGDFANNPNAQQFIDKMVNKHGFDRQQLQEILSQAKRLDSVLRLMDNQAPTTSVKPPSGPNGAWLRYRKKFITPDNVQNGVVFWNQYEDALNRAWQVYGVPPEIIVGIIGVETRWGRVMGKTRILDALATLSFNYPRRAEYFSGELETFLLMARDEQDDPLNLKGSFAGAMGYGQFMPSSYKQYAVDFSGDGHINLWDPVDAIGSVANYFKAHGWVKGDQVAVMANGQAPGLPNGFKTKYSISQLAAAGLTPQQPLGNHQQASLLRLDVGTGYQYWYGLPNFYTITRYNHSTHYAMAVWQLGQAVALARVQ

>1MAT-_

MAISIKTPEDIEKMRVAGRLAAEVLEMIEPYVKPGVSTGELDRICNDYIVNEQHAVSACLGYHGYPKSVCISINEVVCHGIPDDAKLLKDGDIVNIDVTVIKDGFHGDTSKMFIVGKPTIMGERLCRITQESLYLALRMVKPGINLREIGAAIQKFVEAEGFSVVREYCGHGIGRGFHEEPQVLHYDSRETNVVLKPGMTFTIEPMVNAGKKEIRTMKDGWTVKTKDRSLSAQYEHTIVVTDNGCEILTLRKDDTIPAIISHDE

>1MAZ-_

MSMAMSQSNRELVVDFLSYKLSQKGYSWSQFSDVEENRTEAPEGTESEMETPSAINGNPSWHLADSPAVNGATGHSSSLDAREVIPMAAVKQALREAGDEFELRYRRAFSDLTSQLHITPGTAYQSFEQVVNELFRDGVNWGRIVAFFSFGGALCVESVDKEMQVLVSRIAAWMATYLNDHLEPWIQENGGWDTFVELYGNNAAAESRKGQERLEHHHHHH

>1MBA-_

XSLSAAEADLAGKSWAPVFANKNANGLDFLVALFEKFPDSANFFADFKGKSVADIKASPKLRDVSSRIFTRLNEFVNNAANAGKMSAMLSQFAKEHVGFGVGSAQFENVRSMFPGFVASVAAPPAGADAAWTKLFGLIIDALKAAGA

>1MLA-_

MTQFAFVFPGQGSQTVGMLADMAASYPIVEETFAEASAALGYDLWALTQQGPAEELNKTWQTQPALLTASVALYRVWQQQGGKAPAMMAGHSLGEYSALVCAGVIDFADAVRLVEMRGKFMQEAVPEGTGAMAAIIGLDDASIAKACEEAAEGQVVSPVNFNSPGQVVIAGHKEAVERAGAACKAAGAKRALPLPVSVPSHCALMKPAADKLAVELAKITFNAPTVPVVNNVDVKCETNGDAIRDALVRQLYNPVQWTKSVEYMAAQGVEHLYEVGPGKVLTGLTKRIVDTLTASALNEPSAMAAALEL

>1MOQ-_

DAGDKGIYRHYMQKEIYEQPNAIKNTLTGRISHGQVDLSELGPNADELLSKVEHIQILACGTSYNSGMVSRYWFESLAGIPCDVEIASEFRYRKSAVRRNSLMITLSQSGETADTLAGLRLSKELGYLGSLAICNVPGSSLVRESDLALMTNAGTEIGVASTKAFTTQLTVLLMLVAKLSRLKGLDASIEHDIVHGLQALPSRIEQMLSQDKRIEALAEDFSDKHHALFLGRGDQYPIALEGALKLKEISYIHAEAYAAGELKHGPLALIDADMPVIVVAPNNELLEKLKSNIEEVRARGGQLYVFADQDAGFVSSDNMHIIEMPHVEEVIAPIFYTVPLQLLAYHVALIKGTDVDQPRNLAKSVTVE

>1MPP-_

AEGDGSVDTPGLYDFDLEEYAIPVSIGTPGQDFYLLFDTGSSDTWVPHKGCDNSEGCVGKRFFDPSSSSTFKETDYNLNITYGTGGANGIYFRDSITVGGATVKQQTLAYVDNVSGPTAEQSPDSELFLDGIFGAAYPDNTAMEAEYGDTYNTVHVNLYKQGLISSPVFSVYMNTNDGGGQVVFGGVNNTLLGGDIQYTDVLKSRGGYFFWDAPVTGVKIDGSDAVSFDGAQAFTIDTGTNFFIAPSSFAEKVVKAALPDATESQQGYTVPCSKYQDSKTTFSLVLQKSGSSSDTIDVSVPISKMLLPVDKSGETCMFIVLPDGGNQFIVGNLFLRFFVNVYDFGKNRIGFAPLASGYEND

>1MSK-_

KKPRTPPVTLEAARDNDFAFDWQAYTPPVAHRLGVQEVEASIETLRNYIDWTPFFMTWSLAGKYPRILEDEVVGVEAQRLFKDANDMLDKLSAEKTLNPRGVVGLFPANRVGDDIEIYRDETRTHVINVSHHLRQQTEKTGFANYCLADFVAPKLSGKADYIGAFAVTGGLEEDALADAFEAQHDDYNKIMVKALADRLAEAFAEYLHERVRKVYWGYAPNENLSNEELIRENYQGIRPAPGYPACPEHTEKATIWELLEVEKHTGMKLTESFAMWPGASVSGWYFSHPDSKYYAVAQIQRDQVEDYARRKGMSVTEVERWLAPNLGYDAD

>1MUP-_

CVHAEEASSTGRNFNVEKINGEWHTIILASDKREKIEDNGNFRLFLEQIHVLENSLVLKFHTVRDEECSELSMVADKTEKAGEYSVTYDGFNTFTIPKTDYDNFLMAHLINEKDGETFQLMGLYGREPDLSSDIKERFAQLCEEHGILRENIIDLSNANRCLQARE

>1NAR-_

PKPIFREYIGVKPNSTTLHDFPTEIINTETLEFHYILGFAIESYYESGKGTGTFEESWDVELFGPEKVKNLKRRHPEVKVVISIGGRGVNTPFDPAEENVWVSNAKESLKLIIQKYSDDSGNLIDGIDIHYEHIRSDEPFATLMGQLITELKKDDDLNINVVSIAPSENNSSHYQKLYNAKKDYINWVDYQFSNQQKPVSTDDAFVEIFKSLEKDYHPHKVLPGFSTDPLDTKHNKITRDIFIGGCTRLVQTFSLPGVFFWNANDSVIPKRDGDKPFIVELTLQQLLAAR

>1NEU-_

IVVYTDREVYGAVGSQVTLHCSFWSSEWVSDDISFTWRYQPEGGRDAISIFHYAKGQPYIDEVGTFKERIQWVGDPSWKDGSIVIHNLDYSDNGTFTCDVKNPPDIVGKTSQVTLYVFEKVPTR

>1NFP-_

MTKWNYGVFFLNFYHVGQQEPSLTMSNALETLRIIDEDTSIYDVVAFSEHHIDKSYNDETKLAPFVSLGKQIHVLATSPETVVKAAKYGMPLLFKWDDSQQKRIELLNHYQAAAAKFNVDIANVRHRLMLFVNVNDNPTQAKAELSIYLEDYLSYTQAETSIDEIINSNAAGNFDTCLHHVAEMAQGLNNKVDFLFCFESMKDQENKKSLMINFDKRVINYRKEHNLN

>1NG1-_

MFQQLSARLQEAIGRLRGRGRITEEDLKATLREIRRALMDADVNLEVARDFVERVREEALGKQVLESLTPAEVILATVYEALKEALGGEARLPVLKDRNLWFLVGLQGSGKTTTAAKLALYYKGKGRRPLLVAADTQRPAAREQLRLLGEKVGVPVLEVMDGESPESIRRRVEEKARLEARDLILVDTAGRLQIDEPLMGELARLKEVLGPDEVLLVLDAMTGQEALSVARAFDEKVGVTGLVLTKLDGDARGGAALSARHVTGKPIYFAGVSEKPEGLEPFYPERLAGRILGM

>1NIF-_

AAGAAPVDISTLPRVKVDLVKPPFVHAHDQVAKTGPRVVEFTMTIEEKKLVIDREGTEIHAMTFNGSVPGPLMVVHENDYVELRLINPDTNTLLHNIDFHAATGALGGGALTQVNPGEETTLRFKATKPGVFVYHCAPEGMVPWHVTSGMNGAIMVLPRDGLKDEKGQPLTYDKIYYVGEQDFYVPKDEAGNYKKYETPGEAYEDAVKAMRTLTPTHIVFNGAVGALTGDHALTAAVGERVLVVHSQANRDTRPHLIGGHGDYVWATGKFRNPPDLDQETWLIPGGTAGAAFYTFRQPGVYAYVNHNLIEAFELGAAGHFKVTGEWNDDLMTSVVKPASM

>1NKR-_

MHEGVHRKPSLLAHPGPLVKSEETVILQCWSDVMFEHFLLHREGMFNDTLRLIGEHHDGVSKANFSISRMTQDLAGTYRCYGSVTHSPYQVSAPSDPLDIVIIGLYEKPSLSAQPGPTVLAGENVTLSCSSRSSYDMYHLSREGEAHERRLPAGPKVNGTFQADFPLGPATHGGTYRCFGSFHDSPYEWSKSSDPLLVSVT

>1NLS-_

ADTIVAVELDTYPNTDIGDPSYPHIGIDIKSVRSKKTAKWNMQNGKVGTAHIIYNSVDKRLSAVVSYPNADSATVSYDVDLDNVLPEWVRVGLSASTGLYKETNTILSWSFTSKLKSNSTHETNALHFMFNQFSKDQKDLILQGDATTGTDGNLELTRVSSNGSPQGSSVGRALFYAPVHIWESSAVVASFEATFTFLIKSPDSHPADGIAFFISNIDSSIPSGSTGRLLGLFPDAN

>1NNC-_

RDFNNLTKGLCTINSWHIYGKDNAVRIGEDSDVLVTREPYVSCDPDECRFYALSQGTTIRGKHSNGTIHDRSQYRALISWPLSSPPTVYNSRVECIGWSSTSCHDGKTRMSICISGPNNNASAVIWYNRRPVTEINTWARNILRTQESECVCHNGVCPVVFTDGSATGPAETRIYYFKEGKILKWEPLAGTAKHIEECSCYGERAEITCTCRDNWQGSNRPVIRIDPVAMTHTSQYICSPVLTDNPRPNDPTVGKCNDPYPGNNNNGVKGFSYLDGVNTWLGRTISIASRSGYEMLKVPNALTDDKSKPTQGQTIVLNTDWSGYSGSFMDYWAEGECYRACFYVELIRGRPKEDKVWWTSNSIVSMCSSTEFLGQWDWPDGAKIEYFL

>1NOX-_

MEATLPVLDAKTAALKRRSIRRYRKDPVPEGLLREILEAALRAPSAWNLQPWRIVVVRDPATKRALREAAFGQAHVEEAPVVLVLYADLEDALAHLDEVIHPGVQGERREAQKQAIQRAFAAMGQEARKAWASGQSYILLGYLLLLLEAYGLGSVPMLGFDPERVRAILGLPSRAAIPALVALGYPAEEGYPSHRLPLERVVLWR

>1NPK-_

STNKVNKERTFLAVKPDGVARGLVGEIIARYEKKGFVLVGLKQLVPTKDLAESHYAEHKERPFFGGLVSFITSGPVVAMVFEGKGVVASARLMIGVTNPLASAPGSIRGDFGVDVGRNIIHGSDSVESANREIALWFKPEELLTEVKPNPNLYE

>1OBR-_

DFPSYDSGYHNYNEMVNKINTVASNYPNIVKKFSIGKSYEGRELWAVKISDNVGTDENEPEVLYTALHHAREHLTVEMALYTLDLFTQNYNLDSRITNLVNNREIYIVFNINPDGGEYDISSGSYKSWRKNRQPNSGSSYVGTDLNRNYGYKWGCCGGSSGSPSSETYRGRSAFSAPETAAMRDFINSRVVGGKQQIKTLITFHTYSELILYPYGYTYTDVPSDMTQDDFNVFKTMANTMAQTNGYTPQQASDLYITDGDMTDWAYGQHKIFAFTFEMYPTSYNPGFYPPDEVIGRETSRNKEAVLYVAEKADCPYSVIGKSCSTK

>1OPS-_

SQSVVATQLIPMNTALTPAMMEGKVTNPIGIPFAEMSQLVGKQVNTPVAKGQTLMPNMVKTYAA

>1OPY-_

MNLPTAQEVQGLMARYIELVDVGDIEAIVQMYADDATVEDPFGQPPIHGREQIAAFYRQGLGGGKVRACLTGPVRASHNGCGAMPFRVEMVWNGQPCALDVIDVMRFDEHGRIQTMQAYWSEVNLSVREPQ

>1OSA-_

AEQLTEEQIAEFKEAFALFDKDGDGTITTKELGTVMRSLGQNPTEAELQDMINEVDADGNGTIDFPEFLSLMARKMKEQDSEEELIEAFKVFDRDGNGLISAAELRHVMTNLGEKLTDDEVDEMIREADIDGDGHINYEEFVRMMVSK

>1PDO-_

TIAIVIGTHGWAAEQLLKTAEMLLGEQENVGWIDFVPGENAETLIEKYNAQLAKLDTTKGVLFLVDTWGGSPFNAASRIVVDKEHYEVIAGVNIPMLVETLMARDDDPSFDELVALAVETGREGVKALKAKPFAG

>1PEA-_

MGSHQERPLIGLLFSETGVTADIERSQRYGALLAVEQLNREGGVGGRPIETLSQDPGGDPDRYRLCAEDFIRNRGVRFLVGCYMSHTRKAVMPVVERADALLCYPTPYEGFEYSPNIVYGGPAPNQNSAPLAAYLIRHYGERVVFIGSDYIYPRESNHVMRHLYRQHGGTVLEEIYIPLYPSDDDLQRAVERIYQARADVVFSTVVGTGTAELYRAIARRYGDGRRPPIASLTTSEAEVAKMESDVAEGQVVVAPYFSSIDTPASRAFVQACHGFFPENATITAWAEAAYWQTLLLGRAAQAAGNWRVEDVQRHLYDIDIDAPQGPVRVERQNNHSRLSSRIAEIDARGVFQVRWQSPEPIRPDPYVVVHNLDDWSASMGGGPLP

>1PGS-_

APADNTVNIKTFDKVKNAFGDGLSQSAEGTFTFPADVTTVKTIKMFIKNECPNKTCDEWDRYANVYVKNKTTGEWYEIGRFITPYWVGTEKLPRGLEIDVTDFKSLLSGNTELKIYTETWLAKGREYSVDFDIVYGTPDYKYSAVVPVIQYNKSSIDGVPYGKAHTLGLKKNIQLPTNTEKAYLRTTISGWGHAKPYDAGSRGCAEWCFRTHTIAINNANTFQHQLGALGCSANPINNQSPGNWTPDRAGWCPGMAVPTRIDVLNNSLTGSTFSYEYKFQSWTNNGTNGDAFYAISSFVIAKSNTPISAPVVTN

>1PHD-_

TTETIQSNANLAPLPPHVPEHLVFDFDMYNPSNLSAGVQEAWAVLQESNVPDLVWTRCNGGHWIATRGQLIREAYEDYRHFSSECPFIPREAGEAYDFIPTSMDPPEQRQFRALANQVVGMPVVDKLENRIQELACSLIESLRPQGQCNFTEDYAEPFPIRIFMLLAGLPEEDIPHLKYLTDQMTRPDGSMTFAEAKEALYDYLIPIIEQRRQKPGTDAISIVANGQVNGRPITSDEAKRMCGLLLVGGLDTVVNFLSFSMEFLAKSPEHRQELIERPERIPAACEELLRRFSLVADGRILTSDYEFHGVQLKKGDQILLPQMLSGLDERENACPMHVDFSRQKVSHTTFGHGSHLCLGQHLARREIIVTLKEWLTRIPDFSIAPGAQIQHKSGIVSGVQALPLVWDPATTKAV

>1PHK-_

TRDAALPGSHSTHGFYENYEPKEILGRGVSSVVRRCIHKPTCKEYAVKIIDVTGGGSFSAEEVQELREATLKEVDILRKVSGHPNIIQLKDTYETNTFFFLVFDLMKKGELFDYLTEKVTLSEKETRKIMRALLEVICALHKLNIVHRDLKPENILLDDDMNIKLTDFGFSCQLDPGEKLREVCGTPSYLAPEIIECSMNDNHPGYGKEVDMWSTGVIMYTLLAGSPPFWHRKQMLMLRMIMSGNYQFGSPEWDDYSDTVKDLVSRFLVVQPQKRYTAEEALAHPFFQQYVVEEVRHF

>1PHM-_

NECLGTIGPVTPLDASDFALDIRMPGVTPKESDTYFCMSMRLPVDEEAFVIDFKPRASMDTVHHMLLFGCNMPSSTGSYWFCDEGTCTDKANILYAWARNAPPTRLPKGVGFRVGGETGSKYFVLQVHYGDISAFRDNHKDCSGVSVHLTRVPQPLIAGMYLMMSVDTVIPPGEKVVNADISCQYKMYPMHVFAYRVHTHHLGKVVSGYRVRNGQWTLIGRQNPQLPQAFYPVEHPVDVTFGDILAARCVFTGEGRTEATHIGGTSSDEMCNLYIMYYMEAKYALSFMTCTKNVAPDMFRTIPAEANIPI

>1PHR-_

AEQVTKSVLFVCLGNICRSPIAEAVFRKLVTDQNISDNWVIDSGAVSDWNVGRSPDPRAVSCLRNHGINTAHKARQVTKEDFVTFDYILCMDESNLRDLNRKSNQVKNCRAKIELLGSYDPQKQLIIEDPYYGNDADFETVYQQCVRCCRAFLEKVR

>1PHT-_

MSAEGYQYRALYDYKKEREEDIDLHLGDILTVNKGSLVALGFSDGQEARPEEIGWLNGYNETTGERGDFPGTYVEYIGRKKISPP

>1PLC-_

IDVLLGADDGSLAFVPSEFSISPGEKIVFKNNAGFPHNIVFDEDSIPSGVDASKISMSEEDLLNAKGETFEVALSNKGEYSFYCSPHQGAGMVGKVTVN

>1PMI-_

SSEKLFRIQCGYQNYDWGKIGSSSAVAQFVHNSDPSITIDETKPYAELWMGTHPSVPSKAIDLNNQTLRDLVTAKPQEYLGESIITKFGSSKELPFLFKVLSIEKVLSIQAHPDKKLGAQLHAADPKNYPDDNHKPEMAIAVTDFEGFCGFKPLDQLAKTLATVPELNEIIGQELVDEFISGIKLPAEVGSQDDVNNRKLLQKVFGKLMNTDDDVIKQQTAKLLERTDREPQVFKDIDSRLPELIQRLNKQFPNDIGLFCGCLLLNHVGLNKGEAMFLQAKDPHAYISGDIIECMAASDNVVRAGFTPKFKDVKNLVEMLTYSYESVEKQKMPLQEFPRSKGDAVKSVLYDPPIAEFSVLQTIFDKSKGGKQVIEGLNGPSIVIATNGKGTIQITGDDSTKQKIDTGYVFFVAPGSSIELTADSANQDQDFTTYRAFVEA

>1PNE-_

XAGWNAYIDNLMADGTCQDAAIVGYKDSPSVWAAVPGKTFVNITPAEVGILVGKDRSSFFVNGLTLGGQKCSVIRDSLLQDGEFTMDLRTKSTGGAPTFNITVTMTAKTLVLLMGKEGVHGGMINKKCYEMASHLRRSQY

>1POA-_

NLYQFKNMIQCTVPSRSWWDFADYGCYCGRGGSGTPVDDLDRCCQVHDNCYNEAEKISGCWPYFKTYSYECSQGTLTCKGGNNACAAAVCDCDRLAAICFAGAPYNDNDYNINLKARC

>1POC-_

IIYPGTLWCGHGNKSSGPNELGRFKHTDACCRTHDMCPDVMSAGESKHGLTNTASHTRLSCDCDDKFYDCLKNSADTISSYFVGKMYFNLIDTKCYKLEHPVTGCGERTEGRCLHYTVDKSKPKVYQWFDLRKY

>1POT-_

DDNNTLYFYNWTEYVPPGLLEQFTKETGIKVIYSTYESNETMYAKLKTYKDGAYDLVVPSTYYVDKMRKEGMIQKIDKSKLTNFSNLDPDMLNKPFDPNNDYSIPYIWGATAIGVNGDAVDPKSVTSWADLWKPEYKGSLLLTDDAREVFQMALRKLGYSGNTTDPKEIEAAYNELKKLMPNVAAFNSDNPANPYMEGEVNLGMIWNGSAFVARQAGTPIDVVWPKEGGIFWMDSLAIPANAKNKEGALKLINFLLRPDVAKQVAETIGYPTPNLAARKLLSPEVANDKTLYPDAETIKNGEWQNDVGAASSIYEEYYQKLKAGR

>1PRN-_

EISLNGYGRFGLQYVEDRGVGLEDTIISSRLRINIVGTTETDQGVTFGAKLRMQWDDGDAFAGTAGNAAQFWTSYNGVTVSVGNVDTAFDSVALTYDSEMGYEASSFGDAQSSFFAYNSKYDASGALDNYNGIAVTYSISGVNLYLSYVDPDQTVDSSLVTEEFGIAADWSNDMISLAAAYTTDAGGIVDNDIAFVGAAYKFNDAGTVGLNWYDNGLSTAGDQVTLYGNYAFGATTVRAYVSDIDRAGADTAYGIGADYQFAEGVKVSGSVQSGFANETVADVGVRFDF

>1PTF-_

MEKKEFHIVAETGIHARPATLLVQTASKFNSDINLEYKGKSVNLKSIMGVMSLGVGQGSDVTITVDGADEAEGMAAIVETLQKEGLAE

>1PUC-_

SKSGVPRLLTASERERLEPFIDQIHYSPRYADDEYEYRHVMLPKAMLKAIPTDYFNPETGTLRILQEEEWRGLGITQSLGWEMYEVHVPEPHILLFKREKDYQMK

>1RA9-_

MISLIAALAVDRVIGMENAMPWNLPADLAWFKRNTLDKPVIMGRHTWESIGRPLPGRKNIILSSQPGTDDRVTWVKSVDEAIAACGDVPEIMVIGGGRVYEQFLPKAQKLYLTHIDAEVEGDTHFPDYEPDDWESVFSEFHDADAQNSHSYCFEILERR

>1RB9-_

XMKKYVCTVCGYEYDPAEGDPDNGVKPGTSFDDLPADWVCPVCGAPKSEFEAA

>1RCY-_

TTWKEATLPQVKAMLEKDDGKVSGDTVTYSGKTVHVVAAAVLPGFPFPSFEVHDKKNPTLEIPAGATVDVTFINTNKGFGHSFDITKKGPPYAVMPVIDPIVAGTGFSPVPKDGKFGYTDFTWHPTAGTYYYVCQIPGHAATGMFGKIVVK

>1REC-_

GNSKSGALSKEILEELQLNTKFTEEELSSWYQSFLKECPSGRITRQEFQTIYSKFFPEADPKAYAQHVFRSFDANSDGTLDFKEYVIALHMTSAGKTNQKLEWAFSLYDVDGNGTISKNEVLEIVTAIFKMISPEDTKHLPEDENTPEKRAEKIWGFFGKKDDDKLTEKEFIEGTLANKEILRLIQFEPQKVKEKLKEKKL

>1RFS-_

FVPPGGGAGTGGTIAKDALGNDVIAAEWLKTHAPGDRTLTQGLKGDPTYLVVESDKTLATFGINAVCTHLGCVVPFNAAENKFICPCHGSQYNNQGRVVRGPAPLSLALAHCDVDDGKVVFVPWTETDFRTGEAPWWSA

>1RH4-_

XAALAQXKKEIAYLLAKXKAEILAALKKXKQEIAX

>1RHS-_

VHQVLYRALVSTKWLAESVRAGKVGPGLRVLDASWYSPGTREARKEYLERHVPGASFFDIEECRDKASPYEVMLPSEAGFADYVGSLGISNDTHVVVYDGDDLGSFYAPRVWWMFRVFGHRTVSVLNGGFRNWLKEGHPVTSEPSRPEPAIFKATLNRSLLKTYEQVLENLESKRFQLVDSRAQGRYLGTQPEPDAVGLDSGHIRGSVNMPFMNFLTEDGFEKSPEELRAMFEAKKVDLTKPLIATXRKGVTACHIALAAYLCGKPDVAIYDGSWFEWFHRAPPETWVSQGKGGKA

>1RIE-_

VLAMSKIEIKLSDIPEGKNMAFKWRGKPLFVRHRTKKEIDQEAAVEVSQLRDPQHDLERVKKPEWVILIGVCTHLGCVPIANAGDFGGYYCPCHGSHYDASGRIRKGPAPLNLEVPSYEFTSDDMVIVG

>1RKD-_

MQNAGSLVVLGSINADHILNLQSFPTPGETVTGNHYQVAFGGKGANQAVAAGRSGANIAFIACTGDDSIGESVRQQLATDNIDITPVSVIKGESTGVALIFVNGEGENVIGIHAGANAALSPALVEAQRERIANASALLMQLESPLESVMAAAKIAHQNKTIVALNPAPARELPDELLALVDIITPNETEAEKLTGIRVENDEDAAKAAQVLHEKGIRTVLITLGSRGVWASVNGEGQRVPGFRVQAVDTIAAGDTFNGALITALLEEKPLPEAIRFAHAAAAIAVTRKGAQPSVPWREEIDAFLDRQR

>1RMG-_

QLSGSVGPLTSASTKGATKTCNILSYGAVADNSTDVGPAITSAWAACKSGGLVYIPSGNYALNTWVTLTGGSATAIQLDGIIYRTGTASGNMIAVTDTTDFELFSSTSKGAVQGFGYVYHAEGTYGARILRLTDVTHFSVHDIILVDAPAFHFTMDTCSDGEVYNMAIRGGNEGGLDGIDVWGSNIWVHDVEVTNKDECVTVKSPANNILVESIYCNWSGGCAMGSLGADTDVTDIVYRNVYTWSSNQMYMIKSNGGSGTVSNVLLENFIGHGNAYSLDIDGYWSSMTAVAGDGVQLNNITVKNWKGTEANGATRPPIRVVCSDTAPCTDLTLEDIAIWTESGSSELYLCRSAYGSGYCLKDSSSHTSYTTTSTVTAAPSGYSATTMAADLATAFGLTASIPIPTIPTSFYPGLTPYSALAG

>1RSY-_

GGGILDSMVEKLGKLQYSLDYDFQNNQLLVGIIQAAELPALDMGGTSDPYVKVFLLPDKKKKFETKVHRKTLNPVFNEQFTFKVPYSELGGKTLVMAVYDFDRFSKHDIIGEFKVPMNTVDFGHVTEEWRDLQSA

>1RZL-_

ITCGQVNSAVGPCLTYARGGAGPSAACCSGVRSLKAAASTTADRRTACNCLKNAARGIKGLNAGNAASIPSKCGVSVPYTISASIDCSRVS

>1SBP-_

KDIQLLNVSYDPTRELYEQYNKAFSAHWKQETGDNVVIDQSHGGSGKQATSVINGIEADTVTLALAYDVNAIAERGRIDKNWIKRLPDDSAPYTSTIVFLVRKGNPKQIHDWNDLIKPGVSVITPNPKSSGGARWNYLAAWGYALHHNNNDQAKAEDFVKALFKNVEVLDSGARGSTNTFVERGIGDVLIAWENEALLATNELGKDKFEIVTPSESILAEPTVSVVDKVVEKKDTKAVAEAYLKYLYSPEGQEIAAKNFYRPRDADVAKKYDDAFPKLKLFTIDEVFGGWAKAQKDHFADGGTFDQISKR

>1SEK-_

MAGETDLQKILRESNDQFTAQMFSEVVKANPGQNVVLSAFSVLPPLGQLALASVGESHDELLRALALPNDNVTKDVFADLNRGVRAVKGVDLKMASKIYVAKGLELNDDFAAVSRDVFGSEVQNVDFVKSVEAAGAINKWVEDQTNNRIKNLVDPDALDETTRSVLVNAIYFKGSWKDKFNKERTMDRDFHVSKDKTIKVPTMIGKKDVRYADVPELDAKMIEMSYEGDQASMIIILPNQVDGITALEQKLKDPKALSRAEERLYNTEVEIYLPKFKIETTTDLKEVLSNMNIKKLFTPGAARLENLLKTKESLYVDAAIQKAFIEVNEEGAEAAAANAFKITTYSFHFVPKVEINKPFFFSLKYNRNSMFSGVCVQP

>1SFP-_

MDWLPRNTNCGGILKEESGVIATYYGPKTNCVWTIQMPPEYHVRVSIQYLQLNCNKESLEIIDGLPGSPVLGKICEGSLMDYRSSGSIMTVKYIREPEHPASFYEVLYFQDPQA

>1SMD-_

XYSSNTQQGRTSIVHLFEWRWVDIALECERYLAPKGFGGVQVSPPNENVAIHNPFRPWWERYQPVSYKLCTRSGNEDEFRNMVTRCNNVGVRIYVDAVINHMCGNAVSAGTSSTCGSYFNPGSRDFPAVPYSGWDFNDGKCKTGSGDIENYNDATQVRDCRLSGLLDLALGKDYVRSKIAEYMNHLIDIGVAGFRIDASKHMWPGDIKAILDKLHNLNSNWFPEGSKPFIYQEVIDLGGEPIKSSDYFGNGRVTEFKYGAKLGTVIRKWNGEKMSYLKNWGEGWGFMPSDRALVFVDNHDNQRGHGAGGASILTFWDARLYKMAVGFMLAHPYGFTRVMSSYRWPRYFENGKDVNDWVGPPNDNGVTKEVTINPDTTCGNDWVCEHRWRQIRNMVNFRNVVDGQPFTNWYDNGSNQVAFGRGNRGFIVFNNDDWTFSLTLQTGLPAGTYCDVISGDKINGNCTGIKIYVSDDGKAHFSISNSAEDPFIAIHAESKL

>1SRA-_

PPCLDSELTEFPLRMRDWLKNVLVTLYERDEDNNLLTEKQKLRVKKIHENEKRLEAGDHPVELLARDFEKNYNMYIFPVHWQFGQLDQHPIDGYLSHTELAPLRAPLIPMEHCTTRFFETCDLDNDKYIALDEWAGCFGIKQKDIDKDLVI

>1SVB-_

SRCTHLENRDFVTGTQGTTRVTLVLELGGCVTITAEGKPSMDVWLDAIYQENPAKTREYCLHAKLSDTKVAARCPTMGPATLAEEHQGGTVCKRDQSDRGWGNHCGLFGKGSIVACVKAACEAKKKATGHVYDANKIVYTVKVEPHTGDYVAANETHSGRKTASFTISSEKTILTMGEYGDVSLLCRVASGVDLAQTVILELDKTVEHLPTAWQVHRDWFNDLALPWKHEGAQNWNNAERLVEFGAPHAVKMDVYNLGDQTGVLLKALAGVPVAHIEGTKYHLKSGHVTCEVGLEKLKMKGLTYTMCDKTKFTWKRAPTDSGHDTVVMEVTFSGTKPCRIPVRAVAHGSPDVNVAMLITPNPTIENNGGGFIEMQLPPGDNIIYVGELSHQWFQK

>1SVY-_

SGFNHVKPTEYKPRLLHISGDKNAKVAEVPLATSSLNSGDCFLLDAGLTIYQFNGSKSSPQEKNKAAEVARAIDAERKGLPKVEVFXETDSDIPAEFWKLLGGKGAIAAKHETA

>1TCA-_

LPSGSDPAFSQPKSVLDAGLTCQGASPSSVSKPILLVPGTGTTGPQSFDSNWIPLSTQLGYTPCWISPPPFMLNDTQVNTEYMVNAITALYAGSGNNKLPVLTWSQGGLVAQWGLTFFPSIRSKVDRLMAFAPDYKGTVLAGPLDALAVSAPSVWQQTTGSALTTALRNAGGLTQIVPTTNLYSATDEIVQPQVSNSPLDSSYLFNGKNVQAQAVCGPLFVIDHAGSLTSQFSYVVGRSALRSTTGQARSADYGITDCNPLPANDLTPEQKVAAAALLAPAAAAIVAGPKQNCEPDLMPYARPFAVGKRTCSGIVTP

>1TDE-_

GTTKHSKLLILGSGPAGYTAAVYAARANLQPVLITGMEKGGQLTTTTEVENWPGDPNDLTGPLLMERMHEHATKFETEIIFDHINKVDLQNRPFRLNGDNGEYTCDALIIATGASARYLGLPSEEAFKGRGVSACATCDGFFYRNQKVAVIGGGNTAVEEALYLSNIASEVHLIHRRDGFRAEKILIKRLMDKVENGNIILHTNRTLEEVTGDQMGVTGVRLRDTQNSDNIESLDVAGLFVAIGHSPNTAIFEGQLELENGYIKVQSGIHGNATQTSIPGVFAAGDVMDHIYRQAITSAGTGCMAALDAERYLDGL

>1TEN-_

RLDAPSQIEVKDVTDTTALITWFKPLAEIDGIELTYGIKDVPGDRTTIDLTEDENQYSIGNLKPDTEYEVSLISRRGDMSSNPAKETFTT

>1TFE-_

AREGIIGHYIHHNQRVGVLVELNCETDFVARNELFQNLAKDLAMHIAMMNPRYVSAEEIPAEELEKERQIYIQAALNEGKPQQIAEKIAEGRLKKYLEEVVLLEQPFVKDDKVKVKELIQQAIAKIGENIVVRRFCRFELGAMMC

>1THV-_

ATFEIVNRCSYTVWAAASKGDAALDAGGRQLNSGESWTINVEPGTNGGKIWARTDCYFDDSGSGICKTGDCGGLLRCKRFGRPPTTLAEFSLNQYGKDYIDISNIKGFNVPMDFSPTTRGCRGVRCAADIVGQCPAKLKAPGGGCNDACTVFQTSEYCCTTGKCGPTEYSRFFKRLCPDAFSYVLDKPTTVTCPGSSNYRVTFCPTA

>1TML-_

NDSPFYVNPNMSSAEWVRNNPNDPRTPVIRDRIASVPQGTWFAHHNPGQITGQVDALMSAAQAAGKIPILVVYNAPGRDCGNHSSGGAPSHSAYRSWIDEFAAGLKNRPAYIIVEPDLISLMSSCMQHVQQEVLETMAYAGKALKAGSSQARIYFDAGHSAWHSPAQMASWLQQADISNSAHGIATNTSNYRWTADEVAYAKAVLSAIGNPSLRAVIDTSRNGNGPAGNEWCDPSGRAIGTPSTTNTGDPMIDAFLWIKLPGEADGCIAGAGQFVPQAAYEMAIAA

>1TMY-_

MGKRVLIVDDAAFMRMMLKDIITKAGYEVAGEATNGREAVEKYKELKPDIVTMDITMPEMNGIDAIKEIMKIDPNAKIIVCSAMGQQAMVIEAIKAGAKDFIVKPFQPSRVVEALNKVSK

>1TN3-_

ALQTVCLKGTKVHMKCFLAFTQTKTFHEASEDCISRGGTLSTPQTGSENDALYEYLRQSVGNEAEIWLGLNDMAAEGTWVDMTGARIAYKNWETEITAQPDGGKTENCAVLSGAANGKWFDKRCRDQLPYICQFGIV

>1TON-_

IVGGYKCEKNSQPWQVAVINEYLCGGVLIDPSWVITAAHCYSNNYQVLLGRNNLFKDEPFAQRRLVRQSFRHPDYIPLIVTNDTEQPVHDHSNDLMLLHLSEPADITGGVKVIDLPTKEPKVGSTCLASGWGSTNPSEMVVSHDLQCVNIHLLSNEKCIETYKDNVTDVMLCAGEMEGGKDTCAGDSGGPLICDGVLQGITSGGATPCAKPKTPAIYAKLIKFTSWIKKVMKENP

>1TRY-_

IVGGTSASAGDFPFIVSISRNGGPWCGGSLLNANTVLTAAHCVSGYAQSGFQIRAGSLSRTSGGITSSLSSVRVHPSYSGNNNDLAILKLSTSIPSGGNIGYARLAASGSDPVAGSSATVAGWGATSEGGSSTPVNLLKVTVPIVSRATCRAQYGTSAITNQMFCAGVSSGGKDSCQGDSGGPIVDSSNTLIGAVSWGNGCARPNYSGVYASVGALRSFIDTYA

>1TUL-_

MASMSNGTPDIIVNAQINSEDENVLDFIIEDEYYLKKRGVGAHIIKVASSPQLRLLYKNAYSTVSCGNYGVLCNLVQNGEYDLNAIMFNCAEIKLNKGQMLFQTKIWR

>1UCH-_

MEGQRWLPLEANPEVTNQFLKQLGLHPNWQFVDVYGMDPELLSMVPRPVCAVLLLFPITEKYEVFRTEEEEKIKSQGQDVTSSVYFMKQTISNACGTIGLIHAIANNKDKMHFESGSTLKKFLEESVSMSPEERARYLENYDAIRVTHETSAHEGQTEAPSIDEKVDLHFIALVHVDGHLYELDGRKPFPINHGETSDETLLEDAIEVCKKFMERDPDELRFNAIALSAA

>1UOK-_

MEKQWWKESVVYQIYPRSFMDSNGDGIGDLRGIISKLDYLKELGIDVIWLSPVYESPNDDNGYDISDYCKIMNEFGTMEDWDELLHEMHERNMKLMMDLVVNHTSDEHNWFIESRKSKDNKYRDYYIWRPGKEGKEPNNWGAAFSGSAWQYDEMTDEYYLHLFSKKQPDLNWDNEKVRQDVYEMMKFWLEKGIDGFRMDVINFISKEEGLPTVETEEEGYVSGHKHFMNGPNIHKYLHEMNEEVLSHYDIMTVGEMPGVTTEEAKLYTGEERKELQMVFQFEHMDLDSGEGGKWDVKPCSLLTLKENLTKWQKALEHTGWNSLYWNNHDQPRVVSRFGNDGMYRIESAKMLATVLHMMKGTPYIYQGEEIGMTNVRFESIDEYRDIETLNMYKEKVMERGEDIEKVMQSIYIKGRDNARTPMQWDDQNHAGFTTGEPWITVNPNYKEINVKQAIQNKDSIFYYYKKLIELRKNNEIVVYGSYDLILENNPSIFAYVRTYGVEKLLVIANFTAEECIFELPEDISYSEVELLIHNYDVENGPIENITLRPYEAMVFKLK

>1USH-_

MKLLQRGVALALLTTFTLASETALAYEQDKTYKITVLHTNDHHGHFWRNEYGEYGLAAQKTLVDGIRKEVAAEGGSVLLLSGGDINTGVPESDLQDAEPDFRGMNLVGYDAMAIGNHEFDNPLTVLRQQEKWAKFPLLSANIYQKSTGERLFKPWALFKRQDLKIAVIGLTTDDTAKIGNPEYFTDIEFRKPADEAKLVIQELQQTEKPDIIIAATHMGHYDNGEHGSNAPGDVEMARALPAGSLAMIVGGHSQDPVCMAAENKKQVDYVPGTPCKPDQQNGIWIVQAHEWGKYVGRADFEFRNGEMKMVNYQLIPVNLKKKVTWEDGKSERVLYTPEIAENQQMISLLSPFQNKGKAQLEVKIGETNGRLEGDRDKVRFVQTNMGRLILAAQMDRTGADFAVMSGGGIRDSIEAGDISYKNVLKVQPFGNVVVYADMTGKEVIDYLTAVAQMKPDSGAYPQFANVSFVAKDGKLNDLKIKGEPVDPAKTYRMATLNFNATGGDGYPRLDNKPGYVNTGFIDAEVLKAYIQKSSPLDVSVYEPKGEVSWQ

>1UTG-_

GICPRFAHVIENLLLGTPSSYETSLKEFEPDDTMKDAGMQMKKVLDSLPQTTRENIMKLTEKIVKSPLCM

>1VLS-_

MNQQGFVISNELRQQQSELTSTWDLMLQTRINLSRSAARMMMDASNQQSSAKTDLLQNAKTTLAQAAAHYANFKNMTPLPAMAEASANVDEKYQRYQAALAELIQFLDNGNMDAYFAQPTQGMQNALGEALGNYARVSENLYRQTF
