# Supplementary material for: enDNA-Prot: Identification of DNA-Binding Proteins by Applying Ensemble Learning
Source: Biomed Res Int. 2014 May 26;2014:294279. doi: 10.1155/2014/294279 (PMC4058174; doi:10.1155/2014/294279)
Supplement: Supplementary file 1 — Supplementary Material S1 lists all the codes and sequences for the benchmark dataset. It contains 396 proteins, classified into 146 DNA-binding proteins and 250 non DNA-binding proteins. Supplementary Material S2 lists all the codes and sequences for the expanded benchmark dataset. It contains 2271 proteins, classified into 146 DNA-binding proteins and 2125 non DNA-binding proteins. Supplementary Material S3 lists all the codes and sequences for the independent dataset1. It contains 182 proteins, classified into 82 DNA-binding proteins and 100 non DNA-binding proteins. Supplementary Material S4 lists all the codes and sequences for the independent dataset2. It contains 1585 proteins, classified into 770 DNA-binding proteins and 815 non DNA-binding proteins. [file 294279.f1.zip › 294279.f1/S2.docx]

Online Supporting Information S2.The 2271 identifiers and sequences in expanded benchmark dataset are shown here. It contains 146 DNA-binding protein sequences and 2125 non DNA-binding proteins sequences.

|  |
| --- |

(1).DNA-binding proteins:

>D1A04A2

EPATILLIDDHPMLRTGVKQLISMAPDITVVGEASNGEQGIELAESLDPDLILLDLNMPGMNGLETLDKLREKSLSGRIVVFSVSNHEEDVVTALKRGADGYLLKDMEPEDLLKALHQAAAGEMVLSEALTPVLAASL

>D1A0AA_

MKRESHKHAEQARRNRLAVALHELASLIPAEWKQQNVSAAPSKATTVEAACRYIRHLQQNGST

>D1A1IA1

RPYACPVESCDRRFSRSADLTRHIRIHTG

>D1A3QA2

GPYLVIVEQPKQRGFRFRYGCEGPSHGGLPGASSEKGRKTYPTVKICNYEGPAKIEVDLVTHSDPPRAHAHSLVGKQCSELGICAVSVGPKDMTAQFNNLGVLHVTKKNMMGTMIQKLQRQRLRSRPQGLTEAEQRELEQEAKELKKVMDLSIVRLRFSAFLRSLPLKPVISQPIHDSKSPGAS

>D1A73A_

ALTNAQILAVIDSWEETVGQFPVITHHVPLGGGLQGTLHCYEIPLAAPYGVGFAKNGPTRWQYKRTINQVVHRWGSHTVPFLLEPDNINGKTCTASHLCHNTRCHNPLHLCWESLDDNKGRNWCPGPNGGCVHAVVCLRQGPLYGPGATVAGPQQRGSHFVV

>D1AISB1

NLAFALSELDRITAQLKLPRHVEEEAARLYREAVRKGLIRGRSIESVMAACVYAACRLLKVPRTLDEIADIARVDKKEIGRSYRFIARNLNLTPKKLF

>D1AISB2

VKPTDYVNKFADELGLSEKVRRRAIEILDEAYKRGLTSGKSPAGLVAAALYIASLLEGEKRTQREVAEVARVTEVTVRNRYKELVEKLKIKVPIA

>D1AZPA_

MVKVKFKYKGEEKEVDTSKIKKVWRVGKMVSFTYDDNGKTGRGAVSEKDAPKELLDMLARAEREKK

>D1B3TA_

KGGWFGKHRGQGGSNPKFENIAEGLRALLARSHVERTTDEGTWVAGVFVYGGSKTSLYNLRRGTALAIPQCRLTPLSRLPFGMAPGPGPQPGPLRESIVCYFMVFLQTHIFAEVLKDAIKDLVMTKPAPTCNIRVTVCSFDDGVDLP

>D1BG1A1

VVTEKQQMLEQHLQDVRKRVQDLEQKMKVVENLQDDFDFNYKTLKSQGDMQDLNGNNQSVTRQKMQQLEQMLTALDQMRRSIVSELAGLLSAMEYVQKTLTDEELADWKRRQQIACIGGPPNICLDRLENWITSLAESQLQTRQQIKKLEELQQKVSYKGDPIVQHRPMLEERIVELFRNLMKSAF

>D1BG1A2

VVERQPCMPMHPDRPLVIKTGVQFTTKVRLLVKFPELNYQLKIKVCIDKDSGDVAALRGSRKFNILGTNTKVMNMEESNNGSLSAEFKHLTLREQRCGNGGRANCDASLIVTEELHLITFETEVYHQGLKIDLETHSLPVVVISNICQMPNAWASILWYNMLTNNPKNVNFFTKPPIGTWDQVAEVLSWQFSSTTKRGLSIEQLTTLAEKLLGPGVNYSGCQITWAKFCKENMAGKGFSFWVWLDNIIDLVKKY

>D1BG1A3

ILALWNEGYIMGFISKERERAILSTKPPGTFLLRFSESSKEGGVTFTWVEKDISGSTQIQSVEPYTKQQLNNMSFAEIIMGYKIMDATNILVSPLVYLYPDIPKEEAFGKYCRPESQEHPEADPGSAAPYLKTKFICVTPF

>D1BL0A1

DAITIHSILDWIEDNLESPLSLEKVSERSGYSKWHLQRMFKKETGHSLGQYIRS

>D1BL0A2

RKMTEIAQKLKESNEPILYLAERYGFESQQTLTRTFKNYFDVPPHKYRMTNMQGESRFLHPL

>D1CDWA2

NMVGSCDVKFPIRLEGLVLTHQQFSSYEPELFPGLIYRMIKPRIVLLIFVSGKVVLTGAKVRAEIYEAFENIYPILKGFRK

>D1CF7A_

SRHEKSLGLLTTKFVSLLQEAKDGVLDLKLAADTLAVRQKRRIYDITNVLEGIGLIEKKSKNSIQWK

>D1CF7B_

GKGLRHFSMKVCEKVQRKGTTSYNEVADELVSEFTNSNNHLAADSAYDQKNIRRRVYDALNVLMAMNIISKEKKEIKWIGLP

>D1CKQA_

SQGVIGIFGDYAKAHDLAVGEVSKLVKKALSNEYPQLSFRYRDSIKKTEINEALKKIDPDLGGTLFVSNSSIKPDGGIVEVKDDYGEWRVVLVAEAKHQGKDIINIRNGLLVGKRGDQDLMAAGNAIERSHKNISEIANFMLSESHFPYVLFLEGSNFLTENISITRPDGRVVNLEYNSGILNRLDRLTAANYGMPINSNLCINKFVNHKDKSIMLQAASIYTQGDGREWDSKIMFEIMFDISTTSLRVLGRDLFEQLTSK

>D1D02A_

LSGRLNWQALAGLKASGAEQNLYNVFNAVFEGTKYVLYEKPKHLKNLYAQVVLPDDVIKEIFNPLIDLSTTQWGVSPAFAIENTETHKILFGEIKRQDGWVEGKDPSAGRGNAHERSCKLFTPGLLKAYRTIGGINDEEILPFWVVFEGDITRDPKRVREITFWYDHYQDNYFMWRPNESGEKLVQHFNEKLKKYLD

>D1D5YA3

EFTMPEHKFVTLEDTPLIGVTQSYSCSLEQISDFRHEMRYQFWHDFLGNAPTIPPVLYGLNETRPSQDKDDEQEVFYTTALAQDQADGYVLTGHPVMLQGGEYVMFTYEGLGTGVQEFILTVYGTCMPMLNLTRRKGQDIERYYPAEDAKAGDRPINLRCELLIPIRRKLAAA

>D1D66A1

EQACDICRLKKLKCSKEKPKCAKCLKNNWECRYSPKTKRSP

>D1DC1A_

KPFENHLKSVDDLKTTYEEYRAGFIAFALEKNKRSTPYIERARALKVAASVAKTPKDLLYLEDIQDALLYASGISDKAKKFLTEDDKKESINNLIENFLEPAGEEFIDELIFRYLLFQGDSLGGTMRNIAGALAQQKLTRAIISALDIANIPYKWLDSRDKKYTNWMDKPEDDYELETFAKGISWTINGKHRTLMYNITVSLVKKNVDICLFNCEPEIYTPQKVHQQPEKYLLLGELKGGIDPAGADEHWKTANTALTRIRNKFSEKGLSPKTIFIGAAIEHSMAEEIWDQLQSGSLTNSANLTKTEQVGSLCRWIINI

>D1DCTA_

MNLISLFSGAGGLDLGFQKAGFRIICANEYDKSIWKTYESNHSAKLIKGDISKISSDEFPKCDGIIGGPPCQSWSEGGSLRGIDDPRGKLFYEYIRILKQKKPIFFLAENVKGMMAQRHNKAVQEFIQEFDNAGYDVHIILLNANDYGVAQDRKRVFYIGFRKELNINYLPPIPHLIKPTFKDVIWDLKDNPIPALDKNKTNGNKCIYPNHEYFIGSYSTIFMSRNRVRQWNEPAFTVQASGRQCQLHPQAPVMLKVSKNLNKFVEGKEHLYRRLTVRECARVQGFPDDFIFHYESLNDGYKMIGNAVPVNLAYEIAKTIKSAL

>D1DFMA_

MKIDITDYNHADEILNPQLWKEIEETLLKMPLHVKASDQASKVGSLIFDPVGTNQYIKDELVPKHWKNNIPIPKRFDFLGTDIDFGKRDTLVEVQFSNYPFLLNNTVRSELFHKSNMDIDEEGMKVAIIITKGHMFPASNSSLYYEQAQNQLNSLAEYNVFDVPIRLVGLIEDFETDIDIVSTTYADKRYSRTITKRDTVKGKVIDTNTPNTRRRKRGTIVTY

>D1DMUA_

MYNLHREKIFMSYNQNKQYLEDNPEIQEKIELYGLNLLNEVISDNEEEIRADYNEANFLHPFWMNYPPLDRGKMPKGDQIPWIEVGEKAVGSKLTRLVSQREDITVREIGLPTGPDERYLLTSPTIYSLTNGFTDSIMMFVDIKSVGPRDSDYDLVLSPNQVSGNGDWAQLEGGIQNNQQTIQGPRSSQIFLPTIPPLYILSDGTIAPVVHLFIKPIYAMRSLTKGDTGQSLYKIKLASVPNGLGLFCNPGYAFDSAYKFLFRPGKDDRTKSLLQKRVRVDLRVLDKIGPRVMTIDMDK

>D1DP7P_

TVQWLLDNYETAEGVSLPRSTLYNHYLLHSQEQKLEPVNAASFGKLIRSVFMGLRTRRLGTRGNSKYHYYGLRIKA

>D1DSZA_

PCFVCQDKSSGYHYGVSACEGCKGFFRRSIQKNMVYTCHRDKNCIINKVTRNRCQYCRLQKCFEVGMSKESVRND

>D1E3MA3

GTISDEALLQERQDNLLAAIWQDSKGFGYATLDISSGRFRLSEPADRETMAAELQRTNPAELLYAEDFAEMSLIEGRRGLRRRPLWEFEIDTARQQLNLQFGTRDLVGFGVENAPRGLCAAGCLLQYAKDTQRTTLPHIRSITMEREQDSIIM

>D1E3OC2

EEPSDLEELEQFAKTFKQRRIKLGFTQGDVGLAMGKLYGNDFSQTTISRFEALNLSFKNMSKLKPLLEKWLNDAE

>D1ECRA_

DLVDRLNTTFRQMEQELAIFAAHLEQHKLLVARVFSLPEVKKEDEHNPLNRIEVKQHLGNDAQSLALRHFRHLFIQQQSENRSSKAAVRLPGVLCYQVDNLSQAALVSHIQHINKLKTTFEHIVTVESELPTAARFEWVHRHLPGLITLNAYRTLTVLHDPATLRFGWANKHIIKNLHRDEVLAQLEKSLKSPRSVAPWTREEWQRKLEREYQDIAALPQNAKLKIKRPVKVQPIARVWYKGDQKQVQHACPTPLIALINRDNGAGVPDVGELLNYDADNVQHRYKPQAQPLRLIIPRLHLYVAD

>D1EGWA_

GRKKIQITRIMDERNRQVTFTKRKFGLMKKAYELSVLCDCEIALIIFNSSNKLFQYASTDMDKVLLKYTEY

>D1EWNA_

HLTRLGLEFFDQPAVPLARAFLGQVLVRRLPNGTELRGRIVETQAYLGPEDEAAHSRGGRQTPRNRGMFMKPGTLYVYIIYGMYFCMNISSQGDGACVLLRALEPLEGLETMRQLRSTLRKGTASRVLKDRELCSGPSKLCQALAINKSFDQRDLAQDEAVWLERGPLEPSEPAVVAAARVGVGHAGEWARKPLRFYVRGSPWVSVVDRVAEQD

>D1EWQA1

RGQDTLFSVLDETRTAPGRRLLQSWLRHPLLDRGPLEARLDRVEGFVREGALREGVRRLLYRLADLERLATRLELGRASPKDLGALRRSLQILPELRALLGEEVGLPDLSPLKEELEAALVEDPPLKVSEGGLIREGYDPDLDALRAAHREGVAYFLELEERERERTGIPTLKVGYNAVFGYYLEVTRPYYERVPKEYRPVQTLKDRQRYTLPEMKEKEREVYRLEALIRRREEEVFLEVRERAKRQAEALREAARILAELDVYAALAEVAVRYG

>D1EWQA2

YVRPRFGDRLQIRAGRHPVVERRTEFVPNDLEMAHELVLITGPNMAGKSTFLRQTALIALLAQVGSFVPAEEAHLPLFDGIYTRIGASDDLAGGKSTFMVEMEEVALILKEATENSLVLLDEVGRGTSSLDGVAIATAVAEALHERRAYTLFATHYFELTALGLPRLKNLHVAAREEAGGLVFYHQVLPGPASKSYGVEVAAMAGLPKEVVARARALLQAMAAR

>D1EWQA3

LLQESLLPREANYLAAIATGDGWGLAFLDVSTGEFKGTVLKSKSALYDELFRHRPAEVLLAPELLENGAFLDEFRKRFPVMLSEAPFEPEGEGPLALRRARGALLAYAQRTQGGALSLQPFRFYDPGAFMRLPEATLRALEVFEPL

>D1EWQA4

MEGMLKGEGPGPLPPLLQQYVELRDQYPDYLLLFQVGDFYECFGEDAERLARALGLVLTHKTSKDFTTPMAGIPLRAFEAYAERLLKMGFRLAVADQVEPAEEAEGLVRREVTQLLTPGT

>D1F44A1

SDEVRKNLMDMFRDRQAFSEHTWKMLLSVCRSWAAWCKLNNRKWFPAEPEDVRDYLLYLQARGLAVKTIQQHLGQLNMLHRRSGLPRPSDSNAVSLVMRRIRKENVDAGE

>D1F44A2

RAKQALAFERTDFDQVRSLMENSDRCQDIRNLAFLGIAYNTLLRIAEIARIRVKDISRTDGGRMLIHIGRTKTLVSTAGVEKALSLGVTKLVERWISVSGVADDPNNYLFCRVRKNGVAAPSATSQLSTRALEGIFEATHRLIYGAKDDSGQRYLAWSGHSARVGAARDMARAGVSIPEIMQAGGWTNVNIVMNFIRNLDSETGAMVRLLEDGD

>D1FIUA_

MQPLFTQERRIFHKKLLDGNILATNNRGVVSNADGSNTRSFNIAKGIADLLHSETVSERLPGQTSGNAFEAICSEFVQSAFEKLQHIRPGDWNVKQVGSRNRLEIARYQQYAHLTALAKAAEENPELAAALGSDYTITPDIIVTRNLIADAEINRNEFLVDENIATYASLRAGNGNMPLLHASISCKWTIRSDRAQNARSEGLNLVRNRKGRLPHIVVVTAEPTPSRISSIALGTGEIDCVYHFALYELEQILQSLNYEDALDLFYIMVNGKRLKDISDLPLDLAV

>D1FJLA_

KQRRSRTTFSASQLDELERAFERTQYPDIYTREELAQRTNLTEARIQVWFQNRRARLRKQHTSVS

>D1FLOA1

PQFDILCKTPPKVLVRQFVERFERPSGEKIALCAAELTYLCWMITHNGTAIKRATFMSYNTIISNSLSFDIVNKSLQFKYKTQKATILEASLKKLIPAWEFTIIPYYGQKHQSDITDIVSSLQLQFES

>D1FLOA2

KGNSHSKKMLKALLSEGESIWEITEKILNSFEYTSRFTKTKTLYQFLFLATFINCGRFSDIKNVDPKSFKLVQNKYLGVIIQCLVTETKTSVSRHIYFFSARGRIDPLVYLDEFLRNSEPVLKRVNRTGNSSSNKQEYQLLKDNLVRSYNKALKKNAPYSIFAIKNGPKSHIGRHLMTSFLSMKGLTELTNVVGNWSDKRASAVARTTYTHQITAIPDHYFALVSRYYAYDPISKEMIALKDETNPIEEWQHIEQLKGSAEGSIRYPAWNGIISQEVLDYLSSYINRRI

>D1G38A_

VETPPEVVDFMVSLAEAPRGGRVLEPACAHGPFLRAFREAHGTGYRFVGVEIDPKALDLPPWAEGILADFLLWEPGEAFDLILGNPPYGIVGEASKYPIHVFKAVKDLYKKAFSTWKGKYNLYGAFLEKAVRLLKPGGVLVFVVPATWLVLEDFALLREFLAREGKTSVYYLGEVFPQKKVSAVVIRFQKSGKGLSLWDTQESESGFTPILWAEYPHWEGEIIRFETEETRKLEISGMPLGDLFHIRFAARSPEFKKHPAVRKEPGPGLVPVLTGRNLKPGWVDYEKNHSGLWMPKERAKELRDFYATPHLVVAHTKGTRVVAAWDERAYPWREEFHLLPKEGVRLDPSSLVQWLNSEAMQKHVRTLYRDFVPHLTLRMLERLPVRREYGFHT

>D1G9ZA_

NTKYNKEFLLYLAGFVDGDGSIIAQIKPNQSYKFKHQLSLTFQVTQKTQRRWFLDKLVDEIGVGYVRDRGSVSDYILSEIKPLHNFLTQLQPFLKLKQKQANLVLKIIEQLPSAKESPDKFLEVCTWVDQIAALNDSKTRKTTSETVRAVLD

>D1GT0D_

DRVKRPMNAFMVWSRGQRRKMAQENPKMHNSEISKRLGAEWKLLSETEKRPFIDEAKRLRALHMKEHPDYKYRPRRKTKT

>D1H6FA_

DPKVHLEAKELWDQFHKRGTEMVITKSGRRMFPPFKVRCSGLDKKAKYILLMDIIAADDCRYKFHNSRWMVAGKADPEMPKRMYIHPDSPATGEQWMSKVVTFHKLKLTNNISDKHGFTILNSMHKYQPRFHIVRANDILKLPYSTFRTYLFPETEFIAVTAYQNDKITQLKIDNNPFAKGFRD

>D1H9DB_

PRVVPDQRSKFENEEFFRKLSRECEIKYTGFRDRPHEERQARFQNACRDGRSEIAFVATGTNLSLQFFPASWQGEQRQTPSREYVDLEREAGKVYLKAPMILNGVCVIWKGWIDLQRLDGMGCLEFDEERAQQE

>D1HCRA_

GRPRAINKHEQEQISRLLEKGHPRQQLAIIFGIGVSTLYRYFPASSIKKRMN

>D1HLVA1

MGPKRRQLTFREKSRIIQEVEENPDLRKGEIARRFNIPPSTLSTILKNKRAILASERKYGVASTCR

>D1HLVA2

KTNKLSPYDKLEGLLIAWFQQIRAAGLPVKGIILKEKALRIAEELGMDDFTASNGWLDRFRRRRS

>D1I3JA_

KFCKCGVRIQTSAYTCSKCRNRSGENNSFFNHKHSDITKSKISEKMKGKKPSNIKKISCDGVIFDCAADAARHFKISSGLVTYRVKSDKWNWFYIN

>D1I7DA_

MRLFIAEKPSLARAIADVLPKPHRKGDGFIECGNGQVVTWCIGHLLEQAQPDAYDSRYARWNLADLPIVPEKWQLQPRPSVTKQLNVIKRFLHEASEIVHAGDPDREGQLLVDEVLDYLQLAPEKRQQVQRCLINDLNPQAVERAIDRLRSNSEFVPLCVSALARARADWLYGINMTRAYTILGRNAGYQGVLSVGRVQTPVLGLVVRRDEEIENFVAKDFFEVKAHIVTPADERFTAIWQPSEACEPYQDEEGRLLHRPLAEHVVNRISGQPAIVTSYNDKRESESAPLPFSLSALQIEAAKRFGLSAQNVLDICQKLYETHKLITFPRSDCRYLPEEHFAGRHAVMNAISVHAPDLLPQPVVDPDIRNRCWDDKKVDAHHAIIPTARSSAINLTENEAKVYNLIARQYLMQFCPDAVFRKCVIELDIAKGKFVAKARFLAEAGWRTLLGSKERDEENDGTPLPVVAKGDELLCEKGEVVERQTQPPRHFTDATLLSAMTGIARFVQDKDLKKILRATDGLGTEATRAGIIELLFKRGFLTKKGRYIHSTDAGKALFHSLPEMATRPDMTAHWESVLTQISEKQCRYQDFMQPLVGTLYQLIDQAKRTPVRQFRGIVAP

>D1IC8A2

KELENLSPEEAAHQKAVVETLLQEDPWRVAKMVKSYLQQHNIPQREVVDTTGLNQSHLSQHLNKGTPMKTQKRAALYTWYVRKQREVAQQFTHA

>D1IGNA1

KASFTDEEDEFILDVVRKNPTRRTTHTLYDEISHYVPNHTGNSIRHRFRVYLSKRLEYVYEVDKFGKLVRDDDGNLIKTKVLPPSI

>D1IGNA2

KRKFSADEDYTLAIAVKKQFYRDLFQIDPDTGRSLITDEDTPTAIARRNMTMDPNHVPGSEPNFAAYRTQSRRGPIAREFFKHFAEEHAAHTENAWRDRFRKFLLAYGIDDYISYYEAEKAQNREPEPMKNLTNRPKRPGVPTPGNYNS

>D1J1VA_

VTIDNIQKTVAEYYKIKVADLLSKRRSRSVARPRQMAMALAKELTNHSLPEIGDAFGGRDHTTVLHACRKIEQLREESHDIKEDFSNLIRTLSS

>D1J3EA_

PLGSAMRELLLSDEYAEQKRAVNRFMLLLSTLYSLDAQAFAEATESLHGRTRVYFAADEQTLLKNGNQTKPKHVPGTPYWVITNTNTGRKCSMIEHIMQSMQFPAELIEKVCGTI

>D1J75A_

NLEQKILQVLSDDGGPVKIGQLVKKCQVPKKTLNQVLYRLKKEDRVSSPEPATWSIG

>D1JB7A1

YEYVELAKASLTSAQPQHFYAVVIDATFPYKTNQERYICSLKIVDPTLYLKQQKGAGDASDYATLVLYAKRFEDLPIIHRAGDIIRVHRATLRLYNGQRQFNANVFYSSSWALFSTDKRSVTQEINNQDAVSDTTPFSFSSKHATIEKNEISILQNLRKWANQYFSSYS

>D1JB7A2

VISSDMYTALNKAQAQKGDFDVVAKILQVHELDEYTNELKLKDASGQVFYTLSLKLKFPHVRTGEVVRIRSATYDETSTQKKVLILSHYSNIITFIQSSKLAKELRAKIQDDHSVEVASLKKNV

>D1JB7A3

SLNAVVLTEVDKKHAALPSTSLQDLFHHADSDKELQAQDTFRTQFYVTKIEPSDVKEWVKGYDRKTKKSSSLKGASGKGDNIFQVQFLVKDASTQLNNNTYRVLLYTQDGLGANFFNVKADNLHKNADARKKLEDSAELLTKFNSYVDAVVERRNGFYLIKDTKLIY

>D1JB7B_

QQQSAFKQLYTELFNNEGDFSKVSSNLKKPLKCYVKESYPHFLVTDGYFFVAPYFTKEAVNEFHAKFPNVNIVDLTDKVIVINNWSLELRRVNSAEVFTSYANLEARLIVHSFKPNLQERLNPTRYPVNLFRDDEFKTTIQHFRHTALQAAINKTVKGDNLVDISKVADAAGKKGKVDAGIVKASASKGDEFSDFSFKEGNTATLKIADIFVQEKG

>D1JEYA1

RALSRLKLKLNKDIVISVGIYNLVQKALKPPPIKLYRETNEPVKTKTRTFNTSTGGLLLPSDTKRSQIYGSRQIILEKEETEELKRFDDPGLMLMGFKPLVLLKKHHYLRPSLFVYPEESLVIGSSTLFSALLIKCLEKEVAALCRYTPRRNIPPYFVALVPQEEELDDQKIQVTPPGFQLVFLPFADDKRKMPFTEKIMATPEQVGKMKAIVEKLRFTYRSDSFENPVLQQHFRNLEALALDLMEPEQAVDLTLPKVEAMNKRLGSLVDEFKELVYPPDY

>D1JEYB1

RHSIHWPCRLTIGSNLSIRIAAYKSILQERVKKTWTVVDAKTLKKEDIQKETVYCLNDDDETEVLKEDIIQGFRYGSDIVPFSKVDEEQMKYKSEGKCFSVLGFCKSSQVQRRFFMGNQVLKVFAARDDEAAAVALSSLIHALDDLDMVAIVRYAYDKRANPQVGVAFPHIKHNYECLVYVQLPFMEDLRQYMFSSLKNSKKYAPTEAQLNAVDALIDSMSLAKKDEKTDTLEDLFPTTKIPNPRFQRLFQCLLHRALHPREPLPPIQQHIWNMLNPPAEVTTKSQIPLSKIKTLFPLIEAKKK

>D1JEYA2

GRDSLIFLVDASKAMFESQSEDELTPFDMSIQCIQSVYISKIISSDRDLLAVVFYGTEKDKNSVNFKNIYVLQELDNPGAKRILELDQFKGQQGQKRFQDMMGHGSDYSLSEVLWVCANLFSDVQFKMSHKRIMLFTNEDNPHGNDSAKASRARTKAGDLRDTGIFLDLMHLKKPGGFDISLFYRDIISIAEDEDLRVHFEESSKLEDLLRKVRAKETRK

>D1JEYB2

NKAAVVLCMDVGFTMSNSIPGIESPFEQAKKVITMFVQRQVFAENKDEIALVLFGTDGTDNPLSGGDQYQNITVHRHLMLPDFDLLEDIESKIQPGSQQADFLDALIVSMDVIQHETIGKKFEKRHIEIFTDLSSRFSKSQLDIIIHSLKKCDISLQFFLPFSLGKEDGSGDRGDGPFRLGGHGPSFPLKGITEQQKEGLEIVKMVMISLEGEDGLDEIYSFSESLRKLCVFKKIE

>D1JFIB_

DDLTIPRAAINKMIKETLPNVRVANDARELVVNCCTEFIHLISSEANEICNKSEKKTISPEHVIQALESLGFGSYISEVKEVLQECKTVALKRRKASSRLENLGIPEEELLRQQQELFAKARQQQAELAQQEWLQ

>D1JX4A1

VRKSIGRIVTMKRNSRNLEEIKPYLFRAIEESYYKLDKRIPKAIHVVAVTEDLDIVSRGRTFPHGISKETAYSESVKLLQKILEEDERKIRRIGVRFSKFI

>D1JX4A2

MIVLFVDFDYFYAQVEEVLNPSLKGKPVVVCVFSGRFEDSGAVATANYEARKFGVKAGIPIVEAKKILPNAVYLPMRKEVYQQVSSRIMNLLREYSEKIEIASIDEAYLDISDKVRDYREAYNLGLEIKNKILEKEKITVTVGISKNKVFAKIAADMAKPNGIKVIDDEEVKRLIRELDIADVPGIGNITAEKLKKLGINKLVDTLSIEFDKLKGMIGEAKAKYLISLARDEYNEPIRTR

>D1K3XA1

VGPDVLDPNLTPEVVKERLLSPRFRNRQFAGLLLDQAFLAGLGNYLRVEILWQVGLTGNHKAKDLNAAQLDALAHALLEIPRFSYATRG

>D1K3XA2

PEGPEIRRAADNLEAAIKGKPLTDVWFAFPQLKTYQSQLIGQHVTHVETRGKALLTHFSNDLTLYSHNQLYGVWRVVDTGEEPQTTRVLRVKLQTADKTILLYSASDIEMLRPEQLTTHPFLQR

>D1K3XA3

ALFRFKVFHRDGEPCERCGSIIEKTTLSSRPFYWCPGCQH

>D1K4TA1

EKSMMNLQTKIDAKKEQLADARRDLKSAKADAKVMKDAKTKKVVESKKKAVQRLEEQLMKLEVQATDREENK

>D1K4TA2

PSSRIKGEKDWQKYETARRLKKCVDKIRNQYREDWKSKEMKVRQRAVALYFIDKLALRAGNEKEEGETADTVGCCSLRVEHINLHPELDGQEYVVEFDFLGKDSIRYYNKVPVEKRVFKNLQLFMENKQPEDDLFDRLNTGILNKHLQDLMEGLTAKVFRTYNASITLQQQLKELTAPDENIPAKILSYNRANRAVAILCNHQRAPPKTFXQIALGTSKLNYLDPRITVAWCKKWGVPIEKIYNKTQREKFAWAIDMADEDYEF

>D1K61A_

RGHRFTKENVRILESWFAKNIENPYLDTKGLENLMKNTSLSRIQIKNWVSNRRRKEKTIT

>D1KFSA1

MISYDNYVTILDEETLKAWIAKLEKAPVFAFDTETDSLDNISANLVGLSFAIEPGVAAYIPVAHDYLDAPDQISRERALELLKPLLEDEKALKVGQNLKYDRGILANYGIELRGIAFDTMLESYILNSVAGRHDMDSLAERWLKHKTITFEEIAGKGKNQLTFNQIALEEAGRYAAEDADVTLQLHLKMWPDLQK

>D1L3LA1

DAAWLDPKEATYLRWIAVGKTMEEIADVEGVKYNSVRVKLREAMKRFDVRSKAHLTALAIRRKLI

>D1L3LA2

QHWLDKLTDLAAIEGDECILKTGLADIADHFGFTGYAYLHIQHRHITAVTNYHRQWQSTYFDKKFEALDPVVKRARSRKHIFTWSGEHERPTLSKDERAFYDHASDFGIRSGITIPIKTANGFMSMFTMASDKPVIDLDREIDAVAAAATIGQIHARISFLRTTPTAE

>D1LLMC2

EKPFACDICGRKFARSDERKRHRDIQHI

>D1LMB3_

PLTQEQLEDARRLKAIYEKKKNELGLSQESVADKMGMGQSGVGALFNGINALNAYNAALLAKILKVSVEEFSPSIAREIYEMYEAVS

>D1M3QA1

DPIECLFSFICSSNNNIARITGMVERLCQAFGPRLIQLDDVTYHGFPSLQALAGPEVEAHLRKLGLGYRARYVSASARAILEEQGGLAWLQQLRESSYEEAHKALCILPGVGTKVADCICLMALDKPQAVPVEVHMWHIAQRDYSWHPTTSQAKGPSPQTNKELGNFFRSLWGPYAGWAQAVLFSADLRQ

>D1M3QA2

GSEGHRTLASTPALWASIPCPRSELRLDLVLPSGQSFRWREQSPAHWSGVLADQVWTLTQTEEQLHCTVYRGDKSQASRPTPDELEAVRKYFQLDVTLAQLYHHWGSVDSHFQEVAQKFQGVRLLRQ

>D1MDYB_

TTNADRRKAATMRERRRLSKVNEAFETLKRSTSSNPNQRLPKVEILRNAIRYIEGLQALLRD

>D1MNNA_

VILTQLNEDGTTSNYFDKRKLKIAPRSTLQFKVGPPFELVRDYCPVVESHTGRTLDLRIIPRIDRGFDHIDEEWVGYKRNYFTLVSTFETANCDLDTFLKSSFDLLVEDSSVEGRLRVQYFAIKIKAKNDDDDTEINLVQHTAKRDKGPQFCPSVCPLVPSPLPKHQTIREASNVRNITKMKKYDSTFYLHRDHVNYEEYGVDSLLFSYPEDSIQKVARYERVQFASSISVKKPSQQNKHFSLHVILGAVVDPDTFHGENPGIPYDELALKNGSKGMFVYLQEMKTPPLIIRGRSPSNYASSQ

>D1MUSA_

SALHRAADWAKSVFSSAALGDPRRTARLVNVAAQLAKYSGKSITISSEGSKAAQEGAYRFIRNPNVSAEAIRKAGAMQTVKLAQEFPELLAIEDTTSLSYRHQVAEELGKLGSIQKASRGWWVHSVLLLEATTFRTVGLLHQEWWMRPDDPADADEKESGKWLAAAATSRLRMGSMMSNVIAVCDREADIHAYLQDKLAHNERFVVRSKHPRKDVESGLYLYDHLKNQPELGGYQISIPQKGVVDKRGKRKNRPARKASLSLRSGRITLKQGNITLNAVLAEEINPPKGETPLKWLLLTSEPVESLAQALRVIDIYTHRWRIEEFHKAWKTGAGAERQRMEKPDNLERMVSILSFVAVRLLQLRESFTPPQALRAQGLLKEAEHVESQSAETVLTPDECQLLGYLDKGKRKRKEKAGSLQWAYMAIARLGGFMDSKRTGIASWGALWEGWEALQSKLDGFLAAKDLMAQGIKIG

>D1NH2B_

NAEASRVYEIIVESVVNEVREDFENAGIDEQTLQDLKNIWQKKLTE

>D1NH2D1

GYYELYRRSTIGNSLVDALDTLISDGRIEASLAMRVLETFDKVVAETLKD

>D1NH2C_

DYLISEGEEDGPDENLMLCLYDKVTRTKARWKCSLKDGVVTINRNDYTFQKAQVEAEWV

>D1NH2D2

NTQSKLTVKGNLDTYGFCDDVWTFIVKNCQVTVEDSHRDASQNGSGDSQSVISVDKLRIVACNSKKS

>D1NH2A1

SGIVPTLQNIVATVTLGCRLDLKTVALHARNAEYNPKRFAAVIMRIREPKTTALIFASGKMVVTGAKSEDDSKLASRKYARIIQKIGFAAKFTDF

>D1ODHA_

LSWDINDVKLPQNVKTTDWFQEWPDSYVKHIYSSDDRNAQRHLSSWAMRNTNNHNSRILKKSCLGVVVCSRDCSTEEGRKIYLRPAICDKARQKQQRKSCPNCNGPLKLIPCRGHGGFPVTNFWRHDGRFIFFQSKGEHDHPRPETKLEAEARRAMKK

>D1OE4A_

ESPADSFLKVELELNLKLSNLVFQDPVQYVYNPLVYAWAPHENYVQTYCKSKKEVLFLGMNPGPFGMAQTGVPFGEVNHVRDWLQIEGPVSKPEVEHPKRRIRGFECPQSEVSGARFWSLFKSLCGQPETFFKHCFVHNHCPLIFMNHSGKNLTPTDLPKAQRDTLLEICDEALCQAVRVLGVKLVIGVGRFSEQRARKALMAEGIDVTVKGIMHPSPRNPQANKGWEGIVRGQLLELGVLSLLT

>D1OJ8A_

EDWDTFQKKHLTDTKKVKCDVEMKKALFDCKKTNTFIFARPPRVQALCKNIKNNTNVLSRDVFYLPQCNRKKLPCHYRLDGSTNTICLTCMKELPIHFAGVGKCP

>D1OMHA_

MLSHMVLTRQDIGRAASYYEDGADDYYAKDGDASEWQGKGAEELGLSGEVDSKRFRELLAGNIGEGHRIMRSATRQDSKERIGLDLTFSAPKSVSLQALVAGDAEIIKAHDRAVARTLEQAEARAQARQKIQGKTRIETTGNLVIGKFRHETSRERDPQLHTHAVILNMTKRSDGQWRALKNDEIVKATRYLGAVYNAELAHELQKLGYQLRYGKDGNFDLAHIDRQQIEGFSKRTEQIAEWYAARGLDPNSVSLEQKQAAKVLSRAKKTSVDREALRAEWQATAKELGIDFS

>D1OZJA_

FTPPIVKRLLGWKKGEQNGQEEKWCEKAVKSLVKKLKKTGQLDELEKAITTQNVNTKCITIPRSLDGRLQVSHRKGLPHVIYCRLWRWPDLHSHHELRAMELCEFAFNMKKDEVCVNPYHYQRVET

>D1P7HL1

ELPMVERQDTDSCLVYGGQQMILTGQNFTSESKVVFTEKTTDGQQIWEMEATVDKDKSQPNMLFVEIPEYRNKHIRTPVKVNFYVINGKRKRSQPQHFTYHPV

>D1P7HL2

SSVPLEWPLSSQSGSYELRIEVQPKPHHRAHYETEGSRGAVKAPTGGHPVVQLHGYMENKPLGLQIFIGTADERILKPHAFYQVHRITGKTVTTTSYEKIVGNTKVLEIPLEPKNNMRATIDCAGILKLRNADIELRKGETDIGRKNTRVRLVFRVHIPESSGRIVSLQTASNPIECSQRSAH

>D1PDNC_

QGRVNQLGGVFINGRPLPNNIRLKIVEMAADGIRPCVISRQLRVSHGCVSKILNRYQETGSIRPGVIGGSKPRIATPEIENRIEEYKRSSPGMFSWEIREKLIREGVCDRSTAPSVSAISRLV

>D1QRVA_

SDKPKRPLSAYMLWLNSARESIKRENPGIKVTEVAKRGGELWRAMKDKSEWEAKAAKAKDDYDRAVKEFEANG

>D1QTMA1

ALEEAPWPPPEGAFVGFVLSRKEPMWADLLALAAARGGRVHRAPEPYKALRDLKEARGLLAKDLSVLALREGLGLPPGDDPMLLAYLLDPSNTTPEGVARRYGGEWTEEAGERAALSERLFANLWGRLEG

>D1QUQA_

HIVPCTISQLLSATLVDEVFRIGNVEISQVTIVGIIRHAEKAPTNIVYKIDDMTAAPMDVRQWVDTDDTSSENTVVPPETYVKVAGHLRSFQNKKSLVAFKIMPLEDMNEFTTHILEVINAHMVLSK

>D1QUQB_

DMMDLPRSRINAGMLAQFIDKPVCFVGRLEKIHPTGKMFILSDGEGKNGTIELMEPLDEEISGIVEVVGRVTAKATILCTSYVQFKEDSHPFDLGLYNEAVKIIHDFPQFYPLG

>D1QZGA_

VIDSLQLNELLNAGEYKIGELTFQSIRSSQELQKKNTIVNLFGIVKDFTPSRQSLHGTKDWVTTVYLWDPTCDTSSIGLQIHLFSKQGNDLPVIKQVGQPLLLHQITLRSYRDRTQGLSKDQFRYALWPDFSSNSKDTLCPQPMPRLMKTGDKEEQFALLLNKIWDEQTN

>D1R2ZA2

PQLPEVETIRRTLLPLIVGKTIEDVRIFWPNIIRHPRDSEAFAARMIGQTVRGLERRGKFLKFLLDRDALISHLRMEGRYAVASALEPLEPHTHVVFCFTDGSELRYRDVRKFGTMHVYAKEEADRRPPLAEL

>D1R71A_

EADQVIENLQRNELTPREIADFIGRELAKGKKKGDIAKEIGKSPAFITQHVTLLDLPEKIADAFNTGRVRDVTVVNELVTAFKKRPEEVEAWLDDDTQEITRGTVKLLREFLDE

>D1R8EA1

ESYYSIGEVSKLANVSIKALRYYDKIDLFKPAYVDPDTSYRYYTDSQLIHLDLIKSLKYIGTPLEEMKKAQDLEMEELFAFYTEQERQIREKLDFLSALEQTISLVKKRMKRQMEYPA

>D1R8EA2

LGEVFVLDEEEIRIIQTEAEGIGPENVLNASYSKLKKFIESADGFTNNSYGATFSFQPYTSIDEMTYRHIFTPVLTNKQISSITPDMEITTIPKGRYACIAYNFSPEHYFLNLQKLIKYIADRQLTVVSDVYELIIPIHYSPKKQEEYRVEMKIRIA

>D1REPC1

SPRIVQSNDLTEAAYSLSRDQKRMLYLFVDQIRKSDGTLQEHDGICEIHVAKYAEIFGLTSAEASKDIRQALKSFAGKEVVFYRPEEDAGDEKGYESFPWFIKPAHSPSRGLYSVHINPYLIPFFIGLQ

>D1REPC2

NRFTQFRLSETKEITNPYAMRLYESLCQYRKPDGSGIVSLKIDWIIERYQLPQSYQRMPDFRRRFLQVCVNEINSRTPMRLSYIEKKKGRQTTHIVFSFRDIT

>D1RH6A_

MYLTLQEWNARQRRPRSLETVRRWVRESRIFPPPVKDGREYLFHESAVKVDLNRP

>D1RRQA2

VKQVPLAVAVLADDEGRVLIRKRDSTGLLANLWEFPSCETDGADGKEKLEQMVGEQYGLQVELTEPIVSFEHAFSHLVWQLTVFPGRLVHGGPVEEPYRLAPEDELKAYAFPVSHQRVWREYKEWAS

>D1RXWA1

LTREQLIDIAILVGTDYNEGVKGVGVKKALNYIKTYGDIFRALKALKVNIDHVEEIRNFFLNPPVTDDYRIEFREPDFEKAIEFLCEEHDFSRERVEKALEKLKA

>D1RXWA2

ADIGDLFEREEVELEYFSGKKIAVDAFNTLYQFISIIRQPDGTPLKDSQGRITSHLSGILYRVSNMVEVGIRPVFVFDGEPPEFKKAEIEERKKRRAEAEEMWIAALQAGDKDAKKYAQAAGRVDEYIVDSAKTLLSYMGIPFVDAPSEGEAQAAYMAAKGDVEYTGSQDYDSLLFGSPRLARNLAITGKRKLPGKNVYVDVKPEIIILESNLKRLG

>D1RZTA1

ATNHNLHITEKLEVLAKAYSVQGDKWRALGYAKAINALKSFHKPVTSYQEACSIPGIGKRMAEKIIEILESGHLRKLDHI

>D1RZTA2

SESVPVLELFSNIWGAGTKTAQMWYQQGFRSLEDIRSQASLTTQQAIGLKHYSDFLE

>D1RZTA3

RMPREEATEIEQTVQKAAQAFNSGLLCVACGSYRRGKATCGDVDVLITHPDGRSHRGIFSRLLDSLRQEGFLTDDLVSQEENGQQQKYLGVCRLPGPGRRHRRLDIIVVPYSEFACALLYFTGSAHFNRSMRALAKTKGMSLSEHALSTAVVRNTHGCKVGPGRVLPTPTEKDVFRLLGLPYREPAERDW

>D1S32F_

AKRHRKVLRDNIQGITKPAIRRLARRGGVKRISGLIYEETRGVLKVFLENVIRDAVTYTEHAKRKTVTAMDVVYALKRQGRTLYGFGG

>D1SA3A_

MRTELLSKLYDDFGIDQLPHTQHGVTSDRLGKLYEKYILDIFKDIESLKKYNTNAFPQEKDISSKLLKALNLDLDNIIDVSSSDTDLGRTIAGGSPKTDATIRFTFHNQSSRLVPLNIKHSSKKKVSIAEYDVETICTGVGISDGELKELIRKHQNDQSAKLFTPVQKQRLTELLEPYRERFIRWCVTLRAEKSEGNILHPDLLIRFQVIDREYVDVTIKNIDDYVSDRIAEGSKARKPGFGTGLNWTYASGSKAKKMQFKG

>D1SFUA_

CTVNDAEIFSLVKKEVLSLNTNDYTTAISLSNRLKINKKKINQQLYKLQKEDTVKMVPSNPPKWFKNYNC

>D1SKNP_

GRQSKDEQLASDNELPVSAFQISEMSLSELQQVLKNESLSEYQRQLIRKIRRRGKNKVAARTCRQRRTDRHDKM

>D1SX5A_

SLRSDLINALYDENQKYDVCGIISAEGKIYPLGSDTAVLSTIFELFSRPIINKIAEKHGYIVEEPKQQNHYPDFTLYKPSEPNKKIAIDIKTTYTNKENEKIKFTLGGYTSFIRNNTKNIVYPFDQYIAHWIIGYVYTRVATRKSSLKTYNINELNEIPKPYKGVKVFLQDKWVIAGDLAGSGNTTNIGSIHAHYKDFVEGKGIFDSEDEFLDYWRNYERTSQLRNDKYNNISEYRNWIYRGRK

>D1T3NA1

QSFSEEDSFKKCSSEVEAKNKIEELLASLLNRVCQDGRKPHTVRLIIRRYSSEKHYGRESRQCPIPSHVIQKLGTGNYDVMTPMVDILMKLFRNMVNVKMPFHLTLLSVCFCNLK

>D1TC3C_

PRGSALSDTERAQLDVMKLLNVSLHEMSRKISRSRHCIRVYLKDPVSYGTS

>D1TDZA1

GPEPTYEDFDEKLFREKLRKSTKKIKPYLLEQTLVAGLGNIYVDEVLWLAKIHPEKETNQLIESSIHLLHDSIIEILQKAIKLGGSSI

>D1TK5A1

MIVSDIEANALLESVTKFHCGVIYDYSTAEYVSYRPSDFGAYLDALEAEVARGGLIVFHNGHKYDVPALTKLAKLQLNREFHLPRENCIDTLVLSRLIHSNLKDTDMGLLRSGKLPGALEAWGYRLGEMKGEYKDDFKRMLEEQGEEYVDGMEWWNFNEEMMDYNVQDVVVTKALLEKLLSDKHYFPPEIDFTDVGYTTFWSES

>D1TK5A2

LEAVDIEHRAAWLLAKQERNGFPFDTKAIEELYVELAARRSELLRKLTETFGSWYQPKGGTEMFCHPRTGKPLPKYPRIKTPKVGGIFKKPKNKAQREGREPCELDTREYVAGAPYTPVEHVVFNPSSRDHIQKKLQEAGWVPTKYTDKGAPVVDDEVLEGVRVDDPEKQAAIDLIKEYLMIQKRIGQSAEGDKAWLRYVAEDGKIHGSVNPNGAVTGRATHAFPNLAQIPGVRSPYGEQCRAAFGAEHHLDGITGKPWVQAGIDASGLELRCLAHFMARFDNGEYAHEILNGDIHTKNQIAAELPTRDNAKTFIYGFLYGAGDEKIGQIVGAGKERGKELKKKFLENTPAIAALRESIQQTLVESSQWVAGEQQVKWKRRWIKGLDGRKVHVRSPHAALNTLLQSAGALICKLWIIKTEEMLVEKGLKHGWDGDFAYMAWVHDEIQVGCRTEEIAQVVIETAQEAMRWVGDHWNFRCLLDTEGKMGPNWAICH

>D1TTUA1

DKAEYRFFEAMGQVANPISPCPVVGSLEVDGHGEASRVELHGRDFKPNLKVWFGATPVETTFRSEESLHCSIPPVSQVRNEQTHWMFTNRTTGDVEVPISLVRDDGVVYSSGLTFSYKS

>D1TTUA2

QSLTSDRMIDFLSNKEKYECVISIFHAKVAQKSYGNEKRFFCPPPCIYLIGQGWKLKKDRVAQLYKTLKASAQKDAAIENDPIHEQQATELVAYIGIGSDTSERQQLDFSTGKVRHPGDQRQDPNIYDYCAAKTLYISDSDKRKYFDLNAQFFYGCGMEIGGFVSQRIKVISKPSKKKQSMKNTD

>D1TTUA3

CKYLCIASGTKVALFNRLRSQTVSTRYLHVEGNAFHASSTKWGAFTIHLFDDERGLQETDNFAVRDGFVYYGSVVKLVDSVTGIALPRLRIRKVDKQQVILDASCSEEPVSQLHKCAFQMIDNELVYLCLSHDKIIQHQATAINEHRHQINDGAAWTIIST

>D1U3EM1

MEWKDIKGYEGHYQVSNTGEVYSIKSGKTLKHQIPKDGYHRIGLFKGGKGKTFQVHRLVAIHFCEGYEEGLVVDHKDGNKDNNLSTNLRWVTQKINVENQMSRGT

>D1U3EM2

LNVSKAQQIAKIKNQKPIIVISPDGIEKEYPSTKCACEELGLTRGKVTDVLKGHRIHHKGYTFRYKLNG

>D1U4BA1

MAFTLADRVTEEMLADKAALVVEVVEENYHDAPIVGIAVVNEHGRFFLRPETALADPQFVAWLGDETKKKSMFDSKRAAVALKWKGIELCGVSFDLLLAAYLLDPAQGVDDVAAAAKMKQYEAVRPDEAVYGKGAKRAVPDEPVLAEHLVRKAAAIWELERPFLDELRRN

>D1U4BA2

EQDRLLVELEQPLSSILAEMEFAGVKVDTKRLEQMGKELAEQLGTVEQRIYELAGQEFNINSPKQLGVILFEKLQLPVLKKTKTGYSTSADVLEKLAPYHEIVENILHYRQLGKLQSTYIEGLLKVVRPDTKKVHTIFNQALTQTGRLSSTEPNLQNIPIRLEEGRKIRQAFVPSESDWLIFAADYSQIELRVLAHIAEDDNLMEAFRRDLDIHTKTAMDIFQVSEDEVTPNMRRQAKAVNFGIVYGISDYGLAQNLNISRKEAAEFIERYFESFPGVKRYMENIVQEAKQKGYVTTLLHRRRYLPDITSRNFNVRSFAERMAMNTPIQGSAADIIKKAMIDLNARLKEERLQAHLLLQVHDELILEAPKEEMERLCRLVPEVMEQAVTLRVPLKVDYHYGSTWYDAK

>D1U78A2

APRRKALSVRDERNVIRAASNSCKTARDIRNELQLSASKRTILNVIKRSG

>D1UBDC1

TIACPHKGCTKMFRDNSAMRKHLHTHGP

>D1UBDC2

RVHVCAECGKAFVESSKLKRHQLVHTGE

>D1VPWA1

TIKDVAKRANVSTTTVSHVINKTRFVAEETRNAVWAAIKELHYSPSAVARSMKVNH

>D1ZMEC1

SVACLSCRKRHIKCPGGNPCQKCVTSNAICEYLEPS

>D2BOPA_

SCFALISGTANQVKCYRFRVKKNHRHRYENCTTTWFTVADNGAERQGQAQILITFGSPSQRQDFLKHVPLPPGMNISGFTASLDF

>D2DNJA_

LKIAAFNIRTFGETKMSNATLASYIVRIVRRYDIVLIQEVRDSHLVAVGKLLDYLNQDDPNTYHYVVSEPLGRNSYKERYLFLFRPNKVSVLDTYQYDDGCESCGNDSFSREPAVVKFSSHSTKVKEFAIVALHSAPSDAVAEINSLYDVYLDVQQKWHLNDVMLMGDFNADCSYVTSSQWSSIRLRTSSTFQWLIPDSADTTATSTNCAYDRIVVAGSLLQSSVVPGSAAPFDFQAAYGLSNEMALAISDHYPVEVTLT

>D2DRPA1

FTKEGEHTYRCKVCSRVYTHISNFCRHYVTSHKRNVK

>D2DRPA2

VYPCPFCFKEFTRKDNMTAHVKIIHK

>D2GLIA1

ETDCRWDGCSQEFDSQEQLVHHINSEHIHGER

>D2GLIA2

KEFVCHWGGCSRELRPFKAQYMLVVHMRRHTGE

>D2IRFG_

RMRMRPWLEEQINSNTIPGLKWLNKEKKIFQIPWMHAARHGWDVEKDAPLFRNWAIHTGKHQPGIDKPDPKTWKANFRCAMNSLPDIEEVKDRSIKKGNNAFRVYRMLP

>D3PVIA_

SHPDLNKLLELWPHIQEYQDLALKHGINDIFQGNGGKLLQVLLITGLTVLPGREGNDAVDNAGQEYELKSINIDLTKGFSTHHHMNPVIIAKYRQVPWIFAIYRGIAIEAIYRLEPKDLEFYYDKWERKWYSDGHKDINNPKIPVKYVMEHGTKIY

(2).non DNA-binding proteins

>2V2GA

MGITLGEVFPNFEADSTIGKLKFHDWLGNSWGVLFSHPRDFTPVSTTELGRVIQLEGDFKKRGVKLIALSCDNVADHKEWSEDVKCLSGVKGDMPYPIIADETRELAVKLGMVDPDERTSTGMPLTCRAVFIIGPDKKLKLSILYPATTGRNFSEILRVIDSLQLTAQKKVATPADWQPGDRCMVVPGVSAEEAKTLFPNMEVKAVPSGKGYLRYTPQPKSMGGSRSHHHHHH

>3D33A

GANTTETPVKDVELDGRWDDPIRSAATNCPITVFTDGYLLTLKNASPDRDMTIRITDMAKGGVVYENDIPEVQSAYITISIANFPAEEYKLEITGTPSGHLTGYFTKE

>2J9OA

MPAPEAPTSTLPPERPLTNLQQQIQQLVSRQPNLTAGLYFFNLDSGASLNVGGDQVFPAASTIKFPILVAFFKAVDEGRVTLQERLTMRPDLIAPEAGTLQYQKPNSQYAALEVAELMITISDNTATNMIIDRLGGAAELNQQFQEWGLENTVINNPEPDMKGTNTTSPRDLATLMLKIGQGEILSPRSRDRLLDIMRRTVTNTLLPAGLGKGATIAHKTGDIGIVVGDAGMVDMPNGQRYVAAMMVKRPYNDPRGSELIRQVSRMVYQAFEKLSPPEQKLISEEDLNSAVDHHHHHH

>2WI8A

MGSKNESTASKASGTASEKKKIEYLDKTYEVTVPTDKIAITGSVESMEDAKLLDVHPQGAISFSGKFPDMFKDITDKAEPTGEKMEPNIEKILEMKPDVILASTKFPEKTLQKISTAGTTIPVSHISSNWKENMMLLAQLTGKEKKAKKIIADYEQDLKETKTKINDKAKDSKALVIRIRQGNIYIYPEQVYFNSTLYGDLGLKAPNEVKAAKAQELISLEKLSEMNPDHIFVQFSDDENADKPDALKDLEKNPIWKSLKAVKEDHVYVNSVDPLAQGGTAWSKVRFLKAAAEKLTQNKLAAALEHHHHHH

>4JDNA

SLGLLKAFNNFPITNKIQCNGLFTPSNIETLLGGTEIGKFTVTPKSSGSMFLVSADIIASRMEGGVVLALVREGDSKPCAISYGYSSGVPNLCSLRTSITNTGLTPTTYSLRVGGLESGVVWVNALSNGNDILGITNTSNVSFLEVIPQTNA

>2PR7A

GMRGLIVDYAGVLDGTDEDQRRWRNLLAAAKKNGVGTVILSNDPGGLGAAPIRELETNGVVDKVLLSGELGVEKPEEAAFQAAADAIDLPMRDCVLVDDSILNVRGAVEAGLVGVYYQQFDRAVVEIVGLFGLEGEF

>3G8YA

GYQPEKHAVVKSDRGDGRLLSTYAIVHEMLKDTHPQYAYRSGMSAQEFTQWQDGVRAAMVEIMKFPEIKRQPSPVCVKTEKKEGYILEKWEFYPFPKSVSTFLVLKPEHLKGAVPGVLCIPGSGRTKEGLVGEPGICDKLTEDYNNPKVSMALNMVKEGYVAVAVDNAAAGEASDLECYDKGWNYDYDVVSRFLLELGWSWLGYTSYLDMQVLNWMKAQSYIRKDRIVISGFSLGTEPMMVLGVLDKDIYAFVYNDFLCQTQERAVVMTKPDKENRRPFPNSIRHLIPGYWRYFNFPDVVASLAPRPIIFTEGGLDRDFRLVQSAYAASGKPENAEFHHYPKFADKAVRKDVEHLDEGLDSKTYFEAVNVDPPSHYFKNELVIPWLRKVLK

>3MC9A

MLETRATTAKQLVQAPEQPAPQPEVAPPTTEQPAPAPAPGTTPGTENFNTPNATPETTEPRVLVSEVLVRPQSGQLTPELETQVYNVIRTQPGRTTTRSQLQEDINAIFGTGFFSNVQASPEDTPLGVRVSFIVQPNPVLSKVEIQANPGTNVPSVLPQATADEIFRAQYGKILNLRDLQEGIKELTKRYQDQGYVLANVVGAPQVSENGVVTLQVAEGVVENISVRFRNKEGQDVNEQGQPIRGRTQDYIITREVELKPGQVFNRNTVQKDLQRVFGTGLFEDVNVSLDPGTDPTKVNVVVNVVERSLEHHHHHH

>4EIRA

HTIFSSLEVNGVNQGLGEGVRVPTYNGPIEDVTSASIACNGSPNTVASTSKVITVQAGTNVTAIWRYMLSTTGDSPADVMDSSHKGPTIAYLKKVDNAATASGVGNGWFKIQQDGMDSSGVWGTERVINGKGRHSIKIPECIAPGQYLLRAEMIALHAASNYPGAQFYMECAQLNVVGGTGAKTPSTVSFPGAYSGSDPGVKISIYWPPVTAYTVPGPSVFTC

>3O1IC

GSGSDEKICAIYPHLKDSYWLSVNYGMVSEAEKQGVNLRVLEAGGYPNKSRQEQQLALCTQWGANAIILGTVDPHAYEHNLKSWVGNTPVFATVNQLDLDEEQSTLLKGEVGVDWYWMGYEAGKYLAERHPKGSGKTNIALLLGPRTRGGTKPVTTGFYEAIKNSDIHIVDSFWADNDKELQRNLVQRVIDMGNIDYIVGSAVAIEAAISELRSADKTHDIGLVSVYLSHGVYRGLLRNKVLFAPTDKMVQQGRLSVMQAAHYLRHQPYEKQASPIIKPLTPKTLHDDTIEESLSPSEYRPTFS

>4KQ9A

GGGSAAGKKVVYSTFGAQIPFFNRIGEGAKAQATVRRLDFDISTSEIDPGKQIDSIDNAVAQQPDGLIVSPIDGSALVPTIKGAVEDGVPVILLADGLSEDVGQLSFVGSDFAEIGRLKATYIADRLGDGGTVAMVNGTRGMSFVEEQGEAAREVFEERGIEIVDDVYTKAITPDEGLTATQNILTRHSDVGAIYYSGDDGALGGIRAIAARNIAPGKIMVVGTDANEGALAAVRAGTMALTVSQCAYEQGGIAIDVMADYLETGKKPDRRIFTPVIEIDTETIDRVMSGAAWERCENH

>3F47A

MKIAILGAGCYRTHAAAGITNFMRACEVAKEVGKPEIALTHSSITYGAELLHLVPDVKEVIVSDPCFAEEPGLVVIDEFDPKEVMEAHLSGNPESIMPKIREVVKAKAKELPKPPKACIHLVHPEDVGLKVTSDDREAVEGADIVITWLPKGNKQPDIIKKFADAIPEGAIVTHACTIPTTKFAKIFKDLGREDLNITSYHPGCVPEMKGQVYIAEGYASEEAVNKLYEIGKIARGKAFKMPANLIGPVCDMCSAVTATVYAGLLAYRDAVTKILGAPADFAQMMADEALTQIHNLMKEKGIANMEEALDPAALLGTADSMCFGPLAEILPTALKVLEKHKVVEEEGKTKCEIMSQKE

>1GKPA

PLLIKNGEIITADSRYKADIYAEGETITRIGQNLEAPPGTEVIDATGKYVFPGFIDPHVHIYLPFMATFAKDTHETGSKAALMGGTTTYIEMCCPSRNDDALEGYQLWKSKAEGNSYCDYTFHMAVSKFDEKTEGQLREIVADGISSFKIFLSYKNFFGVDDGEMYQTLRLAKELGVIVTAHCENAELVGRLQQKLLSEGKTGPEWHEPSRPEAVEAEGTARFATFLETTGATGYVVHLSCKPALDAAMAAKARGVPIYIESVIPHFLLDKTYAERGGVEAMKYIMSPPLRDKRNQKVLWDALAQGFIDTVGTDHCPFDTEQKLLGKEAFTAIPNGIPAIEDRVNLLYTYGVSRGRLDIHRFVDAASTKAAKLFGLFPRKGTIAVGSDADLVVYDPQYRGTISVKTQHVNNDYNGFEGFEIDGRPSVVTVRGKVAVRDGQFVGEKGWGKLLRREPMYF

>2GK9A

SMHFVQQKVKVFRAADPLVGVFLWGVAHSINELSQVPPPVMLLPDDFKASSKIKVNNHLFHRENLPSHFKFKEYCPQVFRNLRDRFGIDDQDYLVSLTRNPPSESEGSDGRFLISYDRTLVIKEVSSEDIADMHSNLSNYHQYIVKCHGNTLLPQFLGMYRVSVDNEDSYMLVMRNMFSHRLPVHRKYDLKGSLVSREASDKEKVKELPTLRDMDFLNKNQKVYIGEEEKKIFLEKLKRDVEFLVQLKIMDYSLLLGIHDIIRGSEPEEEAPVREDESEVDGDCSLTGPPALVGSYGTSPEGIGGYIHSHRPLGPGEFESFIDVYAIRSAEGAPQKEVYFMGLIDILTQYDAKKKAAHAAKTVKHGAGAEISTVHPEQYAKRFLDFITNIFA

>3JTWA

GMARKVILFIAMSIDNYIADDQGAVDWLEKNVHGTESDDSYEKMYSKIDTVIMGRTTYEQVTQKLSPEKYVYADRQTYIVTSHLGEDTDKIKYWKQSPVELVKRIQKEKGKDVWIVGGAKIIDPLVQANLIDTYILTTVPIFLGSGIRLFDRLEEQVPVRLIDVYQKNELVYSIYQRG

>3CNEA

MAKKVAVLAVNPVNGCGLFQYLEAFFENGISYKVFAVSDTKEIKTNSGMVLIVDDVIANLKGHEDEFDALVFSCGDAVPVFQQYANQPYNVDLMEVIKTFGEKGKMMIGHCAGAMMFDFTGITKGKKVAVHPLAKPAIQNGIATDEKSEIDGNFFTAQDENTIWTMLPKVIEALK

>2VN6A

TVLPKDIPGDSLKVTVGTANGKPGDTVTVPVTFADVAKMKNVGTCNFYLGYDASLLEVVSVDAGPIVKNAAVNFSSSASNGTISFLFLDNTITDELITADGVFANIKFKLKSVTAKTTTPVTFKDGGAFGDGTMSKIASVTKTNGSVTIDP

>3OY2A

MKLIIVGAHSSVPSGYGRVMRAIVPRISKAHEVIVFGIHAFGRSVHANIEEFDAQTAEHVRGLNEQGFYYSGLSEFIDVHKPDIVMIYNDPIVIGNYLLAMGKCSHRTKIVLYVDLVSKNIRENLWWIFSHPKVVGVMAMSKCWISDICNYGCKVPINIVSHFVDTKTIYDARKLVGLSEYNDDVLFLNMNRNTARKRLDIYVLAAARFISKYPDAKVRFLCNSHHESKFDLHSIALRELVASGVDNVFTHLNKIMINRTVLTDERVDMMYNACDVIVNCSSGEGFGLCSAEGAVLGKPLIISAVGGADDYFSGDCVYKIKPSAWISVDDRDGIGGIEGIIDVDDLVEAFTFFKDEKNRKEYGKRVQDFVKTKPTWDDISSDIIDFFNSLLRVESRETPGNEEHPLEHHHHHH

>3HH1A

SNAHKGTLYVVATPLGNLDDMTFRAVNTLRNAGAIACEDTRRTSILLKHFGIEGKRLVSYHSFNEERAVRQVIELLEEGSDVALVTDAGTPAISDPGYTMASAAHAAGLPVVPVPGA

>1Z2NX

GPLGSMTTKQTVSLFIWLPESKQKTLFISTKNHTQFELNNIIFDVTLSTELPDKEPNAIITKRTHPVGKMADEMRKYEKDHPKVLFLESSAIHDMMSSREEINALLIKNNIPIPNSFSVKSKEEVIQLLQSKQLILPFIVKPENAQGTFNAHQMKIVLEQEGIDDIHFPCLCQHYINHNNKIVKVFCIGNTLKWQTRTSLPNVHRCGIKSVDFNNQHLEDILSWPEGVIDKQDIIENSANRFGSKILEDPILLNLTSEAEMRDLAYKVRCALGVQLCGIDFIKENEQGNPLVVDVNVFPSYGGKVDFDWFVEKVALCYTEVAKI

>3GREA

MGHHHHHHGEGDVESIEKFLSTFKILPPLRDYKEFGPIQEIVRSPNMGNLRGKLIATLMENEPNSITSSAVSPGETPYLITGSDQGVIKIWNLKEIIVGEVYSSSLTYDCSSTVTQITMIPNFDAFAVSSKDGQIIVLKVNHYQQESEVKFLNCECIRKINLKNFGKNEYAVRMRAFVNEEKSLLVALTNLSRVIIFDIRTLERLQIIENSPRHGAVSSICIDEECCVLILGTTRGIIDIWDIRFNVLIRSWSFGDHAPITHVEVCQFYGKNSVIVVGGSSKTFLTIWNFVKGHCQYAFINSDEQPSMEHFLPIEKGLEELNFCGIRSLNALSTISVSNDKILLTDEATSSIVMFSLNELSSSKAVISPSRFSDVFIPTQVTANLTMLLRKMKRTSTHSVDDSLYHHDIINSISTCEVDETPLLVACDNSGLIGIFQ

>1EZGA

QCTGGADCTSCTGACTGCGNCPNAVTCTNSQHCVKANTCTGSTDCNTAQTCTNSKDCFEANTCTDSTNCYKATACTNSSGCPGH

>2WNVB

ATQKIAFSATRTINVPLRRDQTIRFDHVITNMNNNYEPRSGKFTCKVPGLYYFTYHASSRGNLCVNLMRGRERAQKVVTFCDYAYNTFQVTTGGMVLKLEQGENVFLQATDKNSLLGMEGANSIFSGFLLFPDMEA

>4IQNA

SNAMDISLTNLIELVKKVNRNKVPTPMSAEEISRLRVRKYRDPQNTETTELPESLKALLAYDRDLLSNYNMPVIETLQKSIDNEGVIHSYSPDEEAYYGVGMDSSGIDIEDLMPVWSNDPRLPALIRIDHVGDQAIFIYITERDANGEYPIARMERNEFWLAESSLVEYLYNIISGAKDIGFTEEDLHLPQWKAQQKMNEQRDAALLDLEDYHEAFWAKLDALVD

>2PA7A

GHMENKVINFKKIIDSRGSLVAIEENKNIPFSIKRVYYIFDTKGEEPRGFHAHKKLEQVLVCLNGSCRVILDDGNIIQEITLDSPAVGLYVGPAVWHEMHDFSSDCVMMVLASDYYDETDYIRQYDNFKKYIAKINLEKEG

>3HL6A

MDIETIVNEFETRAGTLLRYYTGLLERSKVQPCCFKLYNDPFDMVYVMMNSKLFSHVYIKDCKVRQSFELASPKHTEGLIRSIEGHYVGYELHDGKQLSISDMMASQLFEDEYFMYGLQTYAESNNSDVFKCLENGFDTDTLEGIQSSNTDVIANIEMLYQLATGINEPVPELVEGLKLVTEFVQDENATQEDYKALERKLNDLKASYYSLSKLAAALEHHHHHH

>3P06A

NGVELSAVGVLLPVLMDSGRRISGGAFMAVKGDLSEHIKNPKNTRIAQTVAGGTIYGLSEMVNIDEAEKLPIKGAITVLPVVQATATSILVPDNQPQLAFNSWEAAACAADTLESQQTPFLMVTGAVESGNLSPNLLAVQKQLLVAKPAGIGLAANSDRALKVVTLEQLRQVVGDKPWRKPMVTFSSGKNVAQA

>3KP8A

SPLAVGLAAHLRQIGGTMYGAYWCPHCQDQKELFGAAFDQVPYVECSPNGPGTPQAQECTEAGITSYPTWIINGRTYTGVRSLEALAVASGYPLEEGRLEHHHHHH

>3HG9A

MSLTSSAELAEVDTLARSLLLYRSRLAEYAHANPGFSGSPADSALGLPAWFRKPVRLQGYIAAGTSYAFIASPPAGLAAAVDTGTESDLVGVRRNGQLVTRRLGATAIALPAPIPEGAVVAVKEGHHHHHH

>3H0UA

MSLTASYETIKARLDGTVLSATFNAPPMNLIGPEVVRDLVALLEELAHPTAPRVVIFDSADADFFFPHVDMTKVPEYTAEAAKAGGPGDASLGMLFRKLSQLPAVTIAKLRGRARGAGSEFLLACDMRFASRENAILGQPEVGIGAPPGAGAIQHLTRLLGRGRALEAVLTSSDFDADLAERYGWVNRAVPDAELDEFVAGIAARMSGFPRDALIAAKSAINAISLPAPAEVRADAALFQQLVRGEKVQQRTAELFKQGFQTRGATELDLGDALGHLKAVDEGHHHHHH

>1UUYA

VPGPEYKVAILTVSDTVSAGAGPDRSGPRAVSVVDSSSEKLGGAKVVATAVVPDEVERIKDILQKWSDVDEMDLILTLGGTGFTPRDVTPEATKKVIERETPGLLFVMMQESLKITPFAMLARSAAGIRGSTLIINMPGNPNAVAECMEALLPALKHALKQIKGDKR

>3ZBOA

GSHMASMDFKTVMQELEALGKERTKKIYISNGAHEPVFGVATGAMKPIAKKIKLNQELAEELYATGNYDAMYFAGIIADPKAMSESDFDRWIDGAYFYMLSDYVVAVTLSESNIAQDVADKWIASGDELKMSAGWSCYCWLLGNRKDNAFSESKISDMLEMVKDTIHHSPERTKSAMNNFLNTVAISYVPLHEKAVEIAKEVGIVEVKRDNKKSSLLNASESIQKELDRGRLGFKRKYVRC

>3DDEA

GMSIIDLTKLEQKVATMWDSILTNSPFIHEVLDGKATKALYAIYMTETYHYTKHNAKNQALVGIMGKDLPGKYLSFCFHHAHEEAGHELMALSDIASIGFDREDVLSSKPLPATETLIAYLYWISATGNPVQRLGYSYWAENVYGYIDPVLKAIQSTLDLTPQSMKFFIAHSKIDAKHAEEVNEMLHEVCKTQEDVDSVVAVMENSLVLTARILDDVWKEYQLFQSGASDRYAFLRDNA

>1GWMA

MNVRATYTVIFKNASGLPNGYDNWGWGCTLSYYGGAMIINPQEGKYGAVSLKRNSGSFRGGSLRFDMKNEGKVKILVENSEADEKFEVETISPSDEYVTYILDVDFDLPFDRIDFQDAPGNGDRIWIKNLVHSTGSADDFVDPINLEHHHHHH

>3HXLA

MAAGTFTAQNKVRPGVYINFKSEPQAAGTLGERGIVSMPLILSWGEPGKMITIEAGDDVFPKLGYSIMDAQLRLINEALKRAKTLLLYRLNAGTKAAVTVGNLTVTAKWGGARGNDITLVIQENIDDETKFDVSTLVDGAELDKQTVSDIAGLAANDWVIFSGTGALTETAGAPLINGSDGAVTNQAYIDYLAAVEIFDFNTIALPSTDDALKATFTAFAKRLRDDEGKKIQVVLENYPAADYEGVISVKNGVVLADGTILTAAQATAWVAGATAGARVNESLTYQGYDEAVDVAPRYTNAQIIAALQAGEFLFTASDNQALVEQDINTLTSFTADKGKQFAKNRVIRVLDGINNDFVRIFSKFYIGKVSNNADGRNLLKSECINYMNTLQDIDAIKNFDGQTDLTVQSGNDVDAVYIEAYAWPVDSIEKIYVRVRIKLEHHHHHH

>3DCPA

MKRDGHTHTEFCPHGTHDDVEEMVLKAIELDFDEYSIVEHAPLSSEFMKNTAGDKEAVTTASMAMSDLPYYFKKMNHIKKKYASDLLIHIGFEVDYLIGYEDFTRDFLNEYGPQTDDGVLSLHFLEGQGGFRSIDFSAEDYNEGIVQFYGGFEQAQLAYLEGVKQSIEADLGLFKPRRMGHISLCQKFQQFFGEDTSDFSEEVMEKFRVILALVKKRDYELDFNTAGLFKPLCGETYPPKKIVTLASELQIPFVYGSDSHGVQDIGRGYSTYCQKLEHHHHHH

>4H14A

VIGDLKCTTVSINDVDTGAPSISTDTVDVTNGLGTYYVLDRVYLNTTLLLNGYYPTSGSTYRNMALKGTLLLSRLWFKPPFLSDFINGIFAKVKNTKVIKKGVMYSEFPAITIGSTFVNTSYSVVVQPHTTNLDNKLQGLLEISVCQYTMCEYPHTICHPKLGNKRVELWHWDTGVVSCLYKRNFTYDVNADYLYFHFYQEGGTFYAYFTDTGVVTKFLFNVYLGTVLSHYYVLPLTCSSAMTLEYWVTPLTSKQYLLAFNQDGVIFNAVDCKSDFMSEIKCKTHHHHHH

>1O57A

MKFRRSGRLVDLTNYLLTHPHELIPLTFFSERYESAKSSISEDLTIIKQTFEQQGIGTLLTVPGAAGGVKYIPKMKQAEAEEFVQTLGQSLANPERILPGGYVYLTDILGKPSVLSKVGKLFASVFAEREIDVVMTVATKGIPLAYAAASYLNVPVVIVRKDNKVTEGSTVSINYVSGSSNRIQTMSLAKRSMKTGSNVLIIDDFMKAGGTINGMINLLDEFNANVAGIGVLVEAEGVDERLVDEYMSLLTLSTINMKEKSIEIQNGNFLRFFKDNLLKNGETESHHHHHH

>3EZ0A

GMSTSPADTARYNRFVADLFGMMAYGELSAFERFSADARYSPTLHDRAVLGRIAVVEFRHYELVSARLEAMGIDAEDAMLPFQAAVDYFHSRTRPADWYESLMKAYVIDTVSADFYRAISRYVDAGTRDVIEQIQASDETTEVLRERLRSALADDPRLASRLALWGRRLLGEALTQAQRVSYEHAFLGSLIGGEDSAAAKELVSGLIAGLAEKHSKRMTQLGLTG

>2VZPA

SDPVDYQAEDATIVQGAVESNHAGYTGTGFVNYDNVAGSSVEWTVTVPSAGTYDVVVRYANGTTTSRPLDFSVNGSISASGVAFGSTGTWPAWTTKTVRVTLAAGVNKIKAVATTANGGPNVDKITL

>4DOGA

GSHMNSVTVSHAPYTITYHNDWEPVMSQLVEFYNEVASWLLRDETSPIPDKFFIQLKQPLRNKRVCVCGIDPYPKDGTGVPFESPNFTKKSIKEIASSISRLTGVIDYKGYNLNIIDGVIPWNYYLSCKLGETKSHAIYWDKISKLLLQHITKHVSVLYCLGKTDFSNIRAKLESPVTTIVGYHPAARDRQFEKDRSFEIINVLLELDNKAPINWAQGFIY

>2XTCA

MSITSDEVNFLVYRYLQESGFSHSAFTFGIESHISQSNINGTLVPPAALISILQKGLQYVEAEISINEDGTVFDGRPIESLSLIDAVMPD

>2RBDA

GMGILSGNPQDEPLHYGEVFSTWTYLSTNNGLINGYRSFINHTGDEDLKNLIDEAIQAMQDENHQLEELLRSNGVGLPPAPPDRPAARLDDIPVGARFNDPEISATISMDVAKGLVTCSQIIGQSIREDVALMFSQFHMAKVQFGGKMLKLNKNKGWLIPPPLHSDRPIKE

>2I1SA

MKKTFEKVYHLKLSIKGITPQIWRRIQVPENYTFLDLHKAIQAVMDWEDYHLHEFEMVNPKTGMLDKIGAEGDDFDAFGGPLVSEKKAKLSDYFTLENKEALYTYDFGDNWQVKVRLEKILPRKEGVEYPICTAGKRAAVPEDSGGVWGYEEMLEVLKDSEHEEYEDTVLWLGDDFDPEYFDPKDVSF

>3K8GA

GSGAWKASVDPLGVVGSGADVYLYFPVAGNENLISRIIENHESKADIKKIVDRTTAVYGAFFARSKEFRLFGSGSYPYAFTNLIFSRSDGWASTKTEHGITYYESEHTDVSIPAPHFSCVIFGSSKRERMSKMLSRLVNPDRPQLPPRFEKECTSEGTSQTVALYIKNGGHFITKLLNFPQLNLPLGAMELYLTARRNEYLYTLSLQLGNAKINFPIQFLISRVLNAHIHVEGDRLIIEDGTISAERLASVISSLYSKKGSS

>3PVHA

SASEFNILNDGPPKETYVVDDAGVLSRVTKSDLKKLLSDLEYRKKLRLNFITVRKLTSKADAFEYADQVLEKWYPSIEEGNNKGIVVLITSQKEGAITGGPAFIEAVGENILDATVSENLPVLATDEKYNEAVYSSAKRLVAAIDGQPDPGGP

>1TIQA

MSVKMKKCSREDLQTLQQLSIETFNDTFKEQNSPENMKAYLESAFNTEQLEKELSNMSSQFFFIYFDHEIAGYVKVNIDDAQSEEMGAESLEIERIYIKNSFQKHGLGKHLLNKAIEIALERNKKNIWLGVWEKNENAIAFYKKMGFVQTGAHSFYMGDEEQTDLIMAKTLILEHHHHHH

>3BRVA

AMAPAKKSEELVAEAHNLCTLLENAIQDTVREQDQSFTALDWSWLQTE

>4KVHA

SNAMDRVATARAYYRALDEHDYDLLSDVLAPDFVHDRPDRTIEGRERFVRFMREERPQTDTSHPIATIYTGASTVAVEGRLLNSDGAEITQFVDVFAFEDGVIGRIRTHTPEP

>4JERA

MSTTIQYNSNYADYSISSYLREWANNFGDIDQAPAETKDRGSFSGSSTLFSGTQYAIGSSHSNPEGMIAEGDLKYSFMPQHTFHGQIDTLQFGKDLATNAGGPSAGKHLEKIDITFNELDLSGEFDSGKSMTENHQGDMHKSVRGLMKGNPDPMLEVMKAKGINVDTAFKDLSIASQYPDSGYMSDAPMVDTV

>3BA3A

GMDISLLKQVVQSTNKIALSTAVNNEADVKIVNFVWYEAQPDTLYFSSVKTSPALKVYDQNPDIAFITIPNDGTAGNPYLRAQHVKLQRSTKTMTDLLPQYLETVPNYQQVWDAIGSTLVVFELKLTDLFVDAGVGGEKQTLTFN

>2ZAHA

NISYTEGAKPGAISAPVAISRRVAGMKPRFVRSEGSVKIVHREFIASVLPSNDLTVNNGDVNIGKYRVNPSNNALFTWLQGQAQLYDMYRFTRLRFTYIPTTGSTSTGRVSILWDRDSQDPLPIDRAAISSYAHYADSAPWAENVLVVPCDNTWRYMNDTNAVDRKLVDFGQFLFATYSGAGATAHGDLYVEYAVEFKDPQPIAGMVCMFDRLVSFSEVGSTIKGVNYIADRDVITTGGNIGVNINIPGTYLVTIVLNATSIGSLTFTGNSKLVGNSLNVTSSGASALTFTLNSTGVPNSSNSSFSVGTVVALTRVRMTITRCSPETAYLA

>1JQ5A

MAAERVFISPAKYVQGKNVITKIANYLEGIGNKTVVIADEIVWKIAGHTIVNELKKGNIAAEEVVFSGEASRNEVERIANIARKAEAAIVIGVGGGKTLDTAKAVADELDAYIVIVPTAASTDAPTSALSVIYSDDGVFESYRFYKKNPDLVLVDTKIIANAPPRLLASGIADALATWVEARSVIKSGGKTMAGGIPTIAAEAIAEKCEQTLFKYGKLAYESVKAKVVTPALEAVVEANTLLSGLGFESGGLAAAHAIHNGFTALEGEIHHLTHGEKVAFGTLVQLALEEHSQQEIERYIELYLCLDLPVTLEDIKLKDASREDILKVAKAATAEGETIHNAFNVTADDVADAIFAADQYAKAYKEKHRK

>1P9YA

GSHMQVSVETTQGLGRRVTITIAADSIETAVKSELVNVAKKVRIDGLRKGKVPMNIVAQRYGASVRQDVLGDLMSRNFIDAIIKEKINPAGAPTYVPGEYKLGEDFTYSVEFEVYPEVELQ

>3EYEA

MSLSSPNILLTRIDNRLVHGQVGVTWTSTIGANLLVVVDDVVANDDIQQKLMGITAETYGFGIRFFTIEKTINVIGKAAPHQKIFLICRTPQTVRKLVEGGIDLKDVNVGNMHFSEGKKQISSKVYVDDQDLTDLRFIKQRGVNVFIQDVPGDQKEQIPDEGHHHHHH

>3RKCA

SRPFSVLRANDVLWLSLTAAEYDQTTYGSSTNPMYVSDTVTFVNVATGAQGVSRSLDWSKVTLDGRPLTTIQQYSKTFFVLPLRGKLSFWEAGTTKAGYPYNYNTTASDQILIENAPGHRVCISTYTTNLGSGPVSISAVGVLAPHSA

>3DUZA

AEHCNAQMKTGPYKIKNLDITPPKETLQKDVEITIVETDYNENVIIGYKGYYQAYAYNGGSLDPNTRVEETMKTLNVGKEDLLMWSIRQQCEVGEELIDRWGSDSDDCFRDNEGRGQWVKGKELVKRQNNNHFAHHTCNKSWRCGISTSKMYSRLECQDDTDECQVYILDAEGNPINVTVDTVLHRDGVSMILKQKSTFTTRQIKAACLLIKDDKNNPESVTREHCLIDNDIYDLSKNTWNCKFNRCIKRKVEHRVKKRPPTWRHNVRAKYTEGDTATKGDLMHIQEELMYENDLLKMNIELMHAHINKLNNMLHDLIVSVAKVDERLIGNLMNNSVSSTFLSDDTFLLMPCTNPPAHTSNCYNNSIYKEGRWVANTDSSQCIDFSNYKELAIDDDVEFWIPTIGNTTYHDSWKDASGWSFIAQQKSNLITTMENTKFGGVGTSLSDITSMAEGELAAKLTSFMFGHVVNFVIILIVILDYKDDDDK

>3LZQA

GGEVPIGDPKELNGMEIAAVYLQPIEMEPRGIDLAASLADIHLEADIHALKNNPNGFPEGFWMPYLTIAYELKNTDTGAIKRGTLMPMVADDGPHYGANIAMEKDKKGGFGVGNYELTFYISNPEKQGFGRHVDEETGVGKWFEPFKVDYKFKYTGTPK

>2GKPA

SNAMTFNQEQDYWAGYKANERALIIQTWSGFGRYAPDHLYPPHILPLDTDNETLGTTVLQALANSRTFVYDSPEDQDFFDTEKIRQRYEDWVAKLCGNLGYKTRRALFKNMMSVDIWLHNGCLKISPSRHVKLEAWDAIDADDVILSLDNSPEEIGAGLKLALSRCR

>3FO8D

AVDRDTAKNSSPIAGNIEYTISTPGSNYAVGDKITVKYVSDDIETEGKITEVDADGKIKKINIPTAKIIAKAKEVGEYPTLGSNWTAEISSSSSGLAAVITLGKIITDSGILLAEIENAEAAMTAVDFQANLKKYGIPGVVALYPGELGDKIEIEIVSKADYAKGASALLPIYPGGGTRASTAKAVFGYGPQTDSQYAIIVRRNDAIVQSVVLSTKRGGKDIYDSNIYIDDFFAKGGSEYIFATAQNWPEGFSGILTLSGGLSSNAEVTAGDLMEAWDFFADR

>3CL6A

MSVDYPRDLIGYGSNPPHPHWPGKARIALSFVLNYEEGGERNILHGDKESEAFLSEMVSAQPLQGERNMSMESLYEYGSRAGVWRILKLFKAFDIPLTIFAVAMAAQRHPDVIRAMVAAGHEICSHGYRWIDYQYMDEAQEREHMLEAIRILTELTGERPLGWYTGRTGPNTRRLVMEEGGFLYDCDTYDDDLPYWEPNNPTGKPHLVIPYTLDTNDMRFTQVQGFNKGDDFFEYLKDAFDVLYAEGAEAPKMLSIGLHCRLIGRPARLAALQRFIEYAKSHEQVWFTRRVDIARHWHATHPYTGAAK

>1Q5YA

GTQGFAVLSYVYEHEKRDLASRIVSTQHHHHDLSVATLHVHINHDDCLEIAVLKGDMGDVQHFADDVIAQRGVRHGHLQCLPKED

>2XWSA

GMRRGLVIVGHGSQLNHYREVMELHRKRIEESGAFDEVKIAFAARKRRPMPDEAIREMNCDIIYVVPLFISYGLHVTEDLPDLLGFPRGRGIKEGEFEGKKVVICEPIGEDYFVTYAILNSVFRIGRDGKGEE

>2VBKA

DPDQFGPDLIEQLAQSGKYSQDNTKGDAMIGVKQPLPKAVLRTQHDKNKEAISILDFGVIDDGVTDNYQAIQNAIDAVASLPSGGELFIPASNQAVGYIVGSTLLIPGGVNIRGVGKASQLRAKSGLTGSVLRLSYDSDTIGRYLRNIRVTGNNTCNGIDTNITAEDSVIRQVYGWVFDNVMVNEVETAYLMQGLWHSKFIACQAGTCRVGLHFLGQCVSVSVSSCHFSRGNYSADESFGIRIQPQTYAWSSEAVRSEAIILDSETMCIGFKNAVYVHDCLDLHMEQLDLDYCGSTGVVIENVNGGFSFSNSWIAADADGTEQFTGIYFRTPTSTQSHKIVSGVHINTANKNTAANNQSIAIEQSAIFVFVSGCTLTGDEWAVNIVDINECVSFDKCIFNKPLRYLRSGGVSVTDCYLAGITEVQKPEGRYNTYRGCSGVPSVNGIINVPVAVGATSGSAAIPNPGNLTYRVRSLFGDPASSGDKVSVSGVTINVTRPSPVGVALPSMVEYLAI

>2X3MA

GMSAFDEFNEGFGLDVSDTPEELAFETESAIEEIESETSPGDQPKGSEPEEIRVWAEEKARKAVEEGREVTNWADWIMGWRTPNASEKKMEFMYWYTRTYLEEAKDIRPDIADALARGMAGLAFGRTDWVASMLDPQIMRHIYTDPEVARIYSETRDMLRRVSDYYISLTTMELGKVADIIAEAKAKGENPEVVAREIAEAVPRLSPKSLYFNLYYIGRSIGDNYVLEVARVLSKMRRR

>3H8DE

GSSSGGGSSSSGTSSAFSSYFNNKVGIPQEHVDHDDFDANQLLNKINE

>2P8IA

GMTFRDTSAIASWHAHVYFDASSRDAAWTLREQIEAHWSGKLQLGRFHERPVGPHPMWSYQLAFTQEQFADLVGWLTLNHGALDIFLHPNTGDALRDHRDAAVWIGHSHELVLSALN

>2IMZA

CLAEGTRIFDPVTGTTHRIEDVVDGRKPIHVVAAAKDGTLHARPVVSWFDQGTRDVIGLRIAGGAILWATPDHKVLTEYGWRAAGELRKGDRVAQPRRFDGFGDSAPIPARVQALADALDDKFLHDMLAEELRYSVIREVLPTRRARTFDLEVEELHTLVAEGVVVHN

>2IG8A

MTAVRRIRAAALPDLPDASWSNALLVGEELVMSGMTAHPATRQAAERGAALDAHAQALVVLGKVKALLEAAGGHVGNLYKLNVYVTRIADKDAIGRARQEFFAGQGTFPASTLVEVSGLVFPELLVEIDAWARLDIDLANCDEA

>3K5JA

GMDYNQTVLSHLQKFWKHHDIKGFTWTLGRIVEELPDFQVFQVIPNHEDEPWVYVSSGIGQFLGQEFFIISPFETPEHIETLAMLASASMHYPDQFQLGKTVNIGRPWVEQSSFRHFLISLPYPYGQELEYMDNVRFFWLLPITQTERLFLNTHSVEELETKFDEAGIDYLDINRASTVWQAG

>2DY0A

GSSGSSGMTATAQQLEYLKNSIKSIQDYPKPGILFRDVTSLLEDPKAYALSIDLLVERYKNAGITKVVGTEARGFLFGAPVALGLGVGFVPVRKPGKLPRETISETYDLEYGTDQLEIHVDAIKPGDKVLVVDDLLATGGTIEATVKLIRRLGGEVADAAFIINLFDLGGEQRLEKQGITSYSLVPFPGH

>3SNYA

MPTKAVTFYEDINYGGASVSLQPGNYTLSQLNTAKIPNDWMTSLKVPSGWTVDVYENDNFTGTKWTYTSDTPWVGNDANDKMRSVKIYSTTNTGGDT

>3MAHA

SLDTEKDCIKAVAAKDGITVIKVKSSNKLLSWHFMRKLFEIFEFYQEPVDMVATSEVGVSLTIDNDKNLPDIVRALSDIGDVTVDKDMVIICIVGDMEWDNVGFEARIINALKGVPVRMISYGGSNYNVSVLVKAEDKKKALIALSNKLFNSRATKA

>4AQOA

GGTISNNKAPIAKVTGPSTGAVGRNIEFSGKDSKDEDGKIVSYDWDFGDGATSRGKNSVHAYKKAGTYNVTLKVTDDKGATATESFTIEIKN

>2ZBLA

MKWFNTLSHNRWLEQETDRIFNFGKNAVVPTGFGWLGNKGQIKEEMGTHLWITARMLHVYSVAASMGRPGAYDLVDHGIKAMNGALRDKKYGGWYACVNDQGVVDASKQGYQHFFALLGAASAVTTGHPEARKLLDYTIEVIEKYFWSEEEQMCLESWDEAFSQTEDYRGGNANMHAVEAFLIVYDVTHDKKWLDRALRIASVIIHDVARNGDYRVNEHFDSQWNPIRDYNKDNPAHRFRAYGGTPGAWIEWGRLMLHLHAALEARFETPPAWLLEDAKGLFHATIRDAWAPDGADGFVYSVDWDGKPIVRERVRWPIVEAMGTAYALYTLTDDSQYEEWYQKWWDYCIKYLMDYENGSWWQELDADNKVTTKVWDGKQDIYHLLHCLVIPRLPLAPGLAPAVAAGLLDINAKLEHHHHHH

>2CDUA

MKVIVVGCTHAGTFAVKQTIADHPDADVTAYEMNDNISFLSCGIALYLGKEIKNNDPRGLFYSSPEELSNLGANVQMRHQVTNVDPETKTIKVKDLITNEEKTEAYDKLIMTTGSKPTVPPIPGIDSSRVYLCKNYNDAKKLFEEAPKAKTITIIGSGYIGAELAEAYSNQNYNVNLIDGHERVLYKYFDKEFTDILAKDYEAHGVNLVLGSKVAAFEEVDDEIITKTLDGKEIKSDIAILCIGFRPNTELLKGKVAMLDNGAIITDEYMHSSNRDIFAAGDSAAVHYNPTNSNAYIPLATNAVRQGRLVGLNLTEDKVKDMGTQSSSGLKLYGRTYVSTGINTALAKANNLKVSEVIIADNYRPEFMLSTDEVLMSLVYDPKTRVILGGALSSMHDVSQSANVLSVCIQNKNTIDDLAMVDMLFQPQFDRPFNYLNILGQAAQAQADKAHK

>1XE7A

MSANVQEAANAAIEPASFVKVPMPEPPSSLQQLINDWQLIKHREGGYFKETDRSPYTMEVEKPVNGGSGNTEMVTRNQSTLIYYLLTPDSPIGKFHKNINRIIHILQRGKGQYVLVYPDGQVKSFKVGFDYKNGEVSQWVVPGGVFKASFLLPNEEFDNGFLISEVVVPGFDFEDHTFLKGEDELKHLVGPEKAAELAFLAHH

>2EFJA

MELQEVLHMNGGEGDTSYAKNSSYNLFLIRVKPVLEQCIQELLRANLPNINKCFKVGDLGCASGPNTFSTVRDIVQSIDKVGQEKKNELERPTIQIFLNDLFQNDFNSVFKLLPSFYRNLEKENGRKIGSCLIGAMPGSFYSRLFPEESMHFLHSCYCLHWLSQVPSGLVTELGISVNKGCIYSSKASRPPIQKAYLDQFTKDFTTFLRIHSEELISRGRMLLTFICKEDEFDHPNSMDLLEMSINDLVIEGHLEEEKLDSFNVPIYAPSTEEVKRIVEEEGSFEILYLETFNAPYDAGFSIDDDYQGRSHSPVSCDEHARAAHVASVVRSIYEPILASHFGEAILPDLSHRIAKNAAKVLRSGKGFYDSVIISLAKKPEKADM

>3GWRA

GMSEPVFPTPEAAEDAFYAAFEARSLDDMMAVWARDDHVACIHPLAAPLNGRAAVAAGWRSMFGAAGRFRLQVKAVHEIRQADHVIRIVDEFLTIGDETAPRPAILATNVYRREADGWRMVLHHASPLQVGAKAGADTPPVVFH

>1K04A

LSSPADSYNEGVKLQPQEISPPPTANLDRSNDKVYENVTGLVKAVIEMSSKIQPAPPEEYVPMVKEVGLALRTLLATVDETIPLLPASTHREIEMAQKLLNSDLGELINKMKLAQQYVMTSLQQEYKKQMLTAAHALAVDAKNLLDVIDQARLKMLGQTRPH

>3E4WA

MHHHHHHMSGGLTPDQAIDAIRGTGGAQPGCRALHAKGTLYRGTFTATRDAVMLSAAPHLDGSTVPALIRFSNGSGNPKQRDGAPGVRGMAVKFTLPDGSTTDVSAQTARLLVSSTPEGFIDLLKAMRPGLTTPLRLATHLLTHPRLLGALPLLREANRIPASYATTEYHGLHAFRWIAADGSARFVRYHLVPTAAEEYLSASDARGKDPDFLTDELAARLQDGPVRFDFRVQIAGPTDSTVDPSSAWQSTQIVTVGTVTITGPDTEREHGGDIVVFDPMRVTDGIEPSDDPVLRFRTLVYSASVKLRTGVDRGAQAPPV

>3FCNA

GMGMEHKTYEADLFVWCQQQADGLRALSRSRRDLPDDLDLEHIAEEIEDMGRSELREATSLVRQICVRVIMAMSAPEAPDRARWRSEVVSWHNLLLDTITPGMIDRIDIGVIWRRAVSEAKAALIEINVAPQAGLSFQAPLPADHFLDEDFDYDATVARLGPTA

>2SICI

YAPSALVLTVGKGVSATTAAPERAVTLTCAPGPSGTHPAAGSACADLAAVGGDLNALTRGEDVMCPMVYDPVLLTVDGVWQGKRVSYERVFSNECEMNAHGSSVFAF

>3LFTA

SNAKIGVLQFVSHPSLDLIYKGIQDGLAEEGYKDDQVKIDFMNSEGDQSKVATMSKQLVANGNDLVVGIATPAAQGLASATKDLPVIMAAITDPIGANLVKDLKKPGGNVTGVSDHNPAQQQVELIKALTPNVKTIGALYSSSEDNSKTQVEEFKAYAEKAGLTVETFAVPSTNEIASTVTVMTSKVDAIWVPIDNTIASGFPTVVSSNQSSKKPIYPSATAMVEVGGLASVVIDQHDLGVATGKMIVQVLKGAKPADTPVNVFSTGKSVINKKIAQELGITIPESVLKEAGQVI

>3N17A

ANNLGSKLLVGYWHNFDNGTGIIKLKDVSPKWDVINVSFGETGGDRSTVEFSPVYGTDADFKSDISYLKSKGKKVVLSIGGQNGVVLLPDNAAKDRFINSIQSLIDKYGFDGIDIDLQSGIYLNGNDTNFKNPTTPQIVNLISAIRTISDHYGPDFLLSMAPETAYVQGGYSAYGSIWGAYLPIIYGVKDKLTYIHVQHFNAGSGIGMDGNNYNQGTADYEVAMADMLLHGFPVGGNANNIFPALRSDQVMIGLPAAPAAAPSGGYISPTEMKKALNYIIKGVPFGGKYKLSNQSGYPAFRGLMSWSINWDAKNNFEFSNNYRTYFDGLSLQK

>4IQNA

SNAMDISLTNLIELVKKVNRNKVPTPMSAEEISRLRVRKYRDPQNTETTELPESLKALLAYDRDLLSNYNMPVIETLQKSIDNEGVIHSYSPDEEAYYGVGMDSSGIDIEDLMPVWSNDPRLPALIRIDHVGDQAIFIYITERDANGEYPIARMERNEFWLAESSLVEYLYNIISGAKDIGFTEEDLHLPQWKAQQKMNEQRDAALLDLEDYHEAFWAKLDALVD

>3CVOA

GMDDQSGDQMRPELTMPPAEAEALRMAYEEAEVILEYGSGGSTVVAAELPGKHVTSVESDRAWARMMKAWLAANPPAEGTEVNIVWTDIGPTGDWGHPVSDAKWRSYPDYPLAVWRTEGFRHPDVVLVDGRFRVGCALATAFSITRPVTLLFDDYSQRRWQHQVEEFLGAPLMIGRLAAFQVEPQPIPPGSLMQLIRTMTSP

>4KEFA

GSRSGVAVADESLTAFNDLKLGKKYKFILFGLNDAKTEIVVKETSTDPSYDAFLEKLPENDCLYAIYDFEYEINGNEGKRSDIVFFTWSPDTAPVRSKMVYASSKDALRRALNGVSTDVQGTDFSEVSYDSVLERVSRGAGSH

>2Q4XA

MEKRGVIDTWIDKHRSIYTAATRHAFVVSIRDGSVDLSSFRTWLGQDYLFVRRFVPFVASVLIRACKDSGESSDMEVVLGGIASLNDEIEWFKREGSKWDVDFSTVVPQRANQEYGRFLEDLMSSEVKYPVIMTAFWAIEAVYQESFAHCLEDGNKTPVELTGACHRWGNDGFKQYCSSVKNIAERCLENASGEVLGEAEDVLVRVLELEVAFWEMSRGGQ

>1MUWA

SYQPTPEDRFTFGLWTVGWQGRDPFGDATRPALDPVETVQRLAELGAHGVTFHDDDLIPFGSSDTERESHIKRFRQALDATGMTVPMATTNLFTHPVFKDGGFTANDRDVRRYALRKTIRNIDLAVELGAKTYVAWGGREGAESGAAKDVRVALDRMKEAFDLLGEYVTSQGYDIRFAIEPKPNEPRGDILLPTVGHALAFIERLERPELYGVNPEVGHEQMAGLNFPHGIAQALWAGKLFHIDLNGQSGIKYDQDLRFGAGDLRAAFWLVDLLESAGYEGPRHFDFKPPRTEDIDGVWASAAGCMRNYLILKERAAAFRADPEVQEALRASRLDELAQPTAADGVQELLADRTAFEDFDVDAAAARGMAFERLDQLAMDHLLGAR

>3GWBA

ELDGKAPSHRNLNVQTWSTAEGAKVLFVEARELPMFDLRLIFAAGSSQDGNAPGVALLTNAMLNEGVAGKDVGAIAQGFEGLGADFGNGAYKDMAVASLRSLSAVDKREPALKLFAEVVGKPTFPADSLARIKNQMLAGFEYQKQNPGKLASLELMKRLYGTHPYAHASDGDAKSIPPITLAQLKAFHAKAYAAGNVVIALVGDLSRSDAEAIAAQVSAALPKGPALAKIEQPAEPKASIGHIEFPSSQTSLMLAQLGIDRDDPDYAAVSLGNQILGGGGFGTRLMSEVREKRGLTYGVYSGFTPMQARGPFMINLQTRAEMSEGTLKLVQDVFAEYLKNGPTQKELDDAKRELAGSFPLSTASNADIVGQLGAMGFYNLPLSYLEDFMRQSQELTVEQVKAAMNKHLNVDKMVIVSAGPTVAQKPLEHHHHHH

>3KS6A

GMTRIASHRGGTLEFGDSTPHGFTATAAMALEEVEFDLHPTADGAIVVHHDPTLDATTDMTGAIVDMTLAKVKTATIRYGAGSHPMTLEELCALYVDSHVNFRCEIKPGVDGLPYEGFVALVIAGLERHSMLERTTFSSFLLASMDELWKATTRPRLWLVSPSVLQQLGPGAVIETAIAHSIHEIGVHIDTADAGLMAQVQAAGLDFGCWAAHTPSQITKALDLGVKVFTTDRPTLAIALRTEHRMEASV

>3DXPA

GMSSNVSHFEGTRPVADQQRFDTEALEAWMRQHVEGFAGPLSVEQFKGGQSNPTFKLVTPGQTYVMRAKPGPKSKLLPSAHAIEREYRVMDALAGTDVPVAKMYALCEDESVIGRAFYIMEFVSGRVLWDQSLPGMSPAERTAIYDEMNRVIAAMHTVDYQAIGLGDYGKPGNYFQRQIERWTKQYKLSETESIPAMDSLMDWLPQHIPQEDADLTSIVHGDYRLDNLMFHPTEPRVLAVLDWELSTLGHPMGDFGYHCMSWHIAPGQFRGIAGLDHAALGIPDEASYRKLYEQRTGRPITGDWNFYLAFSMFRIAGILQGIMKRVVDGTASSAQALDAGKRARPMAEMGWEYAKKAKQ

>1XQAA

AMGIKHLNLTVADVVAAREFLEKYFGLTCSGTRGNAFAVMRDNDGFILTLMKGKEVQYPKTFHVGFPQESEEQVDKINQRLKEDGFLVEPPKHAHAYTFYVEAPGGFTIEVMC

>2PFZA

TKWDLPTAYPASNLHVENLTQFVKDVDSLSGGKLKITLHNNASLYKAPEIKRAVQGNQAQIGEILLTNFANEDPVYELDGLPFLATGYDASFKLYQAQKPFLEKKLASQGMMLLYSVAWPPQGIFANRDIKQVSDMKGLKWRAYSPVTAKIAELVGAQPVTVQQAELAQAMATGVIDSYMSSGSTGFDTKTYEYIKKFYDTEAWLPKNAVLVNKKAFDALDPATQQALKKAGAQAEERGWKLSQEKNSWYKEQLAKNGMAIIAPTAELKSGLTEVGKRMLDDWLKKAGADGQAMIDAYRKQ

>3H8DE

GSSSGGGSSSSGTSSAFSSYFNNKVGIPQEHVDHDDFDANQLLNKINE

>4A5ZA

MAHHHHHHMDQIKIEENATGFSYESLFREYLNETVTEVWIEDPYIRHTHQLYNFLRFCEMLIKRPCKVKTIHLLTSLDEGIEQVQQSRGLQEIEESLRSHGVLLEVQYSSSIHDREIRFNNGWMIKIGRGLDYFKKPQSRFSLGYCDFDLRPCHETTVDIFHD

>2V8TA

MFLRIDRLQIELPMPKEQDPNAAAAVQALLGGRFGEMSTLMNYMYQSFNFRGKKALKPYYDLIANIATEELGHIELVAATINSLLAKNPGKDLEEGVDPASTPLGFAKDVRNAAHFIAGGANSLVMGAMGEHWNGEYVFTSGNLILDLLHNFFLEVAARTHKLRVYEMTDNPVAREMIGYLLVRGGVHAAAYGKALESLTGVEMTKMLPIPKIDNSKIPEAKKYMDLGFHRNLYRFSPEDYRDLGLIWKGASPEDGTEVVVVDGPPTGGPVFDAGHDAAEFAPEFHPGELYEIAKKLYEKAK

>2VHLA

MAESLLIKDIAIVTENEVIKNGYVGINDGKISTVSTERPKEPYSKEIQAPADSVLLPGMIDIHIHGGYGADTMDASFSTLDIMSSRLPEEGTTSFLATTITQEHGNISQALVNAREWKAAEESSLLGAELLGIHLEGPFVSPKRAGAQPKEWIRPSDVELFKKWQQEAGGLIKIVTLAPEEDQHFELIRHLKDESIIASMGHTDADSALLSDAAKAGASHMTHLYNAMSPFHHREPGVIGTALAHDGFVTELIADGIHSHPLAAKLAFLAKGSSKLILITDSMRAKGLKDGVYEFGGQSVTVRGRTALLSDGTLAGSILKMNEGARHMREFTNCSWTDIANITSENAAKQLGIFDRKGSVTVGKDADLVIVSSDCEVILTICRGNIAFISKEADQI

>3C8LA

GMARKRLIIEMGMGIDQHGQEPTIAASRAVRNAIAHNALPGVWEVAGLSHPNEMIIEVQVAVPYPEQVREEEVLAVLPFGRKTLTVESGGMIVQGRAIPELNDKNDEMLIAIAAVTVLIENE

>4F11A

WARGAPRPPPSSPPLSIMGLMPLTKEVAKGSIGRGVLPAVELAIEQIRNESLLRPYFLDLRLYDTECDNAKGLKAFYDAIKYGPNHLMVFGGVCPSVTSIIAESLQGWNLVQLSFAATTPVLADKKKYPYFFRTVPSDNAVNPAILKLLKHYQWKRVGTLTQDVQRFSEVRNDLTGVLYGEDIEISDTESFSNDPCTSVKKLKGNDVRIILGQFDQNMAAKVFCCAYEENMYGSKYQWIIPGWYEPSWWEQVHTEANSSRCLRKNLLAAMEGYIGVDFEPLSSKQIKTISGKTPQQYEREYNNKRSGVGPSKFHGYAYDGIWVIAKTLQRAMETLHASSRHQRIQDFNYTDHTLGRIILNAMNETNFFGVTGQVVFRNGERMGTIKFTQFQDSREVKVGEYNAVADTLEIINDTIRFQGSEPPKDDYKDDDDK

>4G68A

MAHHHHHHVDDDDKMCSSNNLSKSNTSNSSKTSSSSKKMCSSNNLSKSNTSNSSKTSSSSKKITLTFWNLFTGEPAKTKVKEIIDQWNKENPNVQIVESVTENDAYKTKIKAAIAANEAPDIFQTWAGGFSQPFVEAGKVLQLDSYLNDGTKDQLLPGSFDNVTYNGKIYGIPFDQQASVLYINKELFDKYNVKVPTTFSELIDAIKTFKSKGVTPFALGEKDEWPGMWYYDMIALREGGVQLTRDALNGKASFDNQAFTDAAQKLQDMVNAGAFDSGFMGLTRDEATAEFNQGKAAMYFGGNFDAAAFVSDPSSLVKGKIEAVRFPTIEGGKGDPTEYIGGTVGALMVSANSKYKDEAVRAAKYLAKQLSDMDYLIATGLPAWKYDNIDQSKVDPLEIQIMNNIVANAKGSVPAWDIYLSGDAAQTHKDLVAQLFAKQITPEEYSKQMQQKINGK

>2EB4A

MFDKHTHTLIAQRLDQAEKQREQIRAISLDYPEITIEDAYAVQREWVRLKIAEGRTLKGHKIGLTSKAMQASSQISEPDYGALLDDMFFHDGSDIPTDRFIVPRIEVELAFVLAKPLRGPNCTLFDVYNATDYVIPALELIDARCHNIDPETQRPRKVFDTISDNAANAGVILGGRPIKPDELDLRWISALMYRNGVIEETGVAAGVLNHPANGVAWLANKLAPYDVQLEAGQIILGGSFTRPVPARKGDTFHVDYGNMGSISCRFV

>4A0DA

GSSPDKKWLGTPIEEMRRMPRCGIRLPLLRPSANHTVTIRVDLLRAGEVPKPFPTHYKDLWDNKHVKMPCSEQNLYPVEDENGERTAGSRWELIQTALLNKFTRPQNLKDAILKYNVAYSKKWDFTALIDFWDKVLEEAEAQHLYQSILPDMVKIALCLPNICTQPIPLLAAAMNHSITMSQEQIASLLANAFFCTFPRRNAKMKSEYSSYPDINFNRLFEGRSSRKPEKLKTLFCYFRRVTAAAPTGLVTFTRQSLEDFPEWERCEKPLTRLHVTYEGTIEENGQGMLQVDFANRFVGGGVTSAGLVQEEIRFLINPELIISRLFTEVLDHNECLIITGTEQYSEYTGYAETYRWSRSHEDGSERDDWQRRCTEIVAIDALHFRRYLDQFVPEKMRRELNKAYCGFLRPGVSSENLSAVATGNWGCGAFGGDARLKALIQILAAAAAERDVVYFTFGDSELMRDIYSMHIFLTERKLTVGDVYKLLLRYYNEECRNCSTPGPDIKLYPFIYHAVESCAETADHSGQRTGT

>2R19A

AVTGDTDQPIHIESDQQSLDMQGNVVTFTGNVIVTQGTIKINADKVVVTRPGGEQGKEVIDGYGKPATFYQMQDNGKPVEGHASQMHYELAKDFVVLTGNAYLQQVDSNIKGDKITYLVKEQKMQAFSDKGKRVTTVLVPSQLQDKNNKGQTPAQKKGN

>2O3OA

GSHMATGKEYEVIKNDVEHDMKADHITYEGLNKEATEGYRITANQKSFSKEEIEALKDQKPLMDMPSDDHKVTSLKMKFANPIALSKKDIEDDAQALVSSKIQDGEKYKLWKVDKSKKEIIFFQTYEGHYIYQKTDNPSNMIGQVVLHLNGKNEVVSYDQTTLETFKQIQKESLITEMDAVELLYYQNQLKEYSTVKSCKFGYVAQYPLTSTQVLAPVWRITVEYEKKVNGEKKTVQEYFTVNALESTILDTDQ

>2G82A

MKVGINGFGRIGRQVFRILHSRGVEVALINDLTDNKTLAHLLKYDSIYHRFPGEVAYDDQYLYVDGKAIRATAVKDPKEIPWAEAGVGVVIESTGVFTDADKAKAHLEGGAKKVIITAPAKGEDITIVMGVNHEAYDPSRHHIISNASCTTNSLAPVMKVLEEAFGVEKALMTTVHSYTNDQRLLDLPHKDLRRARAAAINIIPTTTGAAKATALVLPSLKGRFDGMALRVPTATGSISDITALLKREVTAEEVNAALKAAAEGPLKGILAYTEDEIVLQDIVMDPHSSIVDAKLTKALGNMVKVFAWYDNEWGYANRVADLVELVLRKGV

>1GG4A

MISVTLSQLTDILNGELQGADITLDAVTTDTRKLTPGCLFVALKGERFDAHDFADQAKAGGAGALLVSRPLDIDLPQLIVKDTRLAFGELAAWVRQQVPARVVALTGSSGKTSVKEMTAAILSQCGNTLYTAGNLNNDIGVPMTLLRLTPEYDYAVIELGANHQGEIAWTVSLTRPEAALVNNLAAAHLEGFGSLAGVAKAKGEIFSGLPENGIAIMNADNNDWLNWQSVIGSRKVWRFSPNAANSDFTATNIHVTSHGTEFTLQTPTGSVDVLLPLPGRHNIANALAAAALSMSVGATLDAIKAGLANLKAVPGRLFPIQLAENQLLLDDSYNANVGSMTAAVQVLAEMPGYRVLVVGDMAELGAESEACHVQVGEAAKAAGIDRVLSVGKQSHAISTASGVGEHFADKTALITRLKLLIAEQQVITILVKGSRSAAMEEVVRALQENGTC

>4GYXA

GPPGPPGPRGQPGVMGFPGPPGPPGPCCGGV

>1OAOC

MTDFDKIFEGAIPEGKEPVALFREVYHGAITATSYAEILLNQAIRTYGPDHPVGYPDTAYYLPVIRCFSGEEVKKLGDLPPILNRKRAQVSPVLNFENARLAGEATWYAAEIIEALRYLKYKPDEPLLPPPWTGFIGDPVVRRFGIKMVDWTIPGEAIILGRAKDSKALAKIVKELMGMGFMLFICDEAVEQLLEENVKLGIDYIAYPLGNFTQIVHAANYALRAGMMFGGVTPGAREEQRDYQRRRIRAFVLYLGEHDMVKTAAAFGAIFTGFPVITDQPLPEDKQIPDWFFSVEDYDKIVQIAMETRGIKLTKIKLDLPINFGPAFEGESIRKGDMYVEMGGNRTPAFELVRTVSESEITDGKIEVIGPDIDQIPEGSKLPLGILVDIYGRKMQADFEGVLERRIHDFINYGEGLWHTGQRNINWLRVSKDAVAKGFRFKNYGEILVAKMKEEFPAIVDRVQVTIFTDEAKVKEYMEVAREKYKERDDRMRGLTDETVDTFYSCVLCQSFAPNHVCIVTPERVGLCGAVSWLDAKASYEINHAGPNQPIPKEGEIDPIKGIWKSVNDYLYTASNRNLEQVCLYTLMENPMTSCGCFEAIMAILPECNGIMITTRDHAGMTPSGMTFSTLAGMIGGGTQTPGFMGIGRTYIVSKKFISADGGIARIVWMPKSLKDFLHDEFVRRSVEEGLGEDFIDKIADETIGTTVDEILPYLEEKGHPALTMDPIM

>4A5SA

SRKTYTLTDYLKNTYRLKLYSLRWISDHEYLYKQENNILVFNAEYGNSSVFLENSTFDEFGHSINDYSISPDGQFILLEYNYVKQWRHSYTASYDIYDLNKRQLITEERIPNNTQWVTWSPVGHKLAYVWNNDIYVKIEPNLPSYRITWTGKEDIIYNGITDWVYEEEVFSAYSALWWSPNGTFLAYAQFNDTEVPLIEYSFYSDESLQYPKTVRVPYPKAGAVNPTVKFFVVNTDSLSSVTNATSIQITAPASMLIGDHYLCDVTWATQERISLQWLRRIQNYSVMDICDYDESSGRWNCLVARQHIEMSTTGWVGRFRPSEPHFTLDGNSFYKIISNEEGYRHICYFQIDKKDCTFITKGTWEVIGIEALTSDYLYYISNEYKGMPGGRNLYKIQLIDYTKVTCLSCELNPERCQYYSVSFSKEAKYYQLRCSGPGLPLYTLHSSVNDKGLRVLEDNSALDKMLQNVQMPSKKLDFIILNETKFWYQMILPPHFDKSKKYPLLLDVYAGPCSQKADTVFRLNWATYLASTENIIVASFDGRGSGYQGDKIMHAINRRLGTFEVEDQIEAARQFSKMGFVDNKRIAIWGWSYGGYVTSMVLGSGSGVFKCGIAVAPVSRWEYYDSVYTERYMGLPTPEDNLDHYRNSTVMSRAENFKQVEYLLIHGTADDNVHFQQSAQISKALVDVGVDFQAMWYTDEDHGIASSTAHQHIYTHMSHFIKQCFSLPAAASWSHPQFEK

>3T3LA

GTLGHPGSLDETTYERLAEETLDSLAEFFEDLADKPYTFEDYDVSFGSGVLTVKLGGDLGTYVINKQTPNKAIWLSSPSSGPKRYDWTGKNWVYSHDGVSLHELLAAELTKALKTKLDLSSLAYSGKDA

>1VF7A

AESSGKSEAPPPAQTPEVGIVTLEAQTVTLNTELPGRTNAFRIAEVRPQVNGIILKRLFKEGSDVKAGQQLYQIDPATYEADYQSAQANLASTQEQAQRYKLLVADQAVSKQQYADANAAYLQSKAAVEQARINLRYTKVLSPISGRIGRSAVTEGALVTNGQANAMATVQQLDPIYVDVTQPSTALLRLRRELASGQLERAGDNAAKVSLKLEDGSQYPLEGRLEFSEVSVDEGTGSVTIRAVFPNPNNELLPGMFVHAQLQEGVKQKAILAPQQGVTRDLKGQATALVVNAQNKVELRVIKADRVIGDKWLVTEGLNAGDKIITEGLQFVQPGVEVKTVPAKNVASAQKADAAPAKTDSKGHHHHHH

>1JMSA

NSSPSPVPGSQNVPAPAVKKISQYACQRRTTLNNYNQLFTDALDILAENDELRENEGSCLAFMRASSVLKSLPFPITSMKDTEGIPCLGDKVKSIIEGIIEDGESSEAKAVLNDERYKSFKLFTSVFGVGLKTAEKWFRMGFRTLSKIQSDKSLRFTQMQKAGFLYYEDLVSCVNRPEAEAVSMLVKEAVVTFLPDALVTMTGGFRRGKMTGHDVDFLITSPEATEDEEQQLLHKVTDFWKQQGLLLYCDILESTFEKFKQPSRKVDALDHFQKCFLILKLDHGRVHSEKSGQQEGKGWKAIRVDLVMCPYDRRAFALLGWTGSRQFERDLRRYATHERKMMLDNHALYDRTKRVFLEAESEEEIFAHLGLDYIEPWERNA

>3KLQA

GAKDSTVQTSISVENVLERAGDSTPFSVALESIDAMKTIEEITIAGSGKASFSPLTFTTVGQYTYRVYQKPSQNKDYQADTTVFDVLVYVTYDEDGTLVAKVISRRAGDEEKSAITFKPKRLVKPIPPRQPDFPKTPLPLA

>2APLA

MKSTEKKELSHFRLKLETYLNEHFPEMSGNNPFITARSDEALTAYCDAVAQGFSHPEAESMASEVLYQGLHFSRYDTLVSVLEREFEQELPSPLPERLAPILLKNKAIQSVFAKYDLTDDFEASPEYEHLYTELTGTIVLLIESNHLPTIGGGNDTV

>4AMWA

GSTDNPDGIDYKTYDYVGVWGFSPLSNTNWFAAGSSTPGGITDWTATMNVNFDRIDNPSITVQHPVQVQVTSYNNNSYRVRFNPDGPIRDVTRGPILKQQLDWIRTQELSEGCDPGMTFTSEGFLTFETKDLSVIIYGNFKTRVTRKSDGKVIMENDEVGTASSGNKCRGLMFVDRLYGNAIASVNKNFRNDAVKQEGFYGAGEVNCKYQDTYILERTGIAMTNYNYDNLNYNQWDLRPPHHDGALNPDYYIPMYYAAPWLIVNGCAGTSEQYSYGWFMDNVSQSYMNTGDTTWNSGQEDLAYMGAQYGPFDQHFVYGAGGGMECVVTAFSLLQGKEFENQVLNKRSVMPPKYVFGFFQGVFGTSSLLRAHMPAGENNISVEEIVEGYQNNNFPFEGLAVDVDMQDNLRVFTTKGEFWTANRVGTGGDPNNRSVFEWAHDKGLVCQTNITCFLRNDNEGQDYEVNQTLRERQLYTKNDSLTGTDFGMTDDGPSDAYIGHLDYGGGVECDALFPDWGRPDVAEWWGNNYKKLFSIGLDFVWQDMTVPAMMPHKIGDDINVKPDGNWPNADDPSNGQYNWKTYHPQVLVTDMRYENHGREPMVTQRNIHAYTLCESTRKEGIVENADTLTKFRRSYIISRGGYIGNQHFGGMWVGDNSTTSNYIQMMIANNINMNMSCLPLVGSDIGGFTSYDNENQRTPCTGDLMVRYVQAGCLLPWFRNHYDRWIESKDHGKDYQELYMYPNEMDTLRKFVEFRYRWQEVLYTAMYQNAAFGKPIIKAASMYNNDSNVRRAQNDHFLLGGHDGYRILCAPVVWENSTERELYLPVLTQWYKFGPDFDTKPLEGAMNGGDRIYNYPVPQSESPIFVREGAILPTRYTLNGENKSLNTYTDEDPLVFEVFPLGNNRADGMCYLDDGGVTTNAEDNGKFSVVKVAAEQDGGTETITFTNDCYEYVFGGPFYVRVRGAQSPSNIHVSSGAGSQDMKVSSATSRAALFNDGENGDFWVDQETDSLWLKLPNVVLPDAVITIT

>3ZRIA

MRGSHHHHHHTDPIRIELPTLIAKLNAQSKLALEQAASLCIERQHPEVTLEHYLDVLLDNPLSDVRLVLKQAGLEVDQVKQAIASTYSREQVLDTYPAFSPLLVELLQEAWLLSSTELEQAELRSGAIFLAALTRADRYLSFKLISLFEGINRENLKKHFAMILSDSAETT

>3BHDA

MGSSHHHHHHSSGLVPRGSMAQGLIEVERKFLPGPGTEERLQELGGTLEYRVTFRDTYYDTPELSLMQADHWLRRREDSGWELKCPGAAGVLGPHTEYKELTAEPTIVAQLCKVLRADGLGAGDVAAVLGPLGLQEVASFVTKRSAWKLVLLGADEEEPQLRVDLDTADFGYAVGEVEALVHEEAEVPTALEKIHRLSSMLGVPAQETAPAKLIVYLQRFRPQDYQRLLEVNSS

>4DQAA

GNDLLEPKVYFESKEYNFSVEDEMDVMTFDLVSRLSSATSSQVDVSYSVAEPSVVDEYNAKYGTNYEMLDVSQVKLSSTTSSISSGKLYADNIEVELSGLEALKAGNSYVLPMRVHSSSVSTLSGTNIAYFFFSKPLKITKAGNFSNHYISVKFPVGTFFSSFTYEALINVDYFLDNNTIMGTEGVMILRIGDAGGGITPKDYLEVAGGQNYRVTKPLLTNRWYHVALTYDQPTGKTGIYVNGEKWAGSDWGIDGFDPNSDMGFYIGRIYGFKWGERPFHGKMSEVRVWSVARTENQLKQNMLGVDPASEGLALYYKLDGSETQEGGVIKDATGRINGTTNGITIKTLDAPIAIN

>1TWDA

MALLEICCYSMECALTAQQNGADRVELCAAPKEGGLTPSLGVLKSVRQRVTIPVHPIIRPRGGDFCYSDGEFAAILEDVRTVRELGFPGLVTGVLDVDGNVDMPRMEKIMAAAGPLAVTFHRAFDMCANPLYTLNNLAELGIARVLTSGQKSDALQGLSKIMELIAHRDAPIIMAGAGVRAENLHHFLDAGVLEVHSSAGAWQASPMRYRNQGLSMSSDEHADEYSRYIVDGAAVAEMKGIIERHQAKLEHHHHHH

>2APLA

MKSTEKKELSHFRLKLETYLNEHFPEMSGNNPFITARSDEALTAYCDAVAQGFSHPEAESMASEVLYQGLHFSRYDTLVSVLEREFEQELPSPLPERLAPILLKNKAIQSVFAKYDLTDDFEASPEYEHLYTELTGTIVLLIESNHLPTIGGGNDTV

>1RA0A

GSSMANNALQTIINARLPGEEGLWQIHLQDGKISAIDAQSGVMPITENSLDAEQGLVIPPFVEPHIHLDTTQTAGQPNWNQSGTLFEGIERWAERKALLTHDDVKQRAWQTLKWQIANGIQHVRTHVDVSDATLTALKAMLEVKQEVAPWIDLQIVAFPQEGILSYPNGEALLEEALRLGADVVGAIPHFEFTREYGVESLHKTFALAQKYDRLIDVHCDEIDDEQSRFVETVAALAHHEGMGARVTASHTTAMHSYNGAYTSRLFRLLKMSGINFVANPLVNIHLQGRFDTYPKRRGITRVKEMLESGINVCFGHDGVFDPWYPLGTANMLQVLHMGLHVCQLMGYGQINDGLNLITHHSARTLNLQDYGIAAGNSANLIILPAENGFDALRRQVPVRYSVRGGKVIASTQPAQTTVYLEQPEAIDYKR

>2VPAA

MSYYHHHHHHLESTSLYKKAGMSDFYDPRERDPSVSRRPQNRQSDEWIRELLLRGTIARVATLWQGEDGAAFPFITPLAYAYRPEQGDLVYHTNVVGRLRANAGQGHPATLEVSEIGQFLPSNSPLELSVQYRSVMVFGTARVLAGEDARAALTTLSERVFPGLKVGETTRPISEDDLKRTSVYSLSIDRWSGKENWAEQAIQEEDWPALGPEWLG

>2HEWF

GSHMSSSPAKDPPIQRLRGAVTRCEDGQLFISSYKNEYQTMEVQNNSVVIKCDGLYIIYLKGSFFQEVKIDLHFREDHNPISIPMLNDGRRIVFTVVASLAFKDKVYLTVNAPDTLCEHLQINDGELIVVQLTPGYCAPEGSYHSTVNQVPL

>3C8NA

MGSSHHHHHHSSGLVPRGSHMAELKLGYKASAEQFAPRELVELAVAAEAHGMDSATVSDHFQPWRHQGGHAPFSLSWMTAVGERTNRLLLGTSVLTPTFRYNPAVIAQAFATMGCLYPNRVFLGVGTGEALNEIATGYEGAWPEFKERFARLRESVGLMRQLWSGDRVDFDGDYYRLKGASIYDVPDGGVPVYIAAGGPAVAKYAGRAGDGFICTSGKGEELYTEKLMPAVREGAAAADRSVDGIDKMIEIKISYDPDPELAMNNTRFWAPLSLTAEQKHSIDDPIEMEKAADALPIEQIAKRWIVASDPDEAVEKVGQYVTWGLNHLVFHAPGHDQRRFLELFQSDLAPRLRRLG

>2MPRA

VDFHGYARSGIGWTGSGGEQQCFQATGAQSKYRLGNECETYAELKLGQEVWKEGDKSFYFDTNVAYSVNQQNDWESTDPAFREANVQGKNLIEWLPGSTIWAGKRFYQRHDVHMIDFYYWDISGPGAGIENIDLGFGKLSLAATRSTEAGGSYTFSSQNIYDEVKDTANDVFDVRLAGLQTNPDGVLELGVDYGRANTTDGYKLADGASKDGWMFTAEHTQSMLKGYNKFVVQYATDAMTTQGKGQARGSDGSSSFTEELSDGTKINYANKVINNNGNMWRILDHGAISLGDKWDLMYVGMYQNIDWDNNLGTEWWTVGVRPMYKWTPIMSTLLEVGYDNVKSQQTGDRNNQYKITLAQQWQAGDSIWSRPAIRIFATYAKWDEKWGYIKDGDNISRYAAATNSGISTNSRGDSDEWTFGAQMEIWW

>3K3CA

GAMAAEMDWDKTVGAAEDVRRIFEHIPAILVGLEGPDHRFVAVNAAYRGFSPLLDTVGQPAREVYPELEGQQIYEMLDRVYQTGEPQSGSEWRLQTDYDGSGVEERYFDFVVTPRRRADGSIEGVQLIVDDVTSRVRARQAAEARVEELSERYRNVRD

>3LKMA

MGGHHHHHHGENLYFQGISSETGEMGILWEFDPIINKWIRLSMKLKVERKPFAEGALREAYHTVSLGVGTDENYPLGTTTKLFPPIEMISPISKNNEAMTQLKNGTKFVLKLYKKEAEQQASRELYFEDVKMQMVCRDWGNKFNQKKPPKKIEFLMSWVVELIDRSPSSNGQPILCSIEPLLVGEFKKNNSNYGAVLTNRSTPQAFSHFTYELSNKQMIVVDIQGVDDLYTDPQIHTPDGKGFGLGNLGKAGINKFITTHKCNAVCALLDLDVKLGGVLSGNNKKQLQQGTMVMPDILPELMPSDNT

>2XDGA

SMLREDESACLQAAEEMPQTTLGCPATWDGLLCWPTAGSGEWVTLPCPDFFSHFSSESGAVKRDCTITGWSEPFPPYPVACPVPLELLAEEE

>1OF8A

MSESPMFAANGMPKVNQGAEEDVRILGYDPLASPALLQVQIPATPTSLETAKRGRREAIDIITGKDDRVLVIVGPCSIHDLEAAQEYALRLKKLSDELKGDLSIIMRAYLEKPRTTVGWKGLINDPDVNNTFNINKGLQSARQLFVNLTNIGLPIGSEMLDTISPQYLADLVSFGAIGARTTESQLHRELASGLSFPVGFKNGTDGTLNVAVDACQAAAHSHHFMGVTKHGVAAITTTKGNEHCFVILRGGKKGTNYDAKSVAEAKAQLPAGSNGLMIDYSHGNSNKDFRNQPKVNDVVCEQIANGENAITGVMIESNINEGNQGIPAEGKAGLKYGVSITDACIGWETTEDVLRKLAAAVRQRREVNKK

>1BT3A

APIQAPEISKCVVPPADLPPGAVVDNCCPPVASNIVDYKLPAVTTMKVRPAAHTMDKDAIAKFAKAVELMKALPADDPRNFYQQALVHCAYCNGGYDQVNFPDQEIQVHNSWLFFPFHRWYLYFYERILGKLIGDPSFGLPFWNWDNPGGMVLPDFLNDSTSSLYDSNRNQSHLPPVVVDLGYNGADTDVTDQQRITDNLALMYKQMVTNAGTAELFLGKAYRAGDAPSPGAGSIETSPHIPIHRWVGDPRNTNNEDMGNFYSAGRDIAFYCHHSNVDRMWTIWQQLAGKPRKRDYTDSDWLNATFLFYDENGQAVKVRIGDSLDNQKMGYKYAKTPLPWLDSKP

>3BYWA

SNAPVNQVQSSVSWPQNGSLNSVSAPLMSYTPISFDAKIPVASVDKLRKDQDLILGTLPANSEDAGARGLFVRANDDGLQITSHGELVLDLSKRELAQLPADATIAISATEDETTAGIEGDDSTTETVERDVRPIIMGIYTELESNAAADLLNAGLNAHVEINSRFTSSPTLAKYAS

>3L6BA

MDAQYDISFADVEKAHINIRDSIHLTPVLTSSILNQLTGRNLFFKCELFQKTGSFKIRGALNAVRSLVPDALERKPKAVVTHSSGNHGQALTYAAKLEGIPAYIVVPQTAPDCKKLAIQAYGASIVYCEPSDESRENVAKRVTEETEGIMVHPNQEPAVIAGQGTIALEVLNQVPLVDALVVPVGGGGMLAGIAITVKALKPSVKVYAAEPSNADDCYQSKLKGKLMPNLYPPETIADGVKSSIGLNTWPIIRDLVDDIFTVTEDEIKCATQLVWERMKLLIEPTAGVGVAAVLSQHFQTVSPEVKNICIVLSGGNVDLTSSITWVKQAERPASYQSVSVHHHHHH

>4HPVA

GSHMRNINVQLNPLSDIEKLQVELVERKGLGHPDYIADAVAEEASRKLSLYYLKKYGVILHHNLDKTLVVGGQATPRFKGGDIIQPIYIIVAGRATTEVKTESGIDQIPVGTIIIESVKEWIRNNFRYLDAERHVIVDYKIGKGSSDLVGIFEASKRVPLSNDTSFGVGFAPLTKLEKLVYETERHLNSKQFKAKLPEVGEDIKVMGLRRGNEVDLTIAMATISELIEDVNHYINVKEQVRNQILDLASKIAPGYNVRVYVNTGDKIDKNILYLTVTGTSAEHGDDGMTGRGNRGVGLITPMRPMSLEATAGKNPVNHVGKLYNVLANLIANKIAQEVKDVKFSQVQVLGQIGRPIDDPLIANVDVITYDGKLTDETKNEISGIVDEMLSSFNKLTELILEGKATLF

>3N2ZB

KNYSVLYFQQKVDHFGFNTVKTFNQRYLVADKYWKKNGGSILFYTGNEGDIIWFCNNTGFMWDVAEELKAMLVFAEHRYYGESLPFGDNSFKDSRHLNFLTSEQALADFAELIKHLKRTIPGAENQPVIAIGGSYGGMLAAWFRMKYPHMVVGALAASAPIWQFEDLVPCGVFMKIVTTDFRKSGPHCSESIHRSWDAINRLSNTGSGLQWLTGALHLCSPLTSQDIQHLKDWISETWVNLAMVDYPYASNFLQPLPAWPIKVVCQYLKNPNVSDSLLLQNIFQALNVYYNYSGQVKCLNISETATSSLGTLGWSYQACTEVVMPFCTNGVDDMFEPHSWNLKELSDDCFQQWGVRPRPSWITTMYGGKNISSHTNIVFSNGELDPWSGGGVTKDITDTLVAVTISEGAHHLDLRTKNALDPMSVLLARSLEVRHMKNWIRDFYDS

>4A57A

MTDSSSLRGVDADTEKRINVGKKHLQTLRNLETRCHDSLQALVVIDAGSSSTRTNVFLAKTRSCPNKGRSIDPDSIQLIGAGKRFAGLRVVLEEWLDTYAGKDWESRPVDARLLFQYVPQMHEGAKKLMQLLEEDTVAILDSQLNEKQKVQVKALGIPVMLCSTAGVRDFHEWYRDALFVLLRHLINNPSPAHGYKFFTNPFWTRPITGAEEGLFAFITLNHLSRRLGEDPARCMIDEYGVKQCRNDLAGVVEVGGASAQIVFPLQEGTVLPSSVRAVNLQRERLLPERYPSADVVSVSFMQLGMASSAGLFLKELCSNDEFLQGGICSNPCLFKGFQQSCSAGEVEVRPDGSASVNEDVRKNRLKPLATYCSVNNPEISFKVTNEMQCRENSIDPTKPLAERMKIENCSIIKGTGNFDKCVSQVESILVAPKLPLPANIEAASSGFESVDQVFRFASSTAPMIVTGGGMLAAINTLKDHRLLRSDFSGDVEELAEAAREFCSSEVIIRTDGPVIQLPNARGEQKLNSLNFDLCKTMALTVSLLRHMAAGENQPSFIKWEKSIAGPDGKPLADLGWQVGVILHHVLFTEEWGRNAYEAGYSHNLEHHHHHH

>2J7QA

MKIVRASRDQSAPVYGPRAGSQCMSNCFTFLHTCYLMGIDPVLDTTSLDAVLDSGARLDAIADEKVKRQALTDHPYRLGTEIPTVIETPAGITGHALSRPFNGTAETQDLGGYKCLGILDFLTYARGKPLPVYIIVTVGVHTRGVIVARGATYVFDPHTTDLSAEAAVYVCDDFTEAISALSFFTEMIGDFYYDAVLVYFTRCRTTLISPSELLVQIMDQYKDPDIDASVMS

>2DY0A

GSSGSSGMTATAQQLEYLKNSIKSIQDYPKPGILFRDVTSLLEDPKAYALSIDLLVERYKNAGITKVVGTEARGFLFGAPVALGLGVGFVPVRKPGKLPRETISETYDLEYGTDQLEIHVDAIKPGDKVLVVDDLLATGGTIEATVKLIRRLGGEVADAAFIINLFDLGGEQRLEKQGITSYSLVPFPGH

>3P06A

NGVELSAVGVLLPVLMDSGRRISGGAFMAVKGDLSEHIKNPKNTRIAQTVAGGTIYGLSEMVNIDEAEKLPIKGAITVLPVVQATATSILVPDNQPQLAFNSWEAAACAADTLESQQTPFLMVTGAVESGNLSPNLLAVQKQLLVAKPAGIGLAANSDRALKVVTLEQLRQVVGDKPWRKPMVTFSSGKNVAQA

>2E0AA

GPVPREVEHFSRYSPSPLSMKQLLDFGSENACERTSFAFLRQELPVRLANILKEIDILPTQLVNTSSVQLVKSWYIQSLMDLVEFHEKSPDDQKALSDFVDTLIKVRNRHHNVVPTMAQGIIEYKDACTVDPVTNQNLQYFLDRFYMNRISTRMLMNQHILIFSDSQTGNPSHIGSIDPNCDVVAVVQDAFECSRMLCDQYYLSSPELKLTQVNGKFPDQPIHIVYVPSHLHHMLFELFKNAMRATVEHQENQPSLTPIEVIVVLGKEDLTIKISDRGGGVPLRIIDRLFSYTYSTAPTPVMDNSRNAPLAGFGYGLPISRLYAKYFQGDLNLYSLSGYGTDAIIYLKALSSESIEKLPVFNKSAFKHYQMSSEADDWCIPSREPKNLAKEVAM

>2O5NA

EVVRPEVNRTGTVDICQGPMELIFSVSRTSSGATGERISLKNTLSIVSMENGGKPGTYEWSFPANESWPEIQFLLQNREFVSKYYADVVQTPGELVVEYRCPVPQFNCTITHRWKGETIMSFDGAIQTIRSVTSEYTTKNEDTLVKYIRGLNVTLLTDNAKSIEHRWTEICKKLKDADRPDDNQYTLEDDILEDDIEMDIVQCQMTTQVPLKYHMTVWSAGRDSRAIALSADYYTDIEVASYLPVNRSQILNTTCEITSSSGWTVRLRFSEEMVAASKARQAQKRPLLPVEPHGFMSDEHGPAFVQRTINDSRLTLVPR

>3QVLA

SVRIQVINPNTSLAMTETIGAAARAVAAPGTEILAVCPRAGVPSIEGHFDEAIAAVGVLEQIRAGREQGVDGHVIASFGDPGLLAARELAQGPVIGIAEAAMHMATMVATRFSIVTTLPRTLIIARHLLHQYGFHQHCAALHAIDLPVLALEDGSGLAQEKVRERCIRALKEDGSGAIVLGSGGMATLAQQLTRELRVPVIDGVSAAVKMVESLVALGLATSKHGDLAFPEKKALSGQFQSLNPF

>4IAOC

GSMNTIITHPGKMELVYVSDSDDSSSDNDSLTDLESLSSGESNEIKVTNDLDTSAEKDQIQAGKWFDPVLDWRKSDRELTKNILWRIADKTTYDKETITDLIEQGIPKHSYLSGNPLTSVTNDICSVENYETSSAFFYQQVHKKDRLQYLPLYAVSTFE

>1RWRA

QGLVPQGQTQVLQGGNKVPVVNIADPNSGGVSHNKFQQFNVANPGVVFNNGLTDGVSRIGGALTKNPNLTRQASAILAEVTDTSPSRLAGTLEVYGKGADLIIANPNGISVNGLSTLNASNLTLTTGRPSVNGGRIGLDVQQGTVTIERGGVNATGLGYFDVVARLVKLQGAVSSKQGKPLADIAVVAGANRYDHATRRATPIAAGARGAAAGAYAIDGTAAGAMYGKHITLVSSDSGLGVRQLGSLSSPSAITVSSQGEIALGDATVQRGPLSLKGAGVVSAGKLASGGGAVNVAGGGAV

>1HDHA

MSKRPNFLVIVADDLGFSDIGAFGGEIATPNLDALAIAGLRLTDFHTASTXSPTRSMLLTGTDHHIAGIGTMAEALTPELEGKPGYEGHLNERVVALPELLREAGYQTLMAGKWHLGLKPEQTPHARGFERSFSLLPGAANHYGFEPPYDESTPRILKGTPALYVEDERYLDTLPEGFYSSDAFGDKLLQYLKERDQSRPFFAYLPFSAPHWPLQAPREIVEKYRGRYDAGPEALRQERLARLKELGLVEADVEAHPVLALTREWEALEDEERAKSARAMEVYAAMVERMDWNIGRVVDYLRRQGELDNTFVLFMSDNGAEGALLEAFPKFGPDLLGFLDRHYDNSLENIGRANSYVWYGPRWAQAATAPSRLYKAFTTQGGIRVPALVRYPRLSRQGAISHAFATVMDVTPTLLDLAGVRHPGKRWRGREIAEPRGRSWLGWLSGETEAAHDENTVTGWELFGMRAIRQGDWKAVYLPAPVGPATWQLYDLARDPGEIHDLADSQPGKLAELIEHWKRYVSETGVVEGASPFLVR

>4F11A

WARGAPRPPPSSPPLSIMGLMPLTKEVAKGSIGRGVLPAVELAIEQIRNESLLRPYFLDLRLYDTECDNAKGLKAFYDAIKYGPNHLMVFGGVCPSVTSIIAESLQGWNLVQLSFAATTPVLADKKKYPYFFRTVPSDNAVNPAILKLLKHYQWKRVGTLTQDVQRFSEVRNDLTGVLYGEDIEISDTESFSNDPCTSVKKLKGNDVRIILGQFDQNMAAKVFCCAYEENMYGSKYQWIIPGWYEPSWWEQVHTEANSSRCLRKNLLAAMEGYIGVDFEPLSSKQIKTISGKTPQQYEREYNNKRSGVGPSKFHGYAYDGIWVIAKTLQRAMETLHASSRHQRIQDFNYTDHTLGRIILNAMNETNFFGVTGQVVFRNGERMGTIKFTQFQDSREVKVGEYNAVADTLEIINDTIRFQGSEPPKDDYKDDDDK

>2WNPF

SCATGPRNCKDLLDRGYFLSGWHTIYLPDCRPLTVLCDMDTDGGGWTVFQRRMDGSVDFYRDWAAYKQGFGSQLGEFWLGNDNIHALTAQGSSELRVDLVDFEGNHQFAKYKSFKVADEAEKYKLVLGAFVGGSAGNSLTGHNNNFFSTKDQDNDVSSSNCAEKFQGAWWYADCHASNLNGLYLMGPHESFANGINWSAAKGYKYSYKVSEMKVRPA

>1JF3A

GLSAAQRQVVASTWKDIAGADNGAGVGKECLSKFISAHPEMAAVFGFSGASDPGVAELGAKVLAQIGVAVSHLGDEGKMVAEMKAVGVRHKGYGNKHIKAEYFEPLGASLLSAMEHRIGGKMNAAAKDAWAAAYGDISGALISGLQS

>3ESMA

MSLHVTADAPGAAQGGYSVVTFRVPTESETAATTAMTVTLPNVRSARTEPMPGWTARVDRNDKSEAVSVTWTADPGNPGVQPGQFQRFVVSIGPLPSAETVSFPAEQTYSDGRVVAWNQPPAANXSEPEHPAPTLTLATAPGDTEGHHHHHH

>2PLGA

MSLTMVSEVQPVSPASLDAPLENAVEIIETVISSLHQGDAPLVGQTDSGKIWMFRYGSAEVFVQLSGHTEEDFLTIWSPVLPLPVADELALYRKLLTLNWLTTFEAHFAIAEEQVQVVASRTLGGITAGEISRLITIVATLADDYDDALRAEFKGEGHHHHHH

>3LGEE

QAYQGPATGDDDDWDEDWDGPKSSSYFKDSE

>2RINA

AEPESCGTVRFSDVGWTDITATTATATTILEALGYETDVKVLSVPVTYTSLKNKDIDVFLGNWMPTMEADIAPYREDKSVETVRENLAGAKYTLATNAKGAELGIKDFKDIAAHKDELDGKIYGIEPGNDGNRLIIDMVEKGTFDLKGFEVVESSEQGMLAQVARAEKSGDPIVFLGWEPHPMNANFKLTYLSGGDDVFGPNYGGATVHTNVRAGYTTECPNVDKLLQNLSFSLQMENEIMGKILNDGEDPEKAAAAWLKDNPQSIEPWLSGVATKDGGDGLAAVKAALGLEHHHHHH

>3N1MC

GSTERPVAGPYITFTDAVNETTIMLKWMYIPASNNNTPIHGFYIYYRPTDSDNDSDYKKDMVEGDKYWHSISHLQPETSYDIKMQCFNEGGESEFSNVMICETKARKSSGQ

>4F2DA

MTIFDNYEVWFVIGSQHLYGPETLRQVTQHAEHVVNALNTEAKLPCKLVLKPLGTTPDEITAICRDANYDDRCAGLVVWLHTFSPAKMWINGLTMLNKPLLQFHTQFNAALPWDSIDMDFMNLNQTAHGGREFGFIGARMRQQHAVVTGHWQDKQAHERIGSWMRQAVSKQDTRHLKVCRFGDNMREVAVTDGDKVAAQIKFGFSVNTWAVGDLVQVVNSISDGDVNALVDEYESCYTMTPATQIHGEKRQNVLEAARIELGMKRFLEQGGFHAFTTTFEDLHGLKQLPGLAVQRLMQQGYGFAGEGDWKTAALLRIMKVMSTGLQGGTSFMEDYTYHFEKGNDLVLGSHMLEVCPSIAVEEKPILDVQHLGIGGKDDPARLIFNTQTGPAIVASLIDLGDRYRLLVNCIDTVKTPHSLPKLPVANALWKAQPDLPTASEAWILAGGAHHTVFSHALNLNDMRQFAEMHDIEITVIDNDTRLPAFKDALRWNEVYYGFRR

>3MD9A

MAERIVTIGGDVTEIAYALGAGDEIVARDSTSQQPQAAQKLPDVGYMRTLNAEGILAMKPTMLLVSELAQPSLVLTQIASSGVNVVTVPGQTTPESVAMKINAVATALHQTEKGQKLIEDYQQRLAAVNKTPLPVKVLFVMSHGGLTPMAAGQNTAADAMIRAAGGSNAMQGFSRYRPLSQEGVIASAPDLLLITTDGVKALGSSENIWKLPGMALTPAGKHKRLLVVDDMALLGFGLETPQVLAQLREKMEQMQ

>3OD9A

MADGFFKQLTLPSGQVVTVSEGRGEPASTGSYDVRLYSGANPQFPLDQFIDGKVLPRDGSIKELKLLDLNGDKQPELIVVVESAGSGSYLSADAFTLNPQEGLDSFNHVEGLAPNEDVIQALKTPRDLEHHHHHH

>1GY7A

MSLDFNTLAQNFTQFYYNQFDTDRSQLGNLYRNESMLTFETSQLQGAKDIVEKLVSLPFQKVQHRITTLDAQPASPYGDVLVMITGDLLIDEEQNPQRFSQVFHLIPDGNSYYVFNDIFRLNYSA

>3D2OA

MNAIADVQSSRDLRNLPINQVGIKDLRFPITLKTAEGTQSTVARLTMTVYLPAEQKGTHMSRFVALMEQHTEVLDFAQLHRLTAEMVALLDSRAGKISVSFPFFRKKTAPVSGIRSLLDYDVSLTGEMKDGAYGHSMKVMIPVTSLCPCSKEISQYGAHNQRSHVTVSLTSDAEVGIEEVIDYVETQASCQLYGLLKRPDEKYVTEKAYENPKFVEDMVRDVATSLIADKRIKSFVVESENFESIHNHSAYAYIAYP

>1PBWA

MEADVEQQALTLPDLAEQFAPPDIAPPLLIKLVEAIEKKGLECSTLYRTQSSSNLAELRQLLDCDTPSVDLEMIDVHVLADAFKRYLLDLPNPVIPAAVYSEMISLAPEVQSSEEYIQLLKKLIRSPSIPHQYWLTLQYLLKHFFKLSQTSSKNLLNARVLSEIFSPMLFRFSAASSDNTENLIKVIEILISTEWNERQPAPALPPKPPKPTTVAN

>2WW5A

NETEVAKTSQDTTTASSSSEQNQSSNKTQTSAEVQTNAAAYWDGDYYVKDDGSKAQSEWIFDNYYKAWFYINSDGRYSQNEWHGNYYLKSGGYMAQNEWIYDSNYKSWFYLKSDGAYAHQEWQLIGNKWYYFKKWGYMAKSQWQGSYFLNGQGAMIQNEWLYDPAYSAYFYLKSDGTYANQEWQKVGGKWYYFKKWGYMARNEWQGNYYLTGSGAMATDEVIMDGARYIFAASGELKEKKDLNVGWVHRDGKRYFFNNREEQVGTEHAKKIIDISEHNGRINDWKKVIDENEVDGVIVRLGYSGKEDKELAHNIKELNRLGIPYGVYLYTYAENETDAENDAKQTIELIKKYNMNLSYPIYYDVENWEYVNKSKRAPSDTDTWVKIINKYMDTMKQAGYQNVYVYSYRSLLQTRLKHPDILKHVNWVAAYTNALEWENPYYSGEKGWQYTSSEYMKGIQGRVDVSVWY

>2FBAA

AYPSFEAYSNYKVDRTDLETFLDKQKEVSLYYLLQNIAYPEGQFNNGVPGTVIASPSTSNPDYYYQWTRDSAITFLTVLSELEDNNFNTTLAKAVEYYINTSYNLQRTSNPSGSFDDENHKGLGEPKFNTDGSAYTGAWGRPQNDGPALRAYAISRYLNDVNSLNEGKLVLTDSGDINFSSTEDIYKNIIKPDLEYVIGYWDSTGFDLWEENQGRHFFTSLVQQKALAYAVDIAKSFDDGDFANTLSSTASTLESYLSGSDGGFVNTDVNHIVENPDLLQQNSRQGLDSATYIGPLLTHDIGESSSTPFDVDNEYVLQSYYLLLEDNKDRYSVNSAYSAGAAIGRYPEDVYNGDGSSEGNPWFLATAYAAQVPYKLAYDAKSASNDITINKINYDFFNKYIVDLSTINSAYQSSDSVTIKSGSDEFNTVADNLVTFGDSFLQVILDHINDDGSLNEQLNRYTGYSTGAYSLTWSSGALLEAIRLRNKVKALA

>4JFHE

SQTIHQWPATLVQPVGSPLSLECTVEGTSNPNLYWYRQAAGRGPQLLFYWGPFGQISSEVPQNLSASRPQDRQFILSSKKLLLSDSGFYLCAWSETGLGMGGWQFGEGSRLTVLEDLKNVFPPEVAVFEPSEAEISHTQKATLVCLATGFYPDHVELSWWVNGKEVHSGVCTDPQPLKEQPALNDSRYALSSRLRVSATFWQDPRNHFRCQVQFYGLSENDEWTQDRAKPVTQIVSAEAWGRAD

>3CWNA

MGSSHHHHHHSSGLVPRGSHMTDKLTSLRQYTTVVADTGDIAAMKLYQPQDATTNPSLILNAAQIPEYRKLIDDAVAWAKQQSNDRAQQIVDATDKLAVNIGLEILKLVPGRISTEVDARLSYDTEASIAKAKRLIKLYNDAGISNDRILIKLASTWQGIRAAEQLEKEGINCNLTLLFSFAQARACAEAGVFLISPYVGRILDWYKANTDKKEYAPAEDPGVVSVSEIYQYYKEHGYETVVMGASFRNIGEILELAGCDRLTIAPTLLKELAESEGAIERKLSYTGEVKARPARITESEFLWQHNQDPMAVDKLAEGIRKFAIDQEKLEKMIGDLL

>1EG2A

MANRSHHNAGHRAMNALRKSGQKHSSESQLGSSEIGTTRHVYDVCDCLDTLAKLPDDSVQLIICDPPYNIMLADWDDHMDYIGWAKRWLAEAERVLSPTGSIAIFGGLQYQGEAGSGDLISIISHMRQNSKMLLANLIIWNYPNGMSAQRFFANRHEEIAWFAKTKKYFFDLDAVREPYDEETKAAYMKDKRLNPESVEKGRNPTNVWRMSRLNGNSLERVGHPTQKPAAVIERLVRALSHPGSTVLDFFAGSGVTARVAIQEGRNSICTDAAPVFKEYYQKQLTFLQDDGLIDKARSYEIVEGAANFGAALQRGDVAS

>1NWWA

MTSKIEQPRWASKDSAAGAASTPDEKIVLEFMDALTSNDAAKLIEYFAEDTMYQNMPLPPAYGRDAVEQTLAGLFTVMSIDAVETFHIGSSNGLVYTERVDVLRALPTGKSYNLSILGVFQLTEGKITGWRDYFDLREFEEAVDLPLRG

>1EYBA

MGHHHHHHHHHHSSGHIDDDDKHMGSMAELKYISGFGNECSSEDPRCPGSLPEGQNNPQVCPYNLYAEQLSGSAFTCPRSTNKRSWLYRILPSVSHKPFESIDEGHVTHNWDEVDPDPNQLRWKPFEIPKASQKKVDFVSGLHTLCGAGDIKSNNGLAIHIFLCNTSMENRCFYNSDGDFLIVPQKGNLLIYTEFGKMLVQPNEICVIQRGMRFSIDVFEETRGYILEVYGVHFELPDLGPIGANGLANPRDFLIPIAWYEDRQVPGGYTVINKYQGKLFAAKQDVSPFNVVAWHGNYTPYKYNLKNFMVINSVAFDHADPSIFTVLTAKSVRPGVAIADFVIFPPRWGVADKTFRPPYYHRNCMSEFMGLIRGHYEAKQGGFLPGGGSLHSTMTPHGPDADCFEKASKVKLAPERIADGTMAFMFESSLSLAVTKWGLKASRCLDENYHKCWEPLKSHFTPNSRNPAEPN

>1F60B

KPAKPAAKSIVTLDVKPWDDETNLEEMVANVKAIEMEGLTWGAHQFIPIGFGIKKLQINCVVEDDKVSLDDLQQSIEEDEDHVQSTDIAAMQKL

>3VOQA

GAMATVQDMLSSHHYKSFKVSMIHRLRFTTDVQLGISGDKVEIDPVTNQKASTKFWIKQKPISIDSDLLCACDLAEEKSPSHAIFKLTYLSNHDYKHLYFESDAATVNEIVLKVNYILESRASTA

>4ESQA

GHQPVAEERLSALLLNSSEVNAVMGSSSMQPGKPITSMDSSPVTVSLPDCQGALYTSQDPVYAGTGYTAINGLISSEPGDNYEHWVNQAVVAFPTADKARAFVQTSADKWKNCAGKTVTVTNKAKTYRWTFADVKGSPPTITVIDTQEGAEGWECQRAMSVANNVVVDVNACGYQITNQAGQIAAKIVDKVNKE

>2QLWA

MRGSHHHHHHGMASMTGGQQMGRDLYDDDDKDRWGSGDMTLEKHAFKMQLNPGMEAEYRKRHDEIWPELVDLLHQSGASDYSIHLDRETNTLFGVLTRPKDHTMASLPDHPVMKKWWAHMADIMATNPDNSPVQSDLVTLFHMP

>3KVCA

MSSQVEHPAGGYKKLFETVEELSSPLTAHVTGRIPLWLTGSLLRCGPGLFEVGSEPFYHLFDGQALLHKFDFKEGHVTYHRRFIRTDAYVRAMTEKRIVITEFGTCAFPDPCKNIFSRFFSYFRGVEVTDNALVNIYPVGEDYYACTETNFITKVNPETLETIKQVDLCNYVSVNGATAHPHIENDGTVYNIGNCFGKNFSIAYNIVKIPPLQADKEDPISKSEIVVQFPCSDRFKPSYVHSFGLTPNYIVFVETPVKINLFKFLSSWSLWGANYMDCFESNETMGVWLHIADKKRKKYINNKYRTSPFNLFHHINTYEDHEFLIVDLCCWKGFEFVYNYLYLANLRENWEEVKKNARKAPQPEVRRYVLPLNIDKADTGKNLVTLPNTTATAILCSDETIWLEPEVLFSGPRQAFEFPQINYQKYGGKPYTYAYGLGLNHFVPDRLCKLNVKTKETWVWQEPDSYPSEPIFVSHPDALEEDDGVVLSVVVSPGAGQKPAYLLILNAKDLSEVARAEVEINIPVTFHGLFKKS

>3DA7A

SGRTWREADINYTSGFRNSDRILYSSDWLIYKTTDHYQTFTKIRCAQVINTFDGVADYLQTYHKLPDNYITKSEAQALGWVASKGNLADVAPGKSIGGDIFSNREGKLPGK

>3DEEA

GMQPETSAQYQHRFSQAIRGGEAADGLPQDRLNVYIRLIRNNIHSFIDRCYTETRQYFDSKEWSRLKEGFVRDARAQTPYFQEIPGEFLQYCQSLPLSDGILALMDFEYTQLLAEVAQIPDIPDIHYSNDSKYTPSPAAFIRQYRYDVTHDLQEAETALLIWRNAEDDVMYQTLDGFDMMLLEIMGSSALSFDTLAQTLVEFMPKADNWKNILLGKWSGWIEQRIIIPSLSAISENMEGNSPSQNHLSA

>3DORA

SLVCKNALQDLSFLEHLLQVKYAPKTWKEQYLGWDLVQSSVSAQQKLRTQENPSTSFCQQVLADFIGGLNDFHAGVTFFAIESAYLPYTVQKSSDGRFYFVDIMTFSSEIRVGDELLEVDGAPVQDVLATLYGSNHKGTAAEESAALRTLFSRMASLGHKVPSGRTTLKIRRPFGTTREVRVKWRYVPEGVGDLATIAPSIRAPQLQKSMRSFFPKKDDAFHRSSSLFYSPMVPHFWAELRNHYATSGLKSGYNIGSTDGFLPVIGPVIWESEGLFRAYISSVTDGDGKSHKVGFLRIPTYSWQDMEDFDPSGPPPWEEFAKIIQVFSSNTEALIIDQTNNPGGSVLYLYALLSMLTDRPLELPKHRMILTQDEVVDALDWLTLLENVDTNVESRLALGDNMEGYTVDLQVAEYLKSFGRQVLNCWSKGDIELSTPIPLFGFEKIHPHPRVQYSKPICVLINEQDFSCADFFPVVLKDNDRALIVGTRTAGAGGFVFNVQFPNRTGIKTCSLTGSLAVREHGAFIENIGVEPHIDLPFTANDIRYKGYSEYLDKVKKLVCQLINNDGTIILAEDGSFHHHHHH

>2GDMA

GALTESQAALVKSSWEEFNANIPKHTHRFFILVLEIAPAAKDLFSFLKGTSEVPQNNPELQAHAGKVFKLVYEAAIQLEVTGVVVTDATLKNLGSVHVSKGVADAHFPVVKEAILKTIKEVVGAKWSEELNSAWTIAYDELAIVIKKEMDDAA

>4AAJA

MGSSHHHHHHSSGLVPRGSHMFVKICGIKSLEELEIVEKHADATGVVVNSNSKRRIPLEKAREIIENSAIPVFLVSTMVGFSEWAMAIERTGAQYIQVHSNALPQTIDTLKKEFGVFVMKAFRVPTISKNPEEDANRLLSEISRYNADMVLLDTGAGSGKLHDLRVSSLVARKIPVIVAGGLNAENVEEVIKVVKPYGVDVSSGVEKYGIKDPKLVEEFVRRAKNVVW

>3K40A

MEAPEFKDFAKTMVDFIAEYLENIRERRVLPEVKPGYLKPLIPDAAPEKPEKWQDVMQDIERVIMPGVTHWHSPKFHAYFPTANSYPAIVADMLSGAIACIGFTWIASPACTELEVVMMDWLGKMLELPAEFLACSGGKGGGVIQGTASESTLVALLGAKAKKLKEVKELHPEWDEHTILGKLVGYCSDQAHSSVERAGLLGGVKLRSVQSENHRMRGAALEKAIEQDVAEGLIPFYAVVTLGTTNSCAFDYLDECGPVGNKHNLWIHVDAAYAGSAFICPEYRHLMKGIESADSFNFNPHKWMLVNFDCSAMWLKDPSWVVNAFNVDPLYLKHDMQGSAPDYRHWQIPLGRRFRALKLWFVLRLYGVENLQAHIRRHCNFAKQFGDLCVADSRFELAAEINMGLVCFRLKGSNERNEALLKRINGRGHIHLVPAKIKDVYFLRMAICSRFTQSEDMEYSWKEVSAAADEMEQEQ

>3CIHA

MSLILLGALSLASSTFAQTWIWYPGDYEIWLGNQMNNRRTERGAFFPPFWKTDSHYVVVEFSKVLNLSEPEEVFIAAEGTYNVKLDGKLQFGMPETLLLPAGKHSLNIKVWNQATPPTIYVKGKTVNSDSSWRVTYEDKEWIDESGKASDTSATIYMDAGCWNFDGATQRPSQFSLMREPQQPVAKTEQPEGGILYDFGKETFGFITLKNLSGKGKIDLYYGESPEEAKDKAYCETLDKLLLEPGQITDLAIRSTSPLHHSDNEYTLENSKAFRYVYITHEPEVQIGEVSMQYEYLPEEYRGNFRCNDEELNCIWEVGAYTMHLTTREFFIDGIKRDRWVWSGDAIQSYLMNYYLFFDSESVKRTIWLLRGKDPVTSHSNTIMDYTFYWFLSVYDYYMYSGDRHFVNQLYPRMQTMMDYVLGRTNKNGMVEGMSGDWVFVDWADGYLDKKGELSFEQVLFCRSLETMALCADLVGDKDGQQKYEKLASALKAKLEPTFWNNQKQAFVHNCVDGRQSDAVTRYANMFSVFFDYLNADKQQAIKQSVLLNDEILKITTPYMRFYELEALCALGEQETVMKEMKAYWGGMLKAGATSFWEKYNPEESGTQHLAMYGRPYGKSLCHAWGASPIYLLGKYYLGVKPTKEGYKEFAVSPVLGGLKWMEGTVPTPNGDIHVYMDNKTIKVKATEGKGYLTIQSRRQPKANMGTVEKVSEGVWRLWIDSPEERIVTYRLEGHHHHHH

>3LHOA

GMHTDVNALFAALWQDYIKMTPSAAKIHQLLGHGAPIINDHIALRTFNIAKVNLSVLAKHFTSIGYVDSGDYKFEQKKLIAKHFEHPDPKQPKVFISELLVEEFSPEVQKSIHGLIDQVDIAATTADNFIYSGRHWDVDKATYQALLAESEYAAWVAALGYRANHFTVSINDLPEFERIEDVNQALKQAGFVLNSSGGEVKGSPEVLLEQSSTMADKVVVNFTDGDVEIPSCFYEFARRYPMANGQLYTGFVAASADKIFESTNAMM

>1Z70X

EANAPGPVPGERQLAHSKMVPIPAGVFTMGTDDPQIKQDGEAPARRVTIDAFYMDAYEVSNTEFEKFVNSTGYLTEAEKFGDSFVFEGMLSEQVKTNIQQAVAAAPWWLPVKGANWRHPEGPDSTILHRPDHPVLHVSWNDAVAYCTWAGKRLPTEAEWEYSCRGGLHNRLFPWGNKLQPKGQHYANIWQGEFPVTNTGEDGFQGTAPVDAFPPNGYGLYNIVGNAWEWTSDWWTVHHSVEETLNPKGPPSGKDRVKKGGSYMCHRSYCYRYRCAARSQNTPDSSASNLGFRCAADRLPTMDRGSHHHHHH

>3NVOA

GHMEAIKGSDVNVPDAVFAWLLDGRGGVKPLEDNDVIDSQHPCWLHLNYTHPDSARWLASTPLLPNNVRDALAGESSRPRVSRMGEGTLITLRCINGSTDERPDQLVAMRLYMDERFIVSTRQRKVLALDDVVSDLQEGTGPVDCGGWLVDVCDALTDHASEFIEELHDKIIDLEDNLLDQQIPPRGFLALLRKQLIVMRRYMAPQRDVYARLASERLPWMSDDHRRRMQDIADRLGRGLDEIDACIARTGIMADEIAQVMQES

>2A14A

MKGGFTGGDEYQKHFLPRDYLATYYSFDGSPSPEAEMLKFNLECLHKTFGPGGLQGDTLIDIGSGPTIYQVLAACDSFQDITLSDFTDRNREELEKWLKKEPGAYDWTPAVKFACELEGNSGRWEEKEEKLRAAVKRVLKCDVHLGNPLAPAVLPLADCVLTLLAMECACCSLDAYRAALCNLASLLKPGGHLVTTVTLRLPSYMVGKREFSCVALEKGEVEQAVLDAGFDIEQLLHSPQSYSVTNAANNGVCCIVARKKPGP

>1VR4A

MIVTTTSGIQGKEIIEYIDIVNGEAIMGANIVRDLFASVRDVVGGRAGSYESKLKEARDIAMDEMKELAKQKGANAIVGVDVDYEVVRDGMLMVAVSGTAVRI

>4E3YA

DTLESIDNCAVGCPTGGSSNVSIVRHAYTLNNNSTTKFANWVAYHITKDTPASGKTRNWKTDPALNPADTLAPADYTGANAALKVDRGHQAPLASLAGVSDWESLNYLSNITPQKSDLNQGAWARLEDQERKLIDRADISSVYTVTGPLYERDMGKLPGTQKAHTIPSAYWKVIFINNSPAVNHYAAFLFDQNTPKGADFCQFRVTVDEIEKRTGLIIWAGLPDDVQASLKSKPGVLPELMGCKN

>2UXYA

MRHGDISSSNDTVGVAVVNYKMPRLHTAAEVLDNARKIAEMIVGMKQGLPGMDLVVFPEYSLQGIMYDPAEMMETAVAIPGEETEIFSRACRKANVWGVFSLTGERHEEHPRKAPYNTLVLIDNNGEIVQKYRKIIPWCPIEGWYPGGQTYVSEGPKGMKISLIICDDGNYPEIWRDCAMKGAELIVRCQGYMYPAKDQQVMMAKAMAWANNCYVAVANAAGFDGVYSYFGHSAIIGFDGRTLGECGEEEMGIQYAQLSLSQIRDARANDQSQNHLFKILHRGYSGLQASGDGDRGLAECPFEFYRTWVTDAEKARENVERLTRSTTGVAQCPVGRLPYEG

>2CWRA

GPTTPVPVSGSLEVKVNDWGSGAEYDVTLNLDGQYDWTVKVKLAPGATVGSFWSANKQEGNGYVIFTPVSWNKGPTATFGFIVNGPQGDKVEEITLEINGQVI

>2II2A

GPLGSYGDAIPEVKAILEAKNEEELVTFTSRWSAEERKELRTQFQDTTGLEFIAFLKKCIKNGPYEDVMALGWDCNISARVNVIKKAMKNVNDFRAIHDVVLIATPDERLKLAQAYKEKTGNDLLQDFVDQIPLTSAASYLCHLAIRENRTPRGSVASDAEVLKHNLIDADEPDHEAVVRLIITSTADEYKEINHRFEVLTGKSVQEAIETRYADKENARGLCIAHYYNLAPARAVAYAFHSAVETQNDDMAYEQAARITGLFHDLHKFAWVHYACWGVMRDDILSRFQSKEANKVNFRDACLMFWKLAK

>3ZITA

MKKIEVYAQPDCPPCVIVKEFLKHNNVAYEEFDVKKDAAARNRLLYDYDSYSTPTVVIDGEVVAGFQIEKLQQLLNIE

>2VX8A

GSVASVHASISGSSASSTSSTPEVKPLKSLLGDSAPTLHLNKGMAILFAVVARGTTILAKHAWCGGNFLEVTEQILAKIPSENNKLTYSHGNYLFHYICQDRIVYLCITDDDFERSRAFSFLNEVKKRFQTTYGSRAQTALPYAMNSEFSSVLAAQLKHHSENHHHHHH

>4HPVA

GSHMRNINVQLNPLSDIEKLQVELVERKGLGHPDYIADAVAEEASRKLSLYYLKKYGVILHHNLDKTLVVGGQATPRFKGGDIIQPIYIIVAGRATTEVKTESGIDQIPVGTIIIESVKEWIRNNFRYLDAERHVIVDYKIGKGSSDLVGIFEASKRVPLSNDTSFGVGFAPLTKLEKLVYETERHLNSKQFKAKLPEVGEDIKVMGLRRGNEVDLTIAMATISELIEDVNHYINVKEQVRNQILDLASKIAPGYNVRVYVNTGDKIDKNILYLTVTGTSAEHGDDGMTGRGNRGVGLITPMRPMSLEATAGKNPVNHVGKLYNVLANLIANKIAQEVKDVKFSQVQVLGQIGRPIDDPLIANVDVITYDGKLTDETKNEISGIVDEMLSSFNKLTELILEGKATLF

>1JX6A

VLNGYWGYQEFLDEFPEQRNLTNALSEAVRAQPVPLSKPTQRPIKISVVYPGQQVSDYWVRNIASFEKRLYKLNINYQLNQVFTRPNADIKQQSLSLMEALKSKSDYLIFTLDTTRHRKFVEHVLDSTNTKLILQNITTPVREWDKHQPFLYVGFDHAEGSRELATEFGKFFPKHTYYSVLYFSEGYISDVRGDTFIHQVNRDNNFELQSAYYTKATKQSGYDAAKASLAKHPDVDFIYACSTDVALGAVDALAELGREDIMINGWGGGSAELDAIQKGDLDITVMRMNDDTGIAMAEAIKWDLEDKPVPTVYSGDFEIVTKADSPERIEALKKRAFRYSDN

>1VPBA

MGSDKIHHHHHHMITDENKKLAQWAMDYALKNGCQAAKVLLYSSSNTSFELRDAKMDRLQQASEGGLSLSLYVDGRYGSISTNRLNRKELETFIKNGIDSTRYLAKDEARVLADPSRYYKGGKPDLKLYDAKFASLNPDDKIEMAKAVAEEALGKDERIISVGSSYGDGEDFAYRLISNGFEGETKSTWYSLSADITIRGEGEARPSAYWYESSLYMNDLIKKGIGQKALERVLRKLGQKKVQSGKYTMVVDPMNSSRLLSPMISALNGSALQQKNSFLLNKLNEKIASDRLTLTDEPHLVKASGARYFDNEGIATERRSIFDKGVLNTYFIDTYNAKKMGVDPTISGSSILVMETGDKNLDGLIAGVEKGILVTGFNGGNNNSSTGDFSYGIEGFLIENGKLTQPVSEMNVTGNLITLWNSLVATGNDPRLNSSWRIPSLVFEGVDFSGL

>2ZUXA

AARQMEALNRGLVAVKTDGGIFVSWRFLGTENASVLFNVYRDGQKLNAAPVKTTNYVDKNGSAGSTYTVRAVVNGTEQPASEKASVWAQPYHSVPLDKPAGGTTPKGESYTYSANDASVGDVDGDGQYELILKWDPSNSKDNSQDGYTGDVLIDAYKLDGTKLWRINLGKNIRAGAHYTQFMVYDLDGDGKAEVAMKTADGTKDGTGKVIGNANADYRNEQGRVLSGPEYLTVFQGSTGKELVTANFEPARGNVSDWGDSYGNRVDRFLAGIAYLDGQRPSLIMTRGYYAKTMLVAYNFRDGKLSKLWTLDSSKSGNEAFAGQGNHNLSIADVDGDGKDEIIFGSMAVDHDGKGMYSTGLGHGDALHTGDLDPGRPGLEVFQVHEDKNAKYGLSFRDAATGKILWGVYAGKDVGRGMAADIDPRYPGQEVWANGSLYSAKGVKIGSGVPSSTNFGIWWDGDLLREQLDSNRIDKWDYQNGVSKNMLTASGAAANNGTKATPTLQADLLGDWREEVVWRTEDSSALRIYTTTIPTEHRLYTLMHDPVYRLGIAWQNIAYNQPPHTSFFLGDGMAEQPKPNMYTPLEHHHHHH

>2BS2B

MGRMLTIRVFKYDPQSAVSKPHFQEYKIEEAPSMTIFIVLNMIRETYDPDLNFDFVCRAGICGSCGMMINGRPSLACRTLTKDFEDGVITLLPLPAFKLIKDLSVDTGNWFNGMSQRVESWIHAQKEHDISKLEERIEPEVAQEVFELDRCIECGCCIAACGTKIMREDFVGAAGLNRVVRFMIDPHDERTDEDYYELIGDDDGVFGCMTLLACHDVCPKNLPLQSKIAYLRRKMVSVNMS

>3ZE3A

GHHHHHHELANNTTGFTRIIKAAGYSWKGLRAAWINEAAFRQEGVAVLLCVVIAAWLDVDAVTRVLLISSVMLVMIVELLNSAIEAVVDRIGSEYHELSGRAKDLGSAAVLIAIIDAVITWAILLWSHFG

>1W66A

GAMAGSIRSKLSAIDVRQLGTVDYRTAWQLQRELADARVAGGADTLLLLEHPAVYTAGRRTETHERPIDGTPVVDTDRGGKITWHGPGQLVGYPIIGLAEPLDVVNYVRRLEESLIQVCADLGLHAGRVDGRSGVWLPGRPARKVAAIGVRVSRATTLHGFALNCDCDLAAFTAIVPCGISDAAVTSLSAELGRTVTVDEVRATVAAAVCAALDGVLPVGDRVPSHAVPSPL

>4HZ4A

MVMITLHYLKQSCSHRIVWLLEALGLDYELKIYDRLEGTGFAPEELKAQHPLGKAPVLQDGDLVLAEGNAIIQHLLDRYDTENRFTPAHKTDAYSNYVYWLAISASMFSANLLALVSKKGDLGDFAQYTNAQVGLYFSHVEKSLEGKTWIVGEQLTGADFALSFPLQWGLNYVNKADYPNITRYLEQIETHPAYLKANEKTDGGLDLSRFAENLYFQ

>3NUFA

GMTPLDANVELPTEVKAMIEQSSDAQAATALVNYVIKLAAAAEIHFTDLQLQVLTNHLIEMLGRSKSGEQLPAVDPTMFAEVSQKSLDLADQVVQHIGHLEVAEKYVLSIHFEAAQDKI

>1EFDN

AGIDPNRIVALEWLPVELLLALGIVPYGVADTINYRLWVSEPPLPDSVIDVGLRTEPNLELLTEMKPSFMVWSAGYGPSPEMLARIAPGRGFNFSDGKQPLAMARKSLTEMADLLNLQSAAETHLAQYEDFIRSMKPRFVKRGARPLLLTTLIDPRHMLVFGPNSLFQEILDEYGIPNAWQGETNFWGSTAVSIDRLAAYKDVDVLCFDHDNSKDMDALMATPLWQAMPFVRAGRFQRVPAVWFYGATLSAMHFVRVLDNAIGGKA

>2ZQ5A

MTRRPDRKDVATVDELHASATKLVGLDDFGTDDDNYREALGVLLDAYQGEAGLTVLGSKMNRFFLRGALVARLLSQSAWKQYPEHVDVAIKRPIFVTGLVRTGTTALHRLLGADPAHQGLHMWLAEYPQPRPPRETWESNPLYRQLDADFTQHHAENPGYTGLHFMAAYELEECWQLLRQSLHSVSYEALAHVPSYADWLSRQDWTPSYCRHRRNLQLIGLNDAEKRWVLKNPSHLFALDALMATYPDALVVQTHRPVETIMASMCSLAQHTTEGWSTKFVGAQIGADAMDTWSRGLERFNAARAKYDSAQFYDVDYHDLIADPLGTVADIYRHFGLTLSDEARQAMTTVHAESQSGARAPKHSYSLADYGLTVEMVKERFAGL

>2O30A

MPSEAKYTWDQELNEINIQFPVTGDADSSAIKIRMVGKKICVKNQGEIVIDGELLHEVDVSSLWWVINGDVVDVNVTKKRNEWWDSLLVGSESVDVQKLAENKHADMSMLDAEAREVVEKMMHNTSGKDSE

>2CO3A

GSQKSVDIVFSSPQDLTVSLIPVSGLKAGKNAPSAKIAKLVVNSTTLKEFGVRGISNNVVDSTGTAWRVAGKNTGKEIGVGLSSDSLRRSDSTEKWNGVNWMTFNSNDTLDIVLTGPAQNVTADTYPITLDVVGYQP

>4GNRA

GSVEEKTIKIGFNFEESGSLAAYGTAEQKGAQLAVDEINAAGGIDGKQIEVVDKDNKSETAEAASVTTNLVTQSKVSAVVGPATSGATAAAVANATKAGVPLISPSATQDGLTKGQDYLFIGTFQDSFQGKIISNYVSEKLNAKKVVLYTDNASDYAKGIAKSFRESYKGEIVADETFVAGDTDFQAALTKMKGKDFDAIVVPGYYNEAGKIVNQARGMGIDKPIVGGDGFNGEEFVQQATAEKASNIYFISGFSTTVEVSAKAKAFLDAYRAKYNEEPSTFAALAYDSVHLVANAAKGAKNSGEIKDNLAKTKDFEGVTGQTSFDADHNTVKTAYMMTMNNGKVEAAEVVKP

>1GG4A

MISVTLSQLTDILNGELQGADITLDAVTTDTRKLTPGCLFVALKGERFDAHDFADQAKAGGAGALLVSRPLDIDLPQLIVKDTRLAFGELAAWVRQQVPARVVALTGSSGKTSVKEMTAAILSQCGNTLYTAGNLNNDIGVPMTLLRLTPEYDYAVIELGANHQGEIAWTVSLTRPEAALVNNLAAAHLEGFGSLAGVAKAKGEIFSGLPENGIAIMNADNNDWLNWQSVIGSRKVWRFSPNAANSDFTATNIHVTSHGTEFTLQTPTGSVDVLLPLPGRHNIANALAAAALSMSVGATLDAIKAGLANLKAVPGRLFPIQLAENQLLLDDSYNANVGSMTAAVQVLAEMPGYRVLVVGDMAELGAESEACHVQVGEAAKAAGIDRVLSVGKQSHAISTASGVGEHFADKTALITRLKLLIAEQQVITILVKGSRSAAMEEVVRALQENGTC

>1F00I

ASITEIKADKTTAVANGQDAITYTVKVMKGDKPVSNQEVTFTTTLGKLSNSTEKTDTNGYAKVTLTSTTPGKSLVSARVSDVAVDVKAPEVEFFTTLTIDDGNIEIVGTGVKGKLPTVWLQYGQVNLKASGGNGKYTWRSANPAIASVDASSGQVTLKEKGTTTISVISSDNQTATYTIATPNSLIVPNMSKRVTYNDAVNTCKNFGGKLPSSQNELENVFKAWGAANKYEYYKSSQTIISWVQQTAQDAKSGVASTYDLVKQNPLNNIKASESNAYATCVK

>2FD6U

SLRCMQCKTNGDCRVEECALGQDLCRTTIVRLWEEGEELELVEKSCTHSEKTNRTLSYRTGLKITSLTEVVCGLDLCNQGNSGRAVTYSRSRYLECISCGSSDMSCERGRHQSLQCRSPEEQCLDVVTHWIQEGEEGRPKDDRHLRGCGYLPGCPGSNGFHNNDTFHFLKCCNTTKCNEGPILELENLPQNGRQCYSCKGNSTHGCSSEETFLIDCRGPMNQCLVATGTHEPKNQSYMVRGCATASMCQHAHLGDAFSMNHIDVSCCTKSGCNHPD

>2VPBB

AMAAKVVYVFSTEMANKAAEAVLKGQVETIVSFHI

>3VENA

HHHHHHMRVLGLNGWPRDFHDASAALLVDGRIAAFAEEERLTRKKHGYNTAPVQAAAFCLAQAGLTVDDLDAVAFGWDLPAMYRERLGGWPHSDSEALDILLPRDVFPRRTDPPLHFVQHHLAHAASAYYFSGEDRGAVLIVDGQGEEECVTLAHAEGGKITVLDTVPGAWSLGFFYEHVSEYTGLGGDNPGKLMGLAAHGTTVDETLSAFAFDSDGYRLNLIDPQARDPEDWDEYSVTERAWFAHLERIYRLPPNEFVRRYDPAKGRVVRDTRRDPYEYRDLAATAQAALERAVFGLADSVLARTGERTLFVAGGVGLNATMNGKLLTRSTVDKMFVPPVASDIGVSLGAAAAVAVELGDRIAPMGDTAAWGPEFSPDQVRAALDRTGLAYREPANLEREVAALIASGKVVGWAQGRGEVGPRALGQRSLLGSAHSPTMRDHINLRVKDREWWRPFAPSMLRSVSDQVLEVDADFPYMIMTTKVRAAYAERLPSVVHEDWSTRPQTVTEASNPRYHRMLTELGDLVGDPVCLNTSFNDRGEPIVSSPADALLTFSRLPIDALAVGPYLVTKDLRH

>3VL1A

GSHMTKTITVAHIQYDFKAVLEENDENDDEFYINVDKNLNEIKEHKIVVLGNSRGVDAGKGNTFEKVGSHLYKARLDGHDFLFNTIIRDGSKMLKRADYTAVDTAKLQMRRFILGTTEGDIKVLDSNFNLQREIDQAHVSEITKLKFFPSGEALISSSQDMQLKIWSVKDGSNPRTLIGHRATVTDIAIIDRGRNVLSASLDGTIRLWECGTGTTIHTFNRKENPHDGVNSIALFVGTDRQLHEISTSKKNNLEFGTYGKYVIAGHVSGVITVHNVFSKEQTIQLPSKFTCSCNSLTVDGNNANYIYAGYENGMLAQWDLRSPECPVGEFLINEGTPINNVYFAAGALFVSSGFDTSIKLDIISDPESERPAIEFETPTFLVSNDDAVSQFCYVSDDESNGEVLEVGKNNFCALYNLSNP

>2O34A

SLPMQHVHTSPVRDYRNRCARREGETVFQVVVEETDLRVTALAELATPMAAYVGELRAQLKVWMEFQPAFRHSLVPVEVPEGAPEVVRRMAHGARLVGVGPFAAVAGTIAQMVAERFVDVSPELIVENGGDLYLYSERDRVVGILPDPASGDMVGILVRAGTAPVSLCGSSARIGHSLSLGDGDLAVVRARDASLADAAATAFGNMLRRADDVAAVTERAAQLASIGIEGVYAQCGGRIGIWGDMELAVA

>2R8EA

MSKAGASLATCYGPVSADVMAKAENIRLLILDVDGVLSDGLIYMGNNGEELKAFNVRDGYGIRCALTSDIEVAIITGRKAKLVEDRCATLGITHLYQGQSNKLIAFSDLLEKLAIAPENVAYVGDDLIDWPVMEKVGLSVAVADAHPLLIPRADYVTRIAGGRGAVREVCDLLLLAQGKLDEAKGQSI

>1KQFA

MDVSRRQFFKICAGGMAGTTVAALGFAPKQALAQARNYKLLRAKEIRNTCTYCSVGCGLLMYSLGDGAKNAREAIYHIEGDPDHPVSRGALCPKGAGLLDYVNSENRLRYPEYRAPGSDKWQRISWEEAFSRIAKLMKADRDANFIEKNEQGVTVNRWLSTGMLCASGASNETGMLTQKFARSLGMLAVDNQARVCHGPTVASLAPTFGRGAMTNHWVDIKNANVVMVMGGNAAEAHPVGFRWAMEAKNNNDATLIVVDPRFTRTASVADIYAPIRSGTDITFLSGVLRYLIENNKINAEYVKHYTNASLLVRDDFAFEDGLFSGYDAEKRQYDKSSWNYQLDENGYAKRDETLTHPRCVWNLLKEHVSRYTPDVVENICGTPKADFLKVCEVLASTSAPDRTTTFLYALGWTQHTVGAQNIRTMAMIQLLLGNMGMAGGGVNALRGHSNIQGLTDLGLLSTSLPGYLTLPSEKQVDLQSYLEANTPKATLADQVNYWSNYPKFFVSLMKSFYGDAAQKENNWGYDWLPKWDQTYDVIKYFNMMDEGKVTGYFCQGFNPVASFPDKNKVVSCLSKLKYMVVIDPLVTETSTFWQNHGESNDVDPASIQTEVFRLPSTCFAEEDGSIANSGRWLQWHWKGQDAPGEARNDGEILAGIYHHLRELYQSEGGKGVEPLMKMSWNYKQPHEPQSDEVAKENNGYALEDLYDANGVLIAKKGQLLSSFAHLRDDGTTASSCWIYTGSWTEQGNQMANRDNSDPSGLGNTLGWAWAWPLNRRVLYNRASADINGKPWDPKRMLIQWNGSKWTGNDIPDFGNAAPGTPTGPFIMQPEGMGRLFAINKMAEGPFPEHYEPIETPLGTNPLHPNVVSNPVVRLYEQDALRMGKKEQFPYVGTTYRLTEHFHTWTKHALLNAIAQPEQFVEISETLAAAKGINNGDRVTVSSKRGFIRAVAVVTRRLKPLNVNGQQVETVGIPIHWGFEGVARKGYIANTLTPNVGDANSQTPEYKAFLVNIEKA

>3SJRA

SNAMVMDDDITVQPIRGVQPRPAGSHEPFAVPSRAGQHGKRPDGEDSADISLSQGAQAAALLFSAAMDQISRLAELDIEPVRLPESELTGDSHSQHLLLGMEILMELYRQQHPDWTAPAIRQAFAPLARAGLERGYQEACQVLRQLNVYTPAVAGQLQGLLLLTQRLFEERLQIA

>3DNZA

ITGTSTVGVGRGVLGDQKNINTTYSTYYYLQDNTRGNGIFTYDAKYRTTLPGSLWADADNQFFASYDAPAVDAHYYAGVTYDYYKNVHNRLSYDGNNAAIRSSVHYSQGYNNAFWNGSQMVYGDGDGQTFIPLSGGIDVVAHELTHAVTDYTAGLIYQNESGAINEAISDIFGTLVEFYANKNPDWEIGEDVYTPGISGDSLRSMSDPAKYGDPDHYSKRYTGTQDNGGVHINSGIINKAAYLISQGGTHYGVSVVGIGRDKLGKIFYRALTQYLTPTSNFSQLRAAAVQSATDLYGSTSQEVASVKQAFDAVGVK

>3CWRA

GMVEQRNRGRPAVPDAVVRESIVGAAQRLLSSGGAAAMTMEGVASEAGIAKKTLYRFASGRADLIGLLVESWIAPIFPGFEADPQDAAAALERIVYDIAQAVLSREAVSLFRMLASDADLRNRFLPAYNANGIERSRRELARWLDQQASAGRLPLPIPAERVADLLLSAVIAEPLRQITLGLREPLPAWDIAPRVADAVRLIAPGRER

>1QOYA

GSPGISGGGGGILDSMAEIVADKTVEVVKNAIETADGALDLYNKYLDQVIPWQTFDETIKELSRFKQEYSQAASVLVGDIKTLLMDSQDKYFEATQTVYEWCGVATQLLAAYILLFDEYNEKKASAQKDILIKVLDDGITKLNEAQKSLLVSSQSFNNASGKLLALDSQLTNDFSEKSSYFQSQVDKIRKEAYAGAAAGVVVGPFGLIISYSIAAGVVEGKLIPELKNKLKSVQNFFTTLSNTVKQANKDIDAAKLKLTTEIAAIGEIKTETETTRFYVDYDDLMLSLLKEAAKKMINTCNEYQKRHGKKTLFEVPEV

>2H1VA

MSRKKMGLLVMAYGTPYKEEDIERYYTHIRRGRKPEPEMLQDLKDRYEAIGGISPLAQITEQQAHNLEQHLNEIQDEITFKAYIGLAHIEPFIEDAVAEMHKDGITEAVSIVLAPHFSTFSVQSYNKRAKEEAEKLGGLTITSVESWYDEPKFVTYWVDRVKETYASMPEDERENAMLIVSAHSLPEKIKEFGDPYPDQLHESAKLIAEGAGVSEYAVGWQSEGNTPDPWLGPDVQDLTRDLFEQKGYQAFVYVPVGFVADHLEVLYDNDYECKVVTDDIGASYYRPEMPNAKPEFIDALATVVLKKLGR

>3RUIA

GSDPLKIADQSVDLNLKLMKWRILPDLNLDIIKNTKVLLLGAGTLGCYVSRALIAWGVRKITFVDNGTVSYSNPVRQALYNFEDCGKPKAELAAASLKRIFPLMDATGVKLSIPMIGHKLVNEEAQHKDFDRLRALIKEHDIIFLLVDSRESRWLPSLLSNIENKTVINAALGFDSYLVMRHGNRDEQSSKQLGCYFCHDVVAPTDSLTDRTLDQMSTVTRPGVAMMASSLAVELMTSLLQTKYSGSETTVLGDIPHQIRGFLHNFSILKLETPAYEHCPACSPKVIEAFTDLGWEFVKKALEHPLYLEEISGLSVIKQEVERLGNDVFEWEDDESDEIA

>3A9LA

MAQTDTYPNIEALENAETVGVAYNIEVKRQNPSMIYFSPHAGGIEVGTTELIYRVVELTGGSLYLFQGLLPSGNSRLHVTSTHFDEPMAVCMLSKHTDAVSFHGYKDDYNKNTLVGGLNTELRNLIVSKLNSKGIAAEVATDRFTATDPDNIVNRCASGKGVQLEISSAQRRAFFQNNDWSKANRGNVTQEFLDYAEAIKEAEAEYYGLEHHHHHH

>1JB0A

MTISPPEREPKVRVVVDNDPVPTSFEKWAKPGHFDRTLARGPQTTTWIWNLHALAHDFDTHTSDLEDISRKIFSAHFGHLAVVFIWLSGMYFHGAKFSNYEAWLADPTGIKPSAQVVWPIVGQGILNGDVGGGFHGIQITSGLFQLWRASGITNEFQLYCTAIGGLVMAGLMLFAGWFHYHKRAPKLEWFQNVESMLNHHLAGLLGLGSLAWAGHQIHVSLPINKLLDAGVAAKDIPLPHEFILNPSLMAELYPKVDWGFFSGVIPFFTFNWAAYSDFLTFNGGLNPVTGGLWLSDTAHHHLAIAVLFIIAGHMYRTNWGIGHSLKEILEAHKGPFTGAGHKGLYEVLTTSWHAQLAINLAMMGSLSIIVAQHMYAMPPYPYLATDYPTQLSLFTHHMWIGGFLVVGGAAHGAIFMVRDYDPAMNQNNVLDRVLRHRDAIISHLNWVCIFLGFHSFGLYVHNDTMRAFGRPQDMFSDTGIQLQPVFAQWVQNLHTLAPGGTAPNAAATASVAFGGDVVAVGGKVAMMPIVLGTADFMVHHIHAFTIHVTVLILLKGVLFARSSRLIPDKANLGFRFPCDGPGRGGTCQVSGWDHVFLGLFWMYNCISVVIFHFSWKMQSDVWGTVAPDGTVSHITGGNFAQSAITINGWLRDFLWAQASQVIGSYGSALSAYGLLFLGAHFIWAFSLMFLFSGRGYWQELIESIVWAHNKLKVAPAIQPRALSIIQGRAVGVAHYLLGGIATTWAFFLARIISVG

>3MCRA

GMTSNGQQGKPNLPEKDNLPRELGTQRINSPIARMGMFGAKTTGDTSGYGRLRVYRHVPAAAQRPYSDPSDPRTAYFDEVADALERSLKEIGTPYDTAISRVVVDRGEITFHVQREHLLDVATRLRDDPALRFELCLGVTGVHYPEDEGNELHAVYALRSITHNYEIRLEVSCPDSDPHIPSIVSVYPTNDWHEREAWDFFGIIFDGHPALTR

>3DMBA

GMADPKELQDKFWKALKSDRTVMLGLDGVEDGHARPMTAQIEGDSGGPIWFFTSKDNALIAMLGQGRRVIGAFSSKGHDLFASISGSLREDTDPAVVDRLWNPYVAAWYEGGKDDPKLALLRLDADHAQIWLNGSSLLAGIKVLLGV

>2I0KA

STGPVAPLPTPPNFPNDIALFQQAYQNWSKEIMLDATWVCSPKTPQDVVRLANWAHEHDYKIRPRGAMAGWTPLTVEKGANVEKVILADTMTHLNGITVNTGGPVATVTAGAGASIEAIVTELQKHDLGWANLPAPGVLSIGGALAVNAHGAALPAVGQTTLPGHTYGSLSNLVTELTAVVWNGTTYALETYQRNDPRITPLLTNLGRCFLTSVTMQAGPNFRQRCQSYTDIPWRELFAPKGADGRTFEKFVAESGGAEAIWYPFTEKPWMKVWTVSPTKPDSSNEVGSLGSAGSLVGKPPQAREVSGPYNYIFSDNLPEPITDMIGAINAGNPGIAPLFGPAMYEITKLGLAATNANDIWGWSKDVQFYIKATTLRLTEGGGAVVTSRANIATVINDFTEWFHERIEFYRAKGEFPLNGPVEIRCCGLDQAADVKVPSVGPPTISATRPRPDHPDWDVAIWLNVLGVPGTPGMFEFYREMEQWMRSHYNNDDATFRPEWSKGWAFGPDPYTDNDIVTNKMRATYIEGVPTTENWDTARARYNQIDPHRVFTNGFMDKLLP

>3Q64A

MTERSVVHSTFIIERLYPAPPSKVFFALGNADAKRRWFTDPDNPMPGRFEMDFRVGGKEVNAGGPKDGPIHVYTATYQDIVPDQRIVYSYDMLFGETRISVSLATIQLFAEGEGTRLVLTEQGAFLDGHDTPSTREHGTGVLLDLLDAFLDKTTLEHHHHHH

>1XKPA

XQFRGESVQIVSGTLQSIADMAEEVTFVFSERKELSLDKRKLSDSQARVSDVEEQVNQYLSKVPELEQKQNVSELLSLLSNSPNISLSQLKAYLEGKSEEPSEQFKMLCGLRDALKGRPELAHLSHLVEQALVSMAEEQGETIVLGARITPEAYRESQSGVNPLQPLRDTYRDAVMGYQGIYAIWSDLQKRFPNGDIDSVILFLQKALSADLQSQQSGSGREKLGIVISDLQKLKEFGSVSDQVKG

>3TIPA

GPHMIAPGHRDEFDPKLPTGEKEEVPGKPGIKNPETGDVVRPPVDSVTKYGPVKGDSIVEKEEIPFEKERKFNPDLAPGTEKVTREGQKGEKTITTPTLKNPLTGEIISKGESKEEITKDPINELTEYGPET

>1KPTA

LGINCRGSSQCGLSGGNLMVRIRDQACGNQGQTWCPGERRAKVCGTGNSISAYVQSTNNCISGTEACRHLTNLVNHGCRVCGSDPLYAGNDVSRGQLTVNYVNSC

>4EBJA

MGSSHHHHHHSSGRENLYFQGVQHTIARWVDRLREEYADAVAILLKGSYARGDAATWSDIDFDVLVSTQDVEDYRTWIEPVGDRLVHISAAVEWVTGWERDTVDPSSWSYGLPTQETTRLMWAINDETRRRLDRPYKTHPAAEPEVEDTVEALGKIRNAIARGDDLGVYQSAQTVAKLVPTLLIPINPPVTVSHARQAIEAILAFPRVPVGFAADWLTCLGLVEERSARSTAAAAERMVRGVLEMLPTDPDLLGEDIARLMNAGLLEKYVQQ

>1XKPA

XQFRGESVQIVSGTLQSIADMAEEVTFVFSERKELSLDKRKLSDSQARVSDVEEQVNQYLSKVPELEQKQNVSELLSLLSNSPNISLSQLKAYLEGKSEEPSEQFKMLCGLRDALKGRPELAHLSHLVEQALVSMAEEQGETIVLGARITPEAYRESQSGVNPLQPLRDTYRDAVMGYQGIYAIWSDLQKRFPNGDIDSVILFLQKALSADLQSQQSGSGREKLGIVISDLQKLKEFGSVSDQVKG

>2XDGA

SMLREDESACLQAAEEMPQTTLGCPATWDGLLCWPTAGSGEWVTLPCPDFFSHFSSESGAVKRDCTITGWSEPFPPYPVACPVPLELLAEEE

>1LXJA

MPKIFCLADVCMVPIGTDSASISDFVALIEKKIRESPLKSTLHSAGTTIEGPWDDVMGLIGEIHEYGHEKGYVRVHTDIRVGTRTDKHQTAQDKIDVVLKKISQ

>3TWDA

ASRLERVYQSEQAEKLLLAGVMLRDPARFDLRGTLTHGRDVEIDTNVIIEGNVTLGHRVKIGTGCVIKNSVIGDDCEISPYTVVEDANLAAACTIGPFARLRPGAELLEGAHVGNFVEMKKARLGKGSKAGHLTYLGDAEIGDNVNIGAGTITCNYDGANKFKTIIGDDVFVGSDTQLVAPVTVGKGATIAAGTTVTRNVGENALAISRVPQTQKEGWRRPA

>2AUKA

GSHMAAAESSIQVKNKGSIKLSNVKSVVNSSGKLVITSRNTELKLIDEFGRTKESYKVPYGAVLAKGDGEQVAGGETVANWDPHTMPVITEVSGFVRFTDMIDGQTITRQTDELTGLSSLVVLDSAERTAGGKDLRPALKIVDAQGNDVLIPGTDMPAQYFLPGKAIVQLEDGVQISSGDTLARIPQESG

>3KF8B

SSKIILIPSNIPQEFPEASISNPERLRILAQVKDFIPHESTIVIDKVPTITSEQSTYINICIFNLLEACSSRVLVPGTLVNIDAFYDGESINPVDIYEVNGANFTMENIQLIDEMNNSIGKFN

>4F9ZA

SSDGPGAAQEPTWLTDVPAAMEFIAATEVAVIGFFQDLEIPAVPILHSMVQKFPGVSFGISTDSEVLTHYNITGNTICLFRLVDNEQLNLEDEDIESIDATKLSRFIEINSLHMVTEYNPVTVIGLFNSVIQIHLLLIMNKASPEYEENMHRYQKAAKLFQGKILFILVDSGMKENGKVISFFKLKESQLPALAIYQTLDDEWDTLPTAEVSVEHVQNFCDGFLSGK

>3VGPA

GPDLTEDWKEALEWMRTSLEEQNYLNPYEKPEYSVMSWWDYGNWILYVSKKAVVANNFQAGAVDAAKFFTAKSEDEAIKIAKKRGVRYVVTADEITMKDANNTKFPAIMRIAGYNVDLMTEGEILNFFNHTVLYRLHMENAENLTHFRLVKEFGDVKIFEVVGS

>2VN6A

TVLPKDIPGDSLKVTVGTANGKPGDTVTVPVTFADVAKMKNVGTCNFYLGYDASLLEVVSVDAGPIVKNAAVNFSSSASNGTISFLFLDNTITDELITADGVFANIKFKLKSVTAKTTTPVTFKDGGAFGDGTMSKIASVTKTNGSVTIDP

>4GEYA

ANVRLQHHHHHHHLEAEAFSSESKWMTGDWGGTRTELLDKGYDFTLDYVGEVAGNLHGGYNDDKTARYSDQFALGAHLDLQKILGWHDAEFKLAITERSGRNLSNDRISDPRAGQFSSVQEVWGRGQTWRLTQMWIKQKYFDGALDVKFGRFGEGEDFNSFPCDFQNLAFCGSQVGNWVGGIWYNWPVSQWALRVKYNITPAFFVQVGAFEQNPSNLETGNGFKLSGSGTKGAIMPMEAVWSPKVNGLPGEYRLGYYYSTAKADDVYDDVNGNPQALTGEAFKSHSSKHGWWVVAQQQVTAHGGDVNRGLSLFANFTVHDKATNVVDNYQQVGLVYKGAFDARPKDDIGFGVARIHVNDDVKKRAELLNAQSGINDYDNPGFVPLQRTEYNAELYYGFHVTNWLTVRPNLQYIKSPGGVDEVDNALVAGLKIQSSF

>4GB5A

SNAMDAETDRAEIIELFGRYADIADLKEFTDLPRRVHTDPLTIDFESVTGMPPMTVPLSDYGAALRASFGAFSATHHAITGHVVTIDSDRATIHAHVRAEHWLPAEVAGDGPDRWLVVGFYDNEAVRTADGWRLSSVKLTASYQENAHLARAAAAGQAG

>3LDCA

VPATRILLLVLAVIIYGTAGFHFIEGESWTVSLYWTFVTIATVGYGDYSPHTPLGMYFTCTLIVLGIGTFAVAVERLLEFLI

>2R6JA

SGHGMEENGMKSKILIFGGTGYIGNHMVKGSLKLGHPTYVFTRPNSSKTTLLDEFQSLGAIIVKGELDEHEKLVELMKKVDVVISALAFPQILDQFKILEAIKVAGNIKRFLPSDFGVEEDRINALPPFEALIERKRMIRRAIEEANIPYTYVSANCFASYFINYLLRPYDPKDEITVYGTGEAKFAMNYEQDIGLYTIKVATDPRALNRVVIYRPSTNIITQLELISRWEKKIGKKFKKIHVPEEEIVALTKELPEPENIPIAILHCLFIDGATMSYDFKENDVEASTLYPELKFTTIDELLDIFVHDPPPPASAAF

>4DN7A

MHHHHHHSSGVDLGTENLYFQSMQTEQVSLKKRAESAAEKKAAFGEDFELEKYEEGSKVSKPIEDLQSLDEESKKTLLQVGVIPSEEGRSGSFLVLDNAVSHSTLKDKNVELMSTHKAMEKYEWLKDYSWKLVQVDADKYTAKTYLEDADGYFIRVPAGKKTSMPVQTCLMLGSKKAAQTVHNIIIVEEGATLDIITGCTTKKGVEEGLHLGISEMYIKKGGTLNFTMIHNWAEQIGVRPRTVVSVEEGGTYVSNYICLKPVRSVQTYPTVRLEGEGAVTRLNTIAIAHPGSELDLGSKAIFNAPGTRAELISRTITIGGRLIARGEMIGNAKGAKGHLECKGLVLTDKGSQLAIPILEANVDDIELTHEAAVGKIAKDQVEYLMARGLTEDEAVGMIIRGFLDVGIRGIPEELKEEIENTIAQTALGM

>2DWKA

GSSGSSGMANERMNLMNMAKLSIKGLIESALNLGRTLDSDYAPLQQFFVVMEHCLKHGLKAKKTFLGQNKSFWGPLELVEKLVPEAAEITASVKDLPGLKTPVGRGRAWLRLALMQKKLSEYMKALINKKELLSEFYEVNALMMEEEGAIIAGLLVGLNVIDANFCMKGEDLDSQVGVID

>3B5OA

GMEFNHLTKQLNQLLAQDYVAFSITENPVVQMLSQASFAQIAYVMQQYSIFPKELVGFTELARRKALGAGWNGVAQELQENIDEEMGSTTGGISHYTLLADGLEEGLGVAVKNTMPSVATSKLLRTVLSLFDRQVDYVLGATYAIEATSIPELTLIVKLVEWLHEGAIPKDLQYFFSKHLDEWEIEHEAGLRTSVAAYIQPEEFGEFAAGFRAMIDAMQVWWQELAQEAISSEVVLSTAIAQHH

>3B34A

MGSSHHHHHHSSGENLYFQGHMTQQPQAKYRHDYRAPDYQITDIDLTFDLDAQKTVVTAVSQAVRHGASDAPLRLNGEDLKLVSVHINDEPWTAWKEEEGALVISNLPERFTLKIINEISPAANTALEGLYQSGDALCTQCEAEGFRHITYYLDRPDVLARFTTKIIADKIKYPFLLSNGNRVAQGELENGRHWVQWQDPFPKPCYLFALVAGDFDVLRDTFTTRSGREVALELYVDRGNLDRAPWAMTSLKNSMKWDEERFGLEYDLDIYMIVAVDFFNMGAMENKGLNIFNSKYVLARTDTATDKDYLDIERVIGHEYFHNWTGNRVTCRDWFQLSLKEGLTVFRDQEFSSDLGSRAVNRINNVRTMRGLQFAEDASPMAHPIRPDMVIEMNNFYTLTVYEKGAEVIRMIHTLLGEENFQKGMQLYFERHDGSAATCDDFVQAMEDASNVDLSHFRRWYSQSGTPIVTVKDDYNPETEQYTLTISQRTPATPDQAEKQPLHIPFAIELYDNEGKVIPLQKGGHPVNSVLNVTQAEQTFVFDNVYFQPVPALLCEFSAPVKLEYKWSDQQLTFLMRHARNDFSRWDAAQSLLATYIKLNVARHQQGQPLSLPVHVADAFRAVLLDEKIDPALAAEILTLPSVNEMAELFDIIDPIAIAEVREALTRTLATELADELLAIYNANYQSEYRVEHEDIAKRTLRNACLRFLAFGETHLADVLVSKQFHEANNMTDALAALSAAVAAQLPCRDALMQEYDDKWHQNGLVMDKWFILQATSPAANVLETVRGLLQHRSFTMSNPNRIRSLIGAFAGSNPAAFHAEDGSGYLFLVEMLTDLNSRNPQVASRLIEPLIRLKRYDAKRQEKMRAALEQLKGLENLSGDLYEKITKALA

>3DMAA

MLTKVIAQAHIDHFTKWFERADKIVIVSHVSPDGDAIGSSLGLYHFLDSQDKIVNVIVPNAFPDFLKWMPGSKDILLYDRYQEFADKLIMEADVICCLDFNALKRIDEMSDIVAASPGRKIMIDHHLYPEDFCRITISHPEISSTSELVFRLICRMGYFSDISKEGAECIYTGMMTDTGGFTYNSNNREIYFIISELLSKGIDKDDIYRKVYNTYSESRLRLMGYVLSNMKVYKDYNSALISLTKEEQGKFDYIKGDSEGFVNIPLSIKNVCFSCFLREDTEKKMIKISLRSVGKFPCNRLAAEFFNGGGHLNASGGEFYGTMEEAVKVFEQALEKYKPLLKE

>1O75A

CGSSSHETSYGYATLSYADYWAGELGQSRDVLLAGNAEADRAGDLDAGMFDAVSRATHGHGAFRQQFQYAVEVLGEKVLSKQETEDSRGRKKWEYETDPSVTKMVRASASFQDLGEDGEIKFEAVEGAVALADRASSFMVDSEEYKITNVKVHGMKFVPVAVPHELKGIAKEKFHFVEDSRVTENTNGLKTMLTEDSFSARKVSSMESPHDLVVDTVGTGYHSRFGSDAEASVMLKRADGSELSHREFIDYVMNFNTVRYDYYGDDASYTNLMASYGTKHSADSWWKTGRVPRISCGINYGFDRFKGSGPGYYRLTLIANGYRDVVADVRFLPKYEGNIDIGLKGKVLTIGGADAETLMDAAVDVFADGQPKLVSDQAVSLGQNVLSADFTPGTEYTVEVRFKEFGSVRAKVVAQ

>2H1VA

MSRKKMGLLVMAYGTPYKEEDIERYYTHIRRGRKPEPEMLQDLKDRYEAIGGISPLAQITEQQAHNLEQHLNEIQDEITFKAYIGLAHIEPFIEDAVAEMHKDGITEAVSIVLAPHFSTFSVQSYNKRAKEEAEKLGGLTITSVESWYDEPKFVTYWVDRVKETYASMPEDERENAMLIVSAHSLPEKIKEFGDPYPDQLHESAKLIAEGAGVSEYAVGWQSEGNTPDPWLGPDVQDLTRDLFEQKGYQAFVYVPVGFVADHLEVLYDNDYECKVVTDDIGASYYRPEMPNAKPEFIDALATVVLKKLGR

>3P9ZA

SNAMREIVWVHSQRIAPYKTLILNEFCYYPLELDPTPFNALIFTSKNAVFSLLETLKNSPKLKMLQNIPAYALSEPTAKTLQDHHFKVAFMGEKAHGKEFVQEIFPLLEKKSVLYLRAKEIVSSLDTILLEHGIDFKQAVVYENKLKHLTLSEQNALKPKEKSILIFTAISHAKAFLHYFEFLENYTAISIGNTTALYLQEQGIPSYIAKKPSLEACLELALSLRIKEC

>3QHOA

MEGNTILKIVLICTILAGLFGQVVPVYAENTTYQTPTGIYYEVRGDTIYMINVTSGEETPIHLFGVNWFGFETPNHVVHGLWKRNWEDMLLQIKSLGFNAIRLPFCTESVKPGTQPIGIDYSKNPDLRGLDSLQIMEKIIKKAGDLGIFVLLDYHRIGCTHIEPLWYTEDFSEEDFINTWIEVAKRFGKYWNVIGADLKNEPHSVTSPPAAYTDGTGATWGMGNPATDWNLAAERIGKAILKVAPHWLIFVEGTQFTNPKTDSSYKWGYNAWWGGNLMAVKDYPVNLPRNKLVYSPHVFGPDVYNQPYFGPAKGFPDNLPDIWYHHFGYVKLELGYSVVIGEFGGKYGHGGDPRDVIWQNKLVDWMIENKFCDFFYWSWNPDSGDTGGILQDDWTTIWEDKYNNLKRLMDSCSKSSSSTQSVIRSTTPTKSNTSKKICGPAILIILAVFSLLLRRAPR

>3DMYA

MQQLEEALKQLAQGSGSSQALTQVRRWDSACQKLPDANLALISVAGEYAAELANQALDRNLNVMMFSDNVTLEDEIQLKTRAREKGLLVMGPDCGTSMIAGTPLAFANVMPEGNIGVIGASGTGIQELCSQIALAGEGITHAIGLGGRDLSREVGGISALTALEMLSADEKSEVLAFVSKPPAEAVRLKIVNAMKATGKPTVALFLGYTPAVARDENVWFASSLDEAARLACLLSRVTARRNAIAPVSSGFICGLYTGGTLAAEAAGLLAGHLGVEADDTHQHGMMLDADSHQIIDLGDDFYTVGRPHPMIDPTLRNQLIADLGAKPQVRVLLLDVVIGFGATADPAASLVSAWQKACAARLDNQPLYAIATVTGTERDPQCRSQQIATLEDAGIAVVSSLPEATLLAAALIHPLSPAAQQHTPSLLENVAVINIGLRSFALELQSASKPVVHYQWSPVAGGNKKLARLLERLQGHPHHH

>3AHNA

MKFSEFRYERPNIEKLKASFQQALQSFQKASNAEEQNEAMKEINQLRNDFSTMAQICYIRHTIDTNDEFYKQEQDFFDEVEPIVKGLVNDYYRALVSSPFRSQLEGKWGKQLFALAEAELKTYSPDIVEDLQLENKLTSEYTKLVASAKIFFEGEERTLAQLQPFVESPDRDMRKRASEARFTFFQEHEEKFDEIYDQLVKVRTAIAQKLGFKNFVELGYARLGRTDYNAEMVAKFRKQVEKHIVPIAVKLRERQRERIGVEKLKYYDEAFVFPTGNPMPKGDANWIIENGKKMYEELSPETGEFFRYMIEHELMDLVAKKGKASGGYCTYIENYKAPFIFSNFTGTSGDIDVLTHEAGHAFQVYESRHYEIPEYNWPTLEACEIHSMSMEFFTWPWMKLFFKEDAEKYQFYHLSDALLFLPYGVAVDEFQHFVYENPNATPAERKQAWRAIERKYMPTKDYDGNDYLERGGFWQRQSHIYTTAFYYIDYTLAQICAFQFWKRSRENYKEAWNDYLTLCRQGGSKPFTELVRVANLISPFEDGCVQSVVGGIEGWLNSVDDQSL

>3CP7A

QNPADSPHIGKVFFSTNQGDFVCSANIVASANQSTVATAGHCLHDGNGGQFARNFVFAPAYDYGESEHGVWAAEELVTSAEWANRGDFEHDYAFAVLETKGGTTVQQQVGTASPIAFNQPRGQYYSAYGYPAAAPFNGQELHSCHGTATNDPMGSSTQGIPCNMTGGSSGGPWFLGNGTGGAQNSTNSYGYTFLPNVMFGPYFGSGAQQNYNYASTTN

>4A57A

MTDSSSLRGVDADTEKRINVGKKHLQTLRNLETRCHDSLQALVVIDAGSSSTRTNVFLAKTRSCPNKGRSIDPDSIQLIGAGKRFAGLRVVLEEWLDTYAGKDWESRPVDARLLFQYVPQMHEGAKKLMQLLEEDTVAILDSQLNEKQKVQVKALGIPVMLCSTAGVRDFHEWYRDALFVLLRHLINNPSPAHGYKFFTNPFWTRPITGAEEGLFAFITLNHLSRRLGEDPARCMIDEYGVKQCRNDLAGVVEVGGASAQIVFPLQEGTVLPSSVRAVNLQRERLLPERYPSADVVSVSFMQLGMASSAGLFLKELCSNDEFLQGGICSNPCLFKGFQQSCSAGEVEVRPDGSASVNEDVRKNRLKPLATYCSVNNPEISFKVTNEMQCRENSIDPTKPLAERMKIENCSIIKGTGNFDKCVSQVESILVAPKLPLPANIEAASSGFESVDQVFRFASSTAPMIVTGGGMLAAINTLKDHRLLRSDFSGDVEELAEAAREFCSSEVIIRTDGPVIQLPNARGEQKLNSLNFDLCKTMALTVSLLRHMAAGENQPSFIKWEKSIAGPDGKPLADLGWQVGVILHHVLFTEEWGRNAYEAGYSHNLEHHHHHH

>1Y43B

QSEEYCASAWVGIDGDTCETAILQTGVDFCYEDGQTSYDAWYEWYPDYAYDFSDITISEGDSIKVTVEATSKSSGSATVENLTTGQSVTHTFSGNVEGDLCETNAEWIVEDFESGDSLVAFADFGSVTFTNAEATSGGSTVGPSDATVMDIEQDGSVLTETSVSGDSVTVTYV

>3D1PA

MWKAVMNAWNGTESQSKNVSNIQSYSFEDMKRIVGKHDPNVVLVDVREPSEYSIVHIPASINVPYRSHPDAFALDPLEFEKQIGIPKPDSAKELIFYCASGKRGGEAQKVASSHGYSNTSLYPGSMNDWVSHGGDKLDL

>2O1QA

GMLKSKIKEEYVQMDQVDWKPFPAAFSTGGIRWKLLHVSPEMGSWTAIFDCPAGSSFAAHVHVGPGEYFLTKGKMDVRGGKAAGGDTAIAPGYGYESANARHDKTEFPVASEFYMSFLGPLTFVKPDGSPIAVIGWEDAQGAWAA

>1Z67A

SNAMGLFDEVVGAFLKGDAGKYQAILSWVEEQGGIQVLLEKLQSGGLGAILSTWLSNQQRNQSVSGEQLESALGTNAVSDLGQKLGVDTSTASSLLAEQLPKIIDALSPQGEVSAQANNDLLSAGMELLKGKLFR

>3DZAA

GATDSATAAPAAAATTQVQKEAADVLQVAVQGANAMRDIQFARLALFHGQPDSAKKLTDDAAALLAADDASWAKFVKTDAKAKMIADRYVIINASIALSEDYVATPEKESAIQSANEKLAKGDQKGAIDTLRLAGIGVIENQYLMPLNQTRKAVAQSQELLKAGKYYEANLVLKGAEEGIVVDSEMLVAGN

>2H21A

SLSPAVQTFWKWLQEEGVITAKTPVKASVVTEGLGLVALKDISRNDVILQVPKRLWINPDAVAASEIGRVCSELKPWLSVILFLIRERSREDSVWKHYFGILPQETDSTIYWSEEELQELQGSQLLKTTVSVKEYVKNECLKLEQEIILPNKRLFPDPVTLDDFFWAFGILRSRAFSRLRNENLVVVPMADLINHSAGVTTEDHAYEVKGAAGLFSWDYLFSLKSPLSVKAGEQVYIQYDLNKSNAELALDYGFIEPNENRHAYTLTLEISESDPFFDDKLDVAESNGFAQTAYFDIFYNRTLPPGLLPYLRLVALGGTDAFLLESLFRDTIWGHLELSVSRDNEELLCKAVREACKSALAGYHTTIEQDRELKEGNLDSRLAIAVGIREGEKMVLQQIDGIFEQKELELDQLEYYQERRLKDLGLCGENGDILENLYFQ

>1RWRA

QGLVPQGQTQVLQGGNKVPVVNIADPNSGGVSHNKFQQFNVANPGVVFNNGLTDGVSRIGGALTKNPNLTRQASAILAEVTDTSPSRLAGTLEVYGKGADLIIANPNGISVNGLSTLNASNLTLTTGRPSVNGGRIGLDVQQGTVTIERGGVNATGLGYFDVVARLVKLQGAVSSKQGKPLADIAVVAGANRYDHATRRATPIAAGARGAAAGAYAIDGTAAGAMYGKHITLVSSDSGLGVRQLGSLSSPSAITVSSQGEIALGDATVQRGPLSLKGAGVVSAGKLASGGGAVNVAGGGAV

>2OAJA

NKNKIFSLAETNKYGMSSKPIAAAFDFTQNLLAIATVTGEVHIYGQQQVEVVIKLEDRSAIKEMRFVKGIYLVVINAKDTVYVLSLYSQKVLTTVFVPGKITSIDTDASLDWMLIGLQNGSMIVYDIDRDQLSSFKLDNLQKSSFFPAARLSPIVSIQWNPRDIGTVLISYEYVTLTYSLVENEIKQSFIYELPPFAPGGDFSEKTNEKRTPKVIQSLYHPNSLHIITIHEDNSLVFWDANSGHMIMARTVFETEINVPQPDYIRDSSTNAAKISKVYWMCENNPEYTSLLISHKSISRGDNQSLTMIDLGYTPRYSITSYEGMKNYYANPKQMKIFPLPTNVPIVNILPIPRQSPYFAGCHNPGLILLILGNGEIETMLYPSGIFTDKASLFPQNLSWLRPLATTSMAASVPNKLWLGALSAAQNKDYLLKGGVRTKRQKLPAEYGTAFITGHSNGSVRIYDASHGDIQDNASFEVNLSRTLNKAKELAVDKISFAAETLELAVSIETGDVVLFKYEVNQFYSVENRPESGDLEMNFRRFSLNNTNGVLVDVRDRAPTGVRQGFMPSTAVHANKGKTSAINNSNIGFVGIAYAAGSLMLIDRRGPAIIYMENIREISGAQSACVTCIEFVIMEYGDDGYSSILMVCGTDMGEVITYKILPASGGKFDVQLMDITNVTSKGPIHKIDAFSKETKSSCLATIPKMQNLSKGLCIPGIVLITGFDDIRLITLGKSKSTHKGFKYPLAATGLSYISTVEKNNDRKNLTVIITLEINGHLRVFTIPDFKEQMSEHIPFPIAAKYITESSVLRNGDIAIRVSEFQASLFSTVKEQDTLAPVSDTLYINGIRIPYRPQVNSLQWARGTVYCTPAQLNELLGGVNRPASKYKESIIAEGSFSERSSDDN

>3OXPA

NSAMLKTLLTSDVIQVVSQAKDWRDAIAISCQPLIDNGAVEARYVEAIYRSHEAIGPYYVVGPGIAMPHARPEDGVNRLSLALTVITEGVTFNAEGNDPVKLLIVLAATDSNSHIEAISQLAQLFDTASDVQALLNAKTPQDILSVIARY

>4JN3A

GMSENSSVRHGLTSAQHEVWLAQQLDPRGAHYRTGSCLEIDGPLDHAVLSRALRLTVAGTETLCSRFLTDEEGRPYRAYCPPAPEGSAAVEDPDGVPYTPVLLRHIDLSGHEDPEGEAQRWMDRDRATPLPLDRPGLSSHALFTLGGGRHLYYLGVHHIVIDGTSMALFYERLAEVYRALRDGRAVPAAAFGDTDRMVAGEEAYRASARYERDRAYWTGLFTDRPEPVSLTGRGGGRALAPTVRSLGLPPERTEVLGRAAEATGAHWARVVIAGVAAFLHRTTGARDVVVSVPVTGRYGANARITPGMVSNRLPLRLAVRPGESFARVVETVSEAMSGLLAHSRFRGEDLDRELGGAGVSGPTVNVMPYIRPVDFGGPVGLMRSISSGPTTDLNIVLTGTPESGLRVDFEGNPQVYGGQDLTVLQERFVRFLAELAADPAATVDEVALLT

>4A5SA

SRKTYTLTDYLKNTYRLKLYSLRWISDHEYLYKQENNILVFNAEYGNSSVFLENSTFDEFGHSINDYSISPDGQFILLEYNYVKQWRHSYTASYDIYDLNKRQLITEERIPNNTQWVTWSPVGHKLAYVWNNDIYVKIEPNLPSYRITWTGKEDIIYNGITDWVYEEEVFSAYSALWWSPNGTFLAYAQFNDTEVPLIEYSFYSDESLQYPKTVRVPYPKAGAVNPTVKFFVVNTDSLSSVTNATSIQITAPASMLIGDHYLCDVTWATQERISLQWLRRIQNYSVMDICDYDESSGRWNCLVARQHIEMSTTGWVGRFRPSEPHFTLDGNSFYKIISNEEGYRHICYFQIDKKDCTFITKGTWEVIGIEALTSDYLYYISNEYKGMPGGRNLYKIQLIDYTKVTCLSCELNPERCQYYSVSFSKEAKYYQLRCSGPGLPLYTLHSSVNDKGLRVLEDNSALDKMLQNVQMPSKKLDFIILNETKFWYQMILPPHFDKSKKYPLLLDVYAGPCSQKADTVFRLNWATYLASTENIIVASFDGRGSGYQGDKIMHAINRRLGTFEVEDQIEAARQFSKMGFVDNKRIAIWGWSYGGYVTSMVLGSGSGVFKCGIAVAPVSRWEYYDSVYTERYMGLPTPEDNLDHYRNSTVMSRAENFKQVEYLLIHGTADDNVHFQQSAQISKALVDVGVDFQAMWYTDEDHGIASSTAHQHIYTHMSHFIKQCFSLPAAASWSHPQFEK

>2F01A

AEAGITGTWYNQLGSTFIVTAGADGALTGTYESAVGNAESRYVLTGRYDSAPATDGSGTALGWTVAWKNNYRNAHSATTWSGQYVGGAEARINTQWLLTSGTTEANAWKSTLVGHDTFTKVKPSAAS

>1M0KA

MLELLPTAVEGVSQAQITGRPEWIWLALGTALMGLGTLYFLVKGMGVSDPDAKKFYAITTLVPAIAFTMYLSMLLGYGLTMVPFGGEQNPIYWARYADWLFTTPLLLLDLALLVDADQGTILALVGADGIMIGTGLVGALTKVYSYRFVWWAISTAAMLYILYVLFFGFTSKAESMRPEVASTFKVLRNVTVVLWSAYPVVWLIGSEGAGIVPLNIETLLFMVLDVSAKVGFGLILLRSRAIFGEAEAPEPSAGDGAAATSD

>3TEUA

MLDAPTDLQVTNVTDTSITVSWTPPSATITGYRITYTPSNGPGEPKELTVPPSSTSVTITGLTPGVEYVVSVYALKDNQESPPLVGTQTTGGHHHHHH

>2ESSA

GMSEENKIGTYQFVAEPFHVDFNGRLTMGVLGNHLLNCAGFHASDRGFGIATLNEDNYTWVLSRLAIELDEMPYQYEKFSVQTWVENVYRLFTDRNFAVIDKDGKKIGYARSVWAMINLNTRKPADLLALHGGSIVDYICDEPCPIEKPSRIKVTSNQPVATLTAKYSDIDINGHVNSIRYIEHILDLFPIELYQTKRIRRFEMAYVAESYFGDELSFFCDEVSENEFHVEVKKNGSEVVCRSKVIFE

>3A16A

MGSSHHHHHHSSGLVPRGSHMESAIGEHLQCPRTLTRRVPDTYTPPFPMWVGRADDALQQVVMGYLGVQFRDEDQRPAALQAMRDIVAGFDLPDGPAHHDLTHHIDNQGYENLIVVGYWKDVSSQHRWSTSTPIASWWESEDRLSDGLGFFREIVAPRAEQFETLYAFQEDLPGVGAVMDGISGEINEHGYWGSMRERFPISQTDWMQASGELRVIAGDPAVGGRVVVRGHDNIALIRSGQDWADAEADERSLYLDEILPTLQSGMDFLRDNGPAVGCYSNRFVRNIDIDGNFLDLSYNIGHWASLDQLERWSESHPTHLRIFTTFFRVAAGLSKLRLYHEVSVFDAADQLYEYINCHPGTGMLRDAVTIAEH

>3UA3A

MASMSNRTYADNLFPQQVAEQHEEQMSSGSSPKSNSPSRSISSVEAANSRIHIGWMATTLDVAENLDRHVATFCTRLGEFKYNFVVYPIGGVVRAFWTPNGSAENHPPVIDLPDVQLRNDLWESYVVGKISPWIDCDSSDPAFASLSEEHLLKELSYICYLGLQTMAIELTRISSPRTAAILKKWIWTRNSRFTVWVQLPSAIEKCKDYDAFTIEHVDLWTIWADFRKNCGNFSGVYFQVALTISSELPDELTELKLVDRWKAEPLAAFVIESGLFISGRNGEASIPSAHINLLKHLWTTDALRIVLRATTDTFKYNTSIKSEYSQALRHAVRNVNYRSRPDVGEGSNDSTHYLNVIEYKDVLQAPLQPLSENLDSGVYNTFEQDQIKYDVYGEAVVGALKDLGADGRKTVVIYLLGGGRGPIGTKILKSEREYNNTFRQGQESLKVKLYIVEKNPNAIVTLKYMNVRTWKRRVTIIESDMRSLPGIAKDRGFEQPDIIVSELLGSFGDNELSPECLDGVTGFLKPTTISIPQKYTSYVKPIMSTHIHQTIKAQSIPYLSRAIPSHGRGEPELDEDEMWIQKYPQGHVRNNMDQIYVVYLSKYIPLAETTKPVFTFEHPNFMNSSNERSDSIEFVMDRNADLMGFAGYFDLQLYKTVMLSIEPSTHTPGMVSWFPAVIPLRDQLRVGEGDRISLKIDRKVDNTGVWYEWHVEKKKTNGESVSTPIQNPNGESYYMRMLEHHHHHH

>2HO2A

GSDLPAGWMRVQDTSGTYYWHIPTGTTQWEPPGRASPS

>1W7CA

ASAECVSNENVEIEAPKTNIWTSLAKEEVQEVLDLLHSTYNITEVTKADFFSNYVLWIETLKPNKTEALTYLDEDGDLPPRNARTVVYFGEGEEGYFEELKVGPLPVSDETTIEPLSFYNTNGKSKLPFEVGHLDRIKSAAKSSFLNKNLNTTIMRDVLEGLIGVPYEDMGCHSAAPQLHDPATGATVDYGTCNINTENDAENLVPTGFFFKFDMTGRDVSQWKMLEYIYNNKVYTSAEELYEAMQKDDFVTLPKIDVDNLDWTVIQRNDSAPVRHLDDRKSPRLVEPEGRRWAYDGDEEYFSWMDWGFYTSWSRDTGISFYDITFKGERIVYELSLQELIAEYGSDDPFNQHTFYSDISYGVGNRFSLVPGYDCPSTAGYFTTDTFEYDEFYNRTLSYCVFENQEDYSLLRHTGASYSAITQNPTLNVRFISTIGNYDYNFLYKFFLDGTLEVSVRAAGYIQAGYWNPETSAPYGLKIHDVLSGSFHDHVLNYKVDLDVGGTKNRASQYVMKDVDVEYPWAPGTVYNTKQIAREVFENEDFNGINWPENGQGILLIESAEETNSFGNPRAYNIMPGGGGVHRIVKNSRSGPETQNWARSNLFLTKHKDTELRSSTALNTNALYDPPVNFNAFLDDESLDGEDIVAWVNLGLHHLPNSNDLPNTIFSTAHASFMLTPFNYFDSENSRDTTQQVFYTYDDETEESNWEFYGNDWSSCGVEVAEPNFEDYTYGRGTRINKKMTNSDEVY

>3A72A

SSPTSLTNVTIFSPPSDYIVPRTLYPRNEQLPNGDLLATWENYSPEPPAVYFPIYRSKDHGKTWNEISRVHDTVNGYGLRYQPFLYSLPERVGSFKKGTLLLAGSSIPTDLSSTDIVLYASQDDGMTWDFVSHIAAGGEARPNNGLTPVWEPFLLANKGKLICYYSDQRDNATYGQTMVHQVTNDLKNWGPVVEDVTYPTYTDRPGMPVVTKLPNGQYFYVYEYGSFFGTETYSFPLYYRLSSDPENIASAPGQRLVVSSGTQPTSSPYAVWTPYGGENGTIIVSSGTQGTLFINKALGEGEWTEIPCPEEHGYTRALRVLSEDGGRYLVVNSAGVLLGENNRVSVSVMDLKEVL

>3CBZA

GSHMNIITVTLNMEKYNFLGISIVGQSNERGDGGIYIGSIMKGGAVAADGRIEPGDMLLQVNDMNFENMSNDDAVRVLRDIVHKPGPIVLTVAKSGGGSGNEVWIDGP

>2QR4A

MSLSDQEFDEKYLELSEELKQSEKHKGTLDQGASQFLNAIEFVLRVYRQTEVIYVYAHLKNDQDTGNTDYQALYARASSLFSKVSEAVSWFEPEILQLSDDQIWQYFKEEPKLEVYRHYIQQIVDNRAHVLSAEQESLLAGAGEIFDASSDTFAVLNNADLVFPTIEGENGEIVQLSHGVYGQLLESTDRRVREAAFKGLYSVYEQFRNTFASTLGTHIKGHNFKAKVRNYSSAREASLSNNHIPESVYDTLVDVVNKHLPLLHRYMELRKRLLEVEKLHMYDLYTPVLGEAPITFTYEEAKEKALEALKPMGEEYMAIVEKAFSERWIDVVENKGKRSGAYSSGSYDTNPYILLNWHDTLDQLFTLVHEMGHSVHSYFTRSNQPYVYGDYSIFLAEIASTTNENILTEYLLETEKDPRVRAYVLNHYLDGFKGTVFRQTQFAEFEHFMHTEDEKGVPLTSEYLSDSYGKLNAKYYGPAVEEDPEIKFEWSRIPHFYYNYYVFQYSTGFSAASALAKKILNQEPEALENYLAYLKAGNSDYPVEVMKKAGVDMTQAAYIEDAMSMFEQRLNELEELIDREGHHHHHH

>2XFVA

ALEEVVRYLGPHNEIPLTLTRDSETGHFLLKHFLPILQQYHDTGNINETNPDSFPTDEERNKLLAHYGIAVNTDDRGELWIELEKCLQLLNMLNLFGLFQDAFEFEEPETDQDEEDPSHSKLPEN

>2XSKA

MGSSQITFNTTQQGDMYTIIPEVTLTQSCLCRVQILSLREGSSGQSQTKQEKTLSLPANQPIALTKLSLNISPDDRVKIVVTVSDGQSLHLSQQWPPSSEKSLEHHHHHH

>4H5BA

METALLTLDTLAKYLQEKEVQLDIEENGGQRFIRMGWRFEMGDAAVLVSVNDGPNNTSRLEITCVTQKTYADRRAEVAMMLNDRNRERAFARSIDQEGNVWLEYVGFYPTLAEMPQETFDTLFGGVLMHFQDDYAALEGYVPQEGMQIQQPQA

>2NRJA

SLSEIEQTNNGDTALSANEARMKETLQKAGLFAKSMNAYSYMLIKNPDVNFEGITINGYVDLPGRIVQDQKNARAHAVTWDTKVKKQLLDTLNGIVEYDTTFDNYYETMVEAINTGDGETLKEGITDLRGEIQQNQKYAQQLIEELTKLRDSIGHDVRAFGSNKELLQSILKNQGADVDADQKRLEEVLGSVNYYKQLESDGFNVMKGAILGLPIIGGIIVGVARDNLGKLEPLLAELRQTVDYKVTLNRVVGVAYSNINEMHKALDDAINALTYMSTQWHDLDSQYSGVLGHIENAAQKADQNKFKFLKPNLNAAKDSWKTLRTDAVTLKEGIKELKVETVTPQK

>4FIBA

MKVGSQVIINTSHMKGMKGAEATVTGAYDTTAYVVSYTPTNGGQRVDHHKWVIQEEIKDAGDKTLQPGDQVILEASHMKGMKGATAEIDSAEKTTVYMVDYTSTTSGEKVKNHKWVTEDELLEHHHHHH

>4D9IA

MSVFSLKIDIADNKFFNGETSPLFSQSQAKLARQFHQKIAGYRPTPLCALDDLANLFGVKKILVKDESKRFGLNAFKMLGGAYAIAQLLCEKYHLDIETLSFEHLKNAIGEKMTFATTTDGNHGRGVAWAAQQLGQNAVIYMPKGSAQERVDAILNLGAECIVTDMNYDDTVRLTMQHAQQHGWEVVQDTAWEGYTKIPTWIMQGYATLADEAVEQMREMGVTPTHVLLQAGVGAMAGGVLGYLVDVYSPQNLHSIIVEPDKADCIYRSGVKGDIVNVGGDMATIMAGLACGEPNPLGWEILRNCATQFISCQDSVAALGMRVLGNPYGNDPRIISGESGAVGLGVLAAVHYHPQRQSLMEKLALNKDAVVLVISTEGDTDVKHYREVVWEGKHAVAP

>2CJGA

MAAVVKSVALAGRPTTPDRVHEVLGRSMLVDGLDIVLDLTRSGGSYLVDAITGRRYLDMFTFVASSALGMNPPALVDDREFHAELMQAALNKPSNSDVYSVAMARFVETFARVLGDPALPHLFFVEGGALAVENALKAAFDWKSRHNQAHGIDPALGTQVLHLRGAFHGRSGYTLSLTNTKPTITARFPKFDWPRIDAPYMRPGLDEPAMAALEAEALRQARAAFETRPHDIACFVAEPIQGEGGDRHFRPEFFAAMRELCDEFDALLIFDEVQTGCGLTGTAWAYQQLDVAPDIVAFGKKTQVCGVMAGRRVDEVADNVFAVPSRLNSTWGGNLTDMVRARRILEVIEAEGLFERAVQHGKYLRARLDELAADFPAVVLDPRGRGLMCAFSLPTTADRDELIRQLWQRAVIVLPAGADTVRFRPPLTVSTAEIDAAIAAVRSALPVVT

>3RONA

MENLNHCPLEDIKVNPWKTPQSTARVITLRVEDPNEINNLLSINEIDNPNYILQAIMLANAFQNALVPTSTDFGDALRFSMPKGLEIANTITPMGAVVSYVDQNVTQTNNQVSVMINKVLEVLKTVLGVALSGSVIDQLTAAVTNTFTNLNTQKNEAWIFWGKETANQTNYTYNVLFAIQNAQTGGVMYCVPVGFEIKVSAVKEQVLFFTIQDSASYNVNIQSLKFAQPLVSSSQYPIADLTSAINGTL

>1AVGI

AEGDDCSIEKAMGDFKPEEFFNGTWYLAHGPGVTSPAVCQKFTTSGSKGFTQIVEIGYNKFESNVKFQCNQVDNKNGEQYSFKCKSSDNTEFEADFTFISVSYDNFALVCRSITFTSQPKEDRYLVFERTKSDTDPDAKEIC

>4ENEA

MRRRQLIRQLLERDKTPLAILFMAAVVGTLVGLAAVAFDKGVAWLQNQRMGALVHTADNYPLLLTVAFLCSAVLAMFGYFLVRKYAPEAGGSGIPEIEGALEDQRPVRWWRVLPVKFFGGLGTLGGGMVLGREGPTVQIGGNIGRMVLDIFRLKGDEARHTLLATGAAAGLAAAFNAPLAGILFIIEEMRPQFRYTLISIKAVFIGVIMSTIMYRIFNHEVALIDVGKLSDAPLNTLWLYLILGIIFGIFGPIFNKWVLGMQDLLHRVHGGNITKWVLMGGAIGGLCGLLGFVAPATSGGGFNLIPIATAGNFSMGMLVFIFVARVITTLLCFSSGAPGGIFAPMLALGTVLGTAFGMVAVELFPQYHLEAGTFAIAGMGALLAASIRAPLTGIILVLEMTDNYQLILPMIITGLGATLLAQFTGGKPLYSAILARTLAKQEAEQK

>3IO3A

MDLELEPTLESIVQHDSLKWIFVGGKGGVGKTTTSSSVAVQLALAQPNEQFLLISTDPAHNLSDAFCQKFGKDARKVEGLPNLSCMEIDPEAAMSDLQQQASQYNNDPNDPLKSMMSDMTGSIPGIDEALSFMEVLKHIKNQKVLEGEDNSNAISYKTIIFDTAPTGHTLRFLQLPSTLEKLLSKFKDLSGKLGPMLSMMGGGQQQDIFEKLNEVQKNVSEVNEQFTNPELTTFICVCISEFLSLYETERMIQELMSYNMDVNSIVVNQLLFAEGDDHSCKRCESRWKMQKKYLDQMGELYEDYHLVKMPLLGCEIRGVENLKKFSKFLLKPYDPKADSDIVFDLEEK

>3KSNA

GDAASDLKSRLDKVSSFHASFTQKVTDGSGAAVQEGQGDLWVKRPNLFNWHMTQPDESILVSDGKTLWFYNPFVEQATATWLKDATGNTPFMLIARNQSSDWQQYNIKQNGDDFVLTPKASNGNLKQFTINVGRDGTIHQFSAVEQDDQRSSYQLKSQQNGAVDAAKFTFTPPQGVTVDDQRK

>2O5NA

EVVRPEVNRTGTVDICQGPMELIFSVSRTSSGATGERISLKNTLSIVSMENGGKPGTYEWSFPANESWPEIQFLLQNREFVSKYYADVVQTPGELVVEYRCPVPQFNCTITHRWKGETIMSFDGAIQTIRSVTSEYTTKNEDTLVKYIRGLNVTLLTDNAKSIEHRWTEICKKLKDADRPDDNQYTLEDDILEDDIEMDIVQCQMTTQVPLKYHMTVWSAGRDSRAIALSADYYTDIEVASYLPVNRSQILNTTCEITSSSGWTVRLRFSEEMVAASKARQAQKRPLLPVEPHGFMSDEHGPAFVQRTINDSRLTLVPR

>3NYCA

HHHHHHPIEADYLVIGAGIAGASTGYWLSAHGRVVVLEREAQPGYHSTGRSAAHYTVAYGTPQVRALTAASRAFFDNPPAGFCEHPLLSPRPEMVVDFSDDPEELRRQYESGKALVPQMRLLDAEQACSIVPVLRRDKVFGATYDPTGADIDTDALHQGYLRGIRRNQGQVLCNHEALEIRRVDGAWEVRCDAGSYRAAVLVNAAGAWCDAIAGLAGVRPLGLQPKRRSAFIFAPPPGIDCHDWPMLVSLDESFYLKPDAGMLLGSPANADPVEAHDVQPEQLDIATGMYLIEEATTLTIRRPEHTWAGLRSFVADGDLVAGYAANAEGFFWVAAQGGYGIQTSAAMGEASAALIRHQPLPAHLREHGLDEAMLSPRRLSP

>3ZN6A

MGVFDRIRGALGRGLDVFRGDLPQVQPPAPQPAPAPAITPAAVQVGGWGFAWIDNEDFSPTGLAWRSGEYFALAQMKTPETAHFRIAAQERRLRIYLRGQKVVNGRNLSDPDSRTVNLPFLMQTPQGAPTLPSTYHPDVAVWAKVGSTWQPCVITAINYSTGDVTFTEPAGVTASDGIEIYYVHGDGQFRLRVARDAGGVDDSAATVFNQSFSTMHSVDQNNVETMIAWPQQVELVPGTRLVLEVFTTQVPMVWNERSGHYIQIAAMGRRIEVLDKGGLQRLAELEARGGL

>3PUIA

MVDCNYSVACNVMVPMRDGVRLAVDLYRPDADGPVPVLLVRNPYDKFDVFAWSTQSTNWLEFVRDGYAVVIQDTRGLFASEGEFVPHVDDEADAEDTLSWILEQAWCDGNVGMFGVSYLGVTQWQAAVSGVGGLKAIAPSMASADLYRAPWYGPGGALSVEALLGWSALIGTGLITSRSDARPEDAADFVQLAAILNDVAGAASVTPLAEQPLLGRLIPWVIDQVVDHPDNDESWQSISLFERLGGLATPALITAGWYDGFVGESLRTFVAVKDNADARLVVGPWSHSNLTGRNADRKFGIAATYPIQEATTMHKAFFDRHLRGETDALAGVPKVRLFVMGIDEWRDETDWPLPDTAYTPFYLGGSGAANTSTGGGTLSTSISGTESADTYLYDPADPVPSLGGTLLFHNGDNGPADQRPIHDRDDVLCYSTEVLTDPVEVTGTVSARLFVSSSAVDTDFTAKLVDVFPDGRAIALCDGIVRMRYRETLVNPTLIEAGEIYEVAIDMLATSNVFLPGHRIMVQVSSSNFPKYDRNSNTGGVIAREQLEEMCTAVNRIHRGPEHPSHIVLPIIKRKLAAALEHHHHHH

>1YLIA

SANFTDKNGRQSKGVLLLRTLAMPSDTNANGDIFGGWIMSQMDMGGAILAKEIAHGRVVTVAVESMNFIKPISVGDVVCCYGQCLKVGRSSIKIKVEVWVKKVASEPIGERYCVTDAVFTFVAVDNNGRSRTIPRENNQELEKALALISEQPL

>2AHFA

MWQQAIGDALGITARNLKKFGDRFPHVSDGSNKYVLNDNTDWTDGFWSGILWLCYEYTGDEQYREGAVRTVASFRERLDRFENLDHHNIGFLYSLSAKAQWIVEKDESARKLALDAADVLMRRWRADAGIIQAWGPKGDPENGGRIIIDCLLNLPLLLWAGEQTGDPEYRRVAEAHALKSRRFLVRGDDSSYHTFYFDPENGNAIRGGTHQGNTDGSTWTRGQAWGIYGFALNSRYLGNADLLETAKRMARHFLARVPEDGVVYWDFEVPQEPSSYRDSSASAITACGLLEIASQLDESDPERQRFIDAAKTTVTALRDGYAERDDGEAEGFIRRGSYHVRGGISPDDYTIWGDYYYLEALLRLERGVTGYWYERGR

>2YYYA

MPAKVLINGYGSIGKRVADAVSMQDDMEVIGVTKTKPDFEARLAVEKGYKLFVAIPDNERVKLFEDAGIPVEGTILDIIEDADIVVDGAPKKIGKQNLENIYKPHKVKAILQGGEKAKDVEDNFNALWSYNRCYGKDYVRVVSCNTTGLCRILYAINSIADIKKARIVLVRRAADPNDDKTGPVNAITPNPVTVPSHHGPDVVSVVPEFEGKILTSAVIVPTTLMHMHTLMVEVDGDVSRDDILEAIKKTPRIITVRAEDGFSSTAKIIEYGRDLGRLRYDINELVVWEESINVLENEIFLMQAVHQESIVIPENIDCIRAMLQMEEDNFKSIEKTNKAMGIQ

>4DOIA

MSSSNACASPSPFPAVTKLHVDSVTFVPSVKSPASSNPLFLGGAGVRGLDIQGKFVIFTVIGVYLEGNAVPSLSVKWKGKTTEELTESIPFFREIVTGAFEKFIKVTMKLPLTGQQYSEKVTENCVAIWKQLGLYTDCEAKAVEKFLEIFKEETFPPGSSILFALSPTGSLTVAFSKDDSIPETGIAVIENKLLAEAVLESIIGKNGVSPGTRLSVAERLSQLMMKNKDEKEVSDHSVEEKLAKEN

>4DOXA

MSKSSMSTPNIAFPAITQEQMSSIKVDPTSNLLPSQEQLKSVSTLMVAAKVPAASVTTVALELVNFCYDNGSSAYTTVTGPSSIPEISLAQLASIVKASGTSLRKFCRYFAPIIWNLRTDKMAPANWEASGYKPSAKFAAFDFFDGVENPAAMQPPSGLTRSPTQEERIANATNKQVHLFQAAAQDNNFASNSAFITKGQISGSTPTIQFLPPPETSTTRHHHHHH

>4GYTA

GMSLDNDSLHLPKYDDFVQSISVLALTMSGSELHGIMCGYLCAGADSQGEAYIRALLNNKKDEQSRNALLSMFSVFSISQQQMNNFDFEFEMLLPDDDESLVTRAQAFSEWCEGFTQGLTIAGVGMEQFYEEESQDALQHLMEFAELDCESLEVGEEDERALMEVSEYTRMAVLRLHSDLVLHERELGDSGTTH

>1MSPA

AQSVPPGDINTQPSQKIVFNAPYDDKHTYHIKITNAGGRRIGWAIKTTNMRRLSVDPPCGVLDPKEKVLMAVSCDTFNAATEDLNNDRITIEWTNTPDGAAKQFRREWFQGDGMVRRKNLPIEYNL

>1U19A

XMNGTEGPNFYVPFSNKTGVVRSPFEAPQYYLAEPWQFSMLAAYMFLLIMLGFPINFLTLYVTVQHKKLRTPLNYILLNLAVADLFMVFGGFTTTLYTSLHGYFVFGPTGCNLEGFFATLGGEIALWSLVVLAIERYVVVCKPMSNFRFGENHAIMGVAFTWVMALACAAPPLVGWSRYIPEGMQCSCGIDYYTPHEETNNESFVIYMFVVHFIIPLIVIFFCYGQLVFTVKEAAAQQQESATTQKAEKEVTRMVIIMVIAFLICWLPYAGVAFYIFTHQGSDFGPIFMTIPAFFAKTSAVYNPVIYIMMNKQFRNCMVTTLCCGKNPLGDDEASTTVSKTETSQVAPA

>4B0MA

AYTFDSTMLDTNSGESIDVSLFNQGLQLPGNYFVNVFVNGRKVDSGNIDFRLEKHNGKELLWPCLSSLQLTKYGIDIDKYPDLIKSGTEQCVDLLAIPHSDVQFYFNQQKLSLIVPPQALLPRFDGIMPMQLWDDG

>2YMOA

GSAMGNLTCDFNDVYKLEFHPNQQTSVTKLCNLTPNVLEKVTIKCGSDKLNYNLYPPTCFEEVYASRNMMHLKKIKEFVIGSSMFMRRSLTPNKINEVSFRIPPNMMPEKPIYCFCENKKTITINGSNGNPSSKKDIINRGIVEIIIPSLNEKVKGCDFTTSESTIFSKGYSINEISNKSSNNQQDIVCTVKAHANDLIGFKCPSNYSVEPHDCFVSAFNLSGKNENLENKLKLTNIIMDHYNNTFYSRLPSLISDNWKFFCVCSKDNEKKLVFTVEASISSAAALVPR

>1A9XA

MPKRTDIKSILILGAGPIVIGQACEFDYSGAQACKALREEGYRVINVNSNPATIMTDPEMADATYIEPIHWEVVRKIIEKERPDAVLPTMGGQTALNCALELERQGVLEEFGVTMIGATADAIDKAEDRRRFDVAMKKIGLETARSGIAHTMEEALAVAADVGFPCIIRPSFTMGGSGGGIAYNREEFEEICARGLDLSPTKELLIDESLIGWKEYEMEVVRDKNDNCIIVCSIENFDAMGIHTGDSITVAPAQTLTDKEYQIMRNASMAVLREIGVETGGSNVQFAVNPKNGRLIVIEMNPRVSRSSALASKATGFPIAKVAAKLAVGYTLDELMNDITGGRTPASFEPSIDYVVTKIPRFNFEKFAGANDRLTTQMKSVGEVMAIGRTQQESLQKALRGLEVGATGFDPKVSLDDPEALTKIRRELKDAGADRIWYIADAFRAGLSVDGVFNLTNIDRWFLVQIEELVRLEEKVAEVGITGLNADFLRQLKRKGFADARLAKLAGVREAEIRKLRDQYDLHPVYKRVDTCAAEFATDTAYMYSTYEEECEANPSTDREKIMVLGGGPNRIGQGIEFDYCCVHASLALREDGYETIMVNCNPETVSTDYDTSDRLYFEPVTLEDVLEIVRIEKPKGVIVQYGGQTPLKLARALEAAGVPVIGTSPDAIDRAEDRERFQHAVERLKLKQPANATVTAIEMAVEKAKEIGYPLVVRASYVLGGRAMEIVYDEADLRRYFQTAVSVSNDAPVLLDHFLDDAVEVDVDAICDGEMVLIGGIMEHIEQAGVHSGDSACSLPAYTLSQEIQDVMRQQVQKLAFELQVRGLMNVQFAVKNNEVYLIEVNPRAARTVPFVSKATGVPLAKVAARVMAGKSLAEQGVTKEVIPPYYSVKEVVLPFNKFPGVDPLLGPEMRSTGEVMGVGRTFAEAFAKAQLGSNSTMKKHGRALLSVREGDKERVVDLAAKLLKQGFELDATHGTAIVLGEAGINPRLVNKVHEGRPHIQDRIKNGEYTYIINTTSGRRAIEDSRVIRRSALQYKVHYDTTLNGGFATAMALNADATEKVISVQEMHAQIK

>3BJQA

GMSQMSPGQARVVDPILSTHARGYRQSTLIGKKLFPVAPVAQYGGKILTFGKEAFRLYNTKRAPGANTKRIDFGYEGDPYSIVPSALEAKVPRELMRDASQVPGIDLGARSVNTVLRIMALAHEHECAQIALDPAKYNADHKVKLVGSARWTSPDSDPTKDVETAKEAIADSIGMEPNRLMLSRKALSACKYHPKLIERVKYTRAESITIDMLKALWEVEEIVVGTARVATGANDSFGDVWGPDVWLGYVSDNPDPSVEEPSFGYTYQIEGHPLVEVPYWDNNAKSWIYGVSDDNTPALSGMLAGYLIEDAGLPAA

>3LLPA

MTANGTAEAVQIQFGLINCGNKYLTAEAFGFKVNASASSLKKKQIWTLEQPPDEAGSAAVCLRSHLGRYLAADKDGNVTCEREVPGPDCRFLIVAHDDGRWSLQSEAHRRYFGGTEDRLSCFAQTVSPAEKWSVHIAMHPQVNIYSVTRKRYAHLSARPADEIAVDRDVPWGVDSLITLAFQDQRYSVQTADHRFLRHDGRLVARPEPATGYTLEFRSGKVAFRDCEGRYLAPSGPSGTLKAGKATKVGKDELFALEQSCAQVVLQAANERNVSTRQGMDLSANQDEETDQETFQLEIDRDTKKCAFRTHTGKYWTLTATGGVQSTASSKNASCYFDIEWRDRRITLRASNGKFVTSKKNGQLAASVETAGDSELFLMKLINRPIIVFRGEHGFIGCRKVTGTLDANRSSYDVFQLEFNDGAYNIKDSTGKYWTVGSDSAVTSSGDTPVDFFFEFCDYNKVAIKVGGRYLKGDHAGVLKASAETVDPASLWEY

>3U99A

DGRGLSRAIPQNAEYTAECGSCHMAYPANLLPADKWRAITANLENHFGDNASLDPQVTARIEEYLVQHAAQNGKVMKNSTPLLAGAGPQKITEQAFFIRKHDEIPRRMVQDNPKVGSFSQCSNCHNLAEKGIFDEDTVNIPGFGRWDD

>2IN5A

MTHSQQSMVDTFRASLFDNQDITVADQQIQALPYSTMYLRLNEGQRIFVVLGYIEQEQSKWLSQDNAMLVTHNGRLLKTVKLNNNLLEVTNSGQDPLRNALAIKDGSRWTRDILWSEDNHFRSATLSSTFSFAGLETLNIAGRNVLCNVWQEEVTSTRPEKQWQNTFWVDSATGQVRQSRQMLGAGVIPVEMTFLKPAPLEHHHHHH

>4G4SO

MLFKQWNDLPEPKHLLDLPEISKNLQSLEVCPVPKVEFPQDLDVPQYSTAVITTKIMNPLFPKNLLQLTSIGEIKTTLTVKSPSLPQSSGKHSWNYDENFPNEVDPDQKNDTADETVYGFSFPIYSFGKTLLFSMEENFISISPIFGNMISRSIISQLAQFSPDIIVIGTSDKIASMKVMTENECTLQPPEFITGFIGSVLTQLIVGPSKGLKFKCLVAPSEGPNGFEKLSLSDMGSLVDLCGQWLGFEPSRYSEECYRLWRCDSAAIGAQSGLYI

>1UUNA

GLDNELSLVDGQDRTLTVQQWDTFLNGVFPLDRNRLTREWFHSGRAKYIVAGPGADEFEGTLELGYQIGFPWSLGVGINFSYTTPNILIDDGDITRPPFGLNSVITPNLFPGVSISADLGNGPGIQEVATFSVDVSGAEGGVAVSNAHGTVTGAAGGVLLRPFARLIASTGDSVTTYGEPWNMN

>1ZJCA

GSHMTNYKEKLQQYAELLVKVGMNVQPKQPVFIRSSVETLELTHLIVEEAYHCGASDVRVVYSDPTLKRLKFENESVEHFANHEIKSYDVEARMDYVKRGAANLALISEDPDLMDGIDSQKLQAFQQQNARAFKGYMESVQKNQFPWVVAAFPSKAWAKRVYPELSVEEAYIKFIDEVFDIVRIDGNDPVENWRQHIANLSVYAQKLQQKNYHALHYVSEGTDLTVGLAKNHIWEDATSYVNGKEQAFIANIPTEEVFTAPDRNRVDGYVTNKLPLSYNGTIIDQFKLMFKDGEIIDFSAEKGEAVLKDLINTDEGSRRLGEVALVPDDSPISNRNTIFYNTLFDENAACHLAIGSAYAFNIQGGTEMTVEEKIASGLNDSNVHVDFMIGSSDLTIYGIFEDGSKELVFENGNWASTF

>2C71A

MPANKLVALTFDDGPDNVLTARVLDKLDKYNVKATFMVVGQRVNDSTAAIIRRMVNSGHEIGNHSWSYSGMANMSPDQIRKSIADTNAVIQKYAGTTPKFFRPPNLETSPTLFNNVDLVFVGGLTANDWIPSTTAEQRAAAVINGVRDGTIILLHDVQPEPHPTPEALDIIIPTLKSRGYEFVTLTELFTLKGVPIDPSVKRMYNSVPLEHHHHHH

>4EE6A

GSMSESADLTELYSIIEKTAQVVDVTASHDKVWPILNAFQDVIADSVISFRASTGSSADDLDCRFTMLPKGLDPYARALEHGLTPKTDHPVGSLLKEVHENLPITSCGVDFGVAGGFTKTWSFPSAEKLGKVSELVKLPSIPDAVAANRDFFEKWGIADMVSTVGIDYSKRTMNLYFGGGVGDRVPAGVFEEKGVRAILGELGLAAPSEELLKFCERSFVIYVTLSWDSPKINRFTYSVMTPEPLGLPVDLAPTFERLIKSAPYDTEGRNYVYGIASTPKGEYHKIASYYQWQKRVEKLLRSDG

>4E6XA

ERLSTLIHQRMQEAKVPALSVSVTIKGVRQRFVYGVADVASQKANTLDTVYELGSMSKAFTGLVVQILIQEGRLRQGDDIITYLPEMRLNYQGKPASLTVADFLYHTSGLPFSTLARLENPMPGSAVAQQLRNENLLFAPGAKFSYASANYDVLGAVIENVTGKTFTEVIAERLTQPLGMSATVAVKGDEIIVNKASGYKLGFGKPVLFHAPLARNHVPAAYIHSTLPDMEIWIDAWLHRKALPATLREAMSNSWRGNSDVPLAADNRILYASGWFIDQNQGPYISHGGQNPNFSSCIALRPDQQIGIVALANMNSNLILQLCADIDNYLRIGKYA

>2WURA

MSKGEELFTGVVPILVELDGDVNGHKFSVSGEGEGDATYGKLTLKFICTTGKLPVPWPTLVTTLSYGVQCFSRYPDHMKRHDFFKSAMPEGYVQERTIFFKDDGNYKTRAEVKFEGDTLVNRIELKGIDFKEDGNILGHKLEYNYNSHNVYIMADKQKNGIKVNFKTRHNIEDGSVQLADHYQQNTPIGDGPVLLPDNHYLSTQSALSKDPNEKRDHMVLLEFVTAAGITHGMDELYN

>1JUVA

MIKLVFRYSPTKTVDGFNELAFGLGDGLPWGRVKKDLQNFKARTEGTIMIMGAKTFQSLPTLLPGRSHIVVCDLARDYPVTKDGDLAHFYITWEQYITYISGGEIQVSSPNAPFETMLDQNSKVSVIGGPALLYAALPYADEVVVSRIVKRHRVNSTVQLDASFLDDISKREMVETHWYKIDEVTTLTESVYK

>4GQ2M

RMNELKHAVVPIDLQSFCLEGTLALWVPALENDSEDDSEAIETADDNEKLFKKECVAYDAGVYTSNKSKGSQTLRWSIFQNRTLTIFDVSLNSKKEPLSKFNVKIHFPSNVMKDGVAFSFSEHSDTTIIYAITHARVLYYIRLSKTWFQLPDARLDDDWCLCYRPISFLNQKPDLMAAISTSEICVSFFNGGLTKIILNPKDASHYEQHIDDSSYLFSLKKYLSLQAFKADYRSPNTIISMIFLSTYNVLVMLSLDYKLKVLDLSTNQCVETIELSQTILPLQSFPYLTSDHTTNSFIALYYPDNSHGSFSIYKLNANAHSFKLNVVIEKGIIPPSLPDDEFIPWMLSDFQLISSEGSQSKFLLIIAWKSNLNTVIQKCNLSLDQDESFSCVWSHSLDSFSLIEKTFFDVPTNMSSGDISEIWLQHIFAHNTSIESIQVALLSFQNSSSQVSKNKLDKFGALTISELKNAVLSSIVSTIQIEPNSDLTGYDYYEYKRLLYNEWERFAKLVAYLDHFGDEILSINFDPSNAVTYINYANKVAFIRDPYLIESFDEEPLTKLISSLETDDPSLIEGYQILDLGRSLHSCMSFSTLSEIRYSLRELVQDLPSYSLFDTLWVFYDKHIYPNVDPDYISTLIDTLVSLENPMRDIDSLIQRLRSFDIYNHSAQSPSLFLCASVARVLDSILKKFQVSIEGFIFLLSLITSQQDYELQSKFAGCDKLFLSLLEDWRLVSFLLENSALLLEKFEEEDVDSTNCNLNTMEALASVNTALQFFSALNYSECFSESQISPLHATVISSLSAIFIRDDTENDLVTELVEKLFLFKQYNACMQLIGWLNSDPIAVYLKALIYLKSKEAVKAVRCFKTTSLVLYSHTSQFAVLREFQEIAEKYHHQNLLSCYYLHLSKKLFEESAYIDALEFSLLADASKETDDEDLSIAITHETLKTACAAG

>2VK8A

MSEITLGKYLFERLKQVNVNTVFGLPGDFNLSLLDKIYEVEGMRWAGNANELNAAYAADGYARIKGMSCIITTFGVGELSALNGIAGSYAEHVGVLHVVGVPSISAQAKQLLLHHTLGNGDFTVFHRMSANISETTAMITDIATAPAEIDRCIRTTYVTQRPVYLGLPANLVDLNVPAKLLQTPIDMSLKPNDAESEKEVIDTILVLDKDAKNPVILADACCSRHDVKAETKKLIDLTQFPAFVTPMGKGSIDEQHPRYGGVYVGTLSKPEVKEAVESADLILSVGALLSDFNTGSFSYSYKTKNIVEFHSDHMKIRNATFPGVQMKFVLQKLLTTIADAAKGYKPVAVPARTPANAAVPASTPLKQEWMWNQLGNFLQEGDVVIAETGTSAFGINQTTFPNNTYGISQVLWGSIGFTTGATLGAAFAAEEIDPKKRVILFIGDGSLQLTVQEISTMIRWGLKPYLFVLNNDGYTIQKLIHGPKAQYNEIQGWDHLSLLPTFGAKDYETHRVATTGEWDKLTQDKSFNDNSKIRMIEVMLPVFDAPQNLVEQAKLTAATNAKQ

>4GXWA

MVKGTPGNVPAARTGIEITAAHRAFFHALPKVELHCHLLGAVRHDTFVALAQRSGAPIERAEIDAFYARGEKPVGVLHVLRALDRYLLTRPDDLRRIAYEYLEDAAAHNVRHAEFFWNPTGTVRVSGIPYADAQAAIVTGMRDAARDFGIGARLIPSIDREQDPDEAVAIVDWMKANRADEVAGIGIDYRENDRPPELFWKAYRDARAAGFRTTAHAGEFGMPWRNVETAVDLLHVDRVDHGYTIVDNPELCARYAERGIVFTVVPTNSYYLRTLPPDQWAERHPMRKMPGLGLKIHPNTDDPTLHKVNPSEAWELMFSHFGFTIADLKQFMLNGIDGAWVDDDTKAAWRAAWAPEFDMLADTLAADKLAAALEHHHHHH

>1JLYA

XAGLPVIMCLKSNNHQKYLRYQSDNIQQYGLLQFSADKILDPLAQFEVEPSKTYDGLVHIKSRYTNKYLVRWSPNHYWITASANEPDENKSNWACTLFKPLYVEEGNMKKVRLLHVQLGHYTQNYTVGGSFVSYLFAESSQIDTGSKDVFHVIDWKSIFQFPKGYVTFKGNNGKYLGVITINQLPCLQFGYDNLNDPKVAHQMFVTSNGTICIKSNYMNKFWRLSTDDWILVDGNDPRETNEAAALFRSDVHDFNVISLLNMQKTWFIKRFTSGKPGFINCMNAATQNVDETAILEIIELGQNN

>1TE2A

GHMSTPRQILAAIFDMDGLLIDSEPLWDRAELDVMASLGVDISRRNELPDTLGLRIDMVVDLWYARQPWNGPSRQEVVERVIARAISLVEETRPLLPGVREAVALCKEQGLLVGLASASPLHMLEKVLTMFDLRDSFDALASAEKLPYSKPHPQVYLDCAAKLGVDPLTCVALEDSVNGMIASKAARMRSIVVPAPEAQNDPRFVLANVKLSSLTELTAKDLLGGS

>1MKFA

LTLGLAPALSTHSSGVSTQSVDLSQIKRGDEIQAHCLTPAETEVTECAGILKDVLSKNLHELQGLCNVKNKMGVPWVSVEELGQEIITGRLPFPSVGGTPVNDLVRVLVVAESNTPEETPEEEFYAYVELQTELYTFGLSDDNVVFTSDYMTVWMIDIPKSYVDVGMLTRATFLEQWPGAKVTVMIPYSSTFTWCGELGAISEESAPQPSLSARSPVCKNSARYSTSKFCEVDGCTAETGMEKMSLLTPFGGPPQQAKMNTCPCYYKYSVSPLPAMDHLILADLAGLDSLTSPVYVMAAYFDSTHENPVRPSSKLYHCALQMTSHDGVWTSTSSEQCPIRLVEGQSQNVLQVRVAPTSMPNLVGVSLMLEGQQYRLEYFGDH

>1EI9A

DPPAPLPLVIWHGMGDSCCNPLSMGAIKKMVEKKIPGIHVLSLEIGKTLREDVENSFFLNVNSQVTTVCQILAKDPKLQQGYNAMGFSQGGQFLRAVAQRCPSPPMVNLISVGGQHQGVFGLPRCPGESSHICDFIRKTLNAGAYNKAIQERLVQAEYWHDPIREDIYRNHSIFLADINQERGVNESYKKNLMALKKFVMVKFLNDTIVDPVDSEWFGFYRSGQAKETIPLQESTLYTQDRLGLKAMDKAGQLVFLALEGDHLQLSEEWFYAHIIPFLE

>3NO2A

GSSPQHLLVGGSGWNKIAIINKDTKEIVWEYPLEKGWECNSVAATKAGEILFSYSKGAKMITRDGRELWNIAAPAGCEMQTARILPDGNALVAWCGHPSTILEVNMKGEVLSKTEFETGIERPHAQFRQINKNKKGNYLVPLFATSEVREIAPNGQLLNSVKLSGTPFSSAFLDNGDCLVACGDAHCFVQLNLESNRIVRRVNANDIEGVQLFFVAQLFPLQNGGLYICNWQGHDREAGKGKHPQLVEIDSEGKVVWQLNDKVKFGMISTICPIRE

>4KQCA

SNADPYIAVVSKGFQHKFWVTVRDGAEAAAKQNGVKISFVGPETESDSKIQQDLLDSEINKNPDAIAFAAVTGDFTEQIKRIKEKNIPLIGFDSGILPDQAQGAVLATASTDNRAAAAIVADKMFEALKTRIAAFTSDNKAKIAVLQLDNSDTGIGRAEGFVKRFTELADGDAATAGKYALQVIVPTTQNEADIANEVNALRGKSVLGIYLSNEAMARGFLVVYKSAEAGAANTIVGDAQDGGDLVVMGFDSGKPQLDAIRNGIIQGSVTQDPYSIGFQAVTLAYKASKGESVSNIDTGAKWYDKTNIDDPEIAKLLYE

>2H1VA

MSRKKMGLLVMAYGTPYKEEDIERYYTHIRRGRKPEPEMLQDLKDRYEAIGGISPLAQITEQQAHNLEQHLNEIQDEITFKAYIGLAHIEPFIEDAVAEMHKDGITEAVSIVLAPHFSTFSVQSYNKRAKEEAEKLGGLTITSVESWYDEPKFVTYWVDRVKETYASMPEDERENAMLIVSAHSLPEKIKEFGDPYPDQLHESAKLIAEGAGVSEYAVGWQSEGNTPDPWLGPDVQDLTRDLFEQKGYQAFVYVPVGFVADHLEVLYDNDYECKVVTDDIGASYYRPEMPNAKPEFIDALATVVLKKLGR

>2OGBA

STIEYNEILEWVNSLQPARVTRWGGMISTPDAVLQAVIKRSLVESGCPASIVNELIENAHERSWPQGLATLETRQMNRRYYENYVAKRIPGKQAVVVMACENQHMGDDMVQEPGLVMIFAHGVEEI

>1U5XA

GSKKHSVLHLVPVNITSKADSDVTEVMWQPVLRRGRGLEAQGDIVRVWDTGIYLLYSQVLFHDVTFTMGQVVSREGQGRRETLFRCIRSMPSDPDRAYNSCYSAGVFHLHQGDIITVKIPRANAKLSLSPHGTFLGFVKL

>3LGEE

QAYQGPATGDDDDWDEDWDGPKSSSYFKDSE

>1L9XA

MGSSHHHHHHSSGLVPRGSHMRPHGDTAKKPIIGILMQKCRNKVMKNYGRYYIAASYVKYLESAGARVVPVRLDLTEKDYEILFKSINGILFPGGSVDLRRSDYAKVAKIFYNLSIQSFDDGDYFPVWGTCLGFEELSLLISGECLLTATDTVDVAMPLNFTGGQLHSRMFQNFPTELLLSLAVEPLTANFHKWSLSVKNFTMNEKLKKFFNVLTTNTDGKIEFISTMEGYKYPVYGVQWHPEKAPYEWKNLDGISHAPNAVKTAFYLAEFFVNEARKNNHHFKSESEEEKALIYQFSPIYTGNISSFQQCYIFD

>3PNRB

MGHHHHHHHHHHSSGHIEGRHMGDEKCGKSLKLGNISNQTNQETITQSLSVGEILCIDLEGNAGTGYLWVLLGIHKDEPIINPENFPTKLTKKSFFSEEISVTQPKKYKIDEHDSSKNVNREIESPEQKESDSKPKKPQMQLLGGPDRMRSVIKGHKPGKYYIVYSYYRPFSPTSGANTKIIYVTVQ

>4A8JC

MHHHHHHMGSVQRQDLVLFSDQSVLPAHFFQDSNSHNLFFITHQSCTQPLWMINALVETHVLGSPSSLNESSSSMLPSSTRSHAVLASFIHEQNYFTNSLNKLKIPSNNYNVLDFLSDFIVNNIHNKPRDKILSDVLAKFSAAIQNNPTDTIVIIEQPELLLSLVSGLTCSELNNKFITPLLRQCKVLIIVSNSDIFNIDEYDASVHSSNLQNFYKSSFIKSMINLNLNPLKTGFAKDVTGSLHVCRGGAPIATSNTSLHVVENEYLYLNEKESTKLFYR

>1FN9A

MEVCLPNGHQVVDLINNAFEGRVSIYSAQEGWDKTISAQPDMMVCGGAVVCMHCLGVVGSLQRKLKHLPHHRCNQQIRHQDYVDVQFADRVTAHWKRGMLSFVAQMHEMMNDVSPDDLDRVRTEGGSLVELNWLQVDPNSMFRSIHSSWTDPLQVVDDLDTKLDQYWTALNLMIDSSDLIPNFMMRDPSHAFNGVKLGGDARQTQFSRTFDSRSSLEWGVMVYDYSELEHDPSKGRAYRKELVTPARDFGHFGLSHYSRATTPILGKMPAVFSGMLTGNCKMYPFIKGTAKLKTVRKLVEAVNHAWGVEKIRYALGPGGMTGWYNRTMQQAPIVLTPAALTMFPDTIKFGDLNYPVMIGDPMILG

>1ZJCA

GSHMTNYKEKLQQYAELLVKVGMNVQPKQPVFIRSSVETLELTHLIVEEAYHCGASDVRVVYSDPTLKRLKFENESVEHFANHEIKSYDVEARMDYVKRGAANLALISEDPDLMDGIDSQKLQAFQQQNARAFKGYMESVQKNQFPWVVAAFPSKAWAKRVYPELSVEEAYIKFIDEVFDIVRIDGNDPVENWRQHIANLSVYAQKLQQKNYHALHYVSEGTDLTVGLAKNHIWEDATSYVNGKEQAFIANIPTEEVFTAPDRNRVDGYVTNKLPLSYNGTIIDQFKLMFKDGEIIDFSAEKGEAVLKDLINTDEGSRRLGEVALVPDDSPISNRNTIFYNTLFDENAACHLAIGSAYAFNIQGGTEMTVEEKIASGLNDSNVHVDFMIGSSDLTIYGIFEDGSKELVFENGNWASTF

>1XCRA

GSACAEFSFHVPSLEELAGVMQKGLKDNFADVQVSVVDCPDLTKEPFTFPVKGICGKTRIAEVGGVPYLLPLVNQKKVYDLNKIAKEIKLPGAFILGAGAGPFQTLGFNSEFMPVIQTESEHKPPVNGSYFAHVNPADGGCLLEKYSEKCHDFQCALLANLFASEGQPGKVIEVKAKRRTGPLNFVTCMRETLEKHYGNKPIGMGGTFIIQKGKVKSHIMPAEFSSCPLNSDEEVNKWLHFYEMKAPLVCLPVFVSRDPGFDLRLEHTHFFSRHGEGGHYHYDTTPDIVEYLGYFLPAEFLYRIDQPKETHSIGRD

>3O4PA

MEIPVIEPLFTKVTEDIPGAEGPVFDKNGDFYIVAPEVEVNGKPAGEILRIDLKTGKKTVICKPEVNGYGGIPAGCQCDRDANQLFVADMRLGLLVVQTDGTFEEIAKKDSEGRRMQGCNDCAFDYEGNLWITAPAGEVAPADYTRSMQEKFGSIYCFTTDGQMIQVDTAFQFPNGIAVRHMNDGRPYQLIVAETPTKKLWSYDIKGPAKIENKKVWGHIPGTHEGGADGMDFDEDNNLLVANWGSSHIEVFGPDGGQPKMRIRCPFEKPSNLHFKPQTKTIFVTEHENNAVWKFEWQRNGKKQYCETLKFGIF

>2NLVA

GMDKLVKYQELVKKLLTNYASDDVSDQDVEVQLILDTERNHYQWMNVGWQGLNRIYRCVIHFDIKDGKIWLQQNLTDRNPAEELVMMGVPREDIVLGLQAPYKRQYTDYGVA

>3H6RA

MASLEDGTYRLRAVTTHNPDPGVGGEYATVEGARQPVKAEPSTPPFSEQQIWQVTRNSDGQYTIKYQGLNAPFEYGFSYDQLEPNAPVIAGDPKEYILQLVPSTADVYIIRAPIQRVGVDVEVGVQGNTLVYKFFPVDGSGGDRPAWRFTRE

>1BRTA

PFITVGQENSTSIDLYYEDHGTGQPVVLIHGFPLSGHSWERQSAALLDAGYRVITYDRRGFGQSSQPTTGYDYDTFAADLNTVLETLDLQDAVLVGFSTGTGEVARYVSSYGTARIAKVAFLASLEPFLLKTDDNPDGAAPQEFFDGIVAAVKADRYAFYTGFFNDFYNLDENLGTRISEEAVRNSWNTAASGGFFAAAAAPTTWYTDFRADIPRIDVPALILHGTGDRTLPIENTARVFHKALPSAEYVEVEGAPHGLLWTHAEEVNTALLAFLAK

>4I8IA

GNGCHANNDTIKVLAIGNSFSQDAVEQYLHELGEAEGITMIIGNMFIGGCSLERHVQNIRNNAPAYAYRKVEKDGEKTETRSMTIEKALADEKWDYISVQQASPLSGIYDSYKASLPELVNYIRERIGKETVLMMHQTWAYATNANHTGFKNYDQNQMKMYTSIVDAVKKAANLVGIKKIIPSGTAIQNARTSFIGDHMNRDGYHLDLTIGRYTAACTWFEALTHRNVTENPYSPEGIDPIHKKAAQMAAHNAILYPDKVTELTELKKIAD

>3D8UA

SNAYSIALIIPSLFEKACAHFLPSFQQALNKAGYQLLLGYSDYSIEQEEKLLSTFLESRPAGVVLFGSEHSQRTHQLLEASNTPVLEIAELSSKASYLNIGVDHFEVGKACTRHLIEQGFKNVGFIGARGNHSTLQRQLHGWQSAMIENYLTPDHFLTTHEAPSSQLGAEGLAKLLLRDSSLNALVCSHEEIAIGALFECHRRVLKVPTDIAIICLEGSSMGEHAYPSLTSAEFDYERMGTKAAEKLLHAIKGEPEERPTSMGFKLKRRASTAIN

>2WP7A

MEPPNLYPVKLYVYDLSKGLARRLSPIMLGKQLEGIWHTSIVVHKDEFFFGSSGISSCTPGGTLLGPPDSVVDVGNTEVTEEIFLEYLSSLGESLFRGEAYNLFEHNCNTFSNEVAQFLTGRKIPSYITDLPSEVLSTPFGQALRPFLDSIQIQPPGGNSVGRPNGQS

>4A7UA

ATKAVCVLKGDGPVQGIINFEQKESNGPVKVWGSIKGLTEGLHGFHVHEFGDNTAGCTSAGPHFNPLSRKHGGPKDEERHVGDLGNVTADKDGVADVSIEDSVISLSGDHCITGRTLVVHEKADDLGKGGNEESTKTGNAGSRLACGVIGIAQ

>3W07A

GSHMRSRRVDVMDVMNRLILAMDLMNRDDALRVTGEVREYIDTVKIGYPLVLSEGMDIIAEFRKRFGCRIIADFKVADIPETNEKICRATFKAGADAIIVHGFPGADSVRACLNVAEEMGREVFLLTEMSHPGAEMFIQGAADEIARMGVDLGVKNYVGPSTRPERLSRLREIIGQDSFLISPGVGAQGGDPGETLRFADAIIVGRSIYLADNPAAAAAGIIESIKDLRIPEDPAANKARKEAELAAATAEQ

>1YQ5A

GGVTDALSLMYSTSTGGPASIAANALTDFDLSGALTVNSVGTGLTKSAAGIQLAAGKSGLYQITMTVKNNTVTTGNYLLRVKYGSSDFVVACPASSLTAGGTISLLIYCNVLGVVSLDVLKFSLCNDGAALSNYIINITAAKIN

>1YKDA

VTEVEQKLQIVHQTLSMLDSHGFENILQEMLQSITLKTGELLGADRTTIFLLDEEKQELWSIVAAGEGDRSLEIRIPADKGIAGEVATFKQVVNIPFDFYHDPRSIFAQKQEKITGYRTYTMLALPLLSEQGRLVAVVQLLNKLKPYSPPDALLAERIDNQGFTSADEQLFQEFAPSIRLILESSRSFYIATQKQRAAAAMMKAVKSLSQSSLDLEDTLKRVMDEAKELMNADRSTLWLIDRDRHELWTKITQDNGSTKELRVPIGKGFAGIVAASGQKLNIPFDLYDHPDSATAKQIDQQNGYRTCSLLCMPVFNGDQELIGVTQLVNKKKTGEFPPYNPETWPIAPECFQASFDRNDEEFMEAFNIQAGVALQNAQLFATVKQQEQGSRSHHHHHH

>3D1RA

GMRRELAIEFSRVTESAALAGYKWLGRGDKNTADGAAVNAMRIMLNQVNIDGTIVIGEGEIAEAPMLYIGEKVGTGRGDAVDIAVDPIEGTRMTAMGQANALAVLAVGDKGCFLNAPDMYMEKLIVGPGAKGTIDLNLPLADNLRNVAAALGKPLSELTVTILAKPRHDAVIAEMQQLGVRVFAIPDGDVAASILTCMPDSEVDVLYGIGGAPEGVVSAAVIRALDGDMNGRLLARHDVKGDNEENRRIGEQELARCKAMGIEAGKVLRLGDMARSDNVIFSATGITKGDLLEGISRKGNIATTETLLIRGKSRTIRRIQSIHYLDRKDPEMQVHIL

>1K94A

SVYTYFSAVAGQDGEVDAEELQRCLTQSGINGTYSPFSLETCRIMIAMLDRDHTGKMGFNAFKELWAALNAWKENFMTVDQDGSGTVEHHELRQAIGLMGYRLSPQTLTTIVKRYSKNGRIFFDDYVACCVKLRALTDFFRKRDHLQQGSANFIYDDFLQGTMAI

>1XKIA

ASDEEIQDVSGTWYLKAMTVDREFPEMNLESVTPMTLTTLEGGNLEAKVTMLISGRCQEVKAVLEKTDEPGKYTADGGKHVAYIIRSHVKDHYIFYSEGELHGKPVRGVKLVGRDPKNNLEALEDFEKAAGARGLSTESILIPRQSETCSPGSAWSHPQFEK

>4G9FD

KITQTQPGMFVQEKEAVTLDCTYDTSDQSYGLFWYKQPSSGEMIFLIYQGSYDEQNATEGRYSLNFQKARKSANLVISASQLGDSAMYFCAMRDLRDNFNKFYFGSGTKLNVKPNIQNPDPAVYQLRDSKSSDKSVCLFTDFDSQTNVSQSKDSDVYITDKCVLDMRSMDFKSNSAVAWSNKSDFACANAFNNSIIPEDTFFPS

>3BT3A

SLENERLIKMSRFSERGYVVRENGPVYFTKDMDKTVKWFEEILGWSGDIVARDDEGFGDYGCVFDYPSEVAVAHLTPFRGFHLFKGEPIKGVAGFMMIEGIDALHKYVKENGWDQISDIYTQPWGARECSITTTDGCILRFFESIQEG

>1G6HA

MRDTMEILRTENIVKYFGEFKALDGVSISVNKGDVTLIIGPNGSGKSTLINVITGFLKADEGRVYFENKDITNKEPAELYHYGIVRTFQTPQPLKEMTVLENLLIGEICPGESPLNSLFYKKWIPKEEEMVEKAFKILEFLKLSHLYDRKAGELSGGQMKLVEIGRALMTNPKMIVMDEPIAGVAPGLAHDIFNHVLELKAKGITFLIIEHRLDIVLNYIDHLYVMFNGQIIAEGRGEEEIKNVLSDPKVVEIYIGE

>3L39A

MGSDKIHHHHHHMKNSFFSKFTPKEPKFFPLLKQLSDVLSASSVLLVESMEHDLPTERADYYKQIKDMEREGDRLTHLIFDELSTTFITPFDREDIHDLASCMDDVIDGINSSAKRIVIYNPRPISESGKELSRLIHEEAINIGKAMDELETFRKNPKPLRDYCTQLHDIENQADDVYELFITKLFEEEKDCIELIKIKEIMHELEKTTDAAEHVGKILKNLIVKYS

>3FFRA

GMNNKIYFTPGPSELYPTVRQHMITALDEKIGVISHRSKKFEEVYKTASDNLKTLLELPSNYEVLFLASATEIWERIIQNCVEKKSFHCVNGSFSKRFYEFAGELGREAYKEEAAFGKGFYPADITVPADAEIICLTHNETSSGVSMPVEDINTFRDKNKDALIFVDAVSSLPYPKFDWTKIDSVFFSVQKCFGLPAGLGVWILNDRVIEKSKALLAKRKSIGTYHTIPSMLEKARVNQTPETPNAMNIFLLGKVTGDMLQISADGIRKQTEEKAALINTYIESSKVFSFGVEDAKLRSMTTIVANTTMLPGEINKILEPFDMAVGAGYGSKKETQIRIANFPAHSLEQVHKLVQTLKEKIG

>3OYVA

GSDDDNPTVDPANIDYTPENASSWHNYMRNVAALLKTDATNLYNAWNSSYKGGESYASLFKAHSGSPYASALSCVEEIVDKCAEIANEVGTAKIGDPYNLYKAGNTEEALYAVESWYSWHSRDDYTNNIYSIRNAYYGSLDGNINANSLSTVIAGANSSLDTKIKNAIQKAAKAIQDIPQPFRNHIPSNETVAAMDACAELESILKNDLKSYIANNSNNINTDAVLNPVVTQYVDAVVVPTYKSLKEKNDALYNAVIVLADNPSNSAFETACDAWITAREPWEKSEAFLFGPVDEMGLDPNMDSWPLDQNAIVQILNSQSWSDLEWSEGDDEAAVESAQNVRGFHTLEFLLYKNGEPRKVQ

>1SWVA

MDRMKIEAVIFAWAGTTVDYGCFAPLEVFMEIFHKRGVAITAEEARKPMGLLKIDHVRALTEMPRIASEWNRVFRQLPTEADIQEMYEEFEEILFAILPRYASPINGVKEVIASLRERGIKIGSTTGYTREMMDIVAKEAALQGYKPDFLVTPDDVPAGRPYPWMCYKNAMELGVYPMNHMIKVGDTVSDMKEGRNAGMWTVGVILGSSELGLTEEEVENMDSVELREKIEVVRNRFVENGAHFTIETMQELESVMEHIEKQELIIS

>3TUFA

GPAMSPESKNAVQMQSEKSASDSGEVATEKAPAKQDTKEKSGTETEKGKEDGTKGTKDSSADKETSAEASEKGTVVTETADDDLFTTYRLDLEDARSKEREELNAIVSSDDATAKEKSEAYDKMTALSEVEGTEKQLETLIKTQGYEDALVNAEGDKINITVKSDKHSKSKATAIIDLVAKEIKTMKDVAVTFEPSK

>1R4PA

REFTIDFSTQQSYVSSLNSIRTEISTPLEHISQGTTSVSVINHTPPGSYFAVDIRGLDVYQARFDHLRLIIEQNNLYVAGFVNTATNTFYRFSDFTHISVPGVTTVSMTTDSSYTTLQRVAALERSGMQISRHSLVSSYLALMEFSGNTMTRDASRAVLRFVTVTAEALRFRQIQREFRQALSETAPVYTMTPGDVDLTLNWGRISNVLPEYRGEDGVRVGRISFNNISAILGTVAVILNCHHQGARSVRAVNEESQPECQITGDRPVIKINNTLWESNTAAAFLNRKSQFLYTTGK

>2WZPR

MLEANVYDNFNPNYYNISDFSMPNGKKEKRGLPIPKARCQVINYELWETGYLYTSSATLTVSVEVGDIVQILFPEVVPIEEALGKKKKLNLDMVYLVTDVDESNKATLKNYFWAMIESLDVPNAITKTTNFAIIDYLIDPNKNNLMSYGYFFNSSIFAGKATINRKAETSSAHDVAKRIFSKVQFQPTTTIQHAPSETDPRNLLFINFASRNWNRKRITTRVDIKQSVTMDTETIVERSAYNFAVVFVKNKATDDYTDPPKMYIAKNNGDVIDYSTYHGDGTDLPDVRTAKTLFYDRDDHGNPPELSTIKVEISPSTIVTRLIFNQNELLPLYVNDLVDIWYEGKLYSGYIADRVKTEFNDRLIFVESGDKPNVI

>4ACJA

GNEPSDLLEAEQIEKLAKHLPPRTIGYPWNLAFSTSKHGMSIKTLYRAMQDQDSPMLLVIKDSDGQIFGALASEPFKVSEGFYGTGETFLFTFYPEFEAYKWTGDNLFFIKGDMDSLAFGGGSGEFGLWLDGDLYHGRNHSCKTFGNPMLSMKEDFFVQDIEIWSFE

>3GNFB

GPLGMATEEAIIRIPPYHYIHVLDQNSNVSRVEVGPKTYIRQDNERVLFAPVRMVTVPPRHYCIVANPVSRDAQSSVLFDVTGQVRLRHADQEIRLAQDPFPLYPGELLEKDITPLQVVLPNTALHLKALLDFEDKNGDKVMAGDEWLFEGPGTYIPQKEVEVVEIIQATVIKQNQALRLRARKECFDRDGKERVTGEEWLVRSVGAYLPAVFEEVLDLVDAVILTEKTALHLRARQNFKDLRGVAHRTGEEWLVTVQDTEAHVPDVYEEVLGVVPITTLGPRHYCVILDPMGPDGKNQLGQKRVVKGEKSFFLQPGERLERGIQDVYVLSEQQGLLLKALQPLEEGEGEERVAHQAGDRWLIRGPLEYVPSAKVEVVEERQAIPLD

>4JX2A

GHTNNTNNKGLSASINNGVGSSSSNNTYVTPQAFWNLYFDFTGDETPGYPKGKINISQTLFQSEMKKNSSLAQQNEGQLILFINSTLYIYNSDRQLKLKQLMRTAPNSGFTEMTAISHIGPALMYLAKIKENGDASWKSQMENLLKDIQAVKVINAQTPNNWLEQVNAPAWKPHLTTIHNMIDYACSMAGNYMSDVLNEKLSFDMASLQNDFLNGNKTYPIPYNNVMIGTFMLTALQSMDQLHSKISQLKIDWPHAKVIIRFVAGSNVSAGVSKGSNWLVPFVQALSNNKLATDRIYITPYAAVKPSLGAQELTQADYNYYNNTVWGARHNRRIIANEVFTNITSIFLPDRPAIPGDYTYSKPPKIEDFLMRLKFSLAEPTEMLSNTVGFWMAGELAEKNWNYNKISIPGITTGFPEGISTYPNNNPVIQR

>4ACJA

GNEPSDLLEAEQIEKLAKHLPPRTIGYPWNLAFSTSKHGMSIKTLYRAMQDQDSPMLLVIKDSDGQIFGALASEPFKVSEGFYGTGETFLFTFYPEFEAYKWTGDNLFFIKGDMDSLAFGGGSGEFGLWLDGDLYHGRNHSCKTFGNPMLSMKEDFFVQDIEIWSFE

>3DHAA

GRISMTVKKLYFIPAGRCMLDHSSVNSALTPGKLLNLPVWCYLLETEEGPILVDTGMPESAVNNEGLFNGTFVEGQILPKMTEEDRIVNILKRVGYEPDDLLYIISSHLHFDHAGGNGAFTNTPIIVQRTEYEAALHREEYMKECILPHLNYKIIEGDYEVVPGVQLLYTPGHSPGHQSLFIETEQSGSVLLTIDASYTKENFEDEVPFAGFDPELALSSIKRLKEVVKKEKPIIFFGHDIEQEKSCRVFPEYI

>3K2IA

SMSATLILEPPGRCCWNEPVRIAVRGLAPEQRVTLRASLRDEKGALFRAHARYCADACGELDLERAPALGGSFAGLEPMGLLWALEPEKPFWRFLKRDVQIPFVVELEVLDGHDPEPGRLLCQAQHERHFLPPGVWRQSVRAGRVRATLFLPPGPGPFPGIIDIFGIGGGLLEYRASLLAGHGFATLALAYYNFEDLPNNMDNISLEYFEEAVCYMLQHPQVKGPGIGLLGISLGADICLSMASFLKNVSATVSINGSGISGNTAINYKHSSIPPLGYDLRRIKVAFSGLVDIVDIRNALVGGYKNPSMIPIEKAQGPILLIVGQDDHNWRSELYAQTVSERLQAHGKEKPQIICYPGTGHYIEPPYFPLCPASLHRLLNKHVIWGGEPRAHSKAQEDAWKQILAFFCKHLGGTQKTAVPKL

>4G8AA

RSPWDYKDDDDKLAAANSSIPESWEPCVEVVPNITYQCMELNFYKIPDNLPFSTKNLDLSFNPLRHLGSYSFFSFPELQVLDLSRCEIQTIEDGAYQSLSHLSTLILTGNPIQSLALGAFSGLSSLQKLVAVETNLASLENFPIGHLKTLKELNVAHNLIQSFKLPEYFSNLTNLEHLDLSSNKIQSIYCTDLRVLHQMPLLNLSLDLSLNPMNFIQPGAFKEIRLHKLTLRNNFDSLNVMKTCIQGLAGLEVHRLVLGEFRNEGNLEKFDKSALEGLCNLTIEEFRLAYLDYYLDGIIDLFNCLTNVSSFSLVSVTIERVKDFSYNFGWQHLELVNCKFGQFPTLKLKSLKRLTFTSNKGGNAFSEVDLPSLEFLDLSRNGLSFKGCCSQSDFGTISLKYLDLSFNGVITMSSNFLGLEQLEHLDFQHSNLKQMSEFSVFLSLRNLIYLDISHTHTRVAFNGIFNGLSSLEVLKMAGNSFQENFLPDIFTELRNLTFLDLSQCQLEQLSPTAFNSLSSLQVLNMSHNNFFSLDTFPYKCLNSLQVLDYSLNHIMTSKKQELQHFPSSLAFLNLTQNDFACTCEHQSFLQWIKDQRQLLVEVERMECATPSDKQGMPVLSLNITCQMTGHHHHHH

>2CWSA

MPAAAPGKNFDLSHWKLQLPDANTTEISSANLGLGYTSQYFYTDTDGAMTFWAPTTGGTTANSSYPRSELREMLDPSNSKVNWGWQGTHTMKLSGKTVQLPSSGKIIVAQIHGIMDDGTNAPPLVKAVFQDGQLDMQVKQNSDGTGSDVHNYFTGIKLGDLYNMEIRVTDGVAYVTMNGDTRSVDFVGKDAGWKNLKYYFKAGNYVQDNTSTGGSAIAKLYSLSVSHSNLEHHHHHH

>3HRGA

GMIDFTKSKQYTLSIRLSTDGFSFSIYNPINDNSQSLFEKEVDTSLSLTANLKNVFHESDFLSYSYKRVNIMIASKRFTMIPLELFEEEQAELLFYHNHQKRENEIVMYNILKKNNVVIIFGIDKSTYTFLNEQYPEARFYSQSTPLIEYFSIKSRLGNSKKMYASVRKDAIDIYCFERGQLLLANSFECMQTEDRIYYLLYVWKQLEFNQERDELHLTGTLSDKETLMNELKKFILQVFIMNPANNIDMQALLTCE

>3GA4A

MATASHNIDDILQLKDDTGVITVTADNYPLLSRGVPGYFNILYITMRGTNSNGMSCQLCHDFEKTYHAVADVIRSQAPQSLNLFFTVDVNEVPQLVKDLKLQNVPHLVVYPPAESNKQSQFEWKTSPFYQYSLVPENAENTLQFGDFLAKILNISITVPQAFNVQEEFHHHHHHHHHH

>3PQHA

GSFIPKIATATDSSEEVDSEKVIISNNKQTYASFDPNGNISVYNTQGMKIDMTPNSIVLTDAGGGKLTLQGGTMTYKGGTVNLNGLTITPDGRMTDSGGIGLHTHTHPVRGVETGGSTVTSDKPNGG

>1QMYA

MELTLYNGEKKTFYSRPNNHDNAWLNAILQLFRYVEEPFFDWVYSSPENLTLEAIKQLEDLTGLELHEGGPPALVIWNIKHLLHTGIGTASRPSEVCVVDGTDMSLADFHAGIFLKGQEHAVFACVTSNGWYAIDDEDFYPWTPDPSDVLVFVPYDQEPLNGEWKAK

>2BJIA

MADPWQECMDYAVTLAGQAGEVVREALKNEMNIMVKSSPADLVTATDQKVEKMLITSIKEKYPSHSFIGEESVAAGEKSILTDNPTWIIDPIDGTTNFVHGFPFVAVSIGFVVNKKMEFGIVYSCLEDKMYTGRKGKGAFCNGQKLQVSHQEDITKSLLVTELGSSRTPETVRIILSNIERLLCLPIHGIRGVGTAALNMCLVAAGAADAYYEMGIHCWDVAGAGIIVTEAGGVLLDVTGGPFDLMSRRVIASSNKTLAERIAKEIQIIPLQRDDED

>2WURA

MSKGEELFTGVVPILVELDGDVNGHKFSVSGEGEGDATYGKLTLKFICTTGKLPVPWPTLVTTLSYGVQCFSRYPDHMKRHDFFKSAMPEGYVQERTIFFKDDGNYKTRAEVKFEGDTLVNRIELKGIDFKEDGNILGHKLEYNYNSHNVYIMADKQKNGIKVNFKTRHNIEDGSVQLADHYQQNTPIGDGPVLLPDNHYLSTQSALSKDPNEKRDHMVLLEFVTAAGITHGMDELYN

>3I57A

MQKVHVQYIDGETDQMLRQDDLDGYTDETIPYSTAEGIKKFEGDGYELFKDNFPAGEKFDNDDTNDQFYTVIFKHHRENVDPNHSSADGTKGTKTLTETVHYKYANGTKAAEDQTAQVTFTRNGVLDDVTGIVAWGKWNEASQSYKALTSPTIAGYAPSEAVVKRSSNSDAEQGPTLTVIYTADA

>1U7LA

MATALYTANDFILISLPQNAQPVTAPGSKTDSWFNETLIGGRAFVSDFKIPEFKIGSLDTLIVESEELSKVDNQIGASIGKIIEILQGLNETSTNAYRTLPINNMPVPEYLENFQWQTRKFKLDKSIKDLITLISNESSQLDADVRATYANYNSAKTNLAAAERKKTGDLSVRSLHDIVKPEDFVLNSEHLTTVLVAVPKSLKSDFEKSYETLSKNVVPASASVIAEDAEYVLFNVHLFKKNVQEFTTAAREKKFIPREFNYSEELIDQLKKEHDSAASLEQSLRVQLVRLAKTAYVDVFINWFHIKALRVYVESVLRYGLPPHFNIKIIAVPPKNLSKCKSELIDAFGFLGGNAFMKDKKGKINKQDTSLHQYASLVDTEYEPFVMYIINL

>2IGSA

GHMAEINIYQNPGQSLANIYKGFARQCNPGFVFPEAQTIEAWDIPLRLHPEFIPGGDISKADQQYSTLLAQEIANGVTIGFRMVNEKERVCNVEILPLLTSMAQNLDRIKARFGSGYLDRFKGSPNVYPTDVGFSTDASGGISQESGLLVSYGVNLRTLTPGTWQAMTLPEDIKALVGPGVGLRLDAPNFSDVFNTIKSGLRYTTAVTLLLAYFAAIGS

>3MGAA

SNAMKEALATGSEAWWRTKTGPEWIREKDGNYRVTFWWRDPQGNETHSPIRRVWVYITGVTDHHQNAQPQTMARIAGTDVWRWSTALSANWRGSYCFIPTERDDVFAAFAPGETPDRNVLREGWRQLLPQAIADPLNSQSWRGGRGHAVSALEMPDAPLQPGWDRPETPYSPPLMMQWHSERLGNSRRVWILTTGDEAPEERPLAILLDGQFWAENMPVWPALASLTHQRLLPGAVYLLIDAIDTQHRSQELPCNADFWLAVQQELLPQVRAVTPFSDDAGRTVVAGQSFGGLSALYAGLNWPTRFGCVLSQSGSFWWPHRITPPEGEVITRLKTGALCARGLRIVLEAGVREPIVFQANQALYAQLNTSQQSIFWRQVDGGHDALCWRGGLTQGLMLLWQPLIDTL

>2PKEA

GMTPIAQRDGQAIQLVGFDGDDTLWKSEDYYRTAEADFEAILSGYLDLGDSRMQQHLLAVERRNLKIFGYGAKGMTLSMIETAIELTEARIEARDIQRIVEIGRATLQHPVEVIAGVREAVAAIAADYAVVLITKGDLFHQEQKIEQSGLSDLFPRIEVVSEKDPQTYARVLSEFDLPAERFVMIGNSLRSDVEPVLAIGGWGIYTPYAVTWAHEQDHGVAADEPRLREVPDPSGWPAAVRALDAQAGRQQ

>1F32A

QFLFSMSTGPFICTVKDNQVFVANLPWTMLEGDDIQVGKEFAARVEDCTNVKHDMAPTCTKPPPFCGPQDMKMFNFVGCSVLGNKLFIDQKYVRDLTAKDHAEVQTFREKIAAFEEQQENQPPSSGMPHGAVPAGGLSPPPPPSFCTVQ

>3FO8D

AVDRDTAKNSSPIAGNIEYTISTPGSNYAVGDKITVKYVSDDIETEGKITEVDADGKIKKINIPTAKIIAKAKEVGEYPTLGSNWTAEISSSSSGLAAVITLGKIITDSGILLAEIENAEAAMTAVDFQANLKKYGIPGVVALYPGELGDKIEIEIVSKADYAKGASALLPIYPGGGTRASTAKAVFGYGPQTDSQYAIIVRRNDAIVQSVVLSTKRGGKDIYDSNIYIDDFFAKGGSEYIFATAQNWPEGFSGILTLSGGLSSNAEVTAGDLMEAWDFFADR

>1QF8A

MSSSEEVSWISWFCGLRGNEFFCEVDEDYIQDKFNLTGLNEQVPHYRQALDMILDLEPDEELEDNPNQSDLIEQAAEMLYGLIHARYILTNRGIAQMLEKYQQGDFGYCPRVYCENQPMLPIGLSDIPGEAMVKLYCPKCMDVYTPKSSRHHHTDGAYFGTGFPHMLFMVHPEYRPKRPANQ

>3IX1A

ALETVEVMLDWYPNAVHTFLYVAIENGYFAEEGLDVDIVFPTNPTDPIQLTASGAIPLALSYQPDVILARSKDLPVVSVASVVRSPLNHVMFLAEQDFDSPADLVGLTVGYPGIPVNEPILKTMVEAAGGDYEQVHLMDVGFELGASIVSGRADAVVGTYINHEYPVLKHEGHDISYFNPVDYGVPEYDELVLISNEAYVEESGEVLAAFWRAALKGYEWMVENPDEALNVLLTNQDEANFPLIQEVEEESLSILLEKMENPNGPFGGQDAESWEEVISWLDAHDWLEQPVVAEDAFSSITD

>2D42A

AIINLLRELEIYGMQYANSHQYTYGSSYSDDTNPIRIAGLDARIPDPIVTDPVNHIVLDRRIITNTTSNSLEGVFSFSNAYTSRTSSQTRDGVTAGTNITGKYFANLFFEQVGLSGRIAFEGAVTNENKYTLDATQDFRDSQTIRVPPFHRATGVYTLEQGAFEKMTVLECVVSGNGIIRYYRTLPDNSYTEIVQRVNIIDVLQANGTPGFTISKEQNRAYFTGEGTISGQIGLQTFIDVVIEPLPGHA

>3FVSA

MAKQLQARRLDGIDYNPWVEFVKLASEHDVVNLGQGFPDFPPPDFAVEAFQHAVSGDFMLNQYTKTFGYPPLTKILASFFGELLGQEIDPLRNVLVTVGGYGALFTAFQALVDEGDEVIIIEPFFDCYEPMTMMAGGRPVFVSLKPGPIQNGELGSSSNWQLDPMELAGKFTSRTKALVLNTPNNPLGKVFSREELELVASLCQQHDVVCITDEVYQWMVYDGHQHISIASLPGMWERTLTIGSAGKTFSATGWKVGWVLGPDHIMKHLRTVHQNSVFHCPTQSQAAVAESFEREQLLFRQPSSYFVQFPQAMQRCRDHMIRSLQSVGLKPIIPQGSYFLITDISDFKRKMPDLPGAVDEPYDRRFVKWMIKNKGLVAIPVSIFYSVPHQKHFDHYIRFCFVKDEATLQAMDEKLRKWKVEL

>1Z40A

GNYMGNPWTEYMAKYDIEEVHGSGIRVDLGEDAEVAGTQYRLPSGKCPVFGKGIIIENSNTTFLTPVATGNQYLKDGGFAFPPTEPLMSPMTLDEMRHFYKDNKYVKNLDELTLCSRHAGNMIPDNDKNSNYKYPAVYDDKDKKCHILYIAAQENNGPRYCNKDESKRNSMFCFRPAKDISFQNYTYLSKNVVDNWEKVCPRKNLQNAKFGLWVDGNCEDIPHVNEFPAIDLFECNKLVFELSASDQPKQYEQHLTDYEKIKEGFKNKNASMIKSAFLPTGAFKADRYKSHGKGYNWGNYNTETQKCEIFNVKPTCLINNSSYIATTALSHPIEVE

>3GNFB

GPLGMATEEAIIRIPPYHYIHVLDQNSNVSRVEVGPKTYIRQDNERVLFAPVRMVTVPPRHYCIVANPVSRDAQSSVLFDVTGQVRLRHADQEIRLAQDPFPLYPGELLEKDITPLQVVLPNTALHLKALLDFEDKNGDKVMAGDEWLFEGPGTYIPQKEVEVVEIIQATVIKQNQALRLRARKECFDRDGKERVTGEEWLVRSVGAYLPAVFEEVLDLVDAVILTEKTALHLRARQNFKDLRGVAHRTGEEWLVTVQDTEAHVPDVYEEVLGVVPITTLGPRHYCVILDPMGPDGKNQLGQKRVVKGEKSFFLQPGERLERGIQDVYVLSEQQGLLLKALQPLEEGEGEERVAHQAGDRWLIRGPLEYVPSAKVEVVEERQAIPLD

>1RYPL

TTTLAFRFQGGIIVAVDSRATAGNWVASQTVKRVIEINPFLLGTMAGGAADCQFWETWLGSQCRLHELREKERISVAAASKILSNLVYQYKGAGLSMGTMICGYTRKEGPTIYYVDSDGTRLKGDIFCVGSGQTFAYGVLDSNYKWDLSVEDALYLGKRSILAAAHRDAYSGGSVNLYHVTEDGWIYHGNHDVGELFWKVKEEEGSFNNVIG

>3VXJA

ANDTILPLNNIQGDILVGMKKQKERFVFFQVNDATSFKTALKTYVPERITSAAILISDPSQQPLAFVNLGFSNTGLQALGITDDLGDAQFPDGQFADAANLGDDLSQWVAPFTGTTIHGVFLIGSDQDDFLDQFTDDISSTFGSSITQVQALSGSARPGDQAGHEHFGFLDGISQPSVTGWETTVFPGQAVVPPGIILTGRDGDTGTRPSWALDGSFMAFRHFQQKVPEFNAYTLANAIPANSAGNLTQQEGAEFLGARMFGRWKSGAPIDLAPTADDPALGADPQRNNNFDYSDTLTDETRCPFGAHVRKTNPRQDLGGPVDTFHAMRSSIPYGPETSDAELASGVTAQDRGLLFVEYQSIIGNGFRFQQINWANNANFPFSKPITPGIEPIIGQTTPRTVGGLDPLNQNETFTVPLFVIPKGGEYFFLPSISALTATIAA

>1JOVA

MKTTLLKTLTPELHLVQHNDIPVLHLKHAVGTAKISLQGAQLISWKPQNAKQDVLWLSEVEPFKNGNAIRGGVPICYPWFGGVKQPAHGTARIRLWQLSHYYISVHKVRLEFELFSDLNIIEAKVSMVFTDKCHLTFTHYGEESAQAALHTYFNIGDINQVEVQGLPETCFNSLNQQQENVPSPRHISENVDCIYSAENMQNQILDKSFNRTIALHHHNASQFVLWNPWHKKTSGMSETGYQKMLCLETARIHHLLEFGESLSVEISLKG

>1UUQA

MVAESNSAVAPTANVATSPAHEHFVRVNGGHFELQGKPYVITGVNMWYAAYLGAPNEVGDRDRLAKELDNLKAIGVNNLRVLAVSEKSEINSAVKPAVTNGFGNYDETLLQGLDYLLVELAKRDMTVVLYFNNFWQWSGGMTQYMAWIEGEPVQDPNVTNEWEAFMAKSASFYRSEKAQQEYRKTLEKIITRVNSINGKAYVDDATIMSWQLANEPRPGNSQTTAEEKQIYIDWVHAAAAYIKTLDAHHLVSSGSEGEMGSVNDMQVFIDAHATPDIDYLTYHMWIRNWSWFDKTKPAETWPSAWEKAQNYMRAHIDVAKQLNKPLVLEEFGLDRDMGSYAMDSTTEYRDNYFRGVFELMLASLEQGEPSAGYNIWAWNGYGRTTRANYWWQEGDDFMGDPPQEEQGMYGVFDTDTSTIAIMKEFNARFQPKLEHHHHHH

>2FSUA

MRGSHHHHHHGSGSMGMTLETAFMLPVQDAQHSFRRLLKAMSEPGVIVALHQLKRGWQPLNIATTSVLLTLADNDTPVWLSTPLNNDIVNQSLRFHTNAPLVSQPEQATFAVTDEAISSEQLNALSTGTAVAPEAGATLILQVASLSGGRMLRLTGAGIAEERMIAPQLPECILHELTERPHPFPLGIDLILTCGERLLAIPRTTHVEVC

>3A9LA

MAQTDTYPNIEALENAETVGVAYNIEVKRQNPSMIYFSPHAGGIEVGTTELIYRVVELTGGSLYLFQGLLPSGNSRLHVTSTHFDEPMAVCMLSKHTDAVSFHGYKDDYNKNTLVGGLNTELRNLIVSKLNSKGIAAEVATDRFTATDPDNIVNRCASGKGVQLEISSAQRRAFFQNNDWSKANRGNVTQEFLDYAEAIKEAEAEYYGLEHHHHHH

>3OV5A

MTSYTYQATPMDGTLKTMLERWAADSNMQLSYNLPSDYTLIGPVSAISTTSVQQAATELSAVYAAQGVSVSVSANKLLVQPVPVS

>2HLJA

GMPALITYRTTVQEDWVDYNGHLRDAFYLLIFSYATDALMDRIGLDADSRGQSGNSLFTLEAHINYLHEVKLGTEVWVQTQILGFDRKRLHVYHSLHRAGFDEVLAASEQMLLHVDLAGPQSAPFGHTTVCRLNHLVEQQEGAQAPQYMGRTIKLPA

>3TTGA

MGSSHHHHHHSSGRENLYFQGMLTEVSDTRIAHKKFGLFYPSVSRPSIFVEGEDRKNFLQGIASQDILKQDEKSLSYSFFLNPKARILFDAWCGNFEDKIALFPPAGTREEFVNHLKKYLFFRTKAKITDMSDHFREIRLVGPETISVLLSLFDNNFSGSSFRMLKNGGYVLIHPTSFQHNLDVGLQADLFIPIDQFETTQKSLEDFTSNKGGVLLDESSYLAYLTEKGIPLFPSELNDSFFPAEAGLDSVGVSYNKGCYVGQEPVTRLKFQGHLNRSLAGFRLEGGPFPKMEFPVTLFNPKDGNEAGILTRTSSSDILGSGIGLGYIKRNFSENGTELLLPDAQLVRVHSLPFV

>2FEXA

MTRIAIALAQDFADWEPALLAAAARSYLGVEIVHATPDGMPVTSMGGLKVTPDTSYDALDPVDIDALVIPGGLSWEKGTAADLGGLVKRFRDRDRLVAGICAAASALGGTGVLNDVAHTGNALASHKAYPAYRGEAHYRDQPRAVSDGGVVTAAGSAPVSFAVEILKSLGLFGPEAEAELQIFAAEHR

>2ZZVA

MKRVSRRAFLRRLGVGVAATAAFSPLAVAQARRYRWRIQTAWDAGTVGYSLFQKFTERVKELTDGQLEVQPFPAGAVVGTFDMFDAVKTGVLDGMNPFTLYWAGRMPVTAFLSSYALGLDRPDQWETWFYSLGGLDIARRAFAEQGLFYVGPVQHDLNIIHSKKPIRRFEDFKGVKLRVPGGMIAEVFAAAGASTVLLPGGEVYPALERGVIDAADFVGPAVNYNLGFHQVAKYIIMGPPETPAIHQPVDLMDFTINLNRWRSLPKPLQERFIAAVHEYSWIHYAGIQKANLEAWPKYRQAGVEVIRLSNEDVRKFRRLAIPIWFKWAKMDKYSREAFASQLEYMKGIGYVTDEELKGLSL

>2XVYA

GHGAPKAQKTGILLVAFGTSVEEARPALDKMGDRVRAAHPDIPVRWAYTAKMIRAKLRAEGIAAPSPAEALAGMAEEGFTHVAVQSLHTIPGEEFHGLLETAHAFQGLPKGLTRVSVGLPLIGTTADAEAVAEALVASLPADRKPGEPVVFMGHGTPHPADICYPGLQYYLWRLDPDLLVGTVEGSPSFDNVMAELDVRKAKRVWLMPLMAVAGDHARNDMAGDEDDSWTSQLARRGIEAKPVLHGTAESDAVAAIWLRHLDDALARLN

>2O7IA

MQVSLPREDTVYIGGALWGPATTWNLYAPQSTWGTDQFMYLPAFQYDLGRDAWIPVIAERYEFVDDKTLRIYIRPEARWSDGVPITADDFVYALELTKELGIGPGGGWDTYIEYVKAVDTKVVEFKAKEENLNYFQFLSYSLGAQPMPKHVYERIRAQMNIKDWINDKPEEQVVSGPYKLYYYDPNIVVYQRVDDWWGKDIFGLPRPKYLAHVIYKDNPSASLAFERGDIDWNGLFIPSVWELWEKKGLPVGTWYKKEPYFIPDGVGFVYVNNTKPGLSDPAVRKAIAYAIPYNEMLKKAYFGYGSQAHPSMVIDLFEPYKQYIDYELAKKTFGTEDGRIPFDLDMANKILDEAGYKKGPDGVRVGPDGTKLGPYTISVPYGWTDWMMMCEMIAKNLRSIGIDVKTEFPDFSVWADRMTKGTFDLIISWSVGPSFDHPFNIYRFVLDKRLSKPVGEVTWAGDWERYDNDEVVELLDKAVSTLDPEVRKQAYFRIQQIIYRDMPSIPAFYTAHWYEYSTKYWINWPSEDNPAWFRPSPWHADAWPTLFIISKKSDPQPVPSWLGTVDEGGIEIPTAKIFEDLQKATMHHHHHH

>2EX4A

MGSSHHHHHHSSGLVPRGSTSEVIEDEKQFYSKAKTYWKQIPPTVDGMLGGYGHISSIDINSSRKFLQRFLREGPNKTGTSCALDCGAGIGRITKRLLLPLFREVDMVDITEDFLVQAKTYLGEEGKRVRNYFCCGLQDFTPEPDSYDVIWIQWVIGHLTDQHLAEFLRRCKGSLRPNGIIVIKDNMAQEGVILDDVDSSVCRDLDVVRRIICSAGLSLLAEERQENLPDEIYHVYSFALR

>1EI9A

DPPAPLPLVIWHGMGDSCCNPLSMGAIKKMVEKKIPGIHVLSLEIGKTLREDVENSFFLNVNSQVTTVCQILAKDPKLQQGYNAMGFSQGGQFLRAVAQRCPSPPMVNLISVGGQHQGVFGLPRCPGESSHICDFIRKTLNAGAYNKAIQERLVQAEYWHDPIREDIYRNHSIFLADINQERGVNESYKKNLMALKKFVMVKFLNDTIVDPVDSEWFGFYRSGQAKETIPLQESTLYTQDRLGLKAMDKAGQLVFLALEGDHLQLSEEWFYAHIIPFLE

>2ERVA

ADVSAAVGATGQSGMTYRLGLSWDWDKSWWQTSTGRLTGYWDAGYTYWEGGDEGAGKHSLSFAPVFVYEFAGDSIKPFIEAGIGVAAFSGTRVGDQNLGSSLNFEDRIGAGLKFANGQSVGVRAIHYSNAGLKQPNDGIESYSLFYKIPI

>2VLQA

MELKHSISDYTEAEFLQLVTTICNADTSSEEELVKLVTHFEEMTEHPSGSDLIYYPKEGDDDSPSGIVNTVKQWRAANGKSGFKQG

>4DUIA

MRGSHHHHHHGSDLGKKLLEAARAGQDDEVRILMANGADVNATDASGLTPLHLAATYGHLEIVEVLLKHGADVNAIDIMGSTPLHLAALIGHLEIVEVLLKHGADVNAVDTWGDTPLHLAAIMGHLEIVEVLLKHGADVNAQDKFGKTAFDISIDNGNEDLAEILQKLN

>2UWAA

AYVQGPPSPGYYPSSQITSLGFDQGYTNLWGPQHQRVDQGSLTIWLDSTSGSGFKSINRYRSGYFGANIKLQSGYTAGVITSFYLSNNQDYPGKHDEIDIEFLGTIPGKPYTLQTNVFIEGSGDYNIIGREMRIHLWFDPTQDYHNYAIYWTPSEIIFFVDDVPIRRYPRKSDATFPLRPLWVYGSVWDASSWATENGKYKADYRYQPFVGKYEDFKLGSCTVEAASSCNPASVSPYGQLSQQQVAAMEWVQKNYMVYNYCDDPTRDHTLTPEC

>1OI7A

MILVNRETRVLVQGITGREGQFHTKQMLTYGTKIVAGVTPGKGGMEVLGVPVYDTVKEAVAHHEVDASIIFVPAPAAADAALEAAHAGIPLIVLITEGIPTLDMVRAVEEIKALGSRLIGGNCPGIISAEETKIGIMPGHVFKRGRVGIISRSGTLTYEAAAALSQAGLGTTTTVGIGGDPVIGTTFKDLLPLFNEDPETEAVVLIGEIGGSDEEEAAAWVKDHMKKPVVGFIGGRSAPKGKRMGHAGAIIMGNVGTPESKLRAFAEAGIPVADTIDEIVELVKKALG

>3KS7A

GAGGHKNLPAKGDLHIPVFENVNVRFSPDTYPDNYNEADGTGVYHLVNGRIILKKITLPEYKRNVSVSLKVTLASNGDRWDKSGSCFVLPKSSAINLLTIARDGMKFPSVDSLKLEKMVGIVPGKDYLPTVELMRFMTPFGIGHYSNNNDSLSSKRRPVYIPKWESNVTWQQDITDLYPLLEGEAYVGIYIDTWTSEGYLVNADIDVKESRLACDVLPKRHVEPLMNTVYYMGQSYPDIFARRDVSTDFTVPKGAKNIRLKYIVTGHGGHSGGDEFVQKRNIISVDGKEVLNFIPWRDDCASFRRFNPATGVWLIKRLASYIGEKGYTEKEVEEPLASSDLSRSNWCPGSDVVPEEAVIGTLAPGKHTFTVSIPEAQAVDGNKLNHWLVSAYLVWEE

>1YQHA

SNAMSQQVTMSFSVVPQAKTKDVYSVVDKAIEVVQQSGVRYEVGAMETTLEGELDVLLDVVKRAQQACVDAGAEEVITSIKIHYRPSTGVTIDEKVWKYRDEYAKPEAI

>4K6JA

GPLGSGRPELYTVVQHVKHFNDVVEFGENQEFTDDIEYLLSGLKSTQPLNTRCLSVISLATKCAMPSFRMHLRAHGMVAMVFKTLDDSQHHQNLSLCTAALMYILSRDRLNMDLDRASLDLMIRLLELEQDASSAKLLNEKDMNKIKEKIRRLCETVHNKHLDLENITTGHLAMETLLSLTSKRAGDWFKEELRLLGGLDHIVDKVKECVDHLSRDEDEEKLVASLWGAERCLRVLESVTVHNPENQSYLIAYKDSQLIVSSAKALQHCEELIQQYNRAEDSICLADSKPLPHQNVTNHVGKAVEDCMRAIIGVLLNLTNDNEWGSTKTGEQDGLIGTALNCVLQVPKYLPQEQRFDIRVLGLGLLINLVEYSARNRHCLVNMETSCSFDSSICSGEGDDSLRIGGQVHAVQALVQLFLERERAAQLAESKTDELIKDAPTTQHDKSGEWQETSGEIQWVSTEKTDGTEEKHKKEEEDEELDLNKALQHAGKHMEDCIVASYTALLLGCLCQESPINVTTVREYLPEGDFSIMTEMLKKFLSFMNLTCAVGTTGQKSISRVIEYLEHC

>3TC8A

GKETGNMPEIKKQPIASAVPDFNADSAYAYVANQVAFGPRVPNTAAHKACGDYLASELKRFGAKVYQQEAILTAYDGTKLEARNIIGSFDPENSKRVLLFAHWDSRPYSDHDPDPSKHRTPLDGADDGGSGVGALLEIARQIGQKAPGIGIDIIFFDAEDYGTPEFVTDYTPDSWCLGTQFWAKNPHVPNYTAEYGILLDMVGGKNATFFKEQQSLRAAAPIVEMVWSAARDLGYGKYFINAAGGAITDDHQYVISGRNIPSIDIINYDPESKTGFASYWHTQKDNMENIDRETLKAAGQTVLEVIYNR

>4JN3A

GMSENSSVRHGLTSAQHEVWLAQQLDPRGAHYRTGSCLEIDGPLDHAVLSRALRLTVAGTETLCSRFLTDEEGRPYRAYCPPAPEGSAAVEDPDGVPYTPVLLRHIDLSGHEDPEGEAQRWMDRDRATPLPLDRPGLSSHALFTLGGGRHLYYLGVHHIVIDGTSMALFYERLAEVYRALRDGRAVPAAAFGDTDRMVAGEEAYRASARYERDRAYWTGLFTDRPEPVSLTGRGGGRALAPTVRSLGLPPERTEVLGRAAEATGAHWARVVIAGVAAFLHRTTGARDVVVSVPVTGRYGANARITPGMVSNRLPLRLAVRPGESFARVVETVSEAMSGLLAHSRFRGEDLDRELGGAGVSGPTVNVMPYIRPVDFGGPVGLMRSISSGPTTDLNIVLTGTPESGLRVDFEGNPQVYGGQDLTVLQERFVRFLAELAADPAATVDEVALLT

>2WE3A

GAMGSGIPMEACPHIRYAFQNDKLLLQQASVGRLTLVNKTTILLRPMKTTTVDLGLYARPPEGHGLMLWGSTSRPVTSHVGIIDPGYTGELRLILQNQRRYNSTLRPSELKIHLAAFRYATPQMEEDKGPINHPQYPGDVGLDVSLPKDLALFPHQTVSVTLTVPPPSIPHHRPTIFGRSGLAMQGILVKPCRWRRGGVDVSLTNFSDQTVFLNKYRRFCQLVYLHKHHLTSFYSPHSDAGVLGPRSLFRWASCTFEEVPSLAM

>3DYJA

GIDPFTGIDPFTGTQACITAASAVSGIIADLDTTIMFATAGTLNREGAETFADHREGILKTAKVLVEDTKVLVQNAAGSQEKLAQAAQSSVATITRLADVVKLGAASLGAEDPETQVVLINAVKDVAKALGDLISATKAAAGKVGDDPAVWQLKNSAKVMVTNVTSLLKTVKAVEDEATKGTRALEATTEHIRQELAVFCSPEPPAKTSTPEDFIRMTKGITMATAKAVAAGNSCRQEDVIATANLSRRAIADMLRACKEAAFHPEVAPDVRLRALHYGRECANGYLELLDHVLLTLQKPNPDLKQQLTGHSKRVAGSVTELIQAAEAMKGT

>2OIZA

REVLTGGHSVSAPQENRIYVMDSVFMHLTESRVHVYDYTNGKFLGMVPTAFNGHVQVSNDGKKIYTMTTYHERITRGKRSDVVEVWDADKLTFEKEISLPPKRVQGLNYDGLFRQTTDGKFIVLQNASPATSIGIVDVAKGDYVEDVTAAAGCWSVIPQPNRPRSFMTICGDGGLLTINLGEDGKVASQSRSKQMFSVKDDPIFIAPALDKDKAHFVSYYGNVYSADFSGDEVKVDGPWSLLNDEDKAKNWVPGGYNLVGLHRASGRMYVFMHPDGKEGTHKFPAAEIWVMDTKTKQRVARIPGRDALSMTIDQQRNLMLTLDGGNVNVYDISQPEPKLLRTIEGAAEASLQVQFHPVGGT

>2ZZJA

TRSFYNDGHLNGWDYVRKENQGTVSEVSNVVFKGTSALKMTQTYTPGYTGRYHSEVDHNRGYQRGEEQFYGFAFRLSEDWQFQPQSYNIAQFIANRPGAGCGGDDWMPSTMIWIQNNQLYSRYVNGHYRQPNCGRNIVTRPNLATVSAGAWHRVVLQIKWASDNTGYFKIWFDGAKVHEEYNVATTVDDDSVFQFRVGLYANSWHDDGHMTGTQGFRQVWYDEVAVGTTFADVDPDQA

>1Q8DA

ERPNCLSLQDSCKTNYICRSRLADFFTNCQPESRSVSNCLKENYADCLLAYSGLIGTVMTPNYVDSSSLSVAPWCDCSNSGNDLEDCLKFLNFFKDNTCLKNAIQAFG

>3D2OA

MNAIADVQSSRDLRNLPINQVGIKDLRFPITLKTAEGTQSTVARLTMTVYLPAEQKGTHMSRFVALMEQHTEVLDFAQLHRLTAEMVALLDSRAGKISVSFPFFRKKTAPVSGIRSLLDYDVSLTGEMKDGAYGHSMKVMIPVTSLCPCSKEISQYGAHNQRSHVTVSLTSDAEVGIEEVIDYVETQASCQLYGLLKRPDEKYVTEKAYENPKFVEDMVRDVATSLIADKRIKSFVVESENFESIHNHSAYAYIAYP

>1T1JA

NLYFQGHMRKIFLACPYSHADAEVVEQRFRACNEVAATIVRAGHVVFSQVSMSHPINLCLAELDRAAIGRLWAPVDAFYMDHLEELIVLDLPGWRDSAGIRREMEFFEAGGQRVSLWSEVEHEFR

>3JUMA

MGSSHHHHHHSSGLVPRGSHMSDVESLENTSENRAQVAARQHNRKIVEQYMHTRGEARLKRHLLFTEDGVGGLWTTDSGQPIAIRGREKLGEHAVWSLQCFPDWVWTDIQIFETQDPNWFWVECRGEGAIVFPGYPRGQYRNHFLHSFRFENGLIKEQREFMNPCEQFRSLGIEVPEVRRDGLPS

>1XDYA

DLLSWFKGNDRPPAPAGKALEFSKPAAWQNNLPLTPADKVSGYNNFYEFGLDKADPAANAGSLKTDPWTLKISGEVAKPLTLDHDDLTRRFPLEERIYRMRCVEAWSMVVPWIGFPLHKLLALAEPTSNAKYVAFETIYAPEQMPGQQDRFIGGGLKYPYVEGLRLDEAMHPLTLMTVGVYGKALPPQNGAPVRLIVPWKYGFKGIKSIVSIKLTRERPPTTWNLAAPDEYGFYANVNPYVDHPRWSQATERFIGSGGILDVQRQPTLLFNGYADQVASLYRGLDLRENFLEHHHHHH

>3C2QA

SNANIPEIENANLKPALKDSVLPDGFYSTTNHPTHVKVNDEWIEVANPKMDAVIVVYPEEKRAETKVIRKVKKGDFVLIGHNGIRVMPPEKSREAGQLFEFMNSEVSSEKPKEAIIKRIAKEMHEIREEYKKTGTGGIAIVGGPAIIHTGGGPALAKMVELGYIQAILAGNALATHDIESALYGTSLGVNIKTAKPVTGGHKHHIYAINAINDAGNIKNAVESGVLKEGIMYQCIKNNIPYVLAGSIRDDGPIPDVITDSMVAQDKMRTTVMDKKMVIMLSTLLHSVATGNLMPSYIKTVCVDIQPSTVTKLMDRGTSQAIGVVTDVGVFLVLLLKELERLELQE

>2UWAA

AYVQGPPSPGYYPSSQITSLGFDQGYTNLWGPQHQRVDQGSLTIWLDSTSGSGFKSINRYRSGYFGANIKLQSGYTAGVITSFYLSNNQDYPGKHDEIDIEFLGTIPGKPYTLQTNVFIEGSGDYNIIGREMRIHLWFDPTQDYHNYAIYWTPSEIIFFVDDVPIRRYPRKSDATFPLRPLWVYGSVWDASSWATENGKYKADYRYQPFVGKYEDFKLGSCTVEAASSCNPASVSPYGQLSQQQVAAMEWVQKNYMVYNYCDDPTRDHTLTPEC

>3QP4A

GSHMRPLPAGLTASQQWTLLEWIHMAGHIETENELKAFLDQVLSQAPSERLLLALGRLNNQNQIQRLERVLNVSYPSDWLDQYMKENYAQHDPILRIHLGQGPVMWEERFNRAKGAEEKRFIAEATQNGMGSGITFSAASERNNIGSILSIAGREPGRNAALVAMLNCLTPHLHQAAIRVAN

>1NYCA

GSMYQLQFINLVYDTTKLTHLEQTNINLFIGNWSNHQLQKSICIRHGDDTSHNQYHILFIDTAHQRIKFSSFDNEEIIYILDYDDTQHILMQTSSKQGIGTSRPIVYERLV

>3A6FA

MSKSVFVGELTWKEYEARVAAGDCVLMLPVGALEQHGHHMCMNVDVLLPTAVCKRVAERIGALVMPGLQYGYKSQQKSGGGNHFPGTTSLDGATLTGTVQDIIRELARHGARRLVLMNGHYENSMFIVEGIDLALRELRYAGIQDFKVVVLSYWDFVKDPAVIQQLYPEGFLGFDIEHGGVFETSLMLALYPDLVDLDRVVDHPPATFPPYDVFPVDPARTPAPGTLSSAKTASREKGELILEVCVQGIADAIREEFPPT

>2ZF9A

MTHHHHHHAMGPAAGQAYDAGNLDVASSPVKPTLSITKKTLTAAEAPNAKVTMELSVEGAADKYAATGLHIQFDPKLKLIPDEDGALATAGRAARLLELKKAEADTDNSFFTATGSSTNNGKDGVLWSFVLQVPADAQPGDKYDVQVAYQSRTTNEDLFTNVKKDEEGLLMQAWTFTQGIEQGYIQVESTTSLE

>3QQZA

SNASNHAASFQNYHATIDGKEIAGITNNISSLTWSAQSNTLFSTINKPAAIVEMTTNGDLIRTIPLDFVKDLETIEYIGDNQFVISDERDYAIYVISLTPNSEVKILKKIKIPLQESPTNCGFEGLAYSRQDHTFWFFKEKNPIEVYKVNGLLSSNELHISKDKALQRQFTLDDVSGAEFNQQKNTLLVLSHESRALQEVTLVGEVIGEMSLTKGSRGLSHNIKQAEGVAMDASGNIYIVSEPNRFYRFTPQSSH

>4A0DA

GSSPDKKWLGTPIEEMRRMPRCGIRLPLLRPSANHTVTIRVDLLRAGEVPKPFPTHYKDLWDNKHVKMPCSEQNLYPVEDENGERTAGSRWELIQTALLNKFTRPQNLKDAILKYNVAYSKKWDFTALIDFWDKVLEEAEAQHLYQSILPDMVKIALCLPNICTQPIPLLAAAMNHSITMSQEQIASLLANAFFCTFPRRNAKMKSEYSSYPDINFNRLFEGRSSRKPEKLKTLFCYFRRVTAAAPTGLVTFTRQSLEDFPEWERCEKPLTRLHVTYEGTIEENGQGMLQVDFANRFVGGGVTSAGLVQEEIRFLINPELIISRLFTEVLDHNECLIITGTEQYSEYTGYAETYRWSRSHEDGSERDDWQRRCTEIVAIDALHFRRYLDQFVPEKMRRELNKAYCGFLRPGVSSENLSAVATGNWGCGAFGGDARLKALIQILAAAAAERDVVYFTFGDSELMRDIYSMHIFLTERKLTVGDVYKLLLRYYNEECRNCSTPGPDIKLYPFIYHAVESCAETADHSGQRTGT

>1KF6C

TTKRKPYVRPMTSTWWKKLPFYRFYMLREGTAVPAVWFSIELIFGLFALKNGPEAWAGFVDFLQNPVIVIINLITLAAALLHTKTWFELAPKAANIIVKDEKMGPEPIIKSLWAVTVVATIVILFVALYW

>4HSPA

GAPRELTWSQLIPAGAPPAPAPLPIHDLANALSEAGPAASQQSPNAPVVKALDGIEAKLPGYIVPLEISEAGLVTEFLLVPYYGACIHVPPPPSNQIVYVKTAKGVQMDELYQPFWVEGTFKVENASSELAAAGYRMQASKVTPYEYEGG

>4I66A

SNASAFRFGQLALGDRWDIYPQALSRMSREIDKRTSIEAAREPAAVTLSSPTLHETPFLYLAGDREFAIPPEPEVEALRRHLTFGGFLLIDSAEGALGGAFDRSVRRLLQAVFPAPAPGLEIVSGEHVVFKSFYLLERPLGRLALSPVMEGILRDGRLMVAYVQNDLGGAFARDDFGNFQLACVPDGERQRELAFRMLVNLVMYALCLD

>2RFEE

LSPSNSRTPSPKSLPSYLNGVMPPTQSFAPDPKYVSSKAL

>2FELA

MSLSPFEHPFLSGLFGDSEIIELFSAKADIDAMIRFETALAQAEAEASIFADDEAEAIVSGLSEFAADMSALRHGVAKDGVVVPELIRQMRAAVAGQAADKVHFGATSQDVIDTSLMLRLKMAAEIIATRLGHLIDTLGDLASRDGHKPLTGYTRMQAAIGITVADRAAGWIAPLERHLLRLETFAQNGFALQFGGAAGTLEKLGDNAGAVRADLAKRLGLADRPQWHNQRDGIAEFANLLSLVTGTLGKFGQDIALMAEIGSEIRLSGGGGSSAMPHKQNPVNAETLVTLARFNAVQISALHQSLVQEQERSGAGWMLEWLTLPQMVTATGTSLLVAERLAAQIDRLGADESHHHHHH

>2GDMA

GALTESQAALVKSSWEEFNANIPKHTHRFFILVLEIAPAAKDLFSFLKGTSEVPQNNPELQAHAGKVFKLVYEAAIQLEVTGVVVTDATLKNLGSVHVSKGVADAHFPVVKEAILKTIKEVVGAKWSEELNSAWTIAYDELAIVIKKEMDDAA

>4JHMA

MSLEDCRISRVELYALADENAPPIPWADNQEPLLYTNNIVRLFTEDGTEGLGATMSYTENFFDRCIIESLRTIVPGLIGKNPLMTQELNNWLGARCTWGGLPAKSPIDIAAWDIKGKKAGMPLYMLLGGARTKIKSYASTPMFDTVEEYFPYIDDCIEHGFTAIKLHCYCVYDKDVALVEAVEAKYGTSGIRFMLDTAGFYTPEQAMKMAKWMERHNWEWLEAPVSDYDFKTYQRLVANTDLEISSHGNCLLTLQEVTHALSTGMWSDVRQDATVCGGITQLNKCFAIAAGHSKNLEIQSMGYTLTQAANLHVALAHDNCNFFEQFYPYEAFELASKTQIRTDKEGYVHAPAGNGLGVEMDWDAVKEASFASYVFEEGHHHHHH

>3S8GA

MHHHHHHHAVRASEISRVYEAYPEKKATLYFLVLGFLALIVGSLFGPFQALNYGNVDAYPLLKRLLPFVQSYYQGLTLHGVLNAIVFTQLFAQAIMVYLPARELNMRPNMGLMWLSWWMAFIGLVVFALPLLANEATVLYTFYPPLKGHWAFYLGASVFVLSTWVSIYIVLDLWRRWKAANPGKVTPLVTYMAVVFWLMWFLASLGLVLEAVLFLLPWSFGLVEGVDPLVARTLFWWTGHPIVYFWLLPAYAIIYTILPKQAGGKLVSDPMARLAFLLFLLLSTPVGFHHQFADPGIDPTWKMIHSVLTLFVAVPSLMTAFTVAASLEFAGRLRGGRGLFGWIRALPWDNPAFVAPVLGLLGFIPGGAGGIVNASFTLDYVVHNTAWVPGHFHLQVASLVTLTAMGSLYWLLPNLTGKPISDAQRRLGLAVVWLWFLGMMIMAVGLHWAGLLNVPRRAYIAQVPDAYPHAAVPMVFNVLAGIVLLVALLLFIYGLFSVLLSRERKPELAEAPLPFAEVISGPEDRRLVLAMDRIGFWFAVAAILVVLAYGPTLVQLFGHLNPVPGWRLW

>2EABA

MVIASVEDGGDGDTSKDDWLWYKQPASQTDATATAGGNYGNPDNNRWQQTTLPFGNGKIGGTVWGEVSRERVTFNEETLWTGGPGSSTSYNGGNNETKGQNGATLRALNKQLANGAETVNPGNLTGGENAAEQGNYLNWGDIYLDYGFNDTTVTEYRRDLNLSKGKADVTFKHDGVTYTREYFASNPDNVMVARLTASKAGKLNFNVSMPTNTNYSKTGETTTVKGDTLTVKGALGNNGLLYNSQIKVVLDNGEGTLSEGSDGASLKVSDAKAVTLYIAAATDYKQKYPSYRTGETAAEVNTRVAKVVQDAANKGYTAVKKAHIDDHSAIYDRVKIDLGQSGHSSDGAVATDALLKAYQRGSATTAQKRELETLVYKYGRYLTIGSSRENSQLPSNLQGIWSVTAGDNAHGNTPWGSDFHMNVNLQMNYWPTYSANMGELAEPLIEYVEGLVKPGRVTAKVYAGAETTNPETTPIGEGEGYMAHTENTAYGWTAPGQSFSWGWSPAAVPWILQNVYEAYEYSGDPALLDRVYALLKEESHFYVNYMLHKAGSSSGDRLTTGVAYSPEQGPLGTDGNTYESSLVWQMLNDAIEAAKAKGDPDGLVGNTTDCSADNWAKNDSGNFTDANANRSWSCAKSLLKPIEVGDSGQIKEWYFEGALGKKKDGSTISGYQADNQHRHMSHLLGLFPGDLITIDNSEYMDAAKTSLRYRCFKGNVLQSNTGWAIGQRINSWARTGDGNTTYQLVELQLKNAMYANLFDYHAPFQIDGNFGNTSGVDEMLLQSNSTFTDTAGKKYVNYTNILPALPDAWAGGSVSGLVARGNFTVGTTWKNGKATEVRLTSNKGKQAAVKITAGGAQNYEVKNGDTAVNAKVVTNADGASLLVFDTTAGTTYTITKKAS

>2YIZA

SNHTYRVIEIVGTSPDGVDAAIQGGLARAAQTMRALDWFEVQSIRGHLVDGAVAHFQVTMKVGFRLEDS

>2UVKA

SVLPETPVPFKSGTGAIDNDTVYIGLGSAGTAWYKLDTQAKDKKWTALAAFPGGPRDQATSAFIDGNLYVFGGIGKNSEGLTQVFNDVHKYNPKTNSWVKLMSHAPMGMAGHVTFVHNGKAYVTGGVNQNIFNGYFEDLNEAGKDSTAIDKINAHYFDKKAEDYFFNKFLLSFDPSTQQWSYAGESPWYGTAGAAVVNKGDKTWLINGEAKPGLRTDAVFELDFTGNNLKWNKLAPVSSPDGVAGGFAGISNDSLIFAGGAGFKGSRENYQNGKNYAHEGLKKSYSTDIHLWHNGKWDKSGELSQGRAYGVSLPWNNSLLIIGGETAGGKAVTDSVLITVKDNKVTVQNLEHHHHHH

>1ERZA

TRQMILAVGQQGPIARAETREQVVVRLLDMLTKAASRGANFIVFPELALTTFFPRWHFTDEAELDSFYETEMPGPVVRPLFEKAAELGIGFNLGYAELVVEGGVKRRFNTSILVDKSGKIVGKYRKIHLPGHKEYEAYRPFQHLEKRYFEPGDLGFPVYDVDAAKMGMFICNDRRWPEAWRVMGLRGAEIICGGYNTPTHNPPVPQHDHLTSFHHLLSMQAGSYQNGAWSAAAGKVGMEENCMLLGHSCIVAPTGEIVALTTTLEDEVITAAVDLDRCRELREHIFNFKQHRQPQHYGLIAEL

>2YHCA

DNPPNEIYATAQQKLQDGNWRQAITQLEALDNRYPFGPYSQQVQLDLIYAYYKNADLPLAQAAIDRFIRLNPTHPNIDYVMYMRGLTNMALDDSALQGFFGVDRSDRDPQQARAAFSDFSKLVRGYPNSQYTTDATKRLVFLKDRLAKYEYSVAEYYTERGAWVAVVNRVEGMLRDYPDTQATRDALPLMENAYRQMQMNAQAEKVAKIIAANSSNTLEHHHHHH

>3BN8A

MGSDKIHHHHHHMSEAKELIKKMCDLQNSNEEIQKEMAGWSGVVQYKLDGYYFYVEYKSDGTCEFKEGVHSSPTFTVVAPPDFWLAVLKGQEDPVSGFMMGKYRIEGNIMEAQRLAGVIKKFQGKFEL

>1PM4A

LRIPNIATYTGTIQGKGEVCIIGNKEGKTRGGELYAVLHSTNVNADMTLILLRNVGGNGWGEIKRNDIDKPLKYEDYYTSGLSWIWKIKNNSSETSNYSLDATVHDDKEDSDVLTKCPV

>3DNHA

GHMLDVAPPVITPRGTKIEPSAGAPFEAVRVARDVLHTSRTAALATLDPVSGYPYTTATNIGIEPDGTPFFFAAGLTLHARNMETDARISVTLAPFGKGDALTLPRLTLVGRADRIGPDEVPLAIARYIARYPKAKLYLSLPDTRLYRLRTEGVQINGGPARNASNITPADLRTDLSGAEELMAAAESEATRLNAIKGEASRLAVLAGAKTGRWKITSIDPDGIDLASASDLARLWFAERVETLKQFEKALAQLLKGS

>3HR0A

GSQQGKFDTKGIESTDEAKMSFLVTLNNVEVCSENISTLKKTLESDCTKLFSQGIGGEQAQAKFDSCLSDLAAVSNKFRDLLQEGLTELNSTAIKPQVQPWINSFFSVSHNIEEEEFNDYEANDPWVQQFILNLEQQMAEFKASLSPVIYDSLTGLMTSLVAVELEKVVLKSTFNRLGGLQFDKELRSLIAYLTTVTTWTIRDKFARLSQMATILNLERVTEILDYWGPNSGPLTWRLTPAEVRQVLALRIDFRSEDIKRLRL

>3ATSA

TLPAVISRWLSSVLPGGAAPEVTVESGVDSTGMSSETIILTARWQQDGRSIQQKLVARVAPAAEDVPVFPTYRLDHQFEVIRLVGELTDVPVPRVRWIETTGDVLGTPFFLMDYVEGVVPPDVMPYTFGDNWFADAPAERQRQLQDATVAALATLHSIPNAQNTFSFLTQGRTSDTTLHRHFNWVRSWYDFAVEGIGRSPLLERTFEWLQSHWPDDAAAREPVLLWGDARVGNVLYRDFQPVAVLDWEMVALGPRELDVAWMIFAHRVFQELAGLATLPGLPEVMREDDVRATYQALTGVELGDLHWFYVYSGVMWACVFMRTGARRVHFGEIEKPDDVESLFYHAGLMKHLLGEEH

>3UB6A

GSKVMQKDVLAQLMEHLETGQYKKREKTLAYMTKILEQGIHEYYKSFDNDTARKMALDYFKRINDDKGMIYMVVVDKNGVVLFDPVNPKTVGQSGLDAQSVDGVYYVRGYLEAAKKGGGYTYYKMPKYDGGVPEKKFAYSHYDEVSQMVIAATSYYTDINTENKAIKEGVNKVFNENTTRL

>3HWWA

MSVSAFNRRWAAVILEALTRHGVRHICIAPGSRSTLLTLAAAENSAFIHHTHFDERGLGHLALGLAKVSKQPVAVIVTSGTAVANLYPALIEAGLTGEKLILLTADRPPELIDCGANQAIRQPGMFASHPTHSISLPRPTQDIPARWLVSTIDHALGTLHAGGVHINCPFAEPLYGEMDDTGLSWQQRLGDWWQDDKPWLREAPRLESEKQRDWFFWRQKRGVVVAGRMSAEEGKKVALWAQTLGWPLIGDVLSQTGQPLPCADLWLGNAKATSELQQAQIVVQLGSSLTGKRLLQWQASCEPEEYWIVDDIEGRLDPAHHRGRRLIANIADWLELHPAEKRQPWCVEIPRLAEQAMQAVIARRDAFGEAQLAHRICDYLPEQGQLFVGNSLVVRLIDALSQLPAGYPVYSNRGASGIDGLLSTAAGVQRASGKPTLAIVGDLSALYDLNALALLRQVSAPLVLIVVNNNGGQIFSLLPTPQSERERFYLMPQNVHFEHAAAMFELKYHRPQNWQELETAFADAWRTPTTTVIEMVVNDTDGAQTLQQLLAQVSHL

>2YGBA

MAHHHHHHSSGLEVLFQGPNNTIINSLIGGDDSIKRSNVFAVDSQIPTLYMPQYISLSGVMTNDGPDNQAIASFEIRDQYITALNHLVLSLELPEVKGMGRFGYVPYVGYKCINHVSISSCNGVIWEIEGEELYNNCINNTIALKHSGYSSELNDISIGLTPNDTIKEPSTVYVYIKTPFDVEDTFSSLKLSDSKITVTVTFNPVSDIVIRDSSFDFETFNKEFVYVPELSFIGYMVKNVQIKPSFIEKPRRVIGQINQPTATVTEVHAATSLSVYTKPYYGNTDNKFISYPGYSQDEKDYIDAYVSRLLDDLVIVSDGPPTGYPESAEIVEVPEDGIVSIQDADVYVKIDNVPDNMSVYLHTNLLMFGTRKNSFIYNISKKFSAITGTYSDATKRTIFAHISHSINIIDTSIPVSLWTSQRNVYNGDNRSAESKAKDLFINDPFIKGIDFKNKTDIISRLEVRFGNDVLYSENGPISRIYNELLTKSNNGTRTLTFNFTPKIFFRPTTITANVSRGKDKLSVRVVYSTMDVNHPIYYVQKQLVVVCNDLYKVSYDQGVSITKIMGDNN

>3QXFA

ACTWPAWEQFKKDYISQEGRVIDPSDARKITTSEGQSYGMFSALAANDRAAFDNILDWTQNNLAQGSLKERLPAWLWGKKENSKWEVLDSNSASDGDVWMAWSLLEAGRLWKEQRYTDIGSALLKRIAREEVVTVPGLGSMLLPGKVGFAEDNSWRFNPSYLPPTLAQYFTRFGAPWTTLRETNQRLLLETAPKGFSPDWVRYEKDKGWQLKAEKTLISSYDAIRVYMWVGMMPDSDPQKARMLNRFKPMATFTEKNGYPPEKVDVATGKAQGKGPVGFSAAMLPFLQNRDAQAVQRQRVADNFPGSDAYYNYVLTLFGQGWDQHRFRFSTKGELLPDWGQECANSHLEHHHHHH

>1LYQA

AHPELKSSVPQADSAVAAPEKIQLNFSENLTVKFSGAKLTMTGMKGMSSHSPMPVAAKVAPGADPKSMVIIPREPLPAGTYRVDWRAVSSDTHPITGNYTFTVK

>3CNYA

GMSSKAEKDIKWGIAPIGWRNDDIPSIGKDNNLQQLLSDIVVAGFQGTEVGGFFPGPEKLNYELKLRNLEIAGQWFSSYIIRDGIEKASEAFEKHCQYLKAINAPVAVVSEQTYTIQRSDTANIFKDKPYFTDKEWDEVCKGLNHYGEIAAKYGLKVAYHHHMGTGIQTKEETDRLMANTDPKLVGLLYDTGHIAVSDGDYMALLNAHIDRVVHVHFKDVRRSKEEECRAKGLTFQGSFLNGMFTVPGDGDLDFKPVYDKLIANNYKGWIVVEAEQDPSKANPLEMAQIAHRYIKQHLIEN

>2GDMA

GALTESQAALVKSSWEEFNANIPKHTHRFFILVLEIAPAAKDLFSFLKGTSEVPQNNPELQAHAGKVFKLVYEAAIQLEVTGVVVTDATLKNLGSVHVSKGVADAHFPVVKEAILKTIKEVVGAKWSEELNSAWTIAYDELAIVIKKEMDDAA

>2X9ZA

AKPKIDKDFKGKANPDTPRVDKDTPVNHQVGDVVEYEIVTKIPALANYATANWSDRMTEGLAFNKGTVKVTVDDVALEAGDYALTEVATGFDLKLTDAGLAKVNDQNAEKTVKITYSATLNDKAIVEVPESNDVTFNYGNNPDHGNTPKPNKPNENGDLTLTKTWVDATGAPIPAGAEATFDLVNAQAGKVVQTVTLTTDKNTVTVNGLDKNTEYKFVERSIKGYSADYQEITTAGEIAVKNWKDENPKPLDPTEPKVVTYG

>2OIZD

AGGGGSSSGADHISLNPDLANEDEVNSCDYWRHCAVDGFLCSCCGGTTTTCPPGSTPSPISWIGTCHNPHDGKDYLISYHDCCGKTACGRCQCNTQTRERPGYEFFLHNDVNWCMANENSTFHCTTSVLVGLAKN

>2AEBA

MSAKSRTIGIIGAPFSKGQPRGGVEEGPTVLRKAGLLEKLKEQECDVKDYGDLPFADIPNDSPFQIVKNPRSVGKASEQLAGKVAEVKKNGRISLVLGGDHSLAIGSISGHARVHPDLGVIWVDAHTDINTPLTTTSGNLHGQPVSFLLKELKGKIPDVPGFSWVTPCISAKDIVYIGLRDVDPGEHYILKTLGIKYFSMTEVDRLGIGKVMEETLSYLLGRKKRPIHLSFDVDGLDPSFTPATGTPVVGGLTYREGLYITEEIYKTGLLSGLDIMEVNPSLGKTPEEVTRTVNTAVAITLACFGLAREGNHKPIDYLNPPK

>3QQ2A

MAESNALDKRLGELRLRADAGGPWARTFSERQQISNRHARAYDQTVSGLEIGLDRGWSASGGRWYAGGLLGYTYADRTYPGDGGGKVKGLHVGGYAAYVGDGGYYLDTVLRLGRYDQQYNIAGTDGGRVTADYRTSGAAWSLEGGRRFELPNDWFAEPQAEVMLWRTSGKRYRASNGLRVKVDANTATLGRLGLRFGRRIALAGGNIVQPYARLGWTQEFKSTGDVRTNGIGHAGAGRHGRVELGAGVDAALGKGHNLYASYEYAAGDRINIPWSFHAGYRYSF

>1JIWI

SSLILLSASDLAGQWTLQQDEAPAICHLELRDSEVAEASGYDLGGDTACLTRWLPSEPRAWRPTPAGIALLERGGLTLMLLGRQGEGDYRVQKGDGGQLVLRRATP

>2I0KA

STGPVAPLPTPPNFPNDIALFQQAYQNWSKEIMLDATWVCSPKTPQDVVRLANWAHEHDYKIRPRGAMAGWTPLTVEKGANVEKVILADTMTHLNGITVNTGGPVATVTAGAGASIEAIVTELQKHDLGWANLPAPGVLSIGGALAVNAHGAALPAVGQTTLPGHTYGSLSNLVTELTAVVWNGTTYALETYQRNDPRITPLLTNLGRCFLTSVTMQAGPNFRQRCQSYTDIPWRELFAPKGADGRTFEKFVAESGGAEAIWYPFTEKPWMKVWTVSPTKPDSSNEVGSLGSAGSLVGKPPQAREVSGPYNYIFSDNLPEPITDMIGAINAGNPGIAPLFGPAMYEITKLGLAATNANDIWGWSKDVQFYIKATTLRLTEGGGAVVTSRANIATVINDFTEWFHERIEFYRAKGEFPLNGPVEIRCCGLDQAADVKVPSVGPPTISATRPRPDHPDWDVAIWLNVLGVPGTPGMFEFYREMEQWMRSHYNNDDATFRPEWSKGWAFGPDPYTDNDIVTNKMRATYIEGVPTTENWDTARARYNQIDPHRVFTNGFMDKLLP

>1MTYB

ERRRGLTDPEMAAVILKALPEAPLDGNNKMGYFVTPRWKRLTEYEALTVYAQPNADWIAGGLDWGDWTQKFHGGRPSWGNETTELRTVDWFKHRDPLRRWHAPYVKDKAEEWRYTDRFLQGYSADGQIRAMNPTWRDEFINRYWGAFLFNEYGLFNAHSQGAREALSDVTRVSLAFWGFDKIDIAQMIQLERGFLAKIVPGFDESTAVPKAEWTNGEVYKSARLAVEGLWQEVFDWNESAFSVHAVYDALFGQFVRREFFQRLAPRFGDNLTPFFINQAQTYFQIAKQGVQDLYYNCLGDDPEFSDYNRTVMRNWTGKWLEPTIAALRDFMGLFAKLPAGTTDKEEITASLYRVVDDWIEDYASRIDFKADRDQIVKAVLAGLK

>1XR4A

SNAMKETVTMLNQQYVVPEGLQPYQGVTANSPWLASETEKRRRKICDSLEEAIRRSGLKNGMTISFHHAFRGGDKVVNMVMAKLAEMGFRDLTLASSSLIDAHWPLIEHIKNGVVRQIYTSGLRGKLGEEISAGLMENPVQIHSHGGRVKLIQSGELNIDVAFLGVPCCDEFGNANGFSGKSRCGSLGYAQVDAQYAKCVVLLTEEWVEFPNYPASIAQDQVDLIVQVDEVGDPEKITAGAIRLSSNPRELLIARQAANVIEHSGYFCDGFSLQTGTGGASLAVTRFLEDKMRRHNITASFGLGGITGTMVDLHEKGLIKALLDTQSFDGDAARSLAQNPHHIEISTNQYANPASKGAACERLNVVMLSALEIDVNFNVNVMTGSNGVLRGASGGHSDTAAGADLTIITAPLVRGRIPCVVEKVLTTVTPGASVDVLVTDHGIAVNPARQDLLDNLRAAGVALMTIEQLQQRAEQLTGKPQPIEFTDRVVAVVRYRDGSVIDVIRQVKG

>3EO7A

GMPEIHQSIAQHYHERTKYDPETIASKSQRLDWAKQPVPFKEYKIGSAIDLKPYLQETPEVFVNDTNGQWWQRLSRLLFRSYGLTARMPSMGNTVYLRAAPSAGGLYPAEVYVVSRGTPLLSPGLYNYQCRTHSLIHYWESDVWQSLQEACFWHPALESTQLAIIVTAVFYRSAWRYEDRAYRRICLDTGHLLGNIELSAAITDYRPHLIGGFIDEAVNDLLYIDPLQEGAIAVLPLADLLDIQQNISPGCTALPSATETNYPQVPDGELLKYFHHHTQISASITGKLNLPTVIQEKSLEDKYNFPFCLKISTVSAPIYWGENLSDLEITMHKRRSTRAYNGEELTFDELKALLDFTYQPQNYIDQSLDNSPDYFDLNLIETFIAVCGVQGLEAGCYYYAPKAQELRQIRFKNFRRELHFLCLGQELGRDAAAVIFHTSDLKSAIAQYGDRVYRYLHMDAGHLGQRLNLAAIQLNLGVSGIGGFFDDQVNEVLGIPNDEAVIYITTLGRPR

>2OU6A

GMPTFNPELHAQTLNSERAYFVQPDADPAFTPHIGALVEMLTYARLTTLQAVEGLPEDQLWATAPGFANSIGTLLAHIAAVERVYHVLSFQGRDVTPEDDGAAYWGLTMGKEGTAPARLPTLDELRAELADARAETLRVFAAKDDAWLAEPLGPGWANQHWAWFHVMEDEVNHRGQLRLLRQVLAPEEGG

>3IB5A

GESSKSGYQTTGENNSSDYQGIIEDGEYKTSKSRGVGISQNSDNLLNLKSFEAGLTTISKDHFSTKSYIFQEGQYLNKATIQDWLGRKSSSNPEGLNPSDNGKKEANKRNPIYVQQIEEQDYMKQNNGKLELAGMTIGIGMNQKDYYQKEQYGATYSTTISKEKRIEEGKIAAKKVLARVRQKVGNNVPIVIAMFAQAPNDSLVGGYFYSYTVSKSGTDIGSWTETNIKSYVLPATEDNKLPNDNDSTSFDNFQKEVKNFFPNISNVTGQGQYKDKTLQGLHITITTQFYSETEITSFTQYVAQAAKSYLPSGIPVDIKINGSDGETQSFVSTTGGNGGYYTHVFGSY

>4JQSA

GQVPEGYPANYAKAPRFKALIYYTQHAEEAHVQFAEQATTFFKKLNYGDGFVLDITTDFSKYPYEKLKEYNVIIMLNTSPNTKAERDAFEQYMENGGGWVGFHAAAYNDKNTHWPWFVKFLGGGVFYCNNWPPQPVLVEVDNEEHPVTKNLPASFVAPASEWYQWTPSPRQNKDVEVLLSLSPKNYPLGIKDVVNFGDFPIVWSNKNYRMIYLNMGHGDEEFIDGTQNLLLVNAFRWVVSKDKSGNPFLK

>3F0PA

MKLAPYILELLTSVNRTNGTADLLVPLLRELAKGRPVSRTTLAGILDWPAERVAAVLEQATSTEYDKDGNIIGYGLTLRETSYVFEIDDRRLYAWCALDTLIFPALIGRTARVSSHCAATGAPVSLTVSPSEIQAVEPAGMAVSLVLPQEAADVRQSFCCHVHFFASVPTAEDWASKHQGLEGLAIVSVHEAFGLGQEFNRHLLQTMSSRTP

>4AQNA

MSDTMVVNGSGGVPAFLFSGSTLSSYRPNFEANSITIALPHYVDLPGRSNFKLMYIMGFPIDTEMEKDSEYSNKIRQESKISKTEGTVSYEQKITVETGQEKDGVKVYRVMVLEGTIAESIEHLDKKENEDILNNNRNRIVLADNTVINFDNISQLKEFLRRSVNIVDHDIFSSNGFEGFNPTSHFPSNPSSDYFNSTGVTFGSGVDLGQRSKQDLLNDGVPQYIADRLDGYYMLRGKEAYDKVRTAPLTLSDNEAHLLSNIYIDKFSHKIEGLFNDANIGLRFSDLPLRTRTALVSIGYQKGFKLSRTAPTVWNKVIAKDWNGLVNAFNNIVDGMSDRRKREGALVQKDIDSGLLK

>4BQQA

MSYYHHHHHHDYDIPTTENLYFQGAMDTYAGAYDRQSRERENSSAASPATQRSANEDKAADLQREVERDGGRFRFVGHFSEAPGTSAFGTAERPEFERILNECRAGRLNMIIVYDVSRFSRLKVMDAIPIVSELLALGVTIVSTQEGVFRQGNVMDLIHLIMRLDASHKESSLKSAKILDTKNLQRELGGYVGGKAPYGFELVSETKEITRNGRMVNVVINKLAHSTTPLTGPFEFEPDVIRWWWREIKTHKHLPFKPGSQAAIHPGSITGLCKRMDADAVPTRGETIGKKTASSAWDPATVMRILRDPRIAGFAAEVIYKKKPDGTPTTKIEGYRIQRDPITLRPVELDCGPIIEPAEWYELQAWLDGRGRGKGLSRGQAILSAMDKLYCECGAV

>2GWNA

SNAMKILLRNALITNEGKTFPGSVMIDGAFISRIIEGELPADDNLSADEVIECSGLRLFPGCIDDQVHFREPGLTHKATIASESRAAVAGGVTSFMDMPNTNPPTTMWERLLEKRQIGADTAWANYGFFFGGTNDNIDEIKRVDKHLVPGLKLFLGSSTGNMLVDNKETLEKIFGECDLLIATHCEKEEIIRANKEHYKAKYGNDLDIHFHPLIRSEEACYRSSAEAVELAERMNARLHILHLSTEKELSLFRNDIPTAQKRITSEVCVHHLWFSDTDYGRLGNRIKWNPAIKKESDREALRAAVRNGRIDIIATDHAPHLLREKEGSCLQAASGGPLVQHSLLALLELCNQGIFSIEEIVSKTAHIPATLFAIEKRGYIRPGYYADLVLVDPSSPHTVSADNILSLCGWSPFEGFTFSHSVAYTFVNGCLAYAKGRLAESRPTVHPLFFNR

>3CTPA

SLANIREIAKRAGISIATVSRHLNNTGYVSEDAREKIQKVVDELNYTPNALARAMFTKNSKTIGLMVPNISNPFFNQMASVIEEYAKNKGYTLFLCNTDDDKEKEKTYLEVLQSHRVAGIIASRSQCEDEYANIDIPVVAFENHILDNIITISSDNYNGGRMAFDHLYEKGCRKILHIKGPEVFEATELRYKGFLDGARAKDLEIDFIEFQHDFQVKMLEEDINSMKDIVNYDGIFVFNDIAAATVMRALKKRGVSIPQEVQIIGFDNSFIGELLYPSLTTINQPIEALAYTIIELLIKIINGEGVLIEDYIMEVKLIERETTISLKDEG

>1QQP2

DKKTEETTLLEDRILTTRNGHTTSTTQSSVGVTYGYATAEDFVSGPNTSGLETRVVQAERFFKTHLFDWVTSDSFGRCHLLELPTDHKGVYGSLTDSYAYMRNGWDVEVTAVGNQFNGGCLLVAMVPELCSIQKRELYQLTLFPHQFINPRTNMTAHITVPFVGVNRYDQYKVHKPWTLVVMVVAPLTVNTEGAPQIKVYANIAPTNVHVAGEFPSKE

>3L1WA

MKIATYNVRVDTEYDQDWQWSFRKEAVCQLINFHDWSLCCIQEVRPNQVRDLKAYTTFTCLSAEREGDGQGEGLAILYNEQKVQAIDTGYFWLSETPQQPSIHPEAGCPRIALWGLFKETTQNTPFLVINVHLDHISAHARLAGMTVILEELHDKIAQYPTLLMGDFNAESGEEVHQLVQKKFQDSKNLATHYGPRGTFQNFTYTKPWAELEEIDYIYVKGWQVQQTASLTDSIDGRFPSDHFPLEAEVAGENLYFQ

>2AQWA

MHFKTKLKNRRSEVNTCLCIGLDPDEDDIKNFMKNEEQNGYKNIKNNMNSNNNGIENIIKIGKEILLTDGENIQNLSEEDKFFYFFNHFCFYIINNTKEYALVYKMNFAFYIPYGSVGINALKNVFDYLNSMNIPTMLDMKINDIGNTVKNYRKFIFEYLKSDSCTINVYMGTNMLKDICFDYEKNKYYSAYVLIKTTNKDSFIFQNELSINDKQAYIVMADETQKMATELKIEQNNEFIGFVVGSNAFEEMKIIRNKFPDSYILSPGIGAQNGDLYKTLKNGYNKDYEKLLINVGRAITKSPDPKKSSESYYNQIIQIFKDIENGDNIEQV

>2H0QA

RCLFVCRHGERMDVVFGKYWLSQCFDAKGRYIRTNLNMPHSLPQRSGGFRDYEKDAPITVFGCMQARLVGEALLESNTVIDHVYCSPSLRCVQTAHNILKGLQQDNHLKIRVEPGLFEWTKWVAGSTLPAWIPPSELAAANLSVDTTYRPHIPVSKLAISESYDTYINRSFQVTKEIISECKSKGNNILIVAHASSLEACTCQLQGLSPQNSKDFVQMVRKIPYLGFCSCEELGETGIWQLTDPPILPLTHGPTGGFNWRE

>3ISMC

MAKRKAEDTQSDKMATAEKVAQNDYTIGLVDPVKDYQKLIETRVQVDEIVDDDVTKENFDRTAAAARDVIWRLLFDEAGTSQSNTEKASQLLEEYRGDACFYDPTPYNEWIVKLRDEVLKKELLDFWRDVLVKKQLGPCWSRDSDLFDSDDTPPLEFYAHAGCTAPFAASLKVRAALEEQASLDQDGPATPTTPGELSADDAAALSGEFEATLTKENPLEEYRTLMKRFVLTKIIVPDSVHQASVKKIAAAAREIIWKLLFDGTPSAEDQNKAAELLQEYKGDAGFYGPDDYNSWIFNLRDEVLTKELLDFWRDKMVKMELGPSCARDSDYYDNEDPLPFEFYEKAGCKAPFEGPVNDD

>3QE7A

MTRRAIGVSERPPLLQTIPLSLQHLFAMFGATVLVPVLFHINPATVLLFNGIGTLLYLFICKGKIPAYLGSSFAFISPVLLLLPLGYEVALGGFIMCGVLFCLVSFIVKKAGTGWLDVLFPPAAMGAIVAVIGLELAGVAAGMAGLLPAEGQTPDSKTIIISITTLAVTVLGSVLFRGFLAIIPILIGVLVGYALSFAMGIVDTTPIINAHWFALPTLYTPRFEWFAILTILPAALVVIAEHVGHLVVTANIVKKDLLRDPGLHRSMFANGLSTVISGFFGSTPNTTYGENIGVMAITRVYSTWVIGGAAIFAILLSCVGKLAAAIQMIPLPVMGGVSLLLYGVIGASGIRVLIESKVDYNKAQNLILTSVILIIGVSGAKVNIGAAELKGMALATIVGIGLSLIFKLISVLRPEEVVLDAEDADITDK

>3VJZA

HMTALTLPEDIRQQEPSALLYTLVSAYLEHTAQTGDESLSCLSDDQHTLTAFCYLDSQVEEGGFVQLIASGYGEYIFRNPLADSLRRWKIKAVPKVLDKAKALYEQHGKTIETLADGGADIPSLRKQFPEFEEWDGAYYEAAEQDLPLLAEHIQSNWETFAHIGQA

>1Z6OM

TQCNVNPVQIPKDWITMHRSCRNSMRQQIQMEVGASLQYLAMGAHFSKDVVNRPGFAQLFFDAASEEREHAMKLIEYLLMRGELTNDVSSLLQVRPPTRSSWKGGVEALEHALSMESDVTKSIRNVIKACEDDSEFNDYHLVDYLTGDFLEEQYKGQRDLAGKASTLKKLMDRHEALGEFIFDKKLLGIDV

>4E9JA

SHMENSGGNAFVPAGNQQEAHWTINLKDADIREFIDQISEITGETFVVDPRVKGQVSVVSKAQLSLSEVYQLFLSVMSTHGFTVVAQGDQARIVPNAEAKTEAGGGQSAPDRLETRVIQVQQSPVSELIPLIRPLVPQYGHLAAVPSANALIISDRSANIARIEDVIRQLDQKGSHDYSVINLRYGWVMDAAEVLNNAMSRGQAKGAAGAQVIADARTNRLIILGPPQARAKLVQLAQSLDTPTAR

>3PZDA

EFDTPTQQLIQDIKENCLNSDVVEQIYKRNPILRYTHHPLHSPLLPLPYGDINLNLLKDKGYTTLQDEAIKIFNSLQQLESMSDPIPIIQGILQTGHDLRPLRDELYCQLIKQTNKVPHPGSVGNLYSWQILTCLSCTFLPSRGILKYLKFHLKRIREQFPGSEMEKYALFTYESLKKTKCREFVPSRDEIEALIHRQEMTSTVYCHGGGSCKITINSHTTAGEVVEKLIRGLAMEDSRNMFALFEYNGHVDKAIESRTVVADVLAKFEKLAATSEVGDLPWKFYFKLYCFLDTDNVPKDSVEFAFMFEQAHEAVIHGHHPAPEENLQVLAALRLQYLQGDYTLHAAIPPLEEVYSLQRLKARISQSTKTQMLDMWIKEEVSSARASIIDKWRKFQGMNQEQAMAKYMALIKEWPGYGSTLFDVECKEGGFPQELWLGVSADAVSVYKRGEGRPLEVFQYEHILSFGAPLANTYKIVVDERELLFETSEVVDVAKLMKAYISMIVKKRYST

>1QYNA

MSEQNNTEMTFQIQRIYTKDISFEAPNAPHVFQKDWQPEVKLDLDTASSQLADDVYEVVLRVTVTASLGEETAFLCEVQQGGIFSIAGIEGTQMAHCLGAYCPNILFPYARECITSMVSRGTFPQLNLAPVNFDALFMNYLQQQAGEGTEEHQ

>3CA8A

MNITPFPTLSPATIDAINVIGQWLAQDDFSGEVPYQADCVILAGNAVMPTIDAACKIARDQQIPLLISGGIGHSTTFLYSAIAQHPHYNTIRTTGRAEATILADIAHQFWHIPHEKIWIEDQSTNCGENARFSIALLNQAVERVHTAIVVQDPTMQRRTMATFRRMTGDNPDAPRWLSYPGFVPQLGNNADSVIFINQLQGLWPVERYLSLLTGELPRLRDDSDGYGPRGRDFIVHVDFPAEVIHAWQTLKHDAVLIEAMESRSLR

>1EL6A

MSLLNNKAGVISRLADFLGFRPKTGDIDVMNRQSVGSVTISQLAKGFYEPNIESAINDVHNFSIKDVGTIITNKTGVSPEGVSQTDYWAFSGTVTDDSLPPGSPITVLVFGLPVSATTGMTAIEFVAKVRVALQEAIASFTAINSYKDHPTDGSKLEVTYLDNQKHVLSTYSTYGITISQEIISESKPGYGTWNLLGAQTVTLDNQQTPTVFYHFERTA

>1SDDA

AKLRQFYVAAQSIRWNYRPESTHLSSKPFETSFKKIVYREYEAYFQKEKPQSRTSGLLGPTLYAEVGDIMKVHFKNKAHKPLSIHAQGIKYSKFSEGASYSDHTLPMEKMDDAVAPGQEYTYEWIISEHSGPTHDDPPCLTHIYYSYVNLVEDFNSGLIGPLLICKKGTLTEDGTQKMFEKQHVLMFAVFDESKSWNQTSSLMYTVNGYVNGTMPDITVCAHDHISWHLIGMSSGPELFSIHFNGQVLEQNHHKISAITLVSATSTTANMTVSPEGRWTIASLIPRHFQAGMQAYIDIKNCAKKTR

>2W9YA

GAMSVASLPECVKNFFPTEQLEFSSSITADEKPVLHEVFQKHSCFSQCGEMIDEVSKKHPELGKRLATVLEGNKKRLDGLSPAAVEYAKKLIHMVTTTLCSLTVGKPIDDADAKRLHQEFQSLSSEDQAALRKNNPDIKF

>2J7QA

MKIVRASRDQSAPVYGPRAGSQCMSNCFTFLHTCYLMGIDPVLDTTSLDAVLDSGARLDAIADEKVKRQALTDHPYRLGTEIPTVIETPAGITGHALSRPFNGTAETQDLGGYKCLGILDFLTYARGKPLPVYIIVTVGVHTRGVIVARGATYVFDPHTTDLSAEAAVYVCDDFTEAISALSFFTEMIGDFYYDAVLVYFTRCRTTLISPSELLVQIMDQYKDPDIDASVMS

>2Z2NA

MEFKLQELNLTNQDTGPYGITVSDKGKVWITQHKANMISCINLDGKITEYPLPTPDAKVMCLTISSDGEVWFTENAANKIGRITKKGIIKEYTLPNPDSAPYGITEGPNGDIWFTEMNGNRIGRITDDGKIREYELPNKGSYPSFITLGSDNALWFTENQNNAIGRITESGDITEFKIPTPASGPVGITKGNDDALWFVEIIGNKIGRITTSGEITEFKIPTPNARPHAITAGAGIDLWFTEWGANKIGRLTSNNIIEEYPIQIKSAEPHGICFDGETIWFAMECDKIGKLTLIKDNME

>2Q83A

GEEGNSSELPLSAEDAKKLTELAENVLQGWDVQAEKIDVIQGNQMALVWKVHTDSGAVCLKRIHRPEKKALFSIFAQDYLAKKGMNVPGILPNKKGSLYSKHGSFLFVVYDWIEGRPFELTVKQDLEFIMKGLADFHTASVGYQPPNGVPIFTKLGRWPNHYTKRCKQMETWKLMAEAEKEDPFSQLYLQEIDGFIEDGLRIKDRLLQSTYVPWTEQLKKSPNLCHQDYGTGNTLLGENEQIWVIDLDTVSFDLPIRDLRKMIIPLLDTTGVWDDETFNVMLNAYESRAPLTEEQKQVMFIDMLFPYELYDVIREKYVRKSALPKEELESAFEYERIKANALRQLI

>2YF4A

GIDPFTMSDLPCPPTNAERLHEFHRAIGAATPERPTPPPPELLRLRQTLLDEESAEVRAEIDHLLARQAAGEALSAGDLAPLAHELADLLYVTYGALDQLGIDADAVFAEVHRANLSKASGPRRADGKQLKPEGWRPADVRGVIERLQHAPADD

>1PMHX

ESSVNPVVLDFEDGTVMSFGEAWGDSLKCIKKVSVSQDLQRPGNKYALRLDVEFNPNNGWDQGDLGTWIGGVVEGQFDFTGYKSVEFEMFIPYDEFSKSQGGFAYKVVINDGWKELGSEFNITANAGKKVKINGKDYTVIHKAFAIPEDFRTKKRAQLVFQFAGQNSNYKGPIYLDNVRIRPEDA

>1Q0PA

GEQQKRKIVLDPSGSMNIYLVLDGSDSIGASNFTGAKKSLVNLIEKVASYGVKPRYGLVTYATYPKIWVKVSEADSSNADWVTKQLNEINYEDHKLKSGTNTKKALQAVYSMMSWPDDVPPEGWNRTRHVIILMTDGLHNMGGDPITVIDEIRDLLYIGKDRKNPREDYLDVYVFGVGPLVNQVNINALASKKDNEQHVFKVKDMENLEDVFYQMIDESQSLS

>2R2ZA

SNADEVENLYTQVADNEYLVQGRMLIDEFNEVFETDLHMSDVDTMAGYLITALGTIPDEGEKPSFEVGNIKLTAEEMEGTRLLVLRVHFYDEE

>3H9MA

MSLSFTPLHTTSEAFIEKALPWLEDRYFHIAYLNPNGYTAYPQGAFRHYLAFGSEAAIHVSDATRVFETWNEIKKGYTNEWIFVFASYDGKNSVEQLHTSKEAGIAFAAATFFIPEHVWEIQPDGILIHKGSGSSLVTEIQHAEPSTPVQQSDIFVKQVVSKESYFNAFDELQQIIAQGDAYEINYCIPFTAKGNISPAATYQRLNKKTPMPFSVYYKFNTEYILSASPERFIKKTGDTIISQPIKGTSKRGKSKAEDEMLKQQLGTSEKEQSENTMIVDLVRNDLSRTAVAGSVCVPELSGLYTFPNVHQLISTVQSTIDPACSSIDVIQQAFPMGSMTGAPKVNVMKFIDRIESMARGPFSGTVGYMDPHDNFDFNVLIRSIFYNSATQELFMEAGSAITSYAKAETEYEECLLKITPMIHILNNQEGHHHHHH

>3BQ9A

MSLASISPQGSMSLLSQLEIERLKASSNSQLYKLFRNCCLAVLNAGSHTDSSADIYDSYKDFEVNIIRRERGIKLELIEPPEEAFVDGEVIVGIRELLESVLRDILFTGERYSETDLEHADSATLTHVVFDILRNARTLRPQEEPNMVVCWGGHSINEIEYKYTKDVGYHIGLRGLNICTGCGPGAMKGPMKGATIGHAKQRVEGGRYLGLTEPGIIAAEPPNPIVNELVILPDIEKRLEAFVRCAHGIVIFPGGAGTAEELLYLLGILMHPDNQRQSLPVILTGPASSRDYFEALDEFIGATIGDEARQLYKIIIDDPAAVAQHMHAGMAAVKQYRRDSGDAYYFNWTLKINEEFQRPFSPTHENVAALNLHPDQPKERLAADLRRAFSAIVAGNVKDEGIRQIRKNGVFTIHGEQSLMKRLDELLRAFVEQGRMKLPGSVYNPCYKVITDEGHHHHHH

>1K04A

LSSPADSYNEGVKLQPQEISPPPTANLDRSNDKVYENVTGLVKAVIEMSSKIQPAPPEEYVPMVKEVGLALRTLLATVDETIPLLPASTHREIEMAQKLLNSDLGELINKMKLAQQYVMTSLQQEYKKQMLTAAHALAVDAKNLLDVIDQARLKMLGQTRPH

>2IQIA

SLSLGDQKPATIYAPTVRVTPNPAWPQVSWQLLVAKPSAARIIDSPRINVRPTPGELQVYHGAGWAQPATDMLEDSVVRAFEDSGKIAAVARIGAGIRSDYKLAIDVRRFESDYAGQSLPAATIELNAKLLHSSDQRVVASRTFTVARPSSSTDTAAVAAAFEQALTQVTTELVGWTLITGQQDSQTLPRAL

>2O8SA

MAPCLENVACVNLSAMPPAPPSPQPVSHKQAATLCRQGRTCALKSGSNESGGSVTSTYTSYRLISQDIGKSLERVSKQPDVARETEYYREKIGSVKSIDDFMADTRLYNYALKAHGLEDMAYAKAFIRKVLTEGASDKNAFANKLSDNRYAELAKSLDFAGLGAAATATEAAKSGVIGNYARQTLEQEAGDDNNGVRLALYFERKAPTIKSGLDFLADDALAQVFRTTFNLPDAFAAADVDKQAALIEKSINIKDLQDPEKVGKLLERFTIMWEMQNPSTTYDPLAVFGSSSGYGISPDLLISINSLKLGGKAAALEHHHHHH

>2A4XA

MSARISLFAVVVEDMAKSLEFYRKLGVEIPAEADSAPHTEAVLDGGIRLAWDTVETVRSYDPEWQAPTGGHRFAIAFEFPDTASVDKKYAELVDAGYEGHLKPWNAVWGQRYAIVKDPDGNVVDLFAPLPLEHHHHHH

>4DT5A

GYSCRAVGVDGRAVTDIQGTCHAKATGAGAMASGTSEPGSTSTATATGRGATARSTSTGRGTATTTATGTASATSNAIGQGTATTTATGSAGGRATGSATTSSSASQPTQTQTITGPGFQTAKSFARNTATTTVTASHHHHHH

>2X3MA

GMSAFDEFNEGFGLDVSDTPEELAFETESAIEEIESETSPGDQPKGSEPEEIRVWAEEKARKAVEEGREVTNWADWIMGWRTPNASEKKMEFMYWYTRTYLEEAKDIRPDIADALARGMAGLAFGRTDWVASMLDPQIMRHIYTDPEVARIYSETRDMLRRVSDYYISLTTMELGKVADIIAEAKAKGENPEVVAREIAEAVPRLSPKSLYFNLYYIGRSIGDNYVLEVARVLSKMRRR

>1VF7A

AESSGKSEAPPPAQTPEVGIVTLEAQTVTLNTELPGRTNAFRIAEVRPQVNGIILKRLFKEGSDVKAGQQLYQIDPATYEADYQSAQANLASTQEQAQRYKLLVADQAVSKQQYADANAAYLQSKAAVEQARINLRYTKVLSPISGRIGRSAVTEGALVTNGQANAMATVQQLDPIYVDVTQPSTALLRLRRELASGQLERAGDNAAKVSLKLEDGSQYPLEGRLEFSEVSVDEGTGSVTIRAVFPNPNNELLPGMFVHAQLQEGVKQKAILAPQQGVTRDLKGQATALVVNAQNKVELRVIKADRVIGDKWLVTEGLNAGDKIITEGLQFVQPGVEVKTVPAKNVASAQKADAAPAKTDSKGHHHHHH

>3NREA

GMTIYTLSHGSLKLDVSDQGGVIEGFWRDTTPLLRPGKKSGVATDASCFPLVPFANRVSGNRFVWQGREYQLQPNVEWDAHYLHGDGWLGEWQCVSHSDDSLCLVYEHRSGVYHYRVSQAFHLTADTLTVTLSVTNQGAETLPFGTGWHPYFPLSPQTRIQAQASGYWLEREQWLAGEFCEQLPQELDFNQPAPLPRQWVNNGFAGWNGQARIEQPQEGYAIIMETTPPAPCYFIFVSDPAFDKGYAFDFFCLEPMSHAPDDHHRPEGGDLIALAPGESTTSEMSLRVEWL

>1QD6C

FTLYPYDTNYLIYTQTSDLNKEAIASYDWAENARKDEVKFQLSLAFPLWRGILGPNSVLGASYTQKSWWQLSNSEESSPFRETNYEPQLFLGFATDYRFAGWTLRDVEMGYNHDSNGRSDPTSRSWNRLYTRLMAENGNWLVEVKPWYVVGNTDDNPDITKYMGYYQLKIGYHLGDAVLSAKGQYNWNTGYGGAELGLSYPITKHVRLYTQVYSGYGESLIDYNFNQTRVGVGVMLNDLF

>1V0AA

MASAVGEKMLDDFEGVLNWGSYSGEGAKVSTKIVSGKTGNGMEVSYTGTTDGYWGTVYSLPDGDWSKWLKISFDIKSVDGSANEIRFMIAEKSINGVGDGEHWVYSITPDSSWKTIEIPFSSFRRRLDYQPPGQDMSGTLDLDNIDSIHFMYANNKSGKFVVDNIKLIGALEHHHHHH

>3DMBA

GMADPKELQDKFWKALKSDRTVMLGLDGVEDGHARPMTAQIEGDSGGPIWFFTSKDNALIAMLGQGRRVIGAFSSKGHDLFASISGSLREDTDPAVVDRLWNPYVAAWYEGGKDDPKLALLRLDADHAQIWLNGSSLLAGIKVLLGV

>3GN6A

GMTGLSQSQASPMQIQPGNAAFNPWTDAALDTIRDVNQALTLYAEMRVVPAHHDAFLAAIDTVSAKLRVLPGFLSLALKQMSGDSTMVKNYPETYKGVLATAYLDGVAAGTQPYFYNLFVRFADGRAARAAGFEALFETHIHPLLHAMAPRGGDGPELLAYRAVLQSVVAGDRHAIYRGAEEIRSFLRRPVELPERETVTVENHVMVPEDKHAAWEPQVAILLQVAQDTFEPQDEPSGVGLPGARDNRYYRKALSTEILRNAHADGGLRAYIMHGVWESVWDHENSHLDPRFLAAAGPVGAAAVVGPVEPFYLTRRLVVAD

>4GQ4A

GGSSSMGLKAAQKTLFPLRSIDDVVRLFAAELGREEPDLVLLSLVLGFVEHFLAVNRVGLTYFPVADLSIIAALYARFTAQIRGAVDLSLYPREGGVSSRELVKKVSDVIWNSLSRSYFKDRAHIQSLFSFITGTKLDSSGVAFAVVGACQALGLRDVHLALSEDHAWVVFGPNGEQTAEVTWHGKGNEDRRGQTVNAGVAERSWLYLKGSYMRCDRKMEVAFMVCAINPSIDLHTDSLELLQLQQKLLWLLYDLGHLERYPMALGNLADLEELEPTPGRPDPLTLYHKGIASAKTYYRDEHIYPYMYLAGYHCRNRNVREALQAWADTATVIQDYNYCREDEEIYKEFFEVANDVIPNLLKEAASLLEAGSQGSALQDPECFAHLLRFYDGICKWEEGSPTPVLHVGWATFLVQSLGRFEGQVRQKVRIVSVPAPAASPPPEGPVLTFQSEKMKGMKELLVATKINSSAIKLQLTAQSQVQMKKQKVS

>3AI7A

MTSPVIGTPWKKLNAPVSEEALEGVDKYWRVANYLSIGQIYLRSNPLMKEPFTREDVKHRLVGHWGTTPGLNFLIGHINRFIADHGQNTVIIMGPGHGGPAGTSQSYLDGTYTETFPKITKDEAGLQKFFRQFSYPGGIPSHFAPETPGSIHEGGELGYALSHAYGAIMDNPSLFVPAIVGDGEAETGPLATGWQSNKLVNPRTDGIVLPILHLNGYKIANPTILSRISDEELHEFFHGMGYEPYEFVAGFDDEDHMSIHRRFAELWETIWDEICDIKATAQTDNVHRPFYPMLIFRTPKGWTCPKYIDGKKTEGSWRSHQVPLASARDTEAHFEVLKNWLESYKPEELFDANGAVKDDVLAFMPKGELRIGANPNANGGVIRNDLKLPNLEDYEVKEVAEYGHGWGQLEATRTLGAYTRDIIKNNPRDFRIFGPDETASNRLQASYEVTNKQWDAGYISDEVDEHMHVSGQVVEQLSEHQMEGFLEAYLLTGRHGIWSSYESFVHVIDSMLNQHAKWLEATVREIPWRKPIASMNLLVSSHVWRQDHNGFSHQDPGVTSVLLNKCFHNDHVIGIYFATDANMLLAIAEKCYKSTNKINAIIAGKQPAATWLTLDEARAELEKGAAAWDWASTAKNNDEAEVVLAAAGDVPTQEIMAASDKLKELGVKFKVVNVADLLSLQSAKENDEALTDEEFADIFTADKPVLFAYHSYAHDVRGLIYDRPNHDNFNVHGYEEEGSTTTPYDMVRVNRIDRYELTAEALRMIDADKYADKIDELEKFRDEAFQFAVDNGYDHPDYTDWVYSGVNTDKKGAVTATAATAGDNEHHHHHH

>2F1NA

MAHHHHHHVGTDLTDFRVATWNLQGASATTESKWNINVRQLISGENAVDILAVQEAGSPPSTAVDTGRVIPSPGIPVRELIWNLSTNSRPQQVYIYFSAVDALGGRVNLALVSNRRADEVFVLSPVRQGGRPLLGIRIGNDAFFTAHAIAMRNNDAPALVEEVYNFFRDSRDPVHQALNWMILGDFNREPADLEMNLTVPVRRASEIISPAAATQTSQRTLDYAVAGNSVAFRPSPLQAGIVYGARRTQISSDHFPVGVSRR

>3TVJI

GSGEVTCEPGTTFKDKCNTCRCGSDGKSAVCTKLWCNQ

>1EP3B

MSQLQEMMTVVSQREVAYNIFEMVLKGTLVDEMDLPGQFLHLAVPNGAMLLRRPISISSWDKRAKTCTILYRIGDETTGTYKLSKLESGAKVDVMGPLGNGFPVAEVTSTDKILIIGGGIGVPPLYELAKQLEKTGCQMTILLGFASENVKILENEFSNLKNVTLKIATDDGSYGTKGHVGMLMNEIDFEVDALYTCGAPAMLKAVAKKYDQLERLYISMESRMACGIGACYACVEHDKEDESHALKVCEDGPVFLGKQLSL

>2HJNA

MSPVLTTPKRHAPPPEQLQNVTDFNYTPSHQKPFLQPQAGTTVTTHQDIKQIVEMTLGSEGVLNQAVKLPRGEDENEWLAVHCVDFYNQINMLYGSITEFCSPQTCPRMIATNEYEYLWAFQKGQPPVSVSAPKYVECLMRWCQDQFDDESLFPSKVTGTFPEGFIQRVIQPILRRLFRVYAHIYCHHFNEILELNLQTVLNTSFRHFCLFAQEFELLRPADFGPLLELVMELRDR

>1Z0WA

DYKLFITEGYEVGRVNGLAVIGESAGIVLPIIAEVTPSMSKSEGRVIATGRLQEIAREAVMNVSAIIKKYTGRDISNMDVHIQFVGTYEGVEGDSASISIATAVISAIEGIPVDQSVAMTGSLSVKGEVLPVGGVTQKIEAAIQAGLKKVIIPKDNIDDVLLDAEHEGKIEVIPVSRINEVLEHVLEDGKKKNRLMSKFKELELAAV

>4F55A

GGGRTNVPNGYSQNDIQLMANAVYGESRGEPYLGQVAVAAVILNRVTSASFPNTVSGVIFEPRAFTAVADGQIYLTPNETAKKAVLDAINGWDPTGNALYYFNPDTATSKWIWTRPQIKKIGKHIFCK

>2NLVA

GMDKLVKYQELVKKLLTNYASDDVSDQDVEVQLILDTERNHYQWMNVGWQGLNRIYRCVIHFDIKDGKIWLQQNLTDRNPAEELVMMGVPREDIVLGLQAPYKRQYTDYGVA

>3CVOA

GMDDQSGDQMRPELTMPPAEAEALRMAYEEAEVILEYGSGGSTVVAAELPGKHVTSVESDRAWARMMKAWLAANPPAEGTEVNIVWTDIGPTGDWGHPVSDAKWRSYPDYPLAVWRTEGFRHPDVVLVDGRFRVGCALATAFSITRPVTLLFDDYSQRRWQHQVEEFLGAPLMIGRLAAFQVEPQPIPPGSLMQLIRTMTSP

>2PNWA

MSLNTPFSIDEVSFRDLPGWGQDDPRKLFPAMATILSHLRNAKPYRTGALGITAAELVSLLELAERGQVNSPEQARQFFETNSVPFRISPAQGKSGFVTAFYEPELEVSATPDDVWRYPIYRRPPELVDIDNDNRPDGFDPSYAFGKADEEGISYFPDRRAIDEGCLRGRGLEIAWARSKVDLFFVHVQGAARLVFPDGAIKRITYAAKAGHVFSPIGRLLLDRGELDPKTISMQTIRQWLADHPDEVDGVLWHNRSYIFFREADVAGLDMGPIAAAKVPLVAGRALAVDRLIHTFGLPFFIHAPTLTHLDDGKPFARLMLALDTGSAIVGPARGDIFTGSGFEAGELAGTVRNEADFYILLPRIAAERYRREGHHHHHH

>2OWLA

MLWFKNLMVYRLSREISLRAEEMEKQLASMAFTPCGSQDMAKMGWVPPMGSHSDALTHVANGQIVICARKEEKILPSPVIKQALEAKIAKLEAEQARKLKKTEKDSLKDEVLHSLLPRAFSRFSQTMMWIDTVNGLIMVDCASAKKAEDTLALLRKSLGSLPVVPLSMENPIELTLTEWVRSGSAAQGFQLLDEAELKSLLEDGGVIRAKKQDLTSEEITNHIEAGKVVTKLALDWQQRIQFVMCDDGSLKRLKFCDELRDQNEDIDREDFAQRFDADFILMTGELAALIQNLIEGLGGEAQR

>2Z72A

MGNTATEFDGPYVITPISGQSTAYWICDNRLKTTSIEKLQVNRPEHCGDLPETKLSSEIKQIMPDTYLGIKKVVALSDVHGQYDVLLTLLKKQKIIDSDGNWAFGEGHMVMTGDIFDRGHQVNEVLWFMYQLDQQARDAGGMVHLLMGNHEQMVLGGDLRYVHQRYDIATTLINRPYNKLYSADTEIGQWLRSKNTIIKINDVLYMHGGISSEWISRELTLDKANALYRANVDASKKSLKADDLLNFLFFGNGPTWYRGYFSETFTEAELDTILQHFNVNHIVVGHTSQERVLGLFHNKVIAVDSSIKVGKSGELLLLENNRLIRGLYDGTRETLQENSLNQ

>1WUIS

LMGPRRPSVVYLHNAECTGCSESVLRAFEPYIDTLILDTLSLDYHETIMAAAGDAAEAALEQAVNSPHGFIAVVEGGIPTAANGIYGKVANHTMLDICSRILPKAQAVIAYGTCATFGGVQAAKPNPTGAKGVNDALKHLGVKAINIAGCPPNPYNLVGTIVYYLKNKAAPELDSLNRPTMFFGQTVHEQCPRLPHFDAGEFAPSFESEEARKGWCLYELGCKGPVTMNNCPKIKFNQTNWPVDAGHPCIGCSEPDFWDAMTPFYQN

>2Q22A

GMSMPNHPNLTTADAKKILNKFNCLDIAPILKPSEKESVRRALILITKLSDYQILGICADTADEGLLAMKTYSHALGYEVPIDLPVVEGPVYIKLNGKNGLCYLDSYAGHHRGVLVSCQSYYEGGINEMYGHLPLDLFV

>4HR9A

PNSEDKNFPRTVMVNLNIHNRNTNTNPKRSSDYYDRSTSPWNLHRNEDPERYPSVIWEAKCRHLGCINADGNVDYHMNSVPIQQEILVLRREPPHSPNSFRLEKILVSVGCTCVTPIVHHVA

>2GA1A

GMNKKTQLLEVIAALPEELVDQALNYVQMLQNPIQITPGVCGGQARIRNTRIPVWTLVAYRQQGAPDKELLANYPGLTAEDLSAAWHYYEQNPEQIDREIAQDDLV

>1WVGA

SIDKNFWQGKRVFVTGHTGFKGSWLSLWLTEMGAIVKGYALDAPTVPSLFEIVRLNDLMESHIGDIRDFEKLRSSIAEFKPEIVFHMAAQPLVRLSYEQPIKTYSTNVMGTVHLLETVKQVGNIKAVVNITSDKCYDNREWVWGYRENEPMGGYDPYSNSKGCAELVASAFRNSFFNPANYEQHGVGLASVRAGNVIGGGDWAKDRLIPDILRSFENNQQVIIRNPYSIRPWQHVLEPLSGYIVVAQRLYTEGAKFSEGWNFGPRDEDAKTVEFIVDKMVTLWGDDASWLLDGENHPHEAHYLKLDCSKANMQLGWHPRWGLTETLSRIVKWHKAWIRGEDMLICSKREISDYMSATTR

>2YFRA

MDVKQVEKKDSVDKTNAEENKDSSVKPAENATKAELKGQVKDIVEESGVDTSKLTNDQINELNKINFSKEAKSGTQLTYNDFKKIAKTLIEQDARYAIPFFNASKIKNMPAAKTLDAQSGKVEDLEIWDSWPVQDAKTGYVSNWNGYQLVIGMMGVPNVNDNHIYLLYNKYGDNDFNHWKNAGPIFGLGTPVIQQWSGSATLNKDGSIQLYYTKVDTSDNNTNHQKLASATVYLNLEKDQDKISIAHVDNDHIVFEGDGYHYQTYDQWKETNKGADNIAMRDAHVIDDDNGNRYLVFEASTGTENYQGDDQIYQWLNYGGTNKDNLGDFFQILSNSDIKDRAKWSNAAIGIIKLNDDVKNPSVAKVYSPLISAPMVSDEIERPDVVKLGNKYYLFAATRLNRGSNDDAWMATNKAVGDNVAMIGYVSDNLTHGYVPLNESGVVLTASVPANWRTATYSYYAVPVEGRDDQLLITSYITNRGEVAGKGMHATWAPSFLLQINPDNTTTVLAKMTNQGDWIWDDSSENPDMMGVLEKDAPNSAALPGEWGKPVDWDLIGGYNLKPHQHHHHHH

>2IVFC

MKAKRVPGGKELLLDLDAPIWAGAESTTFEMFPTPLVMVKEVSPFLALSEGHGVIKRLDVAALHNGSMIALRLKWASEKHDKIVDLNSFVDGVGAMFPVARGAQAVTMGATGRPVNAWYWKANANEPMEIVAEGFSAVRRMKDKAGSDLKAVAQHRNGEWNVILCRSMATGDGLAKLQAGGSSKIAFAVWSGGNAERSGRKSYSGEFVDFEILK

>3AXGA

MNTTPVHALTDIDGGIAVDPAPRLAGPPVFGGPGNDAFDLAPVRSTGREMLRFDFPGVSIGAAHYEEGPTGATVIHIPAGARTAVDARGGAVGLSGGYDFNHAICLAGGASYGLEAGAGVSGALLERLEYRTGFAEAQLVSSAVIYDFSARSTAVYPDKALGRAALEFAVPGEFPQGRAGAGMSASAGKVDWDRTEITGQGAAFRRLGDVRILAVVVPNPVGVIMDRAGTVVRGNYDAQTGVRRHPVFDYQEAFAEQVPPVTEAGNTTISAIVTNVRMSPVELNQFAKQVHSSMHRGIQPFHTDMDGDTLFAVTTDEIDLPTTPGSSRGRLSVNATALGAIASEVMWDAVLEAGK

>1SBXA

GSHMFMPSDRSTERCETVLEGETISCFVVGGEKRLCLPQILNSVLRDFSLQQINAVCDELHIYCSRCTADQLEILKVMGILPFSAPSCGLITKTDAERLCNALLYG

>3QEKA

SDPKIVNIGAVLSTKKHEQIFREAVNQANKRHFTRKIQLQATSVTHRPNAIQMALSVCEDLISSQVYAILVSHPPAPTDHLTPTPISYTAGFYRIPVIGLTTRMSIYSDKSIHLSFLRTVPPYSHQALVWFEMMRLFNWNHVILIVSDDHEGRAAQKKLETLLEGKESKSKKRNYENLDQLSYDNKRGPKADKVLQFEPGTKNLTALLLEAKELEARVIILSASEDDATAVYKSAAMLDMTGAGYVWLVGEREISGSALRYAPDGIIGLQLINGKNESAHISDAVAVVAQAIHELFEMENITDPPRGCVGNTNIWKTGPLFKRVLMSSKYPDGVTGRIEFNEDGDRKFAQYSIMNLQNRKLVQVGIFNGSYIIQNDRKIIWPGG

>3KU3A

PGDQICIGYHANNSTEKVDTILERNVTVTHAKDILEKTHNGKLCKLNGIPPLELGDCSIAGWLLGNPECDRLLSVPEWSYIMEKENPRDGLCYPGSFNDYEELKHLLSSVKHFEKVKILPKDRWTQHTTTGGSRACAVSGNPSFFRNMVWLTEKGSNYPVAKGSYNNTSGEQMLIIWGVHHPNDETEQRTLYQNVGTYVSVGTSTLNKRSTPEIATRPKVNGQGGRMEFSWTLLDMWDTINFESTGNLIAPEYGFKISKRGSSGIMKTEGTLENCETKCQTPLGAINTTLPFHNVHPLTIGECPKYVKSEKLVLATGLRNVPQIESR

>1DS1A

MTSVDCTAYGPELRALAARLPRTPRADLYAFLDAAHTAAASLPGALATALDTFNAEGSEDGHLLLRGLPVEADADLPTTPSSTPAPEDRSLLTMEAMLGLVGRRLGLHTGYRELRSGTVYHDVYPSPGAHHLSSETSETLLEFHTEMAYHRLQPNYVMLACSRADHERTAATLVASVRKALPLLDERTRARLLDRRMPCCVDVAFRGGVDDPGAIAQVKPLYGDADDPFLGYDRELLAPEDPADKEAVAALSKALDEVTEAVYLEPGDLLIVDNFRTTHARTPFSPRWDGKDRWLHRVYIRTDRNGQLSGGERAGDVVAFTPRG

>1QFTA

NQPDWADEAANGAHQDAWKSLKADVENVYYMVKATYKNDPVWGNDFTCVGVMANDVNEDEKSIQAEFLFMNNADTNMQFATEKVTAVKMYGYNRENAFRYETEDGQVFTDVIAYSDDNCDVIYVPGTDGNEEGYELWTTDYDNIPANCLNKFNEYAVGRETRDVFTSACLEIAAA

>4D9SA

GMAEDMAADEVTAPPRKVLIISAGASHSVALLSGDIVCSWGRGEDGQLGHGDAEDRPSPTQLSALDGHQIVSVTCGADHTVAYSQSGMEVYSWGWGDFGRLGHGNSSDLFTPLPIKALHGIRIKQIACGDSHCLAVTMEGEVQSWGRNQNGQLGLGDTEDSLVPQKIQAFEGIRIKMVAAGAEHTAAVTEDGDLYGWGWGRYGNLGLGDRTDRLVPERVTSTGGEKMSMVACGWRHTISVSYSGALYTYGWSKYGQLGHGDLEDHLIPHKLEALSNSFISQISGGWRHTMALTSDGKLYGWGWNKFGQVGVGNNLDQCSPVQVRFPDDQKVVQVSCGWRHTLAVTERNNVFAWGRGTNGQLGIGESVDRNFPKIIEALSVDGASGQHIESSNIDPSSGKSWVSPAE

>3W06A

GPMGVVEEAHNVKVIGSGEATIVLGHGFGTDQSVWKHLVPHLVDDYRVVLYDNMGAGTTNPDYFDFDRYSNLEGYSFDLIAILEDLKIESCIFVGHSVSAMIGVLASLNRPDLFSKIVMISASPRYVNDVDYQGGFEQEDLNQLFEAIRSNYKAWCLGFAPLAVGGDMDSIAVQEFSRTLFNMRPDIALSVGQTIFQSDMRQILPFVTVPCHILQSVKDLAVPVVVSEYLHANLGCESVVEVIPSDGHLPQLSSPDSVIPVILRHIRNDIAM

>2QTSA

STLHGISHIFSYERLSLKRVVWALCFMGSLALLALVCTNRIQYYFLYPHVTKLDEVAATRLTFPAVTFCNLNEFRFSRVTKNDLYHAGELLALLNNRYEIPDTQTADEKQLEILQDKANFRNFKPKPFNMLEFYDRAGHDIREMLLSCFFRGEQCSPEDFKVVFTRYGKCYTFNAGQDGKPRLITMKGGTGNGLEIMLDIQQDEYLPVWGETDETSFEAGIKVQIHSQDEPPLIDQLGFGVAPGFQTFVSCQEQRLIYLPPPWGDCKATTGDSEFYDTYSITACRIDCETRYLVENCNCRMVHMPGDAPYCTPEQYKECADPALDFLVEKDNEYCVCEMPCNVTRYGKELSMVKIPSKASAKYLAKKYNKSEQYIGENILVLDIFFEALNYETIEQKKAYEVAGLLGDIGGQMGLFIGASILTVLELFDYAYEVIKHR

>4GEYA

ANVRLQHHHHHHHLEAEAFSSESKWMTGDWGGTRTELLDKGYDFTLDYVGEVAGNLHGGYNDDKTARYSDQFALGAHLDLQKILGWHDAEFKLAITERSGRNLSNDRISDPRAGQFSSVQEVWGRGQTWRLTQMWIKQKYFDGALDVKFGRFGEGEDFNSFPCDFQNLAFCGSQVGNWVGGIWYNWPVSQWALRVKYNITPAFFVQVGAFEQNPSNLETGNGFKLSGSGTKGAIMPMEAVWSPKVNGLPGEYRLGYYYSTAKADDVYDDVNGNPQALTGEAFKSHSSKHGWWVVAQQQVTAHGGDVNRGLSLFANFTVHDKATNVVDNYQQVGLVYKGAFDARPKDDIGFGVARIHVNDDVKKRAELLNAQSGINDYDNPGFVPLQRTEYNAELYYGFHVTNWLTVRPNLQYIKSPGGVDEVDNALVAGLKIQSSF

>2PQRC

GSHMQKGQVGIFSFQNNYADSATTFRILAHLDEQRYPLPNGAAEKNLPSLFEGFKATVSIIQQR

>2HOXA

KMTWTMKAAEEAEAVANINCSEHGRAFLDGIISEGSPKCECNTCYTGPDCSEKIQGCSADVASGDGLFLEEYWKQHKEASAVLVSPWHRMSYFFNPVSNFISFELEKTIKELHEVVGNAAAKDRYIVFGVGVTQLIHGLVISLSPNMTATPDAPESKVVAHAPFYPVFREQTKYFDKKGYVWAGNAANYVNVSNPEQYIEMVTSPNNPEGLLRHAVIKGCKSIYDMVYYWPHYTPIKYKADEDILLFTMSKFTGHSGSRFGWALIKDESVYNNLLNYMTKNTEGTPRETQLRSLKVLKEVVAMVKTQKGTMRDLNTFGFKKLRERWVNITALLDQSDRFSYQELPQSEYCNYFRRMRPPSPSYAWVKCEWEEDKDCYQTFQNGRINTQNGVGFEASSRYVRLSLIKTQDDFDQLMYYLKDMVKAKRK

>3HLZA

GMQGKKFISPGAWFSMNYPSDWNEFEDGEGSFLFYNPDVWTGNFRISAFKGNASYGKDAIRQELKENDSASLVKIGTWDCAYSKEMFQEEGTYYTSHLWITGTGNIAFECSFTVPKGGSAKEAEEVIATLEARKEGEKYPAELIPVRLSEIYQINEGYEWVVSTVKQELKKDFQGVEEDLEKIQQVIDSGKISPKKKDEWLAIGITVCAILTNEVEGMEWKTLIDGNREVPVLEYQGRTIDPMKIAWSKVKAGQPCNIAEAYQSAIDHH

>1MTYB

ERRRGLTDPEMAAVILKALPEAPLDGNNKMGYFVTPRWKRLTEYEALTVYAQPNADWIAGGLDWGDWTQKFHGGRPSWGNETTELRTVDWFKHRDPLRRWHAPYVKDKAEEWRYTDRFLQGYSADGQIRAMNPTWRDEFINRYWGAFLFNEYGLFNAHSQGAREALSDVTRVSLAFWGFDKIDIAQMIQLERGFLAKIVPGFDESTAVPKAEWTNGEVYKSARLAVEGLWQEVFDWNESAFSVHAVYDALFGQFVRREFFQRLAPRFGDNLTPFFINQAQTYFQIAKQGVQDLYYNCLGDDPEFSDYNRTVMRNWTGKWLEPTIAALRDFMGLFAKLPAGTTDKEEITASLYRVVDDWIEDYASRIDFKADRDQIVKAVLAGLK

>2WH7A

MGSSHHHHHHSSGLVPRGSHNAVNIVMRQPTTPNFSSALNITSANEGGSAMQIRGVEKALGTLKITHENPSVDKEYDKNAAALSIDIVKKQKGGKGTAAQGIYINSTSGTTGKLLRIRNLNDDKFYVKPDGGFYAKETSQIDGNLKLKDPIANDHAATKAYVDGEVEKLKALLAAKQM

>2V0XA

AKSVVSHSLTTLGVEVSKPPPQHDKIEASEPSFPLHESILKVVEEEWQQIDRQLPSVACRYPVSSIEAARILSVPKVDDEILGFISEATPAAATQASSTESCDKHLDLALCRSYEAAASALQIAAHTAFVAKSLQADISQAAQIINSDPSDAQQALRILNRTYDAASYLCDAAFDEVRMSACAMGSSTMGRRYLWLKDCKISPASKNKLTVAPFKGGTLFGGEVHKVIKKRGNKQ

>4FUSA

MGSSHHHHHHSSGLVPRGSHMASSAEEYNERFMEMWNKIHDPANGYFSADGGPYHSVETLIVEAPDHGHESTSEAYSYFLLLEAYYGKVTGDWSKLRNAWAKMEEHIIPTQEMQPTNNFYNPSKPASYAAEHAQPSGYPSQLEFGVPVGEDPISAKLAQTYGSWDVYGMHWLLDMDNIYGYGNLGDGVSTPSYINTFQRGEQESVWETVTHPSWESFKWGGPNGFLPLFTKDNNYSRQWRYTNAPDADARAVQVMYWAYQWIKEQGKDPEQEVPGLMAKAAKMGDYLRLAMFDKYFKKMGTQDKNAQGGKGYESAHYLMSWYYAWGGAADANAGWAFRIGSSHVHFGYQNPIAAMALSEFDPLKPRTPGATEDWATGLKRSMEFYTWLQSAEGGIAGGATNSWDGSYKPHPQDRADATFYGMVYDENPVYHDPGSGTWFGWQAWSMQRVAEYYYLKGDAQAKQLMDKWAPWVLSNINWLEDGSFEIPATLEWTGKPEKWDPANPKANTNLHVSVVDHGQDLGIAAGVAKALMFYAAAAEKYDTPQNEAKEASKKLLDAMWTHFKTPKGLAAPEKRGDYARFFDKVYVPGEFNGSMANGDAINSESTFLSMRSFYLDDPMFKQVEDALNSGEDPVFTYHRFWAQTEAATAYANYAALFEGDNPCDEGCAPTAQPLSVSTRVNKAVSITLKGTDSDG

>2QZBA

SLAANPWNWFGSSTKVSEQGVGELTASTPLQEQAIADALDGDYRLRSGMKTANGNVVRFFEVMKGDNVAMVINGDQGTISRIDVLDSDIPADTGVKIGTPFSDLYSKAFGNCQKADGDDNRAVECKAEGSQHISYQFSGEWRGPEGLMPSDDTLKNWKVSKIIWRR

>3GIWA

GMGGAALPDNGWPADRIDTESAHSARIYDYIIGGKDYYPADKEAGDAMSREWPALPVHMRANRDWMNRAVAHLAKEAGIRQFLDIGTGIPTSPNLHEIAQSVAPESRVVYVDNDPIVLTLSQGLLASTPEGRTAYVEADMLDPASILDAPELRDTLDLTRPVALTVIAIVHFVLDEDDAVGIVRRLLEPLPSGSYLAMSIGTAEFAPQEVGRVAREYAARNMPMRLRTHAEAEEFFEGLELVEPGIVQVHKWHPDAATADGIRDEDIAMYGAVARKP

>3B79A

SNAMKDPLLNSLIYVSRYYGLANSPEALVNGLPLSDGKLTPFLLPRAAERAGLVAKENRAELEKISSLILPAILVLKGGDSCVLNSINMETREAEVTTLESGMVPISIPLEDLLEQYTGRYFLVKKQFR

>4I6MA

MTLNRKCVVIHNGSHRTVAGFSNVELPQCIIPSSYIKRTDEGGEAEFIFGTYNMIDAAAEKRNGDEVYTLVDSQGLPYNWDALEMQWRYLYDTQLKVSPEELPLVITMPATNGKPDMAILERYYELAFDKLNVPVFQIVIEPLAIALSMGKSSAFVIDIGASGCNVTPIIDGIVVKNAVVRSKFGGDFLDFQVHERLAPLIKEENDMENMADEQKRSTDVWYEASTWIQQFKSTMLQVSEKDLFELERYYKEQADIYAKQQEQLKQMDQQLQYTALTGSPNNPLVQKKNFLFKPLNKTLTLDLKECYQFAEYLFKPQLISDKFSPEDGLGPLMAKSVKKAGASINSMKANTSTNPNGLGTSHINTNVGDNNSTASSSNISPEQVYSLLLTNVIITGSTSLIEGMEQRIIKELSIRFPQYKLTTFANQVMMDRKIQGWLGALTMANLPSWSLGKWYSKEDYETLKRDRKQSQATNATN

>4JPQA

GQSGKSLSVKKVMCTASPEGEAVPSLLDGNGIEFQPLDVVNWKDYPYKPEVSFRIAHTGREILLHYKVKEASVRAVASGDNGRVWEDACVEFFVSPEGDDRYYNFECNCAGRLLIQGGAVNERRPTASQEVLGMVKRWSSLAGEPFEERLGECSWELVMVIPVSAFFQHSVGSLDGKTMKGNFYKCGDKLQTPHFLSWSPIGLERPMFHCPAFFGTLSFE

>4F2EA

GAMGQKAQQKNGYQEIRVEVMGGYTPELIVLKKSVPARIVFDRKDPSPCLDQIVFPDFGVHANLPMGEEYVVEITPEQAGEFSFACGMNMMHGKMIVE

>4IEFA

GPLGSQPAERGRNPQVRLLSAEQSMSKVQFRMDNLQFTGVQTSKGVAQVPTFTEGVNISEKGTPILPILSRSLAVSETRAMKVEVVSSKFIEKKDVLIAPSKGVISRAENPDQIPYVYGQSYNEDKFFPGEIATLSDPFILRDVRGQVVNFAPLQYNPVTKTLRIYTEIVVAVSETAEAGQNTISLVKNSTFTGFEDIYKSVFMNYEATR

>3CP7A

QNPADSPHIGKVFFSTNQGDFVCSANIVASANQSTVATAGHCLHDGNGGQFARNFVFAPAYDYGESEHGVWAAEELVTSAEWANRGDFEHDYAFAVLETKGGTTVQQQVGTASPIAFNQPRGQYYSAYGYPAAAPFNGQELHSCHGTATNDPMGSSTQGIPCNMTGGSSGGPWFLGNGTGGAQNSTNSYGYTFLPNVMFGPYFGSGAQQNYNYASTTN

>1Z3XA

PEFMVTTEPALADLQEQLYNGNEKSQLAAMSTLSTAGTEGYHLLQEFLKDSATFSPPPAPWIRGQAYRLLFHSPEASVQAFLQQHYPQGVIPLRSDRGVDYQELAKLLVAEKFEAADRLTTQKLCELAGPLAQKRRWLYFTEVEQLPIPDLQTIDQLWLAFSLGRFGYSVQRQLWLGCGQNWDRLWEKIGWRQGKRWPRYPNEFIWDLSAPRGHLPLTNQLRGVQVLNALLNHPAWTA

>2J8BA

MLQCYNCPNPTADCKTAVNCSSDFDACLITKAGLQVYNKCWKFEHCNFNDVTTRLRENELTYYCCKKDLCNFNEQLENG

>3F0DA

MAHHHHHHMGTLEAQTQGPGSMDFRIGQGYDVHQLVPGRPLIIGGVTIPYERGLLGHSDADVLLHAITDALFGAAALGDIGRHFSDTDPRFKGADSRALLRECASRVAQAGFAIRNVDSTIIAQAPKLAPHIDAMRANIAADLDLPLDRVNVKAKTNEKLGYLGRGEGIEAQAAALVVREAAA

>3DANA

MDPSSKPLREIPGSYGIPFFQPIKDRLEYFYGTGGRDEYFRSRMQKYQSTVFRANMPPGPFVSSNPKVIVLLDAKSFPILFDVSKVEKKDLFTGTYMPSTKLTGGYRVLSYLDPSEPRHAQLKNLLFFMLKNSSNRVIPQFETTYTELFEGLEAELAKNGKAAFNDVGEQAAFRFLGRAYFNSNPEETKLGTSAPTLISSWVLFNLAPTLDLGLPWFLQEPLLHTFRLPAFLIKSTYNKLYDYFQSVATPVMEQAEKLGVPKDEAVHNILFAVCFNTFGGVKILFPNTLKWIGLAGENLHTQLAEEIRGAIKSYGDGNVTLEAIEQMPLTKSVVYESLRIEPPVPPQYGKAKSNFTIESHDATFEVKKGEMLFGYQPFATKDPKVFDRPEEYVPDRFVGDGEALLKYVWWSNGPETESPTVENKQCAGKDFVVLITRLFVIELFRRYDSFEIELGESPLGAAVTLTFLKRASI

>2WQKA

MPTFLLVNDDGYFSPGINALREALKSLGRVVVVAPDRNLSGVGHSLTFTEPLKMRKIDTDFYTVIDGTPADCVHLGYRVILEEKKPDLVLSGINEGPNLGEDITYSGTVSGAMEGRILGIPSIAFSAFGRENIMFEEIAKVCVDIVKKVLNEGIPEDTYLNVNIPNLRYEEIKGIKVTRQGKRAYKERVFKYIDPYGKPFYWIAAEEFGWHAEEGTDYWAVLNGYVSVTPLHLDLTNYKVMKSIKYLEDSP

>2V73A

GMASIKGEVDEIANYGNLKITKEEERVNITGDLEKFSSLEEGTIVTRFNMNDTSIQSLIGLSDGNKANNYFSLYVSGGKVGYELRRQEGNGDFNVHHSADVTFNRGINTLALKIEKGIGAKIFLNGSLVKTVSDPNIKFLNAINLNSGFIGKTDRANGYNEYLFRGNIDFMNIYDKPVSDNYLLRKTGETK

>1HDHA

MSKRPNFLVIVADDLGFSDIGAFGGEIATPNLDALAIAGLRLTDFHTASTXSPTRSMLLTGTDHHIAGIGTMAEALTPELEGKPGYEGHLNERVVALPELLREAGYQTLMAGKWHLGLKPEQTPHARGFERSFSLLPGAANHYGFEPPYDESTPRILKGTPALYVEDERYLDTLPEGFYSSDAFGDKLLQYLKERDQSRPFFAYLPFSAPHWPLQAPREIVEKYRGRYDAGPEALRQERLARLKELGLVEADVEAHPVLALTREWEALEDEERAKSARAMEVYAAMVERMDWNIGRVVDYLRRQGELDNTFVLFMSDNGAEGALLEAFPKFGPDLLGFLDRHYDNSLENIGRANSYVWYGPRWAQAATAPSRLYKAFTTQGGIRVPALVRYPRLSRQGAISHAFATVMDVTPTLLDLAGVRHPGKRWRGREIAEPRGRSWLGWLSGETEAAHDENTVTGWELFGMRAIRQGDWKAVYLPAPVGPATWQLYDLARDPGEIHDLADSQPGKLAELIEHWKRYVSETGVVEGASPFLVR

>3KVCA

MSSQVEHPAGGYKKLFETVEELSSPLTAHVTGRIPLWLTGSLLRCGPGLFEVGSEPFYHLFDGQALLHKFDFKEGHVTYHRRFIRTDAYVRAMTEKRIVITEFGTCAFPDPCKNIFSRFFSYFRGVEVTDNALVNIYPVGEDYYACTETNFITKVNPETLETIKQVDLCNYVSVNGATAHPHIENDGTVYNIGNCFGKNFSIAYNIVKIPPLQADKEDPISKSEIVVQFPCSDRFKPSYVHSFGLTPNYIVFVETPVKINLFKFLSSWSLWGANYMDCFESNETMGVWLHIADKKRKKYINNKYRTSPFNLFHHINTYEDHEFLIVDLCCWKGFEFVYNYLYLANLRENWEEVKKNARKAPQPEVRRYVLPLNIDKADTGKNLVTLPNTTATAILCSDETIWLEPEVLFSGPRQAFEFPQINYQKYGGKPYTYAYGLGLNHFVPDRLCKLNVKTKETWVWQEPDSYPSEPIFVSHPDALEEDDGVVLSVVVSPGAGQKPAYLLILNAKDLSEVARAEVEINIPVTFHGLFKKS

>2V0XA

AKSVVSHSLTTLGVEVSKPPPQHDKIEASEPSFPLHESILKVVEEEWQQIDRQLPSVACRYPVSSIEAARILSVPKVDDEILGFISEATPAAATQASSTESCDKHLDLALCRSYEAAASALQIAAHTAFVAKSLQADISQAAQIINSDPSDAQQALRILNRTYDAASYLCDAAFDEVRMSACAMGSSTMGRRYLWLKDCKISPASKNKLTVAPFKGGTLFGGEVHKVIKKRGNKQ

>1NOWA

AKPGPALWPLPLSVKMTPNLLHLAPENFYISHSPNSTAGPSCTLLEEAFRRYHGYIFGFYKWHHEPAEFQAKTQVQQLLVSITLQSECDAFPNISSDESYTLLVKEPVAVLKANRVWGALRGLETFSQLVYQDSYGTFTINESTIIDSPRFSHRGILIDTSRHYLPVKIILKTLDAMAFNKFNVLHWHIVDDQSFPYQSITFPELSNKGSYSLSHVYTPNDVRMVIEYARLRGIRVLPEFDTPGHTLSWGKGQKDLLTPCYSRQNKLDSFGPINPTLNTTYSFLTTFFKEISEVFPDQFIHLGGDEVEFKCWESNPKIQDFMRQKGFGTDFKKLESFYIQKVLDIIATINKGSIVWQEVFDDKAKLAPGTIVEVWKDSAYPEELSRVTASGFPVILSAPWYLDLISYGQDWRKYYKVEPLDFGGTQKQKQLFIGGEACLWGEYVDATNLTPRLWPRASAVGERLWSSKDVRDMDDAYDRLTRHRCRMVERGIAAQPLYAGYCNHENM

>3FEGA

GSSHHHHHHSSGLVPRGSRRRASSLSRDAERRAYQWCREYLGGAWRRVQPEELRVYPVSGGLSNLLFRCSLPDHLPSVGEEPREVLLRLYGAILQGVDSLVLESVMFAILAERSLGPQLYGVFPEGRLEQYIPSRPLKTQELREPVLSAAIATKMAQFHGMEMPFTKEPHWLFGTMERYLKQIQDLPPTGLPEMNLLEMYSLKDEMGNLRKLLESTPSPVVFCHNDIQEGNILLLSEPENADSLMLVDFEYSSYNYRGFDIGNHFCEWVYDYTHEEWPFYKARPTDYPTQEQQLHFIRHYLAEAKKGETLSQEEQRKLEEDLLVEVSRYALASHFFWGLWSILQASMSTIEFGYLDYAQSRFQFYFQQKGQLTSVHSSS

>1R75A

MAHHHHHHMGSRISKEAAPVTFKNGKPTVKGTKTYPMFSNILYRIADTEARRWAFYNDSKELIIHVAVLFDYDSQIVPLGDTTAFRIDDPDEGNEDDFGKYLCEVDVRPLETQMFVEGSVTGWRVDTLEARTAEDERGYRL

>4KGDA

MVMKQTKQTNILAGAAVIKVLEAWGVDHLYGIPGGSINSIMDALSAERDRIHYIQVRHEEVGAMAAAADAKLTGKIGVCFGSAGPGGTHLMNGLYDAREDHVPVLALIGQFGTTGMNMDTFQEMNENPIYADVADYNVTAVNAATLPHVIDEAIRRAYAHQGVAVVQIPVDLPWQQIPAEDWYASANSYQTPLLPEPDVQAVTRLTQTLLAAERPLIYYGIGARKAGKELEQLSKTLKIPLMSTYPAKGIVADRYPAYLGSANRVAQKPANEALAQADVVLFVGNNYPFAEVSKAFKNTRYFLQIDIDPAKLGKRHKTDIAVLADAQKTLAAILAQVSERESTPWWQANLANVKNWRAYLASLEDKQEGPLQAYQVLRAVNKIAEPDAIYSIDVGDINLNANRHLKLTPSNRHITSNLFATMGVGIPGAIAAKLNYPERQVFNLAGDGGASMTMQDLATQVQYHLPVINVVFTNCQYGFIKDEQEDTNQNDFIGVEFNDIDFSKIADGVHMQAFRVNKIEQLPDVFEQAKAIAQHEPVLIDAVITGDRPLPAEKLRLDSAMSSAADIEAFKQRYEAQDLQPLSTYLKQFGLDDLQHQIGQGGF

>2VECA

MHHHHHHGTSLYKKAGSENLYFQGITTRTARQCGQADYGWLQARYTFSFGHYFDPKLLGYASLRVLNQEVLAPGAAFQPRTYPKVDILNVILDGEAEYRDSEGNHVQASAGEALLLSTQPGVSYSEHNLSKDKPLTRMQLWLDACPQRENPLIQKLALNMGKQQLIASPEGAMGSLQLRQQVWLHHIVLDKGESANFQLHGPRAYLQSIHGKFHALTHHEEKAALTCGDGAFIRDEANITLVADSPLRALLIDLPV

>2BLNA

MKTVVFAYHDMGCLGIEALLAAGYEISAIFTHTDNPGEKAFYGSVARLAAERGIPVYAPDNVNHPLWVERIAQLSPDVIFSFYYRHLIYDEILQLAPAGAFNLHGSLLPKYRGRAPLNWVLVNGETETGVTLHRMVKRADAGAIVAQLRIAIAPDDIAITLHHKLCHAARQLLEQTLPAIKHGNILEIAQRENEATCFGRRTPDDSFLEWHKPASVLHNMVRAVADPWPGAFSYVGNQKFTVWSSRVHPHASKAQPGSVISVAPLLIACGDGALEIVTGQAGDGITMQGSQLAQTLGLVQGSRLN

>1HYOA

GSMSFIPVAEDSDFPIQNLPYGVFSTQSNPKPRIGVAIGDQILDLSVIKHLFTGPALSKHQHVFDETTLNNFMGLGQAAWKEARASLQNLLSASQARLRDDKELRQRAFTSQASATMHLPATIGDYTDFYSSRQHATNVGIMFRGKENALLPNWLHLPVGYHGRASSIVVSGTPIRRPMGQMRPDNSKPPVYGACRLLDMELEMAFFVGPGNRFGEPIPISKAHEHIFGMVLMNDWSARDIQQWEYVPLGPFLGKSFGTTISPWVVPMDALMPFVVPNPKQDPKPLPYLCHSQPYTFDINLSVSLKGEGMSQAATICRSNFKHMYWTMLQQLTHHSVNGCNLRPGDLLASGTISGSDPESFGSMLELSWKGTKAIDVGQGQTRTFLLDGDEVIITGHCQGDGYRVGFGQCAGKVLPALSPA

>3QBXA

MPRYLGLMSGTSLDGMDIVLIEQGDRTTLLASHYLPMPAGLREDILALCVPGPDEIARAAEVEQRWVALAAQGVRELLLQQQMSPDEVRAIGSHGQTIRHEPARHFTVQIGNPALLAELTGIDVVADFRRRDVAAGGQGAPLVPAFHQALFGDDDTSRAVLNIGGFSNVSLLSPGKPVRGFDCGPGNVLMDAWIHHQRGEHFDRDGAWAASGQVNHALLASLLADEFFAARGPKSTGRERFNLPWLQEHLARHPALPAADIQATLLELSARSISESLLDAQPDCEEVLVCGGGAFNTALMKRLAMLMPEARVASTDEYGIPPAWMEGMAFAWLAHRFLERLPGNCPDVTGALGPRTLGALYPAGSHHHHHH

>2HY5A

MKFALQINEGPYQHQASDSAYQFAKAALEKGHEIFRVFFYHDGVNNSTRLTTPPQDDRHIVNRWAELAEQYELDMVVCVAAAQRRGIVDEGEASRNGKDATNIHPKFRISGLGQLVEAAIQADRLVVFGD

>1MQSA

GKSASMAVEEIASRKDISLRDMQISAILKMLFLNKDLNNNDNITTITDDIFNQQEIIWKVLILDIKSTATISSVLRVNDLLKAGITVHSLIKQDRSPLPDVPAIYFVSPTKENIDIIVNDLKSDKYSEFYINFTSSLPRNLLEDLAQQVSITGKSDKIKQVYDQYLDFIVTEPELFSLEISNAYLTLNDPKTTEEEITGLCANIADGLFNTVLTINSIPIIRAAKGGPAEIIAEKLGTKLRDFVINTNSSSTSTLQGNDSLERGVLIILDRNIDFASMFSHSWIYQCMVFDIFKLSRNTVTIPLESKENGTDNTTAKPLATKKYDIEPNDFFWMENSHLPFPEAAENVEAALNTYKEEAAEITRKTGVTNISDLDPNSNNDTVQIQEVVKKLPELTAKKNTIDTHMNIFAALLSQLESKSLDTFFEVEQDPGSTKTRSRFLDILKDGKTNNLEDKLRSFIVLYLTSTTGLPKDFVQNVENYFKENDYDINALKYVYKLREFMQLSNMSLQNKSLEDGSDSAFKPSNLTLSGIYGLTEGKLQGGVGSLISGIKKLLPEKKTIPITNVVDAIMDPLNSSQKNLETTDSYLYIDPKITRGSHTRKPKRQSYNKSLVFVVGGGNYLEYQNLQEWAHSQLHNPKKVMYGSTAITTPAEFLNEISRLGASNSSNNDA

>1I0RA

MDVEAFYKISYGLYIVTSESNGRKCGQIANTVFQLTSKPVQIAVCLNKENDTHNAVKESGAFGVSVLELETPMEFIGRFGFRKSSEFEKFDGVEYKTGKTGVPLVTQHAVAVIEAKVVKECDVGTHTLFVGEAVDAEVLKDAEVLTYADYHLMKKGKTPRTATVYFESK

>3RLKA

GHHHHHHSKPDPVIPDPPIDPPPGTGKYTCPFAIWSLEEVYEPPTKNRPWPIYNAVELQPREFDVALKDLLGNTKWRDWDSRLSYTTFRGCRGNGYIDLDATYLATDQAMRDQKYDIREGKKPGAFGNIERFIYLKSINAYCSLSDIAAYHADGVIVGFWRDPSSGGAIPFDFTKFDKTKCPIQAVIVVPRA

>1F00I

ASITEIKADKTTAVANGQDAITYTVKVMKGDKPVSNQEVTFTTTLGKLSNSTEKTDTNGYAKVTLTSTTPGKSLVSARVSDVAVDVKAPEVEFFTTLTIDDGNIEIVGTGVKGKLPTVWLQYGQVNLKASGGNGKYTWRSANPAIASVDASSGQVTLKEKGTTTISVISSDNQTATYTIATPNSLIVPNMSKRVTYNDAVNTCKNFGGKLPSSQNELENVFKAWGAANKYEYYKSSQTIISWVQQTAQDAKSGVASTYDLVKQNPLNNIKASESNAYATCVK

>3DMYA

MQQLEEALKQLAQGSGSSQALTQVRRWDSACQKLPDANLALISVAGEYAAELANQALDRNLNVMMFSDNVTLEDEIQLKTRAREKGLLVMGPDCGTSMIAGTPLAFANVMPEGNIGVIGASGTGIQELCSQIALAGEGITHAIGLGGRDLSREVGGISALTALEMLSADEKSEVLAFVSKPPAEAVRLKIVNAMKATGKPTVALFLGYTPAVARDENVWFASSLDEAARLACLLSRVTARRNAIAPVSSGFICGLYTGGTLAAEAAGLLAGHLGVEADDTHQHGMMLDADSHQIIDLGDDFYTVGRPHPMIDPTLRNQLIADLGAKPQVRVLLLDVVIGFGATADPAASLVSAWQKACAARLDNQPLYAIATVTGTERDPQCRSQQIATLEDAGIAVVSSLPEATLLAAALIHPLSPAAQQHTPSLLENVAVINIGLRSFALELQSASKPVVHYQWSPVAGGNKKLARLLERLQGHPHHH

>2YFRA

MDVKQVEKKDSVDKTNAEENKDSSVKPAENATKAELKGQVKDIVEESGVDTSKLTNDQINELNKINFSKEAKSGTQLTYNDFKKIAKTLIEQDARYAIPFFNASKIKNMPAAKTLDAQSGKVEDLEIWDSWPVQDAKTGYVSNWNGYQLVIGMMGVPNVNDNHIYLLYNKYGDNDFNHWKNAGPIFGLGTPVIQQWSGSATLNKDGSIQLYYTKVDTSDNNTNHQKLASATVYLNLEKDQDKISIAHVDNDHIVFEGDGYHYQTYDQWKETNKGADNIAMRDAHVIDDDNGNRYLVFEASTGTENYQGDDQIYQWLNYGGTNKDNLGDFFQILSNSDIKDRAKWSNAAIGIIKLNDDVKNPSVAKVYSPLISAPMVSDEIERPDVVKLGNKYYLFAATRLNRGSNDDAWMATNKAVGDNVAMIGYVSDNLTHGYVPLNESGVVLTASVPANWRTATYSYYAVPVEGRDDQLLITSYITNRGEVAGKGMHATWAPSFLLQINPDNTTTVLAKMTNQGDWIWDDSSENPDMMGVLEKDAPNSAALPGEWGKPVDWDLIGGYNLKPHQHHHHHH

>2BU3A

FCLTLRRRYTMGHHHHHHHHHHSSGHIEGRHMKLEQTLTLSPNLIGFNSNEGEKLLLTSRSREDFFPLSMQFVTQVNQAYCGVASIIMVLNSLGINAPETAQYSPYRVFTQDNFFSNEKTKAVIAPEVVARQGMTLDELGRLIASYGVKVKVNHASDTNIEDFRKQVAENLKQDGNFVIVNYLRKEIGQERGGHISPLAAYNEQTDRFLIMDVSRYKYPPVWVKTTDLWKAMNTVDSVSQKTRGFVFVSKTQDD

>4IOXA

MGSSHHHHHHSSGLVPRGSHMTGDDRPVLTKSAGERFLLYRPSTTTNSGLMAPDLYVYVDPAFTANTRASGTGVAVVGRYRDDYIIFALEHFFLRALTGSAPADIARCVVHSLTQVLALHPGAFRGVRVAVEGNSSQDSAVAIATHVHTEMHRLLASEGADAGSGPELLFYHCEPPGSAVLYPFFLLNKQKTPAFEHFIKKFNSGGVMASQEIVSATVRLQTDPVEYLLEQLNNLTETVSPNTDVRTYSGKRNGASDDLMVAVIMAIYLAAQAGPPHTFAPITRVS

>3E5TA

MRGSHHHHHHGIHMNSLIKENMRMMVVMEGSVNGYQFKCTGEGDGNPYMGTQTMRIKVVEGGPLPFAFDILATSFMYGSKTFIKHTKGIPDFFKQSFPEGFTWERVTRYEDGGVFTVMQDTSLEDGCLVYHAKVTGTNFPSNGAVMQKKTKGWEPNTEMLYPADGGLRGYSQMALNVDGGGYLSCSFETTYRSKKTVENFKMPGFHFVDHRLERLEESDKEMFVVQHEHAVAKFCDLPSKLGRL

>3ZSSA

MGSSHHHHHHSSGLVPRGSHMPATHHSSATSAERPTVVGRIPVLDVRPVVQRGRRPAKAVTGESFEVSATVFREGHDAVGANVVLRDPRGRPGPWTPMRELAPGTDRWGATVTAGETGTWSYTVEAWGDPVTTWRHHARIKIPAGLDTDLVLEEGARLYERAAADVPGREDRRELLAAVDALRDESRPAASRLAAALTPQVDAVLARHPLRDLVTSSDPLPLLVERERALYGAWYEFFPRSEGTPHTPHGTFRTAARRLPAIAAMGFDVVYLPPIHPIGTTHRKGRNNTLSATGDDVGVPWAIGSPEGGHDSIHPALGTLDDFDHFVTEAGKLGLEIALDFALQCSPDHPWVHKHPEWFHHRPDGTIAHAENPPKKYQDIYPIAFDADPDGLATETVRILRHWMDHGVRIFRVDNPHTKPVAFWERVIADINGTDPDVIFLAEAFTRPAMMATLAQIGFQQSYTYFTWRNTKQELTEYLTELSGEAASYMRPNFFANTPDILHAYLQHGGRPAFEVRAVLAATLSPTWGIYSGYELCENTPLREGSEEYLDSEKYQLKPRDWTRAAREGTTIAPLVTRLNTIRRENPALRQLRDLHFHPTDKEEVIAYSKRQGSNTVLVVVNLDPRHTQEATVSLDMPQLGLDWHESVPVRDELTGETYHWGRANYVRLEPGRTPAHVCTVLRPSHPQIGGSHTT

>4J5TA

AEFMEEYQKFTNESLLWAPYRSNCYFGMRPRYVHESPLIMGIMWFNSLSQDGLHSLRHFATPQDKLQKYGWEVYDPRIGGKEVFIDEKNNLNLTVYFVKSKNGENWSVRVQGEPLDPKRPSTASVVLYFSQNGGEIDGKSSLAMIGHDGPNDMKFFGYSKELGEYHLTVKDNFGHYFKNPEYETMEVAPGSDCSKTSHLSLQIPDKEVWKARDVFQSLVSDSIRDILEKEETKQRPADLIPSVLTIRNLYNFNPGNFHYIQKTFDLTKKDGFQFDITYNKLGTTQSISTREQVTELITWSLNEINARFDKQFSFGEGPDSIESVEVKRRFALETLSNLLGGIGYFYGNQLIDRETEFDESQFTEIKLLNAKEEGPFELFTSVPSRGFFPRGFYWDEGFHLLQIMEYDFDLAFEILASWFEMIEDDSGWIAREIILGNEARSKVPQEFQVQNPNIANPPTLLLAFSEMLSRAIENIGDFNSDSYHQVMFNSRTAKFMTNNLEANPGLLTEYAKKIYPKLLKHYNWFRKSQTGLIDEYEEILEDEGIWDKIHKNEVYRWVGRTFTHCLPSGMDDYPRAQPPDVAELNVDALAWVGVMTRSMKQIAHVLKLTQDEQRYAQIEQEVVENLDLLHWSENDNCYCDISIDPEDDEIREFVCHEGYVSVLPFALKLIPKNSPKLEKVVALMSDPEKIFSDYGLLSLSRQDDYFGKDENYWRGPIWMNINYLCLDAMRYYYPEVILDVAGEASNAKKLYQSLKINLSNNIYKVWEEQGYCYENYSPIDGHGTGAEHFTGWTALVVNILGRFRSHHHHHH

>1K5NA

GSHSMRYFHTSVSRPGRGEPRFITVGYVDDTLFVRFDSDAASPREEPRAPWIEQEGPEYWDRETQICKAKAQTDREDLRTLLRYYNQSEAGSHTLQNMYGCDVGPDGRLLRGYHQHAYDGKDYIALNEDLSSWTAADTAAQITQRKWEAARVAEQLRAYLEGECVEWLRRYLENGKETLQRADPPKTHVTHHPISDHEATLRCWALGFYPAEITLTWQRDGEDQTQDTELVETRPAGDRTFQKWAAVVVPSGEEQRYTCHVQHEGLPKPLTLRWEP

>1LMLA

VVRDVNWGALRIAVSTEDLTDPAYHCARVGQHVKDHAGAIVTCTAEDILTNEKRDILVKHLIPQAVQLHTERLKVQQVQGKWKVTDMVGDICGDFKVPQAHITEGFSNTDFVMYVASVPSEEGVLAWATTCQTFSDGHPAVGVINIPAANIASRYDQLVTRVVTHEMAHALGFSGPFFEDARIVANVPNVRGKNFDVPVINSSTAVAKAREQYGCDTLEYLEVEDQGGAGSAGSHIKMRNAQDELMAPAAAAGYYTALTMAIFQDLGFYQADFSKAEVMPWGQNAGCAFLTNKCMEQSVTQWPAMFCNESEDAIRCPTSRLSLGACGVTRHPGLPPYWQYFTDPSLAGVSAFMDYCPVVVPYSDGSCTQRASEAHASLLPFNVFSDAARCIDGAFRPKATDGIVKSYAGLCANVQCDTATRTYSVQVHGSNDYTNCTPGLRVELSTVSNAFEGGGYITCPPYVEVCQGNVQAAKDGGN

>3OT9A

GSHMASNKYKRIFLVVMDSVGIGEAPDAEQFGDLGSDTIGHIAEHMNGLQMPNMVKLGLGNIREMKGISKVEKPLGYYTKMQEKSTGKDTMTGHWEIMGLYIDTPFQVFPEGFPKELLDELEEKTGRKIIGNKPASGTEILDELGQEQMETGSLIVYTSADSVLQIAAHEEVVPLDELYKICKIARELTLDEKYMVGRVIARPFVGEPGNFTRTPNRHDYALKPFGRTVMNELKDSDYDVIAIGKISDIYDGEGVTESLRTKSNMDGMDKLVDTLNMDFTGLSFLNLVDFDALFGHRRDPQGYGEALQEYDARLPEVFAKLKEDDLLLITADHGNDPIHPGTDHTREYVPLLAYSPSMKEGGQELPLRQTFADIGATVAENFGVKMPEYGTSFLNELKK

>3SKVA

MGSSHHHHHHSSGLVPRGSHMTTQNTARARADRSVSPTDPALTYRGAVSLQDRDGWLAPWRAPHEDAYLYFPKGSVGRLAQTSGVRLHLRTDSPWLAVRYEAVGPKPKPGEPQPPAEPALLDVLVDGELARTVELKLDADAELHVDGLPAGDKLVELWLPTLLQFRLAEVRLEAGATLEKDTSSKPHWIHYGDSICHGRGAASPSRTWLALAARAEGLDLQSLSFAADGSHLQPMFARLIRDLPADLISLRVGTSNFMDGDGFVDFPANLVGFVQIIRERHPLTPIVLGSSVYSPFWDELPADDKPTVADYREQVVKVAELLRKHGDQNVHYLDGMRVWGPERGMELYLEKPDKYPTHPNAVGHEIFAESSRREMAALGVLPVRG

>3NOJA

MNTLIGKTGIVVRNIQRAELDSIDALGRLGVATVHEAQNRKGLLSSKMRPIQQGTSLAGSAVTVLVAPGDNWMFHVAVEQCRPGDVLVVSPSSPCTDGYFGDLLATSLQARGVRALIVDAGVRDTQTLRDMGFAVWARAINAQGTVKETLGSVNLPVICGGQLINPGDIVVADDDGVVVVRRDECESTLVAAAERAGLEEEKRLRLAAGELGLDIYKMRERLEAKGLRYVDNIEDLEG

>2WSDA

MTLEKFVDALPIPDTLKPVQQSKEKTYYEVTMEECTHQLHRDLPPTRLWGYNGLFPGPTIEVKRNENVYVKWMNNLPSTHFLPIDHTIHHSDSQHEEPEVKTVVHLHGGVTPDDSDGYPEAWFSKDFEQTGPYFKREVYHYPNQQRGAILWYHDHAMALTRLNVYAGLVGAYIIHDPKEKRLKLPSDEYDVPLLITDRTINEDGSLFYPSAPENPSPSLPNPSIVPAFCGETILVNGKVWPYLEVEPRKYRFRVINASNTRTYNLSLDNGGDFIQIGSDGGLLPRSVKLNSFSLAPAERYDIIIDFTAYEGESIILANSAGCGGDVNPETDANIMQFRVTKPLAQKDESRKPKYLASYPSVQHERIQNIRTLKLAGTQDEYGRPVLLLNNKRWHDPVTETPKVGTTEIWSIINPTRGTHPIHLHLVSFRVLDRRPFDIARYQESGELSYTGPAVPPPPSEKGWKDTIQAHAGEVLRIAATFGPYSGRYVWHCHALEHEDYDMMRPMDITDPHK

>4INWA

SPEIMKDLSINFGKALDTCKKELDLPDSINEDFYKFWKEDYEITNRLTGCAIKCLSEKLEMVDADGKLHHGNAREFAMKHGADDAMAKQLVDLIHGCEKSIPPNDDRCMEVLSIAMCFKKEIHNLKWAPNMEVVVGEVLA

>1YM3A

MAHHHHHHGPNTNPVAAWKALKEGNERFVAGRPQHPSQSVDHRAGLAAGQKPTAVIFGCADSRVAAEIIFDQGLGDMFVVRTAGHVIDSAVLGSIEYAVTVLNVPLIVVLGHDSCGAVNAALAAINDGTLPGGYVRDVVERVAPSVLLGRRDGLSRVDEFEQRHVHETVAILMARSSAISERIAGGSLAIVGVTYQLDDGRAVLRDHIGNIGEEV

>4HCEA

KAISYEQLSLASVGSVERLEGKIVGMNPPQFASINEFKYCTLKLYFTQLLPNVPDKVLVPGVNCIEIVIPTRERICELFGVLNCQSDKISDILLLEKPDRISVEVERILWDNDKTASPGMAVWSLKNISTDTQAQAQVQVPAQSSASIDPSR

>3RBYA

HMPALLKRLLFQVGPHPNERTFTLSSVSTDGHYISLRPFVKPSGDELSFPFEWAFAGTNETVKANDQGNGVVTQDFNFWLDTNVYLNVPNTHRGEVNTTWKNWDSGCVEETGAVYPFGADKESVSFREMWQPVDPSREDLVIVSPNNEKFSSNARSIVLKVTDEAYDGLVIVIGRWIQGFLSQKNNNTIEGLNFIRLLEKDSGKSEFLLSYGKEVNKIPQSYENLKKGSTVTSNGLNWEVIEYHA

>2TRCP

EGQATHTGPKGVINDWRKFKLESEDGDSIPPSKKEILRQMSSPQSRDDKDSKERMSRKMSIQEYELIHQDKEDEGCLRKYRRQCMQDMHQKLSFGPRYGFVYELETGEQFLETIEKEQKVTTIVVNIYEDGVRGCDALNSSLECLAAEYPMVKFCKIRASNTGAGDRFSSDVLPTLLVYKGGELISNFISVAEQFAEDFFAADVESFLNEYGLLPER

>2HNUA

VRTCLPCGPGGKGRCFGPSICCGDELGCFVGTAEALRCQEENYLPSPCQSGQKPCGSGGRCAAAGICCSPDGCHEDPACDP

>3K40A

MEAPEFKDFAKTMVDFIAEYLENIRERRVLPEVKPGYLKPLIPDAAPEKPEKWQDVMQDIERVIMPGVTHWHSPKFHAYFPTANSYPAIVADMLSGAIACIGFTWIASPACTELEVVMMDWLGKMLELPAEFLACSGGKGGGVIQGTASESTLVALLGAKAKKLKEVKELHPEWDEHTILGKLVGYCSDQAHSSVERAGLLGGVKLRSVQSENHRMRGAALEKAIEQDVAEGLIPFYAVVTLGTTNSCAFDYLDECGPVGNKHNLWIHVDAAYAGSAFICPEYRHLMKGIESADSFNFNPHKWMLVNFDCSAMWLKDPSWVVNAFNVDPLYLKHDMQGSAPDYRHWQIPLGRRFRALKLWFVLRLYGVENLQAHIRRHCNFAKQFGDLCVADSRFELAAEINMGLVCFRLKGSNERNEALLKRINGRGHIHLVPAKIKDVYFLRMAICSRFTQSEDMEYSWKEVSAAADEMEQEQ

>1VJNA

MGSDKIHHHHHHMKITWFGHACFALEMEGKTIVTDPFDESVGYPIPNVTADVVTESHQHFDHNAHHLVKGNFRVIDRPGAYTVNGVKIKGVETFHDPSHGRERGKNIVFVFEGEGIKVCHLGDLGHVLTPAQVEEIGEIDVLLVPVGGTYTIGPKEAKEVADLLNAKVIIPMHYKTKYLKFNLLPVDDFLKLFDSYERVGNILELFEKPKERKVVVMEVQ

>2IXDA

MSGLHILAFGAHADDVEIGMAGTIAKYTKQGYEVGICDLTEADLSSNGTIELRKEEAKVAARIMGVKTRLNLAMPDRGLYMKEEYIREIVKVIRTYKPKLVFAPYYEDRHPDHANCAKLVEEAIFSAGIRKYMPELSPHRVESFYNYMINGFHKPNFCIDISEYLSIKVEALEAYESQFSTGSDGVKTPLTEGYVETVIAREKMFGKEVGVLYAEGFMSKKPVLLHADLLGGCKLGHHHHHH

>4IUMA

GYNPPGDGACGYRCLAFMNGATVVSAGCSSDLWCDDELAYRVFQLSPTFTVTIPGGRVCPNAKYAMICDKQHWRVKRAKGVGLCLDESCFRGICNCQRMSGPPPAPVSAAVLDHILEAATFGNVRVVTPEGQGSSGHHHHHH

>3PZ7A

GPHMSSTCTKVLYFTDRSLTPFMVNIPKRLEEVTLKDFKAAIDREGNHRYHFKAMDPEFGTVKEEIFHDDDAIPGWEGKIVAWVEEDHGEN

>2Q88A

RDENKLEELKEQGFARIAIANEPPFTAVGADGKVSGAAPDVAREIFKRLGVADVVASISEYGAMIPGLQAGRHDAITAGLFMKPERCAAVAYSQPILCDAEAFALKKGNPLGLKSYKDIADNPDAKIGAPGGGTEEKLALEAGVPRDRVIVVPDGQSGLKMLQDGRIDVYSLPVLSINDLVSKANDPNVEVLAPVEGAPVYCDGAAFRKGDEALRDAFDVELAKLKESGEFAKIIEPYGFSAKAAMSTTREKLCAAK

>2VFOA

MDPDQYSIEADKKFKYSLKLSDYPTLQDAASAAVDGLLIDRDYNFYGGETVDFGGKVLTIECKAKFIGDGNLIFTKLGKGSRIAGVFMESTTTPWVIKPWTDDNQWLTDAAAVVATLKQSKTDGYQPTVSDYVKFPGIETLLPPNAKGQNITSTLEIRECIGVEVHRASGLMAGFLFRGCHFCKMVDANNPSGGKDGIITFENLSGDWGKGNYVIGGRTSYGSVSSAQFLRNNGGFERDGGVIGFTSYRAGESGVKTWQGTVGSTTSRNYNLQFRDSVVIYPVWDGFDLGADTDMNPELDRPGDYPITQYPLHQLPLNHLIDNLLVRGALGVGFGMDGKGMYVSNITVEDCAGSGAYLLTHESVFTNIAIIDTNTKDFQANQIYISGACRVNGLRLIGIRSTDGQSLTIDAPNSTVSGITGMVDPSRINVANLAEEGLGNIRANSFGYDSAAIKLRIHKLSKTLDSGALYSHINGGAGSGSAYTQLTAISGSTPDAVSLKVNHKDCRGAEIPFVPDIASDDFIKDSSCFLPYWENNSTSLKALVKKPNGELVRLTLATL

>1EYBA

MGHHHHHHHHHHSSGHIDDDDKHMGSMAELKYISGFGNECSSEDPRCPGSLPEGQNNPQVCPYNLYAEQLSGSAFTCPRSTNKRSWLYRILPSVSHKPFESIDEGHVTHNWDEVDPDPNQLRWKPFEIPKASQKKVDFVSGLHTLCGAGDIKSNNGLAIHIFLCNTSMENRCFYNSDGDFLIVPQKGNLLIYTEFGKMLVQPNEICVIQRGMRFSIDVFEETRGYILEVYGVHFELPDLGPIGANGLANPRDFLIPIAWYEDRQVPGGYTVINKYQGKLFAAKQDVSPFNVVAWHGNYTPYKYNLKNFMVINSVAFDHADPSIFTVLTAKSVRPGVAIADFVIFPPRWGVADKTFRPPYYHRNCMSEFMGLIRGHYEAKQGGFLPGGGSLHSTMTPHGPDADCFEKASKVKLAPERIADGTMAFMFESSLSLAVTKWGLKASRCLDENYHKCWEPLKSHFTPNSRNPAEPN

>3OYZA

MTERRHDREFVRTFFTSPTAVEGEDDSAKMLRRAAGLRGMQAPDVWVPDNEDATAPSMRDEGAENIVEVISEQGAEFPGEIHPRMVWHRDSPETRYQGFQHMLDITDPERGAVEHIHGFVIPEVGGIDDWKKADEFFTIVEHEHGLDEGSLAMSVIIESGEAELAMGDLRDEMGKPTNNLERLFLLVDGEVDYTKDMRAMTPTGELPAWPELRHNTSRGASAAGCVAVDGPYDDIRDVEGYRERMTDNQAKGMLGIWSLTPGQVVEANTSPLPPKTGSWLLDADGEEVELASEDGVEAYDGDRLSLEATDGGYELRVGGDARELTADELREELLGLTSYVPSMDDIVDSMEEFEAAKEAGRGAIAMTQSATLRIGGTEIDIEKDRMWDEATYQAAMTPISLFQDVYENRPDQHEELEERYGAGVVERAMEVGL

>1JKXA

MNIVVLISGNGSNLQAIIDACKTNKIKGTVRAVFSNKADAFGLERARQAGIATHTLIASAFDSREAYDRELIHEIDMYAPDVVVLAGFMRILSPAFVSHYAGRLLNIHPSLLPKYPGLHTHRQALENGDEEHGTSVHFVTDELDGGPVILQAKVPVFAGDSEDDITARVQTQEHAIYPLVISWFADGRLKMHENAAWLDGQRLPPQGYAADE

>2I0KA

STGPVAPLPTPPNFPNDIALFQQAYQNWSKEIMLDATWVCSPKTPQDVVRLANWAHEHDYKIRPRGAMAGWTPLTVEKGANVEKVILADTMTHLNGITVNTGGPVATVTAGAGASIEAIVTELQKHDLGWANLPAPGVLSIGGALAVNAHGAALPAVGQTTLPGHTYGSLSNLVTELTAVVWNGTTYALETYQRNDPRITPLLTNLGRCFLTSVTMQAGPNFRQRCQSYTDIPWRELFAPKGADGRTFEKFVAESGGAEAIWYPFTEKPWMKVWTVSPTKPDSSNEVGSLGSAGSLVGKPPQAREVSGPYNYIFSDNLPEPITDMIGAINAGNPGIAPLFGPAMYEITKLGLAATNANDIWGWSKDVQFYIKATTLRLTEGGGAVVTSRANIATVINDFTEWFHERIEFYRAKGEFPLNGPVEIRCCGLDQAADVKVPSVGPPTISATRPRPDHPDWDVAIWLNVLGVPGTPGMFEFYREMEQWMRSHYNNDDATFRPEWSKGWAFGPDPYTDNDIVTNKMRATYIEGVPTTENWDTARARYNQIDPHRVFTNGFMDKLLP

>4A5SA

SRKTYTLTDYLKNTYRLKLYSLRWISDHEYLYKQENNILVFNAEYGNSSVFLENSTFDEFGHSINDYSISPDGQFILLEYNYVKQWRHSYTASYDIYDLNKRQLITEERIPNNTQWVTWSPVGHKLAYVWNNDIYVKIEPNLPSYRITWTGKEDIIYNGITDWVYEEEVFSAYSALWWSPNGTFLAYAQFNDTEVPLIEYSFYSDESLQYPKTVRVPYPKAGAVNPTVKFFVVNTDSLSSVTNATSIQITAPASMLIGDHYLCDVTWATQERISLQWLRRIQNYSVMDICDYDESSGRWNCLVARQHIEMSTTGWVGRFRPSEPHFTLDGNSFYKIISNEEGYRHICYFQIDKKDCTFITKGTWEVIGIEALTSDYLYYISNEYKGMPGGRNLYKIQLIDYTKVTCLSCELNPERCQYYSVSFSKEAKYYQLRCSGPGLPLYTLHSSVNDKGLRVLEDNSALDKMLQNVQMPSKKLDFIILNETKFWYQMILPPHFDKSKKYPLLLDVYAGPCSQKADTVFRLNWATYLASTENIIVASFDGRGSGYQGDKIMHAINRRLGTFEVEDQIEAARQFSKMGFVDNKRIAIWGWSYGGYVTSMVLGSGSGVFKCGIAVAPVSRWEYYDSVYTERYMGLPTPEDNLDHYRNSTVMSRAENFKQVEYLLIHGTADDNVHFQQSAQISKALVDVGVDFQAMWYTDEDHGIASSTAHQHIYTHMSHFIKQCFSLPAAASWSHPQFEK

>2PSPA

EKPAACRCSRQDPKNRVNCGFPGITSDQCFTSGCCFDSQVPGVPWCFKPLPAQESEECVMQVSARKNCGYPGISPEDCAARNCCFSDTIPEVPWCFFPMSVEDCHY

>3FGRB

CSALIKLLPGGHDLLVAHNTWNSYQNMLRIIKKYRLQFREGPQEEYPLVAGNNLVFSSYPGTIFSGDDFYILGSGLVTLETTIGNKNPALWKYVQPQGCVLEWIRNVVANRLALDGATWADVFKRFNSGTYNNQWMIVDYKAFLPNGPSPGSRVLTILEQIPGMVVVADKTAELYKTTYWASYNIPYFETVFNASGLQALVAQYGDWFSYTKNPRAKIFQRDQSLVEDMDAMVRLMRYNDFLHDPLSLCEACNPKPNAENAISARSDLNPANGSYPFQALHQRAHGGIDVKVTSFTLAKYMSMLAASGPTWDQCPPFQWSKSPFHSMLHMGQPDLWMFSPIRVPWDGRGSHHHHHHG

>3MD9A

MAERIVTIGGDVTEIAYALGAGDEIVARDSTSQQPQAAQKLPDVGYMRTLNAEGILAMKPTMLLVSELAQPSLVLTQIASSGVNVVTVPGQTTPESVAMKINAVATALHQTEKGQKLIEDYQQRLAAVNKTPLPVKVLFVMSHGGLTPMAAGQNTAADAMIRAAGGSNAMQGFSRYRPLSQEGVIASAPDLLLITTDGVKALGSSENIWKLPGMALTPAGKHKRLLVVDDMALLGFGLETPQVLAQLREKMEQMQ

>3EKIA

MLKKLKNFILFSSIFSPIAFAISCSNTGVVKQEDVSVSQGQWDKSITFGVSEAWLNKKKGGEKVNKEVINTFLENFKKEFNKLKNANDKTKNFDDVDFKVTPIQDFTVLLNNLSTDNPELDFGINASGKLVEFLKNNPGIITPALETTTNSFVFDKEKDKFYVDGTDSDPLVKIAKEINKIFVETPYASWTDENHKWNGNVYQSVYDPTVQANFYRGMIWIKGNDETLAKIKKAWNDKDWNTFRNFGILHGKDNSSSKFKLEETILKNHFQNKFTTLNEDRSAHPNAYKQKSADTLGTLDDFHIAFSEEGSFAWTHNKSATKPFETKANEKMEALIVTNPIPYDVGVFRKSVNQLEQNLIVQTFINLAKNKQDTYGPLLGYNGYKKIDNFQKEIVEVYEKAIK

>1XS0A

QDDLTISSLAKGETTKAAFNQMVQGHKLPAWVMKGGTYTPAQTVTLGDETYQVMSACKPHDCGSQRIAVMWSEKSNQMTGLFSTIDEKTSQEKLTWLNVNDALSIDGKTVLFAALTGSLENHPDGFNFRSHHHHHH

>2YWIA

GHMEERVLGMPAVESNMFPLGKQAPPFALTNVIDGNVVRLEDVKSDAATVIMFICNHCPFVKHVQHELVRLANDYMPKGVSFVAINSNDAEQYPEDSPENMKKVAEELGYPFPYLYDETQEVAKAYDAACTPDFYIFDRDLKCVYRGQLDDSRPNNGIPVTGESIRAALDALLEGRPVPEKQKPSIGCSIKWKPSA

>3O3MB

MEAILSKMKEVVENPNAAVKKYKSETGKKAIGCFPVYCPEEIIHAAGMLPVGIWGGQTELDLAKQYFPAFACSIMQSCLEYGLKGAYDELSGVIIPGMCDTLICLGQNWKSAVPHIKYISLVHPQNRKLEAGVKYLISEYKGVKRELEEICGYEIEEAKIHESIEVYNEHRKTMRDFVEVAYKHSNTIKPSIRSLVIKSGFFMRKEEHTELVKDLIAKLNAMPEEVCSGKKVLLTGILADSKDILDILEDNNISVVADDLAQETRQFRTDVPAGDDALERLARQWSNIEGCSLAYDPKKKRGSLIVDEVKKKDIDGVIFCMMKFCDPEEYDYPLVRKDIEDSGIPTLYVEIDQQTQNNEQARTRIQTFAEMMSLASAWSHPQFEK

>3I4ZA

GSHGGSMKAANASSAEAYRVLSRAFRFDNEDQKLWWHSTAPMFAKMLETANYTTPCQYQYLITYKECVIPSLGCYPTNSAPRWLSILTRYGTPFELSLNCSNSIVRYTFEPINQHTGTDKDPFNTHAIWESLQHLLPLEKSIDLEWFRHFKHDLTLNSEESAFLAHNDRLVGGTIRTQNKLALDLKDGRFALKTYIYPALKAVVTGKTIHELVFGSVRRLAVREPRILPPLNMLEEYIRSRGSKSTASPRLVSCDLTSPAKSRIKIYLLEQMVSLEAMEDLWTLGGRRRDASTLEGLSLVRELWDLIQLSPGLKSYPAPYLPLGVIPDERLPLMANFTLHQNDPVPEPQVYFTTFGMNDMAVADALTTFFERRGWSEMARTYETTLKSYYPHADHDKLNYLHAYISFSYRDRTPYLSVYLQSFETGDWAVANLSESKVKCQDAACQPTALPPDLSKTGVYYSGLH

>2B06A

MSRSQLTILTNICLIEDLETQRVVMQYRAPENNRWSGYAFPGGHVENDEAFAESVIREIYEETGLTIQNPQLVGIKNWPLDTGGRYIVICYKATEFSGTLQSSEEGEVSWVQKDQIPNLNLAYDMLPLMEMMEAPDKSEFFYPRRTEDDWEKKIF

>3M0ZA

SNAMKLTPNFYRDRVCLNVLAGSKDNAREIYDAAEGHVLVGVLSKNYPDVASAVVDMRDYAKLIDNALSVGLGAGDPNQSAMVSEISRQVQPQHVNQVFTGVATSRALLGQNETVVNGLVSPTGTPGMVKISTGPLSSGAADGIVPLETAIALLKDMGGSSIKYFPMGGLKHRAEFEAVAKACAAHDFWLEPTGGIDLENYSEILKIALDAGVSKIIPHIYSSIIDKASGNTRPADVRQLLEMTKQLVK

>1VH4A

SLMAGLPNSSNALQQWHHLFEAEGTKRSPQAQQHLQQLLRTGLPTRKHENWKYTPLEGLINSQFVSIAGEISPQQRDALALTLDSVRLVFVDGRYVPALSDATEGSGYEVSINDDRQGLPDAIQAEVFLHLTESLAQSVTHIAVKRGQRPAKPLLLMHITQGVAGEEVNTAHYRHHLDLAEGAEATVIEHFVSLNDARHFTGARFTINVAANAHLQHIKLAFENPLSHHFAHNDLLLAEDATAFSHSFLLGGAVLRHNTSTQLNGENSTLRINSLAMPVKNEVCDTRTWLEHNKGFCNSRQLHKTIVSDKGRAVFNGLINVAQHAIKTDGQMTNNNLLMGKLAEVDTKPQLEIYADDVKCSHGATVGRIDDEQIFYLRSRGINQQDAQQMIIYAFAAELTEALRDEGLKQQVLARIGQRLPGGAREGGSHHHHHH

>4HD5A

MRKYAAIALCTSAILAGCNTSNVSQEPKKEKKVQEVAIQKEALQEQGKISYTPITHESTNTSIHITDLKDSLNEVQYKIWRTADGKERAKSFSSKEKEKQFTIPFDIKEFEGKRGEFQIEATGMKEDGKTIPLTKSIITFEQKVPVLMYHAIDDYHGQGIKDLFVSPANFEAQMKHLKDNGYTLLTFERWGDINKVNKPIFVTFDDGMKNNMNAFRVLQKLKDDTFKPAATEYMIVDNVDVEGALSTSEIKEMVDSGIFSVQSHTATHADLPKITNYEEELKGSKEKLEKITGKPVIAIAYXFGHVDDKVVTETKKYYQFATTTKPGQFITKGEPDELLKMKRVRIHHTTTVEQFASSIK

>3MVUA

GMSEPYGKAFSLMRAEAEPAWRAYTHHAFVEGLKAGTLPREAFLHYLQQDYVFLIHFSRAWALAVVKSETHSEMLAAVGTVNALVAEEMQLHIGICEASGISQEALFATRERAENLAYTRFVLEAGYSGDLLDLLAALAPCVMGYGEIGKRLTAEATSTLYGDWIDTYGGDDYQAACKAVGTLLDDALERRLGAEFTSSPRWSRLCQTFHTATELEVGFWQMGLTP

>3RC9A

MGSSHHHHHHENLYFQGHMENPANANPIRVGVIGCADIAWRRALPALEAEPLTEVTAIASRRWDRAKRFTERFGGEPVEGYPALLERDDVDAVYVPLPAVLHAEWIDRALRAGKHVLAEAPLTTDRPQAERLFAVARERGLLLMENFMFLHHPQHRQVADMLDEGVIGEIRSFAASFTIPPKPQGDIRYQADVGGGALLDIGVYPIRAAGLFLGADLEFVGAVLRHERDRDVVVGGNALLTTRQGVTAQLTFGMEHAYTNNYEFRGSTGRLWMNRVFTPPATYQPVVHIERQDHAEQFVLPAHDQFAKSIRAFAQAVLSGEHPREWSEDSLRQASLVDAVRTGARDIYFP

>3RM3A

MGSSHHHHHHSSGLVPRGSHMSEQYPVLSGAEPFYAENGPVGVLLVHGFTGTPHSMRPLAEAYAKAGYTVCLPRLKGHGTHYEDMERTTFHDWVASVEEGYGWLKQRCQTIFVTGLSMGGTLTLYLAEHHPDICGIVPINAAVDIPAIAAGMTGGGELPRYLDSIGSDLKNPDVKELAYEKTPTASLLQLARLMAQTKAKLDRIVCPALIFVSDEDHVVPPGNADIIFQGISSTEKEIVRLRNSYHVATLDYDQPMIIERSLEFFAKHAG

>3B79A

SNAMKDPLLNSLIYVSRYYGLANSPEALVNGLPLSDGKLTPFLLPRAAERAGLVAKENRAELEKISSLILPAILVLKGGDSCVLNSINMETREAEVTTLESGMVPISIPLEDLLEQYTGRYFLVKKQFR

>3BA3A

GMDISLLKQVVQSTNKIALSTAVNNEADVKIVNFVWYEAQPDTLYFSSVKTSPALKVYDQNPDIAFITIPNDGTAGNPYLRAQHVKLQRSTKTMTDLLPQYLETVPNYQQVWDAIGSTLVVFELKLTDLFVDAGVGGEKQTLTFN

>1VZMA

AAKELTLAQTESLREVCETNMACDEMADAQGIVAAYQAFYGPIPF

>3RKCA

SRPFSVLRANDVLWLSLTAAEYDQTTYGSSTNPMYVSDTVTFVNVATGAQGVSRSLDWSKVTLDGRPLTTIQQYSKTFFVLPLRGKLSFWEAGTTKAGYPYNYNTTASDQILIENAPGHRVCISTYTTNLGSGPVSISAVGVLAPHSA

>2NMMA

MGHHHHHHSHMAVADLALIPDVDIDSDGVFKYVLIRVHSAPRSGAPAAESKEIVRGYKWAEYHADIYDKVSGDMQKQGCDCECLGGGRISHQSQDKKIHVYGYSMAYGPAQHAISTEKIKAKYPDYEVTWANDGY

>2DDRA

EVSTTQNDTLKVMTHNVYMLSTNLYPNWGQTERADLIGAADYIKNQDVVILNEVFDNSASDRLLGNLKKEYPNQTAVLGRSSGSEWDKTLGNYSSSTPEDGGVAIVSKWPIAEKIQYVFAKGCGPDNLSNKGFVYTKIKKNDRFVHVIGTHLQAEDSMCGKTSPASVRTNQLKEIQDFIKNKNIPNNEYVLIGGDMNVNKINAENNNDSEYASMFKTLNASVPSYTGHTATWDATTNSIAKYNFPDSPAEYLDYIIASKDHANPSYIENKVLQPKSPQWTVTSWFQKYTYNDYSDHYPVEATISMK

>3RFRA

MKKLVKLAAFGAAAAVAATLGAIAPASAHGEKSQQAFLRMRTLNWYDVQWSKTTVNVNEEMILSGKVHVFSAWPQAVANPRVSFLNAGEPGPVLVRTAQFIGEQFAPRSVSLEIGKDYAFSINLRGRRAGRWHVHAQINVEGGGPIIGPGQWIEIKGDMKDFTDPVTLLDGSTVDLENYGISRIYAWHLPWLAVGAAWILFWFIRKGIIASYVRVAEGRPDDVIGDDDRRIGAIVLALTILATIVGYAVTNSTFPRTIPLQAGLQKPLTPIETEGTVGVGKEQVTTELNGGVYKVPGRELTINVKVKNGTSQPVRLGEYTAAGLRFLNPTVFTQKPDFPDYLLADRGLSNDDVIAPGESKEIVVKIQDARWDIERLSDLAYDTDSQVGGLLFFFTPDGKRFAAEIGGPVIPKFVAGDMP

>3RFRA

MKKLVKLAAFGAAAAVAATLGAIAPASAHGEKSQQAFLRMRTLNWYDVQWSKTTVNVNEEMILSGKVHVFSAWPQAVANPRVSFLNAGEPGPVLVRTAQFIGEQFAPRSVSLEIGKDYAFSINLRGRRAGRWHVHAQINVEGGGPIIGPGQWIEIKGDMKDFTDPVTLLDGSTVDLENYGISRIYAWHLPWLAVGAAWILFWFIRKGIIASYVRVAEGRPDDVIGDDDRRIGAIVLALTILATIVGYAVTNSTFPRTIPLQAGLQKPLTPIETEGTVGVGKEQVTTELNGGVYKVPGRELTINVKVKNGTSQPVRLGEYTAAGLRFLNPTVFTQKPDFPDYLLADRGLSNDDVIAPGESKEIVVKIQDARWDIERLSDLAYDTDSQVGGLLFFFTPDGKRFAAEIGGPVIPKFVAGDMP

>1H8PA

DQDEGVSTEPTQDGPAELPEDEECVFPFVYRNRKHFDCTVHGSLFPWCSLDADYVGRWKYCAQRDYAKCVFPFIYGGKKYETCTKIGSMWMSWCSLSPNYDKDRAWKYC

>4F2EA

GAMGQKAQQKNGYQEIRVEVMGGYTPELIVLKKSVPARIVFDRKDPSPCLDQIVFPDFGVHANLPMGEEYVVEITPEQAGEFSFACGMNMMHGKMIVE

>3N91A

GSDNEFPDFDYQTVYFANQYGLRTIELGESEFVDNTLDNQHKMVIKAAWGGGYTNRNNVVINFKVDESLCDNLYFKDTDQPLVPMPASYYTLASDRIAIPKGQIMAGVEVQLTDDFFADEKSISENYVIPLLMTNVQGADSILQGKPVVENPVLTNAGDWSILPQNFVLYAVKYVNPWHGEYLRRGIDHATVAGTSKDIIRHEQFVENDEVVNISTKSMKDNLLTLKTKDESGKDISYTVRLSFAEDGSCTVHSGSQNVVVSGSGKFVSKGEKNSLGGKDRNAIYLDYTVNLTDNNIQLATKDTLVLRTRNVYGGKSLEVVRK

>2PKFA

GTEDLYFQSHMTIAVTGSIATDHLMRFPGRFSEQLLPEHLHKVSLSFLVDDLVMHRGGVAGNMAFAIGVLGGEVALVGAAGADFADYRDWLKARGVNCDHVLISETAHTARFTCTTDVDMAQIASFYPGAMSEARNIKLADVVSAIGKPELVIIGANDPEAMFLHTEECRKLGLAFAADPSQQLARLSGEEIRRLVNGAAYLFTNDYEWDLLLSKTGWSEADVMAQIDLRVTTLGPKGVDLVEPDGTTIHVGVVPETSQTDPTGVGDAFRAGFLTGRSAGLGLERSAQLGSLVAVLVLESTGTQEWQWDYEAAASRLAGAYGEHAAAEIVAVLA

>2VZPA

SDPVDYQAEDATIVQGAVESNHAGYTGTGFVNYDNVAGSSVEWTVTVPSAGTYDVVVRYANGTTTSRPLDFSVNGSISASGVAFGSTGTWPAWTTKTVRVTLAAGVNKIKAVATTANGGPNVDKITL

>4A9CA

SMDEPDMISVFIGTWNMGSVPPPKNVTSWFTSKGLGKTLDEVTVTIPHDIYVFGTQENSVGDREWLDLLRGGLKELTDLDYRPIAMQSLWNIKVAVLVKPEHENRISHVSTSSVKTGIANTLGNKGAVGVSFMFNGTSFGFVNCHLTSGNEKTARRNQNYLDILRLLSLGDRQLNAFDISLRFTHLFWFGDLNYRLDMDIQEILNYISRKEFEPLLRVDQLNLEREKHKVFLRFSEEEISFPPTYRYERGSRDTYAWHKQKPTGVRTNVPSWCDRILWKSYPETHIICNSYGCTDDIVTSDHSPVFGTFEVGVTSQ

>2WE3A

GAMGSGIPMEACPHIRYAFQNDKLLLQQASVGRLTLVNKTTILLRPMKTTTVDLGLYARPPEGHGLMLWGSTSRPVTSHVGIIDPGYTGELRLILQNQRRYNSTLRPSELKIHLAAFRYATPQMEEDKGPINHPQYPGDVGLDVSLPKDLALFPHQTVSVTLTVPPPSIPHHRPTIFGRSGLAMQGILVKPCRWRRGGVDVSLTNFSDQTVFLNKYRRFCQLVYLHKHHLTSFYSPHSDAGVLGPRSLFRWASCTFEEVPSLAM

>3B5OA

GMEFNHLTKQLNQLLAQDYVAFSITENPVVQMLSQASFAQIAYVMQQYSIFPKELVGFTELARRKALGAGWNGVAQELQENIDEEMGSTTGGISHYTLLADGLEEGLGVAVKNTMPSVATSKLLRTVLSLFDRQVDYVLGATYAIEATSIPELTLIVKLVEWLHEGAIPKDLQYFFSKHLDEWEIEHEAGLRTSVAAYIQPEEFGEFAAGFRAMIDAMQVWWQELAQEAISSEVVLSTAIAQHH

>4G2SA

GSSPGPYIVRLLNSSLNGCEFPLLTGRTLFVVGQSDALTASGQLPDIPADSFFIPLDHGGVNFEIQVDTDATEIILHELKEGNSESRSVQLNTPIQVGELLILIRPESEPW

>1T8SA

MNNKGSGLTPAQALDKLDALYEQSVVALRNAIGNYITSGELPDENARKQGLFVYPSLTVTWDGSTTNPPKTRAFGRFTHAGSYTTTITRPTLFRSYLNEQLTLLYQDYGAHISVQPSQHEIPYPYVIDGSELTLDRSMSAGLTRYFPTTELAQIGDETADGIYHPTEFSPLSHFDARRVDFSLARLRHYTGTPVEHFQPFVLFTNYTRYVDEFVRWGCSQILDPDSPYIALSCAGGNWITAETEAPEEAISDLAWKKHQMPAWHLITADGQGITLVNIGVGPSNAKTICDHLAVLRPDVWLMIGHCGGLRESQAIGDYVLAHAYLRDDHVLDAVLPPDIPIPSIAEVQRALYDATKLVSGRPGEEVKQRLRTGTVVTTDDRNWELRYSASALRFNLSRAVAIDMESATIAAQGYRFRVPYGTLLCVSDKPLHGEIKLPGQANRFYEGAISEHLQIGIRAIDLLRAEGDRLHSRKLRTFNEPPFR

>2XD7A

DGPGDGFTILSSKSLVLGQKLSLTQSDISHIGSMRVEGIVHPTTAEIDLKEDIGKALEKAGGKEFLETVKELRKSQGPLEVAEAAVSQSSGLAAKFVIHCHIPQWGSDKCEEQLEETIKNCLSAAEDKKLKSVAFPPFPSGRNCFPKQTAAQVTLKAISAHFDDSSASSLKNVYFLLFDSESIGIYVQEMAKL

>1VH4A

SLMAGLPNSSNALQQWHHLFEAEGTKRSPQAQQHLQQLLRTGLPTRKHENWKYTPLEGLINSQFVSIAGEISPQQRDALALTLDSVRLVFVDGRYVPALSDATEGSGYEVSINDDRQGLPDAIQAEVFLHLTESLAQSVTHIAVKRGQRPAKPLLLMHITQGVAGEEVNTAHYRHHLDLAEGAEATVIEHFVSLNDARHFTGARFTINVAANAHLQHIKLAFENPLSHHFAHNDLLLAEDATAFSHSFLLGGAVLRHNTSTQLNGENSTLRINSLAMPVKNEVCDTRTWLEHNKGFCNSRQLHKTIVSDKGRAVFNGLINVAQHAIKTDGQMTNNNLLMGKLAEVDTKPQLEIYADDVKCSHGATVGRIDDEQIFYLRSRGINQQDAQQMIIYAFAAELTEALRDEGLKQQVLARIGQRLPGGAREGGSHHHHHH

>2VX8A

GSVASVHASISGSSASSTSSTPEVKPLKSLLGDSAPTLHLNKGMAILFAVVARGTTILAKHAWCGGNFLEVTEQILAKIPSENNKLTYSHGNYLFHYICQDRIVYLCITDDDFERSRAFSFLNEVKKRFQTTYGSRAQTALPYAMNSEFSSVLAAQLKHHSENHHHHHH

>3PE9A

MVKLTAPKSNVVAYGNEFLKITATASDSDGKISRVDFLVDGEVIGSDREAPYEYEWKAVEGNHEISVIAYDDDDAASTPDSVKIFVKQARLEHHHHHH

>2RKNA

AIDLCGMSQDELNECKPAVSKENPTSPSQPCCTALQHADFACLCGYKNSPWLGSFGVDPELASALPKQCGLANAPTC

>1JNIA

DAPAVGKDLTQAAENIPPAFHNAPRQGELPALNYVNQPPMVPHSVANYQVTKNVNQCLNCHSPENSRLSGATRISPTHFMDRDGKVGSSSSPRRYFCLQCHVSQANVDPIVPNDFKPMKGYGN

>1T61A

SVDHGFLVTRHSQTTDDPQCPPGTKILYHGYSLLYVQGNERAHGQDLGTAGSCLRKFSTMPFLFCNINNVCNFASRNDYSYWLSTPEPMPMSMAPITGENIRPFISRCAVCEAPAMVMAVHSQTIQIPQCPTGWSSLWIGYSFVMHTSAGAEGSGQALASPGSCLEEFRSAPFIECHGRGTCNYYANAYSFWLATIERSEMFKKPTPSTLKAGELRTHVSRCQVCMRRT

>3CTPA

SLANIREIAKRAGISIATVSRHLNNTGYVSEDAREKIQKVVDELNYTPNALARAMFTKNSKTIGLMVPNISNPFFNQMASVIEEYAKNKGYTLFLCNTDDDKEKEKTYLEVLQSHRVAGIIASRSQCEDEYANIDIPVVAFENHILDNIITISSDNYNGGRMAFDHLYEKGCRKILHIKGPEVFEATELRYKGFLDGARAKDLEIDFIEFQHDFQVKMLEEDINSMKDIVNYDGIFVFNDIAAATVMRALKKRGVSIPQEVQIIGFDNSFIGELLYPSLTTINQPIEALAYTIIELLIKIINGEGVLIEDYIMEVKLIERETTISLKDEG

>2YGBA

MAHHHHHHSSGLEVLFQGPNNTIINSLIGGDDSIKRSNVFAVDSQIPTLYMPQYISLSGVMTNDGPDNQAIASFEIRDQYITALNHLVLSLELPEVKGMGRFGYVPYVGYKCINHVSISSCNGVIWEIEGEELYNNCINNTIALKHSGYSSELNDISIGLTPNDTIKEPSTVYVYIKTPFDVEDTFSSLKLSDSKITVTVTFNPVSDIVIRDSSFDFETFNKEFVYVPELSFIGYMVKNVQIKPSFIEKPRRVIGQINQPTATVTEVHAATSLSVYTKPYYGNTDNKFISYPGYSQDEKDYIDAYVSRLLDDLVIVSDGPPTGYPESAEIVEVPEDGIVSIQDADVYVKIDNVPDNMSVYLHTNLLMFGTRKNSFIYNISKKFSAITGTYSDATKRTIFAHISHSINIIDTSIPVSLWTSQRNVYNGDNRSAESKAKDLFINDPFIKGIDFKNKTDIISRLEVRFGNDVLYSENGPISRIYNELLTKSNNGTRTLTFNFTPKIFFRPTTITANVSRGKDKLSVRVVYSTMDVNHPIYYVQKQLVVVCNDLYKVSYDQGVSITKIMGDNN

>4J32A

MGHHHHHHLYFQGMNRPSFNEAWLAFRKVNHSVADVGSIIGGNVGKNITGGYFQNACPIRMSYVLNATGFPIARNSPYAKVSGADNKFYIYRVNDMIDYLTHTMGKPDLIVNNPKQSDFIGKKGIIVVKGHGWSNARGHVTLWNGSICSDQCHLLNDPDNGPFVPEVGTLWILP

>3U52C

MSIEIKTNSVEPIRHTYGHIARRFGDKPATRYQEASYDIEAKTNFHYRPQWDSEHTLNDPTRTAIRMEDWCAVSDPRQFYYGAYVGNRAKMQESAETSFGFCEKRNLLTRLSEETQKQLLRLLVPLRHVELGANMNNAKIAGDATATTVSQMHIYTGMDRLGIGQYLSRIALMIDGSTGAALDESKAYWMDDEMWQPMRKLVEDTLVVDDWFELTLVQNILIDGMMYPLVYDKMDQWFESQGAEDVSMLTEFMRDWYKESLRWTNAMMKAVAGESETNRELLQKWIDHWEPQAYEALKPLAEASVGIDGLNEARAELSARLKKFELQSRGVSA

>1VPBA

MGSDKIHHHHHHMITDENKKLAQWAMDYALKNGCQAAKVLLYSSSNTSFELRDAKMDRLQQASEGGLSLSLYVDGRYGSISTNRLNRKELETFIKNGIDSTRYLAKDEARVLADPSRYYKGGKPDLKLYDAKFASLNPDDKIEMAKAVAEEALGKDERIISVGSSYGDGEDFAYRLISNGFEGETKSTWYSLSADITIRGEGEARPSAYWYESSLYMNDLIKKGIGQKALERVLRKLGQKKVQSGKYTMVVDPMNSSRLLSPMISALNGSALQQKNSFLLNKLNEKIASDRLTLTDEPHLVKASGARYFDNEGIATERRSIFDKGVLNTYFIDTYNAKKMGVDPTISGSSILVMETGDKNLDGLIAGVEKGILVTGFNGGNNNSSTGDFSYGIEGFLIENGKLTQPVSEMNVTGNLITLWNSLVATGNDPRLNSSWRIPSLVFEGVDFSGL

>1WC2A

NQKCSGNPRRYNGKSCASTTNYHDSHKGACGCGPASGDAQFGWNAGSFVAAASQMYFDSGNKGWCGQHCGQCIKLTTTGGYVPGQGGPVREGLSKTFMITNLCPNIYPNQDWCNQGSQYGGHNKYGYELHLDLENGRSQVTGMGWNNPETTWEVVNCDSEHNHDHRTPSNSMYGQCQCAHQ

>2CW9A

GSSGSSGDESDNAFIRASRALTDKVTDLLGGLFSKTEMSEVLTEILRVDPAFDKDRFLKQCENDIIPNVLEAMISGELDILKDWCYEATYSQLAHPIQQAKALGLQFHSRILDIDNVDLAMGKMVEQGPVLIITFQAQLVMVVRNPKGEVVEGDPDKVLRMLYVWALCRDQDELNPYAAWRLLDISASSTEQIL

>4HZ4A

MVMITLHYLKQSCSHRIVWLLEALGLDYELKIYDRLEGTGFAPEELKAQHPLGKAPVLQDGDLVLAEGNAIIQHLLDRYDTENRFTPAHKTDAYSNYVYWLAISASMFSANLLALVSKKGDLGDFAQYTNAQVGLYFSHVEKSLEGKTWIVGEQLTGADFALSFPLQWGLNYVNKADYPNITRYLEQIETHPAYLKANEKTDGGLDLSRFAENLYFQ

>1OFLA

EVVASNETLYQVVKEVKPGGLVQIADGTYKDVQLIVSNSGKSGLPITIKALNPGKVFFTGDAKVELRGEHLILEGIWFKDGNRAIQAWKSHGPGLVAIYGSYNRITACVFDCFDEANSAYITTSLTEDGKVPQHCRIDHCSFTDKITFDQVINLNNTARAIKDGSVGGPAMYHRVDHCFFSNPQKPGNAGGGIRIGYYRNDIGRCLVDSNLFMRQDSEAEIITSKSQENVYYGNTYLNCQGTMNFRHGDHQVAINNFYIGNDQRFGYGGMFVWGSRHVIACNYFELSETIKSRGNAALYLNPGAMASEHALAFDMLIANNAFINVNGYAIHFNPLDERRKEYCAANRLKFETPHQLMLKGNLFFKDKPYVYPFFKDDYFIAGKNSWTGNVALGVEKGIPVNISANRSAYKPVKIKDIQPIEGIALDLNALISKGITGKPLSWDEVRPYWLKEMPGTYALTARLSADRAAKFKAVIKRNKEH

>2OAFA

GMQGAGRLMQPRPDSAFVHDVRVTWGDCDPAKIAYTGHLPRFALEAIDAWWSEYHGPGGWYHLELDTNVGTPFVRLEMDFKSPVTPRHILKCHTWPTRLGTKSITFRVDGVQDGVTCFVGAFTCVFTIADQFKSQPAPDHLRALIEPHIPA

>3ML3A

MGSSHHHHHHSSGLVPRGSHMSGTVLINNINAPFLPDPVIVTGNMTLEKNGHVILNNSSSNVGQTYVQKGNWHGKGGILSLGAVLGNDNSKTDRLEIAGHASGITYVAVTNEGGSGDKTLEGVQIISTDSSDKNAFIQKGRIVAGSYDYRLKQGTASGLNTNKWYLTSQMDNQESKQMSNQESTQMSSR

>1W5QA

MSFTPANRAYPYTRLRRNRRDDFSRRLVRENVLTVDDLILPVFVLDGVNQRESIPSMPGVERLSIDQLLIEAEEWVALGIPALALFPVTPVEKKSLDAAEAYNPEGIAQRATRALRERFPELGIITDVCLCEFTTHGQCGILDDDGYVLNDVSIDVLVRQALSHAEAGAQVVAPSDMMDGRIGAIREALESAGHTNVRVMAYSAKYASAYYGPFRDAVGSASNLGKGNRATYQMDPANSDEALHEVAADLAEGADMVMVKPGMPYLDIVRRVKDEFRAPTFVYQVSGEYAMHMGAIQNGWLAESVILESLTAFKRAGADGILTYFAKQAAEQLRRGR

>3IFNP

DAEFRHDSGYEVHHQKLVFFAEDVGSNKGAIIGLMVGGVV

>3KS6A

GMTRIASHRGGTLEFGDSTPHGFTATAAMALEEVEFDLHPTADGAIVVHHDPTLDATTDMTGAIVDMTLAKVKTATIRYGAGSHPMTLEELCALYVDSHVNFRCEIKPGVDGLPYEGFVALVIAGLERHSMLERTTFSSFLLASMDELWKATTRPRLWLVSPSVLQQLGPGAVIETAIAHSIHEIGVHIDTADAGLMAQVQAAGLDFGCWAAHTPSQITKALDLGVKVFTTDRPTLAIALRTEHRMEASV

>3IT3A

MVGYSSKLIFVSMITRHGDRAPFANIENANYSWGTELSELTPIGMNQEYNLGLQLRKRYIDKFGLLPEHYVDQSIYVLSSHTNRTVVSAQSLLMGLYPAGTGPLIGDGDPAIKDRFQPIPIMTLSADSRLIQFPYEQYLAVLKKYVYNSPEWQNKTKEAAPNFAKWQQILGNRISGLNDVITVGDVLIVAQAHGKPLPKGLSQEDADQIIALTDWGLAQQFKSQKVSYIMGGKLTNRMIEDLNNAVNGKSKYKMTYYSGHALTLLEVMGTLGVPLDTAPGYASNLEMELYKDGDIYTVKLRYNGKYVKLPIMDKNNSCSLDALNKYMQSINEKFQKHHHHHH

>1PYAA

SELDAKLNKLGVDRIAISPYKQWTRGYMEPGNIGNGYVTGLKVDAGVRDKSDDDVLDGIVSYDRAETKNAYIGQINMTTAS

>2O3OA

GSHMATGKEYEVIKNDVEHDMKADHITYEGLNKEATEGYRITANQKSFSKEEIEALKDQKPLMDMPSDDHKVTSLKMKFANPIALSKKDIEDDAQALVSSKIQDGEKYKLWKVDKSKKEIIFFQTYEGHYIYQKTDNPSNMIGQVVLHLNGKNEVVSYDQTTLETFKQIQKESLITEMDAVELLYYQNQLKEYSTVKSCKFGYVAQYPLTSTQVLAPVWRITVEYEKKVNGEKKTVQEYFTVNALESTILDTDQ

>1G3KA

TTIVSVRRNGQVVVGGDGQVSLGNTVMKGNARKVRRLYNGKVLAGFAGGTADAFTLFELFERKLEMHQGHLLKSAVELAKDWRTDRALRKLEAMLIVADEKESLIITGIGDVVQPEEDQILAIGSGGNYALSAARALVENTELSAHEIVEKSLRIAGDICVFTNTNFTIEELPN

>3OR1A

MAKHPTPMLDELEKGPWPSFVSDIKQECDNRAKNPKGLDYQIPAECPDDLLGILELSFHEGETHWKHGGIVGVFGYGGGVIGRYCDQPEMFPGVAHFHTVRLAQPAAKYYTAEYLEAICDVWDLRGSGLTNMHGSTGDIVLLGTQTPQLEEIFFEMTHNLNTDLGGSGSNLRTPESCLGISRCEFACYDTQLMCYQLTQDYQDELHRPAFPYKFKFKFDGCPNGCVASMARSDFAVIGTWKDDIKIDQEAVKAYVGGEFKPNAGAHAGRDWGKFDIEAEVVGLCPTGCMTYESGTLSIDNKNCTRCMHCINTMPRALKIGDERGASILVGAKAPVLDGAQMGSLLIPFIAAEEPFDEVKEVIENIWEWWMEEGKNRERLGETMKRVGFQKLLEVTGTKAVPQHVSEPRHNPYIFFKEEEVPGGWSRDISDYRKRHMR

>2VK8A

MSEITLGKYLFERLKQVNVNTVFGLPGDFNLSLLDKIYEVEGMRWAGNANELNAAYAADGYARIKGMSCIITTFGVGELSALNGIAGSYAEHVGVLHVVGVPSISAQAKQLLLHHTLGNGDFTVFHRMSANISETTAMITDIATAPAEIDRCIRTTYVTQRPVYLGLPANLVDLNVPAKLLQTPIDMSLKPNDAESEKEVIDTILVLDKDAKNPVILADACCSRHDVKAETKKLIDLTQFPAFVTPMGKGSIDEQHPRYGGVYVGTLSKPEVKEAVESADLILSVGALLSDFNTGSFSYSYKTKNIVEFHSDHMKIRNATFPGVQMKFVLQKLLTTIADAAKGYKPVAVPARTPANAAVPASTPLKQEWMWNQLGNFLQEGDVVIAETGTSAFGINQTTFPNNTYGISQVLWGSIGFTTGATLGAAFAAEEIDPKKRVILFIGDGSLQLTVQEISTMIRWGLKPYLFVLNNDGYTIQKLIHGPKAQYNEIQGWDHLSLLPTFGAKDYETHRVATTGEWDKLTQDKSFNDNSKIRMIEVMLPVFDAPQNLVEQAKLTAATNAKQ

>3AH7A

MPLVTFLPHEKFCPEGLTVEVKPGTNILELAHDHHIEMESACGGVKACTTCHCIVRKGFDSLEEADELEEDMLDKAWGLEAQSRLGCQVFVADEDLTIEIPKYSLNHAAEAPH

>3R5TA

QQNVWPRTFQNADGSITTIPSQPKRILSTAVTVTGTLLAIDAPVIASAATTQSTFFEQWRKLAELRQVKKLWPAGSVDLESVYVEQPDLIVVSMIGADSARDQIPLLQAIAPTILVDYSDQTWQSLAQQLGLATGLEEQAERTIHNFEQWTKQVRDVLDLPKGRANIVSYHGPGVVNAVAKAQSAHAQLLQSVGVVLEEPDPAWQAGSIVHRDFLRIHYEHLTQLQAETTFLITMTDQQAQAFLHDPILKNLPSIQRKQVYGLGENSFRIDLFSAREIINSLLRRFAGEQAQSLVMPLEHHHHHH

>2DWKA

GSSGSSGMANERMNLMNMAKLSIKGLIESALNLGRTLDSDYAPLQQFFVVMEHCLKHGLKAKKTFLGQNKSFWGPLELVEKLVPEAAEITASVKDLPGLKTPVGRGRAWLRLALMQKKLSEYMKALINKKELLSEFYEVNALMMEEEGAIIAGLLVGLNVIDANFCMKGEDLDSQVGVID

>2RJ2A

GSHMFYFLSKRRRNLLRNPCGEEDLEGWSDVEHGGDGWKVEELPGDGNVEFTQDDSVKKYFASSFEWCRKAQVIDLQAEGYWEELLDTTQPAIVVKDWYSGRTDAGSLYELTVRLLSENEDVLAEFATGQVAVPEDGSWMEISHTFIDYGPGVRFVRFEHGGQDSVYWKGWFGARVTNSSVWVEP

>4IGBB

PTTENLYFQGAMALEEIKNGTDISTLDIRKFNLNINNVSVLSKSQSVDQFHLSNPHYEYLSGGAYPGEMENFTLKVDKSKKQDQVFENPLSLKFTNIGTVNGKQVDAYLNFNKVTLHYLNTAQAESEMNSAQKSTVEFFSISELWESNAFEIGNVPYVDANHDYIMNKAFWIDADVTAEIRYADGTETDLKLVMKPTDIDAIDANNLKETFYVKNYQNDVNLRLMNNANVLVQEEASDRTSWIATQITGGSYNENNVSGLALRSNSNSMNFGYSSTETCSAVFGLYIEKIDPRPVLEVDPAEIPAKDGQDVTYKATFKVPVPGKDILAAPSSIEMVQKFDERLDYKELKVESGGVTLQEGRDYTIEKTGQTVTVKMTPEYLKGNSSSDIIITYKTATNKKVEEKGSEKIDNTVTLHVDNLSAPSNQVSTALLYEK

>3TJ1A

MGSSHHHHHHSSGLVPRGSHMAMMAFENTSKRPPQDFVAPIDQKKRKVQFSDSTGLVTLQPEEIKDEVFSAAMYSRFVKSALDDLDKNDSTQIGIIANQVALPSKNPERINDKNLNILLDILSSNINRIESSRGTFLIQSIINFEKWWELPPHTLSKYIYFIKILCSSIPKWWQDVSMILVSCFILPIKQTVCHHDMLKYFLRMIPSSMGFIDTYLAKFFPNKNDTRRKLVNYTSNLLKLRGYCSELGFQIWSLLIEKIISIDVELQNELDELDDDVDDDDLEEVDLEDDDDLDDDSGDDDDENCGNSNEELRSGAADGSQSDSEDMDIIEGMDGTEEYNVELTQGIKELSTKLDSILTLVSTHVEEQVTPESLESGEGVGVFNTLTTLFKTHVLPTYYTRSIQYIMFHVSQQQLELMDSFLVTLIDISFAVNEAAEKKIKSLQYLGSYIARAKKLSRTQIIFVASYLTSWLNRYVIEREEEVDQRGGMERFKHFYAAFQALCYIFCFRHNIFRDTDGNWECELDKFFQRMVISKFNPLKFCNENVMLMFARIAQQESVAYCFSIIENNNNERLRGIIGKADSDKKENSAQANTTSSSWSLATRQQFIDLQSYFPYDPLFLKNYKILMKEYYIEWSEASGEYESDGSDD

>2BKFA

GAMEPQVTLNVTFKNEIQSFLVSDPENTTWADIEAMVKVSFDLNTIQIKYLDEENEEVSINSQGEYEEALKMAVKQGNQLQMQVHEG

>4G6VA

MGATDRTPPSNAILSNSNSDNNSTQGSQSGTVTKTPNPEATGSLSGKPTQIPPLSDEVTTRSLIRENQSAVTLANKGYDVVQNPEVLGPKNPDYTINGQVFDNYAPATGNVRNIATTISNKVSSGQASNIVVNLADSSASPAAIEAQINSYPIPGLGKVIVIDKLGNITIIKPKGN

>3H0UA

MSLTASYETIKARLDGTVLSATFNAPPMNLIGPEVVRDLVALLEELAHPTAPRVVIFDSADADFFFPHVDMTKVPEYTAEAAKAGGPGDASLGMLFRKLSQLPAVTIAKLRGRARGAGSEFLLACDMRFASRENAILGQPEVGIGAPPGAGAIQHLTRLLGRGRALEAVLTSSDFDADLAERYGWVNRAVPDAELDEFVAGIAARMSGFPRDALIAAKSAINAISLPAPAEVRADAALFQQLVRGEKVQQRTAELFKQGFQTRGATELDLGDALGHLKAVDEGHHHHHH

>3BEMA

MGSDKIHHHHHHMAEFTHLVNERRSASNFLSGHPITKEDLNEMFELVALAPSAFNLQHTKYVTVLDQDVKEKLKQAANGQYKVVSSSAVLLVLGDKQAYQQAADIYEGLKVLGILNKQEYDHMVQDTVSFYENRGEQFKRDEAIRNASLSAMMFMLSAAAAGWDTCPMIGFDAEAVKRILNIDDQFEVVMMITIGKEKTESRRPRGYRKPVNEFVEYM

>2RKQA

EVPIVTRAEWNAKPPNGAIDSMVTPLPRAVIAHTAGGACADDVTCSQHMRNLQNFQMSKQKFSDIGYHYLIGGNGKVYEGRSPSQRGAFAGPNNDGSLGIAFIGNFEERAPNKEALDAAKELLEQAVKQAQLVEGYKLLGHRQVSATKSPGEALYALIQQWPNWSEEML

>3FD3A

RVTLNIATNADSLGTWFLDAVSKFTGGSDYLVNIAVDDQDHTVEWLRGGRVLAAVTAHDKPVQGCRVTPLGVLRYHATASPDFMARHFADGVTPAALARAPGLTFNQKDRLQASWIRTALGEDVSYPTHWLPSTDGFVKASLAGMGWGLNPVQLVAEHLAAGRLVELMPGTPLDIPLYWQVNRLAAERLAGLTANMVGTARVVLMPVG

>1YNFA

MGSSHHHHHHGSNAWEVNFDGLVGLTHHYAGLSFGNEASTRHRFQVSNPRLAAKQGLLKMKALADAGFPQAVIPPHERPFIPVLRQLGFSGSDEQVLEKVARQAPHWLSSVSSASPMWVANAATIAPSADTLDGKVHLTVANLNNKFHRSLEAPVTESLLKAIFNDEEKFSVHSALPQVALLGDEGAANHNRLGGHYGEPGMQLFVYGREEGNDTRPSRYPARQTREASEAVARLNQVNPQQVIFAQQNPDVIDQGVFHNDVIAVSNRQVLFCHQQAFARQSQLLANLRARVNGFMAIEVPATQVSVSDTVSTYLFNSQLLSRDDGSMMLVLPQECREHAGVWGYLNELLAADNPISELKVFDLRESMANGGGPACLRLRVVLTEEERRAVNPAVMMNDTLFNALNDWVDRYYRDRLTAADLADPQLLREGREALDVLSQLLNLGSVYPFQREGGGNG

>1O70A

AAAADTTVTQFLQSFKENAENGALRKFYEVIMDNGGAVLDDINSLTEVTILAPSNEAWNSSNINNVLRDRNKMRQILNMHIIKDRLNVDKIRQKNANLIAQVPTVNNNTFLYFNVRGEGSDTVITVEGGGVNATVIQADVAQTNGYVHIIDHVLGVPYTTVLGKLESDPMMSDTYKMGKFSHFNDQLNNTQRRFTYFVPRDKGWQKTELDYPSAHKKLFMADFSYHSKSILERHLAISDKEYTMKDLVKFSQESGSVILPTFRDSLSIRVEEEAGRYVIIWNYKKINVYRPDVECTNGIIHVIDYPLLEEKDVVVAGGSAAAAA

>3PZDA

EFDTPTQQLIQDIKENCLNSDVVEQIYKRNPILRYTHHPLHSPLLPLPYGDINLNLLKDKGYTTLQDEAIKIFNSLQQLESMSDPIPIIQGILQTGHDLRPLRDELYCQLIKQTNKVPHPGSVGNLYSWQILTCLSCTFLPSRGILKYLKFHLKRIREQFPGSEMEKYALFTYESLKKTKCREFVPSRDEIEALIHRQEMTSTVYCHGGGSCKITINSHTTAGEVVEKLIRGLAMEDSRNMFALFEYNGHVDKAIESRTVVADVLAKFEKLAATSEVGDLPWKFYFKLYCFLDTDNVPKDSVEFAFMFEQAHEAVIHGHHPAPEENLQVLAALRLQYLQGDYTLHAAIPPLEEVYSLQRLKARISQSTKTQMLDMWIKEEVSSARASIIDKWRKFQGMNQEQAMAKYMALIKEWPGYGSTLFDVECKEGGFPQELWLGVSADAVSVYKRGEGRPLEVFQYEHILSFGAPLANTYKIVVDERELLFETSEVVDVAKLMKAYISMIVKKRYST

>4IAUA

MSTAKVTLVTSGGSSQDFTSEQTNITTDFARVRVTKGMWIFYQQANYNDASGGGSLWIKLDESSHLMDLPFTPRSFRPVKTFQVGATLYKHVNFGGKELDLPNSNPRIDIGGVSSALISQGQWRLYEQYDYAGPSTRRGPGVYVNAGALGVANDALKSMEREF

>3A2ZA

MSKGTTSQDAPFGTLLGYAPGGVAIYSSDYSSLDPQEYEDDAVFRSYIDDEYMGHKWQCVEFARRFLFLNYGVVFTDVGMAWEIFSLRFLREVVNDNILPLQAFPNGSPRAPVAGALLIWDKGGEFKDTGHVAIITQLHGNKVRIAEQNVIHSPLPQGQQWTRELEMVVENGCYTLKDTFDDTTILGWMIQTEDTEY

>3V7NA

GPGSMNYISTRGAGIGERHTFSDILLGGLAKDGGLYLPSEYPQVSADELARWRTLPYADLAFEILSKFCDDIAAADLRAITRRTYTADVYRHARRGGNAADITPLTTLGTENGAPVSLLELSNGPTLAFKDMAMQLLGNLFEYTLAKHGETLNILGATSGDTGSAAEYAMRGKEGVRVFMLSPHKKMSAFQTAQMYSLQDPNIFNLAVNGVFDDCQDIVKAVSNDHAFKAQQKIGTVNSINWARVVAQVVYYFKGYFAATRSNDERVSFTVPSGNFGNVCAGHIARMMGLPIEKLVVATNENDVLDEFFRTGAYRVRSAQDTYHTSSPSMDISKASNFERFVFDLLGRDPARVVQLFRDVEQKGGFDLAASGDFARVAEFGFVSGRSTHADRIATIRDVFERYRTMIDTHTADGLKVAREHLRPGVPMVVLETAQPIKFGESIREALGQEPSRPAAFDGLEALPQRFEVVDANAQQVKDFIAAHTGA

>1OSYA

XSATSLTFQLAYLVKKIDFDYTPNWGRGTPSSYIDNLTFPKVLTDKKYSYRVVVNGSDLGVESNFAVTPSGGQTINFLQYNKGYGVADTKTIQVFVVIPDTGNSEEYIIAEWKKT

>2VPAA

MSYYHHHHHHLESTSLYKKAGMSDFYDPRERDPSVSRRPQNRQSDEWIRELLLRGTIARVATLWQGEDGAAFPFITPLAYAYRPEQGDLVYHTNVVGRLRANAGQGHPATLEVSEIGQFLPSNSPLELSVQYRSVMVFGTARVLAGEDARAALTTLSERVFPGLKVGETTRPISEDDLKRTSVYSLSIDRWSGKENWAEQAIQEEDWPALGPEWLG

>4JX2A

GHTNNTNNKGLSASINNGVGSSSSNNTYVTPQAFWNLYFDFTGDETPGYPKGKINISQTLFQSEMKKNSSLAQQNEGQLILFINSTLYIYNSDRQLKLKQLMRTAPNSGFTEMTAISHIGPALMYLAKIKENGDASWKSQMENLLKDIQAVKVINAQTPNNWLEQVNAPAWKPHLTTIHNMIDYACSMAGNYMSDVLNEKLSFDMASLQNDFLNGNKTYPIPYNNVMIGTFMLTALQSMDQLHSKISQLKIDWPHAKVIIRFVAGSNVSAGVSKGSNWLVPFVQALSNNKLATDRIYITPYAAVKPSLGAQELTQADYNYYNNTVWGARHNRRIIANEVFTNITSIFLPDRPAIPGDYTYSKPPKIEDFLMRLKFSLAEPTEMLSNTVGFWMAGELAEKNWNYNKISIPGITTGFPEGISTYPNNNPVIQR

>3LYEA

GSHMAEDEPFSGAKKLRHLLENTDELIVCPGVYDGLSARTAMELGFKSLYMTGAGTTASRLGQPDLAIAQLHDMRDNADMIANLDPFGPPLIADMDTGYGGPIMVARTVEHYIRSGVAGAHLEDQILTKRCGHLSGKKVVSRDEYLVRIRAAVATKRRLRSDFVLIARTDALQSLGYEECIERLRAARDEGADVGLLEGFRSKEQAAAAVAALAPWPLLLNSVENGHSPLITVEEAKAMGFRIMIFSFATLAPAYAAIRETLVRLRDHGVVGTPDGITPVRLFEVCGLQDAMEVDNGAGGKAFSEGV

>4JJPA

SNAMSNYKIPTLTIAGSDSSGGAGIQADLKTFSAIGTYGMSVITAITAQNTKGVFAVEDLNKKIIKKQIEAVFEDIPPRAVKIGMVSSPEIILEIVENLKKYNPKYLVVDPVMISKSGYYLLKPEAKENLIKYLIPLAYIITPNIPEAEEITGIKIHNVDDMKRVGEEILQLGPKFVLMKGGHLDGEAVDILVGKNIFKVYKSERIDKKNTHGTGCTLSSAITSYLALGYEITEAVNLSKIYITEAIKRSFDIGHGVGPVHHFYKFE

>2ODAA

MPLPTFPALLFGLSGCLVDFGAQAATSDTPDDEHAQLTPGAQNALKALRDQGMPCAWIDELPEALSTPLAAPVNDWMIAAPRPTAGWPQPDACWMALMALNVSQLEGCVLISGDPRLLQSGLNAGLWTIGLASCGPLCGLSPSQWQALNNAEREQRRAQATLKLYSLGVHSVIDHLGELESCLADIALRRSKGEKP

>3MT5A

MGRKHIVVCGHITLESVSNFLKDFLHKDRDDVNVEIVFLHNISPNLELEALFKRHFTQVEFYQGSVLNPHDLARVKIESADACLILANKYCADPDAEDASNIMRVISIKNYHPKIRIITQMLQYHNKAHLLNIPSWNWKEGDDAICLAELKLGFIAQSCLAQGLSTMLANLFSMRSFIKIEEDTWQKYYLEGVSNEMYTEYLSSAFVGLSFPTVCELCFVKLKLLMIAIEYKSANRESRILINPGNHLKIQEGTLGFFIASDAKEVKRAFFYCKACHDDITDPKRIKKCGCKRLEDEQPSTLSPKKKQRNGGMRNSPNTSPKLMRHDPLLIPGNDQIDNMDSNVKKYDSTGMFHWCAPKEIEKVILTRSEAAMTVLSGHVVVCIFGDVSSALIGLRNLVMPLRASNFHYHELKHIVFVGSIEYLKREWETLHNFPKVSILPGTPLSRADLRAVNINLCDMCVILSANQNNIDDTSLQDKECILASLNIKSMQFDDSIGVLQANSQGFTPPGMDRSSPDNSPVHGMLRQPSITTGVNIPIITELVNDTNVQFLDQDDDDDPDTELYLTQPFACGTAFAVSVLDSLMSATYFNDNILTLIRTLVTGGATPELEALIAEENALRGGYSTPQTLANRDRCRVAQLALLDGPFADLGDGGCYGDLFCKALKTYNMLCFGIYRLRDAHLSTPSQCTKRYVITNPPYEFELVPTDLIFCLMQFDSNSLEVLFQ

>3ER7A

GMMNTTTLDRYFDLFDASRTDEKAFDDLISLFSDEITFVLNGQEQHGIDAWKQFVRMVFTANQDIKHMYAGWVPSETGDTMETRWAVCGKSADGSVFTQDGTDIARLNADGKIVYLANVPDDTAMFNQYND

>1V2BA

GKPKTDTDFQTYNGDGFKLQIPSKWNPNKEVEYPGQVLRFEDNFDATSNVIVAITPTDKKSITDFGSPEQFLSQVDYLLGRQAYSGKTDSEGGFESDAVAIANVLETSTAEVGGKQYYYLSILTRTADGNEGGKHQLVTATVNDGKLYICKAQAGDKRWFKGAKKFVENTATSFSLA

>3HL1A

GMPPVWTLPRLYQHFQGAIDLELWTIPYYLTVLYSIKDPTTVPYRLIQAAVYQEMLHAQLVSNIANAYGYSPTLSAPEYVGTAVPHIDFDLDTPNPTSIFTPYSAELGPLDLTRVNTMCLIEYPEWRTQREPDLADDVTDYGSIGEFYDALRVGMEQLRGHVRGNQKQMDEFGPFYQNSPPLTVTESGDAGFLQALTLVDIIVDQGEGQTQPVETIPTEFQNTADGFQDAWPHFQRFDFIRRMPNWPGVYTGVTDPPAGSPGAEAQARLIADFAGFLDILNGMFSGGGAPPAFGVQMAKLGGDILSCWKLGAVPRYS

>3QS2A

MAHHHHHHVDDDDKMADVTAQAVATWSATAKKDTTSKLVVTPLGSLAFQYAEGIKGFNSQKGLFDVAIEGDSTATAFKLTSRLITNTLTQLDTSGSTLNVGVDYNGTAVEKTGDTVMIDTANGVLGGNLSPLANGYNASNRTTAQDGFTFSIISGTTNGTTAVTDYSTLPEGIWSGDVSVQFDATWTS

>2PV2A

TELNLSHILIPLPENPTSDQVNEAESQARAIVDQARNGADFGKLAIAHSADQQALNGGQMGWGRIQELPGIFAQALSTAKKGDIVGPIRSGVGFHILKVNDLR

>2WVXA

KDWTQYVNPLMGSQSTFELSTGNTYPAIARPWGMNFWTPQTGKMGDGWQYTYTANKIRGFKQTHQPSPWINDYGQFSIMPIVGQPVFDEEKRASWFAHKGEVATPYYYKVYLAEHDIVTEMTPTERAVLFRFTFPENDHSYVVVDAFDKGSYIKIIPEENKIIGYTTRNSGGVPENFKNYFIIEFDKPFTYKATVENGNLQENVAEQTTDHAGAIIGFKTRKGEQVNARIASSFISFEQAAANMNELGKDNIEQLAQKGKDAWNQVLGKIEVEGGNLDQYRTFYSCLYRSLLFPRKFYELDANGQPIHYSPYNGQVLPGYMFTDTGFWDTFRCLFPLLNLMYPSVNKEMQEGLINTYLESGFFPEWASPGHRGCMVGNNSASILVDAYMKGVKVDDIKTLYEGLIHGTENVHPEVSSTGRLGYEYYNKLGYVPYDVKINENAARTLEYAYDDWCIYRLAKELKRPKKEISLFAKRAMNYKNLFDKESKLMRGRNEDGTFQSPFSPLKWGDAFTEGNSWHYTWSVFHDPQGLIDLMGGKEMFVTMMDSVFAVPPIFDDSYYGQVIHEIREMTVMNMGNYAHGNQPIQHMIYLYDYAGQPWKAQYWLRQVMDRMYTPGPDGYCGDEDNGQTSAWYVFSALGFYPVCPGTDEYVMGTPLFKKATLHFENGNSLVIDAPNNSTENFYIDSMSFNGADHTKNYLRHEDLFKGGTIKVDMSNRPNLNRGTKEEDMPYSFSKELEHHHHHH

>3D5KA

CSLIPDYQRPEAPVAAAYPQGQAYGQNTGAAAVPAADIGWREFFRDPQLQQLIGVALENNRDLRVAALNVEAFRAQYRIQRADLFPRIGVDGSGTRQRLPGDLSTTGSPAISSQYGVTLGTTAWELDLFGRLRSLRDQALEQYLATEQAQRSAQTTLVASVATAYLTLKADQAQLQLTKDTLGTYQKSFDLTQRSYDVGVASALDLRQAQTAVEGARATLAQYTRLVAQDQNALVLLLGSGIPANLPQGLGLDQTLLTEVPAGLPSDLLQRRPDILEAEHQLMAANASIGAARAAFFPSISLTANAGTMSRQLSGLFDAGSGSWLFQPSINLPIFTAGSLRASLDYAKIQKDINVAQYEKAIQTAFQEVADGLAARGTFTEQLQAQRDLVKASDEYYQLADKRYRTGVDNYLTLLDAQRSLFTAQQQLITDRLNQLTSEVNLYKALGGGWNQQTVTQQQTAKKEDPQAHHHHHH

>2GDMA

GALTESQAALVKSSWEEFNANIPKHTHRFFILVLEIAPAAKDLFSFLKGTSEVPQNNPELQAHAGKVFKLVYEAAIQLEVTGVVVTDATLKNLGSVHVSKGVADAHFPVVKEAILKTIKEVVGAKWSEELNSAWTIAYDELAIVIKKEMDDAA

>2AJ7A

MGSDKIHHHHHHMKVIETKYSGKLEVAEDRLIAFDQGIPAFEDEKEFVLLPFAAGTPYYTLQSTKTVDLAFIIVNPFSFFPEYRVKLPEATIAQLNITNENDVAIFSLLTVKEPFSETTVNLQAPIVINANKQMGKQLVLGDTAYNRKQPLFQKELVLAKEAK

>1LKTA

ANVVVSNPRPIFTESRSFKAVANGKIYIGQIDTDPVNPANQIPVYIENEDGSHVQITQPLIINAAGKIVYNGQLVKIVTVQGHSMAIYDANGSQVDYIANVLKY

>1L6RA

GSHMIRLAAIDVDGNLTDRDRLISTKAIESIRSAEKKGLTVSLLSGNVIPVVYALKIFLGINGPVFGENGGIMFDNDGSIKKFFSNEGTNKFLEEMSKRTSMRSILTNRWREASTGFDIDPEDVDYVRKEAESRGFVIFYSGYSWHLMNRGEDKAFAVNKLKEMYSLEYDEILVIGDSNNDMPMFQLPVRKACPANATDNIKAVSDFVSDYSYGEEIGQIFKHFELM

>2JBYA

MGSHHHHHHSQDPMMSRLKTAVYDYLNDVDITECTEMDLLCQLSNCCDFINETYAKNYDTLYDIMERDILSYNIVNIKNTLTFALRDASPSVKLATLTLLASVIKKLNKIQHTDAAMFSEVIDGIVAEEQQVIGFIQKKCKYNTT

>4KSNA

GNSDGQLDTHLADLYLLKYDTGLGVYESFICKYLEDSNDYIASHPQKLSLDEMPRPLESETVSLRQLIVSVLPSRPSI

>3HG9A

MSLTSSAELAEVDTLARSLLLYRSRLAEYAHANPGFSGSPADSALGLPAWFRKPVRLQGYIAAGTSYAFIASPPAGLAAAVDTGTESDLVGVRRNGQLVTRRLGATAIALPAPIPEGAVVAVKEGHHHHHH

>3BF7A

MKLNIRAQTAQNQHNNSPIVLVHGLFGSLDNLGVLARDLVNDHNIIQVDVRNHGLSPREPVMNYPAMAQDLVDTLDALQIDKATFIGHSMGGKAVMALTALAPDRIDKLVAIDIAPVDYHVRRHDEIFAAINAVSESDAQTRQQAAAIMRQHLNEEGVIQFLLKSFVDGEWRFNVPVLWDQYPHIVGWEKIPAWDHPALFIPGGNSPYVSEQYRDDLLAQFPQARAHVIAGAGHWVHAEKPDAVLRAIRRYLNDH

>3HHSA

ADIFDSFELLYDRPGEPMINTKGEDKVLFELTEQFLTPEYANNGLELNNRFGDEEEVSRKIILKNLDKIPEFPKAKQLPNDADFSLFLPSHQEMANEVIDVLMSVTENQLQELLSTCVYARINLNPQLFNYCYTVAIMHRRDTGKVRVQNYAEIFPAKFLDSQVFTQAREAAAVIPKTIPRTPIIIPRDYTATDLEEEHRLAYWREDLGINLHHWHWHLVYPFSASDEKIVAKDRRGELFFYMHQQIIARYNCERLCNSLKRVKKFSDWREPIPEAYYPKLDSLTSARGWPPRQAGMRWQDLKRPVDGLNVTIDDMERYRRNIEEAIATGNVILPDKSTKKLDIDMLGNMMEASVLSPNRDLYGSIHNNMHSFSAYMHDPEHRYLESFGVIADEATTMRDPFFYRVHAWVDDIFQSFKEAPHNVRPYSRSQLENPGVQVTSVAVESAGGQQNVLNTFWMQSDVNLSKGLDFSDRGPVYARFTHLNHRPFRYVIKANNTASARRTTVRIFIAPKTDERNLPWALSDQRKMFIEMDRFVVPLSAGENTITRQSTESSLTIPFEQTFRDLSIQGSDPRRSELAAFNYCGCGWPQHMLVPKGTVGGVAYQLFVMLSNYELDKIEQPDGRELSCVEASMFCGLKDKKYPDARPMGYPFDRPSNSATNIEDFSAMSNMGLQDIVIKLSDVTEPNPRNPPA

>4J32A

MGHHHHHHLYFQGMNRPSFNEAWLAFRKVNHSVADVGSIIGGNVGKNITGGYFQNACPIRMSYVLNATGFPIARNSPYAKVSGADNKFYIYRVNDMIDYLTHTMGKPDLIVNNPKQSDFIGKKGIIVVKGHGWSNARGHVTLWNGSICSDQCHLLNDPDNGPFVPEVGTLWILP

>2AUWA

GHMNEYFFPKLTAVEALAPYRLRTTWSTGEVLEVDVGDILRKIPDLAPILDPEAFARVHIAEWEGSVEWFDTEFGRDNVYAWAKEQAGEVSHEMFGDWMHRNNLSLTTAAEALGISRRMVSYYRTAHKIIPRTIWLACLGWEATRPETKTLPRTLPAAYAKGVSASLSGS

>2Z0BA

GSSGSSGPSQVAFEIRGTLLPGEVFAICGSCDALGNWNPQNAVALLPENDTGESMLWKATIVLSRGVSVQYRYFKGYFLEPKTIGGPCQVIVHKWETHLQPRSITPLESEIIIDDGQFGIHNGVESGPSSG

>1K4ZA

MPPRKELVGNKWFIENYENETESLVIDANKDESIFIGKCSQVLVQIKGKVNAISLSETESCSVVLDSSISGMDVIKSNKFGIQVNHSLPQISIDKSDGGNIYLSKESLNTEIYTSCSTAINVNLPIGEDDDYVEFPIPEQMKHSFADGKFKSAVFEHAG

>2QIPA

SNAMQSDHKEKIAILVDVQNVYYTCREAYRSNFDYNQFWYVATQEKEVVSAKAYAIASNDPKQRQFHHILRGVGFEVMLKPYIQRRDGSAKGDWDVGITLDAIEIAPDVDRVILVSGDGDFSLLVERIQQRYNKKVTVYGVPRLTSQTLIDCADNFVAIDDDFLL

>2FVVA

MHHHHHHSSGVDLGTENLYFQSMMKLKSNQTRTYDGDGYKKRAACLCFRSESEEEVLLVSSSRHPDRWIVPGGGMEPEEEPSVAAVREVCEEAGVKGTLGRLVGIFENQERKHRTYVYVLIVTEVLEDWEDSVNIGRKREWFKIEDAIKVLQYHKPVQASYFETLRQGYSANNGTPVVATTYSVSAQSSMSGIR

>3RWXA

GNNDDDGTNKAQEIAGKYEGYSIGNCAMFTDYVMGEKSVATIVPNEDGTINVTYDSGSGEFKLNNIKVTSKTFEGSGQVELSMNDKPAGAKDFTLTGSIDEQQKLTLKVNVPSVMGGLTIEFIQGTLPISYHVSGTYNKEANLSVSVGSTTYPDITDCKVSIKRSSDDTVELTLKGLSNLNSSQTGRAMNLGDFTVTDVKVTSTDNSIFKIEGSINTTDTNNTPITGTLSGTVSNSETNITFTFKPGAMPIDITAMFKGKK

>2QGYA

MSLNNQDISIGKLSRLKIWITDNHLSDDQWSNTKKFIIIKITTEDGIEGWGEAFSINFREKGIAIIIKELFREISNIPNLSIKSFYNKISLLSDGHRGLDFSSATSAIEIALWDISGKLKNLPLNSLLTKSPKPNVPIYATCWSDLKKDTNDYLRQIEKFYGKKYGGIKIYPMLDSLSISIQFVEKVREIVGDELPLMLDLAVPEDLDQTKSFLKEVSSFNPYWIEEPVDGENISLLTEIKNTFNMKVVTGEKQSGLVHFRELISRNAADIFNPDISGMGGLIDIIEISNEASNNGIFISPHCWNSMSVSASAMLHVCSSIPNSEKAEIFPDYINFSKKFCELPFDIIDNKAHINKSAGLGIVIHEDILSELSIYSLDEKSNDEGHHHHHH

>2ERVA

ADVSAAVGATGQSGMTYRLGLSWDWDKSWWQTSTGRLTGYWDAGYTYWEGGDEGAGKHSLSFAPVFVYEFAGDSIKPFIEAGIGVAAFSGTRVGDQNLGSSLNFEDRIGAGLKFANGQSVGVRAIHYSNAGLKQPNDGIESYSLFYKIPI

>1ITHA

GLTAAQIKAIQDHWFLNIKGCLQAAADSIFFKYLTAYPGDLAFFHKFSSVPLYGLRSNPAYKAQTLTVINYLDKVVDALGGNAGALMKAKVPSHDAMGITPKHFGQLLKLVGGVFQEEFSADPTTVAAWGDAAGVLVAAMK

>1FTRA

MEINGVEIEDTFAEAFEAKMARVLITAASHKWAMIAVKEATGFGTSVIMCPAEAGIDCGYVPPEETPDGRPGVTIMIGHNDEDELKEQLLDRIGQCVMTAPTASAFDAMPEAEKEDEDRVGYKLSFFGDGYQEEDELDGRKVWKIPVVEGEFIVEDSFGITTGVAGGNFYIMAESQPAGLQAAEAAVDAIKGVEGAYAPFPGGIVASASKVGSKQYDFLPASTNDAYCPTVEDNELPEGVKCVYEIVINGLNEEAVKEAMRVGIEAACQQPGVVKISAGNFGGKLGQYEIHLHDLF

>1WLGA

GLDVAISQNGFFRLVDSNGSVFYSRNGQFKLDENRNLVNMQGMQLTGYPATGTPPTIQQGANPAPITIPNTLMAAKSTTTASMQINLNSTDPVPSKTPFSVSDADSYNKKGTVTVYDSQGNAHDMNVYFVKTKDNEWAVYTHDSSDPAATAPTTASTTLKFNENGILESGGTVNITTGTINGATAATFSLSFLNSMQQNTGANNIVATNQNGYKPGDLVSYQINNDGTVVGNYSNEQEQVLGQIVLANFANNEGLASQGDNVWAATQASGVALLGTAGSGNFGKLTNGALEASNVDLSK

>2Y26A

GLAGRGVIYIPKDCQANRYLGTLNIRDMISDFKGVQYEKWITAGLVMPTFKIVIRLPANAFTGLTWVMSFDAYNRITSRITASADPVYTLSVPHWLIHHKLGTFSCEIDYGELCGHAMWFKSTTFESPRLHFTCLTGNNKELAADWQAVVELYAELEEATSFLGKPTLVFDPGVFNGKFQFLTCPPIFFDLTAVTALRSAGLTLGQVPMVGTTKVYNLNSTLVSCVLGMGGTVRGRVHICAPIFYSIVLWVVSEWNGTTMDWNELFKYPGVYVEEDGSFEVKIRSPYHRTPARLLADQSQRDMSSLNFYAIAGPIAPSGETAQLPIVVQIDEIVRPDLSLPSFEDDYFVWVDFSEFTLDKEEIEIGSRFFDFTSNTCRVSMGENPFAAMIACHGLHSGVLDLKLQWSLNTEFGKSSGSVTITKLVGDKAMGLDGPSHVFAIQKLEGTTELLVGNFAGANPNTRFSLYSRWMAIKLDQAKSIKVLRVLCKPRPGFSFYGRTSFPV

>3SC7X

MLNPKVAYMVWMTCLGLTLPSQAQSNDYRPSYHFTPDQYWMNEPNGLIKIGSTWHLFFQHNPTANVWGNICWGHATSTDLMHWAHKPTAIADENGVEAFTGTAYYDPNNTSGLGDSANPPYLAWFTGYTTSSQTQDQRLAFSVDNGATWTKFQGNPIISTSQEAPHDITGGLESRDPKVFFHRQSGNWIMVLAHGGQDKLSFWTSADTINWTWQSDLKSTSINGLSSDITGWEVPDMFELPVEGTEETTWVVMMTPAEGSPAGGNGVLAITGSFDGKSFTADPVDASTMWLDNGRDFDGALSWVNVPASDGRRIIAAVMNSYGSNPPTTTWKGMLSFPRTLSLKKVGTQQHFVQQPITELDTISTSLQILANQTITPGQTLLSSIRGTALDVRVAFYPDAGSVLSLAVRKGASEQTVIKYTQSDATLSVDRTESGDISYDPAAGGVHTAKLEEDGTGLVSIRVLVDTCSVEVFGGQGEAVISDLIFPSDSSDGLALEVTGGNAVLQSVDVRSVSLE

>3DSMA

ASGLFITNEGNFQYSNATLSYYDPATCEVENEVFYRANGFKLGDVAQSMVIRDGIGWIVVNNSHVIFAIDINTFKEVGRITGFTSPRYIHFLSDEKAYVTQIWDYRIFIINPKTYEITGYIECPDMDMESGSTEQMVQYGKYVYVNCWSYQNRILKIDTETDKVVDELTIGIQPTSLVMDKYNKMWTITDGGYEGSPYGYEAPSLYRIDAETFTVEKQFKFKLGDWPSEVQLNGTRDTLYWINNDIWRMPVEADRVPVRPFLEFRDTKYYGLTVNPNNGEVYVADAIDYQQQGIVYRYSPQGKLIDEFYVGIIPGAFCWKLEHHHHHH

>3D1BA

GSHMNPLASLTTDKNDLYINWLKSLSFFQTNSSCAEALVKVIPHYHNKLIDFSQVLQLVFSASEKFPIQENQPLPEQLMFLSNLEKQTPFAKAVGSSIYKLVTGKNLSLDFASQILKEASILEH

>4MT2A

XMDPNCSCATDGSCSCAGSCKCKQCKCTSCKKSCCSCCPVGCAKCSQGCICKEASDKCSCCA

>1JNDA

ASNLVCYYDSSSYTREGLGKLLNPDLEIALQFCSHLVYGYAGLRGENLQAYSMNENLDIYKHQFSEVTSLKRKYPHLKVLLSVGGDHDIDPDHPNKYIDLLEGEKVRQIGFIRSAYELVKTYGFDGLDLAYQFPKNKPRKVHGDLGLAWKSIKKLFTGDFIVDPHAALHKEQFTALVRDVKDSLRADGFLLSLTVLPNVNSTWYFDIPALNGLVDFVNLATFDFLTPARNPEEADYSAPIYHPDGSKDRLAHLNADFQVEYWLSQGFPSNKINLGVATYGNAWKLTKDSGLEGVPVVPETSGPAPEGFQSQKPGLLSYAEICGKLSNPQNQFLKGNESPLRRVSDPTKRFGGIAYRPVDGQITEGIWVSYDDPDSASNKAAYARVKNLGGVALFDLSYDDFRGQCSGDKYPILRAIKYRL

>2YH6A

GDTASLLVENGRGNTLWPQVVSVLQAKNYTITQRDDAGQTLTTDWVQWNRLDEDEQYRGRYQISVKPQGYQQAVTVKLLNLEQAGKPVADAASMQRYSTEMMNVISAGLDKS

>3SDBA

SMNFYSAYQHGFVRVAACTHHTTIGDPAANAASVLDMARACHDDGAALAVFPELTLSGYSIEDVLLQDSLLDAVEDALLDLVTESADLLPVLVVGAPLRHRHRIYNTAVVIHRGAVLGVVPKSYLPTYREFYERRQMAPGDGERGTIRIGGADVAFGTDLLFAASDLPGFVLHVEIAEDMFVPMPPSAEAALAGATVLANLSGSPITIGRAEDRRLLARSASARCLAAYVYAAAGEGESTTDLAWDGQTMIWENGALLAESERFPKGVRRSVADVDTELLRSERLRMGTFDDNRRHHRELTESFRRIDFALDPPAGDIGLLREVERFPFVPADPQRLQQDCYEAYNIQVSGLEQRLRALDYPKVVIGVSGGLDSTHALIVATHAMDREGRPRSDILAFALPGFATGEHTKNNAIKLARALGVTFSEIDIGDTARLMLHTIGHPYSVGEKVYDVTFENVQAGLRTDYLFRIANQRGGIVLGTGDLSELALGWSTYGVGDQMSHYNVNAGVPKTLIQHLIRWVISAGEFGEKVGEVLQSVLDTEITPELIPTGEEELQSSEAKVGPFALQDFSLFQVLRYGFRPSKIAFLAWHAWNDAERGNWPPGFPKSERPSYSLAEIRHWLQIFVQRFYSFSQFKRSALPNGPKVSHGGALSPRGDWRAPSDMSARIWLDQIDREVPKG

>2QQ4A

MSVLDELYREILLDHYQSPRNFGVLPQATKQAGGMNPSCGDQVEVMVLLEGDTIADIRFQGQGCAISTASASLMTEAVKGKKVAEALELSRKFQAMVVEGAPPDPTLGDLLALQGVAKLPARVKCATLAWHALEEALR

>2XD7A

DGPGDGFTILSSKSLVLGQKLSLTQSDISHIGSMRVEGIVHPTTAEIDLKEDIGKALEKAGGKEFLETVKELRKSQGPLEVAEAAVSQSSGLAAKFVIHCHIPQWGSDKCEEQLEETIKNCLSAAEDKKLKSVAFPPFPSGRNCFPKQTAAQVTLKAISAHFDDSSASSLKNVYFLLFDSESIGIYVQEMAKL

>1IZMA

GSMLISHSDMNQQLKSAGIGFNATELHGFLSGLLCGGLKDQSWLPLLYQFSNDNHAYPTGLVQPVTELYEQISQTLSDVEGFTFELGLTEDENVFTQADSLSDWANQFLLGIGLAQPELAKEKGEIGEAVDDLQDICQLGYDEDDNEEELAEALEEIIEYVRTIAMLFYSHFNEGEIESKPVLH

>1NQJA

GGPGNEKLKEKENNDSSDKATVIPNFNTTMQGSLLGDDSRDYYSFEVKEEGEVNIELDKKDEFGVTWTLHPESNINDRITYGQVDGNKVSNKVKLRPGKYYLLVYKYSGSGNYELRVNK

>2MPRA

VDFHGYARSGIGWTGSGGEQQCFQATGAQSKYRLGNECETYAELKLGQEVWKEGDKSFYFDTNVAYSVNQQNDWESTDPAFREANVQGKNLIEWLPGSTIWAGKRFYQRHDVHMIDFYYWDISGPGAGIENIDLGFGKLSLAATRSTEAGGSYTFSSQNIYDEVKDTANDVFDVRLAGLQTNPDGVLELGVDYGRANTTDGYKLADGASKDGWMFTAEHTQSMLKGYNKFVVQYATDAMTTQGKGQARGSDGSSSFTEELSDGTKINYANKVINNNGNMWRILDHGAISLGDKWDLMYVGMYQNIDWDNNLGTEWWTVGVRPMYKWTPIMSTLLEVGYDNVKSQQTGDRNNQYKITLAQQWQAGDSIWSRPAIRIFATYAKWDEKWGYIKDGDNISRYAAATNSGISTNSRGDSDEWTFGAQMEIWW

>2X0QA

MSRTTPPHPAEIVAHLQPEIWNKVNRLLVRKAISEYAHEWLLEPQRLGPGETPGFERFRLTLADGAQYDFDAQVMAMRHWRIPPESIVKTVAGVPAPLDALQFVIEIRDKLGLPVDRLPIYMDEITSTLHGSAYKHGRTTLGAAALARADYQTIETSMIEGHPSFVANNGRLGFDAEDYHGYAPEAATPVRLMWLAVHKDNAHFSCLSDMDYDSLMSEELGESAVTDFAARLREQGLHPADYYFMPAHPWQWFNKLSLAFAPYVAQRKIVCLGYGEEQYLAQQSIRTFFNISRPGKRYVKTSLSILNMGFMRGLSPYYMAGTPAINEYIHDLISADPWLRANGFRILREVASMGFRNYYYEAAIDTDTPYKKMFSALWRENPLTLIAPGQNLMTMAALLHVDPQGRALLPELIQASGLDAGTWLERYVDAYLTPLIHCFYAHDLVFMPHGENVILVIQDGVPVRAFMKDIAEESSILNPQVRLPQAAQRLAADVPEAYKLLTIFVDVFEGYFRHLTQILVETELMPEHDFWRLVAGRIAAYQQAHPQRLDKYRRYDLFAPDMIHSCLNRLQLANNLQMVNLADPIGSFQMAPNLPNPIACFRPSWLGSGEALQTLTAA

>3H6JA

MNTYFDIPHRLVGKALYESYYDHFGQMDILSDGSLYLIYRRATEHVGGSDGRVVFSKLEGGIWSAPTIVAQAGGQDFRDVAGGTMPSGRIVAASTVYETGEVKVYVSDDSGVTWVHKFTLARGGADYNFAHGKSFQVGARYVIPLYAATGVNYELKWLESSDGGETWGEGSTIYSGNTPYNETSYLPVGDGVILAVARVGSGAGGALRQFISLDDGGTWTDQGNVTAQNGDSTDILVAPSLSYIYSEGGTPHVVLLYTNRTTHFCYYRTILLARAVAGSSGWTERVPAYSAPAASGYTSQVVLGGRRILGNLFRETSSTTSGAYQFEVYLGGVPDFESDWFSVSSNSLYTLSHGLQRSPRRVVVEFARSSSPSTWNIVMPSYFNDGGHKGSGAQVEVGSLNIRLGTGAAVWGTGYFGGIDNSATTRLATGYYRVRAWI

>3D33A

GANTTETPVKDVELDGRWDDPIRSAATNCPITVFTDGYLLTLKNASPDRDMTIRITDMAKGGVVYENDIPEVQSAYITISIANFPAEEYKLEITGTPSGHLTGYFTKE

>3IBYA

MTHALLIGNPNCGKTTLFNALTNANQRVGNWPGVTVEKKTGEFLLGEHLIEITDLPGVYSLVANAEGISQDEQIAAQSVIDLEYDCIINVIDACHLERHLYLTSQLFELGKPVVVALNMMDIAEHRGISIDTEKLESLLGCSVIPIQAHKNIGIPALQQSLLHCSQKIKPLKLSLSVAAQQILNDLENQLISKGYKNSFAYYFSRRLAEGDTLIGEKAFTESLLIKLQETEQNLDVLLADARYQKIHEIVTLVQKK

>3FHHA

TETMTVTATGNARSSFEAPMMVSVIDTSAPENQTATSATDLLRHVPGITLDGTGRTNGQDINMRGYDHRGVLVLVDGIRQGTDTGHLNGTFLDPALIKRVEIVRGPSALLYGSGALGGVISYDTVDAKDLLQEGQSSGFRVFGTGGTGDHSLGLGASAFGRTENLDGIVAWSSRDRGDLRQSNGETAPNDESINNMLAKGTWQIDSAQSLSGLVRYYNNDAREPKNPQTVEASESSNPMVDRSTIQRDAQLSYKLAPQGNDWLNADAKIYWSEVRINAQNTGSSGEYREQITKGARLENRSTLFADSFASHLLTYGGEYYRQEQGSHHHHHHHPGGATTGFPQAKIDFSSGWLQDEITLRDLPITLLGGTRYDSYRGSSDGYKDVDADKWSSRAGMTINPTNWLMLFGSYAQAFRAPTMGEMYNDSKHFSIGRFYTNYWVPNPNLRPETNETQEYGFGLRFDDLMLSNDALEFKASYFDTKAKDYISTTVDFAAATTMSYNVPNAKIWGWDVMTKYTTDLFSLDVAYNRTRGKDTDTGEYISSINPDTVTSTLNIPIAHSGFSVGWVGTFADRSTHISSSYSKQPGYGVNDFYVSYQGQQALKGMTTTLVLGNAFDKEYWSPQGIPQDGRNGKIFVSYQW

>2I71A

MGSSHHHHHHSSGRENLYFQGMASIVFSTIGNPKGYQKVTYEIDGEKFESNVSVLALRDLLKVDKTVVILGISVADVYNCKYADYRSCKECIIQNSKNDLGISESYVVAPNVYQKFKGKPDHYFTYIYYHSLRILEKEGINEVFIDTTHGINYMGVLAKEAIQLAVSAYAAKSEKEVKVSLYNSDPVGKDVSDTVKLHEIEAIKISPLSGLKYVTYQILNKDKNFFNKIFSDSVNAIPRFATALDNGLFIYLSEKDSSLHLKRLEDDLSKDPLLTPSENEINVVYKDMKYALSHALFYVISRFSGNVDLDTLRHYAETYADKVTRAIIENEVDKIEKYQMGSERKLLGEYMKVEGKGFDKRILYAHGGLPYAGTYVYKEKDKVYVTYGDKIDEIERQIGS

>3BRKX

MSEKRVQPLARDAMAYVLAGGRGSRLKELTDRRAKPAVYFGGKARIIDFALSNALNSGIRRIGVATQYKAHSLIRHLQRGWDFFRPERNESFDILPASQRVSETQWYEGTADAVYQNIDIIEPYAPEYMVILAGDHIYKMDYEYMLQQHVDSGADVTIGCLEVPRMEATGFGVMHVNEKDEIIDFIEKPADPPGIPGNEGFALASMGIYVFHTKFLMEAVRRDAADPTSSRDFGKDIIPYIVEHGKAVAHRFADSCVRSDFEHEPYWRDVGTIDAYWQANIDLTDVVPDLDIYDKSWPIWTYAEITPPAKFVHDDEDRRGSAVSSVVSGDCIISGAALNRSLLFTGVRANSYSRLENAVVLPSVKIGRHAQLSNVVIDHGVVIPEGLIVGEDPELDAKRFRRTESGICLITQSMIDKLDL

>1VI1A

MSLRIAVDAMGGDHAPKAVIDGVIKGIEAFDDLHITLVGDKTTIESHLTTTSDRITVLHADEVIEPTDEPVRAVRRKKNSSMVLMAQEVAENRADACISAGNTGALMTAGLFIVGRIKGIDRPALAPTLPTVSGDGFLLLDVGANVDAKPEHLVQYAIMGSVYSQQVRGVTSPRVGLLNVGTEDKKGNELTKQTFQILKETANINFIGNVEARDLLDDVADVVVTDGFTGNVTLKTLEGSALSIFKMMRDVMTSTLTSKLAAAVLKPKLKEMKMKMEYSNYGGASLFGLKAPVIKAHGSSDSNAVFRAIRQAREMVSQNVAALIQEEVKEEKTDEEGGSHHHHHH

>4J05A

GPMLADLDHFGKNYKHDEEAQRNQKPWMLTWPQIKLVLLAGVGFFLDAYDLFIINQVAPMLAQVYFPKTGLPAQRQDLMKAAANIGCVVGQVMFGVLGDSFGRKFVYGKELILIIVATIFQMSAPSHWDGNRVLTWITICRVFLGIGIGGDYPMSATVVSDRANIHRRGTLLCFIFANQGWGSFVGSLVTIVTISGFKHRLKSGHTHDVDKAWRILIGLSLIPAFGTLYQRLTLPESRKFELTRDAASSSTVAIDKKDHDATHEVKDAPESEKSSPKVTPADAIDDDRHGVIASKKAHWQEFVAYFSTWNHFRNLLGSMLGWFLVDIAFYGINLNQSVVLAQIGFAGKTGDVYDKLFQLATGNIIVTALGFLPGYYFTLFLIDIVGRKKLQFMGFIMSGLFLAILAGEIDHIGKGPLLACFTFMQFFFNFGANTTTFIVAAELFPTRIRASAHGISAAAGKCGAILSSLVFNQLKAKIGTSAVLWIFFSTCILGFISTFLIDETMGVDPDEKDLEERRARGEIPGGLVPR

>1QTFA

KEYSAEEIRKLKQKFEVPPTDKELYTHITDNARSPYNSVGTVFVKGSTLATGVLIGKNTIVTNYHVAREAAKNPSNIIFTPAQNRDAEKNEFPTPYGKFEAEEIKESPYGQGLDLAIIKLKPNEKGESAGDLIQPANIPDHIDIAKGDKYSLLGYPYNYSAYSLYQSQIEMFNDSQYFGYTEVGNSGSGIFNLKGELIGIHSGKGGQHNLPIGVFFNRKISSLYSVDNTFGDTLGNDLKKRAKLDK

>3LMBA

MNASLTPDQVSKKLKQFFSDHLPISQFMGLEIESYDGDTLILTAPLEPNINDKQTAFGGSLYNAAVMACWGMVYLKTQEENIACNQVVTEGNMKYIAPVYGRIRAICHAPDEEELANFFDHFERKGKARISLEAAIYNDACVMKIEPETKPSVKFNGQYAILKNQ

>4I4OA

VNFPNIPAEGVQFRLRARDTGYVIYSRTENPPLVWQYNGPPYDDQLFTLIYGTGPRKNLYAIKSVPNGRVLFSRTSASPYVGNIAGDGTYNDNWFQFIQDDNDPNSFRIYNLASDTVLYSRTTADPKFGNFTGAKYDDQLWHFELV

>1N62A

MAKAHIELTINGHPVEALVEPRTLLIHFIREQQNLTGAHIGCDTSHCGACTVDLDGMSVKSCTMFAVQANGASITTIEGMAAPDGTLSALQEGFRMMHGLQCGYCTPGMIMRSHRLLQENPSPTEAEIRFGIGGNLCRCTGYQNIVKAIQYAAAKINGVPFEEAAE

>4FWWA

VKYVVPSFSAGGLVQAMVTYEGDRNESAVFVAIRNRLHVLGPDLKSVQSLATGPAGDPGCQTCAACGPGPHGPPGDTDTKVLVLDPALPALVSCGSSLQGRCFLHDLEPQGTAVHLAAPACLFSAHHNRPDDCPDCVASPLGTRVTVVEQGQASYFYVASSLDAAVAASFSPRSVSIRRLKADASGFAPGFVALSVLPKHLVSYSIEYVHSFHTGAFVYFLTVQPASVTDDPSALHTRLARLSATEPELGDYRELVLDCRFAPKLVPRGSPEGGQPYPVLQVAHSAPVGAQLATELSIAEGQEVLFGVFVTGKDGGPGVGPNSVVCAFPIDLLDTLIDEGVERCCESPVHPGLRRGLDFFQSPSFCPNPPGLEALSPNTSCRHFPLLVSSSFSRVDLFNGLLGPVQVTALYVTRLDNVTVAHMGTMDGRILQVELVRSLNYLLYVSNFSLGDSGQPVQRDVSRLGDHLLFASGDQVFQVPIQGPGCRHFLTCGRCLRAWHFMGCGWCGNMCGQQKECPGSWQQDHCP

>3B47A

NAIMDLQTRNTRGLSTLVVRDIGELMMAGDMAVIERYVADVRGKGAVLDLRIYDAAGRPAGKKQDAPDGEVQAALTSGATAEKRHKVDGRHVLSFIVPLANEVRCQSCHEQGARFNGAMLLTTSLEEGYAGARN

>2FWHA

ATHTAQTQTHLNFTQIKTVDELNQALVEAKGKPVMLDLYADWCVACKEFEKYTFSDPQVQKALADTVLLQANVTANDAQDVALLKHLNVLGLPTILFFDGQGQEHPQARVTGFMDAETFSAHLRDRQPHHHHHH

>2H26A

EHAFQGPTSFHVIQTSSFTNSTWAQTQGSGWLDDLQIHGWDSDSGTAIFLKPWSKGNFSDKEVAELEEIFRVYIFGFAREVQDFAGDFQMKYPFEIQGIAGCELHSGGAIVSFLRGALGGLDFLSVKNASCVPSPEGGSRAQKFCALIIQYQGIMETVRILLYETCPRYLLGVLNAGKADLQRQVKPEAWLSSGPSPGPGRLQLVCHVSGFYPKPVWVMWMRGEQEQQGTQLGDILPNANWTWYLRATLDVADGEAAGLSCRVKHSSLEGQDIILYWRNPIXXXXX

>2QYCA

GMTMFLHVVMMEFDDGIDAGFFRTVDEYVARMKRECDGLLLYHFGENVAARSQGYTHATSSAFVDAAAHDAYQVCPAHVAMKAFMGPRIKRVVVYDGEVPAIG

>2AEBA

MSAKSRTIGIIGAPFSKGQPRGGVEEGPTVLRKAGLLEKLKEQECDVKDYGDLPFADIPNDSPFQIVKNPRSVGKASEQLAGKVAEVKKNGRISLVLGGDHSLAIGSISGHARVHPDLGVIWVDAHTDINTPLTTTSGNLHGQPVSFLLKELKGKIPDVPGFSWVTPCISAKDIVYIGLRDVDPGEHYILKTLGIKYFSMTEVDRLGIGKVMEETLSYLLGRKKRPIHLSFDVDGLDPSFTPATGTPVVGGLTYREGLYITEEIYKTGLLSGLDIMEVNPSLGKTPEEVTRTVNTAVAITLACFGLAREGNHKPIDYLNPPK

>3K9TA

GMEEINKYIQNSSETGGEIYNLIEELFPICRSITGNGVRKTMDIIRKHIPLEIHEVKSGTKVFDWTVPKEWNIKDAYVRNSKGEKVIDFKENNLHVMSYSVPVHKTMTLDELKPYLHTIPGNKDRIPYLTSYYKENWGFSLTQNKFDELCDDDYEVVIDSSLEDGSLTYGEYYIRGELEEEILLTTYTCHPSMCNDNLSGVALITFIAKALSKLKTKYSYRFLFAPETIGSITWLSRNEDKLKNIKMGLVATCVGDAGIKNYKRTKFGDAEIDKIVEKVLMHCGSEYYVADFFPWGSDERQFSSPGINLSVGSLMRSCYGFDGYHTSADNLCYMNKDGLADSYKTYLEVIYTIENNRTYLNLNPKCEPQLGKRGIYRMIGGGSDYPFDEFAMFWVLNMSDGKNSLLDIAYKSGMEFRRIKYAADALYRVELLKLV

>3S4EA

ASQVGVIKPWLLLGSQDAAHDLDTLKKNKVTHILNVAYGVENAFLSDFTYKSISILDLPETNILSYFPECFEFIEEAKRKDGVVLVHSNAGVSRAAAIVIGFLMNSEQTSFTSAFSLVKNARPSICPNSGFMEQLRTYQEGKES

>2B0TA

MAKIIWTRTDEAPLLATYSLKPVVEAFAATAGIEVETRDISLAGRILAQFPERLTEDQKVGNALAELGELAKTPEANIIKLPNISASVPQLKAAIKELQDQGYDIPELPDNATTDEEKDILARYNAVKGSAVNPVLREGNSDRRAPIAVKNFVKKFPHRMGEWSADSKTNVATMDANDFRHNEKSIILDAADEVQIKHIAADGTETILKDSLKLLEGEVLDGTVLSAKALDAFLLEQVARAKAEGILFSAHLKATMMKVSDPIIFGHVVRAYFADVFAQYGEQLLAAGLNGENGLAAILSGLESLDNGEEIKAAFEKGLEDGPDLAMVNSARGITNLHVPSDVIVDASMPAMIRTSGHMWNKDDQEQDTLAIIPDSSYAGVYQTVIEDCRKNGAFDPTTMGTVPNVGLMAQKAEEYGSHDKTFRIEADGVVQVVSSNGDVLIEHDVEANDIWRACQVKDAPIQDWVKLAVTRSRLSGMPAVFWLDPERAHDRNLASLVEKYLADHDTEGLDIQILSPVEATQLSIDRIRRGEDTISVTGNVLRDYNTDLFPILELGTSAKMLSVVPLMAGGGLFETGAGGSAPKHVQQVQEENHLRWDSLGEFLALAESFRHELNNNGNTKAGVLADALDKATEKLLNEEKSPSRKVGEIDNRGSHFWLTKFWADELAAQTEDADLAATFAPVAEALNTGAADIDAALLAVQGGATDLGGYYSPNEEKLTNIMRPVAQFNEIVDALKK

>3SD2A

GANSNDIHLLKDSRSNPMGIPIQPTYEKCAILSNILNVSFGRAKDYAIITVTNKATGEIVHSKTYHNTSIVMIDMSSCEKGEYTIHIILNDCLLEGTFTVQ

>1M2DA

AEFKHVFVCVQDRPPGHPQGSCAQRGSREVFQAFMEKIQTDPQLFMTTVITPTGCMNASMMGPVVVVYPDGVWYGQVKPEDVDEIVEKHLKGGEPVERLVISKGKPPGMF

>3K1TA

GMMVPHLTTALTGPLLTLEKRLLDNMPRIEHWFRSQWQEYGAPFYASVDLRNAGFKLAPVDTNLFPGGFNNLNPDFLPLCIQAAMVAVEKICPDARRLLLIPENHTRNTFYLRNVHALTHILRQAGLEVRIGSIAPEITAPTFLETHDGHSILLEPVRRKANRLELDNFDSCAILLNNDLSGGIPDILQGLEQSLIPPLHAGWATRRKSNHFTAYDRVVEEFAPLIDIDPWLLNPYFDTCGGLDFHARLGEEQLAEKVDSLLAKIRRKYAEYGVKQEPFVIVKADAGTYGMGIMTVKSADDVRDLNRKQRNKMSVVKEGLKVSEVILQEGVYTFEHLKDAVAEPVIYMMDHFVVGGFYRVHTSRGADENLNAPGMHFEPLTFETPCSTPDCAGAPDAAPNRFYAYGVVARLALLAATIELQETDPDLLDERT

>3RONA

MENLNHCPLEDIKVNPWKTPQSTARVITLRVEDPNEINNLLSINEIDNPNYILQAIMLANAFQNALVPTSTDFGDALRFSMPKGLEIANTITPMGAVVSYVDQNVTQTNNQVSVMINKVLEVLKTVLGVALSGSVIDQLTAAVTNTFTNLNTQKNEAWIFWGKETANQTNYTYNVLFAIQNAQTGGVMYCVPVGFEIKVSAVKEQVLFFTIQDSASYNVNIQSLKFAQPLVSSSQYPIADLTSAINGTL

>2W2EA

MPDIENQAADGQAEIKPEDAPYITNAYKPAYARWGFGSDSVRNHFIAMSGEFVGTFLFLWSAFVIAQIANQAPETPDGGSNPAQLIMISFGFGFGVMVGVFITYRVSGGNLNPAVTLALVLARAIPPFRGILMAFTQIVAGMAAAGAASAMTPGEIAFANALGGGASRTRGLFLEAFGTAILCLTVLMLAVEKHRATWFAPFVIGIALLIAHLICIYYTGAGLNPARSFGPAVAARSFPNYHWIYWLGPILGAFLAYSIWQMWKWLNYQTTNPGQDSDA

>1ZELA

GAMVVSPAGADRRIPTWASRVVSGLARDRPVVVTKEDLTQRLTEAGCGRDPDSAIRELRRIGWLVQLPVKGTWAFIPPGEAAISDPYLPLRSWLARDQNAGFMLAGASAAWHLGYLDRQPDGRIPIWLPPAKRLPDGLASYVSVVRIPWNAADTALLAPRPALLVRRRLDLVAWATGLPALGPEALLVQIATRPASFGPWADLVPHLDDLVADCSDERLERLLSGRPTSAWQRASYLLDSGGEPARGQALLAKRHTEVMPVTRFTTAHSRDRGESVWAPEYQLVDELVVPLLRVIGKA

>2JCQA

MNQIDLNVTCRYAGVFHVEKNGRYSISRTEAADLCQAFNSTLPTMDQMKLALSKGFETCRYGFIEGNVVIPRIHPNAICAANHTGVYILVTSNTSHYDTYCFNASAPPEEDCTSVTDLPNSFDGPVTITIVNRDGTRYSKKGEYRTHQEDIDAS

>2VBKA

DPDQFGPDLIEQLAQSGKYSQDNTKGDAMIGVKQPLPKAVLRTQHDKNKEAISILDFGVIDDGVTDNYQAIQNAIDAVASLPSGGELFIPASNQAVGYIVGSTLLIPGGVNIRGVGKASQLRAKSGLTGSVLRLSYDSDTIGRYLRNIRVTGNNTCNGIDTNITAEDSVIRQVYGWVFDNVMVNEVETAYLMQGLWHSKFIACQAGTCRVGLHFLGQCVSVSVSSCHFSRGNYSADESFGIRIQPQTYAWSSEAVRSEAIILDSETMCIGFKNAVYVHDCLDLHMEQLDLDYCGSTGVVIENVNGGFSFSNSWIAADADGTEQFTGIYFRTPTSTQSHKIVSGVHINTANKNTAANNQSIAIEQSAIFVFVSGCTLTGDEWAVNIVDINECVSFDKCIFNKPLRYLRSGGVSVTDCYLAGITEVQKPEGRYNTYRGCSGVPSVNGIINVPVAVGATSGSAAIPNPGNLTYRVRSLFGDPASSGDKVSVSGVTINVTRPSPVGVALPSMVEYLAI

>1YQHA

SNAMSQQVTMSFSVVPQAKTKDVYSVVDKAIEVVQQSGVRYEVGAMETTLEGELDVLLDVVKRAQQACVDAGAEEVITSIKIHYRPSTGVTIDEKVWKYRDEYAKPEAI

>3SXUB

MGTSRRDWQLQQLGITQWSLRRPGALQGEIAIAIPAHVRLVMVANDLPALTDPLVSDVLRALTVSPDQVLQLTPEKIAMLPQGSHCNSWRLGTDEPLSLEGAQVASPALTDLRANPTARAALWQQICTYEHDFFPRND

>3CMGA

MSLRQDILLNNNWNFRFSHQVQGDTRRVDLPHTWNAQDALAGKIDYKRGIGNYEKALYIRPEWKGKRLFLRFDGVNSIADVFINRKHIGEHRGGYGAFIFEITDLVKYGEKNSVLVRANNGEQLDIMPLVGDFNFYGGIYRDVHLLITDETCISPLDYASPGVYLVQEVVSPQEAKVCAKVNLSNRAADGTAELQVLVTDGTKVICKESRNVSLKQGADILEQLPLLIQKPRLWNGCEDPFMYQVSISLHKDGKQIDSVTQPLGLRYYHTDPDKGFFLNGKHLPLHGVCRHQDRAEVGNALRPQHHEEDVALMREMGVNAIRLAHYPQATYMYDLMDKHGIVTWAEIPFVGPGGYADKGFVDQASFRENGKQQLIELIRQHYNHPSICFWGLFNELKEVGDNPVEYVKELNALAKQEDPTRPTTSASNQDGNLNFITENIAWNRYDGWYGSTPKTLATFLDRTHKKHPELRIGISEYGAGASIYHQQDSLKQPSASGWWHPENWQTYYHMENWKIIAERPFVWGTFVWNMFDFGAAHRTEGDRPGINDKGLVTFDRKVRKDAFYFYKANWNKQEPMIYLAEKRCRLRYQPEQTFMAFTTAPEAELFVNGVSCGKQKADTYSTVVWKNVKLTSGENIIRVTTPGKKPLTDEVTVEYKEDREGHHHHHH

>3OAKC

DPFTHMSDKIDEMYDIFGDGHDYDWALEIEN

>2GU9A

MQYATLELNNAFKVLFSLRQVQAAEMVIAPGDREGGPDNRHRGADQWLFVVDGAGEAIVDGHTQALQAGSLIAIERGQAHEIRNTGDTPLKTVNFYHPPAYDAQGEPLPAGEG

>2W56A

MSKLTFTASSLPVSKKLHKLLSKQLTAHLLSSEALTTSRYLVFNFRDKSYSADEGGFHPVEMAICQTSTGEWSIEYITDFAYMGNYYPELERNLDFDFRVGQFFVAYRGWLPMQGSRDAKELYRLWESNFLAYVDMDAYNEIAITAQ

>2HY7A

MGVSPAAPASGIRRPCYLVLSSHDFRTPRRANIHFITDQLALRGTTRFFSLRYSRLSRMKGDMRLPLDDTANTVVSHNGVDCYLWRTTVHPFNTRRSWLRPVEDAMFRWYAAHPPKQLLDWMRESDVIVFESGIAVAFIELAKRVNPAAKLVYRASDGLSTINVASYIEREFDRVAPTLDVIALVSPAMAAEVVSRDNVFHVGHGVDHNLDQLGDPSPYAEGIHAVAVGSMLFDPEFFVVASKAFPQVTFHVIGSGMGRHPGYGDNVIVYGEMKHAQTIGYIKHARFGIAPYASEQVPVYLADSSMKLLQYDFFGLPAVCPNAVVGPYKSRFGYTPGNADSVIAAITQALEAPRVRYRQCLNWSDTTDRVLDPRAYPETRLYPHPPTAAPQLSSEAALSHHHHHHH

>2BKRA

MDPVVLSYMDSLLRQSDVSLLDPPSWLNDHIIGFAFEYFANSQFHDSSDHVSFISPEVTQFIKCTSNPAEIAMFLEPLDLPNKRVVFLAINDNSNQAAGGSHWSLLVYLQDKNSFFHYDSHSRSNSVHAKQVAEKLEAFLGRKGDKLAFVEEKAPAQQNSYDCGMYVICNTEALCQNFFRQQTESLLQLLTPAYITKKRGEWKDLIATLAKK

>2C1VA

ETEAIDNGALREEAKGVFEAIPEKMTAIKQTEDNPEGVPLTAEKIELGKVLFFDPRMSSSGLISCQTCHNVGLGGVDGLPTSIGHGWQKGPRNAPTMLNAIFNAAQFWDGRAADLAEQAKGPVQAGVEMSNTPDQVVKTINSMPEYVEAFKAAFPEEADPVTFDNFAAAIEQFEATLITPNSAFDRFLAGDDAAMTDQEKRGLQAFMETGCTACHYGVNFGGQDYHPFGLIAKPGAEVLPAGDTGRFEVTRTTDDEYVFRAAPLRNVALTAPYFHSGVVWELAEAVKIMSSAQIGTELTDQQAEDITAFLGTLTGEQPVIDHPILPVRTGTTPLPTPM

>3BT3A

SLENERLIKMSRFSERGYVVRENGPVYFTKDMDKTVKWFEEILGWSGDIVARDDEGFGDYGCVFDYPSEVAVAHLTPFRGFHLFKGEPIKGVAGFMMIEGIDALHKYVKENGWDQISDIYTQPWGARECSITTTDGCILRFFESIQEG

>2HO0A

GSHMYEYPVFSHVQAGMFSPELRTFTKGDAERWVSTTKKASDSAFWLEVEGNSMTTPTGSKTSFPDGMLILVDPEQAVEPGDFCIARLGGDEFTFKKLIRDSGQVFLQPLNPQYPMIPCNESCSVVGKVIASQ

>2JH1A

VGPEAYGEASHSHSPASGRYIQQMLDQRCQEIAAELCQSGLRKMCVPSSRIVARNAVGITHQNTLQWRCFDTASLLESNQENNGVNCVDDCGHTIPCPGGVHRQNSNHATRHEILSKLVEEGVQRFCSPYQASANKYCNDKFPGTIARRSKGFGNNVEVAWRCYEKASLLYSVYAECASNCGTTWYCPGGRRGTSTELDKRHYTEEEGIRQAIGSVDSPCSEVEVCLPKDENPPLCLDESGQISRT

>2RKQA

EVPIVTRAEWNAKPPNGAIDSMVTPLPRAVIAHTAGGACADDVTCSQHMRNLQNFQMSKQKFSDIGYHYLIGGNGKVYEGRSPSQRGAFAGPNNDGSLGIAFIGNFEERAPNKEALDAAKELLEQAVKQAQLVEGYKLLGHRQVSATKSPGEALYALIQQWPNWSEEML

>3AA0B

MSDQQLDCALDLMRRLPPQQIEKNLSDLIDLVPSLCEDLLSSVDQPLKIARDKVVGKDYLLCDYNRDGDSYRSPWSNKYDPPLEDGAMPSARLRKLEVEANNAFDQYRDLYFEGGVSSVYLWDLDHGFAGVILIKKAGDGSKKIKGCWDSIHVVEVQEKSSGRTAHYKLTSTVMLWLQTNKTGSGTMNLGGSLTRQMEKDETVSDSSPHIANIGRLVEDMENKIRSTLNEIYFGKTKDIVNGLR

>3FM2A

GMSHSLKDFLEACETLGTLRLIVTSSAAVLEARGKIEKLFYAELAKGKYANMHTEGFEFHLNMEKITQVKFETGEAKRGNFTTYAIRFLDEKQESALSLFLQWGKPGEYEPGQVEAWHTLKEKYGEVWEPLPVQL

>3GS9A

GMNSDIIVADFWKNNEEILTDFDKDSFCESWTENEMWSIEFKVAQTPKNAHCYSFLDYESSVYFRGQEFVVKQLSHDAVGKTLSKDIRAPHIYYTCQDGRQDDAITGSFTLEQCLTHIFKTDNRGFSWEIIDPSNILEKVQQENFGNNNYLTLIDQLLDDYGVVVIPDNRHLVFKPREIYGAKTENFIRYKYNTDEASFDIDTLSLKTKIKGYGKVDSNGNNYFSPITYTSPEVEKWGIRWQEPVSDERYTVAGNMQRRLKLELQDYPATTGSVILKNDYECEKGDYVLFIYEPLGIDYDVQIVAYKKYPFTIKAPEITLSNNKKSIVSIMAQLAKVLKGAK

>3ATSA

TLPAVISRWLSSVLPGGAAPEVTVESGVDSTGMSSETIILTARWQQDGRSIQQKLVARVAPAAEDVPVFPTYRLDHQFEVIRLVGELTDVPVPRVRWIETTGDVLGTPFFLMDYVEGVVPPDVMPYTFGDNWFADAPAERQRQLQDATVAALATLHSIPNAQNTFSFLTQGRTSDTTLHRHFNWVRSWYDFAVEGIGRSPLLERTFEWLQSHWPDDAAAREPVLLWGDARVGNVLYRDFQPVAVLDWEMVALGPRELDVAWMIFAHRVFQELAGLATLPGLPEVMREDDVRATYQALTGVELGDLHWFYVYSGVMWACVFMRTGARRVHFGEIEKPDDVESLFYHAGLMKHLLGEEH

>4DUQA

MMSSSLEQALAVLVTTFHKYSSQEGDKFKLSKGEMKELLHKELPSFVGEKVDEEGLKKLMGSLDENSDQQVDFQEYAVFLALITVMSNDFFQGCPDRP

>3SNOA

GMALEPQIKSAPTPVILIVEPYGGSIRQQNPNLPMVFWDDAALTRGDGIFETLLIRDGHACNVRRHGERFKASAALLGLPEPILEDWEKATQMGIESWYSHPNAGEASCTWTLSRGRSSTGLASGWLTITPVSSDKLAQREHGVSVMTSSRGYSIDTGLPGIGKATRGELSKVERTPAPWLTVGAKTLAYAANMAALRYAKSNGFDDVIFTDGDRVLEGATSTVVSFKGDKIRTPSPGGDILPGTTQAALFAHATEKGWRCKEKDLSIDDLFGADSVWLVSSVRGPVRVTRLDGHKLRKPDNEKEIKALITKALG

>3KF8B

SSKIILIPSNIPQEFPEASISNPERLRILAQVKDFIPHESTIVIDKVPTITSEQSTYINICIFNLLEACSSRVLVPGTLVNIDAFYDGESINPVDIYEVNGANFTMENIQLIDEMNNSIGKFN

>2GR8A

ASWSHPQFEKSGGGGGLVPRGSKRADAGTASALAASQLPQATMPGKSMVAIAGSSYQGQNGLAIGVSRISDNGKVIIRLSGTTNSQGKTGVAAGVGYQW

>2WB7A

MNATINDDDIDDVKKALDHATQAAHKAAAELTAKLRSDFVEYGNGGTAGQVLIHIYGPGLIYGFSAFPVQIRLEIPNQPVPFNKVHITEVTAYVIDENNRTYWTRVWNSSTFRQGGYIADTLDLVTVMKAPDPLVYQIRDAIVTGQISRELYDKIWNTSTTHFEIRVIVKGYQEAWKTDSSVSNQSSCPSDGHWYEDACWVHDKDIDFTLKAETTTAWGHVTGTNDVATIDGGMLGSLPIKFLQSLDLSGKWVLYQNKYAGALSDFIIITAASPVHVLNSTAMYKFLITPNPGYFQPANPKISDEYRFVTLRVIEGGRMELADTTTGHIGDLTEPTFFGLTAHYTDAPGTLDYHALGLVYAYVERDDGVKIPIWLAAEPMISVLSNTYTVMKDQDVKNLIDLYKKKDREKINATTKAMINSLQEKIDEAEQLLAKAKGMNNENAIEYAQGAIDEYKAAINDLQKAAQQDDYQMFLNYLNAAKKHEMAGDYYVNAARKALNGDLEQAKIDAEKAKEYSNLAKEYEPG

>3PF0A

GDDNNAAEVDRQVAQDSAEPKTGENAAAGDSSSTNKNAEKIVAVDISAETEKTYLTHVANDMVIPAYADAAKQSDLLHDLAQKHCQKAPVSGDELQALRDQWLVLAQAWASAEMVNFGPATASMSNLYINYYPDERGLVHGGVADLITANPALTAEQLANESAVVQGIPGLEEALYANDSLDAGQCAYVMSASSALGTRLKDIEKNWQQNAIKLLAIDKTAESDQGLNQWFNSLLSLVETMKSNAIEQPLGLSGKAKGHLPAATAGQSRAIINAKLATLNKAMTDPVLTAILGSNNENTVADTLSTALADTTALLAQMPEDLATADKATQQELYDHLTNITRLIKSQLIPTLGIRVGFNSTDGD

>3FEGA

GSSHHHHHHSSGLVPRGSRRRASSLSRDAERRAYQWCREYLGGAWRRVQPEELRVYPVSGGLSNLLFRCSLPDHLPSVGEEPREVLLRLYGAILQGVDSLVLESVMFAILAERSLGPQLYGVFPEGRLEQYIPSRPLKTQELREPVLSAAIATKMAQFHGMEMPFTKEPHWLFGTMERYLKQIQDLPPTGLPEMNLLEMYSLKDEMGNLRKLLESTPSPVVFCHNDIQEGNILLLSEPENADSLMLVDFEYSSYNYRGFDIGNHFCEWVYDYTHEEWPFYKARPTDYPTQEQQLHFIRHYLAEAKKGETLSQEEQRKLEEDLLVEVSRYALASHFFWGLWSILQASMSTIEFGYLDYAQSRFQFYFQQKGQLTSVHSSS

>2NPTA

SMALGPFPAMENQVLVIRIKIPNSGAVDWTVHSGPQLLFRDVLDVIGQVLPEATTTAFEYEDEDGDRITVRSDEEMKAMLSYYYSTVMEQQVNGQLIEPLQIFPRA

>2IN5A

MTHSQQSMVDTFRASLFDNQDITVADQQIQALPYSTMYLRLNEGQRIFVVLGYIEQEQSKWLSQDNAMLVTHNGRLLKTVKLNNNLLEVTNSGQDPLRNALAIKDGSRWTRDILWSEDNHFRSATLSSTFSFAGLETLNIAGRNVLCNVWQEEVTSTRPEKQWQNTFWVDSATGQVRQSRQMLGAGVIPVEMTFLKPAPLEHHHHHH

>2BDRA

MRTLMIEPLTKEAFAQFGDVIETDGSDHFMINNGSTMRFHKLATVETAEPEDKAIISIFRADAQDMPLTVRMLERHPLGSQAFIPLLGNPFLIVVAPVGDAPVSGLVRAFRSNGRQGVNYHRGVWHHPVLTIEKRDDFLVVDRSGSGNNCDEHYFTEEQMLILNPHQLEHHHHHH

>1IUQA

ASHSRKFLDVRSEEELLSCIKKETEAGKLPPNVAAGMEELYQNYRNAVIESGNPKADEIVLSNMTVALDRILLDVEDPFVFSSHHKAIREPFDYYIFGQNYIRPLIDFGNSFVGNLSLFKDIEEKLQQGHNVVLISNHQTEADPAIISLLLEKTNPYIAENTIFVAGDRVLADPLCKPFSIGRNLICVYSKKHMFDIPELTETKRKANTRSLKEMALLLRGGSQLIWIAPSGGRDRPDPSTGEWYPAPFDASSVDNMRRLIQHSDVPGHLFPLALLCHDIMPPPSQVEIEIGEKRVIAFNGAGLSVAPEISFEEIAATHKNPEEVREAYSKALFDSVAMQYNVLKTAISGKQGLGASTADVSLSQPW

>2W5QA

SEDDLTKVLNYTKQRQTEPNPEYYGVAKKKNIIKIHLESFQTFLINKKVNGKEVTPFLNKLSSGKEQFTYFPNFFHQTGQGKTSDSEFTMDNSLYGLPQGSAFSLKGDNTYQSLPAILDQKQGYKSDVMHGDYKTFWNRDQVYKHFGIDKFYDATYYDMSDKNVVNLGLKDKIFFKDSANYQAKMKSPFYSHLITLTNHYPFTLDEKDATIEKSNTGDATVDGYIQTARYLDEALEEYINDLKKKGLYDNSVIMIYGDHYGISENHNNAMEKLLGEKITPAKFTDLNRTGFWIKIPGKSGGINNEYAGQVDVMPTILHLAGIDTKNYLMFGTDLFSKGHNQVVPFRNGDFITKDYKYVNGKIYSNKNNELITTQPADFEKNKKQVEKDLEMSDNVLNGDLFRFYKNPDFKKVNPSKYKYETGPK

>3NSWA

GSHMEYCPKMLSEIRQEDINDVETVAYVTVTGKTARSYNLQYWRLYDVPKTAPSQWPSFGTLRDDCGNIQLTADTDYVLGCKSGNQDCFVKLHDGLSQKEKDLLKE

>3F79A

AANRELQASLNLLQEDQNAGRQVQMNMLPVTPWSIEGLEFSHRIIPSLYLSGDFVDYFRVDERRVAFYLADVSGHGASSAFVTVLLKFMTTRLLYESRRNGTLPEFKPSEVLAHINRGLINTKLGKHVTMLGGVIDLEKNSLTYSIGGHLPLPVLFVEGQAGYLEGRGLPVGLFDDATYDDRVMELPPSFSLSLFSDGILDVLPGATLKEKEASLPEQVAAAGGTLDGLRQVFGLANLAEMPDDIALLVLSRNLA

>3C5PA

SNAMTNIIKIRASVFIPMSWTEAKMDMETGQVIQFEGDSREFTPHAVNTMRSRVEQEVVVDFYKQEVFSYANTGITTEKVISPDGSVNKRTGKASTENIVCTDIVWNSGGVQFKMSASASNPLNVYAPPVDYVLNVCVKKDGSIDVQGEHDGFPCFEFYKQVDFGPFEKIYTHDFRETGDTAAALGGNMDYSFTKRL

>3NQIA

GMDSGESGPQQWAGVVKVNDRMGYVTFTDAAGTELIPTNTIPVTLNARMAYIYCQVDEGQDLSTNPKSIKITLLADPTGIDATAITTPKVGESGDVTTNAPVGSLSFVSGYSTVAPFQFSENTIVLPVLYRVKNVTTTEDIKNELAKHTFTLVCYTDDIKSGDTILKLYLRYKVEDEPAAIAERATRTSSFKAYEISQILREYTLKSGQTKPAKITIVAQQNEYNNKLEDTSTIEKVYEIEYKTAE

>1DS1A

MTSVDCTAYGPELRALAARLPRTPRADLYAFLDAAHTAAASLPGALATALDTFNAEGSEDGHLLLRGLPVEADADLPTTPSSTPAPEDRSLLTMEAMLGLVGRRLGLHTGYRELRSGTVYHDVYPSPGAHHLSSETSETLLEFHTEMAYHRLQPNYVMLACSRADHERTAATLVASVRKALPLLDERTRARLLDRRMPCCVDVAFRGGVDDPGAIAQVKPLYGDADDPFLGYDRELLAPEDPADKEAVAALSKALDEVTEAVYLEPGDLLIVDNFRTTHARTPFSPRWDGKDRWLHRVYIRTDRNGQLSGGERAGDVVAFTPRG

>1KF6C

TTKRKPYVRPMTSTWWKKLPFYRFYMLREGTAVPAVWFSIELIFGLFALKNGPEAWAGFVDFLQNPVIVIINLITLAAALLHTKTWFELAPKAANIIVKDEKMGPEPIIKSLWAVTVVATIVILFVALYW

>1JYHA

MNYEIKQEEKRTVAGFHLVGPWEQTVKKGFEQLMMWVDSKNIVPKEWVAVYYDNPDETPAEKLRCDTVVTVPGYFTLPENSEGVILTEITGGQYAVAVARVVGDDFAKPWYQFFNSLLQDSAYEMLPKPCFEVYLNNGAEDGYWDIEMYVAVQPKHH

>1H97A

TLTKHEQDILLKELGPHVDTPAHIVETGLGAYHALFTAHPQYISHFSRLEGHTIENVMQSEGIKHYARTLTEAIVHMLKEISNDAEVKKIAAQYGKDHTSRKVTKDEFMSGEPIFTKYFQNLVKDAEGKAAVEKFLKHVFPMMAAEI

>3GRAA

MSLAPYRVDFILLEHFSMASFTVAMDVLVTANLLRADSFQFTPLSLDGDRVLSDLGLELVATELSAAALKELDLLVVCGGLRTPLKYPELDRLLNDCAAHGMALGGLWNGAWFLGRAGVLDDYGCSIHPEQRASLSERSPQTRITPASFTLDRDRLSAASPNGAMELMLGLVRRLYGDGLAEGVEEILSFSGAREGHHHHHH

>2IVFC

MKAKRVPGGKELLLDLDAPIWAGAESTTFEMFPTPLVMVKEVSPFLALSEGHGVIKRLDVAALHNGSMIALRLKWASEKHDKIVDLNSFVDGVGAMFPVARGAQAVTMGATGRPVNAWYWKANANEPMEIVAEGFSAVRRMKDKAGSDLKAVAQHRNGEWNVILCRSMATGDGLAKLQAGGSSKIAFAVWSGGNAERSGRKSYSGEFVDFEILK

>1QV9A

MTVAKAIFIKCGNLGTSMMMDMLLDERADREDVEFRVVGTSVKMDPECVEAAVEMALDIAEDFEPDFIVYGGPNPAAPGPSKAREMLADSEYPAVIIGDAPGLKVKDEMEEQGLGYILVKPDAMLGARREFLDPVEMAIYNADLMKVLAATGVFRVVQEAFDELIEKAKEDEISENDLPKLVIDRNTLLEREEFENPYAMVKAMAALEIAENVADVSVEGCFVEQDKERYVPIVASAHEMMRKAAELADEARELEKSNDAVLRTPHAPDGKVLSKRKFMEDPE

>2XTCA

MSITSDEVNFLVYRYLQESGFSHSAFTFGIESHISQSNINGTLVPPAALISILQKGLQYVEAEISINEDGTVFDGRPIESLSLIDAVMPD

>3HTRA

SNAMTPETNETLKLIGSDKVQGTAVYGPDGEKIGSIERVMIEKVSGRVSYAVLSFGGFLGIGDDHYPLPWPALKYNVELGGYQVMVTVDQLERAPKYGPGSEWDWRGARKVDDYYGVALT

>1ZZ1A

MAIGYVWNTLYGWVDTGTGSLAAANLTARMQPISHHLAHPDTKRRFHELVCASGQIEHLTPIAAVAATDADILRAHSAAHLENMKRVSNLPTGGDTGDGITMMGNGGLEIARLSAGGAVELTRRVATGELSAGYALVNPPGHHAPHNAAMGFCIFNNTSVAAGYARAVLGMERVAILDWDVHHGNGTQDIWWNDPSVLTISLHQHLCFPPDSGYSTERGAGNGHGYNINVPLPPGSGNAAYLHAMDQVVLPALRAYRPQLIIVGSGFDASMLDPLARMMVTADGFRQMARRTIDCAADICDGRIVFVQEGGYSPHYLPFCGLAVIEELTGVRSLPDPYHEFLAGMGGNTLLDAERAAIEEIVPLLADIR

>1KJQA

TLLGTALRPAATRVMLLGSGELGKEVAIECQRLGVEVIAVDRYADAPAMHVAHRSHVINMLDGDALRRVVELEKPHYIVPEIEAIATDMLIQLEEEGLNVVPCARATKLTMNREGIRRLAAEELQLPTSTYRFADSESLFREAVADIGYPCIVKPVMSSSGKGQTFIRSAEQLAQAWKYAQQGGRAGAGRVIVEGVVKFDFEITLLTVSAVDGVHFCAPVGHRQEDGDYRESWQPQQMSPLALERAQEIARKVVLALGGYGLFGVELFVCGDEVIFSEVSPRPHDTGMVTLISQDLSEFALHVRAFLGLPVGGIRQYGPAASAVILPQLTSQNVTFDNVQNAVGADLQIRLFGKPEIDGSRRLGVALATAESVVDAIERAKHAAGQVKVQG

>2GUFA

QDTSPDTLVVTANRFEQPRSTVLAPTTVVTRQDIDRWQSTSVNDVLRRLPGVDITQNGGSGQLSSIFIRGTNASHVLVLIDGVRLNLAGVSGSADLSQFPIALVQRVEYIRGPRSAVYGSDAIGGVVNIITTRDEPGTEISAGWGSNSYQNYDVSTQQQLGDKTRVTLLGDYAHTHGYDVVAYGNTGTQAQTDNDGFLSKTLYGALEHNFTDAWSGFVRGYGYDNRTNYDAYYSPGSPLLDTRKLYSQSWDAGLRYNGELIKSQLITSYSHSKDYNYDPHYGRYDSSATLDEMKQYTVQWANNVIVGHGSIGAGVDWQKQTTTPGTGYVEDGYDQRNTGIYLTGLQQVGDFTFEGAARSDDNSQFGRHGTWQTSAGWEFIEGYRFIASYGTSYKAPNLGQLYGFYGNPNLDPEKSKQWEGAFEGLTAGVNWRISGYRNDVSDLIDYDDHTLKYYNEGKARIKGVEATANFDTGPLTHTVSYDYVDARNAITDTPLLRRAKQQVKYQLDWQLYDFDWGITYQYLGTRYDKDYSSYPYQTVKMGGVSLWDLAVAYPVTSHLTVRGKIANLFDKDYETVYGYQTAGREYTLSGSYTF

>2TNFA

LRSSSQNSSDKPVAHVVANHQVEEQLEWLSQRANALLANGMDLKDNQLVVPADGLYLVYSQVLFKGQGCPDYVLLTHTVSRFAISYQEKVNLLSAVKSPCPKDTPEGAELKPWYEPIYLGGVFQLEKGDQLSAEVNLPKYLDFAESGQVYFGVIAL

>4GJZA

STVPRLHRPSLQHFREQFLVPGRPVILKGVADHWPCMQKWSLEYIQEIAGCRTVPVEVGSRYTDEEWSQTLMTVNEFISKYIVNEPRDVGYLAQHQLFDQIPELKQDISIPDYCSLGDGEEEEITINAWFGPQGTISPLHQDPQQNFLVQVMGRKYIRLYSPQESGALYPHDTHLLHNTSQVDVENPDLEKFPKFAKAPFLSCILSPGEILFIPVKYWHYVRALDLSFSVSFWWS

>3A57A

FELPSVPFPAPGSDEILFVVRDTTFNTNAPVNVEVSDFWTNRNVKRKPYKDVYGQSVFTTSGTKWLTSYMTVNINDKDYTMAAVSGYKHGHSAVFVKSDQVQLQHSYDSVASFVGEDEDSIPSKMYLDETPEYFVNVEAYESGSGNILVMCISNKESFFECKHQQ

>2Y7LA

RKTITGVFNSFDSLTWTRSVEYVYKGPETPTWNAVLGWSLNSTTADPGDTFTLILPCVFKFITTQTSVDLTADGVSYATCDFNAGEEFTTFSSLSCTVNSVSVSYARVSGTVKLPITFNVGGTGSSVDLADSKCFTAGKNTVTFMDGDTKISTTVDFDASPVSPSGYITSSRIIPSLNKLSSLFVVPQCENGYTSGIMGFVASNGATIDCSNVNIGISKGLNDWNFPVSSESFSYTKTCTSTSITVEFQNVPAGYRPFVDAYISAENIDKYTLTYANEYTCENGNTVVDPFTLTWWGYKNSEADSDGDVIVV

>2W39A

ATYHLEDNWVGSAFLSTFTHEAIADPTHGRVNYVDQATALAKNLTYASGDTLILRADHTTTLSPSGPGRNSVRIRSIKTYTTHVAVFDVRHMPQGCGTWPAAWETDEGDWPNGGEVDIIEGVNDQSPNAMTLHTGANCAMPASRTMTGHATNNNCDVNTDGNTGCGVQAPTANSYGPSFNANGGGWYAMERTNSFIKVWFFPRNAGNVPNDIASGPATINTDNWGTPTAFFPNTNCDIGSHFDANNIIINLTFCGDWAGQASIFNGAGCPGSCVDYVNNNPSAFANAYWDIASVRVYQ

>1O8BA

MTQDELKKAVGWAALQYVQPGTIVGVGTGSTAAHFIDALGTMKGQIEGAVSSSDASTEKLKSLGIHVFDLNEVDSLGIYVDGADEINGHMQMIKGGGAALTREKIIASVAEKFICIADASKQVDILGKFPLPVEVIPMARSAVARQLVKLGGRPEYRQGVVTDNGNVILDVHGMEILDPIAMENAINAIPGVVTVGLFANRGADVALIGTPDGVKTIVK

>2XU3A

APRSVDWREKGYVTPVKNQGQCGSCWAFSATGALEGQMFRKTGRLISLSEQNLVDCSGPQGNEGCNGGLMDYAFQYVQDNGGLDSEESYPYEATEESCKYNPKYSVANDTGFVDIPKQEKALMKAVATVGPISVAIDAGHESFLFYKEGIYFEPDCSSEDMDHGVLVVGYGFESTESDNNKYWLVKNSWGEEWGMGGYVKMAKDRRNHCGIASAASYPTV

>2CARA

GSMAASLVGKKIVFVTGNAKKLEEVVQILGDKFPCTLVAQKIDLPEYQGEPDEISIQKCQEAVRQVQGPVLVEDTCLCFNALGGLPGPYIKWFLEKLKPEGLHQLLAGFEDKSAYALCTFALSTGDPSQPVRLFRGRTSGRIVAPRGCQDFGWDPCFQPDGYEQTYAEMPKAEKNAVSHRFRALLELQEYFGSLAA

>3A77A

GAMENPLKRLLVPGEEWEFEVTAFYRGRQVFQQTISCPEGLRLVGSEVGDRTLPGWPVTLPDPGMSLTDRGVMSYVRHVLSCLGGGLALWRAGQWLWAQRLGHCHTYWAVSEELLPNSGHGPDGEVPKDKEGGVFDLGPFIVDLITFTEGSGRSPRYALWFCVGESWPQDQPWTKRLVMVKVVPTCLRALVEMARVGGASSLENTVDLHISNSHPLSLTSDQYKAYLQDLVEGMDFQGPGES

>3HG9A

MSLTSSAELAEVDTLARSLLLYRSRLAEYAHANPGFSGSPADSALGLPAWFRKPVRLQGYIAAGTSYAFIASPPAGLAAAVDTGTESDLVGVRRNGQLVTRRLGATAIALPAPIPEGAVVAVKEGHHHHHH

>3DR5A

MSNAFEYLRTYVESTTETDAAVARAREDAAEFGLPAPDEMTGQLLTTLAATTNGNGSTGAIAITPAAGLVGLYILNGLADNTTLTCIDPESEHQRQAKALFREAGYSPSRVRFLLSRPLDVMSRLANDSYQLVFGQVSPMDLKALVDAAWPLLRRGGALVLADALLDGTIADQTRKDRDTQAARDADEYIRSIEGAHVARLPLGAGLTVVTKALEHHHHHH

>2CH5A

PQFMAAIYGGVEGGGTRSEVLLVSEDGKILAEADGLSTNHWLIGTDKCVERINEMVNRAKRKAGVDPLVPLRSLGLSLSGGDQEDAGRILIEELRDRFPYLSESYLITTDAAGSIATATPDGGVVLISGTGSNCRLINPDGSESGCGGWGHMMGDEGSAYWIAHQAVKIVFDSIDNLEAAPHDIGYVKQAMFHYFQVPDRLGILTHLYRDFDKCRFAGFCRKIAEGAQQGDPLSRYIFRKAGEMLGRHIVAVLPEIDPVLFQGKIGLPILCVGSVWKSWELLKEGFLLALTQGREIQAQNFFSSFTLMKLRHSSALGGASLGARHIGHLLPMDYSANAIAFYSYTFS

>4JQSA

GQVPEGYPANYAKAPRFKALIYYTQHAEEAHVQFAEQATTFFKKLNYGDGFVLDITTDFSKYPYEKLKEYNVIIMLNTSPNTKAERDAFEQYMENGGGWVGFHAAAYNDKNTHWPWFVKFLGGGVFYCNNWPPQPVLVEVDNEEHPVTKNLPASFVAPASEWYQWTPSPRQNKDVEVLLSLSPKNYPLGIKDVVNFGDFPIVWSNKNYRMIYLNMGHGDEEFIDGTQNLLLVNAFRWVVSKDKSGNPFLK

>3CTZA

MPPKVTSELLRQLRQAMRNSEYVTEPIQAYIIPSGDAHQSEYIAPCDCRRAFVSGFDGSAGTAIITEEHAAMWTDGRYFLQAAKQMDSNWTLMKMGLKDTPTQEDWLVSVLPEGSRVGVDPLIIPTDYWKKMAKVLRSAGHHLIPVKENLVDKIWTDRPERPCKPLLTLGLDYTGISWKDKVADLRLKMAERNVMWFVVTALDEIAWLFNLRGSDVEHNPVFFSYAIIGLETIMLFIDGDRIDAPSVKEHLLLDLGLEAEYRIQVHPYKSILSELKALCADLSPREKVWVSDKASYAVSETIPKDHRCCMPYTPICIAKAVKNSAESEGMRRAHIKDAVALCELFNWLEKEVPKGGVTEISAADKAEEFRRQQADFVDLSFPTISSTGPTGAIIHYAPVPETNRTLSLDEVYLIDSGAQYKDGTTDVTRTMHFETPTAYEKECFTYVLKGHIAVSAAVFPTGTKGHLLDSFARSALWDSGLDYLHGTGHGVGSFLNVHEGPCGISYKTFSDEPLEAGMIVTDEPGYYEDGAFGIRIENVVLVVPVKTKYNFNNRGSLTLEPLTLVPIQTKMIDVDSLTDKECDWLNNYHLTCRDVIGKELQKQGRQEALEWLIRETQPISKQH

>1T77A

GPVSLSTPAQLVAPSVVVKGTLSVTSSELYFEVDEEDPNFKKIDPKILAYTEGLHGKWLFTEIRSIFSRRYLLQNTALEIFMANRVAVMFNFPDPATVKKVVNFLPRVGVGTSFGLPQTRRISLASPRQLFKASNMTQRWQHREISNFEYLMFLNTIAGRSYNDLNQYPVFPWVITNYESEELDLTLPTNFRDLSKPIGALNPKRAAFFAERYESWEDDQVPKFHYGTHYSTASFVLAWLLRIEPFTTYFLNLQGGKFDHADRTFSSISRAWRNSQRDTSDIKELIPEFYYLPEMFVNFNNYNLGVMDDGTVVSDVELPPWAKTSEEFVHINRLALESEFVSCQLHQWIDLIFGYKQQGPEAVRALNVFYYLTYEGAVNLNSITDPVLREAVEAQIRSFGQTPSQLLIEPHPPR

>3NKUA

GHMMSVNEEQFGSLYSDERDKPLLSPTAQKKFEEYQNKLANLSKIIRENEGNEVSPWQEWENGLRQIYKEMIYDAFDALGVEMPKDMEVHFAGSLAKAQATEYSDLDAFVIVKNDEDIKKVKPVFDALNNLCQRIFTASNQIYPDPIGINPSRLIGTPDDLFGMLKDGMVADVEATAMSILTSKPVLPRYELGEELRDKIKQEPSFSNMVSAK

>2PKEA

GMTPIAQRDGQAIQLVGFDGDDTLWKSEDYYRTAEADFEAILSGYLDLGDSRMQQHLLAVERRNLKIFGYGAKGMTLSMIETAIELTEARIEARDIQRIVEIGRATLQHPVEVIAGVREAVAAIAADYAVVLITKGDLFHQEQKIEQSGLSDLFPRIEVVSEKDPQTYARVLSEFDLPAERFVMIGNSLRSDVEPVLAIGGWGIYTPYAVTWAHEQDHGVAADEPRLREVPDPSGWPAAVRALDAQAGRQQ

>2D4XA

MSDDPIAASQAVVLSQAQAQNSQYALARTFATQKVSLEESVLSQVTTAIQTAQEKIVYAGNGTLSDDDRASLATDLQGIRDQLMNLANSTDGNGRYIFAGYKTEAAPFDQATGGYHGGEKSVTQQVDSARTMVIGHTGAQIFNSITSNAVPEPDGSDSEKNLFVMLDTAIAALKTPVEGNNVEKEKAAAAIDKTNRGLKNSLNNVLTVRAELGTQLSELSTLDSLGSDRALGQKLQMSNLVDVDWNSV

>2W68A

AAMALFDYNATGDTEFDSPAKQGWMQDNTNNGSGVLTNADGMPAWLVQGIGGRAQWTYSLSTNQHAQASSFGWRMTTEMKVLSGGMITNYYANGTQRVLPIISLDSSGNLVVEFEGQTGRTVLATGTAATEYHKFELVFLPGSNPSASFYFDGKLIRDNIQPTASKQNMIVWGNGSSNTDGVAAYRDIKFEIQGD

>2JK9A

SMQELQGLDYCKPTRLDLLLDMPPVSYDVQLLHSWNNNDRSLNVFVKEDDKLIFHRHPVAQSTDAIRGKVGYTRGLHVWQITWAMRQRGTHAVVGVATADAPLHSVGYTTLVGNNHESWGWDLGRNRLYHDGKNQPSKTYPAFLEPDETFIVPDSFLVALDMDDGTLSFIVDGQYMGVAFRGLKGKKLYPVVSAVWGHCEIRMRYLNGLDPE

>1JYHA

MNYEIKQEEKRTVAGFHLVGPWEQTVKKGFEQLMMWVDSKNIVPKEWVAVYYDNPDETPAEKLRCDTVVTVPGYFTLPENSEGVILTEITGGQYAVAVARVVGDDFAKPWYQFFNSLLQDSAYEMLPKPCFEVYLNNGAEDGYWDIEMYVAVQPKHH

>2F6EA

YYFEPNTAIGANGYKIIDNKNFYFRNGLPQIGVFKGPNGFEYFAPANTDANNIEGQAIRYQNRFLHLLGNIYYFGNNSKAVTGWQTINGNMYYFMPDTAMAAAGGLFEIDGVIYFFGVDGVKAPGIY

>3GP6A

MNADEWMTTFRENIAQTWQQPEHYDLYIPAITWHARFAYDKEKTDRYNERPWGGGFGLSRWDEKGNWHGLYAMAFKDSWNKWEPIAGYGWESTWRPLADENFHLGLGFTAGVTARDNWNYIPLPVLLPLASVGYGPVTFQMTYIPGTYNNGNVYFAWMRFQFL

>3DR5A

MSNAFEYLRTYVESTTETDAAVARAREDAAEFGLPAPDEMTGQLLTTLAATTNGNGSTGAIAITPAAGLVGLYILNGLADNTTLTCIDPESEHQRQAKALFREAGYSPSRVRFLLSRPLDVMSRLANDSYQLVFGQVSPMDLKALVDAAWPLLRRGGALVLADALLDGTIADQTRKDRDTQAARDADEYIRSIEGAHVARLPLGAGLTVVTKALEHHHHHH

>3QPAA

GRTTRDDLINGNSASCADVIFIYARGSTETGNLGTLGPSIASNLESAFGKDGVWIQGVGGAYRATLGDNALPRGTSSAAIREMLGLFQQANTKCPDATLIAGGYXQGAALAAASIEDLDSAIRDKIAGTVLFGYTKNLQNRGRIPNYPADRTKVFCNTGDLVCTGSLIVAAPHLAYGPDARGPAPEFLIEKVRAVRG

>1L8WA

MRGSHHHHHHGSSQVADKDDPTNKFYQSVIQLGNGFLDVFTSFGGLVAEAFGFKSDPKKSDVKTYFTTVAAKLEKTKTDLNSLPKEKSDISSTTGKPDSTGSVGTAVEGAIKEVSELLDKLVKAVKTAEGASSGTAAIGEVVADADAAKVADKASVKGIAKGIKEIVEAAGGSEKLKAVAAAKGENNKGAGKLFGKAGAAAHGDSEAASKAAGAVSAVSGEQILSAIVTAADAAEQDGKKPEEAKNPIAAAIGDKDGGAEFGQDEMKKDDQIAAAIALRGMAKDGKFAVKDGEKEKAEGAIKGAAESAVRKVLGAITGLIGDAVSSGLRKVGDSVKAASKETPPALNK

>3EF2A

MSLRRGIYHIENAGVPSAIDLKDGSSSDGTPIVGWQFTPDTINWHQLWLAEPIPNVADTFTLCNLFSGTYMDLYNGSSEAGTAVNGWQGTAFTTNPHQLWTIKKSSDGTSYKIQNYGSKTFVDLVNGDSSDGAKIAGWTGTWDEGNPHQKWYFNRMSVSSAEAQAAIARNPHIHGTYRGYILDGEYLVLPNATFTQIWKDSGLPGSKWREQIYDCDDFAIAMKAAVGKWGADSWKANGFAIFCGVMLGVNKAGDAAHAYNFTLTKDHADIVFFEPQNGGYLNDIGYDSYMAFY

>2FREA

MTNSNNRQSEYPVDPLFLDRWSPRAFDGSPMPKEHLLTILDAAHWAPSASNHQPWRFVYAHKDSEDWPLFVELLMEGNQKWAKNASVLLFVISRDHTISHEGEKKPSATHSFDAGAAWFSLAMQAHLLGYHAHGMGGIFKDRIVEKLDIPDGFKVEAGVAIGTLTDKSILPDDLAEREVPSKRVPLADVAFEGRFTGKAD

>2PRSA

AVVASLKPVGFIASAIADGVTETEVLLPDGASEHDYSLRPSDVKRLQNADLVVWVGPEMEAFMQKPVSKLPGAKQVTIAQLEDVKPLLMKSIHGDDDDHDHAEKSDEDHHHGDFNMHLWLSPEIARATAVAIHGKLVELMPQSRAKLDANLKDFEAQLASTETQVGNELAPLKGKGYFVFHDAYGYFEKQFGLTPLGHFTVNPEIQPGAQRLHEIRTQLVEQKATCVFAEPQFRPAVVESVARGTSVRMGTLDPLGTNIKLGKTSYSEFLSQLANQYASCLKGD

>2EX4A

MGSSHHHHHHSSGLVPRGSTSEVIEDEKQFYSKAKTYWKQIPPTVDGMLGGYGHISSIDINSSRKFLQRFLREGPNKTGTSCALDCGAGIGRITKRLLLPLFREVDMVDITEDFLVQAKTYLGEEGKRVRNYFCCGLQDFTPEPDSYDVIWIQWVIGHLTDQHLAEFLRRCKGSLRPNGIIVIKDNMAQEGVILDDVDSSVCRDLDVVRRIICSAGLSLLAEERQENLPDEIYHVYSFALR

>3M9VA

GHMPPWTARQDSTTGLYAPVTPAGRVLLDRLAAHLPRIRSTAAEHDRDGTFPTDTFDALRKDGLMGATVPAELGGLGVDRLYDVAVALLAVARADASTALALHMQLSRGLTLGYEWRHGDERARTLAERILRGMVAGDAVVCSGIKDHHTAVTTLRPDGAGGWLLSGRKTLVSMAPVGTHFVINARTDGTDGPPRLASPVVTRDTPGFTVLDNWDGLGMRASGTVDIVFDDCPIPADHVLMRDPVGARNDAVLAGQTVSSVSVLGVYVGVAQAAYDTAVAALERRPEPPQAAALTLVAEIDSRLYALRATAGSALTAADALSADLSGDMDERGRQMMRHFQCAKLAVNRLAPEIVSDCLSLVGGASYTAGHPLARLLRDVQAGRFMQPYAYVDAVDFLSAQALGIERDNNYMSTWAKRSGGNGKSADAAGPRRPTPTSR

>1PSWA

MKILVIGPSWVGDMMMSQSLYRTLQARYPQAIIDVMAPAWCRPLLSRMPEVNEAIPMPLGHGALEIGERRKLGHSLREKRYDRAYVLPNSFKSALVPLFAGIPHRTGWRGEMRYGLLNDVRVLDKEAWPLMVERYIALAYDKGIMRTAQDLPQPLLWPQLQVSEGEKSYTCNQFSLSSERPMIGFCPGAEFGPAKRWPHYHYAELAKQLIDEGYQVVLFGSAKDHEAGNEILAALNTEQQAWCRNLAGETQLDQAVILIAACKAIVTNDSGLMHVAAALNRPLVALYGPSSPDFTPPLSHKARVIRLITGYHKVRKGDAAEGYHQSLIDITPQRVLEELNALLLQEEA

>3RC9A

MGSSHHHHHHENLYFQGHMENPANANPIRVGVIGCADIAWRRALPALEAEPLTEVTAIASRRWDRAKRFTERFGGEPVEGYPALLERDDVDAVYVPLPAVLHAEWIDRALRAGKHVLAEAPLTTDRPQAERLFAVARERGLLLMENFMFLHHPQHRQVADMLDEGVIGEIRSFAASFTIPPKPQGDIRYQADVGGGALLDIGVYPIRAAGLFLGADLEFVGAVLRHERDRDVVVGGNALLTTRQGVTAQLTFGMEHAYTNNYEFRGSTGRLWMNRVFTPPATYQPVVHIERQDHAEQFVLPAHDQFAKSIRAFAQAVLSGEHPREWSEDSLRQASLVDAVRTGARDIYFP

>3C8GA

SNAMATLTEDDVLEQLDAQDNLFSFMKTAHSILLQGIRQFLPSLFVDNDEEIVEYAVKPLLAQSGPLDDIDVALRLIYALGKMDKWLYADITHFSQYWHYLNEQDETPGFADDITWDFISNVNSITRNATLYDALKAMKFADFAVWSEARFSGMVKTALTLAVTTTLKELTP

>4FMHA

GSHMLEGGVEVLSVVTGEDSITQIELYLNPRMGVNSPDLPTTSNWYTYTYDLQPKGSSPDQPIKENLPAYSVARVSLPMLNEDITCDTLQMWEAISVKTEVVGISSLINVHYWDMKRVHDYGAGIPVSGVNYHMFAIGGEPLDLQGLVLDYQTQYPKTTNGGPITIETVLGRKMTPKNQGLDPQAKAKLDKDGNYPIEVWCPDPSKNENSRYYGSIQTGSQTPTVLQFSNTLTTVLLDENGVGPLCKGDGLFISCADIVGFLFKTSGKMALHGLPRYFNVTLRKRWVKN

>1JX6A

VLNGYWGYQEFLDEFPEQRNLTNALSEAVRAQPVPLSKPTQRPIKISVVYPGQQVSDYWVRNIASFEKRLYKLNINYQLNQVFTRPNADIKQQSLSLMEALKSKSDYLIFTLDTTRHRKFVEHVLDSTNTKLILQNITTPVREWDKHQPFLYVGFDHAEGSRELATEFGKFFPKHTYYSVLYFSEGYISDVRGDTFIHQVNRDNNFELQSAYYTKATKQSGYDAAKASLAKHPDVDFIYACSTDVALGAVDALAELGREDIMINGWGGGSAELDAIQKGDLDITVMRMNDDTGIAMAEAIKWDLEDKPVPTVYSGDFEIVTKADSPERIEALKKRAFRYSDN

>3MVNA

MHHHHHHSSGVDLGTENLYFQSNAQRRLEVKGVVNNITVYDDFAHHPTAITATIDALRAKVGQQRILAVLEPRSNTMKMGVHKHELATSLQDADSVFIYQPPTIEWQVSEVLANLAQPAISADDVDELVMRIVQQAKPNDHILIMSNGAFGGIHQKLLTALAN

>3CJ1A

MAHHHHHHVGTGSNDDDDKSPDPTQDVREPPALKYGIVLDAGSSHTSMFVYKWPADKENDTGIVGQHSSCDVQGGGISSYANDPSKAGQSLVRCLEQALRDVPRDRHASTPLYLGATAGMRLLNLTSPEATARVLEAVTQTLTQYPFDFRGARILSGQDEGVFGWVTANYLLENFIKYGWVGRWIRPRKGTLGAMDLGGASTQITFETTSPSEDPGNEVHLRLYGQHYRVYTHSFLCYGRDQILLRLLASALQIHRFHPCWPKGYSTQVLLQEVYQSPCTMGQRPRAFNGSAIVSLSGTSNATLCRDLVSRLFNISSCPFSQCSFNGVFQPPVAGNFIAFSAFYYTVDFLTTVMGLPVGTLKQLEEATEITCNQTWTELQARVPGQKTRLADYCAVAMFIHQLLSRGYHFDERSFREVVFQKKAADTAVGWALGYMLNLTNLIPADLPGLRKGTHF

>3OT1A

SNAMEQGMSKRILVPVAHGSEEMETVIIVDTLVRAGFQVTMAAVGDKLQVQGSRGVWLTAEQTLEACSAEAFDALALPGGVGGAQAFADSTALLALIDAFSQQGKLVAAICATPALVFAKQQKFVGARMTCHPNFFDHIPSERLSRQRVCYYATQHLLTSQGPGTALEFALAMIALLAGVELAQHVAAPMVLHPQQLTELSGFIDAQS

>4DVCA

SNAAQFKEGEHYQVLKTPASSSPVVSEFFSFYCPHCNTFEPIIAQLKQQLPEGAKFQKNHVSFMGGNMGQAMSKAYATMIALEVEDKMVPVMFNRIHTLRKPPKDEQELRQIFLDEGIDAAKFDAAYNGFAVDSMVHRFDKQFQDSGLTGVPAVVVNNRYLVQGQSAKSLDEYFDLVNYLLTLK

>3KS6A

GMTRIASHRGGTLEFGDSTPHGFTATAAMALEEVEFDLHPTADGAIVVHHDPTLDATTDMTGAIVDMTLAKVKTATIRYGAGSHPMTLEELCALYVDSHVNFRCEIKPGVDGLPYEGFVALVIAGLERHSMLERTTFSSFLLASMDELWKATTRPRLWLVSPSVLQQLGPGAVIETAIAHSIHEIGVHIDTADAGLMAQVQAAGLDFGCWAAHTPSQITKALDLGVKVFTTDRPTLAIALRTEHRMEASV

>2Z5EA

MEDTPLVISKQKTEVVCGVPTQVVCTAFSSHILVVVTQFGKMGTLVSLEPSSVASDVSKPVLTTKVLLGQDEPLIHVFAKNLVAFVSQEAGNRAVLLAVAVKDKSMEGLKALREVIRVCQVW

>1G5AA

SPNSQYLKTRILDIYTPEQRAGIEKSEDWRQFSRRMDTHFPKLMNELDSVYGNNEALLPMLEMLLAQAWQSYSQRNSSLKDIDIARENNPDWILSNKQVGGVCYVDLFAGDLKGLKDKIPYFQELGLTYLHLMPLFKCPEGKSDGGYAVSSYRDVNPALGTIGDLREVIAALHEAGISAVVDFIFNHTSNEHEWAQRCAAGDPLFDNFYYIFPDRRMPDQYDRTLREIFPDQHPGGFSQLEDGRWVWTTFNSFQWDLNYSNPWVFRAMAGEMLFLANLGVDILRMDAVAFIWKQMGTSCENLPQAHALIRAFNAVMRIAAPAVFFKSEAIVHPDQVVQYIGQDECQIGYNPLQMALLWNTLATREVNLLHQALTYRHNLPEHTAWVNYVRSHDDIGWTFADEDAAYLGISGYDHRQFLNRFFVNRFDGSFARGVPFQYNPSTGDCRVSGTAAALVGLAQDDPHAVDRIKLLYSIALSTGGLPLIYLGDEVGTLNDDDWSQDSNKSDDSRWAHRPRYNEALYAQRNDPSTAAGQIYQDLRHMIAVRQSNPRFDGGRLVTFNTNNKHIIGYIRNNALLAFGNFSEYPQTVTAHTLQAMPFKAHDLIGGKTVSLNQDLTLQPYQVMWLEIA

>1YQSA

ADLPAPDDTGLQAVLHTALSQGAPGAMVRVDDNGTIHQLSEGVADRATGRAITTTDRFRVGSVTKSFSAVVLLQLVDEGKLDLDASVNTYLPGLLPDDRITVRQVMSHRSGLYDYTNDMFAQTVPGFESVRNKVFSYQDLITLSLKHGVTNAPGAAYSYSNTNFVVAGMLIEKLTGHSVATEYQNRIFTPLNLTDTFYVHPDTVIPGTHANGYLTPDEAGGALVDSTEQTVSWAQSAGAVISSTQDLDTFFSALMSGQLMSAAQLAQMQQWTTVNSTQGYGLGLRRRDLSCGISVYGHTGTVQGYYTYAFASKDGKRSVTALANTSNNVNVLNTMARTLESAFCGKPTT

>1P3CA

VVIGDDGRTKVANTRVAPYNSIAYITFGGSSCTGTLIAPNKILTNGHCVYNTASRSYSAKGSVYPGMNDSTAVNGSANMTEFYVPSGYINTGASQYDFAVIKTDTNIGNTVGYRSIRQVTNLTGTTIKISGYPGDKMRSTGKVSQWEMSGSVTREDTNLAYYTIDTFSGNSGSAMLDQNQQIVGVHNAGYSNGTINGGPKATAAFVEFINYAKAQ

>2H5NA

MGLGRQSLNIMTFSGQELTAIIKMAKSMVMADGKIKPAEIAVMTREFMRFGILQDQVDLLLKASDSIEASQAVALIARMDEERKKYVASYLGVIMASDGDIDDNELALWTLISTLCGLPTMTVMEAINNMKNL

>3H3IA

GACDNDTEPGGTAVEKMAGDWWVTVNAFIDGKEVEDPFGAGHLQMSTYNTASNSETEMWLDDLGNFWEYKLKVNVNYAARTFSTTGFVDNVTYESKVKITDGKVLEKAATTPSGMPADSIVYMVQFDDDEDGLTYKVSGFRRTGFPADDF

>3GHAA

MNNKTEQGNDAVSGQPSIKGQPVLGKDDAPVTVVEFGDYKCPSCKVFNSDIFPKIQKDFIDKGDVKFSFVNVMFHGKGSRLAALASEEVWKEDPDSFWDFHEKLFEKQPDTEQEWVTPGLLGDLAKSTTKIKPETLKENLDKETFASQVEKDSDLNQKMNIQATPTIYVNDKVIKNFADYDEIKETIEKELKGKLEHHHHHH

>1JZTA

MSTLKVVSSKLAAEIDKELMGPQIGFTLQQLMELAGFSVAQAVCRQFPLRGKTETEKGKHVFVIAGPGNNGGDGLVCARHLKLFGYNPVVFYPKRSERTEFYKQLVHQLNFFKVPVLSQDEGNWLEYLKPEKTLCIVDAIFGFSFKPPMREPFKGIVEELCKVQNIIPIVSVDVPTGWDVDKGPISQPSINPAVLVSLTVPKPCSSHIRENQTTHYVGGRFIPRDFANKFGFEPFGYESTDQILKL

>3ACHA

MRGSHHHHHHRAVVEAPVEHAPIGKATLPSTFEDSTRQGWAWDATSGVQSALTIKDANESKAISWEVKYPEVKPVDGWASAPRIMLGNVNTTRGNNKYLTFDFYLKPTQASKGSLTISLAFAPPSLGFWAQATGDVNIPLSSLSKMKKTTDGLYHFQVKYDLDKINDGKVLTANTVLRDITIVVADGNSDFAGTMYLDNIRFE

>3ER6A

MSLTNKKNLRVVALAPTGRYFASIISSLEILETAAEFAEFQGFMTHVVTPNNRPLIGRGGISVQPTAQWQSFDFTNILIIGSIGDPLESLDKIDPALFDWIRELHLKGSKIVAIDTGIFVVAKAGLLQQNKAVMHSYFAHLFGELFPEIMLMTEQKALIDGNVYLSSGPYSHSSVMLEIVEEYFGKHTRNLGNQFLSTIESEGHHHHHH

>3BO6A

GPPQMSATNEDLKTNFHSLHNQMRQMPMSHFREALDAPDYSGMRQSGFFAMSQGFQLESHGGDVFMHAHRENPQCKGDFAGDKFHISVQREQVPQAFQALSGLLFSVDSPIDKWKVTDMERVDQQSRVAVGAQFTLYVKPDQENSQYSASSLHNTRQFIECLESRLSESGLMPGQYPESDVHPENWKYVSYRNELRSGRDGGEMQSQALREEPFYRLMAE

>1WLGA

GLDVAISQNGFFRLVDSNGSVFYSRNGQFKLDENRNLVNMQGMQLTGYPATGTPPTIQQGANPAPITIPNTLMAAKSTTTASMQINLNSTDPVPSKTPFSVSDADSYNKKGTVTVYDSQGNAHDMNVYFVKTKDNEWAVYTHDSSDPAATAPTTASTTLKFNENGILESGGTVNITTGTINGATAATFSLSFLNSMQQNTGANNIVATNQNGYKPGDLVSYQINNDGTVVGNYSNEQEQVLGQIVLANFANNEGLASQGDNVWAATQASGVALLGTAGSGNFGKLTNGALEASNVDLSK

>2UVOA

ERCGEQGSNMECPNNLCCSQYGYCGMGGDYCGKGCQNGACWTSKRCGSQAGGATCTNNQCCSQYGYCGFGAEYCGAGCQGGPCRADIKCGSQAGGKLCPNNLCCSQWGFCGLGSEFCGGGCQSGACSTDKPCGKDAGGRVCTNNYCCSKWGSCGIGPGYCGAGCQSGGCDG

>3S6FA

GMTQRSLADIQFQTTLEGVTPAQLGGFFEGWPNPPTPETLWRILDRAAVFVLARTPDGQVIGFVNALSDGILAASIPLLEVQAGWRSLGLGSELMRRVLTELGDLYMVDLSCDDDVVPFYERLGLKRANAMFLRRYDNQAGIPAE

>4ADIA

EEAFTYLCTAPGCATQTPVPVRLAGVRFESKIVDGGCFAPWDLEATGACICEIPTDVSCEGLGAWVPTAPCARIWNGTQRACTFWAVNAYSSGGYAQLASYFNPGGSYYKQYHPTACEVEPAFGHSDAACWGFPTDTVMSVFALASYVQHPHKTVRVKFHTETRTVWQLSVAGVSCNVTTEHPFCNTPHGQLEVQVPPDPGDLVEYIMNYTGNQQSRWGLGSPNCHGPDWASPVCQRHSPDCSRLVGATPERPRLRLVDADDPLLRTAPGPGEVWVTPVIGSQARKCGLHIRAGPYGHATVEMPEWIHAHTTSDPWHPPGPLGLKFKTVRPVALPRALAPPRNVRVTGCYQCGTPALVEGLAPGGGNCHLTVNGEDVGAFPPGKFVTAALLNTPPPYQVSCGGESDRASARVIDPAAQSFTGVVYGTHTTAVSETRFEDDDDKAGWSHPQFEKGGGSGGGSGGGSWSHPQFEK

>4A4JA

AQTINLQLEGMDCTSCASSIERAIAKVPGVQSCQVNFALEQAVVSYHGETTPQILTDAVERAGYHARVL

>3N1MC

GSTERPVAGPYITFTDAVNETTIMLKWMYIPASNNNTPIHGFYIYYRPTDSDNDSDYKKDMVEGDKYWHSISHLQPETSYDIKMQCFNEGGESEFSNVMICETKARKSSGQ

>3QUFA

MGSSHHHHHHSSGRENLYFQGHGTAGGGKTKISFYSYFKDNQIGEVVKGFEKKNPDITLDVQYGQDPAQYISTLQTRLAGGKPPTIFNLTMDNRTDVMKSGAALDISGEDFLDGIDDTNFALFQQDGKTYGMPVSAWVGAFFYNKDILKKAGYDKFPKTWDEFIEMGKKINSNGSTAFLEDFNTQIAGSFTGLLASYYGEQGKSGDLDADIWSGKSTFTKDWTPVFKRWEAAAKAGVIPQKSVGLSADQVKQEFVSGNLGVMRSGPWDLPDLQKSDIDFGVAPFPAYSKEDGQWINGGPDQGFAIASRASDKEKAAAKKFLAYLNSEEGLEAFTSAAGTLSLSSKYNAEPPAELKDVVDNYFKQNKFYWVNWPKSPTVMSTEGIAQQQKIVQGQISAKDAAKALDAKWATLKGS

>2CH5A

PQFMAAIYGGVEGGGTRSEVLLVSEDGKILAEADGLSTNHWLIGTDKCVERINEMVNRAKRKAGVDPLVPLRSLGLSLSGGDQEDAGRILIEELRDRFPYLSESYLITTDAAGSIATATPDGGVVLISGTGSNCRLINPDGSESGCGGWGHMMGDEGSAYWIAHQAVKIVFDSIDNLEAAPHDIGYVKQAMFHYFQVPDRLGILTHLYRDFDKCRFAGFCRKIAEGAQQGDPLSRYIFRKAGEMLGRHIVAVLPEIDPVLFQGKIGLPILCVGSVWKSWELLKEGFLLALTQGREIQAQNFFSSFTLMKLRHSSALGGASLGARHIGHLLPMDYSANAIAFYSYTFS

>3NVWC

DTVGRPLPHLAAAMQASGEAVYCDDIPRYENELFLRLVTSTRAHAKIKSIDVSEAQKVPGFVCFLSADDIPGSNETGLFNDETVFAKDTVTCVGHIIGAVVADTPEHAERAAHVVKVTYEDLPAIITIEDAIKNNSFYGSELKIEKGDLKKGFSEADNVVSGELYIGGQDHFYLETHCTIAIPKGEEGEMELFVSTQNAMKTQSFVAKMLGVPVNRILVRVKRMGGGFGGKETRSTLVSVAVALAAYKTGHPVRCMLDRNEDMLITGGRHPFLARYKVGFMKTGTIVALEVDHYSNAGNSRDLSHSIMERALFHMDNCYKIPNIRGTGRLCKTNLSSNTAFRGFGGPQALFIAENWMSEVAVTCGLPAEEVRWKNMYKEGDLTHFNQRLEGFSVPRCWDECLKSSQYYARKSEVDKFNKENCWKKRGLCIIPTKFGISFTVPFLNQAGALIHVYTDGSVLVSHGGTEMGQGLHTKMVQVASKALKIPISKIYISETSTNTVPNSSPTAASVSTDIYGQAVYEACQTILKRLEPFKKKNPDGSWEDWVMAAYQDRVSLSTTGFYRTPNLGYSFETNSGNAFHYFTYGVACSEVEIDCLTGDHKNLRTDIVMDVGSSLNPAIDIGQVEGAFVQGLGLFTLEELHYSPEGSLHTRGPSTYKIPAFGSIPTEFRVSLLRDCPNKKAIYASKAVGEPPLFLGASVFFAIKDAIRAARAQHTNNNTKELFRLDSPATPEKIRNACVDKFTTLCVTGAPGNCK

>3EA0A

SNAKRVFGFVSAKGGDGGSCIAANFAFALSQEPDIHVLAVDISLPFGDLDMYLSGNTHSQDLADISNASDRLDKSLLDTMVQHISPSLDLIPSPATFEKIVNIEPERVSDLIHIAASFYDYIIVDFGASIDHVGVWVLEHLDELCIVTTPSLQSLRRAGQLLKLCKEFEKPISRIEIILNRADTNSRITSDEIEKVIGRPISKRIPQDEDAMQESLLSGQSVLKVAPKSQLSKTIVDWALHLNGV

>1B5EA

MISDSMTVEEIRLHLGLALKEKDFVVDKTGVKTIEIIGASFVADEPFIFGALNDEYIQRELEWYKSKSLFVKDIPGETPKIWQQVASSKGEINSNYGWAIWSEDNYAQYDMCLAELGQNPDSRRGIMIYTRPSMQFDYNKDGMSDFMCTNTVQYLIRDKKINAVVNMRSNDVVFGFRNDYAWQKYVLDKLVSDLNAGDSTRQYKAGSIIWNVGSLHVYSRHFYLVDHWWKTGETHISKKDYVGKYA

>2P51A

GMNSNFSYPALGVDGISSQISPIRDVWSTNLQQEMNLIMSLIERYPVVSMDTEFPGVVARPLGVFKSSDDYHYQTLRANVDSLKIIQIGLALSDEEGNAPVEACTWQFNFTFNLQDDMYAPESIELLTKSGIDFKKHQEVGIEPADFAELLIGSGLVLQEEVTWITFHSGYDFAYLLKAMTQIPLPAEYEEFYKILCIYFPKNYDIKYIMKSVLNNSKGLQDIADDLQIHRIGPQHQAGSDALLTARIFFEIRSRYFDGSIDSRMLNQLYGLGSTGSVLWHNNSSTPQIQFRDLPGAHPSPTPSNAGIPTTLTNTSSAPNFANSTFRFPPRVV

>1OI7A

MILVNRETRVLVQGITGREGQFHTKQMLTYGTKIVAGVTPGKGGMEVLGVPVYDTVKEAVAHHEVDASIIFVPAPAAADAALEAAHAGIPLIVLITEGIPTLDMVRAVEEIKALGSRLIGGNCPGIISAEETKIGIMPGHVFKRGRVGIISRSGTLTYEAAAALSQAGLGTTTTVGIGGDPVIGTTFKDLLPLFNEDPETEAVVLIGEIGGSDEEEAAAWVKDHMKKPVVGFIGGRSAPKGKRMGHAGAIIMGNVGTPESKLRAFAEAGIPVADTIDEIVELVKKALG

>3FPWA

GAGAMSSRKKPSRRTRVLVGGAALAVLGAGVVGTVAANAADTTEATPAAAPVAARGGELTQSTHLTLEAATKAARAAVEAAEKDGRHVSVAVVDRNGNTLVTLRGDGAGPQSYESAERKAFTAVSWNAPTSELAKRLAQAPTLKDIPGTLFLAGGTPVTAKGAPVAGIGVAGAPSGDLDEQYARAGAAVLGH

>1Y43A

EEYSSNWAGAVLIGDGYTKVTGEFTVPSVSAGSSGSSGY

>1HI9A

MKLYMSVDMEGISGLPDDTFVDSGKRNYERGRLIMTEEANYCIAEAFNSGCTEVLVNDSHSKMNNLMVEKLHPEADLISGDVKPFSMVEGLDDTFRGALFLGYHARASTPGVMSHSMIFGVRHFYINDRPVGELGLNAYVAGYYDVPVLMVAGDDRAAKEAEELIPNVTTAAVKQTISRSAVKCLSPAKRGRLLTEKTAFALQNKDKVKPLTPPDRPVLSIEFANYGQAEWANLMPGTEIKTGTTTVQFQAKDMLEAYQAMLVMTELAMRTSFC

>3TIPA

GPHMIAPGHRDEFDPKLPTGEKEEVPGKPGIKNPETGDVVRPPVDSVTKYGPVKGDSIVEKEEIPFEKERKFNPDLAPGTEKVTREGQKGEKTITTPTLKNPLTGEIISKGESKEEITKDPINELTEYGPET

>4KV7A

SNAAPKSLDATPVDAQLLKFGMSTALSGPAAELGINMRHGILAAFDEAKAKNHLPSKTLKLIALDDGYEPARTAPNMHRLTDEHEVLAVVGNVGTPTAITAIPIAQQTKTPFFGAFTGASALRKTESVEFVINYRASYAEETAAMVDALVAKGIKPEEIGFFTQNDSYGDDGFFGGLAAIRRHQSVKVSSLPHGRYRRNTSQVEDGLADLLMHQPLPKAVIMVGTYEPCSKLIRMARMNNFNPQFLAVSFVGADALQRSLGDLANGIVATQVVPHFDSDLPLVREYRDAMRDYDPELPLSFVSLEGYIVGRILVKAVTSIKGEISRSSIAAALEQLGQFDIGLGAPLTLGPNDHQASSKVWPVLIGADSSQSLAWEELLSE

>1LSHB

KYVPQRKPQTSRRHTPASSSSSSSSSSSSSSSSSSSDSDMTVSAESFEKHSKPKVVIVLRAVRADGKQQGLQTTLYYGLTSNGLPKAKIVAVELSDLSVWKLCAKFRLSAHMKAKAAIGWGKNCQQYRAMLEASTGNLQSHPAARVDIKWGRLPSSLQRAKNALLENGAPVIASKLEMEIMPKANQKHQVSVILAAMTPRRMNIIVKLPKVTYFQQGILLPFTFPSPRFWDRPEGSQSDSLPAQIASAFSGIVQDPVASACELNEQSLTTFNGAFFNYDMPESCYHVLAQECSSRPPFIVLIKLDSERRISLELQLDDK

>3F95A

MVNSDSHPLFVRSLAKNMTWQLADTSTQKVLASGASATSGDKQSLLMQSVNLSYQEDGRGFNWRAQAALSLSYLEPTPLDSKFSTGYLELKMRIDKAPEQGANLQVMCSESNCLRDIDFSSFSQLMADKSWHTLAIPLHCDDSDQAEQPITDALRITSQNLSLAIADVALTIKPSDDSISLTCAKLEHHHHHH

>2ZZVA

MKRVSRRAFLRRLGVGVAATAAFSPLAVAQARRYRWRIQTAWDAGTVGYSLFQKFTERVKELTDGQLEVQPFPAGAVVGTFDMFDAVKTGVLDGMNPFTLYWAGRMPVTAFLSSYALGLDRPDQWETWFYSLGGLDIARRAFAEQGLFYVGPVQHDLNIIHSKKPIRRFEDFKGVKLRVPGGMIAEVFAAAGASTVLLPGGEVYPALERGVIDAADFVGPAVNYNLGFHQVAKYIIMGPPETPAIHQPVDLMDFTINLNRWRSLPKPLQERFIAAVHEYSWIHYAGIQKANLEAWPKYRQAGVEVIRLSNEDVRKFRRLAIPIWFKWAKMDKYSREAFASQLEYMKGIGYVTDEELKGLSL

>3FILA

MQYKLILNGKTLKGVLTIEAVDAATAEKVFKQYANDLGVDGEWTYDDATKTFTVTE

>2GUDA

XSLTHRKFGGSGGSPFSGLSSIAVRSGSYLDAIIIDGVHHGGSGGNLSPTFTFGSGEYISNMTIRSGDYIDNISFETNMGRRFGPYGGSGGSANTLSNVKVIQINGSAGDYLDSLDIYYEQY

>3P0YA

RKVCNGIGIGEFKDSLSINATNIKHFKNCTSISGDLHILPVAFRGDSFTHTPPLDPQELDILKTVKEITGFLLIQAWPENRTDLHAFENLEIIRGRTKQHGQFSLAVVSLNITSLGLRSLKEISDGDVIISGNKNLCYANTINWKKLFGTSGQKTKIISNRGENSCKATGQVCHALCSPEGCWGPEPRDCVSCRNVSRGRECVDKGNSHHHHHH

>3VE2A

GSKDQGGYGFAMRLKRRNWYPGAEESEVKLNESDWEATGLPTKPKELPKRQKSVIEKVETDGDSDIYSSPYLTPSNHQNGSAGNGVNQPKNQATGHENFQYVYSGWFYKHAASEKDFSNKKIKSGDDGYIFYHGEKPSRQLPASGKVIYKGVWHFVTDTKKGQDFREIIQPSKKQGDRYSGFSGDGSEEYSNKNESTLKDDHEGYGFTSNLEVDFGNKKLTGKLIRNNASLNNNTNNDKHTTQYYSLDAQITGNRFNGTATATDKKENETKLHPFVSDSSSLSGGFFGPQGEELGFRFLSDDQKVAVVGSAKTKDKLENGAAASGSTGAAASGGAAGTSSENSKLTTVLDAVELTLNDKKIKNLDNFSNAAQLVVDGIMIPLLPKDSESGNTQADKGKNGGTEFTRKFEHTPESDKKDAQAGTQTNGAQTASNTAGDTNGKTKTYEVEVCCSNLNYLKYGMLTRKNSKSAMQAGGNSSQADAKTEQVEQSMFLQGERTDEKEIPTDQNVVYRGSWYGHIANGTSWSGNASDKEGGNRAEFTVNFADKKITGKLTAENRQAQTFTIEGMIQGNGFEGTAKTAESGFDLDQKNTTRTPKAYITDAKVKGGFYGPKAEELGGWFAYPGDKQTEKATATSSDGNSASSATVVFGAKRQQPVQ

>4A27A

SMEMRAVVLAGFGGLNKLRLFRKAMPEPQDGELKIRVKACGLNFIDLMVRQGNIDNPPKTPLVPGFECSGIVEALGDSVKGYEIGDRVMAFVNYNAWAEVVCTPVEFVYKIPDDMSFSEAAAFPMNFVTAYVMLFEVANLREGMSVLVHSAGGGVGQAVAQLCSTVPNVTVFGTASTFKHEAIKDSVTHLFDRNADYVQEVKRISAEGVDIVLDCLCGDNTGKGLSLLKPLGTYILYGSSNMVTGETKSFFSFAKSWWQVEKVNPIKLYEENKVIAGFSLLNLLFKQGRAGLIRGVVEKLIGLYNQKKIKPVVDSLWALEEVKEAMQRIHDRGNIGKLILDVEKTPTPL

>3MMHA

MHALHFSASDKAALYREVLPQIESVVADETDWVANLANTAAVLKEAFGWFWVGFYLVDTRSDELVLAPFQGPLACTRIPFGRGVCGQAWAKGGTVVVGDVDAHPDHIACSSLSRSEIVVPLFSDGRCIGVLDADSEHLAQFDETDALYLGELAKILEKRFEASRQAV

>2IZWA

ARKKGKSASQVIVLKEKSRKKRQKSRGQQPTRQVTPVSAPAAMGTQITYRGPQVVTQYGDITPAKNSGSLVRVTSSATAGTEVSGTVLFNVRNATELPWLSGQGSRYSKYRVRYAHFTWEPIVGSNTNGEVAMAMLYDVADVTSITIERLMQTRGGTWGPIWSPTRKRLSYDPEHASLPWYLSGVSSGAAAGNIQTPFQIAWAAQSSLVSTTLGRIMAEYLVELTDPVDVTINQ

>3DXTA

METMKSKANCAQNPNCNIMIFHPTKEEFNDFDKYIAYMESQGAHRAGLAKIIPPKEWKARETYDNISEILIATPLQQVASGRAGVFTQYHKKKKAMTVGEYRHLANSKKYQTPPHQNFEDLERKYWKNRIYNSPIYGADISGSLFDENTKQWNLGHLGTIQDLLEKECGVVIEGVNTPYLYFGMWKTTFAWHTEDMDLYSINYLHLGEPKTWYVVPPEHGQRLERLARELFPGSSRGCGAFLRHKVALISPTVLKENGIPFNRITQEAGEFMVTFPYGYHAGFNHGFNCAEAINFATPRWIDYGKMASQCSCGEARVTFSMDAFVRILQPERYDLWKRGQDRAVVDHMEPRVPA

>2OL5A

MYIPKHFAVNDPDVAYQVIEENSFATLVSMHQRELFATHLPLLLDREKTCLYGHFARSNPQWNDIQHQTVLAIFHGPHCYISPSWYETNQAVPTWNYVAVHVYGNVELINDQGEVMQSLHDMVEKYEAPGSRYQLSEVDAGMLSGMNKGIQAFKIIIKRIEGKAKLSQNHPAHRQERIIKQLEQMPFENEKRIASLMKKQRQ

>4HTGA

CVAVEQKTRTAIIRIGTRGSPLALAQAYETREKLKKKHPELVEDGAIHIEIIKTTGDKILSQPLADIGGKGLFTKEIDEALINGHIDIAVHSMKDVPTYLPEKTILPCNLPREDVRDAFICLTAATLAELPAGSVVGTASLRRKSQILHKYPALHVEENFRGNVQTRLSKLQGGKVQATLLALAGLKRLSMTENVASILSLDEMLPAVAQGAIGIACRTDDDKMATYLASLNHEETRLAISCERAFLETLDGSCRTPIAGYASKDEEGNCIFRGLVASPDGTKVLETSRKGPYVYEDMVKMGKDAGQELLSRAGPGFFGN

>4I0XA

SIDEVGALSKFAASLADQMRAGSNSLDRDVQSLFGVWKGSAADAYRSGWDEMQDGATKVWNALTDIASTLGSNAAAFHAQETSTASSITSTQAD

>1KJNA

MKTESTGKALMVLGCPESPVQIPLAIYTSHKLKKKGFRVTVTANPAALRLVQVADPEGIYTDEMVDLESCINELAEGDYEFLAGFVPNDAAAAYLVTFAGILNTETLAIIFDRDADVLEELVNEIMETLDAEIIAARAHHNPAPLRVRIDRFMEEKP

>3VGIA

MSKKKFVIVSILTILLVQAIYFVEKYHTSEDKSTSNTSSTPPQTTLSTTKVLKIRYPDDGEWPGAPIDKDGDGNPEFYIEINLWNILNATGFAEMTYNLTSGVLHYVQQLDNIVLRDRSNWVHGYPEIFYGNKPWNANYATDGPIPLPSKVSNLTDFYLTISYKLEPKNGLPINFAIESWLTREAWRTTGINSDEQEVMIWIYYDGLQPAGSKVKEIVVPIIVNGTPVNATFEVWKANIGWEYVAFRIKTPIKEGTVTIPYGAFISVAANISSLPNYTELYLEDVEIGTEFGTPSTTSAHLEWWITNITLTPLDRPLIS

>3GYCA

GAKSEMAFAGKGEISPRAITMWDFSWLERRWPGAGYEDWDQVLDELSERGYNAIRIDAYPHLIAENPMKKWLLKEVWNQQDWGSPDMNEVQVQPNLNLFLSKCKERDIKVGLSSWYRLDVDEVCLKLDTPEKLADCWLTILRSIEEDGLLDTILYVDLCNEWPGDSWAPFFAKTYPNVGWGNWYKEESLRWMKTSLEKMRQVYPDMPFLYSFDHGDVKKYEEVDCSFLDLYEHHIWMAQQNGGEFYKLVGYGYNRFLPDDYKNVVKNAERVYRERPGYWQKLLTDKIELMASVARKNRRPLVTTECWGLVDYKDWPLLKWDWVKDLCELGTITAARTGMWVGVATSNFCGPQFAGMWRDVEWHKRLTSIIRSSPLDESLTKNNEVAAKLLKRL

>3FO5A

SMRPQPGDGERRYREASARKKIRLDRKYIVSCKQTEVPLSVPWDPSNQVYLSYNNVSSLKMLVAKDNWVLSSEISQVRLYTLEDDKFLSFHMEMVVHVDAAQAFLLLSDLRQRPEWDKHYRSVELVQQVDEDDAIYHVTSPALGGHTKPQDFVILASRRKPCDNGDPYVIALRSVTLPTHRETPEYRRGETLCSGFCLWREGDQLTKVSYYNQATPGVLNYVTTNVAGLSSEFYTTFKACEQFLLDNRNDLAPSLQTL

>2YFRA

MDVKQVEKKDSVDKTNAEENKDSSVKPAENATKAELKGQVKDIVEESGVDTSKLTNDQINELNKINFSKEAKSGTQLTYNDFKKIAKTLIEQDARYAIPFFNASKIKNMPAAKTLDAQSGKVEDLEIWDSWPVQDAKTGYVSNWNGYQLVIGMMGVPNVNDNHIYLLYNKYGDNDFNHWKNAGPIFGLGTPVIQQWSGSATLNKDGSIQLYYTKVDTSDNNTNHQKLASATVYLNLEKDQDKISIAHVDNDHIVFEGDGYHYQTYDQWKETNKGADNIAMRDAHVIDDDNGNRYLVFEASTGTENYQGDDQIYQWLNYGGTNKDNLGDFFQILSNSDIKDRAKWSNAAIGIIKLNDDVKNPSVAKVYSPLISAPMVSDEIERPDVVKLGNKYYLFAATRLNRGSNDDAWMATNKAVGDNVAMIGYVSDNLTHGYVPLNESGVVLTASVPANWRTATYSYYAVPVEGRDDQLLITSYITNRGEVAGKGMHATWAPSFLLQINPDNTTTVLAKMTNQGDWIWDDSSENPDMMGVLEKDAPNSAALPGEWGKPVDWDLIGGYNLKPHQHHHHHH

>1XCRA

GSACAEFSFHVPSLEELAGVMQKGLKDNFADVQVSVVDCPDLTKEPFTFPVKGICGKTRIAEVGGVPYLLPLVNQKKVYDLNKIAKEIKLPGAFILGAGAGPFQTLGFNSEFMPVIQTESEHKPPVNGSYFAHVNPADGGCLLEKYSEKCHDFQCALLANLFASEGQPGKVIEVKAKRRTGPLNFVTCMRETLEKHYGNKPIGMGGTFIIQKGKVKSHIMPAEFSSCPLNSDEEVNKWLHFYEMKAPLVCLPVFVSRDPGFDLRLEHTHFFSRHGEGGHYHYDTTPDIVEYLGYFLPAEFLYRIDQPKETHSIGRD

>3V7NA

GPGSMNYISTRGAGIGERHTFSDILLGGLAKDGGLYLPSEYPQVSADELARWRTLPYADLAFEILSKFCDDIAAADLRAITRRTYTADVYRHARRGGNAADITPLTTLGTENGAPVSLLELSNGPTLAFKDMAMQLLGNLFEYTLAKHGETLNILGATSGDTGSAAEYAMRGKEGVRVFMLSPHKKMSAFQTAQMYSLQDPNIFNLAVNGVFDDCQDIVKAVSNDHAFKAQQKIGTVNSINWARVVAQVVYYFKGYFAATRSNDERVSFTVPSGNFGNVCAGHIARMMGLPIEKLVVATNENDVLDEFFRTGAYRVRSAQDTYHTSSPSMDISKASNFERFVFDLLGRDPARVVQLFRDVEQKGGFDLAASGDFARVAEFGFVSGRSTHADRIATIRDVFERYRTMIDTHTADGLKVAREHLRPGVPMVVLETAQPIKFGESIREALGQEPSRPAAFDGLEALPQRFEVVDANAQQVKDFIAAHTGA

>3EKIA

MLKKLKNFILFSSIFSPIAFAISCSNTGVVKQEDVSVSQGQWDKSITFGVSEAWLNKKKGGEKVNKEVINTFLENFKKEFNKLKNANDKTKNFDDVDFKVTPIQDFTVLLNNLSTDNPELDFGINASGKLVEFLKNNPGIITPALETTTNSFVFDKEKDKFYVDGTDSDPLVKIAKEINKIFVETPYASWTDENHKWNGNVYQSVYDPTVQANFYRGMIWIKGNDETLAKIKKAWNDKDWNTFRNFGILHGKDNSSSKFKLEETILKNHFQNKFTTLNEDRSAHPNAYKQKSADTLGTLDDFHIAFSEEGSFAWTHNKSATKPFETKANEKMEALIVTNPIPYDVGVFRKSVNQLEQNLIVQTFINLAKNKQDTYGPLLGYNGYKKIDNFQKEIVEVYEKAIK

>2P26A

QECTKFKVSSCRECIESGPGCTWCQKLNFTGPGDPDSIRCDTRPQLLMRGCAADDIMDPTSLAETQEDHNGGQKQLSPQKVTLYLRPGQAAAFNVTFRRAKLSSRVFLDHNALPDTLKVTYDSFCSNGVTHRNQPRGDCDGVQINVPITFQVKVTATECIQEQSFVIRALGFTDIVTVQVLPQCECRCRDQSRDRSLCHGKGFLECGICRCDTGYIGKNCECQTQGRSSQELEGSCRKDNNSIICSGLGDCVCGQCLCHTSDVPGKLIYGQYCEHHHHHH

>1MUWA

SYQPTPEDRFTFGLWTVGWQGRDPFGDATRPALDPVETVQRLAELGAHGVTFHDDDLIPFGSSDTERESHIKRFRQALDATGMTVPMATTNLFTHPVFKDGGFTANDRDVRRYALRKTIRNIDLAVELGAKTYVAWGGREGAESGAAKDVRVALDRMKEAFDLLGEYVTSQGYDIRFAIEPKPNEPRGDILLPTVGHALAFIERLERPELYGVNPEVGHEQMAGLNFPHGIAQALWAGKLFHIDLNGQSGIKYDQDLRFGAGDLRAAFWLVDLLESAGYEGPRHFDFKPPRTEDIDGVWASAAGCMRNYLILKERAAAFRADPEVQEALRASRLDELAQPTAADGVQELLADRTAFEDFDVDAAAARGMAFERLDQLAMDHLLGAR

>4HNOA

GSHMIKIGAHMPISKGFDRVPQDTVNIGGNSFQIFPHNARSWSAKLPSDEAATKFKREMKKHGIDWENAFCHSGYLINLASPKDDIWQKSVELLKKEVEICRKLGIRYLNIHPGSHLGTGEEEGIDRIVRGLNEVLNNTEGVVILLENVSQKGGNIGYKLEQLKKIRDLVDQRDRVAITYDTCHGFDSGYDITKKEGVEALLNEIESLFGLERLKMIHLNDSKYPLGAAKDRHERIGSGFIGEEGFAVFFSFKEIQEVPWILETPGGNEEHAEDIKKVFEIIEKFGIE

>3HRZA

ALYTLITPAVLRTDTEEQILVEAHGDSTPKQLDIFVHDFPRKQKTLFQTRVDMNPAGGMLVTPTIEIPAKEVSTDSRQNQYVVVQVTGPQVRLEKVVLLSYQSSFLFIQTDKGIYTPGSPVLYRVFSMDHNTSKMNKTVIVEFQTPEGILVSSNSVDLNFFWPYNLPDLVSLGTWRIVAKYEHSPENYTAYFDVRKYVLPSFEVRLQPSEKFFYIDGNENFHVSITARYLYGEEVEGVAFVLFGVKIDDAKKSIPDSLTRIPIIDGDGKATLKRDTFRSRFPNLNELVGHTLYASVTVMTESGSDMVVTEQSGIHIVASPYQIHFTKTPKYFKPGMPYELTVYVTNPDGSPAAHVPVVSEAFHSMGTTLSDGTAKLILNIPLNAQSLPITVRTNHGDLPRERQATKSMTAIAYQTQGGSGNYLHVAITSTEIKPGDNLPVNFNVKGNANSLKQIKYFTYLILNKGKIFKVGRQPRRDGQNLVTMNLHITPDLIPSFRFVAYYQVGNNEIVADSVWVDVKDTCMGTLVVKGDNLIQMPGAAMKIKLEGDPGARVGLVAVDKAVYVLNDKYKISQAKIWDTIEKSDFGCTAGSGQNNLGVFEDAGLALTTSTNLNTKQRSAAKCPQPAN

>3VSSA

QSGLQDGPEPTIHTQQAYAPEDDFTAKWTRADARQLQRMSDPTAPSRENSMPASVTMPTVPQDFPDMSNEQVWVWDTWPLTDEDANQYSVNGWEIIFSLVADRNLGFDDRHVFAKIGYFYRPAGVPAAERPENGGWTYGGLVFKEGVTGQIFEDQSFSHQTQWSGSARVSKNGEIKLFFTDVAFYRNSDGTNIKPYDPRIALSVGKVKANKKGVTLTGFNKVTDLLQADGTYYQTGAQNEFFNFRDPFTFEDPAHPGETFMVFEGNSAMQRETATCNEADLGYRQGDPYAETVDDVNASGATYQIGNVGLAKAKNKQLTEWEFLPPILSANCVTDQTERPQIYFKDGKSYLFTISHRGTFAAGLDGPEGVYGFVGDGIRSDYQPLNGGSGLALGNPTNLNFLGGQPFAPDFNQHPGHFQAYSHYVMPGGLVQSFIDTIGTHDDFVRGGTLAPTVKMDIGVGGDPTKTAVDYSYGSEGLGGWADIPANKHLFTNGKF

>1JNIA

DAPAVGKDLTQAAENIPPAFHNAPRQGELPALNYVNQPPMVPHSVANYQVTKNVNQCLNCHSPENSRLSGATRISPTHFMDRDGKVGSSSSPRRYFCLQCHVSQANVDPIVPNDFKPMKGYGN

>3RFRC

MSSTTSTAAGAAAEVESVVDLRGMWIGLAVLNVFYLIVRIYEQVFGWRAGLDSFAPEFQTYWMSILWTEIPLELVSGLGLAGYLWKTRDRNVDAVAPREEMRRLVVLVQWLVVYGIAIYWGASFFTEQDGAWHMTVIRDTDFTPSHIIEFYMSYPIYSVIAVGAFFYAKTRIPYFAHGYSLAFLIVAIGPFMIIPNVGLNEWGHTFWFMEELFVAPLHWGFVFFGWMALGVFGVVLQILGRIHALIGKEGVALLTE

>4EG0A

GPGSMSGIDPKRFGKVAVLFGGESAEREVSLTSGRLVLQGLRDAGIDAHPFDPAERPLSALKDEGFVRAFNALHGGYGENGQIQGALDFYGIRYTGSGVLGSALGLDKFRTKLVWQQTGVPTPPFETVMRGDDYAARATDIVAKLGLPLFVKPASEGSSVAVLKVKTADALPAALSEAATHDKIVIVEKSIEGGGEYTACIAGDLDLPLIKIVPAGEFYDYHAKYVANDTQYLIPCGLPAEQETELKRIARRAFDVLGCTDWGRADFMLDAAGNAYFLEVNTAPGMTDHSLPPKAARSIGIGYSELVVKVLSLTLND

>3VL9A

GPLGSASLQRRSDFCGQWDTATAGDFTLYNDLWGESAGTGSQCTGVDSYSGDTIAWHTSWSWSGGSSSVKSYVNAALTFTPTQLNCISSIPTTWKWSYSGSSIVADVAYDTFLAETASGSSKYEIMVWLAALGGAGPISSTGSTIATPTIAGVNWKLYSGPNGDTTVYSFVADSTTESFSGDLNDFFTYLVDNEGVSDELYLTTLEAGTEPFTGSNAKLTVSEYSISIE

>3K8GA

GSGAWKASVDPLGVVGSGADVYLYFPVAGNENLISRIIENHESKADIKKIVDRTTAVYGAFFARSKEFRLFGSGSYPYAFTNLIFSRSDGWASTKTEHGITYYESEHTDVSIPAPHFSCVIFGSSKRERMSKMLSRLVNPDRPQLPPRFEKECTSEGTSQTVALYIKNGGHFITKLLNFPQLNLPLGAMELYLTARRNEYLYTLSLQLGNAKINFPIQFLISRVLNAHIHVEGDRLIIEDGTISAERLASVISSLYSKKGSS

>4DD5A

MGVMNMREVVIASAARTAVGSFGGAFKSVSAVELGVTAAKEAIKRANITPDMIDESLLGGVLTAGLGQNIARQIALGAGIPVEKPAMTINIVCGSGLRSVSMASQLIALGDADIMLVGGAENMSMSPYLVPSARYGARMGDAAFVDSMIKDGLSDIFNNYHMGITAENIAEQWNITREEQDELALASQNKAEKAQAEGKFDEEIVPVVIKGRKGDTVVDKDEYIKPGTTMEKLAKLRPAFKKDGTVTAGNASGINDGAAMLVVMAKEKAEELGIEPLATIVSYGTAGVDPKIMGYGPVPATKKALEAANMTIEDIDLVEANEAFAAQSVAVIRDLNIDMNKVNVNGGAIAIGHPIGCSGARILTTLLYEMKRRDAKTGLATLCIGGGMGTTLIVKR

>3LMBA

MNASLTPDQVSKKLKQFFSDHLPISQFMGLEIESYDGDTLILTAPLEPNINDKQTAFGGSLYNAAVMACWGMVYLKTQEENIACNQVVTEGNMKYIAPVYGRIRAICHAPDEEELANFFDHFERKGKARISLEAAIYNDACVMKIEPETKPSVKFNGQYAILKNQ

>1NP6A

AGKTMIPLLAFAAWSGTGKTTLLKKLIPALCARGIRPGLIKHTHHDMDVDKPGKDSYELRKAGAAQTIVASQQRWALMTETPDEEELDLQFLASRMDTSKLDLILVEGFKHEEIAKIVLFRDGAGHRPEELVIDRHVIAVASDVPLNLDVALLDINDVEGLADFVVEWMQKQNG

>3RONA

MENLNHCPLEDIKVNPWKTPQSTARVITLRVEDPNEINNLLSINEIDNPNYILQAIMLANAFQNALVPTSTDFGDALRFSMPKGLEIANTITPMGAVVSYVDQNVTQTNNQVSVMINKVLEVLKTVLGVALSGSVIDQLTAAVTNTFTNLNTQKNEAWIFWGKETANQTNYTYNVLFAIQNAQTGGVMYCVPVGFEIKVSAVKEQVLFFTIQDSASYNVNIQSLKFAQPLVSSSQYPIADLTSAINGTL

>2Q3ZA

MAEELVLERCDLELETNGRDHHTADLCREKLVVRRGQPFWLTLHFEGRNYQASVDSLTFSVVTGPAPSQEAGTKARFPLRDAVEEGDWTATVVDQQDCTLSLQLTTPANAPIGLYRLSLEASTGYQGSSFVLGHFILLFNAWCPADAVYLDSEEERQEYVLTQQGFIYQGSAKFIKNIPWNFGQFQDGILDICLILLDVNPKFLKNAGRDCSRRSSPVYVGRVGSGMVNCNDDQGVLLGRWDNNYGDGVSPMSWIGSVDILRRWKNHGCQRVKYGQCWVFAAVACTVLRCLGIPTRVVTNYNSAHDQNSNLLIEYFRNEFGEIQGDKSEMIWNFHCWVESWMTRPDLQPGYEGWQALDPTPQEKSEGTYCCGPVPVRAIKEGDLSTKYDAPFVFAEVNADVVDWIQQDDGSVHKSINRSLIVGLKISTKSVGRDEREDITHTYKYPEGSSEEREAFTRANHLNKLAEKEETGMAMRIRVGQSMNMGSDFDVFAHITNNTAEEYVCRLLLCARTVSYNGILGPECGTKYLLNLTLEPFSEKSVPLCILYEKYRDCLTESNLIKVRALLVEPVINSYLLAERDLYLENPEIKIRILGEPKQKRKLVAEVSLQNPLPVALEGCTFTVEGAGLTEEQKTVEIPDPVEAGEEVKVRMDLVPLHMGLHKLVVNFESDKLKAVKGFRNVIIGPA

>2XFRA

MEVNVKGNYVQVYVMLPLDAVSVNNRFEKGDELRAQLRKLVEAGVDGVMVDVWWGLVEGKGPKAYDWSAYKQLFELVQKAGLKLQAIMSFHQCGGNVGDAVNIPIPQWVRDVGTRDPDIFYTDGHGTRNIEYLTLGVDNQPLFHGRSAVQMYADYMTSFRENMKEFLDAGVIVDIEVGLGPAGEMRYPSYPQSHGWSFPGIGEFICYDKYLQADFKAAAAAVGHPEWEFPNDVGQYNDTPERTQFFRDNGTYLSEKGRFFLAWYSNNLIKHGDRILDEANKVFLGYKVQLAIKISGIHWWYKVPSHAAELTAGYYNLHDRDGYRTIARMLKRHRASINFTCAEMRDSEQSSQAMSAPEELVQQVLSAGWREGLNVACENALPRYDPTAYNTILRNARPHGINQSGPPEHKLFGFTYLRLSNQLVEGQNYANFKTFVDRMHANLPRDPYVDPMAPLPRSGPEISIEMILQAAQPKLQPFPFQEHTDLPVGPTGGMGGQAEGPTCGMGGQVKGPTGGMGGQAEDPTSGIGGELPATM

>1T2DA

MAPKAKIVLVGSGMIGGVMATLIVQKNLGDVVLFDIVKNMPHGKALDTSHTNVMAYSNCKVSGSNTYDDLAGADVVIVTAGFTKAPGKSDKEWNRDDLLPLNNKIMIEIGGHIKKNCPNAFIIVVTNPVDVMVQLLHQHSGVPKNKIIGLGGVLDTSRLKYYISQKLNVCPRDVNAHIVGAHGNKMVLLKRYITVGGIPLQEFINNKLISDAELEAIFDRTVNTALEIVNLHASPYVAPAAAIIEMAESYLKDLKKVLICSTLLEGQYGHSDIFGGTPVVLGANGVEQVIELQLNSEEKAKFDEAIAETKRMKALAHHHHHH

>3AQ2A

GSHMTVPTWQVRDLRRILRVSELSQHLRQARTDFRSTLSQLVYFNRSVVNPNEYDDEYLLSDQRLTYVYVDEVTAQLCGLNRLLPSNSPAFGTVATAMPPWLLDPQEMNAILQQSCGQGGFVNYHHGPSTNGFFLAILMSQLFIRIRTDVIRGQGYGWYARQGNYVEEGEDNEGIENEEEEEETREFQLSDLIHYPIVALGSCHLTR

>3OSXA

MRGSHHHHHHGSEGMQFDRGYLSPYFINKPESGSVELENPYILLVDKKISNIRELLPVLEGVAKASKPLVIIAEDVEGEALATLVVNNMRGIVKVASVKAPGFGDRRKAMLQDIATLTNGTVISEEIGLELEKATLEDLGQAKRVVINKDTTTIIDGVGEEGAIAARVTQIRQQIEESTSDYDREKLQERVAKLAGGVKLN

>3H8DE

GSSSGGGSSSSGTSSAFSSYFNNKVGIPQEHVDHDDFDANQLLNKINE

>2FHZA

MTNKLFEHTVLYDSGDAFFELKGNASMKLSPKAAIEVCNEAAKKGLWILGIDGGHWLNPGFRIDSSASWTYDMPEEYKSKIPENNRLAIENIKDDIENGYTAFIITLKM

>2YK4A

EPVNLIFCYTILQMKVAERIMAQHPGERFYVVLMSENRNEKYDYYFNQIKDKAEWAYFFHLPYGLNKSFNFIPTMAELKVKAMLLPKVKRIYLASLEKVSIAAFLSTYPDAEIKTFDDGTINLIQSSSYLGDEFSVNGTIKRNFARMMIGDWSIAKTRNASDEHYTIFKGLKNIMDDGRRKMTYLPLFDASELKAGDETGGTVRILLGSPDKEMKEISEKAAKNFNIQYVAPHPRQTYGLSGVTTLNSPYVIEDYILREIKKNPHTRYEIYTFFSGAALTMKDFPNVHVYALKPASLPEDYWLKPVYALFTQSGIPILTFDDKLVPR

>3COVA

AMAIPAFHPGELNVYSAPGDVADVSRALRLTGRRVMLVPTMGALHEGHLALVRAAKRVPGSVVVVSIFVNPMQFGAGGDLDAYPRTPDDDLAQLRAEGVEIAFTPTTAAMYPDGLRTTVQPGPLAAELEGGPRPTHFAGVLTVVLKLLQIVRPDRVFFGEKDYQQLVLIRQLVADFNLDVAVVGVPTVREADGLAMSSRNRYLDPAQRAAAVALSAALTAAAHAATAGAQAALDAARAVLDAAPGVAVDYLELRDIGLGPMPLNGSGRLLVAARLGTTRLLDNIAIEIGTFAGTDRPDGYR

>3CNYA

GMSSKAEKDIKWGIAPIGWRNDDIPSIGKDNNLQQLLSDIVVAGFQGTEVGGFFPGPEKLNYELKLRNLEIAGQWFSSYIIRDGIEKASEAFEKHCQYLKAINAPVAVVSEQTYTIQRSDTANIFKDKPYFTDKEWDEVCKGLNHYGEIAAKYGLKVAYHHHMGTGIQTKEETDRLMANTDPKLVGLLYDTGHIAVSDGDYMALLNAHIDRVVHVHFKDVRRSKEEECRAKGLTFQGSFLNGMFTVPGDGDLDFKPVYDKLIANNYKGWIVVEAEQDPSKANPLEMAQIAHRYIKQHLIEN

>2JKHL

RKLCSLDNGDCDQFCHEEQNSVVCSCARGYTLADNGKACIPTGPYPCGKQTLERR

>3QPZA

MDIKINDITLGNNSPFVLFGGINVLESLDSTLQTCAHYVEVTRKLGIPYIFKASFDKAARSSIHSYRGVGLEEGLKIFEKVKAEFGIPVITDVHEPHQCQPVAEVCDVIQLPAFLARQTDLVVAMAKTGNVVNIKKPQFLSPSQMKNIVEKFHEAGNGKLILCERGSSFGYDNLVVDMLGFGVMKQTCGNLPVIFDVTHSLQTRDAGSAASGGRRAQALDLALAGMATRLAGLFLESHPDPKLAKCDGPSALPLHLLEDFLIRIKALDDLIKSQPILTIE

>2PXXA

GSGYREVEYWDQRYQGAADSAPYDWFGDFSSFRALLEPELRPEDRILVLGCGNSALSYELFLGGFPNVTSVDYSSVVVAAMQACYAHVPQLRWETMDVRKLDFPSASFDVVLEKGTLDALLAGERDPWTVSSEGVHTVDQVLSEVSRVLVPGGRFISMTSAAPHFRTRHYAQAYYGWSLRHATYGSGFHFHLYLMHKGGKLSVAQLALGAQILSP

>2E6FA

MMCLKLNLLDHVFANPFMNAAGVLCSTEEDLRCMTASSSGALVSKSCTSAPRDGNPEPRYMAFPLGSINSMGLPNLGFDFYLKYASDLHDYSKKPLFLSISGLSVEENVAMVRRLAPVAQEKGVLLELNLSCPNVPGKPQVAYDFEAMRTYLQQVSLAYGLPFGVKMPPYFDIAHFDTAAAVLNEFPLVKFVTCVNSVGNGLVIDAESESVVIKPKQGFGGLGGKYILPTALANVNAFYRRCPDKLVFGCGGVYSGEDAFLHILAGASMVQVGTALQEEGPGIFTRLEDELLEIMARKGYRTLEEFRGRVKTIE

>1V18B

LPDADTLLHFATESTPDGFSCSSSLSALSLDEPFIQKDVELRIMPPV

>3VL9A

GPLGSASLQRRSDFCGQWDTATAGDFTLYNDLWGESAGTGSQCTGVDSYSGDTIAWHTSWSWSGGSSSVKSYVNAALTFTPTQLNCISSIPTTWKWSYSGSSIVADVAYDTFLAETASGSSKYEIMVWLAALGGAGPISSTGSTIATPTIAGVNWKLYSGPNGDTTVYSFVADSTTESFSGDLNDFFTYLVDNEGVSDELYLTTLEAGTEPFTGSNAKLTVSEYSISIE

>2CWRA

GPTTPVPVSGSLEVKVNDWGSGAEYDVTLNLDGQYDWTVKVKLAPGATVGSFWSANKQEGNGYVIFTPVSWNKGPTATFGFIVNGPQGDKVEEITLEINGQVI

>2H21A

SLSPAVQTFWKWLQEEGVITAKTPVKASVVTEGLGLVALKDISRNDVILQVPKRLWINPDAVAASEIGRVCSELKPWLSVILFLIRERSREDSVWKHYFGILPQETDSTIYWSEEELQELQGSQLLKTTVSVKEYVKNECLKLEQEIILPNKRLFPDPVTLDDFFWAFGILRSRAFSRLRNENLVVVPMADLINHSAGVTTEDHAYEVKGAAGLFSWDYLFSLKSPLSVKAGEQVYIQYDLNKSNAELALDYGFIEPNENRHAYTLTLEISESDPFFDDKLDVAESNGFAQTAYFDIFYNRTLPPGLLPYLRLVALGGTDAFLLESLFRDTIWGHLELSVSRDNEELLCKAVREACKSALAGYHTTIEQDRELKEGNLDSRLAIAVGIREGEKMVLQQIDGIFEQKELELDQLEYYQERRLKDLGLCGENGDILENLYFQ

>1WSWA

MAKALIVYGSTTGNTEYTAETIARELADAGYEVDSRDAASVEAGGLFEGFDLVLLGCSTWGDDCIELQDDFIPLFDSLEETGAQGRKVACFGCGDSSYEYFCGAVDAIEEKLKNLGAEIVQDGLRIDGDPRAARDDIVGWAHDVRGAI

>2RA9A

GQHTLKQFAADSALTTTTPLCSEVPLFDINALGDWTYLGTSLPAKFAKLFASILHCIDDEYFLITPVEKVRVQVEDAPLLIVDFERAQPHSLLNVSTSIGTLHHNVDIKQMKLTDDSVYLPLERGLWGKLGRACYYNFVNEFNLSDLNEQ

>1GWYA

ALAGTIIAGASLTFQVLDKVLEELGKVSRKIAVGIDNESGGTWTALNAYFRSGTTDVILPEFVPNTKALLYSGRKDTGPVATGAVAAFAYYMSSGNTLGVMFSVPFDYNWYSNWWDVKIYSGKRRADQGMYEDLYYGNPYRGDNGWHEKNLGYGLRMKGIMTSAGEAKMQIKISR

>1PAQA

MSVNSIYTDREEIDSEFEDEDFEKEGIATVERAMENNHDLDTALLELNTLRMSMNVTYHEVRIATITALLRRVYHFIATQTLGPKDAVVKVFNQWGLLFKRQAFDEEEYIDLMNIIMEKIVEQSFDKPDLILFSALVSLYDNDIIEEDVIYKWWDNVSTDPRYDEVKKLTVKWVEWLQNADEESSSEEE

>1YOVA

MKLMAQLGKLLKEQKYDRQLRLWGDHGQEALESAHVCLINATATGTEILKNLVLPGIGSFTIIDGNQVSGEDAGNNFFLQRSSIGKNRAEAAMEFLQELNSDVSGSFVEESPENLLDNDPSFFCRFTVVVATQLPESTSLRLADVLWNSQIPLLICRTYGLVGYMRIIIKEHPVIESHPDNALEDLRLDKPFPELREHFQSYDLDHMEKKDHSHTPWIVIIAKYLAQWYSETNGRIPKTYKEKEDFRDLIRQGILKNENGAPEDEENFEEAIKNVNTALNTTQIPSSIEDIFNDDRCINITKQTPSFWILARALKEFVAKEGQGNLPVRGTIPDMIADSGKYIKLQNVYREKAKKDAAAVGNHVAKLLQSIGQAPESISEKELKLLCSNSAFLRVVRCRSLAEEYGLDTINKDEIISSMDNPDNEIVLYLMLRAVDRFHKQQGRYPGVSNYQVEEDIGKLKSCLTGFLQEYGLSVMVKDDYVHEFCRYGAAEPHTIAAFLGGAAAQEVIKIITKQFVIFNNTYIYSGMSQTSATFQL

>3FO3A

EPGENLKPVDAMQCFDCHTQIEDMHTVGKHATVNCVHCHDATEHVETASSRRMGERPVTRMDLEACATCHTAQFNSFVEVRHESHPRLEKATPTSRSPMFDKLIAGHGFAFEHAEPRSHAFMLVDHFVVDRAYGGRFQFKNWQKVTDGMGAVRGAWTVLTDADPESSDQRRFLSQTATAANPVCLNCKTQDHILDWAYMGDEHEAAKWSRTSEVVEFARDLNHPLNCFMCHDPHSAGPRVVRDGLINAVVDRGLGTYPHDPVKSEQQGMTKVTFQRGREDFRAIGLLDTADSNVMCAQCHVEYNCNPGYQLSDGSRVGMDDRRANHFFWANVFDYKEAAQEIDFFDFRHATTGAALPKLQHPEAETFWGSVHERNGVACADCHMPKVQLENGKVYTSHSQRTPRDMMGQACLNCHAEWTEDQALYAIDYIKNYTHGKIVKSEYWLAKMIDLFPVAKRAGVSEDVLNQARELHYDAHLYWEWWTAENSVGFHNPDQARESLMTSISKSKEAVSLLNDAIDAQVASR

>2O5HA

SNAMRKLNNHDVHKRYQDRLEEDVEFTINYELPLSCLWSTIKDFSSDFEEKTEAFFILFKELLRRGHLKLQRDGQIIGHTPEEWEQIFREVWPEYEIEPNPLPGYAPFDIGMWLTVEAPAYAVWIDPEDGSEYWAG

>2XHGA

MGGSRSRKSDQGIIAGNVPLTPIQKWFFGKNFTNTGHWNQSSVLYRPEGFDPKVIQSVMDKIIEHHDALRMVYQHENGNVVQHNRGLGGQLYDFFSYNLTAQPDVQQAIEAETQRLHSSMNLQEGPLVKVALFQTLHGDHLFLAIHHLVVDGISWRILFEDLATGYAQALAGQAISLPEKTDSFQSWSQWLQEYANEADLLSEIPYWESLESQAKNVSLPKDYEVTDCKQKSVRNMRIRLHPEETEQLLKHANQAYQTEINDLLLAALGLAFAEWSKLAQIVIHLEGHGREDIIEQANVARTVGWFTSQYPVLLDLKQTAPLSDYIKLTKENMRKIPRKGIGYDILKHVTLPENRGSLSFRVQPEVTFNYLGQFDADMRTELFTRSPYSGGNTLGADGKNNLSPESEVYTALNITGLIEGGELVLTFSYSSEQYREESIQQLSQSYQKHLLAIIAHCLQSHHHHHH

>3H0UA

MSLTASYETIKARLDGTVLSATFNAPPMNLIGPEVVRDLVALLEELAHPTAPRVVIFDSADADFFFPHVDMTKVPEYTAEAAKAGGPGDASLGMLFRKLSQLPAVTIAKLRGRARGAGSEFLLACDMRFASRENAILGQPEVGIGAPPGAGAIQHLTRLLGRGRALEAVLTSSDFDADLAERYGWVNRAVPDAELDEFVAGIAARMSGFPRDALIAAKSAINAISLPAPAEVRADAALFQQLVRGEKVQQRTAELFKQGFQTRGATELDLGDALGHLKAVDEGHHHHHH

>4AC7B

MSNNNYIVPGEYRVAEGEIEINAGREKTTIRVSNTGDRPIQVGSHIHFVEVNKELLFDRAEGIGRRLNIPSGTAARFEPGEEMEVELTELGGNREVFGISDLTNGSVDNKELILQRAKELGYKGVE

>3PFEA

GMFKPQGLYDYICQQWQEEILPSLCDYIKIPNKSPHFDAKWEEHGYMEQAVNHIANWCKSHAPKGMTLEIVRLKNRTPLLFMEIPGQIDDTVLLYGHLDKQPEMSGWSDDLHPWKPVLKNGLLYGRGGADDGYSAYASLTAIRALEQQGLPYPRCILIIEACEESGSYDLPFYIELLKERIGKPSLVICLDSGAGNYEQLWMTTSLRGNLVGKLTVELINEGVHSGSASGIVADSFRVARQLISRIEDENTGEIKLPQLYCDIPDERIKQAKQCAEILGEQVYSEFPWIDSAKPVIQDKQQLILNRTWRPALTVTGADGFPAIADAGNVMRPVTSLKLSMRLPPLVDPEAASVAMEKALTQNPPYNAKVDFKIQNGGSKGWNAPLLSDWLAKAASEASMTYYDKPAAYMGEGGTIPFMSMLGEQFPKAQFMITGVLGPHSNAHGPNEFLHLDMVKKLTSCVSYVLYSFSQKK

>4DJBA

MGSSHHHHHHSQDPMIRCLRLKVEGALEQIFTMAGLNIRDLLRDILRRWRDENYLGMVEGAGMFIEEIHPEGFSLYVHLDVRAVSLLEAIVQHLTEAIISSLAVEFDHATGGERVHLIDLHFEVLDNLLE

>1Y5HA

GIDPFTMTTARDIMNAGVTCVGEHETLTAAAQYMREHDIGALPICGDDDRLHGMLTDRDIVIKGLAAGLDPNTATAGELARDSIYYVDANASIQEMLNVMEEHQVRRVPVISEHRLVGIVTEADIARHLPEHA

>3V7PA

MRIIKPFAILTPQTIIQDKAVAFDKKIEAIDTVENLIKKYPNAAVEHDENSLLLPGFANPHLHLEFSANKATLQYGDFIPWLYSVIRHREDLLPLCDGACLEQTLSSIIQTGTTAIGAISSYGEDLQACIDSALKVVYFNEVIGSNAATADVMYASFLERFHQSKKHENERFKAAVAIHSPYSVHYILAKRALDIAKKYGSLVSVHFMESRAEREWLDKGSGEFAKFFKEFLNQTRPVNDTKSFLELFKELHTLFVHMVWANEEEIQTIASYNAHIIHCPISNRLLGNGVLDLEKIKSIPYAIATDGLSSNYSLNMYEELKAALFVHPNKEATTFAKELIIRATKAGYDALGFEGGEIAVGKDADMQLIDLPEGLTNVEDLYLHVILHTTKPKKVYIQGEEHVREAENLYFQSHHHHHHWSHPQFEK

>2AXCA

MRGSRSGHGNGGGNSNSGGGSNSSVAAPMAFGFPALAAPGAGTLGISVSGEALSAAIADIFAALKGPFKFSAWGIALYGILPSEIAKDDPNMMSKIVTSLPAETVTNVQVSTLPLDQATVSVTKRVTDVVKDTRQHIAVVAGVPMSVPVVNAKPTRTPGVFHASFPGVPSLTVSTVKGLPVSTTLPRGITEDKGRTAVPAGFTFGGGSHEAVIRFPKESGQKPVYVSVTDVLTPAQVKQRQDEEKRLQQEWNDAHPVEVAERRS

>3TMPA

GSHMGAGYNSEDEYEAAAARIEAMDPATVEQQEHWFEKALRDKKGFIIKQMKEDGACLFRAVADQVYGDQDMHEVVRKHCMDYLMKNADYFSNYVTEDFTTYINRKRKNNCHGNHIEMQAMAEMYNRPVEVYQYSTGTSAVEPINTFHGIHQNEDEPIRVSYHRNIHYNSVVNPNKATIGVGLG

>1R31A

MSLDSRLPAFRNLSPAARLDHIGQLLGLSHDDVSLLANAGALPMDIANGMIENVIGTFELPYAVASNFQINGRDVLVPLVVEEPSIVAAASYMAKLARANGGFTTSSSAPLMHAQVQIVGIQDPLNARLSLLRRKDEIIELANRKDQLLNSLGGGCRDIEVHTFADTPRGPMLVAHLIVDVRDAMGANTVNTMAEAVAPLMEAITGGQVRLRILSNLADLRLARAQVRITPQQLETAEFSGEAVIEGILDAYAFAAVDPYRAATHNKGIMNGIDPLIVATGNDWRAVEAGAHAYACRSGHYGSLTTWEKDNNGHLVGTLEMPMPVGLVGGATKTHPLAQLSLRILGVKTAQALAEIAVAVGLAQNLGAMRALATEGIQRGHMALHARNIAVVAGARGDEVDWVARQLVEYHDVRADRAVALLKQKRGQ

>2MPRA

VDFHGYARSGIGWTGSGGEQQCFQATGAQSKYRLGNECETYAELKLGQEVWKEGDKSFYFDTNVAYSVNQQNDWESTDPAFREANVQGKNLIEWLPGSTIWAGKRFYQRHDVHMIDFYYWDISGPGAGIENIDLGFGKLSLAATRSTEAGGSYTFSSQNIYDEVKDTANDVFDVRLAGLQTNPDGVLELGVDYGRANTTDGYKLADGASKDGWMFTAEHTQSMLKGYNKFVVQYATDAMTTQGKGQARGSDGSSSFTEELSDGTKINYANKVINNNGNMWRILDHGAISLGDKWDLMYVGMYQNIDWDNNLGTEWWTVGVRPMYKWTPIMSTLLEVGYDNVKSQQTGDRNNQYKITLAQQWQAGDSIWSRPAIRIFATYAKWDEKWGYIKDGDNISRYAAATNSGISTNSRGDSDEWTFGAQMEIWW

>2IAFA

IGIGPSSSHTVGPMLAANAFLQLLEQKNLFDKTQRVKVELYGSLALTGKGHGTDKAILNGLENKAPETVDPASMIPRMHEILDSNLLNLAGKKEIPFHEATDFLFLQKELLPKHSNGMRFSAFDGNANLLIEQVYYSIGGGFITTEEDFDK

>3PE9A

MVKLTAPKSNVVAYGNEFLKITATASDSDGKISRVDFLVDGEVIGSDREAPYEYEWKAVEGNHEISVIAYDDDDAASTPDSVKIFVKQARLEHHHHHH

>2V78A

MVDVIALGEPLIQFNSFNPGPLRFVNYFEKHVAGSELNFCIAVVRNHLSCSLIARVGNDEFGKNIIEYSRAQGIDTSHIKVDNESFTGIYFIQRGYPIPMKSELVYYRKGSAGSRLSPEDINENYVRNSRLVHSTGITLAISDNAKEAVIKAFELAKSRSLDTNIRPKLWSSLEKAKETILSILKKYDIEVLITDPDDTKILLDVTDPDEAYRKYKELGVKVLLYKLGSKGAIAYKDNVKAFKDAYKVPVEDPTGAGDAMAGTFVSLYLQGKDIEYSLAHGIAASTLVITVRGDNELTPTLEDAERFLNEFKT

>4I6MD

MDPQTLITKANKVSYYGNPTSKESWRYDWYQPSKVSSNVQQPQQQLGDMENNLEKYPFRYKTWLRNQEDEKNLQRESCEDILDLKEFDRRILKKSLMTSHTKGDTSKATGAPSANQGDEALSVDDIRGAVGNSEAIPGLSAGVNNDNTKESKDVKMN

>1EZGA

QCTGGADCTSCTGACTGCGNCPNAVTCTNSQHCVKANTCTGSTDCNTAQTCTNSKDCFEANTCTDSTNCYKATACTNSSGCPGH

>3C8IA

PKQVGNAQHLYDNYDLVPAMIAEVNPRDMVVMALVNTNVDPTLPPRWALATRNITAIPGIEGDTRKVGTRIPAVAVTGQRSVGNQDSWDQISPMPIAWATPDSSVIARAESTIPSEQWTTLSKNLNKLDQVRETKFDLLEL

>3VUSA

QPWPHNGFVAISWHNVEDEAADQRFMSVRTSALREQFAWLRENGYQPVSIAQIREAHRGGKPLPEKAVVLTFDDGYQSFYTRVFPILQAFQWPAVWAPVGSWVDTPADKQVKFGDELVDREYFATWQQVREVARSRLVELASHTWNSHYGIQANATGSLLPVYVNRAYFTDHARYETAAEYRERIRLDAVKMTEYLRTKVEVNPHVFVWPYGEANGIAIEELKKLGYDMFFTLESGLANASQLDSIPRVLIANNPSLKEFAQQIITVQ

>1T1JA

NLYFQGHMRKIFLACPYSHADAEVVEQRFRACNEVAATIVRAGHVVFSQVSMSHPINLCLAELDRAAIGRLWAPVDAFYMDHLEELIVLDLPGWRDSAGIRREMEFFEAGGQRVSLWSEVEHEFR

>4AVAA

MDGIAELTGARVEDLAGMDVFQGCPAEGLVSLAASVQPLRAAAGQVLLRQGEPAVSFLLISSGSAEVSHVGDDGVAIIARALPGMIVGEIALLRDSPRSATVTTIEPLTGWTGGRGAFATMVHIPGVGERLLRTARQRLAAFVSPIPVRLADGTQLMLRPVLPGDRERTVHGHIQFSGETLYRRFMSARVPSPALMHYLSEVDYVDHFVWVVTDGSDPVADARFVRDETDPTVAEIAFTVADAYQGRGIGSFLIGALSVAARVDGVERFAARMLSDNVPMRTIMDRYGAVWQREDVGVITTMIDVPGPGELSLGREMVDQINRVARQVIEAVG

>3RPCA

SNAMTQYTHIRNATGKLTIKNTTFLIDPFLAPKDTYPGFEGTFNYQQRMPMVDLPLSMDDLLSNVTAVVVTHTHLDHWDDTAINSIPKSLPIFVQNTADKELITSQGFIDVRIIFESLEFNGITLRKTGGSHGTVEMYANPVLAPLAGDAMGVIFEAADEPTVYLVGDTVWTSDVEKALLRFDPNVIIMNTGYAQILGFEDSIIMGTKDIGRMVVRKPEAKIIAVHMDTVNHTATSRKDVRKFIKGNNIESHVAVPEDGETITL

>4AC1X

DLPRLIVYFQTTHDSSNRPISMLPLITEKGIALTHLIVCSFHINQGGVVHLNDFPPDDPHFYTLWNETITMKQAGVKVMGMVGGAAPGSFNTQTLDSPDSATFEHYYGQLRDAIVNFQLEGMDLDVEQPMSQQGIDRLIARLRADFGPDFLITLAPVASALEDSSNLSGFSYTALQQTQGNDIDWYNTQFYSGFGSMADTSDYDRIVANGFAPAKVVAGQLTTPEGAGWIPTSSLNNTIVSLVSEYGQIGGVMGWEYFNSLPGGTAEPWEWAQIVTVILRPGL

>2FP8A

SLALSSPILKEILIEAPSYAPNSFTFDSTNKGFYTSVQDGRVIKYEGPNSGFVDFAYASPYWNKAFCENSTDAEKRPLCGRTYDISYNLQNNQLYIVDCYYHLSVVGSEGGHATQLATSVDGVPFKWLYAVTVDQRTGIVYFTDVSTLYDDRGVQQIMDTSDKTGRLIKYDPSTKETTLLLKELHVPGGAEVSADSSFVLVAEFLSHQIVKYWLEGPKKGTAEVLVKIPNPGNIKRNADGHFWVSSSEELDGNMHGRVDPKGIKFDEFGNILEVIPLPPPFAGEHFEQIQEHDGLLYIGTLFHGSVGILVYDKKGNSFVSSH

>4GDHA

GSHMVKVCLFVADGTDEIEFSAPWGIFKRAEIPIDSVYVGENKDRLVKMSRDVEMYANRSYKEIPSADDFAKQYDIAIIPGGGLGAKTLSTTPFVQQVVKEFYKKPNKWIGMICAGTLTAKTSGLPNKQITGHPSVRGQLEEGGYKYLDQPVVLEENLITSQGPGTAMLFGLKLLEQVASKDKYNAVYKSLSMP

>3ZW5A

MHHHHHHSSGVDLGTENLYFQSMLIRRLDHIVMTVKSIKDTTMFYSKILGMEVMTFKEDRKALCFGDQKFNLHEVGKEFEPKAAHPVPGSLDICLITEVPLEEMIQHLKACDVPIEEGPVPRTGAKGPIMSIYFRDPDRNLIEVSNY

>3P02A

GDEWTDEQFKQLISFKTQPGGWGVTDVHVRYANSAKYTYNLPVLVSGSTDNTDDRLVSFSLRDDTLDILNFEKFGNRPELYFRELPQKYYSFPKELTIPAGQSHALLPIEFSLDGLDDSQKWALPLKVCEDANGTYAVNPRKYYRTAVLRPILFNEFSGRFSGSSLLGTMAGESDIKFSSTEIKLNVVTDSIVFFYAGQRTEDYEDRINYKVFLQFTGDKVDSKKDLYKMKIWAENEKLKFNSYSTPTYKVSSEMDATKTYLKHTYIVISDIDFDFVDYTSVPNYEIEYNMKGGLSVSRDLDTRKPDEDQGSDSKWW

>3KU3A

PGDQICIGYHANNSTEKVDTILERNVTVTHAKDILEKTHNGKLCKLNGIPPLELGDCSIAGWLLGNPECDRLLSVPEWSYIMEKENPRDGLCYPGSFNDYEELKHLLSSVKHFEKVKILPKDRWTQHTTTGGSRACAVSGNPSFFRNMVWLTEKGSNYPVAKGSYNNTSGEQMLIIWGVHHPNDETEQRTLYQNVGTYVSVGTSTLNKRSTPEIATRPKVNGQGGRMEFSWTLLDMWDTINFESTGNLIAPEYGFKISKRGSSGIMKTEGTLENCETKCQTPLGAINTTLPFHNVHPLTIGECPKYVKSEKLVLATGLRNVPQIESR

>1ZA7A

RVVQPVIVEPIASGQGRAIKAWTGYSVSKWTASCAAAEAKVTSAITISLPNELSSERNKQLKVGRVLLWLGLLPSVSGTVKSCVTETQTTAAASFQVALAVADNSKDVVAAMYPEAFKGITLEQLAADLTIYLYSSAALTEGDVIVHLEVEHVRPTFDDSFTPVY

>3COVA

AMAIPAFHPGELNVYSAPGDVADVSRALRLTGRRVMLVPTMGALHEGHLALVRAAKRVPGSVVVVSIFVNPMQFGAGGDLDAYPRTPDDDLAQLRAEGVEIAFTPTTAAMYPDGLRTTVQPGPLAAELEGGPRPTHFAGVLTVVLKLLQIVRPDRVFFGEKDYQQLVLIRQLVADFNLDVAVVGVPTVREADGLAMSSRNRYLDPAQRAAAVALSAALTAAAHAATAGAQAALDAARAVLDAAPGVAVDYLELRDIGLGPMPLNGSGRLLVAARLGTTRLLDNIAIEIGTFAGTDRPDGYR

>2WI8A

MGSKNESTASKASGTASEKKKIEYLDKTYEVTVPTDKIAITGSVESMEDAKLLDVHPQGAISFSGKFPDMFKDITDKAEPTGEKMEPNIEKILEMKPDVILASTKFPEKTLQKISTAGTTIPVSHISSNWKENMMLLAQLTGKEKKAKKIIADYEQDLKETKTKINDKAKDSKALVIRIRQGNIYIYPEQVYFNSTLYGDLGLKAPNEVKAAKAQELISLEKLSEMNPDHIFVQFSDDENADKPDALKDLEKNPIWKSLKAVKEDHVYVNSVDPLAQGGTAWSKVRFLKAAAEKLTQNKLAAALEHHHHHH

>2XOCA

GAMDLLELSDVDSESSDISQPYVVCRQCPEYRRQAAQPPHCPAPEGEPGAPQALGDAPSTSVSLTTAVQDYVCPLQGSHALCTCCFQPMPDRRVEREQDPRVAPQQCAVCLQPFCHLYWGCTRTGCYGCLAPFCELNLGDKCLDGVLNNNSYESDILKNYLATRGLTWKNMLTESLVALQRGVFLLSDYRVTGDTVLCYCCGLRSFRELTYQYRQNIPASELPVAVTSRPDCYWGRNCRTQVKAHHAMKFNHICEQTRFKN

>3N2NA

SMACYGGFDLYFILDKSGSVLHHWNEIYYFVEQLAHKFISPQLRMSFIVFSTRGTTLMKLTEDREQIRQGLEELQKVLPGGDTYMHEGFERASEQIYYENRQGYRTASVIIALTDGELHEDLFFYSEREANRSRDLGAIVYAVGVKDFNETQLARIADSKDHVFPVNDGFQALQGIIHSILKKSC

>4F1VA

MDINGGGATLPQALYQTSGVLTAGFAQYIGVGSGNGKAAFLNNDYTKFQAGVTNKNVHWAGSDSKLSATELSTYASAKQPTWGKLIQVPSVGTSVAIPFNKSGSAAVDLSVQELCGVFSGRINTWDGISGSGRTGPIVVVYRSESSGTTELFTRFLNAKCNAETGNFAVTTTFGTSFSGGLPAGAVAATGSQGVMTALAAGDGRITYMSPDFAAPTLAGLDDATKVARVGKNVATNTQGVSPAAANVSAAIGAVPVPAAADRSNPDAWVPVFGPDNTAGVQPYPTSGYPILGFTNLIFSQCYADATQTTQVRDFFTKHYGASNNNDAAITANAFVPLPTAWKATVRASFLTASNALSIGNTNVCNGIGRPLLEAAHHHHHH

>3R4IA

GMRALTPAEVLFDGEVPPAVLPACDHYAGSEKLMLKSLALQQQLGPVFDITLDCEDGAQVGREAQHAELVASLLGSEHDRFGRVGVRIHDFDHAHWRDDVRLILRAAKRAPAYITLPKIRHVHDAAEMVAFIEATRRELGIAQPVPVQLLVETHGALTRVFDLAALPGVEALSFGLMDFVSAHDGAIPDTAMRSPGQFDHPLVRRAKLEISAACHAYGKVPSHNVSTEVRDMSVVANDAARARNEFGYTRMWSIHPAQIEAIVAAFAPRDEEITTATEILLAAQSAQWGPTRYHDTLHDRASYRYYWSVLRRAQATGRAVPQDAAPLFTKVGTNVQAAS

>1JMUA

GNASSIVQTINVTGDGNVFKPSAETSSTAVPSLSLSPGMLN

>2IMRA

SLLRFSAVSRHHRGASIDPMTFSEATTPDALTPDAHTPRLLTCDVLYTGMGGAQSPGGVVVVGETVAAAGHPDELRRQYPHAAEERAGAVIAPPPVNAHTHLDMSAYEFQALPYFQWIPEVVIRGRHLRGVAAAQAGADTLTRLGAGGVGDIVWAPEVMDALLAREDLSGTLYFEVLNPFPDKADEVFAAARTHLERWRRLERPGLRLGLSPHTPFTVSHRLMRLLSDYAAGEGLPLQIHVAEHPTELEMFRTGGGPLWDNRMPALYPHTLAEVIGREPGPDLTPVRYLDELGVLAARPTLVHMVNVTPDDIARVARAGCAVVTCPRSNHHLECGTFDWPAFAAAGVEVALGTDSVASGETLNVREEVTFARQLYPGLDPRVLVRAAVKGGQRVVGGRTPFLRRGETWQEGFRWELSRDL

>1V0WA

ADSATPHLDAVEQTLRQVSPGLEGDVWERTSGNKLDGSAADPSDWLLQTPGCWGDDKCADRVGTKRLLAKMTENIGNATRTVDISTLAPFPNGAFQDAIVAGLKESAAKGNKLKVRILVGAAPVYHMNVIPSKYRDELTAKLGKAAENITLNVASMTTSKTAFSWNHSKILVVDGQSALTGGINSWKDDYLDTTHPVSDVDLALTGPAAGSAGRYLDTLWTWTCQNKSNIASVWFAASGNAGCMPTMHKDTNPKASPATGNVPVIAVGGLGVGIKDVDPKSTFRPDLPTASDTKCVVGLHDNTNADRDYDTVNPEESALRALVASAKGHIEISQQDLNATCPPLPRYDIRLYDALAAKMAAGVKVRIVVSDPANRGAVGSGGYSQIKSLSEISDTLRNRLANITGGQQAAKTAMCSNLQLATFRSSPNGKWADGHPYAQHHKLVSVDSSTFYIGSKNLYPSWLQDFGYIVESPEAAKQLDAKLLDPQWKYSQETATVDYARGICNA

>4AVAA

MDGIAELTGARVEDLAGMDVFQGCPAEGLVSLAASVQPLRAAAGQVLLRQGEPAVSFLLISSGSAEVSHVGDDGVAIIARALPGMIVGEIALLRDSPRSATVTTIEPLTGWTGGRGAFATMVHIPGVGERLLRTARQRLAAFVSPIPVRLADGTQLMLRPVLPGDRERTVHGHIQFSGETLYRRFMSARVPSPALMHYLSEVDYVDHFVWVVTDGSDPVADARFVRDETDPTVAEIAFTVADAYQGRGIGSFLIGALSVAARVDGVERFAARMLSDNVPMRTIMDRYGAVWQREDVGVITTMIDVPGPGELSLGREMVDQINRVARQVIEAVG

>3BWZA

MAPTSSIEIVLDKTTASVGEIVTASINIKNITNFSGCQLNMKYDPAVLQPVTSSGVAYTKSTMPGAGTILNSDFNLRQVADNDLEKGILNFSKAYVSLDDYRTAAAPEQTGTVAVVKFKVLKEETSSISFEDTTSVPNAIDGTVLFDWNGDRIQSGYSVIQPAVINLDMIKASLEHHHHHH

>2XZIA

INDPAKSAAPYHDEFPLFRSANMASPDKLSTGIGFHSFRIPAVVRTTTGRILAFAEGRRHTNQDFGDINLVYKRTKTTANNGASPSDWEPLREVVGSGAGTWGNPTPVVDDDNTIYLFLSWNGATYSQNGKDVLPDGTVTKKIDSTWEGRRHLYLTESRDDGNTWSKPVDLTKELTPDGWAWDAVGPGNGIRLTTGELVIPAMGRNIIGRGAPGNRTWSVQRLSGAGAEGTIVQTPDGKLYRNDRPSQKGYRMVARGTLEGFGAFAPDAGLPDPACQGSVLRYNSDAPARTIFLNSASGTSRRAMRVRISYDADAKKFNYGRKLEDAKVSGAGHEGGYSSMTKTGDYKIGALVESDFFNDGTGKNSYRAIIWRRFNLSWILNGPNN

>1POIB

DYTNYTNKEMQAVTIAKQIKNGQVVTVGTGLPLIGASVAKRVYAPDCHIIVESGLMDCSPVEVPRSVGDLRFMAHCGCIWPNVRFVGFEINEYLHKANRLIAFIGGAQIDPYGNVNSTSIGDYHHPKTRFTGSGGANGIATYSNTIIMMQHEKRRFMNKIDYVTSPGWIDGPGGRERLGLPGDVGPQLVVTDKGILKFDEKTKRMYLAAYYPTSSPEDVLENTGFDLDVSKAVELEAPDPAVIKLIREEIDPGQAFIQVP

>3SFKA

MGHHHHHHAENLYFQGAPFESYNPEFFLYDIFLKFCLKYIDGEICHDLFLLLGKYNILPYDTSNDSIYACTNIKHLDFINPFGVAAGFDKNGVCIDSILKLGFSFIEIGTITPRGQTGNAKPRIFRDVESRSIINSCGFNNMGCDKVTENLILFRKRQEEDKLLSKHIVGVSIGKNKDTVNIVDDLKYCINKIGRYADYIAINVSSPNTPGLRDNQEAGKLKNIILSVKEEIDNLEKNNIMNDEFLWFNTTKKKPLVFVKLAPDLNQEQKKEIADVLLETNIDGMIISNTTTQINDIKSFENKKGGVSGAKLKDISTKFICEMYNYTNKQIPIIASGGIFSGLDALEKIEAGASVCQLYSCLVFNGMKSAVQIKRELNHLLYQRGYYNLKEAIGRKHSKS

>2XTSA

AGTPDPLITEIQPWASEFGEAVDAHPYGLPIHFESHVKRQYVEWLTESPVSSINFTPIHALEGTITPQGCAFERHHSGAIELSKQDYRLMINGLVEKPLVFTFEDLLRFPRTTTTAFCECAANGGMEWGGAQLEGCQYTQGMIHNMEYVGVPLSVLLAEAGVKPEGKWLYAEGADASSNGRSFPMEKVMDDVMLAFFANGEALRKEHGYPARLVVPGWEGNMWVKWVRRLGIYDKAVESREETSKYTDLMPDGRARKWTWVMDAKSVITSPSPQVPIRHGKGPLVISGLAWSGNGRITRVDVSLDGGKNWTTARITGQALPKALTRFHLDIDWDGSEMLLQSRAVDETGYVQPTKDALRAIRGRNNVYHNNGIQTWWVKADGEVENVEIA

>2ZIHA

GSMSTLQRRRVNRADSGDTSSIHSSANNTKGDKIANIAVDGDDDNGTNKKIAYDPEESKLRDNINIPTLTLMEEVLLMGLRDREGYLSFWNDSISYALRGCIIIELALRGKIRILDDSARKRFDLSERLIEVIDSSKTGEVLLDETLQLMKNDEPLSISNWIDLLSGETWNLLKINYQLKQVRERLAKGLVDKGVLRTEMKNFFLFDMATHPIADASCKEAIKRRVLSVLVSRNMELSYNEYFPETTSFKIIRTLALICGSYGANVLENVLTTLEYEKRDKAISRAEEIMAQFSQYPFDLEKETELGVSVNLNKEVKEEIENNPGHDLQLEVIAGVFEVFSRMDMLL

>1DYOA

KPEEPDAGYYYHDTFEGSVGQWTARGPAEVLLSGRTAYKGSESLLVRNRTAAWNGAQRALNPRTFVPGNTYCFSVVASFIEGASSTTFCMKLQYVDGSGTQRYDTIDMKTVGPNQWVHLYNPQYRIPSDATDMYVYVETADDTINFYIDEAIGAVAGTVI

>3S6FA

GMTQRSLADIQFQTTLEGVTPAQLGGFFEGWPNPPTPETLWRILDRAAVFVLARTPDGQVIGFVNALSDGILAASIPLLEVQAGWRSLGLGSELMRRVLTELGDLYMVDLSCDDDVVPFYERLGLKRANAMFLRRYDNQAGIPAE

>1DGWY

MQLRRYAATLSEGDIIVIPSSFPVALKAASDLNMVGIGVNAENNERNFLAGHKENVIRQIPRQVSDLTFPGSGEEVEELLENQKESYFVDGQP

>3TMPA

GSHMGAGYNSEDEYEAAAARIEAMDPATVEQQEHWFEKALRDKKGFIIKQMKEDGACLFRAVADQVYGDQDMHEVVRKHCMDYLMKNADYFSNYVTEDFTTYINRKRKNNCHGNHIEMQAMAEMYNRPVEVYQYSTGTSAVEPINTFHGIHQNEDEPIRVSYHRNIHYNSVVNPNKATIGVGLG

>3LMEA

MSLKIIAPTDKTITPSGTWSIGARAGDFVFIGGMHGTDRVTGKMVDGDEARIRRMFDNMLAAAEAAGATKADAVRLTVFVTDVAKYRPVVNKVQKDIWGDGPYPPRTVLQVPALDQGDIAEIDGTFYAPAEGHHHHHH

>1MJ4A

QESTHIYTKEEVSSHTSPETGIWVTLGSEVFDVTEFVDLHPGGPSKLMLAAGGPLEPFWALYAVHNQSHVRELLAQYKIGEL

>3GDWA

SNANVGVFVLMHGDSTASSMLKTAQELLGTSIGTAMNMPLTMEVQTMYEQLRNQVITQKESLNNGILLLTDMGSLNSFGNMLFEETGIRTKAITMTSTMIVLEAIRMASVGRSLEDIYQNIQLSFESVVREQFRSSLQK

>1RJUV

HECQCQCGSCKNNEQCQKSCSCPTGCNSDDKCPCGN

>4I3MA

GSHMGLFNAHAVAQQRADRIATLLQSFADGQLDTAVGEAPAPGYERHYDSLRALQRQLREQRAELQQVESLEAGLAEMSRQHEAGWIDQTIPAERLEGRAARIAKGVNELVAAHIAVKMKVVSVVTAYGQGNFEPLMDRLPGKKAQITEAIDGVRERLRGAAEATSAQLATAAYN

>3M6NA

MGSSHHHHHHSQDPNSMSAVQPFIRTNIGSTLRIIEEPQRDVYWIHMHADLAINPGRACFSTRLVDDITGYQTNLGQRLNTAGVLAPHVVLASDSDVFNLGGDLALFCQLIREGDRARLLDYAQRCVRGVHAFHVGLGARAHSIALVQGNALGGGFEAALSCHTIIAEEGVMMGLPEVLFDLFPGMGAYSFMCQRISAHLAQKIMLEGNLYSAEQLLGMGLVDRVVPRGQGVAAVEQVIRESKRTPHAWAAMQQVREMTTAVPLEEMMRITEIWVDTAMQLGEKSLRTMDRLVRAQSRRSGLDAG

>3GREA

MGHHHHHHGEGDVESIEKFLSTFKILPPLRDYKEFGPIQEIVRSPNMGNLRGKLIATLMENEPNSITSSAVSPGETPYLITGSDQGVIKIWNLKEIIVGEVYSSSLTYDCSSTVTQITMIPNFDAFAVSSKDGQIIVLKVNHYQQESEVKFLNCECIRKINLKNFGKNEYAVRMRAFVNEEKSLLVALTNLSRVIIFDIRTLERLQIIENSPRHGAVSSICIDEECCVLILGTTRGIIDIWDIRFNVLIRSWSFGDHAPITHVEVCQFYGKNSVIVVGGSSKTFLTIWNFVKGHCQYAFINSDEQPSMEHFLPIEKGLEELNFCGIRSLNALSTISVSNDKILLTDEATSSIVMFSLNELSSSKAVISPSRFSDVFIPTQVTANLTMLLRKMKRTSTHSVDDSLYHHDIINSISTCEVDETPLLVACDNSGLIGIFQ

>3SOYA

SNAATSTVKQEITEGINRYLYSIDKADPTLGKQLFYVSPETSFIHPRGHERGWSQIAENFYGTTMGKTFSKRTLKLDAPPAIHVYGNAAVAEFDWHFTAVRRDNGQTQHTTGRESQVWAKIPNTGWRIVHVHYSGPAKTGVGEGY

>1LQVA

SQDASDGLQRLHMLQISYFRDPYHVWYQGNASLGGHLTHVLEGPDTNTTIIQLQPLQEPESWARTQSGLQSYLLQFHGLVRLVHQERTLAFPLTIRCFLGCELPPEGSRAHVFFEVAVNGSSFVSFRPERALWQADTQVTSGVVTFTLQQLNAYNRTRYELREFLEDTCVQYVQKHISAENTKGSQTSRSYTS

>4ESQA

GHQPVAEERLSALLLNSSEVNAVMGSSSMQPGKPITSMDSSPVTVSLPDCQGALYTSQDPVYAGTGYTAINGLISSEPGDNYEHWVNQAVVAFPTADKARAFVQTSADKWKNCAGKTVTVTNKAKTYRWTFADVKGSPPTITVIDTQEGAEGWECQRAMSVANNVVVDVNACGYQITNQAGQIAAKIVDKVNKE

>1SDDA

AKLRQFYVAAQSIRWNYRPESTHLSSKPFETSFKKIVYREYEAYFQKEKPQSRTSGLLGPTLYAEVGDIMKVHFKNKAHKPLSIHAQGIKYSKFSEGASYSDHTLPMEKMDDAVAPGQEYTYEWIISEHSGPTHDDPPCLTHIYYSYVNLVEDFNSGLIGPLLICKKGTLTEDGTQKMFEKQHVLMFAVFDESKSWNQTSSLMYTVNGYVNGTMPDITVCAHDHISWHLIGMSSGPELFSIHFNGQVLEQNHHKISAITLVSATSTTANMTVSPEGRWTIASLIPRHFQAGMQAYIDIKNCAKKTR

>4FCCA

PGSMDQTYSLESFLNHVQKRDPNQTEFAQAVREVMTTLWPFLEQNPKYRQMSLLERLVEPERVIQFRVVWVDDRNQVQVNRAWRVQFSSAIGPYKGGMRFHPSVNLSILKFLGFEQTFKNALTTLPMGGGKGGSDFDPKGKSEGEVMRFCQALMTELYRHLGADTDVPAGDIGVGGREVGFMAGMMKKLSNNTACVFTGKGLSFGGSLIRPEATGYGLVYFTEAMLKRHGMGFEGMRVSVSGSGNVAQYAIEKAMEFGARVITASDSSGTVVDESGFTKEKLARLIEIKSSRDGRVADYAKEFGLVYLEGQQPWSVPVDIALPCATQNELDVDAAHQLIANGVKAVAEGANMPTTIEATELFQQAGVLFAPGKAANAGGVATSGLEMAQNAARLGWKAEKVDARLHHIMLDIHHACVEHGGEGEQTNYVQGANIAGFVKVADAMLAQGVI

>1Q8DA

ERPNCLSLQDSCKTNYICRSRLADFFTNCQPESRSVSNCLKENYADCLLAYSGLIGTVMTPNYVDSSSLSVAPWCDCSNSGNDLEDCLKFLNFFKDNTCLKNAIQAFG

>3GODA

GSFTMDDISPSELKTILHSKRANLYYLQHCRVLVNGGRVEYVTDEGRHSHYWNIPIANTTSLLLGTGTSITQAAMRELARAGVLVGFCGGGGTPLFSANEVDVEVSWLTPQSEYRPTEYLQRWVGFWFDEEKRLVAARHFQRARLERIRHSWLEDRVLRDAGFAVDATALAVAVEDSARALEQAPNHEHLLTEEARLSKRLFKLAAQATRYGEFVRAKRGSGGDPANRFLDHGNYLAYGLAATATWVLGIPHGLAVLHGKTRRGGLVFDVADLIKDSLILPQAFLSAMRGDEEQDFRQACLDNLSRAQALDFMIDTLKDVAQRSTVSA

>3CR3A

LLTIDTTIEWLGKFNEKIQENKAYLSELDGPIGDGDHGANMARGMSETMKALEVSNFGNVSEIFKKVAMTLMSKVGGASGPLYGSAFLAMSKTAIETLDTSELIYAGLEAIQKRGKAQVGEKTMVDIWSAFLNDLQTDSASKDNLEKVVKASAGLLATKGRASYLGERSIGHIDPGTQSSAYLFETLLEVVA

>2QT1A

MGSSHHHHHHSSGLVPRGSKTFIIGISGVTNSGKTTLAKNLQKHLPNCSVISQDDFFKPESEIETDKNGFLQYDVLEALNMEKMMSAISCWMESARHSVVSTDQESAEEIPILIIEGFLLFNYKPLDTIWNRSYFLTIPYEECKRRRSTRVYQPPDSPGYFDGHVWPMYLKYRQEMQDITWEVVYLDGTKSEEDLFLQVYEDLIQEL

>2WQKA

MPTFLLVNDDGYFSPGINALREALKSLGRVVVVAPDRNLSGVGHSLTFTEPLKMRKIDTDFYTVIDGTPADCVHLGYRVILEEKKPDLVLSGINEGPNLGEDITYSGTVSGAMEGRILGIPSIAFSAFGRENIMFEEIAKVCVDIVKKVLNEGIPEDTYLNVNIPNLRYEEIKGIKVTRQGKRAYKERVFKYIDPYGKPFYWIAAEEFGWHAEEGTDYWAVLNGYVSVTPLHLDLTNYKVMKSIKYLEDSP

>1JMUB

PGGVPWIAIGDETSVTSPGALRRMTSKDIPETAIINTDNSSGAVPSESALVPYNDEPLVVVTEHAIANFTKAEMALEFNREFLDKLRVLSVSPKYSDLLTYVDCYVGVSARQALNNFQKQVPVITPTRQTMYVDSIQAALKALEKWEIDLRVAQTLLPTNVPIGEVSCPMQSVVKLLDDQLPDDSLIRRYPKEAAVALAKRNGGIQWMDVSEGTVMNEAVNAVAASALAPSASAPPLEEKSKLTEQAMDLVTAAEPEIIASLVPVPAPVFAIPPKPADYNVRTLKIDEATWLRMIPKTMGTLFQIQVTDNTGTNWHFNLRGGTRVVNLDQIAPMRFVLDLGGKSYKETSWDPNGKKVGFIVFQSKIPFELWTAASQIGQATVVNYVQLYAEDSSFTAQSIIATTSLAYNYEPEQLNKTDPEMNYYLLATFIDSAAITPTNMTQPDVWDALLTMSPLSAGEVTVKGAVVSEVVPAELIGSYTPESLNASLPNDAARCMIDRASKIAEAIKIDDDAGPDEYSPNSVPIQGQLAISQLETGYGVRIFNPKGILSKIASRAMQAFIGDPSTIITQAAPVLSDKNNWIALAQGVKTSLRTKSLSAGVKTAVSKLSSSESIQNWTQGFLDKVSTHFPAPKPDCPTNGDGSEPSARRVKRDSYAGVVKRGYTR

>3C8GA

SNAMATLTEDDVLEQLDAQDNLFSFMKTAHSILLQGIRQFLPSLFVDNDEEIVEYAVKPLLAQSGPLDDIDVALRLIYALGKMDKWLYADITHFSQYWHYLNEQDETPGFADDITWDFISNVNSITRNATLYDALKAMKFADFAVWSEARFSGMVKTALTLAVTTTLKELTP

>2EZ2A

MNYPAEPFRIKSVETVSMIPRDERLKKMQEAGYNTFLLNSKDIYIDLLTDSGTNAMSDKQWAGMMMGDEAYAGSENFYHLERTVQELFGFKHIVPTHQGRGAENLLSQLAIKPGQYVAGNMYFTTTRYHQEKNGAVFVDIVRDEAHDAGLNIAFKGDIDLKKLQKLIDEKGAENIAYICLAVTVNLAGGQPVSMANMRAVRELTEAHGIKVFYDATRCVENAYFIKEQEQGFENKSIAEIVHEMFSYADGCTMSGKKDCLVNIGGFLCMNDDEMFSSAKELVVVYEGMPSYGGLAGRDMEAMAIGLREAMQYEYIEHRVKQVRYLGDKLKAAGVPIVEPVGGHAVFLDARRFCEHLTQDEFPAQSLAASIYVETGVRSMERGIISAGRNNVTGEHHRPKLETVRLTIPRRVYTYAHMDVVADGIIKLYQHKEDIRGLKFIYEPKQLRFFTARFDYI

>2QWUA

MIMSEMITRQQVTSGETIHVRTDPTACIGSHPNCRMFIDSLTIAGEKLDKNIVAIDGGEDVTKADSATAAASVIRMSITPGSINPTISITLGVLIKSNVRTKIEEKVSSILQASATDMKIKLGNSNKKQEYKTDEAWGIMIDLSNLELYPISAKAFSISIEPTELMGVSKDGMRYHIISIDGLTTSQGSLPVCCAASTDKGVAKIGYIAAA

>3DI5A

GMYQTIEGFLQSWTYETESTQKMLDVLTDESLSQEIAPGHWTLGRVAWHIVTAIPVILSGTGLKFEGETKDYPVPTSAKTIADGYRKVNTAFVEALQSEWTDKDLTTINDFFGRPMPNSIFLMTLINHQNHHRGQMTVLMRQAGLTVPGVYGPAKEEWATAGMEAPKM

>2ZPMA

GIPIEPVLENVQPNSAASKAGLQAGDRIVKVDGQPLTQWVTFVMLVRDNPGKSLALEIERQGSPLSLTLIPESKPGNGKAIGFVGIEPKVI

>2BWRA

SVVVISQALPVPTRIPGVADLVGFGNGGVYIIRNSLLIQVVKVINNFGYDAGGWRVEKHVRLLADTTGDNQSDVVGFGENGVWISTNNGNNTFVDPPKMVLANFAYAAGGWRVEKHIRFMADLRKTGRADIVGFGDGGIYISRNNGGGQFAPAQLALNNFGYAQGWRLDRHLRFLADVTGDGLLDVVGFGENQVYIARNSGNGTFQPAQAVVNNFCIGAGGWTISAHPRVVADLTGDRKADILGFGVAGVYTSLNNGNGTFGAVNLVLKDFGVNSGWRVEKHVRCVSSLTNKKVGDIIGFGDAGVYVALNNGNGTFGPVKRVIDNFGYNQGWRVDKHPRFVVDLTGDGCADIVGFGENSVWACMNKGDGTFGPIMKLIDDMTVSKGWTLQKTVRYAANLYL

>2Z2NA

MEFKLQELNLTNQDTGPYGITVSDKGKVWITQHKANMISCINLDGKITEYPLPTPDAKVMCLTISSDGEVWFTENAANKIGRITKKGIIKEYTLPNPDSAPYGITEGPNGDIWFTEMNGNRIGRITDDGKIREYELPNKGSYPSFITLGSDNALWFTENQNNAIGRITESGDITEFKIPTPASGPVGITKGNDDALWFVEIIGNKIGRITTSGEITEFKIPTPNARPHAITAGAGIDLWFTEWGANKIGRLTSNNIIEEYPIQIKSAEPHGICFDGETIWFAMECDKIGKLTLIKDNME

>2GDMA

GALTESQAALVKSSWEEFNANIPKHTHRFFILVLEIAPAAKDLFSFLKGTSEVPQNNPELQAHAGKVFKLVYEAAIQLEVTGVVVTDATLKNLGSVHVSKGVADAHFPVVKEAILKTIKEVVGAKWSEELNSAWTIAYDELAIVIKKEMDDAA

>1IQZA

PKYTIVDKETCIACGACGAAAPDIYDYDEDGIAYVTLDDNQGIVEVPDILIDDMMDAFEGCPTDSIKVADEPFDGDPNKFE

>1M6IA

EEVPQDKAPSHVPFLLIGGGTAAFAAARSIRARDPGARVLIVSEDPELPYMRPPLSKELWFSDDPNVTKTLRFKQWNGKERSIYFQPPSFYVSAQDLPHIENGGVAVLTGKKVVQLDVRDNMVKLNDGSQITYEKCLIATGGTPRSLSAIDRAGAEVKSRTTLFRKIGDFRSLEKISREVKSITIIGGGFLGSELACALGRKARALGTEVIQLFPEKGNMGKILPEYLSNWTMEKVRREGVKVMPNAIVQSVGVSSGKLLIKLKDGRKVETDHIVAAVGLEPNVELAKTGGLEIDSDFGGFRVNAELQARSNIWVAGDAACFYDIKLGRRRVEHHDHAVVSGRLAGENMTGAAKPYWHQSMFWSDLGPDVGYEAIGLVDSSLPTVGVFAKATAQDNPKSATEQSGTGIRSESETESEASEITIPPSTPAVPQAPVQGEDYGKGVIFYLRDKVVVGIVLWNIFNRMPIARKIIKDGEQHEDLNEVAKLFNIHED

>1R6WA

SHMRSAQVYRWQIPMDAGVVLRDRRLKTRDGLYVCLREGEREGWGEISPLPGFSQETWEEAQSVLLAWVNNWLAGDCELPQMPSVAFGVSCALAELTDTLPQAANYRAAPLCNGDPDDLILKLADMPGEKVAKVRVGLYEAVRDGMVVNLLLEAIPDLHLRLDANRAWTPLKGQQFAKYVNPDYRDRIAFLEEPCKTRDDSRAFARETGIAIAWDESLREPDFAFVAEEGVRAVVIKPTLTGSLEKVREQVQAAHALGLTAVISSSIESSLGLTQLARIAAWLTPDTIPGLDTLDLMQAQQVRRWPGSTLPVVEVDALERLL

>1UUQA

MVAESNSAVAPTANVATSPAHEHFVRVNGGHFELQGKPYVITGVNMWYAAYLGAPNEVGDRDRLAKELDNLKAIGVNNLRVLAVSEKSEINSAVKPAVTNGFGNYDETLLQGLDYLLVELAKRDMTVVLYFNNFWQWSGGMTQYMAWIEGEPVQDPNVTNEWEAFMAKSASFYRSEKAQQEYRKTLEKIITRVNSINGKAYVDDATIMSWQLANEPRPGNSQTTAEEKQIYIDWVHAAAAYIKTLDAHHLVSSGSEGEMGSVNDMQVFIDAHATPDIDYLTYHMWIRNWSWFDKTKPAETWPSAWEKAQNYMRAHIDVAKQLNKPLVLEEFGLDRDMGSYAMDSTTEYRDNYFRGVFELMLASLEQGEPSAGYNIWAWNGYGRTTRANYWWQEGDDFMGDPPQEEQGMYGVFDTDTSTIAIMKEFNARFQPKLEHHHHHH

>1XIPA

GASSLKDEVPTETSEDFGFKFLGQKQILPSFNEKLPFASLQNLDISNSKSLFVAASGSKAVVGELQLLRDHITSDSTPLTFKWEKEIPDVIFVCFHGDQVLVSTRNALYSLDLEELSEFRTVTSFEKPVFQLKNVNNTLVILNSVNDLSALDLRTKSTKQLAQNVTSFDVTNSQLAVLLKDRSFQSFAWRNGEMEKQFEFSLPSELEELPVEEYSPLSVTILSPQDFLAVFGNVISETDDEVSYDQKMYIIKHIDGSASFQETFDITPPFGQIVRFPYMYKVTLSGLIEPDANVNVLASSCSSEVSIWDSKQVIEPSQDSERAVLPISEETDKDTNPIGVAVDVVTSGTILEPCSGVDTIERLPLVYILNNEGSLQIVGLFHVAAIKS

>3VV1A

MDYKDDDDKAAAGSMIGGGIGISFRNEFFNPQTPVNIPVQGFSNGARLRLVLLPTSADSRFHINLRTPDDIVLHFNARFDEGAVVNNSTSGGGWQSEDRHANPFQQNKIYTLEFVSNGGIISIFVNGAHFADFVERTPSHGVHLIEIEGGVHVHSAHVSH

>2GBLA

MTSAEMTSPNNNSEHQAIAKMRTMIEGFDDISHGGLPIGRSTLVSGTSGTGKTLFSIQFLYNGIIEFDEPGVFVTFEETPQDIIKNARSFGWDLAKLVDEGKLFILDASPDPEGQEVVGGFDLSALIERINYAIQKYRARRVSIDSVTSVFQQYDASSVVRRELFRLVARLKQIGATTVMTTERIEEYGPIARYGVEEFVSDNVVILRNVLEGERRRRTLEILKLRGTSHMKGEYPFTITDHGINIFPLGAMRLTQRSSNVRVSSGVVRLDEMCGGGFFKDSIILATGATGTGKTLLVSRFVENACANKERAILFAYEESRAQLLRNAYSWGMDFEEMERQNLLKIVCAYPESAGLEDHLQIIKSEINDFKPARIAIDSLSALARGVSNNAFRQFVIGVTGYAKQEEITGLFTNTSDQFMGAHSITDSHISTITDTIILLQYVEIRGEMSRAINVFKMRGSWHDKAIREFMISDKGPDIKDSFRNFERIISGSPTRITVDEKSELSRIVRGVQEKGPES

>4A27A

SMEMRAVVLAGFGGLNKLRLFRKAMPEPQDGELKIRVKACGLNFIDLMVRQGNIDNPPKTPLVPGFECSGIVEALGDSVKGYEIGDRVMAFVNYNAWAEVVCTPVEFVYKIPDDMSFSEAAAFPMNFVTAYVMLFEVANLREGMSVLVHSAGGGVGQAVAQLCSTVPNVTVFGTASTFKHEAIKDSVTHLFDRNADYVQEVKRISAEGVDIVLDCLCGDNTGKGLSLLKPLGTYILYGSSNMVTGETKSFFSFAKSWWQVEKVNPIKLYEENKVIAGFSLLNLLFKQGRAGLIRGVVEKLIGLYNQKKIKPVVDSLWALEEVKEAMQRIHDRGNIGKLILDVEKTPTPL

>3PIUA

MRMLSRNATFNSHGQDSSYFLGWQEYEKNPYHEVHNTNGIIQMGLAENQLCFDLLESWLAKNPEAAAFKKNGESIFAELALFQDYHGLPAFKKAMVDFMAEIRGNKVTFDPNHLVLTAGATSANETFIFCLADPGEAVLIPTPYYPGFDRDLKWRTGVEIVPIHCTSSNGFQITETALEEAYQEAEKRNLRVKGVLVTNPSNPLGTTMTRNELYLLLSFVEDKGIHLISDEIYSGTAFSSPSFISVMEVLKDRNCDENSEVWQRVHVVYSLSKDLGLPGFRVGAIYSNDDMVVAAATKMSSFGLVSSQTQHLLSAMLSDKKLTKNYIAENHKRLKQRQKKLVSGLQKSGISCLNGNAGLFCWVDMRHLLRSNTFEAEMELWKKIVYEVHLNISPGSSCHCTEPGWFRVCFANLPERTLDLAMQRLKAFVGEYYNV

>3U8VA

SGHTAHVDEAVKHAEEAVAHGKEGHTDQLLEHAKESLTHAKAASEAGGNTHVGHGIKHLEDAIKHGEEGHVGVATKHAQEAIEHLRASEHKSH

>3VSVA

MEYHVAKTGSDEGKGTLKDPFLTINKAASVAMAGDTIIVHEGVYREWVKPKYKGLSDKRRITYKAAEGEKVVIKGSERIQSWQRVEGNVWRCQLPNSFFGEFNPYKEEVFGDWLLTVNEKKHLGDVYLNGMSFYEVTNYEDLFNPQLRTEVLDHWTQKIVPIKNAEQTKYVWYAEVDREKTTIYANFQGADPNEEFVEINVRRSCFYPVETGIDYITVKGFEMAHAATPWAPPTADQPGLIGPNWSKGWIIEDNIIHDAKCSAISIGKEATTGNNYRSIRKDKPGYQYQLEAVFNAKRNGWSKEKIGSHIIRNNTIYDCGQNAIVGHLGGVFSEIYNNHIYNIALKREFYGHEIAGIKLHAAIDVQIHHNRIHDCSLGLWLDWEAQGTRVSKNLFYNNNRDVFVEVSHGPYLVDHNILSSEYAIDNMSQGGAYINNLIAGKMNQRKVLNRSTQYHLPHSTEVAGFAFVYGGDDRFYNNIFIGKEGLENVGTSHYNNCTTSLEEYIEKVNEVPGDLGEFERVEQPVYINKNAYFNGAEPFEKEKDNLVKKDFDPKLAIIDEGDEVYLSLQLPDEFENIVGDIHSTKTLERVRIVDAEYESPDGKELVLDTDYLDAKKPENSSIGPIALLKKGNNYIKVW

>1JUHA

DTSSLIVEDAPDHVRPYVIRHYSHARAVTVDTQLYRFYVTGPSSGYAFTLMGTNAPHSDALGVLPHIHQKHYENFYCNKGSFQLWAQSGNETQQTRVLSSGDYGSVPRNVTHTFQIQDPDTEMTGVIVPGGFEDLFYYLGTNATDTTHTPYIPSSSDSSSTTGPDSSTISTLQSFDVYAELSFTPRTDTVNGTAPANTVWHTGANALASTAGDPYFIANGWGPKYLNSQYGYQIVAPFVTATQAQDTNYTLSTISMSTTPSTVTVPTWSFPGACAFQVQEGRVVVQIGDYAATELGSGDVAFIPGGVEFKYYSEAYFSKVLFVSSGSDGLDQNLVNGGEEWSSVSFPADW

>3SGGA

GAGVVVPKYTPSTENPGGPGEEPGDGLIDPAEPLDRGFMHLKGRELKSLNSISITGLNDGEKVILSTLAGLAARVTGDQVYINEGGPSSVWLKQMQNKYGIPVNTYNALAPLVQHYVETGVIKGYIVYTPYSEGQSHSINVATSLCGLLRGIAVPESLVDKVKAMGVTTELMDVRSYDEKWLYENYKDQLDKSLAADMKPEIFHHLRDYITMTNAFAFYDYNARRDWSWRTSILKDLDKGAYCFGYYDLDEWGMVNNASQLGVSMLPTDQAANLATLSSIYDTTGLKQRPATKEVVTEENVHYVTFLVSDGDNIAFNLWGQQGYMDHDLHGQFPLGYTISPSLYDLAPAALRWYYENSKEGDYFVAGPSGSSYIFPSKMSDADLDDYLAKLNEYVDKSGLNICNILDQKIMDNPKVYNKYLAQPNIDAIFYTGYGEKGDGRIKFSDNGKPVIEQRSVLWEGIDGGSNRGEESTVISQINSRSANPHSADGYTFVFVHCWTKNQQSIKTVIDGLNDNVRVVPVDQFVQLVKQNLGPK

>4DVEA

MHHHHHHHHAMTNNQKVKTLTYSAFMTAFIIILGFLPGIPIGFIPVPIILQNMGIMMAGGLLGPKYGTISVGAFLALALIGLPVLTGGNGGAASFLGPSGGYRIAWLFTPFLIGFFLKKLKITTSQNWFGELIIVLLFGVIFVDFVGAIWLSFQSNIPLLTSLISNLVFIPGDCIKAILTVVIVRRLRKQGGFELYFR

>2Q4MA

MEQPYVYAYPQGSGPSGAPTPQAGGVVVDPKYCAPYPIDMAIVRKMMSLTDGNFVITDVNGNLLFKVKEPVFGLHDKRVLLDGSGTPVVTLREKMVSMHDRWQVFRGGSTDQRDLLYTVKRSSMLQLKTKLDVFLGHNKDEKRCDFRVKGSWLERSCVVYAGESDAIVAQMHRKHTVQSVFLGKDNFSVTVYPNVDYAFIASLVVILDDVNREDRAA

>2G0WA

MGSDKIHHHHHHMTNANGNLKKCPITISSYTLGTEVSFPKRVKVAAENGFDGIGLRAENYVDALAAGLTDEDMLRILDEHNMKVTEVEYITQWGTAEDRTAEQQKKEQTTFHMARLFGVKHINCGLLEKIPEEQIIVALGELCDRAEELIIGLEFMPYSGVADLQAAWRVAEACGRDNAQLICDTWHWARANQTAESIKNVPADRIVSIQLCDVHETPYKELREESLHDRLAPGEGYGDTVGFAKILKEHGVNPRVMGVEVISDSMVATGLEYAALKVYNATKKVLDEAWPEISPR

>3G8YA

GYQPEKHAVVKSDRGDGRLLSTYAIVHEMLKDTHPQYAYRSGMSAQEFTQWQDGVRAAMVEIMKFPEIKRQPSPVCVKTEKKEGYILEKWEFYPFPKSVSTFLVLKPEHLKGAVPGVLCIPGSGRTKEGLVGEPGICDKLTEDYNNPKVSMALNMVKEGYVAVAVDNAAAGEASDLECYDKGWNYDYDVVSRFLLELGWSWLGYTSYLDMQVLNWMKAQSYIRKDRIVISGFSLGTEPMMVLGVLDKDIYAFVYNDFLCQTQERAVVMTKPDKENRRPFPNSIRHLIPGYWRYFNFPDVVASLAPRPIIFTEGGLDRDFRLVQSAYAASGKPENAEFHHYPKFADKAVRKDVEHLDEGLDSKTYFEAVNVDPPSHYFKNELVIPWLRKVLK

>2NXVA

MKPVPTYVQDKDESTLMFSVCSLVRDQAKYDRLLESFERFGFTPDKAEFLAADNREGNQFHGFSWHKQMLPRCKGRYVIFCHEDVELVDRGYDDLVAAIEALEEADPKWLVAGVAGSPWRPLNHSVTAQALHISDVFGNDRRRGNVPCRVESLDECFLLMRRLKPVLNSYDMQGFHYYGADLCLQAEFLGGRAYAIDFHLHHYGRAIADENFHRLRQEMAQKYRRWFPGRILHCVTGRVALGGGWYEAR

>3L6IA

HHHSHMNQLTQYTITEQEINQSLAKHNNFSKDIGLPGVADAHIVLTNLTSQIGREEPNKVTLTGDANLDMNSLFGSQKATMKLKLKALPVFDKEKGAIFLKEMEVVDATVQPEKMQTVMQTLLPYLNQALRNYFNQQPAYVLREDGSQGEAMAKKLAKGIEVKPGEIVIPFTDLEHHHHHH

>1IARB

FKVLQEPTCVSDYMSISTCEWKMNGPTNCSTELRLLYQLVFLLSEAHTCIPENNGGAGCVCHLLMDDVVSADNYTLDLWAGQQLLWKGSFKPSEHVKPRAPGNLTVHTNVSDTLLLTWSNPYPPDNYLYNHLTYAVNIWSENDPADFRIYNVTYLEPSLRIAASTLKSGISYRARVRAWAQAYNTTWSEWSPSTKWHNSYREPFEQH

>4FR9A

GGDDDDTGYLPPSQAIQDALKKLYPNATAIKWEQKGVYYVADCQADGREKEVWFDANANWLMTETELNSINNLPPAVLTAFMESSYNNWVVDDVVILEYPNEPSTEFVVTVEQGKKVDLYFSEGGGLLHEKDVTNGDDTHWPRV

>1OXWA

MHHHHHHAMAQLGEMVTVLSIDGGGIRGIIPATILEFLEGQLQEMDNNADARLADYFDVIGGTSTGGLLTAMISTPNENNRPFAAAKEIVPFYFEHGPQIFNPSGQILGPKYDGKYLMQVLQEKLGETRVHQALTEVVISSFDIKTNKPVIFTKSNLANSPELDAKMYDISYSTAAAPTYFPPHYFVTNTSNGDEYEFNLVDGAVATVADPALLSISVATRLAQKDPAFASIRSLNYKKMLLLSLGTGTTSEFDKTYTAKEAATWTAVHWMLVIQKMTDAASSYMTDYYLSTAFQALDSKNNYLRVQENALTGTTTEMDDASEANMELLVQVGENLLKKPVSEDNPETYEEALKRFAKLLSDRKKLRANKASY

>1HYOA

GSMSFIPVAEDSDFPIQNLPYGVFSTQSNPKPRIGVAIGDQILDLSVIKHLFTGPALSKHQHVFDETTLNNFMGLGQAAWKEARASLQNLLSASQARLRDDKELRQRAFTSQASATMHLPATIGDYTDFYSSRQHATNVGIMFRGKENALLPNWLHLPVGYHGRASSIVVSGTPIRRPMGQMRPDNSKPPVYGACRLLDMELEMAFFVGPGNRFGEPIPISKAHEHIFGMVLMNDWSARDIQQWEYVPLGPFLGKSFGTTISPWVVPMDALMPFVVPNPKQDPKPLPYLCHSQPYTFDINLSVSLKGEGMSQAATICRSNFKHMYWTMLQQLTHHSVNGCNLRPGDLLASGTISGSDPESFGSMLELSWKGTKAIDVGQGQTRTFLLDGDEVIITGHCQGDGYRVGFGQCAGKVLPALSPA

>1JB0K

MVLATLPDTTWTPSVGLVVILCNLFAIALGRYAIQSRGKGPGLPIALPALFEGFGLPELLATTSFGHLLAAGVVSGLQYAGAL

>4ELNA

MGSSHHHHHHSSGLVPRGSHMGLCTSKPSVVGSPVAGSPEHYLTHTAEQTTPSTPSSPEAPMSPSLHGLAALGSPRASSSPRPLSPLVELNTSDLIKQKKQLWQRVQHDGAQFRSTPEERKQFKTALITLWGEQYRPERQQRWNGMMQRMAQMKWNHPELKYMATEDLVALQAWTTDDYEVVQDVLEKEARPTAHGLAFAKCIISALHSLPEEYSYQGTVFTGEDQLPDWVSERYQERSITTDRRFFAASETKNASWQGMAVEWESNSTTGKRISMFSERPNEQEVLFPPGTRFQVTRIEENETHPRLKIYQSQIA

>3LMBA

MNASLTPDQVSKKLKQFFSDHLPISQFMGLEIESYDGDTLILTAPLEPNINDKQTAFGGSLYNAAVMACWGMVYLKTQEENIACNQVVTEGNMKYIAPVYGRIRAICHAPDEEELANFFDHFERKGKARISLEAAIYNDACVMKIEPETKPSVKFNGQYAILKNQ

>3Q1CA

SHMASSWDEMSCAEKLLKVLSFGLWNPTYSRSERQSFQELLTVLEPVYPLPNELGRVSARFSDGSSLRISVTNSESIEAEIRTPDNEKITVLLESNEQNRLLQSLPIDRHMPYIQVHRALSEMDLTDTTSMRNLLGFTSKLSTTLIPHNAQTDPLSGPTPFSSIFMDTCRGLGNAKLSLNGVDIPANAQMLLRDALGLKDTHSSPSRNVIDHGISRHDAEQIARESSGSDNQKAEVVEFLCHPEAATAICSAFYQSFNVPALTLTHERISKASEYNAERSLDTPNACINISISQSSDGNIYVTSHTGVLIMAPEDRPNEMGMLTNRTSYEVPQGVKCTIDEMVRALQPRYAASETYLQNT

>2OXGB

MRGSHHHHHHGSSTVDELTAAFTGGAATGEGGLTLTAPEIAENGNTVPIEVKAPGAVAIMLLAAGNPEPAVATFNFGPAAADQRAATRIRLAQTQDVIALAKMADGSVVKAQTTVKVTIGGCGG

>2WE5A

MGKKMVVALGGNAILSNDASAHAQQQALVQTSAYLVHLIKQGHRLIVSHGNGPQVGNLLLQQQAADSEKNPAMPLDTCVAMTQGSIGYWLSNALNQELNKAGIKKQVATVLTQVVVDPADEAFKNPTKPIGPFLTEAEAKEAMQAGAIFKEDAGRGWRKVVPSPKPIDIHEAETINTLIKNDIITISCGGGGIPVVGQELKGVEAVIDKDFASEKLAELVDADALVILTGVDYVCINYGKPDEKQLTNVTVAELEEYKQAGHFAPGSMLPKIEAAIQFVESQPNKQAIITSLENLGSMSGDEIVGTVVTK

>4ENEA

MRRRQLIRQLLERDKTPLAILFMAAVVGTLVGLAAVAFDKGVAWLQNQRMGALVHTADNYPLLLTVAFLCSAVLAMFGYFLVRKYAPEAGGSGIPEIEGALEDQRPVRWWRVLPVKFFGGLGTLGGGMVLGREGPTVQIGGNIGRMVLDIFRLKGDEARHTLLATGAAAGLAAAFNAPLAGILFIIEEMRPQFRYTLISIKAVFIGVIMSTIMYRIFNHEVALIDVGKLSDAPLNTLWLYLILGIIFGIFGPIFNKWVLGMQDLLHRVHGGNITKWVLMGGAIGGLCGLLGFVAPATSGGGFNLIPIATAGNFSMGMLVFIFVARVITTLLCFSSGAPGGIFAPMLALGTVLGTAFGMVAVELFPQYHLEAGTFAIAGMGALLAASIRAPLTGIILVLEMTDNYQLILPMIITGLGATLLAQFTGGKPLYSAILARTLAKQEAEQK

>3UC2A

MGSDKIHHHHHHENLYFQGEQVQRFGDLDVHYNVFNSSFLQPNVASAVGLVRSKAQGVINVVPMEKGKPVEAAVTGSAKDLTGKVIPLEFRRVSEEGAIYNLAQFPISQRETLVFTIKVEAKGEPAQTFSFNKEIFPDE

>2VTWA

TSSVAAFTSGTIGLSSPTGNFVSSSNNPFNGSYFLQQINTMGMLTTSLYVKVDTTTMGTRPTGAVNENARYFTVWVSSFLTQCNPSNIGQGTLEPSNISMTSFEPARNPISPPVFNMNQNIPYYASRFGVLESYRPIFTGSLNTGSIDVRMQVTPVLATNNTTYNLIAFTFQCASAGLFNPTVNGTVAIGPVVHTCPAARAPVTV

>2XSUA

MHHHHHHNRQQIDALVKQMNVDTAKGPVDERIQQVVVRLLGDLFQAIEDLDIQPSEVWKGLEYLTDAGQANELGLLAGGLGLEHYLDLRADEADAKAGITGGTPRTIEGPLYVAGAPESVGFARMDDGSESDKVDTLIIEGTVTDTEGNIIEGAKVEVWHANSLGNYSFFDKSQSDFNLRRTILTDVNGKYVALTTMPVGYGCPPEGTTQALLNKLGRHGNRPSHVHYFVSAPGYRKLTTQFNIEGDEYLWDDFAFATRDGLVATATDVTDEAEIARRELDKPFKHITFNVELVKEAEAAPSSEVERRRASA

>3EDOA

GMAKKTLILYYSWSGETKKMAEKINSEIKDSELKEVKVSEGTFDADMYKTSDIALDQIQGNKDFPEIQLDNIDYNNYDLILIGSPVWSGYPATPIKTLLDQMKNYRGEVASFFTSAGTNHKAYVSHFNEWADGLNVIGVARDDSEVDKWSK

>2FH1A

MDDDGTGQKQIWRIEGSNKVPVDPATYGQFYGGDSYIILYNYRHGGRQGQIIYNWQGAQSTQDEVAASAILTAQLDEELGGTPVQSRVVQGKEPAHLMSLFGGKPMIIYKGGTSREGGQTAPASTRLFQVRANSAGATRAVEVLPKAGALNSNDAFVLKTPSAAYLWVGTGASEAEKTGAQELLRVLRAQPVQVAEGSEPDGFWEALGGKAAYRTSPRLKDKKMDAHPPRLFACSNKIGRFVIEEVPGELMQEDLATDDVMLLDTWDQVFVWVGKDSQEEEKTEALTSAKRYIETDPANRDRRTPITVVKQGFEPPSFVGWFLGWDDDYWSVDPLDRAMAELAA

>2Q4MA

MEQPYVYAYPQGSGPSGAPTPQAGGVVVDPKYCAPYPIDMAIVRKMMSLTDGNFVITDVNGNLLFKVKEPVFGLHDKRVLLDGSGTPVVTLREKMVSMHDRWQVFRGGSTDQRDLLYTVKRSSMLQLKTKLDVFLGHNKDEKRCDFRVKGSWLERSCVVYAGESDAIVAQMHRKHTVQSVFLGKDNFSVTVYPNVDYAFIASLVVILDDVNREDRAA

>1HUXA

MSIYTLGIDVGSTASKCIILKDGKEIVAKSLVAVGTGTSGPARSISEVLENAHMKKEDMAFTLATGYGRNSLEGIADKQMSELSCHAMGASFIWPNVHTVIDIGGQDVKVIHVENGTMTNFQMNDKCAAGTGRFLDVMANILEVKVSDLAELGAKSTKRVAISSTCTVFAESEVISQLSKGTDKIDIIAGIHRSVASRVIGLANRVGIVKDVVMTGGVAQNYGVRGALEEGLGVEIKTSPLAQYNGALGAALYAYKKAAKSAWSHPQFEK

>3N2NA

SMACYGGFDLYFILDKSGSVLHHWNEIYYFVEQLAHKFISPQLRMSFIVFSTRGTTLMKLTEDREQIRQGLEELQKVLPGGDTYMHEGFERASEQIYYENRQGYRTASVIIALTDGELHEDLFFYSEREANRSRDLGAIVYAVGVKDFNETQLARIADSKDHVFPVNDGFQALQGIIHSILKKSC

>1V5VA

MIQMVKRVHIFDWHKEHARKIEEFAGWEMPIWYSSIKEEHLAVRNAVGIFDVSHMGEIVFRGKDALKFLQYVTTNDISKPPAISGTYTLVLNERGAIKDETLVFNMGNNEYLMICDSDAFEKLYAWFTYLKRTIEQFTKLDLEIELKTYDIAMFAVQGPKARDLAKDLFGIDINEMWWFQARWVELDGIKMLLSRSGYTGENGFEVYIEDANPYHPDESKRGEPEKALHVWERILEEGKKYGIKPCGLGARDTLRLEAGYTLYGNETKELQLLSTDIDEVTPLQANLEFAIYWDKDFIGKDALLKQKERGVGRKLVHFKMIDKGIPREGYKVYANGEMIGEVTSGTLSPLLNVGIGIAFVKEEYAKPGIEIEVEIRGQRKKAVTVTPPFYDPKKYGLFRET

>2DDRA

EVSTTQNDTLKVMTHNVYMLSTNLYPNWGQTERADLIGAADYIKNQDVVILNEVFDNSASDRLLGNLKKEYPNQTAVLGRSSGSEWDKTLGNYSSSTPEDGGVAIVSKWPIAEKIQYVFAKGCGPDNLSNKGFVYTKIKKNDRFVHVIGTHLQAEDSMCGKTSPASVRTNQLKEIQDFIKNKNIPNNEYVLIGGDMNVNKINAENNNDSEYASMFKTLNASVPSYTGHTATWDATTNSIAKYNFPDSPAEYLDYIIASKDHANPSYIENKVLQPKSPQWTVTSWFQKYTYNDYSDHYPVEATISMK

>3VATA

MMSYYHHHHHHDYDIPTTENLYFQGAMGRARKEAVQAAARELLKFVNRSPSPFHAVAECRSRLLQAGFHELKETESWDIKPESKYFLTRNSSTIIAFAVGGQYVPGNGFSLIGAHTDSPCLRVKRRSRRSQVGFQQVGVETYGGGIWSTWFDRDLTLAGRVIVKCPTSGRLEQRLVHVDRPILRIPHLAIHLQRNVNENFGPNMEMHLVPILATSIQEELEKGTPEPGPLNATDERHHSVLTSLLCAHLGLSPEDILEMELCLADTQPAVLGGAYEEFIFAPRLDNLHSCFCALQALIDSCSAPASLAADPHVRMIALYDNEEVGSESAQGAQSLLTELVLRRISASPQHLTAFEEAIPKSYMISADMAHAVHPNYLDKHEENHRPLFHKGPVIKVNSKQRYASNAVSEALIREVASSVGVPLQDLMVRNDSPCGTTIGPILASRLGLRVLDLGSPQLAMHSIRETACTTGVLQTITLFKGFFELFPSLSRSLLVD

>3EOJA

ALFGTKDTTTAHSDYEIILEGGSSSWGQVKGRAKVNVPAAIPLLPTDCNIRIDAKPLDAQKGVVRFTTKIESVVDSVKNTLNVEVDIANETKDRRIAVGEGSLSVGDFSHSFSFEGSVVNMYYYRSDAVRRNIPNPIYMQGRQFHDILMKVPLDNNDLVDTWEGFQQSISGGGANFGDWIREFWFIGPAFAAINEGGQRISPIVVNSSNVEGGEKGPVGVTRWKFSHAGSGVVDSISRWTELFPVEQLNKPASIEGGFRSDSQGIEVKVDGNLPGVSRDAGGGLRRILNHPLIPLVHHGMVGKFNDFTVDTQLKIVLPKGYKIRYAAPQFRSQNLEEYRWSGGAYARWVEHVCKGGTGQFEVLYAQ

>1K4ZA

MPPRKELVGNKWFIENYENETESLVIDANKDESIFIGKCSQVLVQIKGKVNAISLSETESCSVVLDSSISGMDVIKSNKFGIQVNHSLPQISIDKSDGGNIYLSKESLNTEIYTSCSTAINVNLPIGEDDDYVEFPIPEQMKHSFADGKFKSAVFEHAG

>4IQ0A

SNAMLKLGVIGTGAISHHFIEAAHTSGEYQLVAIYSRKLETAATFASRYQNIQLFDQLEVFFKSSFDLVYIASPNSLHFAQAKAALSAGKHVILEKPAVSQPQEWFDLIQTAEKNNCFIFEAARNYHEKAFTTIKNFLADKQVLGADFNYAKYSSKMPDLLAGQTPNVFSDRFAGGALMDLGIYPLYAAVRLFGKANDATYHAQQLDNSIDLNGDGILFYPDYQVHIKAGKNITSNLPCEIYTTDGTLTLNTIEHIRSAIFTDHQGNQVQLPIQQAPHTMTEEVAAFAHMIQQPDLNLYQTWLYDAGSVHELLYTMRQTAGIRFEAEK

>1G6HA

MRDTMEILRTENIVKYFGEFKALDGVSISVNKGDVTLIIGPNGSGKSTLINVITGFLKADEGRVYFENKDITNKEPAELYHYGIVRTFQTPQPLKEMTVLENLLIGEICPGESPLNSLFYKKWIPKEEEMVEKAFKILEFLKLSHLYDRKAGELSGGQMKLVEIGRALMTNPKMIVMDEPIAGVAPGLAHDIFNHVLELKAKGITFLIIEHRLDIVLNYIDHLYVMFNGQIIAEGRGEEEIKNVLSDPKVVEIYIGE

>2QISA

GSSHHHHHHSSGRENLYFQGHMNGDQNSDVYAQEKQDFVQHFSQIVRVLTEDEMGHPEIGDAIARLKEVLEYNAIGGKYNRGLTVVVAFRELVEPRKQDADSLQRAWTVGWCVELLQAFFLVADDIMDSSLTRRGQICWYQKPGVGLDAINDANLLEACIYRLLKLYCREQPYYLNLIELFLQSSYQTEIGQTLDLLTAPQGNVDLVRFTEKRYKSIVKYKSAFYSFYLPIAAAMYMAGIDGEKEHANAKKILLEMGEFFQIQDDYLDLFGDPSVTGKIGTDIQDNKCSWLVVQCLQRATPEQYQILKENYGQKEAEKVARVKALYEELDLPAVFLQYEEDSYSHIMALIEQYAAPLPPAVFLGLARKIYKRRK

>4HHRA

MRGSHHHHHHGSMKVITSLISSILLKFIHKDFHEIYARMSLLDRFLLLIVHGVDKMVPWHKLPVFLGLTYLEVRRHLHQQYNLLNVGQTPTGIRFDPANYPYRTADGKFNDPFNEGVGSQNSFFGRNCPPVDQKSKLRRPDPMVVATKLLGRKKFIDTGKQFNMIAASWIQFMIHDWIDHLEDTHQIELVAPKEVASKCPLSSFRFLKTKEVPTGFFEIKTGSQNIRTPWWDSSVIYGSNSKTLDRVRTYKDGKLKISEETGLLLHDEDGLAISGDIRNSWAGVSALQALFIKEHNAVCDALKDEDDDLEDEDLYRYARLVTSAVVAKIHTIDWTVQLLKTDTLLAGMRANWYGLLGKKFKDSFGHAGSSILGGVVGMKKPQNHGVPYSLTEDFTSVYRMHSLLPDQLHILDIDDVPGTNKSLPLIQEISMRDLIGRKGEETMSHIGFTKLMVSMGHQASGALELMNYPMWLRDIVPHDPNGQARPDHVDLAALEIYRDRERSVPRYNEFRRSMFMIPITKWEDLTEDEEAIEVLDDVYDGDVEELDLLVGLMAEKKIKGFAISETAFYIFLIMATRRLEADRFFTSDFNETIYTKKGLEWVNTTESLKDVIDRHYPDMTDKWMNSESAFSVWDSPPLTKNPIPLYLRIPSR

>3MWCA

MSLTESARIDGVSLYEIVIPMKIPFQISSGTCYTRRSLVVEIREGDLFGYGESAPFEEPFYLGETLETTKVILKNHLLPMILGKEPLSIEEFNHLIKNGIRGNHFARCGVENAYWDLIAKKNKISLKAMIEKKMKNLGVKQEYLASNNYIESGAALGIPEDGRIETLIHQVEESLQEGYRRIKIKIKPGWDVEPLQETRRAVGDHFPLWTDANSSFELDQWETFKAMDAAKCLFHEQPLHYEALLDLKELGERIETPICLDESLISSRVAEFVAKLGISNIWNIKIQRVGGLLEAIKIYKIATDNGIKLWGGTMPESGLGARFLISLASFRGFVFPADVAASEKWYGKGNDLVENTMTDGKIYVPDEPGASFDMTLSHLEALGKKIWESQRGEGHHHHHH

>2FEXA

MTRIAIALAQDFADWEPALLAAAARSYLGVEIVHATPDGMPVTSMGGLKVTPDTSYDALDPVDIDALVIPGGLSWEKGTAADLGGLVKRFRDRDRLVAGICAAASALGGTGVLNDVAHTGNALASHKAYPAYRGEAHYRDQPRAVSDGGVVTAAGSAPVSFAVEILKSLGLFGPEAEAELQIFAAEHR

>2PNQA

ASTPQKFYLTPPQVNSILKANEYSFKVPEFDGKNVSSILGFDSNRLPANAPIEDRRSATTCLQTRGMLLGVFDGHAGCACSQAVSERLFYYIAVSLLPHETLLEIENAVESGRALLPILQWHKHPNDYFSKEASKLYFNGLRTYWQELIDLNTGESADIDVKEALINAFKRLDNDISLEAQVGDPNSFLNYLVLRVAFSGATACVAHVDGVDLHVANTGDSRAMLGVQEEDGSWSAVTLSNDHNAQNERELQRLKLEHPKNEAKSVVKQDRLLGLLMPFRAFGDVKFKWSIDLQKRVIESGPDQLNDNEYTKFIPPNYHTPPYLTAEPEVTYHRLRPQDKFLVLATDGLWETMHRQDVVRIVGEYLTGMHHQQPIAVGGYKVTLGQMHGLLTERRAKMSSVFEDQNAATHLIRHAVGNNEFGAVDHERLSKMLSLPEELARMYRDDITIIVVQFNSHVVGAYQNQEQ

>1YQGA

MNVYFLGGGNMAAAVAGGLVKQGGYRIYIANRGAEKRERLEKELGVETSATLPELHSDDVLILAVKPQDMEAACKNIRTNGALVLSVAAGLSVGTLSRYLGGTRRIVRVMPNTPGKIGLGVSGMYAEAEVSETDRRIADRIMKSVGLTVWLDDEEKMHGITGISGSGPAYVFYLLDALQNAAIRQGFDMAEARALSLATFKGAVALAEQTGEDFEKLQKNVTSKGGTTHEAVEAFRRHRVAEAISEGVCACVRRSQEMERQYQ

>3T4LA

MDDANKIRREEVLVSMCDQRARMLQDQFSVSVNHVHALAILVSTFHYHKNPSAIDQETFAEYTARTAFERPLLSGVAYAEKVVNFEREMFERQHNWVIKTMDRGEPSPVRDEYAPVIFSQDSVSYLESLDMMSGEEDRENILRARETGKAVLTSPFRLLETHHLGVVLTFPVYKSSLPENPTVEERIAATAGYLGGAFDVESLVENLLGQLAGNQAIVVHVYDITNASDPLVMYGNQDEEADRSLSHESKLDFGDPFRKHKMICRYHQKA

>1NJHA

SNAMKAIIKEDVQASLERYADRPVYIHLETTTGSYSAHLNEKNMTVVAYIRNAKVTYHQAKIKGNGPYRVGLKTEEGWIYAEGLTEYTVDEENRLLMAGHLPGGKLAISLQISEKPFTV

>2WLUA

MTNTLVENIYASVTHNISKKEASKNEKTKAVLNQAVADLSVAASIVHQVHWYMRGPGFLYLHPKMDELLDSLNANLDEVSERLITIGGAPYSTLAEFSKHSKLDEAKGTYDKTVAQHLARLVEVYLYLSSLYQVGLDITDEEGDAGTNDLFTAAKTEAEKTIWMLQAERGQGPAL

>3A8GA

MSVTIDHTTENAAPAQAPVSDRAWALFRALDGKGLVPDGYVEGWKKTFEEDFSPRRGAELVARAWTDPEFRQLLLTDGTAAVAQYGYLGPQGEYIVAVEDTPTLKNVIVCSLCACTAWPILGLPPTWYKSFEYRARVVREPRKVLSEMGTEIASDIEIRVYDTTAETRYMVLPQRPAGTEGWSQEQLQEIVTKDCLIGVAIPQVPTV

>3T9OA

MAIKKTTEIDAILLNLNKAIDAHYQWLVSMFHSVVARDASKPEITDNHSYGLCQFGRWIDHLGPLDNDELPYVRLMDSAHQHMHNCGRELMLAIVENHWQDAHFDAFQEGLLSFTAALTDYKIYLLTLEHHHHHH

>3HZ7A

MITIDALGQVCPIPVIRAKKALAELGEAGGVVTVLVDNDISRQNLQKMAEGMGYQSEYLEKDNGVIEVTIVAGEGCAVELEHHHHHH

>3H6PC

MSQIMYNYPAMMAHAGDMAGYAGTLQSLGADIASEQAVLSSAWQGDTGITYQGWQTQWNQALEDLVRAYQSMSGTHESNTMAMLARDGAEAAKWGG

>4EO0A

MDNWESITKSYYTGFAISKTVESKDKDGKPVRKEVITQADLTTACNDAKASAQNVFNQIKLTLSGTWPNSQFRLVTGDTCVYNGSPGEKTESWSIRAQVEGDIQRSVPDHHHHHH

>2QW5A

GMTKLPATSDIYISFFMFTTNLQPDNLDYRRIVVAHIKKLQRFGYSGFEFPIAPGLPENYAQDLENYTNLRHYLDSEGLENVKISTNVGATRTFDPSSNYPEQRQEALEYLKSRVDITAALGGEIMMGPIVIPYGVFPTTDFNEPIWSDELQEHLKVRYANAQPILDKLGEYAEIKKVKLAIEPITHWETPGPNKLSQLIEFLKGVKSKQVGVVIDSAHEILDGEGPEIFKTQVEYLAQQGRLHYVQVSPPDRGALHTSWLPWKSFLTPIVKVYDGPIAVEIFNAIPAFTNSLRLTRRKFWIPDEDPPNQYPNAYDIADEAIKVTRKELKKIGSK

>2J9OA

MPAPEAPTSTLPPERPLTNLQQQIQQLVSRQPNLTAGLYFFNLDSGASLNVGGDQVFPAASTIKFPILVAFFKAVDEGRVTLQERLTMRPDLIAPEAGTLQYQKPNSQYAALEVAELMITISDNTATNMIIDRLGGAAELNQQFQEWGLENTVINNPEPDMKGTNTTSPRDLATLMLKIGQGEILSPRSRDRLLDIMRRTVTNTLLPAGLGKGATIAHKTGDIGIVVGDAGMVDMPNGQRYVAAMMVKRPYNDPRGSELIRQVSRMVYQAFEKLSPPEQKLISEEDLNSAVDHHHHHH

>3ER6A

MSLTNKKNLRVVALAPTGRYFASIISSLEILETAAEFAEFQGFMTHVVTPNNRPLIGRGGISVQPTAQWQSFDFTNILIIGSIGDPLESLDKIDPALFDWIRELHLKGSKIVAIDTGIFVVAKAGLLQQNKAVMHSYFAHLFGELFPEIMLMTEQKALIDGNVYLSSGPYSHSSVMLEIVEEYFGKHTRNLGNQFLSTIESEGHHHHHH

>3VHJA

MVKNNLGVAVIGSKQYAVNLLWGSSQDTETTNQALNKSLTLMSSKLYSVIGRFQGEQFAVGDKNIGHKRGQVTLLSAIDFDGSSFCGLFPADNELWLVIGVDKDGMVHFDKSFHSKDDAKKFFFDHVAYGYPWDRTYSPSDVGVGESRSISELSLIKGKKLKEKGSHHHHHH

>3AMLA

MVTVVEEVDHLPIYDLDPKLEEFKDHFNYRIKRYLDQKCLIEKHEGGLEEFSKGYLKFGINTVDGATIYREWAPAAQEAQLIGEFNNWNGAKHKMEKDKFGIWSIKISHVNGKPAIPHNSKVKFRFRHGGGAWVDRIPAWIRYATFDASKFGAPYDGVHWDPPACERYVFKHPRPPKPDAPRIYEAHVGMSGEEPEVSTYREFADNVLPRIRANNYNTVQLMAIMEHSYYASFGYHVTNFFAVSSRSGTPEDLKYLVDKAHSLGLRVLMDVVHSHASNNVTDGLNGYDVGQNTHESYFHTGDRGYHKLWDSRLFNYANWEVLRFLLSNLRYWMDEFMFDGFRFDGVTSMLYHHHGINKGFTGNYKEYFSLDTDVDAIVYMMLANHLMHKLLPEATIVAEDVSGMPVLCRPVDEGGVGFDFRLAMAIPDRWIDYLKNKEDRKWSMSEIVQTLTNRRYTEKCIAYAESHDQSIVGDKTIAFLLMDKEMYTGMSDLQPASPTINRGIALQKMIHFITMALGGDGYLNFMGNEFGHPEWIDFPREGNNWSYDKCRRQWSLVDTDHLRYKYMNAFDQAMNALEEEFSFLSSSKQIVSDMNEKDKVIVFERGDLVFVFNFHPNKTYKGYKVGCDLPGKYRVALDSDALVFGGHGRVGHDVDHFTSPEGMPGVPETNFNNRPNSFKVLSPPRTCVAYYRVDEDREELRRGGAVASGKIVTEYIDVEATSGETISGGWKGSEKDDCGKKGMKFVFRSSDEDCK

>4E2UA

SQHMAFARDTEVYYENDTVPHMESIEEMYSKYASMNGELPFDNGYAVPLDNVFVYTLDIASGEIKKTRASYIYREKVEKLIEIKLSSGYSLKVTPSHPVLLFRDGLQWVPAAEVKPGDVVVGVRNGELEFHEVSSVRIIDYNNWVYDLVIPETHNFIAPNGLVLHNAQ

>1KXOA

DVYHDGACPEVKPVDNFDWSQYHGKWWQVAAYPDHITKYGKCGWAEYTPEGKSVKVSRYSVIHGKEYFSEGTAYPVGDSKIGKIYHSYTIGGVTQEGVFNVLSTDNKNYIIGYFCSYDEDKKGHMDLVWVLSRSMVLTGEAKTAVENYLIGSPVVDSQKLVYSDFSEAACKVNNSNWSHPQFEK

>3R41A

GHMPDLADLFPGFGSEWINTSSGRIFARVGGDGPPLLLLHGFPQTHVMWHRVAPKLAERFKVIVADLPGYGWSDMPESDEQHTPYTKRAMAKQLIEAMEQLGHVHFALAGHDRGARVSYRLALDSPGRLSKLAVLDILPTYEYWQRMNRAYALKIYHWSFLAQPAPLPENLLGGDPDFYVKAKLASWTRAGDLSAFDPRAVEHYRIAFADPMRRHVMCEDYRAGAYADFEHDKIDVEAGNKIPVPMLALWGASGIAQSAATPLDVWRKWASDVQGAPIESGNFLPEEAPDQTAEALVRFFSAAPGS

>1EL6A

MSLLNNKAGVISRLADFLGFRPKTGDIDVMNRQSVGSVTISQLAKGFYEPNIESAINDVHNFSIKDVGTIITNKTGVSPEGVSQTDYWAFSGTVTDDSLPPGSPITVLVFGLPVSATTGMTAIEFVAKVRVALQEAIASFTAINSYKDHPTDGSKLEVTYLDNQKHVLSTYSTYGITISQEIISESKPGYGTWNLLGAQTVTLDNQQTPTVFYHFERTA

>3FIAA

MGHHHHHHSHVAQFPTPFGGSLDTWAITVEERAKHDQQFHSLKPISGFITGDQARNFFFQSGLPQPVLAQIWALADMNNDGRMDQVEFSIAMKLIKLKLQGYQLPSALPPVMKQQPVAISS

>3VSVA

MEYHVAKTGSDEGKGTLKDPFLTINKAASVAMAGDTIIVHEGVYREWVKPKYKGLSDKRRITYKAAEGEKVVIKGSERIQSWQRVEGNVWRCQLPNSFFGEFNPYKEEVFGDWLLTVNEKKHLGDVYLNGMSFYEVTNYEDLFNPQLRTEVLDHWTQKIVPIKNAEQTKYVWYAEVDREKTTIYANFQGADPNEEFVEINVRRSCFYPVETGIDYITVKGFEMAHAATPWAPPTADQPGLIGPNWSKGWIIEDNIIHDAKCSAISIGKEATTGNNYRSIRKDKPGYQYQLEAVFNAKRNGWSKEKIGSHIIRNNTIYDCGQNAIVGHLGGVFSEIYNNHIYNIALKREFYGHEIAGIKLHAAIDVQIHHNRIHDCSLGLWLDWEAQGTRVSKNLFYNNNRDVFVEVSHGPYLVDHNILSSEYAIDNMSQGGAYINNLIAGKMNQRKVLNRSTQYHLPHSTEVAGFAFVYGGDDRFYNNIFIGKEGLENVGTSHYNNCTTSLEEYIEKVNEVPGDLGEFERVEQPVYINKNAYFNGAEPFEKEKDNLVKKDFDPKLAIIDEGDEVYLSLQLPDEFENIVGDIHSTKTLERVRIVDAEYESPDGKELVLDTDYLDAKKPENSSIGPIALLKKGNNYIKVW

>1EAYC

MSQSPRRIILSRLKAGEVDLLEEELGHLTTLTDVVKGADSLSAILPGDIAEDDITAVLCFVIEADQITFETVEV

>1Z6OM

TQCNVNPVQIPKDWITMHRSCRNSMRQQIQMEVGASLQYLAMGAHFSKDVVNRPGFAQLFFDAASEEREHAMKLIEYLLMRGELTNDVSSLLQVRPPTRSSWKGGVEALEHALSMESDVTKSIRNVIKACEDDSEFNDYHLVDYLTGDFLEEQYKGQRDLAGKASTLKKLMDRHEALGEFIFDKKLLGIDV

>2Z16A

GSSGSSGMSLLTEVETYVLSIIPSGPLKAEIAQKLEDVFAGKNTDLEALMEWLKTRPILSPLTKGILGFVFTLTVPSERGLQRRRFVQNALNGNGDPNNMDRAVKLYKKLKREITFHGAKEVALSYSTGALASCMGLIYNRMGTVTTEVAFGLVCATCEQIADSQ

>4FR9A

GGDDDDTGYLPPSQAIQDALKKLYPNATAIKWEQKGVYYVADCQADGREKEVWFDANANWLMTETELNSINNLPPAVLTAFMESSYNNWVVDDVVILEYPNEPSTEFVVTVEQGKKVDLYFSEGGGLLHEKDVTNGDDTHWPRV

>4HD5A

MRKYAAIALCTSAILAGCNTSNVSQEPKKEKKVQEVAIQKEALQEQGKISYTPITHESTNTSIHITDLKDSLNEVQYKIWRTADGKERAKSFSSKEKEKQFTIPFDIKEFEGKRGEFQIEATGMKEDGKTIPLTKSIITFEQKVPVLMYHAIDDYHGQGIKDLFVSPANFEAQMKHLKDNGYTLLTFERWGDINKVNKPIFVTFDDGMKNNMNAFRVLQKLKDDTFKPAATEYMIVDNVDVEGALSTSEIKEMVDSGIFSVQSHTATHADLPKITNYEEELKGSKEKLEKITGKPVIAIAYXFGHVDDKVVTETKKYYQFATTTKPGQFITKGEPDELLKMKRVRIHHTTTVEQFASSIK

>3BQXA

MSLQQVAVITLGIGDLEASARFYGEGFGWAPVFRNPEIIFYQMNGFVLATWLVQNLQEDVGVAVTSRPGSMALAHNVRAETEVAPLMERLVAAGGQLLRPADAPPHGGLRGYVADPDGHIWEIAFNPVWPIGADGSVTFAAKEGHHHHHH

>3CS3A

MSLKRRQTNIIGVYLADYGGSFYGELLEGIKKGLALFDYEMIVCSGKKSHLFIPEKMVDGAIILDWTFPTKEIEKFAERGHSIVVLDRTTEHRNIRQVLLDNRGGATQAIEQFVNVGSKKVLLLSGPEKGYDSQERLAVSTRELTRFGIPYEIIQGDFTEPSGYAAAKKILSQPQTEPVDVFAFNDEMAIGVYKYVAETNYQMGKDIRIIGFDNSELGAFVQPRLATIAYSKHRWGMVAAEKIIHLMRGEAAESEHIYTRFIEGESFPSEGHHHHHH

>4E3EA

MSAKTNPGNFFEDFRLGQTIVHATPRTITEGDVALYTSLYGSRFALTSSTPFAQSLGLERAPIDSLLVFHIVFGKTVPDISLNAIANLGYAGGRFGAVVYPGDTLSTTSKVIGLRQNKDGKTGVVYVHSVGVNQWDEVVLEYIRWVMVRKRDPNAPAPETVVPDLPDSVPVTDLTVPYTVSAANYNLAHAGSNYLWDDYEVGEKIDHVDGVTIEEAEHMQATRLYQNTARVHFNLHVEREGRFGRRIVYGGHIISLARSLSFNGLANALSIAAINSGRHTNPSFAGDTIYAWSEILAKMAIPGRTDIGALRVRTVATKDRPCHDFPYRDAEGNYDPAVVLDFDYTVLMPRRG

>1MG2D

APQFFNIIDGSPLNFDDAMEEGRDTEAVKHFLETGENVYNEDPEILPEAEELYAGMCSGCHGHYAEGKIGPGLNDAYWTYPGNETDVGLFSTLYGGATGQMGPMWGSLTLDEMLRTMAWVRHLYTGDPKDASWLTDEQKAGFTPFQPKSSGEDQS

>3LHIA

GMFVWHEYENAAEAAQSLADAVADALQGALDEKGGAVLAVSGGRSPIAFFNALSQKDLDWKNVGITLADERIVPTNHADSNTGLVREYLLKNKAAAAVWIPMVEDGKTETELHPDAVVDYALKHYKQPDVLILGMGNDGHTASIFPKAPQFQTAIDGSAGVALVHTTPVTAPHERISMTLDAIAHTGHVFLAIQGEEKKAVFDQAAQGENREYPISLVLNHQGVNCHVFYAE

>2VLQA

MELKHSISDYTEAEFLQLVTTICNADTSSEEELVKLVTHFEEMTEHPSGSDLIYYPKEGDDDSPSGIVNTVKQWRAANGKSGFKQG

>2CAYA

MAHHHHHHMEYWHYVETTSSGQPLLREGEKDIFIDQSVGLYHGKSKILQRQRGRIFLTSQRIIYIDDAKPTQNSLGLELDDLAYVNYSSGFLTRSPRLILFFKDPSSSTEFVQLSFRKSDGVLFSQATERALENILTEKNKHIFN

>3K8WA

TQNSLSTSLQRLSSGLRINSAKDDAAGLAISDRMTAQIKGLTQAQRNANDGISLAQTAEGALGEISNNLQRIRELAVQASNGTNTQTDRDALQAEVTQLQSEIQRVAEQTSFNGQKLLDGSFNGVQFQIGANAGETIGVSKIMNAQTASLGGSLTRTTSTIDATDLTKYDTAMAAGDLTINGVDVGKIDAASTAQERAAQLTEAINRVSSQTNVGASYDKTTGQVTLTSNAAIAVAGAANDATVAGWANNATTGTATTTTGINSLTVSSFTNAQQTITQIDNALKDINTARADLGAVQNRFTSTVANLQSMTENLSSALEHHHHHH

>2G38A

MSFVITNPEALTVAATEVRRIRDRAIQSDAQVAPMTTAVRPPAADLVSEKAATFLVEYARKYRQTIAAAAVVLEEFAHALTTGADKYATAEADNIKTFS

>3DDEA

GMSIIDLTKLEQKVATMWDSILTNSPFIHEVLDGKATKALYAIYMTETYHYTKHNAKNQALVGIMGKDLPGKYLSFCFHHAHEEAGHELMALSDIASIGFDREDVLSSKPLPATETLIAYLYWISATGNPVQRLGYSYWAENVYGYIDPVLKAIQSTLDLTPQSMKFFIAHSKIDAKHAEEVNEMLHEVCKTQEDVDSVVAVMENSLVLTARILDDVWKEYQLFQSGASDRYAFLRDNA

>4HVMA

SNAMTSLSSRPGLRRASFLQRGAWRWLREAPPAAAFAARGLLGSGRIDDDRLAAAADEVLDAFPLLRVNFVDDDGLWMRTRENADALVRSDLRGHPDPQARCVELLRADRDRPTDPERDPLVRLHLVRLSETDVVLGVVAHQMLLDARSRYMVLGAVWQAYYGRFRPAQYRDFAEVADFHPLDRETVRVARHRWWSRRLPALPVRGGDGGPVGPPETSRLRVPGSRWQALTEPGGPLGGNGSLAMAALTAWWLWTQGAGTGTDTGAGTGTGKDSLYLSTEVDLRDHLQLGSVVGPLTDRVVFGVDLTGLREPSFRDLMSRTQAGFLDAVVHYLPYHDVVDLAVDLGVVTPPRVAARWDVAVHLCRNAPSSSLTRGERTLAELGVSIELFREADLIGGDTRSATDTWDGTDTWDGTTTDLSVGELGEDMVIVLDQRRTHPAGGGSALLDGLDAAMAQAVADPSAPLPHSTVDTTTQSTVDKDTVDRNTAHENEE

>2D7VA

MSFGGKSMSEHSAIVTWKRKDSEAFTDNQYSRAHTWEFDGGSKILASASPHVVPVPLSVEANVDPEEAFVAALSSCHMLVFLSIAAKQRYLVESYTDNAVGILGKNSKGKTSVTKVVLRPQVVFSGTSKPTLQQLEKMHHLAHENCFIANSVETEVVTEIIA

>4A57A

MTDSSSLRGVDADTEKRINVGKKHLQTLRNLETRCHDSLQALVVIDAGSSSTRTNVFLAKTRSCPNKGRSIDPDSIQLIGAGKRFAGLRVVLEEWLDTYAGKDWESRPVDARLLFQYVPQMHEGAKKLMQLLEEDTVAILDSQLNEKQKVQVKALGIPVMLCSTAGVRDFHEWYRDALFVLLRHLINNPSPAHGYKFFTNPFWTRPITGAEEGLFAFITLNHLSRRLGEDPARCMIDEYGVKQCRNDLAGVVEVGGASAQIVFPLQEGTVLPSSVRAVNLQRERLLPERYPSADVVSVSFMQLGMASSAGLFLKELCSNDEFLQGGICSNPCLFKGFQQSCSAGEVEVRPDGSASVNEDVRKNRLKPLATYCSVNNPEISFKVTNEMQCRENSIDPTKPLAERMKIENCSIIKGTGNFDKCVSQVESILVAPKLPLPANIEAASSGFESVDQVFRFASSTAPMIVTGGGMLAAINTLKDHRLLRSDFSGDVEELAEAAREFCSSEVIIRTDGPVIQLPNARGEQKLNSLNFDLCKTMALTVSLLRHMAAGENQPSFIKWEKSIAGPDGKPLADLGWQVGVILHHVLFTEEWGRNAYEAGYSHNLEHHHHHH

>2BU3A

FCLTLRRRYTMGHHHHHHHHHHSSGHIEGRHMKLEQTLTLSPNLIGFNSNEGEKLLLTSRSREDFFPLSMQFVTQVNQAYCGVASIIMVLNSLGINAPETAQYSPYRVFTQDNFFSNEKTKAVIAPEVVARQGMTLDELGRLIASYGVKVKVNHASDTNIEDFRKQVAENLKQDGNFVIVNYLRKEIGQERGGHISPLAAYNEQTDRFLIMDVSRYKYPPVWVKTTDLWKAMNTVDSVSQKTRGFVFVSKTQDD

>2CW6A

TLPKRVKIVEVGPRDGLQNEKNIVSTPVKIKLIDMLSEAGLSVIETTSFVSPKWVPQMGDHTEVLKGIQKFPGINYPVLTPNLKGFEAAVAAGAKEVVIFGAASELFTKKNINCSIEESFQRFDAILKAAQSANISVRGYVSCALGCPYEGKISPAKVAEVTKKFYSMGCYEISLGDTIGVGTPGIMKDMLSAVMQEVPLAALAVHCHDTYGQALANTLMALQMGVSVVDSSVAGLGGCPYAQGASGNLATEDLVYMLEGLGIHTGVNLQKLLEAGNFICQALNRKTSSKVAQATCKL

>3A5FA

SIFKGSGVAIITPFTNTGVDFDKLSELIEWHIKSKTDAIIVCGTTGEATTMTETERKETIKFVIDKVNKRIPVIAGTGSNNTAASIAMSKWAESIGVDGLLVITPYYNKTTQKGLVKHFKAVSDAVSTPIIIYNVPGRTGLNITPGTLKELCEDKNIVAVKEASGNISQIAQIKALCGDKLDIYSGNDDQIIPILALGGIGVISVLANVIPEDVHNMCELYLNGKVNEALKIQLDSLALTNALFIETNPIPVKTAMNLMNMKVGDLRLPLCEMNENNLEILKKELKAYNLM

>1QWYA

AETTNTQQAHTQMSTQSQDVSYGTYYTIDSNGDYHHTPDGNWNQAMFDNKEYSYTFVDAQGHTHYFYNCYPKNANANGSGQTYVNPATAGDNNDYTASQSQQHINQYGYQSNVGPDASYYSHSNNNQAYNSHDGNGKVNYPNGTSNQNGGSASKATASGHAKDASWLTSRKQLQPYGQYHGGGAHYGVDYAMPENSPVYSLTDGTVVQAGWSNYGGGNQVTIKEANSNNYQWYMHNNRLTVSAGDKVKAGDQIAYSGSTGNSTAPHVHFQRMSGGIGNQYAVDPTSYLQSR

>3AMRA

KLSDPYHFTVNAAAETEPVDTAGDAADDPAIWLDPKTPQNSKLITTNKKSGLVVYSLDGKMLHSYNTGKLNNVDIRYDFPLNGKKVDIAAASNRSEGKNTIEIYAIDGKNGTLQSMTDPDHPIATAINEVYGFTLYHSQKTGKYYAMVTGKEGEFEQYELKADKNGYISGKKVRAFKMNSQTEGMAADDEYGRLYIAEEDEAIWKFSAEPDGGSNGTVIDRADGRHLTRDIEGLTIYYAADGKGYLMASSQGNSSYAIYDRQGKNKYVADFRITDGPETDGTSDTDGIDVLGFGLGPEYPFGIFVAQDGENIDHGQKANQNFKIVPWERIADQIGFRPLANEQVDPRKLTDRSGK

>4DX5A

MPNFFIDRPIFAWVIAIIIMLAGGLAILKLPVAQYPTIAPPAVTISASYPGADAKTVQDTVTQVIEQNMNGIDNLMYMSSNSDSTGTVQITLTFESGTDADIAQVQVQNKLQLAMPLLPQEVQQQGVSVEKSSSSFLMVVGVINTDGTMTQEDISDYVAANMKDAISRTSGVGDVQLFGSQYAMRIWMNPNELNKFQLTPVDVITAIKAQNAQVAAGQLGGTPPVKGQQLNASIIAQTRLTSTEEFGKILLKVNQDGSRVLLRDVAKIELGGENYDIIAEFNGQPASGLGIKLATGANALDTAAAIRAELAKMEPFFPSGLKIVYPYDTTPFVKISIHEVVKTLVEAIILVFLVMYLFLQNFRATLIPTIAVPVVLLGTFAVLAAFGFSINTLTMFGMVLAIGLLVDDAIVVVENVERVMAEEGLPPKEATRKSMGQIQGALVGIAMVLSAVFVPMAFFGGSTGAIYRQFSITIVSAMALSVLVALILTPALCATMLKPIAKGDHGEGKKGFFGWFNRMFEKSTHHYTDSVGGILRSTGRYLVLYLIIVVGMAYLFVRLPSSFLPDEDQGVFMTMVQLPAGATQERTQKVLNEVTHYYLTKEKNNVESVFAVNGFGFAGRGQNTGIAFVSLKDWADRPGEENKVEAITMRATRAFSQIKDAMVFAFNLPAIVELGTATGFDFELIDQAGLGHEKLTQARNQLLAEAAKHPDMLTSVRPNGLEDTPQFKIDIDQEKAQALGVSINDINTTLGAAWGGSYVNDFIDRGRVKKVYVMSEAKYRMLPDDIGDWYVRAADGQMVPFSAFSSSRWEYGSPRLERYNGLPSMEILGQAAPGKSTGEAMELMEQLASKLPTGVGYDWTGMSYQERLSGNQAPSLYAISLIVVFLCLAALYESWSIPFSVMLVVPLGVIGALLAATFRGLTNDVYFQVGLLTTIGLSAKNAILIVEFAKDLMDKEGKGLIEATLDAVRMRLRPILMTSLAFILGVMPLVISTGAGSGAQNAVGTGVMGGMVTATVLAIFFVPVFFVVVRRRFSRKNEDIEHSHTVDHHLEHHHHHH

>4A6RA

MQKQRTTSQWRELDAAHHLHPFTDTASLNQAGARVMTRGEGVYLWDSEGNKIIDGMAGLWCVNVGYGRKDFAEAARRQMEELPFYNTFFKTTHPAVVELSSLLAEVTPAGFDRVFYTNSGSESVDTMIRMVRRYWDVQGKPEKKTLIGRWNGYHGSTIGGASLGGMKYMHEQGDLPIPGMAHIEQPWWYKHGKDMTPDEFGVVAARWLEEKILEIGADKVAAFVGEPIQGAGGVIVPPATYWPEIERICRKYDVLLVADEVICGFGRTGEWFGHQHFGFQPDLFTAAKGLSSGYLPIGAVFVGKRVAEGLIAGGDFNHGFTYSGHPVCAAVAHANVAALRDEGIVQRVKDDIGPYMQKRWRETFSRFEHVDDVRGVGMVQAFTLVKNKAKRELFPDFGEIGTLCRDIFFRNNLIMRACGDHIVSAPPLVMTRAEVDEMLAVAERCLEEFEQTLKARGLA

>1TUKA

ACQASQLAVCASAILSGAKPSGECCGNLRAQQGCFCQYAKDPTYGQYIRSPHARDTLTSCGLAVPHC

>4KF8A

SLLPKDISQLLSLVSATINGVDNPWSKDQISYFRTLLKVLFVVLRGTKHSNNAAPQKPTAESPVAVTQLVLTTLDRVVARSFRNLAALVHEPDAATTPEDLALITAILQACLSVPGIEQCQLQVLNIMSSHNVLQVATSLFSWSDRLAEKGDPIYGELALLLLLELSALPALAEQLACDGLLGHLTSANLAGFMRRANVSPFTDNAGAARCYAIWAKGILPLLLNILGALGATIAPEVAFVLNQFPNLLRSSVDRLEAPGLSRTVPLSSRDAPGAGPHYFVALVALSEVHSLALLTRVLAALRSGNARDIPEVVWDSGAVLENVEFWLASRKVLRERLLPLNPREAEWRGMKASEGSGCETKLEEKAVGLLEGIRDVLAEEEE

>1FSGA

GSHMASKPIEDYGKGKGRIEPMYIPDNTFYNADDFLVPPHCKPYIDKILLPGGLVKDRVEKLAYDIHRTYFGEELHIICILKGSRGFFNLLIDYLATIQKYSGRESSVPPFFEHYVRLKSYQNDNSTGQLTVLSDDLSIFRDKHVLIVEDIVDTGFTLTEFGERLKAVGPKSMRIATLVEKRTDRSNSLKGDFVGFSIEDVWIVGCCYDFNEMFRDFDHVAVLSDAARKKFEK

>1EW4A

MNDSEFHRLADQLWLTIEERLDDWDGDSDIDCEINGGVLTITFENGSKIIINRQEPLHQVWLATKQGGYHFDLKGDEWICDRSGETFWDLLEQAATQQAGETVSFR

>2GUJA

MALKAQNTISGKEGRLFLDGEEMAHIKTFEANVEKNKSEVNIMGRRMTGHKTTGANGTGTATFYKVTSKFVLLMMDYVKKGSDPYFTLQAVLDDQSSGRGTERVTLYDVNFDSAKIASLDVDSEALEEEVPFTFEDFDVPEKLSDTFLEHHHHHH

>1O13A

MGSDKIHHHHHHMIIAIPVSENRGKDSPISEHFGRAPYFAFVKVKNNAIADISVEENPLAQDHVHGAVPNFVKEKGAELVIVRGIGRRAIAAFEAMGVKVIKGASGTVEEVVNQYLSGQLKDSDYEVHDHHHHEHH

>3NBCA

SITPGTYNITNVAYTNRLIDLTGSNPAENTLIIGHHLNKTPSGYGNQQWTLVQLPHTTIYTMQAVNPQSYVRVRDDNLVDGAALVGSQQPTPVSIESAGNSGQFRIKIPNLGLALTLPSDANSTPIVLGEVDETSTNQLWAFESVSAV

>2X0QA

MSRTTPPHPAEIVAHLQPEIWNKVNRLLVRKAISEYAHEWLLEPQRLGPGETPGFERFRLTLADGAQYDFDAQVMAMRHWRIPPESIVKTVAGVPAPLDALQFVIEIRDKLGLPVDRLPIYMDEITSTLHGSAYKHGRTTLGAAALARADYQTIETSMIEGHPSFVANNGRLGFDAEDYHGYAPEAATPVRLMWLAVHKDNAHFSCLSDMDYDSLMSEELGESAVTDFAARLREQGLHPADYYFMPAHPWQWFNKLSLAFAPYVAQRKIVCLGYGEEQYLAQQSIRTFFNISRPGKRYVKTSLSILNMGFMRGLSPYYMAGTPAINEYIHDLISADPWLRANGFRILREVASMGFRNYYYEAAIDTDTPYKKMFSALWRENPLTLIAPGQNLMTMAALLHVDPQGRALLPELIQASGLDAGTWLERYVDAYLTPLIHCFYAHDLVFMPHGENVILVIQDGVPVRAFMKDIAEESSILNPQVRLPQAAQRLAADVPEAYKLLTIFVDVFEGYFRHLTQILVETELMPEHDFWRLVAGRIAAYQQAHPQRLDKYRRYDLFAPDMIHSCLNRLQLANNLQMVNLADPIGSFQMAPNLPNPIACFRPSWLGSGEALQTLTAA

>1VK3A

MGSDKIHHHHHHMKLRYLNILKEKLGREPTFVELQAFSVMWSEHCGYSHTKKYIRRLPKTGFEGNAGVVNLDDYYSVAFKIESHNHPSAIEPYNGAATGVGGIIRDVLAMGARPTAIFDSLHMSRIIDGIIEGIADYGNSIGVPTVGGELRISSLYAHNPLVNVLAAGVVRNDMLVDSKASRPGQVIVIFGGATGRDGIHGASFASEDLTGDKATKLSIQVGDPFAEKMLIEAFLEMVEEGLVEGAQDLGAGGVLSATSELVAKGNLGAIVHLDRVPLREPDMEPWEILISESQERMAVVTSPQKASRILEIARKHLLFGDVVAEVIEEPVYRVMYRNDLVMEVPVQLLANAPEEDIVEYTPGKIPEFKRVEFEEVNAREVFEQYDHMVGTDTVVPPGFGAAVMRIKRDGGYSLVTHSRADLALQDTYWGTLIAVLESVRKTLSVGAEPLAITNCVNYGDPDVDPVGLSAMMTALKNACEFSGVPVASGNASLYNTYQGKPIPPTLVVGMLGKVNPQKVAKPKPSKVFAVGWNDFELEREKELWRAIRKLSEEGAFILSSSQLLTRTHVETFREYGLKIEVKLPEVRPAHQMVLVFSERTPVVDVPVKEIGTLSR

>3BZWA

MSLEQASVTNDFSENKQGCIQHPWQGKKVGYIGDSITDPNCYGDNIKKYWDFLKEWLGITPFVYGISGRQWDDVPRQAEKLKKEHGGEVDAILVFMGTNDYNSSVPIGEWFTEQEEQVLSAHGEMKKMVTRKKRTPVMTQDTYRGRINIGITQLKKLFPDKQIVLLTPLHRSLANFGDKNVQPDESYQNGCGEYIDAYVQAIKEAGNIWGIPVIDFNAVTGMNPMVEEQLIYFYDAGYDRLHPDTKGQERMARTLMYQLLALPVAFEGHHHHHH

>1NEPA

EPVKFKDCGSWVGVIKEVNVSPCPTQPCKLHRGQSYSVNVTFTSNTQSQSSKAVVHGIVMGIPVPFPIPESDGCKSGIRCPIEKDKTYNYVNKLPVKNEYPSIKVVVEWELTDDKNQRFFCWQIPIEVEA

>2MPRA

VDFHGYARSGIGWTGSGGEQQCFQATGAQSKYRLGNECETYAELKLGQEVWKEGDKSFYFDTNVAYSVNQQNDWESTDPAFREANVQGKNLIEWLPGSTIWAGKRFYQRHDVHMIDFYYWDISGPGAGIENIDLGFGKLSLAATRSTEAGGSYTFSSQNIYDEVKDTANDVFDVRLAGLQTNPDGVLELGVDYGRANTTDGYKLADGASKDGWMFTAEHTQSMLKGYNKFVVQYATDAMTTQGKGQARGSDGSSSFTEELSDGTKINYANKVINNNGNMWRILDHGAISLGDKWDLMYVGMYQNIDWDNNLGTEWWTVGVRPMYKWTPIMSTLLEVGYDNVKSQQTGDRNNQYKITLAQQWQAGDSIWSRPAIRIFATYAKWDEKWGYIKDGDNISRYAAATNSGISTNSRGDSDEWTFGAQMEIWW

>1H2SB

GAVFIFVGALTVLFGAIAYGEVTAAAATGDAAAVQEAAVSAILGLIILLGINLGLVAATL

>1TUKA

ACQASQLAVCASAILSGAKPSGECCGNLRAQQGCFCQYAKDPTYGQYIRSPHARDTLTSCGLAVPHC

>3N17A

ANNLGSKLLVGYWHNFDNGTGIIKLKDVSPKWDVINVSFGETGGDRSTVEFSPVYGTDADFKSDISYLKSKGKKVVLSIGGQNGVVLLPDNAAKDRFINSIQSLIDKYGFDGIDIDLQSGIYLNGNDTNFKNPTTPQIVNLISAIRTISDHYGPDFLLSMAPETAYVQGGYSAYGSIWGAYLPIIYGVKDKLTYIHVQHFNAGSGIGMDGNNYNQGTADYEVAMADMLLHGFPVGGNANNIFPALRSDQVMIGLPAAPAAAPSGGYISPTEMKKALNYIIKGVPFGGKYKLSNQSGYPAFRGLMSWSINWDAKNNFEFSNNYRTYFDGLSLQK

>3IUPA

GMHSALQLRSRIKSSGELELSLDSIDTPHPGPDEVLIRIEASPLNPSDLGLLFGAADMSTAKASGTAERPIVTARVPEGAMRSMAGRLDASMPVGNEGAGVVVEAGSSPAAQALMGKTVAAIGGAMYSQYRCIPADQCLVLPEGATPADGASSFVNPLTALGMVETMRLEGHSALVHTAAASNLGQMLNQICLKDGIKLVNIVRKQEQADLLKAQGAVHVCNAASPTFMQDLTEALVSTGATIAFDATGGGKLGGQILTCMEAALNKSAREYSRYGSTTHKQVYLYGGLDTSPTEFNRNFGMAWGMGGWLLFPFLQKIGRERANALKQRVVAELKTTFASHYSKEISLAEVLDLDMIAVYNKRATGEKYLINPNKGLAG

>1WUBA

MKWNLDPSHTSIDFKVRHMGIASVRGSLKVLSGSVETDEAGRPIQVEAVIDAASIATGEPQRDGHLRSADFLHAEQYPEIRFVSTQIEPLGGNRYRIQGNLTIRDITKPVTLEAEVSAPIKDPWGMQRVAASASGQINRKDWNLTWNQVLELGALLVGEEVKFNLEVEAVAPAPVAAQ

>2YGBA

MAHHHHHHSSGLEVLFQGPNNTIINSLIGGDDSIKRSNVFAVDSQIPTLYMPQYISLSGVMTNDGPDNQAIASFEIRDQYITALNHLVLSLELPEVKGMGRFGYVPYVGYKCINHVSISSCNGVIWEIEGEELYNNCINNTIALKHSGYSSELNDISIGLTPNDTIKEPSTVYVYIKTPFDVEDTFSSLKLSDSKITVTVTFNPVSDIVIRDSSFDFETFNKEFVYVPELSFIGYMVKNVQIKPSFIEKPRRVIGQINQPTATVTEVHAATSLSVYTKPYYGNTDNKFISYPGYSQDEKDYIDAYVSRLLDDLVIVSDGPPTGYPESAEIVEVPEDGIVSIQDADVYVKIDNVPDNMSVYLHTNLLMFGTRKNSFIYNISKKFSAITGTYSDATKRTIFAHISHSINIIDTSIPVSLWTSQRNVYNGDNRSAESKAKDLFINDPFIKGIDFKNKTDIISRLEVRFGNDVLYSENGPISRIYNELLTKSNNGTRTLTFNFTPKIFFRPTTITANVSRGKDKLSVRVVYSTMDVNHPIYYVQKQLVVVCNDLYKVSYDQGVSITKIMGDNN

>1YLIA

SANFTDKNGRQSKGVLLLRTLAMPSDTNANGDIFGGWIMSQMDMGGAILAKEIAHGRVVTVAVESMNFIKPISVGDVVCCYGQCLKVGRSSIKIKVEVWVKKVASEPIGERYCVTDAVFTFVAVDNNGRSRTIPRENNQELEKALALISEQPL

>4EL6A

GADSCKYCLQLYDETYERGSYIEVYKSVGSLSPPWTPGSVCVPFVNDTKRERPYWYLFDNVNYTGRITGLGHGTCIDDFTKSGFKGISSIKRCIQTKDGKVECINQ

>2Q82A

MDFITDMSKNQRLELQNRLAQYETSLMVMSHNGDVPVITGFNVMRVTTMLDALKVELPAVAVLGDDAQDLAYVFGARPLAVGVNIIRVVDVPGQQPSALVDAELGALHEVSMVRVLNDIADEQLVKANM

>3GKEA

MATFVRNAWYVAALPEELSEKPLGRTILDTPLALYRQPDGVVAALLDICPHRFAPLSDGILVNGHLQCPYHGLEFDGGGQCVHNPHGNGARPASLNVRSFPVVERDALIWIWPGDPALADPGAIPDFGCRVDPAYRTVGGYGHVDCNYKLLVDNLMDLGHAQYVHRANAQTDAFDRLEREVIVGDGEIQALMKIPGGTPSVLMAKFLRGANTPVDAWNDIRWNKVSAMLNFIAVAPEGTPKEQSIHSRGTHILTPETEASCHYFFGSSRNFGIDDPEMDGVLRSWQAQALVKEDKVVVEAIERRRAYVEANGIRPAMLSCDEAAVRVSREIEKLEQLEAARLEHHHHHH

>1H8PA

DQDEGVSTEPTQDGPAELPEDEECVFPFVYRNRKHFDCTVHGSLFPWCSLDADYVGRWKYCAQRDYAKCVFPFIYGGKKYETCTKIGSMWMSWCSLSPNYDKDRAWKYC

>1WPXB

XMNQAIDFAQASIDSYKKHGILEDVIHDTSFQPSGILAVEYSSSAPVAMGNTLPTEKARSKPQFQFTFNKQMQKSVPQANAYVPQDDDLFTLVMTDPDAPSKTDHKWSEFCHLVECDLKLLNEATHETSGATEFFASEFNTKGSNTLIEYMGPAPPKGSGPHRYVFLLYKQPKGVDSSKFSKIKDRPNWGYGTPATGVGKWAKENNLQLVASNFFYAETK

>2CSGA

SNAMTTPFTHETLPADPKAAIRQMKQALRAQIGDVQAVFDRLSATIAARVAEINDLKAQGQPVWPIIPFSELAMGNISDATRAEVKRRGCAVIKGHFPREQALAWDQSMLDYLDKNHFDEVYKGPGDNFFGTLSASRPEIYPVYWSQAQMQARQSEEMALAQSFLNRLWQVEHDGKRWFNPDISIIYPDRIRRRPPGTTSKGLGAHTDSGALERWLLPAYQQVFASVFNGNVEQYDPWNAAHRTDVEEYTVDNTTKCSVFRTFQGWTALSDMLPGQGLLHVVPIPEAMAYILLRPLLDDVPEDELCGVAPGRVLPISEQWHPLLMAALTSIPPLEAGDSVWWHCDVIHSVAPVENQQGWGNVMYIPAAPMCEKNLAYARKVKAALETGASPGDFPREDYETTWEGRFTLRDLNIHGKRALGIDV

>3KG7A

SNASGQQVHRLLGNKLELASTGQTIYHQDINLNNHPWIGDHRVYDTPVIPGVSYIAMTLAAVGVPAAVEDINFQQPLFLAESNTTRETQLMLHTADNVGKQFVEVFSRDGAKQEEWQQHASMSVSENPPPPPTLSVDIPALCEQLRPLDTDTLTEIYASISLVYGPMLQAVRQAWIGEETSLLEIEVPKALAFQLAGEPIHPVLIDACTRLTPDLFDFSSDSGVFWAPWRVKEMTLSHPTPSRFYAYVEEPSRVNEQLQTRSYDIQLLDETGQAFGRINGFTVKRAPSQLFLK

>2I0MA

MRNQFDLELHELEQSFLGLGQLVLETASKALLALASKDKEMAELIINKDHAINQGQSAIELTCARLLALQQPQVSDLRFVISIMSSCSDLERMGDHMAGIAKAVLQLKENQLAPDEEQLHQMGKLSLSMLADLLVAFPLHQASKAISIAQKDEQIDQYYYALSKEIIGLMKDQETSIPNGTQYLYIIGHLERFADYIANICERLVYLETGELVDLN

>3IAXB

MPGFNYGGKGDGTGWSSERGSGPEPGGGSHGNSGGHDRGDSSNVGNESVTVMKPGDSYNTPWGKVIINAAGQPTMNGTVMTADNSSMVPYGRGFTRVLNSLVNNPVSLEHHHHHH

>1D2SA

PPAVHLSNGPGQEPIAVMTFDLTKITKTSSSFEVRTWDPEGVIFYGDTNPKDDWFMLGLRDGRPEIQLHNHWAQLTVGAGPRLDDGRWHQVEVKMEGDSVLLEVDGEEVLRLRQVSGHPIMRIALGGLLFPASNLRLPLVPALDGCLRRDSWLDKQAEISASAPTSLRSC

>4A0GA

GSSHHHHHHSSGLVPRGSHMKSTSVSPFHLPLNHPTYLIWSANTSLGKTLVSTGIAASFLLQQPSSSATKLLYLKPIQTGFPSDSDSRFVFSKLDSLSLRRQIPISISNSVLHSSLPAAKSLGLNVEVSESGMCSLNFRDEKTVTGAPELLCKTLYAWEAAISPHLAAERENATVEDSVVLQMIEKCLKEEMECGVKSEKSDLLCLVETAGGVASPGPSGTLQCDLYRPFRLPGILVGDGRLGGISGTIAAYESLKLRGYDIAAVVFEDHGLVNEVPLTSYLRNKVPVLVLPPVPKDPSDDLIEWFVESDGVFKALKETMVLANLERLERLNGMAKLAGEVFWWPFTQHKLVHQETVTVIDSRCGENFSIYKASDNSSLSQQFDACASWWTQGPDPTFQAELAREMGYTAARFGHVMFPENVYEPALKCAELLLDGVGKGWASRVYFSDNGSTAIEIALKMAFRKFCVDHNFCEATEEEKHIVVKVIALRGSYHGDTLGAMEAQAPSPYTGFLQQPWYTGRGLFLDPPTVFLSNGSWNISLPESFSEIAPEYGTFTSRDEIFDKSRDASTLARIYSAYLSKHLQEHSGVRQSAHVGALIIEPVIHGAGGMHMVDPLFQRVLVNECRNRKIPVIFDEVFTGFWRLGVETTTELLGCKPDIACFAKLLTGGMVPLAVTLATDAVFDSFSGDSKLKALLHGHSYSAHAMGCATAAKAIQWFKDPETNHNITSQGKTLRELWDEELVQQISSHSAVQRVVVIGTLFALELKADASNSGYASLYAKSLLIMLREDGIFTRPLGNVIYLMCGPCTSPEICRRLLTKLYKRLGEFNRT

>1SU8A

MAKQNLKSTDRAVQQMLDKAKREGIQTVWDRYEAMKPQCGFGETGLCCRHCLQGPCRINPFGDEPKVGICGATAEVIVARGLDRSIAAGAAGHSGHAKHLAHTLKKAVQGKAASYMIKDRTKLHSIAKRLGIPTEGQKDEDIALEVAKAALADFHEKDTPVLWVTTVLPPSRVKVLSAHGLIPAGIDHEIAEIMHRTSMGCDADAQNLLLGGLRCSLADLAGCYMGTDLADILFGTPAPVVTESNLGVLKADAVNVAVHGHNPVLSDIIVSVSKEMENEARAAGATGINVVGICCTGNEVLMRHGIPACTHSVSQEMAMITGALDAMILDYQCIQPSVATIAECTGTTVITTMEMSKITGATHVNFAEEAAVENAKQILRLAIDTFKRRKGKPVEIPNIKTKVVAGFSTEAIINALSKLNANDPLKPLIDNVVNGNIRGVCLFAGCNNVKVPQDQNFTTIARKLLKQNVLVVATGCGAGALMRHGFMDPANVDELCGDGLKAVLTAIGEANGLGGPLPPVLHMGSCVDNSRAVALVAALANRLGVDLDRLPVVASAAEAMHEKAVAIGTWAVTIGLPTHIGVLPPITGSLPVTQILTSSVKDITGGYFIVELDPETAADKLLAAINERRAGLGLPW

>1YQ5A

GGVTDALSLMYSTSTGGPASIAANALTDFDLSGALTVNSVGTGLTKSAAGIQLAAGKSGLYQITMTVKNNTVTTGNYLLRVKYGSSDFVVACPASSLTAGGTISLLIYCNVLGVVSLDVLKFSLCNDGAALSNYIINITAAKIN

>4AKFA

GSMKLAEIMTKSRKLKRNLLEISKTEAGQYSVSAPEHKGLVLSGGGAKGISYLGMIQALQERGKIKNLTHVSGASAGAMTASILAVGMDIKDIKKLIEGLDITKLLDNSGVGFRARGDRFRNILDVIYMMQMKKHLESVQQPIPPEQQMNYGILKQKIALYEDKLSRAGIVINNVDDIINLTKSVKDLEKLDKALNSIPTELKGAKGEQLENPRLTLGDLGRLRELLPEENKHLIKNLSVVVTNQTKHELERYSEDTTPQQSIAQVVQWSGAHPVLFVPGRNAKGEYIADGGILDNMPEIEGLDREEVLCVKAEAGTAFEDRVNKAKQSAMEAISWFKARMDSLVEATIGGKWLHATSSVLNREKVYYNIDNMIYINTGEVTTTNTSPTPEQRARAVKNGYDQTMQLLDSHKQTFDHPLMAILYIGHDKLKDALIDEKSEKEIFEASAHAQAILHLQEQIVKEMNDGDYSSVQNYLDQIEDILTVDAKMDDIQKEKAFALCIKQVNFLSEGKLETYLNKVEAEAKAAAEPSWATKILNLLWAPIEWVVSLFKGPAQDFKVEVQPEPVKVSTSENQET

>3EF8A

GMTDTNLVEMRAIERMMFDYSYHLDMNHPEELAALFVEDCEVSYAPNFGATGRDAYKKTLEGIGTFFRGTSHHNSNICIDFVSETEANVRSVVLAIHRYTKERPDGILYGQYFDTVVKVDGQWKFKRRELRTTMTTDYHVRAANPIGRAE

>2OU6A

GMPTFNPELHAQTLNSERAYFVQPDADPAFTPHIGALVEMLTYARLTTLQAVEGLPEDQLWATAPGFANSIGTLLAHIAAVERVYHVLSFQGRDVTPEDDGAAYWGLTMGKEGTAPARLPTLDELRAELADARAETLRVFAAKDDAWLAEPLGPGWANQHWAWFHVMEDEVNHRGQLRLLRQVLAPEEGG

>1P3CA

VVIGDDGRTKVANTRVAPYNSIAYITFGGSSCTGTLIAPNKILTNGHCVYNTASRSYSAKGSVYPGMNDSTAVNGSANMTEFYVPSGYINTGASQYDFAVIKTDTNIGNTVGYRSIRQVTNLTGTTIKISGYPGDKMRSTGKVSQWEMSGSVTREDTNLAYYTIDTFSGNSGSAMLDQNQQIVGVHNAGYSNGTINGGPKATAAFVEFINYAKAQ

>1ITHA

GLTAAQIKAIQDHWFLNIKGCLQAAADSIFFKYLTAYPGDLAFFHKFSSVPLYGLRSNPAYKAQTLTVINYLDKVVDALGGNAGALMKAKVPSHDAMGITPKHFGQLLKLVGGVFQEEFSADPTTVAAWGDAAGVLVAAMK

>1PIWA

MSYPEKFEGIAIQSHEDWKNPKKTKYDPKPFYDHDIDIKIEACGVCGSDIHCAAGHWGNMKMPLVVGHEIVGKVVKLGPKSNSGLKVGQRVGVGAQVFSCLECDRCKNDNEPYCTKFVTTYSQPYEDGYVSQGGYANYVRVHEHFVVPIPENIPSHLAAPLLCGGLTVYSPLVRNGCGPGKKVGIVGLGGIGSMGTLISKAMGAETYVISRSSRKREDAMKMGADHYIATLEEGDWGEKYFDTFDLIVVCASSLTDIDFNIMPKAMKVGGRIVSISIPEQHEMLSLKPYGLKAVSISYSALGSIKELNQLLKLVSEKDIKIWVETLPVGEAGVHEAFERMEKGDVRYRFTLVGYDKEFSD

>4J4HA

MALLTPDDLININMQLQKADSAVQEVTGLDIKGICKALYGTFSSSEKVGIVPVTSGNGIIGNFSASLHAITQYFGFDSFVTDMPDVSGYYEAVQNGAEIILMADDRTFLAHNLKNGKMANNQPCTGIIYAEIASRYLKADSKDVLVVGLGKVGFPGAEHLVQKDFRVYGYDADETLLERATSNLGIIPFDPANPKKFSIIFEATPCANTIPEAVLSENCVLSTPGIPCAISEELRDKYEVQLIAEPLGIGTASMLYSVL

>1QWRA

SNAMTQSPIFLTPVFKEKIWGGTALRDRFGYSIPSESTGECWAISAHPKGPSTVANGPYKGKTLIELWEEHREVFGGVEGDRFPLLTKLLDVKEDTSIKVHPDDYYAGENEEGELGKTECWYIIDCKENAEIIYGHTARSKTELVTMINSGDWEGLLRRIKIKPGDFYYVPSGTLHALCKGALVLETQQNSDATYRVYDYDRLDSNGSPRELHFAKAVNAATVPHVDGYIDESTESRKGITIKTFVQGEYFSVYKWDINGEAEMAQDESFLICSVIEGSGLLKYEDKTCPLKKGDHFILPAQMPDFTIKGTCTLIVSHI

>2GX5A

MGSSHHHHHHMALLQKTRIINSMLQAAAGKPVNFKEMAETLRDVIDSNIFVVSRRGKLLGYSINQQIENDRMKKMLEDRQFPEEYTKNLFNVPETSSNLDINSEYTAFPVENRDLFQAGLTTIVPIIGGGERLGTLILSRLQDQFNDDDLILAEYGATVVGMEILREKAE

>2A6ZA

GSDASKLSSDYSLPDLINTRKVPNNWQTGEQASLEEGRIVLTSNQNSKGSLWLKQGFDLKDSFTMEWTFRSVGYSGQTDGGISFWFVQDSNIPRDKQLYNGPVNYDGLQLLVDNNGPLGPTLRGQLNDGQKPVDKTKIYDQSFASCLMGYQDSSVPSTIRVTYDLEDDNLLKVQVDNKVCFQTRKVRFPSGSYRIGVTAQNGAVNNNAESFEIFKMQFFNGV

>2Q2RA

APAGSHMNIKELSLHELCEELKTPAWNAPLTFVGDVGGTSARMGFVREGKNDSVHACVTRYSMKRKDITEIIEFFNEIIELMPASVMKRVKAGVINVPGPVTGGAVGGPFNNLKGIARLSDYPKALFPPGHSAILNDLEAGGFGVLAVSDAHVFSEYFGVMWEGTQWRTCEQEPAGSVIGRGRCLVLAPGTGLGSSLIYYNPPMNQHIVVPLELGSQTLPMRKDIDYIQTLHAELKLFPNYENMVSGAGLEFHYRQVVRGSRPPCSAGEIAKLASEGDANACKAMKKYHEYLMRVGSEASMALLPLTIVLVGDNIVNNAFFYRNPQNLKEMHHEALNHEMERFGFQSRVSYLRQKKLLNLNLMGCYRCGLDLS

>3SXOA

MGSSHHHHHHSSGLVPRGSHMPQSDSVTVTLCSPTEDDWPGMFLLAAASFTDFIGPESATAWRTLVPTDGAVVVRDGAGPGSEVVGMALYMDLRLTVPGEVVLPTAGLSFVAVAPTHRRRGLLRAMCAELHRRIADSGYPVAALHASEGGIYGRFGYGPATTLHELTVDRRFARFHADAPGGGLGGSSVRLVRPTEHRGEFEAIYERWRQQVPGGLLRPQVLWDELLAECKAAPGGDRESFALLHPDGYALYRVDRTDLKLARVSELRAVTADAHCALWRALIGLDSMERISIITHPQDPLPHLLTDTRLARTTWRQDGLWLRIMNVPAALEARGYAHEVGEFSTVLEVSDGGRFALKIGDGRARCTPTDAAAEIEMDRDVLGSLYLGAHRASTLAAANRLRTKDSQLLRRLDAAFASDVPVQTAFEF

>1LNIA

DVSGTVCLSALPPEATDTLNLIASDGPFPYSQDGVVFQNRESVLPTQSYGYYHEYTVITPGARTRGTRRIITGEATQEDYYTGDHYATFSLIDQTC

>2WHMA

RADVKPVTVKLVDSQATMETRSLFAFMQEQRRHSIMFGHQHETTQGLTITRTDGTQSDTFNAVGDFAAVYGWDTLSIVAPKAAGDIVAQVKKAYARGGIITVSSHFDNPKTDTQKGVWPVGTSWDQTPAVVDSLPGGAYNPVLNGYLDQVAEWANNLKDEQGRLIPVIFRLYHENTGSWFWWGDKQSTPEQYKQLFRYSVEYLRDVKGVRNFLYAYSPNNFWDVTEANYLERYPGDEWVDVLGFDTYGPVADNADWFRNVVANAALVARMAEARGKIPVISGIGIRAPDIEAGLYDNQWYRKLISGLKADPDAREIAFLLVWRNAPQGVPGPNGTQVPHYWVPANRPENINNGTLEDFQAFYADEFTAFNRDIEQVYQRPTLIVK

>3HRGA

GMIDFTKSKQYTLSIRLSTDGFSFSIYNPINDNSQSLFEKEVDTSLSLTANLKNVFHESDFLSYSYKRVNIMIASKRFTMIPLELFEEEQAELLFYHNHQKRENEIVMYNILKKNNVVIIFGIDKSTYTFLNEQYPEARFYSQSTPLIEYFSIKSRLGNSKKMYASVRKDAIDIYCFERGQLLLANSFECMQTEDRIYYLLYVWKQLEFNQERDELHLTGTLSDKETLMNELKKFILQVFIMNPANNIDMQALLTCE

>2PH0A

MTMTLNELLATNPDGTLEDIAGKYNTSLFAVVEALPTAQCTLATGDRFDQVWDTIATWGEVTLISHTADAILEFKSELPTGTHRHGYFNLRGKNGLSGHIRATSCQHIAFIERKFMGMDTASVVFFNANGAAMFKIFLGRDSHRQLLSAQVDAFRALASELQPEQVLEHHHHHH

>1G73A

AVPIAQKSEPHSLSSEALMRRAVSLVTDSTSTDLSQTTYALIEAITEYTKAVYTLTSLYRQYTSLLGKMNSEEEDEVWQVIIGARAEMTSKHQEYLKLETTWMTAVGLSEMAAEAAYQTGADQASITARNHIQLVKLQVEEVHQLSRKAETKLAEAQIEELR

>2B97A

AVCPTGLFSNPLCCATNVLDLIGVDCKTPTIAVDTGAIFQAHCASKGSKPLCCVAPVADQALLCQKAIGTF

>1Q9JA

MFPGSVIRKLSHSEEVFAQYEVFTSMTIQLRGVIDVDALSDAFDALLETHPVLASHLEQSSDGGWNLVADDLLHSGICVIDGTAATNGSPSGNAELRLDQSVSLLHLQLILREGGAELTLYLHHCMADGHHGAVLVDELFSRYTDAVTTGDPGPITPQPTPLSMEAVLAQRGIRKQGLSGAERFMSVMYAYEIPATETPAVLAHPGLPQAVPVTRLWLSKQQTSDLMAFGREHRLSLNAVVAAAILLTEWQLRNTPHVPIPYVYPVDLRFVLAPPVAPTEATNLLGAASYLAEIGPNTDIVDLASDIVATLRADLANGVIQQSGLHFGTAFEGTPPGLPPLVFCTDATSFPTMRTPPGLEIEDIKGQFYCSISVPLDLYSCAVYAGQLIIEHHGHIAEPGKSLEAIRSLLCTVPSEYGWIME

>1NOWA

AKPGPALWPLPLSVKMTPNLLHLAPENFYISHSPNSTAGPSCTLLEEAFRRYHGYIFGFYKWHHEPAEFQAKTQVQQLLVSITLQSECDAFPNISSDESYTLLVKEPVAVLKANRVWGALRGLETFSQLVYQDSYGTFTINESTIIDSPRFSHRGILIDTSRHYLPVKIILKTLDAMAFNKFNVLHWHIVDDQSFPYQSITFPELSNKGSYSLSHVYTPNDVRMVIEYARLRGIRVLPEFDTPGHTLSWGKGQKDLLTPCYSRQNKLDSFGPINPTLNTTYSFLTTFFKEISEVFPDQFIHLGGDEVEFKCWESNPKIQDFMRQKGFGTDFKKLESFYIQKVLDIIATINKGSIVWQEVFDDKAKLAPGTIVEVWKDSAYPEELSRVTASGFPVILSAPWYLDLISYGQDWRKYYKVEPLDFGGTQKQKQLFIGGEACLWGEYVDATNLTPRLWPRASAVGERLWSSKDVRDMDDAYDRLTRHRCRMVERGIAAQPLYAGYCNHENM

>1RYP1

QFNPYGDNGGTILGIAGEDFAVLAGDTRNITDYSINSRYEPKVFDCGDNIVMSANGFAADGDALVKRFKNSVKWYHFDHNDKKLSINSAARNIQHLLYGKRFFPYYVHTIIAGLDEDGKGAVYSFDPVGSYEREQCRAGGAAASLIMPFLDNQVNFKNQYEPGTNGKVKKPLKYLSVEEVIKLVRDSFTSATERHIQVGDGLEILIVTKDGVRKEFYELKRD

>3FBIA

MASNDPGNEVSSLYPPPPPYVKFFTQSNLEKLPKYKEKKAASAKQTAPNNSNGGSEEEITCALDYLIPPPMPKNQQYRAFGSIW

>3TEUA

MLDAPTDLQVTNVTDTSITVSWTPPSATITGYRITYTPSNGPGEPKELTVPPSSTSVTITGLTPGVEYVVSVYALKDNQESPPLVGTQTTGGHHHHHH

>3P06A

NGVELSAVGVLLPVLMDSGRRISGGAFMAVKGDLSEHIKNPKNTRIAQTVAGGTIYGLSEMVNIDEAEKLPIKGAITVLPVVQATATSILVPDNQPQLAFNSWEAAACAADTLESQQTPFLMVTGAVESGNLSPNLLAVQKQLLVAKPAGIGLAANSDRALKVVTLEQLRQVVGDKPWRKPMVTFSSGKNVAQA

>2WLUA

MTNTLVENIYASVTHNISKKEASKNEKTKAVLNQAVADLSVAASIVHQVHWYMRGPGFLYLHPKMDELLDSLNANLDEVSERLITIGGAPYSTLAEFSKHSKLDEAKGTYDKTVAQHLARLVEVYLYLSSLYQVGLDITDEEGDAGTNDLFTAAKTEAEKTIWMLQAERGQGPAL

>4DMTA

XGPPGPPGPPGPRGQPGVMGFPGPPGPPGPPX

>3ZXKA

MPRQASTFTNPVLWEDHPALEVFRVGSVFYYSSSTFAYSPGAPVLKSYDLVHWTPVTHSVPRLNFGSNYDLPSGTPGAYVKGIWASTLRYRRSNDRFYWYGCVEGRTYLWTSPGGNALANNGEVPPSAWNWQHTATIDNCYYDAGLLIDDDDTMYIAYGNPTINVAQLSPDGTRQVRVQQRVYAHPQGQTVEGARMYKIRGNYYILVTRPADAEYVLRSTTGSPFGPYEARTLVSRIQGPLANAGFAHQGGIVDAPDGTWHYVAFMDAYPGGRIPVVAPLRWTADGWPEVVTDSQGRWGTSYPIPVRGAKNATEGLASTDLDEFRGTRFSEHWEWNHNPDTSKFTLLGGNEGGLILRTATVTGDLFAARNTLTRRIAGPKASGIFRLDVRGMRDGDRAGAVLFRDRAAYIGVWKQGNEARIVMVDDLRLNEDGWRTASTGRVAANGPVIDTNAQQDIWLRIDADITPAFGTNTERTTTFYYSIDGGRTYTRLGPAFAMTNSWRYFTGYRFGVFNFSTKSLGGEVKVKGFKMNMILEHHHHHH

>2NXPA

GSVAVEDQPDVSAVLSAYNQQGDPTMYEEYYSGLKHFIECSLDCHRAELSQLFYPLFVHMYLELVYNQHENEAKSFFEKFHGDQECYYQDDLRVLSSLTKKEHMKGNETMLDFRTSKFVLRISRDSYQLLKRHLQEKQNNQIWNIVQEHLYIDIFD

>2GRRB

STGEPAPVLSSPPPADVSTFLAFPSPEKLLRLGPKSSVLIAQQTDTSDPEKVVSAFLKVSSVFKDEATVRMAVQDAVDALMQKAFNSSSFNSNTFLTRLLVHMGLLKSEDKVKAIANLYGPLMALNHMVQQDYFPKALAPLLLAFVTKPNSALESCSFARHSLLQTLYKV

>1UWKA

MTDNNKYRDVEIRAPRGNKLTAKSWLTEAPLRMLMNNLDPQVAENPKELVVYGGIGRAARNWECYDKIVETLTRLEDDETLLVQSGKPVGVFKTHSNAPRVLIANSNLVPHWANWEHFNELDAKGLAMYGQMTAGSWIYIGSQGIVQGTYETFVEAGRQHYGGSLKGKWVLTAGLGGMGGAQPLAATLAGACSLNIESQQSRIDFRLETRYVDEQATDLDDALVRIAKYTAEGKAISIALHGNAAEILPELVKRGVRPDMVTDQTSAHDPLNGYLPAGWTWEQYRDRAQTEPAAVVKAAKQSMAVHVQAMLDFQKQGVPTFDYGNNIRQMAKEEGVADAFDFPGFVPAYIRPLFCRGVGPFRWAALSGEAEDIYKTDAKVKELIPDDAHLHRWLDMARERISFQGLPARICWVGLGLRAKLGLAFNEMVRSGELSAPVVIGRDHLDSGSVSSPNAETEAMRDGSDAVSDWPLLNALLNTAGGATWVSLHHGGGVGMGFSQHSGMVIVCDGTDEAAERIARVLTNDPGTGVMRHADAGYDIAIDCAKEQGLDLPMITG

>2WGKA

MAMETGLIFHPYMRPGRSARQTFDWGIKSAVQADSVGIDSMMISEHASQIWENIPNPELLIAAAALQTKNIKFAPMAHLLPHQHPAKLATMIGWLSQILEGRYFLGIGAGAYPQASYMHGIRNAGQSNTATGGEETKNLNDMVRESLFIMEKIWKREPFFHEGKYWDAGYPEELEGEEGDEQHKLADFSPWGGKAPEIAVTGFSYNSPSMRLAGERNFKPVSIFSGLDALKRHWEVYSEAAIEAGHTPDRSRHAVSHTVFCADTDKEAKRLVMEGPIGYCFERYLIPIWRRFGMMDGYAKDAGIDPVDADLEFLVDNVFLVGSPDTVTEKINALFEATGGWGTLQVEAHDYYDDPAPWFQSLELISKEVAPKILLPKR

>3SK2A

GSHMTDLAGPTITPNLQLVYVSNVERSTDFYRFIFKKEPVFVTPRYVAFPSSGDALFAIWSGGEEPVAEIPRFSEIGIMLPTGEDVDKLFNEWTKQKSHQIIVIKEPYTDVFGRTFLISDPDGHIIRVCPLD

>1E29A

VELTESTRTIPLDEAGGTTTLTARQFTNGQKIFVDTCTQCHLQGKTKTNNNVSLGLADLAGAEPRRDNVLALVEFLKNPKSYDGEDDYSELHPNISRPDIYPEMRNYTEDDIFDVAGYTLIAPKLDERWGGTIYF

>3E4WA

MHHHHHHMSGGLTPDQAIDAIRGTGGAQPGCRALHAKGTLYRGTFTATRDAVMLSAAPHLDGSTVPALIRFSNGSGNPKQRDGAPGVRGMAVKFTLPDGSTTDVSAQTARLLVSSTPEGFIDLLKAMRPGLTTPLRLATHLLTHPRLLGALPLLREANRIPASYATTEYHGLHAFRWIAADGSARFVRYHLVPTAAEEYLSASDARGKDPDFLTDELAARLQDGPVRFDFRVQIAGPTDSTVDPSSAWQSTQIVTVGTVTITGPDTEREHGGDIVVFDPMRVTDGIEPSDDPVLRFRTLVYSASVKLRTGVDRGAQAPPV

>4A7UA

ATKAVCVLKGDGPVQGIINFEQKESNGPVKVWGSIKGLTEGLHGFHVHEFGDNTAGCTSAGPHFNPLSRKHGGPKDEERHVGDLGNVTADKDGVADVSIEDSVISLSGDHCITGRTLVVHEKADDLGKGGNEESTKTGNAGSRLACGVIGIAQ

>4F7HA

SSIMTSENHLNNSDKEVDEVDAALSDLEITLEGGKTSTILGDITSIPELADYIKVFKPKKLTLKGYKQYWCTFKDTSISCYKSKEESSGTPAHQMNLRGCEVTPDVNISGQKFNIKLLIPVAEGMNEIWLRCDNEKQYAHWMAACRLASKGKTMADSSYNLEVQNILSFLKMQ

>1EXTA

MDSVCPQGKYIHPQNNSICCTKCHKGTYLYNDCPGPGQDTDCRECESGSFTASENHLRHCLSCSKCRKEMGQVEISSCTVDRDTVCGCRKNQYRHYWSENLFQCFNCSLCLNGTVHLSCQEKQNTVCTCHAGFFLRENECVSCSNCKKSLECTKLCLPQIEN

>2X7QA

MAHHHHHHGHHHQLPTLKVAYIPEHFSTPLFFAQQQGYYKAHDLSIEFVKVPEGSGRLINLLNSNEVDIAIGLTEAFIADIAKGNENIHVLDTYVKSPLLWAVSTGSNRDDVTDAKQLKRIGVSRIGSGSYVMSFVLAHQLGVPSFDQFQVLSNFKNLRDSVNLKDGVEGSDAFMWEYFTSKKYYDNHEIKQIDQIYTPWSSWVVATSSDSLQAKSDVIKNFIDAVNQGIQYYNEHVDEAIEYISSNLDYSAEDAKEWTKTVEFNSRIGKTPLDWDTIVVKTKDTLKLAGVLAESDDVILKRLNSNVKKTNLQLDGDLEAA

>1L1LA

MSEEISLSAEFIDRVKASVKPHWGKLGWVTYKRTYARWLPEKGRSENWDETVKRVVEGNINLDPRLQDSPSLELKQSLTEEAERLYKLIYGLGATPSGRNLWISGTDYQRRTGDSLNNCWFVAIRPQKYGDSKIVPSYLGKQEKAVSMPFSFLFDELMKGGGVGFSVARSNISQIPRVDFAIDLQLVVDETSESYDASVKVGAVGKNELVQDADSIYYRLPDTREGWVLANALLIDLHFAQTNPDRKQKLILDLSDIRPYGAEIHGFGGTASGPMPLISMLLDVNEVLNNKAGGRLTAVDAADICNLIGKAVVAGNVRRSAELALGSNDDQDFISMKQDQEKLMHHRWASNNSVAVDSAFSGYQPIAAGIRENGEPGIVNLDLSKNYGRIVDGYQAGIDGDVEGTNPCGEISLANGEPCNLFEVFPLIAEEQGWDLQEVFALAARYAKRVTFSPYDWEISREIIQKNRRIGISMSGIQDWLLTRLGNRVVTGFKDDFDPETHEAIKVPVYDKRAIKMVDQLYKAVVKADQDYSKTLGCNESIKHTTVKPSGTVAKLAGASEGMHFHYGAYLIQRIRFQDSDPLLPALKACGYRTEADIYTENTTCVEFPIKAVGADNPNFASAGTVSIAEQFATQAFLQTYWSDNAVSCTITFQDSEGDQVESLLRQYRFITKSTSLLPYFGGSLQQAPKEPIDKETYEKRSQEITGNVEEVFSQLNSDVKDLELVDQTDCEGGACPIK

>3IFNP

DAEFRHDSGYEVHHQKLVFFAEDVGSNKGAIIGLMVGGVV

>2I7RA

SNAMNLNQLDIIVSNVPQVCADLEHILDKKADYANDGFAQFTIGSHCLMLSQNHLVPLENFQSGIIIHIEVEDVDQNYKRLNELGIKVLHGPTVTDWGTESLLVQGPAGLVLDFYRMK

>2PSPA

EKPAACRCSRQDPKNRVNCGFPGITSDQCFTSGCCFDSQVPGVPWCFKPLPAQESEECVMQVSARKNCGYPGISPEDCAARNCCFSDTIPEVPWCFFPMSVEDCHY

>2G38A

MSFVITNPEALTVAATEVRRIRDRAIQSDAQVAPMTTAVRPPAADLVSEKAATFLVEYARKYRQTIAAAAVVLEEFAHALTTGADKYATAEADNIKTFS

>3SCYA

GAQPTDPSTTDSELTMLVGTYTSGNSKGIYTFRFNEETGESLPLSDAEVANPSYLIPSADGKFVYSVNEFSKDQAAVSAFAFDKEKGTLHLLNTQKTMGADPCYLTTNGKNIVTANYSGGSITVFPIGQDGALLPASDVIEFKGSGPDKERQTMPHLHCVRITPDGKYLLADDLGTDQIHKFNINPNANADNKEKFLTKGTPEAFKVAPGSGPRHLIFNSDGKFAYLINEIGGTVIAFRYADGMLDEIQTVAADTVNAQGSGDIHLSPDGKYLYASNRLKADGVAIFKVDETNGTLTKVGYQLTGIHPRNFIITPNGKYLLVACRDTNVIQIFERDQATGLLTDIKKDIKVDKPVCLKFVD

>2QQ4A

MSVLDELYREILLDHYQSPRNFGVLPQATKQAGGMNPSCGDQVEVMVLLEGDTIADIRFQGQGCAISTASASLMTEAVKGKKVAEALELSRKFQAMVVEGAPPDPTLGDLLALQGVAKLPARVKCATLAWHALEEALR

>2W39A

ATYHLEDNWVGSAFLSTFTHEAIADPTHGRVNYVDQATALAKNLTYASGDTLILRADHTTTLSPSGPGRNSVRIRSIKTYTTHVAVFDVRHMPQGCGTWPAAWETDEGDWPNGGEVDIIEGVNDQSPNAMTLHTGANCAMPASRTMTGHATNNNCDVNTDGNTGCGVQAPTANSYGPSFNANGGGWYAMERTNSFIKVWFFPRNAGNVPNDIASGPATINTDNWGTPTAFFPNTNCDIGSHFDANNIIINLTFCGDWAGQASIFNGAGCPGSCVDYVNNNPSAFANAYWDIASVRVYQ

>3O12A

MGSSHHHHHHSSGRENLYFQGMVESKNTELSQGTWLNKPKSVFQEAGKVTLETDEKTDFWRETFYGFTRDSGHFLGVETGSAFTAQVRVQGSYESLYDQAGIMVRIDDGHWLKAGIEISDGHAMLSSVLTNGKSDWSTAVYGGNARDFWLRVTVEKGVLRIQVSSDKKTWPLVRLAPFPTSDHYLVGPMACTPERGGLKVTFSEWSLTAPLGKALHDLSGS

>1EDMB

VDGDQCESNPCLNGGSCKDDINSYECWCPFGFEGKNCEL

>2QL8A

GMQDERWNHPLYTTTAINDEELEGHAYIPGGLKVQTSSPMNDHPGTNPEQLLGLSLSTCLEATLEAVEKEHGLPHTGAVRVKVAFIGARAEYQFLVHAQVMVKGVDFDTAKAFTNEIENRCPVSKLLKNSGNYTIETVTDFKD

>1U5HA

MNLRAAGPGWLFCPADAPEAFAAAAAAADVVILDLEDGVAEAQKPAARNALRDTPLDPERTVVRINAGGTADQARDLEALAGTAYTTVMLPKAESAAQVIELAPRDVIALVETARGAVCAAEIAAADPTVGMMWGAEDLIATLGGSSSRRADGAYRDVARHVRSTILLAASAFGRLALDAVHLDILDVEGLQEEARDAAAVGFDVTVCIHPSQIPVVRKAYAASHEKLAWARRVLAASRSERGAFAFEGQMVDSPVLTHAETMLRRAGEATSE

>3CJYA

GMTEAFPALVRQDDARYAITVGPDLAVGPPGHAYLFGGASMALALDVAAETVGRPVVQGSLQFVSFTPLGSVLDLTVEVLQSGRTLAQARVAGTVDGRLVFHSGISLGMREGFSARQWALAPPVPQPDNCPPCTTLPAQDDNARYLEGIEVREAGGPEVPSGRTRLWLRRKDGAPLDAASLAMFADFLPIALGRATGCSGGGNSLDNSLRITGAAAPGWCLCDMIIPSSASGFAQGQVTLWDQSGRLLATGAQSLLLKG

>2VFOA

MDPDQYSIEADKKFKYSLKLSDYPTLQDAASAAVDGLLIDRDYNFYGGETVDFGGKVLTIECKAKFIGDGNLIFTKLGKGSRIAGVFMESTTTPWVIKPWTDDNQWLTDAAAVVATLKQSKTDGYQPTVSDYVKFPGIETLLPPNAKGQNITSTLEIRECIGVEVHRASGLMAGFLFRGCHFCKMVDANNPSGGKDGIITFENLSGDWGKGNYVIGGRTSYGSVSSAQFLRNNGGFERDGGVIGFTSYRAGESGVKTWQGTVGSTTSRNYNLQFRDSVVIYPVWDGFDLGADTDMNPELDRPGDYPITQYPLHQLPLNHLIDNLLVRGALGVGFGMDGKGMYVSNITVEDCAGSGAYLLTHESVFTNIAIIDTNTKDFQANQIYISGACRVNGLRLIGIRSTDGQSLTIDAPNSTVSGITGMVDPSRINVANLAEEGLGNIRANSFGYDSAAIKLRIHKLSKTLDSGALYSHINGGAGSGSAYTQLTAISGSTPDAVSLKVNHKDCRGAEIPFVPDIASDDFIKDSSCFLPYWENNSTSLKALVKKPNGELVRLTLATL

>3MAYA

GASDPCAASEVARTVGSVAKSMGDYLDSHPETNQVMTAVLQQQVGPGSVASLKAHFEANPKVASDLHALSQPLTDLSTRCSLPISGLQAIGLMQAVQGARR

>2XU3A

APRSVDWREKGYVTPVKNQGQCGSCWAFSATGALEGQMFRKTGRLISLSEQNLVDCSGPQGNEGCNGGLMDYAFQYVQDNGGLDSEESYPYEATEESCKYNPKYSVANDTGFVDIPKQEKALMKAVATVGPISVAIDAGHESFLFYKEGIYFEPDCSSEDMDHGVLVVGYGFESTESDNNKYWLVKNSWGEEWGMGGYVKMAKDRRNHCGIASAASYPTV

>2VRSA

ILQTTVDGNSTAISNLKSDISSNGLAITDLQDRVKSLESTASHGLSFSPPLSVADGVVSLDMDPYFCSQRVSLTSYSAEAQLMQFRWMARGTNGSSDTIDMTVNAHCHGRRTDYMMSSTGNLTVTSNVVLLTFDLSDITHIPSDLARLVPSAGFQAASFPVDVSFTRDSATHAYQAYGVYSSSRVFTITFPTGGDGTANIRSLTVRTGIDT

>1ZJCA

GSHMTNYKEKLQQYAELLVKVGMNVQPKQPVFIRSSVETLELTHLIVEEAYHCGASDVRVVYSDPTLKRLKFENESVEHFANHEIKSYDVEARMDYVKRGAANLALISEDPDLMDGIDSQKLQAFQQQNARAFKGYMESVQKNQFPWVVAAFPSKAWAKRVYPELSVEEAYIKFIDEVFDIVRIDGNDPVENWRQHIANLSVYAQKLQQKNYHALHYVSEGTDLTVGLAKNHIWEDATSYVNGKEQAFIANIPTEEVFTAPDRNRVDGYVTNKLPLSYNGTIIDQFKLMFKDGEIIDFSAEKGEAVLKDLINTDEGSRRLGEVALVPDDSPISNRNTIFYNTLFDENAACHLAIGSAYAFNIQGGTEMTVEEKIASGLNDSNVHVDFMIGSSDLTIYGIFEDGSKELVFENGNWASTF

>2HA9A

AMDIRQVTETIAMIEEQNFDIRTITMGISLLDCIDPDINRAAEKIYQKITTKAANLVAVGDEIAAELGIPIVNKRVSVTPISLIGAATDATDYVVLAKALDKAAKEIGVDFIGGFSALVQKGYQKGDEILINSIPRALAETDKVCSSVNIGSTKSGINMTAVADMGRIIKETANLSDMGVAKLVVFANAVEDNPFMAGAFHGVGEADVIINVGVSGPGVVKRALEKVRGQSFDVVAETVKKTAFKITRIGQLVGQMASERLGVEFGIVDLSLAPTPAVGDSVARVLEEMGLETVGTHGTTAALALLNDQVKKGGVMACNQVGGLSGAFIPVSEDEGMIAAVQNGSLNLEKLEAMTAICSVGLDMIAIPEDTPAETIAAMIADEAAIGVINMKTTAVRIIPKGKEGDMIEFGGLLGTAPVMKVNGASSVDFISRGGQIPAPIHSFKN

>1RYPK

MDIILGIRVQDSVILASSKAVTRGISVLKDSDDKTRQLSPHTLMSFAGEAGDTVQFAEYIQANIQLYSIREDYELSPQAVSSFVRQELAKSIRSRRPYQVNVLIGGYDKKKNKPELYQIDYLGTKVELPYGAHGYSGFYTFSLLDHHYRPDMTTEEGLDLLKLCVQELEKRMPMDFKGVIVKIVDKDGIRQVDDFQAQ

>4DOYA

MGSSHHHHHHSSGLVPRGSHMTLSPEKQHVRPRDAADNDPVAVARGLAEKWRATAVERDRAGGSATAEREDLRASGLLSLLVPREYGGWGADWPTAIEVVREIAAADGSLGHLFGYHLTNAPMIELIGSQEQEEHLYTQIAQNNWWTGNASSENNSHVLDWKVSATPTEDGGYVLNGTKHFCSGAKGSDLLFVFGVVQDDSPQQGAIIAAAIPTSRAGVTPNDDWAAIGMRQTDSGSTDFHNVKVEPDEVLGAPNAFVLAFIQSERGSLFAPIAQLIFANVYLGIAHGALDAAREYTRTQARPWTPAGIQQATEDPYTIRSYGEFTIALQGADAAAREAAHLLQTVWDKGDALTPEDRGELMVKVSGVKALATNAALNISSGVFEVIGARGTHPRYGFDRFWRNVRTHSLHDPVSYKIADVGKHTLNGQYPIPGFTS

>1UHVA

MIKVRVPDFSDKKFSDRWRYCVGTGRLGLALQKEYIETLKYVKENIDFKYIRGHGLLCDDVGIYREDVVGDEVKPFYNFTYIDRIFDSFLEIGIRPFVEIGFMPKKLASGTQTVFYWEGNVTPPKDYEKWSDLVKAVLHHFISRYGIEEVLKWPFEIWNEPNLKEFWKDADEKEYFKLYKVTAKAIKEVNENLKVGGPAICGGADYWIEDFLNFCYEENVPVDFVSRHAYTSKQGEYTPHLIYQEIMPSEYMLNEFKTVREIIKNSHFPNLPFHITEYNTSYSPQNPVHDTPFNAAYIARILSEGGDYVDSFSYWTFSDVFEERDVPRSQFHGGFGLVALNMIPKPTFYTFKFFNAMGEEMLYRDEHMLVTRRDDGSVALIAWNEVMDKTENPDEDYEVEIPVRFRDVFIKRQLIDEEHGNPWGTWIHMGRPRYPSKEQVNTLREVAKPEIMTSQPVANDGYLNLKFKLGKNAVVLYELTERIDESSTYIGLDDSKINGY

>3LEDA

MGSSHHHHHHSSGRENLYFQGVRPAVIAATGLYTPPDSVSNAELVEAFNTYVANFNAANKARIEAGEIEPLQPSSSEFIEKASGIKSRYVVAKPGIVDPDVMRPIIPERSNDELSILAEMAVTAAEQAIERWGKPRERIGAVLCACSNMQRAYPAMAIEVQNALGLGGFAFDMNVACSSATFGLKTAADFVGGGSVDAVLMVNPEICSGHLNFRDRDSHFIFGDVATAAIVERADDAQGGWSILGTKLKTQFSNNIRNNAGFLNRAWPEGRDKADKLFVQQGRKVFKEVVPLVSEMIIEHAREIGIDPHGLKRMWLHQANINMNEIIGRKVLGRDPTRDENVIILDDYANTSSAGSIIAFHKHQDDMAQGDLGLICSFGAGYSAGTVFVQKR

>1QQP3

GIFPVACSDGYGGLVTTDPKTADPVYGKVFNPPRNQLPGRFTNLLDVAEACPTFLRFEGGVPYVTTKTDSDRVLAQFDMSLAAKHMSNTFLAGLAQYYTQYSGTINLHFMFTGPTDAKARYMVAYAPPGMEPPKTPEAAAHCIHAEWDTGLNSKFTFSIPYLSAADYTYTASDVAETTNVQGWVCLFQITHGKADGDALVVLASAGKDFELRLPVDARAE

>2UW1A

MQVTHSMPPQKLEIFKSLDDWARNNVLIHLKSVEKSWQPQDYLPDPVSDGFEEQVRELRERAKEIPDDYFVVLVGDMITEEALPTYMSMLNRCDGIKDETGAEPSAWAMWTRAWTAEENRHGDLLNKYLYLSGRVDMRKIEKTIQYLIGSGMDIKSENSPYLGFIYTSFQERATFISHANTAKLAQHYGDKKLAHICGSIASDEKRHATAYTKIVEKLAEIDPDTTVIAFADMMRKKITMPAHLMYDGSDELLFKHFTAVAQRLGVYSALDYCDILEFLVDKWNVERLTGLSDEGRKAQEYVCELGPKIRRLEERAQGRAKEAPTMPFSWIFDRQVKL

>2FE8A

MEVKTIKVFTTVDNTNLHTQLVDMSMTYGQQFGPTYLDGADVTKIKPHVNHEGKTFFVLPSDDTLRSEAFEYYHTLDESFLGRYMSALNHTKKWKFPQVGGLTSIKWADNNCYLSSVLLALQQLEVKFNAPALQEAYYRARAGDAANFCALILAYSNKTVGELGDVRETMTHLLQHANLESAKRVLNVVCKHCGQKTTTLTGVEAVMYMGTLSYDNLKTGVSIPCVCGRDATQYLVQQESSFVMMSAPPAEYKLQQGTFLCANEYTGNYQCGHYTHITAKETLYRIDGAHLTKMSEYKGPVTDVFYKETSYTTTI

>3HZ7A

MITIDALGQVCPIPVIRAKKALAELGEAGGVVTVLVDNDISRQNLQKMAEGMGYQSEYLEKDNGVIEVTIVAGEGCAVELEHHHHHH

>3ETVA

GAMGMNGIDDLLNINDRIKQVQNERNELASKLQNLKQSLASNDTGGGSGGGSDSSDLLQREAILANELNILDNLKTFLNLIKEVKTNLNILELENCYYSLQSLRKKMRNNAAYLKQSFNFQQSISTYVDTLHLELVSTLYKILTNGFWKITENSIQFTPTVEWGKDKVHIEYDTFMDFVAQQYFPKGSLDNQAWFILDMTSADSQEQVRAKLNTIMKEYMNLSRIVSMIKNSIFISGKEISYENEKNILVFSKSSSHGQHCVSTVLTSFEAVCDFMLDGLAFRDRKTLSYELGPLFNTEFTKFVKNNASIILESLDSPLKNLVSVINNKLTRLVAKSEVTNWTHSGKEIQDLLMN

>4EZIA

GALEHEKLVNYIALGEFSRETAEIALKKMPPLDTLTVHYDLQLYKINYKTQSPDGNLTIASGLVAMPIHPVGQVGIISYQHGTRFERNDVPSRNNEKNYIYLAAYGNSAGYMTVMPDYLGLGDNELTLHPYVQAETLASSSIDMLFAAKELANRLHYPISDKLYLAGYSEGGFSTIVMFEMLAKEYPDLPVSAVAPGSAPYGWEETMHFVMLEPGPRATAYLAYFFYSLQTYKSYWSGFDEIFAPPYNTLIPELMDGYHAVDEILQALPQDPLLIFQPKFSNGIISKTDRNTEILKINFNHYDFKPTAPLLLVGTKGDRDVPYAGAEMAYHSFRKYSDFVWIKSVSDALDHVQAHPFVLKEQVDFFKQFERQEAMNK

>4AXOA

MGQIIEEKISGTKDTVDFVRNKDISGITSIKLPTVKVSESDRLDTGNPSDVVYTKDLFTLEESPRLGCGMMEMKETTFDWTLNYDEIDYVIDGTLDIIIDGRKVSASSGELIFIPKGSKIQFSVPDYARFIYVTYPADWASQNLEHHHHHH

>1DYOA

KPEEPDAGYYYHDTFEGSVGQWTARGPAEVLLSGRTAYKGSESLLVRNRTAAWNGAQRALNPRTFVPGNTYCFSVVASFIEGASSTTFCMKLQYVDGSGTQRYDTIDMKTVGPNQWVHLYNPQYRIPSDATDMYVYVETADDTINFYIDEAIGAVAGTVI

>3H9WA

MTKAIPWKINWQTMAFEYIGPQIEALLGWPQGSWKSVEDWATRMHPEDQEWVVNFCVKQSECGVDHEADYRALHRDGHYVWIRDVVHVVRDDSGEVEALIGFMFDISLEHHHHHH

>1THTA

MNNQCKTIAHVLRVNNGQELHVWETPPKENVPFKNNTILIASGFARRMDHFAGLAEYLSTNGFHVFRYDSLHHVGLSSGSIDEFTMTTGKNSLCTVYHWLQTKGTQNIGLIAASLSARVAYEVISDLELSFLITAVGVVNLRDTLEKALGFDYLSLPIDELPNDLDFEGHKLGSEVFVRDCFEHHWDTLDSTLDKVANTSVPLIAFTANNDDWVKQEEVYDMLAHIRTGHCKLYSLLGSSHDLGENLVVLRNFYQSVTKAAIAMDGGSLEIDVDFIEPDFEQLTIATVNERRLKAEIENRTPEMA

>4G55A

GSPEFMAQILPIRFQEHLQLQNLGINPANIGFSTLTMESDKFICIREKVGEQAQVVIIDMNDPSNPIRRPISADSAIMNPASKVIALKAGKTLQIFNIEMKSKMKAHTMTDDVTFWKWISLNTVALVTDNAVYHWSMEGESQPVKMFDRHSSLAGCQIINYRTDAKQKWLLLTGISAQQNRVVGAMQLYSVDRKVSQPIEGHAASFAQFKMEGNAEESTLFCFAVRGQAGGKLHIIEVGTPPTGNQPFPKKAVDVFFPPEAQNDFPVAMQISEKHDVVFLITKYGYIHLYDLETGTCIYMNRISGETIFVTAPHEATAGIIGVNRKGQVLSVCVEEENIIPYITNVLQNPDLALRMAVRNNLAGAEELF

>2APLA

MKSTEKKELSHFRLKLETYLNEHFPEMSGNNPFITARSDEALTAYCDAVAQGFSHPEAESMASEVLYQGLHFSRYDTLVSVLEREFEQELPSPLPERLAPILLKNKAIQSVFAKYDLTDDFEASPEYEHLYTELTGTIVLLIESNHLPTIGGGNDTV

>2EW0A

MTKQYLTHRCLIAPPEMADDFFANTVIYLARHDEEGAQGIIINRPAGIQIKELLNDLDIDADNVNPHEVLQGGPLRPEAGFVLHTGQPTWHSSIAVGENVCITTSKDILDAIAHNEGVGRYQIALGYASWGKNQLEDEIARGDWLICDADMDLIFNLPYDDRWDAAYKKIGVDRTWLASEIGHALEHHHHHH

>1SEFA

MSLMGYKNNRVGYQKELLTSRAVIKKDNYAIIPHDGLVQNAVPGFENVDISILGSPKLGATFVDYIATFHKNGQQTTGFGGDGIQTLVYVIDGRLRVSDGQETHELEAGGYAYFTPEMKMYLANAQEADTEVFLYKKRYQPLAGHQPYKVVGSIHDQQPEEYEGMTDVLLWSLLPKEFDFDMNMHILSFEPGASHAYIETHVQEHGAYLISGQGMYNLDNEWYPVEKGDYIFMSAYVPQAAYAVGREEPLMYVYSKDANREPELEGGSHHHHHH

>1JB0L

AEELVKPYNGDPFVGHLSTPISDSGLVKTFIGNLPAYRQGLSPILRGLEVGMAHGYFLIGPWVKLGPLRDSDVANLGGLISGIALILVATACLAAYGLVSFQKGGSSSDPLKTSEGWSQFTAGFFVGAMGSAFVAFFLLENFLVVDGIMTGLFN

>4IILA

MSGSHHHHHHSSGIEGRGRLIKHRPAVQDERAVRIAVFVPGFRHDSPVYAMLCDGVERAVTQERATGRSIGLDIIEAGPNQALWREKLAHLAAEQRYRLIVSSNPALPHVLEPILRQFPLQRFLVLDAYAPQEHSLITFRYNQWEQAYLAGHLSALVSASAMRFANADKKIGLIAGQSYPVMTQTIIPAFLAGARAVDPAFEVDVRVVGNWYDAAKSADLARILFHEGVDVMMPICGGANQGVLAAARELGFYVSWFDDNGYARAPGYVVGSSVMEQERLAYEQTLRCIRGELPSAGAWTLGVKDGYVRFIEEDPLYLQTVPEPIRVRQSALLRRIQSGELTLPVR

>4ASMB

HHHHHHQYDWDNVPIPANAGAGKTWKLQTAASDDFNYTFNPTNNVVDFGPNGNMKWYNKYHNRPNGQPNNFEGPGPTKWMQNHVAVSGGNLNIWASRIPGATKSFTGSNNTPISRPETRAGCITNKTRVKYPVFVEARVKVMNSTLASDIWLLSPDDTQEIDIMECYGGPGNDNRNSYFASKIHLSHHVFIRPPNFKDYQPADLNSWWGKNGVTQWGGKTIRIGVNWVSPTRLEYFVDGQMVRILDNDAVQTRLADGTWQYTYPAGVTSTGVNGQLIKENGYQKMNIASSLSDAKNKSNISVIDPFNYLNNGRKFSKEMDIIINVEDQSWQAEAYRSPNAAEMANFYDNNLLVDWIRVYKPVN

>1FM0E

MAETKIVVGPQPFSVGEEYPWLAERDEDGAVVTFTGKVRNHNLGDSVNALTLEHYPGMTEKALAEIVDEARNRWPLGRVTVIHRIGELWPGDEIVFVGVTSAHRSSAFEAGQFIMDYLKTRAPFWKREATPEGDRWVEARESDQQAAKRW

>3R5TA

QQNVWPRTFQNADGSITTIPSQPKRILSTAVTVTGTLLAIDAPVIASAATTQSTFFEQWRKLAELRQVKKLWPAGSVDLESVYVEQPDLIVVSMIGADSARDQIPLLQAIAPTILVDYSDQTWQSLAQQLGLATGLEEQAERTIHNFEQWTKQVRDVLDLPKGRANIVSYHGPGVVNAVAKAQSAHAQLLQSVGVVLEEPDPAWQAGSIVHRDFLRIHYEHLTQLQAETTFLITMTDQQAQAFLHDPILKNLPSIQRKQVYGLGENSFRIDLFSAREIINSLLRRFAGEQAQSLVMPLEHHHHHH

>2Y6HA

MLVANINGGFESTPAGVVTDLAEGVEGWDLNVGSSVTNPPVFEVLETSDAPEGNKVLAVTVNGVGNNPFNIQATALPVNVRPGVTYTYTIRARAEQDGAVVSFTVGNQSFDEYGRLHHQQITTEWQPFTFEFTVSDQETVIRAPIHFGYAANVGNTIYIDGLAIVDL

>3ZBDA

MAHHHHHHMSSKQFKILVNEDYQVNVPSLPIRDVLQEIKYCYRNGFEGYVFVPEYCRDLVDCDRKDHYVIGVLGNGVSDLKPVLLTEPSVMLQGFIVRANCNGVLEDFDLKIA

>2GRRB

STGEPAPVLSSPPPADVSTFLAFPSPEKLLRLGPKSSVLIAQQTDTSDPEKVVSAFLKVSSVFKDEATVRMAVQDAVDALMQKAFNSSSFNSNTFLTRLLVHMGLLKSEDKVKAIANLYGPLMALNHMVQQDYFPKALAPLLLAFVTKPNSALESCSFARHSLLQTLYKV

>3RKCA

SRPFSVLRANDVLWLSLTAAEYDQTTYGSSTNPMYVSDTVTFVNVATGAQGVSRSLDWSKVTLDGRPLTTIQQYSKTFFVLPLRGKLSFWEAGTTKAGYPYNYNTTASDQILIENAPGHRVCISTYTTNLGSGPVSISAVGVLAPHSA

>2BU3A

FCLTLRRRYTMGHHHHHHHHHHSSGHIEGRHMKLEQTLTLSPNLIGFNSNEGEKLLLTSRSREDFFPLSMQFVTQVNQAYCGVASIIMVLNSLGINAPETAQYSPYRVFTQDNFFSNEKTKAVIAPEVVARQGMTLDELGRLIASYGVKVKVNHASDTNIEDFRKQVAENLKQDGNFVIVNYLRKEIGQERGGHISPLAAYNEQTDRFLIMDVSRYKYPPVWVKTTDLWKAMNTVDSVSQKTRGFVFVSKTQDD

>4AQOA

GGTISNNKAPIAKVTGPSTGAVGRNIEFSGKDSKDEDGKIVSYDWDFGDGATSRGKNSVHAYKKAGTYNVTLKVTDDKGATATESFTIEIKN

>3ATVA

GSFTMAKAAAIGIDLGTTYSCVGVFQHGKVEIIANDQGNRTTPSYVAFTDTERLIGDAAKNQVALNPQNTVFDAKRLIGRKFGDPVVQSDMKHWPFQVINDGDKPKVQVSYKGETKAFYPEEISSMVLTKMKEIAEAYLGYPVTNAVITVPAYFNDSQRQATKDAGVIAGLNVLRIINEPTAAAIAYGLDRTGKGERNVLIFDLGGGTFDVSILTIDDGIFEVKATAGDTHLGGEDFDNRLVNHFVEEFKRKHKKDISQNKRAVRRLRTACERAKRTLSSSTQASLEIDSLFEGIDFYTSITRARFEELCSDLFRSTLEPVEKALRDAKLDKAQIHDLVLVGGSTRIPKVQKLLQDFFNGRDLNKSINPDEAVAYGAAVQAAILMGDKSENV

>3AA0B

MSDQQLDCALDLMRRLPPQQIEKNLSDLIDLVPSLCEDLLSSVDQPLKIARDKVVGKDYLLCDYNRDGDSYRSPWSNKYDPPLEDGAMPSARLRKLEVEANNAFDQYRDLYFEGGVSSVYLWDLDHGFAGVILIKKAGDGSKKIKGCWDSIHVVEVQEKSSGRTAHYKLTSTVMLWLQTNKTGSGTMNLGGSLTRQMEKDETVSDSSPHIANIGRLVEDMENKIRSTLNEIYFGKTKDIVNGLR

>2C3VA

GSHMASGDATDITIYYKTGWTHPHIHYSLNQGAWTTLPGVPLTKSEYEGYVKVTIEAEEGSQLRAAFNNGSGQWDNNQGRDYDFSSGVHTLADGRILSGTPK

>3CIJA

GHMNVKLKVFHAGSLTEPMKAFKRAFEEKHPNVEVQTEAAGSAATIRKVTELGRKADVIATADYTLIQKMMYPEFANWTIMFAKNQIVLAYRNDSRYADEINSQNWYEILKRPDVRFGFSNPNDDPCGYRSLMAIQLAELYYNDPTIFDELVAKNSNLRFSEDNGSYVLRMPSSERIEINKSKIMIRSMEMELIHLVESGELDYFFIYKSVAKQHGFNFVELPVEIDLSSPDYAELYSKVKVVLANGKEVTGKPIVYGITIPKNAENRELAVEFVKLVISEEGQEILRELGQEPL

>2ZAHA

NISYTEGAKPGAISAPVAISRRVAGMKPRFVRSEGSVKIVHREFIASVLPSNDLTVNNGDVNIGKYRVNPSNNALFTWLQGQAQLYDMYRFTRLRFTYIPTTGSTSTGRVSILWDRDSQDPLPIDRAAISSYAHYADSAPWAENVLVVPCDNTWRYMNDTNAVDRKLVDFGQFLFATYSGAGATAHGDLYVEYAVEFKDPQPIAGMVCMFDRLVSFSEVGSTIKGVNYIADRDVITTGGNIGVNINIPGTYLVTIVLNATSIGSLTFTGNSKLVGNSLNVTSSGASALTFTLNSTGVPNSSNSSFSVGTVVALTRVRMTITRCSPETAYLA

>3OC8A

GSHMEIYPHIKVYEGTLSRLKPGGAMIAVLEYDVNELSKHGYTNLWDVQFKVLVGVPHAETGVIYDPVYEETVKPYQPSNNLTGKKLYNVSTNDMHNGYKWSNTMFSNSNYKTQILLTKGDGSGVKLYSKAYSENFK

>4EA9A

GHMGAASASLAIGGVVIIGGGGHAKVVIESLRACGETVAAIVDADPTRRAVLGVPVVGDDLALPMLREQGLSRLFVAIGDNRLRQKLGRKARDHGFSLVNAIHPSAVVSPSVRLGEGVAVMAGVAINADSWIGDLAIINTGAVVDHDCRLGAACHLGPASALAGGVSVGERAFLGVGARVIPGVTIGADTIVGAGGVVVRDLPDSVLAIGVPAKIKGDRS

>3ARCA

MTTTLQRRESANLWERFCNWVTSTDNRLYVGWFGVIMIPTLLAATICFVIAFIAAPPVDIDGIREPVSGSLLYGNNIITGAVVPSSNAIGLHFYPIWEAASLDEWLYNGGPYQLIIFHFLLGASCYMGRQWELSYRLGMRPWICVAYSAPLASAFAVFLIYPIGQGSFSDGMPLGISGTFNFMIVFQAEHNILMHPFHQLGVAGVFGGALFCAMHGSLVTSSLIRETTETESANYGYKFGQEEETYNIVAAHGYFGRLIFQYASFNNSRSLHFFLAAWPVVGVWFAALGISTMAFNLNGFNFNHSVIDAKGNVINTWADIINRANLGMEVMHERNAHNFPLDLA

>3V6OA

IDVNINISCETDGYLTKMTCRWSTSTIQSLAESTLQLRYHRSSLYCSDIPSIHPISEPKDCYLQSDGFYECIFQPIFLLSGYTMWIRINHSLGSLDSPPTCVLPDSVVKPLPPSSVKAEITINIGLLKISWEKPVFPENNLQFQIRYGLSGKEVQWKMYEVYDAKSKSVSLPVPDLCAVYAVQVRCKRLDGLGYWSNWSNPAYTVV

>3FETA

SNAMKFLTVSDDMNFLRQVNTLVAGKGDMDSVIIGEGDAKGLGSKVLYRAKKGTPFDAVSEGILKIAGNYDYIAIGSTEVGREIAGYLSFKTGFYTATEIFSLEFNGQKAHTKRFFYGGKTVIEEESDARILTVAPGVIEAKDLGTTPEIRDLEIGQSRIKITKFV

>2ODAA

MPLPTFPALLFGLSGCLVDFGAQAATSDTPDDEHAQLTPGAQNALKALRDQGMPCAWIDELPEALSTPLAAPVNDWMIAAPRPTAGWPQPDACWMALMALNVSQLEGCVLISGDPRLLQSGLNAGLWTIGLASCGPLCGLSPSQWQALNNAEREQRRAQATLKLYSLGVHSVIDHLGELESCLADIALRRSKGEKP

>2EZ2A

MNYPAEPFRIKSVETVSMIPRDERLKKMQEAGYNTFLLNSKDIYIDLLTDSGTNAMSDKQWAGMMMGDEAYAGSENFYHLERTVQELFGFKHIVPTHQGRGAENLLSQLAIKPGQYVAGNMYFTTTRYHQEKNGAVFVDIVRDEAHDAGLNIAFKGDIDLKKLQKLIDEKGAENIAYICLAVTVNLAGGQPVSMANMRAVRELTEAHGIKVFYDATRCVENAYFIKEQEQGFENKSIAEIVHEMFSYADGCTMSGKKDCLVNIGGFLCMNDDEMFSSAKELVVVYEGMPSYGGLAGRDMEAMAIGLREAMQYEYIEHRVKQVRYLGDKLKAAGVPIVEPVGGHAVFLDARRFCEHLTQDEFPAQSLAASIYVETGVRSMERGIISAGRNNVTGEHHRPKLETVRLTIPRRVYTYAHMDVVADGIIKLYQHKEDIRGLKFIYEPKQLRFFTARFDYI

>1M1LA

ASLFPPGLHAIYGECRRLYPDQPNPLQVTAIVKYWLGGPDPLDYVSMYRNVGSPSANIPEHWHYISFGLSDLYGDNRVHEFTGTDGPSGFGFELTFRLKRETGESAPPTWPAELMQGLARYVFQSENTFCSGDHVSWHSPLDNSESRIQHMLLTEDPQMQPVQTPFGVVTFLQIVGVCTEELHSAQQWNGQGILELLRTVPIAGGPWLITDMRRGETIFEIDPHLQERVDKGIETD

>3D1BA

GSHMNPLASLTTDKNDLYINWLKSLSFFQTNSSCAEALVKVIPHYHNKLIDFSQVLQLVFSASEKFPIQENQPLPEQLMFLSNLEKQTPFAKAVGSSIYKLVTGKNLSLDFASQILKEASILEH

>2WQKA

MPTFLLVNDDGYFSPGINALREALKSLGRVVVVAPDRNLSGVGHSLTFTEPLKMRKIDTDFYTVIDGTPADCVHLGYRVILEEKKPDLVLSGINEGPNLGEDITYSGTVSGAMEGRILGIPSIAFSAFGRENIMFEEIAKVCVDIVKKVLNEGIPEDTYLNVNIPNLRYEEIKGIKVTRQGKRAYKERVFKYIDPYGKPFYWIAAEEFGWHAEEGTDYWAVLNGYVSVTPLHLDLTNYKVMKSIKYLEDSP

>3CG6A

GSTARMQGAGKALHELLLSAQRQGCLTAGVYESAKVLNVDPDNVTFCVLAADEEDEGDIALQIHFTLIQAFCCENDIDIVRVGDVQRLAAIVGSDEEGGAPGDLHCILISNPNEDTWKDPALEKLSLFCEESRSFNDWVPSITLPE

>2GS5A

MFADRLFNAMERNEPAPGMVLVAAPSMESEDFARSVILIIEHSEYATFGVNLASRSDVAVFNVIPEWVPCVTKPQALYIGGPLNQQSVVGVGVTAQGVDAARVDNLTRLANRLVMVNLGADPEEIKPLVSGMRLFAGHAEWAPGQLAQEIENGDWFVAPALPSDVTAPGSVDVWGDVMRRQPMPLPLYSTFPVNVGEN

>1ZVTA

SEPVTIVLSQMGWVRSAKGHDIDAPGLNYKAGDSFKAAVKGKSNQPVVFVDSTGRSYAIDPITLPSARGQGEPLTGKLTLPPGATVDHMLMESDDQKLLMASDAGYGFVCTFNDLVARNRAGKALITLPENAHVMPPVVIEDASDMLLAITQAGRMLMFPVSDLPQLSKGKGNKIINIPSAEAARGEDGLAQLYVLPPQSTLTIHVGKRKIKLRPEELQKVTGERGRRGTLMRGLQRIDRVEIDSPRRASSGDSEE

>3ZQUA

MSGPERITLAMTGASGAQYGLRLLDCLVQEEREVHFLISKAAQLVMATETDVALPAKPQAMQAFLTEYCGAAAGQIRVFGQNDWMAPPASGSSAPNAMVICPCSTGTLSAVATGACNNLIERAADVALKERRPLVLVPREAPFSSIHLENMLKLSNLGAVILPAAPGFYHQPQSVEDLVDFVVARILNTLGIPQDMLPRWGEQHLVSDE

>3ZXCA

FTCPECRPELCGDPGYCEYGTTKDACDCCPVCFQGPGGYCGGPEDVFGICADGFACVPLVGERDSQDPIVGTCVKIP

>2Y9WA

SDKKSLMPLVGIPGEIKNRLNILDFVKNDKFFTLYVRALQVLQARDQSDYSSFFQLGGIHGLPYTEWAKAQPQLHLYKANYCTHGTVLFPTWHRAYESTWEQTLWEAAGTVAQRFTTSDQAEWIQAAKDLRQPFWDWGYWPNDPDFIGLPDQVIRDKQVEITDYNGTKIEVENPILHYKFHPIEPTFEGDFAQWQTTMRYPDVQKQENIEGMIAGIKAAAPGFREWTFNMLTKNYTWELFSNHGAVVGAHANSLEMVHNTVHFLIGRDPTLDPLVPGHMGSVPHAAFDPIFWMHHCNVDRLLALWQTMNYDVYVSEGMNREATMGLIPGQVLTEDSPLEPFYTKNQDPWQSDDLEDWETLGFSYPDFDPVKGKSKEEKSVYINDWVHKHYG

>3KSNA

GDAASDLKSRLDKVSSFHASFTQKVTDGSGAAVQEGQGDLWVKRPNLFNWHMTQPDESILVSDGKTLWFYNPFVEQATATWLKDATGNTPFMLIARNQSSDWQQYNIKQNGDDFVLTPKASNGNLKQFTINVGRDGTIHQFSAVEQDDQRSSYQLKSQQNGAVDAAKFTFTPPQGVTVDDQRK

>3KZSA

GAQKKTQKTYIPWSNGKLVVSEEGRYLKHENGTPFFWLGETGWLLPERLNRDEAEYYLEQCKRRGYNVIQVQTLNNVPSMNIYGQYSMTDGYNFKNINQKGVYGYWDHMDYIIRTAAKKGLYIGMVCIWGSPVSHGEMNVDQAKAYGKFLAERYKDEPNIIWFIGGDIRGDVKTAEWEALATSIKAIDKNHLMTFHPRGRTTSATWFNNAPWLDFNMFQSGHRRYGQRFGDGDYPIEENTEEDNWRFVERSMAMKPMKPVIDGEPIYEEIPHGLHDENELLWKDYDVRRYAYWSVFAGSFGHTYGHNSIMQFIKPGVGGAYGAKKPWYDALNDPGYNQMKYLKNLMLTFPFFERVPDQSVIAGQNGERYDRAIATRGNDYLMVYNYTGRPMEVDFSKISGAKKNAWWYTTKDGKLEYIGEFDNGVHKFQHDSGYSSGNDHVLIVVDSSKDYVKKDCYQIDTHE

>3ORUA

GMTSFDRPFEAARPDGENPSAHETLAEGGRLRPEATYTIPARQGRAIRMAQGEALMVINRDGSQIGDFWAFVEGDCGEYLSMEHLRPTLRRVSPRPGDVLVSNRRRPILTLLEDSSPGVHDTLVASCDVHRYAQLGHEGYHDNCTDNLRMALGALGLRPTTVPCPLNLWMNTPVVEGGAMEWRPPVSRRGDHVLFRAELDVVVVISCCPMDLLPINGEEAQPRALDVRLRPRPA

>3NFTA

GSALTVRDWPALEALAKTMPADAGARAMTDDDLRAAGVDRRVPEQKLGAAIDEFASLRLPDRIDGRFVDGRRANLTVFDDARVAVRGHARAQRNLLERLETELLGGTLDTAGDEGGIQPDPILQGLVDVIGQGKSDIDAYATIVEGLTKYFQSVADVMSKLQDYISAKDDKNMKIDGGKIKALIQQVIDHLPTMQLPKGADIARWRKELGDAVSISDSGVVTINPDKLIKMRDSLPPDGTVWDTARYQAWNTAFSGQKDNIQNDVQTLVEKYSHQNSNFDNLVKVLSGAISTLTDTAKSYLQI

>3LATA

VSSQKTSSLPKYTPKVNSSINNYIRKKNMKAPRIEEDYTSYFPKYGYRNGVGRPEGIVVHDTANDNSTIDGEIAFMKRNYTNAFVHAFVDGNRIIETAPTDYLSWGAGPYGNQRFINVEIVHTHDYDSFARSMNNYADYAATQLQYYNLKPDSAENDGRGTVWTHAAISNFLGGTDHADPHQYLRSHNYSYAELYDLIYEKYLIKTKQVAPWG

>3N6XA

GMDTAKTKPFDEMFLQDEVIRPIYAEYAAWLQDVPHQQLESKRQEAELLFRRVGITFNVYGEDAGAERLIPFDVVPRILSASEWARLSDGAIQRVKALNMFLHDVYHDQEIIKAGIVPSSILANAQYRPEMFGVDVPGGVYAHIAGVDLVRTGENDFYVLEDNLRTPSGVSYMLENRKMMMRLFPELFRRYPVAPVEHYPQVLLNNLRAVAQAGVHEPTVVLLTPGAYNSAYFEHAFIAQQMGIELVEGQDLFVRNNAVYMRTTEGPKRVDVIYRRIDDDFIDPLSFRPDSMLGVPGLLSVYRNGGVTLANAVGTGVADDKDTYIYVPEMIRFYLGEEPILSNVPTYQLSKADDLKYVLDNLAELVVKEVQGSGGYGMLVGPAASKQELEDFRQRILANPANYIAQPTLALSTCPTLVETGIAPRHVDLRPFVLSGKTVSLVPGALCRVALREGSLVVNSSQGGGTKDTWILKD

>3MD9A

MAERIVTIGGDVTEIAYALGAGDEIVARDSTSQQPQAAQKLPDVGYMRTLNAEGILAMKPTMLLVSELAQPSLVLTQIASSGVNVVTVPGQTTPESVAMKINAVATALHQTEKGQKLIEDYQQRLAAVNKTPLPVKVLFVMSHGGLTPMAAGQNTAADAMIRAAGGSNAMQGFSRYRPLSQEGVIASAPDLLLITTDGVKALGSSENIWKLPGMALTPAGKHKRLLVVDDMALLGFGLETPQVLAQLREKMEQMQ

>3AYFA

MEVNRTVSPNIQTGRKTTNSFLKSILIFTILISSTVLLVGGYWIFKEMAPRPKEVRSESGEVLMTKETIIGGQAVFQKYGLMDYGTVLGHGSYMGPDYTAEALKVYTEGMQDYKAKERYNKPFADLTDDEKSIIREQVIKEMRKNRYNPVTDVLVLTDAQVYGLEKVRDYYRDVFTNGDGWGLKKGLIKESDMPKANRAWVADSDQIQQIADFFFWTAWLSSTLRIGDEITYTNNWPYYEDAGNTMSFSAVWWSGASVTILILFIGIILYVFYRYQLSMQEAYAEGKFPVIDLRRQPLTPSQVKAGKYFVVVSALFFVQTMFGALLAHYYTEPDSFFGINWIYDILPFNIAKGYHLQLAIFWIATAWLGMGIFIAPLVGGQEPKKQGLLVDLLFWALVVLVGGSMIGQWLGVNGYLGNEWFLLGHQGWEYIELGRIWQIILVVGMLLWLFIVFRGVKRGLKRESDKGGLIHLLFYSAIAVPFFYIFAFFIQPDTNFTMADFWRWWIIHLWVEGIFEVFAVVVIGFLLVQLRLVTKKSTVRALYFQFTILLGSGVIGIGHHYYYNGSPEVWIALGAVFSALEVIPLTLLILEAYEQYKMMRDGGANFPYKATFWFLISTAIWNLVGAGVFGFLINLPAVSYFEHGQFLTPAHGHAAMMGVYGMFAIAVLLYSLRNIVKPEAWNDKWLKFSCWMLNIGLAGMVVITLLPVGILQMKEAFIHGYWASRSPSFLQQDVVQNLLLVRAVPDTIFLIGVVALLVFAIKALFHLRKPTHGEGEELPVANHWMKDRLKNSLEHHHHHH

>2RINA

AEPESCGTVRFSDVGWTDITATTATATTILEALGYETDVKVLSVPVTYTSLKNKDIDVFLGNWMPTMEADIAPYREDKSVETVRENLAGAKYTLATNAKGAELGIKDFKDIAAHKDELDGKIYGIEPGNDGNRLIIDMVEKGTFDLKGFEVVESSEQGMLAQVARAEKSGDPIVFLGWEPHPMNANFKLTYLSGGDDVFGPNYGGATVHTNVRAGYTTECPNVDKLLQNLSFSLQMENEIMGKILNDGEDPEKAAAAWLKDNPQSIEPWLSGVATKDGGDGLAAVKAALGLEHHHHHH

>3INGA

GMKEIRIILMGTGNVGLNVLRIIDASNRRRSAFSIKVVGVSDSRSYASGRNLDISSIISNKEKTGRISDRAFSGPEDLMGEAADLLVDCTPASRDGVREYSLYRMAFESGMNVVTANKSGLANKWHDIMDSANQNSKYIRYEATVAGGVPLFSVLDYSILPSKVKRFRGIVSSTINYVIRNMANGRSLRDVVDDAIKKGIAESNPQDDLNGLDAARKSVILVNHIFGTEYTLNDVEYSGVDERSYNANDRLVTEVYVDDRRPVAVSRIISLNKDDFLMSIGMDGLGYQIETDSNGTVNVSDIYDGPYETAGAVVNDILLLSKVQK

>3A07A

SAQFASVTIRNAQTGRLLDSNYNGNVYTLPANGGNYQRWTGPGDGTVRNAQTGRCLDSNYDGAVYTLPCNGGSYQKWLFYSNGYIQNVETGRVLDSNYNGNVYTLPANGGNYQKWYTG

>1YD7A

AHHHHHHGSNKRFPFPVGEPDFIQGDEAIARAAILAGCRFYAGYPITPASEIFEAMALYMPLVDGVVIQMEDEIASIAAAIGASWAGAKAMTATSGPGFSLMQENIGYAVMTETPVVIVDVQRSGPSTGQPTLPAQGDIMQAIWGTHGDHSLIVLSPSTVQEAFDFTIRAFNLSEKYRTPVILLTDAEVGHMRERVYIPNPDEIEIINRKLPRNEEEAKLPFGDPHGDGVPPMPIFGKGYRTYVTGLTHDEKGRPRTVDREVHERLIKRIVEKIEKNKKDIFTYETYELEDAEIGVVATGIVARSALRAVKMLREEGIKAGLLKIETIWPFDFELIERIAERVDKLYVPEMNLGQLYHLIKEGANGKAEVKLISKIGGEVHTPMEIFEFIRREFK

>1WTJA

MSASHADQPTQTVSYPQLIDLLRRIFVVHGTSPEVADVLAENCASAQRDGSHSHGIFRIPGYLSSLASGWVDGKAVPVVEDVGAAFVRVDACNGFAQPALAAARSLLIDKARSAGVAILAIRGSHHFAALWPDVEPFAEQGLVALSMVNSMTCVVPHGARQPLFGTNPIAFGAPRAGGEPIVFDLATSAIAHGDVQIAAREGRLLPAGMGVDRDGLPTQEPRAILDGGALLPFGGHKGSALSMMVELLAAGLTGGNFSFEFDWSKHPGAQTPWTGQLLIVIDPDKGAGQHFAQRSEELVRQLHGVGQERLPGDRRYLERARSMAHGIVIAQADLERLQELAGH

>2II2A

GPLGSYGDAIPEVKAILEAKNEEELVTFTSRWSAEERKELRTQFQDTTGLEFIAFLKKCIKNGPYEDVMALGWDCNISARVNVIKKAMKNVNDFRAIHDVVLIATPDERLKLAQAYKEKTGNDLLQDFVDQIPLTSAASYLCHLAIRENRTPRGSVASDAEVLKHNLIDADEPDHEAVVRLIITSTADEYKEINHRFEVLTGKSVQEAIETRYADKENARGLCIAHYYNLAPARAVAYAFHSAVETQNDDMAYEQAARITGLFHDLHKFAWVHYACWGVMRDDILSRFQSKEANKVNFRDACLMFWKLAK

>1EXSA

VEVTPIMTELDTQKVAGTWHTVAMAVSDVSLLDAKSSPLKAYVEGLKPTPEGDLEILLQKRENDKCAQEVLLAKKTDIPAVFKINALDENQLFLLDTDYDSHLLLCMENSASPEHSLVCQSLARTLEVDDQIREKFEDALKTLSVPMRILPAQLEEQCRV

>3VPZA

SLHSSAQFDPILVADIGGTNARFALITAFDAAKNEFVIEYNHTFPSADFGSLQNATRHYLSTVPHIKPVRACLAVAGPIKAGQVHLTNLGWHFSVSEFKQAFSFLQLEVINDFAAFAYAAPYLDSNQNVVIKAGQADENSNIAVMGPGTGFGAACLVRTAQSSAVLSSEGGHISLAAVTDLDAKLLIELRKEHPHVSLETVFSGPGIAHLYKAMAAVNGITAKHLDAAQISNLANTGECEVCDATLNQFCDWLGSAAGDLALAYGALGGLFIGGGILPRMQSRLLESRFVERFSQKGIMSQYNGQVPVTLVTQDNIPLIGAAACLHNSKQE

>2W3QA

PLGSMPFHAEPLKPSDEIDMDLGHSVAAQKFKEIREVLEGNRYWARKVTSEEPEFMAEQVKGQAPNFLWIGCADSRVPEVTIMARKPGDVFVQRNVANQFKPEDDSSQALLNYAIMNVGVTHVMVVGHTGCGGCIAAFDQPLPTEENPGGTPLVRYLEPIIRLKHSLPEGSDVNDLIKENVKMAVKNVVNSPTIQGAWEQARKGEFREVFVHGWLYDLSTGNIVDLNVTQGPHPFVDDRVPRA

>3EJKA

GMDIMLNTADISAAAILLPVEGAQLSELRQIPAEGGPVLHMLRLDSPQFSQFGEIYFSEVLPRRVKAWKRHSLMTQLFAVPVGCIHVVLYDGREKSPTSGRLAQVTLGRPDNYRLLRIPPQVWYGFAATGDTPALVANCTDIPHRQGESERAPQDAPFIPFSWAGADLSGTPVM

>4F7UP

MVLIMHVSPPEHGLLYTANNIKLKLGDKVVGEGTVYIAQNTLSWQPTELAEGISIEWKQVSLHGISSNPRKCIYFMLDHKVEWNGVYGDVDEQFGEVTECWLMPEDIATVDTMYSAMTTCQALHHHHHH

>3ATVA

GSFTMAKAAAIGIDLGTTYSCVGVFQHGKVEIIANDQGNRTTPSYVAFTDTERLIGDAAKNQVALNPQNTVFDAKRLIGRKFGDPVVQSDMKHWPFQVINDGDKPKVQVSYKGETKAFYPEEISSMVLTKMKEIAEAYLGYPVTNAVITVPAYFNDSQRQATKDAGVIAGLNVLRIINEPTAAAIAYGLDRTGKGERNVLIFDLGGGTFDVSILTIDDGIFEVKATAGDTHLGGEDFDNRLVNHFVEEFKRKHKKDISQNKRAVRRLRTACERAKRTLSSSTQASLEIDSLFEGIDFYTSITRARFEELCSDLFRSTLEPVEKALRDAKLDKAQIHDLVLVGGSTRIPKVQKLLQDFFNGRDLNKSINPDEAVAYGAAVQAAILMGDKSENV

>1Z2WA

GSPEFGTRDRMLVLVLGDLHIPHRCNSLPAKFKKLLVPGKIQHILCTGNLCTKESYDYLKTLAGDVHIVRGDFDENLNYPEQKVVTVGQFKIGLIHGHQVIPWGDMASLALLQRQFDVDILISGHTHKFEAFEHENKFYINPGSATGAYNALETNIIPSFVLMDIQASTVVTYVYQLIGDDVKVERIEYKKS

>4EQBA

SNANSRDSQKLVIYNWGDYIDPELLTQFTEETGIQVQYETFDSNEAMYTKIKQGGTTYDIAIPSEYMINKMKDEDLLVPLDYSKIEGIENIGPEFLNQSFDPGNKFSIPYFWGTLGIVYNETMVDEAPEHWDDLWKLEYKNSIMLFDGAREVLGLGLNSLGYSLNSKDPQQLEETVDKLYKLTPNIKAIVADEMKGYMIQNNVAIGVTFSGEASQMLEKNENLRYVVPTEASNLWFDNMVIPKTVKNQDSAYAFINFMLKPENALQNAEYVGYSTPNLPAKELLPEETKEDKAFYPDVETMKHLEVYEKFDHKWTGKYSDLFLQFKMYRK

>1U5UA

MTWKNFGFEIFGEKYGQEELEKRIKDEHTPPPDSPVFGGLKLKLKKEKFKTLFTLGTTLKGFRRATHTVGTGGIGEITIVNDPKFPEHEFFTAGRTFPARLRHANLKYPDDAGADARSFSIKFADSDSDGPLDIVMNTGEANIFWNSPSLEDFVPVEEGDAAEEYVYKNPYYYYNLVEALRRAPDTFAHLYYYSQVTMPFKAKDGKVRYCRYRALPGDVDIKEEDESGRLTEEEQRKIWIFSRHENEKRPDDYLRKEYVERLQKGPVNYRLQIQIHEASPDDTATIFHAGILWDKETHPWFDLAKVSIKTPLSPDVLEKTAFNIANQPASLGLLEAKSPEDYNSIGELRVAVYTWVQHLRKLKIGSLVPAGQNA

>2VK2A

APLTVGFSQVGSESGWRAAETNVAKSEAEKRGITLKIADGQQKQENQIKAVRSFVAQGVDAIFIAPVVATGWEPVLKEAKDAEIPVFLLDRSIDVKDKSLYMTTVTADNILEGKLIGDWLVKEVNGKPCNVVELQGTVGASVAIDRKKGFAEAIKNAPNIKIIRSQSGDFTRSKGKEVMESFIKAENNGKNICMVYAHNDDMVIGAIQAIKEAGLKPGKDILTGSIDGVPDIYKAMMDGEANASVELTPNMAGPAFDALEKYKKDGTMPEKLTLTKSTLYLPDTAKEELEKKKNMGYLEHHHHHHH

>3V5UA

MVILGVGYFLLGLILLYYGSDWFVLGSERIARHFNVSNFVIGATVMAIGTSLPEILTSAYASYMHAPGISIGNAIGSCICNIGLVLGLSAIISPIIVDKNLQKNILVYLLFVIFAAVIGIDGFSWIDGVVLLILFIIYLRWTVKNGSAEIEENNDKNNPSVVFSLVLLIIGLIGVLVGAELFVDGAKKIALALDISDKVIGFTLVAFGTSLPELMVSLAAAKRNLGGMVLGNVIGSNIADIGGALAVGSLFMHLPAENVQMAVLVIMSLLLYLFAKYSKIGRWQGILFLALYIIAIASLRMGGGSLVPRGSRSHHHHHHH

>3G3SA

GMAEQMRRVARLFGDWPETIIWTCLEGTMGDIYVDDSQSPQSALALYGRQSFFGFLAGQPHRDLLKICEGKNIILVPQNQAWSDLIEEVYGDGVRFFTRYATKKDTEFDLGHLQKLVDDLPESFDMKLIDRNLYETCLVEEWSRDLVGNYIDVEQFLDLGLGCVILHKGQVVSGASSYASYSAGIEIEVDTREDYRGLGLAKACAAQLILACLDRGLYPSWDAHTLTSLKLAEKLGYELDKAYQAYEWR

>1NEPA

EPVKFKDCGSWVGVIKEVNVSPCPTQPCKLHRGQSYSVNVTFTSNTQSQSSKAVVHGIVMGIPVPFPIPESDGCKSGIRCPIEKDKTYNYVNKLPVKNEYPSIKVVVEWELTDDKNQRFFCWQIPIEVEA

>3M6NA

MGSSHHHHHHSQDPNSMSAVQPFIRTNIGSTLRIIEEPQRDVYWIHMHADLAINPGRACFSTRLVDDITGYQTNLGQRLNTAGVLAPHVVLASDSDVFNLGGDLALFCQLIREGDRARLLDYAQRCVRGVHAFHVGLGARAHSIALVQGNALGGGFEAALSCHTIIAEEGVMMGLPEVLFDLFPGMGAYSFMCQRISAHLAQKIMLEGNLYSAEQLLGMGLVDRVVPRGQGVAAVEQVIRESKRTPHAWAAMQQVREMTTAVPLEEMMRITEIWVDTAMQLGEKSLRTMDRLVRAQSRRSGLDAG

>4H2DA

GSFTMPSPQLLVLFGSQTGTAQDVSERLGREARRRRLGCRVQALDSYPVVNLINEPLVIFVCATTGQGDPPDNMKNFWRFIFRKNLPSTALCQMDFAVLGLGDSSYAKFNFVAKKLHRRLLQLGGSALLPVCLGDDQHELGPDAAVDPWLRDLWDRVLGLYPPPP

>2AG4A

HMSSFSWDNCDEGKDPAVIRSLTLEPDPIVVPGNVTLSVVGSTSVPLSSPLKVDLVLEKEVAGLWIKIPCTDYIGSCTFEHFCDVLDMLIPTGEPCPEPLRTYGLPCHCPFKEGTYSLPKSEFVVPDLELPSWLTTGNYRIESVLSSSGKRLGCIKIAASLKGI

>1JX6A

VLNGYWGYQEFLDEFPEQRNLTNALSEAVRAQPVPLSKPTQRPIKISVVYPGQQVSDYWVRNIASFEKRLYKLNINYQLNQVFTRPNADIKQQSLSLMEALKSKSDYLIFTLDTTRHRKFVEHVLDSTNTKLILQNITTPVREWDKHQPFLYVGFDHAEGSRELATEFGKFFPKHTYYSVLYFSEGYISDVRGDTFIHQVNRDNNFELQSAYYTKATKQSGYDAAKASLAKHPDVDFIYACSTDVALGAVDALAELGREDIMINGWGGGSAELDAIQKGDLDITVMRMNDDTGIAMAEAIKWDLEDKPVPTVYSGDFEIVTKADSPERIEALKKRAFRYSDN

>3IFRA

MSLAQGRQVIGLDIGTTSTIAILVRLPDTVVAVASRPTTLSSPHPGWAEEDPAQWWDNARAVLAELKTTAGESDWRPGGICVTGMLPAVVLLDDRGAVLRPSIQQSDGRCGDEVAELRAEVDSEAFLARTGNGVTQQLVTAKLRWIERHEPAVFGAIATVCGSYDYINMLLTGERVVDRNWALEGGFIDLASGTVEADLVALAHIPPSAVPPAHPTHRVLGAVTAEAAALTGLPTGLPVYGGAADHIASALAAGITRPGDVLLKFGGAGDIIVASATAKSDPRLYLDYHLVPGLYAPNGCMAATGSALNWLAKLLAPEAGEAAHAQLDALAAEVPAGADGLVCLPYFLGEKTPIHDPFASGTFTGLSLSHTRGHLWRALLEAVALAFRHHVAVLDDIGHAPQRFFASDGGTRSRVWMGIMADVLQRPVQLLANPLGSAVGAAWVAAIGGGDDLGWDDVTALVRTGEKITPDPAKAEVYDRLYRDFSALYATLHPFFHRSREGHHHHHH

>3GIWA

GMGGAALPDNGWPADRIDTESAHSARIYDYIIGGKDYYPADKEAGDAMSREWPALPVHMRANRDWMNRAVAHLAKEAGIRQFLDIGTGIPTSPNLHEIAQSVAPESRVVYVDNDPIVLTLSQGLLASTPEGRTAYVEADMLDPASILDAPELRDTLDLTRPVALTVIAIVHFVLDEDDAVGIVRRLLEPLPSGSYLAMSIGTAEFAPQEVGRVAREYAARNMPMRLRTHAEAEEFFEGLELVEPGIVQVHKWHPDAATADGIRDEDIAMYGAVARKP

>3DQYA

TWTYILRQGDLPPGEMQRYEGGPEPVMVCNVDGEFFAVQDTCTHGDWALSDGYLDGDIVECTLHFGKFCVRTGKVKALPACKPIKVFPIKVEGDEVHVDLDNGELK

>4IPIA

GAMQNNNEFKIGNRSVGYNHEPLIICEIGINHEGSLKTAFEMVDAAYNAGAEVVKHQTHIVEDEMSDEAKQVIPGNADVSIYEIMERCALNEEDEIKLKEYVESKGMIFISTPFSRAAALRLQRMDIPAYKIGSGECNNYPLIKLVASFGKPIILSTGMNSIESIKKSVEIIREAGVPYALLHCTNIYPTPYEDVRLGGMNDLSEAFPDAIIGLSDHTLDNYACLGAVALGGSILERHFTDRMDRPGPDIVCSMNPDTFKELKQGAHALKLARGGKKDTIIAGEKPTKDFAFASVVADKDIKKGELLSGDNLWVKAPGNGDFSVNEYETLFGKVAACNIRKGAQIKKTDIE

>4ASCA

MFLQDLIFMISEEGAVAYDPAANECYCASLSSQVPKNHVSLVTKENQVFVAGGLFYNEDNKEDPMSAYFLQFDHLDSEWLGMPPLPSPRCLFGLGEALNSIYVVGGREIKDGERCLDSVMCYDRLSFKWGESDPLPYVVYGHTVLSHMDLVYVIGGKGSDRKCLNKMCVYDPKKFEWKELAPMQTARSLFGATVHDGRIIVAAGVTDTGLTSSAEVYSITDNKWAPFEAFPQERSSLSLVSLVGTLYAIGGFATLETESGELVPTELNDIWRYNEEEKKWEGVLREIAYAAGATFLPVRLNVLRLTKMAENLYFQ

>2BLNA

MKTVVFAYHDMGCLGIEALLAAGYEISAIFTHTDNPGEKAFYGSVARLAAERGIPVYAPDNVNHPLWVERIAQLSPDVIFSFYYRHLIYDEILQLAPAGAFNLHGSLLPKYRGRAPLNWVLVNGETETGVTLHRMVKRADAGAIVAQLRIAIAPDDIAITLHHKLCHAARQLLEQTLPAIKHGNILEIAQRENEATCFGRRTPDDSFLEWHKPASVLHNMVRAVADPWPGAFSYVGNQKFTVWSSRVHPHASKAQPGSVISVAPLLIACGDGALEIVTGQAGDGITMQGSQLAQTLGLVQGSRLN

>3TMPA

GSHMGAGYNSEDEYEAAAARIEAMDPATVEQQEHWFEKALRDKKGFIIKQMKEDGACLFRAVADQVYGDQDMHEVVRKHCMDYLMKNADYFSNYVTEDFTTYINRKRKNNCHGNHIEMQAMAEMYNRPVEVYQYSTGTSAVEPINTFHGIHQNEDEPIRVSYHRNIHYNSVVNPNKATIGVGLG

>3ISYA

GMENQEVVLSIDAIQEPEQIKFNMSLKNQSERAIEFQFSTGQKFELVVYDSEHKERYRYSKEKMFTQAFQNLTLESGETYDFSDVWKEVPEPGTYEVKVTFKGRAENLKQVQAVQQFEVK

>2GR8A

ASWSHPQFEKSGGGGGLVPRGSKRADAGTASALAASQLPQATMPGKSMVAIAGSSYQGQNGLAIGVSRISDNGKVIIRLSGTTNSQGKTGVAAGVGYQW

>2EHZA

MSKQAAVIELGYMGISVKDPDAWKSFATDMLGLQVLDEGEKDRFYLRMDYWHHRIVVHHNGQDDLEYLGWRVAGKPEFEALGQKLIDAGYKIRICDKVEAQERMVLGLMKTEDPGGNPTEIFWGPRIDMSNPFHPGRPLHGKFVTGDQGLGHCIVRQTDVAEAHKFYSLLGFRGDVEYRIPLPNGMTAELSFMHCNARDHSIAFGAMPAAKRLNHLMLEYTHMEDLGYTHQQFVKNEIDIALQLGIHANDKALTFYGATPSGWLIEPGWRGATAIDEAEYYVGDIFGHGVEATGYGLDVKLS

>2WLRA

MASAELAKPLTLDQLQQQNGKAIDTRPSAFYNGWPQTLNGPSGHELAALNLSASWLDKMSTEQLNAWIKQHNLKTDAPVALYGNDKDVDAVKTRLQKAGLTHISILSDALSEPSRLQKLPHFEQLVYPQWLHDLQQGKEVTAKPAGDWKVIEAAWGAPKLYLISHIPGADYIDTNEVESEPLWNKVSDEQLKAMLAKHGIRHDTTVILYGRDVYAAARVAQIMLYAGVKDVRLLDGGWQTWSDAGLPVERGTPPKVKAEPDFGVKIPAQPQLMLDMEQARGLLHRQDASLVSIRSWPEFIGTTSGYSYIKPKGEIAGARWGHAGSDSTHMEDFHNPDGTMRSADDITAMWKAWNIKPEQQVSFYCGTGWRASETFMYARAMGWKNVSVYDGGWYEWSSDPKNPVATGERGPDSSKLEHHHHHH

>3CBZA

GSHMNIITVTLNMEKYNFLGISIVGQSNERGDGGIYIGSIMKGGAVAADGRIEPGDMLLQVNDMNFENMSNDDAVRVLRDIVHKPGPIVLTVAKSGGGSGNEVWIDGP

>3UAFA

KTSCLMATGVLKCPTDPEAVKKVHIDLWDAAAAAAESDDLMGRTWSDRNGNFQVTGCASDFGPINTPDPYLYIQHNCPHRDSNATNPIQIDVIPLFLPSIVRLGNVYLDRYLEDYHH

>1PBYC

MNALVGCTTSFDPGWEVDAFGAVSNLCQPMEADLYGCADPCWWPAQVADTLNTYPNWSAGADDVMQDWRKLQSVFPETK

>3VLDA

MGSSHHHHHHSSGLVPRGSHMSEKETNYVENLLTQLENELNEDNLPEDINTLLRKCSLNLVTVVSLPDMDVKPLLATIKRFLTSNVSYDSLNYDYLLDVVDKLVPMADFDDVLEVYSAEDLVKALRSEIDPLKVAACRVIENSQPKGLFATSNIIDILLDILFDEKVENDKLITAIEKALERLSTDELIRRRLFDNNLPYLVSVKGRMETVSFVRLIDFLTIEFQFISGPEFKDIIFCFTKEEILKSVEDILVFIELVNYYTKFLLEIRNQDKYWALRHVKKILPVFAQLFEDTENYPDVRAFSTNCLLQLFAEVSRIEEDEYSLFKTMDKDSLKIGSEAKLITEWLELINPQYLVKYHKDVVENYFHVSGYSIGMLRNLSADEECFNAIRNKFSAEIVLRLPYLEQMQVVETLTRYEYTSKFLLNEMPKVMGSLIGDGSAGAIIDLETVHYRNSALRNLLDKGEEKLSVWYEPLLREYSKAVNGKNYSTGSETKIADCR

>2FWHA

ATHTAQTQTHLNFTQIKTVDELNQALVEAKGKPVMLDLYADWCVACKEFEKYTFSDPQVQKALADTVLLQANVTANDAQDVALLKHLNVLGLPTILFFDGQGQEHPQARVTGFMDAETFSAHLRDRQPHHHHHH

>2HIQA

MTDPALRAATLLQLHFAFNGPFGDAMAEQLKPLAESINQEPGFLWKVWTESEKNHEAGGIYLFTDEKSALAYLEKHTARLKNLGVEEVVAKVFDVNEPLSQINQAKLAGLCGR

>3UAUA

MGHHHHHHHHHHSSGHIDDDDKHMCGNSIDEKTVKKYENQLNQTVKQEIASLSQDSGIKIEFSDFKCNADGDFIACLSPNFKTLAKDNNDEYQELFQAKNIKIRSNEIYKGETNTSISIKEYYNDLFKNQKSIQSNLVFEDFKLGEKVVSDINASLFQQDPKISSFINKLSSDSYTLSFDNSINKQENNYLDNLDIKFYNAKLNFNTNLNINLKEDLLNYLDSKGIKFNTQTLAMDEQAINELLNMVNYEQASDFSNTIQKYIILNNFKIDSTLKTEGVFSSYIATAKENLQTLKAQSQNEEQALIFDKALAILNNITQNDDYKLNLDLKFKNIPVSDYSTQGIDSIEKLSINNQDATEALKIILPFIMFSMLMGGASF

>3D40A

MGSSHHHHHHSSGLVPRGSHMTPDFLAIKVGGSLFSRKDEPGSLDDDAVTRFARNFARLAETYRGRMVLISGGGAFGHGAIRDHDSTHAFSLAGLTEATFEVKKRWAEKLRGIGVDAFPLQLAAMCTLRNGIPQLRSEVLRDVLDHGALPVLAGDALFDEHGKLWAFSSDRVPEVLLPMVEGRLRVVTLTDVDGIVTDGAGGDTILPEVDARSPEQAYAALWGSSEWDATGAMHTKLDALVTCARRGAECFIMRGDPGSDLEFLTAPFSSWPAHVRSTRITTTASA

>3EJNA

GGNLEEMNIDPDNATQTHPKLLLTQICMNAFKRGTDGMYATKKVIQADGESADQYYKWTRGSFGYYDNLRNVQKMGEEAERVNAPVYTALTKFFRAYYFYELTLRFGDIPYSQALKGEKEEIYTPEYDAQEDVFAGILQELREADEILANDASVIDGDIIYNGNSTQWRKLINSFRLKVLMTLSNHTTVGNINIASEFKNIATNSPLMNSLADNGQLVYLDQQGNRYPQFNAQWSGYYMDDTFIQRMRERRDPRLFIFSAQTNKGKTEGKPIDDFSSYEGGDPAAPYSDAIIKVSEGTISPINDRFRTDPIVEPTMLMGYAELQQILAEAVVRGWISGNAQTYYEKGIRASFSFYETHAKDYAGYLNENAVAQYLKEPLVDFTQASGTEEQIERIIMQKYLVTFYQGNWDSFYEQLRTGYPDFRRPAGTEIPKRWMYPQGEYDNNGTNVETAITRQFGAGNDKINQATWWQKKS

>3HVNA

GPLGSRKSSHLILSSIVSLALVGVTPLSVLADSKQDINQYFQSLTYEPQEILTNEGEYIDNPPATTGMLENGRFVVLRREKKNITNNSADIAVIDAKAANIYPGALLRADQNLLDNNPTLISIARGDLTLSLNLPGLANGDSHTVVNSPTRSTVRTGVNNLLSKWNNTYAGEYGNTQAELQYDETMAYSMSQLKTKFGTSFEKIAVPLDINFDAVNSGEKQVQIVNFKQIYYTVSVDEPESPSKLFAEGTTVEDLKRNGITDEVPPVYVSSVSYGRSMFIKLETSSRSTQVQAAFKAAIKGVDISGNAEYQDILKNTSFSAYIFGGDAGSAATVVSGNIETLKKIIEEGARYGKLNLGVPISYSTNFVKDNRPAQILSNSEYIETTSTVHNSSALTLDHSGAYVAKYNITWEEVSYNEAGEEVWEPKAWDKNGVNLTSHWSETIQIPGNARNLHVNIQECTGLAWEWWRTVYDKDLPLVGQRKITIWGTTLYPQYADEVIELERPHRD

>3CWNA

MGSSHHHHHHSSGLVPRGSHMTDKLTSLRQYTTVVADTGDIAAMKLYQPQDATTNPSLILNAAQIPEYRKLIDDAVAWAKQQSNDRAQQIVDATDKLAVNIGLEILKLVPGRISTEVDARLSYDTEASIAKAKRLIKLYNDAGISNDRILIKLASTWQGIRAAEQLEKEGINCNLTLLFSFAQARACAEAGVFLISPYVGRILDWYKANTDKKEYAPAEDPGVVSVSEIYQYYKEHGYETVVMGASFRNIGEILELAGCDRLTIAPTLLKELAESEGAIERKLSYTGEVKARPARITESEFLWQHNQDPMAVDKLAEGIRKFAIDQEKLEKMIGDLL

>3MKCA

MSLALNPAVAPIKSIEFIPVNYQASNWSQNTVVVKVTDENGVYGLGEADGSPDAILAYANIETEHKWLTNITEKAIGRLPIEINAIWDAMYDATQWQGMRGLGMFALSGIDMALYDLAGKQLGVPAYQLLGGTNKDKVHPYLTLYPAIPVDASLDVAIKGYAPLLEKAKAHNIRAVKVCVPIKADWSTKEVAYYLRELRGILGHDTDMMVDYLYRFTDWYEVARLLNSIEDLELYFAEATLQHDDLSGHAKLVENTRSRICGAEMSTTRFEAEEWITKGKVHLLQSDYNRCGGLTELRRITEMATANNVQVMPHNWKTGITSAAAIHYQFAVGNAPYFEYVHPEFCDGELRKYLVTPEAELVDGGFAKPTAPGLGIDLNQEFLASLEGHHHHHH

>2CDUA

MKVIVVGCTHAGTFAVKQTIADHPDADVTAYEMNDNISFLSCGIALYLGKEIKNNDPRGLFYSSPEELSNLGANVQMRHQVTNVDPETKTIKVKDLITNEEKTEAYDKLIMTTGSKPTVPPIPGIDSSRVYLCKNYNDAKKLFEEAPKAKTITIIGSGYIGAELAEAYSNQNYNVNLIDGHERVLYKYFDKEFTDILAKDYEAHGVNLVLGSKVAAFEEVDDEIITKTLDGKEIKSDIAILCIGFRPNTELLKGKVAMLDNGAIITDEYMHSSNRDIFAAGDSAAVHYNPTNSNAYIPLATNAVRQGRLVGLNLTEDKVKDMGTQSSSGLKLYGRTYVSTGINTALAKANNLKVSEVIIADNYRPEFMLSTDEVLMSLVYDPKTRVILGGALSSMHDVSQSANVLSVCIQNKNTIDDLAMVDMLFQPQFDRPFNYLNILGQAAQAQADKAHK

>4ID2A

GCGGKKGSSDNTSTLAMIDSVDAHGLQRMQTSKSETDFKFKGKDYHSLVSRTPDDNLPHVTNELGDTYVDNKIVLHLTRGNETVLNKTFTKNDFSSVVDANFLSKSILEGIVYDKTTPQGIVYAASVCYPQTDLYMPLSITITADGKMSIQKVDILEEDYDDEAPN

>3H9MA

MSLSFTPLHTTSEAFIEKALPWLEDRYFHIAYLNPNGYTAYPQGAFRHYLAFGSEAAIHVSDATRVFETWNEIKKGYTNEWIFVFASYDGKNSVEQLHTSKEAGIAFAAATFFIPEHVWEIQPDGILIHKGSGSSLVTEIQHAEPSTPVQQSDIFVKQVVSKESYFNAFDELQQIIAQGDAYEINYCIPFTAKGNISPAATYQRLNKKTPMPFSVYYKFNTEYILSASPERFIKKTGDTIISQPIKGTSKRGKSKAEDEMLKQQLGTSEKEQSENTMIVDLVRNDLSRTAVAGSVCVPELSGLYTFPNVHQLISTVQSTIDPACSSIDVIQQAFPMGSMTGAPKVNVMKFIDRIESMARGPFSGTVGYMDPHDNFDFNVLIRSIFYNSATQELFMEAGSAITSYAKAETEYEECLLKITPMIHILNNQEGHHHHHH

>3LATA

VSSQKTSSLPKYTPKVNSSINNYIRKKNMKAPRIEEDYTSYFPKYGYRNGVGRPEGIVVHDTANDNSTIDGEIAFMKRNYTNAFVHAFVDGNRIIETAPTDYLSWGAGPYGNQRFINVEIVHTHDYDSFARSMNNYADYAATQLQYYNLKPDSAENDGRGTVWTHAAISNFLGGTDHADPHQYLRSHNYSYAELYDLIYEKYLIKTKQVAPWG

>4HPVA

GSHMRNINVQLNPLSDIEKLQVELVERKGLGHPDYIADAVAEEASRKLSLYYLKKYGVILHHNLDKTLVVGGQATPRFKGGDIIQPIYIIVAGRATTEVKTESGIDQIPVGTIIIESVKEWIRNNFRYLDAERHVIVDYKIGKGSSDLVGIFEASKRVPLSNDTSFGVGFAPLTKLEKLVYETERHLNSKQFKAKLPEVGEDIKVMGLRRGNEVDLTIAMATISELIEDVNHYINVKEQVRNQILDLASKIAPGYNVRVYVNTGDKIDKNILYLTVTGTSAEHGDDGMTGRGNRGVGLITPMRPMSLEATAGKNPVNHVGKLYNVLANLIANKIAQEVKDVKFSQVQVLGQIGRPIDDPLIANVDVITYDGKLTDETKNEISGIVDEMLSSFNKLTELILEGKATLF

>3IO3A

MDLELEPTLESIVQHDSLKWIFVGGKGGVGKTTTSSSVAVQLALAQPNEQFLLISTDPAHNLSDAFCQKFGKDARKVEGLPNLSCMEIDPEAAMSDLQQQASQYNNDPNDPLKSMMSDMTGSIPGIDEALSFMEVLKHIKNQKVLEGEDNSNAISYKTIIFDTAPTGHTLRFLQLPSTLEKLLSKFKDLSGKLGPMLSMMGGGQQQDIFEKLNEVQKNVSEVNEQFTNPELTTFICVCISEFLSLYETERMIQELMSYNMDVNSIVVNQLLFAEGDDHSCKRCESRWKMQKKYLDQMGELYEDYHLVKMPLLGCEIRGVENLKKFSKFLLKPYDPKADSDIVFDLEEK

>1YUMA

MGSSHHHHHHSSGLVPRGSHMGKRIGLFGGTFDPVHIGHMRSAVEMAEQFALDELRLLPNARPPHRETPQVSAAQRLAMVERAVAGVERLTVDPRELQRDKPSYTIDTLESVRAELAADDQLFMLIGWDAFCGLPTWHRWEALLDHCHIVVLQRPDADSEPPESLRDLLAARSVADPQALKGPGGQITFVWQTPLAVSATQIRALLGAGRSVRFLVPDAVLNYIEAHHLYRAPHLEHHHHHH

>3D02A

GAAEKTVVNISKVDGMPWFNRMGEGVVQAGKEFNLNASQVGPSSTDAPQQVKIIEDLIARKVDAITIVPNDANVLEPVFKKARDAGIVVLTNESPGQPSANWDVEIIDNEKFAAEYVEHMAKRMGGKGGYVIYVGSLTVPQHNLWADLLVKYQKEHYPDMHEVTRRMPVAESVDDSRRTTLDLMKTYPDLKAVVSFGSNGPIGAGRAVKEKRAKNKVAVYGMMIPSQAASLIKSGDITEGITYDPATAGYALAAVASTLLNGKTIEPGFELKELGKAEVDSDKHIIRFHKVLLVNKDNIDSLY

>3D1BA

GSHMNPLASLTTDKNDLYINWLKSLSFFQTNSSCAEALVKVIPHYHNKLIDFSQVLQLVFSASEKFPIQENQPLPEQLMFLSNLEKQTPFAKAVGSSIYKLVTGKNLSLDFASQILKEASILEH

>2HBOA

GMSDDLTDAQTAAIPEGFSQLNWSRGFGRQIGPLFEHREGPGQARLAFRVEEHHTNGLGNCHGGMLMSFADMAWGRIISLQKSYSWVTVRLMCDFLSGAKLGDWVEGEGELISEEDMLFTVRGRIWAGERTLITGTGVFKALSARKPRPGELAYKEEA

>3TEWA

EVKQENRLLNESESSSQGLLGYYFSDLNFQAPMVVTSSTTGDLSIPSSELENIPSENQYFQSAIWSGFIKVKKSDEYTFATSADNHVTMWVDDQEVINKASNSNKIRLEKGRLYQIKIQYQRENPTEKGLDFKLYWTDSQNKKEVISSDNLQLPELKQKSSNSRKKRSTSAGPTVPDRDNDGIPDSLEVEGYTVDVKNKRTFLSPWISNIHEKKGLTKYKSSPEKWSTASDPYSDFEKVTGRIDKNVSPEARHPLVAAYPIVHVDMENIILSKNEDQSTQNTDSQTRTISKNTSTSRTHTSEPGSNSNSSTVAIDHSLSLAGERTWAETMGLNTADTARLNANIRYVNTGTAPIYNVLPTTSLVLGKNQTLATIKAKENQLSQILAPNNYYPSKNLAPIALNAQDDFSSTPITMNYNQFLELEKTKQLRLDTDQVYGNIATYNFENGRVRVDTGSNWSEVLPQIQETTARIIFNGKDLNLVERRIAAVNPSDPLETTKPDMTLKEALKIAFGFNEPNGNLQYQGKDITEFDFNFDQQTSQNIKNQLAELNATNIYTVLDKIKLNAKMNILIRDKRFHYDRNNIAVGADESVVKEAHREVINSSTEGLLLNIDKDIRKILSGYIVEIEDTEGLKEVINDRYDMLNISSLRQDGKTFIDFKKYNDKLPLYISNPNYKVNVYAVTKENTIINPSENGDTSTNGIKKILIFSKKGYEIG

>2BLNA

MKTVVFAYHDMGCLGIEALLAAGYEISAIFTHTDNPGEKAFYGSVARLAAERGIPVYAPDNVNHPLWVERIAQLSPDVIFSFYYRHLIYDEILQLAPAGAFNLHGSLLPKYRGRAPLNWVLVNGETETGVTLHRMVKRADAGAIVAQLRIAIAPDDIAITLHHKLCHAARQLLEQTLPAIKHGNILEIAQRENEATCFGRRTPDDSFLEWHKPASVLHNMVRAVADPWPGAFSYVGNQKFTVWSSRVHPHASKAQPGSVISVAPLLIACGDGALEIVTGQAGDGITMQGSQLAQTLGLVQGSRLN

>3B9OA
[truncated: 232,873 more chars]
