# Supplementary material for: enDNA-Prot: Identification of DNA-Binding Proteins by Applying Ensemble Learning
Source: Biomed Res Int. 2014 May 26;2014:294279. doi: 10.1155/2014/294279 (PMC4058174; doi:10.1155/2014/294279)
Supplement: Supplementary file 1 — Supplementary Material S1 lists all the codes and sequences for the benchmark dataset. It contains 396 proteins, classified into 146 DNA-binding proteins and 250 non DNA-binding proteins. Supplementary Material S2 lists all the codes and sequences for the expanded benchmark dataset. It contains 2271 proteins, classified into 146 DNA-binding proteins and 2125 non DNA-binding proteins. Supplementary Material S3 lists all the codes and sequences for the independent dataset1. It contains 182 proteins, classified into 82 DNA-binding proteins and 100 non DNA-binding proteins. Supplementary Material S4 lists all the codes and sequences for the independent dataset2. It contains 1585 proteins, classified into 770 DNA-binding proteins and 815 non DNA-binding proteins. [file 294279.f1.zip › 294279.f1/S3.docx]

Online Supporting Information S3. The sequences in independent dataset1 are shown here. It contains 82 DNA-binding protein sequences and 100 non DNA-binding proteins sequences.

|  |
| --- |

(1).DNA-binding proteins:

>1AM9:A

QSRGEKRTAHNAIEKRYRSSINDKIIELKDLVVGTEAKLNKSAVLRKAIDYIRFLQHSNQKLKQENLSLRTAVHKSKSLKDL

>1AN4:A

MDEKRRAQHNEVERRRRDKINNWIVQLSKIIPDSSMESTKSGQSKGGILSKASDYIQELRQSNHR

>1C9B:A

SDRAMMNAFKEITTMADRINLPRNIVDRTNNLFKQVYEQKSLKGRANDAIASACLYIACRQEGVPRTFKEICAVSRISKKEIGRCFKLILKALETSVDLITTGDFMSRFCSNLCLPKQVQMAATHIARKAVELDLVPGRSPISVAAAAIYMASQASAEKRTQKEIGDIAGVADVTIRQSYRLIYPRAPDLFPTDFKFDTPVDKLPQL

>1CEZ:A

MNTINIAKNDFSDIELAAIPFNTLADHYGERLAREQLALEHESYEMGEARFRKMFERQLKAGEVADNAAAKPLITTLLPKMIARINDWFEEVKAKRGKRPTAFQFLQEIKPEAVAYITIKTTLACLTSADNTTVQAVASAIGRAIEDEARFGRIRDLEAKHFKKNVEEQLNKRVGHVYKKAFMQVVEADMLSKGLLGGEAWSSWHKEDSIHVGVRCIEMLIESTGMVSLHRQNAGVVGQDSETIELAPEYAEAIATRAGALAGISPMFQPCVVPPKPWTGITGGGYWANGRRPLALVRTHSKKALMRYEDVYMPEVYKAINIAQNTAWKINKKVLAVANVITKWKHCPVEDIPAIEREELPMKPEDIDMNPEALTAWKRAAAAVYRKDKARKSRRISLEFMLEQANKFANHKAIWFPYNMDWRGRVYAVSMFNPQGNDMTKGLLTLAKGKPIGKEGYYWLKIHGANCAGVDKVPFPERIKFIEENHENIMACAKSPLENTWWAEQDSPFCFLAFCFEYAGVQHHGLSYNCSLPLAFDGSCSGIQHFSAMLRDEVGGRAVNLLPSETVQDIYGIVAKKVNEILQADAINGTDNEVVTVTDENTGEISEKVKLGTKALAGQWLAYGVTRSVTKRSVMTLAYGSKEFGFRQQVLEDTIQPAIDSGKGLMFTQPNQAAGYMAKLIWESVSVTVVAAVEAMNWLKSAAKLLAAEVKDKKTGEILRKRCAVHWVTPDGFPVWQEYKKPIQTRLNLMFLGQFRLQPTINTNKDSEIDAHKQESGIAPNFVHSQDGSHLRKTVVWAHEKYGIESFALIHDSFGTIPADAANLFKAVRETMVDTYESCDVLADFYDQFADQLHESQLDKMPALPAKGNLNLRDILESDFAFA

>1CF7:B

RSKKGDKNGKGLRHFSMKVCEKVQRKGTTSYNEVADELVSEFTNSNNHLAADSAYDQKNIRRRVYDALNVLMAMNIISKEKKEIKWIGLPTNSAQ

>1CW0:A

ADVHDKATRSKNMRAIATRDTAIEKRLASLLTGQGLAFRVQDASLPGRPDFVVDEYRCVIFTHGCFWHHHHCYLFKVPATRTEFWLEKIGKNVERDRRDISRLQELGWRVLIVWECALRGREKLTDEALTERLEEWICGEGASAQIDTQGIHLLA

>1D0E:A

GSHMTWLSDFPQAWAETGGMGLAVRQAPLIIPLKATSTPVSIKQYPMSQEARLGIKPHIQRLLDQGILVPCQSPWNTPLLPVKKPGTNDYRPVQDLREVNKRVEDIHPTVPNPYNLLSGLPPSHQWYTVLDLKDAFFCLRLHPTSQPLFAFEWRDPEMGISGQLTWTRLPQGFKNSPTLFDEALHRDLADFRIQHPDLILLQYVDDLLLAATSELDCQQGTRALLQTLGNLGYRASAKKAQICQKQVKYLGYLLKEGQR

>1D5Y:A

QAGIIRDLLIWLEGHLDQPLSLDNVAAKAGYSKWHLQRMFKDVTGHAIGAYIRARRLSKSAVALRLTARPILDIALQYRFDSQQTFTRAFKKQFAQTPALYRRSPEWSAFGIRPPLRLGEFTMPEHKFVTLEDTPLIGVTQSYSCSLEQISDFRHEMRYQFWHDFLGNAPTIPPVLYGLNETRPSQDKDDEQEVFYTTALAQDQADGYVLTGHPVMLQGGEYVMFTYEGLGTGVQEFILTVYGTCMPMLNLTRRKGQDIERYYPAEDAKAGDRPINLRCELLIPIRRKLAAA

>1DH3:A

KREVRLMKNREAARESRRKKKEYVKSLENRVAVLENQNKTLIEELKALKDLYSHK

>1EA4:A

MKKRLTITLSESVLENLEKMAREMGLSKSAMISVALENYKKGQEK

>1EBM:A

GSEGHRTLASTPALWASIPCPRSELRLDLVLPSGQSFRWREQSPAHWSGVLADQVWTLTQTEEQLHCTVYRGDKSQASRPTPDELEAVRKYFQLDVTLAQLYHHWGSVDSHFQEVAQKFQGVRLLRQDPIECLFSFICSSNNNIARITGMVERLCQAFGPRLIQLDDVTYHGFPSLQALAGPEVEAHLRKLGLGYRARYVSASARAILEEQGGLAWLQQLRESSYEEAHKALCILPGVGTQVADCICLMALDKPQAVPVDVHMWHIAQRDYSWHPTTSQAKGPSPQTNKELGNFFRSLWGPYAGWAQAVLFSADLRQ

>1EMH:A

MEFFGESWKKHLSGEFGKPYFIKLMGFVAEERKHYTVYPPPHQVFTWTQMCDIKDVKVVILGQDPYHGPNQAHGLCFSVQRPVPPPPSLENIYKELSTDIEDFVHPGHGDLSGWAKQGVLLLNAVLTVRAHQANSHKERGWEQFTDAVVSWLNQNSNGLVFLLWGSYAQKKGSAIDRKRHHVLQTAHPSPLSVYRGFFGCRHFSKTNELLQKSGKKPIDWKEL

>1EOO:A

MSLRSDLINALYDENQKYDVCGIISAEGKIYPLGSDTKVLSTIFELFSRPIINKIAEKHGYIVEEPKQQNHYPDFTLYKPSEPNKKIAIDIKTTYTNKENEKIKFTLGGYTSFIRNNTKNIVYPFDQYIAHWIIGYVYTRVATRKSSLKTYNINELNEIPKPYKGVKVFLQDKWVIAGDLAGSGNTTNIGSIHAHYKDFVEGKGIFDSEDEFLDYWRNYERTSQLRNDKYNNISEYRNWIYRGRK

>1F2I:G

MEPHPMNNLLNYVVPKMRPYACPVESCDRRFSRSDELTRHIRIHTGQKPFQCRICMRNFSRSDHLTTHIRTHT

>1F4K:A

MKEEKRSSTGFLVKQRAFLKLYMITMTEQERLYGLKLLEVLRSEFKEIGFKPNHTEVYRSLHELLDDGILKQIKVKKEGAKLQEVVLYQFKDYEAAKLYKKQLKVELDRSKKLIEKALSDNF

>1F6O:A

KGHLTRLGLEFFDQPAVPLARAFLGQVLVRRLPNGTELRGRIVETEAYLGPEDEAAHSRGGRQTPRNRGMFMKPGTLYVYIIYGMYFCMNISSQGDGACVLLRALEPLEGLETMRQLRSTLRKGTASRVLKDRELCSGPSKLCQALAINKSFDQRDLAQDEAVWLERGPLEPSEPAVVAAARVGVGHAGEWARKPLRFYVRGSPWVSVVDRVAEQDTQA

>1FJX:A

MIEIKDKQLTGLRFIDLFAGLGGFRLALESCGAECVYSNEWDKYAQEVYEMNFGEKPEGDITQVNEKTIPDHDILCAGFPCQAFSISGKQKGFEDSRGTLFFDIARIVREKKPKVVFMENVKNFASHDNGNTLEVVKNTMNELDYSFHAKVLNALDYGIPQKRERIYMICFRNDLNIQNFQFPKPFELNTFVKDLLLPDSEVEHLVIDRKDLVMTNQEIEQTTPKTVRLGIVGKGGQGERIYSTRGIAIGLSAYGGGIFAKTGGYLVNGKTRKLHPRECARVMGYPDSYKVHPSTSQAYKQFGNSVVINVLQYIAYNIGSSLNFKPY

>1FLO:A

PQFDILCKTPPKVLVRQFVERFERPSGEKIALCAAELTYLCWMITHNGTAIKRATFMSYNTIISNSLSFDIVNKSLQFKYKTQKATILEASLKKLIPAWEFTIIPYYGQKHQSDITDIVSSLQLQFESSEEADKGNSHSKKMLKALLSEGESIWEITEKILNSFEYTSRFTKTKTLYQFLFLATFINCGRFSDIKNVDPKSFKLVQNKYLGVIIQCLVTETKTSVSRHIYFFSARGRIDPLVYLDEFLRNSEPVLKRVNRTGNSSSNKQEYQLLKDNLVRSYNKALKKNAPYSIFAIKNGPKSHIGRHLMTSFLSMKGLTELTNVVGNWSDKRASAVARTTYTHQITAIPDHYFALVSRYYAYDPISKEMIALKDETNPIEEWQHIEQLKGSAEGSIRYPAWNGIISQEVLDYLSSYINRRI

>1FZP:B

AITKINDCFELLSMVTYADKLKSLIKKEFSISFEEFAVLTYISENKEKEYYLKDIINHLNYKQPQVVKAVKILSQEDYFDKKRNEHDERTVLILVNAQQRKKIESLLSRVNKRITEANNEIEL

>1GD2:E

RKNSDQEPSSKRKAQNRAAQRAFRKRKEDHLKALETQVVTLKELHSSTTLENDQLRQKVRQLEEELRILK

>1GXP:A

SPMAVEEVIEMQGLSLDPTSHRVMAGEEPLEMGPTEFKLLHFFMTHPERVYSREQLLNHVWGTNVYVEDRTVDVHIRRLRKALEPGGHDRMVQTVRGTGYRFSTRF

>1H88:A

VKSKAKKTVDKHSDEYKIRRERNNIAVRKSRDKAKMRNLETQHKVLELTAENERLQKKVEQLSRELSTLRNLFKQLPE

>1H9D:A

SMVEVLADHPGELVRTDSPNFLCSVLPTHWRCNKTLPIAFKVVALGDVPDGTLVTVMAGNDENYSAELRNATAAMKNQVARFNDLRFVGRSGRGKSFTLTITVFTNPPQVATYHRAIKITVDGPREPRRHRQKL

>1HLV:A

MGPKRRQLTFREKSRIIQEVEENPDLRKGEIARRFNIPPSTLSTILKNKRAILASERKYGVASTCRKTNKLSPYDKLEGLLIAWFQQIRAAGLPVKGIILKEKALRIAEELGMDDFTASNGWLDRFRRRRS

>1I3J:A

KALYSKPGSKNGRWNPETHKFCKCGVRIQTSAYTCSKCRNRSGENNSFFNHKHSDITKSKISEKMKGKKPSNIKKISCDGVIFDCAADAARHFKISSGLVTYRVKSDKWNWFYINA

>1IAW:A

MTELPLQFAEPDDDLERVRATLYSLDPDGDRTAGVLRDTLDQLYDGQRTGRWNFDQLHKTEKTHMGTLVEINLHREFQFGDGFETDYEIAGVQVDCKFSMSQGAWMLPPESIGHICLVIWASDQQCAWTAGLVKVIPQFLGTANRDLKRRLTPEGRAQVVKLWPDHGKLQENLLLHIPGDVRDQIFSAKSSRGNQHGQARVNELFRRVHGRLIGRAVIATVAQQDDFMKRVRGSGGARSILRPEGIIILGHQDNDPKVANDLGLPVPRKGQVVAARVVPADEGDQRQTAEIQGRRWAVAVPGDPIVEAPVVPRKSAE

>1IC8:A

ILKELENLSPEEAAHQKAVVETLLQEDPWRVAKMVKSYLQQHNIPQREVVDTTGLNQSHLSQHLNKGTPMKTQKRAALYTWYVRKQREVAQQFTHAGQGGLIEEPTGDELPTKKGRRNRFKWGPASQQILFQAYERQKNPSKEERETLVEECNRAECIQRGVSPSQAQGLGSNLVTEVRVYNWFANRRKEEAFR

>1JE8:A

MRGSHHHHHHGSATTERDVNQLTPRERDILKLIAQGLPNKMIARRLDITESTVKVHVKHMLKKMKLKSRVEAAVWVHQERIF

>1JEY:A

MSGWESYYKTEGDEEAEEEQEENLEASGDYKYSGRDSLIFLVDASKAMFESQSEDELTPFDMSIQCIQSVYISKIISSDRDLLAVVFYGTEKDKNSVNFKNIYVLQELDNPGAKRILELDQFKGQQGQKRFQDMMGHGSDYSLSEVLWVCANLFSDVQFKMSHKRIMLFTNEDNPHGNDSAKASRARTKAGDLRDTGIFLDLMHLKKPGGFDISLFYRDIISIAEDEDLRVHFEESSKLEDLLRKVRAKETRKRALSRLKLKLNKDIVISVGIYNLVQKALKPPPIKLYRETNEPVKTKTRTFNTSTGGLLLPSDTKRSQIYGSRQIILEKEETEELKRFDDPGLMLMGFKPLVLLKKHHYLRPSLFVYPEESLVIGSSTLFSALLIKCLEKEVAALCRYTPRRNIPPYFVALVPQEEELDDQKIQVTPPGFQLVFLPFADDKRKMPFTEKIMATPEQVGKMKAIVEKLRFTYRSDSFENPVLQQHFRNLEALALDLMEPEQAVDLTLPKVEAMNKRLGSLVDEFKELVYPPDYNPEGKVTKRKHDNEGSGSKRPKVEYSEEELKTHISKGTLGKFTVPMLKEACRAYGLKSGLKKQELLEALTKHFQD

>1JEY:B

MVRSGNKAAVVLCMDVGFTMSNSIPGIESPFEQAKKVITMFVQRQVFAENKDEIALVLFGTDGTDNPLSGGDQYQNITVHRHLMLPDFDLLEDIESKIQPGSQQADFLDALIVSMDVIQHETIGKKFEKRHIEIFTDLSSRFSKSQLDIIIHSLKKCDISLQFFLPFSLGKEDGSGDRGDGPFRLGGHGPSFPLKGITEQQKEGLEIVKMVMISLEGEDGLDEIYSFSESLRKLCVFKKIERHSIHWPCRLTIGSNLSIRIAAYKSILQERVKKTWTVVDAKTLKKEDIQKETVYCLNDDDETEVLKEDIIQGFRYGSDIVPFSKVDEEQMKYKSEGKCFSVLGFCKSSQVQRRFFMGNQVLKVFAARDDEAAAVALSSLIHALDDLDMVAIVRYAYDKRANPQVGVAFPHIKHNYECLVYVQLPFMEDLRQYMFSSLKNSKKYAPTEAQLNAVDALIDSMSLAKKDEKTDTLEDLFPTTKIPNPRFQRLFQCLLHRALHPREPLPPIQQHIWNMLNPPAEVTTKSQIPLSKIKTLFPLIEAKKKDQVTAQEIFQDNHEDGPTAK

>1JFI:A

MPSKKKKYNARFPPARIKKIMQTDEEIGKVAAAVPVIISRALELFLESLLKKACQVTQSRNAKTMTTSHLKQCIELEGDPAANKARKEAELAAATAEQ

>1JFI:B

GPHMASSSGNDDDLTIPRAAINKMIKETLPNVRVANDARELVVNCCTEFIHLISSEANEICNKSEKKTISPEHVIQALESLGFGSYISEVKEVLQECKTVALKRRKASSRLENLGIPEEELLRQQQELFAKARQQQAELAQQEWLQMQQAAQQAQLAAASASASNQAGSSQDEEDDDDI

>1JJ4:A

GSHMTPIIHLKGDRNSLKCLRYRLRKHSDHYRDISSTWHWTGAGNEKTGILTVTYHSETQRTKFLNTVAIPDSVQILVGYMTM

>1JT0:A

MNLKDKILGVAKELFIKNGYNATTTGEIVKLSESSKGNLYYHFKTKENLFLEILNIEESKWQEQWKKEQIKAKTNREKFYLYNELSLTTEYYYPLQNAIIEFYTEYYKTNSINEKMNKLENKYIDAYHVIFKEGNLNGEWSINDVNAVSKIAANAVNGIVTFTHEQNINERIKLMNKFSQIFLNGLSKHHHHHH

>1K79:D

GSGPIQLWQFLLELLTDKSCQSFISWTGDGWEFKLSDPDEVARRWGKRKNKPKMNYEKLSRGLRYYYDKNIIHKTAGKRYVYRFVCDLQSLLGYTPEELHAMLDVKPDAD

>1K82:A

PELPEVETSRRGIEPHLVGATILHAVVRNGRLRWPVSEEIYRLSDQPVLSVQRRAKYLLLELPEGWIIIHLGMSGSLRILPEELPPEKHDHVDLVMSNGKVLRYTDPRRFGAWLWTKELEGHNVLTHLGPEPLSDDFNGEYLHQKCAKKKTAIKPWLMDNKLVVGVGNIYASESLFAAGIHPDRLASSLSLAECELLARVIKAVLLRSIEQGGTTLKDFLQSDGKPGYFAQELQVYGRKGEPCRVCGTPIVATKHAQRATFYCRQCQK

>1KU7:A

SEELEKALSKLSEREAMVLKMRKGLIDGREHTLEEVGAYFGVTRERIRQIENKALRKLKYHESRTRKLRDFLE

>1KX5:A

ARTKQTARKSTGGKAPRKQLATKAARKSAPATGGVKKPHRYRPGTVALREIRRYQKSTELLIRKLPFQRLVREIAQDFKTDLRFQSSAVMALQEASEAYLVALFEDTNLCAIHAKRVTIMPKDIQLARRIRGERA

>1KX5:B

SGRGKGGKGLGKGGAKRHRKVLRDNIQGITKPAIRRLARRGGVKRISGLIYEETRGVLKVFLENVIRDAVTYTEHAKRKTVTAMDVVYALKRQGRTLYGFGG

>1KX5:C

SGRGKQGGKTRAKAKTRSSRAGLQFPVGRVHRLLRKGNYAERVGAGAPVYLAAVLEYLTAEILELAGNAARDNKKTRIIPRHLQLAVRNDEELNKLLGRVTIAQGGVLPNIQSVLLPKKTESSKSKSK

>1KX5:D

PEPAKSAPAPKKGSKKAVTKTQKKDGKKRRKTRKESYAIYVYKVLKQVHPDTGISSKAMSIMNSFVNDVFERIAGEASRLAHYNKRSTITSREIQTAVRLLLPGELAKHAVSEGTKAVTKYTSAK

>1L3L:A

MQHWLDKLTDLAAIEGDECILKTGLADIADHFGFTGYAYLHIQHRHITAVTNYHRQWQSTYFDKKFEALDPVVKRARSRKHIFTWSGEHERPTLSKDERAFYDHASDFGIRSGITIPIKTANGFMSMFTMASDKPVIDLDREIDAVAAAATIGQIHARISFLRTTPTAEDAAWLDPKEATYLRWIAVGKTMEEIADVEGVKYNSVRVKLREAMKRFDVRSKAHLTALAIRRKLI

>1LB2:B

KPEFDPILLRPVDDLELTVRSANCLKAEAIHYIGDLVQRTEVELLKTPNLGKKSLTEIKDVLASRGLSLGMRLENWPPASIADE

>1LPQ:A

KWKWWEEERYPEGIKWKFLEHKGPVFAPPYEPLPENVKFYYDGKVMKLSPKAEEVATFFAKMLDHEYTTKEIFRKNFFKDWRKEMTNEEKNIITNLSKCDFTQMSQYFKAQTEARKQMSKEEKLKIKEENEKLLKEYGFCIMDNHKERIANFKIEPPGLFRGRGNHPKMGMLKRRIMPEDIIINCSKDAKVPSPPPGHKWKEVRHDNKVTWLVSWTENIQGSIKYIMLNPSSRIKGEKDWQKYETARRLKKCVDKIRNQYREDWKSKEMKVRQRAVALYFIDKLALRAGNEKEEGETADTVGCCSLRVEHINLHPELDGQEYVVEFDFLGKDSIRYYNKVPVEKRVFKNLQLFMENKQPEDDLFDRLNTGILNKHLQDLMEGLTAKVFRTYNASITLQQQLKELTAPDENIPAKILSYNRANRAVAILCNHQRAPPKTFEKSMMNLQTKIDAKKEQLADARRDLKSAKADAKVMKDAKTKKVVESKKKAVQRLEEQLMKLEVQATDREENKQIALGTSKLNFLDPRITVAWCKKWGVPIEKIYNKTQREKFAWAIDMADEDYEF

>1LQ1:A

PKKKNLDASITSIIHEIGVPAHIKGYLYLREAISMVYNDIELLGSITKVLYPDIAKKFNTTASRVERAIRHAIEVAWSRGNIDSISSLFGYTVSMTKAKPTNSEFIAMVADKLRLEHKAS

>1LRR:A

VASPAIVEAKPVKTIKDKVRAMRELLLSDEYAEQKRAVNRFMLLLSTLYSLDAQAFAEATESLHGRTRVYFAADEQTLLKNGNQTKPKHVPGTPYWVITNTNTGRKCSMIEHIMQSMQFPAELIEKVCGTI

>1LV5:A

AKMAFTLADRVTEEMLADKAALVVEVVEENYHAAPIVGIAVVNEHGRFFLRPETALADPQFVAWLGDETKKKSMFDSKRAAVALKWKGIELCGVSFDLLLAAYLLDPAQGVDDVAAAAKMKQYEAVRPDEAVYGKGAKRAVPDEPVLAEHLVRKAAAIWELERPFLDELRRNEQDRLLVELEQPLSSILAEMEFAGVKVDTKRLEQMGKELAEQLGTVEQRIYELAGQEFNINSPKQLGVILFEKLQLPVLKKTKTGYSTSADVLEKLAPYHEIVENILHYRQLGKLQSTYIEGLLKVVRPDTKKVHTIFNQALTQTGRLSSTEPNLQNIPIRLEEGRKIRQAFVPSESDWLIFAADYSQIELRVLAHIAEDDNLMEAFRRDLDIHTKTAMDIFQVSEDEVTPNMRRQAKAVNFGIVYGISDYGLAQNLNISRKEAAEFIERYFESFPGVKRYMENIVQEAKQKGYVTTLLHRRRYLPDITSRNFNVRSFAERMAMNTPIQGSAADIIKKAMIDLNARLKEERLQAHLLLQVHDELILEAPKEEMERLCRLVPEVMEQAVTLRVPLKVDYHYGSTWYDAK

>1M5R:A

MKIAIINMGNNVINFKTVPSSETIYLFKVISEMGLNVDIISLKNGVYTKSFDEVDVNDYDRLIVVNSSINFFGGKPNLAILSAQKFMAKYKSKIYYLFTDIRLPFSQSWPNVKNRPWAYLYTEEELLIKSPIKVISQGINLDIAKAAHKKVDNVIEFEYFPIEQYKIHMNDFQLSKPTKKTLDVIYGGSFRSGQRESKMVEFLFDTGLNIEFFGNAREKQFKNPKYPWTKAPVFTGKIPMNMVSEKNSQAIAALIIGDKNYNDNFITLRVWETMASDAVMLIDEEFDTKHRIINDARFYVNNRAELIDRVNELKHSDVLRKEMLSIQHDILNKTRAKKAEWQDAFKKAIDL

>1MM8:A

MVTFMITSALHRAADWAKSVFSSAALGDPRRTARLVNVAAQLAKYSGKSITISSEGSKAAQEGAYRFIRNPNVSAEAIRKAGAMQTVKLAQEFPELLAIEDTTSLSYRHQVAEELGKLGSIQDKSRGWWVHSVLLLEATTFRTVGLLHQEWWMRPDDPADADEKESGKWLAAAATSRLRMGSMMSNVIAVCDREADIHAYLQDKLAHNERFVVRSKHPRKDVESGLYLYDHLKNQPELGGYQISIPQKGVVDKRGKRKNRPARKASLSLRSGRITLKQGNITLNAVLAEEINPPKGETPLKWLLLTSEPVESLAQALRVIDIYTHRWRIEEFHKAWKTGAGAERQRMEKPDNLERMVSILSFVAVRLLQLRESFTPPQALRAQGLLKEAEHVESQSAETVLTPDECQLLGYLDKGKRKRKEKAGSLQWAYMAIARLGGFMDSKRTGIASWGALWEGWEALQSKLDGFLAAKDLMAQGIKIG

>1MNN:A

MNEMENTVPVLQDDLVSKYERELSTEQEEDTPVILTQLNEDGTTSNYFDKRKLKIAPRSTLQFKVGPPFELVRDYCPVVESHTGRTLDLRIIPRIDRGFDHIDEEWVGYKRNYFTLVSTFETANCDLDTFLKSSFDLLVEDSSVEGRLRVQYFAIKIKAKNDDDDTEINLVQHTAKRDKGPQFCPSVCPLVPSPLPKHQTIREASNVRNITKMKKYDSTFYLHRDHVNYEEYGVDSLLFSYPEDSIQKVARYERVQFASSISVKKPSQQNKHFSLHVILGAVVDPDTFHGENPGIPYDELALKNGSKGMFVYLQEMKTPPLIIRGRSPSNYASSQRITVR

>1N6J:A

GRKKIQISRILDQRNRQVTFTKRKFGLMKKAYELSVLCDCEIALIIFNSANRLFQYASTDMDRVLLKYTEYSEPHESRTNTDILETLKRRGIG

>1NLW:A

SRSTHNEMEKNRRAHLRLSLEKLKGLVPLGPDSSRHTTLSLLTKAKLHIKKLEDSDRKAVHQIDQLQREQRHLKRQLEKL

>1NVP:C

GSGAEDGQVEEEPLNSEDDVSDEEGQELFDTENVVVCQYDKIHRSKNKWKFHLKDGIMNLNGRDYIFSKAIGDAEW

>1ODH:A

MELDDFDPEDKEILSWDINDVKLPQNVKTTDWFQEWPDSYVKHIYSSDDRNAQRHLSSWAMRNTNNHNSRILKKSCLGVVVCSRDCSTEEGRKIYLRPAICDKARQKQQRKSCPNCNGPLKLIPCRGHGGFPVTNFWRHDGRFIFFQSKGEHDHPRPETKLEAEARRAMKKVHM

>1P7D:A

MTLHSWLDRYEKILASRGIKQKTLINYMSKIKAIRRGLPDAPLEDITTKEIAAMLNGYIDEGKAASAKLIRSTLSDAFREAIAEGHITTNHVAATRAAKSEVRRSRLTADEYLKIYQAAESSPCWLRLAMELAVVTGQRVGDLCEMKWSDIVDGYLYVEQSKTGVKIAIPTALHIDALGISMKETLDKCKEILGGETIIASTRREPLSSGTVSRYFMRARKASGLSFEGDPPTFHELRSLSARLYEKQISDKFAQHLLGHKSDTMASQYRDDRGREWDKIEIK

>1PP8:F

MDSNDLEASFTSRLPPEIVAALKRKSSRDPNSRFPRKLHMLLTYLASNPQLEEEIGLSWISDTEFKMKKKNVALVMGIKLNTLNVNLRDLAFEQLQHDKGGWTQWKRSGFTRNSVFEDPTQNDSPMHHHHHH

>1PT3:A

KPGKATGKGKPVNNKWLNNAGKDLGSPVPDRIANKLRDKEFKSFDDFRKKFWEEVSKDPELSKQFSRNNNDRMKVGKAPKTRTQDVSGKRTSFELHHEKPISQNGGVYDMDNISVVTPKRHIDIHRGK

>1PUF:B

ARRKRRNFNKQATEILNEYFYSHLSNPYPSEEAKEELAKKCGITVSQVSNWFGNKRIRYKKNIGKFQEEANIY

>1PVR:A

MHHHHHHSNLLTVHQNLPALPVDATSDEVRKNLMDMFRDRQAFSEHTWKMLLSVCRSWAAWCKLNNRKWFPAEPEDVRDYLLYLQARGLAVKTIQQHLGQLNMLHRRSGLPRPSDSNAVSLVMRRIRKENVDAGERAKQALAFERTDFDQVRSLMENSDRCQDIRNLAFLGIAYNTLLRLAEIARIRVKDISRTDGGRMLIHIGRTKTLVSTAGVEKALSLGVTKLVERWISVSGVADDPNNYLFCRVRKNGVAAPSATSQLSNSALGGIFGATHRLIYGAKDDSGQRYLAWSGHSARVGAARDMARAGVSIPEIMQAGGWTNVNIVMNYIRNLDSETGAMVRLLEDGD

>1Q9X:A

MKEFYLTVEQIGDSIFERYIDSNGRERTREVEYKPSLFAHCPESQATKYFDIYGKPCTRKLFANMRDASQWIKRMEDIGLEALGMDDFKLAYLSDTYNYEIKYDHTKIRVANFDIEVTSPDGFPEPSQAKHPIDAITHYDSIDDRFYVFDLLNSPYGNVEEWSIEIAAKLQEQGGDEVPSEIIDKIIYMPFDNEKELLMEYLNFWQQKTPVILTGWNVESFAIPYVYNRIKNIFGESTAKRLSPHRKTRVKVIENMYGSREIITLFGISVLDYIDLYKKFSFTNQPSYSLDYISEFELNVGKLKYDGPISKLRESNHQRYISYNIIAVYRVLQIDAKRQFINLSLDMGYYAKIQIQSVFSPIKTWDAIIFNSLKEQNKVIPQGRSHPVQPYPGAFVKEPIPNRYKYVMSFDLTSLYPSIIRQVNISPETIAGTFKVAPLHDYINAVAERPSDVYSCSPNGMMYYKDRDGVVPTEITKVFNQRKEHKGYMLAAQRNGEIIKEALHNPNLSVDEPLDVDYRFDFSDEIKEKIKKLSAKSLNEMLFRAQRTEVAGMTAQINRKLLINSLYGALGNVWFRYYDLRNATAITTFGQMALQWIERKVNEYLNEVCGTEGEAFVLYGDTDSIYVSADKIIDKVGESKFRDTNHWVDFLDKFARERMEPAIDRGFREMCEYMNNKQHLMFMDREAIAGPPLGSKGIGGFWTGKKRYALNVWDMEGTRYAEPKLKIMGLETQKSSTPKAVQKALKECIRRMLQEGEESLQEYFKEFEKEFRQLNYISIASVSSANNIAKYDVGGFPGPKCPFHIRGILTYNRAIKGNIDAPQVVEGEKVYVLPLREGNPFGDKCIAWPSGTEITDLIKDDVLHWMDYTVLLEKTFIKPLEGFTSAAKLDYEKKASLFDMFDF

>1QSS:A

ALEEAPWPPPEGAFVGFVLSRKEPMWADLLALAAARGGRVHRAPEPYKALRDLKEERGLLAKDLSVLALREGLGLPPGDDPMLLAYLLDPSNTTPEGVARRYGGEWTEEAGERAALSERLFANLWGRLEGEERLLWLYREVERPLSAVLAHMEATGVRLDVAYLRALSLEVAEEIARLEAEVFRLAGHPFNLNSRDQLERVLFDELGLPAIGKTEKTGKRSTSAAVLEALREAHPIVEKILQYRELTKLKSTYIDPLPDLIHPRTGRLHTRFNQTATATGRLSSSDPNLQNIPVRTPLGQRIRRAFIAEEGWLLVALDYSQIELRVLAHLSGDENLIRVFQEGRDIHTETASWMFGVPREAVDPLMRRAAKTINFGVLYGMSAHRLSQELAIPYEEAQAFIERYFQSFPKVRAWIEKTLEEGRRRGYVETLFGRRRYVPDLEARVKSVREAAERMAFNMPVQGTAADLMKLAMVKLFPRLEEMGARMLLQVHDELVLEAPKERAEAVARLAKEVMEGVYPLAVPLEVEVGIGEDWLSAK

>1R71:A

RYRGSKWAGKKSIPAFIDNDYNEADQVIENLQRNELTPREIADFIGRELAKGKKKGDIAKEIGKSPAFITQHVTLLDLPEKIADAFNTGRVRDVTVVNELVTAFKKRPEEVEAWLDDDTQEITRGTVKLLREFLDEKGRDPNTVDAFNGQTDAERDAEAGDGQDGEDGDQDGKDAKEK

>1R7M:A

MKNIKKNQVMNLGPNSKLLKEYKSQLIELNIEQFEAGIGLILGDAYIRSRDEGKTYCMQFEWKNKAYMDHVCLLYDQWVLSPPHKKERVNHLGNLVITWGAQTFKHQAFNKLANLFIVNNKKTIPNNLVENYLTPMSLAYWFMDDGGKWDYNKNSTNKSIVLNTQSFTFEEVEYLVKGLRNKFQLNCYVKINKNKPIIYIDSMSYLIFYNLIKPYLIPQMMYKLPNTISSETFLK

>1R8D:A

MKYQVKQVAEISGVSIRTLHHYDNIELLNPSALTDAGYRLYSDADLERLQQILFFKEIGFRLDEIKEMLDHPNFDRKAALQSQKEILMKKKQRMDEMIQTIDRTLLSVD

>1S9F:A

MIVLFVDFDYFYAQVEEVLNPSLKGKPVVVCVFSGRFEDSGAVATANYEARKFGVKAGIPIVEAKKILPNAVYLPMRKEVYQQVSSRIMNLLREYSEKIEIASIDEAYLDISDKVRDYREAYNLGLEIKNKILEKEKITVTVGISKNKVFAKIAADMAKPNGIKVIDDEEVKRLIRELDIADVPGIGNITAEKLKKLGINKLVDTLSIEFDKLKGMIGEAKAKYLISLARDEYNEPIRTRVRKSIGRIVTMKRNSRNLEEIKPYLFRAIEESYYKLDKRIPKAIHVVAVTEDLDIVSRGRTFPHGISKETAYSESVKLLQKILEEDERKIRRIGVRFSKFIEAIGLDKFFDT

>1SFU:A

MDLLSCTVNDAEIFSLVKKEVLSLNTNDYTTAISLSNRLKINKKKINQQLYKLQKEDTVKMVPSNPPKWFKNYNC

>1SKN:P

MGHHHHHHSGQRKRGRQSKDEQLASDNELPVSAFQISEMSLSELQQVLKNESLSEYQRQLIRKIRRRGKNKVAARTCRQRRTDRHDKMSHYI

>1T2K:A

MGTPKPRILPWLVSQLDLGQLEGVAWVNKSRTRFRIPWKHGLRQDAQQEDFGIFQAWAEATGAYVPGRDKPDLPTWKRNFRSALNRKEGLRLAEDRSKDPHDPHKIYEFVNS

>1T2K:D

KRRKFLERNRAAASRSRQKRKVWVQSLEKKAEDLSSLNGQLQSEVTLLRNEVAQLKQLLLA

>1T3N:A

SRVIVHVDLDCFYAQVEMISNPELKDKPLGVQQKYLVVTCNYEARKLGVKKLMNVRDAKEKCPQLVLVNGEDLTRYREMSYKVTELLEEFSPVVERLGFDENFVDLTEMVEKRLQQLQSDELSAVTVSGHVYNNQSINLLDVLHIRLLVGSQIAAEMREAMYNQLGLTGCAGVASNKLLAKLVSGVFKPNQQTVLLPESCQHLIHSLNHIKEIPGIGYKTAKCLEALGINSVRDLQTFSPKILEKELGISVAQRIQKLSFGEDNSPVILSGPPQSFSEEDSFKKCSSEVEAKNKIEELLASLLNRVCQDGRKPHTVRLIIRRYSSEKHYGRESRQCPIPSHVIQKLGTGNYDVMTPMVDILMKLFRNMVNVKMPFHLTLLSVCFCNLK

>1T8E:A

MIVSDIEANALLESVTKFHCGVIYDYSTAEYVSYRPSDFGAYLDALEAEVARGGLIVFHNGHKYDVPALTKLAKLQLNREFHLPRENCIDTLVLSRLIHSNLKDTDMGLLRSGKLPGALEAWGYRLGEMKGEYKDDFKRMLEEQGEEYVDGMEWWNFNEEMMDYNVQDVVVTKALLEKLLSDKHYFPPEIDFTDVGYTTFWSESLEAVDIEHRAAWLLAKQERNGFPFDTKAIEELYVELAARRSELLRKLTETFGSWYQPKGGTEMFCHPRTGKPLPKYPRIKTPKVGGIFKKPKNKAQREGREPCELDTREYVAGAPYTPVEHVVFNPSSRDHIQKKLQEAGWVPTKYTDKGAPVVDDEVLEGVRVDDPEKQAAIDLIKEYLMIQKRIGQSAEGDKAWLRYVAEDGKIHGSVNPNGAVTGRATHAFPNLAQIPGVRSPYGEQCRAAFGAEHHLDGITGKPWVQAGIDASGLELRCLAHFMARFDNGEYAHEILNGDIHTKNQIAAELPTRDNAKTFIYGFLYGAGDEKIGQIVGAGKERGKELKKKFLENTPAIAALRESIQQTLVESSQWVAGEQQVKWKRRWIKGLDGRKVHVRSPHAALNTLLQSAGALICKLWIIKTEEMLVEKGLKHGWDGDFAYMAWVHDEIQVGCRTEEIAQVVIETAQEAMRWVGDHWNFRCLLDTEGKMGPNWAICH

>1T8E:B

SDKIIHLTDDSFDTDVLKADGAILVDFWAEWCGPCKMIAPILDEIADEYQGKLTVAKLNIDQNPGTAPKYGIRGIPTLLLFKNGEVAATKVGALSKGQLKEFLDANLA

>1T9I:A

MNTKYNKEFLLYLAGFVDGNGSIIAQIKPNQSYKFKHQLSLTFQVTQKTQRRWFLDKLVDEIGVGYVRDRGSVSDYILSEIKPLHNFLTQLQPFLKLKQKQANLVLKIIEQLPSAKESPDKFLEVCTWVDQIAALNDSKTRKTTSETVRAVLDSLSEKKKSSP

>1TTU:A

GPLGSGDSVQSLTSDRMIDFLSNKEKYECVISIFHAKVAQKSYGNEKRFFCPPPCIYLIGQGWKLKKDRVAQLYKTLKASAQKDAAIENDPIHEQQATELVAYIGIGSDTSERQQLDFSTGKVRHPGDQRQDPNIYDYCAAKTLYISDSDKRKYFDLNAQFFYGCGMEIGGFVSQRIKVISKPSKKKQSMKNTDCKYLCIASGTKVALFNRLRSQTVSTRYLHVEGNAFHASSTKWGAFTIHLFDDERGLQETDNFAVRDGFVYYGSVVKLVDSVTGIALPRLRIRKVDKQQVILDASCSEEPVSQLHKCAFQMIDNELVYLCLSHDKIIQHQATAINEHRHQINDGAAWTIISTDKAEYRFFEAMGQVANPISPCPVVGSLEVDGHGEASRVELHGRDFKPNLKVWFGATPVETTFRSEESLHCSIPPVSQVRNEQTHWMFTNRTTGDVEVPISLVRDDGVVYSSGLTFSYKSLER

>1TX3:A

SFIKPIYQDINSILIGQKVKRPKSGTLSGHAAGEPFEKLVYKFLKENLSDLTFKQYEYLNDLFMKNPAIIGHEARYKLFNSPTLLFLLSRGKAATENWSIENLFEEKQNDTADILLVKDQFYELLDVKTRNISKSAQAPNIISAYKLAQTCAKMIDNKEFDLFDINYLEVDWELNGEDLVCVSTSFAELFKSEPSELYINWAAAMQIQFHVRDLDQGFNGTREEWAKSYLKHFVTQAEQRAISMIDKFVKPFKKYIL

>1U3E:M

MEWKDIKGYEGHYQVSNTGEVYSIKSGKTLKHQIPKDGYHRIGLFKGGKGKTFQVHRLVAIHFCEGYEEGLVVDHKDGNKDNNLSTNLRWVTQKINVENQMSRGTLNVSKAQQIAKIKNQKPIIVISPDGIEKEYPSTKCACEELGLTRGKVTDVLKGHRIHHKGYTFRYKLNG

>1VAS:A

TRINLTLVSELADQHLMAEYRQLPRVFGAVRKHVANGKRVRDFKISPTFILGAGHVTFFYDKLEFLRKRQIELIAECLKRGFNIKDTTVQDISDIPQEFRGDYIPHEASIAISQARLDEKIAQRPTWYKYYGKAIYA

>1X9N:A

MTPRKPAVKKEVKEEEPGAPGKEGAAEGPLDPSGYNPAKNNYHPVEDACWKPGQKVPYLAVARTFEKIEEVSARLRMVETLSNLLRSVVALSPPDLLPVLYLSLNHLGPPQQGLELGVGDGVLLKAVAQATGRQLESVRAEAAEKGDVGLVAENSRSTQRLMLPPPPLTASGVFSKFRDIARLTGSASTAKKIDIIKGLFVACRHSEARFIARSLSGRLRLGLAEQSVLAALSQAVSLTPPGQEFPPAMVDAGKGKTAEARKTWLEEQGMILKQTFCEVPDLDRIIPVLLEHGLERLPEHCKLSPGIPLKPMLAHPTRGISEVLKRFEEAAFTCEYKYDGQRAQIHALEGGEVKIFSRNQEDNTGKYPDIISRIPKIKLPSVTSFILDTEAVAWDREKKQIQPFQVLTTRKRKEVDASEIQVQVCLYAFDLIYLNGESLVREPLSRRRQLLRENFVETEGEFVFATSLDTKDIEQIAEFLEQSVKDSCEGLMVKTLDVDATYEIAKRSHNWLKLKKDYLDGVGDTLDLVVIGAYLGRGKRAGRYGGFLLASYDEDSEELQAICKLGTGFSDEELEEHHQSLKALVLPSPRPYVRIDGAVIPDHWLDPSAVWEVKCADLSLSPIYPAARGLVDSDKGISLRFPRFIRVREDKQPEQATTSAQVACLYRKQSQIQNQQGEDSGSDPEDTY

>1XPX:A

TPLHSSTLTPMHLRKAKLMFFWVRYPSSAVLKMYFPDIKFNKNNTAQLVKWFSNFREFYYIQMEKYARQAVTEGIKTPDDLLIAGDSELYRVLNLHYNRNNHIEVPQNFRFVVESTLREFFRAIQGGKDTEQSWKKSIYKIISRMDDPVPEYFKSPNFLEQLE

>1YFJ:A

MLGAIAYTGNKQSLLPELKSHFPKYNRFVDLFCGGLSVSLNVNGPVLANDIQEPIIEMYKRLINVSWDDVLKVIKQYKLSKTSKEEFLKLREDYNKTRDPLLLYVLHFHGFSNMIRINDKGNFTTPFGKRTINKNSEKRFNHFKQNCDKIIFSSLHFKDVKILDGDFVYVDPPYLITVADYNKFWSEDEEKDLLNLLDSLNDRGIKFGLSNVLEHHGKENTLLKEWSKKYNVKHLNKKYVFNIYHSKEKNGTDEVYIFN

>1Z63:A

MGSSHHHHHHSSGLVPRGSHMASKSFQLLEPYNIKANLRPYQIKGFSWMRFMNKLGFGICLADDMGLGKTLQTIAVFSDAKKENELTPSLVICPLSVLKNWEEELSKFAPHLRFAVFHEDRSKIKLEDYDIILTTYAVLLRDTRLKEVEWKYIVIDEAQNIKNPQTKIFKAVKELKSKYRIALTGTPIENKVDDLWSIMTFLNPGLLGSYSEFKSKFATPIKKGDNMAKEELKAIISPFILRRTKYDKAIINDLPDKIETNVYCNLTPEQAAMYKAEVENLFNNIDSVTGIKRKGMILSTLLKLKQIVDHPALLKGGEQSVRRSGKMIRTMEIIEEALDEGDKIAIFTQFVDMGKIIRNIIEKELNTEVPFLYGELSKKERDDIISKFQNNPSVKFIVLSVKAGGFGINLTSANRVIHFDRWWNPAVEDQATDRVYRIGQTRNVIVHKLISVGTLEEKIDQLLAFKRSLFKDIISSGDSWITELSTEELRKVIELSVGGY

>3ORC:A

EQRITLKDYAMRFGQTKTAKDLGVYQSAINKAIHAGRKIFLTINADGSVYAEEVKDGEVKPFPSN

(2). non DNA-binding proteins

>P77610

MSKHDTDTSDQHAAKRRWLNAHEEGYHKAMGNRQVQMIAIGGAIGTGLFLGAGARLQMAGPALALVYLICGLFSFFILRALGELVLHRPSSGSFVSYAREFLGEKAAYVAGWMYFINWAMTGIVDITAVALYMHYWGAFGGVPQWVFALAALTIVGTMNMIGVKWFAEMEFWFALIKVLAIVTFLVVGTVFLGSGQPLDGNTTGFHLITDNGGFFPHGLLPALVLIQGVVFAFASIEMVGTAAGECKDPQTMVPKAINSVIWRIGLFYVGSVVLLVMLLPWSAYQAGQSPFVTFFSKLGVPYIGSIMNIVVLTAALSSLNSGLYCTGRILRSMAMGGSAPSFMAKMSRQHVPYAGILATLVVYVVGVFLNYLVPSRVFEIVLNFASLGIIASWAFIIVCQMRLRKAIKEGKAADVSFKLPGAPFTSWLTLLFLLSVLVLMAFDYPNGTYTIAALPIIGILLVIGWFGVRKRVAEIHSTAPVVEEDEEKQEIVFKPETAS

>Q9USX1

MQVTIPKSTSKEDDKNRNLLPKNVKPIHYDLSLYPDLETFTYGGKVVVTLDVLEDSNSITLHGINLRILTAALEWGSQTVWASEVSYGDERIVLQFPSTVPANSVAVLTLPFTARISSGMEGFYRSSYVDSDGNTKYLATTQMEPTSARRAFPCWDEPALKATFTIDITAKENYTILSNMNAVEETVKDGLKTARFAETCRMSTYLLAWIVAELEYVEYFTPGKHCPRLPVRVYTTPGFSEQGKFAAELGAKTLDFFSGVFGEPYPLPKCDMVAIPDFEAGAMENWGLVTYRLAAILVSEDSAATVIERVAEVVQHELAHQWFGNLVTMQFWDGLWLNEGFATWMSWFSCNHFYPEWKVWESYVTDNLQSALSLDALRSSHPIEVPIMHDYEINQIFDAISYSKGSCVIRMVSKYVGEDTFIKGIQKYISKHRYGNTVTEDLWAALSAESGQDISSTMHNWTKKTGYPVLSVSETNDGELLIEQHRFLSTGDVKPEEDTVIYWAPLKLKTMKDGKAVVDEKAVLSDRSKKIKVDKEALESYKLNSEQSGIYRVNYSADHLKKLSQIAVEKPDYLSVEDRAGLIADVASLSRAGYGKVSSTLDLIKTWKDEPNFVVFAEMLARLNGIKSTLRFESSDIIAAMKKLVLEVSATKAHSLGWEFKANDDHIIRQFKSTVYNYAGLFGDDKVVKDALSKFDAYASGNKSAINDNLRSAVFNIAIRYGGAKSWDQLLEIYTKTNDPYVRNSCLRAFGVTEDEKYIQKTLDLTLDPIVKEQDIYLILVTLSTHKNGVLAMWKFATSNWDKLLSRLPVAGTMRGYVVRFVTSGFTHASAIDKIKEFFADKDTKLYERALQQSLDTISANSSFIDKSLDDITRWLKENRYM

>Q9HL27

MMKSIILIVLDGLGDRPGSDLQNRTPLQAAFRPNLNWLASHGINGIMHPISPGIRCGSDTSHMSLLGYDPKVYYPGRGPFEALGLGMDIRPGDLAFRANFATNRDGVIVDRRAGRENKGNEELADAISLDMGEYSFRVKSGVEHRAALVVSGPDLSDMIGDSDPHREGLPPEKIRPTDPSGDRTAEVMNAYLEEARRILSDHRVNKERVKNGRLPGNELLVRSAGKVPAIPSFTEKNRMKGACVVGSPWLKGLCRLLRMDVFDVPGATGTVGSNYRGKIEKAVDLTSSHDFVLVNIKATDVAGHDGNYPLKRDVIEDIDRAMEPLKSIGDHAVICVTGDHSTPCSFKDHSGDPVPIVFYTDGVMNDGVHLFDELSSASGSLRITSYNVMDILMQLAGRSDKFGS

>P17690

MPPPALVLLLGFLCHVAIAGRTCPKPDELPFSTVVPLKRTYEPGEQIVFSCQPGYVSRGGIRRFTCPLTGLWPINTLKCMPRVCPFAGILENGTVRYTTFEYPNTISFSCHTGFYLKGASSAKCTEEGKWSPDLPVCAPITCPPPPIPKFASLSVYKPLAGNNSFYGSKAVFKCLPHHAMFGNDTVTCTEHGNWTQLPECREVRCPFPSRPDNGFVNHPANPVLYYKDTATFGCHETYSLDGPEEVECSKFGNWSAQPSCKASCKLSIKRATVIYEGERVAIQNKFKNGMLHGQKVSFFCKHKEKKCSYTEDAQCIDGTIEIPKCFKEHSSLAFWKTDASDVKPC

>P60087

MTKTKAIDIIGAPSTFGQRKLGVDLGPTAIRYAGLISRLKQLDLDVYDKGDIKVPAVNIEKFHSEQKGLRNYDEIIDVNQKLNKEVSASIENNRFPLVLGGDHSIAVGSVSAISKHYNNLGVIWYDAHGDLNIPEESPSGNIHGMPLRILTGEGPKELLELNSNVIKPENIVLIGMRDLDKGERQFIKDHNIKTFTMSDIDKLGIKEVIENTIEYLKSRNVDGVHLSLDVDALDPLETPGTGTRVLGGLSYRESHFALELLHQSHLISSMDLVEVNPLIDSNNHTAEQAVSLVGTFFGETLL

>P57684

MASPPPIVEYAVFFTILAVLVFVAGEYLAWVYREQANSDHRPPGYLSWFERLDEIFTPIENGLYRLSGINPRREMTWKGYLKAVLVFNVCIWVLLFVVLMFQDALPMNFVGVGGESWDLAFHTASSFTSNTNQQHYSGETLSVFTHTFGIGIAMFLTPATGLALMPAFARAFTNKEDPRLGNFYENVVRGLVRFLLPISLLIAIILMAEGSVQTILGGQLTANTFTMGIQNIRIGPHAGIEAIKMFGTNGGGINAANAATAFENPTPLSNLVLTLAMPIGTFSAIYAWGAWVGNRSHGVAIVAAFFVIYMALTGVAVVGETGTNAGMVVTGNGLHVDQTVGNMEGKETRFGPTASAIWGLSTTGTTNGGVNSMHNSWTALGAFSLLFAFATNNISNGVGTGLLNILMFVILTAFIGALMIGRRPQYLGKKLEWQEMRYVFVVILVLPILVLIPQAAAVVYQGAIDSMNNPGFRGFSEVLYEFFSASANNGSGFEGLGDGTLFFNLVNGVQVLLARYVPITAQLAIAGYLANKKVSPESKGSLDTDTPAFVGLLIGVIIIVSALVFLPALVFGPIGELLSGGI

>O24562

MGSLASERKVVGWAARDATGHLSPYSYTLRNTGPEDVVVKVLYCGICHTDIHQAKNHLGASKYPMVPGHEVVGEVVEVGPEVAKYGVGDVVGVGVIVGCCRECSPCKANVEQYCNKKIWSYNDVYTDGRPTQGGFASTMVVDQKFVVKIPAGLAPEQAAPLLCAGVTVYSPLKHFGLTNPGLRGGILGLGGVGHMGVKVAKAMGHHVTVISSSSKKRAEAMDHLGADAYLVSSDAAAMAAAADSLDYIIDTVPVHHPLEPYLALLKLDGKLVLLGVIGEPLSFVSPMVMLGRKAITGSFIGSIDETAEVLQFCVDKGLTSQIEVVKMGYVNEALERLERNDVRYRFVVDVAGSNVEAEAAAADAASN

>Q92051

MAHAWGYGPADGPESWAESFPIANGPRQSPIDIVPTQAQHDPSLKHLKLKYDPATTKSILNNGHSFQVDFVDDDNSSTLAGGPITGIYRLRQFHFHWGSSDDKGSEHTIAGTKFPCELHLVHWNTKYPNFGEAASKPDGLAVVGVFLKIGAANPRLQKVLDALDDIKSKGRQTTFANFDPKTLLPASLDYWTYEGSLTTPPLLESVTWIVLKEPISVSPAQMAKFRSLLFSSEGETPCCMVDNYRPPQPLKGRKVRASFK

>Q8Z9L5

SESLHLTRNGPILEITLDRPKANAIDAKTSFAMGEAFLNFRDDPELRVAIITGGGEKFFSAGWDLKAAAEGEAPDADFGPGGFAGLTEIFDLDKPVIAAVNGYAFGGGFELALAADFIVCAENASFALPEAKLGIVPDSGGVLRLPKLLPPAIVNEMVMTGRRMSAEEALRWGIVNRVVSQSELMESARELAQQLVNSAPLAIAALKEIYRATSEMPVEEGYRYIRSGVLKHYPSVLHSEDALEGPQAFAEKRAPVWKGR

>P00431

MTTAVRLLPSLGRTAHKRSLYLFSAAAAAAAAATFAYSQSQKRSSSSPGGGSNHGWNNWGKAAALASTTPLVHVASVEKGRSYEDFQKVYNAIALKLREDDEYDNYIGYGPVLVRLAWHTSGTWDKHDNTGGSYGGTYRFKKEFNDPSNAGLQNGFKFLEPIHKEFPWISSGDLFSLGGVTAVQEMQGPKIPWRCGRVDTPEDTTPDNGRLPDADKDADYVRTFFQRLNMNDREVVALMGAHALGKTHLKNSGYEGPWGAANNVFTNEFYLNLLNEDWKLEKNDANNEQWDSKSGYMMLPTDYSLIQDPKYLSIVKEYANDQDKFFKDFSKAFEKLLENGITFPKDAPSPFIFKTLEEQGL

>P46092

MGTEATEQVSWGHYSGDEEDAYSAEPLPELCYKADVQAFSRAFQPSVSLTVAALGLAGNGLVLATHLAARRAARSPTSAHLLQLALADLLLALTLPFAAAGALQGWSLGSATCRTISGLYSASFHAGFLFLACISADRYVAIARALPAGPRPSTPGRAHLVSVIVWLLSLLLALPALLFSQDGQREGQRRCRLIFPEGLTQTVKGASAVAQVALGFALPLGVMVACYALLGRTLLAARGPERRRALRVVVALVAAFVVLQLPYSLALLLDTADLLAARERSCPASKRKDVALLVTSGLALARCGLNPVLYAFLGLRFRQDLRRLLRGGSCPSGPQPRRGCPRRPRLSSCSAPTETHSLSWDN

>P51480

MESAADRLARAAAQGRVHDVRALLEAGVSPNAPNSFGRTPIQVMMMGNVHVAALLLNYGADSNCEDPTTFSRPVHDAAREGFLDTLVVLHGSGARLDVRDAWGRLPLDLAQERGHQDIVRYLRSAGCSLCSAGWSLCTAGNVAQTDGHSFSSSTPRALELRGQSQEQS

>Q9U8F1

MSMYTHQSNNELIPLKTVSTLNDLIKTIRIIFNQKEINVNEIHKILNDFQCDFTEWQKYIYFNKTHYTRNLIDEGNGRYNLFLLCWSEDQGTRIHDHSGAHCFVKLIKGCIKETIFEWPKYFTVEKSNYSINQIDLPLTVKSVSEMRPGDVTYMHDKIGIHRLHNPSTTETAITLHLYFPPYTNSMIFEESTSRMKKMDVTFHSKFGKQIPQ

>Q06850

MGNTCVGPSRNGFLQSVSAAMWRPRDGDDSASMSNGDIASEAVSGELRSRLSDEVQNKPPEQVTMPKPGTDVETKDREIRTESKPETLEEISLESKPETKQETKSETKPESKPDPPAKPKKPKHMKRVSSAGLRTESVLQRKTENFKEFYSLGRKLGQGQFGTTFLCVEKTTGKEFACKSIAKRKLLTDEDVEDVRREIQIMHHLAGHPNVISIKGAYEDVVAVHLVMECCAGGELFDRIIQRGHYTERKAAELTRTIVGVVEACHSLGVMHRDLKPENFLFVSKHEDSLLKTIDFGLSMFFKPDDVFTDVVGSPYYVAPEVLRKRYGPEADVWSAGVIVYILLSGVPPFWAETEQGIFEQVLHGDLDFSSDPWPSISESAKDLVRKMLVRDPKKRLTAHQVLCHPWVQVDGVAPDKPLDSAVLSRMKQFSAMNKFKKMALRVIAESLSEEEIAGLKEMFNMIDADKSGQITFEELKAGLKRVGANLKESEILDLMQAADVDNSGTIDYKEFIAATLHLNKIEREDHLFAAFTYFDKDGSGYITPDELQQACEEFGVEDVRIEELMRDVDQDNDGRIDYNEFVAMMQKGSITGGPVKMGLEKSFSIALKL

>Q99VC0

PKIVVVGAVAGGATCASQIRRLDKESDIIIFEKDRDMSFANCALPYVIGEVVEDRKYALAYTPEKFYDRKQITVKTYHEVIAINDERQTVTVLNRKTNEQFEESYDKLILSPGASANSLGFESDITFTLRNLEDTDAIDQFIKANQVDKVLVIGAGYVSLEVLENLYERGLHPTLIHRSDKINKLMDADMNQPILDELDKREIPYRLNEEIDAINGNEITFKSGKVEHYDMIIEGVGTHPNSKFIESSNIKLDRKGFIPVNDKFETNVPNIYAIGDIATSHYRHVDLPASVPLAWGAHRAASIVAEQIAGNDTIEFKGFLGNNIVKFFDYTFASVGVKPNELKQFDYKMVEVTQGAHANYYPGNSPLHLRVYYDTSNRQILRAAAVGKEGADKRIDVLSMAMMNQLTVDELTEFEVAYAPPYSHPKDLINMIGYKAK

>Q9ZDA8

RMITQKEKEHLAKDKQNIYLRIISGIALVSLFVIAILCLKTLFYILMILVGLGMLSEWYNMTYPSINYLLIGLIIIPIPISLLIFLSMEESNRLVIMLYFCILWSVDTFAMIGGKTFKGIKLAPKISPKKTWTGLITGTVSAGLVSVLVSLIPNYHIEHYYFSNKIYLFIISCILALIAQSSDLFISYFKRKFNIKDSGHIIPGHGGVLDRFDSIILTAPVFFCINIYL

>P12212

MKKKKALPSFLYLVFIVLLPWGVSFSFNKCLELWIKNWWNTRQSQTLLTAIQEKRVLERFMELEDLFILDEMIKEKPNTHVQNPPIGIRKEIIQLAKIDNEGHLHIILHFSTNIICLAILSGSFFLGKEELVILNSWVQEFFYNLNDSVKAFFILLVTDFFVGFHSTRGWELLIRWVYNDLGWVPNELIFTIFVCSFPVILDTCLKFWVFFCLNRLSPSLVVIYHSISEA

>Q8LPK5

MMESRSPICNTCGEEIGVKSNGEFFVACHECSFPICKACLEYEFKEGRRICLRCGNPYDENVFDDVETKTSKTQSIVPTQTNNTSQDSGIHARHISTVSTIDSELNDEYGNPIWKNRVESWKDKKDKKSKKKKKDPKATKAEQHEAQIPTQQHMEDTPPNTESGATDVLSVVIPIPRTKITSYRIVIIMRLIILALFFNYRITHPVDSAYGLWLTSVICEIWFAVSWVLDQFPKWSPINRETYIDRLSARFEREGEQSQLAAVDFFVSTVDPLKEPPLITANTVLSILALDYPVDKVSCYVSDDGAAMLSFESLVETADFARKWVPFCKKYSIEPRAPEFYFSLKIDYLRDKVQPSFVKERRAMKRDYEEFKIRMNALVAKAQKTPEEGWTMQDGTSWPGNNTRDHPGMIQVFLGYSGARDIEGNELPRLVYVSREKRPGYQHHKKAGAENALVRVSAVLTNAPFILNLDCDHYVNNSKAVREAMCFLMDPVVGQDVCFVQFPQRFDGIDKSDRYANRNIVFFDVNMRGLDGIQGPVYVGTGTVFRRQALYGYSPPSKPRILPQSSSSSCCCLTKKKQPQDPSEIYKDAKREELDAAIFNLGDLDNYDEYDRSMLISQTSFEKTFGLSTVFIESTLMENGGVPDSVNPSTLIKEAIHVISCGYEEKTEWGKEIGWIYGSITEDILTGFKMHCRGWRSIYCMPLRPAFKGSAPINLSDRLHQVLRWALGSVEIFLSRHCPLWYGCSGGRLKLLQRLAYINTIVYPFTSLPLVAYCTLPAICLLTGKFIIPTLSNLASMLFLGLFISIILTSVLELRWSGVSIEDLWRNEQFWVIGGVSAHLFAVFQGFLKMLAGLDTNFTVTSKTADDLEFGELYIVKWTTLLIPPTSLLIINLVGVVAGFSDALNKGYEAWGPLFGKVFFAFWVILHLYPFLKGLMGRQNRTPTIVILWSILLASVFSLVWVRINPFVSKTDTTSLSLNCLLIDC

>P13569

MQRSPLEKASVVSKLFFSWTRPILRKGYRQRLELSDIYQIPSVDSADNLSEKLEREWDRELASKKNPKLINALRRCFFWRFMFYGIFLYLGEVTKAVQPLLLGRIIASYDPDNKEERSIAIYLGIGLCLLFIVRTLLLHPAIFGLHHIGMQMRIAMFSLIYKKTLKLSSRVLDKISIGQLVSLLSNNLNKFDEGLALAHFVWIAPLQVALLMGLIWELLQASAFCGLGFLIVLALFQAGLGRMMMKYRDQRAGKISERLVITSEMIENIQSVKAYCWEEAMEKMIENLRQTELKLTRKAAYVRYFNSSAFFFSGFFVVFLSVLPYALIKGIILRKIFTTISFCIVLRMAVTRQFPWAVQTWYDSLGAINKIQDFLQKQEYKTLEYNLTTTEVVMENVTAFWEEGFGELFEKAKQNNNNRKTSNGDDSLFFSNFSLLGTPVLKDINFKIERGQLLAVAGSTGAGKTSLLMMIMGELEPSEGKIKHSGRISFCSQFSWIMPGTIKENIIFGVSYDEYRYRSVIKACQLEEDISKFAEKDNIVLGEGGITLSGGQRARISLARAVYKDADLYLLDSPFGYLDVLTEKEIFESCVCKLMANKTRILVTSKMEHLKKADKILILHEGSSYFYGTFSELQNLQPDFSSKLMGCDSFDQFSAERRNSILTETLHRFSLEGDAPVSWTETKKQSFKQTGEFGEKRKNSILNPINSIRKFSIVQKTPLQMNGIEEDSDEPLERRLSLVPDSEQGEAILPRISVISTGPTLQARRRQSVLNLMTHSVNQGQNIHRKTTASTRKVSLAPQANLTELDIYSRRLSQETGLEISEEINEEDLKECFFDDMESIPAVTTWNTYLRYITVHKSLIFVLIWCLVIFLAEVAASLVVLWLLGNTPLQDKGNSTHSRNNSYAVIITSTSSYYVFYIYVGVADTLLAMGFFRGLPLVHTLITVSKILHHKMLHSVLQAPMSTLNTLKAGGILNRFSKDIAILDDLLPLTIFDFIQLLLIVIGAIAVVAVLQPYIFVATVPVIVAFIMLRAYFLQTSQQLKQLESEGRSPIFTHLVTSLKGLWTLRAFGRQPYFETLFHKALNLHTANWFLYLSTLRWFQMRIEMIFVIFFIAVTFISILTTGEGEGRVGIILTLAMNIMSTLQWAVNSSIDVDSLMRSVSRVFKFIDMPTEGKPTKSTKPYKNGQLSKVMIIENSHVKKDDIWPSGGQMTVKDLTAKYTEGGNAILENISFSISPGQRVGLLGRTGSGKSTLLSAFLRLLNTEGEIQIDGVSWDSITLQQWRKAFGVIPQKVFIFSGTFRKNLDPYEQWSDQEIWKVADEVGLRSVIEQFPGKLDFVLVDGGCVLSHGHKQLMCLARSVLSKAKILLLDEPSAHLDPVTYQIIRRTLKQAFADCTVILCEHRIEAMLECQQFLVIEENKVRQYDSIQKLLNERSLFRQAISPSDRVKLFPHRNSSKCKSKPQIAALKEETEEEVQDTRL

>Q29411

MGLRVAQTGFVALVLLQSCAAYKLVCYYTSWSQYREGDGSCFPDAIDPFLCTHIIYSFANISNNEIDTWEWNDVTLYDTLNTLKNRNPNLKTLLSVGGWNFGSQRFSKIASKTQSRRTFIKSVPPFLRTHGFDGLDLAWLYPGRRDKRHLTTLVKEMKAEFIREAQAGTEQLLLSAAVSAGKVAIDRGYDIAQISQHLDFISLLTYDFHGAWRQTTGHHSPLFRGQEDASSDRFSNADYAVSYVLRLGAPANKLVMGIPTFGRSFTLASSKTDVGAPVSGPGIPGRFTKEKGILAYYEICDFLQGATTHRFRDQQVPYATKGNQWVGYDDQESVKNKAKYLKSRQLAGAMVWALDLDDFRGNFCGQNLRFPLTSAIKDVLAAA

>Q9FRV0

MRSLAVVVAVVATVAMAIGTAHGSVSSIISHAQFDRMLLHRNDGACQAKGFYTYDAFVAAANAFPGFGATGSTDARKRDVAAFLAQTSHETTGGWATAPDGAFAWGYCFKQERGAAADYCTPSAQWPCAPGKRYYGRGPIQLSHNYNYGPAGRAIGVDLLRNPDLVATDPTVSFKTALWFWMTAQAPKPSSHAVITGKWSPSGADRAAGRAPGFGVITNIINGGLECGHGQDSRVADRIGFYKRYCDILGVGYGDNLDCYNQRPFA

>P17652

MKLAYWMYAGPAHIGTLRVASSFKNVHAIMHAPLGDDYFNVMRSMLERERDFTPVTTSIVDRHVLARGSQEKVVENITRKDNEESPDLIILTPTCTSSILQEDLQNFVNRASMSENSTSDVLLADVNHYRVNELQAADRTLEQIVRFYLEKEKSTLFRQGVSSVDDNNKTLTNNFLSIKTEKPSANIIGIFTLGFHNQHDCRELKRLLNNLGIEINEVIPEGGSVKNLKNLPKAWFNIIPYREVGLMSAIYLEKEFNMPYVAVSPIGIIDTAVCIREIERILNKIYFESLEGNVNTQSVLTTNPYFNSHINSTNSETRDHNVKPFDFEFYIENQTRFISQAAWFSRSIDCQNLTGKKAVVFGDATHAAGITKILAREMGIKVVCSGTYCKHDADWFREQVFGFCDQILITDDHTQVGDMIAKLEPSAIFGTQMERHIGKRLDIPCGVISAPVHIQNFPLSYRPFLGYEGTNQIADLVYNSFSLGMEDHLLEIFSGHDTKEPITKSLSTENELNWDAEALKELSNVPGFVRGKVKRNTEKYARQNAIPSITLDVLFAAKEALSA

>P06276

MHSKVTIICIRFLFWFLLLCMLIGKSHTEDDIIIATKNGKVRGMNLTVFGGTVTAFLGIPYAQPPLGRLRFKKPQSLTKWSDIWNATKYANSCCQNIDQSFPGFHGSEMWNPNTDLSEDCLYLNVWIPAPKPKNATVLIWIYGGGFQTGTSSLHVYDGKFLARVERVIVVSMNYRVGALGFLALPGNPEAPGNMGLFDQQLALQWVQKNIAAFGGNPKSVTLFGESAGAASVSLHLLSPGSHSLFTRAILQSGSFNAPWAVTSLYEARNRTLNLAKLTGCSRENETEIIKCLRNKDPQEILLNEAFVVPYGTPLSVNFGPTVDGDFLTDMPDILLELGQFKKTQILVGVNKDEGTAFLVYGAPGFSKDNNSIITRKEFQEGLKIFFPGVSEFGKESILFHYTDWVDDQRPENYREALGDVVGDYNFICPALEFTKKFSEWGNNAFFYYFEHRSSKLPWPEWMGVMHGYEIEFVFGLPLERRDNYTKAEEILSRSIVKRWANFAKYGNPNETQNNSTSWPVFKSTEQKYLTLNTESTRIMTKLRAQQCRFWTSFFPKVLEMTGNIDEAEWEWKAGFHRWNNYMMDWKNQFNDYTSKKESCVGL

>Q85FG6

MKTPETLAFECETGNYHTFCPISCVAWLYQKIEDSFFLVVGTKTCGYFLQNALGVMIFAEPRYAMAELEEGDISAQLNDQKELEKICLQIRDDRNPSVIIWIGTCTTEIIKMDLEGIAPKLEKQIGIPIIVARANGLDYAFTQGEDTVLAAMAHRCPEYKHCTTNLKNRTDEMARIDNHTTNKSPFEASKLTRFNLVLFGSLSSSIASQLNLELKRQSISVSGWLPSQKYEELPGLGEGIYVCGVNPFLSRTATTLMRRRKCKLVGAPFPIGPDGTRAWIEKICSVFDIKSYGLENRESEIWENVKDYIQVINGKSVFFMGDNLLEISLARFLIRCGMIVYEVGIPYMDRRYQAAELLFLQHTCRKMKTPLPRIVEKPDNYGQIQRMHELKPHLAITGMAHANPLEARNIDTKWSVEFTFAQIHGFSSVRDILELVTRPLRRRGVITPGFRSLSKQTTGD

>P93324

MGNSYITKEDNQISATSEQTEDSACLSAMVLTTNLVYPAVLNAAIDLNLFEIIAKATPPGAFMSPSEIASKLPASTQHSDLPNRLDRMLRLLASYSVLTSTTRTIEDGGAERVYGLSMVGKYLVPDESRGYLASFTTFLCYPALLQVWMNFKEAVVDEDIDLFKNVHGVTKYEFMGKDKKMNQIFNKSMVDVCATEMKRMLEIYTGFEGISTLVDVGGGSGRNLELIISKYPLIKGINFDLPQVIENAPPLSGIEHVGGDMFASVPQGDAMILKAVCHNWSDEKCIEFLSNCHKALSPNGKVIIVEFILPEEPNTSEESKLVSTLDNLMFITVGGRERTEKQYEKLSKLSGFSKFQVACRAFNSLGVMEFYK

>P17957

MVSVEEIRKAQRAEGPATVMAIGTATPPNCVDQSTYPDYYFRITNSEHMTELKEKFKRMCDKSMIKKRYMYLNEEILKENPSVCAYMAPSLDARQDMVVMEVPKLGKEAATKAIKEWGQPKSKITHLIFCTTSGVDMPGADYQLTKLLGLRPSVKRYMMYQQGCFAGGTVLRLAKDLAENNKGARVLVVCSEITAVTFRGPTDTHLDSLVGQALFGDGAAAVIVGSDPLPVEKPLFQLVWTAQTILPDSEGAIDGHLREVGLTFHLLKDVPGLISKNIEKALVEAFQPLGISDYNSIFRIAHPGGPAILDQVEAKLGLKPEKMEATRHVLSEYGNMSSACVLFILDQMRKKSIENGLGTTGEGLDWGVLFGFGPGLTVETVVLRSVTV

>Q6IQX7

MRASLLLSVLRPAGPVAVGISLGFTLSLLSVTWVEEPCGPGPPQPGDSELPPRGNTNAARRPNSVQPGSERERPGAGAGTGESWEPRVLPYHPAQPGQATKKAVRTRYISTELGIRQKLLVAVLTSQATLPTLGVAVNRTLGHRLEHVVFLTGARGRRTPSGMAVVALGEERPIGHLHLALRHLLEQHGDDFDWFFLVPDATYTEAHGLDRLAGHLSLASATHLYLGRPQDFIGGDTTPGRYCHGGFGVLLSRTLLQQLRPHLESCRNDIVSARPDEWLGRCILDATGVGCTGDHEGMHYNYLELSPGEPVQEGDPRFRSALTAHPVRDPVHMYQLHKAFARAELDRTYQEIQELQWEIQNTSRLAADGERASAWPVGIPAPSRPASRFEVLRWDYFTEQYAFSCADGSPRCPLRGADQADVADVLGTALEELNRRYQPALQLQKQQLVNGYRRFDPARGMEYTLDLQLEALTPQGGRWPLTRRVQLLRPLSRVEILPVPYVTEASRLTVLLPLAAAERDLASGFLEAFATAALEPGDAAALTLLLLYEPRQAQRAAHSDVFAPVKAHVAELERRFPGARVPWLSVQTAAPSPLRLMDLLSKKHPLDTLFLLAGPDTVLTPDFLNRCRMHAISGWQAFFPMHFQAFHPAVAPPQGPGPPELGRDTGHFDRQAASEACFYNSDYVAARGRLVAASEQEEELLESLDVYELFLRFSNLHVLRAVEPALLQRYRAQPCSARLSEDLYHRCRQSVLEGLGSRTQLAMLLFEQEQGNST

>Q8TJJ1

MRDGEQTPGVALTREKKLLIARALDEMRINVIEAGSAITSAGERESIKAVANAGLDAEICSYCRIVKMDVDHALECDVDSIHLVAPVSDLHIKTKIKKDRDTVRQIAAEVTEYAKDHGLIVELSGEDASRADPEFLKAIYSDGIDAGADRLCFCDTVGLLVPEKTTEIFRDLSSSLKAPISIHCHNDFGLATANTVAALAAGAKQSHVTINGLGERAGNASLEEVVMSLEWLYKYDTGIKHEQIYRTSRLVSRLTGIPVSPNKALVGGNAFTHEAGIHVHGLLADKSTYEPMSPEYIGRQRQIVLGKHAGRSSITLALKEMGLEADEAQTEEIFNRVKQMGDQGKHITDADLQTIAETVLDIYKEPIVKLEEFTIVSGNRVTPTASIKLNVKDKEIVQAGIGNGPVDAVINAIRRAVSSCAEDVVLEEYHVDSITGGTDALVEVRVKLSKNGKVITASGARTDIIMASVEAVMNGMNRLIREE

>P43635

MVQRLLPGAHICRRSFNSSAIIKSSALTLKEALENVIPKKRDAVKKLKACYGSTFVGPITISSVLGGMRGNQSMFWQGTSLDPEHGIKFQGLTIEECQNRLPNTGIDGDNFLPESMLWLLMTGGVPTFQQAASFRKELAIRGRKLPHYTEKVLSSLPKDMHPMTQLAIGLASMNKGSLFATNYQKGLIGKMEFWKDTLEDSLNLIASLPLLTGRIYSNITNEGHPLGQYSEEVDWCTNICSLLGMTNGTNSSNTCNLTSQQSLDFINLMRLYTGIHVDHEGGNVSAHTTHLVGSALSDPYLSYSSGIMGLAGPLHGLAAQEVVRFLIEMNSNISSIAREQEIKDYLWKILNSNRVIPGYGHAVLRKPDPRFTAMLEFAQKRPIEFENDKNVLLMQKLAEIAPKVLLEHGKSKNPFPNVDSASGILFYHYGIRELLFFTVIFGCSRAMGPLTQLVWDRILGLPIERPKSLNLEGLEALTKASNVNKL

>P0A2G0

MTNMTQASATEKKGASDLLRFKIFGMPLPLYAFALITLLLSHFYNAIPTDLVGGFALMFVMGAIFGEIGKRLPIFNKYIGGAPVMIFLVAAYFVYAGIFTQKEIDAISNVMDKSNFLNLFIAVLITGAILSVNRKLLLKSLLGYIPTILAGIVGASLFGIVIGLCFGIPVDRIMMLYVLPIMGGGNGAGAVPLSEIYHSVTGRSREEYYSTAIAILTIANIFAIIFAALLDMIGKKYTWLSGEGELVRKASFKTEDDEKAGQITHRETAVGMVLSTTCFLLAYVVAKKILPSIGGVSIHYFAWMVLIVAALNASGLCSPEIKAGAKRLSDFFSKQLLWVLMVGVGVCYTDLQEIIDALTFANVVIAAIIVVGAVVGAAIGGWLIGFYPIESSITAGLCMANRGGSGDLEVLSACNRMNLISYAQISSRLGGGIVLVIASIVFSMMV

>Q03059

PILEKVPPKMPVQASSCEEVLDLPKLPVPPLQQTLATYLQCMQHLVPEEQFRKSQAIVKRFGAPGGLGETLQEKLLERQEKTANWVSEYWLNDMYLNNRLALPVNSSPAVIFARQHFQDTNDQLRFAASLISGVLSYKALLDSQSIPTDWAKGQLSGQPLCMKQYYRLFSSYRLPGHTQDTLVAQKSSIMPEPEHVIVACCNQFFVLDVVINFRRLSEGDLFTQLRKIVKMASNEDERLPPIGLLTSDGRSEWAKARTVLLKDSTNRDSLDMIERCICLVCLDGPGTGDLSDTHRALQLLHGGGCSLNGANRWYDKSLQFVVGRDGTCGVVCEHSPFDGIVLVQCTEHLLKHMMTGNKKLVRVDSVSELPAPRRLRWKCSPETQGHLASSAEKLQRIVKNLDFIVYKFDNYGKTFIKKQKCSPDGFIQVALQLAYYRLYQRLVPTYESASIRRFQEGRVDNIRSATPEALAFVQAMTDHKAAVLASEKLQLLQRAIQAQTEYTVMAITGMAIDNHLLALRELARDLCKEPPEMFMDETYLMSNRFILSTSQVPTTMEMFCCYGPVVPNGYGACYNPHAEAITFCISSFHGCKETSSVEFAEAVGASLVDMRDLCSSRQPADSKPPTAKERARGPSQAKQS

>O67987

MANTRVIELFDEFTDLIRDFIVRHEITTPEYETIMQYMISVGEAGEWPLWLDAFFETTVDSVSYGKGNWTSSAIQGPFFKEGAPLLTGKPATLPMRADEPGDRMRFTGSVRDTSGTPITGAVIDVWHSTNDGNYSFFSPALPDQYLLRGRVVPAEDGSIEFHSIRPVPYEIPKAGPTGQLMNSYLGRHSWRPAHIHIRITADGYRPLITQLYFEGDPYLDSDSCSAVKSELVLPVNKIDIDGETWQLVDFNFILQHN

>P08082

MAEDFGFFSSSESGAPEAAEEDPAAAFLAQQESEIAGIENDSGFGAPAASQVASAQPGLASGGGSEDMGTTVNGDVFQEANGPADGYAAIAQADRLTQEPESIRKWREEQKKRLQELDAASKVTEQEWREKAKKDLEEWNQRQSEQVEKNKINNRIADKAFYQQPDADTIGYVASEEAFVKESKEETPGTEWEKVAQLCDFNPKSSKQCKDVSRLRSVLMSLKQTPLSR

>Q06393

MEELVGLREGSSGKPVTLQELWGPCPRIRRGVRRGLEWLKERLFRVGEDWHFLVALGVLMALISYAMNFAIGRVVRAHKWLYREVGDGHLLRYLSWTVYPVALLSFSSGFSQSISPFSGGSGLPELKTMLSGVVLEDYLDIKNFGAKVVGLSCTLATGSTIFLGKVGPFVHLSVMISAYLGRVRAKTIGETENKAKEIEMLSAAAAVGVATVFAAPFSGVLFSIEVMSSHFSVWNYWRGFFAATCGAFMFRLLGVFNSEQETITSIYKTRFRVDVPFDLPEIFFFVALGFICGVLSCAYLFCQRTFLRFIKTNRYTSRLLATSKPSYAALVALVLASITYPPGVGRFMASRLSMAEHLHSLFDNNSWALMTRNSSPPWPAEPDPQNLWLEWCHPRFTIFGTLAFFLVMKFWMLILATTIPMPAGYFMPIFIIGAAIGRLLGEALSVAFPEGIVAGREVNPIMPGGYALAGAAAFSGAVTHTISTALLAFELTGQIVHALPVLMAVLAANAISQNCQPSFYDGTIMAKKLPYLPWIRGRQIGSYPVTVEHFMNCNLTTLAKDTPLEEVVKVVTSTEVSQYPLVETRESQTLVGIVERTHLVQALQTQPASWAPGQERFLQDILAGGCPTQPVTLQLSPETSLYQAHSLFERLTLQSLFVTSRGKAVGSVSWAELKKAISTLINPPAPK

>Q9N2G5

MAETAEPEGGAPSPQGPPEGSALLEERPGEPDPAGPEASEGAAKAPSGEGAGAAAKAGATEEASGGRDGEGAGEQAPDAGTESGGETPDAKGAQIEAEGAPEGTKAPQLGEEGSGGKQVEESGPDCELRGEAAREAEGQAAAPAAPGAQEEAVPGDSVDAEGSIDAGGSVDAAGSVDAGGSIDAGGSMDAGGSVDAGGSIDTGGSVDAAGSVDAGGSIDTGRNVDAGGSIDAGGSVDAGGSMDAEGPAGGAHGAGGEPQDLGAGSPQPRSEAVEVAAAENEGHSPGESVEDAAAEEAAGTREPEGSEDAAGEDGDQGRPQEETEQQAERQEPGPETQSEEEERPPDRSPDGEAAASTRAAQPEAELSNHLAAEEGGQRGEGPANGRGEDGEASEEGDPGQEHDITLFVKAGYDGESIGNCPFSQRLFMILWLKGVIFNVTTVDLKRKPADLQNLAPGTNPPFMTFDGDVKTDVNKIEEFLEEKLAPPRYPKLATQHPESNSAGNDVFAKFSAFIKNTKKDANEIYEKSLLKALKKLDAYLNSPLPDEVDAYSTEDVAVSGRKFLDGDDLTLADCNLLPKLHIIKIVAKKYRDFEFPPEMTGIWRYLNNAYARDEFINTCPADQEIEHAYSDVAKRMK

>Q9Z832

MADGEVHKLRDIIEKELLEARRVFFSEPVTEKSASDAIKKLWYLELKDPGKPIVFVINSPGGSVDAGFAVWDQIKMLTSPVTTVVTGLAASMGSVLSLCAAPGRRFATPHSRIMIHQPSIGGPITGQATDLDIHAREILKTKARIIDVYVEATNQPRDIIEKAIDRDMWMTANEAKDFGLLDGILFSFNDL

>O66043

MKNRLNVLAFFALLFAALYISRGFLQSWMVGTLSVVFTLSVIFIGIIIFFENRHPTKTLTWLLVLAAFPVVGFFFYLMFGQNHRKSKRFSKKAIEDERAFQKIEGQRQLNEEQLKKMGGHQQLLFRLAHKLGKNPISFSSETKVLTDGKETYAHILQALKMAEHHIHLEYYIVRHDDLGNQIKDILISKAKEGVHVRFLYDGVGSWKLSKSYVEELRDAGVEMVSFSPVKLPFLTHTINYRNHRKIIVIDGVVGFVGGLNIGDEYLGKDAYFGYWRDTHLYVRGEAVRTLQLIFLQDWHYQTGETILNQTYLSPSLSMTKGDGGVQMIASGPDTRWEVNKKLFFSMITSAKKSIWIASPYFIPDDDILSALKIAALSGIDVRILVPNRPDKRIVFHASRSYFPELLEAGVKVYEYNRGFMHSKIIIVDHEIASIGTSNMDMRSFHLNFEVNAYLYRTSSVTKLVSDYVYDLEHSNQINFSLFKNRPFFHRLIESTSRLLSPLL

>P92692

MFADRWLFSTNHKDIGTLYLLFGAWAGVLGTALSLLIRAELGQPGNLLGNDHIYNVIVTAHAFVMIFFMVMPIMIGGFGNWLVPLMIGAPDMAFPRMNNMSFWLLPPSFLLLLASATVEAGAGTGWTVYPPLAGNYSHPGASVDLTIFSLHLAGISSILGAINFITTIINMKPPAMSQYQTPLFVWSVLITAVLLLLSLPVLAAGITMLLTDRNLNTTFFDPAGGGDPILYQHLFWFFGHPEVYILILPGFGMISHIVTHYSGKEEPFGYMGMVWAMVSIGFLGFIVWAHHMFTVGMDVDTRAYFTSATMIIAIPTGVKVFSWLATLHGSNTKWSAAILWALGFIFLFTVGGLTGIVLANSSLDIVLHDTYYVVAHFHYVLSMGAVFAIMGGFIHWFPLFSGYTLDQTYAKIHFITMFIGVNLTFFPQHFLGLSGMPRRYSDYPDAYTTWNILSSAGSFISLTAVMLMIFMIWEAFASKRKVPMVEQPSTSLEWLYGCPPPYHTFEEPVYMKPE

>P48892

MTHQTHAYHMVNPSPWPLTGALSALLMTSGLAMWFHYNLTLLLTLGMTTNLLTMYQWWRDIIRESTFQGHHTPIVQKGLRYGMILFIISEVFFFAGFFWAFYHSSLAPTPELGGCWPPTGIIPLNPLEVPLLNTSVLLASGVSITWAHHSLMEGNRKHMLQALFITISLGVYFTLLQASEYYETSFTISDGVYGSTFFMATGFHGLHVIIGSTFLIVCFLRQLKYHFTSNHHFGFEAAAWYWHFVDVVWLFLYVSIYWWGS

>P05093

MWELVALLLLTLAYLFWPKRRCPGAKYPKSLLSLPLVGSLPFLPRHGHMHNNFFKLQKKYGPIYSVRMGTKTTVIVGHHQLAKEVLIKKGKDFSGRPQMATLDIASNNRKGIAFADSGAHWQLHRRLAMATFALFKDGDQKLEKIICQEISTLCDMLATHNGQSIDISFPVFVAVTNVISLICFNTSYKNGDPELNVIQNYNEGIIDNLSKDSLVDLVPWLKIFPNKTLEKLKSHVKIRNDLLNKILENYKEKFRSDSITNMLDTLMQAKMNSDNGNAGPDQDSELLSDNHILTTIGDIFGAGVETTTSVVKWTLAFLLHNPQVKKKLYEEIDQNVGFSRTPTISDRNRLLLLEATIREVLRLRPVAPMLIPHKANVDSSIGEFAVDKGTEVIINLWALHHNEKEWHQPDQFMPERFLNPAGTQLISPSVSYLPFGAGPRSCIGEILARQELFLIMAWLLQRFDLEVPDDGQLPSLEGIPKVVFLIDSFKVKIKVRQAWREAQAEGST

>P00178

MEFSLLLLLAFLAGLLLLLFRGHPKAHGRLPPGPSPLPVLGNLLQMDRKGLLRSFLRLREKYGDVFTVYLGSRPVVVLCGTDAIREALVDQAEAFSGRGKIAVVDPIFQGYGVIFANGERWRALRRFSLATMRDFGMGKRSVEERIQEEARCLVEELRKSKGALLDNTLLFHSITSNIICSIVFGKRFDYKDPVFLRLLDLFFQSFSLISSFSSQVFELFPGFLKHFPGTHRQIYRNLQEINTFIGQSVEKHRATLDPSNPRDFIDVYLLRMEKDKSDPSSEFHHQNLILTVLSLFFAGTETTSTTLRYGFLLMLKYPHVTERVQKEIEQVIGSHRPPALDDRAKMPYTDAVIHEIQRLGDLIPFGVPHTVTKDTQFRGYVIPKNTEVFPVLSSALHDPRYFETPNTFNPGHFLDANGALKRNEGFMPFSLGKRICLGEGIARTELFLFFTTILQNFSIASPVPPEDIDLTPRESGVGNVPPSYQIRFLAR

>Q33800

MINLRKTHPLMKIINHSFIDLPAPSNISAWWNFGSLLGACLIIQILTGFFLAMHYTSDTLTAFSSVAHICRDVNYGWLIRNLHANGASMFFMCLFLHIGRGIYYGSYLYKETWNIGVILLLTVMATAFVGYVLPWGQMSFWGTTVITNLLSAIPYIGTTLAEWIWGGFAVDKATLTRFFAFHFILPFIIMALAMVHLLFLHETGSNNPTGINPDSDKIPFHPYYTIKDALGMTLLLLVLLLLALFSPDSLGDPDNFSPANPLNTPPHIKPEWYFLFAYAILRSIPNKLGGVLALLASILILLIMPLLHTANQRSMMFRPVSQTLFWILAADLITLTWVGGQPVEQPYIIIGQLASMLYFLLILILMPLAGMFENYMLKPKW

>Q9PIT2

MIKIGIYGAKGRMGKQIEECLKSETQARISILYDKGGNLGELFEKSDVIIDFSSPSGTHELLNYARTMPKPLVIGTTGLDEKILHLMQSASEVMPIFYATNMSLGVAVLNYLASKASQMLRNFDIEILEMHHRHKKDAPSGTAMTLAQSVAKARNLELEKVRVSGRDGIIGERSKDEIAVMSLRGGDIVGRHTVGFYEDGEFLELNHTATSRATFAKGAIKIAIWLSKQEAKMYSINDFLGI

>Q9I4L1

MPGAVDFKERISRQRPHDRETYGHAGNTDLQDIVYQLESDRGRIVNSAAVRRLQQKTQVFPLERNAAVRSRLTHSLEVQQTGRFIVRTLFRQLGPRAAEVGLDGLEGALESLVEMACLMHDVGNPPFGHFGEYAINDWFERNLDALFERRIPPGQGDGLLQQRMLTDLKHFEGNAQAIRLVVKLLRLNLTYTQTAGLLKYVRPAYEPKPDKAAANHYLNKKPGFYLSEEAFVDELRRVLGMRPGTRHPVAYIMEAADDISYCLADIEDSVEKGILDIRQLADLLVKKFAVHHSPDAPIPGDADNMSFQRMVDYSLEKAEREPINKVSEFFIRLRVKMIHPLVQHAAQQFIDNFEAVHAGTLGRALMEDGSLPHAIVQTFKDVAMEWVFCHPEVETLELQGYRIIQGLLDFYAPLLRLPAEEFQALAEGRQAAAPHPQLLVRRLPSQQIKAYLEAMKGVAEDPLQRQWEFYHRCRMLQDFVSGMTDQHAQDEYRALSAL

>P71019

MSKIAFLFPGQGSQFIGMGKELYEQVPAAKRLFDEADETLETKLSSLIFEGDAEELTLTYNAQPALLTTSIAVLEKFKESGITPDFTAGHSLGEYSALVAAGALSFKDAVYTVRKRGEFMNEAVPAGEGAMAAILGMDAEALKQVTDKVTEEGNLVQLANLNCPGQIVISGTAKGVELASELAKENGAKRAIPLEVSGPFHSELMKPAAEKLKEVLDACDIKDADVPVISNVSADVMTEKADIKEKLIEQLYSPVRFEESINKLIAEGVTTFIEIGPGKVLSGLVKKVNRRLKTIAVSDPETIELAIQTLKEENDNA

>P48623

MVVAMDQRTNVNGDPGAGDRKKEERFDPSAQPPFKIGDIRAAIPKHCWVKSPLRSMSYVVRDIIAVAALAIAAVYVDSWFLWPLYWAAQGTLFWAIFVLGHDCGHGSFSDIPLLNSVVGHILHSFILVPYHGWRISHRTHHQNHGHVENDESWVPLPERVYKKLPHSTRMLRYTVPLPMLAYPLYLCYRSPGKEGSHFNPYSSLFAPSERKLIATSTTCWSIMFVSLIALSFVFGPLAVLKVYGVPYIIFVMWLDAVTYLHHHGHDEKLPWYRGKEWSYLRGGLTTIDRDYGIFNNIHHDIGTHVIHHLFPQIPHYHLVDATKAAKHVLGRYYREPKTSGAIPIHLVESLVASIKKDHYVSDTGDIVFYETDPDLYVYASDKSKIN

>Q8ZNA5

MVMSQKTLFTKSALAVAVAIISTQAWSAGFQLNEFSSSGLGRAYSGEGAIADDAGNVSRNPALITMFDRPTFSAGAVYIDPDVNISGTSPSRRTLDADNIAPTAWVPNVHFVAPINDQFGWGASITSNYGLATEFNDTYAGGSVGGTTDLETMNLNLSGAYRLNEAWSFGLGFDAVYARAKIERFAGDLGQLVAAQNPALAPVAGQIPSDTKIAHLNGNQWGFGWNAGILYELDKNNRYALTYRSEVKIDFKGNYSSDLPIAINRFNLPIPTATGGATQSGYLTLNLPEMWEVSGYNRVAPQWAIHYSLAYTSWSQFQELKAKSTAGDTLFEKHEGFKDAYRIALGTTYYYDDNWTFRTGIAFDDSPVPAQNRSISIPDQDRFWLSAGTTYAFNKDASVDVGVSYMHGQSVKINEGPYQFESEGKAWLFGTNFNYAF

>Q9NW38

MAVTEASLLRQCPLLLPQNRSKTVYEGFISAQGRDFHLRIVLPEDLQLKNARLLCSWQLRTILSGYHRIVQQRMQHPPDLMSFMMELKMLLEVALKNRQELYALPPPPQFYSSLIEEIGTLGWDKLVYADTCFSTIKLKAEDASGREHLITLKLKAKYPAESPDYFVDFPVPFCASWTPQSSLISIYSQFLAAIESLKAFWDVMDEIDEKTWVLEPEKPPRSATARRIALGNNVSINIEVDPRHPTMLPECFFLGADHVVKPLGIKLSRNIHLWDPENSVLQNLKDVLEIDFPARAILEKSDFTMDCGICYAYQLDGTIPDQVCDNSQCGQPFHQICLYEWLRGLLTSRQSFNIIFGECPYCSKPITLKMSGRKH

>Q92SC4

MKALVENLKATARETDATDIRAAFAADPNRFSRFSTAFDDLLFDYSKCAVNDRIIDGLEALAKAAKVEEKRDAMFRGDIINITEERAVLHTALRNRSNRPVLVDGKDVMPDVNAVLEAMGKFADDIRSGALKGATGKKITDVVNIGIGGSDLGPVMATLALAPFHDGPRLHFVSNVDGAHIADTLTLLDPETSLFIVASKTFTTIETMTNAATARAFIAGKLGEAAVGHHFAAVSTALDKVGAFGIDAARVFGFWDWVGGRYSIWSAIGLPLMIAIGKENFGRFLDGGHAIDEHFRSAPLRQNIPMLLGLIGFYNRNVLGYPSRAILPYDQRLTRFPAYLQQLDMESNGKGVTLDSQPVEFSTGPVVWGEPGTNGQHAFYQLIHQGTDVIPAEFMIAANGHEKDLRHQHQLLMANCLAQSEALMKGRTLAEAKAQLTSKGMDDAKADKIAPHRVFTGNRPSLTIVYDQLDPFALGRLIALYEHRVFVEGALFNINSFDQWGVELGKELATGLLPVIEGKESAEGHDSSTAGLVAALLKAAR

>Q5RDY7

MATEGLHENETLASLKSEAESLKGKLEEERAKLRDVELHQVAERVEALGQFVMKTRRTLKGHGNKVLCMDWCKDKRRIVSSSQDGKVIVWDSFTTNKEHAVTMPCTWVMACAYAPSGCAIACGGLDNKCSVYPLTFDKNENMAAKKKSVAMHTNYLSACSFTNSDMQILTASGDGTCALWDVESGQLLQSFHGHGADVLCLDLAPSETGNTFVSGGCDKKAMVWDMRSGQCVQAFETHESDINSVRYYPSGDAFASGSDDATCRLYDLRADREVAIYSKESIIFGASSVDFSLSGRLLFAGYNDYTINVWDVLKGSRVSILFGHENRVSTLRVSPDGTAFCSGSWDHTLRVWA

>P50573

MVLAFWLAFFTYTWITLMLDASAVKEPHQQCLSSPKQTRIRETRMRKDDLTKVWPLKREQLLHIEDHDFSTRPGFGGSPVPVGIDVQVESIDSISEVNMDFTMTFYLRHYWKDERLSFPSTTNKSMTFDRRLIQKIWVPDIFFVHSKRSFIHDTTVENIMLRVHPDGNVLFSLRITVSAMCFMDFSRFPLDTQNCSLELESYAYNEEDLMLYWKHGNKSLNTEEHISLSQFFIEEFSASSGLAFYSSTGWYYRLFINFVLRRHIFFFVLQTYFPAMLMVMLSWVSFWIDRRAVPARVSLGITTVLTMSTIVTGVSASMPQVSYVKAVDVYMWVSSLFVFLSVIEYAAVNYLTTVEEWKQLNRRGKISGMYNIDAVQAMAFDGCYHDGETDVDQTSFFLHSEEDSMRTKFTGSPCADSSQIKRKSLGGNVGRIILENNHVIDTYSRIVFPVVYIIFNLFYWGIYV

>P02229

MKFLILALCFAAASALSADQISTVQASFDKVKGDPVGILYAVFKADPSIMAKFTQFAGKDLESIKGTAPFEIHANRIVGFFSKIIGELPNIEADVNTFVASHKPRGVTHDQLNNFRAGFVSYMKAHTDFAGAEAAWGATLDTFFGMIFSKM

>Q89RJ4

MDDFVRVGGLGAVSAALPRALRPFADIRIMLPGYRDIIEQLTHIQIVGRCPSFADLPACSLGRAATKDGLPVYVLLCSQLYDRPGNPYGDESGRDWPDNDIRFARFASAAAELAMGKLDKNWAADLIHANDWQASLVPAYLAWRGAKLPSILTIHNLAYQGLFPKDSLRRIGAPESAFHIDGLEFYDQVSFLKAGLVYASHLTTVSGTYAREITTAEFGCGLEGLLRLRSDAAELTGILNGIDESWDPRSCAQLAQQFGAGDWVGKKANADYVRKQFGLAVSRGPMFGIVARLVHQKGIDLVLSAADEIIDAGGQIVVTGSGEPALEQALIDAHRRRPDAIGVAIGFNDAQARRIFAGSDFTLMPSRFEPCGLSQMYAQRFGSLPIGHQTGGLAETITDGETGFLFSRPSHESFLGGVRRAFEAFMAQDQLDSMRRSAMGRSFSWSISADSYSALYRKLAAV

>Q63T91

MTDAPFDRADIDALLGARHPDPFACLGPHRVGDATVVRTLLPGALRVRAIAAGGGVLGELRQVDPAGCFAGALPDGQERGERPRYRLSIDWPDARQDVEDAYAFGTLLDEDALARFAAGDPRAALACLGARALDMDGVPGVRFAVWAPGASRVSVVGDFNGWDARRHPMRLRRPWGVWELFVPRIGAGERYKFALRARDGAALPLKADPCACRTEAPPRTASIVADLDALERFGWHDDAWLRARASLDLAHAPVSIYEVHPESWLRVAAEGNRSATWDELAQRLIPYAAGMGFSHVELTPIAEYPFGGSWGYQSLSPFAPSARFGPPEGFARFVEHAHAAGLGVIVDWVPAHFPDDPHGLGKFDGTALFEHADPREGWHPDWHTHVFNVGRREVGAFLIASALAWAHRYHVDGIRVDAVASMLYRDYSRAAGEWVPNVYGGRENLESIAFLKHFNDTLHGPAAPPGVATFAEESTAWPGVTAPTAEHGLGFDFKWNMGWMHDTLAYLREDPIHRRHHHDRLTFGLVYAFSERFVLPLSHDEVVHGKGSLAAKMPGDAWQRLANLRAYFGFMWAHPGKKLLFMGGEFAQWGEFAHDATPQWDLLDAPAHRGVQRLVRDLNRLHAAEPALHALDDRPAGFAWLVGDDRNNSVFAFVRRDDAGRMLVAVCNFTPVPRTDYRLGLPAPGRWAEVLNTDGAAYGGTDAGNGGAVQADEIPAHGERWSAALRLPPLATLWLRPA

>P38410

MACCLSEEAEEARRINDEIERQLRRDKRDARRELKLLLLGTGESGKSTFIKQMRIIHGSGYSDEDKRGFTKLVYQNIFSAMQAMIRAMETLKIPYKYEHNKGHALLVREVDVEKVASFENPYVDAIKYLWNDPGIQECYDRRREYQLSDSTKYYLNDLDRIATHGYLPTQQDVLRVRVPTTGIIEYPFDLQSVIFRMVDVGGQRSERRKWIHCFENVTSIMFLVALSEYDQVLVESDNENRMEESKALFRTIITYPWFQNSSVILFLNKKDLLEEKIMYSHLVDYFPEYDGPQRDAQAAREFILKMFVDLNPDSDKIIYSHFTCATDTENIRFVFAAVKDTILQLNLKEYNLV

>P36552

MALRLGRLGSDPWWRAVLGDYAQLRAASPRCASARVCQLPGTAGPQPRRGLGYGPWARGGSGLGTRLAATLAGLAGLAAAAFGHVQRAEMVPKSSGARSPSPGRREEDGDELARRCSTFMSSPVTELRELRRRPEDMKTKMELMIMETQAQVCRALAQVDGVADFTVDRWERKEGGGGITCVLQDGRVFEKAGVSISVVHGNLSEEAANQMRGRGKTLKTKDSKLPFTAMGVSSVIHPKNPYAPTMHFNYRYFEVEEADGNTHWWFGGGCDLTPTYLNQEDAVHFHRTLKEACDQHGPDIYPKFKKWCDDYFFIVHRGERRGIGGIFFDDLDSPSKEEAFRFVKTCAEAVVPSYVPIVKKHCDDSYTPRDKLWQQLRRGRYVEFNLLYDRGTKFGLFTPGSRIESILMSLPLTARWEYMHSPPENSKEAEILEVLRHPKDWVH

>Q8G4S7

MARTAHIVRETSESHIDLELNLDGTGKTDIDTSVPFYNHMMTALGKHSLIDLTIHAHGDTDIDVHHTVEDTAIVFGEALKQALGDKKGIRRFADATVPLDEALAKAVVDISGRPYCVCSGEPEGYEFCMIGGHFTGSLVRHVMESIAFHAGICLHMQVLAGRDPHHIAEAEFKALARALRFAVEIDPRVDGVPSTKGAL

>P41565

MALKVAIAAGSAAKAIFKPALLCRPWEVLAAHEAPRRSISSQQTIPPSAKYGGRHTVTMIPGDGIGPELMLHVKSVFRHACVPVDFEEVHVSSNADEEDIRNAIMAIRRNRVALKGNIETNHDLPPSHKSRNNILRTSLDLYANVIHCKSLPGVVTRHKDIDILIVRENTEGEYSSLEHESVAGVVESLKIITKAKSLRIAEYAFKLAQESGRKKVTAVHKANIMKLGDGLFLQCCREVAARYPQITFDSMIVDNTTMQLVSRPQQFDVMVMPNLYGNIVNNVCAGLVGGPGLVAGANYGHVYAVFETATRNTGKSIANKNIANPTATLLASCMMLDHLKLHSYATSIRKAVLASMDNENMHTPDIGGQGTTSQAIQDIIRHIRIINGRAVEA

>Q8GGL2

MMAYLVFLGPPGAGKGTYAKRIQEKTGIPHISTGDIFRDIVKKENDELGKKIKEIMEKGELVPDELVNEVVKRRLSEKDCEKGFILDGYPRTVAQAEFLDSFLESQNKQLTAAVLFDVPEDVVVQRLTSRRICPKCGRIYNMISLPPKEDELCDDCKVKLVQRDDDKEETVRHRYKVYLEKTQPVIDYYGKKGILKRVDGTIGIDNVVAEVLKIIGWSDK

>Q7N8Z5

MIKVYAPASIGNVSVGFDVLGAAVSPVNGALLGDCVTVRAAKSFSLRNEGQFVGKLPEKLEHNIVYQCWQLFCQHLGKQLPVEMTLEKNMPIGSGLGSSACSVVAGLMALNEFAGLPFNESQLLAMMGELEGRISGSIHYDNVAPCYLGGLQLIMEQGDIICQPVPSFDEWLWVMAYPGIKVSTAEARAILPVKYSKQDVIDHGRFLAGFIHACHTRQPALAARLMKDVVAEPYRTQLLPGFANARETAKRVGALACGISGSGPTLFSICNDIATAEEIAEWLQQHYVQNDEGFVHICRLDLAGARQIG

>Q9UIQ6

MEPFTNDRLQLPRNMIENSMFEEEPDVVDLAKEPCLHPLEPDEVEYEPRGSRLLVRGLGEHEMEEVEEDYESSAKLLGMSFMNRSSGLRNSATGYRQSPDGACSVPSARTMVVCAFVIVVAVSVIMVIYLLPRCTFTKEGCHKKNQSIGLIQPFATNGKLFPWAQIRLPTAVVPLRYELSLHPNLTSMTFRGSVTISVQALQVTWNIILHSTGHNISRVTFMSAVSSQEKQAEILEYAYHGQIAIVAPEALLAGHNYTLKIEYSANISSSYYGFYGFSYTDESNEKKYFAATQFEPLAARSAFPCFDEPAFKATFIIKIIRDEQYTALSNMPKKSSVVLDDGLVQDEFSESVKMSTYLVAFIVGEMKNLSQDVNGTLVSIYAVPENIGQVHYALETTVKLLEFFQNYFEIQYPLKKLDLVAIPDFEAGAMENWGLLTFREETLLYDSNTSSMADRKLVTKIIAHELAHQWFGNLVTMKWWNDLWLNEGFATFMEYFSLEKIFKELSSYEDFLDARFKTMKKDSLNSSHPISSSVQSSEQIEEMFDSLSYFKGSSLLLMLKTYLSEDVFQHAVVLYLHNHSYASIQSDDLWDSFNEVTNQTLDVKRMMKTWTLQKGFPLVTVQKKGKELFIQQERFFLNMKPEIQPSDTSYLWHIPLSYVTEGRNYSKYQSVSLLDKKSGVINLTEEVLWVKVNINMNGYYIVHYADDDWEALIHQLKINPYVLSDKDRANLINNIFELAGLGKVPLKRAFDLINYLGNENHTAPITEALFQTDLIYNLLEKLGYMDLASRLVTRVFKLLQNQIQQQTWTDEGTPSMRELRSALLEFACTHNLGNCSTTAMKLFDDWMASNGTQSLPTDVMTTVFKVGAKTDKGWSFLLGKYISIGSEAEKNKILEALASSEDVRKLYWLMKSSLNGDNFRTQKLSFIIRTVGRHFPGHLLAWDFVKENWNKLVQKFPLGSYTIQNIVAGSTYLFSTKTHLSEVQAFFENQSEATFRLRCVQEALEVIQLNIQWMEKNLKSLTWWL

>P07956

MVKYEDKISLYDAKGNLVAENVPLEAISPLYNPTIKSMLKNIKRTVAVNLADIENTLATGSIGGKGCKVPGRTLDLSVVSNAQAIADEVEKILKVSKDDDTAIKLINGGKQMAVQVPSERLEVAAEYSVSMLATAMALKEAIIKTFNVDMFEGSTVHASIMGNYPQVMDYAGGNIASLLGAPSNLEGLGYALRNIPVNHAVATTKKNMMNAIAFSSVMEQTATFEMGDAIGSFERQHLLGLAYQGLNADNLVIEFIKANGKGTVGTVVQSVVERALADGVIVVDKTMGSGFNMYKPADVNKWNAYAAAGLVAAAAVSCGAARAAQNIASVILYYNDILEYETGLPGVDYGRSMGTAVGFSFFSHSIYGGGGPGIFNGNHVVTRHSKGFAIPPVCAAMCADAGTQMFSPEHTSALVGAVYSAFDEFREPMKYVIERALNIKDKL

>Q5LXE1

MARPKIALIGAGQIGGTLAHLVALKELGDVVLFDIAEGTPEGKALDIAESGPSEGFDAKLKGTQSYADIAGADVCIVTAGVPRKPGMSRDDLLGINLKVMKSVGEGIRDNAPDAFVICITNPLDAMVWALQQFSGLPANKVCGMAGVLDSARFRHFLAEEFNVSMKDVTAFVLGGHGDTMVPSVRYSTVAGIPLPDLIKMGWTSQEKLDAIVQRTRDGGAEIVGLLKTGSAYYAPATSAIEMAEAYLKDQKRVLPCAAYCNGELGVKGMYVGVPTVIGAGGIERIIDVSLTKEEQDMFDNSVNAVKGLVEACKGIDGSLA

>P82159

MPKAPAKKAEPAPAPAPAPEPAPAPAAPAVDLSAVKVEFSADQIEDYREAFGLFDRVGDNKVAYNQIADIMRALGQNPTNKEVSKLLGNPSADDMTNKRVEFEAFLPMLQTIINSPNKAGFEDYVEGLRVFDKEGNGTVMGAELRIVLSTLGEKMTEAEIDALMAGQEDENGVVNYEAFVKHIMSV

>Q9NRE1

MQLVILRVTIFLPWCFAVPVPPAADHKGWDFVEGYFHQFFLTKKESPLLTQETQTQLLQQFHRNGTDLLDMQMHALLHQPHCGVPDGSDTSISPGRCKWNKHTLTYRIINYPHDMKPSAVKDSIYNAVSIWSNVTPLIFQQVQNGDADIKVSFWQWAHEDGWPFDGPGGILGHAFLPNSGNPGVVHFDKNEHWSASDTGYNLFLVATHEIGHSLGLQHSGNQSSIMYPTYWYHDPRTFQLSADDIQRIQHLYGEKCSSDIP

>Q9CMG7

MQDKDLSTVQTFKRLWPIISPFKLGLVVSGIALVINALADAGLISLLKPLLDEGFGKADVSFLRTMSYVVVLVIFLRGISNFISSYCLSWVSGKVVMIMRRRIFKHLMFMPVPFFDQNSSGRLLSRITYDSELVANSSSGALITIVREGAYIISLLAVMLYTSWQLSIVLFLIGPIIAVLIRFVSKRFRELSKNMQNSMGELTSTAEQMLKGHKVVLSFGGQIVEEERFNHVSNDMRRKGMKMAVADAISNPVVQIIASFALAAVLYLATVPTIMDQNLTAGSFTVVFSSMLAMMRPLKSLTNVNAQFQKGMAACQTLFALLDLETEKDLGTHKGENVQGYLSFKNVTFTYQSRDEPALRNLSFDVEKGKTVALVGRSGSGKSTIANLVTRFYDVDQGEITLDGINIQDYRLSSLRKNCAVVSQQVHLFNDTIANNIAYAAKDKYSREEIIKAAKDAYAMEFIEKLEHGLDTVIGENGVNLSGGQRQRLAIARALLRNSPVLILDEATSALDTESERSIQLALEKLQKERTVIVIAHRLSTIENADEILVIEHGEIKERGSHSELLALNGAYKQLHHIQVNH

>O78701

MLLTNILCLMMPILLAVAFLTLTERKILGHMQLRKGPNTVGPHGLLQPVADAVKLFTKEPLHPLTSSKLMFIIAPTLAFTLALSLWAPLPMPHPLINLNLSILFILAMSSLAVHSILWSGWASNSKYALIGALRAVAQTISYEVTLAIILLSIMLINGSFTLSTLTITQEQMWLILPTWPLAMMWFISTLAETNRTPFDLSEGESELVSGFNVEYAAGPFALFFMAEYTNIILMNALTATLFFGAFHNPLFPELHTINLTTKTLILVFLFLWIRASYPRFRYDQLMHLLWKNFLPLTLALCTWHMTMPISLAGIPPQT

>Q89AU1

MTIIFVDNEEYNVDKSDNLLQACLSSGINIPYFCWHPVLGSIGSCRQCAVTIYKDLEDKVGQLVMSCMTSVLDGMIVSTSDKISRNFRKGIIELLMLNHPHDCPICEEGGSCHLQDMTVMAGHTVRRYRFTKRTHKNQYLGHFITHEMNRCISCYRCVRYYKDYSGGTDLGVFGISNNVYFGRYNDGCLESEFSGNLVEVCPTGVFTDKTYSKKYSRKWDMQYAPSICQHCCVGCNISVGEKYGKISRIENRYHNAINHYFLCDLGRFSYDYSNVDERLTYSIYRSQNKTKIINDVNKTIDKLAMKFKKSSKIIGIGSCRASVESNFSLQKLVGSENFYLGISQKEYDCLMLIKDILKDNQIHVPTLREIEKSDVIFLLGEDVTKTSPLIALSIRQSIKGQVKTQDVSKNIPIWHADAVKNSFRNNKNKLFITNLMNSSLDDIADESYYASTFDQVLLGAEVYKCISNNCISNVTLLKQDLLSCAKRIATALTLSKCPLIISGSHSYNLDLIKVSFNIAKSLKVIGKNVGLILLSSNVNSIGVSLLEGISIEKVINKVLLKQIDKIIVLENDLYRYLPESIVDTLFKSSSCTVVIDHLNTRTLKQADIAIPTCNSFESSGTVVNYEGRAQRFFKTYHPNSSENKKSILESWKWLHLLYCKLHKISVFWHSLDDVIEEISLKIFSFSKLKDVAPNSSFKIFGQKLARSHHRASGRTALYSNINIHEPRPPQDNDTMFSFSMEGCQNVQNYLPYVPFSWFPGWNSVQSWNTYKKINNENYGKHLFQDTTKFVLTYYKLNCKNVNKIEDLYLIVPCYFLFCNNELAQYSPVIQENVLKNAYGIINTEDAKVLLIESGSKIEFSYLNKNFSIKVQLSKEFKKGQLGLPLGMADFPFFLAEKQVKVFRKISI

>Q6NK10

MVIGVLTLQGGFAEHIAILESLGVEHRRVRVPNDLLGLDGLIIPGGESTVMDKLARAFDLAEPLRAAINNGLPVFATCAGLIYLGTVENPAKGQQTLGCLDVVVRRNAFGRQVDSFDAVVDVEGIDANVAFIRAPEVISCGAGVTVTARVGDHVVGVRQGKIHAYAFHPESAGEVRLHQAWLASI

>Q00645

MRFTPLFLLAAVAIASPAPDLNARHELTRRQASESCPIGYCTQNGGTTGGAAGDTVTVTNLADLTEAAESDGPLTIIVSGSISGSAKIRVASDKTIFGESGSSITGIGFYIRRVSNVIMRNLKISKVDADNGDAIGIDASSNVWVDHCDLSGDLSGGKDDLDGLVDISHGAEWITVSNTYFHDHWKGSLIGHSDNNEDEDLGHLHVTYANNYWYNVYSRTPLIRFATVHIINNYWDSLIDTGVNCRMDAQVLIQSSAFHNCPDRAIFFADSDYTGYAVVDDVDLGGSSNSVPEGTLTPSSLPYAAITALGSGQVASVIPGTAGQKL

>Q52982

MRTWFPYPLLSIALLLMWLLLSQSVTPGSIVLGLVVSTVLAWVTLNLQPARSRLHRWSRIAGFILRVVGDVIRSNIAVTLIILRAGRRPVNAGFMTVSLDLDDENALALLACVVTATPGTAWLEYDRRQKILLFHVLDIENEDLWRKTITRYAADLKEIFE

>Q02180

MLDAFSRSVVSADAKTAPVGGSDLAGLRSYVRDGNKRLDAVNAITSNASCIVSDAVTGMICENTGLIQAGGNCYPNRRMAACLRDGEIVLRYISYALLAGDASVLDDRCLNGLKETYIALGVPTQSAGRAVAIMKASATAHIGETNTPGLGGKRFRKMETTQGDCAALVAEAGAYFDRVIGAIS

>Q99X73

MSQIEFKNVSKVYPNGHVGLKNINLNIEKGEFAVIVGLSGAGKSTLLRSVNRLHDITSGEIFIQGKSITKAHGKALLEMRRNIGMIFQHFNLVKRSSVLRNVLSGRVGYHPTWKMVLGLFPKEDKIKAMDALERVNILDKYNQRSDELSGGQQQRISIARALCQESEIILADEPVASLDPLTTKQVMDDLRKINQELGITILINLHFVDLAKEYGTRIIGLRDGEVVYDGPASEATDDVFSEIYGRTIKEDEKLGVN

>P23545

MNKYRVRLFSVFVVCMILVFCVLGLFLQQLFETSDQRKAEEHIEKEAKYLASLLDAGNLNNQANEKIIKDAGGALDVSASVIDTDGKVLYGSNGRSADSQKVQALVSGHEGILSTTDNKLYYGLSLRSEGEKTGYVLLSASEKSDGLKGELWGMLTASLCTAFIVIVYFYSSMTSRYKRSIESATNVATELSKGNYDARTYGGYIRRSDKLGHAMNSLAIDLMEMTRTQEMQRDRLLTVIENIGSGLIMIDGRGFINLVNRSYAKQFHINPNHMLRRLYHDAFEHEEVIQLVEDIFMTETKKCKLLRLPIKIERRYFEVDGVPIMGPDDEWKGIVLVFHDMTETKKLEQMRKDFVANVSHELKTPITSIKGFTETLLDGAMEDKEALSEFLSIILKESERLQSLVQDLLDLSKIEQQNFTLSIETFEPAKMLGEIETLLKHKADEKGISLHLNVPKDPQYVSGDPYRLKQVFLNLVNNALTYTPEGGSVAINVKPREKDIQIEVADSGIGIQKEEIPRIFERFYRVDKDRSRNSGGTGLGLAIVKHLIEAHEGKIDVTSELGRGTVFTVTLKRAAEKSA

>P11084

MAEISDLDRQIEQLLRCELIKESEVKALCAKAREILVEESNVQRVDSPVTVCGDIHGQFYDLKELFRVGGDVPETNYLFMGDFVDRGFYSVETFLLLLALKVRYPDRITLIRGNHESRQITQVYGFYDECLRKYGSVTVWRYCTEIFDYLSLSAIIDGKIFCVHGGLSPSIQTLDQIRTIDRKQEVPHDGPMCDLLWSDPEDTTGWGVSPRGAGYLFGSDVVAQFNAANDIDMICRAHQLVMEGYKWHFNETVLTVWSAPNYCYRCGNVAAILELDEHLQKDFIIFEAAPQETRGIPSKKPVADYFL

>P24555

MLPKAARIPHAMTLHGDTRIDNYYWLRDDTRSQPEVLDYLQQENSYGHRVMASQQALQDRILKEIIDRIPQREVSAPYIKNGYRYRHIYEPGCEYAIYQRQSAFSEEWDEWETLLDANKRAAHSEFYSMGGMAITPDNTIMALAEDFLSRRQYGIRFRNLETGNWYPELLDNVEPSFVWANDSWIFYYVRKHPVTLLPYQVWRHAIGTPASQDKLIYEEKDDTYYVSLHKTTSKHYVVIHLASATTSEVRLLDAEMADAEPFVFLPRRKDHEYSLDHYQHRFYLRSNRHGKNFGLYRTRMRDEQQWEELIPPRENIMLEGFTLFTDWLVVEERQRGLTSLRQINRKTREVIGIAFDDPAYVTWIAYNPEPETARLRYGYSSMTTPDTLFELDMDTGERRVLKQTEVPGFYAANYRSEHLWIVARDGVEVPVSLVYHRKHFRKGHNPLLVYGYGSYGASIDADFSFSRLSLLDRGFVYAIVHVRGGGELGQQWYEDGKFLKKKNTFNDYLDACDALLKLGYGSPSLCYAMGGSAGGMLMGVAINQRPELFHGVIAQVPFVDVVTTMLDESIPLTTGEFEEWGNPQDPQYYEYMKSYSPYDNVTAQAYPHLLVTTGLHDSQVQYWEPAKWVAKLRELKTDDHLLLLCTDMDSGHGGKSGRFKSYEGVAMEYAFLVALAQGTLPATPAD

>Q7WEU9

MSASASTAADFVRFALDEGVLRFGSFKVKSGRISPYFFNAGLFNSGRSVGALAGFYAQALVDSGVAFDMLFGPAYKGIPLATATSVALAGHRAMAGRDVPFAFNRKEAKDHGEGGTLVGAPLTGKVVIIDDVITAGTSVRESVEIIRAAGAEPAAVLIALDRMERAGPDDALSPHSAVQDVARTYGIPVVSIASLADIMTLLQDDAQFAEHREAVQAYRSKYGV

>P18631

MSNQMTDSTSAGSGTEHSVDTNTALKAGSPNDLKVSHEEDLNDLEKTAEETLQQKPAKEYIFVSLCCVMVAFGGFVFGWDTGTISGFVNQTDFLRRFGQEKADGSHYLSNVRTGLIVSIFNIGCAVGGIVLSNIGDRWGRRIGLITVIIIYVIGIIIQIASVDKWYQYFIGRIISGLGVGGITVLSPMLISETAPKHLRGTLVSCYQLMITFGIFLGYCTNYGTKNYSNSVQWRVPLGLCFAWAIFMVLGMMFVPESARFLVETDQIEEARKSLAKTNKVSIDDPVVKYELLKIQSSIELEKAAGNASWGELITGKPSMFRRTLMGIMIQSLQQLTGDNYFFYYGTTIFQSVGMDDSFETSIVLGIVNFASTFFALYTVDHFGRRNCLLYGCVGMVACYVVYASVGVTRLWPDGPDHPDISSKGAGNCMIVFACFYIFCFATTWAPIAYVVISESYPLRVKGKAMAIASASNWIWGFLIGFFTPFITSAIHFYYGYVFMGCMVFAFFYVYFFVPETKGLTLEEVNEMYSEGVLPWKSSSWVPSSRRGAEYDVDALQHDDKPWYKAML

>P29703

MEEYDYSDVKPLPIETDLQDELCRIMYTEDYKRLMGLARALISLNELSPRALQLTAEIIDVAPAFYTIWNYRFNIVRHMMSESEDTVLYLNKELDWLDEVTLNNPKNYQIWSYRQSLLKLHPSPSFKRELPILKLMIDDDSKNYHVWSYRKWCCLFFSDFQHELAYASDLIETDIYNNSAWTHRMFYWVNAKDVISKVELADELQFIMDKIQLVPQNISPWTYLRGFQELFHDRLQWDSKVVDFATTFIGDVLSLPIGSPEDLPEIESSYALEFLAYHWGADPCTRDNAVKAYSLLAIKYDPIRKNLWHHKINNLN

>Q24145

MSRDSDPMKWYHGNLSREAADELLKQGYEDGTFLVRESSTAAGDFVLSLLCQGEVCHYQVRRHGGEDAFFSIDDKVQTKILHGLDTLVDYYQQAANGLPTKLTVPLIRDLPPHNTRSHGVTNLLHRATSKNESKVVFELLKCGYRNFDAKNQDGQTALHLAALHSDEDILKHLLNAKVQVNSSDSFGCQPLHYAARSKPASFIRTLISAQANVQGRNIDNGYVPLHEAAKHGNLEAVQELLLAEAPPLPRTSSGEFPFDLAKEAGQTAVEEFLLNYKLPPANTTRDQWYHGTLTREEAVAILKKHAKELLAKQPEVDTSGCFLVRYSESPAASGLVLTLLCDQVVKNFRISQADLYQNGNKVQSGGSKFLYIDDGPYWPSVEHLIAHFMRFSYGLPVSLKYPVPPQPKPEVPSFATIPRSNMKPKAASPATPPTPVSPHSHHQHPHVPALTITKKKQKENSSSMFNTLRLTSPKKALFDMNSLRKNKSKGKRSDSESSVSGSLAGTEQELQAAAPMLKSLSFSTEFSTFNADGVTGSGAAAAGEVYNVPRNNTPIEIDLPPIAQKTEAEVEYFTKSDVAIERERAGQWIGNGYQPTMDVLSLLDQQIKAPAVARLNSLGPNASTESEMASYLHRKCSGTPSTPSATEVEAAKLRFFIEPEKLVLDREIGHGEFGSVHSGWLLRKSGAGEESRLEVAIKMLSDEHSNKQEFLREASVMMRLEHKCIVRLIGIAKGEMLMMVQELAPLGSMLQYILDHGHEITANAELKVWASQIACGMHYLESQHFVHRDLAARNILLTARHQAKISDFGMSRSLRPGSTEYQFTQGGRWPIRWYAPESFNLGIFSHASDVWSFGVTIWEMFSLGAPPYGEISNVDAIKLVDSGERLPQPNLCPAYIYAVMQSCWKERPKDRPTFVYLTEFFARDPDYQNLPELVQTVHI

>Q11201

ST3GMVTLRKRTLKVLTFLVLFIFLTSFFLNYSHTMVATTWFPKQMVLELSENLKRLIKHRPCTCTHCIGQRKLSAWFDERFNQTMQPLLTAQNALLEDDTYRWWLRLQREKKPNNLNDTIKELFRVVPGNVDPMLEKRSVGCRRCAVVGNSGNLRESSYGPEIDSHDFVLRMNKAPTAGFEADVGTKTTHHLVYPESFRELGDNVSMILVPFKTIDLEWVVSAITTGTISHTYIPVPAKIRVKQDKILIYHPAFIKYVFDNWLQGHGRYPSTGILSVIFSMHVCDEVDLYGFGADSKGNWHHYWENNPSAGAFRKTGVHDADFESNVTATLASINKIRIFKGR

>Q9I8X5

MSRPSSAGPCASKPCGKQKQPPPPPPHAPSLPATISGGPGASAPPAPTAAAITGPLSQQHQELTSLFECPVCFDYVLPPILQCQAGHLVCNQCRQKLSCCPTCRASLTPSIRNLAMEKVASAVLFPCKYASTGCSLSLHHTEKPEHEDICEYRPYSCPCPGASCKWQGSLENVMQHLTHSHKSITTLQGEDIVFLATDINLPGAVDWVMMQYCFNHHFMLVLEKQEKYEGHQQFFAIVLLIGTRKQAENYAYRLELNGNRRRLTWEATPRSIHDGVAAAIMNSDCLVFDTAIAHLFADNGNLGINVTISTCCP

>P56584

MSAPTLDVESPLAASTSSLRAMNMVSSHTTVAKDEIYDLLGIGFGPAHLALSISLRESSEANETNFKAHFLEKRGHFAWHPALLLPGSQLQVSPLKDLVTLRDPASTYSFYNYLHSHGRLARYINKEQGVPSRREWTSYLAWAARRMNQAVSYGQDVISIEPLALASASPDAKQDTVAVRPASAQEADSLCLYQVRIRDESTGHIVNRYARNLSVAVGGVPKLPPAFQAAWDEQQRAPHSIPRLVHSGFYIPSMLKLEPELHKAASLRHPDAAAQLDDSSRLRLAVIGAGQSSTEMFMNLHSRFPSAIVTMIFRASALVPSDDTGFVNSAAFDPERTDEFWQASETQRRKWLQEFKRTNYSVVRTDLLNELHDAMYDKYEVQLPEELQDPTEKQAGRMEMRRCTEVVEVTPLDDGIQLTMRDNLRNAKLETIRFDAVFLGTGFIRSPSKMRFLEQLKPFYPALDAEWMSRDTIAEEDEVSKSIDVEDEEVIERRREMLRGITRDYRLVPASAMQSMQFVVANLRRDQEAMLRRLLPSKRWLAKIRPRTYPKPRSTCWAATRQRTACRTVC

>Q9Z4N3

MKNIVLASLLGFGLISSAWATETVNIHERVNNAQAPAHQMQSAAAPVGIQGTAPRMAGMDQHEQAIIAHETMTNGSADAHQKMVESHQRMMGSQTVSPTGPSKSLAAMNEHERAAVAHEFMNNGQSGPHQAMAEAHRRMLSAG

>Q92581

MARRGWRRAPLRRGVGSSPRARRLMRPLWLLLAVGVFDWAGASDGGGGEARAMDEEIVSEKQAEESHRQDSANLLIFILLLTLTILTIWLFKHRRARFLHETGLAMIYGLLVGLVLRYGIHVPSDVNNVTLSCEVQSSPTTLLVTFDPEVFFNILLPPIIFYAGYSLKRRHFFRNLGSILAYAFLGTAISCFVIGSIMYGCVTLMKVTGQLAGDFYFTDCLLFGAIVSATDPVTVLAIFHELQVDVELYALLFGESVLNDAVAIVLSSSIVAYQPAGDNSHTFDVTAMFKSIGIFLGIFSGSFAMGAATGVVTALVTKFTKLREFQLLETGLFFLMSWSTFLLAEAWGFTGVVAVLFCGITQAHYTYNNLSTESQHRTKQLFELLNFLAENFIFSYMGLTLFTFQNHVFNPTFVVGAFVAIFLGRAANIYPLSLLLNLGRRSKIGSNFQHMMMFAGLRGAMAFALAIRDTATYARQMMFSTTLLIVFFTVWVFGGGTTAMLSCLHIRVGVDSDQEHLGVPENERRTTKAESAWLFRMWYNFDHNYLKPLLTHSGPPLTTTLPACCGPIARCLTSPQAYENQEQLKDDDSDLILNDGDISLTYGDSTVNTEPATSSAPRRFMGNSSEDALDRELAFGDHELVIRGTRLVLPMDDSEPPLNLLDNTRHGPA

>P56707

MSSPLITDFLHQAGRAAVIAGGLGTELQRHGADLNDPLWSAKCLLSCPHLIRQVHLDYLENGADIIITASYQATIQGFKAKGFSDEEGEALLRRSVEIAREARDLYYQRCAESSSDNGDDSRILKQRPILIAGSVGSYGAYLADGSEFSGNYGDAIKSETLKDFHRRKVQILADSGVDLLAFEAVPNKLEAQAYADLLEEENIITPAWFAFTSKDGNNVVSGDSIEECGSIAESCDKVVAVGINCTPPRFIHDLILLLKKVTAKPIVIYPNSGETYDAIRKEWGQNSGVTDEDFVSYVDKWCESGASLVGGCCRTTPDTIRGIYKILSSGQSPTFSAK

>Q29495

MSTPSVHCLKPSPLHLPSGIPGSPGRQRRHTLPANEFRCLTPEDAAGVFEIEREAFISVSGNCPLNLDEVQHFLTLCPELSLGWFVEGRLVAFIIGSLWDEERLTQESLALHRPRGHSAHLHALAVHRSFRQQGKGSVLLWRYLHHVGAQPAVRRAVLMCEDALVPFYQRFGFHPAGPCAIVVGSLTFTEMHCSLRGHAALRRNSDR

>Q59966

MSTSGTFFADNSQTIGKTPLVRLNRIVKGAPATVLAKIEGRNPAYSVKCRIGAAMIWDAEQRGLLGPGKELIEPTSGNTGIALAFVAAARGIPLTLTMPETMSLERRKLLAAYGAKLVLTEGVKGMTGAVRRAEDIAASDPDRYVLLQQFRNPANPAIHEQTTGPEIWEDTGGAIDILVSGVGTGGTITGVSRYIKQTQGKPILSVAVEPEASPVISQQRSGLPLKPGPHKIQGIGAGFIPENLDLSLVDQVERVSNEEAIAYARRLAQEEGLISGISCGAAVAAAVRLAQQSEHAGKTIVVVLPDSGERYLSTALFDGIFNEQGLAVV

>O83889

MALLDISSGNVRKTIETNPLVIVDFWAPWCGSCKMLGPVLEEVESEVGSGVVIGKLNVDDDQDLAVEFNVASIPTLIVFKDGKEVDRSIGFVDKSKILTLIQKNA

>P31549

MATRRQPLIPGWLIPGVSATTLVVAVALAAFLALWWNAPQDDWVAVWQDSYLWHVVRFSFWQAFLSALLSVIPAIFLARALYRRRFPGRLALLRLCAMTLILPVLVAVFGILSVYGRQGWLATLCQSLGLEWTFSPYGLQGILLAHVFFNLPMASRLLLQALENIPGEQRQLAAQLGMRSWHFFRFVEWPWLRRQIPPVAALIFMLCFASFATVLSLGGGPQATTIELAIYQALSYDYDPARAAMLALLQMVCCLGLVLLSQRLSKAIAPGTTLLQGWRDPDDRLHSRICDTVLIVLALLLLLPPLLAVIVDGVNRQLPEVLAQPVLWQALWTSLRIALAAGVLCVVLTMMLLWSSRELRARQKMLAGQVLEMSGMLILAMPGIVLATGFFLLLNNTIGLPQSADGIVIFTNALMAIPYALKVLENPMRDITARYSMLCQSLGIEGWSRLKVVELRALKRPLAQALAFACVLSIGDFGVVALFGNDDFRTLPFYLYQQIGSYRSQDGAVTALILLLLCFLLFTVIEKLPGRNVKTD

>P29363

MRYISTRGQAPALNFEDVLLAGLASDGGLYVPENLPRFTLEEIASWVGLPYHELAFRVMRPFVAGSIADADFKKILEETYGVFAHDAVAPLRQLNGNEWVLELFHGPTLAFKDFALQLLGRLLDHVLAKRGERVVIMGATSGDTGSAAIEGCRRCDNVDIFIMHPHNRVSEVQRRQMTTILGDNIHNIAIEGNFDDCQEMVKASFADQGFLKGTRLVAVNSINWARIMAQIVYYFHAALQLGAPHRSVAFSVPTGNFGDIFAGYLARNMGLPVSQLIVATNRNDILHRFMSGNRYDKDTLHPSLSPSMDIMVSSNFERLLFDLHGRNGKAVAELLDAFKASGKLSVEDQRWTEARKLFDSLAVSDEQTCETIAEVYRSSGELLDPHTAIGVRAARECRRSLSVPMVTLGTAHPVKFPEAVEKAGIGQAPALPAHLADLFEREERCTVLPNELAKVQAFVSQHGNRGKPL

>P52197

MDDFASLPLVIEPADLQARLSAPELILVDLTSAARYAEGHIPGARFVDPKRTQLGQPPAPGLQPPREQLESLFGELGHRPEAVYVVYDDEGGGWAGRFIWLLDVIGQQRYHYLNGGLTAWLAEDRPLSRELPAPAGGPVALSLHDEPTASRDYLLGRLGAADLAIWDARSPQEYRGEKVLAAKGGHIPGAVNFEWTAAMDPSRALRIRTDIAGRLEELGITPDKEIVTHCQTHHRSGLTYLIAKALGYPRVKGYAGSWGEWGNHPDTPVEL

>O86840

MTDIPADDPKIELRSDITVELVKSAATDSDVLFAARVSTAGEQSLDELKKDPERSKGLINYLMRDRHGSPFEHNSMTFFVSAPIFVFREFMRHRVGWSYNEESGRYRELQPVFYAPDASRKLVQQGRPGKYVFVEGTPEQHELVGSAMEDSYRQAYATYQQMLAAGVAREVARAVLPVGLYSSMYATCNARSLMHFLGLRTQHELAKVPSFPQREIEMAGEKMEAEWARLMPLTHAAFNANGRVAP

>P50163

MAGRWNLEGCTALVTGGSRGIGYGIVEELASLGASVYTCSRNQKELNDCLTQWRSKGFKVEASVCDLSSRSERQELMNTVANHFHGKLNILVNNAGIVIYKEAKDYTVEDYSLIMSINFEAAYHLSVLAHPFLKASERGNVVFISSVSGALAVPYEAVYGATKGAMDQLTRCLAFEWAKDNIRVNGVGPGVIATSLVEMTIQDPEQKENLNKLIDRCALRRMGEPKELAAMVAFLCFPAASYVTGQIIYVDGGLMANCGF

>Q40287

MGSTDLNSKPHIVLLSSPGLGHLIPVLELGKRIVTLCNFDVTIFMVGSDTSAAEPQVLRSAMTPKLCEIIQLPPPNISCLIDPEATVCTRLFVLMREIRPAFRAAVSALKFRPAAIIVDLFGTESLEVAKELGIAKYVYIASNAWFLALTIYVPILDKEVEGEFVLQKEPMKIPGCRPVRTEEVVDPMLDRTNQQYSEYFRLGIEIPTADGILMNTWEALEPTTFGALRDVKFLGRVAKVPVFPIGPLRRQAGPCGSNCELLDWLDQQPKESVVYVSFGSGGTLSLEQMIELAWGLERSQQRFIWVVRQPTVKTGDAAFFTQGDGADDMSGYFPEGFLTRIQNVGLVVPQWSPQIHIMSHPSVGVFLSHCGWNSVLESITAGVPIIAWPIYAEQRMNATLLTEELGVAVRPKNLPAKEVVKREEIERMIRRIMVDEEGSEIRKRVRELKDSGEKALNEGGSSFNYMSALGNEWEKSWKTQRSERSLW

>P50047

MSFKMDREEYAQHYGPTVGDSVRLGDTNLFAAIEKDFTVYGQESKFGGGKVLRDGMGVSATETRDNPSVVDTIITGATIIDYTGIIKADIGIRDGKIVAIGRGGNPDTMDNVDFVVGASTEAIAAEGLIVTAGGIDLHVHYISADLPEFGLDNGITTLFGGGTGPADGSNATTCTPGKFHITRMLQAVDDMPANFGFLAKGVGSETEVVEEQIKAGAAGIKTHEDWGATYAGIDNSLKVADKYDVSFAVHTDSLNEGGFMENTLESFQGRTVHTFHTEGSGGGHAPDIMVFAGKENILPSSTNPINPYTTNAIGELLDMVMVCHHLDPKIPEDVSFAESRVRKQTVAAEDVLHDMGALSIMTSDAMAMGRVGEVAMRCWQLADKMKAQRGPLEGDSEFNDNNRIKRYVAKYTINPAITNGIADYIGSVEVGKFADLVIWEPAQFGAKPKLVLKGGMLTYGVMGDAGSSLPTPQPRIMRKLYGAYGQAVHETNLTFVSQYAYDHGIKEEIGLNKIVLPVKNTRNLTKRDMKLNDYAPKTIRIDPQTFDVFIDDELVTCEPIHTTSLSQRYFLF

>P53241

MTISNKSWRSYFPHLRKLPEDDQYLYSDDTNSSIIAEEELHHSVDKSSKTDVTAETTAVEPHPHNLRHDLPYEVRDEAGRKWWKYFDEFEYRVNKEYKKSRKWYEFLYPNHTTQTKAERRLLYKLDIIIALYFFMLCWSKSVDLNNYTNAYVSNMKEDLNMKGNDYVYTSTIANVGAIVFQLPFMYLLPRFPSHIILPVMDLGWTWFTFACYRANSLAELRAYRFILSAFGAAYYPVSQYILGCWYAPDEINSRVCLFFCGQQLGSVTSGLLQSRIFKSLNGVHGLAGWRWMFLIDAIAISLPTAIIGFFVIPGVPSKCYSLFLTDEEIRIARARNKRNQIKDGVDKSKLAPLWSRKLWKKVFCTPAFWVLVVFDTCSWNNMTAYSGSYTLWLKSNTKYSIAQVNNLSVIPACLGFAYVIFCAFGADLFRCKWIFMVFAAIMNTVSCALLIKWDIPSKAKWYAFFTTYFSVAASPCLWSFINDFLRFDPQVKAITWIAIYSFSQSTYAWIPTLAWPTVESPRFKTGYTVSLIFGAIYGLWTFVVLFFYKRNEKKHALGNGIILYDSNKGEELPEFVKKNMEERDGYYYLKRSS

>P48789

MRKLTQFCLGLMLLPIAAVAQNQPTMKDVLGKYFLVGTALNSHQIWTHDPKIVHAITDNFNSVVAENCMKGEIIHPEEDYYDWHDADQLVKFAEQHKMTVHGHCLVWHSQAPKWMFTDKEGKEVTREVLIDRMYHHITNVVKRYKGKIKGWDVVNEAILDNGEYRQSPYYKIIGPDFIKLAFIFAHQADPDAELYYNDYSMSIPAKRNAVVKLVKELKAAGCRIDAVGMQSHNGFNYPNLEDYENSIKAFIAAGVDVQFTELDVNMLPNPKSFGGAEISQNYKYNKELNPYVNGLTKAAQKTFDQQYLSFFKIYRKYVDHIKRVTVWGVDDGSSWLNGWPVPGRTNYGLLIDRNYKVKPVVKEIIKLYE

>P77337

MSDDKFDAIVVGAGVAGSVAALVMARAGLDVLVIERGDSAGCKNMTGGRLYAHTLEAIIPGFAVSAPVERKVTREKISFLTEESAVTLDFHREQPDVPQHASYTVLRNRLDPWLMEQAEQAGAQFIPGVRVDALVREGNKVTGVQAGDDILEANVVILADGVNSMLGRSLGMVPASDPHHYAVGVKEVIGLTPEQINDRFNITGEEGAAWLFAGSPSDGLMGGGFLYTNKDSISLGLVCGLGDIAHAQKSVPQMLEDFKQHPAIRPLISGGKLLEYSAHMVPEGGLAMVPQLVNEGVMIVGDAAGFCLNLGFTVRGMDLAIASAQAAATTVIAAKERADFSASSLAQYKRELEQSCVMRDMQHFRKIPALMENPRLFSQYPRMVADIMNEMFTIDGKPNQPVRKMIMGHAKKIGLINLLKDGIKGATAL

>Q8DRY7

MSQHLFTAFILTALAGLSTGIGSLIAFVTKHTNKTFLSVSLGFSAGVMIYVSMIEIFPTAQTILTKAMDKKSGSWLTVLAFFGGILLIAIIDKLIPSEENPHEIKTIEEEDQKPTKLMRMGLMTAIAIGIHNFPEGLATFISGLQDASIAIPIVIAIAIHNIPEGIAVSVPIYQATGSKKKAFTYSFLSGLAEPLGAIIGWFLLMPIMNNIVYGAIFSAVAGIMVFISLDELLPAAEEYGKHHLAIYGVISGMLIMAVSLLLFI
