# Supplementary material for: enDNA-Prot: Identification of DNA-Binding Proteins by Applying Ensemble Learning
Source: Biomed Res Int. 2014 May 26;2014:294279. doi: 10.1155/2014/294279 (PMC4058174; doi:10.1155/2014/294279)
Supplement: Supplementary file 1 — Supplementary Material S1 lists all the codes and sequences for the benchmark dataset. It contains 396 proteins, classified into 146 DNA-binding proteins and 250 non DNA-binding proteins. Supplementary Material S2 lists all the codes and sequences for the expanded benchmark dataset. It contains 2271 proteins, classified into 146 DNA-binding proteins and 2125 non DNA-binding proteins. Supplementary Material S3 lists all the codes and sequences for the independent dataset1. It contains 182 proteins, classified into 82 DNA-binding proteins and 100 non DNA-binding proteins. Supplementary Material S4 lists all the codes and sequences for the independent dataset2. It contains 1585 proteins, classified into 770 DNA-binding proteins and 815 non DNA-binding proteins. [file 294279.f1.zip › 294279.f1/S4.docx]

Online Supporting Information S4. The sequences in independent dataset2 are shown here. It contains 770 DNA-binding protein sequences and 815 non DNA-binding proteins sequences.

|  |
| --- |

(1). DNA-binding proteins

>ASCL3_MOUSE/94-145 PF00010

IRKRNERERQRVKCVNEGYARLRRHLPEDYLEKRLSKVETLRAAIKYISYLQ

>FIGLA_MOUSE/60-112 PF00010

RRRVANAKERERIKNLNRGFAKLKALVPFLPQSRKPSKVDILKGATEYIQILG

>HLH4_CAEEL/6-57 PF00010

AKRNARERTRVHTVNQAFLVLKQHLPSLRQFTKRVSKLRILNAAITYIDTLL

>ID1_HUMAN/56-106 PF00010

LLDEQQVNVLLYDMNGCYSRLKELVPTLPQNRKVSKVEILQHVIDYIRDLQ

>ID2_HUMAN/24-76 PF00010

RSKTPVDDPMSLLYNMNDCYSKLKELVPSIPQNKKVSKMEILQHVIDYILDLQ

>ID4_HUMAN/53-105 PF00010

AEAAADEPALCLQCDMNDCYSRLRRLVPTIPPNKKVSKVEILQHVIDYILDLQ

>NUC1_NEUCR/668-758 PF00010

KRTSHKIAEQGRRNRINSALQEIATLLPKAPAKEGGDGDGDGHSSSGGGGGSGGADREDKREKDKDKAGGGIPNSKASTVEMAIEYIKQLQ

>O42447_ONCMY/31-83 PF00010

KIPMLDEQMSVFLQDMNSCYSKLKELVPTLPANKKASKMEILQHVIDYIWDLQ

>O76488_JUNCO/77-138 PF00010

ARRNARERNRVKQVNDGFNALRRHLPASVVAALSGGARRGSGKKLSKVDTLRMVVEYIRYLQ

>Q9W7E6_ORYLA/51-103 PF00010

KREMVNAKERLRIRNLNTMFSRLKRMLPLMQPDKKPSKVDTLKAATEYIRLLL

>ATF1_HUMAN/211-271 PF00170

DPQLKREIRLMKNREAARECRRKKKEYVKCLENRVAVLENQNKTLIEELKTLKDLYSNKSV

>ATF4_HUMAN/276-340 PF00170

EKLDKKLKKMEQNKTAATRYRQKKRAEQEALTGECKELEKKNEALKERADSLAKEIQYLKDLIEE

>BZLF1_EBV/170-231 PF00170

ECDSELEIKRYKNRVASRKCRAKFKQLLQHYREVAAAKSSENDRLRLLLKQMCPSLDVDSII

>CPC1_NEUCR/214-270 PF00170

DPSDVVAMKRARNTLAARKSRERKAQRLEELEAKIEELIAERDRWKNLALAHGASTE

>CPRF1_PETCR/267-331 PF00170

DRDLKRERRKQSNRESARRSRLRKQAEAEELAIKVDSLTAENMALKAEINRLTLTAEKLTNDNSR

>CPRF3_PETCR/194-258 PF00170

ERELKRQRRKQSNRESARRSRLRKQAKSDELQERLDNLSKENRILRKNLQRISEACAEVTSENHS

>CREB2_BOVIN/265-325 PF00170

EAARKREVRLMKNREAARECRRKKKEYVKCLENRVAVLENQNKTLIEELKALKDLYCHKSD

>CREBA_DROME/439-503 PF00170

EKSLKKIRRKIKNKISAQESRRKKKEYMDQLERRVEILVTENHDYKKRLEGLEETNANLLSQLHK

>CREM_HUMAN/272-332 PF00170

EATRKRELRLMKNREAAKECRRRKKEYVKCLESRVAVLEVQNKKLIEELETLKDICSPKTD

>EMBP1_WHEAT/248-312 PF00170

ERELKRERRKQSNRESARRSRLRKQQECEELAQKVSELTAANGTLRSELDQLKKDCKTMETENKK

>GBF1_ARATH/220-284 PF00170

ERELKRQKRKQSNRESARRSRLRKQAECEQLQQRVESLSNENQSLRDELQRLSSECDKLKSENNS

>GBF2_ARATH/247-311 PF00170

EKEVKREKRKQSNRESARRSRLRKQAETEQLSVKVDALVAENMSLRSKLGQLNNESEKLRLENEA

>GBF3_ARATH/257-321 PF00170

ERELKRERRKQSNRESARRSRLRKQAETEELARKVEALTAENMALRSELNQLNEKSDKLRGANAT

>GBF4_ARATH/185-249 PF00170

KAAAQRQKRMIKNRESAARSRERKQAYQVELETLAAKLEEENEQLLKEIEESTKERYKKLMEVLI

>GCN4_YEAST/223-280 PF00170

ESSDPAALKRARNTEAARRSRARKLQRMKQLEDKVEELLSKNYHLENEVARLKKLVGE

>HBP1A_WHEAT/250-314 PF00170

ERELKKQKRKLSNRESARRSRLRKQAECEELGQRAEALKSENSSLRIELDRIKKEYEELLSKNTS

>JRA_DROME/210-274 PF00170

QEKIKLERKRQRNRVAASKCRKRKLERISKLEDRVKVLKGENVDLASIVKNLKDHVAQLKQQVME

>JUNB_HUMAN/266-330 PF00170

QERIKVERKRLRNRLAATKCRKRKLERIARLEDKVKTLKAENAGLSSTAGLLREQVAQLKQKVMT

>JUN_CHICK/233-297 PF00170

QERIKAERKRMRNRIAASKCRKRKLERIARLEEKVKTLKAQNSELASTANMLREQVAQLKQKVMN

>TAF1_TOBAC/192-256 PF00170

ERELKREKRKQSNRESARRSRLRKQAEAEELAIRVQSLTAENMTLKSEINKLMENSEKLKLENAA

>YAP4_YEAST/235-295 PF00170

GKPLRNTKRAAQNRSAQKAFRQRREKYIKNLEEKSKLFDGLMKENSELKKMIESLKSKLKE

>YEN5_YEAST/382-446 PF00170

AKAWKRARLLERNRIAASKCRQRKKMSQLQLQREFDQISKENTMMKKKIENYEKLVQKMKKISRL

>DBHA_PSEAE/1-90 PF00216

MRKPELAAAIAEKADLTKEQANRVLNALLDEITGALNRKDSVTLVGFGTFLQRHRGARTGKNPQTGQPVKIKASNTVAFKPGKALRDAVN

>DBH_AQUAE/1-94 PF00216

MTKKEIAEEIYDRLIRDYNVKIQKKEVYNLVSEVFTIIQECLLKGEKVKISGFGTFVVKTRKPKKGMNIKKREIVEVPERKIVLFKPSKNFIKS

>DBH_BORAF/9-104 PF00216

VTKSDIVDQISLNIRNNNLKLEKKYIRLVIDAFFEELKSNLCSNNVIEFRSFGTFEVRKRKGRLNARNPQTGEYVKVLDHHVAYFRPGKDLKERVW

>DBH_CHLTR/4-94 PF00216

MTKKKLISTISQDHKIHPNHVRTVIQNFLDKMTDALVQGDRLEFRDFGVLQVVERKPKVGRNPKNAAVPIHIPARRAVKFTPGKRMKRLIE

>DBH_GUITH/1-90 PF00216

MNKSQLISKIAYYTKYSKTDIEKIITSMLEIIVDTVATGEKVTLVGFGSFEARERKAREGRNPRTGEKLFLPASRIPTFSVGNFFRNKVN

>DBH_HELPY/1-91 PF00216

MNKAEFIDLVKEAGKYNSKREAEEAISAFTLAVETALSKGESVELIGFGKFETAEQKGKEGKVPGSDKTYKTEDKRVPKFKPGKTLKQKVE

>DBH_RICPR/1-99 PF00216

MNKTEFIAFMTEHGHNNKHAAHKTLTKADAEKALNLVIESVISAIKSKHNVNLTGFGSFEIHHRKEREGRNPKTGAKMKIDAYNQPTFRAGRKLKEACN

>DBH_THEMA/1-90 PF00216

MTKKELIDRVAKKAGAKKKDVKLILDTILETITEALAKGEKVQIVGFGSFEVRKAAARKGVNPQTRKPITIPERKVPKFKPGKALKEKVK

>DBH_TREPA/6-95 PF00216

RTRSFVVDALCDEVDLSRRHVARVVDSFVSVVTAALERGETVELRDFGVFESRVRKASVGKSIKTGEVVSIPSHCVVVFRPSKRLKSAVR

>DBH_UREPA/8-97 PF00216

KTRSQMIDELSKMLNIEKKQTKAFMDTYEAFLILELSRAKEVRLGNIGKFKVSVRAERKGINPKTGETVIIPEKTIPKFTFTKGIKEIIN

>HLIK_ASFM2/9-101 PF00216

ITKQELYSLVAADTQLNKALIERIFTSQQKIIQNALKHNQEVIIPPGIKFTVVTVKAKPARQGHNPATGEPIQIKAKPEHKAVKIRALKPVHD

>O67923_AQUAE/1-90 PF00216

MTKSDIAKELARRHGISYKKALLIVNMTFEILKAKILNGEKVEVRGLGTFKLKRKPGRFVKNPKTGIEIYVKERYVPYYKMSKLLRKKLN

>O85384_LISMO/2-83 PF00216

ANKTDLVNSVAELADLSKKTQRKQQLYSKLFKLLHLLGFGNFEVRERAARKGRNPATKEEIDIPASKVPAFKPGKALKEAVK

>Q49060_MYCCA/1-89 PF00216

MLLEELYEDPEFVEMTKVSIKTIFDKAFIVASKNLAAGDHVVLAKFGKFETIEKEAQMKMNPSTQEEIEVPAHKVVKFKLSKSIKEKMN

>Q49504_MYCGA/7-96 PF00216

LSAAEYLKEMADETNLKVQDIRLVVTSLQKVLAKELATTGEVRLFDIGKFKLVATKPRTGINPKTKQKIQIPAGKKIKLTVSKILTDAVD

>Q97CE1_THEVO/2-90 PF00216

VGISELSKDVAKKANTTQKVARTVIKSFLDEIVAQANGGQKINLAGFGIFERRTQGPRKARNPQTKKVIEVPSKKKFVFRASSKIKYQQ

>Q97EA3_CLOAB/1-90 PF00216

MNKAELITSISEKSKLTKKDAEVALKAFIESVEETLEKHEKVQLVGFGTFETRERAERKGRNPRSKEEIVIPASTVPVFKAGKEFKERVN

>Q98RC2_MYCPU/36-125 PF00216

MTKKEFMQKISEHSFISQRDVEAIINSMVFVIKESLISEEKVTIPMLGTFETKIKAAREGIKFTTGEKILIPEKRVVKFKPTKYIKEVVN

>Q9CH59_LACLA/2-91 PF00216

ASKQELIDYVADRTELSKVNAAKAINAFVEAITYYISEGTPVHISNFGTFEVRKRAARVSHDVHSREKILVGEQNIPVFRAGKALKLATK

>Q9ZCL7_RICPR/3-91 PF00216

ITKNKISLMLSSKLGFSNNLCEAIVNTVFSNILEIAKVQKLTLKNFGSFEVKQKTPRPGINFHTKSPVMIASKKNLRFTPSEKLKALIN

>Q9ZHZ9_MYCHY/7-96 PF00216

MNKKELIEQIAIKTNLPIKNIELVLNEFFGITADVVKKQGKLVINSFGTFQGVFKPASSSFNPLTKTQIQVNAKTTVRFKPSKVLKDFIA

>TF1_BPSP1/1-90 PF00216

MNKTELIKAIAQDTELTQVSVSKMLASFEKITTETVAKGDKVQLTGFLNIKPVARQARKGFNPQTQEALEIAPSVGVSVKPGESLKKAAE

>ADA2_HUMAN/72-118 PF00249

DPSWTAQEEMALLEAVMDCGFGNWQDVANQMCTKTKEECEKHYMKHF

>ARR11_ARATH/195-246 PF00249

RVVWSFELHHKFVNAVNQIGCDHKAGPKKILDLMNVPWLTRENVASHLQKYR

>ARR18_ARATH/196-246 PF00249

RVVWSQELHQKFVSAVQQLGLDKAVPKKILDLMSIEGLTRENVASHLQKYR

>ARR20_ARATH/213-268 PF00249

RMQWTPELHHKFEVAVEKMGSLEKAFPKTILKYMQEELNVQGLTRNNVASHLQKYR

>MYBF_ARATH/25-76 PF00249

RLRWTPELHRSFVHAVDLLGGQYKATPKLVLKIMDVKGLTISHVKSHLQMYR

>NCOR1_HUMAN/625-670 PF00249

TSRWTEEEMEVAKKGLVEHGRNWAAIAKMVGTKSEAQCKNFYFNYK

>O04322_ARATH/118-165 PF00249

GVPWTEEEHRLFLVGLQKLGKGDWRGISRNYVTSRTPTQVASHAQKYF

>O04605_ARATH/59-104 PF00249

RENWTDQEHDKFLEALHLFDRDWKKIEAFVGSKTVVQIRSHAQKYF

>O23486_ARATH/88-134 PF00249

CPDWSADDEMLLLEGLEIYGLGNWAEVAEHVGTKSKEQCLEHYRNIY

>O81713_ARATH/24-69 PF00249

RERWTEDEHERFLEALRLYGRAWQRIEEHIGTKTAVQIRSHAQKFF

>O94481_SCHPO/365-410 PF00249

PEKWNAMDTEKFYKALSQWGTDFALIANMFPTRNRRQIKLKFKQEE

>Q15044_HUMAN/294-339 PF00249

NARWTTEEQLLAVQAIRKYGRDFQAISDVIGNKSVVQVKNFFVNYR

>Q42575_ARATH/107-152 PF00249

RNSFTEVEDQAIIAAHAIHGNKWAVIAKLLPGRTDNAIKNHWNSAL

>Q9FG27_ARATH/193-244 PF00249

RIRWTQDLHEKFVECVNRLGGADKATPKAILKRMDSDGLTIFHVKSHLQKYR

>Q9FH42_ARATH/25-76 PF00249

RLRWTADLHDRFVDAVAKLGGADKATPKSVLKLMGLKGLTLYHLKSHLQKYR

>Q9FJV5_ARATH/107-158 PF00249

RMRWTSTLHAHFVHAVQLLGGHERATPKSVLELMNVKDLTLAHVKSHLQMYR

>Q9FK47_ARATH/47-98 PF00249

RLKWTPDLHERFVEAVNQLGGGDKATPKTIMKVMGIPGLTLYHLKSHLQKYR

>Q9LRN5_ARATH/43-110 PF00249

RLRWTTELHERFVDAVTQLGGPDSEYYFVYLSLCSVSLLEATPKTIMRTMGVKGLTLYHLKSHLQKFR

>Q9LSH8_ARATH/243-294 PF00249

RMRWTPELHESFVKAVIKLEGPEKATPKAVKKLMNVEGLTIYHVKSHLQKYR

>Q9LTH4_ARATH/143-193 PF00249

RLVWTPQLHKRFVDVVAHLGIKNAVPKTIMQLMNVEGLTRENVASHLQKYR

>Q9M0H0_ARATH/227-278 PF00249

RMRWTPELHEAFVEAVNSLGGSERATPKGVLKIMKVEGLTIYHVKSHLQKYR

>Q9S807_CHLRE/189-240 PF00249

RLRWTPELHNRFVNAVNSLGGPDKATPKGILKLMGVDGLTIYHIKSHLQKYR

>Q9SIZ5_ARATH/84-135 PF00249

RLRWTPELHICFLQAVERLGGPDRATPKLVLQLMNVKGLSIAHVKSHLQMYR

>Q9SJW0_ARATH/17-68 PF00249

RLRWTHELHERFVDAVAQLGGPDRATPKGVLRVMGVQGLTIYHVKSHLQKYR

>Q9SVP8_ARATH/39-98 PF00249

RLRWTSELHERFVDAVTQLGGPDISTKNLTTKATPKTIMRTMGVKGLTLYHLKSHLQVLM

>Q9XEB2_ARATH/16-67 PF00249

RMHWTDDLDIRFIQVIEKLGGEESATPKRILSLMGVRDLTISHVKSHLQMYR

>Q9ZPZ8_ARATH/88-139 PF00249

RLRWSSDLHDCFVNAVEKLGGPNKATPKSVKEAMEVEGIALHHVKSHLQKFR

>TFC5_YEAST/417-462 PF00249

TDPWTVEEMIKFYKALSMWGTDFNLISQLYPYRSRKQVKAKFVNEE

>CSDE1_RAT/25-90 PF00313

RETGVIEKLLTSYGFIQCSERQARLFFHCSQYNGNLQDLKVGDDVEFEVSSDRRTGKPIAIKLVKI

>CSDE1_RAT/673-738 PF00313

LRRATVECVKDQFGFINYEVGDSKKLFFHVKEVQDGIELQAGDEVEFSVILNQRTGKCSACNVWRV

>CSP_AQUAE/2-68 PF00313

SFRGTVKWFSKDKGYGFITREDTNADVFVHFTDIQMEGFKTLQKGQKVEFDVVEDTKGPRAKNVRVL

>O46173_DROME/62-131 PF00313

KVTGTVKWFNVKSGYGFINRNDTREDVFVHQSAIANNPKKAVRSVGDGEVVEFDVVIGEKGNEAANVTGP

>O62213_CAEEL/20-90 PF00313

KVKGTVKWFNVKNGYGFINRTDTNEDIFVHQTAIINNNPNKYLRSLGDNEEVMFDIVEGSKGLEAASVTGP

>P91398_CAEEL/63-134 PF00313

GLQGKVKWYSVLRRYGFISRSDGEKDVFVHQTAISKSDTEKFYLRTLADEEEVLFDLVDGKNGPEAANVTGP

>P91602_CAEVU/52-120 PF00313

RYFGSCKWFNVSKGYGFVIDDITREDLFVHQSNLNMQGFRSLDEGERVSYYIQERSNGKGREAYAVSGE

>Q27277_SCHMA/25-95 PF00313

RVKGVVKWFNVKAGYGFINRQDTSTDIFVHQSAISRNNPEKLQRSLQEGEEVEFYVVEGDKGDEASEVTGP

>MCM1_YEAST/24-74 PF00319

KFIENKTRRHVTFSKRKHGIMKKAFELSVLTGTQVLLLVVSETGLVYTFST

>Q9FKQ5_ARATH/4-54 PF00319

ASSSSSSRNSTSLTNRLKTIFKKAEELSILCAIEVCVIYYGPDGELRTWPK

>SRF_HUMAN/149-199 PF00319

EFIDNKLRRYTTFSKRKTGIMKKAYELSTLTGTQVLLLVASETGHVYTFAT

>O51971_HALSA/5-90 PF00352

KDTITIENVVASTAIEQELDLSRVAMDLEGADYDPEQFPGLVYRLDEPSVVALLFGSGKLVITGGKHPVDAEHAVDTIDSRLEDLG

>O74068_CERSY/97-181 PF00352

CTRPVVRNMVATVDAGRTVPIDRISSRIPGAVYDPGSFPGMILKGLGSCSFLVFASGKVVIAGARSPGELYRSSFDLLARLNGAG

>P90869_CAEEL/262-346 PF00352

DIDIQIRNVVCNYTLPLHIDLRKLAMNTHNVTYEREKGVMMKQKRSPGCYIKVYSSGKVYIVGCRSEADCKRAARSIARHVQRVM

>P90869_CAEEL/355-441 PF00352

IRNYRVNNVLATCRLPFGIKIEEVAAKYPSESTYEPELSVGLVWRSVTPKATLRIHTTGSITVTGAQSEADVLEVLSKIYPIVLEFR

>Q07450_ONCVO/92-178 PF00352

FTEFKVQNMVGSCDVRFPIQLEGLCLTHTQFSTYEPELFPGLIYRMVKPRVVLLIFVSGKVVITGAKYKKDIDDAFNQIYPILKGFK

>Q8T052_DROME/288-375 PF00352

FLNFRIVNVLGTCSMPWAIKIVNFSERHRENASYEPELHPGVTYKMRDPDPKATLKIFSTGSVTVTAASVNHVESAIQHIYPLVFDFR

>Q9BIE4_LEIDO/195-279 PF00352

FPVVVAVQAQASIPVGINLAELSCATRNVEYMPNNRIPPATMRLHEPTAVVMMHNSGALSIIGAASVSEARQAAELAARIIRKAL

>Q9U7A4_ANTLO/171-257 PF00352

FADFKIQNIVSSCDTQFSIRLEGLAFAHSNFCSYEPELFPGLIYRMVKPKIVLLIFVSGKIVLTGAKMRDEIYEAFDNIYPVLTQYK

>Q9XG30_GUITH/161-247 PF00352

FIDFRIQNIVASCDVRFPIRLESLAHAHNQFCSYEPELFPGLIYRMITPKVVLLIFVSGKLVLTGAKQRNDIFQAFSNIYSVLCLYK

>Q9XZP5_BRUMA/139-225 PF00352

IRNYRVCNVLATCKMPFGVKIEELAQKYPDCSQYEPELSVGLIWRSTNPRATLRIHTTGSITVTGAASESDVMKAVEVIYPIVKEFR

>Q9XZP5_BRUMA/46-130 PF00352

CFEFQIRNVVCNYTLPLHIDLHRVALNSGNVAFDRGRGVLLKQKRNPSCYVKIYSSGKIYIVGCRSESECKRAARGVARMVQKTM

>TBP_AERPE/6-91 PF00352

KPEVKIENIVATVILENQLDLNLIETKIQDVDYNPDQFPGLVYRLESPRVTVLIFKSGKMVITGAKSINQLIHVVKKLLKAFADQG

>TBP_ARCFU/3-88 PF00352

DYKIKIENVVASTQIGENIDLNKISREIKDSEYKPKQFPGLVLRTKEPKAAALVFRSGKVVCTGSKSVEDARRAVKQIVKMLKEIG

>TBP_ARCFU/94-181 PF00352

EPEVKVQNIVASADLGVDLNLNAIAIGLGLENIEYEPEQFPGLVYRLDNPRVVVLIFGSGKMVVTGGKSPEDARKAVERISEELRTLG

>TBPB_HALSA/96-183 PF00352

NPPIEVQNIVSSASLEQSLNLNAIAIGLGLEQIEYEPEQFPGLVYRLDDPDVVVLLFGSGKLVITGGQNPDEAEQALAHVQDRLTELG

>TBPC_HALSA/1-87 PF00352

MTVEIANIVGSGDLGVELDVEPLEADLSTPYSEYDPSNYHGLYVRLEENGPLITVYRSGKYIITGCASMESLHETNEEFLGMLADLG

>TBPC_HALSA/94-181 PF00352

QTGFTVENVVCTAMLDELVSLNALAIGLGLEVTEYEPEQFPGLVYRPEEIGAVLLVFANGKMVITGAKDTETAESAYEYFQSKVQELV

>TBPD_HALSA/5-90 PF00352

TDTIQIENVVASTDLSQELALEQLATDLPGAEYNPGDFPGVIYRLDDPKSATLIFDSGKAVCTGAQSVDDVHDAISIVVEDLRDLG

>TBPD_HALSA/96-183 PF00352

SPPVHVQNIVCSGSLDQDLNLNAIAIGLGLEDVEYEPEQFPGLVYRLNDPDVVVLLFGSGKLVITGGSNPDDAHHALEIIHERLTDLG

>TBP_DICDI/112-198 PF00352

FTDFKIQNIVGSCDVKFPIKLELLHNAHTSFTNYEPEIFPGLIYKMIQPKVLLLIFVSGKIVLTGAKVREYIYEAFENIYPVLSAFK

>TBP_DROVI/260-346 PF00352

FLDLKIQNMVGLCDVKFPIRLEGLVLTHCNFSRYEPELFPGLIYRMVRPRIVLLLPAPGKVVLTGAKVRQEIYDAFDKIFSILKKFK

>TBPE_HALSA/96-183 PF00352

DPEIVVQNIVTSADLGRQLNLNAIAIGLGLENIEYEPEQFPGLVYRLDDPEVVALLFGSGKLVITGGKEPKDAEHAVDKITSRLEELG

>TBP_ENTHI/141-227 PF00352

YSNFNVQNIVGSCDVKFQIALRTLVDSYLAFCQYEPEVFPGLVYRMASPKVTLLVFSTGKVVLTGAKDEESLNLAYKNIYPILLANR

>TBPF_HALSA/5-90 PF00352

ADTIHIENVVASSDLGQELALDQLATDLDGAEYNPEDFPGVVYRLQEPKSATLIFRSGKVVCTGAKSVDAVHDALEIVFDDLRELG

>TBPL1_HUMAN/8-92 PF00352

ALDILITNVVCVFRTRCHLNLRKIALEGANVIYKRDVGKVLMKLRKPRITATIWSSGKIICTGATSEEEAKFGARRLARSLQKLG

>TBPL1_HUMAN/97-182 PF00352

FTDFKVVNVLAVCNMPFEIRLPEFTKNNRPHASYEPELHPAVCYRIKSLRATLQIFSTGSITVTGPNVKAVATAVEQIYPFVFESR

>TBP_METJA/96-181 PF00352

NPEIKIQNMVATADLGIEPNLDDIALMVEGTEYEPEQFPGLVYRLDDPKVVVLIFGSGKVVITGLKSEEDAKRALKKILDTIKEVQ

>TBP_METTL/93-177 PF00352

NPEVNVQNMVATADLGIEPNLDDISTLEGTEYEPEQFPGLVYRLSDPKVVVLIFGSGKVVITGLKKKDDAYLALDKILSTLKELE

>TBP_MOUSE/227-313 PF00352

FLDFKIQNMVGSCDVKFPIRLEGLVLTHQQFSSYEPELFPGLIYRMIKPRIVLLIFVSGKVVLTGAKVRAEIYEAFENIYPILKGFR

>TBP_PLAFA/138-227 PF00352

FCNFKIENIIASANCNIPIRLEVLAHDHKEYCNYEPELFAGLVYRYKPTSNLKSVILIFVSGKIIITGCKSVNKLYTVFQDIYNVLIQYK

>TBP_PLAFA/47-132 PF00352

NISLNIHNIISSANLCIDINLRLVAVSIRNAEYNPSKINTLIIRLNKPQCTALIFKNGRIMLTGTRTKKDSIMGCKKIAKIIKIVT

>TBP_PYROC/109-194 PF00352

RPKVQIQNIVASANLNVCVDLERAALTLENSMYEPEQFPGLIHRMDEPRVVLLIFSSGKMVITGAKREEEVYEAVNKIYEKLKKLR

>TBP_SOLTU/110-196 PF00352

FKDFKIQNIVGSCDVKFPIRLEGLAYAHGAFSSYEPELFPGLIYRMKQPKIVLLIFVSGKIVITGAKVRDETYTAFENIYPVLTEFR

>TBP_SULSH/100-185 PF00352

KPKIQIQNIVASANLHVNVNLDKAAFLLENNMYEPEQFPGLIFRMDDPRVVLLIFSSGKMVITGAKREDEVSKAVKRIFDKLAELD

>TBP_TETTH/138-224 PF00352

FKDFKIQNIVGSTDVKFPINLDHLEQDHKKFVQYEPEIFPGKIYREFNTKIVLLIFVSGKIVLTGAKTRENINKAFQKIYWVLYNYQ

>TBP_THEAC/4-89 PF00352

REKITIENIVASTSLAEHLDLSRIALALDGSEYEPEQFPGLIYRLQEPKTAVLIFRSGKVNCTGAKNIEDVKRTIKIIIDKLKAAD

>TRF_DROME/136-222 PF00352

FMEYKLQNIVATVDLRFPIRLENLNHVHGQFSSYEPEMFPGLIYRMVKPRIVLLIFVNGKVVFTGAKSRKDIMDCLEAISPILLSFR

>TF2B_DROME/120-190 PF00382

ISSMADRINLPKTIVDRANNLFKQVHDGKNLKGRSNDAKASACLYIACRQEGVPRTFKEICAVSKISKKEI

>TF2B_DROME/214-284 PF00382

MCRFCANLDLPNMVQRAATHIAKKAVEMDIVPGRSPISVAAAAIYMASQASEHKRSQKEIGDIAGVADVTI

>TF2B_KLULA/137-207 PF00382

ITMMCDAAELPKIVKDCAKEAYKLCFEERVLKGKSQESIMASVILVGCRRAEVGRSFKEILSLTNVRKKEI

>TF2B_YEAST/133-203 PF00382

ITMLCDAAELPKIVKDCAKEAYKLCHDEKTLKGKSMESIMAASILIGCRRAEVARTFKEIQSLIHVKTKEF

>TF3B_CANAL/190-263 PF00382

IQHFVEKLDFKDKATKVAKDAVKLAHRMAADWIHEGRRPAGIAGACVLLAARMNNFRRSHAEIVAVSHVGEETL

>TF3B_CANAL/95-165 PF00382

IKRIAAALKIPDYIAEAAGEWFRLALTLNFVQGRRSNNVLATCLYVACRKERTHHMLIDFSSRLQISVYSL

>TF3B_KLULA/193-266 PF00382

IQHFAEKLELGDKKIKVIRDAVKLAQTMSRDWMYEGRRPAGIAGACLLLACRMNNLRRTHSEIVAISHVAEETL

>TF3B_KLULA/98-168 PF00382

LKAVSYALNIPEYVTDAAFQWYRLALSNNFVQGRKSQNVIAACLYIACRKERTHHMLIDFSSRLQVSVYSI

>HSF1_ARATH/51-234 PF00447

PPFLSKTYDMVEDPATDAIVSWSPTNNSFIVWDPPEFSRDLLPKYFKHNNFSSFVRQLNTYGFRKVDPDRWEFANEGFLRGQKHLLKKISRRKSVQGHGSSSSNPQSQQLSQGQGSMAALSSCVEVGKFGLEEEVEQLKRDKNVLMQELVKLRQQQQTTDNKLQVLVKHLQVMEQRQQQIMSFL

>HSF1_CHICK/21-216 PF00447

SAFLTKLWTLVEDPETDPLICWSPSGNSFHVFDQGQFAKEVLPKYFKHNNMASFVRQLNMYGFRKVVHIEQGGLVKPEKDDTEFQHPYFIRGQEHLLENIKRKVTSVSSIKNEDIKVRQDNVTKLLTDIQVMKGKQESMDSKLIAMKHENEALWREVASLRQKHAQQQKVVNKLIQFLISLVQSNRILGVKRKIPL

>HSF2_CHICK/22-214 PF00447

PAFLSKLWALVGEAPSNQLITWSQNGQSFLVLDEQRFAKEILPKYFKHNNMASFVRQLNMYGFRKVVHVDSGIVKLERDGLVEFQHPYFKQGREDLLEHIKRKVSSSRPEENKISQEDLSKIISSAQKVEIKQETIESRLSALKRENESLWREVAELRAKHLKQQQVIRKIVQFIVTLVQNNQLVSLKRKRPL

>HSF30_LYCPE/30-197 PF00447

PPFLSKTYEMVEDSSTDQVISWSTTRNSFIVWDSHKFSTTLLPRFFKHSNFSSFIRQLNTYGFRKVDPDRWEFANEGFLGGQKHLLKTIKRRRNVGQSMNQQGSGACIEIGYYGMEEELERLKRDKNVLMTEIVKLRQQQQSTRNQIIAMGEKIETQERKQVQMMSFL

>HSF3_CHICK/17-209 PF00447

PGFLAKLWALVEDPQSDDVICWSRNGENFCILDEQRFAKELLPKYFKHNNISSFIRQLNMYGFRKVVALENGMITAEKNSVIEFQHPFFKQGNAHLLENIKRKVSAVRTEDLKVCAEDLHKVLSEVQEMREQQNNMDIRLANMKRENKALWKEVAVLRQKHSQQQKLLSKILQFILSLMRGNYIVGVKRKRSL

>HSF8_LYCPE/40-225 PF00447

PPFLVKTYDMVDDPSTDKIVSWSPTNNSFVVWDPPEFAKDLLPKYFKHNNFSSFVRQLNTYGFRKVDPDRWEFANEGFLRGQKHLLKSISRRKPAHGHAQQQQQPHGHAQQQMQPPGHSASVGACVEVGKFGLEEEVERLKRDKNVLMQELVRLRQQQQSTDNQLQGMVQRLQGMELRQQQMMSFL

>HSF_DROME/47-241 PF00447

PAFLAKLWRLVDDADTNRLICWTKDGQSFVIQNQAQFAKELLPLNYKHNNMASFIRQLNMYGFHKITSIDNGGLRFDRDEIEFSHPFFKRNSPFLLDQIKRKISNNKNGDDKGVLKPEAMSKILTDVKVMRGRQDNLDSRFSAMKQENEVLWREIASLRQKHAKQQQIVNKLIQFLITIVQPSRNMSGVKRHVQL

>HSF_KLULA/195-394 PF00447

PAFVNKLWSMVNDKSNEKFIHWSTSGESIVVPNRERFVQEVLPKYFKHSNFASFVRQLNMYGWHKVQDVKSGSMLSNNDSRWEFENENFKRGKEYLLENIVRQKSNTNILGGTTNAEVDIHILLNELETVKYNQLAIAEDLKRITKDNEMLWKENMMARERHQSQQQVLEKLLRFLSSVFGPNSAKTIGNGFQPDLIHEL

>HSF_SCHPO/51-248 PF00447

TQFSNKLYNMVNDSSTDSLIRWSDRGDSFLVIGHEDFAKLVLPRYFKHNNFSSFVRQLNMYGFHKVPHIQQGVLQSDSPNELLEFANPNFQRDQPELLCLVTRKKAGSQPVEESNTSLDMSTISSELQNIRIQQMNLSNELSRIQVDNAALWQENMENRERQRRHQETIDKILRFLASVYLDGKQKPPSKVMPKSRRL

>SKN7_YEAST/83-283 PF00447

PANEFVRKLFRILENNEYPDIVTWTENGKSFVVLDTGKFTTHILPNHFKHSNFASFVRQLNKYDFHKVKRSPEERQRCKYGEQSWEFQHPEFRVHYGKGLDNIKRKIPAQRKVLLDESQKALLHFNSEGTNPNNPSGSLLNESTTELLLSNTVSKDAFGNLRRRVDKLQKELDMSKMESYATKVELQKLNSKYNTVIESLI

>O09242_9DELA/922-973 PF00552

APANWYYVLTPGLTNQRWKGPLHLSRKLQERLLSIDGSPQWIPWRLLKKTVC

>O11404_9PLVG/986-1036 PF00552

FKNFRVYYREGRDQLWKGPGELLWKGEGAVILKVGTDIKVVPRRKAKIIKD

>O12274_9DELA/821-873 PF00552

TTPKWYYYKIPGLTSSRWSGPVQSLKEAAGAALIPVGGSHLWIPWRLLKRGVC

>O39643_ALV/789-842 PF00552

VLTEGPPVKIRIETGEWEKGWNVLVWGRGYAAVKNRDTDKVIWVPSRKVKPDIT

>O56220_9BETR/1648-1702 PF00552

SADPKPMVMWKDLLTGSWKGPDVLITAGRGYACVFPQDAETPIWVPDRFIRPFTE

>O56224_MPMV/1711-1765 PF00552

PKKQFAMVKWKDPLDNTWHGPDPVLIWGRGSVCVYSQTYDAARWLPERLVRQVSN

>O56228_9DELA/1390-1443 PF00552

KQTHWYYFKLPGLNSRQWKGPQEALQEAAGAALIPVSASSAQWIPWRLLKRAAC

>O70641_9DELA/1396-1448 PF00552

APTNWYYYKLPGLTNQRWKGPLQSLQEAAGAALLSIDGSPQWIPWRLLKKTVC

>O90273_SIVCZ/966-1016 PF00552

ILKFKVYYRQGRDPQWKGPAQLLWKGEGAVVVKEGENIFSVPRRKAKLVKD

>O92812_BLV/1350-1401 PF00552

GSDKLFLYKLPGQNNRRWLGPLPALVEASGGALLATNPPVWVPWRLLKAFKC

>P90246_9RETR/1091-1142 PF00552

IKNSWIYYKDRRDKEWKGPTQVEYWGQGAVLIKHPEHGYMLIPRRHIRRVPE

>POL_BIV06/997-1046 PF00552

KIEKWCYVRNRRKEWKGPYKVLWDGDGAAVIEEEGKTALYPHRHMRFIPP

>POL_CAEVC/1048-1103 PF00552

QKIQFCYYRIRKRGHQESGKDQPRYCGKGKEPIVVKDIESEKYLVIPYKDAKFIPP

>POL_IPHA/755-809 PF00552

PDRPKEMVKWKDVLTDLWKGPDPILIRSRGAVCVFPQEEENPLWIPERLTRRAPS

>POL_IPMA/802-862 PF00552

PDRPNEMVKWKNVLHNKWYGPDPILIRSRGAVCVFHRMKTTHFGYQKDSPEKSRLTKGIPD

>POL_JSRV/804-858 PF00552

SSCKKPLVRWKDPLTNLWYGPDPVLIWGRGHVCVFPQDAEAPRWIPERLVRAAEE

>POL_OMVVS/1024-1079 PF00552

SKFRFCYYRVRKRGHPGEWLGPTQVLWEGEGAIVIKDKNLEKYLVIAKKDVKFIPQ

>POL_SIVGB/944-994 PF00552

NLNFKVYFREGRDQLWKGPGILLWKGEGAVVLKYQEEIKIVPRRKCKIIKD

>POL_SIVVT/990-1040 PF00552

ILNFRVYYREGRDPVWKGPAQLIWKGEGAVVLKDGSDLKVVPRRKAKIIKD

>POL_SMRVH/812-866 PF00552

TSQQHATVMWRDPLTSVWKGPDPVLIWGRGSACIYDQKEDGPRWLPERLIRHINN

>POL_VILV/1043-1098 PF00552

EKIRFCYYRTRKRGHPGEWQGPTQVLWGGDGAIVVKDRGTDRYLVIANKDVKFIPP

>Q03261_SIVCZ/963-1013 PF00552

FSNFRVYYRQGKNEWKGPARLLWKGEGAVVVQTEEGDIFAVPRRKAKIITD

>Q82851_9RETR/1373-1422 PF00552

KTEKWAYVRDKRKVWKGPYKVLWDGEGAAVVEENAMPTLYPHRHMRFIPP

>Q84809_9RETR/1015-1066 PF00552

LKDSWIYYKDRKDKLWKGPTQVYYWGEGAVLIKDENNKYLLIPRRRIRRVPA

>Q8AGY0_9RETR/1546-1596 PF00552

HQRPLVYYRCLPDPAWRGPAQLITWGRGYAAIQLPDKVLWVPGRCVRPCHL

>Q8C2B1_MOUSE/722-776 PF00552

PDRPNEMVKWKNVLDNKWYGPDPILIRSRGAICVFPQNEDNPFWVPERLTRKIQT

>Q8J4V1_9RETR/1060-1115 PF00552

SKIRFCYYRVRKKGHPGDWQGPTQVLWEGEGAIVVKDKPTERYFVVTNKDVRFIPP

>Q8J8R9_9RETR/24-75 PF00552

SSKKFCFYKIPGEHDWKGPTRVLWKGDGAVVVNDERKGIIAVPLTRTKLLIK

>Q8JAI0_SIVCZ/939-990 PF00552

FQKFRVYYRQGANPHWQGPAVLLWKGEGAVVVQTQAGEIITVPRRKAKIIKP

>Q8N9I4_HUMAN/35-86 PF00552

REGKLIWWKDNKNKIWEIGKVIIWGRGCACVSPGENQLPVWIPTRHLKFYNE

>Q8NFP1_HUMAN/800-852 PF00552

ESIQWIMWKDLQDNQWKGPFPLLRRIRGAVCFFPQGAAQPIWTPERRIRLVNL

>Q8Q5Q7_9RETR/1070-1121 PF00552

LQSQWIYYKDQKDKNWKGPMRVEYWGQGSVLLKDEERGYFLVPRRHIRRVPE

>Q8Q6V8_9HIV1/220-270 PF00552

IQNFRVYYRDSRDPIWKGPAQLLWKGEGAVVIQDKGEIKVVPRRKAKIIRD

>Q90PX4_SIVCZ/953-1003 PF00552

IQNFRVYFREGRDQQWKGPAKLIWEGEGAVVIQDQEDLKVVPRRKCKIIKD

>Q99FI1_SIVCZ/914-964 PF00552

FKNFKAYWKEHTGEWQGPGELVWKGEGAVVIRNSQGTLFVKPRRKVKITRL

>Q9DKV8_CAEV/1046-1101 PF00552

IQKKFCYYRIRKRGHPGEWNGPTEVLWEGEGAIVVKDKESDRYLVIPYKDAKFIPP

>Q9GLF8_TRIVU/118-172 PF00552

SAHDYAQVKWKDPLTHQWHGPDPVLIWGKGHACIYDSNAQNARWLPERLLKLVDH

>IRF1_HUMAN/5-114 PF00605

RMRMRPWLEMQINSNQIPGLIWINKEEMIFQIPWKHAAKHGWDINKDACLFRSWAIHTGRYKAGEKEPDPKTWKANFRCAMNSLPDIEEVKDQSRNKGSSAVRVYRMLPP

>IRTF_HUMAN/10-117 PF00605

RKLRNWVVEQVESGQFPGVCWDDTAKTMFRIPWKHAGKQDFREDQDAAFFKAWAIFKGKYKEGDTGGPAVWKTRLRCALNKSSEFKEVPERGRMDVAEPYKVYQLLPP

>ACE1_YEAST/1-40 PF00649

MVVINGVKYACETCIRGHRAAQCTHTDGPLQMIRRKGRPS

>AMT1_CANGA/1-40 PF00649

MVVINGVKYACDSCIKSHKAAQCEHNDRPLKILKPRGRPP

>CRF1_YARLI/1-40 PF00649

MVVIEGIKYACERCIRGHRVSSCTHTQQPLIRIKPKGRPA

>CUF1_SCHPO/1-40 PF00649

MVVINNVKMACMKCIRGHRSSTCKHNDRELFPIRPKGRPI

>CUF2_SCHPO/1-40 PF00649

MIIIDGKNYACVVCLRGHRGSSCQHQERALIEVRTRGRPL

>HAA1_YEAST/1-40 PF00649

MVLINGIKYACERCIRGHRVTTCNHTDQPLMMIKPKGRPS

>MAC1_YEAST/1-40 PF00649

MIIFNGNKYACASCIRGHRSSTCRHSHRMLIKVRTRGRPS

>Q7S636_NEUCR/1-41 PF00649

MPIINGQKMACAPCIRGHRSTKCTHASERVMVPVRKPGRPL

>Q7S6K5_NEUCR/1-39 PF00649

MLINGEKWACEACVRGHRVSNCQHHDRPLQHINKKGRPV

>DNBI_HHV11/1-1147 PF00747

METKPKTATTIKVPPGPLGYVYARACPSEGIELLALLSARSGDSDVAVAPLVVGLTVESGFEANVAVVVGSRTTGLGGTAVSLKLTPSHYSSSVYVFHGGRHLDPSTQAPNLTRLCERARRHFGFSDYTPRPGDLKHETTGEALCERLGLDPDRALLYLVVTEGFKEAVCINNTFLHLGGSDKVTIGGAEVHRIPVYPLQLFMPDFSRVIAEPFNANHRSIGEKFTYPLPFFNRPLNRLLFEAVVGPAAVALRCRNVDAVARAAAHLAFDENHEGAALPADITFTAFEASQGKTPRGGRDGGGKGAAGGFEQRLASVMAGDAALALESIVSMAVFDEPPTDISAWPLFEGQDTAAARANAVGAYLARAAGLVGAMVFSTNSALHLTEVDDAGPADPKDHSKPSFYRFFLVPGTHVAANPQVDREGHVVPGFEGRPTAPLVGGTQEFAGEHLAMLCGFSPALLAKMLFYLERCDGAVIVGRQEMDVFRYVADSNQTDVPCNLCTFDTRHACVHTTLMRLRARHPKFASAARGAIGVFGTMNSMYSDCDVLGNYAAFSALKRADGSETARTIMQETYRAATERVMAELETLQYVDQAVPTAMGRLETIITNREALHTVVNNVRQVVDREVEQLMRNLVEGRNFKFRDGLGEANHAMSLTLDPYACGPCPLLQLLGRRSNLAVYQDLALSQCHGVFAGQSVEGRNFRNQFQPVLRRRVMDMFNNGFLSAKTLTVALSEGAAICAPSLTAGQTAPAESSFEGDVARVTLGFPKELRVKSRVLFAGASANASEAAKARVASLQSAYQKPDKRVDILLGPLGFLLKQFHAAIFPNGKPPGSNQPNPQWFWTALQRNQLPARLLSREDIETIAFIKKFSLDYGAINFINLAPNNVSELAMYYMANQILRYCDHSTYFINTLTAIIAGSRRPPSVQAAAAWSAQGGAGLEAGARALMDAVDAHPGAWTSMFASCNLLRPVMAARPMVVLGLSISKYYGMAGNDRVFQAGNWASLMGGKNACPLLIFDRTRKFVLACPRAGFVCAASSLGGGAHESSLCEQLRGIISEGGAAVASSVFVATVKSLGPRTQQLQIEDWLALLEDEYLSEEMMELTARALERGNGEWSTDAALEVAHEAEALVSQLGNAGEVFNFGDF

>DSX_DROME/40-86 PF00751

TPPNCARCRNHGLKITLKGHKRYCKFRYCTCEKCRLTADRQRVMALQ

>MAB3_CAEEL/24-70 PF00751

KNYYCQRCLNHGELKPRKGHKPDCRYLKCPCRECTMVEQRRQLNNLL

>MAB3_CAEEL/90-137 PF00751

RDPHCARCSAHGVLVPLRGHKRTMCQFVTCECTLCTLVEHRRNLMAAQ

>O01582_CAEEL/57-103 PF00751

RTLFCRKCEGHGQQVVLKGHASRCPFNNCSCKTCTNVMSMRANAIIR

>O62159_CAEEL/41-87 PF00751

RILYCRKCEGHGEKVILKNHSPQCPYILCNCKSCEKLNYKRLKSFNK

>Q18435_CAEEL/39-85 PF00751

KIYYCQRCLNHDVPRPRKNHKCECPYADCTCEKCGLVEKRRILNIRL

>Q18436_CAEEL/36-83 PF00751

RVPNCQKCGQHGRKSRLKGHKRSCPFRECPCAKVCVFSNKRSSRVSLE

>Q19291_CAEEL/29-75 PF00751

RTPKCARCRNHGTTSALKGHKRYCQWKDCMCAKCTLIAERQRVMAAQ

>O17072_CAEEL/108-174 PF00808

MSVPMARVKKIMRIDDDVRNFMIASDAPIFMAQAAEFFIEEMTAMGWQYVSEARRRILQKADIASAV

>O73745_XENLA/39-103 PF00808

QDLPLARIKKIMKLDEDVKMISAEAPVLFAKAAQIFITELTLRAWIHTEDNKRRTLQRNDIAMAI

>Q9GSP1_DROME/10-74 PF00808

ARFPAGRIKKIMQSDEEIGKVAQAVPVIISRTLELFVESLLTKTLRITNARNAKTLSPSHMRQCI

>Q9LHG0_ARATH/7-71 PF00808

TRFPAARIKKIMQADEDVGKIALAVPVLVSKSLELFLQDLCDRTYEITLERGAKTVSSLHLKHCV

>Q9LN09_ARATH/108-172 PF00808

IKFPMNRIRRIMRSDNSAPQIMQDAVFLVNKATEMFIERFSEEAYDSSVKDKKKFIHYKHLSSVV

>Q9V452_DROME/18-83 PF00808

TFLPLSRVRTIMKSSMDTGLITNEVLFLMTKCTELFVRHLAGAAYTEEFGQRPGEALKYEHLSQVV

>P53_ORYLA/80-270 PF00870

TTVPVTTDYPGSYELELRFQKSGTAKSVTSTYSETLNKLYCQLAKTSPIEVRVSKEPPKGAILRATAVYKKTEHVADVVRRCPHHQNEDSVEHRSHLIRVEGSQLAQYFEDPYTKRQSVTVPYEPPQPGSEMTTILLSYMCNSSCMGGMNRRPILTILTLETEGLVLGRRCFEVRICACPGRDRKTEEESR

>P73_HUMAN/113-309 PF00870

PVIPSNTDYPGPHHFEVTFQQSSTAKSATWTYSPLLKKLYCQIAKTCPIQIKVSTPPPPGTAIRAMPVYKKAEHVTDVVKRCPNHELGRDFNEGQSAPASHLIRVEGNNLSQYVDDPVTGRQSVVVPYEPPQVGTEFTTILYNFMCNSSCVGGMNRRPILIIITLEMRDGQVLGRRSFEGRICACPGRDRKADEDHY

>Q27937_LOLFO/120-314 PF00870

PSVPSNIKYPGEYVFEMSFAQPSKETKSTTWTYSEKLDKLYVRMATTCPVRFKTARPPPSGCQIRAMPIYMKPEHVQEVVKRCPNHATAKEHNEKHPAPLHIVRCEHKLAKYHEDKYSGRQSVLIPHEMPQAGSEWVVNLYQFMCLGSCVGGPNRRPIQLVFTLEKDNQVLGRRAVEVRICACPGRDRKADEKAS

>ADAB_BACSU/88-173 PF01035

GTQFQLAVWNALCEIPYGQTKSYSDIANDINKPAAVRAVGAAIGANPVLITVPCHRVIGKNGSLTGYRGGFEMKTLLLDLEKRASS

>MGMT_CRIGR/96-182 PF01035

DSFTRQVLWKLLKVVKFGEMVSYQQLAALAGNPKAARAVGGAMRNNPVPILIPCHRVICSNGSIGNYSGGGQAVKEWLLAHEGIPTR

>MGMT_HUMAN/92-177 PF01035

ESFTRQVLWKLLKVVKFGEVISYQQLAALAGNPKAARAVGGAMRGNPVPILIPCHRVVCSSGAVGNYSGGLAVKEWLLAHEGHRLG

>MGMT_YEAST/116-201 PF01035

GTDFQRKVWNELLNVEHGHVVTYGDIAKRIGKPTAARSVGRACGSNNLALLVPCHRIVGSNRKLTGYKWSCKLKEQLLNNEKENSL

>O25387_HELPY/74-159 PF01035

GTFFQKQVWSALMTIPYGKTKSYDEIAKLINNPRSCRAVGNANRNNPISLIVPCHRVVRKNGTLGGYNGGIEVKKWLLEFESKILN

>OGT_ARCFU/61-144 PF01035

ASSFTRRVLEEVSRIPYGMVRMYSDIAKALNTSPRAVGQAVKRNPLPVIIPCHRVVGKKEIGGYTVSCSDIDGKSLKKRLLRLE

>OGT_METJA/77-159 PF01035

VPEFTKKVLDIVKDIEFGKTLTYGDIAKKLNTSPRAVGMALKRNPLPLIIPCHRVVAKNSLGGYSYGLDKKKFILERERLNMV

>OGT_MYCLE/73-158 PF01035

GTEFQQRVWQALLTIPYGETRSYGEIAEQVGAPGAARAVGLANSRNPIAIIVPCHRVIGASGQLIGYGGGLNRKLTLLELEKHQVL

>OGT_SHIFL/86-171 PF01035

GTPFQREVWKTLRTIPCGQVMHYGQLAEQLGRPGAARAVGAANGSNPISIVVPCHRVIGRNGTMTGYAGGVQRKEWLLRHEGYLLL

>Q50855_MYXXA/37-125 PF01035

PLPFSKAVWKAVRAIPRGQVRSYAQVALYAGRPGAARGVGREMATLPQQPELPLPWWRVTRSDGTLAPQVAQEQARRLRAEGVEVTQRG

>Q84444_PBCV1/141-179 PF01096

LQCGKCKSRKTSYYEMQTRSADEPMTVFAKCHSCGSRWK

>RPO5_VARV/157-195 PF01096

TPCPNCKSRNTTPMMIQTRAADEPPLVRHACRDCKQHFK

>RPOM_SULAC/70-108 PF01096

ISCPSCGNDEAYFWILQTRSADEPATRFYKCTKCGKVWR

>TCEA1_HUMAN/261-299 PF01096

FTCGKCKKKNCTYTQVQTRSADEPMTTFVVCNECGNRWK

>TFS2_ASFB7/204-242 PF01096

YKCPNCKQRMCTYREVQTRALDEPSTIFCTCKKCGHEFI

>TFS2_YEAST/269-307 PF01096

FTCGKCKEKKVSYYQLQTRSADEPLTTFCTCEACGNRWK

>IF5A1_CAEEL/87-156 PF01287

VVKRREYILMSIEDGFCSLMDPESCELKDDLKMPEGDLGNTIREALEKDEGSVLVQVVAACGEEAILGYK

>IF5A1_CHICK/83-150 PF01287

NIKRNDYQLIGIQDGYLSLLTESGEVREDLKLPEGDLGKEIEGKFNANEDVQISVISAMNEECAVAIK

>IF5A1_TOBAC/85-154 PF01287

HVNRTDYQLIDISEDGFVSLLTENGNTKDDLRLPTDDNLLALIKDGFAEGKDLVLSVMSAMGEEQICGIK

>IF5A1_YEAST/83-150 PF01287

FVKRSEYQLLDIDDGYLSLMTMDGETKDDVKAPEGELGDSMQAAFDEGKDLMVTIISAMGEEAAISFK

>IF5A_DICDI/98-165 PF01287

NVSRKEYTVMDVQDGYLSLLDAGGEVKEDLALPEDDIGKEITQMLKEGKEPLVSVISALGKEGVVSVK

>IF5A_METJA/70-129 PF01287

IIDRRKGQVLAIMGDMVQIMDLQTYETLELPIPEGIEGLEPGGEVEYIEAVGQYKITRVI

>IF5A_NEUCR/90-157 PF01287

NVKRTDYQFSYIDEDFLVLIDSNGEEKRELKMPEGELAKRIEKLEEEGKDFFVGVQTAMGEEAAIDVK

>IF5A_SULAC/69-131 PF01287

IIEKHVGQILADKGDNLTIMDLESYETFDLEKPTENEIVSKIRPGAEIEYWSVMGRRKIVRVK

>ARGR1_CLOPE/4-73 PF01316

LEKSLRHEVILDIIESKCICKQEELIIELKECGINVTQATLSRDLHEMNIIRKSFENHEHRYIVTKEDKF

>ARGR_BACLD/1-70 PF01316

MNKGQRHIKIREIITANEIETQDELVDILKKDGYNVTQATVSRDIKELHLVKVPTNNGSYKYSLPADQRF

>ARGR_BACST/1-70 PF01316

MNKGQRHIKIREIIMSNDIETQDELVDRLREAGFNVTQATVSRDIKEMQLVKVPMANGRYKYSLPSDQRF

>ARGR_CHLPN/2-70 PF01316

KKKVTIDEALKEILRLEGAATQEELCAKLLAQGFATTQSSVSRWLRKIQAVKVAGERGARYSLPSSTEK

>ARGR_CORGL/16-85 PF01316

VTRTARQALILQILDKQKVTSQVQLSELLLDEGIDITQATLSRDLDELGARKVRPDGGRAYYAVGPVDSI

>ARGR_DEIRA/6-75 PF01316

IGKDQRQKRIQDIILRESVSTQAELVKLLAKEGVQVTQATVSRDINELRLVRVPIGKGRHRYALAQYGGD

>ARGR_MYCTU/16-86 PF01316

ANRAGRQARIVAILSSAQVRSQNELAALLAAEGIEVTQATLSRDLEELGAVKLRGADGGTGIYVVPEDGSP

>ARGR_SHIFL/5-75 PF01316

AKQEELVKAFKALLKEEKFSSQGEIVAALQEQGFDNINQSKVSRMLTKFGAVRTRNAKMEMVYCLPAELGV

>ARGR_STRR6/1-69 PF01316

MRKRDRHQLIKKMITEEKLSTQKEIQDRLEAHNVCVTQTTLSRDLREIGLTKVKKNDMVYYVLVNETEK

>ARGR_THEMA/3-72 PF01316

ISKKRRQELIRKIIHEKKISNQFQIVEELKKYGIKAVQPTVARDLKEIGAVKIMDESGNYVYKLLDETPV

>DTXR_CORDI/3-63 PF01325

DLVDTTEMYLRTIYELEEEGVTPLRARIAERLEQSGPTVSQTVARMERDGLVVVASDRSLQ

>MNTR_BACSU/1-61 PF01325

MTTPSMEDYIEQIYMLIEEKGYARVSDIAEALAVHPSSVTKMVQKLDKDEYLIYEKYRGLV

>O26316_METTH/6-66 PF01325

HLSENIEEYLETIYRLSDSRKPVTTTDISREMKIAPASVTQMLKKLDSNGYVKYSPYRGAV

>O27019_METTH/11-70 PF01325

ELTRSMENYLETLYRLSSHGIVKTSDLSSRMNVRPASVTQMLKRLHEMGLVIYHPYLGAE

>O28295_ARCFU/7-68 PF01325

KFVTKTEVVLRTVYTALEKGLAVIGPTQVAEELGVSKSTAQKMLNELSKAGYGVYVPKKGLV

>O28489_ARCFU/1-60 PF01325

MERIEEYLEAIYDIQEETSKVAKTGELAKILNVKPSSVTEMLIKLRDMGYVDYQPYKGAK

>O30276_ARCFU/1-61 PF01325

MLGSRAEDYLEAIYSLSKEKGYAKVMELSEVLNVKPATVSEMLEKLSRMGYVEYKKRSHVK

>P72424_STAEP/1-61 PF01325

MLTEEKEDYLKAILTNDGDVSFVSNKKLSQFLNIKPPSVSEMVGRLEKEGYVETKHYKGAR

>Q50379_MYCSM/3-63 PF01325

DLVDTTEMYLRTIYDLEEEGVVPLRARIAERLDQSGPTVSQTVSRMERDGLLHVAGDRHLE

>ARI1A_HUMAN/1014-1119 PF01388

LGGEPERKMWVDRYLAFTEEKAMGMTNLPAVGRKPLDLYRLYVSVKEIGGLTQVNKNKKWRELATNLNVGTSSSAASSLKKQYIQCLYAFECKIERGEDPPPDIFA

>ARI3A_MOUSE/240-350 PF01388

LDADPKRKEFLDDLFSFMQKRGTPVNRIPIMAKQVLDLFMLYVLVTEKGGLVEVINKKLWREITKGLNLPTSITSAAFTLRTQYMKYLYPYECERRGLSSPNELQAAIDSN

>ARI4A_HUMAN/306-416 PF01388

ELDPEERDNFLQQLYKFMEDRGTPINKPPVLGYKDLNLFKLFRLVYHQGGCDNIDSGAVWKQIYMDLGIPILNSAASYNLKTAYRKYLYGFEEYCRSANIQFRTVHHHEPK

>DRI_DROME/290-400 PF01388

INDDPKRKEFLDDLFSFMQKRGTPINRLPIMAKSVLDLYELYNLVIARGGLVDVINKKLWQEIIKGLHLPSSITSAAFTLRTQYMKYLYPYECEKKNLSTPAELQAAIDGN

>ECM5_YEAST/182-294 PF01388

EFQRTKIVDFYAKLYNFHNKIKKSTLTRIPSIDKRTLDLYRLRSCVKLRGGFNAVCEKKLWAQIGRELGYSGRIMSSLSTSLRSAYAKILLDFDIYEEEEQAARNNEKNEDMV

>JAD1A_HUMAN/81-189 PF01388

EAMTRVRLDFLDQLAKFWELQGSTLKIPVVERKILDLYALSKIVASKGGFEMVTKEKKWSKVGSRLGYLPGKGTGSLLKSHYERILYPYELFQSGVSLMGVQMPNLDLK

>JAD1C_HUMAN/76-184 PF01388

EAQTRVKLNYLDQIAKFWEIQGSSLKIPNVERRILDLYSLSKIVVEEGGYEAICKDRRWARVAQRLNYPPGKNIGSLLRSHYERIVYPYEMYQSGANLVQCNTRPFDNE

>JARD2_MOUSE/616-726 PF01388

GRRWGPNVQRLACIKKHLRSQGITMDELPLIGGCELDLACFFRLINEMGGMQQVTDLKKWNKLADMLRIPKTAQDRLAKLQEAYCQYLLSYDSLSPEEHRRLEKEVLMEKE

>P91019_CAEEL/296-407 PF01388

MKVMAERRGFFERLIEFCEHNGEPLTMVPQVSKQSIDLHRLYIGVRAKGGFQQVTKDKYWKNLCTEANPDLAESSAAGYQLRKHYQRHLLMLECRETGRNPEDEVAFADKMK

>Q23541_CAEEL/118-233 PF01388

EAIVKEKHTFIDRLINFNRYSGLTFEFPVDRDGNIVDLYRLHRIVQNFGGCEEVNEDEKWRDVAREYLPKEQMARGVPSAFINLIRSHYNLHIEPFNRNLKEKAMKNDDESDDEME

>SWI1_YEAST/403-508 PF01388

ELNNKQYELFMKSLIENCKKRNMPLQSIPEIGNRKINLFYLYMLVQKFGGADQVTRTQQWSMVAQRLQISDYQQLESIYFRILLPYERHMISQEGIKETQAKRIFL

>YP83_CAEEL/21-131 PF01388

PEKQRKMAEFYNSLRMFYKRRWNATLKLPHVQGVEVNLYRLYDTVMALGGWQKVAASDKWSDIAEMFGCKDDILCGDHAIKIIYMRYLSKFEQVETIGDVDDYVDNEMSRS

>Q43888_STRAG/238-272 PF01396

VGKCPKCGNNIVLKKSFYGCSNYPECKFTLAEHFR

>Q60149_STRPY/630-664 PF01396

VGKCPKCGNNIVLKKSFYGCSNYPECKFTLAEHFR

>TOP1_BACSU/616-656 PF01396

GVKCPSCGEGNIVERKSKKKRVFYGCDRYPDCEFVSWDKPI

>TOP1_ECOLI/708-745 PF01396

IVECEKCGSEMHLKMGRFGKYMACTNEECKNTRKILRN

>TOP1_METJA/677-715 PF01396

DRICPKCGAKLILKKGVYGAFYGCSNYPKCKYTEPINKK

>TOP1_MYCGE/615-655 PF01396

ERDCPKCNQPLVYRYTKRGNEKFVGCSDFPKCKYSEFSNPK

>TOP1_MYCGE/664-705 PF01396

DELCPECNNKLVKRRTKFNAKKTFIGCSNFPNCRFIKKDNAA

>TOP1_MYCPN/621-661 PF01396

GRDCPSCASPLLYRYTKRGNEKFVGCSNFPNCKYNEFSQNK

>TOP1_MYCPN/670-711 PF01396

EELCPECNSQLVKRRTKFNPNKTFVGCSNFPRCRYIKKDNAS

>TOP3A_HUMAN/655-694 PF01396

IRKCPQCNKDMVLKTKKNGGFYLSCMGFPECRSAVWLPDS

>Y656A_HAEIN/101-141 PF01396

KITCPECKTGHLISRRGRQGKIFYGCDNFPKCKFSLPAKPY

>Y664_METJA/263-301 PF01396

DNTCPWCGAKLRVVRTKKGEFLGCTNYPNCLYRRFPKKN

>YRDD_ECOLI/103-143 PF01396

AITCPQCRTGHLVQRRSRYGKTFHSCDRYPECQFAINFKPI

>LEXA_BACSU/1-65 PF01726

MTKLSKRQLDILRFIKAEVKSKGYPPSVREIGEAVGLASSSTVHGHLARLETKGLIRRDPTKPRA

>LEXA_HAEIN/1-65 PF01726

MRPLTARQQEVLDLLKRHLETTGMPPTRAEISRELGFKSANAAEEHLKALSRKGAIEIIPGASRG

>LEXA_MYCLE/2-66 PF01726

DSGLTERQRTILNVIRASVTSRGYPPSIREIADAVGLTSTSSVAHQLRTLERKGYLRRDPNRPRA

>LEXA_PSEAE/1-65 PF01726

MQKLTPRQAEILSFIKRCLEDHGFPPTRAEIAQELGFKSPNAAEEHLKALARKGAIEMTPGASRG

>LEXA_SHIFL/1-65 PF01726

MKALTARQQEVFDLIRDHISQTGMPPTRAEIAQRLGFRSPNAAEEHLKALARKGVIEIVSGASRG

>LEXA_STRCO/25-89 PF01726

SSGLTDRQRRVIEVIRDSVQRRGYPPSMREIGQAVGLSSTSSVAHQLMALERKGFLRRDPHRPRA

>LEXA_THEMA/1-64 PF01726

MKDLTERQRKVLLFIEEFIEKNGYPPSVREIARRFRITPRGALLHLIALEKKGYIERKNGKPRA

>P73722_SYNY3/1-65 PF01726

MEPLTRAQKELFDWLVSYIDETQHAPSIRQMMRAMNLRSPAPIQSRLERLRNKGYVDWTDGKART

>DNBP_METTH/7-110 PF01984

IRRKKMLELQQKAQQQAMEAEAQEQMRQQLEMQKKQIMMQILTPEARSRLANLRLTRPDFVEQIELQLIQLAQMGRVRSKITDEQLKELLKRVAGKKREIKISR

>PDCD5_HUMAN/8-112 PF01984

LRRQRLAELQAKHGDPGDAAQQEAKHREAEMRNSILAQVLDQSARARLSNLALVKPEKTKAVENYLIQMARYGQLSEKVSEQGLIEILKKVSQQTEKTTTVKFNR

>Y1060_PYRHO/11-115 PF01984

IRRRKLMELQRKYLEQQKAQEEAERQQALIEAQIQAILRKILTPEARERLARVKLVRPELARQVELILVQLYQAGQITERIDDAKLKRILAQIEAKTRREFRIKW

>Y2068_ARCFU/10-110 PF01984

IRRRKLMELQRQKELEELQKEEMRRQVEAQKKAILRAILEPEAKERLSRLKLAHPEIAEAVENQLIYLAQAGRIQSKITDKMLVEILKRVQPKKRETRIIR

>Y691_METJA/6-108 PF01984

IKRKKLLELQKKLAEQQQQEEALLEAEMQKRALLRKILTPEARERLERIRLARPEFAEAVEVQLIQLAQLGRLPIPLSDEDFKALLERISALTKRKREIKIVR

>YF69_SCHPO/8-118 PF01984

IRQARLAQLQAEHGSAPSNIASGPSSNQQQQEVQDEMRQNLLSQILEHPARDRLRRIALVRKDRAEAVEELLLRMAKTGQISHKISEPELIELLEKISGEVSKRNETKIVI

>HAP2_KLULA/184-240 PF02045

EQPFYVNAKQYYRILKRRYARAKLEENLKISRERRPYLHESRHKHAMRRPRGQGGRF

>NFYA1_ARATH/171-227 PF02045

QEPVYVNAKQYEGILRRRKARAKAELERKVIRDRKPYLHESRHKHAMRRARASGGRF

>NFYA2_ARATH/135-195 PF02045

DSTIYVNSKQYHGIIRRRQSRAKAAAVLDQKKLSSRCRKPYMHHSRHLHALRRPRGSGGRF

>NFYA3_ARATH/178-234 PF02045

TDPVFVNAKQYHAIMRRRQQRAKLEAQNKLIRARKPYLHESRHVHALKRPRGSGGRF

>NFYA4_ARATH/97-153 PF02045

EEPVFVNAKQYHGILRRRQSRAKLEARNRAIKAKKPYMHESRHLHAIRRPRGCGGRF

>NFYA_RAT/256-312 PF02045

EEPLYVNAKQYHRILKRRQARAKLEAEGKIPKERRKYLHESRHRHAMARKRGEGGRF

>O49915_ORYSA/137-193 PF02045

EEPIYVNAKQYHAILRRRQLRAKLEAENKLVKNRKPYLHESRHQHAMKRARGTGGRF

>P87249_EMENI/231-288 PF02045

ESPLYVNAKQFHRILKRRVARQKLEEQLRLTSKGRKPYLHESRHNHAMRRPRGPGGRF

>Q20858_CAEEL/302-358 PF02045

QQPMLVNPKQFNRIMRRREMRQQLEASGRLPLARQKYLHESRHLHALKRKRGLDGRF

>Q42471_BRANA/176-232 PF02045

QEPVYVNAKQYQAIMRRRQARAKAELEKKLIKSRKRYLHESRHQHAMRRPRGTGGRF

>DNB2_ADE05/176-264 PF02236

PIVSAWEKGMEAARALMDKYHVDNDLKANFKLLPDQVEALAAVCKTWLNEEHRGLQLTFTSKKTFVTMMGRFLQAYLQSFAEVTYKHHE

>DNB2_ADE07/161-249 PF02236

PLVSAWEKGMEVMAVLMERYRLDNDLRTSFKLMPEQHEQYKRICHQYVNEEHRGIPLTFSSMKTLTAMMGRFMQGLVHSYSEIAHNNWE

>DNB2_ADE12/131-219 PF02236

PLVSAWEKGMEAMAMLMEKYHVDHDERATFRFLPDQGSVYKKICTTWLNEEKRGLQLTFSSQKTFQELMGRFLQGYMQAYAGVQQNSWE

>DNB2_ADE40/122-210 PF02236

PLVSAWEKGMDLMIKLMEKYHVEAEEKNGFKFLPEQSNVYRKICQTWLNEEHRGLPLTFTSHKTFVEMMGRFLRAYVESYAGVKNNEWE

>DAF19_CAEEL/243-324 PF02257

DDADSINLPNNQRASPATVNWLFENYEIGEGSLPRCELYDHYKKHCAEHRMDPVNAASFGKLIRSVFHNLKTRRLGTRGNSK

>Q14267_HUMAN/254-336 PF02257

DEEKENNRASKPHSTPATLQWLEENYEIAEGVCIPRSALYMHYLDFCEKNDTQPVNAASFGKIIRQQFPQLTTRRLGTRGQSK

>RFX1_MOUSE/407-488 PF02257

GNASQSYSHTTRASPATVQWLLDNYETAEGVSLPRSTLYCHYLLHCQEQKLEPVNAASFGKLIRSVFMGLRTRRLGTRGNSK

>RFX1_YEAST/270-350 PF02257

LQLAEQNRERERQVFALLWLMKNCKSQHDSYVPRGKIFAQYASSCSQNNLKPLSQASLGKLIRTVFPDLTTRRLGMRGQSK

>RFX5_HUMAN/76-158 PF02257

DKSSEPSTLSNEEYMYAYRWIRNHLEEHTDTCLPKQSVYDAYRKYCESLACCRPLSTANFGKIIREIFPDIKARRLGGRGQSK

>SAK1_SCHPO/86-166 PF02257

MDHVSANSEKFRQVFGICWLKRACEEQQDAAVQRNQIYAHYVEICNSLHIKPLNSASFGKLVRLLFPSIKTRRLGMRGHSK

>Q38013_BPD31/8-139 PF02316

TKLAGLPGLPTTDRGVRKLAEREGWKKQKHSGRGGGYEYHVSALPKETRAALLNAALGEVATKAVRQETQLALVETNRQQLVADARQGVLHALDLMMARTGYSRKRSITLMLDMARLGQVEPQLLAMLKMAR

>TRA_BPMU/8-156 PF02316

KECANLPGLPKTSAGVIYVAKKQGWQNRTRAGVKGGKAIEYNANSLPVEAKAALLLRQGEIETSLGYFEIARPTLEAHDYDREALWSKWDNASDSQRRLAEKWLPAVQAADEMLNQGISTKTAFATVAGHYQVSASTLRDKYYQVQKFA

>Q20619_CAEEL/95-164 PF02319

RKEKSLGLLCQRFLIAINEETVGSSTREVHLETVARKMNVEKRRIYDIVNVMEALDAMQKTNKSYYQWQG

>Q7YY02_CRYPV/368-434 PF02319

RFENGLVLMTKRFIQYLYEQGESRIIDLAAAEAHMDVQRRRLYDITNVLEGIGILKKMGRNAFMCSA

>Q8BQJ5_MOUSE/113-182 PF02319

RKEKSLGLLCHKFLARYPKYPNPAVNNDICLDEVAEELNVERRRIYDIVNVLESLHMVSRLAKNRYTWHG

>Q8BRE2_MOUSE/143-212 PF02319

RKQKSLGLLCQKFLARHPSYPLSTEKTTISLDEVAVSLGVERRRIYDIVNVLESLHLVSRVAKNQYGWHG

>Q8BRE2_MOUSE/283-368 PF02319

RKDKSLRIMSQKFVMLFLVSKTKIVTLDVAAKILIEESQDTPDHSKFKTKVRRLYDIANVLTSLALIKKVHVTEERGRKPAFKWIG

>Q8BSQ3_MOUSE/143-212 PF02319

RKQKSLGLLCQKFLARYPSYPLSTEKTTISLDEVAVSLGVERRRIYDIVNVLESLHLVSRVAKNQYGWHG

>Q8LSZ4_ARATH/169-250 PF02319

RREKSLGLLTQNFIKLFICSEAIRIISLDDAAKLLLGDAHNTSIMRTKVRRLYDIANVLSSMNLIEKTHTLDSRKPAFKWLG

>Q8RWL0_ARATH/145-225 PF02319

KKEKSLWLLAQNFVKMFLCSDDDLITLDSAAKALLSDSPDSVHMRTKVRRLYDIANVFASMNLIEKTHIPVTRKPAYRWLG

>Q8SRS9_ENCCU/13-81 PF02319

NKREGLKYITQAVFQVLRENGACTYSFICKNIVFPNTETLNRRIYDVLNVMKAVRLVDKKGKRYFLVDD

>Q8SSD8_ENCCU/14-79 PF02319

RSENSLYNLTRRFLKLVSMSPDRNVSIHQASIELNVGKRRIYDITNVLEGLGLLSKWSVSNAKWIG

>Q9FNY3_ARATH/49-133 PF02319

TSGGGLRQFSVMVCQKLEAKKITTYKEVADEIISDFATIKQNAEKPLNENEYNEKNIRRRVYDALNVFMALDIIARDKKEIRWKG

>Q9GT27_BRUMA/100-183 PF02319

DKSKGLRHFSTKVCEKVKKRHTNYNEVADELVSEYFDSADVQPTDTEKQQYDMKNIRRRVYDALNVLMAMNIIEKEKKEIRWVG

>Q9LFQ9_ARATH/138-217 PF02319

RKERTLWLLAQNFVKLFLCSDDDLVTFDSATKALLNESQDMNMRKKVRRLYDIANVFSSMKLIEKTHVPETKKPAYRWLG

>Q9LZE7_ARATH/99-199 PF02319

KTGRGLRQFSMKGLISFSAPIMLSSKCLSICEKVESKGRTTYNEVADELVAEFALPNNDGTSPDQQQYDEKNIRRRVYDALNVLMAMDIISKDKKEIQWRG

>Q9NZ54_HUMAN/46-128 PF02319

KNGMGLCRLSMKVWETVQRKGTTSCQEVVGELVAKFRAASNHASPNESAYDVKNIKRRTYDALNVLMAMNIISREKKKIKWIG

>TDP1_CAEEL/68-152 PF02319

DKPTGLRHFSTKVCEKVKEKGLTNYNEVADELVADYFQNNLIKQIDVVKQEYDMKNIRRRVYDALNVLLAMNIITKSKKDIRWIG

>TDP1_MOUSE/111-193 PF02319

KNGKGLRHFSMKVCEKVQRKGTTSYNEVADELVAEFSAADNHILPNESAYDQKNIRRRVYDALNVLMAMNIISKEKKEIKWIG

>TDP2_MOUSE/127-213 PF02319

KNGKGLRHFSMKVCEKVQRKGTTSYNEVADELVSEFTNSNNHLAADSAYDQENIRRRVYDALNVLMAMNIISSLPTGKKRNQVDCNS

>ARFA_ARATH/123-228 PF02362

SFCKTLTASDTSTHGGFSVLRRHADDCLPPLDMSQQPPWQELVATDLHNSEWHFRHIFRGQPRRHLLTTGWSVFVSSKKLVAGDAFIFLRGENEELRVGVRRHMRQ

>ARFC_ARATH/158-263 PF02362

MFCKTLTASDTSTHGGFSVPRRAAEDCFPPLDYSQPRPSQELLARDLHGLEWRFRHIYRGQPRRHLLTTGWSAFVNKKKLVSGDAVLFLRGDDGKLRLGVRRASQI

>ARFM_ARATH/125-232 PF02362

FFSKILTASDVSLSGGLIIPKQYAIECFPPLDMSQPISTQNLVAKDLYGQEWSFKHVFRGTPQRHMFTSGGGWSVFATTKRLIVGDIFVLLRGENGELRFGIRRAKHQ

>ARFQ_ARATH/118-223 PF02362

TFAKILTPSDANNGGGFSVPRFCADSVFPLLNFQIDPPVQKLYVTDIHGAVWDFRHIYRGTPRRHLLTTGWSKFVNSKKLIAGDSVVFMRKSADEMFIGVRRTPIS

>ARFR_ARATH/127-232 PF02362

SFVKILTASDTSTHGGFSVLRKHATECLPSLDMTQATPTQELVTRDLHGFEWRFKHIFRGQPRRHLLTTGWSTFVSSKRLVAGDAFVFLRGENGDLRVGVRRLARH

>ARFT_ARATH/125-230 PF02362

SFTKVLTASDTSAYGGFFVPKKHAIECLPPLDMSQPLPAQELLAKDLHGNQWRFRHSYRGTPQRHSLTTGWNEFTTSKKLVKGDVIVFVRGETGELRVGIRRARHQ

>O23110_ARATH/169-277 PF02362

LFEKAVTPSDVGKLNRLVIPKQHAEKHFPLPSPSPAVTKGVLINFEDVNGKVWRFRYSYWNSSQSYVLTKGWSRFVKEKNLRAGDVVTFERSTGLERQLYIDWKVRSGP

>O23625_ARATH/570-674 PF02362

LLQKVLKQSDVGNLGRIVLPKKEAETHLPELEARDGISLAMEDIGTSRVWNMRYRFWPNNKSRMYLLENTGDFVKTNGLQEGDFIVIYSDVKLIRGVKVRQPSGQ

>O49236_AVEFA/503-607 PF02362

LLQKVLKQSDVGALGRIVLPKEAETHLPELKTRDGISIPMEDIGTSRVWSMRYRFWPNNKSRMYLLENTGDFVRSNELQEGDFIVIYSDVKSGKYLIRGVKVRPP

>O65420_ARATH/320-421 PF02362

YNFKILSATDTGKRLVLPKKYAEAFLPQLSHTKGVPLTVQDPMGKEWRFQFRFWPSSKGRIYVLEGVTPFIQTLQLQAGDTVIFSRLDPERKLILGFRKASI

>O81781_ARATH/170-257 PF02362

CFVAHVTDSNLREDTLFLPRKFDRSDGLIKGSNKIVLMNEEARTWTLILKFRNSKLKSVSTKDFNKIKRKESGDSSQKVPSSSSSVSE

>O81782_ARATH/284-379 PF02362

QTQSCFIFDHVAKSLPRNFVRSDGLIKGSNKIVLMNEGARTWTLILKFRDSRRSFYSRGGWRSFCRENGLKPGDSVTFKLESSNTKTPLLLFSIAE

>O82411_ARATH/90-195 PF02362

LFQKELKNSDVSSLRRMILPKKAAEAHLPALECKEGIPIRMEDLDGFHVWTFKYRYWPNNNSRMYVLENTGDFVNAHGLQLGDFIMVYQDLYSNNYVIQARKASEE

>O82595_ARATH/30-122 PF02362

MFDKVLTPSDVGKLNRLVIPKQHAENFFPLEDNQNGTVLDFQDKNGKMWRFRYSYWNSSQSYVMTKGWSRFVKEKKLFAGDTVSFYRGYIPDD

>Q7XKC4_ORYSA/367-472 PF02362

ILRKELTNSDVGNIGRIVMPKRDAEAHLPALHQREGVTLKMDDFKFETTWNFKYRFWPNNKSRMYVLESTGGFVKHHGLQTGDIFIIYKSSESGKFVVRGEKAIKP

>Q7XKC5_ORYSA/296-399 PF02362

ILRKELTNSDVGNIGRIVMPKRDAEAHLPALHQREGVMLKMDDFKLETTWNFKYRFWPNNKSRMYVLESTGGFVKQHVLQTGDIFIIYKSSESEKLVVRGEKAI

>Q7XKK6_ORYSA/120-225 PF02362

SFAKTLTQSDANNGGGFSVPRFCAETIFPELDYSSEPPVQSVCAKDVHGVEWTFRHIYRGTPRRHLLTTGWSPFVNKKQLTAGDSIVFMRDEGGNIHVGLRRAKRG

>Q7XS75_ORYSA/20-120 PF02362

TQLKVLVPSSFRKMRICDELAAQLGVGVGGGGAPRAATARVASPLGKAWDVGVVRDGDGRAFLGRGWAEFAAAHGLGVGWFVVLRHGGGVLAVEAFDTTCC

>Q7XSS9_ORYSA/127-236 PF02362

YYAKQLTQSDANNGGGFSVPRLCADHIFPALNLDDDPPVQSLTMGDLQGDSWEFRHIYRGTPRRHLLTTGWSKFVNAKQLVAGDTVVFMWCGAPAPERKLLVGVRRAARY

>Q84R27_ARATH/29-124 PF02362

KFFKLVLPSTMKDKMMKIPPRFVKLQGSKLSEVVTLETPAGFKRSIKLKRIGEEIWFHEGWSEFAEAHSIEEGHFLLFEYKENSSFRVIIFNVSAC

>Q851V0_ORYSA/300-391 PF02362

YSTVGVSPEFAGRYLGPAMAREVVMERGGGGGGGDQWHVRFVRRESSRGFHGTGWRRFARDNGLLAHDVCLFELRLVDGAGAGDRLRRRPRP

>Q851V0_ORYSA/35-125 PF02362

YMVGDFTESMIVPSRFANNFNGHISEVVNLKSPSGKTWSIGVAYSDTGELVLRSGWKEFVDANGVQENDCLLFRYSGVSSFDVLIFDPSGC

>Q851V0_ORYSA/413-504 PF02362

TATGAIWPTTTVPARFANNFNGHISEEVNLRSPSGETWSIGVANSDAGELVLQPGWKEFVDGNGIEEGDCLLFRYSGVSSSFDVLIFDPSGC

>Q851V5_ORYSA/149-242 PF02362

YFFKVMIGGFRRQMTIPYKFAENFRDQIQGTIKLKARNGNTCSVLVDKCSNKLVLTKGWAEFANSHDIKMGDFLVFRYTGNSQFEVKIFDPSGC

>Q851V5_ORYSA/452-545 PF02362

YFFKVMIGDFHKRMTIPDKFARHFKGVISKTIKLEPRSGYTFDVQVTKKLNILVLGSGWESFVNAHDLNMGDFLVFKYNGDFLLQVLIFDPSGC

>Q851V5_ORYSA/742-826 PF02362

FRERMIIPNEFLQYFRGKIPRTIKLQLRDGCTYDVQVTKNLGKISLQSGWKAFVTAHDLQMGDFLVFSYDGISKLKVLIFGPSGC

>Q851V5_ORYSA/939-1029 PF02362

KSRVHGKSQTVDICREYADVYLPFKELNMTLQRHGKNWEVLCRTKDTRTKRLSTGWSRFAQENNLQVGDICLFELLKKKEYSMNVHIIPKK

>Q851W4_ORYSA/28-121 PF02362

CFHRQMSANFEHSMIIPNKFLDQFGGKISRTVELESPKGNVYVVKVSKHMNKTVLQCGWEAFVDAHQIEENDSLLFRHIKNSRRASGVQERNAD

>Q851W4_ORYSA/440-542 PF02362

IYVSIMNKSNVGTDGLYIITLGRQFAIRYLPEGEQTLTLLTTGTGKAWQVKMRPRSGDARMFTLGWRDFVRDNRLQTEDICLFQLTKNSERGLAMKVHIIRHN

>Q851W5_ORYSA/247-351 PF02362

VFVAIMKHSNVNSRRACLVIPKRYASAHFPLESQTITLQRQGKNKKWYPMFYIRKDGSGYMLYGCWKNFVRDNHVKEGDMCIFHLTKFTGGEFGATVHLLRETKS

>Q851W5_ORYSA/415-518 PF02362

MYVSIMNKSNVGTDGLYIIIFGRQFATRYLPEGEQTLTLLMTGKSNAWQVKMRPRSGDAQMITTGWRHFVHDNHLQIEDICLFQLMNDESKLTMTVHIIRRNEK

>Q8GT89_PRUPE/148-253 PF02362

FFCKTLTASDTSTHGGFSVPRRAAEKLFPPLDFTMQPPSQELVVRDLHDNSWTFRHIYRGQPKRHLLTTGWSLFVGAKRLRAGDSVLFIRDEKSQLMIGVRRANRQ

>Q8GYJ2_ARATH/37-146 PF02362

LFEKPLTPSDVGKLNRLVIPKQHAERYFPLAAAAADAVEKGLLLCFEDEEGKPWRFRYSYWNSSQSYVLTKGWSRYVKEKHLDAGDVVLFHRHRSDGGRFFIGWRRRGDS

>Q8H0U7_ARATH/197-288 PF02362

EFSMFIKKSYLIYMWFPKSVQSIHMPKQRTIFKIHHPNMKKSWNVVYVVSGTKSSFSAGWKGLAQEYPLAVGDTCKFSFIKQHELILFVSKP

>Q8H506_ORYSA/123-224 PF02362

YFVKTLMSSDAEYRDRFAVPMDVAKDVFPPLVDAKAVQPLIVKDLQGSPMTFDYGRNGNRVTLAKVWKKFRDDMDFVDGDSVIFMRRRDDDELYVGVRRQRT

>Q8H506_ORYSA/441-543 PF02362

SFVKPLTCTDAVKNRYRFIVPKRETAMGVLPQLQLNEHVPLYIKDMHGKEWVINYTWKEYTHMLSSGWIKFANANRLVTGDNVVFMRSMDSGERYMGLRRTLK

>Q8H507_ORYSA/123-229 PF02362

YFVKTLMISDFDFRIRFSAPMADAKGVFPPLVDAKAVQPLLVKDLHGSPMTFDYGRKGKRVTLAKVWKKFRDDMDFVDGDSVIFMRRRDDDDDDGELYVGVRRQRTL

>Q8H508_ORYSA/119-235 PF02362

FFEKQLSPADVTSNALVLPAGAEHVLPPLDIAAYQTARLFDVRDLRGKRFEFVHIWDKKRCRYMLGDLGVNDNDGWRGFVKAKRLATRDTVVFMRRGGGDGDGDGELLVGVRRAPRA

>Q8H6E4_MARPO/71-175 PF02362

LFEKAVTPSDVGKLNRLVIPKQHAERCFPLDLALNVPCQTLSFEDVSGKHWRFRYSYWNSSQSYVFTKSWSCFLKGKKLEAGDTVSFERGPNQELYIDFRRRLNN

>Q8L3W1_ARATH/243-340 PF02362

FFRVVLRPSYLYRGCIMYLPSGFAEKYLSGISGFIKVQLAEKQWPVRCLYKAGRAKFSQGWYEFTLENNLGEGDVCVFELLRTRDFVLKVTAFRVNEY

>Q8LAA3_ARATH/182-291 PF02362

LFEKTVTPSDVGKLNRLVIPKHQAEKHFPLPLGNNNVSVKGMLLNFEDVNGKVWRFRYSYWNSSQSYVLTKGWSRFVKEKRLCAGDLISFKRSNDQDQKFFIGWKSKSGL

>Q8LEW1_ARATH/6-115 PF02362

MFDKVVTPSDVGKLNRLVIPKQHAERYFPLDNSTTNDSNKGLLLNFEDRSGNSWRFRYSYWNSSQSYVMTKGWSRFVKDKKLDAGDIVSFQRDSCNKDKLYIDWRRRPKI

>Q8LMR9_ORYSA/36-144 PF02362

MFDKVVTPSDVGKLNRLVIPKQHAEKYFPLDAASNEKGLLLSFEDRTGKPWRFRYSYWNSSQSYVMTKGWSRFVKEKRLDAGDTVSFGRGVGEAARGRLFIDWRRRPDV

>Q8RYD1_ARATH/223-323 PF02362

FLVFMKRSHVVSKCFLTIPYKWCVKNMLITRQEVVMQVDQTKWEMKFNIFGARGSGGISTGWKKFVQDNNLREGDVCVFEPANSETKPLHLNVYIFRGEET

>Q8RYD3_ARATH/28-142 PF02362

LFEKSLTPSDVGKLNRLVIPKQHAEKYFPLNNNNNNGGSGDDVATTEKGMLLSFEDESGKCWKFRYSYWNSSQSYVLTKGWSRYVKDKHLDAGDVVFFQRHRFDLHRLFIGWRRR

>Q8S2E6_ORYSA/20-123 PF02362

HFFKVLVGDFKQRLKIPPNFCKHIPWEESRKAKGLKEASMAATLEGPSGRTWLVVIRRTAEGTFFTSGWPKFVQDQALRELEFVVFRYDGNTRFTAMVFDRTAC

>Q8S2E6_ORYSA/288-383 PF02362

YCVIRMSTMHVYYSFMMRFPTGFSRQHLPRERTDVVLRDPGGKVWSVLYIPNTRDRLSRGWCAFARGNCLEEGDYCVFELVAAAEFRVHIFRVVEP

>Q8S8E8_ARATH/588-682 PF02362

FLTLTLAPEDVKDGNLHLPCQFMRINGINKPGQITLLGRGGMKWFAYLLSGDGTVVVGNGWKGFCEANGVMLGETFVLEFIPKDDTNHVFKFYTK

>Q8S975_ORYSA/117-222 PF02362

SFCKILTPSDTSTHGGFSVLRRHANECLPPLDMSMATPTQELITKDLHGSEWRFKHIYRGQPRRHLLTTGWSTFVTSKKLISGDAFVYLRSETGEQRVGVRRLVQK

>Q94HG5_ORYSA/136-230 PF02362

SFVKPMLHSHVVRGFWLGLPRHFCETYLPKHDAIVTLLDEKDEQFDTNYLAYKNGLSGGWAGFALDHGLLDGDATVFQLVKPTTFKVHIIRATVD

>Q9AV47_ORYSA/127-232 PF02362

SFAKTLTQSDANNGGGFSVPRYCAETIFPKLDYRADPPVQTVLAKDVHGVVWKFRHIYRGTPRRHLLTTGWSTFVNQKKLVAGDSIVFLRTRHGELCVGIRRAKRM

>Q9AWS0_ORYSA/181-294 PF02362

LFEKAVTPSDVGKLNRLVVPKQHAEKHFPLRRAASSDSASAAATGKGVLLNFEDGEGKVWRFRYSYWNSSQSYVLTKGWSRFVREKGLRAGDTIVFSRSAYGPDKLLFIDCKKN

>Q9AWS7_ORYSA/177-286 PF02362

LFEKAVTPSDVGKLNRLVVPKQQAERHFPFPLRRHSSDAAGKGVLLNFEDGDGKVWRFRYSYWNSSQSYVLTKGWSRFVREKGLRPGDTVAFSRSAAAWGTEKHLLIDCK

>Q9C688_ARATH/177-288 PF02362

LFQKELTPSDVGKLNRLVIPKKYAVKYMPFISDDQSEKETSEGVEDVEVVFYDRAMRQWKFRYCYWRSSQSFVFTRGWNGFVKEKNLKEKDIIVFYTCDVPNNVKTLEGQSK

>Q9FGD2_ARATH/220-316 PF02362

HFVRNITRGSLQKLELPLTFLRSNGIELEEDIELCDESGKKWPLKILNHDRGFKFSHESWLCFCKSHEMILTNKCLFEFIVPSNGRCSEILVRIVSG

>Q9FJG2_ARATH/216-307 PF02362

EFKLTIKKSHLLFLGIPKKFVDMHMPTETTMFKIHYPRGKKSWDVTYVVTDVQSRFSGGWSRLAKELGLLVGDVCTFKLIKPTEMRVKVSKE

>Q9FK61_ARATH/211-294 PF02362

EFTLTIKKSYLIFLGIPKMFEELHMPTEATMFKIHDPEGKRSWDVMYKFSNNQTRFCAGWIRLAKELGLEIGDVCTFTLIKPTE

>Q9FNI3_ARATH/45-161 PF02362

LFEKSLTPSDVGKLNRLVIPKQHAEKYFPLNAVLVSSAAADTSSSEKGMLLSFEDESGKSWRFRYSYWNSSQSYVLTKGWSRFVKDKQLDPGDVVFFQRHRSDSRRLFIGWRRRGQG

>Q9FNS6_9CONI/549-652 PF02362

LLQKVLKQSDVGNLGRIVLPKKEAETHLPELEARDGISIAMEDIVTSRVWNMRYRFWPNNKSRMYLLENTGDFVRSNGLQEGDFIVLYSDTKTGKYMIRGVKVP

>Q9LJL1_ARATH/139-233 PF02362

SFTKPMLQSHVTGGFWLGLPLPFCKAHMPKRDVIMTLVDEEEEESQAKYLAQKNGLSGGWRGFAIDHQLVDGDAVVFHLIARTTFKVYIIRVNDD

>Q9LSP6_ARATH/201-294 PF02362

EFKITIRKSYLKFLAIPKHFVDDHIPNKSKIFTIRHPNGGSWKVLCLVREIRTIFSGGYSKLAREFPLMVGDKCTFKLIKPFEFVLLTSKKNRE

>Q9SJ97_ARATH/120-216 PF02362

RFVTFTPEDIRDCILILPSQFIKANGINNLGEITLLGQNRMKWFAYLLSMSKDGSLALGSGWKGICEANGVNTGEAFTLEYIDEQETAHKTSQCVGE

>Q9SJA0_ARATH/449-545 PF02362

SNDSCLVVVSLLYFDMRLPKVFTRENGINKPGRITLLGKDGIKQQTNLLFDKANGAMSLGHGWKDFVKDNGLKTGDSFTLKLIWEDQTPVLSLCPAD

>Q9SJA0_ARATH/878-973 PF02362

FVTLALTPEDVTACKLILPSQFMKANGINNKLGKITLLGENGVEWPGYMLSLDGTLALGNGWEGFCEANGVKLGQTFTLEFVNEQDTTTTPRIPVA

>Q9SZA5_ARATH/94-187 PF02362

SSIKITTFCNLLQVIPRKFSTHCKRKLPQIVTLKSPSGVTYNVGVEEDDEKTMAFRFGWDKFVKDHSLEENDLLVFKFHGVSEFEVLVFDGQTL

>Q9XJ59_DAUCA/513-620 PF02362

LLQKVLKQSDVGCLGRIVLPKREAETQLPQLEDRDGIQIVMEDIGTSKVWNLRYSLRYWPNNKSRMYVLENTGEFVKENGLQEGDFIVIYSDIKCGKYLIRGVKVRQP

>Q9ZR14_ARATH/137-240 PF02362

KKQLMSSDVDKDQCMLMLSKEQVKEKMLPFLEDSENPVKGIDVSVYGPDGKVQQMEFKMWNGDKTPVLTSGWKQFVEDYGLSMTCDFVTVWMFRHIKTRKLCFA

>O53353_MYCTU/21-84 PF02467

WQDRALCAQTDPEAFFPEKGGSTREAKKICMGCEVRHECLEYALAHDERFGIWGGLSERERRRL

>O69649_MYCTU/31-93 PF02467

WVSKALCRTTDPDELFVRGAAQRKAAVICRHCPVMQECAADALDNKVEFGVWGGMTERQRRAL

>O84993_RHOOP/17-87 PF02467

WRFHASCRSVSPDVFFGPDGERYGPRIRREREAKRICSSCLVLADCRAYAEESREGFGIWGGASEHERRAL

>O88103_STRCO/17-87 PF02467

WQLLAACRGVDSSLFFHPEGERGAARSARENSAKEVCMRCPVRAECAAHALAVREPYGVWGGLTEDEREEL

>P71592_MYCTU/12-83 PF02467

WFGYPDDDGSDGAAKARAYERSATQARIQCLRRCPLLQQRRCAQHAVEHRVEYGVWAGIKLPGGQYRKREQL

>Q49871_MYCLE/17-87 PF02467

WQLQGLCRGVDSSMFFHPDGERGRARMQREQRAKEMCRRCPVIEECRAHALDVGEPYGVWGGLSESERDLL

>Q9ZX29_BPMT4/7-70 PF02467

GDPSAICAQTDPELWFPDKGQSTRDAKRMCMRCPLLDECRALALRDPHLVGVWGGLSAQERRRI

>STA5A_BOVIN/332-583 PF02864

FIIEKQPPQVLKTQTKFAATVRLLVGGKLNVHMNPPQVKATIISEQQAKSLLKNENTRNECSGEILNNCCVMEYHQATGTLSAHFRNMSLKRIKRADRRGAESVTEEKFTVLFESQFSVGSNELVFQVKTLSLPVVVIVHGSQDHNATATVLWDNAFAEPGRVPFAVPDKVLWPQLCEALNMKFKAEVQSNRGLTKENLVFLAQKLFNSSSSHLEDYNGMSVSWSQFNRENLPGWNYTFWQWFDGVMEVLKK

>STAT_DROME/342-588 PF02864

FIVDKQPPQVMKTNTRFAASVRWLIGSQLGIHNNPPTVECIIMSEIQSQRFVTRNTQMDNSSLSGQSSGEIQNASSTMEYQQNNHVFSASFRNMQLKKIKRAEKKGTESVMDEKFALFFYTTTTVNDFQIRVWTLSLPVVVIVHGNQEPQSWATITWDNAFAEIVRDPFMITDRVTWAQLSVALNIKFGSCTGRSLTIDNLDFLYEKLQREERSEYITWNQFCKEPMPDRSFTFWEWFFAIMKLTKD

>O13463_EMENI/239-462 PF02919

IKWTTLEHNGVVFPPPYEPLPKHVKMKYDGIPVDLHPEAEEVAGFFGSMLNSTQHTENPTFQKNFFADFKEILKKTGGAKDQKGNKVDIKEFSKCDSQPIFQYYDAQRQEKKALPPAEKKRLKAEKDAQEAPYMYCMWDGRKQKVGNFRVEPPSLFRGRGEHPKTGRVKARVQPEQITINIGKEARVPPPPEGHKWKEVKHDQEGTWLAMWQENINGNYKYVML

>O24307_PEA/343-555 PF02919

RKWTTLVHNGVIFPPPYKPHGVKMLYMGRPVDLTPELRRGCYKYAVMRDTDYMQKDRFKENFWNDWRKLLGRNHVIQNLKDCDFTPIYDWCQSEKEKKKQMTTEEKKALKEEKLKQEEKYTWAIVDGVKRRVGNFRVEPPGLFRGRGEHPKMGRLKKRIHPSDIVINIGSEAQVPECPIPGEGWKDIRNDNTVTWLCYWSDPINPKLFKYVFL

>O59891_CRYNE/220-437 PF02919

SKWTVLEHNAVLFPPPYVPLPKDVKMKYDGVSLTLPPESEEVAGFFGALLETDYAQDAKFRENFFRDFKAIVEKYPPKEDVKVKKLEKCDFRPMFEYFEKEKEKKKALTKEEKKAIKAEKDKLEAPYLYANVDGRKEKVGNFRAEPPGLFKGRGEHPKKGTVKNRLRPEDIIINIGKEAPIPVPNIPGQWKGIQHDNTVTWLAHWKENVNGNAKYVFL

>O60013_PNECA/140-356 PF02919

IKWKTLEHNGVLFPPPYEPLPSYVKMKYKGEPISLPPEAEEVAGFFGAILSSEQYVKNPTFQKNFFHDFQETCKKTNAPVIPEKFEYCDFTPMFEYFEKKKEEKKNKSKEEKKKIKEEKDALEEKYKYCFLDGRKEKVGNFRIEPPGLFRGRGDHPKTGKLKTRVVPEQVIINIGKDSVIPSPPPGHNWKEVRHDNTVTWLATWNENVNNNVKYVFL

>Q26024_PLAFA/154-380 PF02919

IQWNYLEHRGLIFSPPYVQHHVPIFYKSIKIELNAKSEELATYWCSAIGSDYCTKEKFILNFFKTFINSLENDNIIKQENETKLKKGDISNFKFIDFMPIKDHLLKLREEKLNKTKEEKEEEKKMRMEKELPYTYALVDWIREKISSNKAEPPGLFRGRGEHPKQGLLKKRIFPEDVVINISKDAPVPRLYDNMCGHNWGDIYHDNKVTWLAYYKDSINDQIKYTFL

>Q27529_CAEEL/257-471 PF02919

VKWNSLQHCGPLFAPPYIPLPSHVHFKYGGEKMKLTLETEEIAQFYAGVLDHEYSTKEAFNKNFMKDWRKVMTVEERERIHDLKKCDFRAIDAYQKEQREIRKAMTKEEKLKIKEEKEAEVKIYGIAIIDGHRQKVANFRIEPPGVFRGRGGHPKMGLIKKRIMPEDVIINCGKDTEIPKPPPGHKWKEVRHDNTVTWLCSWTESVLGQNKYIML

>Q94705_PHYPO/410-622 PF02919

KRWTSLVHNGVVFVEPYVAHGVKFYYDGKPINLSPEAEEVATFYAKYLETDYPKKENFNTNFWSDFRTYLTPEQKKVIKDFSKADFTHIHKYLVEQKELKANRTAEEKKAEKAEKEKLLQKYGFAMINGHRQRISNWAIERPGLFLGRGAHPKTGLVKKRIEAEDITINIGKGVPVPEPPPGHKWCKVVHDDKVAWLAMWKENINNGFKYVWL

>TOP1_ARATH/369-581 PF02919

KKWTTLVHNGVIFPPPYKPHGIKILYKGKPVDLTIEQEEVATMFAVMRETDYYTKPQFRENFWNDWRRLLGKKHVIQKLDDCDFTPIYEWHLEEKEKKKQMSTEEKKALKEEKMKQEEKYMWAVVDGVKEKIGNFRVEPPGLFRGRGEHPKMGKLKKRIHPCEITLNIGKGAPIPECPIAGERWKEVKHDNTVTWLAFWADPINPKEFKYVFL

>TOP1_CANAL/155-371 PF02919

IKWQTLEHNGVMFPPPYEPLPSHVKLYYNNKPVNLPPEAEEVAGFYGAMLETDHAKNPVFQKNFFNDFLEVLKECGGCGVEIKKFEKLDFSKMYAHFEKLREEKKAMSREEKKRIKEEKEKEEEPYRTCYLNGRKELVGNFRIEPPGLFRGRGAHPKTGKLKRRVVSEQVTLNLGKDAKIPEPPAGHQWGEIRHDNEVTWLAMWKENISDSLKYVRF

>TOP1_DAUCA/215-430 PF02919

RNGQPLVHNGVIFPPLYKPHGVKMLYRENPVDLTPEQEEVATMFAVMLETEYMTKPKFRENFMSDWRKILGEKHIIQNLEDCDFTPIYEWHQREKEKKKQMSTDEKKAIKEGKDETRKEKYMWAVVDGMSREKVGNFRVGTTRVVQRSWRASKDPQVKKNVYSQIDITINIGKDAPIPEPPIPGERWKEIRHDNTVTWLAFWNDPINPKEFKYVFL

>TOP1_DROME/438-652 PF02919

VKWSTLEHKGPVFAPRYERVPRNVRFYYDGKPLELSEETEEAATFYAKMLNHDYCTKEVFNNNFFKDFRKSMTPREREIIKDFRKCNFQEMFNYFQAESEKRKAASKEEKLIKKNENEALMKEFGFCMIDGHKEKIGNFRLEPPGLFRGRGEHPKMGMIKRRIQASDVSINCGKDSKVPSPPPGSRWKEVRHDNTVTWLASWIENVQGQVKYIML

>TOP1_MOUSE/217-431 PF02919

IKWKFLEHKGPVFAPPYEPLPESVKFYYDGKVMKLSPKAEEVATFFAKMLDHEYTTKEIFRKNFFKDWRKEMTNDGKNTITNLSKCDFTQMSQYFKAQSEARKQMSKEEKLKIKEENEKLLKEYGFCVMDNHRERIANFKIEPPGLFRGRGNHPKMGMLKRRIMPEDIIINCSKDAKVPSPPPGHKWKEVRHDNKVTWLVSWTENIQGSIKYIML

>TOP1_SCHPO/193-408 PF02919

QKWTTLEHNGVIFAPPYEPLPKNVKLIYDGNPVNLPPEAEEVAGFYAAMLETDHAKNPVFQDNFFRDFLKVCDECNFNHNIKEFSKCDFTQMFHHFEQKREEKKSMPKEQKKAIKQKKDEEEEKYKWCILDGRKEKVGNFRIEPPGLFRGRGSHPKTGSLKRRVYPEQITINIGEGVPVPEPLPGHQWAEVKHDNTVTWLATWHENINNNVKYVFL

>TOP1_USTMA/161-383 PF02919

KKWDVLIHKGPRFPDPYQPLAKDVKLKYDGRPVDLPCQTEEIAMFYAVKLETQHAQNAIFNRNFFDDFKTDLKKYPPRDGTQIKSFDKLDFRDMYNYWRSLKDAELERKKALAPSARKREIEERKAEETKWKICLVDGVEQRVGNVNVEPPGLFLGRGAHPKAGKVKRRISPGDITINHSGDHPAPQPPAGMGDWAEVVEKKDVTWLAYWKENINGQYKYVFL

>TOP1_XENLA/271-485 PF02919

IKWKFLEHKGPVFAPPYEPVPDNVKFYYDGNLVKLSPKAEEVATFFAKMLDHEYTTKDIFRKNFFKDWKKEMTTDERNLITNLSKCDFNAMSLYFKEQSEARKNMTKEEKLKIKAENERLLQEYGYCIMDNHKERIANFRIEPPGLFRGRGDHPKMGKLKKRIMPEDIIINCSKDSKIPVAPAGHKWKEVRHDGKVTWLVSWTENIQGSIKYIML

>TOP1_YEAST/143-361 PF02919

IKWVTLKHNGVIFPPPYQPLPSHIKLYYDGKPVDLPPQAEEVAGFFAALLESDHAKNPVFQKNFFNDFLQVLKESGGPLNGIEIKEFSRCDFTKMFDYFQLQKEQKKQLTSQEKKQIRLEREKFEEDYKFCELDGRREQVGNFKVEPPDLFRGRGAHPKTGKLKRRVNPEDIVLNLSKDAPVPPAPEGHKWGEIRHDNTVQWLAMWRENIFNSFKYVRL

>LIPB2_HUMAN/192-256 PF02920

EKEQREQEEKQRKAEELLQELRHLKIKVEELENERNQYEWKLKATKAEVAQLQEQVALKDAEIER

>O85368_ENTFC/3-69 PF02920

EKRRDSKNRVLRSGESQRKDGRYAYKYVDTFGKPQFVYSWKLVPTDKTPAGKREDISLREKEKEIQK

>Q56367_9THEM/120-186 PF02920

EEIHKAKIVYVEAVWDTGKAIRYMMKYLSKEMEGRFGYSWKWIFKGAAQVWKWLCRALRYEMKEIIK

>TNR6_ENTFA/3-69 PF02920

EKRRDNRGRILKTGESQRKDGRYLYKYIDSFGEPQFVYSWKLVATDRVPAGKRDCISLREKIAELQK

>O22844_ARATH/1-160 PF03101

MFYDDYSRRLGFVMRVMSCRRSEKDGRILARRFGCNKEGHCVSIRGKFGSVRKPRPSTREGCKAMIHVKYDRSGKWVITKFVKEHNHPLVVSPREARHTLDEKDKRIQELTIELRNKKRLCAAYKEQLDAFAKIVEEHSNQIAKKVENVVNNLKEFEHLE

>O49426_ARATH/35-208 PF03101

EFYKEYANSVGFTTIIKASRRSRMTGKFIDAKFVCTRYGSKKEDIDTGLGTDGFNIPQARKRGRINRSSSKTDCKAFLHVKRRQDGRWVVRSLVKEHNHEIFTGQADSLRELSGRRKLEKLNGAIVKEVKSRKLEDGDVERLLNFFTDMQSLRNIFWVDAKGRFDYTCFSDVVS

>Q9S793_ARATH/80-294 PF03101

SFYNSYARELGFAIRVKSSWTKRNSKEKRGAVLCCNCQGFKLLKDAHSRRKETRTGCQAMIRLRLIHFDRWKVDQVKLDHNHSFDPQRAHNSKSHKKSSSSASPATKTNPEPPPHVQVRTIKLYRTLALDTPPALGTSLSSGETSDLSLDHFQSSRRLELRGGFRALQDFFFQIQLSSPNFLYLMDLADDGSLRNVFWIDARARAAYSHFGDVLL

>Q9SFW7_ARATH/11-214 PF03101

LFYKDYAKSVGFGTAKLSSRRSRASKEFIDAKFSCIRYGSKQQSDDAINPRASPKIGCKASMHVKRRPDGKWYVYSFVKEHNHDLLPEQAHYFRSHRNTELVKSNDSRLRRKKNTPLTDCKHLSAYHDLDFIDGYMRNQHDKGRRLVLDTGDAEILLEFLMRMQEENPKFFFAVDFSEDHLLRNVFWVDAKGIEDYKSFSDVVS

>Q9SRR6_ARATH/55-208 PF03101

SFYDNYATCMGFVMRVDAFRRSMRDGTVVWRRLVCNKEGFRRSRPRRSESRKPRAITREGCKALIVVKREKSGTWLVTKFEKEHNHPLLPLSPNDEKDAKIRELSAELSRERRRCTALQQQLDMVLKEMEEHSNHLTININSVIQSVRDIESNT

>Q9SSQ4_ARATH/99-299 PF03101

NYYNCYASEVGFRVRVKNSWFKRRSKEKYGAVLCCSSQGFKRINDVNRVRKETRTGCPAMIRMRQVDSKRWRVVEVTLDHNHLLGCKLYKSVKRKRKCVSSPVSDAKTIKLYRACVVDNGSNVNPNSTLNKKFQNSTGSPDLLNLKRGDSAAIYNYFCRMQLTNPNFFYLMDVNDEGQLRNVFWADAFSKVSCSYFGDVIF

>Q9SU01_ARATH/24-179 PF03101

DFYVEYSKRLGFVVRMMQRRRSGIDGRTLARRLGCNKQGFGPNNQRSSSSSSSSREGCKATILVKMEKSGKWVVTRFIKEHNHSLQFIGSSSYDSFADKERKIKELTEEIECQDRLCDVYRDRLVSFIDNVEHYTEELSLKVRDIVENVKKLECQI

>Q9SWG3_ARATH/65-277 PF03101

IFYQEYAKSMGFTTSIKNSRRSKKTKDFIDAKFACSRYGVTPESESSGSSSRRSTVKKTDCKASMHVKRRPDGKWIIHEFVKDHNHELLPALAYHFRIQRNVKLAEKNNIDILHAVSERTKKMYVEMSRQSGGYKNIGSLLQTDVSSQVDKGRYLALEEGDSQVLLEYFKRIKKENPKFFYAIDLNEDQRLRNLFWADAKSRDDYLSFNDVVS

>Q9SY66_ARATH/62-279 PF03101

EFYSTFAKRCGFSIRRHRTEGKDGVGKGLTRRYFVCHRAGNTPIKTLSEGKPQRNRRSSRCGCQAYLRISKLTELGSTEWRVTGFANHHNHELLEPNQVRFLPAYRSISDADKSRILMFSKTGISVQQMMRLLELEKCVEPGFLPFTEKDVRNLLQSFKKLDPEDENIDFLRMCQSIKEKDPNFKFEFTLDANDKLENIAWSYASSIQSYELFGDAVV

>Q9SZL8_ARATH/87-301 PF03101

AFYNSYARRIGFSTRVSSSRRSRRDGAIIQRQFVCAKEGFRNMNEKRTKDREIKRPRTITRVGCKASLSVKMQDSGKWLVSGFVKDHNHELVPPDQVHCLRSHRQISGPAKTLIDTLQAAGMGPRRIMSALIKEYGGISKVGFTEVDCRNYMRNNRQKSIEGEIQLLLDYLRQMNADNPNFFYSVQGSEDQSVGNVFWADPKAIMDFTHFGDTVT

>Q9ZV55_ARATH/52-221 PF03101

YFYREYARSVGFGITIKASRRSKRSGKFIDVKIACSRFGTKREKATAINPRSCPKTGCKAGLHMKRKEDEKWVIYNFVKEHNHEICPDDFYVSVRGKNKPAGALAIKKGLQLALEEEDLKLLLEHFMEMQDKQPGFFYAVDFDSDKRVRNVFWLDAKAKHDYCSFSDVVL

>Q9ZVC9_ARATH/63-248 PF03101

SFYDEYSRQLGFTSKLLPRTDGSVSVREFVCSSSSKRSKRRLSESCDAMVRIELQGHEKWVVTKFVKEHTHGLASSNMLHCLRPRRHFANSEKSSYQEGVNVPSGMMYVSMDANSRGARNASMATNTKRTIGRDAHNLLEYFKRMQAENPGFFYAVQLDEDNQMSNVFWADSRSRVAYTHFGDTVT

>MAFF_MOUSE/22-116 PF03131

PHLSDEALMGLSVRELNRNLRGLSAEEVTRLKQRRRTLKNRGYAASCRVKRVCQKEELQKQKSELEREVDKLARENAAMRLELDALRGKCEALQG

>MAF_RAT/255-349 PF03131

DRFSDEQLVTMSVRELNRQLRGVSKEEVIRLKQKRRTLKNRGYAQSCRFKRVQQRHVLESEKNQLLQQVDHLKQEISRLVRERDAYKEKYEKLVS

>NRL_HUMAN/130-224 PF03131

ERFSDAALVSMSVRELNRQLRGCGRDEALRLKQRRRTLKNRGYAQACRSKRLQQRRGLEAERARLAAQLDALRAEVARLARERDLYKARCDRLTS

>O42290_CHICK/170-264 PF03131

ERFSDDQLVSMSVRELNRQLRGFSKEEVIRLKQNRRTLKNRGYAQSCRYKRVQQRHILENEKCQLQSQVEQLKQEVSRLAKERDLYKEKYEKLAA

>O73679_BRARE/241-335 PF03131

DRFSDDQLVTMSVRELNRHLRGFTKDEVIRLKQKRRTLKNRGYAQSCRFKRVQQKHLLENEKTQLINQVEQLKQEINRLARERDAYKLKCEKLTG

>Q6IRI7_RAT/22-116 PF03131

PVLSDDELVSMSVRELNQHLRGLTKEEVTRLKQRRRTLKNRGYAASCRIKRVTQKEELERQRVELQQEVEKLARENSSMRLELDALRSKYEALQT

>Q7PRH4_ANOGA/20-116 PF03131

PDISDDELVSITVRDLNRTLKMRGLTREEIVRMKQRRRTLKNRGYAASCRIKRIEQKDELETEKSQEWRDMELMHEETGRLQEENDSLRNKYEALRK

>Q7T3H0_BRARE/22-116 PF03131

PVLSDSELMSLSVRELNMHLRGLSREEVQKLKQRRRTLKNRGYAASCRVKRVSQREALEQQKKELQQEVERLGAENAGMRRELEGLGARLAALQR

>Q93564_CAEEL/1107-1201 PF03131

DHLSDEELAQISVRQLNQKLMGQDRNVVMQWKQKRRTLKNRGYALNCRARRVNNQVQLEADNMMLRNQIKTLREALSEAQMRLHYYEPVFYQAYP

>Q98UK2_BRARE/202-296 PF03131

SRFSDQQLVSMSVRELNRHLRGMSKDDIIRLKQKRRTLKNRGYAQSCRHKRVQQKHLLEHEKTSLATQVEQLKHELGRLVRERDAYKLKCERLVV

>Q9PUA5_XENLA/148-242 PF03131

ERFSDEQLVGMSVRELNRQLRGFSKEEALRLKQRRRTLKNRGYAQSCRYKRVQQRHVLETEKCHLSRQLQQLQQEVARITRERDGWRARYEKLLS

>Q9V958_DROME/20-116 PF03131

PDITDDDLVSISVRDLNRTLKMRGLNREEIVRMKQRRRTLKNRGYAASCRIKRIEQKDELETKKSYEWTELEQMHEDNEQVRREVSNWKNKYKALLQ

>Q9VIW0_DROME/393-487 PF03131

DCLNDDMLTTLTVRELNKRLHGCPREEVVRLKQKRRTLKNRGYAQNCRSKRLHQRHELEKANRVLNQDLHRLKLEYSRVCQERDALMQRLQRAAN

>O76923_DROME/233-440 PF03299

PSEVFCAVPGRLSLLSSTSKYKVTIAEVQRRLSPPECLNASLLGGVLRRAKSKNGGRLLREKLEKIGLNLPAGRRKAANVTLLTSLVEGEATHLAKDFHFVCETEFPARQLAEYIVRHQTEPQDSYRRKELILHSQQITKELMQILSQDRTTHFGTRSQHLLEPSMQRHLTHFSLITHGFGSPAIMAVLHAFQTFLNESLNYLEKLYP

>Q19863_CAEEL/228-434 PF03299

GNVVAEVVDGRLPAVGTRSKYDLTVDELRRRCGAPEHMNQSALYCFFRKSKKKEAINRVKKVLTDYNITVRTMQRQRKVTCFSPFLEEEATALARDLDSITEEFLPIDAIALEMLEKLIFNNKNLDICLRILKNTNKTITRVIKTLEVRQPKITGQKEKLKGNSLDLSYHNFSLTTHGFGHPNSLSHYRSYQKIVEQAALYCEKMQK

>OTX1_BRARE/173-261 PF03529

APPSVSLPEPVAPSNTSCMQRSVSGTASSTYPMPYNQTTGYSQGYPTPSGSYFSGVDCGSYLAPMHSHHHPHQLSPMTASSMPTHPHHH

>OTX1_HUMAN/187-280 PF03529

APASVSVPEPLAAPSNTSCMQRSVAAGAATAAASYPMSYGQGGSYGQGYPTPSSSYFGGVDCSSYLAPMHSHHHPHQLSPMAPSSMAGHHHHHP

>OTX1L_BRARE/180-276 PF03529

APPSLPDISPPASASCMQRAMSSGGGTTGVPSYPMPYNQAPSYAQGYPTSNAASYFGGMDCGSYLAPMTGHAHHHPHAHHSQLSTAAAVSGHHHPHH

>OTX2_HUMAN/153-235 PF03529

SPASISPLSDPLSTSSSCMQRSYPMTYTQASGYSQGYAGSTSYFGGMDCGSYLTPMHHQLPGPGATLSPMGTNAVTSHLNQSP

>OTX2_XENLA/153-235 PF03529

SPASVSPLSDPLSTSSSCMQRSYPMTYTQASGYSQGYAGSTSYFGGMDCGSYLSPMHHQLSGPGATLSPMGTNAVTSHLNQSP

>Q98SI7_XENLA/188-274 PF03529

APGSGPDPLGTGSASCMQRSGSSSAASYPMSYSQAAGYTQAYPAPSSSYFSGVDCSSYLGPMHSHHHPHQLSPMAPSSMSGHHHHHH

>Q9PVM0_XENLA/152-237 PF03529

SPASISPIPDPLSIATTPCMQRSAGYPMTYSQAPAYTQSYGGSSSYFTGLDCGSYLSPMHPQLSAPGSTLSPIASSTMGSHLSQSP

>O23875_ORYSA/40-161 PF03634

SDGATPRRVRPRKSPSSSDRHSKVAGRGRRVRIPAMVAARVFQLTRELGHRTDGETIEWLLRQAEPSIIAATGTGVTPEEAPPAAVAIGSSSVAAAAAAGGHGGAFVHVPYYTALLMQPPNA

>O64647_ARATH/59-280 PF03634

SLAPPSSTGPPLKRASTKDRHTKVEGRGRRIRMPATCAARIFQLTRELGHKSDGETIRWLLENAEPAIIAATGTGTVPAIAMSVNGTLKIPTTTNADSDMGENLMKKKRKRPSNSEYIDISDAVSASSGLAPIATTTTIQPPQALASSTVAQQLLPQGMYPMWAIPSNAMIPTVGAFFLIPQIAGPSNQPQLLAFPAAAASPSSYVAAVQQASTMARPPPLQ

>Q9C518_ARATH/44-302 PF03634

LATTSSTATATTTKRSTKDRHTKVDGRGRRIRMPALCAARVFQLTRELGHKSDGETIEWLLQQAEPAIVAATGTGTIPANFSTLSVSLRSSGSTLSAPPSKSVPLYGALGLTHHQYDEQGGGGVFAAHTSPLLGFHHQLQHHQNQNQNQDPVETIPEGENFSRKRYRSVDLSKENDDRKQNENKSLKESETSGPTAAPMWAVAPPSRSGAGNTFWMLPVPTTAGNQMESSSNNNTAAGHRAPPMWPFVNSAGGGAGGGG

>Q9C758_ARATH/40-241 PF03634

RIIRVSRASGGKDRHSKVLTSKGLRDRRIRLSVATAIQFYDLQDRLGFDQPSKAVEWLINAASDSITDLPLLNTNFDHLDQNQNQTKSACSSGTSESSLLSLSRTEIRGKARERARERTAKDRDKDLQNAHSSFTQLLTGGFDQQPSNRNWTGGSDCFNPVQLQIPNSSSQEPMNHPFSFVPDYNFGISSSSSAINGGYSSR

>Q9CA43_ARATH/74-283 PF03634

VDGSTSQEVQWRRTVKKRDRHSKICTAQGPRDRRMRLSLQIARKFFDLQDMLGFDKASKTIEWLFSKSKTSIKQLKERVAASEGGGKDEHLQVDEKEKDETLKLRVSKRRTKTMESSFKTKESRERARKRARERTMAKMKMRLFETSETISDPHQETREIKITNGVQLLEKENKEQEWSNTNDVHMVEYQMDSVSIIEKFLGLTSDSSSS

>Q9FLM6_ARATH/52-227 PF03634

EDQEIQILYEKEKKKPNKDRHLKVEGRGRRVRLPPLCAARIYQLTKELGHKSDGETLEWLLQHAEPSILSATVNGIKPTESVVSQPPLTADLMICHSVEEASRTQMEANGLWRNETGQTIGGFDLNYGIGFDFNGVPEIGFGDNQTPGLELRLSQVGVLNPQVFQQMGKEQFRVLH

>Q9FTA2_ARATH/15-194 PF03634

EQTSNKGPLNAVKKPPSKDRHSKVDGRGRRIRMPIICAARVFQLTRELGHKSDGQTIEWLLRQAEPSIIAATGTGTTPASFSTASLSTSSPFTLGKRVVRAEEGESGGGGGGGLTVGHTMGTSLMGGGGSGGFWAVPARPDFGQVWSFATGAPPEMVFAQQQQPATLFVRHQQQQQASAA

>Q9FUE7_POPDE/106-351 PF03634

DTSKQPIRQRRTGKDRHSKIHTAQGPRDRRMRLSLQISKKFFDLQDMLGFDKASKTIEWLFTKSKAGIKELTDSVPGGRRRCSICADGKSVCSTPESEVVSGIKLTPETNGDKRGTKAKNDSLVSNPKEKRSKKVHKPVFNLVDRDSREKARARARERTRERMKNQGIDTSSDRSSQANPNKLEKFESSSPLEYGENLAPVNQEMSSPLKVGDVKESSNHDLLPHQMDYVSITGNFFWITNSPRSS

>Q9FYG7_ARATH/77-314 PF03634

EVSKEIKKVVKKDRHSKIQTAQGIRDRRVRLSIGIARQFFDLQDMLGFDKASKTLDWLLKKSRKAIKEVVQAKNLNNDDEDFGNIGGDVEQEEEKEEDDNGDKSFVYGLSPGYGEEEVVCEATKAGIRKKKSELRNISSKGLGAKARGKAKERTKEMMAYDNPETASDITQSEIMDPFKRSIVFNEGEDMTHLFYKEPIEEFDNQESILTNMTLPTKMGQSYNQNNGILMLVDQSSSS

>Q9LEZ9_ARATH/17-195 PF03634

QRWNNPRIVRVSRAFGGKDRHSKVCTVRGLRDRRIRLSVMTAIQVYDLQERLGLSQPSKVIDWLLEVAKNDVDLLPPLQFPPGFHQLNPNLTGLGESFPGVFDLGRTQREALDLEKRKWVNLDHVFDHIDHHNHFSNSIQSNKLYFPTITSSSSSYHYNLGHLQQSLLDQSGNVTVAFS

>Q9LII4_ARATH/132-395 PF03634

ISTSQDPKMKKAKKPSRTDRHSKIKTAKGTRDRRMRLSLDVAKELFGLQDMLGFDKASKTVEWLLTQAKPEIIKIATTLSHHGCFSSGDESHIRPVLGSMDTSSDLCELASMWTVDDRGSNTNTTDKLSYTLETRGNKVDGRSMRGKRKRPEPRTPILKKLSKEERAKARERAKGRTMEKMMMKMKGRSQLVKVVEEDAHDHGEIIKNNNRSQVNRSSFEMTHCEDKIEELCKNDRFAVCNEFIMNKKDHISNESYDLVNYKPN

>Q9LKA2_ARATH/15-280 PF03634

GEIVEVQGGHIVRSTGRKDRHSKVCTAKGPRDRRVRLSAHTAIQFYDVQDRLGFDRPSKAVDWLIKKAKTSIDELAELPPWNPADAIRLAAANAKPRRTTAKTQISPSPPPPQQQQQQQQLQFGVGFNGGGAEHPSNNESSFLPPSMDSDSIADTIKSFFPVIGSSTEAPSNHNLMHNYHHQHPPDLLSRTNSQNQDLRLSLQSFPDGPPSLLHHQHHHHTSASASEPTLFYGQSNPLGFDTSSWEQQSSEFGRIQRLVAWNSGGG

>Q9LSD5_ARATH/62-246 PF03634

NKKSQNQNQLGPKRSSNKDRHTKVEGRGRRIRMPALCAARIFQLTRELGHKSDGETIQWLLQQAEPSIIAATGSGTIPASALASSAATSNHHQGGSLTAGLMISHDLDGGSSSSGRPLNWGIGGGEGVSRSSLPTGLWPNVAGFGSGVPTTGLMSEGAGYRIGFPGFDFPGVGHMSFASILGGNH

>Q9M1U4_ARATH/1-165 PF03634

MDSKNGINNSQKARRTPKDRHLKIGGRDRRIRIPPSVAPQLFRLTKELGFKTDGETVSWLLQNAEPAIFAATGHGVTTTSNEDIQPNRNFPSYTFNGDNISNNVFPCTVVNTGHRQMVFPVSTMTDHAPSTNYSTISDNYNSTFNGNATASDTTSAATTTATTTV

>Q9S7W5_ARATH/58-293 PF03634

MKSKDPRIVRVSRAFGGKDRHSKVCTLRGLRDRRVRLSVPTAIQLYDLQERLGVDQPSKAVDWLLDAAKEEIDELPPLPISPENFSIFNHHQSFLNLGQRPGQDPTQLGFKINGCVQKSTTTSREENDREKGENDVVYTNNHHVGSYGTYHNLEHHHHHHQHLSLQADYHSHQLHSLVPFPSQILVCPMTTSPTTTTIQSLFPSSSSAGSGTMETLDPRQMVSHFQMPLMGNSSSS

>Q9SJK7_ARATH/27-186 PF03634

GVSKSSSSGGGCISARTKDRHTKVNGRSRRVTMPALAAARIFQLTRELGHKTEGETIEWLLSQAEPSIIAATGYGTKLISNWVDVAADDSSSSSSMTSPQTQTQTPQSPSCRLDLCQPIGIQYPVNGYSHMPFTAMLLEPMTTTAESEVEIAEEEERRRR

>Q9XGR9_LINVU/79-321 PF03634

DPANMAETFQTKQSTVKKDRHSKIYTAQGPRDRRVRLSIGIARKFFDLQEMLGFDKPSKTLDWLLTKSKTAIKELVQSKSTKSNSSNSHSDDDCDDEVVSAEGDSENAADSKGKSVLIKGNYKCKEAASAMDSQQAALNLVKESRAKARARARERTKEKMCIKQQLNEARNNNKGGDWINNPFNNVIQSNRNHQQFESREAAFVHHPVFGFHQQNYSNASASSHENWDQSNQLCAILNQHKFI

>DNB2_ADE12/349-474 PF03728

VRCECLNKQDGLPRMGRQLCKITPFNLSNVDNIDINEVTDPGALASIKYPCLLVFQCANPVYRNARGNAGPNCDFKISAPDVMGALQLVRQLWGENFDGSPPRLVIPEFKWHQRLQYRNISLPTNH

>DNB2_ADE41/341-466 PF03728

LRCECLNKKDLVPQLGRQMCKVTPFALSGAEDLKTSEVTDKSALASILHPCVLVFQCANPVYRNSRGSAGPNCDFKISAPDVISALQLVRQFWKENVEDPLPKLIIPEFKWSTRLQYRNVALPTGH

>Q83907_ADEO7/103-217 PF03728

SGCYLWYHDWEEDRPRCFHGDFMFKRVNEIEMPPTSEAGVQALKEGRGVLSNGRMNKQVVKIVQEHYVLCAEDAHQRFGQCSPRSCGLNFSDEKKALLAMQNAIETTKAVFPKAK

>Q9YUQ9_9ADEN/203-337 PF03728

TKCFCNYGHENIQLGRQICKMTAFEIPGANDIDPESCHDDMLLATAKYKHTFVFQCCNPIRLKRNAKDKDNQTHKHCDFKLSMIDVRQAMKISKDIYTKLKETIDDGSPTKIMLPAFVFNPKKHSFKQAIVAQHE

>Q9RPY7_PSEAE/13-62 PF03869

SRTADKFVVRLPEGMREQIAEVARSHHRSMNSEIIARLEQSLLQEGALQD

>RARC_BPP22/4-53 PF03869

MSKMPQFNLRWPREVLDLVRKVAEENGRSVNSEIYQRVMESFKKEGRIGA

>RMNT_BPP22/1-50 PF03869

ARDDPHFNFRMPMEVREKLKFRAEANGRSMNSELLQIVQDALSKPSPVTG

>CAT8_YEAST/518-619 PF04082

SLPSLQLLSLASFYYLNVGDISAIYGVRGRIVSMAQQLRLHRCPSAVLSVHSNPVLQKFEQSERRLLFWAIYYVDVFASLQLGVPRLLKDFDIECALPISDV

>CTF1A_FUSSO/337-431 PF04082

RVTIVQSLLLMGWYWEGPEDVTKNVFYWSRVATIVAQGSGMHRSVEQSQLSRSDKRLWKRIWWTLFTRDRSVAVALGRPVHINLDDADVEMLTED

>CTF1B_FUSSO/342-437 PF04082

RLVLVQALLLMTYWYETPDDQKDTWHWMGVAISLAHTIGLHRNPGSTSMAPAKQKLWKRIWWSCFMRDRLIALGMRRPTRIKDEDFDVPMLEESDF

>DAL81_YEAST/397-491 PF04082

GRPKLSIIQTGLLILQCRSECHNNWVLCSSVVALAEELGLGVECNDWKLPKWEKDLRKRLAWAVWLMDKWCALNEGRQSHLILGRNWMIKLLNFD

>GAL4_YEAST/322-416 PF04082

SIILVTALHLLSRYTQWRQKTNTSYNFHSFSIRMAISLGLNRDLPSSFSDSSILEQRRRIWWSVYSWEIQLSLLYGRSIQLSQNTISFPSSVDDV

>MAL13_YEAST/169-269 PF04082

DLDNSNIFNIMTYYCLHRSFAQISNARTSYRLCCEAVGLITVAGLHREETYGSLTFEEQQLRRKLYYLLLMTERYYAIYLHCATSLDATIAPPQLELVTDP

>MAL33_YEAST/164-264 PF04082

DLSGRDIFRIMTYYCLLRCFSQSSDVRNSYRLCREAIGLVIVAGLHREKAYESLSFREQQLLRKVYYLLLLTERYYSVYVHCVTSLDTTIAPPQPEFVTDP

>MAL63_YEAST/166-267 PF04082

DLSNSDIFRIMTYYCLHRCYAQFADTRTSYRLSCEAVGLIIKIAGFHREETYEFLPFGEQQLRRKVYYLLLMTERFYAVYIKCVTSLDATIAPPLPEVVTDP

>O13414_ASPNG/290-386 PF04082

SITTMQCYVLAQMYCMTKGDYTSLLRYRGLAVSLSQQLRLHQSQKRFSSNALVAETRKKVFWCQYTLDRFTAALTGLPVLLREEDIKTEYPDDIDDE

>O13461_EMENI/292-388 PF04082

SIPTLQCYVLAQMYCLTKGDYTNVLRYRGVAVGVCHQLKLQKSQKAFSSNALLAETRKKVFWCQYVLDRLCASLTGLPVLLREEHIQTEYPEDIDDE

>O60201_ASPOR/256-356 PF04082

VVDEINMENLLTSFFLFAAYGNLDRQDQAWFYLCQTTSMVFTLGLQRESTYSKLSVEEAEEKRRVFWLLFVTERGYALQQAKPVMLRNSIHKPQVLCSDDP

>O74541_SCHPO/256-354 PF04082

HITSAQCLLCLGFYKIAMGNTSHGWLLCGMAFRMGQDLGFHLDPRDWHINNVPVVSEEQAALRSRIYWGCYVADVFVSFILGRPTTLSKSDTSVPTSDD

>O74915_SCHPO/283-383 PF04082

HLTSVQCLLCLGYYDIGMGNTSLGWLLSGLAFRMGQDLGFQLNPENWYIDNSPAISSADSDIRRRVFWGSYVADKFIGFIMGRPTMLKRSDASIPGSNQLP

>O93984_PICAN/225-321 PF04082

DEYLLQGLLLLSCYEMGMGNDSMSYLYISMACSLTQHMGLHISYDEHSSAAKFAPRTTPFQSALLWSICTQDRIITSRIGVPSCIHFKRIISPVYEV

>O94490_SCHPO/385-477 PF04082

SLETVQSFFLAGMYLSPTLAHEVVYMYFGIAMRAAVANGMHKKSANAQFSGDVAELRKRLFWSVYCMERKIGISLGRPESLVRSEIDIHFPEY

>PDR1_YEAST/462-563 PF04082

VDFTCDITHLEQLLYFLDLLFWLSEIYGFEKVLNVAVHFVSRVGLSRWEFYVGLDENFAERRRNLWWKAFYFEKTLASKLGYPSNIDDSKINCLLPKNFRDV

>PPR1_YEAST/437-533 PF04082

RLEALAGTLLMVIYSIMRPNVPGVWYTMGSVLRLTVDLGLHSEKINKNYDAFTREIRRRLFWCVYSLDRQICSYFGRPFGIPEESITTRYPSLLDDS

>PRIB_LENED/299-400 PF04082

DEYAVQAFACLTYWKGHDDNRTSTWTFVGYACRMRVEIGLNRYVPNVPSNETELQRLERRNRERTYLVLFIHDRSLSTQTGRHWMLPEDDFIRHSDRWHESS

>PUT3_YEAST/457-553 PF04082

KKGGIEVLLLYAFFLQVADYTLASYFYFGQALRTCLILGLHVDSQSDTLSRYEIEHHRRLWWTVYMFERMLSSKAGLPLSFTDYTISTALPADIDDE

>Q12180_YEAST/469-565 PF04082

GTRTLQALLLLNRYFQLTYDTELANCILGTAIRLAVDMELNRKSSYKSLDFEEAIRRRRMWWHCFCTDKLYSLMLSRPPIVGERDMDMLTDQNYYEV

>Q9HE23_NEUCR/497-602 PF04082

RLDSVQARLLQDLYLLSTCRLNKAWYTFGNTLQMITSLGLHRRVGRNRGLGRDITKRPDYAKLQCERRTFWTGYIIDKQLSMVFGRPSHFRDDFINQELPDAVNDE

>Q9P7H9_SCHPO/387-482 PF04082

SLSRLSTLQAAIIFLTGRPWINVAGNWSILTRAIALAQILGFHLDCSEWQIPKEEKILRNRVWWALFVNEKWLSMYIGINASIRQDDYLVPPLTDN

>Q9UTA7_SCHPO/313-409 PF04082

TQPRLSTIQAALLYLISRPLHNMYSLSSILSRTTVLSQLLGFNHDCTEWKIPNEEKTIRKRIWWAIFIADKWYSMYFGLATNIHEDDFVVPKIESDE

>Q9UVH4_SACPS/164-264 PF04082

DLSNSDIFRIMTYYCLHRCYAQFADTRTSYRLSCEAIGIIKMGGFHREETYEFLPFGEQQLRRKVYYLLLMTERYYAVYFHVVASLDAIVAPPLPEIVTDP

>Q9Y8A1_ASPNG/254-354 PF04082

PVDGMNIESLLTSFFLFASYGNLDKQDHAWFYLCQATSMVFTLALHRESSYVDLSTEEAEERRKVFWLLFVTERGYALQQSKPVMLRNSIRKPQVLCSEDP

>QA1F_NEUCR/372-465 PF04082

QVQHCRALLLLCLVSLGRDDWESAWLLVGFAVRVLLVVRTQLPPDDDRPRPRMRALLVACFIVDTIVSMRHNVPAHLKPDDIADLPLPEDGQDQ

>RDR1_YEAST/246-350 PF04082

TVEQVSAWVLRTIYLRATSRPHVAWLASCVTIHLSEAIGLHHEIDREDIAISNNVPPKRTTVVSEHTRRLFWCAWSINTILSYDYGRSSVTLNRITCKPVKETDG

>SEF1_KLULA/410-512 PF04082

STHISQALLILCNWPLPNQKVLDDCSYRFVGLAKSLSFQLGLHRGKFMTEFTRTQTSLPEAEKWRTRTWLGIFFAEQLWASILGLPPTSQTDYLIEKARLGDD

>SEF1_YEAST/421-524 PF04082

STHISQALLILCIWPLPNQKVLDDCSYRFVGLAKSLSYQLGLHRGEFISEFTRTQTSMPNAEKWRTRTWLGIFFAELCWASILGLPPTSQTDYLLEKALSCGDE

>STB4_YEAST/512-609 PF04082

SFELIQSWLLITFYFRTCYRQTACWNALSQAVNMCNGMSLYLNKFPEIHSTYDESKAWHCFWCCFIMDKLISFQMGRFYQLSLPASEMCEQMNLVKSK

>STB5_YEAST/392-492 PF04082

DIETVRCLLLLGIYSFFEPKGSSSWTISGIIMRLTIGLGLNRELTAKKLKSMSALEAEARYRVFWSAYCFERLVCTSLGRISGIDDEDITVPLPRALYVDE

>SUC1_CANAL/166-265 PF04082

YQMTPTLETILTSFFLHVAEVNKGSKPAAIIYLREAITMAQIIGLHNESTYKSKPVAEAHRMRKIYFMLMVTERFMCIDDLIPVVLENSIKEFSLDDEQY

>THI1_SCHPO/339-435 PF04082

DVSAVQSSLLIGLYLQSTIYEKSSFAYFGLAIKFAVALGLHKNSDDPSLTQNSKELRNRLLWSVFCIDRFVSMTTGRRPSIPLECISIPYPVILPDL

>XLNR_ASPNG/481-576 PF04082

ELKLGRELPPNVSHARQDGERDGDGEADKRHPPTLITSLGHGSGSSGINVTEEEREERRRLWWLLYATDRHLALCYNRPLTLLDKECGGLLQPMND

>YAO7_SCHPO/249-344 PF04082

SIDKVQALIVMTQYAAYLSSSSLCRTLCGQACLMAQQLNLHRKQSTDVEPEKAESWKRIFWMCYILDKNISLIFGTPSVFNDKDIDCNLPDSKYEL

>YB00_YEAST/464-556 PF04082

DMTALKALLLLAKFAQQKISASSAVKVLSVAIKVALDLRLNLHSTYEDLELDEIIKRRRLWCYCFSTDKFFSVVLSRPPFLKEENTDVLTDES

>YE14_YEAST/310-410 PF04082

LFSTMYDLTILKGLVSLMKHRYWIDDPFVLGRIISTMSRRSLDAGLNRWEYYIGQDEDTAEEYRKLWWDCYWWDRWYSLVTGKQPLIPHEMTSCLFPKDVV

>YFF2_YEAST/159-259 PF04082

DFNGSDIFKIMTYYCLNRCYAQMSNSRTSYRLSCEAVGLIKLAGFHREETLKLLPFDEQQLGRKVYYLLLLTERYFSVYTHCATSLDTTIAPPQPENVTDP

>YHDD_SCHPO/261-357 PF04082

DITTLQTLLVLGIREIGRGLTFKGWLFSGMAFRLVYDMGLHLDPDHWDHSEESRIDREVRRRCFWGCFTLDKLISLCYGRPPGLYLKQTDVRNTTQL

>YKW2_YEAST/291-392 PF04082

YTERVQFLFLRYLYINVAGLDGGDQSHCIFIHGLTIDTAIHMGLNEDLRRLYLSKNHPIEEIPYLERLWLWILFTDVKISLSTGIPVRINDDFVNKVRLENY

>YL278_YEAST/417-509 PF04082

YVQQIELLTLLVLYIIRTDRDSLILYDIIKDVMGISKKKLHLNQWYPNDPFANKKLRLFWCVYLLERMICVAVGKPYTIKESEINLPLFNNDS

>MRE11_CAEEL/311-533 PF04152

YVASSEAVGDGFYILQPGSTVATSLTPEEALQKNAFLIKIKGRKFASKPIPLQTVRPMVCDELLLDKIPPGSRPILKTDRPKHTDGRYIDEIAIEAKINEMITTAKAKRRPRQPELPLIRLKVIYDGDWLNITPANAKRIGLRYENVVANAVDMVFIKKNNKPKEGKLQTENEKNITEMADEMGQVSATNLQTIINDYFINQPLVDQMTVLKPIGIGRALEQY

>MRE11_CHICK/250-462 PF04152

KITPAQNEQQHFYVTQPGSSVVTSLSPGEAVKKHIGLLRVKGKKMKMQRIALETVRTFYMEDVVLADHPELFNPDNPKVTQAIQAFCMEKVEMMLDNAERERLGNPRQPQKPLIILRVDYTGGFEPFIVHRFSQKYMDRVANPKDIIHFFRHREQKEKNDNDINFGKLLSRPASEEVTLRVEDLVKQYFQTAEKKVQLSLLTERRMGEAVQEF

>MRE11_MOUSE/250-462 PF04152

KIGPIKNEQQLFYVSQPGSSVVTSLSPGEAVKKHVGLLRIKGRKMNMQKLPLRTVRRFFIEDVVLANHPNLFNPDNPKVTQAIQSFCLEKIEEMLDSAERERLGNPQQPGKPLIRLRVDYSGGFEPFNVLRFSQKFVDRVANPKDVIHFFRHREQKGKTGEEINFGMLITKPASEGATLRVEDLVKQYFQTAEKNVQLSLLTERGMGEAVQEF

>MRE11_XENLA/251-464 PF04152

KIAPTRNEQQLFYVSQPGSSVATSLSPGEAEKKHVGLLRIKGKKMNMQKIPLQTVRQFFIEDLVLSDYPDIFNPDNPRVTQEIETFCIEKVEAMLDTAERERLGNPRQPDKPLIRLRVDYTGGFEPFNTLRFSQKFVDRTANPKDIIHFFRHKEQKDKKDSITINFGKIDSKPLLEGTTLRVEDLVKEYFKTAEKNVQLSLLTERGMGEAVQEF

>Q9GZJ8_BOMMO/249-457 PF04152

PMKGNKTEKDSFFVVQPGSTVATSLAAGEALPKHCGLLEIHKGNFKLTPLPLQTVRPFIFKTIVLSEENIGSDDVNENEKVQEFLKNRVNEAIDEASKLKTADLRQPLLPLIRLSIFYERESQNFNRIRFGQNFNGLVANPNDLLIMKKEKKIREKRECDPEEEGDMTGVAAEAADVESLLRAYYEAQPKDKRLSVLSVRVITDAVRDF

>Q9XYZ4_DROME/263-474 PF04152

RIEPEENAKKRFYVSQPGSSVPTSLSEGEAKKKHVGLLEIYKGKFKLKPLPLETVRPFVYESVVLADHADELGLVEGDASTKVFKFAQERVEAMIERAVAQHTGHPKQPTLPLIRLRLLYTDESCMFNAIRFGEMLSTRVANVQDVVQFSKVVKRTKTEAVNLDKEALRRALEADNATRVEELVDRYFEEAKSNKPLKLFHSKALAEMTYRL

>JERKY_MOUSE/14-66 PF04218

KRKRVVLTLKEKIDICTRLERGESRKALMQEYNVGMSTLYDIKAHKAQLLRFF

>P91673_DROME/7-60 PF04218

RKPRTSLTLEEKMEVIQSQERNKLSVRDLAKRFNIGKTQAADILKHKQSIKEGL

>Q9BXZ0_HUMAN/4-56 PF04218

RGKYTTLNLEEKMKVLSRIEAGRSLKSVMDEFGISKSTFYDIKKNKKLILDFV

>Q9VP44_DROME/27-80 PF04218

RNERNILTFYEKIAVIRYYDETNISRNSLAKMFHCCATQIRRILDKKKDLLQQL

>LYTR_STAAU/147-244 PF04397

DKIHMLKQQNIIGIGTHNGITTIHTTNHKYETTEPLNRYEKRLNPTYFIRIHRSYIINTKHIKEVQQWFNYTYMVILTNGVKMQVGRSFMKDFKASIG

>LYTR_STAES/153-250 PF04397

ERIHILNFTDIIALSVNNGITTIDTTKQSYETTETLNHYEKKLPSSLFIKIHRATIVNKEHIQTIEHWFNYTYQLTLTHEFKYQVSRSYMKTFKQQLG

>LYTR_STRA5/148-245 PF04397

DRSVVVKMQDIVAASVEDGELTVSTVQKTYTIRKTLNWFKSRAVAPYFLQIHRNTVINLEMIEEIQPWFNHTLLLIMSNGEKFPVGRSYLKDLNEHLT

>LYTR_STRMU/146-243 PF04397

DRSIVLKMPDIVAASIEDGELTVSTKNTSYTIKKTLNWFKTRAKTNYFLQIHRNTVVNLEMIQEIQPWFNHTLLLVMVNGEKFPVGRSYMKELNAHLT

>LYTT_BACCR/148-245 PF04397

ESIVLVNIEDIVYVGLVDGKVTVKTMRETYVTHDTLVILEKKLPQVSFMRVHRSFIANINHITEIQPWFNSTYNLIMKEGSKVPVSRTYAKELKKLLR

>LYTT_BACSU/143-240 PF04397

ESIVIVDTKDIIYAGTEDGHVNVKTFDHSYTVSDTLVVIEKKLPDSDFIRVHRSFVVNTEYIKEIQPWFNSTYNLIMKDGSKIPVSRTYAKELKKLLH

>LYTT_ENTFA/143-240 PF04397

DRIYLVAPEDIYLVSVEERQLSIFVDQQVYKMTGTLNSIEQKLPATLFIKTHRSFILNRTKIQEIQPWFNNTLQVILTNGSKVPVSRSYVKEFKEKLG

>Q892V7_CLOTE/158-254 PF04397

GKIILVPLSQIYFCYTEKDKVYVKTYNKEYISNYTLNKLQKKTKFFRAHRSYLVNLNNIKELFSWFNGTYKLVMKDDKNTEIPISRGNVKHLKDILD

>Q8DZT1_STRA5/144-241 PF04397

DRIYLVSADDILLIEAMQGKLIIQTPDKNYEIDGSLQQWQDKLPSSQFVRVHRSYIVNINAIKTIEPWFNQTLQLHLCNKITVPVSRANVKPLKQMLG

>Q92UV1_RHIME/150-249 PF04397

GQTLVLPTDGIAMLAAEADFTRIVMADARDHLVCRLLGQFDAELPTPPFIRVSRSLIINLDRVVRVRSQDKGRSLLSLGRSLTPIMLGRVATTRLRRALD

>Q97IH1_CLOAB/156-252 PF04397

GKIVLVDVNDIYFFYIKSEKTYVKLNDIIYVTSSTLNDMEKKTGFFRAHRSYLVNLNKIFEIYSWFNGTYKLIMSDYRKSEIPVSRGNVRKLKEIIN

>Q9CE27_LACLA/141-236 PF04397

SGYPISIDSLLYIEADKTHRNYLRAITKDNECLVRGTISDVKKKIPNLLALGRGLLVDTNKIRRIEEDGLTLCFKQSLKKVKAPAVCKKELKELKK

>Q9ETK5_STRPN/10-108 PF04397

KTVYKVNIDDIYYIQTHPTKAHTVQIVTEEASFNMLQNLSNLENQCGETLMRCHRNCLVNLDKLKSIDFQERILFLGEEGQYAVKYARRRYREIRQKWL

>Q9KWM3_STAWA/142-241 PF04397

KSLYFLEIDKITTAECCGRKIIITLDDKETVSYNGSLKYLEKKLYSYGFILVSRSLLIPLKSIKNIIFDKHSKCYILKSIKNNTVKITPEKYKKVKQTLS

>Q9KX23_OLICA/286-384 PF04397

GQRCTMLISRLFAVQPQAHYTLLFDGEATWFCPLSLSQVAKVLDSANFAQVHRSHIIDLDRFRLVRGTGNGGMFEAISKTPYKVPLSRARRAWIKQQLQ

>Q9ZGQ0_BUTFI/136-230 PF04397

GENHFFKESTISYVEGMGKNCILHFCDGREKMECRETLGAIEARLSSKKFYRCYKSYLINLAQVDSYNHEEVTMSTGEKLLISRLKYKEFNNIYA

>O94619_SCHPO/157-606 PF04503

GSEPAKAYMSHISNLRKKSRLNLQEIQKNSLHTGNTSHPYANASFPHDPANAMGQQIDSSQFHQGAGGLNDRNQHLMRQAMLNNQSRETFPPTAAQLQQLKQLHYRQLQSVQQQQKQHQQKKTPQSGSTPQMQNTTSQPTTHDTHPPKQQGPISDFRSIPSSPKTEGAPSNAQFRPSLPATPNGSVPQSNPLYDTTGLNGGQYPVVQNSAQPLLHEINFASNRNPHLKQGGAVPSSTLPQQQKSLDKPKPAQQPSTGQFSGNQMNQYGFSNSPYSQNMLYNFNGNANPSRLNPALKNYMEELKLLEQQNKKRLLLVSQEKERKGYTSASPDRPLSQTITESSVAKTKSTTPKSTDTPTEATTSPVKVSTKNSNTTENLNGINESNMPMLQNGLPLRTSGDHPSNYSNLIENSSTSDTNNADNGMDVMGNWQLQQTHSSRPTPNASSPLDV

>Q9BWW5_HUMAN/81-295 PF04503

CEHSGEAKAFQDYSAAAAPSPVMGSMAPGDTMAAGSMAAGFFQPFMSPRFPGGPRPTLRMPSQPPAGLPGSQPLLPGAMEPSPRAQGHPSMGGPMQRVTPPRGMASVGPQSYGGGMRPPPNSLAGPGLPAMNMGPGVRGPWASPSGNSIPYSSSSPGSYTGPPGGGGPPGTPIMPSPGDSTNSSENMYTIMNPIGQGAGRANFPLGPGPEGPMAA

>Q9U291_CAEEL/91-234 PF04503

GGDPFSAEAKYFHEAMIGMPPGMNGHFAPPPMGMEMMGGHPGAFGGRFAPGRMPPGAMAPGGMPPGAFPMFPPDPRLQRMAPNQGMRMPPPPVGQPFPGAVGMPRPVGPGAPMVTYLKNRKIEDFSKTKSIHIFRKNNKIRSFL

>SSBP3_CHICK/80-302 PF04503

CEHSSEAKAFHDYSAAAAPSPVLGNIPPNDGMPGGPIPPGFFQGPPGSQPSPHAQPPPHNPNSMMGPHSQPPGAVPGTQPLLPNSMDPTRQQGHPNMGGPMQRMNPPRGMGPMGPGPQNYGSGMRPPPNSLGPGMPGINMGPGAGRPWPNPSSANSIPYSSSSPGTYVGPPGGGGPPGTPIMPSPADSTNSSDNIYTMINPVPPAGSRSNFPMGPGSDGPMGG

>ELF1_DROME/870-1093 PF04516

STNGSTRSRPWHDFGRQNDADKIQIPKIFTNVGFRYHLESPISSSQRREDDRITYINKGQFYGITLEYVHDAEKPIKNTTVKSVIMLMFREEKSPEDEIKAWQFWHSRQHSVKQRILDADTKNSVGLVGCIEEVSHNAIAVYWNPLESSAKINIAVQCLSTDFSSQKGVKGLPLHVQIDTFEDPRDTAVFHRGYCQIKVFCDKGAERKTRDEERRAAKRKMTAT

>Q12800_HUMAN/37-264 PF04516

AYSMSDVLALPIFKQEESSLPPDNENKILPFQYVLCAATSPAVKLHDETLTYLNQGQSYEIRMLDNRKLGELPEINGKLVKSIFRVVFHDRRLQYTEHQQLEGWRWNRPGDRILDIDIPMSVGIIDPRANPTQLNTVEFLWDPAKRTSVFIQVHCISTEFTMRKHGGEKGVPFRVQIDTFKENENGEYTEHLHSASCQIKVFKPKGADRKQKTDREKMEKRTPHEKEK

>Q6GMM0_BRARE/18-240 PF04516

TYIRDALAPFLKHEEEHQAAEIGASPFHYVLCAATSPAVKLHDETLTYLNQGQSYEIRMLNGKLVEYTDVSSKYVKSIVRVVFHDRRLQYTEHQQLEEWRWNRPGDRILDIDIPLSVGIIEPNAHPLQLNTIEFLWDPDKNASVFIQVNCISTEFTPRKHGGEKGVPFRIQIDTFTASAHGEYTEHMCSASCQVKVFKPKGADRKLKTDREKIEKKSLQDKEK

>Q6ISB3_HUMAN/210-440 PF04516

STPDSTYSESFKDAATEKFRSASVGAEEYMYDQTSSGTFQYTLEATKSLRQKQGEGPMTYLNKGQFYAITLSETGDNKCFRHPISKVRSVVMVVFSEDKNRDEQLKYWKYWHSRQHTAKQRVLDIADYKESFNTIGNIEEIAYNAVSFTWDVNEEAKIFITVNCLSTDFSSQKGVKGLPLMIQIDTYSYNNRSNKPIHRAYCQIKVFCDKGAERKIRDEERKQNRKKGKGQ

>Q6NWN8_HUMAN/216-444 PF04516

TFKEGVQEVFFPSDLSLRMPGMNSEDYVFDSVSGNNFEYTLEASKSLRQKPGDSTMTYLNKGQFYPITLKEVSSSEGIHHPISKVRSVIMVVFAEDKSREDQLRHWKYWHSRQHTAKQRCIDIADYKESFNTISNIEEIAYNAISFTWDINDEAKVFISVNCLSTDFSSQKGVKGLPLNIQVDTYSYNNRSNKPVHRAYCQIKVFCDKGAERKIRDEERKQSKRKVSDV

>Q99PF2_MOUSE/18-246 PF04516

SYLRDVLALPIFKQEEPQLSPENGARLPPLQYVLCAATSPAVRLHEETLTYLNQGQSYEIRLLENRKLGDFQDLNTKYVKSIIRVVFHDRRLQYTEYQQLEGWRWSRPGDRILDIDIPLSVGILDPRASPTQLNAVEFLWDPSKRASAFIQVHCISTEFTPRKHGGEKGVPFRVQIDAFKQNEKWGLLGASTLLPACQIKGFKAQGELIGNKKTDREKMEKRTAQEKEK

>Q9N3N7_CAEEL/170-389 PF04516

SMLERSSERTLSHRDSPLVIPKLYNNLGFQYVLEAPISTSVRRDDDRMTYVNKGQFYTVSLEYTPDLNKCLKSQTVKSQLMVVFREDKTYEEEIKTWQSWHARQHVSKQRILEIDSKNSSGMIGQIEEIGNNAVQFYWNPSDPSGVRISIAVQCLSTDFSTQKGVKGLPLHVQIDTYDGENDKVPFHRGYCQIKVFCDKGAERKLRDEDKRAQKRKVQEY

>O51406_BORBU/263-415 PF04552

TSENPQKQKKAKWLIESLRYRDEILAKIGIAIYTLQKEFLRRGFKSLRPMNLSILSEKISVSKSTISRAIKNKYLKCEWGTILIKELFSSVGGAKTNEFSKLSIKITVKKLLEANKKMSDKEISVILKSKGISISRRTVNKYRNELKSEKGRT

>O54393_MYXXA/344-503 PF04552

KDFIQDKLRSAMWLIRSIHQRQRTIYKVTESIVKFQRDFLDKGIAYLKPLILRDVAEDIGMHESTVSRVTTSKYVHTPQGIFELKYFFNSSIARVSGEDTASEAVKHHIKQLVAQEDARNPYSDQKIVELLRSQGTEIARRTVAKYREVLGILPSSKRKR

>O66858_AQUAE/244-398 PF04552

QKELKEAFERYESIRKVLDIRRRNLRKVLEKIVERQKDFLTGKGSLKPLTLREVSSEIGIHESTLSRIVNSKYVKTPVGTYSLRTFFVRESAEGLTQGELMKLIKEIVENEDKRKPYSDQEIANILKEKGFKVARRTVAKYREMLGIPSSRERRI

>O69080_PLALI/335-494 PF04552

REYIKRKVESAKWLIESIEQRHNTLKRVAQAIVDHQIEFLEKGPEFISPLKMQQIADIVKVHVTTVSRAVDDKWIQTPRGLFPLKRFFGGGTTTAEGEEVAWHIIRLKLKEIVDGENKDDPLSDDALVEELAKHGFNLARRTVTKYRKALNIPSSRQRRS

>O69778_RHIET/312-472 PF04552

QSFLNECFQSASWLVRSLDQRATTILKVATEIVRHQDVFLEDGIAHLRPLNLKTIADAIDMHESTVSRVTSNKYILTPRGVFELKYFFTVAIPSSQGGDAHSAEAVRHQIRALIAAETLHDVLSDDGIVAKLKETGVDIARRTVAKYREAMRIPSSTQRRR

>Q59085_AZOBR/359-519 PF04552

KEYITERFQSANWLVKSLHQRATTILKVASEIIRQQDAFFIHGVSHLKPLILRDIAEAIGMHESTVSRVTTNKFMATPRGVFELKYFFTSAIQGADGQAAHSAEAVRYRIKAMIDAEKPDDVLSDDKIVEILRGEGIDIARRTVAKYREAMRIPSSVQRRR

>Q98AS6_RHILO/303-463 PF04552

QSFLNECLQNANWLIRSLDQRAKTILKVAAEIIRQQDAFFEHGVAHLRPLNLRTVADAINVHQSTVSRVTSNKYMLTPRGVFELKYFFTVAIGSSEGGDAYSAEAVRHQIKAMVAVESPNEVLSDDDIATRLKETGIDIARRTVAKYREALNIPSSARRRR

>Q98GS5_RHILO/338-498 PF04552

KDFLAECLQNANWLTRSLDQRAKTILKVASEIVRQQDAFLVHGVRQLKPLNLRTVADAIGMHESTVSRVTANKYMLTPRGVFELRYFFTASIAASGGGDAHSSEAVRDRIKQLIDEEKPVDVLSDDAIVDMLKESGVDIARRTVAKYREGMNIPSSVQRRR

>Q9F0Q3_ENTFA/280-441 PF04552

QEYIKDKKNEFEWLERAVNQRGDTILRVGQEIVRRQEAFFLEADRPLKPMTLKEIADALSIHESTVSRAVNGKYLETTFGVFELRSFFSTSILSSEEDGEDVSTTMVKKQLQKLIDQEVKSKPLSDQKLVERLKDSGMEISRRTVAKYREALGIPSSSKRKR

>Q9F1E7_ACEDI/298-457 PF04552

RTYLNDTLTSANWLIRALQQRSMTILRVSTEILRRQEDFLQHGPQALQPLNLRTVAEALNIHESTVSRVTANKYVATPRGVLPLKFFFVAAMTGNDGEIRSNIAIQSVIRRMIQGERPDAVLSDEAISLTLRRQGIDIARRTVAKYREAMGFPNSLQRLQ

>Q9K710_BACHD/282-443 PF04552

AQYAKEKYHQMQWLVKSIQQRQQTLLKVTEAILRKQRAFFEEKEGALQPLTLREVAEEIGVHESTVSRATTNKYAQTPRGLLELKSFFVSSISSRFGEKGPSSDSVKKLVRKLVEEENKAKPLSDQKIAHLLKDQYNVEASRRVIAKYRDELGIPSSTKRKR

>Q9KP49_VIBCH/326-485 PF04552

SNYIRTNLQEAKWLIKSLESRNETLLKVAKCIVEHQHDFFEYGEEAMKPMVLNDVAMAVEMHESTISRVTTQKYMHTPRGIFELKYFFSSHVSTDNGGECSSTAIRALIKKLVAAENPAKPLSDSKIATLLADQGIQVARRTIAKYRESLGIAPSSQRKR

>Q9PDH1_XYLFA/301-460 PF04552

ASYLRNQLQEARWLLKGLEARGETLLKVVNSLIRHQTGFLEFGQHALRPLTIRELATELALHESTVSRAIAGKYVRTPRGTLPLRTFFASGISTDSGGETSNSAIQAIIRRLIETENPRKPLSDAKLAELLKTSGIPVARRTIAKYRDAMNISASHERVR

>Q9PJD0_CHLMU/264-419 PF04552

KDHLSQQIRAAKQLLRNIKKREETLLAVLRVLIPYQEEFLLKKRSVPKAFSVKQLARELSLHETTICRAINNKTLATPIGLISMRSLFPQAVGACPDQSKATILHWIHQWISTEKNPLSDEAISQKIIAKGIPCARRTVAKYRSQLNIPPAHQRKH

>Q9PPN1_CAMJE/254-410 PF04552

HDFLSHYIKEAKNLVDALAMRKATLYKIGLMIVEYQYDFFMGKEIKPMTFKDLALDLERNASTISRAVANKYLSCERGLIPLRDFFAFALDEEGETSNVGVKEFVANLVKNEDRNKPLSDSKILELIKEEFKVDIGRRTITKYRKHLNIASSTDRKK

>Q9RII3_RHOSH/270-426 PF04552

TPAARAAWTQAQAVGRMIENRNATLLRVAREILARQEAALDEGPSALVALTMTEVAEALGIHESTVSRVVAGTCVDTPRGTWWLRRMFSGRLAEGGPSAAAIRAAIARLVAQEDPAAPLSDGALVEALAAEDMQLARRTVAKYREMLNIPPGHRRRR

>Q9Z7D5_CHLPN/269-424 PF04552

QKNLSQQILSAKWLIKNLRKREQTLLQVMETLLPKQEDFLLGKIPAPYPLGIKDLAEDLSFHESTIFRAIENKAVAAPIGIFPLKHLFPRGIHQDSSHSKENVLQWIRQWIATEQTPLSDSVISDRITAKGIPCARRTVAKYRAQLKILPANKRKK

>RP541_RHIME/343-503 PF04552

QAFLNECLQNANWLTRSLDQRARTIMKVASEIVRQQDAFLIHGVGHLRPLNLRIVADAIKMHESTVSRVTSNKYMLTPRGLFELKYFFTVSIGSAENGDAHSAESVRHRIRTMINQESADAVLSDDDIVDVLKQAGVDIARRTVAKYREAMSIPSSVQRRR

>RP54_ACIGB/321-480 PF04552

NQYLRNQMLEAKNFIKSVDERHKTLLKVASCIVQHQREFLEIGAEGMKPLVLRDVAEEVELHESTVSRVTTNKFLLTPRGLFELKYFFSSHVGTTSGGEASSTAIRAKIKKLVADENPRKPLSDNTIANLLKEEGIDVARRTVAKYRESLHIPSSSDRKV

>RP54_AZOCA/341-501 PF04552

KTFLADCLQSASWLTRSLDQRARTILKVASEIVRQQDAFLVHGVRHLRPLNLRTVADAIGMHESTVSRVTSNKYISTPRGVLEMKFFFSSSIASSGGGEAHAAEAVRHRIKSLIEAESADDVLSDDTLVQKLKDDGIDIARRTVAKYRESMNIPSSVQRRR

>RP54_AZOVI/341-500 PF04552

NTFMRNQLQEARWFIKSLQSRNETLMKVSTQIVEHQRGFLDYGEEAMKPLVLHDIAEAVGMHESTISRVTTQKYMHTPRGIYELKYFFSSHVSTAEGGECSSTAIRAIIKKLIAAENPKKPLSDSKIAGLLEEQGIQVARRTVAKYRESLSIAPSSERKR

>RP54_BACSU/274-434 PF04552

VSYLSAKYQEWRWLSRALRQRKQTITRIINELITRQKDFFLKGRSAMKPLTLREVADCLSLHESTVSRAIKGKTIQTPYGLFEMKLFFSAKAEASGDGDASNYAVKTHLENLINQEDKTKPLSDQKLVDLLYEQHGIQISRRTVAKYRDQMNIPSSAARKR

>RP54_CAUCR/330-491 PF04552

KTFVADCMASANWLVKSLDQRAKTILKVASEIVRQQDGFLAFGVEHLRPLNLKTVADAIGMHESTVSRVTSNKYIATPRGVFELKFFFTSAIQSSEGGEAHSAASVRHKIKGLVDAEKCEADVHSDDRIVEILKAAGVDIARRTVAKYREAMRIPSSVERRR

>RP54_ECOLI/316-475 PF04552

SQFIRSNLQDAKWLIKSLESRNDTLLRVSRCIVEQQQAFFEQGEEYMKPMVLADIAQAVEMHESTISRVTTQKYLHSPRGIFELKYFFSSHVNTEGGGEASSTAIRALVKKLIAAENPAKPLSDSKLTSLLSEQGIMVARRTVAKYRESLSIPPSNQRKQ

>RP54_HELPJ/253-408 PF04552

SDYLKEKLKEAKDLIDALNLRKATIYKIGLMLLEYQYDFFKGKELRPLKLLDLANEFNHSVSTISRAISNKYLACERGVFPIKHFFSIALDNSETSNAVIKDYLLELIKNEDKKEPLSDAKILELIEEKFHLKMVRRTITKYRQLLNIASSSERKR

>RP54_RALEU/333-492 PF04552

TAGLQQKLQEARWLIKNIQQRFDKILRVSQAIVERQKNFFSHGEIAMRPLVLREIADTLGLHESTISRVTTNKYMATPMGTFELKYFFGSHVSTETGGAASSTAIRALIKQLIGAEDPRNPLSDSRIAELLGEQGFVVARRTVAKYREALKIPAVNLRKS

>RP54_RHOCA/263-421 PF04552

RAERRRRGRGPGAGEALERRRDTLLRTAAVLVARQSAFLDKGPAHLVPLTLEDVASELGLHASTISRAVSGRMIQTQTRALPLRAFFSRAVSTQGGGEAVSRDSLDFVQRTWAAKIRQNPLSDDAIVTLAERAGLRIARRTVAKYRSTLGLASSYERRR

>RP54_RHOSH/269-429 PF04552

DAWLARARSQARWLERAVERRQATLLRTAVCLVRHQADFLDQGPRALRPLSMEEVALELDLHPSTISRATATRLIETPRGLIPLRAFFSRSVSSDGPEAPQSQDALMALVREIIAREDRTKPFSDDAIVKQAKLAGAVLARRTVTKYRETLGIPSSYDRKR

>RP54_THIFE/315-474 PF04552

HKYIQDQLNEARWFIKSLQSRQDTILKVARAIVERQKDFFANGPESMRPMVLRHIADAVEMHESTVSRVTNQKYMITPRGLYEFKYFFSSHVGTDSGGSASATAIRALLIKMTQAEDAQHPLSDAEIARVLADQGIQIARRTVAKYREAANVPPASQRRR

>RP55_BRAJA/353-513 PF04552

KSYFTDALQNATWLVRALDQRARTILKVATEIVRQQDGFFTHGVAHLRPLNLKAVADAIQMHESTVSRVTANKYMATNRGTFELKYFFTASIASADGGEAHSAEAVRHHIKQLIDSEAPAAILSDDTIVERLRASGIDIARRTVAKYREAMRIPSSVQRRR

>ETV1_HUMAN/1-333 PF04621

MDGFYDQQVPYMVTNSQRGRNCNEKPTNVRKRKFINRDLAHDSEELFQDLSQLQETWLAEAQVPDNDEQFVPDYQAESLAFHGLPLKIKKEPHSPCSEISSACSQEQPFKFSYGEKCLYNVSAYDQKPQVGMRPSNPPTPSSTPVSPLHHASPNSTHTPKPDRAFPAHLPPSQSIPDSSYPMDHRFRRQLSEPCNSFPPLPTMPREGRPMYQRQMSEPNIPFPPQGFKQEYHDPVYEHNTMVGSAASQSFPPPLMIKQEPRDFAYDSEVPSCHSIYMRQEGFLAHPSRTEGCMFEKGPRQFYDDTCVVPEKFDGDIKQEPGMYREGPTYQRRG

>ETV4_HUMAN/8-339 PF04621

GYLDQQVPYTFSSKSPGNGSLREALIGPLGKLMDPGSLPPLDSEDLFQDLSHFQETWLAEAQVPDSDEQFVPDFHSENLAFHSPTTRIKKEPQSPRTDPALSCSRKPPLPYHHGEQCLYSSAYDPPRQIAIKSPAPGALGQSPLQPFPRAEQRNFLRSSGTSQPHPGHGYLGEHSSVFQQPLDICHSFTSQGGGREPLPAPYQHQLSEPCPPYPQQSFKQEYHDPLYEQAGQPAVDQGGVNGHRYPGAGVVIKQEQTDFAYDSDVTGCASMYLHTEGFSGPSPGDGAMGYGYEKPLRPFPDDVCVVPEKFEGDIKQEGVGAFREGPPYQRRG

>ETV5_MOUSE/1-366 PF04621

MDGFCDQQVPFMVPGKSRSEDCRGRPLIDRKRKFVDTDLAHDSEELFQDLSQLQEAWLAEAQVPDDEQFVPDFQSDNLVLHAPPPTKIKRELHSPSSELSSCSHEQALGAKYGEKCLYNYCAYDRKPPSGFKPLTPPATPLSPTHQNSLFPPPQATLPTSGLTPGAGPVQGVGPAPTPHSLPEPGSQQQTFAVPRPPHQPLQMPKMMPESQYPSEQRFQRQLSEPSHPFPPQSGVPGDSRPSYHRQMSEPIVPAAPPPLQGFKQEYHDPLYEHGVPGMPGPPAHGFQSPMGIKQEPRDYCADSEVPNCQSSYMRGGYFSSSHEGFPYEKDPRLYFDDTCVVPERLEGKVKQEPTMYREGPPYQRRG

>PEA3_BRARE/5-348 PF04621

MDGYLDQQVPYTLANRSQGNGPLNRLLMATKRKYMDAELPPQESEDLFQDLSQLQETWLTEAQVPDSDEQFVPDFHSENSVAFHSPPVKIKKEPQSPGSDPSQSCSHKQSFSYPNGEQCLYASAYEQKRAAVAGAGGSKSSCPATPMSPMQHYSPKPTVGTRQESGYMNPPSSSQSHACHSHSYPMNPSSRFPSGSAEMCPPFASQGQALQRIDPAHASGGGGGGYHRQHSDPCLPYPPQQTFKQEYMDPLYDRAAHINGPQPQRFPPAHMMVKQEPTDYTYEPDVPGCPSMYHHNEGYSNPQHNSEGYMFENDSRVVPEKFEGEVKQEGGSVFREGAPYQRRG

>O49689_ARATH/31-247 PF04640

NRWPPWLKPLLKEQFFVHCKFHGDSHKSECNMYCLDCTNGPLCSLCLAHHKDHRTIQIRRSSYHDVIRVNEIQKYLDIGGIQTYVINSAKVVFLNERPQPRPGKGVTNTCKVCYRSLVDDSFRFCSLGCKFHLTSPCISVNSFLILTFSMFLGNEIKIAGTSRGFEKGRENLLMETEDSSSSIAIGKNITNLQSFSPSTPPLTTSSNCRIVKRRKGI

>Q9C7R9_ARATH/6-210 PF04640

VMTPPWLTPMLRADYFVTCSIHSQSSKSECNLFCLDCSGNAFCSSCLAHHRTHRVIQIRRSSYHNVVRVSEIQKHIDISCIQTYVINSAKIFFLNARPQCRTGKSLNKTCQICSRNLLDSFLFCSLACKLEGVKNGEDPNLTLFHSGKSDDSSKIINTGICSRLIDGISIAVDDQRSETAGVLSPETPSIESHRNYPMKSRRKGI

>Q9C9J9_ARATH/12-236 PF04640

LNPPPWLIPMLRANYFIPCSIHAASNKSECNMFCLDCSSEAFCSYCLLNHRNHRVLQIRRSSYHNVVRVNEIQKYIDISCVQTYIINSARIVFLNERPQPRIGKGVTNTCEICCRSLLDSFRFCSLGCKLGGMKRDPSLTFSLRGKHGREYEGEWESDEATTPTKIRKTCAFNRLMSGLSISTVKCDYLSGDQPSSSSGDESGFKLSPGTPPIYNHRNSSRRKGV

>Q9FIQ4_ARATH/25-219 PF04640

NQWPIWLKPLLNQHFFAQCKFHGHLPRTECKMYCLDCTNDSFCSLCLSEHENHRTIQIRISSYHNVTKVDEIQKYLDISSIQTYVINSSKVLFLNERPQSKPGKGFTNACMVCYRGLAENCFRFCSIGCKVAGTSGVFQKRVKHTTNDSDNSNNSSGVENNSSGAENGNSNLQSLSPPTPQFPPRSLRKRLRKGI

>Q9FYJ5_ARATH/16-234 PF04640

ATKPAWLEGLMAETFFSSCGIHETRRKSEKNVFCLLCCLSVCPHCLPSHRSHPLLQVRRYVYHDVVRLSDLEKLIDCSYVQPYTINGAKVIFLNQRQQSRAKVSSNVCFTCDRILQEPFHFCSLSCKVIILQQKFGNYFTFRIDESDFTFEGLRMDGHDQLGEISTMEDGEDILVISDESEQGNNSHKKEKKKSKKKKPESNYLPGMVLSSLGNRRKGA

>Q9LZZ6_ARATH/4-238 PF04640

GEFPAWLEVLLKDKFFNACLDHEDDKKNEKNILCIDCCLTICPHCLSSHTSHRLLQIRRYVYRDVLRVEDGSKLMDCSLIQPYTTNSSKVVFINERPQSRQFRGSGNICITCDRSLQSPYLFCCLSCKISDVIMRQRGLSGFLRVCNVLDLTDEVTTTTPSSTLEPTGSNRTSSESSGNEGEDMFWCQALACTATTEIVRKKRSSLSTTCRRVTEVVSTTNTEAPVNFLNRRKNP

>Q9SJJ8_ARATH/1-134 PF04640

MEEPKWLEGLLRTNFFSICPRHRETPRNECNMFCLSCQNAAFCFYCRSSFHIDHPVLQIRRSSYHDVVRVSEIENALDIRGVQTYVINSARVLFLNERPQPKNSSHEPFLIPFASVPWVARFVFLHLFSMQNSF

>Q9SYN7_ARATH/10-237 PF04640

YTSPPWLMPMLRGSYFVPCSIHVDSNKNECNLFCLDCAGNAFCSYCLVKHKDHRVVQIRRSSYHNVVRVNEIQKFIDIACVQTYIINSAKIVFLNERPQPRIGKGVTNTCEICCRSLLDSFRFCSLGCKLGGMRRGDLSLTFSLKGKHGREYLGGSESDEATTPTKMRKTNAFNRLMSGLSISTVRFDDYGPNGDQRSSSSGDEGGFSFSPGTPPIYNHRNSSRRKGV

>Q9WII1_CNPV/14-288 PF04661

TVQRRRGNDEDNKFTCIQALEHAKSLCTKNNKIVKSVKLSQSLFKSSNNISVILEPEYKDKLVTPLIIVEGEGKIYHNKNDSFNREEPYFLKIRPTLMNPILYQIMECIYRDLNYLDPENTMDEKTFKDCHLYINGNRIMSADVKYLKNGKPVGEKLSVSKEIDKLVKKDPQMIKAVLVASTFFDNGNMCKISFSLKSLIMEKICKTTLIDTNGEVISIVTSGETDTEDDSDTETDIDKECKKIQQIKLDDKKHNNKVNECDDEDCEEEQSLFSL

>S1FA1_ORYSA/6-76 PF04689

NNMIIEEVNKGLNPGTIVLLVVATLLILFFVGNYALYMYAQKTLPPRKKKPVSKKKLKREKLKQGVSAPGE

>S1FA_SPIOL/1-70 PF04689

MAVNEVEAKGLNPGLIVLLVIGGLLLTFLVGNFILYTYAQKNLPPKKKKPISKKKMKRERLKQGVAPPGE

>DESM_BOVIN/8-105 PF04732

QRVSSYRRTFGGAPSFPLGSPLSSPVFPRAGFGTKGSSSSVTSRVYQVSRTSGGAGGLGALRASRLGSTRVPSSYGAGELLDFSLADAVNQEFLTTRT

>DESM_XENLA/9-94 PF04732

QRASSYRRTFGGGSPSFSTRSSFGSKGASSSSVSSRVYQVSRSTAAPSLSSFRATRVAPVRSSYGADVLDFSLADAMNQEFLQTRT

>GFAP_MOUSE/4-64 PF04732

RRITSARRSYASETVVRGLGPSRQLGTMPRFSLSRMTPPLPARVDFSLAGALNAGFKETRA

>IF3T_TORCA/22-95 PF04732

VRVSSIRRSYTARGNPQGSLIIPSSSRSRVSYVTPISSRSVRLVRSSAPVVASSSNLDFTLVDAMNSEFKVNRT

>NFL_COTJA/8-88 PF04732

FFPSYKRRYADSPRLHVSAMRSGGYSSARSAYSSLSAPVSSVSVRRSYATSSASGSLLHSVDSLDLSQVAAISNDLKSIRS

>NFL_PIG/8-86 PF04732

YYSTSYKRRYVETPRVHISSVRSGYSTARSAYSSYSAPVSSSLSVRRSYSSSSGLMPSLENLDLSQVAAISNDLKSIRT

>NFL_RAT/8-87 PF04732

YFSTSYKRRYVETPRVHISSVRSGYSTARSAYSSYSAPVSSSLSVRRSYSSSSGSLMPSLENLDLSQVAAISNDLKSIRT

>NFL_XENLA/8-81 PF04732

YYTPYKRRVVESSPRVHIRSSYVSPSRTTYSPLVSTTMRRSYATSSSSSFLPSVDTMDLSQVAAISSDLKIVRT

>NFM_CHICK/8-93 PF04732

GNPSYRRVMTETRATYSRASASPSSGFRSQSWSRGSGSTVSSSYKRTNLGAPRTAYGSTVLSSAESLDVSQSSLLNGAAELKLSRS

>NFM_RAT/8-97 PF04732

GNPSAYRRVPTETRSSFSRVSGSPSSGFRSQSWSRGSPSTVSSSYKRSALAPRLAYSSAMLSSAESSLDFSQSSSLLNGGSGGDYKLSRS

>O13099_XENLA/9-84 PF04732

GNPSYRRMAETRTSYSRTSSTPSSGFRSQSWSRSSPTTISSYKRTNLAAPRVYSSNDSLDFSQSSAFNGDLKQVRF

>P79933_XENLA/8-89 PF04732

YLSSSYRKIFGDPPRASSARLGPSGSSRTTVAGGYRSHSQSRSNVPSSSYRRAPRGAGYLAADTVDLSQTSAVNNEYKIIRT

>P87359_BRARE/6-71 PF04732

YRTSHHRSYGTPSVSSRIGGRYTSSIPSRPVDFRSRSSAPAPRLSYDKVDFSLAEAVNQEFLATRS

>P87360_BRARE/8-86 PF04732

YSASSYRKIFGDSTRFSASPSRLSSSRSGFKSQSVNRPNIPGSYKRSTRSGFPSSSLNLDSFDFTQSTVLNNEFKIIRT

>PERI_XENLA/9-86 PF04732

TSTSYRRTLGSSPVPSSYSSSSRLSTSRHFGSPSPGPSSRSSSSAFRVRSSTPVRVSLDRVDFSVAEAVNQEFLTTRS

>PLST_CARAU/1-75 PF04732

MSHSTFSHLFSPHFGAPVYSPVSSRIGGRYVSSSVPTRSVDFRSRSSAPAPRLSYDKVDFSSAEAINQEFFATRS

>Q90307_CARAU/7-82 PF04732

TIGSPYRRVMDTRTSYSSPSSGFRSQTWSRASPSSSSYKRTFNVPVARRYGSTVLSSTDNLDYTQTSILNGDYKRS

>Q90441_BRARE/10-100 PF04732

ESASSYRRTFGSGLGSSIFAGHGSSGSSGSSRLTSRVYEVTKSSASPHFSSHRASGSFGGGSVVRSYAGLGEKLDFNLADAINQDFLNTRT

>Q9DDB3_9CHON/3-87 PF04732

TSTSTYRRMFAEAPFQRASSSRSYATRQQPRIVSSVSRTSYSQPSMIKRRSVRVNRSSAPGIAMGNSLNFSLVDAMNSEFKVNRS

>Q9DDC8_LAMFL/5-105 PF04732

QRVSSYRRTFGPVASSSATRSFSSRSSGYGGFGGGGAGFGGSIGGGGGMIRSSRSVGLGSSAQSGYGGRSSSASYVKALSGQNVDFSLADALNADYRTTRT

>Q9YHX5_BRARE/5-92 PF04732

TSTSSYKRMFGGERPAAARSTFSSRQYSSPVRVSSSRTSYNYSSAPPSVYASKGLRVRSGAPLPRLATDTLDFGLAEAINTEFKANRT

>VIM1_XENLA/4-94 PF04732

TKSSYRRIFGGNPRSSSSGNRYATSSTRYTLGSAMRPSTSSRMVYSTSSSPAVFKSSSVRLRSSLPPARMADSVDFALADAVNLEFKANRT

>VIMB_CARAU/5-87 PF04732

TSTSSYKRMFGAERQAMVRSTYSSRQYSSPGRTTSRVSYSSASSTSPSLYMSKSARSATRLATETLDFGLADAINTEFKANRT

>VIME_ONCMY/10-97 PF04732

TSSSSYKRMFGGEGRPSVGMARSTLSSRQYSSPVRSSRMSYSVSAPPSIYASKNVRLRSSAPMPRLSSDTVDFALSDAINSEFKANRT

>XNIF_XENLA/6-86 PF04732

LYTSSYKKIFGDWGRSSSLLYTTNSSSSTRSQSYRPREAYTSNISSYRKVSRSPGHLSSAQDHFDLSQSTALSNELKIVRT

>Q83608_9POXV/7-102 PF04767

YHTHTPFYIDTKEGKYLVLKAVKVCDIRTIECSGEKASCVLKVEKTSPTCDRKPMTPCERAARSSSPQNRGVPFMRTNMLEDLQASNRNVVSRILG

>Q9DHT1_YLDV/8-105 PF04767

YYTHTPFFIDTKEGRYLVLKAIKACDIRKVDCDVSKASCVLKVEKPTSTCDRPMSPCERAARSSSPNGRNNSVIPFMRTNMLEDIQTNNRNVMSRILG

>VF17_VARV/6-101 PF04767

ASAHTPFYINTKEGRYLVLKAVKVCDVRTVEFEGSKASCVLKVDKPSSPASERRPSSPSRCERMNNPGKQVPFMRTDMLQNMFAANRDNVASRLLS

>O55580_NPVLS/67-314 PF04786

WVKVFVHNLQRRNISVVRCTDKFNYLNECLQFVSQPNYLPFQHLGREVEEVPNRFFDQDELKLRRPSNWHHHRPQFRRQLYFAQVLLFDRVRMKRLKSTFGESLSVTLTNGSIYHNVFGYMSKLYHKCNEGLLTPSSIVNLPIADTEREMFVRRFFWCPQERNPLLYAANDILDIVNVQPYTLDAFNQQFAFLAPHNRSNEAEMIMGGLIEGFKQSKNETKMQTMELEGTLYKEYTVAIKPMVFFNLS

>O92392_NPVBM/71-316 PF04786

WQEQMAINVKRGNFSILNCSCFEGRFLKNEFCRLANLNSLHEWEDKLYPEPDKNIVVLEPANGKTTYTIGPRVQGKPCGFWFSDFGTIKRAKSNFGQFFSIQYGDIHKHNNIFGNILQRHLQSDFPLKMEPNVCIHLPDKNKTSERDMLIRRFYIINRDNNGSIYATGKIRNVPLDMQRMSVEDFDRLFEMDKIDGPSEEIKMYMMGTIDGVKYGKEMQMTDMNNKKITEKPYSLAFKPGIFVIIE

>Q99H22_9NUCL/72-319 PF04786

WVDKFTHNLQKKNLTVLRCHTPFNKLFESLGFLNESISLESWIDKLYPQVNDKVVIEPLKPPKLTYKIGVLVKGGMFHFYFFDMVKMKRYKSVYGEFFMITWPNMHVHNKIFGNIMKNHLQEENLKLQNSVLVNLPEDNVSYANKMMFVRKFFNITQSQNEKVFSTGDLVKSVRCEPFTVDTFNDVFQFESNTDPPKPSEEVEMLMGALIEGVKISKNETQFETVTGKKLFEKSYSLSIKPMVFFRIE

>Q9J814_9NUCL/81-327 PF04786

WVDELRYNLQAKNLTVLRCDVAFNKLFECLSFLRESLPLDNYLDSFLPEVAADIGILKPKAPAVVYLVGMLVKGGVEPFYVFDMAKVRRCMSNFGEFLSIRWSKQYIHNDAYANVIIKYKGFDCDKMKLQNSACVNLPSDDAVGRKTTFVRKFFDIKHHNNEKNYMTGRLIKSVKCEPFTVKRFNELFQFEQDSKSSDEVEMLVGIQIDGFKQGKNDIEFDTLVNNKKVSEKSYSLAIKPMVFFHIE

>Q9YMS7_NPVLD/7-256 PF04786

WIDELMYNLKRGNFTVVTCDSPNKNLLNSLLKVQHKLNIGQYMQQLYPDVSEELAPKLVIRKPKPPRLVYEVGMHVHGGKLPFYFVDTVVLRRCVSDFGEFMTARWTEMNAHNSIFAQMVLRYNSWEGEMITMRDNVIIKLPNDKAAFARMFFDIKRQTNEQVYDKGIGGSEKQVMCEMFDADRFEELFQFNMVANECTPSDEVNMVMVAIIDGFRQGKDDIEMETVNNKKVKERWFSLAVQPVVFFNIE

>Q9YMT7_NPVLD/12-238 PF04786

WRDKFVHALHCAGERAVVCDEPASRLDEYLDRWRDRCVPLTTACTGEAVRALRLTNGALRAACDPRKRPSVLERVRVEPARGGYAVRWPRLPGLYRRTVDLLTQRRDAEPDDQWTDDDDVCAFLERAGDEDPTSRELLARKFYRIARADNLPLYLSGELLARARARPVPVDRLDSTLAAPFEALMLAAVDGVRENRAGVEYKSVNNKTYRCKTFSLAIKPVLFFIVD

>Y025_NPVOP/59-299 PF04786

WQCLVLLNCQALDFSAINFHYGDLQYLKDKFTELQNLSNYYEWRKQERPEDKVCIMEAAVGKCTYTIGLRVKGRPNGFAIAEFGSVHRSKSTFGQFLSTTWSAIHEHNSVFGKIMDSYYKHEYPLKLESSVCVHLPEKDYEREIKARQFLWVRRDNNPELYATGQLDRPLEVAPMTLAEFDRLFEVNKTDGPSQEVPVLVCGRIDGVKYGKEIQMTDVNGRKFSEKPYSLAFKPVLYLILE

>BAT_HALSA/617-669 PF04967

LTDRQLTALQKAYVSGYFEWPRRAEGKQLAESMDIVPSTYHQHLQAAKQKLVG

>O28843_ARCFU/150-202 PF04967

LTEAQRRALKIAYDLGYFSEKRDVTINDIAMALGVAKSTAHKHLKIAISKLVE

>O29453_ARCFU/167-219 PF04967

LTDLQVKTIKQAYVNGYYDYPRRCTLGDLAKINGISKNAMAKRLKSVEKKIFE

>O54637_HALSA/175-227 PF04967

ITDRQLAALQLALESGYYEQPRKTSLRDLAERTAVARSTYEEHLRKAENKLLT

>Q97A82_THEVO/160-212 PF04967

LTETERRIVNSSLRQGFFSWPRSYDLTSISREFGLTKPTILYHIRNAERKVME

>Q97BL0_THEVO/325-377 PF04967

YTPSEMQIIAEAVSRGYYSIPRHVGIRDLAEYFGMSKSTVQEYLRKAEQKTMN

>Q97BT7_THEVO/149-201 PF04967

LTPREKEIINAAFKLGYFDIDRQISMKDLAKNFGIKTASISDVMRRALKKIVM

>Q97C66_THEVO/163-215 PF04967

LTEKQLRYLVSAYHNGYFDVPKRTNIVELAKQFNISYSTLQEHLEKAVNKIMK

>Q97UC0_SULSO/162-214 PF04967

LTPKELSILKLAFDKGYFETPKRIGLEELANELGISKPAIMQVLRKAIAKLTE

>Q97UD7_SULSO/162-214 PF04967

LTSKEFEIIYKAYKMGYFDWPKKVDLKELSEELGISKAATLQALRRAMGKLIR

>Q97UG0_SULSO/190-242 PF04967

LTEKERMLLRKARELGYFNVPRDRNSQELAKQLGVSKMNISFSLRKILKKISD

>Q97WR0_SULSO/150-202 PF04967

LTEWEKKVLTVSYSYGYLDYPRRATADELAKLLGISKVTFLYHLRNAQRKLIA

>Q97X87_SULSO/145-197 PF04967

LTDRQMEILKLAYKMGYFDDDRRVTLTELAKQLGISTPTLEEILRRALRKVVK

>Q97Y14_SULSO/153-205 PF04967

LTDRNLSFLKIAHKQGLFDYPKRKTLLSLSKELGIKPNTLLYHIRKSESSLLE

>Q97YL7_SULSO/164-216 PF04967

FSEQERNAVLHAIRLGYYEYPRRINLEELGKIMGISKPTLEEYLRKAEKKIMS

>Q97YT8_SULSO/151-203 PF04967

LTPYELKTLILAYKNGYFDFPRRIKSDKISKLINISKSTFTYHLRSAESRIIK

>Q97ZB6_SULSO/152-204 PF04967

LTEKEILVLKTAITMGYFNYPRQIKAKEIAEKLGISKQDFLYHLRKSIEKIIF

>Q97ZX5_SULSO/134-186 PF04967

ITARQEQILRIALEAGFFDYPRRIGLKDLAKKLNISPSSLSEIIRRAEKNVIT

>Q9HK57_THEAC/173-225 PF04967

FTPIEMQIIIEAYNLGYYDIPRRAGIREIAEYFGLSKSTVQEYIRNAESKTMS

>Q9HKC6_THEAC/159-211 PF04967

LTEMQEKVLRNAFEMGFFNYPKDVHLKDIAQKIGVSAVSVDQYLREAQRKLIR

>Q9HLR0_THEAC/170-222 PF04967

LTTKQLKYLVNAYHMGYFDTPKRVQIGEMAKEFGISYSTLQEHLEKAVNRILK

>Q9HPS7_HALSA/155-207 PF04967

LTDRQLEVLTTAYEAGYFERPRGATAQALADDLGIAPSTFTEHLLAAQRKLLG

>Q9HQD9_HALSA/153-205 PF04967

LTARQAAVLRAAVDAGYYERPRETTTAALADRFDIARSTLEEHLRRAEGKLAR

>Q9HQU9_HALSA/529-581 PF04967

LTDKQAAVLRAAFHAGYFEWPRESTAEDLAADMGVTAPTLHNHLRRAQQKLLA

>Q9HR58_HALSA/160-212 PF04967

LTDAQREAVRVALDVGYLDVPRSASLSAVSEQLGVTEQSASERLRRATRNLAA

>Q9HR76_HALSA/158-210 PF04967

LTDKQREAAAAAVAKGYYATPRGADLSDLATALGISKSAVSQRLSAVESKLAT

>Q9HSM5_HALSA/800-852 PF04967

LTERQVEVAQVAYHSGFFDPSHAVSGAVVAETLGISNTAFYDHVNRIESKVFD

>Q9HSM9_HALSA/169-221 PF04967

LTDRQREVLALATRMGYYDEAATVRVADIADEIGLADTTTWEHLSRAEETVMA

>Q9HSR5_HALSA/159-211 PF04967

LTDKQYRAIRTAFARGYYDSPRQCSTRELAAELGVSAAAASDLLRRAERQLIG

>Q9UXH2_SULSO/156-208 PF04967

LTEKEVLILKIALNMGYFNYPRNIKAKDIAEILGISKQDFLYHLRNSINKIIT

>O60428_HUMAN/20-165 PF05224

AFVCQKKNHFQVTVYIGMLGEPKYVKTPEGLKPLDCFYLKLHGLEALNQSINIEQSQSDRSKRPFNPVTVNLPPEQVTKVTVGRLHFSETTANNMRKKGKPNPDQRYFMLVVALQAHAQNQNYTLAAQISERIIVRASNPGQFESD

>Q01652_PENCH/156-329 PF05224

ELTCYRRNLFQITGSVTLPRGMRYIMTDQGDRIPILAQELTVSATESVEGNPVKIISVPWKTPSAAAANSGPAPEGNNPSTGAKIEKEPPPIPLDIMAGQDLDSDYATFPIVWKRLQFRVATANNGRRKELQQHFVVRLKVVATLSTGAKIPISEVQSGPVIVRGRSPRNFQSR

>Q23567_CAEEL/283-435 PF05224

CFVNQKKNHFQISVNVEASDTMPPKYVNFNNRLVPIRDFKLSFCGVKAEMPSSEITIRQSRADRKPHTHTPVLFEIQERRMTKVCVPRLHFSETTLNNQRKQKNRPNPEQKFFLLVVRLFASIDESEHGVLIQSYASEKVIVRATNPGSFEPQ

>Q6BNX4_DEBHA/343-545 PF05224

VISCYRRNYIQISMSMNLSGFSNDDDNNNKVLKLQTNEYGYSITRVIKWFKVEIFANTNVSEIRSIPIVIYDDNGQRDKERPKDEIVTDENNDFVNPISIDKAQSTITLNESVIKNSEIDNYYTVKKLQFKSATPNNGNLTFQNYYHLKIKVSAIVADLYYDDYIDEDYSSNTYNERNEVSLFELVSEPIIVRGRNPSFYADR

>Q6C7W3_YARLI/226-376 PF05224

HLTFYRRNLLQVRASVHNDHKVVYVQTESGERAKTQSLWLDVEVTDNSADSDPQRLLFCSPKSATSLPADDEAKAKMVFPDNPTANDEVEWKRLQFKGATAHNGRTKHQQFFYVSVKCLAELENGTKTCLASALSRPVVVRGRNPRFYLNR

>Q6NL96_DROME/413-560 PF05224

AYVCQKKNHFQVTCHARLQGDAKFVKTPSGFEKIKSFHLHFYGVKFEAPNQTIRVEQSQSDRSKKAFHPVPIDLQKHIVSKITVGRLHFSETTNNNMRKKGRPNPEQRFFQLVVGLHVHTISGNFPVVSHGSEKIIVRASNPGQFESD

>Q8MQ67_CAEEL/301-451 PF05224

CFVNQKKNHFQVTVKIEAIDPSPPQCFKINGVCKPIENFQLSFVGAKSESQNSEIPIRQSTTERKPILHTPVLFKIVERRMTIVTVPRLHFSETTLNNQRKNLRPNPDQKYFNLVVRLYATATDGTTVLMQAFASERVIVRATNPGSFEPP

>Q94474_DICDI/88-267 PF05224

QWIYCRRNHFQLDITAVYPKLFQETQYGHNGVTLSNSSSSVDPTQTPSYMLISGVKTPINGLTLTIKGIKNRADMSQQESEVELFQTNSKREKQGEHAPKPVAIQFGSLVSIQRLHFRKATLNNARRHGQPNPHQEFNQLVVSLYGRCMGQEYCIVSYVSPALIVRTATQVTPPGDLSPV

>Q9C2N1_NEUCR/175-340 PF05224

ELTCYRRNLFQISGNICFPQIPLSVMLETGETSQIKNMEVTISAIESVDGHPVRLIVIPWKTPPPNSPEVNQAPDQEPPSLPLIPWSEEEEDNGGDHYAIYPIGWRRLQFRIATANNGRRKELQQHFVLHLKLHGTLANGTKLVLSELTTAPIVVRGRSPRNFQAR

>Q9LNA6_ARATH/43-112 PF06331

MVNAIKGVFVSCDIPMTQFIVNMNNSMPPSQKFIIHVLDSTHLFVQPHVEQMIRSAISDFRDQNSYEKPT

>Q9N390_CAEEL/1-70 PF06331

MVNVKKGVLVTSDPAFRQLLIHLDDSRQLGSKFIVRELDDTHLFIEKEIVPMLENKVEQIMENMNPEAVD

>TFB5_SCHPO/1-68 PF06331

MPRAQKGLLLVECDPTVKQLILNMDEQSPGIVIEEIDEERLLVNESRLEQVKAELERRLEENTYQVEE

>HSF1_CHICK/251-491 PF06546

AYSGSNIYSPDSSTNSGPIISDVTELAQSSPSASPSGSLDERSSPVVRIKEEPPSPSRSPKENEPSTTTAAAGNSTEQPQPQEKCLSVACLDKNELNDHLDTIDSNLDNLQTMLSTHGFSVDTTALLDLFSPSMTVTDMNLPDLDSSLASIQDLLSSQEQQKPSEADAAAADTGKQLVHYTAQPLFLVDSSAVDVGSGDLPIFFELGEGSYFTDGDEYNEDPTISLLSGTEQPKPKDPTVS

>HSF1_HUMAN/246-529 PF06546

AYSSSSLYAPDAVASSGPIISDITELAPASPMASPGGSIDERPLSSSPLVRVKEEPPSPPQSPRVEEASPGRPSSVDTLLSPTALIDSILRESEPAPASVTALTDARGHTDTEGRPPSPPPTSTPEKCLSVACLDKNELSDHLDAMDSNLDNLQTMLSSHGFSVDTSALLDLFSPSVTVPDMSLPDLDSSLASIQELLSPQEPPRPPEAENSSPDSGKQLVHYTAQPLFLLDPGSVDTGSNDLPVLFELGEGSYFSEGDGFAEDPTISLLTGSEPPKAKDPTVS

>HSF2_CHICK/295-541 PF06546

SSNCSRSPDIVIVEDDNEEEYAPVIQGDKSTESVAVSANDPLSPVSDSTSPLMSSAVQLNNQSTLTAEDPVSMMDSILNENGVISQNINLLGKVELLDYLDSIDCSLEDFQAMLSGRQFSIDPDLLFDLFTSSVQMNPTDHIPNTKMETKGIETTKSNAGPAASQETQVSKPKSDKQLIQYTAFPLLAFLDGNPGSTVESGSSATETPSSVDKPLEVDELLESSLDPEPTQSKLVRLEPLTEAEASE

>HSF2_HUMAN/229-498 PF06546

PHSRTEGLKPRERISDDIIIYDVTDDNADEENIPVIPETNEDVISDPSNCSQYPDIVIVEDDNEDEYAPVIQSGEQNEPARESLSSGSDGSSPLMSSAVQLNGSSSLTSEDPVTMMDSILNDNINLLGKVELLDYLDSIDCSLEDFQAMLSGRQFSIDPDLLVDLFTSSVQMNPTDYINNTKSENKGLETTKNNVVQPVSEEGRKSKSKPDKQLIQYTAFPLLAFLDGNPASSVEQASTTASSEVLSSVDKPIEVDELLDSSLDPEPTQS

>HSF_XENLA/236-448 PF06546

SYPVSGFTDSSAGPIISDVTELPESSPSPSPCPSLEASPSPVILIKTEPLTPSQSPEQSPAPPKLDDTPISPSTFIDSILLETETSVCPGGNKNDEMSESHPPEPCLSVACLDNISLSRQMSEVSRLFPTSCSSVPGRAEPPGLDMAVAELNDHVDNIDFNLDTLQNLLNGQSFSVDTSALMDLFSPSLGIPDLSLPDPDSSLASVSSTPIYT

>Q8UWM7_BRARE/243-497 PF06546

AFTGTGVFSPEPPVKTGPIISDITELAQSSPVATDEWIEDRTSPLVHIKEEPSSPAHSPEVEEVCPVEVEVGAGSDLPVDTPLSPTTFINSILQESEPVSALTPPPSEQKCLSVACLDKTELHDHLESIDSGLENLQQILNAQSINFDSSPLFDIFSSAASDVGLDSLASIQDLLSPDPVKETESGLDTDSGKQLVQYTSQPIVLPDPLSTDSSSTDLPMLLELQDDSYFSSEPAEDPTIALLNLQPVPEDPKLS

>REX1_THEMA/3-52 PF06971

EKIPKPVSKRLVSYYMCLERLLDEGVEVVSSEELARRLDLKASQIRKDLS

>REX2_THEMA/5-51 PF06971

IHLPRSTFERLKMYRKVLEATKKPYISSDEIARFLEINPDLVRKDFS

>REX_BACSU/6-55 PF06971

SKIPQATAKRLPLYYRFLKNLHASGKQRVSSAELSDAVKVDSATIRRDFS

>REX_BACTN/13-62 PF06971

TKVPEPTLRRLPWYLSNVKLLKQRGERFVSSTQISKEINIDASQIAKDLS

>REX_CLOAB/5-54 PF06971

KNISMAVIRRLPKYHRYLEELLKSDVDRISSKELSEKIGFTASQIRQDLN

>REX_CLOPE/5-54 PF06971

KGISMAVIKRLPKYHRYLQELMENDVDRISSKELSEKIGFTASQIRQDLN

>REX_CLOTE/5-54 PF06971

RNISMAVIKRLPKYHRYLEELLRNEVDRISSKELSKKIGFTASQIRQDFN

>REX_LACLC/8-57 PF06971

KSLPKATAKRLPQYYRLFKSLVEENVTRTNSQLISEKIGVDAATIRRDFS

>REX_LACPL/4-53 PF06971

TKIPRATAKRLPIYYRYLNILLDADKKRVSSTELSEAVKVDSATIRRDFS

>REX_LACSS/6-55 PF06971

HDLPEAVAKRIPIYYRYFKLLETDGIERIKSEQLAKLVAIPSATIRRDFS

>REX_LISMO/6-55 PF06971

TKIPQATAKRLPLYHRYLKYLDESGKERVSSAELSEAVKVDSATIRRDFS

>REX_STAES/5-54 PF06971

FKIPRATLKRLPLYYRLVSILKGKGIDRVNSKTISEALQIDSATIRRDFS

>REX_STRCO/14-63 PF06971

RGIPEATVARLPLYLRALTALSERSVPTVSSEELAAAAGVNSAKLRKDFS

>REX_STRMU/5-54 PF06971

KTIPKATIKRLSLYYRIFKRFHSENIEKASSKQIAEAIGIDSATVRRDFS

>REX_STRP3/5-54 PF06971

KSIPKATAKRLSLYYRIFKRFHADQVEKASSKQIADAMGIDSATVRRDFS

>REX_THEAQ/2-50 PF06971

KVPEAAISRLITYLRILEELEAQGVHRTSSEQLGELAQVTAFQVRKDLS

>REX_THETN/5-54 PF06971

TIVSMAVIRRLPRYHRCLEELLKNDIKRISSKELSERMGVTASQIRQDLN

>Q48345_9NOST/296-364 PF07282

WTQFRQWVEYFGKVFGVVTVAVPPHHTSQNCSNCGEVVKKSLSTRTHACPHCGHIQDRDWNAARNILEL

>Q54132_SACER/310-380 PF07282

LGEFRRQLEYKTTRAGKTLVVIDRWYPSSKTCSACGHLLEKLSLSTRAWTCPGCRTRHDRDINAAKNILAA

>Q56249_BACP3/292-361 PF07282

WGMFTTFLQYKLVEQGKKLIKIDKWFPSSKTCSCCGRVKESLSLSERTFRCECGFESDRDVNAAINIKHE

>Q897V1_CLOTE/106-177 PF07282

WSEFRRMLEYKASWYGRNIIIAPSNYASSQLCSECGYKNEDVKNLALREWICPNCGVHHDRDINASKNLLKL

>Q8CME0_SYNEL/290-360 PF07282

WGELVRQLEYKAQWYGRTLVKIDRWFPSSKRCGQCGHIVERLPLSVREWDCPKCGAHHDRDINAAGNILAV

>Q8DGA6_SYNEL/12-78 PF07282

AGRVWQMLSYKAQKLAMKTTLQNEAYTSQECPVCLHRQKVKGKNYHCTNCGFKYHRDGVGSINIRRK

>Q8FDH2_ECOL6/309-378 PF07282

WAEMRRQLEYKQAWRGGDVLAINPAYTSQKCACCGHTSKNNRRTQASFICTACGYTANADVNGARNILTA

>Q8GF05_ENTFC/302-372 PF07282

WSKFVTKLQYKADWYGRKIIKVDKWFPSSQICSECGHKDGKKSLEIREWTCPVCHTHHDRDINASINILTE

>Q8GJN4_SYNP7/299-367 PF07282

WTQFTHWLDYYGKLWRKVVVAVNPAYTSQDCSGCGYRVQKSLSTRTHDCPHCGLTICRDQNAALNILKR

>Q8KNE3_MICEC/292-362 PF07282

WAEFRTLLTYKAHRDGRTLAVVDRWYPSSKTCSACGHLLATLSLGTRHWTCPSCGTRHDRDVNAAKNITVA

>Q8TXQ5_METKA/374-440 PF07282

YKRIIREIKRKCEEYGIEVIEASPAYTSVTCPMCRSKCKRNGGLVICSKCGKTMNADIVRAYNLLTR

>Q8TZH0_PYRFU/310-371 PF07282

WKKIVGKLSYRVPIEFVNPAYTSSTCPICGSKLESRNGLVECFNCGFKADRQFVGAFNILMR

>Q8U235_PYRFU/288-354 PF07282

FRKLQRIIEYKAKLRGIKVIFVNPAYTSTLCPICGGKLSPNGHRVLKCKCGFEADRDVVGSWNVRLK

>Q8YKG9_ANASP/321-388 PF07282

WGAFLQIMQAVAVRRGKHTRGVDPRGTSINCSGCGQRVEKTLAVRVHNCSCGLVIDRDWNSAINLLNY

>Q8YRS1_ANASP/285-355 PF07282

FGELLQILEWVAKNKNKLVVFIDPWYPSSKTCSSCGHILENLDLSIRRWRCPSCQSENDRDGNASLNIKRV

>Q8YWC9_ANASP/289-354 PF07282

HGETRAFLTYKSQRLGMKVEIQCERYSSQVCPSCGQRHKPKNRTYKCKCGFEFHRDGVGCVNIRRK

>Q8YXQ5_ANASP/292-359 PF07282

WYQFRKWLEFFGVKFGRITVAVNPAYTSQECSNCGAMVKKSLSTRTHACECGFVMDRDWNAAINILKL

>Q93EQ5_PHOLU/291-361 PF07282

WHGFIKKLEYKAAAAGVHLVKLDQWFASSKTCHCCGHKMPEMPLRKRIWPCPCCRVEHDRDINAAINIRRK

>Q96XP8_SULTO/295-367 PF07282

FSSTKNAIMEKAKEFGVPIILVDPAYTSSTCPVHGSKIIYRPDGGSAPRVGVCVVGKERWHRDVVSLYNLMRR

>Q96XT5_SULTO/297-363 PF07282

YRRVQYWIEWQAKKHGLLVVYVNPHYSSVSCPKCGKKMVEVSYRWFKCICGYENDRDVIGIMNLNRR

>Q97AM7_THEVO/154-220 PF07282

FYQIEKMIEYKARLLGIPVVYVDPYNTSKECSRCGLKGNRSGKEFKCPGCGHVDNADANASFNIALR

>Q97WZ3_SULSO/295-367 PF07282

FSSMKNAIIEKAREFGVPVVLVNPSYTSTVCPIHGANIVYQLDGGDAPRVGVCEKGKEKWHRDVVALYNLARR

>Q9FCX3_CLODI/170-241 PF07282

WSEFIRQLEYKANWYGRQIVKVGKFFASSQICNKCGYKNEEVKNLNIREWICPSCNETHDRDINASINILKE

>Q9HHG8_HALSA/296-366 PF07282

FDRLQTRLADKAADAGIPVQYVDPAYTSQICHACGHIGTRPEQAEFRCPNDDCWVSVYQADINAAANIAGR

>Q9HHX8_HALSA/177-247 PF07282

FAKLHAQIRYKAVEKGIPVETVNPCNTSKECHACGEVGYRPRQATFKCTNDACWMGESQADVNGAINIADR

>Q9HSD4_HALSA/268-335 PF07282

FARLLNCIEYKAHDAGIDVQLVNEYDTSKTCNRCACEGVRATQGRFECPECGLDDNADKNGALNIGKR

>Q9HSY4_HALSA/184-253 PF07282

FRTLYEQVAYKAEAEGISVKQVGSAYTSQRCAECGFTADENRPTRNDFQCGKCGAEANADYNAAKNIGLR

>Q9PFX1_XYLFA/291-361 PF07282

FFEFRRQLEYKAMMRGGQVVVANRFFASSKRCLTCGHTLNELPLSVRQWACPACGARHDRDVNAAVNLKNM

>Q9UYW1_PYRAB/257-329 PF07282

MNGFQRFVLEKAVEFNVPVVFVSPSYSSKICPGCGAFNVKPDDDALRRRVFNCPVCGFSMDRDFVAVLNLLGL

>Q9V0F3_PYRAB/297-364 PF07282

VRELQRLIEYKAKWFGVPVVYVNPKNSSKTCPACGGHLIPQEGRLMKCLKCGLVEDRDFIAVLNLRMW

>Q9V1M1_PYRAB/302-369 PF07282

FLGIQKAIERKAKEYGVPVIKVNPKNSSRLCPIHNAVVKYESGRFGVCSVGGEVWHRDILAVWNLYLR

>Q9WXQ4_THEMA/302-368 PF07282

FRKLMSQIEYKAQIFGIEVVRISEAHTSKTCPVCGTQNRPSGRNYKCAGCGFEYHRDGVGAINIWKR

>Q9X0B6_THEMA/300-366 PF07282

FRKLIEMIRYKAEQFGIEVKLISEANTSKTCPVCGAKNKPNGRRYHCKTCGFEYHRDGVGAINIWKR

>Q9X8Z6_STRCO/301-371 PF07282

WSSFVGMLKYKAERYGRTLVVIGRFEPTSQTCSTCGVKDGPKPLQVREWTCTACGTVHDRDHNAAINVKQA

>Y012_METJA/58-119 PF07282

WRKIAKKLEYKSVVLYVNPHYTSKTCPVCGSKMKSQEGQVVKCDKCGIFDRQFVRCYNIFKR

>Y1635_METJA/304-366 PF07282

WNGLIEKISYKTIVILVNPAYTSTICPICGSRMESQEGQVVYCSNCLNSFNRQLVGCYNIFKR

>Y751_METJA/192-259 PF07282

FSKLQFFIEYKAKWDGLDVEYVNPSRTSKLCPICGCKLDPNGQRLLKCNNCNLVFDRDVVATFNLFKK

>Y85A_METJA/6-82 PF07282

AKKFLGYLKNKCLEFGVKVIEGNPAYTSIKCPNCGSRLSQLYKLADERALPSRLMYCFDCGFYADRDTVAVFNLIKR

>O58279_PYRHO/30-135 PF07381

PVIDPHVLRSLHRSELRRRILQYLYEIYPSATYLSEIARVVGSDPSNVRGALVGLGNRYNGESSLVYLGLVEEIRNNGFKYYRLTEYGKKVVEMLRDYQAYYRKFM

>Q9HJ88_THEAC/8-118 PF07381

PIVDGNILRSLNRSELRRKVLFYLLSIYPYRSYLSEISRAVKSDPSNVKGCLEGLGVRYTGEESLIGLGLVIVEQSKNGFKYFKINPEIVEEVRNMKNMFSDKKVYTVEIG

>Y905_METJA/4-109 PF07381

AFIDPMIIRSLNKSKLRKKILYLLYKMYPHGIYLSEISRRVRSDPSNVLGCLKGMNGRYNGHFSLIELGLVECVERGGVKIYKLTDYGKKIVEVLKDQDSDFIESL

>GCFC_MOUSE/327-830 PF07842

DLVKRQLKDRLDSMKELHKTNQQQHEKHLQSRVDSTRAIERLEGSSGGIGERYKFLQEMRGYVQDLLECFSEKVPLINELESAIHQLYKQRASRLVQRRQDDIKDESSEFSSHSSQALMAPNLDSFGRDRALYQEHAKRRIAEREARRTRRRQAREQTGQMADHLEGLSSDDEETSTDITNFNLEKDRILKESSKVFEDVLESFYSIDCIKAQFEAWRSKYYMSYKDAYIGLCLPKLFNPLIRLQLLTWTPLEAKCRDFETMLWFESLLFYGCEDREQEKDEADVALLPTIVEKVILPKLTVIAETMWDPFSTTQTSRMVGITMKLINGYPSVVNADNKNTQVYLKALLLRMRRTLDDDVFMPLYPKNVLENKNSGPYLFFQRQFWSSVKLLGNFLQWYGIFSNKTLQELSIDGLLNRYILMAFQNSEYGDDSIRKAQNVINCFPKQWFVNLKGERTISQLENFCRYLVHLADTIYRNSIGCSDVEKRNARENIKQIVKLLASV

>GCF_HUMAN/272-759 PF07842

EIIKKQLNTRLTLLQETHRSHLREYEKYVQDVKSSKSTIQNLESSSNQALNCKFYKSMKIYVENLIDCLNEKIINIQEIESSMHALLLKQAMTFMKRRQDELKHESTYLQQLSRKDETSTSGNFSVDEKTQWILEEIESRRTKRRQARVLSGNCNHQEGTSSDDELPSAEMIDFQKSQGDILQKQKKVFEEVQDDFCNIQNILLKFQQWREKFPDSYYEAFISLCIPKLLNPLIRVQLIDWNPLKLESTGLKEMPWFKSVEEFMDSSVEDSKKESSSDKKVLSAIINKTIIPRLTDFVEFLWDPLSTSQTTSLITHCRVILEEHSTCENEVSKSRQDLLKSIVSRMKKAVEDDVFIPLYPKSAVENKTSPHSKFQERQFWSGLKLFRNILLWNGLLTDDTLQELGLGKLLNRYLIIALLNATPGPDVVKKCNQVAACLPEKWFENSAMRTSIPQLENFIQFLLQSAHKLSRSEFRDEVEEIILILVKI

>O01989_CAEEL/338-817 PF07842

EDILAKLKLRIQERDEALNFRKEEKRKLEQNIEENKSMIAKIEMELPNQSTKYTMYQELRVYSRSLLECLNEKVGEINSIIDKKRDCGKSRTSRLSVRRRQDMRDQHAECMQGRNARMGEAAGRAAERDARRGRRRREREFTLARINHEEGLSTDDEEPTPQSMNDQKICDEVEAVASVLFADALDEYSDLRKVFGRMTDWLAVDPKSFQDAYVYLCIPKLSSPYVRLQILRADFLRKETILTSMQWFHIAMLAGSENAEIDQSHEILVELAPAIVEKVVIPFLIGLFLKLSSNFDTVKEEWDPMSLRQTRHLTTFCSLFEKLPNLTEKSKQFNAFLNAIRERICDCISEDLFMPIFMPNALEQPICRQFHDRQFWTCIKLIKSINALSPLISIAARFELVVEKCVNSQCVMALRTGSKNDVTAERKVRGLLAELDDSLLKMGGRTSFRQLIGTLELIAEEQSKAGRSFHKEIRKFLEKL

>Q84T85_ORYSA/411-936 PF07842

DVASKALQENIRKLKETHKTTVDALVKTDTHLTEALSEISSLESGLQDAERKFVYMQELRNYISVMCDFLNDKAFYIEELEEHMQKLHENRVTAVSERRAADLADESSVIEAAVNAAVSVLSKGSSSAYLSAASNAAQAAAAAARESSNLPPELDEFGRDINMQKRMDLKRREEDRRRRKIRSESKRLSSEGRSANNEHIEGELSTDESDSESSAYLSSRDELLKTADLVFSDAAEEYSSLRIVKDKFEGWKTQYPLAYRDAHVALSAPSVFTPYVRLELLKWDPLHETTDFFGMEWHKILFDYGEQNSESGTDPNNVDKDLIPVLVEKVALPILHHRIMHCWDILSTQRTKNAVDAINMVISYLPTSSKALHQLLAAVNSRLTEAIADISVPAWGSMVTRTVPGASQYAAHRFGVAIRLLKNVCLWKDIFAKPVLEKLALEELLKGKILPHMKSIILDAHDAIARAERISALLKGVWSSPSQKLQPFIDLVVELGNKLERRHMSGISEEETRGLARRLKDILVEL

>Q8BKT3_MOUSE/68-554 PF07842

EIIKKQLNNRLTLLQESHRSHQREYEKYEQDIKSSKTAIQNLESASDHAQNYRFYRGMKSYVENIIDCLNEKIVSIVELESSMYTLLLKRSEALLKRRQDELKCESSYLQQLSRKDETSANGSLAVDEKDQRILEEIEARRMQRRQARELSGSCDHQEGMSSDDELSPAEMTNFHKCQGDILQDCKKVFEDVHDDFCNVQNILLKFQQWREKFPDSYYEAFVGFCLPKLLSPLIRVQLLDWNPLKMDSIGLDKMPWFTAITEFMESSMDDIGKEDGSDKKILAAVINKTVVPRLTDFVETIWDPLSTSQTRSLTVHCRVAFEQFASENEVSKNKQDLLKSIVARMKKSIEDDIFIPLYPKSSEEGKMSPHSKFQERQFWGALKLFRNILLWNGLLPDDTLQDLGLGKLLNRYLIISLTNAVPGPDVVKKCSQIAACLPERWFENSAMRTSIPQLENFIKFLLQSAQKLSSSEFRNEVSEIILILVKV

>Q8MRQ8_DROME/96-578 PF07842

QEILAAIQSRLSELKERSADHSASMARISTELKALKLQQLECQQNAPTAAAKYKFYQEIKCYVNDLVDCLSEKAPVIYDLEKRALQQYGKNQRYLVNRRRQDVRDQAKEIAESAKPITAASRRTPDYEEQVRRAAEREGRRTRRRCERERNDLLSSHLDGMSSDDEIADQQQELSVTTMAQIESQSVDALEDVTDDFSKIELILMKFFAWRKTDMSSYQDAFVSLCLPKVLAPLVRHELVLWSPLLDVYADIENMRWYQACMLYASQADETVEQLKIDPDINLVPALIEKIVLPKVTALVTECWDPLSTTQTLRLVGFINRLGREFPLSGTNKQLNKLFESIMERMRLALENDVFIPIFPKQVQEAKTSFFQRQFCSGLKLFRNFLSWQGILADKLLRELAIGALLNRYLLLAMRVCTPNDAINKAYIIVNTLPTVWLLPNSETLKNMELFIGYIKQTLENCDASNPIFMQSSDKAKQILQRL

>O68272_ALCEU/10-73 PF07879

RLIKKYPNRRLYDTQTSTYITLADVKQLVMDSEEFKVVDAKSGDELTRSILLQIILEEETGGVP

>P71142_COMTE/18-81 PF07879

RIIKKYPNRRLYDTNTSSYITLAEVKQLVMDSEPVLIRDAKTNEDLTRSILLQIILEEEAGGAP

>Q6MR75_BDEBA/17-83 PF07879

KIIKRYQNRKLYDTQQSCYVTLDDIAKMIRTNEEVMVIDNKSKNDITAATLTQIIFEAEKKASQYAP

>Q6NCE2_RHOPA/34-98 PF07879

TTIKKYANRRLYNTGTSTYVTLEDLATMVKDGEDFLVYDAKTGDDITRSVLAQIIFEQENKAGQN

>Q72MS0_LEPIC/13-77 PF07879

KLLKRYANRRLYDPETSKTITLEDVAEMIIKGEEIRVIDNMSGSDITPKILGQTFLKVSLGQRNE

>Q7NYA7_CHRVO/6-69 PF07879

RVIKKYPNRRLYDTATSSYITLGDVKQLVLDNVDIQVLDAKTQEDITRSVLLQIILEEENGGMP

>Q8KRF0_9GAMM/10-73 PF07879

RLIKKYPNRRLYDTRTSCHITLADIRQLVISEEPFQVVDAKTGEDLTRSILLQIIQEAESDGEP

>Q8P8E7_XANCP/19-82 PF07879

RIIKKYPNRRLYDTEISSYITIEDVRQLIIDGEEFEVRDAKSGEDLSRAVLLQIIADQEQDGEP

>Q9WX82_PARDE/10-73 PF07879

LLIKRYASRRLYNTETSDYVTLEDIAGFIRAGRQVRIVDLKTGDDLTRQYLLQIIAEHEGRGEN

>YPH4_THIVI/5-68 PF07879

RIIKKYPNRRLYDTEVSRYITLADVRNLVMDCTSFKVVDTANESDITRSILLQIMLEEETGGEP

(2).non DNA-binding proteins:

>A0YHF6_9GAMM/9-569 PF06315

LAKVILNGFDAFFGDFQNVTLGAQARFEQADWLLVHASMSHRLEMYKKKVRDVAALSDGLADGLADDRALWREAKAEYAVLVETHSNYEIAQTFFNSVYCYVFGHEKIRDVHSFVLAPNSLPEHRDAEVIFTEYANVSDMATTARQILVDTHFNIPFEDIDRDVERIVEITDRLLRGRLRRGQSIKAQVLNSLFYRNKAAYLVGRIVVDGAMLPFVFPFLNNEDGRVYVDTVLFSPDDVSMLFSFTRSYFMVDTAVPSQYVGFLKSIMPQKELFELYSAIGFGKHAKTVFYRRAVAHTAETDDSYIIAPGIKGMVMLVFTLPSYDYVYKVIKDRFTPPKDMTREQVKGKYKLVKRWDRAGRMADTQEFNNLAFDRRRFSDELVAELEKEAPSLLEQKGNALILKHVYVERRMIPLNLYIKDATQDQLYSVMDEYGNAIKQLAAANIFPGDMLLKNFGVTRHGRVVFYDYDEICPLVDCSFRTIPLPKTEEQEMASQPWYNVAANDVFPEEFRLFFSGNRRARDAFDELHPDLYRADFWSDLQRQVKDGRVGDVYPYRRKYR

>Q6SEY0_9BACT/16-195 PF02737

SVSVVGAGLIGCGWAIVFARAGWQVTLQDIDLAKLQGAPKVLAVQLRMLEQHDLCADPAGILARISYESDLKTAVCEVDYVQECGPEVLGLKQELFSELDALTPPETILASSTSGLMASQFSAHLAGRHRALVAHPVNPPHLVPVVEISPSEWTDPEIVRVVVDVMTGVGQTPVTVQKEI

>A7NKD4_9CHLR/227-607 PF01314

LSAIMDERNITSPRKGGLSVYGTNVLMNITSTMGALPTKNSTLTSFGPGAEKISGEYVKEHILVDDPTCHACPVACKKEVEIKEGPWKGLRMESLEYEPAWSLGANCGNDDVNAVAKLIDLCNDYGMDAIETGHPISIYMEATERGYTNGDGGLVWGDTMGMVELVRKIAFREGIGNVMANGADATAKYFGHPELAMTVKGQGVPAYDPRGLKGMGIAYATSNRGACHLRAYTPAAELGVTPLGALKVDPLAWKGKGELTKIFQDLHAFSDSLDLCKFSAFAEGAEEYAAQYSAMVGVECTAEDVLKIGERIYNLERYYNNLAGFGPGSDYLPARFTQEPSTMPGSQGHVCELDDMLAEYYAARGWENGVVPESKLRELGI

>OXAA_MYCTU/40-278 PF02096

WALSVMFLVFTLRALLYKPFVRQIRTTRQMQELQPQIKALQKKYGKDRQRMALEMQKLQREHGFNPILGCLPMLAQIPVFLGLYHVLRSFNRTTGGFGQPHLSVIENRLTGNYVFSPVDVGHFLDANLFGAPIGAYMTQRSGLDAFVDFSRPALIAVGVPVMILAGIATYFNSRASIARQSAEAAANPQTAMMNKLALYVFPLGVVVGGPFLPLAIILYWFSNNIWTFGQQHYVFGMIE

>O88029_STRCO/104-167 PF01648

LGIDAEPDGPLPDGVLESIALPAEVALLRRLGGARPGVHWDRLLFSAKESVYKAWYPLTGQWLD

>Q0U449_PHANO/246-624 PF03081

GTNGIGTYVAAIEGIFVAEYDNITNIFPREEWSSVCEATCQEPLGEFSKTLRELNGHIQKNLLTDCFLGYEICGMVRRLSIRLQDTTGALKGQIYDSVKPVRETSKMSMGKLLDDVRSKTQSLIALPIDGGAVPITTETMRRLQEMTNYLEPLSSILASLGEGGWNAGSASNSSTTLDVGPDSIKLFGQYAADTIDTLLSNLAAKARALLKGKNLQGIFIANNVAIVIRMIRSSELAPLLDINSKKMVDWRKQGTAMYLEAWREPSGHLLDVQYTNRSKERPQSGGLDSAAIVKALGSKDKDAIKEKFKNFNTSFDTLVASHKGYAMEPEVRNQLSKEVQNIIEPLYIRFYDRYREIDKGKGKYVKYDKSELVKALSSF

>Q9RYB8_DEIRA/290-522 PF00198

EDRETRVPLRGMRRAIANQMQASHLYTVRTLTVDEVNLTKLVEFRQRVKDEAKAADVKLSYLPFIFKAITVALKKYPSLNTSFDEATQEIVQKSYYNLGMAVATEAGLTVPVIRDVDRKSIFDLARDVVDLAGRANAGKLSPDELTGSSFSVTNIGSIGALFSFPIINVPDAAIMGVHSIVKRPIVDEHDNITVAHMMYLSLSFDHRLIDGAEAARFCKEVIRLLENPDRLML

>OXAA_BORBU/341-544 PF02096

PNWGLSIIFLTIVVRILIFPLTFKGFRATAELSKLQPKMKELQAKFKHDPKKLNEEMGRLYKEEGVNPLGGCLPVILQLPIFFALYSLVNNLFLLRGASFIPGWIDDLSIGDSVYHFGYKLYFVSWTDIRILPFIMMFTQLGSTIVSSNMDLKNLGAQQKFLYFGMPIMFFFILYNMPSGLLIYWITTNIFTILQQYYIKMHLS

>A4YBG1_SHEPC/1-800 PF01295

MDQQDLFPDIAERLNEVRIARALALLSPLQKHLFHLIPFLIHQNSVQYPGYHHPNTPSGISGYKEGKNASLACDVFKLPFIPQEMDTYAFEGVYAMGSTASFGQNVKSDVDVWLVHHPQLCDEDLALIRTKANRLTAWFADYHFEVNFYLVHPQQFSGGQKGRSACQASMAHEHSGSTQHWLLLEEFYRSQIRLAGKTIAWWPNAKLNPDLLYLGDVHELPASEYFGASLWQLYKGLNKPHKALIKVLLLEAYASEYPHSHLLCDRLWQKTLEGDFSASNDAYYAIYEAIEIYLLKQNDTRRLEIVRRCFYLKCGVYLSRAKQGKDWRYPKMQQLVQDWQWPESLINTLDDCEHWHSGQLNWFNEQLNELLLASYQTLLRFASTHELNEGLRIEELGMLTRKLHTYFTQDEDQIAKLNLLWSSSVAESEVTMVSSSRESQYYLYRQGPQPKNLLGESAICKGKTPSALMIWACLNGVSTPDTKWYEFGQSKLKSKRLTDAAKRLLHFIDHDWRVSKRDLCQPWHFRKLIFVLNLDCDPTINWRGQEMMVDFMNANVFSLGRKKENMLGALDAICLNSWGEWQCHRFEGDTAVLQALAFVTPGLRRASLPVDMDVISCSQKLRPQLKLAVKNLLKQTARLCQQVQQSSILVQPLQISHIRYGIFFNPLGMAYEDLSDAKSFYQQLSRSHLVRLPRPALGDDPFSSMPNIIQNFAAKGAIQYFLRQRGDNLDVFILDEENQLSHYVQHGSNMSELVNKVSHHYVFDEHYAARARFNIPQFFHLVRVAGDLTVAPFGIDVTDQ

>3HAO_YEAST/1-155 PF06052

MFNTTPINIDKWLKENEGLLKPPVNNYCLHKGGFTVMIVGGPNERTGYHINPTPEWFYQKKGSMLLKVVDETDAEPKFIDIIINEGDSYLLPGNVPHSPVRFADTVGIVVEQDRPGGENDKIRWYCSHCRQVVHESELQMLDLGTQVKEAILDFE

>YA33_SCHPO/2-437 PF07247

STGRLEIYSVKRNVLNMFSIVIVSATYSISLDSTVLYPAVYHAIVNQPMLGARIHNCHSKIPIVKKLKTIDLDKVVKYADDQKVDSFCNNALQNFKLCYDDETLPLWMVYVLNDKSELVFIYDHSLFDGGSGPLFHKYVLEGLQMSKTNFSSSTVPVSELPLPKNLEKLIDVHPSWFCLMKALWTNSGLPFSKGFRSPSYKGHAPVRPFSSHTIFFSISNAVVKNIKQLSKNIDASFTSIFYSVFMLSIYYAIAKNGKVNLDMLIDVNARRFLPVAKQTMGNYVFSYVHHLNGFQPSQRQEDYKHTMVDLATEFSHRLKAALSNPREMSQQIGLLSYIDIEDYLLKSCEKTRGNTAEISNLGYFSFPADSSVKIKNMAFAQPCSSLSAPFVLNVITVADGPCSFSLSIFDDGNTEQTHELAIKIRDKFLSILEKVS

>Q6KYW3_PICTO/2-174 PF02737

RVTVIGAGTMGSGIAEVFALNNHEVLLSDVSNDILNNGRKKIEASLEKFKEKGRIKSVEDVLEKISMNTDINAQESDLYIEAVLERIDVKRDVLSRIRSDSIIATNTSSISITYLSKFVRNPEKFIGMHFFNPPPIMSLIEIVRGNSTSDETTKRIVDISRSLGKTPVEVNDF

>Q7PSK0_ANOGA/629-720 PF00207

SWLWKNVTIGSSGSLKLTEVVPDTMTSWYLTGFSIDPVVGLGIINNPIEFTTVQPFFILECLPYSIKRDEASEIQFIVVSNLQEGHTVDVTL

>Q7PMK4_ANOGA/424-515 PF00207

SWLWKTDKIGSSGSATTKESVPDTITAWHLTGFSIDPVYGLGIIKQPLQLTTVQPFYIVPNMPYSIKRGELVELQFIVFNNFPKKYKASVTL

>Q703Z6_THETE/222-607 PF01314

FIAESTKLAQRLMSGPTAKALHIYGTNLLTAVINSIGAYPTRNFETAYAEEADKLTGEYIKKNYVVETHACMMCPIGCTQHVMVKGGPFKFIGKAKYEYENTWSLGANIGLFDPEADLRLQKLANELGFDTISLGNTLAAALELAKMGKLPVNIDWGDAGALVDLVIRMANRSDIGDDLAEGDYRLALKYGEPRVFRGSRGQGLPAYDPRSLKGFALSYFTANRGGDHLEAYSPTWEVLGVPEKVDPLCETPECIEKQARIVIYAQHLMALTDSVTYCKFATLDREGIFEKDLAYLFNLAYDWDVTPEDMLTIGERIFNVERLFHVKEGKWVKDELNPHFRNPIPTGPAKGHTAAKMFDEGIKIYYKLRGWVDGKPTYDTLKRLGL

>O30159_ARCFU/214-578 PF01314

YREVYDEIYRMAIESDAMKKYHLLGTAANVIPLNELNALPTKNLKAARFEKAEEISGEALAEHNLGRRIACSHCPVACIHLAALRLPYENEPYFFRTIMISYDYELIYSLGSMLGIGRRDDLLRLIHRVETYGLDAMSTGVCLAWATEALERGIITERETLVKLNFGDVESYLKAIDYIYEQPTEFYKHLAMGVQHASEIYGGREFALAFGGNEMPGYHTGYAAVLGYLTGMRHSHLDSAGYSLDQKVKDLSPEEVINRLIEEESWRQVLSSLVVCFFARGIYTPEVISRAFEPLGYSISPDELKNLGTEIYREKARLKVAMGFNPKKLSVPKRIFETPSLHGKLSEEFMARALDYYRKRLEEML

>A1I9B8_9DELT/215-617 PF01314

FDKIKKTATDYINRNPWTSDGQRKYGTLSIVNITRTTGILPVRNFQDGTSDNAYKLTGELIREKYNTSHHTCKPCTILCGKKGVFDGKEMPVPEYETVGLMGSNLEIYDPVIIARWNKVCGDMGMDTISAGGTLSWVMEATEKDLVKSNLKFGSPEGVDEALADIAYCRGFGAEMAGGSRRLSEKYGGTEFAMHVKGMELSAYDPRGSYGHGLSYAVANRGACHLSSTMMAMENFMCFLAPHTTLSKARWTKVFEDMWCCVNSLHTCLFTSYAYTTELLIPRMSSWISTLIGMQFFPQVLTMVVDFSVVYRKMWQAVTGISISKSEFLRAGERIHVLERYMNTRMGASVKTDTLPKRLLTESRKCDKKGRTVPLEEMRDQYYRLRGFTPEGKPSPELLKKLEI

>Q7VXA7_BORPE/103-331 PF03417

WRGDEDTRWIAHNEDGDPFLYGRCHMVDVRPDDAPGYISFYYPGSLPGHTFAANRAGLVQTINNVRIRQRHAGVPRMLLARAVLDCATLDDALAVLRDHPRAGGFHHTLGAAGEPRLYSVEASPAACSIGEVARGNGHANHLVHPGSEAIGQIVTDSSRSRQRHIENLMDHWQPPVDGARLVATLLDREGELPILRCSADDPDEENTLATALFEMRDGGLTLQVHDRRD

>Q2RKJ9_MOOTA/216-599 PF01314

FREAVLASLAKIKANDVTHGGLPAYGTGVLVNVINAHGGLPTRNFQTGIFPGAEKISGEALAATYLVRKKACLACPMACGRATMVPSGPYAGHGEGPEYEAQWSLGADCGIDDLAAILKANFLANELGYDPISFGSTLACAMELYEKGYLPAGDTEVPLEFGNAAVMVETARKVGYREGIGDLLAEGSYRLASRYGHPELSMTSKKQEYPAYDPRAFQGIGLNYATSNRGGCHVRGYTIAAEALGTPVQADPLSSEGKAALDKAFQDLTALVDASGICLFTTFALGAPDVASMLATATGVPYTEESGLLAGERIYNLERLFNFAAGLTKADDTLAPRLLNEPMPEGPAKGKTSALTKMLAEYYQLRGWDEEGRVTAATRERLGL

>EXO70_SCHPO/244-610 PF03081

GKEDVSINLVALSRLLPAVASELLLLFDQVTAKALYPKIVKPAINTVTNATRQLEGVYEKRGAAENFVLLSLIDCIVVTRQNMNNLMPFEDASFLGFVNGVGREMEKILISSISRLYNGTCHNNKTVPLTTDRVSEMTHGIMSFLNELAEHENASYLLESIGNWGWRHEINADLSPARSVQDITRNYVMDCMDSYLTSVQTAAQAVDTIGWKMGVMLLNISVYFEAKCLESKIASFLQDVDLEKLGDRSQKYSTMYMEVWRQCSQNMLDSTYTKSQNKSTMSAKEREITKEKFRNFNEQVTSVVQVHRESVRFETGVATFLLQEVKKTVLPLYQRFYDKYINSDFTKNKDKYIKFTKADLDSFITSA

>CYAA_AERHY/1-827 PF01295

MLVKLDTLVARYDELNRLKTQRALGLMSRYGQQVFQLLPVMLHFNHPLLPGYVAGDVPHGIWSFTANEAQQAFIQDLCQNANCQNGLTTHDKSIQGLYSMGSTSSIGQCCHSDLDIWVCHVAGLSQERLELLDLKCQQLSKWAEQRGVDLNFFLIPEDKFRQRNDAQMQGESCGSAQHLLLLDEFYRSAMLIAGKRLLWYLVPAEYDDRYDEYVNGLFAHGKLSQDDWLDLGGFDRIPAEEYFGSALWQLYKGIDSPYKAVLKSVLMEAYSHEYPNTRLLSVTSRDWFQHNEGMHYRLDNYCLMLDKVTNYLKSIGDMQRLDLVRRCFYLKVCDGLSHPKEDHSPAWRREQMTQLVAYWGWSPERLHHLDHRQEWKVEDVKVAHAELLEALMQSYRNLIQFARRNNISESINPEDIGILSRKLYAAFESLPGKVQRINLTIAPDLSEPDLSLVQVPHGRLNRAGWYLYKHSLEPADIIGRAPLEYNGYISKLVSWAYFNGLLTPQSRVHLFNQGSDLHIDNLHQFCRDLSGTFPVKYPRATNLALSRPCEIRQLSIFLNLETDPTSHWVGQVIEFDANAADVFSFGRNLECLVGSVDLVYRNSWSEIRTLHFQGDEAVVDALTTILGKMHQDAAAPEMIEVFCYSQHFRSLVRTRFQQLVAECIELRLARDKQQLVKTLALGKEKYGIFFERRGVSVKKLENAIDFYRHISHNKLDHLPLRLDKTHSQHLPGIVDSYASEGLVQFFFDTRDAGTNIYILDEANRVEIYQHFAGNKDELVQGVNRFYTSSHERFSDAGQFSNFNLPPYYEIVQVNGELEVIPYRSQGP

>Q0AWA3_SYNWW/216-597 PF01314

FMQAITDARNKLKAHPVTGAGLAAYGTNVLVNILNEHGGLPVKNFSEAAVFAKAENVSGEYQAEHCLVRNKGCFGCSIGCGRVSRNRGKYKGIGEGPEYEATWGLGPNLYIDDFEAISKANFLCNELGLDPISLAGTLACATEMMEKGFIPQEKAELCWGDADMLVEMTIKTAYREGFGDELAEGSYRLAEKYGHPEFSMSVKKQELPAYDPRGQQGIGLNYATSNRGGCHVRGYMTSPEVLGIPEKVDPDSTEGKAALLKIFQDLTALVDSAGICLFTTFGQGLPEIAEQLRQATGLDLSDEEFLLAGERIWNLERSFNLQAGISSKDDTLPPRLLREPMKGGPHQGNVVRLELMLPEYYTLRGWDQAGVPTPEKLQELSL

>3HAO_CAEEL/1-151 PF06052

MSGVTAIEIPQWIQDNQEDFVPPVCNKCMFSDQLKVFYVGGPNQRKDFHLEEGEEFFFQRKGDMVLKVIEKGQVRDLVIKQGEMFMLPARVEHSPQRFSNSIGLVVERERKNTEFDCVRFLVGSSNITLFERWFYLTDVVKDLPPLIKEFY

>O84058_CHLTR/134-364 PF00198

DHRESRESMSAIRKTISRRLVQSLHDSAMLTTFNEIHMGPLIALRKERQEDFVAKYGVKLGFMSFFVRAVVDSLKKYPRVNAYIEDNEIVYRHYYDISIAIGTDRGLVVPVIRNCDQLSSGEIELQLADLASRAREGKLAIHELEGGGFTITNGGVYGSLLSTPIINPPQVGILGMHKIEKRPVVREDAIVIADMMYVAMSYDHRIIDGKEAVGFLVNVKEQLEQPELLLK

>O24813_STRSQ/92-155 PF01648

VGIDAEESAPLPDGVLDLVGLPXERDQVERLGAQSDAVPWDRLLFSCKEAVYKVWFPLAQRLLG

>Q9IIH9_ADEF1/1-444 PF01686

MQTPARVFAPTEGRNSIVYGNLPPVQDTTKIFFIDNKAADIDAYNQQKDHSNYFTNIIQTQNLNAEDSSVRQIIMDERSRWGGDLHTITKTCAVNCCDFFQSNSCKVKLMVDKNKELYEWFDLVIPEGNYVLNEVIDLLNESIVQIYLANGRQNGVLESDIGVKFDSRYMFLGRDPVTSLVTPGAYVYKGYHADIFLLPNCAVDFSKSRLSNILGIRKRDSYTDGFILTYEDLQSGNIPALLDIKKFQQNGEISPVLQDSSNRSYHVTGEPGNYETLYRSFLCAYLNNQSTAFKNYLLVNSDISAGIGQLYWSLPDIFKPPVTFKMEQKVEMSPVVGTQLFPLIGKSVYSGASVYNQMIESATNSVHVFNRFPDNQILMQAPCMNTHLVSENVPLSTNQGTLPIHTVIPGVQRVILTDDQRRPCPYVVKSIATVQPKVISPATL

>Q21UJ2_RHOFD/217-617 PF01314

FMAATKAAKKILAENGVTGTGLPAMGTQVLMSVINEIGALPTRNHQEVQFEGAKDIGAEAMATPRKTDGKKQLVTNQACFGCTIACGRISKMDEGHFTIENKPQYRGANGGLEYEAAWALGAANGVNDLEALQYVNLLCNEEGIDPITFGATIGAVMELYGMGILTKEQLGIEAPFGSARAVAFLAEETVNGRGFGKEIAMGSKRLTAKYGHPELSMSSKGQEFPAYDSRAIQGIGLAYATSNRGGCHLRGYTIASEVLGIPVKTDPLESKGKPELVKAFQDATAAFDSAGVCIFTTFAWGLQDLSPQMQGACGEQYTIEELAKIGERIWNMEREFNNRAGFTKADDSLPPRLTSVEGACKTGPAKGKFNELATMLPLYYEARGWDTEGRPTAATRERLSL

>ODO2_SCHPO/217-451 PF00198

SRNEDRVKMNRMRLRIAERLKESQNRAASLTTFNECDMSAVVALRKKYKDEILKETGVKIGFMSFFSKACTQAMKQIPAINGSIEGEGKGDTLVYRDFCDLSIAVATPKGLVTPVIRNAESMSLLEIESAIATLGSKARAGKLAIEDMASGTFTISNGGIFGSLYGTPIINLPQTAVLGLHAIKERPVVINGQVVPRPMMYLALTYDHRMVDGREAVTFLRLVKEYIEDPAKMLL

>Q93QG7_9MICO/6-185 PF02737

HVGIFGAGSIGTAFALLFADAGFAVRIFDPDPSALERSRHVIDQRITELQRFTLLASNPSEVRELIEIVSSARTAASGAILVQEAGPEDVQTKQHIFEDLTAVTSDETILASASSAIPSSRFVDVHSAFRSLIGHPGNPPYLLRVVELVGNPSTEEQTILRAGQLYEQAGLSAVRVNREV

>Q3V653_9PULM/716-807 PF00207

TWLWLETSIGTNGTASISTQVPDTITPWVAGAFAVNSVTGLGVVPTQTHLRVFRPFFVSLNLPYSVTRGEQLALQAIVFNYMSDDMQVRVTL

>Q9ZPI6_ARATH/492-585 PF00725

GFAVNRTFFPYSQAAHMLANLGVDLFRIDSVITSFGLPLGPFQLGDLAGHGIGLAVGPIYAKVYGDRMFRSPMTELLLKSGRNGKINGRGYYIY

>A7PSW5_VITVI/298-662 PF03081

IKNWLEGIKISITTLFTGERILCDHVFAASDSMRESCFAEISKEGATLLFEFPRLLVSKSKRSPDMAFRTLDVYTAISDNWPDIESIFSFESTSSVRLHALTTLSKLGESVRMMLSEFESVIQKDSSKSPVAGGGLHPLTQYVMNYLSHLADYSSILGDIIGDSPPPVQSPLPEFYFESSDTDNTPAPAISVRLAWIILFLLCKLDGKAKQYKEVSLSYLFLANNLQHVVSKVRTSNLRYLLGDEWISMHESKLRQFAANYERLGWGHVISSMPENPKAAISPEEAKETFRKFNLEFEQAYRKQSSYIVPDPKLRDEIKASIARKLDSVYQEFYETHRETLATIRNAEMLIRFAPEDVRNCLSDL

>A0Y6R6_9GAMM/6-567 PF06315

IAELILTGFKKHYQLFQKITAQAPLAFAKQDWQAINDISRLRISYYDERVNETIHTLKQDHPSYELDETLWLEVKKLYQGFLCFHPQAELAETFYNSVFCRLYHRRYFHNDFIFVQATLKDAPSVPVESEYRSYFPVVDGLKPTIKRIINHFDFKAPFVDLERDIRLLVKAFYKQAPDTHHQPWKMRFDILHTAFYRNKAAYIVGRVVSESGVQPFIIAVLHHEDKGLYLDALLTKSSQMRVIFGFARAYFMVETHAPSALVRFLNELMPNKTMAELYNAIGFHKQGKTEFYREFLNHLTHSNDEFTIAPGTPGMVMMVFTLPSFGYVFKVIKDKFGESKPFGRDTVLKRYQLVKSHDRVGRMADTIEYSNVVFPLARFDSNLLEQLHKTIGSSMVIEGDWLIIKHLYIERRMTPLNLFLENADDESAADAIEEYGQALKEMIAVNIFPGDMLLKNFGVSKHKRIIFYDYDEVQYLTDMNFRALPKAKSYDDYLMDEQTYSVAPQDVFPEQLCTFVTPNPTYKQFLISTHPELIDVNFWKQAQQNIKNGLINHIYPYPTAQR

>O17761_CAEEL/41-220 PF02737

SVAVIGGGTMGRGIAIAFCLSGFETYLVEVNNKAAEFCKNELEITYKREKAFRRLNDSKVEKLRKNLQITTDFQKLNNCDLIVEAVFEDMKLKKELFTKLDKICKPSCIFGTNTSSLDLNEMSSVLRDPTKVVGIHFFNPANLIRMVEVIYGSKTSSKAVATAFEACRSIKKLPVLVGNC

>O93737_PYRFU/231-578 PF01314

MKVLLREFWKELFSMTTFREWGTGAGGYSVGHDRSSEPIRNWQEEYHDNEEISVVNFENRTWIKKYWADYGCPVNCMKISYLRYGPYKGSISDAPDYELQAYMGTNLGIFEPEKIVYLSYLVDELGLDGINTGNILGFAAELYQRGILTKEDLGFELNWGDEKAFAKLLHLIVEKEGIGKILAEGTYRAALKISEIKGIDVTKYAVHVKGIAVGAHGIRSELDYTKDISYAVSVQGGDHTSTAALPAKGYTGELVEAFYDSAVICNFVTKPGFEKIIEFGNALSGFNITPEQWLNEIGLRIIHLQRILLLLGGPDVYWDPRKDDDNPPRFYEPLPSVLSKERHPTGGY

>A0IZJ0_9GAMM/1-802 PF01295

MVNDQSHFVETAQRLNRIRLARALAILSPLKRDLLRLIPLLFHFHRVGYPGYNGPLTPSGIFNYQASESEYSACSTLGLVPPNLHLVNHPAIEGIYSMGSMASFGQNPKSDVDLWLVHDRLLSPNECRLLEEKSALISQWFEQYGLEVNIYLVHPEQFIKSSDSDEEFQTSIGLEHSGSAQHWLLLEEFYRSQICLAGKTVAWWPDAKRSEELLFLGDVSQIPASEYFGASLWQLYKGLNKPHKALLKVLLLEAYASQYPNTCLVSGKVWQRTMEGDFSAENDAYFLLYESIESYLLAQDDLRRLDIVRRCFYLKCGLRLSEKNQAKDWRYHKLSMLVENWKWSNSLLQTLDHCEDWHCGQLQWFNEQLNELMLGSYQTLLQFASTQKLSKSLKLSELGLLTRKLHTYFSSDIQQIISLNRLWSDSVCEADLTIIYSKNSQEYCLYRCKPAPRNFIGQRAVYRSKSKAKLMAWACLNGVANDATAWYEFSEGKRKSSSLSKASKRLVTFFEHADLRVSKMDFCQPWRYKKLVFLLNFNHDPTGLWQGQEIMVDYMNANIFSLGREQKNMLESIDIICLNSWGEWHCHHYEGEFGLLDALCFITAGIRRGAGDIAIEVISCSTKLKSQFERTVGNLMQRAIRLSLQVSSSATLVYPLKVSGIEYGLFFNSKGMVYQNLNDTKGLFQQLTKKKLLELPRPDLGNEPFSKVPEVIQDFAARGAIQYFLRQNEQELDVFILNESNELDHYQQSGIDIDELVSKVSHHHAFEEFKASQQRFNLPQFFRLIRVDGRLRALPFGISVDE

>FADB_PSESM/316-495 PF02737

QAAVLGAGIMGGGIAYQSAVKGTPILMKDIREEAIQLGLNEASKLLGGRLEKGRLTAAKMAEALNAIRPTLSYGDFGNVDLVVEAVVENPKVKQAVLAEVEANVGENTILASNTSTISISLLAQALKRPENFVGMHFFNPVHMMPLVEVIRGEKSSEEAVATTVAYAKKMGKNPIVVNDC

>Q6DEG7_DANRE/20-280 PF00797

DLDTLRTIHMLHVMKIPFENLSIHCGEKNTTDLNIIYHKLVKSNRGGWCCENNLLFSWVLKEMGYKYTTLGSKVFNKFQNDFYPVDSHLINMVEIDGKFYLTDVSYGVSCQLWYPLEMISGKDQPQPPGVFRLLKEGVRWTLEKTSRKQVVKDKAYANSSLIDKRLTKTMYSFPLTPRAKEHFVDTLDCLQTSPDSRFVLKSICSLQLPEGFRALIGWTYSEITYNPEDDSDMVEMKEIPNCDIETVLREKFNVVLVNKLT

>A2WYH2_ORYSI/258-597 PF03081

IINIRGGHHMAAASQWHMAQREIDAMVKEIHSKAGRQHALVWSYKLRLLAFADEITKLQLSPDKLFVVLRLLKVLNPDFFLVSQCRPEEFSVARYDDTLQKLRMAMYHMLRELKILIQTRASRRVPPGGGIHEVTRYVMNYIRLLLHHKTTLGLILGNDDCNKDNERMDSLDHIVQDLIICLESMLNKAPEAYESQGLQCFFLMNNLHFVVKQVEGSELISLLGQSWVQVHREFIEQYLKTYVDLSWGPAISCLSARTGMLGGCFSQPSSTVRFSLQFDSTYYNQECWKVEDPQLREKVRRAVCDKVILAYQAHLDKYMKAKRKHEWYTPELLKAQLMKL

>Q88DP3_PSEPK/28-172 PF07696

LPLGKAMQVYEDPDGNASIAQVSAPGFAKHFQPHHEDVLNAGYSTSVFWLKVELRPTAAPNAAPRSWLLELAYPPLDHLELYLPDSSGLYRLAQRTGDALPYDSRQIRQNNYLFELQLPPGKVTTAYLRLHSQGSVQAPLALWSP

>Q73Q34_TREDE/9-188 PF02737

KVAVVGDGTMGHGISEVFAKAGHTVQIIGLNDASLKSALDRIKLSLNEFVAEGLVSASDIDTIVGRISFSTDIQKAEDAAIVIEALPENMDLKTETFGKLEKICPQDTILATASGHSVSEVIAQVKKRDRVIATHFWFPPQLLPLVEVCGAPETSKATIDTTCELLKGIGKKPVVIDKEI

>Q8WPV9_SUBDO/1-152 PF06052

MAGLEIINVDRRVEENKGSFLPPVCNKLMHGAGQLKVMFIGGPNQRKDYHIEEGEEFFLQLKGDMCLKIVEKDAHKDVNIKEGECYLHPSRIPHSPQRFDNTVGLVIERDRSKDETDGLRYYVDGTTDPLWEEWFYCYDLGSQLGPVIKKYF

>Q1ZCS9_9GAMM/1-830 PF01295

MNPEIKRLRDKADQFNRLRIQSAQVLMTENEYNVFQTFPILLHYNQVNLPGYINSDVPIGICQFQITAKQTQLLSQFLNVDKLYKTSQSCDILGLYAMGSTSSIGQCSHSDFDIWICYPHQIDSRRIQLLNEKATLITAWAETFNVELNFFLIPDNKFRVKNNAGMNSDSCGSSQHMLLLDEFYRTALRVAGKRILWPLIPIDHEKNYNEYVENLYKNKELNENDWLDLGGFYRIPAEEYFGATLWQLYKGIDTPYKAVLKTILMEAYSSEYPNTELVAMSYKRCFQNQAHYDERLDPYCLMLEKVTHYLIKIDDLKRLDVVRACFYLKTEENLSTPCHNNNTAWRRTILNRFINQWQWDENQILDLDNHLNWKVCAVEKAQNVLLDALMISYRKLHSFARRNNISESISVEDLGILSRKLYAAHEKLPGKIDLINANGRHNLSEPDLSFIQVPENRKSNNAPGWYLYNSSLDNHSLINTPLLMHSQYLSKLIAWCHFNGLYKKQTQLYLYNQGSDLMDAKLNQFMDDLHSIFPLRIAKATNKALTQPCEIKHLAIFLNVEKDPTQHWQDTTESNNDEIGNVLSYGINDECLIGSIDLIYRNSWNEVRTLHFNSNYSVVEALNTLLGKMHQDAKQPEKIEIFSYSRHFKKQLSDTFLTKLQEFIQLRLTTTSRKSLQTLWTGGKKFGFYFERTGVSLQHLESSVDIYSHISEKKVSSSAINLRNTHFEQTVEMIESHLSEGLIQFFFENHEHGFNVYIANVDNEIETFQHFSGNKDDLVKSVNRFYASNRTQSDNTNNTINFSLPQFYDIQLEAQEGLSLRSFKGAIKNQ

>O58641_PYRHO/235-620 PF01314

LMKVNFEIQRRIKESPSFKKYENWHANISLSLLKARRPYFGDYEEEFWEEAEEAAKNAKKFFEEKASLRPSSCFSCPLRCWAWVEYKGEEAPMVACQGTFPAIIFILKIKDPELAWKVYLKLQREGMDIMSTVAIIAYASRLGLVKLGSEDVLNLIDKIINREGPGNILAEGIKRASEHFGVPAVYVKGGMESWSSDIRPFRGVALAGAVSESGSISRAIHGFPASSYYTKPEKAKKVAKEIVGDESAAEPTNYKGKAKLVAYFENEHIIADSLGVCDTPMLSVPLELWAESFTAATGIEMSPEKLHFYAEKIRTLERMFNVRRGVNREKDTLSPRLFESVIHSGPWKGVKLDREKFEEMKDEYYMLRGWNENGIPTEETLKKYGL

>ALB3_ARATH/140-356 PF02096

YGFAIILLTIIVKAATYPLTKQQVESTLAMQNLQPKIKAIQQRYAGNQERIQLETSRLYKQAGVNPLAGCLPTLATIPVWIGLYQALSNVANEGLFTEGFFWIPSLGGPTSIAARQSGSGISWLFPFVDGHPPLGWYDTVAYLVLPVLLIASQYVSMEIMKPPQTDDPAQKNTLLVFKFLPLMIGYFALSVPSGLSIYWLTNNVLSTAQQVYLRKLG

>Q23571_CAEEL/214-445 PF00198

LKEDVAVPIRGYTRAMVKTMTEALKIPHFGYNDEINVDSLVKYRAELKEFAKERHIKLSYMPFFIKAASLALLEYPSLNSTTDEKMENVIHKASHNICLAMDTPGGLVVPNIKNCEQRSIFEIAQELNRLLEAGKKQQIKREDLIDGTFSLSNIGNIGGTYASPVVFPPQVAIGAIGKIEKLPRFDKHDNVIPVNIMKVSWCADHRVVDGATMARFSNRWKFYLEHPSAMLA

>Q88L88_PSEPK/9-188 PF02737

QVAVIGAGTMGRGIVISLANAGLSVLWLDCNAAALEAGLGMVSQAWAQQVDKQRITQAQADACLARVQAVDGYPALAEADLVIEAVYENLALKQEIFCALDAHLKPRAILASNTSALDIDAIAAVTQRPSQVLGLHFFSPAHVMKLLEIVRGTHTDQKVLDAAKALGERMGKVAIVAGNC

>Q9RUA4_DEIRA/309-488 PF02737

SAGIIGAGTMGGGIAMNFLNVGIPVTIVETSQEALDRGLGVIRKNYENTAKKGRMTQDDVEKRMGLLTPTLKMEDLAGADIIIEAVFENMDVKKDIFTRLDKIAKPGAILASNTSTLDVNEIASVTGRPEQVIGLHFFSPANVMKLLEIVRADKTSDSVLATSLALAKRIKKVGVVVGVC

>Q734X0_BACC1/92-272 PF02826

IASLIMSSRNIINGVSWTKNLEGEEVPQLVESGKKQFVGSEIAGKRLGVIGLGAIGALVANDALALGMDVVGYDPYISVETAWRLSTHVQRAFSLDEIFATCDYITLHIPLTNQTKGIIGEHAIEKMKKGMRLFNFSRGELVDEKVLQKALEEDVIAHYVTDFPNENVIKMKNVTATPHLG

>Q54813_STRPE/21-91 PF03992

TLSGSAEDFEAAFAETAEFLCRRPGFRWHALLVPADTGPGSADARPQYVNIAVWDDEASFRAAVAHPEFPA

>A1L1H6_XENLA/735-826 PF00207

TFVWKMVVVDSEGKLSFTEKVPDTITEWKGSMFCLSEEQGFGMTTYTANYTSFLPFFVEPSLPYSITRGETMVLRAFVSNYLQKCITISVTL

>ERG26_YEAST/8-280 PF01073

LIIGGSGFLGLHLIQQFFDINPKPDIHIFDVRDLPEKLSKQFTFNVDDIKFHKGDLTSPDDMENAINESKANVVVHCASPMHGQNPDIYDIVNVKGTRNVIDMCKKCGVNILVYTSSAGVIFNGQDVHNADETWPIPEVPMDAYNETKAIAEDMVLKANDPSSDFYTVALRPAGIFGPGDRQLVPGLRQVAKLGQSKFQIGDNNNLFDWTYAGNVADAHVLAAQKLLDPKTRTAVSGETFFITNDTPTYFWALARTVWKADGHIDKHVIVLKR

>Q9F0Q6_9ACTO/105-168 PF01648

LGIDAEPNGPLPDGVLAMVSLPSEREWLAGLAARRPDVHWDRLLFSAKESVFKAWYPLTGLELD

>Q9SF50_ARATH/227-599 PF03081

MNRWIEAVKVSMKTLFNGEKTLCDEIFESSVSLREFCFRDISKEGALLLFGFPETITLRDKKNPHPEKIFPLLDMYCTITDNLLAIEAIFSFPSISNVRTQAHSSLSRLSESILAHLMDFESQIRKDSSKTVVRGGGVHPMTISAMNHISRLAEYSNALINILKGSSSSSSAKALLPKSYFNVSESEESPVSELKARFAWMILVLLCKIDGKAEMYKDFSMQYLFLANNLQHVVSRARSTNVKHVLGNDWIAKNSEKVRQFARSYERLAWGPLASMCPAISTSEAVEMSPEEAMMQFKKFNETFESTCEAQSECIVLDPKLLDEMRISIGRKLLPVYRDFYNAHRNAVMLAGTEGQWNVRYNPEDIGNHLSEL

>Q82BZ8_STRAW/22-269 PF00797

TVDVLRELQLHHLRTVPFENLSIHLGEEIVLDEKRLLDKVVGARRGGFCYELNGAFGALLESLGFDVTLLAARVYGEEGRLGVPYDHLALRVRTVDGGDWLADVGFGAHSHYPLAFGERGEQVDPGGTFRVIEAGVDAAGARSSGGSAASGDLDVLRDGKPQYRLETRPRVLGDFVAGLWWHSTSPKSHFTQSLVCSRVTEDGGRITLSGRRFTVTAADGRKEVSDLGTDEEVLGVYRERFGIELDQV

>Q6L725_STRKN/313-492 PF02737

AVGVAGSGTMASGIAEVFAKAGYDVVLAARSEEKAQLARARIGKSLARSVEKGRITAESAAETLERITPAGSYEAFADVDLALEAIAEDLDVKQQLFATLDKVCKPGAILATTTSSLPVVACARATSRPRDVIGMHFFNPAPAMKLVEVVRTVLTSDEVHATVREVCAAIRKHPVDCGDR

>MFPA_BRANA/314-493 PF02737

KVAIIGGGLMGSGIATALILSNYSVILKEVNEKFLEAGIGRVKANLQSRVKKGKMSKEKFEKTMSLLKGSLDYESFRDVDMVIEAVIENISLKQQIFADLEKYCPQHCILASNTSTIDLNKIGERTKSQDRIIGAHFFSPAHVMPLLEIVRTNHTSAQVIVDLLDVGKKIRKTPVVVGNC

>ODO2_RICPR/169-399 PF00198

EERTQRVRMSRLRKTIAQRLKDSQNTAAILTTFNEIDMSKVIALRNQYKEEFEKKHTVKLGFMSFFVKATIEALKLIPSINAEIDGDDLLYKNYYDIGVAVGTDQGLVVPVVRDADKMGFADVEQAIGDLAKKAREGKLSMSDLSGGTFSISNGGVYGSLLSTPIINPPQSGILGLHKTEERAVVIDGKIEIRPMMYIALSYDHRIIDGKEGVSFLVKIKNLIENPEKLLL

>Q92Y06_RHIME/13-76 PF03992

ITPGNEDAFETYAAELSVATRAEAGCLSYHLHRHLNQKGVYVFVEEWASRQVWEQHMSGEAIRA

>GRM7_RAT/594-855 PF00003

PVFLAMLGIIATIFVMATFIRYNDTPIVRASGRELSYVLLTGIFLCYIITFLMIAKPDVAVCSFRRVFLGLGMCISYAALLTKTNRIYRIFEQGKKSVTAPRLISPTSQLAITSSLISVQLLGVFIWFGVDPPNIIIDYDEHKTMNPEQARGVLKCDITDLQIICSLGYSILLMVTCTVYAIKTRGVPENFNEAKPIGFTMYTTCIVWLAFIPIFFGTAQSAEKLYIQTTTLTISMNLSASVALGMLYMPKVYIIIFHPELN

>O45279_CAEEL/97-322 PF00198

LRHHQDIPLSNIRATIAKRLTASKQQIPHEYQGVDVRIDDILALRQKLKKSGTAVSLNDFIIKAAALALRSVPTVNVRWTPEGIGLGSVDISVAVATPTGLITPIVENSDILGVLAISSKVKELSGLARESKLKPQQFQGGSFTISNLGMFGSVTNFTAIINPPQCAILTIGGTRSEVVSVDGQLETQKLMGVNLCFDGRAISEECAKRFLLHFSESLSDPELLIA

>Q84T13_EUGGR/24-211 PF02737

TVGVVGMGAMGHGIAQMTAAAGYKVVAVDIDANMLSKGIKAVEDSLSKVAAKAVKDGKADKATAEKNAADVRSRITTSGDIGALSSCDLVIESIIEDLNIKKKFFADLGKVAGANAILASNTSSFPITQLGEASGRTSNFLGLHFFNPVQMMKLVEVIKTKDTKEDVYKLGFAFSKSIGKEPVACGDT

>Q98PG1_MYCPU/82-314 PF00198

KLEARREKVTTIRKAIARAMKNSQDNVAYVSLVHEIDMTKLWDLRKSVVEKVKDLTGIKLTFLPFILKAIAIAIKDFQIFGAKYDEKTEELVYPDTVNLGVAVDTDHGLMVPVIKNAQSLNLVEFSQEIIRLANLARTKTIKPADMSGATFTITNYGSVGSLFGTPVINYPELAIAGVGAIVDKVYWKNGAAVPGKVMWITIAADHRWIDGATMGKFISKVKSLLEQPEILGV

>Q9XYS5_ASCSU/133-356 PF00198

PPRYTDIPLTNMRSVIARRLCESKQGIPHTYAIQKIDSDNVNKLRAKLKKEGISVSINDFIIKACACALRAVPELNVKWMKDHAEALPNVDISVAVATPAGLITPIVFKADTLGVSQIGAKVRELAKKARANKLTLEEFQGGTFTVSNLGMYGSISHFTAIINPPQAAIMAIGGGIDELETDLSSTNRFQVTLCFDGRAITVPDAHRFLEHFAMTFKEPDLMVA

>Q9HUN5_PSEAE/81-220 PF07696

AWLNGSLDLLEDPDGNLAVEDLEQAEQAGRFVAAAGRTSVGLSRSAWWLRLDLPRREAVSGGWWLEVASASLHDLRLYLPDERGGFREHRSGEAVPFAEGRDHAYRHPLFRIPPGDGPLRVYLRSYDPGGNAFPLRLWSH

>Q90XS7_ONCMY/389-481 PF00207

SWLWEVQPVRSGQITLNRNLPDSLTTWEIKAVGVFKKEGSSGICVADPIKVSVTQAVSVDVPLPYSMVRGEQIELRGSVYNQAEDNIKYCVTL

>FADJ_ECOL6/310-490 PF02737

SVGILGGGLMGGGIAYVTACKAGLPVRIKDINPQGINHALKYSWDQLEGKVRRRHLKASERDKQLALISGTTDYRGFAHRDLIIEAVFENLELKQQMVAEVEQNCAAHTIFASNTSSLPIADIAAHAARPEQVIGLHFFSPVEKMPLVEIIPHAGTSAQTIATTVKLAKKQGKTPIVVRDK

>OXAA2_LISMO/55-247 PF02096

GNYGIAIIITTLLIRALIMPLNLRTAKAQMGMQSKMAVAKPEIDEIQARLKRATSKEEQATIQKEMMAVYSKYNINPMQMGCLPLLIQMPILMAFYYAIRGSSEIASHTFLWFNLGSPDMVLAIIAGLVYLAQYFVSMIGYSPEQKKQMKIIGLMSPIMILFVSFTAPSALALYWAVGGLFLAGQTLLTKKLY

>A0LH72_SYNFM/230-689 PF01314

LSLIGANNQIVVPSSPQPWAEHYNPGSRWVASRGRTWGAANPPIETGSCEPQNLNRIAYRTNNAAFFLGEQAWRYTVRGNGCTGCPIRCHTMIKVPTVASKYGIPEIGQSTCVRMIFGKWFFKSFPDGPLGETSIEAAMVGMHLADDLGLWDNYGQLQRDFQKCYSEGIFQSKVGEKEFRRYSWDKFEKGDPAFLFELLPRIAGKDGELASALGLGTGFMLEGWGISEEAWKSDRTLQYWKLGHPKHHSNEDAGQCGVIINTQYNRDAQCHSHTNFTRNGLPLEIQKRLAADLWGSDAAVDAAGDFTPMNVYKAKMAKWALLRKELHDSISLCNWMGPWSSSPLKERGYKGDDTLESLLYSLVTGDRKSPRELDEVAERIFVLHRALTIRGMGTREMRAKHDTIPDWVYGDDKDRIPFTKGTIQMDKDDIRKAMQMFYEEAGWDKETGVPTGATYRRLGL

>Q9KG97_BACHD/180-411 PF00198

SSAEKRIPLKGIRKAIAEKMIKSVATIPHVTHVDEIEMDALKELREQLKHYSEQKGIKLTFLPFFIKAIVSALKEFEYFNASIDEETNEIVLKKDYHIGIATDTEKGLIVPVIQNADQKSLLELAGEITQLSTQARKGTLNVQQMTGSTFTISNVGPIGGLHATPIINYPEVAILALHKMEPRNVVREWESVIKLMMNMSLSFDHRLVDGATAVRFTNRMKELIENPNLLLM

>BMH1_YEAST/5-241 PF00244

REDSVYLAKLAEQAERYEEMVENMKTVASSGQELSVEERNLLSVAYKNVIGARRASWRIVSSIEQKEESKEKSEHQVELICSYRSKIETELTKISDDILSVLDSHLIPSATTGESKVFYYKMKGDYHRYLAEFSSGDAREKATNASLEAYKTASEIATTELPPTHPIRLGLALNFSVFYYEIQNSPDKACHLAKQAFDDAIAELDTLSEESYKDSTLIMQLLRDNLTLWTSDMSESG

>14333_SOLLC/9-246 PF00244

REENVYMAKLADRAESDEEMVEFMEKVSNSLGSEELTVEERNLLSVAYKNVIGARRASWRIISSIEQKEESRGNEEHVNSIREYRSKIENELSKICDGILKLLDSKLIPSATSGDSKVFYLKMKGDYHRYLAEFKTGAERKEAAESTLTAYKAAQDIASAELAPTHPIRLGLALNFSVFYYEILNSPDRACNLAKQAFDEAIAELDTLGEESYKDSTLIMQLLRDNLTLWTSDMQDDG

>Q8FRT3_COREF/22-196 PF02737

VVGVLGGGRMGAGIAHSFLAAGAHVTVVDINDAAVEAARERITNDIEGSIKRGAEGTVEQWLDRLTLSTDTAAFADHPVVVEAVPEIIDLKADSFRKIAAAAPGAVIATNTSSLSVSDLALSVDNPVIGLHYFNPVPASKLVEVVVADSTPEALVDLAREWVAGLGKTPIVVKDA

>A2XSN9_ORYSI/302-674 PF03081

MKKWVHGVKTVVRSLLTGERRICDQVLAVSDELRDECFVESTKGCIMQILNFGDAVAVCSRSPEKLSRILDMYEALAEVIPELKELFFGNSGNDVICDLEGVLERLGDAVKGTLLEFGKVLQQESSRRPMMAGEIHPMTRYVMNYLRLLVVYSDTLDKLLGDDSAGDVDHSDTHRGGDDEEEYLESLSPLGRHLVKLISYLEANLEEKSKLYEDGALQCIFSMNNILYIVQKVKDSELGRILGDHWIRRRRGKIRQNSKNYLRISWTKVLSFLKDDAHGGRSGSGSGSGNSSRIKEKFKNFNLAFDEIYRSQTLWKVPDPQLREELKISISENVIPAYRAFLGRYGSLVDSGRNSGRYIKYTPEDLENQLSDL

>Q9CLX7_PASMU/32-323 PF06472

LAKTTQWGRQFWLLAEDYFHPKHNLLPLFYFMIIVFFNLLSVRIDILVSNWYNALYKSLQDMNETVFWQQMVVFAVIASSSITNALLTYYLSKRFLIHWRMWLNNRMLNKWTENQAYYKTQYVEAQLDNPDQRIQQDVSSFVSTSLDFATGLISSIVSIIAFTIILWNLSGPMTIANIEIPHAMVFLVFIYVLFSSIVAFKIGRPLIQLNFANERLNANYRYSLIRLKEYAESIAFYRGEKMEKRLLTTQFNQVIDNVWQVIYRTLKLSGFNLIITQISVVFPLVIQVTRYF

>O35189_MOUSE/588-839 PF00003

LGCMALSFSAITILILVTFVKYKDTPTVKANNRILSYILLISLVFCFLCSLLFIGPPDQVTCIFQQTTFGVLFTVSVSTVLAKTITVVMAFKLTTPGRRMRGMMMTGAPKLVIPICTLIQLVLCGIWLVTSPPFIDRDIQSEHGKIVILCNKGSVIAFHVVLGYLGSLALGSFTLAFLARNLPDTFNEAKFLTFSMLVFCSVWITFLPVYHSTRGRVMVVVEVFSILASSAGLLMCIFVPKCYVILIRPDSN

>Q9ZRQ1_ARATH/230-460 PF00198

KDRERRVPMTRLRKRVATRLKDSQNTFALLTTFNEVDMTNLMKLRSQYKDAFLEKHGVKLGLMSGFIKAAVSALQHQPVVNAVIDGDDIIYRDYVDISIAVGTSKGLVVPVIRDADKMNFADIEKTINGLAKKATEGTISIDEMAGGSFTVSNGGVYGSLISTPIINPPQSAILGMHSIVQRPMVVGGSVVPRPMMYVALTYDHRLIDGREAVYFLRRIKDVVEDPQRLLL

>A0JMP8_DANRE/731-820 PF00207

TWIWELAEVGDSGSAEVPVTVPDTITTWETEAFCLSSTGLGLAPPAQLTVFQPFFLELSLPYSIIRGEIFELKATVFNYLSKCIMVKVSP

>Q2W349_MAGMM/216-615 PF01314

FIKATIEQKKVLADNAVTGQGLPKYGTQVLMNVINEIGAMPTRNFKEVQFEGAHKISAEAMHEPRATDGKANLATNGACFGCTIACGRISRMDPGHFSITSRPQYKEPSGGVEYEAAWAMGSDCGVDDLEACTFANFMCNEHGIDPISFGSTLAAAMEMFEMGVITKEQTGGVELKFGSAEALVKMAELTGKGEGFGLELGQGSRRLCAKYGHPELSMTVKSQEFPAYDPRGIQGMGLTYATSNRGACHLRSYTVASEVLGIPFKSDPLATDGKAALVKAFQDATAAFDASGICIFTTFAWSLENLAPQIDAACEGEWTPEILLEVGERIWTLERQFNLAAGMTAADDTLPKRLLKDAAKTGPAKGLTSGLEKMLPEYYQLRGWTTDGVPTTETLKRLQL

>14331_ENTHI/4-239 PF00244

REDCVYTAKLAEQSERYDEMVQCMKQVAEMEAELSIEERNLLSVAYKNVIGAKRASWRIISSLEQKEQAKGNDKHVEIIKGYRAKIEKELSTCCDDVLKVIQENLLPKASTSESKVFFKKMEGDYYRYFAEFTVDEKRKEVADKSLAAYTEATEISNAELAPTHPIRLGLALNFSVFYFEIMNDADKACQLAKQAFDDAIAKLDEVPENMYKDSTLIMQLLRDNLTLWTSDACDEE

>Q9SKF0_ARATH/1-361 PF04724

MTIRSLRKPPPVKLVFPTLIILFLTCLLCILTNFQTISYLFRPLWDKPPPPFKRIPHYYAENVSMGHLCELHGWTPRLEPRRVFDAIIFSNELDLLEVRWRELEPYVSKFVILESNTTFTGIPKPLFFDSNKERFAFAEGKIVHGVFPGKKRSTGQPYEDPFLLEGQQRVAMNWLLREAGVSDGDAVIMSDADEIPSPHTVKFLQWCDGIPDVMHLEMREYMYSFEFPVDYSSWRASVHIYSRKWTQYRHSRQTDLILSDAGWHCSFCFRRLNEFVFKMKGYSHADRVKRKEFLDYQRIQKHICKGYDLFDMLPEKYSFQELISKIGPIPPSASAVHLPAFLIQNAARFRFLLPGGCLREP

>Q828C1_STRAW/13-194 PF02737

RVGVVGCGQMGAGIAEVCARAGLDVKVAETTGEALEIGRTRLFNSLSKAAERGKISEEERDATQARLSFTTDLGEFADRDLVIEAVVENEQVKTEIFQVLDQVVTRPDAILASNTSSIPLVKLAVATSRPDQVIGIHFFNPAPVQKLVELIPALTTSEGTLSRAQGLVEKILGKHAIRAQDR

>Q8VWH7_ARATH/734-1015 PF06472

QTSQRALPSRVAAMLNVLIPTIFDKQGAQLLAVACLVVSRTLISDRIASLNGTTVKYVLEQDKAAFVRLIGLSVLQSGASSIIAPSLRHLTQRLALGWRIRLTQHLLRNYLRNNAFYKVFHMSGNSIDADQRLTRDLEKLTADLSGLLTGMVKPSVDILWFTWRMKLLTGQRGVAILYTYMLLGLGFLRRVAPDFGDLAGEEQQLEGKFRFMHERLNTHAESIAFFGGGAREKAMVDKKFRALLDHSLMLLRKKWLYGILDDFVTKQLPNNVTWGLSLLYAL

>Q8FXG6_BRUSU/180-469 PF00393

GHFVKTIHNGIEYADMQMIAEIYGILRDGLGLSAPAIGDVFEKWNEGPLNSYLIEITAKVLKSTDPETGKAMVDMILDEAGQKGTGRWAAIEAQILGVPATGIEAAVAARSLSSMKGEREEAAKAYKSGARTLDIANQTRFLSDLEQGLLAGKIAAYAQGFAVMEAASREHGWNIPLATTARIWRAGCIIRSQLLDDIAQAFEGNESRNLLMAPAFVPRMETSVKSLRQIVAQAALASLPLPALGSALSYFDSYTQALGTANLIQGQRDFFGSHGFKRIDKEGDFHGPWG

>Q9L4Z1_STAEP/200-431 PF00198

DFPETTEKIPAMRKAIAKAMVNSKHTAPHVTLMDEIDVQELWDHRKKFKEIAAEQGTKLTFLPYVVKALVSALKKYPALNTSFNEEAGEVVHKHYWNIGIAADTDKGLLVPVVKHADRKSIFEISDEINELAVKARDGKLTSEEMKGATCTISNIGSAGGQWFTPVINHPEVAILGIGRIAQKPIVKDGEIVAAPVLALSLSFDHRQIDGATGQNAMNHIKRLLNNPELLLM

>Q9AE39_RHILE/10-77 PF03992

VATGSEGDFETVWRNRDSSLPEVPGFVEFRLLRGKVNDEEGYTLYSSHTVWKSEADFQNWTKSMSFRA

>Q82MI0_STRAW/323-502 PF02737

KVAVLGAGMMGAGIAYSCARAGIEVVLKDVSAEAAAKGRGYSEKLCAKAVSRGRTTQEKADALLARITPTADPADLAGCDAVIEAVFEDTALKHKVFEEIEHIVEPDALLCSNTSTLPITALAEGVERQADFIGLHFFSPVDKMPLVEIIKGERTGDEALARAFDLVRQINKTPIVVNDS

>ACEK1_PSEHT/22-590 PF06315

LARAVFAGFEAMFATFLNITLGAQSRFEQRQYHEVQSAMRERLQVYERQVKSVSEAVKVIAYAELSCPQTWQLAKNIYGNMVKNHENEPIAHTFFNSTFGAIWDDKKIRTVHLFVLKAKYRTQPRPYDSLVKRISLQHGFNSAIKTLITNQVFRVPFSNLNQDVATLQATLTQGAKQQCRQVYELINLNDGYIEYAYSHFYRNKACYLIGRCIAKNGDNMPFAIAILNTPKGLKIDAVMMGADQLSLLFGFARTYFMVDTDQPARYVDYLSVLMPHKQRFELFNAIGFIKHAKTEFYRYKVDTTKNSPASFKYVAAPGTPGMVMLVFTIAGSDHVYKVIKDKFSAPKTATKAQVKEKYNFVKQADRVGRLVDTHEFRYLAFDLSRFSEQLLQQMKEHIGSSLIISGKALILKHVYVERKMTPLNLYINDCDSKALAQVMLDYGRAIKDLAGANIFPGDMLMKNFGVTRWGRVVFYDYDEICPLTDCNFREVPQTQNALEELSSDSYFDIEPNDIFPSQFKVFFSANELAFNAFNSHHSDLFNAQFWQTCQQQVQQGYLPDVYPYKQSWR

>CRYL1_BOVIN/11-193 PF02737

GVAVVGSGLIGRSWAMLFASAGFRVKLFDIEPRQVTDALVSLRKEMKMLELSGYLKGELGAEEQLSLISGCSDLREAVEGALHVQECVPENLELKRKLFAQLDKIADDHVILSSSSSCLLPSKLFAGLAHVKQCLVAHPVNPPYYVPLVELVPHPETAPATVDRTYALMRRVGQSPVRLLREI

>Q711M5_MUSMC/20-280 PF00797

DLATLTEVLQHQMRAVPFENLNMHCGEAMHLDLQDIFDHIVRKKRGGWCLQVNHLLYWALTKMGFETTMLGGYVYITPVSKYSSEMVHLLVQVTISDRKYIVDSAYGGSYQMWEPLELTSGKDQPQVPAIFLLTEENGTWYLDQIRREQYVPNEEFVNSDLLEKNKYRKIYSFTLEPRVIEDFEYVNSYLQTSPASVFVSTSFCSLQTSEGVHCLVGSTFTSRRFSYKDDVDLVEFKYVNEEEIEDVLKTAFGISLERKFV

>MDR1_HUMAN/70-348 PF00664

LVFGEMTDIFANAGNLEDLMSNITNRSDINDTGFFMNLEEDMTRYAYYYSGIGAGVLVAAYIQVSFWCLAAGRQIHKIRKQFFHAIMRQEIGWFDVHDVGELNTRLTDDVSKINEGIGDKIGMFFQSMATFFTGFIVGFTRGWKLTLVILAISPVLGLSAAVWAKILSSFTDKELLAYAKAGAVAEEVLAAIRTVIAFGGQKKELERYNKNLEEAKRIGIKKAITANISIGAAFLLIYASYALAFWYGTTLVLSGEYSIGQVLTVFFSVLIGAFSVGQA

>Q6GDA7_STAAR/21-259 PF00797

SIEALNYYATRFMLTVPFENIDVQNGKPISVDIDALFNKIVHDKRGGFCYELNTFFKAYLQQKGFNPELMSATIHTPGGGRSQNGSHASLVVSINNVFYVTDVGFGDLPLNAIPITLPDDTQQITDISGTFRAIFNSEDKDIFYVQKYENNHWHTKYEAEFKAREIEEFDQNIEYNQTHPDSVFVKHLLITMPQSFGRATMFENHLTLTKDGTPEKLAITKDNYKQFLTKYFGLNVTIS

>Q7QEK0_ANOGA/123-370 PF03417

VNEPDCVVLGHTEDALSEVLNHFYFVSAHIISESPEGKHKVVEERFTSLCYAGHLPGYTMNANHHGLVFSINTLSAKTLVGGKTPRHFITRALLAAENFVQAQEILRDHGVGAADGCSINMTFLRQEGDRLFHNAEMGPATNGDESQLNILTASPGEHIIHVNSYQRLPVPEVSGLIIESSIERMKTFAKYAPPKTVADVVRMLSDVTASEHKVFRDHGKNQKVKTICVGIIDCIKRTWTLYAENPAY

>Q82J95_STRAW/4-184 PF02737

KLAVIGAGLMGSGIAQVSAQAGWDVVLRDVTDEALTRGTDGIKASYDKFVSKGRLSADDAGAALARITTTTDLDAVADADIVVEAVFEKLEVKHEIFRALDKIVRDETVLASNTSAIPITKIAAVTERPERVVGAHFFSPVPMMQLCELVRGYKTSDEALATTREFAESVGKTCIVVNRDV

>Q9RYX3_DEIRA/36-123 PF07694

DSRLQVPRFLLVSLMCVPLMFFPAQVAPGVFVDLRAVPIAFLTLRLGWGWGLVGAVPLLVYRYLLGGVGWPPAMVSAIGVVLVAGLFH

>Q8IZJ3_HUMAN/750-841 PF00207

TWIWHCLNISDPSGEGTLSVKVPDSITSWVGEAVALSTSQGLGIAEPSLLKTFKPFFVDFMLPALIIRGEQVKIPLSVYNYMGTCAEVYMKL

>Q67MQ7_SYMTH/215-665 PF01314

FRDLQKDWVQELRSHPITGRQLPRLGTAGLLSGMQAHRMLATRNFSRGRFDQFDAVSGETLAEEHLVRNGGCTGCPVHCGREVILPEKAGNGAPEAGDAAATGWGPEVTPAAANGRASGGGRKGRRVKGPELETIVLLGPNLENADLAEIIRLNHQLDELGMDSISTGGTIAFAMELNEKGLWENGLRFGRVENLSQLLDDIAHRRGIGDLLAEGSRRLAERFGGKGFAMQVKGMELPAYEPRAAVGQGLGYAVSNRGGCHLNAGYLAVLEGLGLEMDPYTPRAKAALTILMQDLMEAISAAGNCLFSSYDVFPAALLNRPNGWLTRAVNRLLPWAGGPVALLLRLRWMPVHLPGLPLTRALEAVTGMKMDLGRLKLIGERGYNLERLVNTRLGMTAADDDLPERLKAVEQVPGDPRTKVPLEPMKRQYYRLRGWTDAGVPTPGLLRRLGI

>Q9C9A1_ARATH/49-274 PF02485

FAYYITGGRGDNDRISRLLLAVYHPRNRYLIHLGAEATDAERLALLSDLKSVPAVNAFGNVDVLGKVDRLSENGASKIASTLHAVSILLKLDPTWNWFIELSALDYPLITQDDLSHVFASVNRSLNFIDHTSDLAWKESQRIKPIVVDPALYLARRTQLFTATEKRPTPDAFKVFTGSPWIVLSRPFLEYCIFGWDNLPRILLMYFNNVILSEECYFHTVICNAPE

>A0Z1J2_9GAMM/7-569 PF06315

LARTILNGFTAYFAEFENITLAARTRFELAEWGIMQEISTRRIDLYKEKVLETLAYVNVIADDRISDFVFWTETRAVYVELVRGMTNFEIAETFYNSIYNSVFGHHRIRNEHAFVFSPQGDMPPVDVRRVVKRFSGDLHDSVATLLSQYAMHIPYENIERDSASIVAAIENQLGEVDGASDSAVELHTLEHHFYRNKAVYIVGRIVAEGLQIPFVLPMLHNGDAVAPAVYIDTILFGSDRVSLLFSFTRSYFMVDASIPSQYVLFLHQLMPKKDISEIYSAIGHHRHGKTYYYRTAYRHMRATTDQFIVAPGIKGMVMTVFTLPSYEFVFKIIKDRFTPPKDVTHQLVKDKYNLVKRWDRAGRMADTQEFNNLVFDRSRFSEELMAELYEACPSQLVISGTALIIKHCYVERRMTPLNLYLQNASDEDVDEVMNDYGKSIRELAAANIFPGDMLLKNFGVTRHGRVVFYDYDEIQPLLECNFRRIPPPRDEYEEMASRPWYSVGPNDVFPEEFRLFFSGNQRARKAFDARHSEIYDADFWTGLQDRIREGHVDDFYPYPRVMR

>OXAA_DEIRA/211-394 PF02096

GNWGLVIMALTILLRLVMWPLMQAQGRTTARMQLVQPLQKEIQEKYKGKTDPESQRAMQMEMAQLMRDYQVNPAGCFSTLLPFPVLIALWATIRNFEFDSGFLWLPDLATPDPFYILAVIYLFVNLGQLYVSTRKTPEMFRQQAMIYLVFLYFALTFPSGVTLYIILSTIIGIVQQIIINKQVE

>Q9X6X2_MYXXA/180-413 PF00198

GAADERVPLRGLRKKIAEKMVRSKFTAPHFAFVEEVDATELVALRARLNAQLAAAGENIKLNYLPFIIKATVAALKKFPHLNANFDEASQELVVRGEFNIGMAAATPDGLTVAVVKSADRLTLAELARETARLGAAARDRKLKMEELTGGTFTISSLGQSGGLFATPIINHPEVGILGVHRLKKRPAVVGDQVVVRDMMNLSLSCDHRVIDGSVAADFTYEIIKYLEKPDLLFL

>O69856_STRCO/340-521 PF02737

KVGVVGAGLMASQLALLFLRRLEVPVVLTDIDQERVDKGVGYVHAEIDKLLGKGRVNQDKANRLKALVTGVLDKAEGFADADFVIEAVFEEMGVKQKVFAEVEAVAPAHAILATNTSSLSVSEMASKLKHPERVVGFHFFNPVAILPLLEIVRGEQTDEAALATAFGVAKKLKKTAVLVKDA

>1433F_RAT/4-241 PF00244

REQLLQRARLAEQAERYDDMASAMKAVTELNEPLSNEDRNLLSVAYKNVVGARRSSWRVISSIEQKTMADGNEKKLEKVKAYREKIEKELETVCNDVLALLDKFLIKNCNDFQYESKVFYLKMKGDYYRYLAEVASGEKKNSVVEASEAAYKEAFEISKEHMQPTHPIRLGLALNFSVFYYEIQNAPEQACLLAKQAFDDAIAELDTLNEDSYKDSTLIMQLLRDNLTLWTSDQQDEE

>Q2NKT0_BOVIN/1-82 PF06446

MALNTQIRATCLLLLVLLSLTSGSVLPPQTRQLTDLQTKDTAGAAAGLTPVLQRRRRDTHFPICIFCCGCCRKGTCGMCCRT

>Q9HX42_PSEAE/28-172 PF07696

LPLGQSIDVFEDVRGSADINDITSRAIDSSFRRHDKDVLNAGYSRSVFWLRLDLDYRPVASSDPRTWLLELAYPPLDKLDLYLPDGQGGYRLAQRTGDTLPFASRPIRQNNYLFELGLEPNKPQRVYLRLESQGSIQAPLTLWSP

>Q7T170_DANRE/20-265 PF00797

TLETLRYLHLNHLLTVPFENLTIHTRGRVRLELPLLYEKIVVNHRGGFCFEINGLFSWLLSQMGYDVTLLSAQIRSRFTGAYGPPFDHLFLMVKVDEHRWLCDVGFGSGFQLPLSLETDSPQIQSHGVYRLRSEGNLIFMESKSEIGGECWTEQYKFTLEPRDRADFRAMCDYHQSSVSSLMFCKSLCSLLLPTGRITIMGRRLIISSLGSGDGEHASRTITDLSDEEITELLREKFGIVLQAPLI

>GRM1_CAEEL/685-934 PF00003

PTILAVFGIIATLFVIVVYVIYNETPVVKASGRELSYILLISMIMCYCMTFVLLSKPSAIVCAIKRTGIGFAFSCLYSAMFVKTNRIFRIFSTRSAQRPRFISPISQVVMTAMLAGVQLIGSLIWLSVVPPGWRHHYPTRDQVVLTCNVPDHHFLYSLAYDGFLIVLCTTYAVKTRKVPENFNETKFIGFSMYTTCVVWLSWIFFFFGTGSDFQIQTSSLCISISMSANVALACIFSPKLWIILFEKHKN

>MUG4_MOUSE/738-828 PF00207

TWVWDIVTVSSTGVAEVEMTVPDTITEWKAGALCLSNDTGLGLSSVVPLQAFQPFFVEVSLPYSVVRGEAFMLKATVMNYLPTSMRMSVQL

>Q62591_RATSO/49-139 PF00207

TWIWDLVTVNSSGVTEVEMTVPDTITEWKAGALCLSNDTGLGLSSVATLQAFQPFFVELTMPYSVIREEAFMLKATVMNYRPTSLRTGVQL

>Q17505_CAEEL/548-799 PF00002

QTLLTLLTYVGCIISIICLLLTFFAYLIFSRNGGDRVFIHENLCLSLAIAEITFLAGITRTEDSLQCGIIAVALMYMFLSALTWMLLEGYHIHRMLTEVFPSDPRRFTYLLVGYIPPAIITLVAYLYNSDGFGTPDHCWLSTQNNFIWFFAGPACFIFCANSLVLVKTLCTVYQHTSGGYLPCRHDVDSGRSIRNWVKGSLALASLLGVTWIFGLFWVEDSRSIVMAYVFTISNSLQGLFIFLFHVVFAEKM

>ABCD2_MOUSE/78-365 PF06472

PGLNAAFFKQLLELRKILFPKLVTTETGWLCLHSVALISRTFLSIYVAGLDGKIVKSIVEKKPRTFIIKLIKWLMIAIPATFVNSAIRYLECKLALAFRTRLVDHAYETYFANQTYYKVINMDGRLANPDQSLTEDIMMFSQSVAHLYSNLTKPILDVILTSYTLIRTATSRGASPIGPTLLAGLVVYATAKVLKACSPKFGSLVAEEAHRKGYLRYVHSRIIANVEEIAFYRGHKVEMKQLQKCYKALAYQMNLILSKRLWYIMIEQFLMKYVWSSCGLIMVAIPII

>HCDH2_CAEEL/24-209 PF02737

NVTIIGAGLMGSGIAQVSANAKLNVTVVDSNQSALEKAQQGIANSLKRVAKKKHADDAAAQTALVSSVLDRIKMSTNVSDSVKDADLVIEAIVENIDIKRKLFAEVEVAAKPTTLITTNTSSLRLADIGLNLKDKSRFGGLHFFNPVPMMKLLEVVRHTETSDATFNQLVDYGKTVGKTTVACKDT

>GPR64_HUMAN/625-886 PF00002

MMALTFITYIGCGLSSIFLSVTLVTYIAFEKIRRDYPSKILIQLCAALLLLNLVFLLDSWIALYKMQGLCISVAVFLHYFLLVSFTWMGLEAFHMYLALVKVFNTYIRKYILKFCIVGWGVPAVVVTIILTISPDNYGLGSYGKFPNGSPDDFCWINNNAVFYITVVGYFCVIFLLNVSMFIVVLVQLCRIKKKKQLGAQRKTSIQDLRSIAGLTFLLGITWGFAFFAWGPVNVTFMYLFAIFNTLQGFFIFIFYCVAKENV

>Q9LNN5_ARATH/64-289 PF02485

FAYLVSGSKGDLESLWRTLRALYHPRNQYIVHLDLESPVDERSELASRISNDPMYSKAGNVYMITKANLVTYTGPTMVANTLHACAILLKRTPDWDWFINLSASDYPLVTQDDLIHTFSTLDRNLNFIEHTSSLGWKYKKRAMPLIIDPGLYMLNKSNVLLVRPNRSLPSAFKLFTGSAWMALSHAFVEYIIYGWDNLPRTLLMYYTNFVSSPEGYFHTVICNVPE

>Y3718_BACHK/2-69 PF07288

IFKVFYQEKMTEVPVRENTKVLYLEATSEKDVRTKLNKFAYNIEFVQSVTGNHLEYEKANADLTLAEI

>Q39UP3_GEOMG/239-638 PF01314

VMTILSGIGSPQEMLHTDEKWHTENFAWGNARTRRRDFWTEEIDESWSKSQHGAVKRLISCYNCPTHCGALISHKDTPRYMAKCFGKLTYAMAAYVDDLDFSWKILQRATEYGVDSFSTPQILAFAVELYEAGILTDKDFKGCPSDKEGRFFWLLDRVARREGIGDILADGVYFAARKIGNGAEAFDHNTIKKHEQLPVKLGTLDPLYFLMYSTNEKISITQMEGQWPQSAFPTMEQREEFVRDWPQIPDEKFKQIVLDWELRGEKSIPYFPTPDMCSEIVDWMEMMHNIDDAVGMCCGMSSFCLKPPYHIHNYPKIISAATGLDLDESGLKQIANRSRNLHRAYNNRLGIRRVDEKPPADHWKKRFPELEEQLLSTYYTYKGWNHDGIPTREKLEELDL

>Q8CAU4_MOUSE/1-127 PF06214

MDPKGSLSWRILLFLSLAFELSYGTGGGVMDCPVILQKLGQDTWLPLTNEHQINKSVNKSVRILVTMATSPGSKSNKKIVSFDLSKGSYPDHLEDGYHFQSKNLSLKILGNRRESEGWYLVSVEENV

>Q96XV4_SULTO/5-183 PF02737

KVAVIGAGVMGHGIAEVFSLYGNEVYLYDKYPDALEKGLKNILWSLNKLKEKGKITDTERVFSRIKPVNDLSQISDAELVIEAVSENLDLKSSVFKQVSKIVSKDSIIATNTSSLPISELAQSVENPHRFLGLHFFNPPVLMKLVEVVKGVKTDDLIFSKGIEIIKSIEKVPIPVRKDV

>Q5AEJ2_CANAL/10-465 PF07247

KPEFNERYYICRTIENYSTNFSIIVQYNRQISHNLLSNALYSLIKKNSWFVQNFFQIDQRNPATANGHNFEVRILEHVKFNDVVKFHQIDKFDEIIMESLNDHIFSMNNATLPLWKINVFEEMRPNGNQFISVSFDHSNYDGLSGVQFQKDLAKELLTAKDDLFYDVLFDYQRDFGNLPAKILPAVDNLTDLFDLGVLSSSNSILKKWVPFYDTICGFIWPSDPPIFDTDTPVTKNLQTKYKLLKLTSNQIGQISKYCRSHGITLTTYFDIICICALQETVFSVVKSSATHTSSLVAINGRRYYSDEIKNFLYGTLVCGAPIILPTIENKLEAMQIFHQEMTNDINTKKSFQSTGNLLKHANVWEYFQNKINKIGGRFTLTISNLGKISNSNDIFKFEQMYFVSNTGVVYNFVLNITTLPNGELTAVVGYIPEFEKYELNNKPIMNTFMEKFYDLL

>Q9SIB0_ARATH/209-553 PF03081

IRKWLRTTTRAVNTLFSGEQILSDHVFSSSSSTIRESAFAEITSQTALALFTFPEKMAKCRKSPEKIFLTLDVYQTIVDLLPKINELFSSDSTSTVRSQVDLTLVNLREGVVSMIDEFESSISKESSKSLISGGGIHQLTRYVMNFIVFLADYSDTLSDIISKPSLPSPEEEKDSGDSSPVKSRISRLILFLLCKIDAKSRLYNDVALSYLFLINNVNYVVVKVRSSNLKTVLSEDWVKKHEAKVKKYVAKFEEIVWGEMMTSLSDDVTMTAEEGIKRFSDGFEEAYKRQTGWIVPDSKLRDEIKRSVGMMIIPRYSGFCERNRVRLLENVGFDPEDIGNYLSDL

>Q8G825_BIFLO/10-192 PF02737

TIANVGTGTMGHAITLQFALAGYPVHLVGRSEASLEKAMKAIRSDAEDFAEAGLLKAGDTVDTVLARITGYADYASGVADVDFVIESVAENLDVKKSVWTEVEHAAPKDAILSTNTSGLSPTALQSVMGHPERFVVAHFWNPAQLMPLVEVVPGEKTDPKVVDITFDLMAKIGKKPAKIKKES

>Q8YJI4_BRUME/5-184 PF02737

TVGIIGAGQMGSGIAHVCALAGYNVLLHDAAPDRLEKGIATINGNMARQVASGKLQEEQRADAMKLIRPANSMEDLAGVDLAIEAATEDETIKRKIFAQLCPVLNPEAILATNTSSISITRLASTTDRPERFIGIHFMNPVPVMKLVELVRGIATEEDTFRKSNDFVTALGKTVTVAEDF

>PDXJ_CAUCR/3-245 PF03740

LRLGVNIDHVATIRNARGASYPEPVRAAELALIAGADGITAHLREDRRHISDADIAVLTDLCHKRGKPLNFEMAVTDEMVGIALNARPHAACLVPERREEVTTEGGLDVIKGQKRIADATARLRTVGARVSLFIEPDPDQIRACVTAGAQVVELHTGAYCDAARAGETARAEAILKRLKAGAALAHELGLEVHAGHGIDYATVKPVAAIPQIAELNIGHFLIGEAIFVGLPEAIHRMRALMEA

>Q9LU48_ARATH/29-257 PF03767

IESRHKKAAEEDVNLHCTTWRFAAEMNNLAPWKTIPVECADYVKDYVMGKGYLTDLERVSEEALIFARSIEFSGDGKDIWIFDIDETLLSNLPYYIDHGFGLELFDHSEFDKWVERGVAPAIAPSLKLYQRVLDLGYKVFLLTGRKESHRLVTVENLINAGFQNWDKLILRSPEEQHKMATLYKSEKRDEMVKEGYRIRGNSGDQWSDLLGTSMSQRSFKLANPMYYIP

>Q4LEN8_PAROL/2-89 PF06446

KAFSIAVAVTLALAFVCIQDSSAVPFQGVQELEEAGGNDTPVAAHQMMSMESWMESPVRQKRHISHISMCRWCCNCCKAKGCGPCCKF

>ECHP_CAVPO/301-479 PF02737

SVGVLGLGTMGRGIAISFARVGIPVIAVESDPKQLETAQKLITSILEKEASKSRQQCGQQRSGPKPRFSSSMKDLASVDLVVEAVFEDMNLKKRVFAELSAVCKPEAFLCTNTSALDVDEIATSTNRPQQVIGTHFFSPAHVMKLLEVIPSRHSSPTTIATVMDLAKKIKKVAVVVGNC

>Q98D42_RHILO/25-263 PF00797

SLDTLKALHFAHPQAIPFENIDPFLGRPVRLDLAALQDKIVLGGRGGYCFEHNLLFMHALKALGFEVGGLAARVLWGQSEDAITARSHMLLRVELDGRTYIADVGFGGLTLTAPLLLEPGREQKTPHEPFRIVEADDHFRLQAAIGGDWRSLYRFDLQPQYEVDYSVTNYFLSTSPTSHFLSSVIAARAAPDRRYALRGNRLSIHHLGGRTEQTEIATAADLADTLQGLLGIIIPDRTA

>PAAH_ECOLI/7-186 PF02737

TVAVIGSGTMGAGIAEVAASHGHQVLLYDISAEALTRAIDGIHARLNSRVTRGKLTAETCERTLKRLIPVTDIHALAAADLVIEAASERLEVKKALFAQLAEVCPPQTLLTTNTSSISITAIAAEIKNPERVAGLHFFNPAPVMKLVEVVSGLATAAEVVEQLCELTLSWGKQPVRCHST

>O32959_MYCLE/290-530 PF00198

HLRGTTQKVSRIRQITAKKTRESLQATAQLTQTHEVDMAKIVGLRAKAKAAFAEREGVNLTFLPFIAKAAIDALKIHPNINASYNEDTKEITYYDAEHLGFAIDTDKGLLSPVIHYAGDLSLAGLARAIVDIAARARSGNLKPEELSGGTFTITNIGSQGALFDTPILVPPQAAMLGIGAIVKRPRVVIDASGNESIGVRAICYLPLTYDHRLIDGADAGRFLTTIKHRLEEGAFEADLGL

>Q6NBL0_RHOPA/329-508 PF02737

KLAIIGAGFMGASVGYVSAKAGIEVVLIDRDQESADKGKAHCQSVIDGLIKKGRAKEADRDALMSRITATPDFNAISDCDLVIEAVFEDRKVKAETYAKAQPLLKEGAIFASNTSTLPINSLAEEFKDQSKFIGIHFFSPVEKMMLVEVILGNNTSDVTLATALDYTRQIGKTPIVVNDS

>Q97HG6_CLOAB/546-624 PF06725

RVIAVDPSVIKLGTRVYLQFPDNKRYQTKNGQRYDLNGWYTAHDTGGAIKGNHIDLFMGFGGAEDTARCDNFGTVNIKV

>Q8YAL6_LISMO/10-73 PF03992

VKKEQTEAFLQAAKEVIAASRAEAGNHGYELVQSTENETVFYMLEKWADMDAIQQHNDSEHFKK

>14332_ENTHI/4-238 PF00244

REDLVYLSKLAEQSERYEEMVQYMKQVAEMGTELSVEERNLISVAYKNVVGSRRASWRIISSLEQKEQAKGNTQRVELIKTYRAKIEQELSQKCDDVLKIITEFLLKNSTSIESKVFFKKMEGDYYRYYAEFTVDEKRKEVADKSLAAYQEATDTAASLVPTHPIRLGLALNFSVFYYQIMNDADKACQLAKEAFDEAIQKLDEVPEESYKESTLIMQLLRDNLTLWTSDMGDDE

>Y2424_BACSK/2-69 PF07288

IFKVYYQEDAAQMPVRERTKSLYIEGESEADVRLKLAKQNFNIEYVTAVTGAYLEYEQANSDFKVVNI

>CELR3_RAT/2534-2777 PF00002

LELLAVFTHVVVAASVTALVLTAAVLLSLRSLKSNVRGIHANVAAALGVAELLFLLGIHRTHNQLLCTVVAILLHYFFLSTFAWLLVQGLHLYRMQVEPRNVDRGAMRFYHALGWGVPAVLLGLAVGLDPEGYGNPDFCWISIHEPLIWSFAGPIVLVIVMNGIMFLLAARTSCSTGQREAKKTSVLRTLRSSFLLLLLVSASWLFGLLAVNHSVLAFHYLHAGLCGLQGLAVLLLFCVLNADA

>GLR_HUMAN/138-407 PF00002

YSSFQVMYTVGYSLSLGALLLALAILGGLSKLHCTRNAIHANLFASFVLKASSVLVIDGLLRTRYSQKIGDDLSVSTWLSDGAVAGCRVAAVFMQYGIVANYCWLLVEGLYLHNLLGLATLPERSFFSLYLGIGWGAPMLFVVPWAVVKCLFENVQCWTSNDNMGFWWILRFPVFLAILINFFIFVRIVQLLVAKLRARQMHHTDYKFRLAKSTLTLIPLLGVHEVVFAFVTDEHAQGTLRSAKLFFDLFLSSFQGLLVAVLYCFLNKEV

>OXAA_LEPIN/420-618 PF02096

PNYGWSIIIFAILFKLVFYPLNQKQADSMKKMQELSPQLKTINEKFANDPKMRQQKTMELYKKNNVNPVGGCLPMVIQIPIFIALYTAFSDTIDLWNSPFLWVKDLSEPDVIWTSPAIPYFTQTGIGLNLLALLMVGTQVFQTRMTSVSMDPNQKMLMYVMPVMMLYIFWNMPSGVTLYWTFQNVLSIGQQWITNHLKK

>Q9HIA5_THEAC/179-398 PF00198

PGREEILEMHGLRRIIFDKMTKAKQIMPHFTVMEEVDVTSMVSILDSAKARNRKVTVTGFLARIVPSILKQYPYLNAIYDETRRVYILKKYYNIGIAVDTPDGLNVFVIKDADRKSMVEISAEISDKASRARENKLQLDEVQDSTFTITNVGTIGGIMSTPIINYPEVAILGVHRILEREGRKYMYLSLSCDHRLIDGAVATRFIVDLKKVIEDPNAIIY

>Q53926_STRCO/3-173 PF02737

TVTVIGAGTIGLGWINLFSARGLTVRVNSRRPDVRRVVHEALELFSPGRVDELAARIEYEPDVGRAVAGADVVSENAPDDLPLKQRLFAEIGAAAPDHALVLSSTSKLLPDELSRDMPGPGRLVVAHPFNPPHIVPLVEVVRGERTDPEAVERTLAFLASVGRTPVVVRRA

>O45730_CAEEL/67-356 PF06472

KAFDPQFLKQLKELLKIMIPGVFSKEAGIIGMHSIILICRTFLTIFVAQLEGSMVQAIVEKNVLQFVLHLVKWILVALPATFVNSMIRFFESYLGLAFRTRLTKHAYKQYFSDQTYYAVSNLDTRLQNADQCLTEDITMFSQSVAHLYSHLTKPVLDVALITFTLIKLAIQRGTGRSTFLPSCMAIMAVSLTAKILRAVSPRFGHMVAEEARRKGHLRYLHSRIITNSEEIAFYGGHQAEYKQLDGAFNSLYQQMMLIFKKRIPYIMIEQFLMKYVWSGTGMVMIALPIL

>P93712_PHAVU/28-255 PF03767

SMTTGYGHGASDTEVRCASWRLAVEAQNIFGFETIPQQCVDATANYIEGGQYRSDSKTVNQQIYFFARDRHVHENDVILFNIDGTALSNIPYYSQHGYGSEKFDSERYDEEFVNKGEAPALPETLKNYNKLVSLGYKIIFLSGRLKDKRAVTEANLKKAGYNTWEKLILKDPSNSAENVVEYKTAERAKLVQEGYRIVGNIGDQWNDLKGENRAIRSFKLPNPMYYTK

>Q9DDV9_ONCMY/794-889 PF00207

SWLWKEENLPQCLKDEECVIQKSIDLPDSITSWQITVIGLSKTHGICVSEPLTLIVRKNFFLDLKLPYSAVRNEQLEIKAILHNYIDDTITVRVEL

>OXAA_STRCO/38-259 PF02096

WGLSIVSLVILIRICLIPLFVKQIKATRGMQTLQPEMKKIQERYKNDKQRQSEEMMKLYKETGTNPLSSCLPILAQSPFFFALYHVLNGIASGDTIGKINQPLLESAQKAHIFGAPLAAKFTDGASKVESLGASLTDVRVITAVMIVLMSASQFFTQRQLMTKNVDTSVKTPFMQQQKMLMYVFPVMFAVFGINFPVGVLVYWLTTNVWTMGQQMYVIRNNP

>Q804B4_NOTVI/91-189 PF00207

SWFWKVEQLVEPPDANGISSKTLQMFLKDSITTWEVLAVSLSPGKGICVADPYEITVFKKYFIDLRLPYSVVRNEQVEIRAVLFNYEEDQDEIKVRVEL

>ODP2_RICPR/178-408 PF00198

TEEYRSVPNNNIRKIIAKRLLESKQTVPHFYLSIECNVDKLLDVREDINKSFSEDKVTKISVNDFIILAVAKALQEVPNANASWSEDAIRYYNNVDISVAVAIENGIVTPIVKDANKKNIIELSREMKTLIKKAKDNKLTPIEFQGGGFTISNLGMYGIKNFNAIINTPQSCIMGVGASTKRAIVKNDQIIIATIMDVTLSADHRVIDGAVSAEFLASFKRFIENPVLMLI

>Q9L8D9_AZOVI/5-184 PF02737

CIAILGSGSMGVGIATHLARHGHEVLLIYPSMEQLAEVLAMARSILAGLVEAGRFAPEQVAATLARLRTSTRLKDVAGVRLLIETLPERIELKRALYAELERIVDAEAVIASDTGGLSPERLAEGMRHPGRLLIAHFRSPPHRVPLVAVVAGRQTRSEHLAYVRTLLAGTNLEVVVVPDG

>Q6BRP8_DEBHA/9-477 PF07247

KLGFLEKYYVCRNVEWYKEDICVTAQYNQHIDEVLLSNALRSLILKNTVFALNVYRDGDPQYDAKMNGDNFTVRPVEKIAFSDVVSFKDIDELFGVVELNALSEEVSPVNDTRPLWRLKVYKSSSSKKQYVTFHCDHTFFDGLSAAQFHKDLIVEFGIYSGDAALKFQETLFDYTEDKSKLPDILDSPDKIVDLYNPGILFSLLAIVQSMLLPKWLADGFWSLFSTNAEQKPLYTHLPVQKGAKSNQRHVNIKPDDVKRLRAFCRSQSTTLTPYITAIAMYALQETVFPVVSNSQCSFQVDIPIDGRRYFPDKANDTKYGIYVGAFSCKMGPVSKSTGSYDDLKVPINTLTTQLSSSMADNSPFRYFGLLKYVNCWSFLASKIGQTDSRLTLEVSNLGLHLISHDNWSIENLWFNQTNGILNHFCLSLIATESNGLNLLTSYLADLDMVQTSLRKLVIDEFMALFTTKL

>Q4RUJ7_TETNG/680-771 PF00207

TWVWHCLNVSSETGQAELRLDVPDSITTWVTEAVGLSEEKGLGLARRAELRTFKPFFVDFTLPYSLIRGEQTKVPLTVYNYLPTCAEVHVKV

>Q7W811_BORPA/8-191 PF02737

QAAVLGAGVMGAQIAAHLANAGVPVMLYDLTAPQGERNAVVHKALAGLRKLEPAPLARPDRLALITPAHYDDDLERLGGCDLVIEAIAERLDWKTDLYHRIAAHVAPHAIVASNTSGLSIETLAGALPEALRPRFCGIHFFNPPRYMRLVEIIATRHTRPEVVDQLEGWLTSRLGKGVIRALDT

>Q9UX37_SULSO/10-191 PF02737

KVAVIGAGVIGVGWTTLLLAKGYKVNLYTEKKETLEKALAKVSAYLVNLKNLGMINEEPESYITNLTGITKIDDAIHNVDFVIEAIIEDYTAKKNLFKLLDTQLPQDIIIASSTSGLLMTEIQKAMIRHPERGVIAHPWNPPHLLPLVEIVPGEKTSKETVDLTREFMEKLDRVVVLLRKEV

>Q8XYL6_RALSO/104-332 PF03417

LPGKGRRQIAHNEDGDPGFAGHCALAQCRIDGSPGFAAFVYPGSLPGHTFAVTDAGLAMTVNNLRLREAVAGVPRMVLTRAVLDAASLDTAVAVLRESPRAGGFHLTLGHCAHPALLSVEFGAHGCSVREIAEPALHANHATHPPMRDAPQIVTDSSRHRQARGDALLAQARAAGQPIDPLAILADQEDASLPIYRADPHDPDDENTLATADIAILPAHIEWAVYERPG

>ABCD3_RAT/57-338 PF06472

AVVDKVFLSRLSQILKIMVPRTFCKETGYLILIAVMLVSRTYCDVWMIQNGTLIESGIIGRSSKDFKRYLFNFIAAMPLISLVNNFLKYGLNELKLCFRVRLTRYLYEEYLQAFTYYKMGNLDNRIANPDQLLTQDVEKFCNSVVDLYSNLSKPFLDIVLYIFKLTSAIGAQGPASMMAYLLVSGLFLTRLRRPIGKMTIMEQKYEGEYRFVNSRLITNSEEIAFYNGNKREKQTIHSVFRKLVEHLHNFIFFRFSMGFIDSIIAKYIATVVGYLVVSRPFL

>Q6XBS9_SACPS/47-542 PF07247

RMGSVEDLYAALNRQKLYRNFSTYSELNDYCTKDQLALALRNICLKNPTLLHIVLPARWPDHKKYYLSSEYYSQPRPKHDYISVLPELKLDGVILNEQPEHNALMKQILEEFANSNGSYTAKIFKLTTALTIPYTGPTSPTWRLICLPEEDDTNKWKKFIFVSNHCMCDGRSSIHFFQDLRDELNNIKTLPKKLDYIFEYEKDYQLLRKLPEPIENMIDFRPPYLFIPKSLLSGFIYSHLRFSSKGVCTRMDEIEKSDEIVTEIINISPSEFQKIRTKIKLNIPGKCTITPFLEVCWFVTLHKWGKFFKPLKFEWLTDVFIPADCRSLLPEDEEVRAMYRYGANVGFVDFTPWISKFNMNDSKENFWPLIAHYHEVISGAIKDKKHLNGLGFNIQSLVQKYVNIDKVMRDRALGKSRGGTLLSNVGMFHQSEETEHKYRIRDLAFGQFQGSWHQAFSLGVSSTNVKGMNILISSTKNVVGSQELLEELCAMYKALL

>O08351_FLAME/1-234 PF03767

MKKILLTGGLILSFISCSAQKADHDTKDLVNATAWMQNAGEYKALTIQAYQLAQIRLAQILTQEVSEKPRAIVLDIDETVLDNSPYQAYQIENKKNFNQEDWSKWTRLAQAEPIAGALNFLNFTKNNGVEIFYVSNRSEAERVPTLENLQKKNFPYADNDHLILKTDKSSKESRRQKLSEKYNIVLFFGDNLSDFSDMYYYNNEGKTSSEKVLEHPELFGSKFIILPNAMYGDW

>1433B_VICFA/7-242 PF00244

RENFVYIAKLAEQAERYEEMVDSMKNVANLDVELTIEERNLLSVGYKNVIGARRASWRILSSIEQKEESKGNDVNAKRIKEYRHKVETELSNICIDVMRVIDEHLIPSAAAGESTVFYYKMKGDYYRYLAEFKTGNEKKEAGDQSMKAYESATTAAEAELPPTHPIRLGLALNFSVFYYEILNSPERACHLAKQAFDEAISELDTLNEESYKDSTLIMQLLRDNLTLWTSDIPEDG

>Q9HUV7_PSEAE/44-178 PF07696

PSANQNWRLLRDESAQLRIADVLQRKEQFRPLAKRSFIFPASPQAVWLQVQLPAQKVPSWLWIFAPRVQYLDYYLVQDGQLVRDQHTGESRPFQERPLPSRSYLFSLPVDGKPMTLYVRMTSNHPLMAWFDQIDE

>Q98977_ONCMY/717-813 PF00207

SWLWIEESLPRCPDNKQCPVHNRKITLPGSITSWQITAIGLSKTHGICVSEPLKLIVWKNFFLDLKLPYSAVRNEQLEIKAILHNYMEGSITVRVEL

>CYAA_HAEIN/1-843 PF01295

MECNLAQAKQWVSALDQRRFERALQGSGDAFQHVLAIVPLLLHLNHPQLPGYVIHAPSGIASFLASDYQKKWLTNEYGIHYADHKPSTLKSAVNFHEVFPPILGVYVMGSFGSISQTSSSDLDTWICVRDGLSLDEYTLLTQKAKRISEWAMQFNVEINFYLMDQQRFRNEHYADPLTIENSGSAQYMLLLDEFYRSAVRLAGKPLLWLHLWVENEKDYEKEVARLITEGEIDPNDWVDFGGLGQFSANEYFGASLWHLYKGIDSPYKSVLKILLLEAYSKEYPNTCLIARTFKRDLLAGNTNPDHHFDPYIAILAKVTQYLTALSEFKRLDFVHRCFYVKATEDFARYQANNWRIRYMEILAQEWGWSAETVKHLNKRPFWKIKAVKENHDNIMKFLMLSYRNLVEFARKHHIHSSVVPQDINILSRKLYTAFEELPGKVSLLNTQISHNLSEAHLTFVEVRGNKHFKDGWYLINQPIHHIMFSKERVIEYGESLNKLVSWAYFNHLLTEKTELSIFSKNVTLSTLQRFVTNLRQSFPSTIAKQPKNSDLLNQCEIRSLFIAINLTTDPTSKVEEVLTGISSRDLFSFGSLEQSLVGSIDFTYRNVWNEIRTLHFEGQNAILLALKVLSNKIYRGVNRPDSIQVYCYSERYRQDLRQLVMGLVNRCVSIQVGDIQQPCQTSRLRVAGKNWQLFFEDRGISLQEIGNESVCNEAESAVDFDEVLQTPIEDGETNQESRRYPPEMDAFASEGFLQFFFEDNSDHSFNVYILDESNHLEIYRHCDGEKDEKVREINQLYQNAKQEGDKNPYNIVQHNFNYPQFYQLQNGKNGISIVPFKFRQMNK

>O29815_ARCFU/4-184 PF02737

KIGVVGFGLMGTQITQFFAQQGLEVVAIDVSEERLRKGMEAIKAGRFGLQRLVEKGKITEEEMNAVLSRISTSTSHSALKDCDLVIEAVFEDVNLKLKVLREIDAVTDAVIGSNTSSISITKLSSAVSNPERFLGIHFFNPAQIQKLVELVKGLLSDEKLVNGIRDWFLKLGKVPIVVNDS

>O29237_ARCFU/5-184 PF02737

TVAVLGAGLMGHGIAEVCAMAGYNVTMRDIKQEFVDRGMNMIKESLAKLEQKGKIKSAEEVLSRIKPTVDLEEAVKDADLVIEAVPEVVEIKKQVWEEVDKLAKPDCIFTSNTSTMRITMLADFTSRPEKFAGLHFFNPPVLMRLVEVIRGEKTSDEVMDLLVEFVKSIGKTPVRVEKDV

>Q82NF5_STRAW/7-184 PF02737

AAAVVGAGTIGLSWAALFAGHGIRVRITDPRPDLAEAVAEALDQAAGQLAAQGLDVHGLAERVLIADDLADAVRDADVVQENGPENAAFKRELFTDLVGTAPAHALLLSSSSAIPATAFTDTIEDAGRILIGHPFNPPHLVPLVEVVPGERTSEESVARAVEFYRFVGRVPVVERKEI

>O93552_CARAU/574-825 PF00003

LVFFSLFGVGLTLLVAILFYNKKDTPMVKANNSELSFLLLFSLTLCFLCSLTFIGRPTEWSCMLCHTAFGITFVLCISCVLGKTIVVLMAFKATLPGNNIMKWFGPAQQRLSVLAFTLIQVIICVLWLTISPPFPYKNMKYYKEKIILECSLGSTIGFWAVLTYISLLAFLCFILAFLARTLPDKFNEAKFITFSMLIFCAVWITFIPAYVSSPGKFTVAVEIFAILSSSFGLLFGIFAPKCYIILLKPEQN

>A1K9P1_AZOSB/217-615 PF01314

FLQATTAAKKVLADNAVTGEGLPKYGTQVLMNVINEMGALPTRNHRDVQFEDAGKISAEAMHEKRPTDGKAHLVTNAACFGCTIACGRISEIDKGHFTVKNSPKYWGASGGLEYEAAWALGAANGVGDLEALQYANLICNEQGMDPISFGATIGAVMELYEMGVLTKEQIGIDAPFGSAEALARLVEMTANGEGFGKEIGLGSKRLCAKYGHPELSMSVKGQEFPAYDSRGVQGIGLAYATSNRGACHLRGYTIASEVLGIPVKTDPHATEGKPELVKAFQDATAVFDAAGICVFTSFAWTLADVQPQLAAACEGDWSMEKLNEVGERIWNMERRFNNAAGFTAADDNLPPRLTSEPAKSGPAKGMVNELSKMLPEYYKVRGWTPEGAPTAETLARLGL

>Q6FND8_CANGA/4-456 PF07247

HLGGIEQYFYQRSSLNLHSCFSVGVELNSLPNRSDFICALRRVVNSHFQLYCNAYKSNNEVVVNPIGEPIKFDDVVDYMDWDTYGEEEINSVFRKYNFEYGVDKPLWKVIVVPKANKMIFATDHLFFDGIATVLFWTELLKSLEELKQDNDLDVDEIIYRPGGSTDSIDQCHPYEKLPIPFSWKVKRPIVKALFTIAPGAVVSQDASIIQFDEYKIPEDYLKKNSNDDGCYQIKNTNRQISLKMSSNKLKVIMKDCKQHHVSFTAYLTAVLYLALGKIPKEKYSGSTIKYEVPMNTRNRISNSGEYSNSYGTFVAGGEFIETLDFERDEWELAQSIHKKIVEKSTTGVMDAINEARLLELVDAKKFMKMKFETTTGPSSTFEVTNLGFQNFSQNEHSKYTVTDAFFNEPQLFTNLITYSVISTPSGGLNCSMAYPEKLDSILCNSVAFLKNKF

>CYAA_YERIN/1-830 PF01295

MYLYIETLKQRLDAINQLRVDRALAAMGPTFQKVYSLLPTLLHCHHPLMPGYLDGNVPHGVCLFTPNETQQDYLSEVEAKWGEPLQQSVGGELPITGVYSMGSTSSIGQCHTSDLDVWVCHQAWLDSEERNRLQEKCSLLEKWAASMGVEVSFFLIDENRFRHNASGSLGGEDCGSTQHILLLDEFYRSAVRLAGKRILWNMVPVEEENNYDDYVLSLYAQGVLTPNEWLDLGGLSTLSAEEYFGASLWQLYKSIDSPYKAVLKTLLLEAYSWEYPNSQLLAMEIKQRLHAGEIVAFGLDAYCMMLDRVTRYLTQINDTTRLNLVRRCFYLKVCEKLSRSPASVGWRREILSQLVSEWGWSDESLAVLDNRANWKIERVREAHNELLDAMMQSYRNLIRFARRNNLSVSASPQDIGVLTRKLYAAFEALPGKVTLVNPQISPDLSEEHLTFIHVPAGRANRAGWYLYNQAPSMDAIVSHQPLEYNRYLNKLVSWAYFNGLLTSKTRLHIKSANLCDTVKLQELVTDISHHFPLRLPAPTPKALYSPCEIRHLAIIVNLEHDPTAAFRNQVVHFDFRKLDVFSFGEQQQCLVGSIDLLYRNSWNEVRTLHFSGEQAVLEALKTILGKMHQDAAPPESVDVFCYSQHLRGLIRTRIQQLVSECIELRLSSTRQEPGRFKAVRVSGHTWGLFFERLSVSVQKLENAVEFYGAISNNKLHGLSIQVETDQIHLPPVVDGFASEGIIQFFFEGTADEKGFNIYILDESNRVEVYHHCEGSKEALVRDVSRFYSSSHDRFTYGSSFINFNLPQFYQIVQLDGRTQVIPFRSNTLSH

>PTHR1_HUMAN/184-466 PF00002

FDRLGMIYTVGYSVSLASLTVAVLILAYFRRLHCTRNYIHMHLFLSFMLRAVSIFVKDAVLYSGATLDEAERLTEEELRAIAQAPPPPATAAAGYAGCRVAVTFFLYFLATNYYWILVEGLYLHSLIFMAFFSEKKYLWGFTVFGWGLPAVFVAVWVSVRATLANTGCWDLSSGNKKWIIQVPILASIVLNFILFINIVRVLATKLRETNAGRCDTRQQYRKLLKSTLVLMPLFGVHYIVFMATPYTEVSGTLWQVQMHYEMLFNSFQGFFVAIIYCFCNGEV

>Q9L164_STRCO/53-116 PF03992

VYDGAQQQFLETYENLRSHVESVPGHLGEQLCQSIENPSQWLITSEWESAPPFLNWVSSEEHVR

>COMB_CLOAB/6-235 PF04029

IISADDIKEEKVKNKTAVVIDMLRATSVITTALNNGCKRVVPVLTVEEALKKVKEYGKDAILGGERKGLKIEGFDFSNSPMEYTEDVVKGKTLIMTTTNGTRAIKGSETARDILIGSVLNGEAVAEKIVELNNDVVIVNAGTYGEFSIDDFICSGYIINCVMDRMKKLELTDAATTAQYVYKTNEDIKGFVKYAKHYKRIMELGLKKDFEYCCKKDIVKLVPQYTNGEIL

>Q988C8_RHILO/4-182 PF02737

NIAIIGLGTMGPGMAARLARGGLQVVAYDVAPAAIERARSMLSVAETVLDALGIALPSAGVGTVRFTDDIGDAVSGADLVIENVPENISIKADVYRTIDGLIGQDTIVASDTSGIPITKLQAHISYPERMVGMHWSNPPHIIPMIEVIAGEKTAPQTVATIRDLIRSIGLLPVVVKKDV

>Q46EL8_METBF/210-589 PF01314

FRELEIKLLKLFDASPVLSKGLANYGTSALVKLLDYMNLIPSRNFTGKKTLFADELSGECIKSTFELENESCPGCPLGCKKKIKGAGQMFKETGSILPERAILPEGAILPDYDSLWAFGFNLENPDLTSVLKADRICKDYGLDPISAGSVLGAYAELKEGIIEANELESRLFEIGEGGKLGNGARRYLSGLGRKDLSMDVKGLELGGFDPRGIRGQALAYATSSHGGDYLTAFMVGPEVLGRPVSLNRLSLKGKAGILQVFENLNAVLDSFVFCPYSSFALNEELGSALLLFGAGVEISPAELLRIGERIYNLERTYNLKAGFSWTDDTLPERLFENEGENEGYGLPRQEFEAALKEYYHYRGWDEEGVPGSEKLKELGI

>Q9RX69_DEIRA/10-73 PF03992

VKPEFAAQFEESFRNRAGLVDGMPGFIRNEVLKPTKPGDPYIVLTYWQDEPSFRAWTESDEFKQ

>1433E_SHEEP/4-239 PF00244

REDLVYQAKLAEQAERYDEMVESMKKVAGMDVELTVEERNLLSVAYKNVIGARRASWRIISSIEQKEENKGGEDKLKMIREYRQMVETELKLICCDILDVLDKHLIPAANTGESKVFYYKMKGDYHRYLAEFATGNDRKEAAENSLVAYKAASDIAMTELPPTHPIRLGLALNFSVFYYEILNSPDRACRLAKAAFDDAIAELDTLSEESYKDSTLIMQLLRDNLTLWTSDMQGDG

>FAS2_SCHPO/1725-1788 PF01648

VGVDVELVSAISIDNETFIERNFTDTERKYCFAAPNPQASFAGRWSAKEAVFKSLGISGKGAAA

>O02314_CAEEL/99-289 PF02485

LSYGLLVYKELSQVLFMLSSIYQPQNEYCIAVGENSASTFLILLEELSDCFPNVHFMKRPPITWGSYEIINSVYDCLKFLSHLKSNWKYFQYLSGVDIPLKTNLEMVRILKSLNGTANVEIKVYENRRLLGQNETESPLPLFKSSLSSLIPRKAANYLASSSIPQQLLEFLRNTWVADEGFWGTLFGNKGL

>A1HUB8_9FIRM/220-567 PF01314

FAAKGREFHQLLLNNPQTGEYFPKYGTAAMMEITNAIGGLPTRNFRTGRFAGADKLNGEALYQTIVSRGGAGRPTHACMPGCLVRCSNVYPDQEGREVVAPLEYETLGLLGSNLEIANLDVVAQANRLCNDYGVDTIEIGCAIGVAMEAGVLPFGDEDAFLKLMEEVVTGTYLGRIIASGCVVTAKVFGVRRIPAVKGQGMPAYEPRAIKGTAVTYATTAMGADHTAGNVARANVKHHLKDGQVALSQGAQIKIGMLDALGFCMMVAPALKDSTILAELVNARYGSSLTAQDLEQLTRQYLRTEKEFNRRAGFTAAHDRLPEHFYDEANPETGTVFDITDEELASLTF

>OXAA_COXBU/367-550 PF02096

KNWGWSIIITTILIKIVFYWFSAKSFRSMARMREMQPRIQALKERHGDDRQALSRATMELYRKEKINPLGGCLPMLIQVPVFIAFYYVIIESVQLRQAPFIFWIHDLSVKDPYYILPIIMGLSMLAQQWVSPTSPDPTQQKMMWILPVIFTVFFINFPAGLVLYWITNNVVQTLQQWYVNKTYE

>VIPR1_RAT/140-397 PF00002

YNTVKTGYTIGYSLSLASLLVAMAILSLFRKLHCTRNYIHMHLFMSFILRATAVFIKDMALFNSGEIDHCSEASVGCKAAVVFFQYCVMANFFWLLVEGLYLYTLLAVSFFSERKYFWGYILIGWGVPSVFITIWTVVRIYFEDFGCWDTIINSSLWWIIKAPILLSILVNFVLFICIIRILVQKLRPPDIGKNDSSPYSRLAKSTLLLIPLFGIHYVMFAFFPDNFKAQVKMVFELVVGSFQGFVVAILYCFLNGEV

>Q89HA7_BRAJA/6-195 PF02737

NIACLGAGRMGRGIAVAFAYAGHRVTMIDVKPRSAEDFAKLETDALGEVRKTFASLSNLGLLTEADVDPLVARVSVATASQSGTALADAGMVFEGVPEVVELKREVLGAASRQVKPDTIIASTTSTILVDDLSGAIVNPHRFLNVHWLNPAYLIPLVEVSPGKATDPAIIDEVKALLEGIGKVPVVCAAT

>Q92XD1_RHIME/155-360 PF03417

WLGEGGPALIRNFDYPPSIVSDRFEMTEWSGLKVIAKAQRPWGGCVDGMNEEGLAASITLGGGRSQGRGFSIILVIRYVLETCHQVEQAVKALCRIPVALAQNVTVLDSAGNYATLFLGPGQRPIITRLRACANHQRAARPSSFSVARQQFILRALEDPSMSLEKLTDGFLRSPLYSVSATHPTLYTAVYRPAKGRVDYIWPGKRW

>O29090_ARCFU/4-179 PF02737

VIAVIGAGTMGAAIALLFANAGFEVTLVDKSRGALRRAEDRHRGESLEELEEAGLRKQDNPASLITYTTELRVYECDFIVEAIVERLRDKIELFRKIEEINSPAVLATNTSSFMPSEIARHLANPERLTLFHFSNPPILMPLVEVGGEIVSDETVERAVEMAKSIGKEPVVLRKEC

>Q9KES1_BACHD/206-436 PF00198

EDKGNRVKLSGLRKVVAKRMVDSAFSAPHVTITTEIDMSSTIKIRSQLLGMIEQETGYRLSYTEIVMKAVAHALMSHPTINASFFENEIVYHEDVHIGLAVAVEGGLVVPVVKHVDKKGLAQLTNECKTVAMAARDNRLSQEMMSGGTFTISNLGMYAIDVFTPVINQPESAILGVGRIQEKPVGIDGQIELRPMMTASLSFDHRVIDGAPAAAFLTDVKSMLEQPFQLLM

>CELR1_MOUSE/2480-2723 PF00002

VLPLKIITYAALSLSLVALLVAFVLLSLVRTLRSNLHSIHKNLIAALFFSQLIFMVGINQTENPFLCTVVAILLHYVSMGTFAWTLVENLHVYRMLTEVRNIDTGPMRFYHVVGWGIPAIVTGLAVGLDPQGYGNPDFCWLSLQDTLIWSFAGPVGTVIIINTVIFVLSAKVSCQRKHHYYERKGVVSMLRTAFLLLLLVTATWLLGLLAVNSDTLSFHYLFAAFSCLQGIFVLLFHCVAHREV

>Q9CJD7_LACLA/298-530 PF00198

GAGDRREAMNPTRKVVSKVMTAQHTHIPPVTNFDQVEVSKLVKHRAVFKEIAAKQDIKLTYLAYVAKALATTAHKFPDINASVDYEKQEIVYHEHVNLGIAVNAPTGLYVPVIHEAETKSILEIAKEIAELATATREGTLKPQQMQGSTITISNIGSARGSWFTPIINGSDVVILGLGSIVKEPIVNGEGEIVVGQNMKLSMTYDHRLIDGMLGQTSLNYLKSLLADPEFMLM

>Q9V397_DROME/375-554 PF02737

TVGVLGAGLMGAGIVQVSVDKGYQVVMKDATEAGLARGIGQVQKGLETAVKRKRISALERDQTLASLRPTLDYSDFKNADIIIEAVFEDIKVKHRVIKELEAVVPEHCVIATNTSAIPITKIAAGSSRPEKVVGMHYFSPVDKMQLLEIITHPGTSKDTIAQAVAVGLKQGKVVITVGDG

>Q9LIQ3_ARATH/69-294 PF02485

IAYLISGSSGDTRRILRLLYATYHPRNRYLLHLDSLATQSERDRLAVDVQDVPIFRAARNVDVIGKPDFAYQRGSSPMASTLHGASILLRLSGTWDWFVSISVDDYPLVTQDELLHIMSHLPKDLNFVNHTSYIGWKESRKLKPVIVDPGLYLVEKTDMFFASQKRELPKAFKLFSGPSFSILSRNFMEHCVLGTDNFPRTLLMYLSNTPDSLSNYFPTILCNTDT

>Q6ZW52_HUMAN/236-326 PF00207

TWLWDLFPIGNSGKEAVHVTVPDAITEWKAMSFCTSQSRGFGLSPTVGLTAFKPFFVDLTLPYSVVRGESFRLTATIFNYLKDCIRVQTDL

>RAD24_SCHPO/6-241 PF00244

REDAVYLAKLAEQAERYEGMVENMKSVASTDQELTVEERNLLSVAYKNVIGARRASWRIVSSIEQKEESKGNTAQVELIKEYRQKIEQELDTICQDILTVLEKHLIPNAASAESKVFYYKMKGDYYRYLAEFAVGEKRQHSADQSLEGYKAASEIATAELAPTHPIRLGLALNFSVFYYEILNSPDRACYLAKQAFDEAISELDSLSEESYKDSTLIMQLLRDNLTLWTSDAEYSA

>OXAA_CHLTR/568-773 PF02096

GSWGISIILLTIVLKLLLYPLNAWSIRSMRRMQKLSPYIQEIQQKYKREPKRAQMEIMALYKMNKVNPITGCLPLLIQIPFLIAMFDLLKSSFLLRGASFIPGWIDNLTAPDVLFSWETPIWFIGKEFHLLPILLGVVMFAQQKISAVKRSGPASDQQRQQEAMGTMMALLFTFMFYNFPSGLNIYWFSSMLLGVIQQWVTNKILD

>Q6G5X6_STAAS/23-263 PF00797

SIEALNYYATRFMLTVPFENIDVQNSKPISINIDALFNKIVHDKRGGFCYELNTFFKAYLQQKGFNPALMSATIHTPGGGRSLNGSHASLVVSINDVFYVTDVGFGDLPIHAIPITSSEHTQPITDISGTFRAIFNNEDKDIFYVQKFENDHWHTKYEAEFKPKQIEDFNSNIEYNQTNPDSIFVQHLLITMPQSFGRATMSENHLTLTRNGSSEKLAVTKNNYKHFLEKYFGLDITINRI

>O35267_RAT/404-655 PF00003

LAILALCFSALTAFVLSIFLKHQETPTVKANNRTLSYVLLISLISCFLCSLLFIGHPSFTTCIMQQTTFAVVFTVAASTVLAKTIIVILAFKVTNTSRKMRWLLVSGAPKFIIPICTMIQLILCGIWLGTSPPFVDADGHVEKGHILIFCNKGSILAFYCVLGYLVSIAIASFTLAFFARNLPDTFNEAKFLTFSMLVFCSVWVTFLPVYHSTKGKSMVAVEVFCILASSAGLLFCIFAPKCFIILLRPEKK

>Q8XXV0_RALSO/3-182 PF02737

AIGIVGTGAMGRGIAQIAAQAGLRVRLFDASPQAVEAARAALADTLARLAAKGRLTADQADAALARLMPAAALAELADCDLVVEAIVENLEAKRDLFRQLEAIVGPETILASNTSSLSITAIAAACRRPERVAGFHFFNPVPLMKVVEVIAGLRSAPAACDALAALAGRMGHAAVRCTDM

>Q67J86_SYMTH/230-603 PF01314

LKAIMEGALTAPRKGGLSLYGTNVLMNIINEVGALPAFNGKETFHPDAEAISGETIRAQYLVEEPTCHACPVACKKLVEIKEGPYAGVRTESFEYETAWALGVNCGLTDAGAVAKLLDLCNDYGMDTIELGNVLSTTMEATELGLVKDGIAWGDAARMIEVAGLIARAEGDLGKTLGLGAYGAAKAFGRPDLANSVKGQAIPAYDPRGIKGIGLGYATSNRGACHLRGYTVASEIAGIPEQTDRLATEGKGALLKVFQDLHAFSDSLDLCKFSAFAMGAEEYAQAYSAVTGVPFTAEDVMRTGERIYNLERYYNNLAGLGEGSDYLPKRFLTEPATGGSAGQVSELDVMLQEYYAARGWQNGVVPVEKLRELEI

>Q90633_CHICK/762-858 PF00207

SWLWQVEELTEPPNEQGISMKTLPIYLKDSITTWEVLAVSISENKGLCVADPYEITVMKEFFIDLRLPYSAVRNEQVEVRAILYNYWTNKIKVRVEL

>O67671_AQUAE/388-548 PF02872

EIGTAEVMLYKRDTFFSTWDWLVGEAINDYYGGDLDVVTSPGYRWGTVVLPGQKITVDHVYAFTAITYPNVYVLKRTGEQLKAVWEDVADNVFNPNPFYQQGGDMSRIWNVEYEIEVNGPQYNRIKRVWIGGKELKPKKEYLVAVYGGPPPPPEAVEPGYK

>A2M_MOUSE/750-840 PF00207

TWIWDLVPLDVSGDGELAVKVPDTITEWKASAFCLSGTTGLGLSSTISLQAFQPFFLELTLPYSVVRGEAFTLKATVLNYMSHCIQIRVDL

>VSP1_ARATH/41-269 PF03767

EAELLEKEGLSINYPNCRSWHLGVETSNIINFDTVPANCKAYVEDYLITSKQYQYDSKTVNKEAYFYAKGLALKNDTVNVWIFDLDDTLLSSIPYYAKYGYGTENTAPGAYWSWLESGESTPGLPETLHLYENLLELGIEPIIISDRWKKLSEVTVENLKAVGVTKWKHLILKPNGSKLTQVVYKSKVRNSLVKKGYNIVGNIGDQWADLVEDTPGRVFKLPNPLYYVP

>ARY2_RABIT/20-280 PF00797

DLESLTDIFQHQIRTVPYENLSIHCGESMELDLEAIFDQIVRRNRGGWCLQVNYLLYWALTTTGFETTMLGGFVYGSNNDKYSTGMIHLIVQVTINGRNYIVDAGFGRSYQMWQPVELISGKDQPQVPSIFRLREEGETWYLDQIRRQQHVPDQEFLNSELLEKKIYQKLYCFTLQPRTIEEFESANTYLQESPSSVFLDKSICSLQTPEGVHCLVGLTLTSRTYNYKENTDLVEFKVLTEEEVEGVLKTIFNISLGKKLV

>Q088L2_SHEFN/1-814 PF01295

MTDKTAYYQKDLAEKLNGIRLARVLNVLPESQLHLFHLIPFLIHHNQINVPGIIDPDTPCGIHGFTLSDAIVSACDALSLSIPEMIQPSDCVFEGIYAMGSTASFGQNPQSDVDIWLVYNSKLTDDQLKLIEYKNKLISDWFAGFEFEVNFYLVHPMQFRECSAFDNCQPVGLEHSGSSQHWLLLEEFYRSHIRLAGKVVAWWPDANSQNTEFDASLLYLGDINSLPAAEYFGASLWQLYKGLNKPHKALLKVLLLETYASEYPSTTLITQQIWQYCEQQDFSVDNDAYLLLYQRIETYLIAQGDDNRLEIVRRCFYLKSGVTLSCLSPNSAADWRVEKIQNLVKQWAWSHELVATLDNSPYWHAGQLKWFNQQLSELLLVSYKNLLQFASKQTLSERMRVEELGLLARKLHTYFSEDTHLLQPLNRLWSLSTAEKSLTIFHCRQASRFYLYRQAYLDKDPSQAVDNDPAHEFDDHSIHEADNICNLVAWSVLNGLATADTQWFQVGRGKRRTDKLSYLTRKLIPIMHNVPTVYKRDLCEPWCYQKIVLITNMDRDPTTQLSEQELILEHVNANILSFGQTKISMVSSVAIVCLNSWGEWQSHRFNGKTALLEAISFIILGLKRSNEQVDLSIISCSAKLKQPIFNQLKTLLLRCYGLMKKVNQTNTLMHPITIGEQHYSMHFNSLGMMYRKVDLITGTGNFNSSVLPYADLAEDPYLNSPSVIQKFIVMGAKQYFLRERHQTLDVFIADDNNQLEHLQYQHTSINDFVAKESHLYVFDEQKQRSPVFNMPQFFQLVDIEGKLTVIPFGLSVDE

>Q4RYQ7_TETNG/703-792 PF00207

TWIWDLVSVGDAGSVDLDKTVPDTITKWAAGAFCVSSAGLGVAPGAALTAFQPFFVSLTLPYSVVRGEVFPLRATVFNYLSDCIMVQLTL

>RAD25_SCHPO/5-240 PF00244

RENSVYLAKLAEQAERYEEMVENMKKVACSNDKLSVEERNLLSVAYKNIIGARRASWRIISSIEQKEESRGNTRQAALIKEYRKKIEDELSDICHDVLSVLEKHLIPAATTGESKVFYYKMKGDYYRYLAEFTVGEVCKEAADSSLEAYKAASDIAVAELPPTDPMRLGLALNFSVFYYEILDSPESACHLAKQVFDEAISELDSLSEESYKDSTLIMQLLRDNLTLWTSDAEYNQ

>1433T_HUMAN/3-236 PF00244

KTELIQKAKLAEQAERYDDMATCMKAVTEQGAELSNEERNLLSVAYKNVVGGRRSAWRVISSIEQKTDTSDKKLQLIKDYREKVESELRSICTTVLELLDKYLIANATNPESKVFYLKMKGDYFRYLAEVACGDDRKQTIDNSQGAYQEAFDISKKEMQPTHPIRLGLALNFSVFYYEILNNPELACTLAKTAFDEAIAELDTLNEDSYKDSTLIMQLLRDNLTLWTSDSAGEE

>O73640_FUGRU/613-867 PF00003

LTAVAVSGAVVTTAVFVVFLHYRHTPMVRANNSELSFLLLLSLKLCFLCSLVFIGRPSVWSCRFQQAAFGISFVLCVSCLQVKTIVVLAAFRSARPGAGALMKWFGPSQQRGSVCIFTCVQARVIICIVWLSLSPPVPQADLDVPGLQVTLECAMASVVGFSLVLGYIGLLACTCLLLAFLARKLPDNFNEAKLITFSMLIFCAVWVAFVPAYISSPGKYSVAVEIFAILASSYGLLFCIFAPKCFIILLRPEKN

>Q8R5U1_THETN/3-182 PF02737

KIFVVGAGTMGSGIAQVFAENGFEVVVRDIDMKFVERALGVIESNLKRNVEKGKITEEKKNEVLSRIRGTVDIDEAREADFVIEAAVENMEIKKEIFRELDNVCRKEVILATNTSSLSITEIASSTQRPEKVIGMHFFNPVPVMKLVEVIRGMKTSDETFNTVKELAQKLGKTPVEVNEA

>Q17M04_AEDAE/556-648 PF00207

TWLWTEMIEVDDKGHVDITDIIPDTMTSWSISAFAINTNHGLGVVKNPVALTVLKPFFVTVNLPYSIVKTEQAVVEVFVHNYLNQAQHVTVRV

>Q9HYE4_PSEAE/32-170 PF07696

LSLGAYAEYYRDAGGKARLGDILALPAQAFAALRGDHANFGKNAAAWWFRVRLDNRNGADLAGFLEVNYPLLDDLKVYLLTADGRIEQQESGDLFAFSQRPVQVRNFWFPLRLPPGESTLLLRVQSTSTVYLPLYFSTY

>Q75PR1_EPTBU/720-810 PF00207

TWIWTDAITGPNGSVSISATVPDTITSWVASAFSLGPKLGLGIAETQQLQAFQPFFISLKLPYSVVRGESFLLMVTVFNYLSEEQKVFVTL

>ACEK_BURS3/16-580 PF06315

VAQTMLENFDRHYRIFREAAVEAKTLYEHGDWHGLQRLARERITSYDDRVKECVEVLEDEYDAENIDDEVWQQIKLHYIGLLTSHRQPECAETFFNSVCCKILHRSYFSNDFIFVRPAISTEYLENDEPAAKPTYRAYYPGTDGLATTLERIVTNFQLEPAFDDLPRDIGCVMQAIHDEFGHFDEAPNFQIHVLSSLFFRNKSAYIVGRIINADRVLPFAVPIRHVRPGVLSLDTVLLRRDQLMIIFGFSHSYFLVDMGVPSAYVDFLCTIMPGKPKAEIYTSVGLQKQGKNLFYRDLLHHLSHSSDRFIIAPGIKGLVMLVFTLPSFPYVFKIIKDHFPPPKETTRAQIMEKYQLVKRHDRLGRMADTLEYSSVALPIARLDHALVRELEKEVPSLLEYEDGNLVIEHLYIERRMTPLNLYLQNGSDSDVEHGVKEYGNAVKELMKANIFPGDMLYKNFGVTRHGRVVFYDYDEIEYLTDCNVRRVPPPRNEEDELSGEPWYTVGPHDIFPETYGPFLLGDPRVRDVFMKHHADFFDPALWQASKDKLIQGELPDFYPYDTALR

>O44344_STRPU/784-875 PF00207

TWFFDVVSMPEDGSPYLYPVSIPSSITDWHLTAVSLSPTQGMCVEDETTVSVFQDFFIQLHLPYSVVRLEQTQVIATIFNYGFSDFEVSVNF

>OXAA_TREPA/415-618 PF02096

PNWGVAIILVTIAIKVLFFPLTKRSFIAMQKMQELQPHMQRIQERYKGNTQKIHEEMAKLYREAQYNPLSGCLPTLVQMPIIFAMYRLFNNYFEFRGAMFIPYWIPDLSLADSVWTLPFALPVTQWTQMRMLPVLYVVSQIMFSKLTQVPHTEQQKTSMTIMTYVMPLFFFFFFYDAPSGLLVYWTAMNGVTLVQQLVMKRTAN

>Q9A7J4_CAUCR/199-428 PF00198

AGSYDLVPLDGMRKTIARRMTESFRDVPHFPLTIDLEIDALLAARAKINSLLEKQGVKVSVNDIVIKAAAVALKQVPEANASYTPEGIAMHHHADIAVAVAVDGGLITPIIRKAETKGLAQISAEMKDLAQRAKDKKLKPEEFQGGTFSISNLGMFGIKSFASIINEPQGAIMSVGAGEQRPVVKNGEIKVATVMTVTLTCDHRVVDGSVGAKFLAAFRPLIEEPLTLIV

>Q6MM12_BDEBA/320-499 PF02737

GLGVLGAGTMGGGIAYVAADKGIQVRMKDLNTDALGKGLKHASDLWMKLVKRKSIDKYQFQQKMDLVSVSTDYAGFKNLDVVVEAIVEDMGIKQKVIGECAGQMRPDAIIATNTSSLSVTEMAKGHPRPEYFAGMHFFNPVNKMPLIEVIRGEKTSDETIATIYELSKKMGKMPVVVKDG

>Q8Y2T8_RALSO/5-184 PF02737

TVGIVGAGTMGNGIAQACAVAGLDVVMVDISEAAVQKGLATVAGSLDRLIKKEKLTEADKAAALARIHGATAYGDLKRADIVIEAATENLDLKIKILGQLEAVAAPHAIIASNTSSISITKLAAVLQDASRFIGMHFFNPVPMMALVEIIRGLQTGDATHAAVEALAQRLGKTPITVKNS

>ODO2_STAAM/188-420 PF00198

TKPVIREKMSRRKKTAAKKLLEVSNNTAMLTTFNEVDMTNVMELRKRKKEQFMKDHDGTKLGFMSFFTKASVAALKKYPEVNAEIDGDDMITKQYYDIGVAVSTDDGLLVPFVRDCDKKNFAEIEAEIANLAVKAREKKLGLDDMVNGSFTITNGGIFGSMMSTPIINGNQAAILGMHSIITRPIAIDQDTIENRPMMYIALSYDHRIIDGKEAVGFLKTIKELIENPEDLLL

>Q9JYI1_NEIMB/12-75 PF03992

VKPEYTETLAAQFKELVKASRAEEGNISYDLHQEIGKPNRFVFVENWKSQAAIDEHNASAHFQA

>Q7QGM3_ANOGA/633-724 PF00207

SWLWKTAIIGNSGTLKLIEVVPDTTTTWYLTGFSIDPVYGLGIIKKPIELTTVQPLIVMESLPYSIKRGEAIEIQFILISNLQEEYTVDVTL

>ODP2_ZYMMO/210-439 PF00198

DTPHNSIKLSNMRRVIARRLTESKQNIPHIYLTVDVQMDALLKLRSELNESLAVQNIKISVNDMLIKAQALALKATPNVNVAFDGDQMLQFSQADISVAVSVEGGLITPILKQADTKSLSALSVEMKELIARAREGRLQPQEYQGGTSSISNMGMFGIKQFNAVINPPQASILAIGSGERRPWVIDDAITIATVATITGSFDHRVIDGADAAAFMSAFKHLVEKPLGILA

>YGIN_SHIFL/12-83 PF03992

PGQHHRQAVLDQFAKIVPTVLKEEGCHGYAPMVDCAAGVSFQSMAPDSIVMIEQWESIAHLEAHLQTPHMKA

>1433Z_DROME/6-239 PF00244

KEELVQKAKLAEQSERYDDMAQAMKSVTETGVELSNEERNLLSVAYKNVVGARRSSWRVISSIEQKTEASARKQQLAREYRERVEKELREICYEVLGLLDKYLIPKASNPESKVFYLKMKGDYYRYLAEVATGDARNTVVDDSQTAYQDAFDISKGKMQPTHPIRLGLALNFSVFYYEILNSPDKACQLAKQAFDDAIAELDTLNEDSYKDSTLIMQLLRDNLTLWTSDTQGDE

>Q9LDF5_ARATH/6-185 PF02737

SVGVVGAGQMGSGIAQLAATSGLDVWLMDADRDALSRATAAISSSVKRFVSKGLISKEVGDDAMHRLRLTSNLEDLCSADIIVEAIVESEDIKKKLFKDLDGIAKSSAILASNTSSISITRLASATRRPSQVIGMHFMNPPPIMKLVEIIRGADTSEETFLATKVLAERFGKTTVCSQDY

>O29062_ARCFU/4-186 PF02737

KVACIGAGTVGASWASLFAWRGCDVAVYDPFPEALNRAEASIARTVSTLSEIFSGSEDDVKSALSRVKFTENLEEALKGAYYVQESAVEKLEVKRDLFEKMDAIAEPETILATSTSGLSISEIQTAARKHPERCITAHPYNPPHLIPLVEVVPRKQTDESCTEKTVEFMERMGKKPIVVKKDV

>Q6DI22_DANRE/24-209 PF02737

HVTVIGGGLMGAGIAQVAASTGHSVVLVDTSADILNKSAKGIENSLKRVAKKKFAEKPEDGEAFVQKVLKNVSTSTDAASVVHGTDLVVEAIVENLKVKQDLFGALDKVAPEHTIFASNTSSLPIADIASCTARLDRFGGLHFFNPVPMMKLVEVIKTPATSQQTFDALLEFSKALGKHPVSCKDT

>Q68M54_ACASC/2-88 PF06446

KTFSVAVAVAVVLTFICLQESSAGSFTEVQEPEEPMNNESPVAAHEEKSEESWKMPYNNRHKRSPKDCQFCCGCCPDMSGCGICCRF

>Q91076_LAMJA/760-849 PF00207

TWIWDLYPVSESGLEQVAVKVPDSITEWKASAFCSSPAGFGLSEVSSLRVFTPFFVEPVLPYSVVRGETFPLAISVHNYLHSCLKIEVTL

>O73636_FUGRU/608-859 PF00003

LAVFSVGGACLAVITAAVFFHHRTSPIVRANNSELSFLLLFSLTLCFLCSLTFIGAPSHLSCMLRHTAFGITFVLCISCVLGKTVVVLMAFRATLPGSNVMKWFGPPQQRMTVVTFTSIQVLICIVWLVVNPPFPVRNLTTYKERIILECALGSSVGFWAVLGYIGLLAAVCLVLAVLARKLPDNFNEAKMITFSMLIFCAVWITFIPAYVSSPGKFTVAVEIFAILASSFGLILCIFAPKCFIILFKPEKN

>ODP2_DICDI/363-592 PF00198

SGEFTDIPHSNIRKVTAARLTESKQTIPHYYLTMECRVDKLLKLRSELNAMNTVKISVNDFIVKASLPALRDNPVVNSTWTDQFIRRYHNIDINVAVNTPQGLFTPIVRGVDMKGLNSISTSVKQLAEKAQNGKLHPSEFESGTFTISNLGMLGIKQFAAVINPPQAAILALVPQKLVSFLSNKPDSPYETATILSVTLSCDHRVIDGAVGAEWLKSFKDYVENPIKLIL

>Q29L41_DROPS/945-1036 PF00207

VWLWRDVNIGPHGRYIFNVEVPDRPAYWMVSAFSVSPSKGFGMLNKALEYVGVQPFFINVEMPETCRQGEQVGVRVTVFNYMTTPIEATVVL

>Q99Y38_STRP1/51-257 PF03767

SRENTMSVLWYQRAAETQALYLQGYQLATDRLKEQLNKPTDKPYSIVLDIDETVLDNSPYQAKNVLEGTGFTPESWDYWVQKKEAKPVAGAKDFLQFADQNGVQIYYISDRSTTQVDATMENLQKEGIPVQGRDHLLFLEKGVKSKESRRQKVKETTNVTMLFGDNLLDFADFSKKSQEDRTALLSDLQEEFGRRFIIFPNPMYGSW

>A0RZG1_AUSSU/761-857 PF00207

SWLWLTEDLKEPPNSQGISSKTLSFYLRDSITTWEVLAVSIAPTKGICVAEPYEITVMKDFFIDLRVPYSVVKNEQVEIRAVLYNYADEDIYVRVEL

>Q5XJ69_DANRE/20-278 PF00797

DLDSLFTIHKLHVMSVPFENFSVHNGEKNSMDLHVIYNKIVKSNRGGWCCENNLLFSWALKEMGYKSTILGGRVFNSLEQDFLPSDSHLINLVEIDGKQYIADVSFGMSYQIWYPLELISGKDQPQPPGVFRLTNNGEKWILQKTGRKQIIVDNGFVDSTLVDKRLTKTLYSLTLTPRDADHFLEMSVMLQTNPDSLFLLKTICSLQTVTGYRALLGSTYSEVTFKEDSDSVEMKKILDDEIEDVLKEKFNMVLVNKFT

>Q6FVS8_CANGA/40-557 PF07247

RMGHLENYFAIMQRQKLYTNFNMYGELNKEVTREQLAVAIRQILLRHPIMMQAIIPKKFPEHEEYYTSDDYYNTPFPENDFLRVITSKIKLSDIIINEQSEDYGEIIDMILSEYKKNGYKFDAYMQELIGNIVIPIGNPNKPNWRLLCLPSAEGGGAQWKKFVYISNHCCSDAISAVNLFQDIAENVSLIEQNSWAVPYADDVIVDYEQDVADIAKLPAPITERVEYRPPLSKLPKIMLVSFLKTALNFKSDALETRCNDEYSGEPETSAVQMGDVCYDSILNYTCEEVAVIRDRIKHNVHGKCTVTPFIQAAFFVAMHQSRKLLGQKQGFKEWMSEWGVDMATPSSTRRYLPEDPEVRDMYKYGSNVGGIHYLYMISGMKVEREETEKFWSLVEYYHDILLASHSNGDQTVGLGTLMLDVIVDKKNVDKLIRDEYLYQKRGGVIMSNAGYFHQDPAQAYHVTDLVFGQRPGALKFSFGVNVVSTNIGGMNLNVGMVRRTLRDRAEFREFIGILDRVI

>ODO2_BUCAI/188-418 PF00198

NKVTNRVKMTRLRQRIAERLLDSKNNTAMLTTFHEVNMKPIILLRKKYGEDFEKKHNVRIGFMSFFVKAVIQALKNFPEINAYIDQTDIVFYKNFDISIAISTPRGLITPVIRNADTMTMAEIEKKIKDFSIKGLQNKINIKELMGGNFTITNGGVFGSLMSTPIINPPQTAILGMHVIQERPVVVNGQIKILPMMYLALSYDHRLIDGKESVGFLINIKNILEDFNRIAI

>OXAA_BUCAP/347-531 PF02096

GNWGFSIILITFMMKAITYPLTKAQYTSMSKMRELQPKINELKKNFGHDKQRMSKEIMALYKKEKINPLGGCLPVFIQMPIFLSLYYMLIGSVELRHAPFLFWIKDLSDQDPYYVLPIFMGLTMFFIQRTSSNNISDPFQQKIMHFMPFIFTVFFLWFPSGLVLYYIVSNLVTIIQQKYILSNFK

>DIHR_ACHDO/130-393 PF00002

DAAMAFVFFVGFCLSLVAIAVAIWIFLYFKDLRCLRNTIHTNLMATYICNDATWIISAVVQEYVENGGLCSVLAVLMHYFYLTNFFWMFVEGLYLFLLVVATFTGEKVKLQIYIIIGWGIPGVIVVTWAIIKHLGKTAPDNAGESHPMVLLIKHCPWMAEDYFDWIHQAPVITVLAVNLVFLFSIMWVLITKLQSANTAETQQYRKATKALLVLFPLLGITYILMMQGPMDGVAGHVFRNAQALLLSLQGFTVALFYCFLNTEV

>A2WY32_ORYSI/235-579 PF03081

IKRWILATKLVAKALAVMQRQLQAQSCGAFDRFKNDYFMAIAKNSIFVLLRFANGFTTTEAPDKLVYVLEMYEALSNATPGLLLLFTEQRVELVSRQVEVVLAKLARALRAMIGGLIARIRTADCPQTTGSAARGVGVHPLTRYAMTCVELLSPHRAALDLILANGAGESVTSLGSLVAVLVTSLERHLEEINPKLSNDDDDAAAAAAASRHLFLATNASYVARRAVDAGVEPLLGDGWAARRGSLIARYVASYVEACWAPVAACLETAGRKPVKVAAKFSSAFDEAYESQVHREIPDPALRDALRKAASEMVVPAYSAYLQNHPKLQKNVRHTAGELDRLLWEL

>ACPS_MYCTU/6-73 PF01648

VGIDLVSIPDFAEQVDQPGTVFAETFTPGERRDASDKSSSAARHLAARWAAKEAVIKAWSGSRFAQRP

>A5INK5_9THEM/716-804 PF00207

TVLWIPDVKLHDGTARISFKVPDSITSFRATAYGFSKDRFSQGEETIVVSKDFYITPHLPSFLREGDIMRLSATVFNRTGKELFVEIRI

>ODP2_CAEEL/272-507 PF00198

GQDYTDIPLSNMRKTIAKRLTESKSTIPHYYLTSEIQLDTLLQVREKLNGLLAKGTSGQATKISINDFIIKASALACQRVPEANSYWMDSFIRENHHVDVSVAVSTPAGLITPIIFNAHAKGLATIASEIVELAQRAREGKLQPHEFQGGTFTVSNLGMFGSVSDFTAIINPPQSCILAIGGASDKLVPDEAEGYKKIKTMKVTLSCDHRTVDGAVGAVWLRHFKEFLEKPHTMLL

>Q9AA61_CAUCR/39-553 PF03806

GLLGVVERLGNLLPEPVMIFVWLILGLMVLSAIGQALGWSASITYAGDEAPQFGELENGVLTYAASSLFSEANLARLFTEMPKTLTSFAPLGLVLVVILGAAVAERSGLFSALIRASLREAPKRILTPLVVIIGMVSHHASDAAYVVFIPLAGLLYAAVGRHPLAGIAAGFAAVSGGFAGNLTPGQFDVVLFGFTQEAARIIDPTWTMNPLGNWWYILAIVVVFTPIAWFLTDKVVEPRLGPWGGQADDALKAELAKSAVTADEKRGLKFAGLAALAIVALFAALSLIPGFTPLIDETKTGPAQLTPFYGALIAGFMMLFLAGGVAYGVGVGTVKTEGDVVNMMADGVRSVAPYIVFAFFAAHFVAMFNWSRLGPIAAIHGAETLKAMNLPAPLLLVSVLGFSSVLDLFIGSASAKWSALAPVVVPMFMLLGISPEMTTAAYRMGDSFTNLMTPLMSYFPLVLAMTRRWDPSMGVGSLLALMLPYALAFMVAGVAMTLAWVAFDWPLGPAAQVHY

>Q49SH4_PAROL/2-81 PF06446

KTFSVAVTVAVVLVFICIQQSSATSPEVQELEEAVSSDNAAAEHQEQSADSWMMPQNRQKRDVKCGFCCKDGGCGVCCNF

>Q9RSI3_DEIRA/19-82 PF03992

IRPGKEAEYEALLSEAIAMLAGVPGHRGTGIVRPGAGDREYTLMARFDNVDSAAAWEHSPQRLA

>S25K_SOYBN/25-251 PF03767

RMNTGYGARTPEVKCASWRLAVEAHNIFGFETIPEECVEATKEYIHGEQYRSDSKTVNQQAYFYARDLEVHPKDTFVFSIDGTVLSNIPYYKKHGYGVEKFNSTLYDEWVNKGNAPALPETLKNYNKLVSLGFKIIFLSGRTLDKQAVTEANLKKAGYHTWEKLILKDPQDPSTPNAVSYKTAAREKLIRQGYNIVGIIGDQWSDLLGGHRGESRTFKLPNPCTTFS

>Q896G2_CLOTE/101-321 PF03417

RRRENGSFIIGHNEDDEYRGNPSYMTTYIKDNEGWFTTYDYFNMPFGNAFSYNSSGIIKTINYCHSEEVNLEGIPRYFIQRHITEATSIEDFIKRCNIEDRASGFHAIALDANKNIAVSVEVTKDDISVKEIKDYYAHTNHYVHEKFKNKKIQQGSTTLFRLEKVYDLLKDKISTKGENIEIEDFNDILNYRGTSYEDSILALKSEPNFTCSRIVFDSTIK

>CASR_HUMAN/616-867 PF00003

LTLFAVLGIFLTAFVLGVFIKFRNTPIVKATNRELSYLLLFSLLCCFSSSLFFIGEPQDWTCRLRQPAFGISFVLCISCILVKTNRVLLVFEAKIPTSFHRKWWGLNLQFLLVFLCTFMQIVICVIWLYTAPPSSYRNQELEDEIIFITCHEGSLMALGFLIGYTCLLAAICFFFAFKSRKLPENFNEAKFITFSMLIFFIVWISFIPAYASTYGKFVSAVEVIAILAASFGLLACIFFNKIYIILFKPSRN

>A3BAD3_ORYSJ/211-560 PF03081

MEIWIQALRVIIGTVLPEERQACTQIFGSDSKVEEDCFARATMRFIQQLFAFGSLIANVKDEQYEKVPLLVQMLEEFLKLKPSIEALRYGDAKDAISQEADMLLEKLREEAVRLLLKFSEAQINHESYDNETIVLNGSVLSFPQYTMGVIKLLAGYSDTLNIILPVEVGGVGTVTTSPWKSYVLTLLTRLQLNIEEKSKSYKDECLRNVFLMNNAMYVLEKARSPDLKILLGDNWVTKQLVQVEQHATAYLRASWTEPLFQLKDKGINYTERSLILTKKFKNFNSIFGEISRVQTTWKVPNPQLRQHLRLVILQQVIPAYRAFVGRFGMLLNSKFIKYTLEDIENNVLDL

>O29077_ARCFU/8-190 PF02737

VIGVVGAGVMGHGIAQVAARTGYDVVMVDVSEEVLKKAMELIESGPFGLRRLVEKGKMSEDEAKAVMARIRTSTSLEALKDADFIIEAVTEKADLKKKIFAELDRICKPETIIASNTSAIMISDLATAVERKDKFIGMHWFNPAPVMRLIEVIRGALTSDETFNITVELSKKMGKIPIEAGDG

>A2MG_HUMAN/738-828 PF00207

TWIWDLVVVNSAGVAEVGVTVPDTITEWKAGAFCLSEDAGLGISSTASLRAFQPFFVELTMPYSVIRGEAFTLKATVLNYLPKCIRVSVQL

>OXAA_RICPR/359-560 PF02096

GNFGISILIVTVIIKLLMFTLANKSYRSMKKMKNLQPEIDRIKNLYNNDKARLNQEIMALYKKEKVNPVAGCLPILVQIPVFFSIYKVLYVTIEMRQAPFFGWIKDLSSPDPTTIFNLFGLLPFAPPSFLMIGAWPILMAITMFLHQKMSPELADPIQAQVMKFMPLIFLFMFSSFPVGLLIYWSWNNILSIIQQYYINKFN

>OVOS_CHICK/748-838 PF00207

TWIWDIILINSTGKASVSYTIPDTITEWKASAFCVEELAGFGMSVPATLTAFQPFFVDLTLPYSIIHGEDFLVRANVFNYLNHCIKINVLL

>ACEK_COLP3/9-578 PF06315

IANTIINGFERHFAIFTEITQSARNRFQQCQWNEIHRSARARTNFYDERVKETFNDIKEDFNISSLDDALWQRVKAVYSDLLINHKQPELAETFYNSVFCHLFERKYYHNDYIYVESTAHRLDDKTQPEIYTSYQPKELGLKQTICDIMNSHRTVIPFEDLDRDVDALINTFRRKAHKTRVKLEDLKFDILNFTFYRNKGAYLIGRVLSPAGETPFIIAVLNNEKGGLYIDALLTSSESMAVVFGFARAYFFVDCEHPYALVNFLQGLMPHKTKADLYSAIGFHKQGKTQFYRDFLNHLDSSDDQFELAAGIKGMVMSVFTLPSYPYVFKIIKDKFSPSKNITKKDVKGKYRLVKLHDRVGRMADTMEYSEVAFPKSRFNDELLAELQKVAPSIIRYEGEGEEALIIIEHLYIERRMVPLNLYLMDALKNKAQQKIDDALFGYGQAIKQLISADIFPGDMLLKNFGVTRHGRVIFYDYDEIAYMNEINFRVKPKAVTEEQLYAAEPWYSVMPGDMFPEELATFALANPSYLKAFKIHHEDLLTAAYWQQCQQDVANGIYKDVFPYPDKYR

>Q8I7P1_9CNID/768-858 PF00207

TWLYEHMKADKDGRVSFRVTVPDTITTWIMQAIAVSNTTGFGLTPPFNLKAFKSFFVSLKLPYSAQRGEQVSVIATVFNYKDQAEMVRIYL

>14331_CAEEL/5-237 PF00244

VEELVQRAKLAEQAERYDDMAAAMKKVTEQGQELSNEERNLLSVAYKNVVGARRSSWRVISSIEQKTEGSEKKQQLAKEYRVKVEQELNDICQDVLKLLDEFLIVKAGAAESKAFYLKMKGDYYRYLAEVASEDRAAVVEKSQKAYQEALDIAKDKMQPTHPIRLGLALNFSVFYYEILNTPEHACQLAKQAFDDAIAELDTLNEDSYKDSTLIMQLLRDNLTLWTSDVGAED

>Q9A803_CAUCR/19-262 PF00797

TLDTLRAIAFRHPDAIPFENLDVLLGRGISIVPSDVDAKLIGAGRGGYCYEQNGLLKRVLQALGFQVEGLMARVLWMAPEGAPPRPRSHQVLGVTIDGETWLADAGFGGCVLTAPLRLFSDEVQDSPHGKFRIVDTQTNGVAERQVQADLSGRWAPLYQVSQGAWAEVDYEQANFYTYTHPSSHFTWSMTVGRTTPTARYALKNNRFTHRDVTGAVVEQRDLTVDELEATLRQVIGLPVEADWR

>Q9PD30_XYLFA/159-389 PF00198

TRLEERVPMTRIRQRIAERLMQSKNSTAMLTTFNEINLAKVSNIRKELQEEFQKAHGIKLGFMSFFVKAAANALQRFPLVNASIDGTDIIYHGYSDISIAVSTDKGLVTPVLRNVERMSFADIEHHIADYAKKARDGKLSLEELQGGTFTVTNGGTFGSLLSTPIVNPPQSAILGMHTIKERPIAENGHIVIAPMMYVALSYDHRIIDGKDSVQFLVDIKNQLEAPGRMLF

>Q5CAP7_SCOMX/2-84 PF06446

KTLTVAVAVAVVLAFIWIQESAATFHGAQQPEEAVSNEDPAADPQETPVDSWMMPSNRQKRGMKCKFCCNCCNLNGCGVCCRF

>Q7VWZ3_BORPE/14-192 PF02737

RVAVVGAGTIGASWAALFAARGLRVTVTDPAPGAAESLRGRIADIWPALAAAGAIQDGADPDAVRFDADLQEALRDADFVQENAPERPDFKADLFTRMDAALPPHTIIASSSSGLPMSALQAGCRHPERCVIGHPFNPPHLIPLVEVVAGRQTAPAAIERAMAFYRSLGKYPIRIDKEI

>Q994E4_9ADEN/18-471 PF01686

LDPLFVPPRYLGPTEGRSSIRYSQFSPLYDTTKLYFIDNKSADIATLNYQNNHSNFLTSVVQNSDFTPLEASTQTINFDDRSRWGAEFKTILHTNMPNVTAFMFSNSFRVRVMTAKSGGVATYDWVTLSIPEGNFSDITVIDLMNNAITEHYLAVGRQNGVEVSDIGVKIDTRNFRLGFDPVTGLVMPGKYTNMAFHPDIVLAPGCAIDFTTSRLNNLLGIRKRYPFQEGFIITYEDLVGGNIPALLDVENYDEADPNTIQPLRHDSKNRSYHVGEDSSAGETFTWYRSWYLAYNYGADTGIRSTTLLVSTDVTCGAEQVYWSVPGMYTEPVTFRASQNVSNYPVVGAELLPLMSRSYYNAQAVYAQMIQESTNQTLVFNRFPENQILVRPPESTITSISENVPTQTDHGTLPIRNSISGVQRVTLTDARRRACPYVYKSIAIAQPKVLSSKTF

>A2YA16_ORYSI/142-483 PF03081

VRVWAQALSTMDRVFRLRHREARNPANEAAAAQLAALGELASASAGAMLRLATAVAALGASPSALLAALDVYVPVSEAYPGLARMFSWSTAAADAALAALVDAARRCVRGLPASIRSHYPWRMPQGGEVHPCVGFWMGYFRCMLRNRVSLYLVLAGGDGGETATTPALAPGGEGGLVADLISRLEAVLEEKSGELAFPGLRQVFMLNNTHAIVRRAVRSDLAMFLPPGWARAREERMEGYVKSYLDASWAPVVSRLAAAATKPAAVSVLRRRRDPLAAFNSALENACSAQRCWKVPSPVLRRVLRRTVSEHVVPAYRRCLEAAETPAAARTVEELERQLSEL

>A5INI5_9THEM/712-800 PF00207

TALWIPSLELHNGIARVSFKVPDSITSFRATAYGFSKDRFSQAESEMVVSKKFYLMPHLPSFLREGDVIKISATVFNRTSKTLPVQLMV

>GABR2_RAT/484-753 PF00003

LSALTILGMIMASAFLFFNIKNRNQKLIKMSSPYMNNLIILGGMLSYASIFLFGLDGSFVSEKTFETLCTVRTWILTVGYTTAFGAMFAKTWRVHAIFKNVKMKKKIIKDQKLLVIVGGMLLIDLCILICWQAVDPLRRTVERYSMEPDPAGRDISIRPLLEHCENTHMTIWLGIVYAYKGLLMLFGCFLAWETRNVSIPALNDSKYIGMSVYNVGIMCIIGAAVSFLTRDQPNVQFCIVALVIIFCSTITLCLVFVPKLITLRTNPDAA

>VSPA_SOYBN/28-254 PF03767

RMNTGYGARTPEVKCASWRLAVEAHNIFGFETIPEECVEATKEYIHGEQYRSDSKTVNQQAYFYARDLEVHPKDTFVFSIDGTVLSNIPYYKKHGYGVEKFNSTLYDEWVNKGNAPALPETLKNYNKLVSLGFKIIFLSGRTLDKQAVTEANLKKAGYHTWEKLILKDPQDPSTPNAVSYKTAAREKLIRQGYNIVGIIGDQWSDLLGGHRGESRTFKLPNPLYYIQ

>Q8E9Q9_SHEON/36-171 PF07696

NLMPWLTVTHLNTTSELADIQALPKTKWHQFTSGDIQRLSQHNFWLTFSIQSGDESLSRILALDNPLLDKVTLYHLVGNQLINTTYMGDTLPYQQRPLLSNIFLYPFKINANEQHTFYLHIETEGNAAVPINLWSA

>Q8RH78_FUSNN/95-316 PF03417

LTNKNCLVFARNSDFLVDIKKVSDSTFYKLNSNFSFIGNTTAMIQMEDGINEKGLACGLTFVYPTVKNYGFNAGFLIRYILEKCETTEQAVDFLNKVPIGSSQNIIIIDRFRNLAVAELNSSHKTIRINEANVVYRTNHFIEQTMLKYKYLGDDDVFSHLRYKTLNSQNYTEFNLSGIFELLKGKNGFICQYDKTKKFDTIWSSVFDIKNKVIYRCEGNPKQ

>Q8ZXA0_PYRAE/3-186 PF02737

KVAVIGAGTMGHGIAELFAIAGYEVALVDVAEDFLKRALQNIEWSLKKLAEKGQIKEDVGVILGRIKPIVNDVCKAVEGAELMVEAVVEDIEIKRKVFAEADRCAPPSAILATNTSSLPITEIAEAVKPERRPLVVGMHFFNPPVLMPLVEIIKGAYTSDETVKKTAEYASKLGKQTVVVNKDV

>Q3IMT3_NATPD/205-570 PF01314

FEKLRDRYEAAFKNSNTGQWLAASETLETVDFADAVGVLPTRGWQDAEFDGAEDIGIETARERAHGRERPDDPVPGGFRVDSDAGESVPRGATPIVLGAGLGIDDFDAVATLGTVCDRLALDVITAGNAVAWAVRASQEGLIDRSISFGDEAAARDLIEEIAERSTPLGDTLADGVDAAAERYGGEGLIPTVKGMELSSYDPRGAAAMALAYATSDRGGCHRRARPVEAEPIAGGDWSTERRAATVVDEQDRRAVLWSLIADDFLEEVFRADLGAEWLEALGYDHDPEDLEAVGERVWTLVRLFNVREGFCREDDELPAVLTEPPESGGEGIDPDAFDALLDRYYRQRGWDQRGRPTAELLRRLDI

>A2XIF9_ORYSI/186-499 PF03081

VQSWHTAAGFAFNFAFSRERVLCHRVFAADAALADKAAVMRARRAPERLFHVLDVHATLAEILPAIACILGDKSEAAARATAALRNAGNAARGILMSLEQAIQKTTSSKAAVTGSAVHPLTRYVMNYLVLLADYEDTLARIYQQGESTLTSGSGSASRVSPSSSADSIGRLVSVLQRKLEAMAVGYRPSALRSLFMANNTHYVSKKVRGSSKLEGIVGEDWIEEQMAETRRHVDAFVHSAWRDVLVAGGEGADAAVKEAVATQRSWVVADDEMGDAVRAAAAAVVVPAYRALYRRHGTAAWMTPGDVNAMISRQ

>ACEK_RALEJ/16-586 PF06315

VARTMLDGFDKHYRLFREVSRQAKVKFEAGDWHSLQQMQRDRIAFYNERVRETSVILEDEYDAENIEDEIWQQIKLHYIGLLTNHHQPELAETFFNSVCTRLLHRSYFNNDFIFVRPAISTEYIENEESPTRPTFRAYYPGSRQGMAECFERIVHNFQLETPFEDLRRDVGHVVRGVEEHFGDFRIAPNFQIHVLSSLFFRNKTAFIIGRIVNADRTYPLAIPIVHGPSGKLTLDAVLLKKVQVLILFSFTHSYFMVDMEIPSAYVTFLRDIMPRKPRAEIYTSLGLQKQGKNLFYRDFLHHLQHSSDKFISAPGIKGLVMLVFTLPSYPYVFKVIKDFYPAPKETTRELIKSKYQLVKQHDRVGRMADTLEYSNVAFPLSRFDEDLVRELEHHAPSMIEYQRGKDGGEEIVVRHVYIERRMTPLNIWLQEGTDEQLEHGIIEYGNAIKELIAANIFPGDMLYKNFGVTRHGRVVFYDYDEIEYFTDCNVRRVPQPRNEEEEMSGDIWYTVRPHDIFPETFRTFLLGNPRVREAFLRHHEDLFDPAMWQSHKDRLLAGHVHDFFAYPISER

>Q9A6G2_CAUCR/10-74 PF03992

VQPAKAAEFEKVFLDLAAKVKANEPGCLVYQLTRSKTEEGVYKVLELYASMDALKHHGGTDYFKA

>Q8ZT58_PYRAE/216-594 PF01314

YQKLAVEAVREGSASPSYGFWMRQGTTSTVEWAQEASVLPTYNFSEGQFEDFFKIGGNMVEKFETDLKSCPLCFMACGHWVPAESGTVEVDYENLAMLGSNLGIADLSKVGELNKIADTMGIDTISLGNVLGYVMEASERGFGDRLGFVVEWGDYEAAKQLAYDIAYRRGVGDLLAEGVKRISERIGGEDFAMHIKGLEVSAYDCHAAPAMALAYATSPIGAHHKDAWVISWEVRTDRFGYTREKASKVIELQRIRGGWFETFVGCRFPWVEVGLSLDWYPKLFKTATGLDATPDYFNEVGDRIYALIRAFWARELGYWDRELDMPPAKWFKEPLSKGPLRGAHLDYDKYHELLSHYYDLRGWDERGIPRRSTLKRLGL

>Q8WPD7_CIOIN/796-888 PF00207

RLSWPTIRIRPNGHITSYKTARDSITTFVVGAVGMQDSPDGFCIAPTKEMKVFKDVFVQINLPYSIRKLEQAQLKITIFNYNAQNNYTLRLHA

>Q20073_CAEEL/1028-1271 PF00003

LALITVLVVVAIAVLVLVLVKLYLRVVKGNQSLGISLLIGIIILYSTAFFFVFDPTDSVCRLRVILHGLGYTICFGVMIAKATQLRNAETLGFGTAIHISFWNYWLLLFFIVGVQIALSISWFLEPFMSTIGVIDTNVQRMMCTMGKVEFVVSNFYVMILIFMALFISMLNRNIKRNYKETKWLLYSTVLCFFTWVAWITLYLVLDHEFRDTVIVVELVACATILLGFLFGPKIYILLSYEPVV

>Q6V1N6_9ACTO/7-171 PF02737

TIAVIGLGTTGSVLASMAARSGRRVIAVDTDASALDLAGARLTDTGPGTIDLTTRSADIVSADLVIEAVPERMKTKCELLSHAHNACAPGAVFATTTSGLAVTDIAFGSGRPCRTVGLHLFPQGPMDPATAVEVVGTPLTDGSVLADVQALIRDLGQVPVSVPDR

>E41L2_HUMAN/886-999 PF05902

RTEISTKEVPIVQTETKTITYESPQIDGGAGGDSGTLLTAQTITSESVSTTTTTHITKTVKGGISETRIEKRIVITGDGDIDHDQALAQAIREAREQHPDMSVTRVVVHKETEL

>Q4RP15_TETNG/180-271 PF00207

TLLWIDSDVSENIWTSDKMSVPDGFTALRAVALVMSDNLGLGFTPVPQQLSVTKDFSLALNVPSCLIRGEEIVLEVHVINHLERQMEVILLL

>GLP1R_RAT/141-409 PF00002

LLSLYIIYTVGYALSFSALVIASAILVSFRHLHCTRNYIHLNLFASFILRALSVFIKDAALKWMYSTAAQQHQWDGLLSYQDSLGCRLVFLLMQYCVAANYYWLLVEGVYLYTLLAFSVFSEQRIFKLYLSIGWGVPLLFVIPWGIVKYLYEDEGCWTRNSNMNYWLIIRLPILFAIGVNFLVFIRVICIVIAKLKANLMCKTDIKCRLAKSTLTLIPLLGTHEVIFAFVMDEHARGTLRFVKLFTELSFTSFQGFMVAVLYCFVNNEV

>K6PF_PYRFU/3-447 PF04587

DEVRELGIYTAYNANVDAIVNLNAEIIQRLIEEFGPDKIKRRLEEYPREINEPLDFVARLVHALKTGKPMAVPLVNEELHQWFDKTFKYDTERIGGQAGIIANILVGLKVKKVIAYTPFLPKRLAELFKEGILYPVVEEDKLVLKPIQSAYREGDPLKVNRIFEFRKGTRFKLGDEVIEVPHSGRFIVSSRFESISRIETKDELRKFLPEIGEMVDGAILSGYQGIRLQYSDGKDANYYLRRAKEDIRLLKKNKDIKIHVEFASIQDRRLRKKVVNNIFPMVDSVGMDEAEIAYILSVLGYSDLADRIFMYNRIEDAILGGMIILDELNFEILQVHTIYYLMYITHRDNPLSEEELMRSLDFGTILAATRASLGDINDPRDVKVGMSVPYNERSEYIKLRFEEAKRKLRLKEYKVVIVPTRLVPNPVSTVGLGDTISTGTFLSYL

>Q6QZI2_PSEAM/50-149 PF00207

SWLWSDIKLPSCPQQTPNCDSTSIVKSVPLQDSITTWQFTGISLSRTRGICIGEPLEVIVRKDFFIDLRLPYSAVRGEQLEVKAILHNYTPDPITVRVDL

>GSP_ANEMI/103-165 PF01648

VGIDIERISEIDIKIAEQFFHENEYIWLQSKAQNSQVSSFFELWTIKESYIKAIGKGMYIPIN

>Q8ZVZ4_PYRAE/211-593 PF01314

MPEKLAEFLKKFVPYFVGEKSVKALFEGGTPRLVEIANQMGFFPTYNWRRVSMDGWERIAWPALKRDYFVKPAACLHCPAACHRLVRSKKYGVDVDIEYETIFALGGLTGCADPDELIRLNDLADRLGMDTISLGGVLAFAIEAAEEGKLKLEAEWGCGGLAKLIEDIAYRRGVGDILADGVKVAAERLGVGEAAVHVRGLEPAGYDPRVLKGMALNYAIGYRGADHLATMAYAIDIGGYAGGPQSLGEEKVRAVAHMEEASAVFDSLVLCKFGRGVYDMYPGGRGFEIAAELLTYVTGEDWSAKSLREAALRIINLTRVLNLKMGAGPDGLPERFFKPVRFEGREYVLTRGELEAALRSYYALRGWDEEGRPRPETLRELQL

>OXAA_ZYMMO/358-560 PF02096

GNYGLAIILMVFTIRALIFPIANKQYASMASMRRLQPKMQAVRERYKNDEARMRQELVTLYQKEKVNPFAGCLPMFIQFPIFIALYKTLLVTIESRHQPFILWIKDLSAPDPLTPFNLFGLLHFTPPHFLMIGVLPIILGITMWLQFRASPQQLEPAQQQIMSFLPLISVIFMAPLAAGLQVYYIFNNLISLAQMMWLQHRHS

>PACR_MOUSE/150-435 PF00002

YLSVKALYTVGYSTSLVTLTTAMVILCRFRKLHCTRNFIHMNLFVSFMLRAISVFIKDWILYAEQDSSHCFVSTVECKAVMVFFHYCVVSNYFWLFIEGLYLFTLLVETFFPERRYFYWYTIIGWGTPTVCVTVWAVLRLYFDDAGCWDMNDSTALWWVIKGPVVGSIMVNFVLFIGIIIILVQKLQSPDMGGNESSIYFSCVQKCYCKPQRAQQHSCKMSELSTITLRLARSTLLLIPLFGIHYTVFAFSPENVSKRERLVFELGLGSFQGFVVAVLYCFLNGEV

>CALCR_RAT/145-435 PF00002

AYVLYYLALVGHSMSIAALIASMGIFLFFKNLSCQRVTLHKNMFLTYILNSIIIIIHLVEVVPNGDLVRRDPMHIFHHNTYMWTMQWELSPPLPLSAHEGKMDPHDSEVISCKILHFFHQYMMACNYFWMLCEGIYLHTLIVMAVFTEDQRLRWYYLLGWGFPIVPTIIHAITRAVYYNDNCWLSTETHLLYIIHGPVMAALVVNFFFLLNIVRVLVTKMRQTHEAEAYMYLKAVKATMVLVPLLGIQFVVFPWRPSNKVLGKIYDYLMHSLIHFQGFFVATIYCFCNHEV

>Q82P90_STRAW/346-481 PF05270

VTPVRFSSYNYPDRYIRHWDFRARIEANVTNLADSQFRVVTGLAGSGTISLESANYPGYYLRHKNYEVWVEKNDGSSAFKNDASFSRRAGLADSADGIAFESYNYPGRYLRHYENLLRIQPVSTALDRQDATFYAE

>OXAA_HAEIN/348-532 PF02096

SNWGLAIICVTIVVKAILYPLTKAQYTSMAKMRILQPKMQEMRERFGDDRQRMSQEMMKLYKEEKVNPLGGCLPILLQMPIFIALYWTFLEAVELRHAPFFGWIQDLSAQDPYYILPILMGISMFLLQKMSPTPVTDPTQQKVMNFMPLVFMFFFLWFPSGLVLYWLVSNLITIAQQQLIYRGLE

>Q0YMR8_9DELT/231-597 PF01314

FLAALEKARSVLYGSAYAKSWAEQGTARAIVANSNAGTEAVRNYREGTFPEADKIGGDAARRDVWVKDIACYCCPLACKKSGMTKGKYGGIVHDGPEYETGVMLGSNLLIADMPGLLKAIYTIDDLGLDQISTGNVIGFLMEAYEKGLIDRGFLDGIDLKWGNVDATLAMIEKIAAKEGVGALAAEGVRALSWHIGKGSEKFAIQVKGLELAAHNIQANQPRGLSYATAERGACHMSGDDIATQNRRAMIDSIGLCLFPTFEPALEEPMLALLSAITGRDYDKAAFEKTGERIFNLEKMFNYREGFRRADDCLPDRFFEDAFTIGTKKGAVLDRSKFEEMLTRYYKDRGWDPETTKPGKAKLQELGL

>O18673_CAEEL/4453-4530 PF05902

GEYVSSKSVTQGNRTIETITYKTEKDGIVETHVEHRVTIHSDGDIDHDAELSQAILEATQMNPDMVVEKIEVRQETTQ

>OXAA_PSEPU/369-552 PF02096

GNWGWSIIVLTMLIKGLFFPLSAASYRSMARMRAVAPKLAALKERFGDDRQKMSQAMMELYKKEKINPLGGCLPILVQMPVFLALYWVLLESVEMRQAPWILWITDLSIKDPFFILPIIMGATMFIQQRLNPTPPDPMQAKVMKMMPIIFTFFFLWFPAGLVLYWVVNNCLSISQQWYITRRIE

>OXAA_THEMA/246-429 PF02096

KNFGWAIMLFTLIVRLILYPLYHAQTKSLINMRKLQPQIEAIKKKYKDPTKQQEALLKLYREAGVNPASGCLMLLIQLPIFMLLWSVIRYYVEEFAYSGSFLIWKDLSAGGFSNNWLFLVITIVASYYTTLLTSQDARTAWQGIIMSVIFPFLFVGLPSGLFLYYATNTLIQLAVTYYTYKRYK

>Q8XYB2_RALSO/32-95 PF03992

LDKADEEAFLRVWQDDANFMKRQPGFISTQLHRAIGDSPAYLNYAVWESNAHFRAAFMHPEFRA

>A1I8Q2_9DELT/218-619 PF01314

FEKTKQKAARYIKGNAMTSATYTTFGTRANVTPSNRANILPINNFFDGQDDRAVNLSGEVINKAHHTSHSTCKPCSILCGQKGEFGGEVLPVPEFETVGLLGSNIGIFDTNRIAEFNRVCGEMGMDTISAGGTLAWVMEAAEKGMVESPLRFGAAEGVSQALLDIAHCRGFGAEMAEGVRVLSEKYGGKEFAMHVKGMEMAAYDPRGSVGQGLAYAVANRGACHLSAYMIAQEIYFKLLDPYRTRAKPEFVKFFESITCCVNSLHTCQFTMFAYLLEPPSAKYSPDLVLGFFMQNVPEIAIRLIDFSVYTRLWSAVTGIPMSNGAFLAAGDRIHVLERYMNTRMGISRKDDTLPGRLLMEGRASDPRKRVVPLDKMLSKYYKLRGFDDNGIPTEKTLKRLGI

>BAI1_HUMAN/944-1191 PF00002

ATLPSVTLIVGCGVSSLTLLMLVIIYVSVWRYIRSERSVILINFCLSIISSNALILIGQTQTRNKVMCTLVAAFLHFFFLSSFCWVLTEAWQSYMAVTGHLRNRLIRKRFLCLGWGLPALVVAISVGFTKAKGYSTMNYCWLSLEGGLLYAFVGPAAAVVLVNMVIGILVFNKLVSKDGITDKKLKERAGASLWSSCVVLPLLALTWMSAVLAVTDRRSALFQILFAVFDSLEGFVIVMVHCILRREV

>Q8EYE8_LEPIN/42-182 PF07696

NISSLIEYRYRGQQFAGCSPEHIDGLEDLEWHQIPTEVLRVKRTPFGNWLRFSVKNSESTIQSRILLLGWLNVPDTQLCFFDKSGKFVSARSGYSNDIEDEKILTNLPHFRIDLEPNENRIFYLFVLSNEDINYRIQIMGL

>O93553_CARAU/589-840 PF00003

LTTISLVGAFITIIIAVIFFRYKNTPIVKANNSELSFLLLFSLMLCFLCSLTFIGRPTEWSCMLRHTAFGITFVLCISCVLGKTIVVLMAFRATLPGSNVMKWFGPPQQRLSVFSFTLIQVIICVLWLTIYPPFPFKNLNYFKEKIILECNVGSVVGFWAVLGYIGLLAILCFFLAFLARKLPDNFNEAKFITFSMLIFCAVWIAFIPAYVSSPGKFTVAVEVFAILASTYGMLFCIFIPKCYIILLKPDKN

>3HAO_HUMAN/1-149 PF06052

MERRLGVRAWVKENRGSFQPPVCNKLMHQEQLKVMFVGGPNTRKDYHIEEGEEVFYQLEGDMVLRVLEQGKHRDVVIRQGEIFLLPARVPHSPQRFANTVGLVVERRRLETELDGLRYYVGDTMDVLFEKWFYCKDLGTQLAPIIQEFF

>AAAA_EMENI/108-335 PF03417

CKTPNGALQGQNWDFFTATKENLIQLTICQPGLPTIKMITEAGIIGKVGFNSAGVAVNYNALHLHGLRPTGLPSHLALRMALESTSPSEAYEKIVSQGGMAASAFIMVGNAHEAYGLEFSPISLCKQVADTNGRIVHTNHCLLNHGPSAQELNPLPDSWSRHGRMEHLLSGFDGTKEAFAKLWEDEDNYPLSICRAYKEGKSRGSTLFNIVFDHVGRKATVRLGRPNN

>OXAA_ECOLI/352-536 PF02096

GNWGFSIIIITFIVRGIMYPLTKAQYTSMAKMRMLQPKIQAMRERLGDDKQRISQEMMALYKAEKVNPLGGCFPLLIQMPIFLALYYMLMGSVELRQAPFALWIHDLSAQDPYYILPILMGVTMFFIQKMSPTTVTDPMQQKIMTFMPVIFTVFFLWFPSGLVLYYIVSNLVTIIQQQLIYRGLE

>ODB2_BACSU/191-424 PF00198

AAGDKEIPVTGVRKAIASNMKRSKTEIPHAWTMMEVDVTNMVAYRNSIKDSFKKTEGFNLTFFAFFVKAVAQALKEFPQMNSMWAGDKIIQKKDINISIAVATEDSLFVPVIKNADEKTIKGIAKDITGLAKKVRDGKLTADDMQGGTFTVNNTGSFGSVQSMGIINYPQAAILQVESIVKRPVVMDNGMIAVRDMVNLCLSLDHRVLDGLVCGRFLGRVKQILESIDEKTSVY

>Q92MY2_RHIME/10-73 PF03992

AHAGKGDEVVALTTPLIEATLKEAGCVSYELYRKPTDPDALVFVETWKDRAAIDAHFAEPHLKA

>O73637_FUGRU/604-856 PF00003

LTAASLLGTVISVVVLGIFIHHRSTPVVRANNSELSFLLLVSLKLCFLCSLLFIGRPRLWTCQLRHAAFGISFVLCVSCILVKTMVVLAVFRASKPGGGATLKWFGAVQQRGTVLGLTSIQAAICFAWLLSSSPKPHKNIQYHKDKIVFECVVGSTVGFAVLLSYIGLLAILSFLLAFLARNLPDNFNEAKLITFSMLIFCAVWVAFVPAYINSPGKYADAVEVFAILTSSFGLLVALFGPKCYIILFRPERN

>41_HUMAN/747-861 PF05902

KSEIPTKDVPIVHTETKTITYEAAQTDDNSGDLDPGVLLTAQTITSETPSSTTTTQITKTVKGGISETRIEKRIVITGDADIDHDQVLVQAIKEAKEQHPDMSVTKVVVHQETEI

>Q9HEY2_COCCA/355-499 PF05270

VSLRATTSGYTDRYLAHSGATVNTQVVSSSSTALLKRQASWIVRAGFTNSECFAFESKDTAGSFLRHANFVLQVNANDGSKGFKEDATFCPQAGLTGKGSSIRTWAYPTRWIRHFNNVGYISSNGGVKDFDNVSSFNDDITWLVE

>FAS2_CANAL/1768-1831 PF01648

VGVDVELLSAINIDNETFIERNFTGNEVEYCLNTAHPQASFTGTWSAKEAVFKALGVESKGAGA

>Q7WLF5_BORBR/307-482 PF02737

RVHVVGAGVMGGDIAAWCAYRGLTVTLQDQDMARIAPALARAGAFFARKLKDRRAARAAFDRLVPDPQGDGVAQADLVIEAISENAQAKQALYRQLEPRLKPGALLATNTSSLSLAGLAQVLDQPQRLVGIHFFNPVARMPLVEVVLAADGDPRALARACAFVGRIGKLPLPVRDA

>EXO70_GIBZE/245-630 PF03081

GTNGMGTYAQAMEGLFISEYDNVCSVFSREDWGVIFLSTCQTALAEQARCLRELNAHIKSHLNTDCYLAYEITEIISALSGKLETRTGELKGALAAALKPVRETAKSSLAELLEETRRKVGMLQILPSDGAPIPLVSETMQRLQTMVHFLRPISSIMISIGDGGWKANAATNGRSTDAIPSLASFDIGADGKEIFSHYCLDTIEMLLSGLDQKSRVLMKSRAVAGVFMANSVVIIGRMVQTSELNDLLENKLDILEQWRKKATASYTDICKDLSVHLFDTVHTNRTNRPTSGPVDSTSIVKGLGSKDKDKIKEKFTQFNGAFDDMVSRHKSYSMEREVRRIFGEDIRQKLQPLYERFWDRYHEIDKGKGKYVKYDKTSIAAVFASL

>Q8NK89_ASPKA/350-494 PF05270

VSLRVTTPGYTTRYIAHTDTTVNTQVVDDDSSTTLKEEASWTVVTGLANSQCFSFESVDTPGSYIRHYNFELLLNANDGTKQFHEDATFCPQAALNGEGTSLRSWSYPTRYFRHYENVLYAASNGGVQTFDSKTSFNNDVSFEIE

>A2ZH43_ORYSI/264-579 PF03081

IQSWLAAARIAFSSVFPAEKELCDTVFAGDASVGDAVFEDVANNQAANLLDVAEAAVARARRAPERLFRVLDVHDALTEILPEIMSVFGDRSEVAKRGCSALFKAGEAARGALANLEVAIEKEPSKATVAGGGVHPLTRYVMNYLVFLADYEGALDRINQQQGSPERSWSIGWLVQVLMRKIEAKAGSYREAALRHLFMANNTHYVARKVAKIPSLGDDDGEAQDAARRHVEAYVRAAWGKVLKAIAAADGVEVEEAVMQAVAKQEKWVAADEEMGQVLRAAATAAVVPKYRMLYRRHGATLRLTPGDVNAIIAAL

>Q97QX5_STRPN/52-343 PF04392

KIGVLQFVSHPSLDLIYKGIQDGLAEEGYKDDQVKIDFMNSEGDQSKVATMSKQLVANGNDLVVGIATPAAQGLASATKDLPVIMAAITDPIGANLVKDLKKPGGNVTGVSDHNPAQQQVELIKALTPNVKTIGALYSSSEDNSKTQVEEFKAYAEKAGLTVETFAVPSTNEIASTVTVMTSKVDAIWVPIDNTIASGFPTVVSSNQSSKKPIYPSATAMVEVGGLASVVIDQHDLGVATGKMIVQVLKGAKPADTPVNVFSTGKSVINKKIAQELGITIPESVLKEAGQVI

>Q9FD14_STRCU/17-80 PF03992

IKSGMEHEFEKVWLEVGDSVTTHPANLGQWLSRSAEEDGIYYIVSDWVDEPLFREFETSDRHLE

>O35266_RAT/506-757 PF00003

LALIAFCLSAFTAVVLWVFVKHHDTPIVKANNRILSYILIMSLMFCFLCSFFFIGHPNRGTCILQQITFGIVFTVAVSTVLAKTITVILAFKLRDPGRSLRNFLVSGAPNYIIPICSLLQCILCAIWLAVSPPFVDIDEHSEHGHIMIVCNKGSIMAFYCVLGYLACLALGSFTTAFLAKNLPDTFNEAKFLTFSMLVFCSVWVTFLPVYHSTRGRVMVAVEIFSILASSAGMFGCIFAPKIYIILMKPERN

>ACEK_ECO57/9-568 PF06315

IAQTILQGFDAQYGRFLEVTSGAQQRFEQADWHAVQQAMKNRIHLYDHHVGLVVEQLRCITNGQSTDAEFLLRVKEHYTRLLPDYPRFEIAESFFNSVYCRLFDHRSLTPERLFIFSSQPERRFRTIPRPLAKDFHPDHGWESLLMRVISDLPLRLHWQNKSRDIHYIIRHLTETLGPENLSKSHLQVANELFYRNKAAWLVGKLITPSGTLPFLLPIHQTDDGELFIDTCLTTTAEASIVFGFARSYFMVYAPLPAALVEWLREILPGKTTAELYMAIGCQKHAKTESYREYLVYLQGCNEQFIEAPGIRGMVMLVFTLPGFDRVFKVIKDKFAPQKEMSAAHVRACYQLVKEHDRVGRMADTQEFENFVLEKRHISPALMELLLQEAAEKITDLGEQIVIRHLYIERRMVPLNIWLEQVEGQQLRDAIEEYGNAIRQLAAANIFPGDMLFKNFGVTRHGRVVFYDYDEICYMTEVNFRDIPPPRYPEDELASEPWYSVSPGDVFPEEFRHWLCADPRIGPLFEEMHADLFRADYWRALQNRIREGHVEDVYAYRRRQR

>ODO2_RAT/220-452 PF00198

LRSEHREKMNRMRQRIAQRLKEAQNTCAMLTTFNEVDMSNIQEMRARHKDAFLKKHNLKLGFMSAFVKASAFALQEQPVVNAVIDDATKEVVYRDYIDISVAVATPRGLVVPVIRNVETMNYADIERTINELGEKARKNELAIEDMDGGTFTISNGGVFGSLFGTPIINPPQSAILGMHGIFDRPVAVGGKVEVRPMMYVALTYDHRLIDGREAVTFLRKIKAAVEDPRVLLL

>Q9I6L9_PSEAE/11-73 PF03992

ARNGQSERLGLRLQQLVEPGLEAPGCLEYQVRRDALEPDLWLVCSQWREAQQMFDYLAGGIQQ

>Q4PDG8_USTMA/293-725 PF03081

GQAGAVELFEAYYGMLDNDYRILQGLMTSAELDKQTLLLASTFSQLASQPLTALIESLGTVQSHVRRHLATHTSFLLDLIGALSGIVLTGRWDVLLRAMEDSPEAPLHSTSPIDRAELDLNTGFAPAAEVLEIYSKLKNTAIGIFPRFIEDVKAIPARKVSEVPSTSVNEITYLGLQFIRQITEYSDVVSPLLHTLGNGNWMMSSGVAPILSLGLDSDASKQSIVGDYLNDVVAVVLTSLEARSRAIRQPSTASVFLLNNIGHLRRSVSAPLPSYLGAAEDGSSVSIISLHLGEMGNDLLGTALRQANTSYLDAWSPVVAPLMDDQPLNATQYHRHATSKLIGVGSGSEKNQVKDRFARFYEALEDLERLHRAYPVNREDHELKERLTRDVTRLVCPMYARFLAKHKASDFTKNPSKHIRMTEQEVEDKIASL

>A2DS47_TRIVA/251-594 PF03081

KEHPIHTFGYLISFFFKREKEFAKKVFGDKYKSPFQNSFQPLFQHFKKTCVNVSDPDLKSHVDVLFDVDIVSTIVDVLDSQVNDEDFQTYAVQLAQLSHMFHAGIERCLSNYRVAVEMNDPDNVPANGSVIANVSNVIIFLNVLGQYKAGIEQVGGLSFELYIPSVLEALFKNIIEKSTRYTDIVLRQLFLMNNSHYILAQIEQSPLLTSTIPLAFKETLEKTMQDAQKVFVDETWNKAFQILDYDRAFDGLKKGDKLNTNQKKLVKQKFKKFKDAVLTIQMKQNNYCLKNTKLMEPIMNEAIQKTHTKFETFYTRWFDSGFASHPEKYTGVQPSTLEGIINRL

>Y2045_SYNY3/23-279 PF00950

QYEFMQNAIVASLLVSLACGLIGSFIVINRMVFISGGVAHAAYGGIGLGYYFAFNPLWGAFVFSLVMALAMGWVARKYQQRSDTLIGVMWALGMAIGIMLIDLTKGYKADVASYLFGSILTVPRQELWLMAGLDMLIIILLFLLYKEFLAISFDPVYATTRNLPVDILYLTLVAAIALTVVMVMQLVGLIMVIALLSIPAAIAGQYVRDVPQMMAVASGLGMVFCGVGLALSYSFNLSSGATIILVASIAYLISLAF

>A3B510_ORYSJ/298-655 PF03081

IARWIPAFNMVFRILIPSERRLCDRVFDGLAPFGDLAFVAAVRTQALQLISFGDAISSSSRAPERLFRVVDMYEAVRDLLPDLDPVFADPYSAALRAEVTAVCNTLGSSIKGIFMELENLIRRDPARVAAQGGGIHPITRYVMNYLRAACGSRQTLEEVMEGDFGAVGGAAAAVDPDRPTSSLAVHIAWIMDVLHKNLDIKSKIYRDPSLACVFLMNNGKYIIQKVNDSELGVLLGDEWIKQMTNRVRRWSMDYQRVTWGKVTTVLQTGGPGVGGLPATAMKQKLRMFNTYFQEIYEVQSEWVIADEQLRVDVRAAVAEAVMPVYTALISRLKSSPEARHDLYIKYTPEDVEACIQHL

>Q9ZBJ9_STRCO/15-194 PF02737

TVAVVGLGTMGTGIAEILAGAGREVVGIDVSEAQAVKAVAALESSTARAVERGRLTEEERAAALGRVRTSTDLRAAAGADLVIEVVPESYEIKQRVLRELDGIVRPEAILATGTNALSVTRLAADSARPERVLGLHFFNPAPAMKLVEVVSSVLTAPAAVTAVTDLALDLGKEPVAVGDR

>Q1YY77_PHOPR/1-837 PF01295

MHNYIQTLKNRLDGHNQLRIERARAAMNVQSEQVFDLLSILLHYNHPAVPGYLEQKVPFGIANFAVSAFQQQFIDDCGLGAQSLIPTTKIDDSECSITGLYAMGSTSSIGQSLTSDLDIWVCIRTTLSVSCREKLDAKCSLVSEWAMTQGVEANFFLIDENRFRDNFSEKMTGENCGSSQHLLLLDEFYRSAVCLAGQPLLWFMVPPEMEECYDEYIEYLESAGYIRRDEWIDFGGLTRIPAEEYFGSSLWQLYKSIDSPYKSVLKAILLEAYSWEYPHTQLLSVDGKRRFFSEDRTDFCMDAYYLMLEKVTRYLERINDHRRLDLVRRCFYLKTHEKLTREPSSGSVPWRRKVLQQLTTDWHWSQEVLEELDNRRDWKVGQVKRAHNELLDALMLSYRNLIRFARRNNITSAISPEDISILARKLYAAFEVLPGKVTLLNPQISPDLHEPDLTLIQVPAGRTNTAGWYLYKQPLDPLAILGQPSLEHNRYLSKLVAWSYFNGLLTESTCLHSVANGTDMDIDKLYQLVSDMRNTFPIRRPNPGLQALSSPCEIRQLGLFINLENDPTTELKNRSVRFDFKNTDIFSYGTEQRCLVGSVDLVYRNSWNEVRTLNFSGENSMLDALKTVLGKMHQDAIPPESVDVFCYSKHMRGLIRNLVYQLIAECIEMRLKPIEQEKRRRFKAICIGDQTHGLFFERRGVSVQKLENSVDFYSCISTNKLTGTPNVVMGKMDEPHPPEIVDAYASEGLIQFFFEDCEDGYNIYILDETNRIEMYRQCSGDKDEMVHGVNRFYTSSQDRFSYSANFINFNLPQFYEIVHTDNGELRVMPFKSGQQSP

>E41L1_HUMAN/777-869 PF05902

ATEIRSLSPIIGKDVLTSTYGATAETLSTSTTTHVTKTVKGGFSETRIEKRIIITGDEDVDQDQALALAIKEAKLQHPDMLVTKAVVYRETDP

>APHA_SALTI/10-221 PF03767

AVCLLFTLNHSANALVSSPSTLNPGTNVAKLAEQAPVHWVSVAQIENSLTGRPPMAVGFDIDDTVLFSSPGFWRGKKTYSPDSDDYLKNPAFWEKMNNGWDEFSIPKEVARQLIDMHVRRGDSIYFVTGRSQTKTETVSKTLADNFHIPAANMNPVIFAGDKPGQNTKVQWLQEKNMRIFYGDSDNDITAARDCGIRGIRILRAANSTYKPL

>Q61E84_CAEBR/258-601 PF03081

DVDSCHVMCSALLSLLELEEKLMVRAIPDTSKRAQVFRELVSRPLAYVVLQTQKVVNEKDIGIIPLLPLLHLLSQNLARFHNLAANSIGDVQFDSLMRQLQVKCSSYVNEVVEHLNEDTTKFVPPDGNVHPTTASTLNFLSSLTAHRVTVTQHILALTAPQGTNTNLLLPKLFARILSALGSMLKKKANLYDDPTLATIFLLNNYNYIAKTLADEKDGLLPAITEMNSNILSFYHEEISTCTNEYLKSWNGIASVLKSTDRIGEDKQMAKQIMSTFIRDFDQILAQQMDYCISDPKICAHVQSEVRARIWKNYSQLLDACQRLHLFPQGIKYTENTFEMAIKNL

>A2MP_MOUSE/738-828 PF00207

TWIWDLVIVDSTGVAEMEVTVPDTITEWKAGAFCLSNDTGLGLSPVIDFQAFQPFFVDLTMAYSVIRGEAFTLKATVLNYLQTCIRVGVQL

>Q99TX8_STAAM/191-418 PF00198

NSENSTIPVNGVRKAIAQNMVNSVTEIPHAWMMIEVDATNLVNTRNHYKNSFKNKEGYNLTFFAFFVKAVADALKAYPLLNSSWQGNEIVLHKDINISIAVADENKLYVPVIKHADEKSIKGIAREINTLATKARNKQLTTEDMQGGTFTVNNTGTFGSVSSMGIINHPQAAILQVESIVKKPVVINDMIAIRSMVNLCISIDHRILDGLQTGKFMNHIKQRIEQYTL

>Q93KC8_EUBAC/216-599 PF01314

TKQVSSDKTGILMKDPVAGGGLPTYGSAVLVNIINENGVLPVRNFQESYTDQADKISGETLTEKYLIKKNPCYRCPIACGRWVKLDDGTECGGPEYETIWSFGADCDNYDLNSVDVANMLCNEYGLDTISVGATIAAAMELYQRGYIKDEEIKGDGVSLDWGDSKAIVAWTKKIGLREGFGDRMADGSYRLCDSYGVPEYSMTVKKQELPAYDPRAIQGHGITYAVNNRGGCHIKGYMISPEILGYPEKLDRFSDEGKAAYAKVFHDLTAVIDSLGLCIFTTFGLGLPDYVDMYNAVVGEEHDNESLLRAGDRIWTLEKLFNLKAGLTKADDTLPKRLLEDPIADGPSKDNVHHLDVLLPEYYSVRGWDENGLPTDETLRTLGL

>Q9YFI0_AERPE/217-601 PF01314

LLRISREIASRIMASSLLNREKGLLAVYGTPVAMEALGPAEAVPHLNYRNPVLERWEALSGRIMSKTILAGRLTCSSCPVACRRDTVGVSLSFRTEGPDYAQISSLGSNTGLISLEGVGYLTSLSYRLGLDPIEAGNILAMYAEITELEDLGREGLVWGDLEAMEKLLVSTAYRRGVGDLLAGGARVVAEKLKRPEVRTDVKGVTIQNADPRVEKAWGVINSVEAFGGAAHIWVYGGIVASFKTLGVNVAVDWSFNPETTAKAVYEHQLLVAAADTLQVCAFSQYAAGWRDYARALSAVTGVKWSVEKLRGAAETLLDLERLLNQQLGIEPTEDVLPPKFTENPVPKGKHKGETADVSSHIEEYYSLRNLKNGKIGSERMREATS

>Q9JMB2_RAT/843-956 PF05902

KLETSTKEVPVVHTETKTITYESSQVDPGADLEPGVLMSAQTITSETTSTTTTTHITKTVKGGISETRIEKRIVITGDADIDHDQALAQAIKEAKEQHPDMSVTKVVVHKETEI

>O45148_CAEEL/231-461 PF00198

ARDEVRVKANRMRMRIAQRLKDAQNTYAMLTTFNEIDMSSLIEMRKTYQKDFVAKHGVKLGMMSPFVRAAAYALQESPVVNAVLDENEIVYRHFVDISVAVATPKGLVVPVLRNVESMNYAQIELELANLGVKARDGKLAVEDMEGGTFTISNGGVFGSMFGTPIINPPQSAILGMHGVFDRVVPVNGKPEIRPIMQIALTYDHRLIDGREAVTFLKKIKTAVEDPRIMFM

>O28262_ARCFU/2-161 PF02737

KVFVIGAGLMGRGIAIAIASKHEVVLQDVSEKALEAAREQIPEELLSKIEFTTTLEKVKDCDIVMEAVFEDLNTKVEVLREVERLTNAPLCSNTSVISVDDIAERLDSPSRFLGVHWMNPPHVMPLVEIVISRFTDSKTVAFVEGFLRELGKEVVVCKGQ

>Q6ZMU1_HUMAN/10-104 PF00207

SWLWRKFTLPKSKSGISHYPISVKVPDSITTWQFVVVSLKAGQGLCVSDPFELTVMKSFFVDLKLPSSVIRNEQVQIQAMLYNFRDRQAKVRVEF

>1433S_HUMAN/3-238 PF00244

RASLIQKAKLAEQAERYEDMAAFMKGAVEKGEELSCEERNLLSVAYKNVVGGQRAAWRVLSSIEQKSNEEGSEEKGPEVREYREKVETELQGVCDTVLGLLDSHLIKEAGDAESRVFYLKMKGDYYRYLAEVATGDDKKRIIDSARSAYQEAMDISKKEMPPTNPIRLGLALNFSVFHYEIANSPEEAISLAKTTFDEAMADLHTLSEDSYKDSTLIMQLLRDNLTLWTADNAGEE

>VSP2_ARATH/36-264 PF03767

VAELLEKEKLSINYANCRSWHLGVETSNIIDFDTVPANCKDYVEDYLITSKQYQYDSKTVCKEAYFYAKGLALKNDTVNVWIFDLDDTLLSSIPYYAKYGYGTEKTDPGAYWLWLGTGASTPGLPEALHLYQNIIELGIEPIILSDRWKLWKNVTLDNLEAAGVTYWKHLILKPNGSNLRQVVYKSKVRKSLVKKGYNIVGNIGDQWADLVEDTPGRVFKLPNPLYYVP

>Q6UAQ6_CTEID/754-847 PF00207

SWLWEEIDLCENCPAPVKDKVLYLKDSITTWQILAVSLSPTLGICVAEPEEIVVFKHLFIDLKMPYSAVRGEQLEIKAIIHNYTPKKQKVRVEF

>A0A1G5_9BIVA/869-959 PF00207

TWLWDLHTIGDDGVVNVTTEIPHTVTEWVGNTLCSNSKDGVGISPMIGITVFQPFFLSFTLPYSAIREENLPVLVTVFNYMTECLTMEVRM

>Q92TG8_RHIME/46-225 PF02737

SAAVIGGGTMGTGIAAALLHAGLPLVLVERDEAAVERALARLRTIFDGAVKRGRISAGLAAERLAGVTGSTDYTAIAEADLIIEAVFEDLDVKRDVFRRLAAVCRADAILATNTSYLDPERIAADIGSRERFLGLHFFSPAQVMKLLEIVPTQATAPDVLATGFALARMLNKIPVRAGIS

>Q983R7_RHILO/19-79 PF03992

VPEGGQQALIDLLREAAMSCRGIPGWMSASLHRSLDGTRVVNYAQAQDQAAMQRVFEHLRG

>Y2021_HALSA/410-473 PF03992

TKPEHTAEFVEKFDTVAGVLADMDGHFDTDLMVNVENDDDMFIASQWRSQEDAMAFFRSDDFGD

>Q9YIA6_CYPCA/272-370 PF00207

SWLWEENDLPTCRETNCGTTSVTKVIYLKDSITTWQILAVSLSPTLGICVAEPEEVVVLKSFFIDLKMPYSAVRGEQLEIKAIIHNYSQMNMKKVRVEF

>Q9SZS3_ARATH/50-268 PF02485

FGYLVSGSRGDLESLWRVLRTLYHPRNQYVVHLDLESPAEERLELAKRVSQDPVFSDVGNVHMITKANLVTYRGPTMVANTLHACAILLKQSKEWDWFINLSASDYPLVTQDDLIDTFSGLDRNLNFIDHSSKLGWKEEKRAKPLIIDPGLYSTKKSDVFWVTPRRTMPTAFKLFTGNSVIKYCIWGWDNLPRTLLMYYTNFLSTPEGYFHTVICNAPE

>Q2LYI2_SYNAS/210-579 PF01314

LKKANEDVRRLMAASPVLLGELGIGQYGTAALYDLLDARRMMPTDNFRRTRFDAARRMNAHAFRQRYAPRRTGCRGCSILCKKIAADGRSLPEFETLSHFSALVGNTNLETVMEANRLCNDLGMDTISAAATLACHGEIRGRPLEPSEILSLLMDIGTGRGIGQALGQGAAAYARACGRPETAMTVKNLELPGYDPRGAYGMALAYATSTRGGCHLRAYPISHEILRKPVATDRFTFSGKARIIKGAEDLFAVVDSLTACKFVFFAASLEEYARIYTAVTGVESSADDLLTAGERIDYSERIMNDNNGFRGTDDDLPARFFELPGTGDGHLEIPPLDRAEFLKARAAYRRIRGLNEEGRPTREKAAEMAI

>HEPC_MORCS/2-85 PF06446

KTFSVAVAVAVVLAFICLQESSAVPVTEVQELEEPMSNEYQEMPVESWKMPYNNRHKRHSSPGGCRFCCNCCPNMSGCGVCCRF

>EMR1_HUMAN/599-851 PF00002

DFSLYIISHVGIIISLVCLVLAIATFLLCRSIRNHNTYLHLHLCVCLLLAKTLFLAGIHKTDNKTGCAIIAGFLHYLFLACFFWMLVEAVILFLMVRNLKVVNYFSSRNIKMLHICAFGYGLPMLVVVISASVQPQGYGMHNRCWLNTETGFIWSFLGPVCTVIVINSLLLTWTLWILRQRLSSVNAEVSTLKDTRLLTFKAFAQLFILGCSWVLGIFQIGPVAGVMAYLFTIINSLQGAFIFLIHCLLNGQV

>GCNT2_HUMAN/95-308 PF02485

LAYIMVIHHHFDTFARLFRAIYMPQNIYCVHVDEKATTEFKDAVEQLLSCFPNAFLASKMEPVVYGGISRLQADLNCIRDLSAFEVSWKYVINTCGQDFPLKTNKEIVQYLKGFKGKNITPGVLPPAHAIGRTKYVHQEHLGKELSYVIRTTALKPPPPHNLTIYFGSAYVALSREFANFVLHDPRAVDLLQWSKDTFSPDEHFWVTLNRIPGV

>O76634_CAEEL/162-372 PF02485

VAFARIVYTDYEMIEKQVQMSYHPQNSFCFAIDKKAPSQFHERLRAMAACLPNVLLLPDEESVDSAGHNINLAHYNCLRVLINKPGWNYAILLQNHDVIVKSVYEIEQIYDWLGGANDIEITPEAGRVDNKKFKWDPVSLKMFRNETGIDKNVLTTSMKFAKGAVQGSLSRGAVDWMVRTVDLSTYINQWNEGSSGVDEQFIQSFQVSADL

>GRM1_RAT/596-845 PF00003

AIAFSCLGILVTLFVTLIFVLYRDTPVVKSSSRELCYIILAGIFLGYVCPFTLIAKPTTTSCYLQRLLVGLSSAMCYSALVTKTNRIARILAGSKKKICTRKPRFMSAWAQVIIASILISVQLTLVVTLIIMEPPMPILSYPSIKEVYLICNTSNLGVVAPVGYNGLLIMSCTYYAFKTRNVPANFNEAKYIAFTMYTTCIIWLAFVPIYFGSNYKIITTCFAVSLSVTVALGCMFTPKMYIIIAKPERN

>Q9KBV0_BACHD/180-410 PF00198

QDGAKITPASAMRQVIATRMHGSLMQSAQLTMNMKADVTDLMALREEVNHTVQTRYGMKLTVTDFIARAVVLALQEHSNMNSAYIDEHIVTYEYVHLGMAVSLTQGLVVPVVQHAEALSVVELSKQIKSLSEQARTGKLQSDQLTGSTFTVTNLGVYGVDHFTPILNPPEAGILGVGAATDAPVFQGGDWQTRAMLPLSLTFDHRIVDGAPAAEFLQTIKQFLEKPTHLLL

>Q4SVH8_TETNG/907-1003 PF00207

SWLWRTVDLPAQADRDGMATKNLDSALPDSITEWGVLAISASPDTGFCVAQPYNVRAWKRFFVDLRLPYSVARNEQVEIKAVIHNYGDEDMHVRVVL

>Q9RSM4_DEIRA/27-90 PF03992

ASAEHAAHLRQLLVHIAQATRQEDGCLLYLVSEDLSQPGHFLITEHWDNLGAMHTHLALPGVTQ

>OXAA_HELPY/350-534 PF02096

GNWGWAIILLTIIVRIILYPLSYKGMVSMQKLKELAPKMKELQEKYKGEPQKLQAHMMQLYKKHGANPLGGCLPLILQIPVFFAIYRVLYNAVELKSSEWILWIHDLSIMDPYFILPLLMGASMYWHQSVTPNTMTDPMQAKIFKLLPLLFTIFLITFPAGLVLYWTTNNILSVLQQLIINKVLE

>O71096_ADEB3/30-482 PF01686

FDEIHVPPRYAAASAGRNSIRYSMLPPLYDTTKIYLIDNKSSDIQTLNYQNDHSDYLTTIVQNSDFTPLEASNHSIELDERSRWGGNLKTILYTNLPNITQHMFSNSFRVKMMASKKDGVPQYEWFPLRLPEGNFSETMVIDLMNNAIVELYLALGRQEGVKEEDIGVKIDTRNFSLGYDPQTQLVTPGVYTNEAMHADIVLLPGCAIDFTHSRLNNLLGIRKRFPYQEGFVISYEDLKGGNIPALMDVEEFNKSKTVRALREDPKGRSYHVGEDPEARENETAYRSWYLAYNYGDPEKGVRATTLLTTGDVTCGVEQIYWSLPDMALDPVTFKASLKTSNYPVVGTELLPLVPRSFYNAQAVYSQWIQEKTNQTHVFNRFPENQILVRPPAPTITSISENKPSLTDHGIVPLRNRLGGVQRVTLTDARRRSCPYVYKSLGIVTPQVLSSRTF

>A5D3S9_9FIRM/218-592 PF01314

LNFMNYEAGKWIKANPITSKGLPEFGTPVLVNLFNEMGVLPTRNFQFSRFAGALKISGEAITETMFAGRRGCYGCPVQCAMLVKTSTGITAGPEYESVWALGPQCGIDDLEVIVEANRLCNDLGLDTISTGVTIGCAMELAEKGFLKEGLNFGDGPGLLSAIRRIAFREGTGNLMAEGSRYLAGNCMAPGYAMQAKGLELPAYDPRGLQGMGLALATSNRGGCHLRAYMAGPEALGVPKMVNRFSTEGKAGLAINQQNIGAAADSLAVCRFINLAVTEEYFARILSAATGVDYRPQDLYRAGERIWNLERLYNLKAGLDSSCDTLPPRLLEEPVPDGPSRGKTVVLKPMLEEYYRFRGWDRAGRPTRKKIQELQL

>Q5V0N4_HALMA/231-629 PF01314

AMQVIQESDVTAPNEGGLSMYGTNVLMNPTEEMDGLPTKNGKYSSTRAYSDAEGDGERIIDSENVSGENVRENILVDEPTCHSCPVACKKEVEVQAMHKGEEMNVRTESYEYESAWALGPNSGHVERDKIAVMLDRCNDLGVDTIDVGNTMAMTMEMTEEGKLDELDDGLDWGDAETMIDMIEMIAHRETELADHLAEGPDHLAEEFDAHTNSLAVKGQTMAAYDPRCMKGMAIGYATSNRGACHLRGYTPAAEILGIPEKVDPREWEGKGELCATFQDLHAISDSFDICKFNAFAEGIEEYVLQYNGMTGLDVSEEELMEAGERVYNLERYYNNLAGFDGSDDDLPNRFVEGDEHAMPAQGGSEGELAELSKMKDEYYEVRGWENGVVPDEKLDELGI

>ARY3_MOUSE/20-280 PF00797

DLQTLTEILQHQIRAIPFENLNIHCGKTMELSLEDTFHQIVRKKRGGWCLQVNHLLYWALAMIGFETTMLGGCVYVPSACKYSNTMIHLLLQVTISGKTYIVDSAFPFSCQLWEPLELTSGKDQPQVPAIFHLREENGTWYLEQTKRQEYVSNQEFIDSNFLEKNTHRKIYSFTLEPRTIEDFWSISTYYQVSRTSVMTNTSLCSLHTKDGVHGLMGTILAYKKFNYKDNIDLVEFKTLKEEEIEEVLKSVFGIHLETKLV

>O64968_ARATH/249-480 PF00198

NFEDKTVPLRGFSRAMVKTMTMATSVPHFHFVEEINCDSLVELKQFFKENNTDSTIKHTFLPTLIKSLSMALTKYPFVNSCFNAESLEIILKGSHNIGVAMATEHGLVVPNIKNVQSLSLLEITKELSRLQHLAANNKLNPEDVTGGTITLSNIGAIGGKFGSLLLNLPEVAIIALGRIEKVPKFSKEGTVYPASIMMVNIAADHRVLDGATVARFCCQWKEYVEKPELLML

>Q97HJ3_CLOAB/219-623 PF01314

IRSISKLWIEQSHTSIQFKVMGLSGTLLLMDKYGEAKCLPVENAQRGYDKKCENLSPMVFKKEFETKKVPCKFCTLGCGKAYEIKKGKYKGEKGERIEYGSATSFGPNVGIYDYSDVLHLKLLCDKLGMDTIEVAATIALILECQKRGVLREDITEGRKFQFGNVDDIEYLMHKISDRSGIGDILAEGAYRASKTLKVEKYAFCIKKASTGLHSNQKKAWSLGYITSTRGGDHLKDFPFTSVFSNPLTDSLGTHIFKRKFKGTLSKDKEQGRVVWWHENYKYVIDSLGICLFCMQSITPQGHAFFDEFAEILSALFNIEVTKEDMFYAGERIYQLQNAFNINCGMKLEDYKWPSREKEEDIEDEFIKDSTIEVRDSPGMLPEYFYFRGLSSEGFPTTKRFKELQL

>Q8NLF1_CORGL/11-74 PF03992

VPEGAGEELEKRFAARQNAVDSAPGFEGFQLLRPVSGEDRYFVVTQWADEDSYNAWRDAEKAKG

>A1HS03_9FIRM/228-594 PF01314

MIKLRKEMFATLESWNAYDQWRRWGTASMLIASSQAGTHVTRNFREGTYEQVDKIGAPISEKEFWVKHRSCYQCPLHCMKIGQVTSGPYKGTIAEGPEYETGTMHGSNCLVTDLGGMMKSIELADDLGLDSITTGNILGFLMELYEKGIVTRADLDGIDMKWGNIEAMLAIQQKIAKREGVGDILAEGVKKAAAKFGQDAEKYAIQIKGQELAAWNIPANHGFAIVYGTSNRGACHQVGPTVEEQHRRTMCDTLVICRFPYYGIGTALYQKALNVITGWKLDDAGFFKVAERIWNLEKVFNAREGFRRADDYVPKRFTTEAFTVGPKKGAILPPETQEKILDEYYTKRGWDVKTSLPGEAKLKELGL

>Q91741_XENLA/767-856 PF00207

TWLWRTVKVYNGLFSEAVYMPDSITTWEIQAIGMSREKGFCIAEPLKVKVFKDFHIYLRVPYSVKRFEQMELRPILYNYNNKDLEVKVYM

>O97802_BOVIN/769-1016 PF00002

ELLLTVITWVGIVISLVCLAICIFTFCFFRGLQSDRNTIHKNLCINLFIAEFIFLIGIDKTKYMIACPIFAGLLHFFFLAAFAWMCLEGVQLYLMLVEVFESEYSRKKYYYVAGYLFPATVVGVSAAIDYKSYGTEKACWLHVDNYFIWSFIGPVTFIILLNIIFLVITLCKMVKHSNTLKPDSSRLENIKSWVLGAFALLCLLGLTWSFGLLFINEETIVMAYLFTIFNAFQGVFIFIFHCALQKKV

>Q7SD69_NEUCR/139-422 PF06472

PGLNLAFLHQFLSLMSIMVPRWKSKEAGLLVSHGIFLMLRTYLSLVVARLDGEIVRDLVAGNGKQFLLGIVKWCGLGGFASYVNATIKYLESKVSIAFRTRLTRYIHDLYLNDNLNYYKLSNLDGGVGQGADQFITQDLTLFCAAAANLYSSLGKPFVDICVFNYQLYRSLGPLALTGLLSNYFLTASILRRLSPPFGKLKAVEGRKEGDFRGLHARLIANAEEIAFYGGAEMEKTFLNREFKSLKNWMEGIYMLKIRYNILEDFILKYSWSAYGYLLSSLPVF

>GRM4_HUMAN/591-852 PF00003

PLFLAVVGIAATLFVVITFVRYNDTPIVKASGRELSYVLLAGIFLCYATTFLMIAEPDLGTCSLRRIFLGLGMSISYAALLTKTNRIYRIFEQGKRSVSAPRFISPASQLAITFSLISLQLLGICVWFVVDPSHSVVDFQDQRTLDPRFARGVLKCDISDLSLICLLGYSMLLMVTCTVYAIKTRGVPETFNEAKPIGFTMYTTCIVWLAFIPIFFGTSQSADKLYIQTTTLTVSVSLSASVSLGMLYMPKVYIILFHPEQN

>Q20081_CAEEL/103-293 PF02485

MFFIRVVSKDYDFVEEVLAMMHSPIHFFCFVLDINSEPLFKERMFRLGDCMVNVLVPRELFNTSTAHGTLNAHRRCLREIDEFDWKHAVITAEHDIPLHSTKFLSRRSRRLRDMVEMNGIHFKEETLANANPNDTSSQLFQVSRNWLQHLCSVATISHSQHKVLYDYLLKTPDFDLPNDQHREISVVEQKC

>Q9HJ31_THEAC/215-615 PF01314

LMETIKKAQIQFRNSMNLVNPWHMFGTTQITESSHLNGDTPIKNWAGVGIVDFGEENAKKISGEEIRKDVLKSYGCAQCTLACGGHVKRETRYGTVEGHRLEYEGTGAFGGLNLIADLDAMSMSFELCNRYGLDIITTGAVIAFANELYERGILTEKDIGFKIGFGNPDAEVKLTELIGKGEGIGRILGMGQRYAAKVIGKGAEESAMEIGGQDLPMHDPRLMPSLGNTYISDATPGRHTAGGIGFNEGFELVLPFKHKDSWTKIPRYEYHGKAYWQLLSVAGQEILNSTGMCLFSTNIWPNSYPYTELIKAITGWDMTEDDLVNIGWRIQIARHVFSAKQGINQYEIKPPGRVMGYPPLKAGPTAGVSIDYETLRNEYLSQLGLARDGKPNPEVIKKLGI

>Q804B3_NOTVI/122-212 PF00207

SWMWKTHKITDRSGSQSLSFTLPDSLTSWEIQGIGISDQGLCVADPVRVEVVKDVFLNAHVPYSVVRGEQIELIGSVYNYKDSRVRVCVTM

>Q8WPD8_CIOIN/773-864 PF00207

ALYLDPSTLEQAGRRQITAKARDSITTYEIDAMASADTPDGFCIAPTTNVKVFKNVFVQVYTPYSLKKREQALIKLSVFNYGDTLVTVDIMM

>O32924_MYCLE/40-357 PF06472

LSFITRWGRQYWRITRGYFIGPQSVGVWLMLAVLLFSVVISVRLNVLFSYQSKDLYNALQAAFEGAGAQNDLVKQSGMHGFWMSLGIFSILAVIFIARVMADIYLTQRFIIAWRMWLTNHLTTDWLDQRAYYREQFIDNTIDNPDQRIQQDIDIFTAGAGGTPNGPSNGTGSTLLFGAVESVISVISFTAILWNLSGRLAVFEFEIPRAMFWIVIVYVLLATIITFWIGRPLIGLNFANEKLNAAFRYALVRLRDAAEAVAFYRGEEAERKQLEQRFTPIIDNYRRYVSRTIRFLGWNASVSQTIVPLPWILQAPRLF

>Q96VA0_ASPOR/358-502 PF05270

ISLQVTTAGYTTRYLAHDGSTVNTQVVSSSSTTALRQQASWTVRTGLANSACLSFESVDTPGSYIRHYNFALLLNANDGTKQFYEDATFCPQAGLNGQGNSIRSWSYPTRYFRHYENVLYVASNGGVQTFDATTSFNDDVSWVVS

>Q9FNC4_ARATH/40-272 PF03767

SQTGVTSLKAPNLNGYCESWRVNVELNNIRDFKVVPQECVWFVQKYMTSSQYEDDVERAVDEAILYLGKTCCEKKTCDGMDAWIFDIDDTLLSTIPYHKSNGCFGGEQLNTTKFEEWQNSGKAPAVPHMVKLYHEIRERGFKIFLISSRKEYLRSATVENLIEAGYHSWSNLLLRGEDDEKKSVSQYKADLRTWLTSLGYRVWGVMGAQWNSFSGCPVPKRTFKLPNSIYYVA

>Q4S019_TETNG/605-702 PF00207

SWLWTDVVLPACPATQPACTSVKRNIPLQDSITTWQFIGISLSRSLGICVAEPLEVVVRKEFFLDLKLPSFAVRGEQLEIKVVVYNYSPDPDFVTIDL

>Q8T8P3_DICDI/93-376 PF06472

TRVDAVFFRRLAKIIRIVIPSLKSKEFLSLLYLTALLFARTMLSVSIAEIAGKNAQNLVARKWKEMRNGVLKFALVSIPASFVNASLKYETDMLALRFRKRLSEYVHKEYLEGVNFYKASHLGGADRIDNADQRVTSDIEQFCNSMSSLYTTLFKPFLDLVLFTRKLVVVMGWGSPLLMFSYFIVSGFLKKLIMPPFGRLTAKQSELEGNYRTVHQRLITNAEEIAFYDGSRKERQIINLSFGDIYNHTGYVSYLKCLVGIFDGFLVKYCASIVGYGCMVLPIY

>GHRHR_MOUSE/126-383 PF00002

FSTVKIIYTTGHSISIVALCVAIAILVALRRLHCPRNYIHTQLFATFILKASAVFLKDAAIFQGDSTDHCSMSTVLCKVSVAISHLATMTNFSWLLAEAVYLSCLLASTSPRSKPAFWWLVLAGWGLPVLCTGTWVGCKHSFEDTECWDLDNSSPCWWIIKGPIVLSVGVNFGLFLNIICILLRKLEPAQGGLHTRAQYWRLSKSTLLLIPLFGIHYIIFNFLPDSAGLDIRVPLELGLGSFQGFIVAVLYCFLNQEV

>CD97_HUMAN/544-793 PF00002

DWKLTLITRVGLALSLFCLLLCILTFLLVRPIQGSRTTIHLHLCICLFVGSTIFLAGIENEGGQVGLRCRLVAGLLHYCFLAAFCWMSLEGLELYFLVVRVFQGQGLSTRWLCLIGYGVPLLIVGVSAAIYSKGYGRPRYCWLDFEQGFLWSFLGPVTFIILCNAVIFVTTVWKLTQKFSEINPDMKKLKKARALTITAIAQLFLLGCTWVFGLFIFDDRSLVLTYVFTILNCLQGAFLYLLHCLLNKKV

>CO4B_MOUSE/779-867 PF00207

NWLWRVEPVDSSKLLTVWLPDSMTTWEIHGVSLSKSKGLCVAKPTRVRVFRKFHLHLRLPISIRRFEQFELRPVLYNYLNDDVAVSVHV

>A0LKL7_SYNFM/216-608 PF01314

FAEIVRKIIKRFRDSLKGGKHPLNLHGTAVTVMATQNFGVFPTRNFQQGTFDAWEEIHGESLTRKFLVRPKACFNCPIGCGRVTKVDEPGFQGTGEGPEYETVYAMGSNCGVSNLAAVTKANYICNEMGMDTISMGATIACAMELAERGFLPEADVGGPLRFGDARALVELTLKTASREGFGDLLARGSYRLAEHYGHPELAMVSKKQEFAGYDPRGAQGMGLAYATSPVGASHMRGDSAYIELLGVPMVLDPLSWEDKSELVKDWQDVFTVIDAAGLCVFFSVRNLVTPTRDIRPQGIMELLNAATGADYGLDELVRAGERIFNAERLFMIHAGLSCKDDSLPPRMTGEPLPDGPARGSVCRLPEMLRSYYQLRGWDENGIPTWTKLRELGL

>Q4KSK3_9ADEN/23-520 PF01686

LEVPFVPPRYMAPTEGRNSIRYSELAPQYDTTRVYLVDNKSADIASLNYQNDHSNFLTTVVQNNDFTPAEASTQTINFDERSRWGGDLKTILHTNMPNVNEYMFTSKFKARVMVARKHPENVAKEDLSQDILEYKWFEFTLPEGNFSETMTIDLMNNAILENYLQVGRQNGVLESDIGVKFDSRNFKLGWDPVTKLVMPGVYTYEAFHPDVVLLPGCGVDFTESRLSNLLGIRKKQPFQEGFKIMYEDLEGGNIPALLDTKKYLESKKELEDAAKEAAKQQGDGAVTRGDTHLTVAQEKAAEKELVIVPIEKDESGRSYNLIKDTHDTLYRSWYLSYTYGDPEKGVQSWTLLTTPDVTCGAEQVYWSLPDLMQDPVTFRSTQQVSNYPVVGAELMPFRAKSFYNDLAVYSQLIRSYTSLTHVFNRFPDNQILCRPPAPTITTVSENVPALTDHGTLPLRSSIRGVQRVTVTDARRRTCPYVYKALGIVAPRVLSSRTF

>P87500_9ADEN/1-451 PF01686

MESFVPPPRVFAPTEGRNSITYNAFAPLQDTTNLYYIDNKTSDIEALNLTNDHSDYFTNIIQNADVSPTESATQDIKLDERSRWSGNLVTLLKTNCPNVTEYNNSNKVRVRLMTDKTDPQNPVYEWVEIEIPEGNYTGNEIIDLLNNAVLEHYLKVGRQNNVEVSDIGVKFDTRMFGLGQDPVTSLIVPGRYTYKAFHPDIVLLPNCGVDFTFSRLNNILGIRKRNPYMKGFIIMYDDLEQGNIPALLDTTKYPAEVLPVLADADNVSYRVQQISTDPPAWQTEYRSWALAYHNKGPIRTTTLLTVPDITGGLGQLYWSIPDSFKAPITFTSNTSNTETLPVVAMQLFPLQQRIVYNASAVYSQLVEQMTNNTKVFNRFPNNEILMQPPYGTLTWISENVPSVADHGQQPLKNSLPGVQRITLTDDRRRTCPYIYKSLARVSPRVISSATL

>NHOA_SALTY/21-257 PF00797

TVEALRTLHLAHNCAIPFENLDVLLPREIQLDETALEEKLLYARRGGYCFELNGLFERALRDIGFNVRSLLGRVILSHPASLPPRTHRLLLVDVEDEQWIADVGFGGQTLTAPLRLQAEIAQQTPHGEYRLMQEGSTWILQFRHHEHWQSMYCFDLGVQQQSDHVMGNFWSAHWPQSHFRHHLLMCRHLPDGGKLTLTNFHFTRYHQGHAVEQVNVPDVPSLYQLLQQQFGLGVNDV

>Q9LG77_ORYSJ/43-279 PF03767

RLRQMMIPAAVGDGDYCDSWRVGVEANNVRGWTAAPRKCDNYVENYMRGHHYRRDSKVVVDEAAAYAEAAVLSGDPAADANATWVFDVDETALSHVKFYKKHGFGYHRTDEPAFMEWLIAGRASALPNTVTLYKKLLLLGVKIVFLSDRPDTPELRNATATNLIKEGFDCWDELILRSENSTATGSVVEYKSGERKKLEEEKGMVIIGNIGDQWSDLLGSPEGRRTFKLPNPAYYID

>Q7QFE4_ANOGA/61-342 PF06472

AHVNAVFFNQLRTLLGIIIPKKWSVENGLLVVIALSLIARSVSDIWMIQNATAIESTIITMNKKQFRTALVKYLSALPAIAVVNNVLKWSIGELKLRFRTNLSQYLYNEYLKGFTYYKMSNLDNRIANADQLLTTDIDKFCESVTDLYSNICKPLLDIVIYVYRLTTNLGGTTPGILLLYLFFSGVFLTNLRKPTGRLTVLEQKLEGEFRYVNSRLITNSEEIAFYKGNNREKLTILASFNKLVGHLRKFLEFRVGMGIVDNMVAKYIATVVGFYAVSLPFF

>Q67KC9_SYMTH/230-602 PF01314

QKAIMAAALTAPNKGGLSLYGTNVLMNILNEAGSLPTRNAKESHFEHGEAISGEAFREFNLASEPVCHACPVHCKKEVEVKEGRWKAKTESMEYETAWALGALCGLGDREPLVYMLDQCNDYGLDTIEMGVTLAMAMEASEKGLTPEVINWGDAEKMSELIRKTAYREGFGDILANGAYGAACLLGDPDIALSVKGQAIPAYDPRGVQGIGLGYATSNRGACHLRGYTIASEIAGIPFPTDRTVTEGKAELLKTFQDLFGFLDSMDVCKFASFAQGAEEFSAQVRALTGFDDITPEEAMRIGERVYNLERHYNNLNGFTGKDDTLPKRFFTEPATHNSAGMLSQLPVMLEEYYRLRGWKDGVVPEEKLKELEI

>A3CSC2_METMJ/217-591 PF01314

FLALKSDIAEKIRENAISGGGLPRFGTAVLVNIINENYILPVRNFQIAHFPAAENVSGERMADTILTGKMGCQGCVIQCGRDVEVEGKRTAGPEYETIWAFGPDCGIDDLAAVVKANNLCNDLGLDTISTGSTIACAMELSEKGYIDEEVRFGDAEQMLDLVHRIGYRDGIGDELAEGSFRFARRHGHPELSMSVKRQELPAYDPRGLQGHGLAYATSVRGGDHVYAYLIAPEVLGSPEKLDPYSSEGKAVWTKTFQDLTAFIDSSGSCLFTSFPLGAADYGAMVSAVTGYDIDAGEVLRIGERIWNLQKVFNIRAGCTREDDTLPPRLLREPLTEGAPKGRVWEREPLLDEYYRVRGWDREGRPTPEKLRDLGI

>Q81DJ8_BACCR/98-334 PF03417

VLPKKTDLEHTYVLRNYDLSPVIDDMRFCSTHVEGAYAHSGFSTQYFGRTEGINEHGLAVTFSACGQPVGNIAGLRKPMVSGLQCFAVIRLLLEKCKNVQEAKLLIEEIPIASNINLIIADPLNAAYIEIFDGHKSTITIDGEKQAFIVSTNHAVSSSIQKLNNRKLEQSTKRYHLLHEHLNRCEQVSIESLKKLVEEEYPAGLTVHNYEEWFGTLHSVLFDLHDRTMKICFGSPLL

>Q4RZT6_TETNG/759-856 PF00207

SWLWSEITLPSCPKPNCDTSSLVKNIPLPDSITTWQFTGISLSRTHGICVGESLKVIVRKEFFIDLRLPYSAVRGEQIEIKAILHNYSPDLITVRVNL

>A0ZWM6_DROME/176-267 PF00207

TWIFADIESTEEEVFKWVKTIPDTITNWVVTGFSLHPQKGLGVTNDQTNIKTFQPFFVSVRLPYSVKRGEVINVPALVFNYLPKTLDVELTL

>ABCD4_HUMAN/14-294 PF06472

PRLDLQFLQRFLQILKVLFPSWSSQNALMFLTLLCLTLLEQFVIYQVGLIPSQYYGVLGNKDLEGFKTLTFLAVMLIVLNSTLKSFDQFTCNLLYVSWRKDLTEHLHRLYFRGRAYYTLNVLRDDIDNPDQRISQDVERFCRQLSSMASKLIISPFTLVYYTYQCFQSTGWLGPVSIFGYFILGTVVNKTLMGPIVMKLVHQEKLEGDFRFKHMQIRVNAEPAAFYRAGHVEHMRTDRRLQRLLQTQRELMSKELWLYIGINTFDYLGSILSYVVIAIPIF

>O73638_FUGRU/594-844 PF00003

LAAVSLFGAALTSLVFCVFFRFRHTPLVKASNSELSFLLLFSLTLCFLCSLTFIGRPSRWSCVLRHTAFGITFALCMSCVLAKTVAVLFAFTAKRPGNTVFYCSVPLQRTSVFACITLQVIICVLWLTLAPPHPHKNTAHAKERIILECNLGSPVWFWVVLGYIGLLAVICFILAFLARKLPDNFNEAKFITFSMLIFCAVWVTFIPAYVSSPGKFTVAVEIFAILASSFGLLFCIFAPKCYILILKPEKN

>ACEK_IDILO/8-559 PF06315

LAKSILDGFHSHYRRFQVLTQGARERFLKRDWTAVVSAASERIHYYDHQVGTTAKKVERRVGTELDEGLWLATRQRYQQLLKFHPQAELAETFYNSVFCRVFDRAYFNNDYIFVETVLANHIPVPVENECHSYFPVVDGLEGTLTRVFEDIGLGGEFENFENDIEQLRDKFFERATETDIEAHNLRIDVLKSPFYRNKAAYIVGRVVTENNHYPFIVPVLINSQGKLYVDAFITRSDRMATIFGFARSYFMVETEAPSALVRFLKDLMPHKTLAELYSSVGFHKQGKTEFYREFLHHLRRTDDQLSAAPGVKGMVMTVFTLPSFPYVFKVIKDRLGGTKEFGRQTVIDRYRMVKRHDRVGRMADTLEFVDVALPLKRISADLLDEFKQTIANSISIEGDTLVIHQLFVERRMTPLNLYLEYANDEEVDAAMDDYGRALKEMMAANIFPGDMLLKNFGVTRHKRVVFYDYDEVRYLTDMSFRRLPENDWEVSYAPDDVFPQQLAQFAVPQAKYRNKLLKRHPELIDSAYWCRVQKNIRKGELTDVFPYDADLR

>Q9W6G1_PROAT/13-109 PF00207

SWLWNVETLPAVPNEKELSSKLVQLFLKDSITTWEVQAVSISPQKGVCVAEPYEIIVMKDFFIDLRLPYSTVRNEQIEIKAILYNYSPQEITVRMQL

>SEB1_CAEEL/164-436 PF00002

ARNARKLEFVGLGLSLVSLILAISIFSYFRRLRVFRNLLHLHLMIAMLMVVILRLVLYIDLIFTGENGPHTNSAEGKTINTMPIVCEGMFFFLEYFKTVTFCWMFLEGIYLNNQIVFGFFNSEPKLLPYFIAGYGIPLVHTMLWLLVVLIKKDFKVERCLGSYYLEPEFWILDGPRMAELVINLFFICNVIRVLYSKVRESNNTSEAGLKKSVKAAMMLLPLLGVPNIMQTIPFAPTRDNIMVFAVWTYTASFTYMYQGLMVASIYCFTNKEV

>Q9VLY7_DROME/697-788 PF00207

TWLFLNISASSDGRNSIHRRIPSEMTSWVVSAFALDPVNGLGLSPPNGKLEAYKEFYISTELPYSIKRDELIAIPFVVHNNRDSDLNVEVTF

>GPC5B_HUMAN/59-302 PF00003

VEAVAGAGALITLLLMLILLVRLPFIKEKEKKSPVGLHFLFLLGTLGLFGLTFAFIIQEDETICSVRRFLWGVLFALCFSCLLSQAWRVRRLVRHGTGPAGWQLVGLALCLMLVQVIIAVEWLVLTVLRDTRPACAYEPMDFVMALIYDMVLLVVTLGLALFTLCGKFKRWKLNGAFLLITAFLSVLIWVAWMTMYLFGNVKLQQGDAWNDPTLAITLAASGWVFVIFHAIPEIHCTLLPALQE

>ARY1_HUMAN/20-280 PF00797

DLETLTDILQHQIRAVPFENLNIHCGDAMDLGLEAIFDQVVRRNRGGWCLQVNHLLYWALTTIGFETTMLGGYVYSTPAKKYSTGMIHLLLQVTIDGRNYIVDAGFGRSYQMWQPLELISGKDQPQVPCVFRLTEENGFWYLDQIRREQYIPNEEFLHSDLLEDSKYRKIYSFTLKPRTIEDFESMNTYLQTSPSSVFTSKSFCSLQTPDGVHCLVGFTLTHRRFNYKDNTDLIEFKTLSEEEIEKVLKNIFNISLQRKLV

>Q9M3D4_ARATH/241-604 PF03081

IKNWIKAAKIGIATLFRGEKLLCDHVFSASNSTRESCFYEIANEAATNLFKFPEFVAKEKKSHERIFPLMDLQAAISDLWQDIEMIFHFDAVAGVKSQALTSLQKLKVSIHSALTDFESIIQKDTTKALTPGGGIHKLTRSTMNFISSLSKYSGVLSEILADHPLPRNTRLLESYVRAPISEDEQHNHALSVHFAWLILVLLCKLDTKAEHYKDVSLSYLFLANNLQIIIETVGSTPLRNLLGDDWLNKHEDKLCAYAGNYEIAAWSNVFMSLPEEPTDLSPEEAKIYFRRFHTAFEEAYMKQSSRVVPNAKLRDELKVSIAKKLVPEYREFYRKYLPMLGQERNIEILVRFKPDNLENYISDL

>Q9VWC8_DROME/61-342 PF06472

AHVNSVFFKQLRQLLPILIPGFWSVETGLLFLVAAALIGRSVSDIWMIQNATVVESTIIHMNRTKFKTALLKYLTALPAISVVTNVLKWSLGELKLRFRTNLTHHLYSQYLNGYTYYKMSNLDNRIANADQLLTTDIDKFCESATDLYSNISKPVLDIFIYVYRLTVNLGGKTPSILMLYLLFAGVFLTRLRRPTGRLTVEEQKLEGEFRYVNSRLITNSEEVAFYQGNVREKLTLLASYSKLRSHLRKFLEFRVSMGIIDNIIGKYFASIVGFYAVSIPFF

>OXAA_AQUAE/310-491 PF02096

GSWVLSILVLTFIVRIFLFPLGYKSVVSMQKLQELAPKMEKIKQKYKDDPVKMQEEMMKLYAETGFNPMAGCLPILLQIPIFFALYKVLIITVDLKVSSFLWIPSLADKDPYYILPVIMGLTMILQQKMTPSPDPKQALVGYITSVAFTLLFINFPAGLVLYWTLNNVFNIIQNYLIKEVLL

>ACPS_CHLPN/6-73 PF01648

IGTDIIEISRIREAIATHGNRLLNRIFTEAEQKYCLEKTDPIPSFAGRFAGKEAVAKALGTGIGSVVA

>A0H3S2_9CHLR/212-595 PF01314

MQSVLQSIQQRGERHPTANAIRAIGSAGLLPAAIKLGALTSRDARTPADGVAIARMFSEIARRGGRQERGCAGCPLPCYIDLRTRTGETHPLPSLELIAGFGARVGITDADTMLAIADRCLRLGIDPAAAAAAITFMTEAQDEGLVRQRTLNWGDGAAVIAALDRMSQRQEKRDILSLGVGEMQEAVWGSAAFAPQVKGLAMPALDPRALTEIGLAMATSPIGGDYRYAMAFEELVAEPPAWLPPPASGPRETEGKALRLIWHERFAAVLDASGLCRRLGLMAYQVTPGELIALLSAVSGRTISGADLVRIGERIVTLDRMFTRRYAANSQDTLPDRYLREPLSSGPTAGYTPPLDVLLAEYYARHGWDSAGDPTPARLAELGI

>ODP2_YEAST/248-482 PF00198

TASYEDVPISTMRSIIGERLLQSTQGIPSYIVSSKISISKLLKLRQSLNATANDKYKLSINDLLVKAITVAAKRVPDANAYWLPNENVIRKFKNVDVSVAVATPTGLLTPIVKNCEAKGLSQISNEIKELVKRARINKLAPEEFQGGTICISNMGMNNAVNMFTSIINPPQSTILAIATVERVAVEDAAAENGFSFDNQVTITGTFDHRTIDGAKGAEFMKELKTVIENPLEMLL

>Q18597_CAEEL/65-346 PF06472

ASLNAEFFAKLKKLLKILIPGPFSSEVFYMIVIGFVLLARTIADVYMITNATSVEASIVDRSPIMFAMSVFKYFLNLPAISLINALLKFSLSELKLRFRENLTKYLYGKYLGGFTYYQISNLDSRIQNPDQLLTQDVEKFCDGIVELYSNMTKPILDVFLYVFKLGRALGWEGPGLLFGYLMASMVVLTKLRRPIAKLTVEEQVLEGEYRYVNSRLIMNSEEIAFYQGNKPEKQALMGSFNNLVYHLRKTIMFRFTLGFVDNIVGKYITNIVGWVACAKTFF

>A2WVD1_ORYSI/227-593 PF03081

IKRWNRAMKVFVQVYLTSEKRLSNHVFGELSESTADLCFYEISLSSVMQLLTFYESVAIGPPKPEKLFRLLDMYEVLNDLLPEVEFLFQEGCDDIVLTEYNEVLLQLGESVRKTITEFKYAVQSYTSSNAMARGEVHPLTKYVMNYIKALTAYSKTLDSLLKDTDRRCQHFSTDIQSMANQCPHFTVSALHLQSVTAILEENLEAGSRLYRDDRLRNIFMMNNIYYMVQKVKNSELKIFLGDDWIRVHNRKFQQQAMSYERASWSQVLSFLSDDGLCAAGDGASRKIIKEKFKNFNLSFEDAYRTQTGWSIPDDQLREDVRISISLKIIQAYRTFMGRYYSRLDGTRHLERYIKYKPEDLEKLLLDL

>O05471_STREQ/39-257 PF03767

QTKVTYSDEQLRSNENTMSVLWYQRAAEAKALYLQGYQLATDRLKNQLGQATDKPYSIVLDIDETVLDNSPYQAKNILEGTSFTPESWDVWVQKKEAKPVAGAKEFLQFADQNGVQIYYISDRAVSQVDATMENLQKEGIPVQGRDHLLFLEEGVKSKEARRQKVKETTNLIMLFGDNLVDFADFSKKSEEDRTALLSELQEEFGRQFIIFPNPMYGSW

>CD97_MOUSE/526-777 PF00002

DPRLELITKVGLLLSLICLLLCILTFLLVKPIQSSRTMVHLHLCICLFLGSIIFLVGVENEGGEVGLRCRLVAMMLHFCFLAAFCWMALEGVELYFLVVRVFQGQGLSTWQRCLIGYGVPLLIVAISMAVVKMDGYGHATYCWLDFRKQGFLWSFSGPVAFIIFCNAAIFVITVWKLTKKFSEINPNMKKLRKARVLTITAIAQLLVLGCTWGFGLFLFNPHSTWLSYIFTLLNCLQGLFLYVMLCLLNKKV

>Q98HP0_RHILO/10-70 PF03992

ASRGQRDAVMDLLRASTGALPGCLSYIIATDPADADAIWVTEVWTDQASHKASLQLPEVQA

>ABCD1_HUMAN/65-352 PF06472

AGMNRVFLQRLLWLLRLLFPRVLCRETGLLALHSAALVSRTFLSVYVARLDGRLARCIVRKDPRAFGWQLLQWLLIALPATFVNSAIRYLEGQLALSFRSRLVAHAYRLYFSQQTYYRVSNMDGRLRNPDQSLTEDVVAFAASVAHLYSNLTKPLLDVAVTSYTLLRAARSRGAGTAWPSAIAGLVVFLTANVLRAFSPKFGELVAEEARRKGELRYMHSRVVANSEEIAFYGGHEVELALLQRSYQDLASQINLILLERLWYVMLEQFLMKYVWSASGLLMVAVPII

>STCJ_EMENI/1429-1495 PF01648

IGVDTVTLSSFNAHENAIFLQRNYTERERQSLQLQSHRSFRSAVASGWCAKEAVFKCLQTVSKGAGA

>O44608_CAEEL/12-200 PF02737

LVAIFGAGMMGSGIAQVCLQAGYPVNLYGRSEKKLLEARETIKKNLIRVASKKKTDVPMEPAALEEIAQIQLDLLQIHTDIPSAAEDAAMAIEAVAENLDLKLDIFQTIQKTCPQNCMLITNTSSLKLSQMLPVIQNPALFAGLHFFNPVPVMKLVEVVSTDETSPETTNFLFNFCKEIKKLPVAAKDT

>Q978T2_THEVO/5-184 PF02737

KVTVIGSGIMGHGIAETIALAGYDVNLEDISDDVLAKAKAEIDASLDRLVKSGKLSDKTKVLGRIHYFTSIPESVKDADLVIEAVPEILDIKRQVFAQLDQSTKEDAILATNTSNIRLTEIAEGVKKKGKVVGMHFFNPPVVLKLVEVIRSDYTEDEVFEAVYDFSKKIGKIPIKVYKDT

>A4J5K2_9FIRM/208-622 PF01314

LLATTLEAQNLIKSASSYGAFCHYGSMLATIPYGNFKALSAHNFSKGTLPNWNERFGRQVVDEYSSRHIGCQSCIIACAHWVEIKEGKYKGTELKDMEISPTVAFGGNVGLSLEATVVASKLCRQNGLDMLSTGGVIAFAIELFQKGIITREDLGYDLSFGDDEAAFRLLNDIVKRNGVGNILAEGTKRAASQLKGSEQCAIHIKGLEVPMIDPRNRWSTWTLGLLTNIRGGDHLRCRNPVENLRFNENKHDYIKERFGHKGPMYDQLDMPEDLKKDIIDLDGDLVDIAKMSKWAEDLINLYNAIGICIRPPVLEKIGPTILSEAYTVHTGIKITPEDLIKSAERSWNLIKLFNIREGEDIKETKFPRRFFDEELYGKVLDEEKVQRVLEKYFVARGWEPSTGKPTKEKLRELGL

>Q8KSV9_9ACTO/13-76 PF03992

HLSPSGEDVLAAYHEASRRMAGTPGLLGNQLMSEVGRPDSYVVVSHWDAWESFETWESGSEHKE

>O35268_RAT/599-850 PF00003

LSLMALCLAALTVVVLGVFVKHHRTPIVKANNCTLTYILLIALIFCFLCPLFFIGHPNSATCILQQITFGVVFTVAISTVLAKTTTVILAFRVTAPHRMMKYFLVSRASNYIIPICTLIQIIVCAIWLGASPPSVDIDAQSEHGHIIIACNKGSVTAFYCVLGYLACLAFVSFTLAFLSRNLPVTFNEAKSMTFSMLVFCSVWVTFLPVYHGTKGKVMVAVEIFSTLASSAGMLGCIFAPKCYTILFRPDRN

>Q9L6H8_BRUME/176-406 PF00198

ASREERVKMTRLRQTIARRLKDAQNTAAMLTTFNEVDMSAVMELRNKYKDVFEKKHGVKLGFMGFFTKAVTHALKEIPAVNAEIDGTDIVYKNFCHVGMAVGTDKGLVVPVIRDADQMSIAEVEKELARLARAARDGSLSMADMQGGTFTITNGGVYGSLMSTPILNAPQSGILGMHKIQERPMVVGGQIVVRPMMYLALSYDHRIVDGKEAVTFLVRVKESLEDPERLVL

>Q0A941_ALHEH/217-616 PF01314

FLQATEQGKKILADNEVTGQGLPTYGTQVLMNVINEMGAMPTRNMREVQFEGAHKISGEAMHEPRPTDQRPNLTTNAACFGCTIACGRISRIDNGHFTVEHKPKYWGNSGGLEYEAAWALGSDTGVDDLEALTYANFLCNEDGFDPISFGATVAAAMELYENGAITDAHTDGLQLRFGSAEALSRCAELVAAGEGFGKDLGLGSKRLCEKYGQPELSMTVKGQEFPAYDPRGIQGMGLAYATSNRGACHLRGYTVSSEVLGIPEKTDPLTTEGKPKLVKAFQDAAAMVDSSGLCLFTTFAWTPDDIAPQIDAACGGGWDTERLLEMGERIWNLERDFNNRAGLTGADDTLPKRLLKEAAKVGPAKGRANDLGEMLPRYYEIRGWTPDGQVSETTRQRLRL

>Q6TL26_9BIVA/826-916 PF00207

TFLWDLELIGDEGEVVLTRNLPHTITEWVGNTICANTEVGIGTSPLATITAFQPFFLSFTLPYSAVRGETVPVTVTIFNYLQECLVMLVRL

>A7PAA6_VITVI/270-638 PF03081

IRRWVRAMKIFVRVYLASEKWLSDQVFGEVGSVSSACFVEASRASIFQLLNFGEAIVIGPHKPEKLMRILDMYEVLADLLPDIDGIYQEDIGSSVRTECREVLGGLGDCVRATFLEFENAIASNTSTNPFAGGGIHPLTRYVMNYIKILTDYSNTINLLFEDHDRADPGSLSSNTSPVTEEENKSGSSSCSTPTGLHFRALISVLECNLEDKSKLYRDVALQHLFLMNNIHYMTEKVKNSELRDVFGDEWIRKHNWKFQQHAMNYERASWSSILLLLKEEGIQNSNSNSPSKTVLKDRLRSFNVAFEELYKSQTAWLIPDSQLRDELQISTSLKVVQAYRTFVGRHNPHISDKHIKYSPDDLQNFLLDL

>A2BLI7_HYPBU/221-613 PF01314

AKKLSKAIVEHSISQSLTKYGTAVLVNIINEHGGLPTKNWTRGVFEKAQQISGEYLAEHYLKTNKGCWGCAIRCSRVAEVKSGPYRTPVSEGPEYETIWANGANTMIGNMEAIIKINYLLNDMGLDTISFGNTAATLMELYEKAQKGELPEDKAKKLLDLLEDVEPTWGNADAVIRLIWKTAYRDGIGDYTAEGAARLAEEFGCPDCAIHVRGLELPAYDPRAINSMALSYATSNRGGCHLRAYGVSFDVLGVPKKFDPLKIDLEKVKLIKWQQDYFAVIDSLVVCKFNTFADAPEYYVELLKYAMGWEDLTVEELLTIGERIYNVERLFAVREGRGYRDYLPKRLLEEPLPDGPAKGRTAKEALETYLPEYYKLRGWVDGKPTPETLKRLGL

>A8GZG5_9GAMM/1-803 PF01295

MLEDIDKLQHAAQRLDIIRYARAIALLSPLKRHLLRLIPAFLHYHAPKLPGYNGPLTPSGIVDYHTDSETLDACETLEIDLSTEVVSDTPSIEGIYSMGSTSSFGQNPQSDIDVWVVHSEQLSKDQCELLANKAVLLTQWFAQFDFEVNFYLVHPEQFIRDQGEHADRYNCMGQEHSGSAQHWLLLEEFYRSQFRLAGKPIGWWPSAGKSSNLLSLGDANALPASEYFGASLWQLYKGVDKPHKALLKVLLLEAYASEYPHTRLVSERIWQRTLEGDFSSSNDAYYLLYESIETYLLGRGEGRRLEIARRCFYLKCGVKLSQSDQAVDWRYYKMKKLVNHWNWSESLLKTLDNCEDWHCGQLQWFNEQLNELMLGSYRTLLQFASTHRLSESLRISELGLLTRKLHTYFSEDAHQIMRLNSLWSRSILEPYLSVIYSQQDEQYYLYRCSPEPRNFLEHSAVFHSKSKAKLLVWASLNGVANDKTRWYEVRQSKRKCIYLTHAANRLDGLVVHESTKVSKMALYQPWHFRKLVFLLNFNSDPTESWSGQDIMVDYMNSNVFSIGRRHQNMVDSIDVISLNSWGEWHCHHFEGEKSILDALSFVTPGMKRAIADVSVEVISCSSKLRSQTEHTVQDLLSRAVRLSRQAQSSATFGYALQVGKLRYGLFFNNRGMHYENLSDAKSFYQQLSQRKLLELPRPSLGDEPFAKLPAVIQDYAAKGAVQYFLRQRKQGLDVFILDEMNELNHYVQEGTDVNALVNQLSHYHTFEDPLLTKDNFNLPQFFKLDRVKGVLTAVPFGVTLEAS

>Q4RYP1_TETNG/701-790 PF00207

TWVWKLVKVGESGETDVALTVPDTITTWETEAFCVSPQGFGLAPRKEITVFQPFFLELSLPYSIVRGEQFELKATVFNYQTSCMMVSVKT

>PDXJ_CAMJE/1-257 PF03740

MLLGVNIDHIAVLRQARMVNDPDLLEAAFIVARHGDQITLHVREDRRHAQDFDLENIIKFCKSPVNLECALNDEILNLALKLKPHRVTLVPEKREELTTEGGLCLNHAKLKQSIEKLQNANIEVSLFINPSLEDIEKSKILKAQFIELHTGHYANLHNALFSNISHTAFALKELDQDKKTLQAQFEKELQNLELCAKKGLELGLKVAAGHGLNYKNVKPVVKIKEICELNIGQSIVARSVFTGLQNAILEMKELIKR

>Q8AYN9_CYPCA/782-869 PF00207

SWLWEEHTSKSGSVSITKIIPDSLTIWELKAVGVFSEGICVSEEKIQVSQDISVDVPLPYSMVRGEQIELRGSVYNQHLSKTEFRVTL

>Q6PCK2_XENLA/729-818 PF00207

TWIWELLPVGESGTTELHRSAPDTITDWNAGAFCMGPSGFGISPPTSLQVFQPFFVELTLPYSVVRGESFTLKASVFNYLRECMKVQTSL

>ARY2_CHICK/20-280 PF00797

DLETLTEIFQHHIQAVPFENLSIHCGETIELDLAATYDKIVRKKRGGWCMENNHLLSWALKTLGYNVTLLGAKVYIPEHDAYADDIDHLLLKVVLHDKSYIVDGGFGMAYQLWQPMELISGKDQPQTPGIFRFVEENGTWYLEKVKRKQYVPNHSDSAPHNVDKEVCRRVYLFTLQPRDIEEFRARNLHLQTAPDSLFVTKSICSLQTPDGVRALVGWKLTEIKYNYKDNMDLVEIRILADEEMEKTLKEKFNITLDKKFV

>O58642_PYRHO/203-577 PF01314

LMSVFEEIQESIISSGEFLRFKDWHVNFVPTILKLRMPYFGDYEREWEKAEEAAMKAKEFFEEHTIGRASCFSCPLRCWGLVNYDGETLPINLCQGTFPAATFILKIEDPELAWRVYRKCQSEGLDMMSATAVIAYASRLGKVKLGSEEILDFIDKLVKREGDGDIFAEGIKRASEHFGVPAVYVKGGMESWSSDIRPFVGSALIAAVADSGSVNRALYSFPEFYYYIKKEQAEMVATKFVGDVEAAYPWSYSSSKVKLAVIWENLHIIADSLGVCVIALLTTPLKLWARAYKAVTGNSITEEDLMTIAERIRTMERSFNLKYGKVKDDLSPRLFSEEPRLDREKLEEMKRVYYSLRGWDENGVPTSETLKKLGL

>Q8CVA0_OCEIH/100-312 PF03417

VFTSSNYMIRNYDYHPKTYEGRLVLFQPTDTGLTTIGPSQRITGRLDGMNQYGLVLGYNFTHRKQPGEGFICNMISRIVLETCSSVEEAVHLLQEIPHRYAFSYLVLDQHEETFIIEGSARGTSVRKGNLCTNHFENLTAENRNYLDDSYRRLLAMQNNSAKKLSAEEAFRMMNDTDRGVFSKQYKNWGGTIHTSGYLPAERKVWFSLGGDAD

>Q87VR7_PSESM/25-161 PF07696

AEHGAGWSTLLDEKARLSLDEIRSARYQNQFSPIELERVAAAERDSAVWLHYRLHPNQHEQLVRIFAPDLASADMYVMDGDQQIDHLRTGNAVPTEDQRLPSNDFLLPIPQSAASLDIYLRLVSSQKMRPSITLEPA

>Q97UK9_SULSO/5-184 PF02737

KIGVVGAGTMGHGIAEVSALANYNVSVVDISWDFLNRAKERIMESLNKFYEKGQIKEKPEDIMKRIEFSTSYDVMRDADFVIEAVPEIIELKRKVFETLDSITPSHTFLASNTSSIPISTIAEVTKRKEKIIGMHFFNPPPIMKLVEIVPSKYTSDETIEVTIDLAKKMNKIPVKLKVEV

>Q8CUR6_OCEIH/102-312 PF03417

TEADFLIRNYDNAPRSYEGRYVIYKPTDAGYATVGPTMQITGRTDGINEKGFAMGYNFVNRIKSDDGFVCNMIGRLLLENCASVEEAKQLLQELPHRHTFSYVLLDKNGYSVVAEVSPRNVKFREANMCTNHFEELTEENRYRTDESMERLNKISSQQSSVENPYEAYQLLNNLEKGVFSKKYDAWAGTLHTAAYLPKEMKAWIALGANRP

>O73768_CARAU/133-390 PF00002

LSTLKQLYTAGYATSLISLITAVIIFTCFRKFHCTRNYIHINLFVSFILRATAVFIKDAVLFSDETQNHCLMSTVACKTAVTFFQFCILTNYFWLLVEGLYLQTILTLTFVSQRKYFWWYILIGWGVPSVVLIVWVLTRQFYDNRGCWDDTDNMNIWWIIKGPITVSLIANIIIFLNVIRILVQKLKSPGVGGNDTGHFMRLAKSTLFLIPLFGMHYTLFAFLPENTGEIVRFYIELGLGSFQGFVVALLYCFLNGDV

>Q7W0C8_BORPE/7-181 PF02737

NLAVVGAGAMGSGIAALFASKGFDVVLIDPMEGALTRAAQVIERQLGVYAPDAIAPAMQRIRMDAGLEAACSAQLVIEAVPEKLALKRDIFARLDTLCDPQAIFATNTSGLSINDIAQAVTRRDRFVGTHFFTPADVIPLVEVVRNDDTSEQTVARVMDMLRAGGKRPVLVRKDI

>A1Z0M0_PSECR/2-85 PF06446

KTFSVAVAVAVVLAVICLQESSAVPANEEQELEQQIYFADPEMPVESCKMPYYMRENRQGSPARCRFCCRCCPRMSGCGICCRF

>Q12VC6_METBU/707-796 PF00207

TWIWMPEILTDDNGLATLDLNAPDSITKWRLHAVSSGPEGIGISEAGLTVFQDFFIDPDLPYAVIRGEEFPVQVQVYNYLDMPQNVKLTL

>Q98UJ6_CHICK/259-490 PF00198

SGKDKTEPITGFHKAMVKTMSAALKIPHFGYCDEIDLTHLVQLREELKPLAQSRGVKLSFMPFFIKAASLGLLQYPILNASLDEGCQNVTYKASHNIGVAMDTEQGLIVPNVKNVQVSSIFEIASELNRLQALGSASQLGTNDLTGGTFTLSNIGTIGGTYAKAVILPPEVAIGALGKIQVLPRFNGKGEVFKAQIMNVSWSADHRIIDGATMARFSNLWKSYLENPALMLL

>SCHA_STRHA/32-95 PF03992

IRDGTQAEVLDAYERMSDRVAAVPGHISDQLCQSLENPTQWLITSEWESAPEFLAWANSEEHLE

>Q3L2T4_MACEU/761-857 PF00207

SWLWTVERLTEKPDKHGISTKTHTVFLKDSITTWEILAVSLSDTKGICVADPYEITVMQDFFIDLRLPYSVVRNEQVEIRAILYNYGENPLKVRVEL

>PDXJ_NEIMB/1-237 PF03740

MLLGVNIDHIATVRNARGTTYPSPVEAALVAETHGADLITMHLREDRRHIKDADVFAVKNAIRTRLNLEMALTEEMLENALKVMPEDVCIVPEKRQEITTEGGLDVLAQQEKIAGFTKILTDAGIRVSLFIDADDRQIQAARDVGAPVVELHTGAYADARSHAEQIRQFERIQNGAHFAGDLGLVVNAGHGLTIHNVTPIAQILAIRELNIGHSLIAQALFLGLPEAVRQMKEAMFR

>O55225_MOUSE/1241-1384 PF05270

PYQLSSVAAGGTLVATKAVDSDIALVRAEDLAPGDISSFLLTAALYKAKAHDPDVVSLEAADRPNFFLHTTANGSIGLAKWQRDEAFHQHASFSLHRGTWQAGLVALESLAKPGSFLHSSGLELALRAYEHTEVFRGGALFRLL

>Q2C7Y9_9GAMM/9-568 PF06315

IAHTILQGFDAMYGRFLDVTAEAQERFEKQHWGLVHNALKKRINFYDHHVELVTNQIQIMLGERYANHDFLMAVKMAYQDLLLDYPRYDIAESFFNSVYCRIFKHRNINRDKLFVHTSQEDRVPPYPTSLTRHYRVKTTLSAMFERMLDDTPFTLTWQDKSRDTQLLLKHLQRDLGNKFDQDTTLELIREPFYRNKAAYLIGKLTFSNGDFCPFVVPILNNSSHEIYLDACICDVNDVSIIFGFARSYFMVYAPAPAALVRFLSQLIPNKTLAELYTAIGCQKHGKTEFYREFLAHLATTDDQFVIAPGIKGMVMSVFTLPSYDFVFKIIKDKFAPQKDISHNTVKEKYQLVKEHDRVGRMADTQEYRNFCFPRERFSEELIKELQSIAPSIVTLTDDTVMIEHLYMERRMVPFNLYIEKADDDELRYAINEYGEAIKQLAAANIFPGDMLFKNFGVTRHKRVVFYDYDEITYMTEMNFRKIPPPRYPEDELAAEPWYSVGINDVFPEEFRTFLLINPKVRTLFDELHSDLFEASYWQKLQQNITEENYPDVTPYPSEYR

>PEN3_ADECC/22-477 PF01686

LAPLVPPRYKGATEGRNSIRYSQLPPLFDTTKLYLIDNKSSDIQALNYQNDHSNFLTTVVQNANYTPMEASTQSIQLDERSRWGGDFRSILHMNMPNVTEYMFSNSFKAYLPATADASGKVLTYEWYTLTIPEGNYSEVMLLDLLNNAVVENYLAHGRQHNVKEEDIGLKFDTRNFYLGFDPETELVMPGFYTNEAFHPDIILSPGCAVDFTHSRLNNFLGIRKRLPYQEGFIIKWEDLQGGNIPALLDLEIYNPDTPGDNITPLLQDSKARSYHVGEDPSAGSTFTSYRSWFLAYNYGPVDGIKSKTVLVAPDITCGVEQIYWSLPDMAVDPVTFTSSHNPSSYPVVGTELLPLLPRSFYNGSSVYSQLLQESTAQTHVFNRFPENAILKRPPAPTIISISENVPALSNHGTLPLKNNIPGVQRVTITDARRRVCPYVYKSLGVVVPRVLSSKTF

>Q88DK1_PSEPK/25-161 PF07696

AEHDGGWTVLLDEQANLQLSDVRSERYRNQFSPLPLADLDAAPAGQALWLHYRLAPGDQEQLLRVFAPDLSGLDLYALEGEQLLRQLHHGRQAGNASPTLRGSDHVLPLPNSRQPLDIYLRLVSEHQLRPAISLEPA

>A5C6J8_VITVI/270-630 PF03081

IKKWIRAMKIIVRVYLASEKRLCDHILGDFGSINPICFVETSKVSMLRLLNFGEAVAIGQHLPEKLFSLLNMYEALADLLLHIDALFSEEAGASIRIDFHKLQRELGDAAGATFMEFETAIASYTSTSPFPGGGILHLTRYVMNYIKILTEYSNTLNLLLKDQNGEDPEPLIEAENAQGVPSQVVCPVAHHLRSIASLLESNLESRSKLYKDVSLQHIFLMNNIHYMVQKVKGSELRGFFGDEWIRKHMVKVQQRVTSYERTTWSSVLSLLREDGNSGSSSPWKMILKERCRGFSIAFEEVYKNQTAWFIPDPQLRDNLRILTSQKIIQAYRGFIGRNSENLSDKHIKYSADDLENYVHNL

>APLP2_RAT/42-204 PF02177

GTGFAVAEPQIAMFCGKLNMHVNIQTGKWEPDPTGTKSCLGTKEEVLQYCQEIYPELQITNVMEANQPVNIDSWCRRDKKQCRSHIVIPFKCLVGEFVSDVLLVPENCQFFHQERMEVCEKHQRWHTVVKEACLTEGMTLYSYGMLLPCGVDQFHGTEYVCCP

>GABR1_HUMAN/595-867 PF00003

VSVLSSLGIVLAVVCLSFNIYNSHVRYIQNSQPNLNNLTAVGCSLALAAVFPLGLDGYHIGRNQFPFVCQARLWLLGLGFSLGYGSMFTKIWWVHTVFTKKEEKKEWRKTLEPWKLYATVGLLVGMDVLTLAIWQIVDPLHRTIETFAKEEPKEDIDVSILPQLEHCSSRKMNTWLGIFYGYKGLLLLLGIFLAYETKSVSTEKINDHRAVGMAIYNVAVLCLITAPVTMILSSQQDAAFAFASLAIVFSSYITLVVLFVPKMRRLITRGEWQ

>GIPR_HUMAN/134-399 PF00002

LERLQVMYTVGYSLSLATLLLALLILSLFRRLHCTRNYIHINLFTSFMLRAAAILSRDRLLPRPGPYLGDQALALWNQALAACRTAQIVTQYCVGANYTWLLVEGVYLHSLLVLVGGSEEGHFRYYLLLGWGAPALFVIPWVIVRYLYENTQCWERNEVKAIWWIIRTPILMTILINFLIFIRILGILLSKLRTRQMRCRDYRLRLARSTLTLVPLLGVHEVVFAPVTEEQARGALRFAKLGFEIFLSSFQGFLVSVLYCFINKEV

>RIFF_AMYMD/22-251 PF00797

DLETLAKLQKSHLMAIPYSSLAYELRDAVNVVDLDEDDVFVTSIAEGQGGACYHLNRLFHRLLTELGYDVTPLAGSTAEGRETFGTDVEHMFNLVTLDGADWLVDVGYPGPTYVEPLAVSPAVQTQYGSQFRLVEQETGYALQRRGAVTRWSVVYTFTTQPRQWSDWKELEDNFRALVGDTTRTDTQETLCGRAFANGQVFLRQRRYLTVENGREQVRTITDDDEFRALV

>Q7D836_MYCTU/9-175 PF02737

RAAVVGAGLMGRRIAGVLASAGLDVAITDTNAEILHAAAVEAARVAGAGRGSVAAAADLAAAIPDADLVIEAVVENLAVKQELFERLATLAPDAVLATNTSVLPIGAVTERVEDGSRVIGTHFWNPPDLIPVVEVVPSARTAPDTADRVVALLTQVGKLPVRVGRDV

>LDHD_STAAU/4-330 PF00389

IMFFGTRDYEKEMALNWGKKNNVEVTTSKELLSSATVDQLKDYDGVTTMQFGKLENDVYPKLESYGIKQIAQRTAGFDMYDLDLAKKHNIVISNVPSYSPETIAEYSVSIALQLVRRFPDIERRVQAHDFTWQAEIMSKPVKNMTVAIIGTGRIGAATAKIYAGFGATITAYDAYPNKDLDFLTYKDSVKEAIKDADIISLHVPANKESYHLFDKAMFDHVKKGAILVNAARGAVINTPDLIAAVNDGTLLGAAIDTYENEAAYFTNDWTNKDIDDKTLLELIEHERILVTPHIAFFSDEAVQNLVEGGLNAALSVINTGTCETRLN

>Q9SW12_ARATH/32-260 PF03767

IFETQLKNINDNVNLHCTSWRFAAETNNLAPWKTIPAECADYVKDYLMGEGYVVDVERVSEEAKVYASSFESNGDGKDIWIFDIDETLLSNLPYYMEHGCGLEVFDHSKFDMWVEKGIAPAIAPSLKLYQKVIHLGYKVILLTGRRENHRVITVENLRNAGFHNWDKLILRSLDDRNKTATMYKSEKREEMVKEGYRIRGNSGDQWSDLLGSAMSERSFKLPNPMYYIP

>A7NH89_9CHLR/210-592 PF01314

LDQALESINRRIAASDVAAGFRQYGSLCYAQRAEELGALSARNGQSHTVSHASAISRTALAQRGRRESRGCEGCLLACHSAYIRKNGEPVAYPDLEALAGFGWRCGLSTPDAIILVNDLCLRLGLDVSETSAALAFVMECRERGLIHAGNLAWGDLESVVGALRRLGQRQEKRDILSLGVGEMQEVYYGSSAFAPQVRQLAFPALDPRALPEIALADATAPIGGDYRYAMIYEPFLVEPPVWLPTDPSNPHSIQGVVPRLIWHERFAAAVDAAGLCRRLALLAYQIAPAELNELLAATLGRSFTSVDVAKIGERIVTLERWLAVQYGAGRDTLPHRWTEEPLEDGPAAGKLPLLNDMLAEYYRRHGWDEQGRPSEARLTELGI

>A0HKN8_COMTE/14-594 PF06315

IAQALMHGYNQHYRMFSAEAARAKQRFEAMDWPGQQQAQRERIEFYDRQVRACIQRLEEEFSASLQSMAVWQQVKLHYIGLMVDHLQPELAETFFNSVTTKILHRTHFQNDFIFVRAAVSTEYLENTAPGAVPIYRAYYPGNLAQLEGTLQTLFEQLQLQCSFENLPRDVHLLAERIRERLAGLTLRANFQLQVLSSLFYRNKGAYLVGKIITGYTELPLAIPILHGEHEQLILHAALFGEDDLHALFSFARAYFMVEMEVPSAYVSFLRSLMPRKPKAEIYNALGLAKQGKTLFYRDFLHHLRHSSDKLRIAPGIKGLVMLVFDLPSFPYVFKLIKDRIASSKDVSRPQVKAKYQLVKKHDRGGRMADTMEYSLVAFPKERFSDELLAELQAQAPEQIEISDRDGDGQMEVIISHLYIERRLVPLNLFLMECLAEADTKPERRAAMERAILEYGNAIKDLVATNIFPGDMLWKNFGVTRGGKVVFYDYDEIEYLTDCNFRQVPQPRTEEEEMSGGVWYSVGPRDVFPETFGPFLLGHPAVRDIFMRHHADLLEADFWRAHQSRIRDGHFLDVFPYERSRF

>Q65VX6_MANSM/1-834 PF01295

MKYDLQFAKKQVDDLHRLRVERVLQGSTADFQHVFQLIALLLHLNHPALPGYVTDAPAGVAHFKLSDYQKNFLAQQFPTGFDFVRLEQESNAHQQEKTPIYGVYVMGSIASISQTAKSDLDTWVCHSPDLTPYALNKLQQKTQLLKIWAKKFNTDITLFLMDEFYFNHYRYSNTLSVENCGSAQHMLLLDEFYRSAIRLAGKPLLWLHLNVENEADYGKEVQRLQQTKQINRADWIDFGGLGAFSANEYFGASLWQLYKGIDSPYKSVLKIVLLESYSQEYPNAKLISMQFKQQLFNLKPVKEQCFDAYLAMLERVTEYLTKLKDEKRLDFIRRCFYIKVTETVRERPLAPWRAKILKNLTAQWGWSEETIKHLNRIHTWKIRSVRETHNKLIRVLMLSYRNLVNFARKHNVNASIAPQDISILTRKLYTAFEVLPGKVTLMNPQLALDLSEKNLTFIEVTEEHGVKPGWYVVNQMPSVVYPSQNRYIEYNPILIKLIAWTYFNGLLTSKTKVHISSTHVDIEKINQCITDLRVSFPVKASPPTDEELTHPCEIRSLAVMINLTKDPTPYSDINRTEIQQSDLFSLDGENESLIGSVDLLYRNKWNEIKTLHYEGDKAMLSALKVLSNKIHRGSGVPESVNVFCYNQYYQEEISELVVGLLNKCISIQLGTTQLPMSSVPRMTGKNWKLFFEEHDATLHQPQTEPVFISQVIAEQKQVKVKRNQPYKHLLNYPRQIDSFASEGFLQFFFEDNEDETFNVYILDENNRLEIYRQCDGSKEQKIREISQIYNLSGSDQNDNHYKIIKRDFNYPQFYQLKHQQKGILILPFSGSCMV

>ACEK_XANCP/17-577 PF06315

IGRAVYEAFQDYHAQFSQITARARQRFETRDWSGAREDAVARIALYDHYISECMLRLRAVLLGQAHDRALWMRARTHYAELLTGLIDQELYKTFYNTLTRRYFRTQGVDAQIEFIALDIEPTDAITVPVARHTYAVSPGRLTEMLVRVLGDYPFEVPYAHRTRCAAAIAVRLLDDLAHWGEHPVRSVELLETVFYRERRAYLVGRLFGEHRFSPCVIALVNDDAGLRAEAVLTRRSDVAQLFSNSRSYFQADLTTVGDAVVFLRSLLTHKPIDELYTMLGRAKQGKTERYRTFFRHFQAHPAEQLVHADGTPGMVMVVFTLPSYPLVFKLIRDRFAYPKTMSRAQVEGKYELVFQLDRIGRLLDAQPYRFLRFPKARFSPALLQDLQSSCAMSLSEDGDDVLIALCYVQRRLRPLNLYLREQLPAAAHAAALDYGQAIKDMARNNIFPGDMLLKNFGITRHQRAVFYDYDELCLITECTFRDWPTPTSYEEQMAAEPWFHVGPRDVFPERFALFMGLPSSQLEAVKHMHPELFDPQWWRDLQARLREDDYPDTPPYADAQK

>Q5ANM4_CANAL/12-486 PF07247

PLSVSENFFRCRTASGNYRNFVVTATYNQSLKKKLYVLVKALRKTILEYPILATTVKFDKTVNAFAYVPLKTINLSDILFFEGDEKWLTNGVINENYMKYCNETQFKLYEENVLFKLILVGDFHLSAIFEHTIGDGLVANYFHEVLLKNLAECESIEDAAFETQFGPLVFENIKAEDEIFNFSKDKLYIKNSLPPPIDPFLEDIDLDYSFGDPHFQDKIIPEGFPSKWKGRFDTVDTHDISFKLINFSKQETKIILSKCKEHKVGITSYIEVVHALTLQPIFGDNSFTTHRTAMTLRRHYTPDLAEPPYKKILSDSEYKIFGTSAHMGFVQNFPPIKVFSWDLVKKVNLDLSKAVKNKRALNQMKLWKDTGDLLDEKNMAFFDTQYNKPKADAVKISNLGFIELPEYVTKNDKKWVISDMVFSQDMAPYASEFMLSVVSTPIGGMNFVLSYYDYSFEDGNWENFDEFVTKLHDNM

>Q0ETM8_THEET/216-599 PF01314

VREVARNKTKMLRENGVTGEGLPTYGTAVLVNIINEHGIFPVANFQRAYTEHADKISGETMTREILVRKNPCYGCPIACGRWIRLKDGREVGGPEYETLWAFGADCEVYDINAIAEANYWCNEYGLDTISTGATIAAAMELYEKGYIKDDEIEKDGYSLKFGDAEAVVGWVKKIGAREGFGDKMAEGSYRLCESYGVPELSMSVKKQELPAYDPRGAQGHGLEYATSNRGGCHVRGYMISPEILGIPQKLDRFSLEGKAEWVKTFQDLTAVIDSLGLCLFTSFALGAQDYVDLVNAVCGTDYTAETLLEVGSRIYTLERLFNLKAGIDPKEDKLPKRLLEEPIPEGPSKGHVHKLSELLPKYYEVRGWDDNGYPTKETLQKLGI

>Q5V2K1_HALMA/208-573 PF01314

LAELREQYAKRYREGDTGQWLNASGTVETVDFANAIGALSTRGWEDGQFEGADSVGIEAVQELAAGREYDDADSPGGFRVQTEDGETVPRGATAMSLGAGLGINDFDAVATLGETCNRLGLDLISAGSAVAWAIKAGDAGLLAESLDYGSPDDARALLEEIVARESTLGDALADGVDAASARLGGDDLLPTVKAMELPAYDPRGARSMALAYATSDRGACHRRALPIEREGFDGNWGPERAAAAVICEQDQRSVLWCLVVDDFVGDAFDDLGAEWLDAVGLDTDGDLATVGERVWTLTRLFNVREGISRADDELPAKLQEPLDSGPNAGAAIDTESFDAMLDEYYRQRGWDADGHPTPETIERLGL

>Q8T770_BRAFL/749-839 PF00207

TWIFEDVQVDDGGQAIVPVTVPGSITTWVIQAVGISTANGMCVAEPFHMESFKKFFIHLQLPYSIIRGEQVAVRATIFNYDQQDLRVNVYM

>VIPR_CARAU/100-359 PF00002

FRSVKIGYTIGHSVSLISLTTAIVILCMSRKLHCTRNYIHMHLFVSFILKAIAVFVKDAVLYDVIQESDNCSTASVGCKAVIVFFQYCIMASFFWLLVEGLYLHALLAVSFFSERKYFWWYILIGWGGPTIFIMAWSFAKAYFNDVGCWDIIENSDLFWWIIKTPILASILMNFILFICIIRILRQKINCPDIGRNESNQYSRLAKSTLLLIPLFGINFIIFAFIPENIKTELRLVFDLILGSFQGFVVAVLYCFLNGEV

>O23808_PHAVU/28-255 PF03767

SMTTGYGDGASDTEVRCASWRLAVEAQNIFGFETIPQQCVDATANYIEGGQYRSDSKTVNQQIYFFARDRHVHENDVILFNIDGTALSNIPYYSQHGYGSEKFDSERYDEEFVNKGEAPALPETLKNYNKLVSLGYKIIFLSGRLKDKRAVTEANLKKAGYNTWEKLILKDPSNSAENVVEYKTAERAKLVQEGYRIVGNIGDQWNDLKGENRAIRSFKLPNPMYYTK

>Q14CJ0_HUMAN/772-859 PF00207

SWLWEVHLVPRRKQLQFALPDSLTTWEIQGIGISNTGICVADTVKAKVFKDVFLEMNIPYSVVRGEQIQLKGTVYNYRTSGMQFCVKM

>O32459_9ACTO/14-77 PF03992

APGDDPEAVVQAYKLVCEELRGTPGLLGSELLASTLDEGRFAVLSLWSDAARFQEWEQGPAHKG

>ACEK_DECAR/15-581 PF06315

IALAMIQGFNKHYTLFRQTSREAKTRFEQADWLGVHKAVKERIRFYDDRVDECVERLRNQFDAASIDDTTWQQVKLLYIGLLLNHKQPELAETFFNSVTTKILHRNYFHNDFIFVRPSISTENIEGDDTQTYRSYYAKEDGLRGTVLKIVKDFDWHRPFVDLEHDVEHVYHAVRHFLNGMPPREVNFQIQVLGSAFYRNKAAYIIGKAINGATEYPFTIPVLQNEAGQLYLDTILLDAWRIGLLFSLSRAYFMVDMEVPSGYVQFLRSILPAKPRSELYIMLGLGKQGKTMFFRDLIYHLHHSEDKFIMAPGIRGLVMLVFTLPSYPYVFKLIKDVFGSSKEMDRATVKKKFMMVKQVDRVGRMADTLEFSNVMFPLKRFNDEVLAELQQLAPSCFEVDGDQLIIKHLYIERRMEPLNIHLDRMERANNVERLEHVIREYGSAIREMAQANIFPGDMLWKNFGVTRFGRVVFYDYDEIEYMTDIKFRQIPPAPDFETEMSGEVWYAVSRNDVFPEEFATFLLTSPQVRKIFIKYHKDLLSPRFWLEAQEKIRSGYVEDFFPYPQELR

>Q95MM6_BOVIN/1-126 PF06214

MDPKGLLSSNVLLLFSLIIELSCRTGEGLTSSTKTILGQLGSSVLLPLASEEISRSMNKSIHILVTMAESPKDTVKKKIVSLDLRKGDSPRLNDGYEFHLENLSLRILKSRKEDEGWYFISLEENV

>A1RM97_SHESW/227-692 PF01314

LGIIGANNNGVVPSTPQSWAEYSAPISRWKARPGLKWGAADQEIELGEVPWNERNRIGFRTSMAESYIGVEYANKWMKRTGGCHSCPIRCFCELKVPELKSKYGRKTEHISNVCMGFLVPQYLMGTKNGTEAHAMAGALGAHILDDYGIWCNYGSISHLFKFLKTKDMFKHLLPEDEFNSIPWALWDNQDPAFLLDFYRRVAFKEGEISHMADGLYDLIKRWGLDGTNPALGYWDIHKESATKVFNKKTNAAVHHASETAGQVGTLINVVFNRDAQCHSHINIVNSGLPHDMTVAIAEKKWGEGAFDKVNWYTPINAAKIRFAKWSLVKNVLHDSLTLCNWMFPLLASPDKNRNYEGDSTIESQMFSLVTGIETTEAELDFKAERVLHLHRALTVLQMNESNMREEHDVLSDWVYDLNPDKNFGDQGTIKMDRADMQKALDMFYEAFGWDIQTGAPTRATLNKFGL

>CD109_HUMAN/693-784 PF00207

TWIWLDTNMGYRIYQEFEVTVPDSITSWVATGFVISEDLGLGLTTTPVELQAFQPFFIFLNLPYSVIRGEEFALEITIFNYLKDATEVKVII

>O97019_HALRO/778-868 PF00207

SWISEEKKIGDDGTLVFRENAPDSITIYEIAAFGMSTVSSFCIAKPKEVKVHKNVFIQLYLPYSVRVREQAVIRFAVFNYGPQEIEVTVKV

>O31302_CORAM/10-89 PF01648

VGVDLVHIPGFAEQLSRPGSTFEQVFSPLERRHAQTRRDAAADATNSSLAGSRTEHLAGRWAAKEAFIKAWSQAIYGKPP

>Q99ZX6_STRP1/235-469 PF00198

PEGVEHKPMSAMRKAISKGMTNSYLTAPTFTLNYDIDMTEMIALRKKLIDPIMAKTGLKVSFTDLIGMAVVKTLMKPEHEYMNASLINDANDIELHRFVNLGIAVGLDDGLIVPVIHGANKMCLSDFVLASKDVIKKAQTGKLKAAEMSGSTFSITNLGMFGTKTFNPIINQPNSAILGVGATIPTPTVVDGEIVSRPIMAMCLTIDHRLVDGMNGAKFMVDLKKLMENPFELLI

>Q9HKW7_THEAC/5-187 PF02737

TTAVVGSGVMGQGIAQVFARSGYPVTIIDVRDDILANAVRSIKEGRYGLMNLVKKGTMTESEVDKIMGKIRTSTSYGSLSDADIVVEAVPENLDLKRKVFIDIEKNVSENAIIASNTSGITIAEIAQDLKKKDRAIGMHWFNPAGIMKLIEVVRAKMTSEDTISTVVDFSRRIGKTPVVVADV

>CO3_ONCMY/747-846 PF00207

SWMWEDTNLPECPAQNKHCESTSVIRNNFLKDSITTWQITAISLSKTHGICVADPFEMIVLKEFFIDLKLPYSAVRNEQLEVKAILHNYSEDPIIVRVEL

>CO3_EPTBU/736-829 PF00207

SFMWEIIKLSRSAENGKSRITKKMPDSITTWDIQAVEVSQSKGLCVGPSLELTVFKQFFLKVHTPYALKQYEQVELRVVIYNYMNQDVKGEIQV

>SLAF1_CANFA/1-126 PF06214

MDSRGFLSLRCLLVLALASKLSCGTGESLMNCPEVPGKLGSSLQLSLASEGISKRMNKSIHILVTRAESPGNSIKKKIVSLDLPEGGSPRYLENGYKFHLENLTLRILESRRENEGWYFMTLEENF

>Q981H5_RHILO/99-326 PF03417

VQTRDGALIAQNWDGPLGSDVEQALFIHVGTSGFEFATVAAVGALGWVGFNRAGFGFVNNDLVLKSRCDGIPSQIVRRVFLGCPDVNAAVEAAKSLPHMAGRAYLFGDASNKIASIEVSARHGVSVCRSGSFLAHTNHALNDQIRADEDAELLARQYPSSQKRLEVLKMREAGCADACEVMDILRDRTNAPDSVCKQTSLREPTQTAFSVVMQCRNRTLFLVRGMPSA

>Q3ABB7_CARHZ/216-594 PF01314

LKELAKQLTAAYKKEMELFTQYGTLGYLDIGYYFGDTPVKYFTKGLFPIDKVNARKFREEYSIHPRACWGCPIACGRELNFKGRIVDGPEYETAVTYGPLLENFDMDTIIEANDIANRYGFDTISTGVSLAFLMYLKEENLLPENLKAEVPDFGDSKGILEALRLTGENSGIGQLIGKGVARVAEELGLSQDYAAAVRRLEIPMHEPRAFTGQALSYATGPRGACHLRGSFFEVDLGIAKDPDFGVVPGPRFNLDGRVEAVVKFQSLRELDNCLVKCSFAFTPLKAVVGALSYTTGVAWTPEKVVEAANNSITIKRAISFNLGSSPADDRLPGHVKKPLAEGGAFGNVPEIEKYLPEFYQLRGWNSDGTISREFLARLL

>Q8FGB4_ECOL6/3-184 PF02737

NVAVIGAGVMGTGVAHNMAQYGISTNVVDISQSQLDKCRQMIEANLRLYNFHPQHKKKTHSTAEIMENIRFTTELDDIVECDLVIENITEDIEKKNALYTRMNTICGASTVFGVNTSAISITALSKLMRHPENVVGVHFMNPVPLMHTVELIRGVHTAERTLNIFHHLFAQLNKTGIVVNDS

>Q84CL9_9ACTO/25-261 PF00797

TLATLSALHRAHTVAIPFENLDVALGRPVPLDVKSIQRKLVGQSRGGYCYEHNTLFAAVVERLGFEVAARGARNRTRGAVLTAVTHALLLVTVEGERWLCDTGFGSQGPLEPVPLRDGAVVEQGGWTFGITSEAEGIHVLSSLRPEGWVGLYSFAPQTLYPADFAVMNHYSSSHPLSKFVGQVVAQRRAPGVRRALVRDELTTVRTDGSSEQVTVPAEELGRVLTEVFGIVLEDDEV

>Q8FH59_ECOL6/253-713 PF01314

TELIGANNNHVVPSTPQSWAEYSDPKSRWTARKGLFWGAAEGGPIETGEIPPGNQNTVGFRTYKSVFDLGPAAEKYTVKMSGCHSCPIRCMTQMNIPRVKEFGVPSTGGNTCVANFVHTTIFPNGPKDFEDKDDGRVIGNLVGLNLFDDYGLWCNYGQLHRDFTYCYSKGVFKRVLPAEEYAEIRWDQLEAGDVNFIKDFYYRLAHRVGELSHLADGSYAIAERWNLGEEYWGYAKNKLWSPFGYPVHHANEASAQVGSIVNCMFNRDCMTHTHINFIGSGLPLKLQREVAKELFGSEDAYDETKNYTPINDAKIKYAKWSLLRVCLHNAVTLCNWVWPMTVSPLKSRNYRGDLALEAKFFKAITGEDMTQEKLDLAAERIFTLHRAYTVKLMQTKDMRNEHDLICSWVFDKDPQIPVFTEGTDKMDRDDMHASLTMFYKEMGWDPQLGCPTRETLQRLGL

>Q97AK1_THEVO/217-598 PF01314

YKIVLPKMLKKIKENPVTSQGLTQFGTEVLVNIINKSGIYSNKNLTESGDDPLADDVSGETLAETYLLHNQPCFACPIGCGRVVRYNERESEGPEYESTWALGPNTGIHDLYTIIAANDNADRLGYDTISAGLTMSAAMELYENGKIPDKDVGPTKPKFGNQSALLEMSVKLAYRKDFGSKLAEGSKKLSEMYGDPSVSMSVKGQEIAAYDPRGVWGMALEYATSNIGGSHMRAYTISNEILGVEPVSDPLKLEGKAELVKYVQDFTEVMDCSGLCQFPSFALNLDDYLELVNAVTGFNYTKEEIMKAAERVWNLERLFNLKAGIKPEDDKLPERFLKDPIPKGPKKGNTVPFEKLIKDYYKVRGWTENGYPTPEKLKELGL

>Q20406_CAEEL/129-319 PF02485

LAYGLVVYKTIVQVLTQMSLFYQPQHMFCITVDDQSPNEYKSVIQALPSCFPNMHVFIGEPSQWGSFGILKNVYTCFNWLSKSKQKWKYYQYLSGTDLPIRTNLEMVRIFKALNGSMNTDVSTFEVDRYKNMEGVLPPMPVYKSSMSVVVPREGADYLISSPRVQKLLKYLSKTWIPDESFWSTVLGSPAL

>Q076D6_CHICK/727-815 PF00207

SWLWRRIHVAGTARLSVLLPDSITTWEIQAVAIVPGHGLCVAEPQRVTVTQDVRVALRLPPSIRPLEQMQLQPLIHSRLPRSINVTVTL

>Q930L9_RHIME/12-75 PF03992

AKPGRSGELGDELLQLVTPSRNEEGCLRYEIHQSNDVPDVWMVLEDWRHASDFKLHMNTPYVQA

>Q18XF6_DESHD/222-584 PF01314

MEKILKDSRTTIRRTYDLMRWGGSMTNMPHSDEGHLDVANYREGHWPEINKIGGLAYERLCRARSRGCFGCPMGCMPLGVVREGEFSGNVVCPDLEAAATMGSGILVNDLNAMVYLTRWADEYGFDSTSLGNITGFAMECYENGLLPEAELEGIDLSWGNVEGVLALWNKILKREGIGALFAEGVKKAAAKIGGGSEHYAMQVKGREFACFTPQADHKQGLQYAVSDKGPAHHFGGKADHTQQRTWADLLTACTWQRRMIKPEVYLKLLNAVTDWQLQAGDWPLTARRILLLARAYNIREGLLPLRDDVPPERVHVDKLTTGIGAGQVYPREQFSMDRKAWYGELGCDAGGFPTPEALKEHGL

>LYS5_SCHPO/123-188 PF01648

IGVDIVECKPLAFEASWMEDFMSVFTPCEWKLIKSSISSIDVFFLLWTCKEAILKALGIGLSGNPL

>Q5SGB5_LAMJA/746-837 PF00207

SWGWNKYKIPASGRHPQIRLQLPDTITTWNMQAVSISKTRGVCLADPLLLVSTKDFFIKLHLPYSVKRGEQTEIRVILYNYMEESLTILTEM

>Q9KTV7_VIBCH/137-199 PF01648

LGIDIEHRLAHQTASEVQAIIGTAQEWALLAQQFDLASAVTLLFSAKESLFKALFPQVHLYLD

>Q0LHA9_HERAU/215-590 PF01314

FLSSFKPITERIDRDPLATAIRDYGSSYYVDLAANHGAITGRNGQDPEPSVMPTTGDYAGEIYDRGCPRCPLPCYHDFATTNQPRPEIEAIVGFGARCGLQSPAAIIEANQRCLRYGLDPNATANAIAFLMECRQRELTRQYDLQWGDEAAILAAIDAIATKQGIGGLLSLGVYEMSQVFYGSEQFAPLANNLTISPLDPRAAQGWALHLATSSIGGDARGAMPWYEWLDTIPQWLKGNDDHQPNVVNGKPERLIWHERFVAGLDSAGICRRLAALAYQITPKELATILSADIGKSVSPTDLAKVGERIITVERLLALQWGHSDDLSTRWQQQPLTSGIAANQIPTLDTLLTKYYALHGWDSDGIPTNARLAELSI

>Q76KI4_TRISC/754-844 PF00207

SWLWKTVEVLSAGDHKIRNYMPDSITTWEIQAVGMFANKGFCVAEPKMIKVFRPFFISVKLPYSVKRNEQLDLTVILYNYLHEDLEVAVYM

>Q5JEI3_PYRKO/217-622 PF01314

LRELIKKWALIFKDHPATKADMEYGSGEFLDWMNRERGTFPVRNWQMGFFKKAYEKAKEEGREHIGIDPYFWAPKYRAGRRPCPLCNKPCSQYVKVESKKWGTFMVDGPEYETLYSFGGVLEIDDFETVAYLNYLADQYGLDTISAGVTIAWAMEAYERGLLTKEDTDGIELTFGNGEAAVEALRKMAYREGNLGKLLADGVKRASERLGKESWKFAMHVKGMEPPAYDVRGIKGMALAFAVSVRGADHLTAGAYGTELVGRWWKFDGVDRTKGENKGFEIAFHENLMAIYDATGTCKFSRHMYFLEGFPELIEAVTGMNIGEAELMVIGERIMNIARAFNVREGFSRKDDTLPYRIMWEPIPEGVSKGLHVPPWELDRMLDEYYQARGWSRDGIPTKAKLIALDL

>Q8T398_CIOIN/737-827 PF00207

TWLWDEQISGADGSATFNTTAPDTITSWIFSAFSVSDQHGLGVSEQHKVTVFRNFFITLNLPVRVIRGELIIVQAIVFNYLSTEVDAVLTL

>O74288_EMENI/357-506 PF05270

VSLKVTTSGYDTRYIAHTGSTINTQVVSSSSSSTLKQQASWTVRTGLASTAAANGCVSFESVDTPGSYIRHSNFALLLNANDGTKLFSEDATFCPQDSFNDDGTNSIRSWNYPTRYWRHYENVLYVASNGGVNTFDAATAFTDDVSWVVA

>CRFR1_RAT/116-370 PF00002

YHVAVIINYLGHCISLVALLVAFVLFLRLRSIRCLRNIIHWNLISAFILRNATWFVVQLTVSPEVHQSNVAWCRLVTAAYNYFHVTNFFWMFGEGCYLHTAIVLTYSTDRLRKWMFVCIGWGVPFPIIVAWAIGKLHYDNEKCWFGKRPGVYTDYIYQGPMILVLLINFIFLFNIVRILMTKLRASTTSETIQYRKAVKATLVLLPLLGITYMLFFVNPGEDEVSRVVFIYFNSFLESFQGFFVSVFYCFLNSEV

>Q6U2I5_HUMAN/90-178 PF00207

NWLWRVETVDRFQILTLWLPDSLTTWEIHGLSLSKTKGLCVATPVQLRVFREFHLHLRLPMSVRRFEQLELRPVLYNYLDKNLTVSVHV

>Q62BJ7_BURMA/23-263 PF00797

TLDVLRQLQLLHPQSIPFENLNPFTGARVALELESVVDKLVGQRRGGYCFEQNKLFYTVLVQLGFRVTPLIARVRWQRPPEQSTPQTHMLLRIDLDGDTWFADVGFGSTTLTAPLRHDPNVAQPTPHGTFRVVDAPVAGEFEIECETPNGWHPLYRFSLKPVEWVDYEVANWYTSRHPDSFFTHDFIACRILPDARALLFNDALTLRTADGAAHTTRLTDADAWAACVRERFGLDLDGFDA

>PDXJ_PSEAE/7-243 PF03740

ILLGVNIDHVATLRQARGTRYPDPVKAALDAEEAGADGITVHLREDRRHIQERDVRVLKEVLQTRMNFEMGVTEEMLAFAEEIRPAHSCLVPERREELTTEGGLDVAGQEQRIRDAVRRLAAVGSEVSLFIDPDPRQIEASARVGAPAIELHTGRYADAEDPEEQARELQRVREGVALGRSLGLIVNAGHGLHYHNVEPVAAIDGINELNIGHAIVAHALFVGFRQAVAEMKALMLA

>CYAA_PASMU/1-838 PF01295

MNYDLFSAQKKVEYLDKLRIERALSGSSGEFQHVFQLLTLLLHINHPNLPGYVADAPVGIADFVISPYQKQYLLTTVPSLEANQSLLPSFSYRSTNAILGVYVMGSIASISQTPKSDLDTWVCHRDDLSTKEKEALQRKTHLLKNWAKQFNIEINFYLMDQKRFRCFRYAEPLTAENCGSAQYMLLLDEFYRSAIRLAGKPLLWLHLLIEQEENYESEVERLVRTQQICLDDWVDFGGLGQLSANEYFGASLWQLYKGIDAPYKSVIKILLLETYSSEYPNTYLIARQFKEELLTGKLNPSHHFDPYLAMLQRATRYLTKHNELKRLGFVRRSVYLKATEGMCWQDPNATNNWRLQHLQKLIQEWDWSDALIEELNQRANWKIKQVKKAHNSLIKFLMLSYRNLVAFARKHKVNSSIMPQDISVLTRKLYTAFEELPGKITLLNPQISLNLSEKNLLFFEVKGSKTFKDGWYVVNQTPSVAGFVQKRYTEYSESLNKLVAWAYFNRILTANTDLHIISPNVSLTTLRHFVTDLRLSFPVTVSSVTNEDLTHACEIRSLIVAVNLTVDPTKKITQVKSRIQASDLFSFGPKEESLVGSIDITYRNLWNEIRTLHFEGPNAILLALKVLSNKIHRGAPSPKLIQVFSYSHRYRRTLSNIVTALINRCISIQIGDALPPQNNLLRVAGKNWQFFFEERGISLQEIHSNEELEATGFDTALQTEVEEKESALPDTSRTYPPEIDHFASEGFLQFFFEDNSDGSFNVYILDEANRIEIYRNCDGQKEKKILEINHIYQSSGLDENNNPYKIVQRDFNYPQFYQLLLQENGVKIVPFHSRLAMS

>Q9ZBJ4_STRCO/11-73 PF03992

TRPGFREEVVSLLLSAADGLRDAGCELYVVGLSEDDETTIWVTEVWRTREDHDASLELPAAKD

>PEN3_ADEGX/48-524 PF01686

SELYMPLQRVMAPTGGRNSIKYRDYTPCRNTTKLFYVDNKASDIDTYNKDANHSNFRTTVIHNQDLDADTAATESIQLDNRSCWGGDLKTAVRTNCPNVSSFFQSNSVRVRMMWKRDPPTSTAPPSAVGSGYSVPGAQYKWYDLTVPEGNYALCELIDLLNEGIVQLYLSEGRQNNVQKSDIGVKFDTRNFGLLRDPVTGLVTPGTYVYKGYHPDIVLLPGCAIDFTYSRLSLLLGIGKREPYSKGFVITYEDLQGGDIPALLDLDSVDVNDADGEVIELDNAAPLLHDSAGVSYNVIYDQVTGKPVTAYRSWMLAYNVPNSQANQTTLLTVPDMAGGIGAMYTSLPDTFIAPTGFKEDNTTNLCPVVGMNLFPTYNKIYYQAASTYVQRLENSCQSATAAFNRFPENEILKQAPPMNVSSVCDNQPAVVQQGVLPVKSSLPGLQRVLITDDQRRPIPYVYKSIATVQPTVLSSATL

>Q72M90_LEPIC/6-182 PF02737

TVTVLGANGTMGAGSAAIVASFGKAKVHMLARDTNKAKEGIEKAIGSVKTDTIRPRLIPGSYDADLEKAVSESDWVFELVAESYEVKEPINKRIASSRRPGTIVSTVSSGLSIERLSKAFDEDGQKHYFGTHFFNPPYKMILCELVSHKGSDKKVLKQLGEYLEKVLGRAVVYTNDT

>Q7BQ74_BACCE/4-184 PF02737

KIGIVGAGTMGIGMAVDLVLHGLETVLIDVTEEQLEKAEEKILETVRFAPLINKAFPRMHKEEVLSLISSSTNLDEVAECDYIVENVPENWQIKEPIYRRLDEICKKDTIFGVNTSCISITKVGGVTKRPDKIIGMHFMNPVYMKPSIEVIRGHLTSDETVEKAQSFLKQLDKDAIVVNDQ

>Q9XA62_STRCO/236-467 PF00198

TTRETRVPVKGVRKATAAAMVGSAFTAPHVTEFVTVDVTRTMKLVEELKQDKEYTGLRVNPLLLIAKALLVAIRRNPDVNASWDEAAQEIVVKHYVNLGIAAATPRGLIVPNIKDAHAQTLPQLAGSLGELVSTAREGKTSPAAMQGGTVTITNVGVFGVDTGTPILNPGESAILAVGAIKLQPWVHKGKVKPRQVTTLALSFDHRLVDGELGSKVLADVAAVLEQPKRLIS

>CD109_MOUSE/695-786 PF00207

TWIWLDAYMGSKIYEEFEVTVPDSITSWVASAFVISEDLGFGLTTVPAELQAFQPFFLFLNLPYSVIRGEEFALEVSIVNYLKDTIKVVILI

>GRM_DROME/630-881 PF00003

PMAIAIFGIALTSIVIVLFAKNHDTPLVRASGRELSYTLLFGILVCYCNTFALIAKPTIGSCVLQRFGIGVGFSIIYSALLTKTNRISRIFHSASKSAQRLKYISPQSQVVITTSLIAIQVLITMIWMVVEPPGTRFYYPDRREVILKCKIQDMSFLFSQLYNMILITICTIYAIKTRKIPENFNESKFIGFTMYTTCIIWLAFVPIYFGTGNSYEVQTTTLCISISLSASVALVCLYSPKVYILVFHPDKN

>YHJ3_YEAST/5-238 PF00696

YTIVIKLGSSSLVDESTKEPKLSIMTLIVETVTNLKRMGHKVIIVSSGGIAVGLDALNIPHKPKQLSEVQAIAAVGQGRLIARWNMLFSQYGEQTAQILLTRNDILRWNQYNNARNTINELLAMGVIPIVNENDTLSISEIEFGDNDTLSAITAALVGADFLFLLTDVDCLYTDNPRTNPDARPIVLVPELSEGLPGVNTSSGSGSEVGTGGMRTKLIAADLASNAGIETIVMK

>HEPC_CANFA/1-85 PF06446

MALSTRIQAACLLLLLLASVASVSVLPHQTGQLTDLRAQDTAGAEAGLQPTLQLRRLRRRDTHFPICIFCCGCCKTPKCGLCCKT

>Q9KQB4_VIBCH/172-402 PF00198

GRSEKRVPMTRLRKRIAERLLEAKNNTAMLTTFNEVNMKPIMDMRKQYQDVFEKRHGIRLGFMSFYVKAVTEALKRYPEVNASIDGDDLVYHNYFDVSIAVSTPRGLVTPVLKNCDTLSLAQIEKGIKELAEKGRDGKLTVDELTGGNFTITNGGVFGSLMSTPIINPPQAAILGMHKIQDRAMVVDGKIEILPMMYLALSYDHRSIDGRESVGFLVTVKELLEDPARLLL

>Q7XPT9_ORYSJ/273-640 PF03081

IAEWTQFSRITVKLLFGAERILCDQVFEGKYTWKDHCFAEVTAKSLSILLSFGDAVVQSQILPDKLYILLDMYKATLELQSKVDAIFEGNACSENQKSALTLTKSLAQTAKKTIGDFMEYILNHSVTSTTVDGAVHYMTSYVTDYIKFLFDYQSSIKQIFGDPCVEDEKDTDVVSQIVGAIHALETNLAMKAKQYKDLALGHLFLMNNIHYIVKYIGRSELKDLLGADWIERQRRIVQQHATRYRRVAWLKVLECLSTQGLTSSVGSSIDVTQGSFRNIKNSTTSRSVIKERLKCFNMRFEEICQKQMNWGVPDRDLRDSLILMIAEILLPAYRSFLKHFGPLVENSHSALKYMKYTPESLEQALGNL

>Q7JKM0_CAEEL/750-840 PF00207

TWIWSDLNSTSGEVEMEIEAPDTITSWVASTFAINEENGLGVAPTTSKLRVFRPFFIQLNLPYAVRRGEKFALLVLVFNYMEKEQDVTVTL

>PPA1_SOLLC/27-255 PF03767

PLIIEYPEKQLRDELKCTTWRFVVETNNLSPWKTIPEECADYVKEYMVGPGYKMEIDRVSDEAGEYAKSVDLGDDGRDVWIFDVDETLLSNLPYYSDHRYGLEVFDDVEFDKWVENGTAPALGSSLKLYQEVLKLGFKVFLLTGRSERHRSVTVENLMNAGFHDWHKLILRGSDDHGKTATTYKSERRNAMVEEGFRIVGNSGDQWSDLLGSSMSYRSFKLPNPMYYIL

>Q3LA38_SCOMX/2-90 PF06446

KAFSIAVAVTLVPAFVCILESSAVPFPGVQELEEAGSNDTPAAAHQETSMEPWTVPSHIRQKRQSHISLCRWCCNCCKAYKGCGFCCRF

>Q99ZR9_STRP1/5-226 PF03767

FTSILFTVSFCGIIALPVEASGPKVPYTQEGITAISNQATVKLISIADIASSLEGQKPITVSFDIDDTLLFTSQYFQYGKEYITPGSFDFLHKQKFWDLVAKRGDQDSIPKEYAKQLIAMHQKRGDKIVFITGRTRGSMYKKGEIDKTAKSLAKDFKLDKPIAINYTGDKAVKPYQYDKTYYIKKNGSQIHYGDSDEDINAAKEAGARPIRILRAPNSTNLP

>O06159_MYCTU/163-391 PF00198

AGPDVRPVHGVHARMAEKMTLSHKEIPTAKASVEVICAELLRLRDRFVSAAPEITPFALTLRLLVIALKHNVILNSTWVDSGEGPQVHVHRGVHLGFGAATERGLLVPVVTDAQDKNTRELASRVAELITGAREGTLTPAELRGSTFTVSNFGALGVDDGVPVINHPEAAILGLGAIKPRPVVVGGEVVARPTMTLTCVFDHRVVDGAQVAQFMCELRDLIESPETALL

>Q9X7I0_PORGI/1-243 PF03767

MNSRHLTITIIAGLSLFVLTLGGCSVTQQDPQWTLGGKLFTSAWIQRSAEYQALCIQAYNIATERVDALPAERKQGDRPYAIVTDIDETILDNTPNSVYQALRGKDYDEETWGKWCAQADADTLAGALSFFLHAANKGIEVFYVTNRRDNLREQALQNLQRYGFPFADEEHLLTTHGPSDKEPRRLKIQEQYEIVLLIGDNLGDFHHFFNTKEESGRKQALGLTAGEFGRHFIMLPNPNYGSW

>ODB2_PSEPU/192-422 PF00198

TDSEQVPVIGLRRKIAQRMQDAKRRVAHFSYVEEIDVTALEALRQQLNSKHGDSRGKLTLLPFLVRALVVALRDFPQINATYDDEAQIITRHGAVHVGIATQGDNGLMVPVLRHAEAGSLWANAGEISRLANAARNNKASREELSGSTITLTSLGALGGIVSTPVVNTPEVAIVGVNRMVERPVVIDGQIVVRKMMNLSSSFDHRVVDGMDAALFIQAVRGLLEQPACLFV

>CRFR2_XENLA/115-368 PF00002

YKIALIINYLGHCISILALVIAFLLFLCLRSIRCLRNIIHWNLITTFILRNIMWFLLQMIDHNIHESNEVWCRCITTIYNYFVVTNFFWMFVEGCYLHTAIVMTYSTDKLRKWVFLFIGWCIPSPIIVTWAICKLFYENEQCWIGKEPGKYIDYIYQGRVILVLLINFVFLFNIVRILMTKLRASTTSETIQYRKAVKATLVLLPLLGITYMLFFVNPGEDDVSQIVFIYFNSFLQSFQGFFVSVFYCFLNGEV

>Q0ZM39_SCYSE/789-877 PF00207

TWLWNIVVPENGTRKVDQTLPDTITQWVGKAVCVHPQVGVGLSERESIATFTSFFVDLTLPPTVKRKETLPVKSVFNYHDKDLPITITL

>ADPGK_HUMAN/68-492 PF04587

PVRRWRRVAVGVNACVDVVLSGVKLLQALGLSPGNGKDHSILHSRNDLEEAFIHFMGKGAAAERFFSDKETFHDIAQVASEFPGAQHYVGGNAALIGQKFAANSDLKVLLCGPVGPKLHELLDDNVFVPPESLQEVDEFHLILEYQAGEEWGQLKAPHANRFIFSHDLSNGAMNMLEVFVSSLEEFQPDLVVLSGLHMMEGQSKELQRKRLLEVVTSISDIPTGIPVHLELASMTNRELMSSIVHQQVFPAVTSLGLNEQELLFLTQSASGPHSSLSSWNGVPDVGMVSDILFWILKEHGRSKSRASDLTRIHFHTLVYHILATVDGHWANQLAAVAAGARVAGTQACATETIDTSRVSLRAPQEFMTSHSEAGSRIVLNPNKPVVEWHREGISFHFTPVLVCKDPIRTVGLGDAISAEGLFYSE

>A1HP75_9FIRM/218-610 PF01314

LAAAIDAAVPRVRERTRLMQEYGTAGGIIGAERIGDLPLKNWTQGTWDGVEKISGQAMAETIVTGRYHCASCPIGCGRQVRVERSPYGRVEGAGPEYETVGMFGGACLVSDLAAIAMANELCNRYGLDTISTGAVIAFAMELYEHGILTDRDLGGCPAPVWGDGRAVVGLIQAIASQEGIGRLLGSGVRRAAELIGGRAGEFAMHVKGLELPAHDPRAFYSLALGYATSNRGACHLQGASYFFEKAAVLPEAGINEVLDRFRTDDQGLIQARLQDTMCLMDSLKLCKFLFYGGIDLTTVTGWLNHLTGWDCTVAELLTTGERIFNLKRLYNVKCGVSRRDDVLPERILRQPRPDGGAAGALPPLDAMLREYYRVRGWDEVGRPRPETLARLGI

>A2QCL9_ASPNG/266-651 PF03081

GTNGIGIYSNALESFIYAEHDILVRVFTGDQRGLALQATCRSALTEYAQTLRELNQYIRANFMTDCFLAFEIIEIVTAMSYRVESKTGELKSLFIEALRPVRETAKSSLSELLEETKRKAAGIAMLPPDGGSVPLVNEVMSSLTTLTGYSGPLASILTSLGDGNWRSTTNAAPTAPLDVSPDSMTLLSHFILDMIEALMIALEARGRALHRSKAVQGVFLSNVFCTVDRSIRQSSELARYLGSPDSIARIDTFRKRATSTYLDAWKETSHYLLDVQYTSRGSGGSARPASGGVVDSAAIVKSLSSKDKDAIKDKFKAFNASFDELVARHKALYMEREVRGVLAREVQAVLEPLYARFWDRYHEIDKGRGKYVKYDKGSLSAQLAAL

>Q2TLD4_9ADEN/1-447 PF01686

MESSNTATRIFAPTEGRNSIIYSNLPPVQDTTKIFYIDNKAIDIESYNQEKDHSNYYTNIIQTQNISTIDSSIQQIQLDERSRWGGELHTSLVTSVMNCTKHFNSDRCLVKIQTIKSPPTFEWKELKIPEGNYVLNEFIDLLNEGITSLYLQYGRQQGVLEEDIGIKFDTRNFEIGKDPTTNLVTPGKYLFKGYHADIILLPGWAIDFSFSRLGNILGIRKRETYKAGFLIEYDDLTNGNIPPLLDVANYKSTSQAKPLLQDPSGRSYHVMDSDSNRPVTAYRSFVLSYNNEGAAKLKFLMCMSDITGGLNQLYWCLPDSYKPPVSFKQETQVDKLPVVGMQLFPFVSKSVYSGAAVYTQLIEQQTNLTQIFNRFHDNEILKQAPYVNQVLLAENVPINVNQGTIPIFSTLPGVQRVVVEDDRRRTVPYVTKSLATVYPKVLSSKTL

>WH42_STRCO/54-117 PF03992

VHDGMQQEFLDAYERIRDRVAAVPGHVSDQLCQSLENPTQWLLTSEWESAAPFLAWVNSDEHLD

>NAT_MYCTU/21-260 PF00797

TLDVLQDLVTVHSRTIPFENLDPLLGVPVDDLSPQALADKLVLRRRGGYCFEHNGLMGYVLAELGYRVRRFAARVVWKLAPDAPLPPQTHTLLGVTFPGSGGCYLVDVGFGGQTPTSPLRLETGAVQPTTHEPYRLEDRVDGFVLQAMVRDTWQTLYEFTTQTRPQIDLKVASWYASTHPASKFVTGLTAAVITDDARWNLSGRDLAVHRAGGTEKIRLADAAAVVDTLSERFGINVADI

>Q9A6W1_CAUCR/122-182 PF03992

AAPGKRDELLAILAEGTADMPGCLSYVLATDPANPEAIWITEVWTDKAAHAGSLKLPAVQA

>ACEK_RHOPA/48-607 PF06315

LARVVLSAFDNYYAVSRRIPALAQAAFEARDWPVTVRLSKIRIGLYTACIDQLVPLLKAGLPELTTDEQLWPTAEAELLAAIEGRYEADFAFAFWQSLRRKLVSDEWRPVSYDAGSTARRTTSPAAVLKTTATTLPITAEVIAGILDEAGFRVPWRDRDGDAALAAQAIETALEPLSPRPGEPVKIEIADSGFLRNRGACLVGRIKLRDRGDMPMRNLPLLIALLNEKDGLVVDAVLTDSDELQYAFSSTLANYHATNPRYHELARLLYELMPKRPLGTQYSCIGFHHLGKVAVMSEILAEHRKTKEKLATAPGFKGTVAIAFTMPSSAYVLKIIRDHPTDDYKFDYFDGLDEVLRKYNLVHEIDRAGSMLDNIIYSNVKLDRAMFAPELLDELLEAGIGTVTLDRGALVFRHLIVQIKLTPLPLYLANASAAESRAAVINLGDCIKNNAAADIFNKDLDGRNYGVSRIRKVYLFDYDAVEPLTSVTVSRDGAAPGEFDNGMVFRPQEMLEGLRIDDPGLRRAFRDAHPELMQADYWEGMQQALRDGKVPKVMNYPASRR

>Q7PYR2_ANOGA/105-392 PF06472

PGLNIDFLLQLRQLIRIMVPRLLCEESGLLAVHTLCLVSRTFLSIYVASMEGAIVKFIVRKDVRNFVLMLLKWFGIAIPATFINSMIRYLENKLALAFRTRLVKHAYGMYFKNETYYKVSNLDGRIENADHRLTDDISTFSSSVAHLYSSLTKPCFDLLLIGIAMARSSRRMKANIVMGPALATVVIGTTAHILRIVSPKFGQLVAEEANRTGYLRHVHSKIITNAEEIAFYGGHKVEHTQLQEAYGRLVGQMNTIFTQKLWFIMLEQFFMKYVWSGTGMVMVSLPIL

>Q19729_CAEEL/116-306 PF02485

LSYGMLVYKDLPQVLFLLSSIYHPQNEYCIAVGENSAPIFQNLLREVSTCFSNVHFMKRPPISWGSHEIIDSVYDCLEFLSHLETDWRYFQYLSGVDIPLKTNLEMVQILKHLNGTSNVEITNYQQARLTGKNENESPLPLFKSSLSAIIPRKAANQLASSNTARKLLEFLWNTEIADEGFWGTLFGNKDQ

>Q9Z6I4_STRSO/373-612 PF00198

PLRGQTVKMTRIRKVIGDNMVKALTEQAQLSSVVEVDVTRLMKLRGKAKDSFAAREGVKLSPMPFFVKAAAQALKAHPVINARINEAEGTITYFDTENVGIAVDSEKGLMTPVIKHAGDLNLAGIAKATAELAGKVRANKITPDELSGATFTISNTGSRGALFDTIIVPPNQVAILGIGATVKRPAVLETEEGTVIAVRDMTYLTLSYDHRLVDGADAARYLTTVKQILEAGEFEVELGL

>GRM6_RAT/583-844 PF00003

PLLLAVLGIMATTTIMATFMRHNDTPIVRASGRELSYVLLTGIFLIYAITFLMVAEPCAAICAARRLLLGLGTTLSYSALLTKTNRIYRIFEQGKRSVTPPPFISPTSQLVITFGLTSLQVVGVIAWLGAQPPHSVIDYEEQRTVDPEQARGVLKCDMSDLSLIGCLGYSLLLMVTCTVYAIKARGVPETFNEAKPIGFTMYTTCIIWLAFVPIFFGTAQSAEKIYIQTTTLTVSLSLSASVSLGMLYVPKTYVILFHPEQN

>O35269_RAT/511-762 PF00003

LCFMSLGFSSLTAAVLVVFLKNRDTPIVKANNLALSYTLLITLMLCFLCPLLFIGRPSTASCILQQNIFGLLFTVALSTVLAKTITVVIAFKITSPGRIRRWLLISRAPNFIIPLCTLLQVFLSGIWLTTSPPFIDKDAHSEHGHIIIICNKGSAVAFHCNLGYLGALALVSYFMAFLSRNLPDTFNEAKFLAFSMLVFCSVWVTFLPVYHSTKGKNMVAMEVFSILASSTSLLGIIFAPKCYLILLRPERN

>APHA_SHIFL/10-221 PF03767

AVCLLFALNSSAVALASSPSPLNPGTNVARLAEQAPIHWVSVAQIENSLAGRPPMAVGFDIDDTVLFSSPGFWRGKKTFSPESEDYLKNPVFWEKMNNGWDEFSIPKEVARQLIDMHVRRGDAIFFVTGRSPTKTETVSKTLADNFHIPATNMNPVIFAGDKPGQNTKSQWLQDKNIRIFYGDSDNDITAARDVGARGIRILRASNSTYKPL

>ODP2_NEUCR/224-458 PF00198

AAAYTDVPISGMRKTIAARLKESVTENPHFFVSTNLSVSKLLKLRQALNSSADGRYKLSVNDFLIKAMGIASKRVPTVNSSWRDGVIRQFETVDVSVAVATPNGLITPIVKGVEGKGLESISAAVKELAKKARDGKLKPEEYQGGSISISNMGMNPAVQSFTAIINPPQAAILAVGAPQKVAVPVENEDGTTGVSWDEQIIVTASFDHKVVDGAVGAEWIRELKKVIENPLELLL

>PXA2_YEAST/100-383 PF06472

NHKGGERKGKVDFLFKLLLHDKKCLILFITQAILLNIRTLLSLRVATLDGQLVSTLVRAQYANFTKILLGKWMILGIPASFINSLISYTTKLCAVTINRKVSDFLLSKYLSNHHTFYSVASAESVSEIQDNLTKDIYTFSMNSSLLLNQLLKPMLDLILCSFKLLTSNTSVMGEGTLALGLIVYASNSLLKLIQPNFTRLTMASASLESWFRSLHSNLHSSNEEIALLRGQKRELENVDYSFYRLVLFLNREIKARAIYDVATAFVIKYTWGAAGLVLCSIPIF

>14334_SOLLC/6-243 PF00244

REENVYLAKLAEQAERYEEMIEFMEKVAKTADVEELTVEERNLLSVAYKNVIGARRASWRIISSIEQKEESRGNEDHVNTIKEYRSKIEADLSKICDGILSLLESNLIPSASTAESKVFHLKMKGDYHRYLAEFKTGTERKEAAENTLLAYKSAQDIALAELAPTHPIRLGLALNFSVFYYEILNSPDRACNLAKQAFDEAISELDTLGEESYKDSTLIMQLLRDNLTLWTSDNADDV

>O31405_BACSU/168-396 PF00198

EQKAQEIPVTGMRKVIAARMQESLANSAQLTITMKADITKLATLQKQLSPTAEERYGTKTDDHSFLSQEPPVLALQAHPVLNSFYQNERIITHPHVHLGMAVALENGLVVPVIRHAEKLSLIELAQSISENAKKAREGRAGSEELQGSTFSITNLGAFGVEHFTPILNPPETGILGIGASYDTPVYQGEEIVRSTILPLSLTFDHRACDGAPAAAFLKAMKTYLEKPQH

>A1S182_THEPD/218-581 PF01314

YREVYDKIFKLIVDSPLMKKYHEIGTPINVRVLNALGALPSLNLQKASIDSADAISGEYIATNYLGRRVSCAHCPVACIHLAVVREPYEEEPYFYKTTFIGYDYEPIYALGSMLGAGEARDVLRLIDLVDSYGLDAMTTGVVLAWATEALSRGIISEKDLAGVKLRWGDYNAYMKAVQYIVEQPTELYRDLARGALYAARKWGGEDFALSFGGNEMAGYHTGPAAYANFAFGARHSHLDSAGYDLDQETIGRTPEPGELVRRLYEEESWRQVLTSLVVCLFARKVYKPEVVSEALKPLGIEMSVDDLYSLGRKIYREKYRLKLREGFRPEDVSFPRRIFETPTPHGLLSPEYMEKVKLEYVRLI

>Q84G15_STRHY/9-185 PF02737

RLTVLGAGTMGLGITSLVVGHGIPVTVVEVDEAKAGRTRAAVTERLRMAQLMGALPAGRPQGELTVTASLADGRNATAVVEAVTEDTPTKAKVLEAVAGLTGARVPLISNTSSIPIDELAGHIADPARLVGTHFMNPPYLIPTVEVIRGPRTGEAVMTAVTDLLRALERKPVVVGDG

>GCNT1_MOUSE/123-332 PF02485

IAYSIVVHHKIEMLDRLLRAIYMPQNFYCIHVDRKAEESFLAAVQGIASCFDNVFVASQLESVVYASWTRVKADLNCMKDLYRMNANWKYLINLCGMDFPIKTNLEIVRKLKCSTGENNLETEKMPPNKEERWKKRYAVVDGKLTNTGIVKAPPPLKTPLFSGSAYFVVTREYVGYVLENENIQKLMEWAQDTYSPDEFLWATIQRIPEV

>Q8XW80_RALSO/20-84 PF03992

VVPDSGEAFERVFAVQAAAVRANEPGNRLYELFRSQTVPNSYTLVEIYEGEAALAAHRASAHMAA

>ODO2_RALEH/184-414 PF00198

DRPEERVPMSRLRARIAERLLQSQSTNAILTTFNEVNMKPVMDLRNKYKDRFEKEHGVKLGFMSFFVKAAVHALKKFPLINASIDGNDIVYHGYFDIGIAVGSPRGLVVPILRNADQMSLADIEKKIAEFGVKARDGKLSLEELTGGTFSISNGGVFGSMLSTPIINPPQSAILGVHATKDRPVVEDGQIVIRPMNYLAMSYDHRIIDGREAVLGLVAMKDALEDPARLLL

>Q76DK1_PENJP/756-846 PF00207

TWLWDIVVLPSSGVLSQKVTLPDTITEWVGKAVCANAELGVGLSELESITAFTPFFVDLTLPPSVKRGEVLPVKISIFNYLGQPLPVTVDL

>Q7VND7_HAEDU/1-798 PF01295

MQRVDALNDFRIERALAANNPQFQHVFSLLPLLLHTNCPELPVYVKNAPSGIAQFELSAYQQAYLANLMVSDDVVEAFSSHSAFDALYSMGSTGSITQTSLSDLDLWLCYSDRLTAVEYQLIEQKLNKIKQWSQQFGVEINFYLMNPSHFKAHLDHKDVNEEHSGSTQHFFLLDEFYRSAIRLAGKRLLWQHLSDEDYQVCVAQGAFKSKEWIDFGDFSSLSTAEFFGASLWQLYKGIACPYKSAIKILLLESYSQTYPATDLISKEFKRKLLSQQRCSYHFDPYLAMLERVTAYLNGQQERIRLAFLRMCFYIKAMEGEYIESWRTAALDELVAEWKWEDEYLQLIQHRHTWKIKQAVKHQQMIVDQLLHSYRNLIHFARKFHIDPSIMTSDIDTLMRKLYSVFEVLPGKVPLINPNIAQNLAESALTFIEVQGNSAIQPGWYLINQAPKSPYNSALRYVRNGKSLIKIVAWSYFNGLITSSTQLHLISPSLDLNKLRQFITDLRLSFPVQAPAINEQDMLHPNEIRSLILAVNLVKDPTQKLEARRTAVQPSDLFNFGSCQESLVGSVSIIYRNMWNEIRTQHFEGNDAILKALKLISNKIYRSSAPPQSVNVFCYSRRLRSELRDFIADLAHKCITIQTGTISQKQPLNTLKVAGKIWQFVFGKKTVYIQPLTQQAVDLLENNANKYVLKQTKPQLGFPKEIDEFASEGFLQFFFEDNLDNSFNVYILDERNRLETYYNCFDAKEEKIREINRLYSEQKTNILAVASFNFPQFYQIINVSDEIKIVPFQSKQHRD

>OXAA2_BACSU/61-262 PF02096

GEYGLSIILVTIIVRIVVLPLFVNQFKKQRIFQEKMAVIKPQVDSIQVKLKKTKDPEKQKELQMEMMKLYQEHNINPLAMGCLPMLIQSPIMIGLYYAIRSTPEIASHSFLWFSLGQSDILMSLSAGIMYFVQAYIAQKLSAKYSAVPQNPAAQQSAKLMVFIFPVMMTIFSLNVPAALPLYWFTSGLFLTVQNIVLQMTHH

>Q3IMQ9_NATPD/230-627 PF01314

AMELIQESEVTAPNEGGLSLYGTNVLMNATEEMSGLPTRNAKYTSTNDAREDGFGDEGFDAEAVSGENVRENILVDEPTCHSCPVACKKEVEADVVHKGEELNVRTESYEYESAWALGPNSGHTERDEIALMLQRCNDHGIDTIEAGNMLAMAMEMTEEGKLDSLGEGIEWGDTEEMVEMITKIATRDGELADLLAEGPRRVADRMDAHDNSLAVKGQTMAAYDPRCMKGMAIAYATSNRGACHLRGYTPAAEILGIPEKVDPVEWEGKGELTATFQDMHAISDSFDICKFNAFAEGIEEYVTQYNGMTGRDVGEEELLEAGERIYTLERYYNNLAGFDGSDDDLPDRFVEGREGAVPGQGGSEGSLVELEELKNEYYETRGWVDGVVPDERLDELGI

>Q7WM92_BORBR/8-178 PF02737

RIAVVGAGLMGHGIAQAFMVAGFPVSIWDPAEAARASAPQRIAAHLALLGMERPVTVRVCDTLASCVAGSQLVIEAVPEKLELKRELLAVLDELNPDAIVATNTSVLRITEIAADSARPERVVGTHWWNPPYLIPVVEVVRGERTGEDVARRVSVWLAQAGKTPVDVYRDV

>Q9RBX7_PSEIN/180-241 PF01648

LGVDCEPLAAGERLDDIVSACCTAADRACLPSRAAGTMATLIFSAKEAGYKALSHRFGRIVD

>Q566M0_XENTR/734-824 PF00207

TWLWDLVETDADGKADLAVTVPDTITTWKAGMFCTSQSAGFGLSETISLVAFQPFFLELTLPYSAIRGEKFILKATVFNYLSQTIRVAISL

>Q9CHY3_LACLA/10-73 PF03992

IKEDKKSQFLKEIEGLISASKKDEGCLEYSLYESTDNQLEFVMIENWESQEAIEKHNTNPLLLA

>Q8EZ63_LEPIN/36-171 PF07696

SLSFFVYYFSSDSDENFRERIFAKDSSLSFQNIPAEVFSLGFTSNTVWFYIPLKNDTEKDYRGEFEIFNSYLEEVDIFYRYGSKDSIHEILAGTSRVYEKSFPALNFYLRPGEEIQIVCKIKSGTPMRIPIVLESE

>Q72LP6_THET2/219-612 PF01314

LKELARRMAKERMDRAAGLVTMGTVGTVKPFNLRGVLPSHNFLDGFSEGAEALDGTSLDALGIRIGRDTCYACAIRCKQVVKIEGTGKYDVRPEYGGPEYEGLGALGSTCGVTDPYAVTKANTLCNQYGLDVIGVGVTIACAMEAVEKGYLDDEGLGLRFGNGDALIAAIEKLARREGRLGELLAQGAKRLAESLGHPELAMHVKGQEVPMHDPRYKRALGVGYAVSPTGADHNHNLHDTAFAKEGRALRELRFYGEDFQPLPIEDLSEAKIRMLWTKTRERGFVNSLVMCDFVPWTPEEWREALYAATGWRLSPEEMLEVGERTLQLTRLFNLREGISPEEDRLPERFFQPFRKGNPEARLDPEAFREGVRAYRRLAGWEGGVDPERLRALGL

>A1YWG4_XENTR/2-80 PF06446

KSLLLCCLLLLLSLICHRGHSASLSGNEIKAPEHPISESEQGESDALGPLFRTKRHLNICVYCCKCCKKQKGCGMCCFT

>Q6IE37_HUMAN/484-574 PF00207

TWMWDLVSVSSSGSANLSFLIPDTITQWEASGFCVNGDVGFGISSTTTLEVSQPFFIEIASPFSVVQNEQFDLIVNVFSYRNTCVEVSYIW

>ARY1_CHICK/20-280 PF00797

DLETLTDIFQHHIRAVPFENLSIHCGEKITLELEHVYNKIVHKKRGGWCMENNQLLGWVLKCLGYDTSFLGAYVFNPHENAYATIMTHLLVKVVIEGKAYIVDAGFGVSYQMWQPMELVSGKDQPQAPGIFRFTEKNAIWYLEKMRRKQYIPNQNFSNSDLLEKKDCRKVYMFSLEPRTVEDFCFQCTYLQTSPDSLFTKKSICTLQTTDGFRALIGWTLTETKYNYKENMDLVEFITLKDEEVEKTLKDKFNITLERKLV

>Q5JI10_PYRKO/213-598 PF01314

FQRLWQEYYNEFATNPKYEHTRTYGTTDALRSAASLGMSPAYNFSRPYIPDELASKLGGDEVKKYEVEPEWFVHGKSCPIKCARYVEVEYKGKKIRVKPEYESIAMLGAATGVFDFPAVAYFIHLVNDYGMDSIATGATIGWLFEMVERGLISEDEIGFPVKGFGDAEAEERLIKLMAERKGIGAILADGVKRACERLGRGCEFAVHVKGMESPAWDPRGRRTYGLSYATADVGASHLRGWPRPHQLPNQGPAKELVPSLIEGRDESYITDMLGTCKFVPYKMEDLAKLYSVATGEEWTVEELRKRAWGVESIARIHDALDWVTPPLDDTIPPRWWEPEPDGPAKGNAAFIDYNDFLEARREFYRLRGWHEELGVPLPETMEKLGL

>AOR_PYRFU/217-601 PF01314

FMLVVREKVNKLRNDPVAGGGLPKYGTAVLVNIINENGLYPVKNFQTGVYPYAYEQSGEAMAAKYLVRNKPCYACPIGCGRVNRLPTVGETEGPEYESVWALGANLGINDLASIIEANHMCDELGLDTISTGGTLATAMELYEKGHIKDEELGDAPPFRWGNTEVLHYYIEKIAKREGFGDKLAEGSYRLAESYGHPELSMTVKKLELPAYDPRGAEGHGLGYATNNRGGCHIKNYMISPEILGYPYKMDPHDVSDDKIKMLILFQDLTALIDSAGLCLFTTFGLGADDYRDLLNAALGWDFTTEDYLKIGERIWNAERLFNLKAGLDPARDDTLPKRFLEEPMPEGPNKGHTVRLKEMLPRYYKLRGWTEDGKIPKEKLEELGI

>Q70TF5_ONCMY/766-854 PF00207

SFAFAVVDINGKGSHTVALPDSITTWEIQAVSLSASHGICVAEPHEFRVFKKVFVSLRLPYSVKRFEQMSIAPVVYNYCDEAAQIALHM

>Q9IBG9_ORYLA/769-857 PF00207

SFAFTDFEVNKEGRYDLALPDSITTWEIQVVTFSPTSGLCVVKPQEIKAFKKSFVSLRLPYSVKRYEQLSVTPVIYNYDDKELKVAVHM

>Q5NKN5_CARRO/777-868 PF00207

TWIFDDVYVGPKGRVEKELSLPHSITTWVVQAVGISNTGGMCIAEPLKITTFKSIFVQLNIPYSVVRNEQVEIQATVFNNHAHQSVRASVYM

>Q8PEE0_XANCP/16-79 PF03992

VPASARAIVLALLPELQRQSLQEPGCLGYEVLHAPQALECIVLIERYRDSAAIEAHRSSTHYSS

>Q98L66_RHILO/10-77 PF03992

VQNGSEADFEAVWKNRDSSLAEMKGFREFHLLRGPVNETEGCTLFASHTVWASQDDFVAWTKSENFRA

>DIHR_MANSE/83-351 PF00002

TDVASLIYLAGYSLSLAVLSLAVFVFLYFKDLRCLRNTIHTNLMSTYILSACSWILNLVLQNWSDESQQDQTSCMILVICMNYFYLTNFFWMLVEGLYLYMLVVETFTAENIKLKVYTTIGWGAPAVFITIWVISRCFVNVLPSTGPDGLAMFPEAKMCIWMHEHQVDWIHKAPALVGLALNLFFLIRIMWVLITKLRSANTLETEQYRKATKALLVLIPLLGITNLLVLCGPSDDSWFAYAFDYTRALMLSTQGFTVALFYCFMNTEV

>Q9LFQ0_ARATH/88-313 PF02485

LAYLISGSSGDGQMLKRTLMALYHPNNQYVVHLDRESSPEERLDLSGFVANHTLFQRFQNVRMIVKANFVTYRGPTMVANTLHAAAILLREGGDWDWFINLSASDYPLVTQDDLLHTFSYLPRDLNFIDHTSNIGWKESHRAKPIIIDPGLYMSKKADVFWVSQKRSMPTAFKLFTGSAWMMLSRPFVDYFIWGWDNLPRIVLMYYANFLSSPEGYFHTVICNARE

>Q8MT94_DROME/69-160 PF00207

NWIFNIFENVGEEEFTLTKKIPDTITSWVVTGFSLNPTSGIALTKNPSKIRVFQPFFVSTNLPYSVKRGEVIAIPVVIFNYLDKTLDADVVM

>Q7R7A0_PLAYO/224-479 PF01835

SALVTNLSVHFKWGRESSXVWVTTLDSARPVPNADVRISRYCKDETLWQGRTDANGVALIQGPALPNPSDSGESCDWGDGPLMVSARTEDDMSFTLSSWTNGISPQNFGLAVGFYGNPEIVHSVLDRSLFRAGETVSMKHFQRLRTSNGFGLPDTRPAKIKVRHTGSGQEYELPADESATSAXFRVEQFRVPTMRADIQPQSDALINARXATLDLHVSYLSGGGAANAPVKVRTLVEPRAVSFPGYPDFDFEVQAI

>MGAT3_MOUSE/155-509 PF04724

HVLSSRERLGSRGTRRKWVECVCLPGWHGPSCGVPTVVQYSNLPTKERLVPREVPRRVINAININHEFDLLDVRFHELGDVVDAFVVCDSNFTAYGEPRPLKFREMLTNGTFEYIRHKVLYVFLDHFPPGGRQDGWIADDYLRTFLTQDGVSRLRNLRPDDVFIIDDADEIPARDGVLFLKLYDGWTEPFAFHMRKSLYGFFWKQPGTLEVVSGCTMDMLQAVYGLDGIRLRRRQYYTMPNFRQYENRTGHILVQWSLGSPLHFAGWHCSWCFTPEGIYFKLVSAQNGDFPRWGDYEDKRDLNYIRSLIRTGGWFDGTQQEYPPADPSEHMYAPKYLLKNYDQFRYLLENPYREP

>AAAA_PENCH/108-335 PF03417

CQLPNGALQGQNWDFFSATKENLIRLTIRQAGLPTIKFITEAGIIGKVGFNSAGVAVNYNALHLQGLRPTGVPSHIALRIALESTSPSQAYDRIVEQGGMAASAFIMVGNGHEAFGLEFSPTSIRKQVLDANGRMVHTNHCLLQHGKNEKELDPLPDSWNRHQRMEFLLDGFDGTKQAFAQLWADEDNYPFSICRAYEEGKSRGATLFNIIYDHARREATVRLGRPTN

>Q8TJ01_METAC/210-570 PF01314

FEELETKMLKLFDANPVLSKGLANYGTSALVKLLDYMGLIPCRNFSGKGTPFADLFSGEYIKASFELERENCPACPLGCKRRIKKTRQLLPDYDSLWAFGFNLENSDFDSVLRADRICKDYGFDPVSAGSVLGACAEFQSGKIEPRELETLLLGIGEGNEPGKGARNYLSARKREDLSMDVKGLELGGFDPRGIRGQALAYATSCHGGDYLTAFMVGPEVLGKPLLLDRLSLRGKAGILQVFENLTAVLDSLVLCSFSIFAINEELCSALLRAGAGLEIAPAELLKAGERIWNLERIYNLKAGFTRKADTLPERLFEAGSEERVDGIPRQEFETALQEYYLYRVWDREGVPSPEKLMKLGL

>Q9ADL9_POLCB/11-194 PF02737

VVGVVGAGVMGVGVAQSLAQTGHDVVLVDVSEAALARARMGIRNGLRAVTLFGSAEDKKRAGDPKAVLERVAFTTDYGRLAGADFVVENVTEKWDIKREVYARLEGVCRPEIIFAADTSAISITRIGSVTKRPSQVVGMHFMNPVPLKPMVEVIRGFHTSPETLGAAKRFLAEMGKTCVVVEDA

>Q9VLT3_DROME/663-804 PF07703

RNQHIKVTTSTEKPVVGEYIIFHIRTNFYLEEFNYLIMSKGVILVNDRETITEGIKTIAVVLSSEMAPVATIVVWKINQQGQVVADSLTFPVNGISRNNFTVYINNRKARTGEKVEVAIFGEPGSYVGLSGIDSAFYTMQAG

>Q2RM47_MOOTA/217-617 PF01314

FADLVQAIDREIYDHWEYQTRVLLGTTKLVHALNEAGCLATRHFTTGRFEAAEDVSGERLAETVKLKSKACFACTIPCSRFFRIKEGPYRGLASEGPEFEGLAGFSSRVGNPDLDFALQAVDCCNRLGMDVITTSEVISFVMELYARGMLTSSEADGLDLTWGNKETILSLINKIARREGFGDILADGVRAAARRLGKGEDLAMHIKGLEVFQADPRGLKGYALGLAVASRGGDHLRSEPSFEFYEDPEAGRRRFGAPEAAFRLEYKGKGRVVKYYEERCALADSLNACKNTLVNMEILPYEQAAALLRAAVGWDYTPEELRQIGERIVNLERAYIVSLGIRRADDTLPRRFLEEPLPEGSGPSTGQVVELEPMLDEYYTARGWNRDTGIPEPEKLAALGL

>Q89DA2_BRAJA/1-296 PF06472

MKNISATLAIVWRIAVPYFRSEDKWVGRGLLAAVIAMELALVAIDVLVNQWQNRFYSALQASDWDAFVTQIWIFVALASMFIALAVYKLYLNQWLQIRWRQWLTRHYLGEWLQGATHYRMQLKGDAADNPDQRITEDVKNFVEQTLTIGLGLLSSIVTLFSFVIILWGLSNAAPLHLFGTDLMIPGYLCWGALVYAIFGTALTHWIGAPLVNLNFEQQRYEADFRFHLVRVRENSEQIALLKGEGAERGRLLGRFGLVIGNWYAIMSRTKRLTAFTASYQQAAVIFPYVLVAPAFF

>Q7Q4I0_ANOGA/824-915 PF00207

VWLWKDVNIGPHGRYIFNLDVPQVPALWSVSAFGISGTRGYGMIRKPIEYVGIQPFFINVEMPTACHQGEQVGIRVAVFNYQTVDIEATVVL

>GLP2R_RAT/175-443 PF00002

LYTLQLMYTVGYSVSLISLFLALTLFLFLRKLHCTRNYIHMNLFASFILKVLAVLVKDMVSHNSYSKRPDDESGWMSYLSETSVSCRSVQVLLHYFVGTNHLWLLVEGLYLHTLLEPTVFPERRLWPKYLVVGWAFPMLFVIPWGFARAHLENTRCWATNGNLKIWWIIRGPMLLCVTVNFFIFLKILKLLISKLKAHQMCFRDYKYRLAKSTLLLIPLLGVHEVLFTFFPDDQVQGFSKRIRLFIQLTLSSVHGFLVALQYGFANGEV

>Q9IX55_9ADEN/1-446 PF01686

METYNPPPRVLAPTEGRNSITYAPLAPLQDTTHIYYVDNKTSDIQTLNYQQDHSDFYTNIIHNADLNPSDAATQTIRLDQRSRWGGELKTFLKTNSPNVCEFFNSNTFKARVMVSKSDSSNPVYEWVDLSIPEGNFTVDEVIDLMNNAIVEQYLAVGRQQGVEISDIGVKFDTRNFLLGLDPETGLVTPGKYTFKAYHPDIVLLPGCGVDFTNSRLNNILGIRKRQPYQEGFTIMYEDLTSGNIPPLLDVSAYPTEIRPCLEDPSGNTYHVTQVSTNVWECAYRSWNVSYQKKGNAYTTTVLTVPDVTGGIGQLYWSFPDSFKAPITFSNNAAEPPVNGMQMFPLQQKIVYNPNAVYAQLVEQMTNETRIFNRFPSNAILMQPPYNTVTWISENVPSITDHGVQPLRNSLRGVQRVLLTDDRRRACPYIYKSLATVSPRVLSSATL

>P78615_EMENI/1743-1806 PF01648

IGVDVESIDSINISNETFIERILPASEQQYCQNAPSPQSSFAGRWSAKEAVFKSLGVCSKGAGA

>GRM5_RAT/582-831 PF00003

AVVFACLGLLATLFVTVIFIIYRDTPVVKSSSRELCYIILAGICLGYLCTFCLIAKPKQIYCYLQRIGIGLSPAMSYSALVTKTNRIARILAGSKKKICTKKPRFMSACAQLVIAFILICIQLGIIVALFIMEPPDIMHDYPSIREVYLICNTTNLGVVTPLGYNGLLILSCTFYAFKTRNVPANFNEAKYIAFTMYTTCIIWLAFVPIYFGSNYKIITMCFSVSLSATVALGCMFVPKVYIILAKPERN

>Q8EGN0_SHEON/28-160 PF07696

IALDSPYLFHAEAKQLPPADFKEVSQWMGQLKEASSVSLTGGDYWMVSPVMVNSRQTRWVVDASNSIIESVDYWLLGSDGSVQFAHSGYYAPYEFLFDYGRKVRLNMGTDYWLVTRLSSQYFSSAPEVALESQ

>BAI2_HUMAN/917-1197 PF00002

AGSPSVPLVIGCAVSCMALLTLLAIYAAFWRFIKSERSIILLNFCLSILASNILILVGQSRVLSKGVCTMTAAFLHFFFLSSFCWVLTEAWQSYLAVIGRMRTRLVRKRFLCLGWGLPALVVAVSVGFTRTKGYGTSSYCWLSLEGGLLYAFVGPAAVIVLVNMLIGIIVFNKLMARDGISDKSKKQRAGSERCPWASLLLPCSACGAVPSPLLSSASARNAMASLWSSCVVLPLLALTWMSAVLAMTDRRSVLFQALFAVFNSAQGFVITAVHCFLRREV

>P74510_SYNY3/208-433 PF00198

VPVGQTVPLTTFQKALVQNMVAAMAAPTFRVGYTITTDGLDQLYKQIKGKGVTMTALLAKAVALALKKHPIVNASYTDQGIIYHKDVNIALAVAMPDGGLITPVLQNADQVDIYSLSRRWKELVERARAKQLQPEEYSTGTFTISNLGMFGVDRFDAILPPGQGGILAVGASRPQVVANEEGLIGTKRQMAVNVTCDHRVIYGAHAAAFLKDLAVIIEENAQSLTM

>Q9RPS3_ENTFA/199-432 PF00198

TSPDKIVSADPVRKAIAKKMVQSVNEIPHAWLMVEADVTNLVQLRNSLKDEFKQQEGLSLSFFPFFAKAVIQALKKNPKINTSWDDGSIIYHKDVNLSIAVTTDEHLYVPVIQQADNYSIAGLAKEINRLAQEVRQGTLASKEMQGGTFTLNNTGTLGSVQSMGIINHPQAAILQVESINKRLVPTADGGFKVADMVNLCLSIDHRILDGQQAGKFLRDVKDNLAKYNADTDVY

>Q98JF2_RHILO/115-172 PF01648

LGIDVEPAEPLPDDIFAIVATGADRTGAADPRLAGRILFAAKEAVYKAAYPLDREVLG

>Q7W855_BORPA/3-183 PF02737

HVAVIGGGIIGASWAVVFARRGLEVTIVERDAACLAGLPARLAGMIERSASLLGAGEQPGDVAARIGATDALAAAVGRADYVQEAVSENLALKRTLFAELDALAPAHALLASSTSTYGASQFTEALAGRARCLVAHPMTPPHLSPVVEMAASAWTDPQVLAGAEAFMRSLGQHPVRIRKEI

>Q22481_CAEEL/108-317 PF02485

VVFARVVYKDYEFLEKQVQMSYHPQNIFCFFIDSKSKDDFKWRIRRLGRCLPNVFVIDEELRIDSAGHNMNLAHYKCMEKMVKLPDWDYFILMQNHDVVGKSVYEISRIFEILDGANDIDIDKEFGRIDESLKWDLKTLRLFRDESALNSTYLNSTLRVSKGSVQGSLSRAAVEWMVKTVNPRVYLDQWNEGAYGVDEQWISTFQANDFL

>ISDG_LISMO/10-82 PF03992

VEKGAAEHVIRQFTGANGDGHPTKDIAEVEGFLGFELWHSKPEDKDYEEVVVTSKWESEEAQRNWVKSDSFKK

>Q9HHS4_HALSA/10-73 PF03992

FDPDARDDALELIADLVEQSQTEDGLIDYRAAVDVSDPNVVRFFEQYEDAAAFDAHSQTDHFQA

>Q6Z5U7_ORYSJ/196-558 PF03081

TTRWLHTLEYVRCVAAVMHRGGQARARALSAAAEKPVETLLEFATAVSRVSGSPEKLFHMLHMHKALAHAAPLLLAAFIGDAKERFAGELERTLASLGVAVRGILSKTKALIHSYGGSPGQNVVVVVVVPDGGGIHVVTSYLARYVELLAQHAASLNVILAGDVDVDDDDGSQSQMMSPLGRLVAGVIGSLGVMLRRTAELYETEGGEGLRHLFLLNNEHAILQAIETTTLLPLAAEWTQAYRHGIEQHKQGYIQTWAAVATSCLPRDDPPPPPTSAKKAGFLRRRRRSPPLREFAASLEETSVEQMQWKAASPHLRDELRRAVKECVAQAYSEFMDKHPTSNAGEEFATVDDLILRCQIDQI

>ODPX_HUMAN/272-499 PF00198

VGTFTEIPASNIRRVIAKRLTESKSTVPHAYATADCDLGAVLKVRQDLVKDDIKVSVNDFIIKAAAVTLKQMPDVNVSWDGEGPKQLPFIDISVAVATDKGLLTPIIKDAAAKGIQEIADSVKALSKKARDGKLLPEEYQGGSFSISNLGMFGIDEFTAVINPPQACILAVGRFRPVLKLTEDEEGNAKLQQRQLITVTMSSDSRVVDDELATRFLKSFKANLENPIR

>Q9VUB9_DROME/54-501 PF04587

ALEPAPKVAIGYGACTDLQINATEFLDRYYGRRIPVAAATTGSRAVVNNEDELLQSFAYYFQNGAAAERVMANSTLFTQLVGYAKVMDKERINWYMGGNAPLMAVRFFMEGAQVLLGAHMSRKLRPLLPKEIRLAGDEIPNDDIHLILEYKAGDKWGPYVAPRANRYILHNDRNNPHLRAVEQLTDALKMYQPQLLVVSGLQMMDMFTFKSGEREARLQQVQRQLTSQPQGTLNHFEMASYVELQLLQQLRHFVLPYVDSLGMNEQELSNLQQVLAHGRTTLATDWNPRIAHTLDQMRQVFISLLEDYEDRSSSDAKRRSISRIHVHTLAYQAILTTAGSKWKNTRAAAAKAALTAHRYVCKSQFINPEAVLQVLDDSFATSAQADAPRMRIGAASPVPCWREYIQYGRHRQRLEVEICVAPVLVCREARKTAGAGDNISASGLAAQL

>A5D4X4_9FIRM/219-613 PF01314

LQEFTKEFTQVLKTNAAILHDFGTLGTIQGVESEGDLPIKNWTLGSWEEGAYKISGQMLAETVQTGHHACHACTIRCGKEAQVTVGPYKGAIGHAPEYETGAAFGSLILNDDLEIIVACNDLCNRYGMDTIEAGSTIAMAIEAYEKGLLTDRDTEGIELKWGMKEDLLVVLEKMANNKVGLGRLLAQGVKRAADEIGGIAPEFAIHTKGQALAMHDPRAYTTMVADYATCNRGGCHLECLGYFSEGGAYPAKCVGFTKPYDPHGYENKAEYAVRLMNFMTVFNALGLCKFIMLGHITPEMASQWINAATGWDLTGKDVELAGDRLFNIKRMYNNRLGISRKDDVLPQRLLLHDRQTGAAAGSLPYYSRIMKDLYDYRKWTPEGLPSQEKLKELGL

>A2YXE5_ORYSI/221-597 PF03081

IRRWSHAVRAVVKTLLAGERRLCDEVFASDEELGHECFADVARGCLLQLIGFADAVAMSTPATEKLYRMLGMYEALTAVEPDIESLFTGDARDFFSSEVAGVAAQLGSTIRHTIDQFVNVIHGESSRRPVLGGEIHPMTRYVLNYCGLLAECRATLDMVLADNNTSNHDTNDDDHDGGGGGGASSTPSGRCMREILTHLLRNLDEKSRLYDDAGLKNIFLMNNIYYIVQKMMVEFPALRELLGDDWVRRHRGQIRQYETGYLRASWMSVLASLRDDASPAAAHGHGGRAALKEKARSFNAAFEELYRSQTAWKVTDPQLREELRIAVSERLIPAYRSFVGRSRQLLESGSSSGRHSSSAAKHIKYSLEDLEDYMLDF

>Q2LXC3_SYNAS/220-623 PF01314

FELCLRHSQEIAASPFNGDLMAIEWNDAFHHDNFAWGNSRVRRKNYWSQELEDRWKDYTLEIRDRLQGCYNCPKNCHLVVKPPGRQRYMLKCFGKGTWHMAAFEELPFTFDILALAQEYGVDSYAAPQTIAFAIELYEDGILTDKELPDFPASGADRFYYLLEKLVRREGIGDVLANGVYAAARLIGKGAEKYDHNTIKKFEQIPLKLGRVNFPYFLMYCTSDKMAINQSEGSYPQDAIKDPVERQKFADEWISAPERFRRFFMEWEPRTDPSPEASINICDWNETMHYVDDSVGTCAFCSSFRGQFGGGAAYHIYNIPHFINLATGMNMDADDLWQVARRNRNLVRAINVSRGLRRVDEKPPENHWSKREPEKEQALLSEYYTFKGWTDDGIPSKATLDKLGL

>Q8NRX6_CORGL/10-72 PF03992

PLPEYVDTFREQVAEFTEKTRAEEGNIFFDWSINTDNPNEFILIEAFQDDAAEAHVNSDHFKA

>ACEK_ERWCT/9-568 PF06315

VAQTILQGFDAQYGRFLEVTAGAQQRFEQADWPAVQQAMKQRIHLYDHHVGLVVAQLRCITGIRCDDADFLARMKHIYTGLLPDYPRFEIAESFFNSVYCRLFNHRELAPDKLFVFSSQPEKRFHEIPRPIAKTFVPTDGWQRMLEKLLGDVPLRLPWEDLPRDIDYIVTYLQSTFSAEQLEQATLQVANELFYRNKAAWLAGKLSLPDGVFPFLLPIHHNERGALFIDTCLTAQADASMVFGFARSYFMVYAPQPSALVAWLRDILPGKTTAELYLAIGCQKHSKTEYYREYLHYIAESEEQFIIAPGVKGMVMLVFTLPSFDRVFKVIKDRFAPQKEVSAERVMACYQLVKEHDRVGRMADTQEYENFVIDKHRISPELLDELWREVPEKLEDLGDQLVIRHLYMERRMTPLNLYLEQANAQQLHDVIEEYGNAIKQLAAANIFPGDMLFKNFGVTRHGRVVFYDYDEICYMTEVNFRKIPPPRYLEDELAAEPWYSVAPNDVFPEEFPHFLCSDRHIRTLFEEMHGDLFCADYWRALQQRIREGHIEDVYAYRRRKR

>Q0W0W8_UNCMA/218-614 PF01314

YKASMDELLDSTYNNVISGSLLPKNGVTGIMDMVNKHGVLPTRNHQSGTFEHAHDISGQKMSGTMLTRTRGCYCCNIYCTRLINVPYGPYHGLRGKGPDYDATVAFGSQCGNGNLEAAAQANLWCDQYGLDAVTTGETIAWAMELYERGIIDRHDTRGLDLRFGNHEAMVAMVPKIATRMEFGAVLADGLAEAAKKIGKGSEQYVMAVKNMDLPGFEARGSKAMGLQYAVDNRGGDNLRPFGVMTECFGFRSKELNMPEQYDPLSEANKTQWLIPAQNYSVAVNSLICCMFTIIGYSVEPSQYARQLSAITGFSYDTDRLLLAGERIWNLQRAFNVREGFTRKDDRLPDRLTTEPAPSGPAAGSTVHLDPMLDAYYEARGWDRSGIPTMEKLRSLGL

>O96954_GEOCY/141-418 PF00003

YVALAVGGLVFAIVCVFFTVIFRKRKLIRLSSPNLNYLIGLGAIILYFNVITLVIPTTDTVIAAILCNINPWLTSLGYSLCYGTILAKTIRIWFIFNKPRVPSVTKSIVIKDYALALFVVSLVVIDVIILGIFAIVEGLRGELAVHRTSNKENIEDTIGPTCEFHQYYLYICKSKGQVALFTVLFGYKGLLQVTALILAFNTRKVKVKGLDDSKYIAAAIYVTSIVLAVAAISTYTLRDYVNIYPAVVGIGFLLGTTMILGLVFVPRMVGLYQDPQGD

>Q8KUF7_ACTPA/19-248 PF00797

DRAALTALHRAHLRALHYDNTAAATQDGPVPDNLADLDVDATFDGLVTAGRGGICFELNLLFHRLLTDLGFTTTVLSAGVADEEGGFSPDLAHRFTAVHLDGEVLLADVGFAGPSYLDPIRLAPDEQVQHGCAFRVVEQDGRHLVLRRSRTTDWRPLYEFATTPRTLSDWDGFTPRLRRYLDRAVIAGTTLLCRAVDDGHRALVGKRHLVVRDGHETVTTLLDPAEHARV

>Q93HM4_STRAW/25-88 PF03992

LHDGAQKQFLEAYEQLRNQVASVPGHISDQLCQSIENPSQWLITSEWESAPPFLAWVNSEEHVE

>Q8UH99_AGRT5/49-183 PF07696

LDLTATTDIYANQGEAFQVSTAPGPDGIRRRIEVRASSEDHQGDWAVFALANVSEEQLERVIVAPHFRLVNSKLFWPDLGSQRIIAITPSEGFALDRQPSPDADVFRITLNPGSVITFVAELSTPQLPQIYLWEP

>Q65EH4_BACLD/24-251 PF00797

DLPEFLSLLALQFPFENGAVLRNERISMTKTELTEALLNNKRGGLCYDLNAFLYYVLTELGFSVHMVRGTVFNAKEQAWALTGTHVAVILREGDETYLLDTGFGINLPLAPVPFSGEPVPSKTGAYRIRETKTDKGDYLLEMDKGDGWQIGYAFSLTPIDEAVLTCVRDAIFDEKASPFNKNPLASKLIKDGKLILTKDHFTKQTGGGLTKEDVNAGDFQTIFVHSFF

>Q9VM14_DROME/280-512 PF00198

GARYEDIPVTNMRAVIAKRLLESKTQLPHYYVTVQCQVDKLLKFRAKVNKKYEKQGARVSVNDFIIKAVAIASLKVPEANSAWMDTVIRKYDDVDVSVAVSTDKGLITPIVFNADRKGVLEISKDVKALAAKARDNKLQPHEFQGGTISVSNLGMFGVNQFAAVINPPQSCILAIGTTTKQLVADPDSLKGFKEVNMLTVTLSADHRVVDGAVAARWLQHFRDYMEDPSNMVL

>ACEK_ACISJ/14-593 PF06315

IAKAMMDGFNRHYRLFRAESARAKHRFETADWHGQQRAQRERIEFYDLRVRECVRRLDKEFNAGALPMDVWHQIKLHYIGLMVNHLQPELAETFFNSVTTKILHRTHFHNDFIFVRPAVSTEYIESDDPGARPTYRAYYPSRDNLHETVVRIVEHCALQRDFENLPRDAGHVVQALQQRLGAVKLRTNFQVQVLSSLFFRNKGAYLVGKVINGYNELPFALPILHGEDGRLLIDAVLFGENDLQMLFSFARAYFMVDMEIPSAYVQFLRSLMPRKPRAELYTALGLAKQGKTLFYRDFLHHLRYSTDKFRIAPGIKGMVMLVFDLPSFPYVFKLIKDQFPAPKDTTREQVQGKYLLVKQHDRVGRMADTLEYSLVAFPRERFSDELIEEIRRHAPSQIEISDRDGDGRQEVIIAHLYIERRMIPLNIHLQECFDTGLDKPEARSALEHAVTEYGNAIKDMVAANIFPGDMLWKNFGITRNGKVVFYDYDEIEYLTDCNFRRVPPPRCEEDEVSGEVWWPVGPHDVFPETFGPFLLGNDSVREAFMRHHADLLDVEFWQSHKERIQAGHLYDVFPYDSARR

>O93736_PYRFU/213-598 PF01314

FQQLWSEFYKKFSTDPKYADTRKYGTTTALLWAAEVGMGSAYNFSKPHIPEELAKKLSGLEIERYEIEPEWYIHGKSCPIKCSMYMEIEYKGKKIRVKPEYESLGMLGAATGVFDLPAVSYFIWLVNNYGLDSIATGNTIAWFLELVERGLITEEEIGFPVKGFGDAEAVERLIHLIAERKGIGAVLADGVKRACERLGRGCEFAVHVKGLESPAWDPRGRRTYALSYATADIGASHLRGWPSPHQLPNDGPAKELVPSLIEDRDYMYIINMLGVCKFVPYTLDDLAKLYSLATGEEWSVEKLRRVAQAVESMARIYNALEWITPPLDDTIPPRWWEPEKDGPAKGNAAFIDYNDFLEARREFYRLRGWDEELGVPLPETMEKLGY

>Q9I933_CYPCA/769-859 PF00207

SWMWTVIQTDSTGIIRHDAVAPDSITTWEIQAVGISPTKGFCIAEPKPLRVFQDFFLSVNLPYSVKRNEQLQVKAVVYNYKEESLKIIVKM

>NAT_MYCSM/21-265 PF00797

DLGTLHAIVAAHNRSIPFENLDPLLGIPVADLSAEALFAKLVDRRRGGYCYEHNGLLGYVLEELGFEVERLSGRVVWMRADDAPLPAQTHNVLSVAVPGADGRYLVDVGFGGQTLTSPIRLEAGPVQQTRHEPYRLTRHGDDHTLAAQVRGEWQPLYTFTTEPRPRIDLEVGSWYVSTHPGSHFVTGLTVAVVTDDARYNLRGRNLAVHRSGATEHIRFDSAAQVLDAIVNRFGIDLGDLAGRDV

>Q87EM4_XYLFT/65-199 PF07696

AQSLMLERLDDDPPAHEVLAGVYDAMLRPNDTGGASIYETARHPVWWRIRADRQISAAGQPKLQIEFPYLNWVEAWVPGRSVPSHHAIYGAAADRRYATRALVIDLPEGLPQGRAVWLRVHAQSTISMPVSIVSN

>Q8TQ38_METAC/261-647 PF01314

LKERVAEAMKKIRENPVTAPDGGLHTYGTAVLVNLINESGAYPTRNFQEAYFPEADEQSGETLVKKYLTGRYGCWGCPIVCGRKSDVPDGPFSIRNTEGPEYETIFAFGSNCGITELDALIKANHLCDELGLDTISMGDTIACAMELVERGKIPEEKLQGMNLRFGDPSSMIEAIWRTAYRAGIGADLALGSKKLAEKYGAPELSIAVKGMELPAYDPRAIQGIGLNYATANRGGDHVYGYMISPEILELPEKLDPYAVEDKPRWTIILQDLTSAINSSVVCLFTSFALGLPEYAGMLAAITGFDLDADKLLKLGERVTNLERLMNNMYGFDRKEDVLPKRLTEEPVPAGPSKGQISHVPEMINEYYTIRGWVDGKPTKEKLDELEI

>Q2KSF3_ADE04/32-535 PF01686

LEAPYVPPRYLAPTEGRNSIRYSELTPLYDTTRLYLVDNKSADIASLNYQNDHSNFLTTVVQNNDFTPTEASTQTINFDERSRWGGQLKTIMHTNMPNVNQFMYSNKFKARVMVSRKTPNGVTVGDNYDGSQDELKYEWVEFELPEGNFSVTMTIDLMNNAIIDNYLAVGRQNGVLESDIGVKFDTRNFRLGWDPVTELVMPGVYTNEAFHPDIVLLPGCGVDFTESRLSNLLGIRKRQPFQEGFQIMYEDLDGGNIPALLDVEAYEKSKEESVAAATTAVATASTEVRDDNFASAAAVAAVKADETKSKIVIQPVEKDSKERSYNVLSDKKNTAYRSWYLAYNYGDRDKGVRSWTLLTTSDVTCGVEQVYWSLPDMMQDPVTFRSTHQVSNYPVVGAELLPVYSKSFFNEQAVYSQQLRAFTSLTHVFNRFPENQILVRPPAPTITTVSENVPALTDHGTLPLRSSIRGVQRVTVTDARRRTCPYVYKALGIVAPRVLSSRTF

>Q4RJA3_TETNG/94-194 PF00207

SWLWTDVNLPVNCPVDEPSCKTTSLEKTFPLQDSITTWLFTGISLSENHGICVSAPLEVIVRKDFFVDLQLPYSAVRGEQLEIKAILHNYSPDLITVRVDL

>A6VL92_ACTSC/1-845 PF01295

MKYDLEFARKQVADLEKFRLFRALSGTTDEFRYVFQLIALLLHANHPNLPGFVADAPTGIADFKLTDYQRHYLHEILAQSSDIQFPQIFDRTSYAIDGVYVMGSIASIAQTTSSDLDIWVCLREGLKAREREKLQQKAAALQRWAKQYEVDVNLFLMDQNRFRNFQSSGVMTKENCGSTQYMLLLDEFYRSAIRLAGKPLLWLHLLVEDEKNYESEVDDLIRRGEIDPLEWVDFGGLGKFSANEYFGASLWQLYKGIDAPYKAVIKILLLEEYSWEYPNTRLIASDFKFHLLMGHTEDHHFDPYLEMLERVTDYLTYRKDFKRLDDMRHCFYLKATEDWWYRNESNWRVDLINRLAQDWGWSKETIQDLNLRPFWKIKRVKQSYNKLMQMLMVSYRNLIDFARKHHVDANIVPQDISILTRKIYTAFEELPGKVLLINPQISTDLSEPYLTFVEVTATDRPVKKGWYMLNQAPEVSGFSHPRYTEYSATLHKLVASAYFNGLLMPHTRLHIQSPNVSLPILQEFITNLAETLPVHALPPTNDDLHHPCEIRQLMVAINLSNDPTKQLTDSKMPIQQSDLFSFGSEQQNLVGSIDFIYRNLWNEIRTLHFEGPNAILMALKVLSNKIHHGSTALANIEVYSYSRNYRYSLANIVTALIKKCIDIQLGTNQAANSQSMLRVAGKNWQFFFEERGISLHELPENQELPVNELDKELYAEINKNDSVLPVRYEQNMPEKLQYPSEIDSFASEGFLQFFFEDNSDDTFNVYILDEKNHVEIYHRCDGEKEQKINEINYIYTNADSSGNNPYGIIQQNFNYPQFYRIIHNRYYEGNNPPVHIVPFQRQAKY

>Q9YIA8_CYPCA/762-857 PF00207

SWLWEEVDLPTSDKGETTSIPKVIYLKDSITTWQILAVSLSPTLGICVAEPEEMAVFKQLFIDLKMPYSAVRGEQLEIKAIIHNYTPNKQKVRVEL

>Q5QLT4_ORYSJ/188-532 PF03081

IMCWIPAIRVVFHILIPSERHLCDSIFEGFTSYSDVAFVTACHPFLQLLSFGNFIAAAGKNPECLFRIVDMYDAVRDILPVLDDAFNPEVAALRECLGLSIKAILMALENLVRRDPSESCPLDGGVHPMTRYVMNYLVTACVSRHTLEEVMLLEFGSSDPSGNCPIEPDRPTSSLAIHLAWIVDVLTGNLVSKSKVYSHAPLSCVFLVNNGIYIIKKVNGCELKVLLGEDWIKVIHSKVNQWILEYRRATWGKAIMILEMDKRFCSNVNVITEKLSRFNNFVEAICQVQSRWVLVDKQQGVDFSILVEELVIPAYRDMAEMLKATGSAGESYMRLEDVRSRIQQL

>O01717_9CHEL/791-881 PF00207

TWLWELQNIGATGELSLKRDIPHTITEWVGSAICISEETGLGVSEAATVKGFQPFFVSFTLPYSVIRGEKVPIIVTVFNYLSECLPIKLSL

>A6SNP3_BOTFB/246-636 PF03081

GTNGIGTYAMGMEGAFLAEYDNICALFTRDEWGKVFNLTCQGAIAELSRTLRELNNHIKSNLTTDCYLAYEIVEIVSNLSSNLESRTGELKPSFASALKPIRETAKGSLADLLDDTRRRINLLQTLPPDAATVPMTTETMMRLQTMVEFLRPISSIMISIGDGGWKSSATPQGSTDQIPSLKSFDVNADGKQIFANYCIDTIEALLTSLDQKAKALLKGGKPALGIFIANNATIVKRMIETSDLNGLLAPKMGEVERWIKTGTTLYSAAWREPSGYLLDVQYTNRGNVRPQSGSGNTGIDSAAVVKALGSKEKDQIKEKFKMFNQSFDDLIQKHKSLMMEKEVREILARQISSLIKPLYDRFYDKYYEIDKGKGKYVKWDKAAMNAVFSSL

>Q21796_CAEEL/155-364 PF02485

IAFVRTIYKIYELQEALLSISYHPDNVFCFVMDSKSTDRLKESVRIMSSCFTNVVVLGKEYSLNSGGHGQDPAHFDCLKTILDRKWDHAIILQNFDLIIKTPYQLSDISESLNYTSIMGFDHGFSYRYNTKAKWTPAGMKLFKIETGVPNEILNRNLIVRKSLNEVIVSKVFVKSMFEKLNMDIIIKLFDDNDYYGVDEMLVQTLYENYL

>ACEK_PSEAB/10-571 PF06315

IAALILRGFDDYREQFREITDGARARFEQAQWQEAQRASTQRINLYEEKVAETVAGLRVGLADSELLDVERWPIIKSAYIAQIDLRLDDELAETWFNSIFCGLFSHDNISDGTMFVHTTRPSLRAHARAPYTRTYRPGGDLRQALEKIFDDYRFDVPYDDRERDLERIDALLHSNLPDWVCKDPDLAIELIGSVFYRNKGAYLVGRLFTPDEQWPLVFPLLHREGHGIQFDTVITDEAEVSIIFSFTRSYFMVDVPVPAELVAFLKRLLPGKHLAELYTSIGFYKQGKSEFYRALINHLATTDDRFVMAPGVRGMVMSVFTLPGFNTVFKIIKDRFNPSKSVDHATVIQKYQLVKNHDRVGRLADTQQFADFRFPVSKFEPECLAELLEVAPSTVVMEGDVVLIRHCWTERRMTPLNIYLENASEAQTREALNDYGLAIKQLAAANIFPGDMLLKNFGVTRHGRVVFYDYDEICYLTEVNFRYIPEPRFPEDEMSSEPWYSVGPNDVFPEEFPRFLFVDLNQRRLFAKLHGNLYDAKYWQGLQEQIREGKVIDVFPYRRQET

>ACEK_AZOSE/14-575 PF06315

IAQAMIEGFNKHYRIFRETSRRAKESFEAAEWQAQIDAVRERVQFYDERVDEAVRRLHEEFDADSLDDSTWQQLKLQYIGILMRHKQPELAETFFNSVCCKILHRTYFNNDYLFARPAVSTEYIESYPPVYSSYYPQDEGLRTTVRRIIEDFDWQRPFANLDRDIDNILRAVHEHIGAWPDMEVNCQIQVLYSAFYRNKTAYIIGKAINGYQEYPFALAVRHNPAGRLEADTILLDPWRISVLFSLSRAYFLVDMEVPSGYVHFLRSIMPNKHRSELYTMLGLGKQGKTMFFRDLIAHLRHSNDQFIIAPGIRGLVMLVFTLPSYPYVFKIIKDVFGASKNMDRATVKRKYLMVKQVDRVGRLADTLEFSYAALPLSRFHPELLDELRALAPSSFEIEGDSVIIKHLYIERRMTPLNIYLEHADDDQVEYAVREYGNAIRELATANIFPGDMLWKNFGVTRYGRVVFYDYDEIEFMTAMNFRRIPPAPYPEMEMAAEPWYSAGPMDVFPEEFATFLLGAPRVRKAFLKHHRDLLDAKFWQDVQASIRKGYLEDFFPYPTELR

>OXAA1_BACSU/59-246 PF02096

DNYGLSIILVTILIRLLILPLMIKQLRSSKAMQALQPEMQKLKEKYSSKDQKTQQKLQQETMALFQKHGVNPLAGCFPILIQMPILIGFYHAIMRTQAISEHSFLWFDLGEKDPYYILPIVAGVATFVQQKLMMAGNAQQNPQMAMMLWIMPIMIIVFAINFPAALSLYWVVGNLFMIAQTFLIKGPD

>Q5KMS6_CRYNE/297-673 PF03081

MSIWEAIMHAAEQQGETMLAESLFPNHPPQTILTQTLASPITLANKALSPTLSTLKKYLSRHTFTALDLYASVSRLLPQWDHIMSSCLSRLGTPQSPATTELVTALDQSVNTLRSLSLRSFPEMLVDVRSATTEGPPTSVISDITYSTITYLESLMEYEQVVEGLLGKSKSERSWLMGLNELPSNVRSADEEGGIVKFFVADVLGTLLNHLHAKAKGMKRPIGQAFLLNNVSHIRNMLIIQSNSDITGPGAEAMLNKAVRDARTQFISEFQSLTSLLTSAPHSHTQRFAVPQLPTSERHNLKESSIAFFERLGELEGVMARYPLNRQDPEMRDSVGREVEGVVRKGYEAFAARCQSKGAEKYVRSSPDEVSRRVQSM

>Q8PTR7_METMA/221-607 PF01314

LAEKVAESMSLIRENPVTGPKGGLHVYGTAVLVNIVNAHGAYSSRNFQQSYFPEADEQSGEKLAESYLTGTTGCWGCPIVCGRKSFVPDGPFSVKYTEGPEYETIFALGSNCGVKELDAVIKANHFCNEFGIDTISMGGTIACAMELVEKGKIPEEKLLGFNLRFGDPGAMVECVWRAAYKAGFGADLALGSRKLAEKYQAPEYAINVKGMELPAYDPRPIQGIGLEYATSNRGGDHVYGYTIPSELLGIPEKRDPYSTEGKPEWTIFMQDLGSVINSSVICLFTSFALGLPQYAGMLQAITGMELDPEKLLKIGERITNLERHMNNRYGFNRVQDALPKRLTAEPTPAGPSRGQVSHVPEMIDKYYELRGWIDGVPTEEKLKELGI

>Q9KBD3_BACHD/5-185 PF02737

TVGVVGAGTMGSGIANLAAMSGLQVVLLDLDDNQLDIAWQKINTFMEKSVAKGKMSEAEKEAALGRIKSTTTYEELAEADLVIEAVIENLDVKKEVFHTLDTCLANDTIIATNTSSMSITEIAAATNRPDRVVGMHFFNPAQLMKLVEVVRGYQTSDDTVETVKQFARQLKKEPIEVKKDT

>A5CZI7_9FIRM/217-604 PF01314

ILKLNKEIFKFLKSHPMIPLMKKFGTTGGFIPSLLSGDAGVKNFAISLEESGLTVEDFKEVGAELLNEKFKVDDFGCSACPVRCGAFFDVEHEKYPMKHVSRPEYETIGWFTSSILNTDPVIMIKCNELCNEYGMDTISVGGTVGWVLECFNEGLLTKDQLDGIEPYWGNGDAVVALTEKICKMEGCGEILGYGSQWAANHFGVGHDYLLVSSGIEMAQHDPRRAPGYIRTYQLDPSIGRHPKGGLAKANDRMTWEEKYNFRVTGFQDVSEIANTEYINNSGLCIFSIRMFPEGSIYKFLELVTGIHYSRKDIREMGIRSFTMRHAFNIREGLRRKDFTVTNRMIGKPPMKAGPLKGITLDEVRLGDNLYNALGYNVDGVPSLDMLEL

>YCNE_BACSU/10-73 PF03992

VKPEKREEFLSEAQSLVQHSRAEEGNAQYDLFEKVGEENTFVMLEKWKDEAAMKFHNETAHFQG

>O16776_CAEEL/131-337 PF02485

TAFARIVFADYEFIEKQVQASYHPQNVFCFAIDANSSAEFQRKMKKLEQCLPNVVVLPVTESYDSKGHNINLAHYNCMKKLESLRGWGYLMLLQNHDVITKSVYELDRIFELLGGVNDVFMSREIPERRKKHLKWDLKSLKLFRNDSKNSDYELEISSGSVHSSLSRAAVKWLVEEVDLSIFIDQWNQTDYGADEQFISTFQMSPDL

>ACEK_PSEA6/12-574 PF06315

VAYLILHGFDKSYRWHSRITRDAQQRFEQALWQETQKAVKERIAIYERTLADAVGEIYQQVFPHQENNQFWFDLKTRYQKILSDHPQYELAETFYNSVLGRIFKHQKINDEMMFIMPTRCYLAGLQRHLVVHSFDTSGTVRRMLEDIFSQYHFDIAFQDMQRDLQHLDGALRARLNREQLASVHTVEMLKPVFYRSKSAYLIGRICMPDETLPFVIPLSIAEAETSGEKHKIVVEALLTERQDLSVVFSFARAYFMADTQHPAEVVAFLHELLPHKKKFELYIALGLYKHGKTVFYRNFLAHLEESNDQFSIAPGIRGLVMAVFHLPSYGVVFKIIKDEFPESKKITRQHVKDCYKLVKMTDRVGRMADTHEYVNFRLPRHRVEQALVDELLDCCASSIELTDEEVIIKHLYIERKMTPLNIFLEQQPDPALITSALNDLGLCIKQIAAAHIFAGDMLHKNFGITRGGRVIFYDYDEICYLTEREFRTLPKSDDPYAIDTLSVGPTDVFPEQFEHFIVGKKHLKQELKALHGEIMTAEYWQHMQAQSLKGDVPDFIPYNQAKR

>Q8RES5_FUSNN/2-179 PF02737

KVGIIGAGTMGAGIAQAFAQTEGFTVVLCDINNEFAANGKKKIAKGFEKRIAKGKMEQADADKILERITTGTKDICGDCDLIIEAAIENMEIKKQTFKELDDICKPEAIFATNTSSLSITEIGAGLKRPMIGMHFFNPAPVMKLVEIIAGLNTPTDIVDKIKKVSEDIGKVPVQVEEA

>Q2BHU2_9GAMM/9-573 PF06315

IAAQVMVFFAEYRLSFQEITDCAQSYFESANWREIQTLSSERIDLYEEMVEEAAAALEQQLGDSIYQPLLWHQAKSYYSKLIKQRTDPELAETFYNSVYCRLFQHHLIDSENMFIESTRTGKEISSGDSIYSTYSTDDSGLVQLLGRMLDEVPLNLAWEHKRRDIRNLIVFMRDNAALDVLGQLESIDLVNRVFFRNKAAYLVGRLKLEKGSQPFVLPVLNNDDGGLYIDTALTHEDDVSIVFSFTRSYFLVEVDVPSEFVSFLHSLIPEKSRAELYSSIGFYKQGKAEFYRSLQGHLKATDDQFVIAPGIRGMVMSVFTLPSYPVVFKIIKDRFSSSKNVTREVVKQKYQLVKKHDRVGRMADTQEFTNFSFPRERFSDHLLEELKEVAGSSIYISGNEVVIRHLWTERYMTPLNMYIDSLVMEEDLEGLRHVIDEYGKAIKQLAAANIFAGDMLFKNFGVTRHGRVVFYDYDEILYLTDCNFREVPKPIYPEQELASEPWYSVAENDVFPEEFAMLTACHQKIRAIFNELHGDLLSVPFWKQAQDNVRSGVIVDVFPYRGEQR

>Q6PKM9_LATJA/2-86 PF06446

KTFSVAVAVAVVLTFICIQESSAVPVTEVQELEEPMSNDNPVAAHEETSVDSWKMPYNSRHKRAIKCKFCCGCCTPGVCGVCCRF

>Q9LQP9_ARATH/237-588 PF03081

IKIWVIVARVAITTLFNGERILSDHIFSSSVAESCFVDITLQSALNLFIFSLTVAKSRKTAEKIFPTLDVYQTILQLTPKIDQIFSYDSTAAVRLQANESLEKLSESVNAMMTEFQSSITKESSKSAISGGGVHQLTRYVMNFIVFLADYSDSLATILKESSLPLPEDYFSSSGEENPGSGDRSPMAARLAWLILVLLCKIDAKSRLYNDSALSYLFLANNLHYVVTKVRTSNLRLVLGDDWVANHEVKVNQYLEKYEKMAWGDVIASLPGDSTAGTEAEESLRRFNEAFEEAYKKHKTWVVPDPNLRDEIQASIARKLMPGYTGFYKKHPVGSCNIVRFTPEDLNNYITDL

>Q9V1N1_PYRAB/217-615 PF01314

FEKLVEEIELETYAHEEYWSRRIMGTSRILLAANRIGVLPGRHFTEPEVDYAYLVSGERLALEYNVKTRGCFSCVVPCSRVFLIKRGKYAGIMGEGPEYEALAGMTVRIGNSDLDAALYAVKLINDLGLDVISTSEVISWLMELYERGDITSDEIGGLKPVWGDMETVLTLIEDIAYRRGVGDVLADGVLKAAEKLGKGKDIAMQVKGLEMIQADPRGLKGYGLGFAVSTRGADHLRSEPFVELTDNPELCRKLLGIPEACKRLGVRGKGVLVAHYENLCAVVDAIEVCKNLAENMNILDYEKVARLIHVTTGMKMTARDVELVGERIINMERAYIARLGVRREHDRLPERFLRKPLPKGASKGHVIELEPMLEEYYRIRKWDPGKGIPTEKRLKELGL

>Q8PYE1_METMA/216-575 PF01314

FGDLRAKMLKLFEANPVLSKGLSNYGTPAFVKLLDYMDLIPGRNFSERRTSFADRFSGEYIKASFNLEKESCPSCPLSCKRRIKKTGQILPDYDSLWAFGFNLENPDFNSVLLADRICKDYGLDPVSAGSVLGALAELKSEKIEARELEGLLEEMGEGGKAGEGARKYLSSRGREDISMDVKGLELAGFDPRGIKGQALAYATSCHGGDYLTAFMVGPEVLGKPLMLDRFSLKGKAGILQAFENMTAVLDSLVLCPFSVFAVGEELCSALLYAGTGIKISPAELLKAGERIWNLERVYNIKAGFSREDDTLPQRLFETSEWKGIGIDKQEFNAALREYYHYRGWDREGVPLQEKLEELEI

>LYS5_YEAST/133-197 PF01648

VGIDIASPCNYGGREELELFKEVFSEREFNGLLKASDPCTIFTYLWSLKESYTKFTGTGLNTDLS

>A7PVM8_VITVI/289-648 PF03081

VDLWSKHLEYAVKNLLELEYQLCNDVFEKIGSDVSMDCFARIAIQSGFLAFIQFGNTVTESKKDAVKLFKLLKIFHTLNELRLDFNRLFGGKSCIEIRIPTRHLIKRVIDGACEIFWELLPQVEAHKGTSPPSNGSVPSLVSFVVDYCNQLLQDDYRPTMIQVLEIHQNWKHQKFQEELLRKEVRNIVEAVQRNLDAWSKAYEDTSLSYIFLMNNHCHLYKALKGTSLGNLIGDSQLKEHKKNKDYYASIYLRESWGMLPGLLGHEDETLFSGGRAMACSLVKKKLKAFNEALDGTYKKQSNWDVADENLRKRICQLVVDAIVPVYRSYIQKYGHFIEQDGIKNVKIYSEEGLVSMLSSM

>Q74EQ2_GEOSL/217-600 PF01314

YREAARESYRLLKGHPVTAEGLPALGTAVLVNVINQSGALPTRNFQSGTFEGAEDISGERLAQTWLRRNKGCFGCVIACGRVTRLADPRWTESGEGPEYETLWALGAACGVSDLAAITKANYLCNEYGMDTITAGGTVACAMELFERGLLTEGEAGMPLRFGDGDALVRMFQLMGTAEGFGAKLGLGSWRLAESYGAPELSMTVKKQEFPAYDPRGIQGMALEYATSNRGACHVRGYMVSPEILGVPEKLDPAVTEGKAGWTKAFQDFTAVVDSAGICLFTTFAIGAPQVSAFLNAATGLGYSLEQLLEAGDRIWNLERQFNLKAGIDPTQDTLPPRLLSEPLPDGPMQGSVARLAEMLPDYYRARGWDEQGIPTDEKLKSLGL

>Q9UZE9_PYRAB/215-607 PF01314

LKKLSGEAYNDILNAPGYPFWKRQGTMAAVEWTNENSALPTRNFSDGSFEFARSIDGYTMEGMKVKQRGCPYCNMPCGNVVLDAEGQESELDYENVALLGANLGIGKLNEVAVLNRIADDMGMDTISLGGVIGFVMEAKEKGLIKDDEAPEFGDFKKAKELALNIAYRRTELGNFAAEGVKRMAEKLGDDSFAMHVKGLEVSGYNCFIYPAMALAYGTSSIGAHHKEAWVIAWEIGTAPIEGEQAKKVEYKITYDPVKAEKVVELQRLRGGLFEMLTACRLPWVEIGLSLEYYPKLLKAITGVTYTWDDLYKAADRIYALIRAYWVREFNGNWGREMDYPPKRWFNEGLKSGPYKGQHLDREKYDALLSEYYRIRGWDERGIPTKETLKKLDL

>Q97QN9_STRPN/115-347 PF00198

YGKIERIPMTPMRKVIAQRMVESYLTAPTFTLNYEVDMTEMLALRKKVLEPIMEATGKKTTVTDLLSLAVVKTLMKHPYINASLTEDGKTIITHNYVNLAMAVGMDNGLMTPVVYNAEKMSLSELVVAFKDVIGRTLDGKLAPSELQNSTFTISNLGMFGVQSFGPIINQPNSAILGVSSTIEKPVVVNGEIVIRPIMSLGLTIDHRVVDGMAGAKFMKDLKELIETPISMLI

>Q9HYJ0_PSEAE/158-369 PF00198

RFGGERLRGVRRSMALNMARSHAEVVPVTIYGDADLHRWKTARDPLIRLAQALAEACRAEPTLNAWFDGASLSLKLHERLDLGIAVDTPDGLFVPVLRDVGARSAEDLRAGMRRLREDVQARSIPPAEMLGATLTLSNFGTLFGRYANPVVVPPQVAILGAGGIRDEVVAWRGEMAIHPILPLSLSFDHRAATGGEAARFLKVLVNALEQPD

>Q3A1K5_PELCD/219-612 PF01314

YEELVKEIRAKTKEVYDGPLGTYGTSCAVEIFNDCGDLPIKNWLWGTWDKAAKVSGQELAKTVLKKRYHCGGCLIGCGRTVEIPAGAFKMKEGGGPEYETLGLLGSNCLVDDVEAICKGNEICNDYGVDTIEAAGIISFLMEAWEHGMIDEGDTDGLEMTWGNGLAMCEMLEKVCLRKGIGDPCSQGIFEAVKRVGPASEEFAIHTKGMMFPAHDPRGRGGLGVAYATSNRGACHMQAYNQDFEGEGCFNIADLGYDAPLPPYTNEGKGKFVADQQHFMSMMDSLKLCKFSIFGGMTVGPMTQFLNHIVGWDFTNEQWLECGERIFNLKRLFNTREGVSRKDDTLPPRILASPRQGGSGDYVPDLGYMLRDYYRARGWDEWGIPTPETLKRLSL

>Q4G2S5_9TELE/2-96 PF06446

KPMSIACAVAVIIACVCALQSAALPSEVRLDPEVRLEEPEDSEAARSVDQGVAAALAKETSPEALFRTKRQSHLSLCRYCCNCCKNKGCGFCCRF

>Q0C7A6_AEDAE/183-274 PF00207

SWFWKNSTMKNQKTQTFHDVVPDSITSWYVTGFALSPTLGLGLMHAPRKFTVTKPFYMVANLPYSIKRGEVVRIQIMLFNFLNSDLTTDVTL

>ENTD_ECOLI/107-170 PF01648

IGIDIEEIFSVQTARELTDNIITPAEHERLADCGLAFSLALTLAFSAKESAFKASEIQTDAGFL

>O84249_CHLTR/201-428 PF00198

PGSYIEEPLSPVREVISKRLQAAKTFIPHFYVRQRIYASPLLALLKELQEQNIKLSINDCIVRACALALKEFPEINSGFNSVDNKIIRFSTIDISIAVAIPDGVIAPIVRCADRKNIGMISAEIKGLATKAKQQSLAEEEYKGGSFCVSNLGMTGISDFTAILNPPQAAILAVGSVEEQPVVLNGELAVGLTCMLTLSVDHRVIDGYPAAMFMKRLQRLLEAPSVLLL

>Q7SZG2_FUGRU/766-854 PF00207

SFAFMEFDVNDKGRYTLALPDSITTWELQVITLSAATGFCVAEPTDIRAFKDTFVSLRLPYSVRKYEQLSIAPVIYNYGLEPIQLAVHM

>ODP2_MYCCT/207-438 PF00198

PLSWDEVPMNGVRKATVKAMTKSHTEIAAFTGMKNTDITETHKMRTELKDHAAASGIKLTYLAFIIKAVAKSLRDMPNINVRGDFANNKIQFMHNINIGIAVDTPNGLMVPVIKGADHLSVFEIAIKISELANKAKDGKLTRAEMTEATFTVSNFGSVGLDYATPIINSPESAILGVGTMSQTPLYINGELQKRFIMPLSMTCDHRIIDGADAGRFLIKVQDYLSKPVLLFM

>ACEK_POLSJ/14-593 PF06315

IAKAMMDGFNRHYQLFRTESARAKHRFETADWHGQQRAQRERIEFYDLRVREASTRLEREFKAGEQSMDVWHQVKLHYIGLLVNHHQPELAETFFNSVTTKILHRSYFQNDFIFVRPAVSTEYIENEEPTAQPTYRAYYPTRDNLREIIVRLVRDFDLRLEFEDLERDAGYVLEAVSERLSDVKLRANFQIQVLSGLFFRNKGAYVVGKIINGFNEVPLALPILHSKSGKLVIDAALFGEDDLLILFSFARAYFMVDMGIPSAYVQFLRSMMPHMPRAEIYNALGLAKQGKTLFYRDFLHHLRHSTDKFRIAPGIKGMVMLVFDLPSFPYVFKVIKDYYPPQKDTSREQIKGKYLLVKQHDRVGRMADTQEYSEVAFPRARFDDELIAEIEKFAPSQLEISDRDRDGNTEVIIKHVYIERRMIPLNIYLQEAFDAGVGEPAAKNQIERAVVEYGNAIKDLVAANIFPGDMLWKNFGITRHGKVVFYDYDEIEYITDCKFRRVPAPRNDEEEMSGEVWYSVGPKDVFPETFAPFLLGNDAVREVFMKHHGDLLDAEFWQSHQARIQAGHVHDVFPYEREKR

>Q9I1K5_PSEAE/256-317 PF03992

HRSAVDDGYAEAAERMLELASRQPGYLGVESVRGANGFGITVSYWDSEAAIRAWSRHAEHRD

>PDXJ_RHILO/3-246 PF03740

AKLSVNLNAIAMLRNRRDLPWPSVTGIGRLALAAGAHGLTVHPRPDERHTRHSDLPEIRALIDDEFPQAEFNIEGYPSEDFLALVEKHQPEQVTLVPDDPAQATSDHGWNFVADAALLTPIVRRLKKGGFRVSLFSDADPAGMTAARDTGADRIELYTGPYGSYHSDSAKADKELERLGKTADAAFAAGLQVNAGHDLTVGNLPALAKRIPALAEVSIGHGLTADALEYGMAGTVGRFLRACGW

>K6PF_PYRAB/2-445 PF04587

SVPQDVSIFTAYNANVDAITKLNGETIQKLINEFGEKEIAERIEEYPREIREPIDFVARLIHALRLGKPTAVPLVDESLNSWFDEKFEYELERLGGQAGIIANVLAGLGIKKVIAYTPFLPKRLADLFKEGVLYPTVENGELKLKPIREAYRDEDPLKINRIFEFRKGTKFKFLGESVEVPASGRFIVSARFESISKIETKEELRPFLDDIGKEVDGAIFSGYQGLRLKYSDGKDANYYLRRAKEDIISLKEEDVKVHVELASIQDRKLRKKVITNILPIADSVGIDEAEIAQLLSVLGYRDLADRIFTYNRLEDSILGGMIILDELNFEILQVHTIYYLMYITHRDNPLSEEELMKSLEFGTTLAAARASLGDINRPEDYEIGLKVPFNERSEYVKLRFEEAKTKLRMREYKVVVIPTRLVPNPVLTVGLGDTISAGAFITYV

>GRM2_HUMAN/571-824 PF00003

PVTIACLGALATLFVLGVFVRHNATPVVKASGRELCYILLGGVFLCYCMTFIFIAKPSTAVCTLRRLGLGTAFSVCYSALLTKTNRIARIFGGAREGAQRPRFISPASQVAICLALISGQLLIVVAWLVVEAPGTGKETAPERREVVTLRCNHRDASMLGSLAYNVLLIALCTLYAFKTRKCPENFNEAKFIGFTMYTTCIIWLAFLPIFYVTSSDYRVQTTTMCVSVSLSGSVVLGCLFAPKLHIILFQPQKN

>Q9YBC6_AERPE/175-406 PF00198

EEAEERIPVRGIKRSMAQSMSLSKSKIPHAYIAEEVDFTELSKLREALKRDAEEKGVRLTYLPFVFKAVAKAIRKYPLVNSEFDEEKMEIVVKKAVNIGFAVDTPHGLVVPVVKNVEKKGLFAIAREIADLTAKAREMRLSLEEVSGATFTITNVGSIGSVIGFPVIYPPNVAILGVHRLVERPVYVDGELKPRKIGFVSLSFDHRALEGAYATRFLMEVKRLLENPALLFA

>Q84G21_STRHY/20-249 PF00797

TLDTLRHLHKRHLMAVPYDNSTAPDRLPASRHLTNVPLDLVFGHVVTEGHGGVCYELNRLFHTLLAELGYDVRMVAAAVRQANGTFGPEREHTFDLVHLDGRTHLVDVGFPGPSYSEPLYLSEEEQHQYGCSYRVTEHDGYRVVERRPKGSDWQPVYRFRPELADPSGWDAVRLDSLDDYAQDSVLAGTTFRSRATDNGKIVLIGRRYFTVEDGVERTKVLVKADEFQDV

>Q9VXY3_DROME/229-461 PF00198

VPADRVEVLKGVRKAMLKSMTESLKIPHFAYSDEIDMTQLMQFRNQLQLVAKENGVPKLTFMPFCIKAASIALSKYPIVNSSLDLASESLVFKGAHNISVAIDTPQGLVVPNIKNCQTKTIIEIAKDLNALVERGRTGSLSPADFADGTFSLSNIGVIGGTYTHPCIMAPQVAIGAMGRTKAVPRFNDKDEVVKAYVMSVSWSADHRVIDGVTMASFSNVWKQYLENPALFLL

>Q93SM2_STAAU/5-187 PF02737

KVTVLGAGTMGAQLAALFVNAGLKVKLLDIVVDKNDPNLIAKKSYDKITDKKRPLLFDLNLASHLTYGNFDDDLVNDDADLYIEAVKEDIEIKHAVWQQVLQHAKEDALFATNTSGIPINAIAQAFNEKDQERFFGLHFFNPPRIMKLVELIPTSHTKESIILDVKNFAQNVLGKGVIVVNDV

>Q1EVU3_9CLOT/228-685 PF01314

TEIIGSNNQHVVPSTPQPWAEYHWTGSRWTGQPGLFWGAAEPPVETGECPVGDPNRVGLRTQKAIFDLGAVAEERTIKMGGCHSCPIRCQSNLKVPELEKYGLTPYATSTCIGFINPGTVMNVNWQGTEQELIIKSLGSRLADDYGIWSNYGQLGRDFRYMLTTGKFKEVLPKEEYDSIRWDLAESGDPEFLLEFYRRIALKEGEFWKVAQGPYWMAKEWKLGNDYWDNYGNNVWSKMAFPKHHSNEAGAQVGVLVNIMFNRDANSHTHMNLLDNGLPIEILKDILAEKFGSGDALDPKANYTPMNEYKAKFAKWSVDRNFLHDSITLCNWVWPMTASPSKERNYRGDTALEAKFFSLATGIETTEDELDFAAERISTLHRALTVKNMNTVDMRNEHDTIPEWAFVDLKNPDAKPFTEGTVIMDRDDMQLALTMLYKEYGWDEKTGAPTRACLERLNL

>O45866_CAEEL/125-337 PF02485

VAFARVVYMDYELIEKHVEMSYHPQNSFCFAIDKKAAKEFKERMQAMASCLPNVLLLPDDLSVDSHGHNTNLAHYNCLRALINKPGWNYAILLQNHDLITKSVYELEKIFNWLGGANDVAIRPELGRLDKKHFKWDPMSLKLFRNVSESEIDPVILNTTLKFAKGAVQSSLSRAAVDWMTRTVDLTTFIDQWNHGTYGVDEQFTQAFQISDFL

>HEL_HAEIN/6-244 PF03767

KMTALAALSAFVLAGCGSHQMKSEGHANMQLQQQAVLGLNWMQDSGEYKALAYQAYNAAKVAFDHAKVAKGKKKAVVADLDETMLDNSPYAGWQVQNNKPFDGKDWTRWVDARQSRAVPGAVEFNNYVNSHNGKVFYVTNRKDSTEKSGTIDDMKRLGFNGVEESAFYLKKDKSAKAARFAEIEKQGYEIVLYVGDNLDDFGNTVYGKLNADRRAFVDQNQGKFGKTFIMLPNANYGGW

>GRM3_RAT/580-833 PF00003

PVTIACLGFLCTCIVITVFIKHNNTPLVKASGRELCYILLFGVSLSYCMTFFFIAKPSPVICALRRLGLGTSFAICYSALLTKTNCIARIFDGVKNGAQRPKFISPSSQVFICLGLILVQIVMVSVWLILETPGTRRYTLPEKRETVILKCNVKDSSMLISLTYDVVLVILCTVYAFKTRKCPENFNEAKFIGFTMYTTCIIWLAFLPIFYVTSSDYRVQTTTMCISVSLSGFVVLGCLFAPKVHIVLFQPQKN

>ACEK_YERPA/9-568 PF06315

IAQTILQGFDAQYGRFLEVTAGAQHRFEQADWHAVQQAMKKRIHLYDHHVGLVVEQLKYITDQRHFDVEFLARVKEIYTGLLPDYPRFEIAESFFNSVYCRLFKHRDLTPDKLFVFSSQPERRFREIPRPLARDFIPKGDLSGMLQMVLNDLSLRLHWENLSRDIDYIVMAIRQAFTDEQLASAHFQIANELFYRNKAAWLVGKLRLNGDIYPFLLPIHHNESGELFIDTCLTSKAEASIVFGFARSYFMVYVPLPAAMVEWLREILPGKSTAELYTAIGCQKHGKTESYREYLAFIHQSSEQFIIAPGVKGMVMLVFTLPSFDRVFKVIKDQFAPQKEVTQARVLECYQLVKEHDRVGRMADTQEYENFVIDKHRISPELLAELQHEVPEKLEDLGDKIVIKHLYMERRMTPLNLYMEQADDQQLKDAIEEYGNAIKQLAAANIFPGDMLFKNFGVTRHGRVVFYDYDEICYMTEVNFRDIPPPRYPEDEMASEPWYSVSPNDVFPEEFRHFLCSDRKVRHFFEEMHGDLFQASYWRGLQQRIRDGHVEDVFAYRRKQR

>PDXJ_VIBCH/4-240 PF03740

IYLGVNIDHVATLRNARGTQYPDPVHAAEIAERAGADGITIHLREDRRHITDRDVRILRETLQTRMNLEMAVTDEMVEIALQTQPEYVCLVPEKREELTTEGGLDVLGQLERVKAATEKLTAAGIKVSLFIDADREQIDAAKACGAPFIELHTGHYSDAKSDVDQQNELKKIAAAAAYAHDLGITVNAGHGLTYHNVAAIAAIPEIYELNIGHAIIGRAVFDGLAKAVADMKAIMVA

>Q8T2V4_TRYCR/62-342 PF06472

VHFDRTLLRRIIELLRVCFPRIVSAESGLVLLLTSLLMLRTTLTLTFSQISASNTKALMQKNFRHFIFGLLDVAVYAIPATITGVGINYATATLEQCFRGNLQNALHQEYFEGCKMYDLAIKGLVDNPAHRVTHDVQRFCSELSGLFPAVIKPILDIAIFSSALAGFGGYGVPLVMMLYYAFVALMFRMLLPNFAGWVARSREKEGNLRLLHTQLIQHAEEVAFYRGAEIEGENAERLLESFICVERRLKRAKWWSTFINGILVKYAATGVGYAVCAVVVA

>A4J6T0_9FIRM/219-575 PF01314

FKAAAKRFAKGLIEHPVTGQGLPGFGTNVLVNILNEAGGLPTKNFRAGRNEWANNISGETMSATIEARGGKVSHGCHAGCVIRCSQHYHDKEGKYLTSGFEYETIWALGANACINDLDVIAECDRLMDDIGVDSIETSVTIGVAMEAGIIPWGDATAAVDLVKQIGQGTPLGRIIGSGAAFTGQAFGVTRVPVVKKQAIPAYDPRAVKGVGLTYATTPMGADHTAGYAVATNILKVGGFVDPLEKGGQVELSRNLQIATAAVDSTGLCLFVAFCILDNADAFQAIIDMLNAQYSLALTADDVTELGKTVLRAERKFNELAGFTKADDRLPEFFKEECPPHNTTWDFTEEEVDEVFNF

>Q91588_9PIPI/693-789 PF00207

SWFWKVEQMVEKPDVNGISSKTLNVFLKDSITTWEVLAVSLSENKGLCVGQPYEIKVMKDFFIDLKLPYSVVRNEQVEIRAILYNYRNDRIKVRVEL

>A2WY31_ORYSI/215-549 PF03081

IKRWSLATHLVGKALVVMQRQLYTHNSPGAFDALKDEYFLAITKNRILNLLKFADDFTSITSHEKLIYILGMYQALSEAAPGLLLMFTGPHKELVAERSEEILAKLAMSIRSMVASLIAKVRDGVSNTKNIVGVGVGVHPLTKYAVLCIERLAPHRDTLDLILASGGDDVASLSDLASRVVGSLEEKPVLPCDDDATAAATGSRHHLFHANNANFVLQSCKPLLGDEWAAARESVVERHVAGYAEACWAPVVACLEPAGRKPAAKVVAKFSAAFDRAYESQARCEVRDPALRDALRRAVSDKVVTAYGVYLKTHPKLEKKLRYTAGELGERLSEL

>Q82P18_STRAW/329-465 PF05270

VSRSFQSVNYPTRYWQEQSSLLNLPVVSSAASSAEKAASAFTVVAGLADAGGYSFRDAAGNYLRHYDYRARFDADDGTPTFAKDATYVARTGTAAGSVRFESYNYPGYYLRHYNYQLRVDPTDGTDQFRQDSSFNPV

>A0ZWM0_DROME/233-325 PF00207

TWLFSNITDVGANGEYIIKETVPDTLTSWVITGFSLSPQSGLAVTRNPSRIRVFQPFFITTNLPYSVKRGEVIAIPVIVFNYLGMDVKAKVLM

>Q87VY9_PSESM/28-172 PF07696

LPLGRAVQVFEDPTGTATIDSVSSPAGAQAFRPAPAGTFNAGYSRSAFWLKVELSYRPADADIHNDWLLELAYPPMDRVDFYAPDANGRPTLTWQTGDMLPFASRQFAQNNYLFQLELPPGQTRTLYVRISSEGSVQAPLNLWST

>Q3ABF2_CARHZ/218-622 PF01314

FAAAIEKAEQWLKNYPLSSIPTLGTVGLVSLNNSLGILPTKNFQETYFENADQISGEVLNRKYQIKRRACYGCSFACGRYTSVMSGKYATPPMEGPEYETVDMFGPICGVDDLEAIIRANYLCNVYGLDTVSTGMSIGFAMECYEKGLLTEKDTEGMPLRWGDGEVMVKLVEKIAHREGIGEFLAQGVKRMAEQLGPAAEEAAIHVKGLELPAHEPRSESKVLALQYAVSPRGGCHMHPNWASTWDFGQLDCGMKEFGMPWPPKEIQDESPQKGVAYRYVALQGEISEILGACIFYSWGTEGSCITPQLYAEIVSALTGWDVTAEELMLAAERSWNLKRCFNAREGFTREHDKLPKRFTKAIPNGPAKGLKVVNLDAMLDAYYEAMGWDKATGNPTPEKLKELGL

>O02312_CAEEL/156-366 PF02485

IAFARIVYKDYEFIEKQVQVSFHPQNAFCFVIDINASEEFKKRMRALAACMPNVIVLADEDPVYSSGHNVNLVHNKCLKALLDIPGWNYALLLQNHDLIMKSVYEMEQIFEWLGGANDIFVTHEIGRVDVKKLKWDPMSLKLFINETEMDKLLLTTPMKIVKGWVHCSLSRASVEWMFQKLDPSIFMHQLNQGRYGVDEQYFPILQANAEF

>ARY2_HUMAN/20-280 PF00797

DLETLTDILEHQIRAVPFENLNMHCGQAMELGLEAIFDHIVRRNRGGWCLQVNQLLYWALTTIGFQTTMLGGYFYIPPVNKYSTGMVHLLLQVTIDGRNYIVDAGSGSSSQMWQPLELISGKDQPQVPCIFCLTEERGIWYLDQIRREQYITNKEFLNSHLLPKKKHQKIYLFTLEPRTIEDFESMNTYLQTSPTSSFITTSFCSLQTPEGVYCLVGFILTYRKFNYKDNTDLVEFKTLTEEEVEEVLKNIFKISLGRNLV

>Q0WPV2_ARATH/303-668 PF03081

MKKWIQAVKITVRVLLVGEKKICDEIFSSSESSKEVCFNETTKSCVMQLLNFGEAVAIGRRSSEKLFRILDMYDALANVLQTLEVMVTDCFVCNETKGVLEALGDAARGTFVEFENNVRNETSKRPTTNGEVHPMIRYVMNYMKLIVDYAVTLNSLLESNESSGVSGDDSTEEMSPLAKRILGLITSLESNLEDKSKLYEDGGLQHVFMMNNIYYIVQKVKDSELGKLLGDDWVRKRRGQIRQYATGYLRASWSRVLSALRDESMGGSSSGSPSYGQRSNNSSKMALKERFRGFNASFEELYRLQTAWKVPDPQLREELRISISEKVIPAYRAFFGRNRSQLEGGRHAGKYIKYTPDDLESYLPDL

>Q9I932_CYPCA/764-852 PF00207

SFEFQQIDVKEKVRHKIHLPHSITTWEIQSLSFSPSHGFCVAEPIDLTVFKSLFISLRLPYSVKRFEQLSIVAVIFNYGKMERELVVQM

>Q7BRJ6_9PSED/8-188 PF02737

KVVVVGAGLMGTGIAHGFVSAGFHTVLVDTSAQSLSNARTAIENILKAGVSLGKVSAQQADASMARLSTAGDLGEAAIGANWLVETVSEQLAIKKSIVAQAAPLMAPDAIIATNTSALSVTEIAATIDSADRVIGMHFFNPVHKMKLVELVRGLATSDETVARTRDLCDALGKTSIIVNES

>LPHN3_BOVIN/942-1198 PF00002

DLLLDVITWVGILLSLVCLLICIFTFCFFRGLQSDRNTIHKNLCISLFVAELLFLIGINRTDQPIACAVFAALLHFFFLAAFTWMFLEGVQLYIMLVEVFESEHSRRKYFYLVGYGMPALIVAVSAAVDYRSYGTDKVCWLRLDTYFIWSFIGPATLIIMLNVIFLGIALYKMFHHTAILKPESGCLDNINYEDNRPFIKSWVIGAIALLCLLGLTWAFGLMYINESTVIMAYLFTIFNSLQGMFIFIFHCVLQKKV

>Q8ZYU2_PYRAE/222-608 PF01314

FLKVATEYSVKLMNAATSKALHQYGTNLLTNIINSIGGYPTRNFETGYFEEAEKISGEYIKQNFVKETHGCMLCPIQCTQMTVVTTGPYKVAGEKIKYEYESTWALGGNLGLSQTDAVLKMEKLANELGMDTISLGNTLGTFLELVKRGKIQYDTNWGDPAALVDLVYKTAYRIDIGDDLAEGDWRLANKYGAPDAFVGSRGQGFPAYDPRALKGFAISYVTANRGGDHLEAYSPTWEVLGVPEKVDPLCETPECISKQVKLVIYAQHLMALTDSVTYCKFDTLDKDGLFEQHLADLFNAAYGWDVTGQDMLTIGERIFNVERLFHVKEGKWVKDELPPKMREPIKTGPAKGHTASKMFDEGIKEFYKLRGWVDGKPTYETLKRLGL

>Q98DZ9_RHILO/206-437 PF00198

NDAVEDIKVVGLRRKIAEKMTLSKSRIPHITYVEEIDVTALEELRAALNKEKRADRPKLTLLPFLMRAMVKAIAEQPQLNSLFDDEAGIIHQHGGIHIGIAAQTPSGLVVPVVKHAEARDIWDCGAEVNRLAEAAKSGTATRDELSGSTITITSLGAMGGIATTPVINHPEVAIIGVNKMMVRPVWDGTQFIPRKMMNLSSSFDHRVIDGWDAAVFVQRIKALLETPALIFV

>A2WYG7_ORYSI/282-641 PF03081

MKKWIQALKVVVRGLLAEERRICNQIFAADAEAEEDCFTEAAKGCILQLLNFGDAIAIGKRSSEKLFRILGMYEALDEVLPELEGLFSGDARDFIKEEAVGILMRLGDAVRGTVAEFANAIQGETSRRALPGGEIHPLTRYVMNYVRLLADYSRSLNQLLEDWDTELENGGDNVNMTPLGQCVLILITHLQAKIEEKSKLYEDEALQNIFLMNNLLYIVQKVKDSELKTLLGDNWIRQRRGQIRRYSTGYLRSSWTRVLACLRDDGLPQTMGSSSALKASLKERFKNFNLAFEELYKTQTTWKVVDPQLREELKISISEKVLPAYRSFVGRFRGQLEGGRNSARYIKYNPEDLENQVSDF

>Q8H150_ARATH/227-595 PF03081

IKTWLKAVKLAVRKLFFGERILADHVFSSSGLIVESSFTEITQEGALILFTFPEYASKIKKLTPEKMFRFLDMYEALANLYVEIESIFYFESAAAVRSQVINSLARLGDATRLMMTDFESAIQKETSKTPIIGGGVHPLTRYVMNYLSFLADYSDSIAAIFENWKLSVPTPLPDSLYISGGDEANPEDLYSSTVSVRIAWVILLTLCKIDGKAQPYKDVALSYLFLANNLQYVVVKVRSSTLKVLLGDDWVFRHEEKVKLYADKFEKLAWGKVLDLLPEIPTDEISPEEAKVLVARFNDEFETSYRKQTSWVIPDPKLRDQIKITLSQKLMLVCTEFYRMNRFAYGMVGDNEAISRYTPEDIGNYLSDL

>Q6CJX7_KLULA/18-514 PF07247

RAGYVEEVLLMEHRQKLYSNFVVHGELKSAASELQLAHALRALFLRYPILATTIIPEYWNEKETYYTSEAFYNKPGLAEDYVSVVDQLKLEDVIMNKQPEHSKFYNEILNQWEHDGFKYGDDLSVVVSQYTFSCWDPTKPHFRLVLLPSADDKKGARGFKDILYITNHVTSDGTSGANLLEDLSIELGKFSGTNLQQLEYLLHYGRDYEQLPKLPDPIEEKVSYGVNPSFIASFLLSQMGKKLLTKKWDQPITRPIDASPASHMAHIIKIDPTGMQKMRARVKDKLNGKATLTPFMQACWFVSCYEAGLFDDRKWNEFFINMAVPMNTRQYLPEDPEVRDRFRYGTVVGGTNFNFLISSFNITSNQQFWDLTEYYQQWFTKTREEGLATKTFGTLFADFVSKSNNLDKLIKQDMMHQRRAFALLSNVGYRPQKAQESNPFQLQDLIFSQAACEMPFVFSLCCVSTDIGGMSLTLTCCEESISQQQWHKICQVFEKNL

>Q8TP46_METAC/185-453 PF03417

WAGGDGKLIFGKNEDNFNMPEQLSNRMLVVASPDDGIGHAFLTYPGMIGLDGGINADGLAMMTQLSSMQHESMKGCGIATFTRLLLTHARTVEDAIRIFQEHPRCAGIAYHVADARAKKAVVVETSSRKVCCRYPMPGVEALWQTNHSNCYPGWMGYSGYNMVADQVPVNQLKDISTIENWQNSLKEPYNFYVQAPSRFERYQQLIHEYYSNITVENAIKILSDCYDPYTRQTRDVLFPSWTNNILCTICALYPDFAYKAKEPVGQFKA

>Q9RY67_DEIRA/185-415 PF00198

PRPEERVPMTRIRARIAERLKEVQNTAALLTTFNEVNMQPTMELRKKYQDQFVKKHGVKLGFMSLFVRAATEALKAFPMVNASVDGKDVIYHGYYDIGIAVASERGLVVPILRDTDNMSLADIEKQIAEFATRARAGKLTMEDMSGGTFSITNGGTFGSMMSTPIINAPQSAILGMHNIIERPIAQNGQVVIAPMMYLAVSYDHRLIDGKEAVQFLVMIKNLLEDPARMLL

>O06156_MYCTU/11-74 PF03992

VPAGAGPELEKRFAHRAHAVENSPGFLGFQLLRPVKGEERYFVVTHWESDEAFQAWANGPAIAA

>Q9QM76_9ADEN/94-569 PF01686

SELFVPVQRVMAPTGGRNSIKYRDYAPCQNTTKLFYVDNKLSDIDTFNPEANHSNFRTTVIHNQDLDPATAATETIQLDNRSCWGGELKTAVKTNCPNVSSFFQSNTVRVKLMSYRDPVPPGTAAPTSPEPYAPAGAQYKWYDLTIPEGNYALHEIIDLLNEGVVQIYLKEGRQNNVLRSDIGVKFDTRYFNLLQDPVTGLVTPGTYVYKGYHPDVVLLPGCAVDFTYSRLSLMLGIAKREPYSKGFLITYEDLEGGNVPALLDVESAQMTGVDQDVIELADAKPLLKDSKGVSYNVIYDSNNRPVTAYRSWLIAYNQSGSTANETTLLTVPDVGGGIGAMYTSMPDTFVAPTGFKEDNTTNLAPVVGMNLFPALNKVYYQGASTYVQQLENSCQSATAAFNRFPENEILKQAPPINVSAVCDNQPAVVQQGVLPLKNSLAGLQRVLITDDQRRPIPYVYKSLATVQPRVLSSSTL

>Q16YC2_AEDAE/682-773 PF00207

TWLFENAYSGFSGEKTITKKVPDTITSWMITGFSVSPVYGLGLTRQPRKLNVFLPFFVSTNLPYSVKRGEVVSIPIVVFNYMDSDQTAEVTF

>ODP2_SCHPO/252-483 PF00198

PGDYEDLPLSNMRKIIASRLAESKNMNPHYYVTVSVNMEKIIRLRAALNAMADGRYKLSVNDLVIKATTAALRQVPEVNAAWMGDFIRQYKNVDISMAVATPSGLITPVIRNTHALGLAEISTLAKDYGQRARNNKLKPEEYQGGTFTISNLGMFPVDQFTAIINPPQACILAVGTTVDTVVPDSTSEKGFKVAPIMKCTLSSDHRVVDGAMAARFTTALKKILENPLEIML

>Q0IKU9_BALAM/843-933 PF00207

AFLFSIETLDAEGVKTVTSEMPDTITSWVGSAICTNSKDGFGISNKTSITTFKPFFTEVSLPYSMKRGEILSMSVSVFNFLDSSLSVYLEV

>Q9CLY5_PASMU/4-242 PF03767

FKLSVIAVSSLVLLAACSNQQAVEQANQAKLQQQVAMGLIWTQQSGEYAALAHQAFNSAKMAFDHAKAKKGKKKAVVVDLDETMIDNSAYAGWQVQSGQGFSPKTWTKWVDARQSAAIPGAVEFSNYVNANGGTMFFVSNRRDDVEKAGTVDDMKRLGFTGVNDKTLLLKKDKSNKSVRFKQVEDMGYDIVLFVGDNLNDFGDATYKKSNAERRDFVAKNSKAFGKKFIVLPNTQYGDW

>Q9ZVI2_ARATH/22-251 PF03767

STSTWMPMDGNYGASYCLSWRLAVETNNVRAWRIVPLQCLRYVEVYMLAGQYDRDVQLTVDQIKVYLNEIILPGDGMDAWILDVDDTCFSNVFYYRLKRYGCDPYDPTGFRTWAMKGESPAIQPVLELFYKLIETGFKVFLVTGRDEETLRQATLENLHNQGFTGYERLIMRTADNKRQSATTYKTRIRKEMMEEGYRIWGNVGDQWSDLQGEYSGDRTFKIPNPMYFVP

>Q8IT76_ORNMO/797-887 PF00207

TWLWELKELDEHGNLDFKEKIPHTVTEWVGSAVCINNQDGIGVSDPARIKAFQPFFASFSLPYSVIRGEVFPVTLSVFNYLDKCLPVELTL

>Q8VWH7_ARATH/80-361 PF06472

KKKKGGGLKSLQVLTAILLSQMGKMGARDLLALVATVVFRTALSNRLAKVQGFLFRAAFLRRAPLFLRLISENIMLCFMLSTLHSTSKYITGALSLRFRKILTKIIHSHYFENMVYYKISHVDGRITHPEQRIASDVPRFSSELSDLILDDLTAVTDGILYAWRLCSYASPKYIFWILAYVLGAGTAIRNFSPSFGKLMSKEQQLEGEYRQLHSRLRTHSESIAFYGGETREESHIQQKFKNLVSHMSHVLHDHWWFGMIQDFLLKYLGATVAVILIIEPFF

>O42881_SCHPO/6-221 PF02230

NSVIINPSVAHTATVIFLHGLGDSGQGWSFMANTWSNFKHIKWIFPNAPSIPVTVNNGMKMPAWYDIYSFADMKREDENGILRSAGQLHELIDAELALGIPSDRILIGGFSQGCMVSLYAGLTYPKRLAGIMGHSGFLPLASKFPSALSRVAKEIPILLTYMTEDPIVPSVLSSASAKYLINNLQLKCLDRPFEGDAHSLSSESFMAMYKFTQTVI

>Q99SW3_STAAM/102-313 PF03417

FQGKDFMVRNYDYHPATYDGRYLLYQPTDSGLAQIGPVSRVTGRMDGMNESGLTMGYNFMHRKKPANGFVCYMIGRLILENCRNVTEAIQLLKEIPHRSSFSYILMDKSLNHAIVEVTPRSIDVRYDNICTNHFEILTHENRNYTKESKERLARTISQTNDNLDMTTAFKLFNNPQYEIYSKLFKSWSGTIHTSMYQPETLTAYFTLGENAP

>O02309_CAEEL/146-356 PF02485

VAFARIVYKDYELVEKQVQMSYHPQNSFCFAIDKKAPTRFKNQMRAMAACLPNVLLLPDQEPIDSNGHNVNLAHLNCLRALINKPGWNYAMLLQNHDLLTKSVYELEQVYEWLGGANDVELLPEAQRLDEENFKWDPRSLKMFPDESKVDETILNEKIKFSKGGVQGSMSRAAVDWMTRKVNLSTYIDQWNQGRWGVDEMLISSLQISAFL

>Q89UB5_BRAJA/24-156 PF07696

IDLTGVLEHQRSDADRIQVSTAPGTDGIVRRIEVRAREGGQNWVVFALANNTDDQLDRLIVAPHYRIVSSGLLWPDLGLSRIATITPSTGDRPERQESPTADVFRVTLDPGAVITFVAELRTDKLPQLYLWEP

>ISDG_STAAW/11-75 PF03992

LTKGTAKDIIERFYTRHGIETLEGFDGMFVTQTLEQEDFDEVKILTVWKSKQAFTDWLKSDVFKA

>Q8Y476_LISMO/10-73 PF03992

VKPDLVAEFLVEVNLVIQGSLSETGNHGYELVCSVENANIFYILEKWADESAIQFHNGTEHYKL

>SCTR_RABIT/135-391 PF00002

LLKLKVMYTVGYSSSLVMLLVALGILCAFRRLHCTRNYIHMHLFLSFILRALSNFIKDAVLFSSDDAIHCDAHRVGCKLVMVFFQYCIMANYAWLLVEGLYLHSLLVVSFFSERKCLQGFVVLGWGSPAMFVTSWAVTRHFLEDSGCWDINANAAIWWVIRGPVILSILINFILFINILRILTRKLRTQETRGQDMNHYKRLARSTLLLIPLFGVHYIVFVFSPEGAMEIQLFFELALGSFQGLVVAVLYCFLNGEV

>Q743U0_MYCPA/21-260 PF00797

NLEVLRALMAAHTGSIPFENLDPLMGVPVDDLSPAALTDKLVHRRRGGYCYEQNGLLGYALAEIGFRVRRLAGRVVWMQPPDTPPRAQTHTVLAVTFPGSQGAYLVDVGFGGQTLPSPIRFETGNAQQTTHEPYRLDDRGEGLVLQALVRDEWPPLYVFGTRTVPQIDLLVGSWYVSTHPSSMFVTGLMVARTTADARWNLAGRELTVHRAQSSEKIRLDDADAVLDVLGERFGIDVAGI

>Q7MA94_WOLSU/219-575 PF01314

FREAAKVFATSLKEDDVSGKGLPAFGTNVLVNIINEAGALPTRNFRSGRFEFAENISGETMAENIKARGGKTTHGCHAGCIIQCSQVYNTPEGEYQTSGFEYEMIWAYGAHTGINDLDVIAQIDSILDDYGLDAIETGVTFGVAVDAGILEYGDGKRVVELLEEINNATPLGRILGSGAANLGRCYGLSRVPVVKGQSIPAYDPRAIKGQGITYATTTMGADHTAGYAVAVNILGSGGTLDPLKKEGQIELSRNLQIATAAVDSTGMCIFTAFPMLSDPKAMPALVQMIEAKTGATLGMDGLVEYGKKVLKLERNFNERAGLNRAHDRLPEFFKEPLPPHNVVWDFTDEELDSLWNF

>O84405_CHLTR/158-387 PF00198

SKEENRIPLSPLRRAIASSLRQSSEEVPHASLVVDVDVTDLMNLISAERERFTAAHGVKLTITSFIIQCLAKSLEQFPLLNGSLDGDTIVLKKAINVGVAVNLNKEGVVVPVIHNCQDRGLVSIAKVLADLSSRARSNKLDSSETKGGSVTVTNFGMTGALIGMPIIRYPEVAILGIGTIQKRVVVRDDDSLAIRKMMCVTLTFDHRILDGIYGGEFLTALKNRLESVTM

>FAS2_PENPA/1740-1803 PF01648

IGVDVEHIDSVNIENETFVERNFTQSEQDYCRKAASPQSSFAGRWSAKEAVFKSLGVSSKGAGA

>Q9PPW4_UREPA/178-425 PF03767

NTLSAEKDGMLLTEYNSAKHQFDAMIKQSDFDTSKVKVEKDSNGNITKVTVSNPESNKAIPVVFMDIDETILNNYANQNYQLLNNKAYNPKDWDAFVNDKVSKRLAGAFEFIKYVWEHGGVVMFNSNREQENQVDPTIENLVSEGLERSLLPKWIFWMQGVDFSSDKPWANIKKDAQGKRVKSTKEDRMNAMNKRTQGYDLSEFGSGNAVVLKTVMRIGDNFDDFNDNASKGKLNSERVALLKEYGKL

>Q997I0_ADEB4/1-449 PF01686

MQKYVPPPRVLAPTDGRNSITYTPIASLQDTTKVYYIDNKTSDIESLNYYNDHSNFFTNVIQNADLDSAEASTQDIKLDERSRWGGELKTYLKTNCPNVSEFFNSNSLRVKLMVDKTDEKPVYDWVSLTVPEGNYSVGELIDALNNAIIEHYMAVGRQLEVEISDIGVKFDTRDFSLGLDPITSLVTPGKYTYKAFHPDIILLPECGVDFTYSRINNILGIRKKNPYEKGFKIMYEDLTNGNIPPLLDIEKLPSIEPLMNDENDVSYNVVKVTSDPDTWETMYRSWALSYHRKGGAYLNTLLTVPDVTGGVGQVYWSLPDTFKPPITFTNNTTNAETLPVIGMHMFPLKAGLVHNTNAVYSQLLEQVTNRTAVYNRFPQNAILMQPPYNTVTWISENIPFVADHGIQPVKNTLTGVQRVTITDDRRRPCPYIQKSLATVVPKVLSSATL

>VIPR2_HUMAN/123-382 PF00002

YILVKAIYTLGYSVSLMSLATGSIILCLFRKLHCTRNYIHLNLFLSFILRAISVLVKDDVLYSSSGTLHCPDQPSSWVGCKLSLVFLQYCIMANFFWLLVEGLYLHTLLVAMLPPRRCFLAYLLIGWGLPTVCIGAWTAARLYLEDTGCWDTNDHSVPWWVIRIPILISIIVNFVLFISIIRILLQKLTSPDVGGNDQSQYKRLAKSTLLLIPLFGVHYMVFAVFPISISSKYQILFELCLGSFQGLVVAVLYCFLNSEV

>A1WCS5_ACISJ/217-615 PF01314

FMAETFARKKILHDNAVTGQGLPTYGTQVLMNVINEVGALPTRNHRDVQFEGAKDISAEAMATPRASDGKKQLVTNQACFGCTIACGRISKIDETHFTVQNKPQYWGASGGLEYEAAWALGAANGVNDLECLQYANLLCNEQGIDPISFGATVGAVMELYEMGVLSKEQIGIAAPFGSAEALAFLAEETVNGRGFGKEVGQGSRRLCTKYGHPELSMTSKSQEFPAYDGRAIQGIGLAYATSNRGGCHLRGYTIASEILGIPVKTEPTASEGKPELVKAFQDATAAFDSAGVCVFTTFAWSLPDLAPQLQAACDEGYTVQELEKIGERIWNMEREFNNAAGFTKADDSLPRRLLTEAAKTGGSKGTVSHLPEMLPKYYAVRGWDPEGRPTPETRARLGL

>Q9Z9V5_BACHD/5-185 PF02737

QVTVVGAGVMGRGIAYVFALAGFQTTLTDISEEQLNRAQQYVEETAEKGVAKGKVTEEQKAAIVHGLNVTTHLKDAVKQADLIVEAVPEKRELKQAVLEVADEYSPPHAVIASNTSTISPTELGSYTKRSNQTAVMHFFNPVPLMPLVEIVKGLETSQQTVDTIREVSEQIGKETVEVNEF

>CYAA_PROMI/1-835 PF01295

MYLYIETLKQRLDAINQLRLERAFASMSDVFKQVYGLIPVLLHYHHPQLPGYIQGNVPHGTCFFEPDDVQRQWVNKLTNASCDEPMNGYTSGELPITGIYSMGSTSSIGQSHCSDIDIWVCHQSWLDQDERARLQRKCLLIEQWAGELGIDVTFFLIDENRFRHHASGSLGGEDCGSTQHILLLDEFYRTAVRLAGKRLLWTMVPVEEEYHYDEYVNSLYAQGVLTPNEWLDLGGLGELSAEEYFGASLWQLYKSVDSPYKAVLKSILLEAYSADYPNGKLLALEMKQHLHRGEIVNYGLDAYCMMLERVTRYLVSINDLTRLDLIRRCFYLKVCEKLSNEKNENEPAGWRRQVLSQLVTQWQWDHERLAILDNRDSWKIERVRNAHNELLDTMMQSYRNLIRFARRNNLSVSASPQDIGVLTRKLYAAFEALPGKVTLVNPQISPDLSEPHLTFIYVPPGRANRSGWYLYNRAPDFAHIVGHQPLEYNRYLNKLVAWSYFNGLLTKDSQVYIHQGDSSCDEIKLHELVRDISSHFPIRLPAPTPKALYSPCEIRHLAIIVNLEVDPTERFSDQVVHFDFRKLDVFSFGEEEQCLIGSIDLLYRNSWNEVRTLHFNGTQSMLESLKTILGKMHQDAAPPASVEVFCYSQHLRGLIRTRVQQLVSECIELRLSTNRLEPGRFKALRIAGQTWGLFFERLNVSVQKLENAIEFYGAISYNKLHGLPVKLGKDARYLPAVIDGFACEGIIQFFFETTEDNNVFNIYILDEANRVEIYSHCEGSKEELVKDVSRFYSSSHDRFTYGSSFINFNLPQFYQIVKVDGATQVLPFAGGSFGK

>Q4KWL1_9ADEN/43-514 PF01686

SELYLPPQRVLAPLGGRSSIRYRNMSPSQNTTKLFYIDNKKSDIDTYNTNANHSNFRTSVIHNQELDPETAATETIQLDNRSNGEANKTSVKTTALMYQFFQSNTVRVRMMCSREPVSSPQTTNRTHMPLPNAKYKWYDIKIPEGNYSLSDLIDLLNEGIIQAYLKEGPQNNVFAPDMGVKFDTRYLDLLKDPVTNLVTPGVYVNKGYHPDIILLPGCAVDFTYSRLSLLLGIGKRQPYTQGFIIQYEDLTGGNVPALLDLSSVNVDDEDGEIIELQNVKPVIIMDSQGVSYNVIKEGSKVHLAYRSWLIAYQNEGSPAQQSTLLTLPDMSGGIGAMYYSLPDTFQPPTGFKDDNTTNLAPVVAMNLFPALSKVTYVGASAYVQQLENSCNGATAAFNRFPENEILKQAPPMNVSAVSDNQPAVVHQGTLPVKNLLPGLQRVLITDDQRRPIPYIHKTIATVQPSVLSSATL

>Q8GNN3_STRHY/41-259 PF00797

NLQTLAELQWLHLCSLPYDTGYILHQPYEDFDMPRVFEAVMKRGGVCFELNFLFHRLLVEMGFDAHVNSASTALPGGQWGSEIEHMAIRVRIDDVDWLVDVGHGSVAITEPLRIDEQAGSVVQMGTEFRLATRGEWRVLQYKPKGRDWRDAYRMKIKDRAISDWNTWREELPPDADPVVPRKRRRGVENGQVTLVANLFRSIIGGEETVKHVRDEAELI

>A1RWC4_THEPD/218-599 PF01314

LRKTAAEAYASAKSKPPYSFWMRQGTMATIQWSQENSVLPTFNFSEGVFDESSGIDGFAMERLKVSQRGCPNCNSICGNVILDDEGAESELDYENVAMLGSNIGLGDLRKVARLNRLADMWGIDTIGLGSALGFAIEASQRGLLKDRIEWGDFDKILELSREISLGEGPVGSVLSEGVEHASKVLGCEECAVHVKGLSVSAYDCHAAPGMALSYGVSSVGAHHKDAWVISWEVAHGRFEYSKAKAKRVYELQRIRGGFFENLVACRLPWVELGLELDWYVKLFNYATGLSWTLDDHLKVADRTITLIRSYWVREYLAEGRRWGRQLDYPPLKWFTKPYTRGPLKGARLDPQKYDELLGNYYELVGWDHRGVPRASTLERLGL

>Q8TFX8_ASPFU/85-366 PF06472

VEVNREFFRNLLRLLKIVIPGWKSKEFRLLIGHSVFLVLRTMLSLYVAELDGRLVSSLVRGKGKDFLLGLTWWMIVAVPATFTNSMLSYHQCKLALSYRKRLTDYIHDKYLSNMTFYAISALDDRVKNPDQLITVDVSRFSDSLAELYSNLAKPILDMVIYNYSLSKSVGGEGLFIMSLLVQLSANIMRALTPPFGKYVADEARLEGEFRFLHSRLIDYSEEVALYHGQEAEKDTLDKGYFTLIKHVNRILRRRLYHGFMEDFVIKYFWGALGLILCSMPVF

>ODP2_HUMAN/384-614 PF00198

TGVFTDIPISNIRRVIAQRLMQSKQTIPHYYLSIDVNMGEVLLVRKELNKILEGRSKISVNDFIIKASALACLKVPEANSSWMDTVIRQNHVVDVSVAVSTPAGLITPIVFNAHIKGVETIANDVVSLATKAREGKLQPHEFQGGTFTISNLGMFGIKNFSAIINPPQACILAIGASEDKLVPADNEKGFDVASMMSVTLSCDHRVVDGAVGAQWLAEFRKYLEKPITMLL

>Q8CRS5_STAES/102-313 PF03417

YKGRDFLVRNYDYHPATYDGRYLLFQPNDGGLSQIGPTSRVTGRMDGMNEYGLVMAYNFMHRKKPANGFVCYMVGRLILENCKNVTEAIKFLKEVPHRSSFSYILMDRHSNYAIVEVTPRSIDVRYEHICTNHFELLTHENRNYTRESKERLNRVINKTTPSTNKDIAFKLFNDPQYEIYSNLFKSWSGTIHTSLYEPNSLIAWMALGQNSH

>Q9KLV5_VIBCH/10-73 PF03992

AFPDSAQQVQALLEALLEPSRNEEGCCQYELYRDNSIEGLFLMQEIWCSEESLQKHQQSDHFQH

>3HAO_XANCP/1-150 PF06052

MLVPPINLHAWVEQHRHLLKPPVGNKCIQQDGFIIMIVGGPNARTDYHYDEGPEWFFQLEGEMVLKVQDDGTARDIPIRAGEIFLLPPKVPHSPQRAAGSIGLVIERERLPHEQDGLQWYCPQCNHKLYEAMFPLENIETDFPPVFDHFY

>Q9W369_DROME/127-375 PF03417

VNQKNCRLLGHTEDALTETLNHYYFVVAHIISDKPQGKYNVKEEHFMSLCYAGHLPGYTMSHNHHGLVFSINTISAELLRSGKTPRHFITRALLATSNVDDAFRVLKDAGVGAADACSINFTFLADPRQMCYNVEMAPSPDRKNESHLNIKEVPLGEHNYHVNQFDRIRQDQANDLMISSSISRMQTFGAYNPPMSEQDVRHMLGDVSGGVYCVWRENNSCDEVVKTIAVGIFDLSARTISLYSDNPSE

>Q0YNK6_9DELT/217-637 PF01314

YMDLCNEVLDYIKHREENPIPDVMPILAGLGSPQEMKVHDEKWHTENFNWGNARTRRKDFWTEEVDHAWAETMEKARTRLISCYNCPMKCGATISMEGLPTYMMKCFTKLTYTMAAYSDLDFGLRIAQKATEYGLDGFSAPQVMAFAFELLEKGILTEKDFPGLPEDNEEKFFYLLDKIVRRDGIGDVLANGTYWAAQEIGKGAEEYAHNNIKKHEQLPLKLSMLNPIYYLMYCTGEKINITQIEGQFPQAPYPKREQREEFVKDWFQVPDDKFKQIFLDWEPRGEKSMPHYPTVDMCCDIVDWQERMHYIDDALGQCAGLSSFPLKPPYHIHNYPKFIEAGAGIEMDEEKLTLAAKRYRTLVRAINIRRGMRRIDETPPANHWKNRFPELEKELLDSYYKLKGWNDDGIPTKETLDELGL

>ODP2_ACHLA/312-543 PF00198

QGDVEVVKITRLRKAVSNAMTRSKSIIPETVLMDEINVDALVNFRNEAKGLAESKGIKLTYMAFIAKAVLIALKEFPMFNASFNHDTDEVYIKKFINLGMAVDTPDGLIVPNIKNADRLSVFELASQVRSLADDTIARKISMDQQTGGTFTITNFGSAGIAFGTPVINYPELAILGIGKIDRKPWVVGNEIKIAHTLPLSLAVDHRIIDGADGGRFLMRVKELLTNPTLLLL

>A1YWG3_XENTR/2-81 PF06446

KPVPICCLLLLLSFICHRGHSASLSGNEVTVTGNQIPETQMEESNALEPLLRSKRQSHLSICVHCCNCCKYKGCGKCCLT

>Q9RKA2_STRCO/10-73 PF03992

VLPEHADQWPQVVEDFTRATRAEPGCLWFDWSRSLDDPTEYVLVEAFRDDEAGAAHVGSAHFKA

>Q7Q4F0_ANOGA/205-294 PF00207

TWLWESIKAKNGQTSIEKSLPDTITTWVVSGFSVGPANGLQILKKPLQIKSQKRIFVQLHMPPSIKRFEEVSVHCLVHNYGKAINVSLEV

>Q0W315_UNCMA/218-610 PF01314

FMTLVKEQNMRLNKNPVTSDALRYRGTPNILLGVNAAGGLPTRNFQTGQFEEAEKIDGEAMRKELWNDGRNWHPCWNCTIKCTHFHVLDAPGYEGKIDDGPEYETTALLGSNCGISDIKAISLADYIIDGYGMDTISLGNTIAFLMECYEKGLIGKDMTNGLDLRFGNKEAWMAAIHAAGKGEGELGRLASNGVRRAAEEIGQGSADFAAHTKGQEMPAYDPRSGQGTALSYARGIRGADHLKPWVFNKEWLTSGERTDPFSTEDKPALIKRDDADSAILDCICVCRFVANELNLENDFLMLVNAATGFGYDLQEFLDIGERAINLARAFYSREGFGRREDVLPKRFNTEPLKDGLAKGNVAKIEEMIGRYYELCGWDDNGVPTKEKLRSLGL

>Q9LIA2_ARATH/265-644 PF03081

IKKWVRVMRDIVQVYLLSEKSLDNQIFGDLNEIGLTCFVDTVKAPMMQLLNFGEAVSLGPRQPEKLLRILEMYELASELLPEIDALFLDHPGSSVRTEYREVMRRLGDCARTTFLEFKSAIAADVSSHPFPGGAVHPLTNYVMNYLMALTDFKHTLDSLLMEHDDAEDLTIPPSPDIINPVMVEEESTYENSSSPEKFLAMTRHFYSITSVLEANLQEKSKLYKDVSLQHIFLLNNIHYMTRKVLKSELRHIFGDKWNRKHTWKFQQQATEYERATWLPVLSFLKDDGSGSGPGSGSGSGSKNLRPRERFQGFNTAFEEVYKAQTGWLISDEGLREDVRTKASMWVIQAYWTFYSRHKNSVSERYIKYTTDDIERLLLDL

>Q7NWB3_CHRVO/36-263 PF03417

SMEGSLLAKNRDWKPDHAQSLRLLHPEHGYAYLGLYADNGSEPGIKAGVNQKGLAVVAAEASSLPRALRADSARHGVLTRLLRDYGSLDEVASAADKLFAQARPVFLLLADAGGLMQVEIGQHGRYRLIRQQSGTLAHTNHYADTSLLDGAQTIGPSSQARLERIRFLLDQHPAHTLSEFERLSRDRHDGPDNSLWRSGREHTLAGWRIALPAGAPPRLQLTLANPGR

>Q8QGD4_RANCA/723-812 PF00207

TWLFELVVVGPEGHTVLNLNTPDSITKWETDAVCLGKSGIGEIRNVGLVAFQPYFIDLVIPYSVVQGEKFKIDAQIFSYEKKCILVAVSL

>O66049_ENTCL/1-240 PF03767

MMKTSAKLAASGLVALLLTGCASSTHQTAQQQLGQQSVLAVNWFQQSGEYQALTWQAFNTARMAFDQAPSLTGKPKAVIVDLDETMLDNSAYSAWQAKNGQPFSSKTWSAWTQARQAKAVPGAVEFARHVTENGGTLFYVSNRDQKDYAATVANMQQLGFPNVSDKTVRLNTDSSNKQARFDAIKNAGYNVVLYVGDNLNDFGGATWHKGNQTRRDFVNLNHQQFGTQFIVLPNPLYGDW

>Q8KY33_STRCU/12-75 PF03992

HLSDEPDLVLPAYHEASRRMAGTAGLLGNKLLHALGDPRSYVVVSRWSDWEAFTAWESGSAHKD

>Q59298_9CLOT/219-443 PF00198

KYTEKIVPMTQIRKIISARMHESWITSPTVTYDIKVDMTSLKRFKDALKDVCKVTYTDLIVKIVSKVLLQFPLLNCSINGNELITRNYVNMGVAVAIDGGLVVPVVKYANEKGLKEISTEVKDLAKKAKSNQLKPENMTGGTFTITNLGMFGIEYFSPIINQPEVAILGVNKITETPVVQNGEIVIKPLMNLSLTANHRAVDGSVAAQFLSKVKEYMEKPELLML

>EXO70_YARLI/227-601 PF03081

QSNGIHIYAEALLKMLQAEHDNLRCAFPKDEARVKQYFTQLAQTPLQTYQAAGEEISNHVQRNISTDALLIFELIEGTVALKRGLEPLTNSAAPLDRLIQSSYNVSQGVFTEILKICEARVQQVMTLPSDNGVCDATVEVMSRIRRFAEYKDSAVLAISGMKYQQWIPQPRPAWMSTFSSAPAGYTTTKPQELLSAVFSDSIDAFYVTLEMKAKQLNPKKPSQVGFFLLTNLTLIERFVTKSEVYKVLGGQGRERLEKLRKRGLNLFLEGWKATASLLMDTTVVNSKGSLSSKDRELVKDKFKTFNADFEELVKNHKTYTITDPALKQLLAKEVAFICPLYHRYYDKHIGGDFSKNVDKYIKYDKAQFDRVLQEL

>Q9FNR3_ARATH/272-623 PF03081

MRKWTKVVKIITQVYLASEKQLCDQILGDFESISTACFIEISKDAILSLLNFGEAVVLRSCKPEMLERFLSMYEVSAEILVDVDNLFPDETGSSLRIAFHNLSKKLADHTTTTFLKFKDAIASDESTRPFHGGGIHHLTRYVMNYLKLLPEYTDSLNSLLQNIHVDDSIPEKTGEDVLPSTFSPMARHLRSIVTTLESSLERKAQLYADEALKSIFLMNNFRYMVQKVKGSELRRLFGDEWIRKHIASYQCNVTNYERSTWSSILALLRDNNDSVRTLRERCRLFSLAFDDVYKNQTRWSVPDSELRDDLHISTSVKVVQSYRGFLGRNAVRIGEKHIRYTCEDIENMLLDL

>Q9YHC6_RANRI/126-382 PF00002

FGAIKTGYTIGHSLSLISLTAAMIILCIFRKLHCTRNYIHMHLFMSFIMRAIAVFIKDIVLFESGESDHCHVGSVGCKAAMVFFQYCIMANFFWLLVEGLYLHNLLVISFFSEKKYFWWYILIGWGAPSVFITAWSLARVYFEDTGCWDTIESHLWWIIKTPILVSILVNFILFICIIRILVQKLHSPDVGRNENSQYTRLAKSTLLLIPLFGVHYIMFAFFPDNFKVEVKLVFELILGSFQGFVVAVLYCFLNGEV

>Q87K26_VIBPA/22-260 PF00797

DLESLHKIHVAQHRCLPFENFDISLQRGISVEIEDIIQKTVYHSRGGYCFELNGLMLDVLKTLGFEARSLLGRVHVMGTPTGRSHQITLVTLDEQAWIVDVGFGSNTPRAPLPFILNQVIQTDLQTFRFVEDAQFGYFLQVLSTDGTDTWNNLYSFDLEFVFAGDIACGNFFTSTSPNSRFTSARVAAKATESGLVTLLNYTLKYTSQGEQTELELEPGQAYLDALKEYFGIELDAQYG

>PTHR2_HUMAN/141-420 PF00002

FERLYVMYTVGYSISFGSLAVAILIIGYFRRLHCTRNYIHMHLFVSFMLRATSIFVKDRVVHAHIGVKELESLIMQDDPQNSIEATSVDKSQYIGCKIAVVMFIYFLATNYYWILVEGLYLHNLIFVAFFSDTKYLWGFILIGWGFPAAFVAAWAVARATLADARCWELSAGDIKWIYQAPILAAIGLNFILFLNTVRVLATKIWETNAVGHDTRKQYRKLAKSTLVLVLVFGVHYIVFVCLPHSFTGLGWEIRMHCELFFNSFQGFFVSIIYCYCNGEV

>Q6R523_OREMO/40-107 PF08403

VISAFGHDTLDRVPHIDFYRNAGSMSGHRAVRPSLQELHDVFQKNGAISVPDTLEDDGERSIGTPSDD

>Q1V614_VIBAL/1-833 PF01295

MQAYTQKIIQRLDNLNQQRVDRALALMDSQSQQVFHLIPALLNYNHPVIPGYYDADVPFGVHGLELNSIQQQFIDDIQLAIGQPLKTVEKPAILGLYTMGSTSSIGQSTSSDLDIWVCISPEMDCDERELLTNKCLLITDWAQSKGVEANFFLMDKERFRSNHSEEMTGDNCGSSQHLLLLDEFYRSAVRIAGQRLLWQIVPPEMEECYDEYVSQLCSDGYIDCSEWIDFGKLNCIPAEEYFGSNLWQLYKSIDSPYKSVLKAILLEAYSWEYPHTQLLSIDTKRRFFAHEPDLYGMDAYYLMLEKVTRYLERIQDDTRLDLVRRCFYLKTHEKLSREPNIGSVAWRREALSDMIAKWNWDASVVAELDDRRNWKVEQVKVVHHALLDALMQSYRNLIQFARRNDITSAISPQDISILARKLYAAFEVLPGKVTLLNPQISPDLHEPDLSFIEVKKGGVNKSGWYLYKQPLIAHRILGQPYLEHHEYLSKLVAWAFFNGLITESTRLHAVVREAQLDIDKFYQMVSDLRNTFALRKRRPSMQALASPCEISQLAMFINFENDPTSELSGRSLKVDVKNTDIFSFGPEHKNLVGSVDLVYRNSWHEVRTLHFKGETAMLDALKTILGKMHQDALPPESVDVFCYAKNMRGVMRNMVYQLLAECIDLRLKPVEQEKRRRFKAMRLGNQTYGLFFERRGVSVQKLENSIDFYRSISTNKLKGSPLLMLDREQEYQMPEAVDGFASEGLVQFFFEDNEDGFNIYVLDESNQVEVYHQFSGSKDEMISSVNSFYTSVKDDSRVASKFINFNLPQYYQIIHPEEGNAYIIPYRNDGCSP

>A1RWK6_THEPD/232-616 PF01314

FRELSKSLIERIRKNPVTGGGLPKYGTAVLVNIINQAGMLPYKNWQFGYNPEADKISGETLEKTYLISRRPCWGCQIGCGRVVKVPSGPFQILYSEGPEYESIWALGNATGVMDLAAIIKANHYCDEFGLDTISMGSTIAAAMELYEKGYIPQEDLQGLDLRFGNAAALVEAVWRTAYKSGFGAKLALGSRRLAEMYGAPELSMSVKGLEMPAYDPRGAKGIGLEYATANRGGCHVTGYTISPEILGIPQKIDPLTPEGKAQWVKVFQDLTSVVNSAVNCLFTTFEIGAKDYADLFNTIAGFNFTEKDVLTIGERIYNLERYMMSLYGFSAKDDTLPPRLLKEPMPEGPAKGQVVELDKMLEEYYKIRGWVNGVPTKEKLKELGI

>Q17K55_AEDAE/621-713 PF00207

SWLWKSFSMDGRNNFKAIEDSVPDTITTYHVSGFALSPTLGLGVIQQPVSFTVRKKFYLVANLPYSIKRGEVALIQVTVFNFLGSSVTTDVTL

>Q8FSQ9_COREF/9-190 PF02737

HITVIGSGTMGSQIGMVAALSGFETTIIDIAEDALQRAREQLESRMARDVEKGRRTQSEVDEAFTRLDFSTDRDAVVAGTDFVIEAAVEDLSIKRKLFAALDQIAPPHAILATNSSNIVSSRIADATSRPEKVCNMHFFNPVLVMKAVEVVAHPGTSQDTVAITAALAEAMGKQVIHVHKEI

>Q9CFE4_LACLA/11-76 PF03992

GEGEKAHLFADEMVSSGLVYKIRQQSGNLRYEYFVPLDNQQAILLIDEWENQEAIDIHHKSPMMTK

>Q0PIE0_HELMO/216-599 PF01314

FLAASMDARTKLKAHPVTGVGLPTYGTEVLVNVLNQSGALPTKNFASGKFDRADETGGETLREKYLVRNKGCFGCSIGCARVTRISDGPYQGFGEGPEYEAGWSYGANCGIHDLAAITKANFICNELGLDPITMGATIACAMEMIKKGILSREQVGRDLVFGDADAIVELTRQTGLRVGFGDQLAEGSYRLAESYGCPELSMTVKKQELPAYDPRGVQGMGLEYATSNRGGCHVRGYLTAPEILGVPEKLDPLTTEGKAFWLKIFQDLTAVIDSAGICLFTSLGIGLPEIAAQIRTALGWDCSDEAILELGERIWNLEKLFNLKAGFSKADDTLPKRLLEEPLPDGPAKGKVVELEPMLEEYYALRGWNADGIPTAEKTKALTI

>CURD_STRCN/25-86 PF03992

PSRRRQQEFLDAYEQMRDRVASVPDLGDQLCQSLENPSQWLITSEWAAPPYLAWVNSEDHLE

>Q8Y824_LISMO/13-76 PF03992

THPGERDHLVKILLEAAESLEEYNTCIQYIVSESENEADTVFVSEIWVDKGHHEASLDNPAVKA

>O54100_STRCO/38-102 PF03992

VAAPAQGAFENNCTASMRATLSNVPGLRNARLLRPRKGAHGYLAVLNFEDDAAFTAYTSSEAFRA

>PDXJ_XYLFA/5-252 PF03740

TRLSVNVNKIAVLRNSRGDGAPDVIRAASACIDAGAHGITVHPRPDARHIRHDDVIGLSALTRARGVEFNIEGNPFAEPRAGYCGLLALCRETRPHQVTLVPDGDQQITSDHGFDFAREGPGLRPLIDEIKQWGCRVSLFVDVNVTGLADAAIWGVDRIELYTGPYAEMHHAGCSDAVLREFATTARLAQDVGLGVNAGHDLSQTNLGVFLGAVPDVLEVSIGHALISEALYEGLVPTVRRYLDILDS

>Q5P0J8_AZOSE/236-686 PF01314

LKEHNKVIGKKITSTSVIKNALKQVGTPFLYKPSRVLGALGTMNNQKTAWHESLDADNFDPYRPGMDGCYKCPVHCRNLNDMTPEGKGGWGSAALKGLKGNASYDKAQADVEHGKERTYNGIHNDGQFDKYDKGDGPEYVTVGKFGPMIGLKEPEHVLRLNNILNDLGLDSSSTGSAIAWAMELWQRGIIDASHTGGLDLSWGNYETVEKLLFMTAKREGFGDTIADSSRAVERGKYPKEALDYRMAVKGLFQSDPHDARILKAFALGLSVATRGMDHLRNRVTLEINARINDDAAFKTQLYGGAVVPEPNGYGGKEFAVRRCENTFAVGDSVGMCRFNTKLFNSPTTPDCGDFATQVSTATDVTLSADELNEIGRNITGIERLLNFRLGLRAKDDTLPRRWFDEEIEVGPFKGEKVDRKEFEAMKSRFYTVTGLNAEGVPAADWHEQLAH

>Q83901_ADEO7/1-451 PF01686

MQKFVPPPRILAPTEGRNSITYTPLAPLQDTTKVFFIDNKSSDIESLNFTNNHSNFFTNIIQNADLAADEAATQDIKLDERSRWGGELKTFIKTNCPNVSEFFNSNSFLARLMVDKTDPEHPKYEWVQITIPEGNYTGSELIDQLNNGILNNYLEVGRQKGVEIEDIGVKFDTRDFSLGYDPETGLITPGKYTYKAFHPDIILLPECGVDFTYSRINNMLGIRKRFPYTKGFQILYSDLTKGNISPLLNLNNYPHSIEPVMQDENGVSYNVEKISDNPPRWQTKYRSWTLSYKNNGGAKALTVLTVPDITGGLGQIYWSMPDTFKAPITFTNNTTKPETLPIVGLHMFPLKAGLVHNINAVYSQLLEQITNTTQVFNRFPKNAILMQPPYSTVTWISENVPFVADHGIQPLKNSLTGVQRVTITDDRRRSCPYIQKSLATVVPKVLSSATL

>Q7U9C9_SYNPX/100-377 PF06472

LMGGAAFLAMRQQLRNRRWLHWLMLGVIVLMLLTVNGINAGIGFIARDLTNALVAKQEDGFYRILIIYACCFVVALPIRVSQIFFTFKLGIIWRDWLSRSLIGDYMRNRAYYVLNPNDEQATDVDNPDQRITDDTRSFTSQSLQFTLGVFDALLTFSLNILILWSISTTLTLSLFGYASFATAVLVISGRKLVKINFDQLRYEADFRYGLVHIRNNAESIAFYSGEEPEAAETERRLGSVVRNFNLLIIWRVIIDVMRRSIGYAGNFFPYLVMAVPYF

>Q86ZN9_FUSOX/351-495 PF05270

ISLKVTTSGYTDRYLTHSDSTVNTQVVSSSSATALKQSASWTVRTGLANSGCVSFESNDTPGSYIRHSGFTLYVNKGDGSKSFNEDATFCPQKGLSGSGSSIRSWNYPTRYIRHYNNLGYASSNGGVHDWDAAKSFVDDVTFVVA

>Q2QX89_ORYSJ/323-687 PF03081

IATWTKAFRHTINVGLSTEHDLCARVFAGRHAAVGRGMFADLARCVMLHMLNFTEAVTMTKRAAEKLFKVLDMYEATRDASPVIDAFLTADDGNNSTALTDLKHELNSVRSRLGEFAAAIFRELESSIRADAGKQPVPGGAVHPLTRYVMNYLKYACEYNSTLEQVFREHGAHGGGGGGDGENPFAAQLMEVMELLHGNLEGKSRLYKDPSLSNIFLMNNGRYMLQKIRGSPETNAMLGEAWARKQSTNLRQYHKNYQRETWSRVLGLLRDDGVLTVKGSVQKPVLKERFKQFNAAMDEIQRTQGAWVVSDEQLQSELRVSIAAVVVPAYRSFLGRFAQTFSAGRQSEKYVKLSADDVEAIIDEL

>Q2I626_GLOMM/313-404 PF00207

TWLFDNIESTNEEEFTYVTKIPDTITSWLISGFSMNPNKGLGITADKTKVVTFQPFFISIRLPYSVKRGEVINVPALVFNYLNKDLDVEVIL

>Q3ADZ1_CARHZ/217-597 PF01314

TKELLSRLLKKIREDGVTGQGLPNYGTAVLVNIINENGILPTNNFQKSYFPTADAISGETLSEKYLVKKDPCYRCPIACGRYCKVDDEEGGGPEYETIWAFGADCGVDDLAAIIKANNLCNEYGLDTISAGATIACAMELYEKGHIKKEELDGPELKFGSAEAVIEWTRKMGAGEGFGAKLALGSYRLAESYGVPELSMSVKKQELPAYDPRGVQGHGLQYATSNRGGCHVRGYLISPEILGSPEKIDRFALEGKANWAKLFQDLTAVIDSLGLCLFTSFALNADDYRELFNYIVGENYTTEDILTAGERIWNLERVFNLKAGIDAKEDTLPKRLLEEPVPDGPSKGHKHRLAELLPEYYKVRGWDEKGVPTAEKLQALGL

>CALRL_HUMAN/138-391 PF00002

ALNLFYLTIIGHGLSIASLLISLGIFFYFKSLSCQRITLHKNLFFSFVCNSVVTIIHLTAVANNQALVATNPVSCKVSQFIHLYLMGCNYFWMLCEGIYLHTLIVVAVFAEKQHLMWYYFLGWGFPLIPACIHAIARSLYYNDNCWISSDTHLLYIIHGPICAALLVNLFFLLNIVRVLITKLKVTHQAESNLYMKAVRATLILVPLLGIEFVLIPWRPEGKIAEEVYDYIMHILMHFQGLLVSTIFCFFNGEV

>ODP2_MYCGE/151-383 PF00198

KTEQKTIAISTMRKAIAEAMTKSHAIIPTTVLTFYVNATKLKQYRESVNGYALSKYSMKISYFAFFVKAIVNALKKFPVFNASYDPDQNEIVLNDDINVGIAVDTEEGLIVPNIKQAQTKSVVEIAQAIVDLANKARTKKIKLTDLNKGTISVTNFGSLGAAVGTPIIKYPEMCIVATGNLEERIVKVENGIAVHTILPLTIAADHRWVDGADVGRFGKEIAKQIEELIDLTV

>A1SR20_PSYIN/9-865 PF01295

LNPEIKRLRDKADELTRLRIQSAELLMNADTLTVFRTFPLLLHYNHPSLPGYTKGDTPTGIHQFNVNKKQKKQLAELFPYQEEPQKKAQATNLITKPTDLKIQLNNLLSFQDEPQKKPQQADLIGLYAMGSTASIGQSSESDLDIWICYPHQMDKQRVQLLKNKSWLITHWAEGYGVELNFFLIPDNKFRIDNKSDMTMDACGTSQHLLLLDEFYRTELHIAGKRILWWQVPFEHEKNYNEYVKKLYDNGGLNPDDWLDLGGFDRIPAEEYFGATLWQLYKGIDTPYKAVLKTILMEAYSWEYPNTELIALYYKKRFQEQQTDLEYLDPYCLMLEKVTSYLNKINDFKRLEVVRACFYLKTEENLSKICTLNPPTWRRQVLTKFVNRWDWNKDHIADLDNRKKWKVRAVSNAKDILLDTLMASYSKLLRFARDNNIEGSISAEDLGILSRKLYAAHESLPGKVDLINPKISPDLSEPDLSFIQVPKNRKNAAGWYLYNCSLDSFTLINTPTLMYAKYIAKLVAWCHMNGLYEEETQLHLYNQGSDLVDKKLNQFIQDLYSVFPIYIPKASNNALSRPCEIKHLTIFLNLERDPTRHWESTSESEDDQQGDIFSYGEDKECLIGSIDLIYRNSWNEVRTLHFNSNQSVIDALHTILGKMHQGACPPEQIDLFCYSCQFQAQISQAFKSKLNEYIQLRLDSLSQRSVQTLKTGEENFAFYFEQTGVSVQHLKNLVDIYSHISNKKVSNSALNLKNTYFDKTVSIIGSHISEGIVQFFFENYHDGFNVYIANADNEIETLHSFTGGKDDLVQNVNRFYASNNNEMNKQDDRINFNLPQFYEIELSDNKELILSSFTSTTK

>Q6NRX1_XENLA/725-816 PF00207

TFLWRLVSVDSEGQNTITETVPDTITKWQGSMFCVSEKEGFGITKYSANFTSFLPFFVELSLPYSLTREEILVMKAFVSNYLEECIKIIVTL

>PXA1_YEAST/181-467 PF06472

QLFYSKFLNQMNVLSKILIPTVFDKNFLLLTAQIFFLVMRTWLSLFVAKLDGQIVKNIIAGRGRSFLWDLGCWFLIAVPASYTNSAIKLLQRKLSLNFRVNLTRYIHDMYLDKRLTFYKLIFDAKASNSVIKNIDNSITNDVAKFCDATCSVFANIAKPVIDLIFFSVYLRDNLGTVGVAGIFVNYFITGFILRKYTPPLGKLAGERSASDGDYYNYHLNMINNSEEIAFYQGTAVERTKVKELYDVLMEKMLLVDKVKFGYNMLEDYVLKYTWSGLGYVFASIPIV

>A1HS18_9FIRM/216-573 PF01314

FRAGAKKLANALLKHPVTSQGLPTYGTDVLVNTLSEAGGLPTRNFSVGRFEGANNIGGETLHAICKERGGKTGHACSPGCIIRCSNIYKDAAGNVVTGGLEYESCWSLGADCGIDNLDDIAVLNRMCDDYGVDTIEMGVTLGVAMEAGLAKFGDGKAAIELLKEVGKASPLGRILGAGAAVTGKAFGVRRVPVVKGQAIPAYDPRAVKGIGVTYATTTMGADHTAGYAVAANILGVGGKVDPLSPAGQVELSRNLQIATSFLDSTGLCLFVAFAILDIPEGLDGIVEMCNARYGWNKSLDDYLELGKQVLRDERAFNKAAGFNDDADDLPDFFRTEPLPPHNTVFDVPKEELKSLFNF

>GLKA_PYRAB/17-448 PF04587

SIGKIKGVMLAYNTNIDAIKYLKREDLERRIEEAGKDEVLRYSDELPKKINTIQQLLGSILWSVKRGKAAELLVEDREVRNYMRQWGWDELRMGGQVGIMANLLGGVYGIPVIAHVPQISKLQASLFLDGPIYVPTFEEGLKLVHPRNFEGNEEDCIHYIYEFPRGFKVLNFTAPRENRFIGAADDYNPRLYIRKEWVERFEEIAERAELAIVSGLHSLTEETYREPIKVVREHLKVLKDLNIKTHLEFAFTADEKVRREILGLLSLVYSVGLNEVELASVLEIMNERELADRILAKDPADPVAVIEGLMKLIEEGVERIHFHTYGYYLAITKYRGEHVRDALLFSALAAATKAMLGNIEKLDDLRKGLEVPIGRQGLEVYEVVKREFNVEKGIGEVGDYQIAFVPTKIVEKPKSTVGIGDTISSSAFVSEF

>VSPB_SOYBN/29-254 PF03767

RMKTGYGERSSEVKCASFRLAVEAHNIRAFKTIPEECVEPTKDYINGEQFRSDSKTVNQQAFFYASEREVHHNDIFIFGIDNTVLSNIPYYEKHGYGVEEFNETLYDEWVNKGDAPALPETLKNYNKLLSLGFKIVFLSGRYLDKMAVTEANLKKAGFHTWEQLILKDPHLITPNALSYKSAMRENLLRQGYRIVGIIGDQWSDLLGDHRGESRTFKLPNPMYYIE

>A0T1M1_MACRS/759-849 PF00207

TWLWDLVRLPETGVSSEPVTVPDTITEWAGKVVCVHPEKGIGLSKRTTIKTFTSFFLDLTLPPSVKRGEILPVKISIFNYLSEKLPVTVSL

>PEN3_ADEM1/23-488 PF01686

VQQPWVPPRYFAPTEGRNSIVYDQFPTCYDTTKLFLVDNKSADITDLNMQNDHSHFATTVVQNSEFTPREASTQHITLDNRSRWGAKLKTLIQTNLPSVTDYMYTNSLRVKLMESYDEATGTATYEWHDITLPEGNFDSGRIIDLLNNAIWELYLTYGRQNGVREDQIGIKFDTRNFRLGFDPLTNLIMPGHYTYEYFHPDIVLMKGCAVDFSKTRLNNVLGWRKRYPYQPGFVITYDDLVGGDIPPLLDLAAYLKKPREGAGGPIIRALQKDSKGRSYHVQYTDLGEVTGYRSLYLAYNYTSDVTHLRKSTVRSWLVLTAPDITGGAQQLYWSLPDMALAPTTFRPSGQTPATFPVVSTEPLPIAARTIFNAQPGYAQIVNQNTSQTMVYNRFPENAILMRPPQPFMVQVPENVTTVTDHGTLPLQNTLSGVQRVAVTDSRRRTCPYVYKCAATLEPHIMSSRTL

>Q9RH46_COXBU/173-403 PF00198

ERTEKRVPLSRIRQRVAERLVQVQQEAALLTTFNEINMQLVMELRKKYREEFEKKFKVRLGFMSFFTKAVVEALKRFPMVNASIDGSDIIYHNYYDIGIAIGTERGLIVPILRNAEKMNMADIEKQIREYASRAQEGRLNIEELTGGTFTITNGGTYGTLLSTPIINPPQTAILGMHKIMDRPTVENGEVVVRPIMQVALSYDHRVIDGREAVLFLVTIKELLEDPARMIL

>Q9VIT8_DROME/418-510 PF00207

TWLFTDIRKSQKEVTDLEIKLPDTLGTWIVKGFSLHPEKGLGIFQSNLTQIRTIKPYSLFIHLPYSVKLGETVRIPVLIVNLFFTCFFFKVEL

>PEN3_ADEP3/28-484 PF01686

TEPLYVPPRYLGPTEGRNSIRYSQLPPLYDTTKIYLIDNKSADIASLNYQNNHSDFLTSVVQNSDFTPMEASTQTINLDERSRWGGEFKSILTTNIPNVTQYMFSNSFRVRLMSARDKETNAPTYEWFTLTLPEGNFSDIAVIDLMNNAIVENYLAVGRQQGVKEEDIGVKIDTRNFRLGYDPETKLVMPGSYTNMAFHPDVVLAPGCAIDFTFSRLNNLLGIRKRYPYQEGFMLTYEDLAGGNIPALLDLTTYDQENSSTIKPLKQDSKGRSYHVGEDPEAGDTFTYYRSWYLAYNYGDPATGTASQTLLVSPDVTCGVEQVYWSLPDLMQDPVTFRPSQTPSNYPVVATELLPLRSRAFYNTQAVYSQLLQQATNNTLVFNRFPENQILLRPPESTITSISENVPSLTDHGTLPLRNSIPGVQRVTVTDARRRVCPYVYKSLGVVTPRVLSSRTF

>Q7WJQ5_BORBR/15-188 PF02737

HAVVIGGGTMGADVAVVLARALCRTTVVEPDAARAAALPRRAADNLAQAGRAEGAAHIAVVPGLDAVEWDSVGLVIECVPERLDIKQALFAELARRAPAHAILASNSSSFPISAIGAGLPTRERMLGLHFFMPAHLVPLVEVVLCEASDPACADVLIAFMRRCGMVPVRVAQDL

>O49855_SOYBN/36-264 PF03767

RMKTGHGGHYIPEVSCQSWRLGVEAHNVIDWKTVPQDCEGYIGNYMLGEQYRSDSKIVNQQAYFYAKTLNITAKTAWVFDIDETTLSNLPYYADHGFGVELYNETSFNKWVDLGEAPALPESLKLYKKLLSLGIKIVFITGRPLDQKAVTATNLNLKLAGYHTWEKLITKNTSEYHGKTAVTYKSTERKKLEEKGYKIIGNIGDQWSDLLGTNTGDRTFKLPDPMYYIS

>Q9LNK4_ARATH/241-467 PF00198

VELGSVVPFTTMQGAVSRNMVESLGVPTFRVGYTISTDALDALYKKIKSKGVTMTALLAKATALALAKHPVVNSSCRDGNSFVYNSSINVAVAVAIDGGLITPVLQNADKVDIYSLSRKWKELVDKARAKQLQPQEYNTGTFTLSNLGMFGVDRFDAILPPGTGAIMAVGASQPSVVATKDGRIGMKNQMQVNVNVTADHRVIYGADLAQFLQTLASIIEDPKDLTF

>Q8ETF3_OCEIH/100-313 PF03417

TNEYYVRNYDFTPLLYDHLFQLVDPEKSFASAGYSQQVLGRIDGANSEGLVIGLHFVSYTEYQIGISAWTAIRMVLDTCSSTSQAVNMLKEIPHAACYNFSIGDKSGDIAVVEASPNKVVMREHNSYLTCVNHFQNQDLHQKNRQHIEFSEKRNMHMQELNKQGFSEKQMFEQFKDSHSPLFFKNYDDFFGTLHTFSYSFHDSKVTTCLAQGDN

>O29907_ARCFU/221-594 PF01314

FNRVVDEVLQRIKDDFTCNMFTQLGTSGYMETAEGFGDLPIKYYTQGTWDGATKISGAAMAASVLRGNDGCLGCVVRCGRVVEHKGKQIHGPEYETLAAFGALQLNDDLESLIEINYLVNDLGMDSISAGVSVAFAMYLTEKGVGDFNIKWGDAEAVKQMVKDIACRRGKGEELAEGVRFIGLKYGKEAWSAHVKGLEIPMHDARAFSSLAAAYATHPRGACHLPHQMYLYEMGKTISEYGIKSDDRFSNEGKGILAAKVQNFSELFNAITMCAFMPVKPRHIAEMLRAVTGYNYTVENIYTAGERMFTLKRVYNVKCGVKAEHDTLPEIVLQPLEGGSAGNVPDVKKQVSELYEFRKWPEGVPSREKLEELGL

>Q38WN9_LACSS/2-70 PF07288

IYKVLFQADKTQSPLREATKSLYLEANSAVEARQLVEDNTPYNIEFVQELTGEHLAYEQESEDFKLTEF

>ACEK_AERHH/9-568 PF06315

VAETILQRFESFYSRFLEITQGSKSRFENSDWLGVQLAGRERIRLYDHHVGATTALIKQMMGATLPSQALLKQVKGAFSDLLPKCENFEVAESFFNSVYRRIFRHRNIRDENLYIHPFRSRGEHPDLSALLRIYRTDLAHLPQTLSQLLGDYSFTLPYEDKQRDIVDIQRHLAENGPAILHDEPFAIELLKEVFYRNKGAYLVGLIRVKGQVFPFILPLLSTGQSIYVDTVIFEPELASIVFGFARAYFMVYAPMPALFVLFLRQIMPHKPDYEIYNAIGCQKHGKTELYRHYQQHLAQSREQFVIAPGIKGMVMSVFTLPSYDVVFKVIKDEFTPPKDVSHEQVKAKYRLVKQHDRVGRMADTQEFTNFEFPLDRISPELLAELKTVAPSALTLTDDKLVIKHLYTERKMIPLNLYLDKADEQQTRLALEEYGNAIKQLAAANIFPGDMLFKNFGVTRHGRVVFYDYDEICYMTECNFRQIPPPRYPEDEWSAEPWYSVAPNDIFPEEFATFLLQKPQVRDIMMQLHKELFDANYWKMLQSNIKEGVFEDVYPYRRKKR

>Q20449_CAEEL/27-98 PF04083

DDPELNMNTSQIIERWGYKAEVHTVTTEDGYILQMQRIPYGKTSVTWPNGKRPVILLQHGLLACASDWVDNL

>Q72KB3_THET2/12-194 PF02737

KVGVVGAGTMGSGIAALVASAGIPVVLLDIPGKEDRNEYAKRGLERALKARPAAFMDPERARYVEIGNTEDDLEKLRDCDWVVEAIVEKPEPKQALYARLESLLKPTAIVSSNTSGIPMRVLLEGRSEGFRRRFLGTHFFNPPRYLHLLELIPTPETDPKVLEEIRRFGERILGKGTVLAKDS

>Q9K3H2_STRCO/147-371 PF00198

ASPGRQLSKRQRTVGAVVTESMRTVPAAAAYAKVDVGQAEELARQLSERTGSFVSLPVLLIKAVARRHAAHPLMFAALTDDGAVRESERAHVGVTMDAGRGLYTPVVHDAAELSGDRIADLLTGFRSKAFRGTFQAGELTGANIMVAPHTTEGMVLATPIVFPGQTCVVSVGAVDDQVLLDAQGAPRTHRFVHLGLVYDHRVVNGRDAMALLKDVKEELEAPAAL

>O30212_ARCFU/216-588 PF01314

LKEHYSALLESVAANPGKELWHTYGTLMYTTQGYELGDTPAKYFTEGVFPVFRISGEAMLENYEVKPEGCANCPVICGHRVRGVKMEYESVASLGSLCGIYDLDGVLDATQYCNEAGLDVISAGVSVAFAMYLTEKGLLKDGVRFGDAEGLLEMLKKIVGREGLGELLAEGTKVAAKKLGVSEEETAQVKGLEIPMHDPRAFSGMALTYATSSRGACHLHSDYFTVDIGAAPVPELGVIPTNRFDESEEKVRMHVIHQSAKEMWNSYILCMLGLIGVSDAANFHSAITGESVTPSDVAKIGERTYMLKRMINLKLGMKKEDEKLPEIVRRPLKKGGTGGYSPNVKRMLEIYYRLRGLDDNGHPTKEKLEDLGL

>Q8EZ72_LEPIN/39-171 PF07696

ICEFDQIEFALDPDLSNEVPKEPKKSLVFLPKENSFLKLGFIKESVWIRFNIKQYPRSRCFLRIPQVTLDGAALFAKSSVQITGDRFRYSERFVDDYYPVFYLEPLDIQKEKNQYYLWIKTSSIINFPIFLES

>GLKA_PYRFU/18-451 PF04587

SVPKVKGVLLGYNTNIDAIKYLDSKDLEERIIKAGKEEVIKYSEELPDKINTVSQLLGSILWSIRRGKAAELFVESCPVRFYMKRWGWNELRMGGQAGIMANLLGGVYGVPVIVHVPQLSRLQANLFLDGPIYVPTLENGEVKLIHPKEFSGDEENCIHYIYEFPRGFRVFEFEAPRENRFIGSADDYNTTLFIREEFRESFSEVIKNVQLAILSGLQALTKENYKEPFEIVKSNLEVLNEREIPVHLEFAFTPDEKVREEILNVLGMFYSVGLNEVELASIMEILGEKKLAKELLAHDPVDPIAVTEAMLKLAKKTGVKRIHFHTYGYYLALTEYKGEHVRDALLFAALAAAAKAMKGNITSLEEIREATSVPVNEKATQVEEKLRAEYGIKEGIGEVEGYQIAFIPTKIVAKPKSTVGIGDTISSSAFIGEF

>Q9M0F4_ARATH/28-256 PF03767

RAASFIKLPRSSIASYCESWRLAAETNNVGPWKVIPSQCENYIKNYINGGQFDKDYDVVASYAIDYAKTVKVGGDGKDAWVFDIDETLLSNIEYYKANGYGSEPYDSIKYNEVVEKGKDPGYDASLRLYKALKKLGFTIILLTGRDEGHRSVTEKNLRDAGYFGWNRLLLRGQNDQGKTATQYKSEQRSQVVKEGYTIHGNTGDQWSDLLGFAVASRSFKVPNPMYYVA

>ARYL_CHICK/20-281 PF00797

DLQTLTAIFQHHIQAIPFENLSMHCGETIDLDLQATYNKIVKKKRGGWCMETNYLLFWALKEMGYDICVLGGNSYEPAKKAYTDEINHILLKVVIKGSSYIVDAGFGGGPYQTWLPMLLISGKDQPQIPGIFRFIEDNGIWYLEKVKRKHYVPEGSVPLTDNPEMGNIRKLYSFTLEPKHIDDFQELNAYLQVAPDTILQKKSICSLQTTDGFYALVGWTFSEMKYKYKEDADLLQTTTLTDEEVEKTLKDKFNIVLENKLI

>V2RX_MOUSE/624-874 PF00003

LVILSIFGALVVLAVTVVYVIHRHTPLVKANDRELSFLIQMSLVITVLSSLLFIGKPCNWSCMARQITLALGFCLCLSSILGKTISLFFAYRISVSKTRLISMHPIFRKLIVLVCVVGEIGVCAAYLVLEPPRMFKNIEIQNVKIIFECNEGSVEFLCSIFGFDVLRALLCFLTTFVARQLPDNYYEGKCITFGMLVFFIVWISFVPAYLSTKGKFKVAVEIFAILASSYGLLGCLFLPKCFIILLRPKRN

>ODO2_BACSU/184-415 PF00198

DKPVEVQKMSRRRQTIAKRLVEVQQTSAMLTTFNEVDMTAVMNLRKRRKDQFFEQNEVKLGFMSFFTKAVVAALKKYPLLNAEIQGDELIVKKFYDIGIAVAAVEGLVVPVVRDADRLTFAGIEKEIGELAKKARNNKLTLSELEGGSFTITNGGTFGSLMSTPILNSPQVGILGMHKIQLRPVAIDEERFENRPMMYIALSYDHRIVDGKEAVGFLVTIKNLLEDPEQLLL

>Q0AVX1_SYNWW/215-629 PF01314

VLELSAKGQKLIKSASSYQAFCAYGTMNATIPYGGFNALSVHNYSRGTLPDWKQKAGRQIVDIYGSRHIACQSCIIACGHLAEINEGKYAGTLVKDMEITPTVSYSSNVGLSTEASIKSSELCQRYGIDMSSSGSVIAFAMELYQKGIINKDDVGYELAFGDDDAAFALLRDISLRQGIGDILAEGVKRAAEHWPGADDYAIHVKGVEVPMIDPRGRWSTWTLGMLTNIRGGDHLRCRNPVENLRYNENLYHYQKERFGFKKPMYDRLDMPENLKSAAIDLESDTVDIAIMSKWAEDLINLYNSVGICIRPPIMETIGPTLLAEIYTCMTGIPMSPDELMMGSERAWNLMKLFNIRHGEVAGDSKFPRRFYRELQSGNIVDEDKVQAVLEQYWQARGWDPGTGHPLPETEKRLGI

>O73639_FUGRU/617-868 PF00003

LLVVSLIGSFLTCAVALVFFYHRTSPIVRANNSDLSFLLLFSLTLCFLCSLTFISPPSQWSCMLRHTAFGITFVLCISCILGKTIVVLMAFRATLPGSDVMKWFGPGKQKAIITFSTLVQVVICTVWLVVAPPTPRQYMPRESAIIILLCDEGSTIAFSLVLGYIGVLACMCFLLAFLARKLPDNFNEARLIAFSMLIFCAVWVAFVPAYISSPGKYSTLTEIFAILASSYGLLGCIFAPKCYIILMKSEKN

>ABF1_TRIRE/352-496 PF05270

ISLRATTACCTTRYIAHSGSTVNTQVVSSSSATALKQQASWTVRAGLANNACFSFESRDTSGSYIRHSNFGLVLNANDGSKLFAEDATFCTQAGINGQGSSIRSWSYPTRYFRHYNNTLYIASNGGVHVFDATAAFNDDVSFVVS

>Q7Q7T0_ANOGA/564-655 PF00207

SWLWQNVSIGRTGSRTVHEVLPDTTTSWYLTGFSIDPVYGLGIIKKPIEFITVKPFYIVDSLPYSIKRGEAAVLQFTLFNNLEAEYIADVTL

>ACEK_PHOPR/9-571 PF06315

VAHTILQGFDAMYGRFLDVTAGAQERFEQQDWSSVHLALKKRISFYDHHVGLVTSQIQIMLGERYANRSFLMAVKSSYENLLLDYPRYEIAESFFNSVYCRIFEHRNINRDKLFVHSSQENRIPTYPTQLTRIYHAHSGLTSLFDRVLDDTPFTQVWEDKQRDTKLIINHLKQELGAAFNDETSLELIREPFYRNKAAYLIGKITLNHDGQYQTIPLVLPVLNNSNKQLYVDACICHVNDASIIFGFARSYFMVYAPAPGALVRFLSNLIPNKTNAELYTAIGCQKHGKTELYREFLTHLDNSDDQFVTAPGIKGMVMSVFTLPSYDFVFKIIKDKFAPQKDISHATVKEKYKLVKEHDRVGRMADTQEYRHFTFERHRFSDELLAELLAVAPSIIKVTSDQIIISHLYMERRMIPFNIYVEQANDDDLRDAVDEYGKAIKQLAAANIFPGDMLFKNFGVTRHKRVVFYDYDEISYMTEMNFRKIPEPRTPEDEMSAEPWYSVGIYDVFPEEFRTFLLINPKVKALFNELHSDLFEAKYWQSLQQNITHGQYEDVYPYRGVHR

>Q18843_CAEEL/4-286 PF06472

LALDFLFFRRFWRLLKILFPCHRRSITAWLAGALLIATALDQVMTYLVGIQPSLFYVALGNKDADTFKILCARGAAIILGKAFTLAVFKYLTNMLAIKSRQVCNLTMHRLYFKRQAFFKLSSSGDMLDNPDQRLTQDIEKATRILSNDLLAPIATAPFIIGYYTWLTYESSGWIGPAAIYTYFCIQTIINKMILSPIVQKVSEQEKMEGDFRQRHMEVRSNVEAIAFYRAGVLENIMTNQKLKNLIETQKSLTEWRMVLNSITNVFDYFGGILSYLIIGVPVF

>Q9X8M8_STRCO/32-237 PF03767

TRSRTWTRVTGTAAVVAAALTATVTPSVAAPAETPPASVTAAAADVGYDTWQRDCRAVMDAALPYLKERIAGSAPGEKQAVVLDIDNTSLETDFGFSYPQPANRPVLEVAEYAQEHGVALFFVTARPGIIEAPTEWNLAHAGYESSGLYVRGFLDLFKDVAEYKTEQRAEIESKGYTIIANIGNSASDLSGGHAEKTFKLPDYDGQ

>FAS2_YEAST/1769-1832 PF01648

VGVDVELITSINVENDTFIERNFTPQEIEYCSAQPSVQSSFAGTWSAKEAVFKSLGVKSLGGGA

>OXAA1_BACHD/57-241 PF02096

GSFGLAIIVVTLLIRLLILPLMIKQLKSTRAMQALQPEMQALREKYSAKDQRTQQKLQQETMALFQKHGVNPLAGCFPVLIQMPILLAFYHAIMRTREIGDEHFLWFVLNQPDPILLPIIAGITTFLQQKMMMVTDNPQMKVLLYVMPVMILVFAMFLPSSLALYWVIGNLFMILQTYFITGPNV

>HEPC1_SALSA/2-86 PF06446

KAFSVAVVLVIACMFILESTAVPFSEVRTEEVGSFDSPVGEHQQPGGESMHLPEPFRFKRQIHLSLCGLCCNCCHNIGCGFCCKF

>Q9RZ10_DEIRA/56-237 PF02737

TVTVCGSGVLGSQIAFQTAFHGFDVHLYDINDAAIAKARETLGKLQARYQQDLKVDAQQTGDAFARISFFTDIAEAVKGVDLVIEAIPENMDIKRKFYNQLGEVADPNTIFATNSSTLLPSQFMEETGRPEKFLALHFANEIWKFNTAEIMRTPRTDDAVFDTVVQFAKDIGMVALPMYKEQ

>Q6XBT1_SACPS/23-529 PF07247

RMGHLENYFAVLSRQKMYSNFTVYAELNKGVNKRQLMLVLKLLLQKYSTLAHTIIPKHYPHHEAYYSSEEYLSKPFPQHDFIKVISHLEFDDLIMNNQPEYREVMEKISEQFKKDDFKVTNRLIELISPVIIPVGNPKRPNWRLICLPGKDTDGFETWKNFVYVTNHCGSDGVSGSNFFKDLALLFCKIEEKGFDYDEEFIEDQVIIDYDRDYTEISKLPKPITDRIDYKPALTSLPKFFLTTFIYEHCNFKTSSESTLTARYSPSTNANASYNYSLHFSTKEVEQIRAQIKKNVHDGCTLTPFIQACFLVALYRLDKLFTKSLLEYGFDVAIPSNARRFLPNDEELRDSYKYGSNVGGSHYAYLISSFDIPEGDNDKFWSLVEYYYDRFLESYDNGDHLIGLGVLQLDFIVQNKNIDSLLANSYLHQQRGGAIISNTGLVSQDTTKPYYVRDLIFSQSAGALRFAFGLNVCSTNVNGMNMDMSVVQGTLRDRGEWESFCKLFYQTI

>Q8YAK8_LISMO/127-348 PF07859

FIFYHGGGFVGGTPAVVENFCKGIAEKLPAVVINVDYHLAPEFPAPAAPKDCYRVLEWVVEQSNELGIDASKIGVSGDSAGGTLAAAVSYMDYEAETNYVGFQALLYPALTLVDEDNDKYQWDISKFGASEDTLPLVAPGIIGMNSSGELLRKAYVRDENPAAPIYSPLSAVDKSIYPPTLIASAEFDALRAFADVFAKELRASGVQTKAIVYQGMCHAFID

>O28003_ARCFU/228-612 PF01314

FKAASKEMLDKIKQNPVTGEGLPKYGTAVLVNVINNAGALPYKNWQEAYNEKADEISGETLEAKYLRKRVACWGCSIGCGRATEVKTGPFKVLNTEGPEYESIWALGNDTAVINLEAVIKANHFCDELGIDTISMGSTIACAMELYEKGYIKDEDLQGLDLSFGSDAAMVEAVWRTAYKTGIGKYLALGSKRLAEIFGAPELSMSVKGLEMPAYDPRGIKGIGLNYATANRGGCHVTGYTVSPEIVGLPEKIDPLTYEGKAAWVKAFQDFTCVVNSAVNCLFTTFALGAEDYAVLLSHITGWDLNADEIMKIGERIYNLERVILNKYGFDGKDDTLPKRLLTEPLKEGPAKGQVVELDRMKEEYYQLRGWENGVPTKEKLKELDI

>YQJZ_BACSU/21-82 PF03992

VKSENDTGYGETAERMVSLAADQPGFLGVESVREADGRGITVSYWDSMDAINHWRHHTEHQA

>NHOA_ECOLI/21-257 PF00797

NIDTLRALHLKHNCTIPFENLDVLLPREIQLDNQSPEEKLVIARRGGYCFEQNGVFERVLRELGFNVRSLLGRVVLSNPPALPPRTHRLLLVELEEEKWIADVGFGGQTLTAPIRLVSDLVQTTPHGEYRLLQEGDDWVLQFNHHQHWQSMYRFDLCEQQQSDYVMGNFWSAHWPQSHFRHHLLMCRHLPDGGKLTLTNFHFTHYENGHAVEQRNLPDVASLYAVMQEQFGLGVDDA

>Q704C0_THETE/215-595 PF01314

INKIGIEAVEQGRKMPGYSFWMKQGTTSTVEWAQEASVLPTYNFSEGQFDEFEKIGGAMVEKMEVDLKSCPLCFMPCGHWVPSEEGTAEVDYENIALLGSNLGIGDLSKVAELNRVADLMGMDTISLGNTLGFAMEASERGLTKEFGYKIEWGDYKAARELAIDIALRRGFGDLLAEGTMRASQRLGKGAEELAVQVKGLEVSGYDCHAAPAMALAYATSPIGPHHKDAWVISWEVKTDRLGYTREKAAKVIELQRIRGGWFEAFVACRLPWVEVGLSLDWYPKLFKAATGIDATPDYFNEVGDRIYALIRAFWIREYGYWSRELDVPPPKWFKRPLTKGPLKGAHLDYDKYNELLSHYYDLRGWGQNGVPRRSTLKRLGL

>Q92N21_RHIME/10-77 PF03992

VAAGQEEAFEAVWKARDSSLSEMPGFIEFHLLRGDSVPEEGYTPFISKSAWENRDAFIAWTKSDNFRA

>Q98AX6_RHILO/20-257 PF00797

TVDGLVALQQAQMRAIPFENIDVLLGDIPNLTENSIWAKLINARRGGYCFELNKLFGLALEALGFTIQPILCRVRMGAAEGGPRTHQAFILTIEGVDWLADAGFGGPAPIAPLRIDTEELQTAGRDVFRLRADSASGELVVERKNGNEWFPLYGFDRATALPSDFEGANFICARWDRSPFPSSLMMSVLTAEGPANLFNKDFSLIRNQIEETETLKTKSELQRVLSDVFRLHLPRSTI

>Q18598_CAEEL/60-341 PF06472

AHVDGVFLGKLARILKILIPGFFSKESFYLILIAVSLLCRTYADVYMIITSTKIEASIIDRNPLLFALEAFKYVLNLPAISVTNAILKFGIAELKLRFRERLSTHLYSQYLKGFTFYKMSNLDTRIQNADQLLTQDVDRFCDGIVELYSNLSKPILDVFLYLFRLGTSLGFSSPSILFSYLLFTGVGLTYLRRPIGRLTVEEQALEGEYRYVNSRLIMNSEEIAFYQGNQSEKETIMSTFASLVQHLRKIILFRFSIGFVDNIVAKYLATVVGWYAVGSSFF

>O33291_MYCTU/11-71 PF03992

AKPESVDTVRDILTRAVDDVHREPGCQLYALHETGETFIFVEQWADAEALKAHSGAPAVAT

>Q7TZG9_MYCBO/41-358 PF06472

LRYLTPWGRQFWRITRAYFVGPNSVRVWLMLGVLLLSVVLAVRLNVLFSYQGNDMYTALQKAFEGIASGDGTVKRSGARGFWMSIGVFSVMAVLHVTRVMADIYLTQRFIIAWRVWLTHHLTQDWLDGRAYYRDLFIDETIDNPDQRIQQDVDIFTAGAGGTPNAPSNGTASTLLFGAVQSIISVISFTAILWNLSGTLNIFGVSIPRAMFWTVLVYVFVATVISFIIGRPLIWLSFRNEKLNAAFRYALVRLRDAAEAVGFYRGERVEGTQLQRRFTPVIDNYRRYVRRSIAFNGWNLSVSQTIVPLPWVIQAPRLF

>A1S030_THEPD/234-608 PF01314

VKLLIREAWSALNRNVLWKHWGTSSGGYEVASLTSSEPVRNWQEEWHDSRSMGVQNYEAHWVKRYWGDYGCPQTCMKISRLRSGKFSGATTDGPDYELQAYLGPNLGVFEPRANVYLSSLADELGLCGIQTGNVLGFAAELYEKGVLTREDLGFELKWGDAEAFARLMSMIARREGIGDCLAEGTARAALKLGKLKGVDLSKYAVHVKGVGVGAHGARSGKDFPQAFTYAVGVQGGDHTSPPRLPVDRQWGEFTSAFEDSAVICSFNVHGDLPFQFLRAITGWDITRETWMRVHGRRIITLQRVLLLLGGPDLYWDPRRDDDNPPRFYEPLPSGPFKGRSVSREEVESQKREYFGYLGWDSLGIPTEETLRELGL

>Q8UCM2_AGRT5/10-75 PF03992

VVPGYEETFESIWRERKSHLSELPGYIEFHMLKGPKADDHTLYASHTVWATKDDFLAWTKSEQFRA

>O35265_RAT/279-530 PF00003

LTLMSLGFFVVTGLVIGVFIIHRNTPIVKANNRSLSYILLITLTLCFLCPLLFIGLPNTATCILQQNLFGLLFTVALSTVLAKTITVVMAFKITAPGRKTRWLLILRAPQFIIPLCALMQILFSGIWLGTSPPFVDMDAHSEHGHIIILCNKGSAIGFYCTLAYLGVMAFGSYLLAFMSRNLPDTFNESKALAFSMLMFCSVWVTFLPVYHSTTGKVRVAMEMFSILASSASILTLIFVPKCYIVLFRPERN

>Q9BHG2_LEIMA/117-397 PF06472

FNQEVSVLNRFMQLLRVAIPNCHGREARSIYILFVLMVVRAYVSVRLVNVSGLVSRTAIEGNLRHAIRALALFAVSCVPATLLNVTLDYYSELLGLHCRDNLAAYFSDRYLKRRVFFQMAGLHEVDHVDQRITEDVRNWARVSASLFTSIPRPLIEAITFSFTLARQTGWRGTLLTWSYYLSFAVWICCYAPNLDWMVQQRMAKEGAVRGEHQRLLAHAEEITLTKGFQFHEKVLQRLFKAVTDQSRYAAYVRSRFEFTETLHNKYGSVLLGYVVCAMAAM

>Q0YLF5_9DELT/218-631 PF01314

VWQHTTQFLTSLETDLNDFGKKMKQAGTAGTLTDSTTSGDSPLLNWQGVPADFPAEKANKIDLFSVTRYETRKYHCYGCPFGCGGLCMIPDEPLLKETHRPEYETICGFGAQLLNDNIESIFMVNELLNRAGIDTISCATTVNWAFEAYAKGILTQEDTDGLELTWGNHGALVALVQKIVAGEGIGAILANGVKKSAEHFGGEELAAPMHVHGQELPMHDSRSTSGGLDLGVGYETEPTPGRHTATFAGWDQYKHSDHPKNRLFDKFRLKSRYEKPVDDDHEKQGERLRGASCAEDIINGAGLCNFGFYLGPAPPLVEWLNATTGWRKTFDDYLLVGQRIKTVRHAFNIREGLEVAEIRMPERARGNPPLTTGPNAYSGNVLAWDDAKKDYYRAMGWDEITGRPLRETLRSLEL

>Q9X5D6_ZYMMO/15-78 PF03992

AKADQQEALQQALLHYADQSKTEEGYLAFHIGYDKSQPPRFILNERWQDHAAFFAHEKSDIFQD

>Q9M0F5_ARATH/27-255 PF03767

IRTSFIKLPGSDGSRYCDSWRLAAETNNVGTWDLIPSICVDSVAEYLNGDQFLSDYSVIVDYALAFAKSVEISGDGKDVWIFDIDETLLTNIDYYKAHGYGSEPYDDNKFSEWVEQGTAPAFDASLRLYNALKKLGFTIILLTGRDEHQRTSTETNLRDAGYSGWERLLLRGPNDQGKSATNYKSEQRSKLIEEGFKIRGNSGDQWSDLQGFAVADRSFKVPNPMYYIP

>Q53908_STRCO/20-83 PF03992

DGPATQHKLVELATGGVQEWIREVPGFLSATYHASTDGTAVVNYAQWESEQAYRVNFGADPRSA

>O97227_PLAF7/204-445 PF00198

MDIIEEVSLKGIKLAMCKSMNESLQVPLFHLNEMCIINNLIKMRKEYKEQQKNLQTKETNITITCILIKLISNVLKEFPILNSKFNFKTNTYTMYKNHNISIAVDTPHGLLVPNIKNVQNKNILDIQKDLLSLRDKANNMQLDKSDITNGTITVSNFGAISGTFATPIVFDNQACIIGIGKMEKKLLLKDESNNLNSLNDILVADTINFTFGADHRYIDGATLAQFSKMLKMNIENCASLGP

>Q9F0Y4_XANAL/160-226 PF01648

VGIDLERPITPAARAALLSIAIDADEAARLAKAADAQWPQDLLLTALFSAKESLFKAAYSAVGRYFD

>K6PF_THEZI/7-452 PF04587

EKARGLSIYTAYNTNVDAIVYLNGETVQRLIDEFGADAVRKRMEDYPREINEPLDFVARLVHALKTGKPMAVPLVNEELHTWFDSHFRYDVERMGGQAGIIANLLSNLDFREVIVYTPHLAKRQAEMFVRKPNLFYPVVEGGRLVLKHPWEAYREGDPVKVNRIFEFRAGTAFKLGDERIVVPFSGRFIVSARFESIRIYTEPGLRPFLPEIGERVDGAILSGYQGINLRYSDGKDANYYLRKAKEDIMLLKREKDLKVHLEFASIQSRELRKKVIYNLFPLADSVGMDEAEIAYVLSALGYDELADRIFTYNRIEDTVLGGKILLDEMNLDVLQIHTIYYIMYITHADNPLSEEELRRSLELATTLAASRASLGDITSPDQIEIGLRVPYNERGEYVKLRFEEAKRKLRTKEYKLVIIPTRLVQNPVSTVGLGDTISTGAFASYL

>Q8FRN7_COREF/14-196 PF02737

QAVVIGAGSMGAGIATLLANAGITVTLLDRHSGDPEDPNRLAESGLERQIQRGAFYRPEFSSRIQTGNIVDDTAALTRADWIIEAVFEDLTVKHDTFRLIEEHRSPGSLVSSNTSTIPLAQLTEVMGTPMRLDFAIVHFFNPPTTMRLVELVTGPDTTPKTATDLTRIIEQQLGKVVLHCRDT

>Q1YQU1_9GAMM/1-561 PF06315

MAKTILNGFRSYFADYLNSTLSAKARFEKADWHGVQQANVDRLEMYKAKVAQTVMYLGMVTNKDIADLDLWAESKKAYTQLVFNFPNFEIAETFFNSVFADLHDHDKINDDIIYVLSSHMIEAPAAEYSIFVRYEGDDRRDIFRRILKESEFSLPGEDIELDLDCILSVFCSEVTPNLIGSPDDLKFDLLESTFYRSKAAYMVGRVIDGDQTFPMAIVILNNEKGQLYVDTAIFNTDDLSVIFSFTRNHFMVDAPLPYQYAHFLKTLMPKKLDYEIYNSLGFPKHAKTEFYRQLVHHLDSSNDQFVIAPGIKGMVMTVFTLPSYNIVFKIIKDKFAPPKEVTHQIVRDKYRLVSRHDRIGRMADTQDFDNLVFPLDRFSPELLEELQKVAASTIEIRGDKLVIKHLYTERYMTPLNIYLETANDEQMRSAMEEYGNCIKQLAAANIFPGDMPLKNFGVTRHARVVFYDYDEIAALTDCNFRKIPQPRTEEEEMQAGTWYTVGPDDIFPEEFRLFFSGNTKARRMFEEMHSDLYDVDFWQGLQEKIRDGFVLDVFPYRRAKR

>Q9XA49_STRCO/261-489 PF00198

RSDGTRVPLKGVRGAVADKLSRSRREIPDATCWVDADATELMRARTAMNASGGPKISLVALLARICTAALARFPELNSTVDTDAREVVRLDRVHLGFAAQTDRGLVVPVVRDAHTRDAEALTAEFARLTEAARAGRLTPGELTGGTFTLNNYGVFGVDGSTPIINHPEAAMLGVGRIVPKPWVHEGELAVRQVVQLSLTFDHRVCDGGTAGGFLRYVADCVEQPAVLLR

>Q99IW7_9BACT/10-70 PF03992

ATPGQRDALIAILVEGASSMPGCLSYVVAQDPKDPDAIWITEVWDSPESHKASLSLPSVQD

>Q9BXP1_HUMAN/42-536 PF00324

GVVVPTVLSMFSIVVFLRIGFVVGHAGLLQALAMLLVAYFILALTVLSVCAIATNGAVQGGGAYFMISRTLGPEVGGSIGLMFYLANVCGCAVSLLGLVESVLDVFGADATGPSGLRVLPQGYGWNLLYGSLLLGLVGGVCTLGAGLYARASFLTFLLVSGSLASVLISFVAVGPRDIRLTPRPGPNGSSLPPRFGHFTGFNSSTLKDNLGAGYAEDYTTGAVMNFASVFAVLFNGCTGIMAGANMSGELKDPSRAIPLGTIVAVAYTFFVYVLLFFLSSFTCDRTLLQEDYGFFRAISLWPPLVLIGIYATALSASMSSLIGASRILHALARDDLFGVILAPAKVVSRGGNPWAAVLYSWGLVQLVLLAGKLNTLAAVVTVFYLVAYAAVDLSCLSLEWASAPNFRPTFSLFSWHTCLLGVASCLLMMFLISPGAAGGSLLLMGLLAALLTARGGPSSWGYVSQALLFHQVRKYLLRLDVRKDHVKFWRPQLLL

>Q704C8_THETE/213-589 PF01314

LEEFLRDFTPRFIGDKSVKSLFEAGTLRLVELGNIMGFFPSYYWGQTSADGWERLAWPTLRSYLLRPAACLHCPAACHRLVRSGKYGVSVDLDYETVFALGGLVGCFDPDEVIKLNDLADRLGMDTISLGNVVAFAVEASRLGKLPPLEWGCDTAERLAVDIAYRRGLGDLLAEGVAAAAAKIGAREIAVHVRGLEPAGYDPRALKGMALGYAIGYRGADHLATMAYAIDYAGMAGGPQSLGDEKIHAVAHMEEVAAIMDSLVLCKFGRGVYDLYPGGRGLETIARLLSDVTGDGWTAGLVRESALRVINLTRALNVMMGDPGDGLPERWYKPVKFGDRILQLNREEVEDALRKYYELRGWDERGLPRPAALSELGL

>Q9X9W3_STRCO/138-196 PF03992

PDPDRQRAWVDAVFEALESEPHPHPGGISAHFHLSTDGTRVLNYAEWESAQAHLDALAA

>Q59658_PELCA/228-450 PF00198

PAQPTSKPMSAIGAAISNTVTNSWTIPQFPVTMGIEMGAAKEFRAGLKAAGKAVSMNDMVIRACGKAIEQYPMVNATLGGKEYGLNADVNIAVAVGTDDALMMPVVKGCQALSLEEVASASRAVIDKVKAGTCGPAEMAGGNFAISNLGMLGVDSFGALVPPGMSAILAVGGIKDEVVVKDGEMVPVSTMKVTLVADHRVVDGLYSAQFLVELKRLLENPEEL

>Q648Z2_9ARCH/105-488 PF01314

MKEHVRSAIRKIKENPVTGEALPTYGTAVLVNLINEHGAYPARNFQTGYFPDAARQSGETLADKYLVGKKACWGCPIGCGRSTAVPDGAFSVTSGEGPEYESIFAFGSDCGITELDAITKANHLCNELGLDTISMGTTIAAAMELVETGKIPESRLHGLNLTFGNAGAMVEAVWMTAYRAGIGDDLALGSLGLGEKYGAPELSMSVKGLELPAYDPRAVQGIGLNYATANRGGCHVSGYLISPEIVGFPEKLDPYTTEGKAQWVKTFQDFTSLVNSSVVCLFNTFALGLPDYADMLSCITGWDVDDQELLMIGERITNLERLMINRYGFDEKDDALPKRFDEPMPDGPSKGHVSLLDEMLSEYYELRGWEHGKPGSGKLRELGL

>Q9I267_PSEAE/52-115 PF03992

ARSGQSHRLGLRLQELAQAGQAAPGCLRYELRQADGDADLWLLHSEWSDEAAMQAYLSGDAQRV

>Q8G252_BRUSU/44-179 PF07696

LDLSRAVELLRNKGESVQVSTMPGPDGIVRRIEVQSDQNANASGDWAAFSIANPTDEQIDRLIVAPHFRLVGSGVIWPDLGSPRIASITPSEGFALDRQPSADADVFRITLNPGSVITFVAELSSHNLPQLYLWEP
